# Catalog of the Neotropical Trichoptera (Caddisflies)

**DOI:** 10.3897/zookeys.654.9516

**Published:** 2017-02-09

**Authors:** Ralph W. Holzenthal, Adolfo R. Calor

**Affiliations:** 1 Department of Entomology, University of Minnesota, 219 Hodson Hall, 1980 Folwell Ave., St. Paul, Minnesota, USA, 55108; 2 Departamento de Zoologia, Instituto de Biologia, Universidade Federal da Bahia, Rua Barão de Jeremoabo, 147, Campus Ondina, CEP 40170-115, Salvador, Bahia, Brazil

**Keywords:** Caddisflies, Trichoptera, catalog, Neotropical, taxonomy, distribution, valid names, synonyms, bibliography

## Abstract

The Neotropical caddisfly (Trichoptera) fauna is cataloged from a review of over 1,000 literature citations through 2015 (partial 2016) to include 3,262 currently recognized, valid species-group names in 25 families and 155 extant genera. Fourteen subspecies are included in the total as well as 35 fossil species and 1 fossil genus. The region covered includes all of Mexico, Central America, the Caribbean, and South America. Genus-group and species-group synonyms are listed. For each nominal species, information on the type locality, type depository, sex of type, distribution by country, and other pertinent taxonomic or biological information is included. Summary information on taxonomy, phylogeny, distribution, immature stages, and biology are provided for each family and genus where known. An extensive index to all nominal taxa is included to facilitate use of the catalog. The glossosomatid species *Mexitrichia usseglioi* Rueda Martín & Gibon, is transferred to *Mortoniella* **comb. n**.

## Introduction

Trichoptera are an order of holometabolous insects that have aquatic egg, larval, and pupal stages. These immature stages are ubiquitous in the world’s freshwaters, but are especially diverse in rivers and streams. In the Neotropics, larvae can be found in small trickles and seeps in the high Andean páramo, down to the very large, slowly flowing, lowland rivers, and all kinds of rivers and streams between these extremes. It is in intermediate sized, forested, mountain streams where the fauna seems to reach its greatest diversity. Intermittent streams also support a trichopterous fauna, with a few species especially adapted to seasonal drying. A very few species can be found in waters of thermal origin or with water chemistry that has been affected by volcanic activity. In addition to lotic species, those that frequent standing waters also occur, but this lentic fauna is not as diverse as that of northern, Holarctic lakes and ponds. However, floodplain lakes and pools, swamps and marshes in lowland areas, and mountain lakes all harbor species. There are also species that frequent the spray and splash zones of waterfalls and similar torrential situations as well as hygropetric habitats. These species often occur above the waterline, where a few venture quite far from the aquatic habitat. Only a single species has been reported from a container habitat (bromeliad tanks) and none of the Neotropical species is known from the marine littoral, unlike some Australian and New Zealand species. Recent reviews of Trichoptera diversity, biology, and natural history include those of Wiggins (2004) and Holzenthal et. al (2007, 2015).

Trichoptera larvae are important participants in energy flow and nutrient dynamics in the aquatic environment. They display a wide diversity of trophic adaptations, being surpassed only by aquatic Diptera in the type of food eaten and the manner in which it is obtained. Similarly, the larvae exploit a wide variety of aquatic microhabitats. This trophic and habitat diversity has been attributed to the larvae’s ability to use silk to construct capture nets, retreats, cases, and pupal shelters. In fact, the order has been divided into taxonomic units based on the differences in the way silk is used, whether to spin nets or tubes, or as mortar to make portable cases. The case makers use sand and small mineral fragments, pieces of leaves or other vegetable material, or silk alone to construct cases. Other larvae are “free-living,” but nevertheless lay down a strand of silk as they move across the substrate. Not only do larvae exhibit great diversity in their biology, they also respond to pollution in various manners, most being intolerant to most forms of pollution. As such, they have been used extensively as biological indicators of water quality, especially in temperate regions, where a large field of study has developed around this application. However, in the Neotropics, where larval taxonomy is poorly known, progress in this area has been hampered.

Like most holometabolous larvae, Trichoptera have well-developed mandibulate mouthparts, although the maxillae and labium are closely associated and the latter is modified to spin silk. The thoracic legs are well developed, but the abdomen lacks prolegs, except for a pair of terminal anal prolegs, each bearing a strong anal claw. Highly branched or single filament abdominal gills may be present. The exarate pupae are also aquatic and have dectitious mandibles, at least in the Neotropical families.

Trichoptera adults are less familiar to the aquatic ecologist or taxonomic non-specialist. Adults are small, generally drab colored, and usually begin to fly after the sun sets, when they are attracted to artificial lights, often in great numbers. However, it is this life history stage that is of paramount taxonomic importance because the species level taxonomy of the order is based mainly on structures of the adult male genitalia. Females and larvae must be positively associated with the adult males before their identities can be established. In contrast to that of larvae, the ecology and behavior of adult Trichoptera are poorly known. They, too, are certainly important components of the aquatic and riparian environments, where they serve as food for fish, birds, bats, lizards, frogs, and other vertebrates, as well as spiders and other invertebrates. Adult female flight behavior, especially upstream flight prior to oviposition, is an important compensation for downstream larval drift. The adults have specialized, lapping type mouthparts and most probably imbibe liquids, including nectar. Adult Trichoptera certainly depend on the riparian habitat for mating and oviposition sites, shelter, food, etc. and therefore may be good indicators of riparian health and integrity, and thus that of the entire watershed as shown by Houghton (2006) and Houghton et al. (2011).

Adult Trichoptera have greatly modified mouthparts. Although most species have reduced mandibles and maxillae, almost all possess well-developed maxillary and labial palps. The compound eyes are well developed, and the head may or may not bear ocelli. Antennae are long and filiform in most species. The head and thorax bear characteristic “setal warts.” Two pairs of wings are present with the forewings longer, but often narrower than the hind wings. Both pairs of wings, as well as the body and other appendages, are covered with setae, or hairs, and occasionally scales. The hairs and scales are usually plainly colored, but the Neotropical fauna contains species with brightly colored or intricate patterns of setae, especially on the forewings. Tibial spurs on the legs are conspicuous.

The world fauna contains about 15,000 described species, but it has been estimated that as many as 50,000 species may occur. In this catalog we record **3,262** valid names of extant species-group taxa from the Neotropics, including **14** extant subspecies. One species is listed as Trichoptera, *incertae sedis*. This represents 1,050 more species-group taxa than included in the catalog published by Flint et al. (1999b). In addition, we list **35** fossil species, these from Dominican and Mexican amber (the identity of two additional fossil species is discussed below). These species and subspecies are distributed among **155** extant genera, **1** fossil genus (†*Palaehydropsyche*), and **25** families (Table 1).

**Table 1.** Number of extant and fossil species and genera of Neotropical Trichoptera, by family.

Family	No. Species		No. Genera	
	Extant	Fossil	Extant	Fossil
Anomalopsychidae	28	–	2	–
Atriplectididae	2	–	1	–
Calamoceratidae	74	1	2	–
Ecnomidae	44	–	2	–
Glossosomatidae	266	5	11	–
Helicophidae	16	–	5	–
Helicopsychidae	121	3	1	–
Hydrobiosidae	183	1	22	–
Hydropsychidae	476	4	15	1
Hydroptilidae	946	7	36	–
Kokiriidae	2	–	1	–
Lepidostomatidae	28	–	1	–
Leptoceridae	224	2	16	–
Limnephilidae	51	–	10	–
Odontoceridae	45	–	3	–
Philopotamidae	377	6	5	–
Philorheithridae	6	–	2	–
Polycentropodidae	283	1	5	–
Pseudoneureclipsidae	4	4	1	–
Psychomyiidae	2	–	1	–
Rhyacophilidae	1	–	1	–
Sericostomatidae	20	–	6	–
Stenopsychidae	3	–	1	–
Tasimiidae	2	–	2	–
Xiphocentronidae	58	1	3	–
TOTAL	3262	35	155	1

## Biogeography

The Neotropical fauna is divided into two distinct faunal elements – the Chilean and Brazilian, equivalent to the Neotropical and Patagonian of de Moor and Ivanov (2008). The Chilean fauna is distinct, highly endemic, and very closely related to the faunas of Australia and New Zealand. It occurs in southern Chile and adjacent Argentina, from about the level of the Río Negro south. It shares almost nothing in common with the Brazilian fauna. The Brazilian fauna occurs in southern Mexico, Central America, the Antilles, and all of tropical and subtropical South America, exclusive of the Chilean region. There is broad overlap of the Neotropical and Nearctic faunas from the southwestern states of the United States through Mexico and Central America to Panama and Costa Rica. The fauna of the Greater Antilles is highly endemic, that of the Lesser Antilles much less so. Areas with an apparent great concentration of endemic species and high species richness include the northern Andes, the Amazon basin, and the mountains of southern and southeastern Brazil.

## Taxonomic history of the fauna

The first descriptions of Neotropical Trichoptera occurred in the 1830s in the works of Perty (1830-1834) (*Phryganea maculata* = *Macrostemum maculatum*), Pictet (1836) (*Macronema lineatum*; *Hydropsyche hyalina* = *Macrostemum hyalinum*), and Burmeister (1839) (*Barypenthus concolor*; *Barypenthus rufipes*; *Chimarrha morio* = *Chimarra morio*; *Mystacides albicornis* = *Marilia albicornis*; *Mystacides gracilis* = *Triplectides gracilis*; *Mystacides princeps* = *Triplectides gracilis*; *Macronema speciosum* = *Leptonema speciosum*). Ulmer (1913), who listed 162 names, produced the first checklist of the region. Since then, from about the end of the 19th century until the first third of the 20th century, numerous other early workers, principally among them Banks, Brauer, Hagen, McLachlan, Müller, Navás, and Ulmer, described and recorded numerous genera and species from the region. However, many of these early works, especially those of Navás, suffer from inadequate descriptions and illustrations, those of Ulmer and Müller being exceptions. Fortunately, many of the early species have been redescribed and lectotypes designated, especially in the works of Betten and Mosely (1940), Flint (1966a, 1967c), Kimmins (1957), Kimmins and Denning (1951), Ross (1938a, 1952), Schmid (1949a), Tomaszewski (1961), and Weidner (1964).

M.E. Mosely was among the first of the “modern” workers to produce several important works and revisions on the fauna (e.g., Mosely 1933, 1936, 1937, 1939a, 1954). H.H. Ross and D.G. Denning, primarily workers on the North American fauna, produced several important works covering the Neotropical fauna (e.g., Denning 1947a, 1962b, 1964; Ross 1956b, 1959; Ross and King 1952). Beginning in the 1950s, F. Schmid produced a series of works, principally on the Chilean fauna (Schmid 1955a, 1957, 1958b, 1959, 1964), and subsequently published several important revisions covering the Neotropical fauna (e.g., Schmid 1982, 1989). Since about the early 1960s O.S. Flint, Jr., has produced a large body of work on the entire Neotropical fauna, which includes major contributions to the taxonomy of most families and genera. Finally, L. Botosaneanu made significant contributions to our knowledge of the fauna of the Antilles.

Notable recent workers include E.B. Angrisano, W. Bravo, P. Rueda Martín, and J. Sganga on the fauna of Argentina and Uruguay, J. Oláh on Hydropsychidae and Hydroptilidae, R.J. Blahnik and D.R. Robertson on the Philopotamidae and Glossosomatidae, respectively, J. Bueno-Soria, S. Santiago-Fragoso, and R. Barba-Álvarez on the fauna of Mexico, S.C. Harris, R.E. Thomson, and A.P.M. Santos on Hydroptilidae, A. Prather on Calamoceratidae, K.A. Johanson on Helicopsychidae, F. Muñoz-Quesada on *Wormaldia* and other taxa, R.W. Holzenthal on Trichoptera across the region, many active Brazilian workers and their students (A.R. Calor, A.M.O. Pes, L.L. Dumas, D.M. Takiya, A.P.M. Santos, H. Paprocki, J.L. Nessimian, and others), and W. Wichard on fossil taxa.

## Other checklists, catalogs, and bibliographies

In preparing this catalog several published (or electronic) checklists, catalogs, and bibliographies of the regional and world Trichoptera faunas were consulted. In all cases, the accuracy of the names, citations, or listings in these works were checked and corrected as necessary before inclusion in the present catalog. However, as these former works may be useful to the user of this catalog in further research on the Neotropical fauna or other regional faunas, these works are listed and discussed below, beginning with those covering the world fauna.

The world catalog, *Trichopterorum Catalogus*, Volumes I–XV + Index, 1960–1973b, by F.C.J. Fischer is an indispensable and first source of taxonomic and associated literature pertaining to Trichoptera. The catalog and its supplements cover all literature from 1758 to the end of 1960. As a planned continuation to Fischer’s catalog, A.P. Nimmo published the first volume of *Bibliographica Trichopterorum* (Nimmo 1996) covering the literature from 1961 through 1970. This valuable work includes full literature citations by first author, an index of key words, including species-group and genus-group names, a list of expanded journal titles, and an alphabetical list of secondary authors. In addition, Nimmo compiled an annual bibliography of Trichoptera literature in the series *Current and Selected Bibliographies on Benthic Biology* published by the North American Benthological Society, now the Society for Freshwater Science. A similar bibliography is published in *Braueria*, formerly *Trichoptera Newsletter*, by H. Malicky, Lunz am Zee, Austria. The searchable *World Trichoptera Checklist* is available over the World Wide Web [www.clemson.edu/cafls/departments/esps/database/trichopt/]. Morse (1997b) discussed the format of this checklist. Three other important sources of information with application to the Neotropical fauna are the checklists of Poole (1996), Morse (1993), and Rasmussen and Morse (2014) on the Nearctic fauna, the latter ones also covering Mexico fully or in part. *Zoological Record* and other electronic abstracting services (e.g., *Web of Science*) are of paramount importance in accessing the taxonomic literature. The *Trichoptera Literature Database* [www.trichopteralit.umn.edu] is also useful, especially for searching the literature, for obtaining uniformly formatted titles and journal names, and for downloading copies of the older literature, including the entire *Trichopterorum Catalogus*.

Regional bibliographies of a general nature include those of Flint (1977, 1981d) and Bueno-Soria and Santiago-Fragoso (1982) published in the *Aquatic Biota* series edited by S.H. Hurlbert and others. These works summarize the state of taxonomic knowledge and distribution of the Trichoptera fauna of the respective regions and list all pertinent literature until about the late 1970s and early 1980s. More regionally restricted catalogs, checklists, and faunal works are listed next by country or region:

**Argentina** (Angrisano and Sganga 2007, Angrisano 1995c, Brand 2009, Flint, 1982c, Isa Miranda and Rueda Martín 2014, Mangeaud 1996, Manzo et al., 2014, Muzón et al., 2005); **Brazil** (Almeida and Marinoni 2000, Barcelos-Silva et al. 2012, Blahnik et al. 2004, Calor 2011, Costa et al. 2014, Dumas and Nessimian 2012, Dumas et al., 2009, Dumas et al., 2010, Flint 1992d, Marinoni and Almeida 2000, Moretto and Bispo 2015, Nogueira and Cabette 2011, Oliveira and Froehlich 1997, Paprocki and França 2014, Paprocki et al. 2004, Quinteiro et al. 2014, Speis and Forehlich 2009, Souza et al., 2013a); **Caribbean Islands** (Botosaneanu 2002); **Chile** (Flint 1974e, Rojas 2006, Schmid 1952); **Colombia** (Flint 1991, Medellín et al., 2004, Muñoz-Quesada 2000, Rincón-Hernández 1999); **Costa Rica** (Holzenthal 1988c, Muñoz-Quesada 1999, Springer 2010); **Cuba** (Botosaneanu 1977, 1979, 1980, Flint 1996c, González Lazo et al. 2005, Kumanski 1987, López del Castillo et al., 2004, 2007, Naranjo López and González Lazo 2005); **Curaçao** (Flint 1992a); **Dominican Republic** (Botosaneanu 1995, 1996, Flint and Sykora 2004, Wichard 1987, 2007a, b); **Grenada** (Flint and Sykora 1993); **Guadaloupe** (Botosaneanu 1994a, 2000); **Haiti** (Botosaneanu 1991a); **Hispaniola** (Flint and Pérez-Gelabert 1999, Pérez-Gelabert 2008); **Jamaica** (Botosaneanu and Hyslop 1998, 1999, Botosaneanu and Thomas 2004, Flint 1968a, Malicky 1999); **Lesser Antilles** (Flint 1968b, Malicky 1983); **Martinique** (Botosaneanu and Thomas 2005, Botosaneanu 1988, 1989a); **Mexico** (Banks 1901, Bueno-Soria and Barba-Álvarez 2011, Bueno-Soria and Flint 1978, Bueno-Soria et al. 2005, 2007, Denning 1964, Flint 1967d, Rojas-Ascencio et al. 2002, Ross 1951a [the checklist of Arizona species by Blinn and Ruiter (2005) may contain species that will eventually be discovered in adjacent Mexico]); **Nicaragua** (Chamorro-Lacayo et al., 2007, Maes 1999, Maes and Flint 1988); **North America** (Morse 1993, Poole 1996, Rasmussen and Morse 2014); **Panama** (Aguila 1992, Armitage et al., 2015a, 2015b, Armitage and Cornejo 2015); **Peru** (Flint and Reyes 1991, Flint 1996b); **Puerto Rico** (Flint 1964a); **Suriname** (Flint 1974c); **Trinidad and Tobago** (Botosaneanu and Alkins-Koo 1993, Botosaneanu and Sakal 1992, Flint 1996a); **Uruguay** (Angrisano 1994, 1995b, 1997b); **Venezuela** (including Isla Margarita) (Botosaneanu and Viloria 2002, Botosaneanu 1989b, Flint 1981a).

In addition, several works have been published containing keys for identifying families and genera of the Neotropical fauna. Angrisano (1995d) produced a key to all the families and genera of caddisflies known from South America. Flint provided keys to adults of the Neotropical families (1991) and families and genera (1996b), but the latter does not include all genera from the region covered by this catalog, and excludes genera from the Chilean Subregion. Other important keys to regional faunas include those of Angrisano and Korob (2001), Angrisano and Sganga (2007), Huamantinco and Ortiz (2010), Pes et al. (2005, 2014), Posada-García and Roldán-Pérez (2003), Roldán-Pérez (1988), Ruiter (2000), Springer (2006), and Springer et al. (2010a, b). Finally, Wiggins (1996), Wiggins and Currie (2008), and Morse and Holzenthal (2008) provided various keys for North American families and genera of adults, pupae, and larvae. These works will adequately serve those studying the fauna of much of Mexico, and, to a lesser degree, Central America, but they should be used with caution in those regions south of the United States.

## Purpose of the catalog

The Neotropical Trichoptera fauna is diverse, second only in numbers of species to that of the Oriental fauna (Morse 1997b, 2011). However, despite recent advances in our knowledge of the fauna of Argentina, Brazil, Costa Rica, and Venezuela, for example, and building on investigations since the early 1960s, the fauna is still underexplored and poorly known. New collections or examinations of museum material, especially from unexplored regions (Chamorro and Holzenthal 2010), but even from well-explored areas (Flint 1996a) always yield many new species. Epansive regions, especially in South America have hardly been explored for caddisfly diversity (e.g., Amazon basin, Guyana shield, northern Andes). The likelihood that the Neotropical fauna is substantially more diverse than presently known is very high, even with the addition of over 1,000 new species described since 1999, when the fauna was most recently cataloged (Flint et al., 1999b).

A catalog is a list of nominal species and associated taxonomic and nomenclatural references arranged in a logical, easily accessible format. Catalogs are important tools to anyone requiring knowledge of currently accepted names, including synonyms and distributional data. Because the binomen is usually the starting point of the information storage and retrieval system afforded by the Linnean hierachy, an accurate list of currently accepted species names is essential for anyone needing information about a species, be it for basic or applied research. By accumulating and organizing all the previously published Neotropical Trichoptera taxonomic information into a single, easily accessed source, we hope to facilitate and stimulate further exploration and research on the fauna. Furthermore, we hope that this catalog benefits research beyond Trichoptera systematics, such as ecology, behavior, conservation, and the application of Trichoptera as biological indicators of water quality.

The need for a comprehensive catalog for the Neotropical Trichoptera grew from discussions between Flint and Holzenthal in 1993. These discussions and effort resulted in the 1999 publication by the Ohio Biological Survey of the *Catalog of the Neotropical Caddisflies (Insecta: Trichoptera)* by Flint, Holzenthal and Harris. The need for an update of the “*Catalog*” emerged from discussions between Holzenthal and Calor in 2015, some 15 years after the publication by Flint et al., (1999b). It was also agreed that a new catalog should be published in an “open access” format so that it could be readily available for free download from the Internet. Work on a new catalog began immediately and the result is presented here.

## Definition of the region covered

The current catalog lists names of all species described or recorded from south of the United States, to include all of Mexico, Central and South America, the Greater and Lesser Antilles, and all of the off-shore islands pertaining politically to the countries of the region (although the latter contain very few Trichoptera or have not been surveyed). We realize that this region extends northward beyond the traditional northern boundary of the Neotropical Region to include northern, Nearctic Mexico. However, with regard to Trichoptera, the traditional demarcation between the Neotropical and Nearctic regions, the Isthmus of Tehuantepec, does not apply. There is broad overlap and interdigitation of the Trichoptera faunas of the two regions from at least the southwestern states of the United States through Mexico and Central America until the mountains of eastern Costa Rica and western Panama. Although the region covered by this catalog is artificial with regard to biogeography, it has allowed us to be more objective as to which species to include in the catalog.

## Fossil species

Few fossil species of caddisflies have been discovered in the Neotropical Realm. A single species of the fossil family Necrotaulidae has been described from the Rhaetic (upper Triassic) of western Argentina. The larval cases of a Brazilian Tertiary caddis, provisionally placed in the Limnephilidae, has also been described. Otherwise a number of caddisflies have been described in recent years from Dominican and now Mexican amber (Johanson and Wichard 1996, Lewis 1989, Wells and Wichard 1989, Wichard 1981, 1983a, 1983b, 1985, 1986, 1987, 1989, 1995a, 1995b, 2000, 2007a, b, Wichard et al., 2006). The first two fossils will only be mentioned here, but the amber fossils are treated in their proper systematic position in the catalog.

Wieland (1925) described *Necrotaulius affinis* as the type of his new genus *Tipulitides* placed in the Diptera, Tipulidae. In 1926 he, with the help of Tillyard, who also examined the fossil, transferred the species to *Necrotaulius* and presented a more accurate figure of its wing venation. It is still the only member of the family known from the New World.

Martins-Neto (1989) described a caddisfly case from the Oligocene of São Paulo state in Brazil. The species, *Indusia suguioi*, was placed in the form genus *Indusia*, and suggested that it was a member of the Limnephilidae. The photo of the fossil is too vague to either support or refute this placement.

## Format of the catalog

The catalog is organized alphabetically by family, genus, and species. For each family introductory information, including literature citations, of a general nature is given concerning distribution, diversity, taxonomy, biology, habitat, and knowledge of immature stages, if available. Valid generic names are next presented in **boldface** type, centered on the page, and followed by the author and, in square brackets, the number of currently recognized valid species-group taxa in the region, followed by the number of fossil species. A generic synonymy follows. The currently recognized, valid genus name is followed by its author, date and bibliographic citation of publication, and page number on which the name was formally established. Following this, in square brackets, the type species in its original combination with author and date is presented, along with any synonyms of the type species name, the manner in which the type species was established (e.g., original designation, monotypy, subsequent selection, etc.), and the family in which it was originally described if different from the current family. Other citations containing other important nomenclatural acts, generic revisions, or larval descriptions are next included with annotations contained in square brackets. Generic synonyms follow, in chronological order (oldest names first), and are presented in the same format and with the same information as presented for the valid genus name, with the addition of the citation where the generic synonymy was established. Subgeneric names are presented as generic synonyms and with the same information, but the subgeneric status is so indicated and the citation included. Following the generic synonymy, introductory information on the genus, similar to that presented for the family, is given.

All currently recognized, valid species and subspecies names (specific epithets only), in their current orthography, are then listed in alphabetical order and in ***boldface*** type. Fossil species (and genera) are preceded by the symbol †. In cases where subgenera are used, the subgenus name follows the specific epithet, in parentheses. Each species name is followed by its author, date and bibliographic citation of publication, and page number on which the name was formally established. Following, in square brackets, the type locality is presented, as annotated by us for clarity, but otherwise given as indicated in the original publication, except the country of origin is always listed first. The type depository is then given if known, and so indicated if unknown, according to the institution codes presented below. Sex of the type is presented next, if known, and so indicated if not known. Sex of type is followed (separated by a semicolon) by the sex or stage of any other specimens illustrated and described with the type specimen (these also separated by semicolons). Finally, still in square brackets and separated by a semicolon, the genus of the original combination, or the original orthography of the specific epithet if different from present orthography, is presented. In addition, citations for any significant publications containing redescriptions, lectotype or neotype designations or other nomenclatural acts, systematic revisions, larval descriptions, or new distribution records follow their appropriate species’ entries. Synonyms are indicated in *italics*, preceded by an em dash (—), and listed in chronological order (if more than one) and in their present orthography under the valid species entry. All species-group synonyms are included in the catalog. Information presented for synonyms is the same as presented for the senior name (date and bibliographic citation of the synonymy, sex of type, type depository, genus of original combination or original orthography), but also includes the date and bibliographic reference where the synonymy was established. Lastly, for each species entry the distribution by country, based on published records, is presented.

In addition to original citations and important taxonomic or nomenclatural works, all of the recent and important literature published after 1960 is included in the catalog. However, the extensive bibliographies presented by Fischer (1960-1973a) for the literature prior to 1961 are NOT repeated in this catalog if not of primary importance. The reader is referred to Fischer’s catalog for this additional literature; again, Fischer’s catalog is available from the *Trichoptera Literature Database* (see above). Furthermore, genus-group synonyms are included only if those synonyms pertain to type species described from the region covered by the catalog. Other sources (Fischer 1960-1973a, Poole 1996, Morse 1993, *Trichoptera World Checklist*) should be consulted for a complete list of genus-group synonyms.

All literature cited in the introduction and catalog itself is listed in the References section. The complete title of the journal, book, or other bibliographic source is given to assist the user in obtaining literature. In all cases, the original citation was consulted by the authors in compiling the catalog to ensure accuracy of information or to check date of issue.

The catalog includes all literature known to us up to the end of 2015, as well as several important works published in 2016 and any other literature published after 2015 that has come to our attention. The user is cautioned, however, that we make no claims to have included all the literature published in 2015, and certainly not 2016, but we have done our best to do so. Some literature is not abstracted in *Zoological Record* or *Web of Science* until several years after its date of publication and thus may have been missed. Again, the user should check the appropriate bibliographic sources to ensure complete coverage and overlap by several years the bibliography in this catalog when searching the literature in the future.

The catalog ends with an **Index** that lists all names presented in the catalog and the primary page number where the name occurs. Format of names in the index generally follows that presented in the catalog: valid species and subspecies epithets are presented in bold italics, followed by the current genus in italics; synonyms of species or subspecies names are presented in italics, followed by the current genus in italics. The original orthography of species names, including synonyms, is also indexed, but referred to the species in its current combination and orthography. For subspecies names, the trinomen is also indexed, but referred to the name in combination with the nominotypical name. Homonyms are also indexed, but with the author of the name and date of publication included. Valid genus names are presented in bold, followed by the family in square brackets. Generic synonyms are presented in italics, except that currently recognized subgeneric names are presented in bold italics, both followed by the family in square brackets. Fossil species are followed by the symbol †.

## Trichoptera classification

Since the publication of Flint et al., (1999b), much research has been done on Trichoptera phylogeny and classification, including analyses using molecular data (e.g., Kjer et al., 2001, Holzenthal et al., 2007a, Malm et al., 2013) corroborating or refuting earlier hypotheses (e.g., Ross 1967, Weaver 1984, Weaver and Morse 1986, Wiggins and Wichard 1989, Frania and Wiggins 1997, Ivanov 1997 among others; see Morse 1997a for a review). We have chosen to present taxonomic names in our catalog in alphabetical order for ease of use only. Suborder and family concepts in this catalog follow the classification presented by Holzenthal et al. (2011) as presented below (with genera and subgenera listed in alphabetical order). Only subgenera with representation in the region covered by this catalog are included. Publications containing hypotheses on relationships within families are presented, where known, under the family treatments. The *Trichoptera World Checklist* can be consulted for additional details on classification below the family.

Order TrichopteraKirby, 1813

Suborder AnnulipalpiaMartynov, 1924

Superfamily PhilopotamoideaStephens, 1829

Family **Philopotamidae**Stephens, 1829

*Alterosa*Blahnik, 2005

*Chimarra*Stephens, 1829

*Chimarra*Stephens, 1829

*Chimarrita*Blahnik, 1997

*Curgia*Walker, 1860

*Otarrha*Blahnik, 2002

*Chimarrhodella*Lestage, 1925

*Sortosa* Navás, 1918

*Wormaldia*McLachlan, 1865

Family **Stenopsychidae**Martynov, 1924

*Pseudostenopsyche*Döhler, 1915

Superfamily PsychomyioideaWalker, 1852

Family **Ecnomidae**Ulmer, 1903

*Austrotinodes* Schmid, 1955

*Chilocentropus* Navás, 1934

Family **Polycentropodidae**Ulmer, 1903

*Cernotina* Ross, 1938

*Cyrnellus* Banks, 1913

*Nyctiophylax*Brauer, 1865

*Polycentropus*Curtis, 1835

*Polyplectropus* Ulmer, 1905

Family **Pseudoneureclipsidae**Ulmer, 1913

*Antillopsyche*Banks, 1941

Family **Psychomyiidae**Walker, 1852

*Tinodes*Curtis, 1834

Family **Xiphocentronidae** Ross, 1949

*Cnodocentron*Schmid, 1982

*Caenocentron*Schmid, 1982

*Machairocentron*Schmid, 1982

*Xiphocentron*Brauer, 1870

*Antillotrichia*Banks, 1941

*Glyphocentron*Schmid, 1982

*Rhamphocentron*Schmid, 1982

*Sphagocentron*Schmid, 1982

*Xiphocentron*Brauer, 1870

Superfamily HydropsychoideaCurtis, 1835

Family **Hydropsychidae**Curtis, 1835

*Blepharopus*Kolenati, 1859

*Calosopsyche*Ross and Unzicker, 1977

*Centromacronema* Ulmer, 1905

*Cheumatopsyche*Wallengren, 1891

*Diplectrona* Westwood, 1839

*Hydropsyche*Pictet, 1834

*Ceratopsyche*Ross and Unzicker, 1977

*Hydropsyche*Pictet, 1834

*Leptonema*Guérin-Méneville, 1843

*Macronema*Pictet, 1836

*Macrostemum*Kolenati, 1859

† *Palaehydropsyche*Wichard, 1986

*Plectromacronema*Ulmer, 1906

*Plectropsyche*Ross, 1947

*Pseudomacronema* Ulmer, 1905

*Smicridea*McLachlan, 1871

*Rhyacophylax* Müller, 1879

*Smicridea*McLachlan, 1871

*Streptospyche*Ross and Unzicker, 1977

*Synoestropsis* Ulmer, 1905

Suborder IntegripalpiaMartynov, 1924

Superfamily GlossosomatoideaWallengren, 1891

Family **Glossosomatidae**Wallengren, 1891

*Canoptila* Mosely, 1939

*Cariboptila* Flint, 1964

*Culoptila*Mosely, 1954

*Glossosoma*Curtis, 1834

*Itauara*Müller, 1888

*Mastigoptila* Flint, 1967

*Merionoptila*Schmid, 1959

*Mortoniella*Ulmer, 1906

*Protoptila* Banks, 1904

*Scotiotrichia* Mosely, 1934

*Tolhuaca*Schmid, 1964

Superfamily Hydroptiloidea Stephens, 1836

Family **Hydroptilidae** Stephens, 1836

*Acostatrichia* Mosely, 1939

*Alisotrichia* Flint, 1964

*Anchitrichia*Flint, 1970

*Angrisanoia*Özdikmen, 2008

*Ascotrichia* Flint, 1983

*Betrichia* Mosely, 1939

*Bredinia* Flint, 1968

*Byrsopteryx* Flint, 1981

*Celaenotrichia* Mosely, 1934

*Cerasmatrichia* Flint, Harris and Botosaneanu, 1994

*Ceratotrichia* Flint, 1992

*Costatrichia*Mosely, 1937

*Dicaminus* Müller, 1879

*Flintiella* Angrisano, 1995

*Hydroptila*Dalman, 1819

*Ithytrichia*Eaton, 1873

*Kumanskiella*Harris and Flint, 1992

*Leucotrichia* Mosely, 1934

*Mayatrichia*Mosely, 1937

*Mejicanotrichia*Harris and Holzenthal, 1997

*Metrichia* Ross, 1938

*Neotrichia*Morton, 1905

*Nothotrichia* Flint, 1967

*Ochrotrichia* Mosely, 1934

*Orinocotrichia* Harris, Flint and Holzenthal, 2002

*Orthotrichia*Eaton, 1873

*Oxyethira*Eaton, 1873

*Argyrobothrus*Barnard, 1934

*Dactylotrichia*Kelley, 1984

*Dampfitrichia*Mosely, 1937

*Kelleyella*Özdikmen, 2007

*Loxotrichia*Mosely, 1937

*Oxytrichia* Mosely, 1939

*Tanytrichia*Kelley, 1984

*Peltopsyche* Müller, 1879

*Ragatrichia*Oláh and Johanson, 2011

*Rhyacopsyche* Müller, 1879

*Scelobotrichia*Harris and Bueno-Soria, 1993

*Taraxitrichia*Flint and Harris, 1991

*Tizatetrichia* Harris, Flint and Holzenthal, 2002

*Tricholeiochiton* Kloets and Hinks, 1944

*Tupiniquintrichia* Santos, Nessimian and Takiya, 2016

*Zumatrichia*Mosely, 1937

Superfamily Rhyacophiloidea Stephens, 1836

Family **Hydrobiosidae** Ulmer, 1905

*Amphichorema*Schmid, 1989

*Androchorema*Flint, 1979

*Apatanodes* Navás, 1934

*Atopsyche*Banks, 1905

*Atopsaura*Ross, 1953

*Atopsyche*Banks, 1905

*Dolochorema* Banks, 1913

*Australobiosis* Schmid, 1958

*Cailloma*Ross and King, 1951

*Clavichorema* Schmid, 1955

*Heterochorema*Schmid, 1989

*Iguazu*Ross and King, 1951

*Isochorema*Schmid, 1989

*Metachorema*Schmid, 1957

*Microchorema* Schmid, 1955

*Neoatopsyche* Schmid, 1955

*Neochorema*Schmid, 1957

*Neopsilochorema* Schmid, 1955

*Nolganema* Navás, 1934

*Parachorema*Schmid, 1957

*Pomphochorema* Flint, 1969

*Pseudoradema* Schmid, 1955

*Rheochorema* Schmid, 1955

*Schajovskoya*Flint, 1979

*Stenochorema* Schmid, 1955

Family **Rhyacophilidae** Stephens, 1836

*Rhyacophila*Pictet, 1834

Infraorder BrevitentoriaWeaver, 1984

Superfamily LeptoceroideaLeach, 1815

Family **Atriplectididae**Neboiss, 1977

*Neoatriplectides*Holzenthal, 1997

Family **Calamoceratidae** Ulmer, 1905

*Banyallarga* Navás, 1916

*Banyallarga* Navás, 1916

*Histricoverpa*Prather, 2004

*Phylloicus* Müller, 1880

Family **Leptoceridae**Leach, 1815

*Achoropsyche*Holzenthal, 1984

*Amazonatolica*Holzenthal and Pes, 2004

*Amphoropsyche*Holzenthal, 1985

*Atanatolica* Mosely, 1936

*Brachysetodes* Schmid, 1955

*Grumichella* Müller, 1879

*Hudsonema* Mosely, 1936

*Mystacides*Berthold, 1827

*Nectopsyche* Müller, 1879

*Neoathripsodes*Holzenthal, 1989

*Notalina* Mosely, 1936

*Neonotalina* Holzenthal, 1986

*Oecetis*McLachlan, 1877

*Osflintia*Calor and Holzenthal, 2008

*Setodes*Rambur, 1842

*Triaenodes*McLachlan, 1865

*Triplectides*Kolenati, 1859

Family **Odontoceridae**Wallengren, 1891

*Anastomoneura* Huamantinco and Nessimian, 2004

*Barypenthus*Burmeister, 1839

*Marilia* Müller, 1880

Family **Philorheithridae** Mosely, 1936

*Mystacopsyche* Schmid, 1955

*Psilopsyche* Ulmer, 1907

Superfamily Sericostomatoidea Stephens, 1836

Family **Anomalopsychidae** Flint, 1981

*Anomalopsyche* Flint, 1967

*Contulma* Flint, 1969

Family **Helicophidae** Mosely, 1953

*Alloecentrellodes*Flint, 1979

*Austrocentrus*Schmid, 1964

*Eosericostoma* Schmid, 1955

*Microthremma* Schmid, 1955

*Pseudosericostoma*Schmid, 1957

Family **Helicopsychidae**Ulmer, 1906

*Helicopsyche*Siebold, 1856

*Cochliopsche*Müller, 1885

*Feropsyche*Johanson, 1998

Family **Sericostomatidae** Stephens, 1836

*Chiloecia* Navás, 1930

*Grumicha* Müller, 1879

*Gumaga*Tsuda, 1938

*Myotrichia* Schmid, 1955

*Notidobiella* Schmid, 1955

*Parasericostoma*Schmid, 1957

Superfamily TasimioideaRiek, 1968

Family **Tasimiidae**Riek, 1968

*Charadropsyche* Flint, 1969

*Trichovespula* Schmid, 1955

Infraorder PlenitentoriaWeaver, 1984

Family **Kokiriidae**McFarlane, 1964

*Pangullia* Navás, 1934

Superfamily LimnephiloideaKolenati, 1848

Family **Limnephilidae**Kolenati, 1848

*Anomalocosmoecus*Schmid, 1957

*Antarctoecia* Ulmer, 1907

*Austrocosmoecus* Schmid, 1955

*Clistoronia*Banks, 1916

*Hesperophylax*Banks, 1916

*Limnephilus*Leach, 1815

*Metacosmoecus* Schmid, 1955

*Monocosmoecus*Ulmer, 1906

*Platycosmoecus*Schmid, 1964

*Verger* Navás, 1918

Superfamily PhryganeoideaLeach, 1815

Family **Lepidostomatidae**Ulmer, 1903

*Lepidostoma*Rambur, 1842

## List of type depositories

AMNH American Museum of Natural History, New York, New York, USA 


ASL  Academy of Sciences, St. Petersburg, Russia 


BMNH Natural History Museum, London, England 


CAS California Academy of Sciences, San Francisco, California, USA 


CMNH Carnegie Museum of Natural History, Pittsburgh, Pennsylvania, USA 


CNC  Canadian National Collection, Ottawa, Ontario, Canada 


CNIN  Colección Nacional de Insectos, Instituto de Biología, Universidad Nacional Autonoma de México, Mexico City, Mexico, formerly IBUNAN 


**Collection** Apollinaris he worked in Colombia, sent material to Navás, material presumed lost

**Collection** Malicky private collection, Hans Malicky, Lunz am See, Austria

**Collection** Martynov private collection in Warsaw, material not in ASL, presumed lost

**Collection** Navás some material transferred to MZBS and survived, remainder mostly lost

**Collection** Poinar collection of George O. Poinar, Oregon State University, Corvallis, Oregon, USA

**Collection** Wichard private collection, Wilfried Wichard, Bonn, Germany

CU Cornell University, Ithaca, New York, USA 


CZNC  Coleção Zoológica Norte Capixaba, Universidade Federal do Espírito Santo, São Mateus, Brazil 


CZMA  Coleção Zoológica do Maranhão, Universidade Estadual do Maranhão, Caxias, Maranhão, Brazil 


DEI  Institut für Pflanzenschutzforschung (former Deutsches Entomologisches Institut), Eberswalde, Germany 


DZRJ  Coleção Entomológica Prof. José Alfredo Pinheiro Dutra, Departamento de Zoologia, Universidade Federal do Rio de Janeiro, Rio de Janeiro, Brazil 


DZUP  Coleção de Entomologia Padre Jesus Santiago Moure, Departamento de Zoologia, Universidade Federal do Paraná, Curitiba, Paraná, Brazil 


FHCU  Facultad de Humanidades y Ciencias (Departamento de Artropodos), Univeridad de la Republica, Montevideo, Uruguay 


FMNH Field Museum of Natural History, Chicago, Illinois, USA 


FSCA  Florida State Collection of Arthropods, Gainesville, Florida, USA 


GPIMH  Geological-Palaeontological Institute and Museum, University of Hamburg, Germany 


HNHM Hungarian Natural History Museum, Budapest, Hungary 


IBN  Instituto de Biodiverdidad Neotropical, Facultad de Ciencias Naturales e Instituto Miguel Lillo, Tucumán, Argentina 


IBUNAM  now CNIN



IESHC Instituto de Ecología y Sistemática, Havana, Cuba 


INBIO Instituto Nacional de Biodiversidad, Santo Dominge de Heredia, Costa Rica 


IHNEC  Museo de Paleontología, Instituto de Historia Natural y Ecología de Chiapas, Tuxtla Gutiérrez, Chiapas, Mexico 


IML Instituto Miguel Lillo, Tucumán, Argentina 


INHS Illinois Natural History Survey, Champaign, Illinois, USA 


INPA Instituto Nacional de Pesquisas da Amazonia, Manaus, Brazil 


IRSNB Institut Royal des Sciences Naturelles de Belgique, Brussels, Belgium 


ISMA  Instituto San Miguel, Buenos Aires, Argentina 


IZAM  Instituto de Zoología Agrícola, Maracay, Venezuela 


KMUL  Karl-Marx-University, Leipzig, Germany 


LACM  Los Angeles County Museum of Natural History, California, USA 


MACN Museo Argentino de Ciencias Naturales “Bernardino Rivadavia”, Buenos Aires, Argentina 


MCZ Museum of Comparative Zoology, Harvard University, Cambridge, Massachusetts, USA 


MHNG Muséum d’Histoire Naturelle, Geneva, Switzerland 


MHNJP Museo de Historia Natural “Javier Prado”, Universidad Nacional Mayor de San Marcos Lima, Peru 


MIUP Universidad de Panamá Museo de Invertebrados, Panama 


MNHNP Muséum National d’Histoire Naturelle, Paris, France 


MNHNS Museo Nacional de Historia Natural, Santiago, Chile 


MNRJ Museu Nacional, Universidade Federal do Rio de Janeiro, Rio de Janeiro, Brazil [F. Müller material. Müller did not designate types nor indicate any depository for his material, but the material he examined, primarily larval cases, is in the MNRJ (A.P.M. Santos, personal communication)]. 


MZBS Museo de Zoologia, Barcelona, Spain 


MZUSP  Museu de Zooologia, Universidade de São Paulo, São Paulo, Brazil 


NMB Naturhistorisches Museum, Basel, Switzerland 


NMNH National Museum of Natural History, Washington, DC, USA 


NMSB National Museum, Sofia, Bulgaria 


NMW  Naturhistorisches Museum, Vienna, Austria 


NRS Naturhistoriska Riksmuseet, Stockholm, Sweden 


OPC  Jánus Oláh private collection, Debrecen, Hungary, presently under protection of Hungarian Natural History Museum, Budapest, Hungary 


PAN Polish Academy of Sciences, Warsaw, Poland 


PSUC Pennsylvania State University, Frost Entomological Museum, University Park, Pennsylvania, USA 


RNH  Rijksmuseum van Natuurlijke Historie, Leiden, Netherlands 


SDMNH  San Diego Museum of Natural History, San Diego, California, USA 


SEMC  Snow Entomological Museum Collection, University of Kansas, Lawrence, Kansas, USA 


SMNS  Staatlichen Museum für Naturkunde, Stuttgart, Germany 


UASC Museo de Historia Natural Noel Kempff Mercado, Santa Cruz de la Sierra, Bolivia 


UCB University of California, Berkeley, California, USA 


UCD University of California, Davis, California, USA 


UChS Universidad de Chile (Investigaciones Entomológicas), Santiago, Chile 


UMQ University of Montreal, Montreal, Quebec, Canada 


UMSP University of Minnesota, St. Paul, Minnesota, USA 


UNLP  Museo de la Plata, Universdad Nacional de La Plata, La Plata, Argentina 


UZMC  Universitetets Zoologiske Museet, Copenhagen, Denmark 


WSU Washington State University, Pullman, Washington, USA 


ZIUH Zoologisches Institut der Universität, Halle an der Salle, Germany 


ZMHU  Zoologisches Museum, Museum für Naturkunde der Humboldt-Universität, Berlin, Germany 


ZMUA  Zoölogische Museum, Universiteit van Amsterdam, Netherlands 


ZSM  Zoologischen Staatssamlung München, Munich, Germany 


ZSZMH  Zoologische Staatsinstitut und Zoologisches Museum, Hamburg, Germany 


## Catalog

### Family Anomalopsychidae

The family Anomalopsychidae was established by Flint (1981c) for two Chilean species previously placed in the Sericostomatidae: *Contulma cranifer* Flint and *Anomalopsyche minuta* (Schmid). Two additional *Contulma* species were described by Holzenthal and Flint (in Flint 1991) from Colombia. Since then, 24 new species have been described from Costa Rica, Colombia, Ecuador, Peru, Bolivia, and Brazil (Holzenthal and Flint 1995, Holzenthal and Robertson 2006, Jardim and Nessimian 2011, Holzenthal and Ríos-Touma 2012).

Flint (1981c) described the immature stages of *Anomalopsyche* and those of several species of *Contulma* were described by Holzenthal and Flint (1995) and Holzenthal and Ríos-Touma (2012). Larvae of both genera build cases of sand grains and inhabit small streams in forested areas or at high elevations.

#### Genus *Anomalopsyche* Flint [1]

*Anomalopsyche* Flint, 1967a:66 [Type species: *Anomalopsyche ocellata* Flint, 1967a = *Myotrichia minuta* Schmid, 1957, original designation; in Sericostomatidae]. —Flint, 1981c:75 [to Anomalopsychidae].

A single species is known from Chile. Larvae and pupae were described by Flint (1981c). They build slightly curved, tapered, cylindrical cases of sand grains. The immature stages are found in spring runs, in waterfalls, and hygropetric habitats, often in aquatic moss.

***minuta*** (Schmid), 1957:392 [Type locality: Chile, Ñuble, Tregualemu, NMNH; ♂; in *Myotrichia*]. —Flint, 1967a:66 [to *Anomalopsyche*]. —Flint, 1974e:91 [checklist]. —Flint, 1981c:75 [♂; ♀; larva; pupa; redescription].

—*ocellata* Flint, 1967a:66 [Type locality: Chile, Valdivia, Punucapa, near Valdivia; NMNH; ♂]. —Flint, 1974e:84, 91 [to synonymy].

**Distribution.** Chile.

#### Genus *Contulma* Flint [27]

*Contulma* Flint, 1969b:513 [Type species: *Contulma cranifer* Flint, 1969b, original designation; in Sericostomatidae]. —Flint, 1981c:82 [to Anomalopsychidae]. —Holzenthal and Flint, 1995:1 [♂; ♀; larva; pupa; phylogeny].

The 27 known species of *Contulma* range in distribution from Costa Rica, through the Andes of Colombia, Ecuador, Peru, and Bolivia, to Chile and the mountains of southeastern Brazil. Certainly, many more undescribed species await discovery.

*Contulma* species are generally found associated with the spray and splash zones of waterfalls, small first order streams, and seeps in lush, montane forests. Several species have been taken from small streams flowing through the páramo. Holzenthal and Ríos-Touma (2012) described the life history stages and biology of an uncommon species living above 3,800 m in the Ecuadorian páramo. The larvae fed on diatoms and were univoltine with continuous larval growth. In some Colombian streams between 2,500-2,900 m a.s.l., *Contulma* larvae were a dominant component of the benthic fauna (Medellín et al., 2004). Adults of species living in high elevations are not readily attracted to lights because of cold nighttime temperatures; those of the Ecuadorian species *Contulma paluguillensis* were collected in sticky traps as well as Malaise and emergence traps.

***adamsae*** Holzenthal and Flint, 1995:11 [Type locality: Peru, Cuzco, Paucartampo, nr. park entrance station, nr. km 106, seeps; NMNH; ♂; ♀]. —Flint, 1996b:429 [distribution].

**Distribution.** Peru.

***bacula*** Holzenthal and Flint, 1995:11 [Type locality: Ecuador, Napo, 1 mi E of Papallacta; NMNH; ♂]. —Medellín et al., 2004:201 [distribution; biology].

**Distribution.** Colombia, Ecuador.

***boliviensis*** Holzenthal and Robertson, 2006:50 [Type locality: Bolivia, Santa Cruz, Parque Nacional Amboró, 17°50'15"S, 64°23'29"W, el. 2030 m; UASC; ♂].

**Distribution.** Bolivia.

***caldensis*** Holzenthal and Flint, 1995:12 [Type locality: Colombia, Caldas, 1.1 km E Termales de Ruíz; NMNH; ♂]. —Muñoz-Quesada, 2000:274 [checklist].

**Distribution.** Colombia.

***cataracta*** Holzenthal and Flint, 1995:12 [Type locality: Ecuador, Napo, Río Maspa Chico, 2 km W Cuyuja; NMNH; ♂].

**Distribution.** Ecuador.

***colombiensis*** Holzenthal and Flint, in Flint, 1991:106 [Type locality: Colombia, Dpto. Antioquia, 12 km N Fredonia, road to Medellín; NMNH; ♂]. —Holzenthal and Flint, 1995:14 [♂; ♀; redescription; distribution]. —Muñoz-Quesada, 2000:274 [checklist].

**Distribution.** Colombia.

***costaricensis*** Holzenthal and Flint, 1995:14 [Type locality: Costa Rica, Cartago, Reserva Tapantí, unnamed tribs. (Quebrada Palmitos and falls), ca. 9 km (road) NW tunnel, 9.72°N, 83.78°W; NMNH; ♂].

**Distribution.** Costa Rica.

***cranifer*** Flint, 1969b:513 [Type locality: Chile, Malleco, Río Manzanares, near Purén; NMNH; ♂]. —Flint, 1974e:91 [checklist]. —Flint, 1981c:82 [to Anomalopsychidae]. —Holzenthal and Flint, 1995:14 [♂; ♀; redescription].

**Distribution.** Chile.

***echinata*** Holzenthal and Flint, 1995:15 [Type locality: Colombia, Caldas, 5 km W Termales de Ruíz; NMNH; ♂; ♀]. —Muñoz-Quesada, 2000:274 [checklist].

**Distribution.** Colombia.

***ecuadorensis*** Holzenthal and Flint, 1995:16 [Type locality: Ecuador, Imbabura, Otavalo/Apuila; NMNH; ♂; ♀].

**Distribution.** Ecuador.

***fluminensis*** Holzenthal and Robertson, 2006:52 [Type locality: Brazil, Rio de Janeiro, Rio Macaé, Macaé da Cima, 22°23'41"S, 42°30'08"W, el. 1000 m; MZUSP; ♂]. —Dumas et al., 2009:371 [distribution]. —Paprocki and França, 2014:3 [checklist].

**Distribution.** Brazil.

***inornata*** Holzenthal and Flint, 1995:16 [Type locality: Colombia, Caldas, 5 km W Termales de Ruíz; NMNH; ♂]. —Muñoz-Quesada, 2000:274 [checklist].

**Distribution.** Colombia.

***lanceolata*** Holzenthal and Flint, 1995:17 [Type locality: Ecuador, Napo, Baeza (72 km E), in seep at waterfall; NMNH; ♂].

**Distribution.** Ecuador.

***meloi*** Holzenthal and Robertson, 2006:54 [Type locality: Brazil, São Paulo, Estação Biológica de Boracéia, Rio Venerando, 23°39'11"S, 45°53'25"W, el. 850 m; MZUSP; ♂]. —Calor, 2011:319 [checklist]. —Paprocki and França, 2014:3 [checklist].

**Distribution.** Brazil.

***nevada*** Holzenthal and Flint, 1995:17 [Type locality: Colombia, Caldas, 0.7 km S entrance P.N. Los Nevados; NMNH; ♂; ♀; larva]. —Muñoz-Quesada, 2000:274 [checklist].

**Distribution.** Colombia.

***paluguillensis*** Holzenthal and Ríos-Touma, 2012:444 [Type locality: Ecuador, Pichincha, Reserva Paluguillo, Quebrada Saltana, 0°19'1.80"S, 78°13'8.8"W, 3848 m; UMSP; ♂; ♀; larva; pupa; biology].

**Distribution.** Ecuador.

***papallacta*** Holzenthal and Flint, 1995:17 [Type locality: Ecuador, Napo, 1 mi E of Papallacta; NMNH; ♂].

**Distribution.** Ecuador.

***penai*** Holzenthal and Flint, 1995:18 [Type locality: Ecuador, Zamora-Chinchipe, 30 km E Loja; NMNH; ♂; ♀; larva]. —Muñoz-Quesada, 2000:274 [checklist].

**Distribution.** Colombia, Ecuador.

***sana*** Jardim and Nessimian, 2011:226 [Type locality: Brazil, Rio de Janeiro: Macaé municipality, Sana, Córrego da Ilha, 22°20'41.8"S, 42°11'03.7"W, 381 m; DZRJ; ♂]. —Paprocki and França, 2014:3 [checklist].

**Distribution.** Brazil.

***sancta*** Holzenthal and Flint, 1995:19 [Type locality: Costa Rica, Alajuela, Quebrada Virgencita, 10.2 km S Bajos del Toro, 10.168°N, 84.326°W; NMNH; ♂].

**Distribution.** Costa Rica.

***spinosa*** Holzenthal and Flint, in Flint, 1991:106 [Type locality: Colombia, Dpto. Antioquia, Quebrada La Iguaná, 17 km NW Medellín, on road to San Jerónimo; NMNH; ♂]. —Holzenthal and Flint, 1995:19 [♂; ♀; larva; distribution]. —Muñoz-Quesada, 2000:274 [checklist]. —Medellín et al., 2004:200 [distribution; biology].

**Distribution.** Colombia, Ecuador.

***talamanca*** Holzenthal and Flint, 1995:21 [Type locality: Costa Rica, Puntarenas, Río Jaba at rock quarry, 1.4 km (air) W Las Cruces, 8.79°N, 82.97°W; NMNH; ♂; ♀].

**Distribution.** Costa Rica.

***tapanti*** Holzenthal and Flint, 1995:21 [Type locality: Costa Rica, Cartago, Reserva Tapantí, unnamed trib. (Quebrada Palmitos and falls), ca. 9 km (road) NW tunnel, 9.72°N, 83.78°W; NMNH; ♂; ♀].

**Distribution.** Costa Rica.

***tica*** Holzenthal and Flint, 1995:22 [Type locality: Costa Rica, Puntarenas, Río Bellavista, ca. 1.5 km NW Las Alturas, 8.951°N, 82.846°W; NMNH; ♂].

**Distribution.** Costa Rica.

***tijuca*** Holzenthal and Flint, 1995:22 [Type locality: Brazil, Rio de Janeiro, Parque Nacional Tijuca, Repressa dos Ciganos; MZUSP (on indefinite loan to NMNH); ♂; ♀; probable larva]. —Dumas et al., 2009:371 [distribution]. Paprocki et al., 2004:5 [checklist]. —Dumas and Nessimian, 2012:8 [distribution]. —Paprocki and França, 2014:3 [checklist].

**Distribution.** Brazil.

***tripui*** Holzenthal and Robertson, 2006:56 [Type locality: Brazil, Minas Gerais, Estação Ecológica do Tripuí, Córrego Tripuí, 20°23'22"S, 43°32'32"W, el. 1070 m; MZUSP; ♂]. —Calor, 2011:319 [distribution]. —Dumas et al., 2009:371 [distribution]. —Paprocki and França, 2014:3 [checklist].

**Distribution.** Brazil.

***valverdei*** Holzenthal and Flint, 1995:22 [Type locality: Costa Rica, Cartago, Reserva Tapantí, waterfall, ca. 1 km (road) NW tunnel, 9.69°N, 83.76°W; NMNH; ♂; ♀; larva].

**Distribution.** Costa Rica.

### Family Atriplectididae

Neboiss (1978) established a new family, Atriplectididae, for the Australian species *Atriplectides dubius* Mosely and at the same time transferred *Hughscottiella auricapilla* Ulmer, from the Seychelles, to the family. The larvae of both genera were described in the same year (Marlier 1978, Neboiss 1978). Roback (1966) described an unusual caddisfly larva from the Río Bella, near Tingo Maria, Peru that he was unable to place in a known family at the time, but it, too, belongs to the Atriplectididae. The larvae of all three are unusual within the Trichoptera in that the head, pro- and mesonota are narrow, elongate and retractile. Holzenthal (1997) reviewed the family and described the first Neotropical species, placing it in a new genus, *Neoatriplectides*. The unusual larval morphology is apparently an adaptation that allows the larva to feed internally in small dead arthropods found in the stream (Malicky 1997).

#### Genus *Neoatriplectides* Holzenthal [2]

*Neoatriplectides* Holzenthal, 1997:157 [Type species: *Neoatriplectides froehlichi* Holzenthal, 1997].

Unknown family 1, Roback, 1966:256 [larva only]. —Holzenthal, 1997:157 [to synonymy].

Only two species are known in the genus, with definitive records of adults of one from only Peru and Bolivia, and the other from southeastern Brazil. Larval only records are known from Colombia and Ecuador, as well as Peru The larvae were described by Roback (1966) and Holzenthal (1997). Larvae are found on sandy substrates or among leaf liter in shallow, lateral pools of small streams (Dumas and Nessimian 2008). Posada-García and Roldán-Pérez (2003) recorded a single larva from a small stream containing bryophytes at an elevation of 2,800 m in northwestern Colombia, and Villarreal-Grisales and García-Cárdenas (2013) recorded larvae from Quindío, Colombia, in streams at about 3000 m elevation. These larvae were found on rocks in the center of the streams where the current was fastest. Villada-Bedoya et al. (2015) also recorded a larva from Colombia. These larval records and the one from Ecuador (Holzenthal 1997) are here tentatively listed under *Neoatriplectides froehlichi*, but adults must be collected or reared to confirm their identity.

***desiderata*** Dumas and Nessimian, 2008:64 [Type localiy: Brazil, Minas Gerais, Itamonte, Rio Aiuruoca, 22°20'56.9"S, 44°41'37.9"W, 1860 m; DZRJ; ♂; pupa]. —Dumas et al., 2009:367 [distribution]. —Dumas and Nessimian, 2012:8 [checklist]. —Paprocki and França, 2014:4 [checklist].

—*Neoatriplectides* sp., Holzenthal, 1997:160 [larva; case]. —Dumas and Nessimian, 2008:64 [to synonymy].

**Distribution.** Brazil.

***froehlichi*** Holzenthal, 1997:159 [Type locality: Peru, Cuzco, Paucartambo, Puente San Pedro, ca. 50 km NW Pilcopata, MHNJP (temporarily at NMNH); ♂; /female]. —Muñoz-Quesada 2000:272 [probable distribution]. —Posada-García and Roldán-Pérez, 2003:175 [probable distribution]. —Villarreal-Grisales and García-Cárdenas, 2013:261 [probable distribution]. —Villada-Bedoya et al., 2015:369 [probable distribution].

—Probable n. gen., n. sp., Flint, 1996b:423 [teneral ♂]. —Holzenthal, 1997:159 [described as new species].

**Distribution.** Bolivia, Colombia [probable], Ecuador, Peru.

### Family Calamoceratidae

This is a small, but cosmopolitan family of seven genera and about 200 species, most of which are tropical. Only two genera, *Banyallarga* and *Phylloicus*, are to be found in the Neotropics, with 17 and 58 species respectively, including one fossil species from Domincan amber.

Adults are more diurnal in their activity than most Trichoptera. The immature stages and cases of several species of *Phylloicus* have been described: Flint (1964a), Roldán Pérez (1988), Wiggins (1996), Bowles and Flint (1997), Prather (2003), Huamantinco et al. (2005), Quinteiro et al. (2011). The same stages are also known for *Banyallarga argentinica* (Flint and Angrisano 1985). Larvae of the Neotropical species are found in standing, backwater areas of streams and rivers where they feed on leaf detritus; they are often very abundant.

#### Genus *Banyallarga* Navás [17]

*Banyallarga* Navás, 1916b:78 [Type species: *Banyallarga testacea* Navás, 1916b, original designation]. —Fischer, 1963:175 [in Hydropsychidae: Macronematinae]. —Botosaneanu and Flint, 1982:24 [larva; as *Phylloicus*]. —Flint, 1983a:77 [to Calamoceratidae]. —Flint and Angrisano, 1985:688 [larva; pupa; distinguished from *Phylloicus*]. —Prather, 2004:3, 11 [revision; ♂; ♀; key to species]. —Rueda Martín, 2013:328 [case; differentiation of larvae from *Phylloicus*].

*Histricoverpa* Prather, 2004:22 [Type species: *Ganonema molliculum* McLachlan, 1871, original designation; as subgenus of *Banyallarga*; ♂; ♀; revision; key to species].

This genus of 17 known species is endemic to the Neotropics, being found from Nicaragua to Argentina. Adults exhibit a preference for flying and swarming during the day, and only rarely are attracted to collecting lights.

The larvae are found in slowly flowing areas of small streams on sandy-stony bottoms or among vegetation (Flint and Angrisano 1985). Larvae build tubular cases primarily of mineral fragments with some plant matter incorporated or flat cases of leaf framents, similar to those of *Phylloicus* (Rueda Martín 2013). They appear to be omnivorous.

***acutiterga*** (*Histricoverpa*) (Denning and Hogue), in Denning et al., 1983:188 [Type locality: Costa Rica, San José Province, Motel Prado, San Isidro del General; LACM; ♂; in *Murielia*]. —Holzenthal, 1988c:71 [distribution]. —Flint et al., 1999a:73 [to *Banyallarga*]. —Prather, 2004:23 [♂; ♀; redescription; to *Histricoverpa*].

**Distribution.** Costa Rica.

***argentinica*** (*Banyallarga*) Flint, 1983a:77 [Type locality: Argentina, Pcia. Salta, Cañada la Gotera, Rt. 59, km 23.5; NMNH; ♂]. —Flint and Angrisano, 1985:691 [larva; pupa; biology]. —Mangeaud, 1996:154 [distribution]. —Cohen, 2004:74 [list of type material, distribution]. —Prather, 2004:12 [♂; ♀; distribution]. —Rueda Martín, 2013:326 [♂; case]. —Isa Miranda and Rueda Martín, 2014:199 [distribution].

**Distribution.** Argentina, Bolivia, Peru.

***columbiana*** (*Banyallarga*) (Navás), 1934a:174 [Type locality: Colombia, Santander, Pamplona; MNHNP; original description implies ♀; but type is ♂; in *Anisocentropus*]. —Flint, 1983a:77 [to *Banyallarga*]. —Muñoz-Quesada, 2000:274 [checklist]. —Prather, 2004:12 [♂; redescription].

**Distribution.** Colombia.

***echinata*** (*Histricoverpa*) Prather, 2004:24 [Type locality: Peru, Madre de Dios, Manu Biosphere Res[serve], Pakitza Bio[logical] Sta[tion], 11°56'00"S, 71°18'00"W, el. 350 m; NMNH; ♂; ♀].

—“n. sp. 1” Flint, 1996b:424. —Prather, 2004:24 [as synonymy].

**Distribution.** Peru.

***fortuna*** (*Histricoverpa*) (Resh), in Denning et al., 1983:190 [Type locality: Panama, Rio Chiriqui at Fortuna; UCB; ♂; in *Murielia*]. —Aguila, 1992:543 [distribution]. —Flint et al., 1999a:73 [to *Banyallarga*]. —Prather, 2004:26 [♂; ♀; redescription; to *Histricoverpa*]. —Armitage et al., 2015b:8 [checklist]. —Armitage and Cornejo, 2015:197 [checklist].

—*undescribed* genus, undescribed species “A” McElravy et al., 1981:153. —Denning et al., 1983:190 [to synonymy].

**Distribution.** Costa Rica, Panama.

***loxana*** (*Banyallarga*) (Navás), 1934a:173 [Type locality: Ecuador, Loja; MNHNP; original description implies ♂; but the type is female; in *Phylloicus*]. —Flint, 1983a:77 [to *Banyallarga*]. —Flint, 1996b:424 [distribution]. —Cohen, 2004:77 [distribution]. —Prather, 2004:15 [♂; ♀; redescription]. —Rueda Martín, 2013:326 [♂]. —Isa Miranda and Rueda Martín, 2014:199 [distribution].

**Distribution.** Argentina, Bolivia, Ecuador, Peru.

***mexicana*** (*Histricoverpa*) Prather, 2004:27 [Type locality: Mexico, Oaxaca, La Esperanza; IBUNAM; ♂; ♀].

**Distribution.** Mexico.

***mollicula*** (*Histricoverpa*) (McLachlan), 1871:127 [Type locality: Venezuela; BMNH; ♂; in *Ganonema*]. —Flint, 1983a:77 [to *Banyallarga*]. —Prather, 2004:28 [♂; ♀; redescription; to *Histricoverpa*].

**Distribution.** Venezuela.

***nica*** (*Histricoverpa*) Prather, 2004:29 [Type locality: Nicaragua, Jinotega, Peñas Blancas, 13°17'00"N, 85°33'00"W, el. 1300 m; UMSP; ♂; ♀]. —Chamorro-Lacayo et al., 2007:40 [checklist].

**Distribution.** Nicaragua.

***penai*** (*Banyallarga*) Prather, 2004:16 [Type locality: Bolivia, La Paz, Unduavi/Coroico, el. 2500 m; NMNH; ♂; ♀].

**Distribution.** Bolivia, Ecuador.

***quincemil*** (*Histricoverpa*) Prather, 2004:30 [Type locality: Peru, Cuzco, Quincemil; CNC; ♂; ♀].

**Distribution.** Peru.

***sanchezi*** (*Histricoverpa*) Prather, 2004:31 [Type locality: Colombia, Huila, Quebrado Juancho, 10 km W Iquira; NMNH; ♂].

**Distribution.** Colombia.

***sylvana*** (*Histricoverpa*) Prather, 2004:32 [Type locality: Costa Rica, Alajuela, Reserva Forestal San Ramón, Río San Lorencito & tribs., 10°12'58"N, 84°36'25"W, el. 980 m; UMSP; ♂; ♀]. —Chamorro-Lacayo et al., 2007:40 [checklist].

**Distribution.** Costa Rica, Nicaragua.

***tapanti*** (*Histricoverpa*) Prather, 2004:33 [Type locality: Costa Rica, Cartago, Reserva Tapanti, Quebrada Segunda at administration building, 09°45'40"N, 83°47'13"W, el. 1250 m; UMSP; ♂; ♀].

**Distribution.** Costa Rica.

***vicaria*** (*Banyallarga*) (Walker), 1852:114 [Type locality: Venezuela; BMNH; ♀; in *Hydropsyche*]. —McLachlan, 1871:127 [♂; in *Ganonema*]. —Betten and Mosely, 1940:218 [redescription; in *Ganonema*]. —Flint, 1983a:77 [to *Banyallarga*]. —Prather, 2004:17 [Neotype: Venezuela, Mérida, Parque Nacional Sierra Nevada, Mucuy Fish Hatchery, 7 km E Tabay, Queb. La Mucuy, el. 2012 m; UMSP; ♂; ♀; distribution].

—*testacea* Navás, 1916b:78 [Type locality: Colombia, Muzo; collection Appolinaris, now lost?; ♂]. —Muñoz-Quesada, 2000:274 [checklist]. —Prather, 2004:17 [to synonymy].

**Distribution.** Bolivia, Colombia, Venezuela.

***villosa*** (*Banyallarga*) (Navás), 1934a:174 [Type locality: Ecuador, Loja; MNHNP; original description implies ♂; but type is ♀; in *Anisocentropus*]. —Flint, 1983a:77 [to *Banyallarga*]. —Prather, 2004:19 [♂; /female; redescription].

**Distribution.** Ecuador.

***yungensis*** (*Banyallarga*) Flint, 1983a:79 [Type locality: Argentina, Pcia. Tucumán, Horco Molle, near Tucumán; NMNH; ♂]. —Martynov 1912:7 [♂; misidentified as *Ganonema vicarium*]. —Flint, 1996b:424 [distribution]. —Cohen, 2004:74 [list of type material]. —Prather, 2004:20 [♂; /female; redescription; distribution]. —Rueda-Martin, 2013:327 [♂]. —Isa Miranda and Rueda Martín, 2014:199 [distribution].

**Distribution.** Argentina, Bolivia, Peru, Venezuela.

#### Genus *Phylloicus* Müller [57 + †1]

*Phylloicus* Müller, 1880a:113, 131 [Type species: *Phylloicus major* Müller, 1880a, subsequent selection of Flint, 1964a, not Fischer, 1965]. —Müller, 1880b:180 [♂; ♀; cases]. —Müller, 1888:274 [larva]. —Ulmer, 1905b:77 [adults]. —Ulmer, 1907d:120 [adults]. —Lestage, 1925:42 [checklist; key]. —Betten, 1934:236 [adults]. —Flint and Angrisano, 1985:688 [larva; pupa; distinguished from *Banyallarga*]. —Roldán Pérez, 1988:146 [larva]. —Wiggins, 1996:224 [larva]. —Prather, 2003:17 [revision; ♂; ♀; key to species]. —Wichard, 2007a:34 [diagnosis]. —Rueda Martín, 2013:328 [case; differentiation of larvae from *Banyallarga*].

*Homoeoplectron* Ulmer, 1905a:33 [Type species: *Homoeoplectron assimile* Ulmer, 1905a = *Phylloicus major* Müller, 1880a, subsequent selection of Fischer, 1965]. —Ulmer, 1905b:77 [to synonymy].

*Notiomyia* Banks, 1905:18 [Type species: *Heteroplectron mexicanum* Banks, 1900, original designation]. —Flint, 1967c:17 [to synonymy].

*Murielia* Hogue and Denning, in Denning et al., 1983:187 [Type species: *Phylloicus farri* Flint, 1968a, original designation]. —Flint et al., 1999a:73 [to synonymy].

The genus is limited to Latin America, except for two species which extend into the southwestern United States. As in *Banyallarga*, the often strikingly colored adults are day active, although they do appear at collecting lights, especially teneral individuals.

Larvae have been described a number of times (Ulmer 1955, Wiggins 1996, Botosaneanu and Sykora 1973, Botosaneanu and Flint 1982, Bowles and Flint 1997, Huamantinco et al. 2005, Quinteiro et al. 2011, Rueda Martín 2013). The flat case, made of oval pieces of leaves, is distinctive. Larvae live in still, backwater pools of rivers and streams; they can occur in very large numbers. One species is known to occur in water trapped in the leaf axils of Bromeliaceae (Müller 1880a, Banks 1912). Larvae are detritivorous, feeding in leaf litter depositional areas. Moretti et al. (2009) showed that *Phylloicus* selected leaves for case-building that were chemically protected againt microbial degradation and consumption by other shredders, but this was dependant on leaf abundance. As a typical and important leaf shredder in Neotropical aquatic ecosystems, *Phylloicus* has been been the subject of many different kinds of biological and ecological studies, perhaps the most of any caddisfly in the region (e.g., Rincón and Martínez 2006, Wantzen and Wagner 2006, Becker et al. 2009, Encalada et al. 2010, Landeiro et al. 2010, Navarro et al. 2013, Vidovix et al. 2013, Guzmán-Soto and Tamarís-Turizo 2014, Martins et al. 2014, Rezende et al. 2015).

***abdominalis*** (Ulmer), 1905a:34 [Type locality: Brazil, “Are-as”, probably in Santa Catarina; ZIUH; type destroyed; ♂; in *Homoeoplectron*]. —Ulmer, 1913:398 [distribution]. —Prather 2003:15 [Neotype: Brazil, Santa Catarina, Itajai; MCZ; ♂; ♀]. —Paprocki et al., 2004:5 [checklist]. —Huamantinco et al., 2005:20 [larva; pupa; distribution]. —Dumas et al., 2009:367 [distribution]. —Calor, 2011:319 [checklist]. —Dumas and Nessimian, 2012:8 [checklist]. —Paprocki and França, 2014:4 [checklist]. —Quinteiro et al., 2014:230 [distribution].

**Distribution.** Argentina, Brazil.

***aculeatus*** (Blanchard), 1851:138 [Type locality: Chile; MNHNP; ♀; in *Macronema*]. —Flint, 1974e:84, 90 [♀; lectotype; to *Phylloicus*]. —Flint, 1990:119 [distribution]. —Prather, 2003:17 [♂; ♀; distribution]. —Cohen, 2004:77 [distribution]. —Guevara-Cardona et al., 2007:123 [checklist].

—*distans* Navás, 1918c:226 [Type locality: Chile, Marga-Marga, Los Perales; MZBS; ♂]. —Flint, 1974e:84 [to synonymy].

**Distribution.** Argentina, Chile.

***adamsae*** Prather, 2003:19 [Type locality: Peru, Madre de Dios, Manu Biosphere Res[erve], Pakitza Bio[logical] Sta[tion], 11°56'00"S, 71°18'00"W, el. 350 m; MHNJP; ♂; ♀].

—“n. sp. 3” Flint, 1996: 425. ­—Prather, 2003:19 [to synonymy].

**Distribution.** Peru.

***aeneus*** (Hagen), 1861:285 [Type locality: Mexico, [Veracruz]; MCZ; ♀; in *Macronema*]. —Hagen, 1864b:804 [to *Anisocentropus*]. —Ulmer, 1905b:79 [to *Phylloicus*; redescription; ♂; in *Phylloicus nigripennis*]. —Ross, 1952:34 [lectotype; ♂]. —Flint, 1967c:17 [♂; in *Phylloicus nigripennis*]. —Flint, 1967d:174 [distribution]. —Bueno-Soria and Flint, 1978:210 [distribution]. —Denning et al., 1983:182 [redescription]. —Aguila, 1992:543 [distribution]. —Wiggins, 1996:224 [larva]. —Bowles and Flint, 1997:58 [variation]. —Maes, 1999:1196 [checklist]. —Prather, 2003:21 [♂; ♀; distribution]. —Baumgardner and Bowles, 2005:11 [distribution]. —Blinn and Ruiter, 2006:332 [biology]. —Bowles et al., 2007:23 [distribution; biology]. —Bueno-Soria et al., 2007:32 [distribution]. —Chamorro-Lacayo et al., 2007:40 [checklist]. —Ruiter and Blinn, 2009:4 [distribution]. —Bueno-Soria and Barba-Álvarez, 2011:354 [checklist]. —Djernaes, 2011:52 [♂; ♀]. —Armitage et al., 2015b:8 [checklist]. —Armitage and Cornejo, 2015:197 [checklist].

—*ornatus* (Banks), 1909:342 [Type locality: United States, Texas, Brownwood; MCZ; ♀; in *Notiomyia*]. —Flint, 1967c:17 [to *Phylloicus*]. —Flint, 1967d:173 [distribution]. —Bueno-Soria and Flint, 1978:211 [distribution]. —Holzenthal, 1988c: 72 [distribution]. —Bowles and Flint, 1997:53 [redescription; ♂; ♀; larva; pupa]. —Norwood and Stewart, 2002:44 [biology]. —Prather, 2003:21 [to synonymy].

—*centralus* (Navás), 1924c:82 [Type locality: Costa Rica; MNHNP; ♂; in *Macronema*]. —Holzenthal, 1988:72 [to *Phylloicus*]. —Prather, 2003:21 [to synonymy].

***amazonas*** Prather, 2003:26 [Type locality: Venezuela, Amazonas, Cerro de la Neblina, Basecamp, 00°51'N, 66°10'W, el. 140 m; NMNH; ♂]. —Paprocki et al., 2004:5 [checklist]. —Paprocki and França, 2014:4 [checklist].

**Distribution.** Brazil, Guyana, Peru, Venezuela.

***angustior*** Ulmer, 1905b:78 [Type locality: Brazil, Rio Grande do Sul; NMW; ♂]. —Flint, 1966a:11 [lectotype; ♂]. —Flint, 1981a:36 [♂; distribution]. —Botosaneanu and Flint, 1982:24 [larva]. —Flint, 1991:98 [♂; distribution]. —Botosaneanu and Alkins-Koo, 1993:38 [♂; distribution]. —Flint, 1996a:102 [distribution; likely *hansoni*]. —Flint, 1996b:424 [distribution]. —Muñoz-Quesada, 2000:274 [checklist]. —Botosaneanu, 2002:96 [checklist]. —Prather, 2003:27 [♂; ♀; distribution]. —Cohen, 2004:77 [distribution]. —Paprocki et al., 2004:5 [checklist]. —Angrisano and Sganga, 2007:41 [♂; ♀; distribution]. —Calor, 2011:319 [checklist]. —Dumas and Nessimian, 2012:8 [checklist]. —Manzo et al., 2014:167 [distribution]. —Paprocki and França, 2014:4 [checklist]. —Isa Miranda and Rueda Martín, 2014:199 [distribution].

**Distribution.** Argentina, Brazil, Colombia, Paraguay, Trinidad[?], Uruguay, Venezuela.

***auratus*** Prather, 2003:29 [Type locality: Peru, Madre de Dios, Manu Biosphere Res[erve], Pakitza Bio[logical] Sta[tion], Aquajal, 11°56'00"S, 71°18'00"W, el. 250 m; MHNJP; ♂; ♀]. —Paprocki et al., 2004:5 [checklist]. —Paprocki and França, 2014:4 [checklist].

—“n. sp. 4” Flint, 1996:425. —Prather, 2003:29 [to synonymy].

**Distribution.** Brazil, Peru.

***bertioga*** Prather, 2003:31 [Type locality: Brazil, São Paulo, Bertioga, 23°51'00"S, 46°09'00"W, el. 5 m; MZUSP; ♂; ♀]. —Paprocki et al., 2004:5 [checklist]. —Calor, 2011:319 [checklist]. —Paprocki and França, 2014:5 [checklist].

**Distribution.** Brazil.

***bicarinatus*** Prather, 2003:32 [Type locality: Peru, Madre de Dios, Manu, Biosphere Res[erve], Pakitza Bio[logical] Sta[tion], Quebrada Trompetero, trail 2, marker 15, 11°56'39"S, 71°16'59"W, el. 350 m; MHNJP; ♂; ♀].

**Distribution.** Bolivia, Peru.

***bidigitatus*** Prather, 2003:34 [Type locality: Brazil, Rio de Janeiro, Itatiaia; NMW; ♂]. —Paprocki et al., 2004:5 [distribution]. —Calor, 2011:319 [checklist]. —Dumas et al., 2009:367 [distribution]. —Dumas et al., 2010:7 [distribution]. —Dumas and Nessimian, 2012:9 [checklist]. —Paprocki and França, 2014:5 [checklist]. —Quinteiro et al., 2014:230 [distribution].

**Distribution.** Brazil.

***blahniki*** Prather, 2003:35 [Type locality: Costa Rica, Puntarenas, Parque Nacional Corcovado, unnamed stream, Piedra el Arco, 08°34'55"N, 83°42'32"W, el. 20 m; UMSP; ♂; ♀]. —Armitage et al., 2015b:8 [checklist]. —Armitage and Cornejo, 2015:197 [checklist].

**Distribution.** Costa Rica, Panama.

***brevior*** Banks, 1914b (1915):632 [Type locality: Guyana, Bartica; MCZ; ♂]. —Flint, 1967c:18 [♂]. —Flint, 1974c:139 [♂; distribution]. —Prather, 2003:36 [♂; ♀; distribution]. —Paprocki et al., 2004:5 [checklist]. —Dumas et al., 2010:9 [distribution as Pará state, Brazil, not Paraná as reported by Prather 2003 and Paprocki et al. 2004]. —Paprocki and França, 2014:5 [checklist].

**Distribution.** Brazil, Guyana, Suriname.

***bromeliarum*** Müller, 1880a:131 [Type locality: Brazil, Santa Catharina [sic]; MNRJ; case]. —Ulmer, 1906:56 [♀]. —Ulmer, 1913:398 [♂; distribution]. —Ulmer, 1955:418 [larva]. —Prather, 2003:38 [Lectotype: Brazil, Santa Catarina, Blumenau, 26°56'0"S, 49°3'0"W; MCZ; ♂; ♀; distribution]. —Paprocki et al., 2004:5 [checklist]. —Calor, 2011:319 [checklist]. —Paprocki and França, 2014:5 [checklist].

**Distribution.** Argentina, Brazil.

***camargoi*** Quinteiro and Calor, in Quinteiro et al., 2011:39 [Type locality: Brazil, São Paulo, Faculdade de Filosofia, Ciências e Letras de Ribeirão Preto, 21°10'04"S, 47°51'25"W; MZUSP; ♂; ♀; /larva/; /pupa/]. —Calor, 2011:319 [checklist]. —Paprocki and França, 2014:5 [checklist].

**Distribution.** Brazil.

***chalybeus*** (Hagen), 1861:285 [Type locality: Cuba; MCZ; ♂; in *Macronema*]. —Ross, 1952:34 [lectotype; ♂]. —Flint, 1967c:18 [♂]. —Flint, 1968b:83 [checklist]. —Botosaneanu, 1979:52 [distribution]. —Botosaneanu, 1980:114 [♂; restriction of type locality]. —Kumanski, 1987:32 [distribution]. —Botosaneanu, 1994b:468 [larva]. —Flint, 1996c:15 [checklist]. —Botosaneanu, 2002:96 [checklist].—Prather, 2003:39 [♂; ♀; distribution]. —López del Castillo et al., 2004:229 [distribution]. —Naranjo López and González Lazo, 2005:150 [checklist]. —López del Castillo et al., 2007:171 [distribution; seasonal abundance].

**Distribution.** Cuba.

***cordatus*** Prather, 2003:41 [Type locality: Venezuela, Amazonas, Cerro de la Neblina, Camp IV, 00°58'00"N, 65°57'00"W, el. 760 m; NMNH; ♂; ♀].

**Distribution.** Venezuela.

***crenatus*** Navás, 1916b:79 [Type locality: Colombia, Muzo; collection Apollinaris, now lost?; ♂; in *Banyallarga*]. —Lestage, 1925: 44 [perhaps *Phylloicus*]. —Flint, 1983a:77 [to *Phylloicus*]. —Muñoz-Quesada, 2000:274 [checklist]. —Prather, 2003:42 [as *nomen dubium*].

**Distribution.** Colombia.

***cressae*** Prather, 2003:43 [Type locality: Venezuela, Lara, Parque Nacional Dinira, Quebrada Las Pinetas, 09°46'19"N, 70°01'45"W, el. 1889 m; UMSP; ♂; ♀].

**Distribution.** Bolivia, Ecuador, Venezuela.

***cubanus*** Banks, 1924:445 [Type locality: Cuba; MCZ; [♂]. —Banks, 1938:298 [distribution]. —Flint, 1967c:18 [♂]. —Flint, 1968b:83 [checklist]. —Botosaneanu and Sykora, 1973:399 [♂; larva; pupa]. —Botosaneanu, 1979:52 [distribution]. —Botosaneanu, 1994b:468 [larva]. —Flint, 1996c:15 [checklist]. —Botosaneanu, 2002:97 [checklist]. —Prather, 2003:45 [♂; ♀; distribution]. — López del Castillo et al., 2004:229 [distribution]. —González Lazo et al., 2005:260 [distribution]. —Naranjo López and González Lazo, 2005:150 [checklist].

**Distribution.** Cuba.

***dumasi*** Santos and Nessimian, 2010a:322 [Type locality: Brazil, Amazonas State, Rio Preto da Eva municipality, tributary to Rio Preto da Eva, 02°32'09.4"S, 59°49'59.3"W; INPA; ♂; ♀]. —Paprocki and França, 2014:5 [checklist].

**Distribution.** Brazil.

***elegans*** Hogue and Denning, in Denning et al., 1983:184 [Type locality: Panama, Canal Zone, Barro Colorado Island; WSU; ♂]. —Flint, 1991:98 [♂; distribution]. —Maes and Flint, 1988:6 [distribution]. —Holzenthal, 1988c:72 [distribution]. —Aguila, 1992:543 [distribution]. —Maes, 1999:1196 [checklist]. —Muñoz-Quesada, 2000:274 [checklist]. —Prather, 2003:46 [♂; ♀; distribution]. —Chamorro-Lacayo et al., 2007:40 [checklist]. —Armitage et al., 2015b:8 [checklist]. —Armitage and Cornejo, 2015:197 [checklist].

**Distribution.** Colombia, Costa Rica, Ecuador, Nicaragua, Panama.

***elektoros*** Prather, 2003:48 [Type locality: Venezuela, Amazonas, Cerro de la Neblina, Basecamp, in rain forest, 00°51'N, 66°10'W, el. 140 m; NMNH; ♂; ♀]. —Martins et al., 2014:337 [biology]. —Paprocki and França, 2014:5 [checklist].

**Distribution.** Brazil, Peru, Venezuela.

***ephippium*** Prather, 2003:50 [Type locality: Ecuador, Tungurahua, 13 km E Baños, el. 1550 m; NMNH; ♂; ♀].

**Distribution.** Ecuador.

***farri*** Flint, 1968a:56 [Type locality: Jamaica, St. Andrew, Hope River near Newcastle at mile post 16.5; NMNH; ♂; ♀; larva; pupa; case]. —Flint, 1968b:83 [checklist]. —Denning et al., 1983:188 [type species of *Murielia*]. —Botosaneanu and Hyslop, 1998:21 [distribution]. —Malicky, 1999:116, 117 [distribution]. —Flint et al., 1999a:73 [returned to *Phylloicus*]. —Botosaneanu, 2002:97 [checklist]. —Prather, 2003:51 [♂; distribution].

**Distribution.** Jamaica.

***fenestratus*** Flint, 1974c:139 [Type locality: Suriname, Nickerie River, Stondansi; RNH; ♂]. —Flint, 1996b:425 [distribution]. —Prather, 2003:53 [♂; ♀; distribution]. —Paprocki et al., 2004:5 [checklist]. —Dumas et al., 2010:9 [distribution as Pará state, Brazil, not Paraná as reported by Prather 2003 and Paprocki et al. 2004]. —Paprocki and França, 2014:5 [checklist].

**Distribution.** Brazil, Ecuador, Guyana, Peru, Suriname, Venezuela.

***flinti*** Prather, 2003:55 [Type locality: Peru, Madre de Dios, Manu Biosphere Res[erve], Pakitza Bio[logical] Sta[tion], trail 1, 1st stream, 11°56'00"S, 71°18'00"W, el. 250 m; MHNJP; ♂; ♀]. —Paprocki et al., 2004:5 [checklist]. —Paprocki and França, 2014:6 [checklist].

—“n. sp. 2” Flint, 1996:425. ­—Prather, 2003:55 [to synonymy].

**Distribution.** Brazil, Peru.

***hansoni*** Denning, 1983, in Denning et al., 1983:184 [Type locality: Trinidad, Simla Research Station; CAS; ♂]. —Botosaneanu and Flint, 1982:24 [larva; as synonym of *Phylloicus angustior*]. —Botosaneanu and Alkins-Koo, 1993:38 [as synonym of *Phylloicus angustior*]. —Flint, 1996a:102 [distribution; as *angustior*]. —Flint et al., 1999a:57 [as synonym of *Phylloicus angustior*]. —Botosaneanu, 2002:96 [as *Phylloicus angustior*]. —Prather, 2003:57 [resurrected; distribution].

**Distribution.** Trinidad, Venezuela.

***holzenthali*** Prather, 2003:59 [Type locality: Venezuela, Tachira, Quebrada La Honda, 10 km E La Grita, 08°08'49"N, 71°56'02"W, el. 2300 m; UMSP; ♂; ♀].

**Distribution.** Colombia, Venezuela.

***iridescens*** Banks, 1941:397 [Type locality: Dominican Republic, Constanza to V. Nuevo; MCZ; ♂]. —Flint, 1967c:18 [lectotype; ♂]. —Flint, 1968b:83 [checklist]. —Botosaneanu, 1996:20 [distribution]. —Flint and Pérez-Gelabert, 1999:36 [checklist]. —Botosaneanu, 2002:97 [checklist]. —Prather, 2003:61 [♂; ♀; distribution]. —Flint and Sykora, 2004:3 [distribution]. —Pérez-Gelabert, 2008:298 [checklist].

**Distribution.** Dominican Republic.

***lituratus*** Banks, 1920:350 [Type locality: Colombia, Mariquito; MCZ; ♂]. —Flint, 1967c:19 [♂]. —Denning et al., 1983:182 [redescription]. —Holzenthal, 1988c:72 [distribution; as *priapulus*]. —Aguila, 1992:543 [distribution]. —Muñoz-Quesada, 2000:274 [checklist]. —Prather, 2003:62 [♂; ♀; distribution]. —Chamorro-Lacayo et al., 2007:40 [checklist]. —Rueda Martín, 2013:322 [♂; larva; pupa; case; biology; distribution]. —Armitage et al., 2015b:8 [checklist]. —Armitage and Cornejo, 2015:197 [checklist].

—“species 1” Flint, 1991:98. —Prather, 2003:62 [to synonymy].

—*priapulus* Denning and Hogue, 1983, in Denning et al., 1983:187 [Type locality: Costa Rica, Puntarenas, Province, 1.8 miles west of Rincón, Osa Peninsula; LACM; ♂]. —Graça et al., 2001:951 [biology]. —Prather, 2003:62 [to synonym].

**Distribution.** Argentina, Colombia, Costa Rica, Ecuador, Nicaragua, Panama, Venezuela.

***llaviuco*** Prather, 2003:65 [Type locality: Ecuador, Azuay, Río Llaviuco, 16 km W Quenca, el. 3010 m; NMNH; ♂].

**Distribution.** Ecuador.

***maculatus*** (Banks), 1901:369 [Type locality: Mexico, Veracruz, Presidio; MCZ; ♀; in *Heteroplectron*]. —Flint, 1967c:19 [♀; to *Phylloicus*]. —Bueno-Soria and Flint, 1978:211 [distribution]. —Holzenthal, 1988c:72 [distribution]. —Prather, 2003:66 [♂; ♀; distribution].

**Distribution.** Costa Rica, Guatemala, Mexico.

***magnus*** Banks, 1913a:236 [Type locality: Colombia, Monte Socorro; MCZ; ♂]. —Flint, 1967c:19 [♂]. —Muñoz-Quesada, 2000:275 [checklist]. —Prather, 2003:68 [♂; ♀].

**Distribution.** Colombia.

***major*** Müller 1880a:113, 131 [Type locality: Brazil, Santa Catarina; MNRJ; case]. —Ulmer, 1905b:77, 78 [as synomyn of *assimilis*]. —Flint, 1964a:65 [type species of genus]. —Flint, 1966a:11 [♂; taxonomic remarks]. —Prather, 2003:69 [♂; ♀; distribution]. —Paprocki et al., 2004:5 [checklist]. —Dumas et al., 2009:367 [distribution]. —Calor, 2011:319 [checklist]. —Paprocki and França, 2014:6 [checklist].

—*assimilis* (Ulmer), 1905a:36 [Type locality: Brazil, Santa Catarina; PAN; ♂; in *Homoeoplectron*]. —Flint, 1966a:11 [♂; lectotype; to synonymy].

**Distribution.** Brazil, Paraguay.

***medius*** Müller 1880a:132 [Type locality: Brazil, Santa Catarina; no type nor type depository designated; sex not stated]. —Ulmer, 1955:418 [literature, discussion]. —Prather, 2003:71 [as *nomen dubium*]. —Paprocki et al., 2004:5 [checklist].

**Distribution.** Brazil.

***mexicanus*** (Banks), 1900:257 [Type locality: Mexico, Morelos, Cuernavaca; MCZ; ♂ (description implies type is female, but specimen with type label is a male according to Prather, 2003); in *Heteroplectron*]. —Banks, 1901 :369 [distribution]. —Flint, 1967c:17 [as synonym of *Phylloicus aeneus*]. —Wiggins, 1996:224 [larva; as *Phylloicus aeneus*]. —Prather, 2003:71 [resurrected; ♂; ♀; distribution]. —Blinn and Ruiter, 2006:332 [biology]. —Blinn and Ruiter, 2009a:307 [biology]. —Blinn and Ruiter, 2009b:185 [phenology, distribution]. —Ruiter and Blinn, 2009:4 [distribution].

**Distribution.** Mexico, U.S.A.

***monneorum*** Dumas and Nessimian, 2010a:309 [Type locality: Brazil, Rio de Janeiro, Itatiaia, Parque Nacional do Itatiaia, Rio Campo Belo tributary in the track to Lago Azul, 22°27'8.38"S, 44°36'40.99"W, el. 790 m; DZRJ; ♂; ♀]. —Dumas and Nessimian, 2012:9 [checklist]. —Paprocki and França, 2014:6 [checklist]. —Quinteiro et al., 2014:231 [distribution].

**Distribution.** Brazil.

***monticolus*** Flint, 1968b:74 [Type locality: Dominica, 1.6 miles west of Pont Casse; NMNH; ♂; ♀; larva; pupa; case]. —Flint and Sykora, 1993:50 [checklist]. —Botosaneanu 1994a:51 [distribution]. —Botosaneanu, 2000:256 [distribution]. —Botosaneanu, 2002:97 [checklist]. —Prather, 2003:74 [♂; ♀]. —Botosaneanu and Thomas, 2005:53 [distribution].

**Distribution.** Dominica, Guadeloupe, Martinique.

***munozi*** Prather, 2003:75 [Type locality: Costa Rica, Cartago, Reserva Tapantí, Quebrada Palmitos & falls, ca. 9 km (road) NW tunnel, 09°43'12"N, 83°46'48"W, el. 1400 m; UMSP; ♂; ♀]. —Armitage et al., 2015b:8 [checklist]. —Armitage and Cornejo, 2015:197 [checklist].

—“species 2” Flint, 1991:99. —Prather, 2003:75 [to synonymy].

**Distribution.** Colombia, Costa Rica, Panama.

***nigripennis*** (Banks), 1900:256 [Type locality: Mexico, Puebla, Santa Maria; MCZ; ♀; in *Heteroplectron*]. —Banks, 1901 :369 [distribution]. —Flint, 1967c:17 [♂; as synonym of *Phylloicus aeneus*]. —Prather, 2003:77 [resurrected; ♂; ♀]. —Chamorro-Lacayo et al., 2007:40 [checklist].

—*latus* (Navás), 1924c:83 [Type locality: Costa Rica; MNHNP; ♂; as *Macronema latum*]. —Holzenthal, 1988c:53, 71 [as synonym of *Phylloicus aeneus*]. —Prather, 2003:77 [to synonymy].

—*sagittosa* (Ross), 1951a:72 [Type locality: Mexico, Lower California, Todos Santos; CAS; ♂; in *Notiomyia*]. —Denning, 1964:134 [distribution]. —Flint, 1967:17 [as synonym of *Phylloicus aeneus*]. —Prather, 2003:77 [to synonymy].

**Distribution.** Costa Rica, Guatemala, Honduras, Mexico, Nicaragua.

***obliquus*** Navás, 1931b:458 [Type locality: Brazil, Minas Gerais; DEI; ♀]. —Prather, 2003:79 [♂; ♀]. —Paprocki et al., 2004:5 [checklist]. —Dumas et al., 2009:368 [distribution]. —Paprocki and França, 2014:6 [checklist]. —Quinteiro et al., 2014:230 [distribution].

**Distribution.** Brazil.

***panamensis*** Prather, 2003:80 [Type locality: Panama, Chiriqui, Guadalupe Arriba, 08°52'26"N, 82°33'13"W; NMNH; ♂; ♀]. —Armitage et al., 2015b:8 [checklist]. —Armitage and Cornejo, 2015:197 [checklist].

**Distribution.** Costa Rica, Panama.

***paprockii*** Prather, 2003:82 [Type locality: Brazil, Minas Gerais, Aldeia de Cachoeira das Pedras, 20°06'49"S, 44°01'25"W, el. 925 m; MZUSP; ♂; ♀]. —Paprocki et al., 2004:5 [checklist]. —Paprocki and França, 2014:6 [checklist].

**Distribution.** Brazil.

***passulatus*** Prather, 2003:83 [Type locality: Venezuela, Amazonas, Puerto Ayacucho (40kmS) El Tobogán, Caño Coromoto; NMNH; ♂; ♀].

**Distribution.** Venezuela.

***paucartambo*** Prather, 2003:84 [Type locality: Peru, Cuzco, Paucartambo to Pilcopata rd., Río San Pedro at Puente San Pedro, 13°03'18"S, 71°32'47"W, el. 1445 m; MHNJP; ♂; ♀].

—“n. sp. 1” Flint, 1996:424. —Prather, 2003:84 [to synonymy].

**Distribution.** Ecuador, Peru.

***perija*** Prather, 2003:86 [Type locality: Venezuela, Zulia, Parque Nacional Perijá, Río Negro in Toromo, 10°03'04"N, 72°42'43"W, el. 360 m; UMSP; ♂; ♀].

**Distribution.** Venezuela.

***pirapo*** Prather, 2003:86 [Type locality: Paraguay, Itapua, Pirapo; NMNH; ♂; ♀].

**Distribution.** Argentina, Paraguay.

***plaumanni*** Flint, 1983a:76 [Type locality: Brazil, Edo. Santa Catarina, Seara, 27°09'S, 52°15'W; NMNH; ♂]. —Prather, 2003:88 [♂; ♀]. —Paprocki et al., 2004:5 [checklist]. —Paprocki and França, 2014:6 [checklist].

**Distribution.** Argentina, Brazil.

***pulchrus*** Flint, 1964a:65 [Type locality: Puerto Rico, Maricao Forest; NMNH; ♂; ♀; larva; pupa]. —Flint, 1968b:83 [checklist]. —Botosaneanu, 1996:21 [♂; misidentificated as *Phylloicus superbus* according to Flint and Sykora, 2004]. —Botosaneanu, 2002:97 [checklist]. —Prather, 2003:90 [♂; ♀; distribution]. —Flint and Sykora, 2004:3 [distribution]. —Pérez-Gelabert, 2008:298 [checklist].

**Distribution.** Cuba, Dominican Republic, Puerto Rico.

***quadridigitatus*** Prather, 2003:91 [Type locality: Brazil, São Paulo, Alto da Serra; NMW; ♂]. —Paprocki et al., 2004:5 [checklist]. —Calor, 2011:319 [checklist]. —Paprocki and França, 2014:6 [checklist].

**Distribution.** Brazil.

***quitacalzon*** Prather, 2003:92 [Type locality: Peru, Madre de Dios, Toma del Agua, Amazonia Lodge, 12°52'13"S, 71°22'34"W, el. 415 m; MHNJP; ♂; ♀].

**Distribution.** Peru.

***spectabilis*** Martynov, 1912:9 [Type locality: Peru, Callanga; ASL; ♂]. —Prather, 2003:94 [♂].

**Distribution.** Peru.

***spinulacolis*** Prather, 2003:95 [Type locality: Venezuela, Falcón, Río Ricoa near Dos Bocas, 11°17'19"N, 69°26'04"W, el. 157 m; UMSP; ♂; ♀].

**Distribution.** Venezuela.

***superbus*** Banks, 1938:298 [Type locality: Cuba, Oriente, Pico Turquino; MCZ; ♂]. —Flint, 1967c:19 [lectotype; ♂]. —Flint, 1968b:83 [checklist]. —Botosaneanu, 1979:52 [distribution]. —Flint and Pérez-Gelabert, 1999:36 [checklist]. —Botosaneanu, 2002:97 [checklist]. —Prather, 2003:96 [♂]. —Naranjo López and González Lazo, 2005:150 [checklist].

**Distribution.** Cuba, Dominican Republic.

***tricalcaratus*** (Ulmer), 1905a:37 [Type locality: Brazil, Bahia, Freyreiss; ZIUH; ♂; in *Homoeoplectron*]. —Ulmer, 1905b:78 [to *Phylloicus*; key]. —Prather, 2003:97 [as *nomen dubium*]. —Paprocki et al., 2004:5 [checklist].

**Distribution.** Brazil.

***trichothylax*** Prather, 2003:98 [Type locality: Ecuador, Cotopaxi, Latacunga, 13 km W, el. 1372 m; NMNH; ♂].

**Distribution.** Ecuador.

† ***velteni*** Wichard, 2007a:35 [Type locality: Dominican Republic; SMNS; ♂; in amber].

**Distribution.** Dominican Republic.

***yolandae*** Prather, 2003:98 [Type locality: Brazil, Paraná, Municipio Corbélia, Rio Novo, headwaters, 24°53'53"S, 53°14'54"W, el. 700 m; MZUSP; ♂]. —Paprocki et al., 2004:5 [checklist]. —Paprocki and França, 2014:6 [checklist].

**Distribution.** Brazil.

### Family Ecnomidae

This family now contains 10 genera and almost 400 species, mostly confined to the Southern Hemisphere, with Australia home to several endemic genera. In the Northern Hemisphere, species extend into the Palearctic and just barely into the Nearctic regions. In their recent phylogeny, Johanson and Espeland (2009) placed the putative origin of the family as Gondwanan. In the New World, all species but one are placed in the genus *Austrotinodes*.

The larvae of a number of the Old World species of *Ecnomus* have been described (Ulmer 1957, Lepneva 1970, Scott 1968) as well as those of *Austrotinodes* (Flint 1973a, Bowles 1995, Wiggins 1996). They construct silken tubes with fine sand grains incorporated, attached to rocks, wood, or submerged vegetation, and occur in both lotic and lectic waters. The pupa is found in an oval, silken cocoon, firmer than the retreat, with silken sieve openings at each end (Ulmer 1957).

#### Genus *Austrotinodes* Schmid [43]

*Austrotinodes* Schmid, 1955a:132 [Type species: *Austrotinodes latior* Schmid, 1955a, original designation]. —Flint, 1973a:127 [review of genus]. —Flint and Denning, 1989b:108 [review]. —Bowles, 1995:160 [checklist]. —Wichard, 2007a:25 [♀; fossil, diagnosis]. —Cartwright, 2009:3 [Australian species, key].

*Austrotinodes*, originally described for a series of species from southern Chile and adjacent Argentina, is now known to occur throughout the entire Neotropics, including the West Indies and Texas (Bowles 1995), and Australia (Cartwright 2009). Wichard (2007a) recorded a female from Dominican amber, but he did not formally describe it as a new species.

The pupa of *Austrotinodes recta* and the larva of an unknown Chilean species were described by Flint (1973a). The larva of *Austrotinodes cubanus* was described (Botosaneanu 1994b) and the larva and pupa of *Austrotinodes texensis* are also known (Bowles 1995); the latter species is known only from Texas, USA, and is therefore not included below. The adults are generally collected, most commonly by net or rarely at UV lights, near flowing waters, from small streams to rather large rivers. The larvae live in elongate, rather flimsy shelters of sand and silk on the undersides of rocks. Larval food is probably fine detrital matter.

***abrachium*** Thson and Holzenthal, 2010:39 [Type locality: Brazil, Minas Gerais, Rio Paraúna, 3 km S Santana do Riacho, 19°10.986'S, 43°43.485'W, el. 650 m; MZUSP; ♂]. —Paprocki and França, 2014:7 [checklist].

**Distribution.** Brazil.

***adamsae*** Flint, 1996a:76 [Type locality: Tobago, Hermitage River, 5 km S Charlotteville, 11°19'N, 60°34'W; NMNH; ♂; ♀]. —Botosaneanu, 2002:96 [checklist].

**Distribution.** Tobago.

***amazonensis*** Flint and Denning, 1989b:119 [Type locality: Brazil, Amazonas State, Hwy. AM 010, km 246, 20 km W Itacoatiara; MZUSP; ♂]. —Bowles, 1995:160 [checklist]. —Paprocki et al., 2004:5 [checklist]. —Paprocki and França, 2014:7 [checklist].

**Distribution.** Brazil.

***ancylus*** Flint and Denning, 1989b:114 [Type locality: Ecuador, Pastaza Province, Tzapino, 32 km NE Tigueno at 1°11'S, 77°14'W; NMNH; ♂]. —Bowles, 1995:160 [checklist].

**Distribution.** Ecuador.

***angustior*** Schmid, 1955a:133 [Type locality: Chile (ile de Chiloé) Aucar; NMNH; ♂]. —Flint, 1974e:87 [checklist]. —Flint and Denning, 1989b:109 [distribution]. —Bowles, 1995:160 [checklist].

**Distribution.** Argentina, Chile.

***ariasi*** Flint and Denning, 1989b:118 [Type locality: Brazil, Amazonas State, Reserva Ducke, Hwy. AM 010, km 26; MZUSP; ♂]. —Bowles, 1995:160 [checklist]. —Paprocki et al., 2004:5 [distribution]. —Dumas et al., 2010:7 [distribution]. —Paprocki and França, 2014:7 [checklist].

**Distribution.** Brazil.

***armiger*** Flint, 1983a:23 [Type locality: Chile, Pcia. Malleco, Cabrería, Cordillera Nahuelbuta; NMNH; ♂]. —Schmid, 1958b:202 [example of *angustior* this species]. —Flint and Denning, 1989b:109 [distribution]. —Bowles, 1995:160 [checklist].

**Distribution.** Argentina, Chile.

***belchioris*** Thomson and Holzenthal, 2010:39 [Type locality: Brazil, Minas Gerais, Parque Estadual do Itacolomi, Córrego Belchior, 20°25.048'S, 43°25.737'W; MZUSP; ♂; ♀]. —Paprocki and França, 2014:7 [checklist].

**Distribution.** Brazil.

***boliviensis*** Thomson and Holzenthal, 2010:42 [Type locality: Bolivia, Dept. La Paz, AMNI Madidi, Comun. San Miguel de Bala, Arroyo Bacuatra Grande, 14°30.737'S, 67°31.385'W, el. 280 m; UASC; ♂; ♀].

**Distribution.** Bolivia.

***bracteatus*** Flint and Denning, 1989b:119 [Type locality: Brazil, São Paulo State, Paranapiacaba Biological Station; MZUSP; ♂]. —Bowles, 1995:160 [checklist]. —Paprocki et al., 2004:5 [distribution]. —Calor, 2011:319 [checklist]. —Paprocki and França, 2014:7 [checklist].

**Distribution.** Brazil.

***brevis*** Schmid, 1958b:201 [Type locality: Chile, Contulmo (Palo Botado); NMNH; ♂]. —Flint, 1973a:135 [distribution]. —Flint, 1974e:87 [checklist]. —Bowles, 1995:160 [checklist].

**Distribution.** Chile.

***canoabo*** Flint and Denning, 1989b:116 [Type locality: Venezuela, Carabobo State, near Canoabo; NMNH; ♂]. —Bowles, 1995:160 [checklist].

**Distribution.** Venezuela.

***cekalovici*** Flint, 1969b:507 [Type locality: Chile, Prov. Cautin, Puente Hilquilco, south of Quepe; NMNH; ♂]. —Flint, 1974e:87 [checklist]. —Bowles, 1995:160 [checklist].

**Distribution.** Chile.

***chihuahua*** Flint and Denning, 1989b:109 [Type locality: Mexico, Cuiteco, Chihuahua State; UCD; ♂]. —Bowles, 1995:160 [checklist].

**Distribution.** Mexico.

***contubernalis*** Flint and Denning, 1989b:116 [Type locality: Panama, Chiriqui Province, Fortuna Dam Site, 8°44'N, 82°16'W; NMNH; ♂]. —Aguila, 1992:537 [distribution]. —Muñoz and Holzenthal, 1993:568 [♂; distribution]. —Bowles, 1995:160 [checklist]. —Armitage et al., 2015b:4 [checklist]. —Armitage and Cornejo, 2015:192 [checklist].

—*species* B McElravy et al., 1981:152 [in part, same data as *contubernalis*]. —Flint and Denning, 1989b:116 [to synonymy].

**Distribution.** Costa Rica, Panama.

***cressae*** Thomson and Holzenthal, 2010:45 [Type locality: Venezuela, Sucre, Península de Paria, Puerto Viejo, Rio el Pozo, 10°43.073'N, 62°28.569'W, el. 20 m; UMSP; ♂; ♀].

**Distribution.** Venezuela.

***cubanus*** Kumanski, 1987:10 [Type locality: Cuba, Province Pinar del Rio, torrent in the Cueva Fuentes; NMSB; ♀]. —Botosaneanu, 1994b:461 [larva]. —Bowles, 1995:160 [checklist]. —Flint, 1996c:15 [checklist]. —Botosaneanu, 2002:96 [checklist]. —González Lazo et al., 2005:260 [distribution]. —Naranjo López and González Lazo, 2005:150 [checklist]. —López del Castillo et al., 2007:171 [checklist].

**Distribution.** Cuba.

***doublesi*** Muñoz and Holzenthal, 1993:565 [Type locality: Costa Rica, Parque Nacional Guanacaste, Estación Pitilla, Río Orosí, 10.991°N, 85.428°W; NMNH; ♂]. —Bowles, 1995:160 [checklist]. —Maes, 1999:1184 [checklist]. —Chamorro-Lacayo et al., 2007:40 [checklist].

**Distribution.** Costa Rica, Nicaragua.

***fortunata*** Flint and Denning, 1989b:114 [Type locality: Panama, Chiriqui Province, Fortuna Dam Site, 8°44'N, 82°16'W; NMNH; ♂]. —Aguila, 1992:537 [distribution]. —Bowles, 1995:160 [checklist]. —Armitage et al., 2015b:4 [checklist]. —Armitage and Cornejo, 2015:192 [checklist].

—*species* B McElravy et al., 1981:152 [same data as *fortuna*]. —Flint and Denning, 1989b:114 [to synonymy].

**Distribution.** Panama.

***freytagi*** Flint and Denning, 1989b:110 [Type locality: Honduras, El Zamorano; CAS; ♂]. —Flint, 1973a:140 [Belize example of *sedmani* this species]. —Bowles, 1995:160 [checklist].

**Distribution.** Belize, Honduras.

***fuscomarginatus*** Flint and Denning, 1989b:117 [Type locality: Venezuela, Amazonas Federal Territory, Cerro de la Neblina, camp IV, 0°58'N, 65°57'W; NMNH; ♂]. —Bowles, 1995:160 [checklist].

**Distribution.** Venezuela.

***inbio*** Muñoz and Holzenthal, 1993:565 [Type locality: Costa Rica, Alajuela, Reserva Forestal San Ramón, Río San Lorencito and tribs., 10.216°N, 84.607°W; NMNH; ♂]. —Bowles, 1995:160 [checklist].

**Distribution.** Costa Rica.

***irwini*** Flint, 1973a:135 [Type locality: Chile, Prov. Malleco, Parque Nacional Nahuelbuta; UCR; ♂]. —Flint, 1974e:87 [checklist]. —Bowles, 1995:160 [checklist].

**Distribution.** Chile.

***labiatus*** Flint and Sykora, 2004:4 [Type locality: Dominican Republic, Pedernales Province, Río Mulito, 13 km N Pedernales, 18°09'N, 71°46'W, el. 230 m; CMNH; ♂]. —Pérez-Gelabert, 2008:298 [checklist].

**Distribution.** Dominican Republic.

***lineatus*** (Navás), 1934a:166 [Type locality: none given [presumably Chile]; collection Navás, now lost?; ♂; in *Tinodes*]. —Schmid, 1955a:132 [to *Austrotinodes*]. —Flint, 1974e:87 [checklist]. —Bowles, 1995:160 [checklist].

**Distribution.** Chile.

***longispinum*** Thomson and Holzenthal, 2010:45 [Type locality: Brazil, São Paulo, Cachoeira do Paredão, Lajeado, Serra da Bocaina, 22°43.533'S, 44°37.274'W, el. 1550 m; MZUSP; ♂; ♀]. —Calor, 2011:319 [checklist]. —Paprocki and França, 2014:7 [checklist].

**Distribution.** Brazil.

***madininae*** Botosaneanu, 1990a:40 [Type locality: Martinique, tributaries of Rivière Morose (Morne Vert, quartier Bernadette, at the foot of Pitons du Carbet; ZMUA; ♀]. —Flint and Sykora, 1993:49 [checklist]. —Bowles, 1995:160 [checklist]. —Botosaneanu, 2002:96 [checklist]. —Botosaneanu and Thomas, 2005:51 [♂; distribution].

**Distribution.** Martinique.

***mexicanus*** Flint, 1973a:136 [Type locality: Mexico, San Luis Potosi, El Salto Falls; NMNH; ♂; ♀; wings]. —Bueno-Soria and Flint, 1978:198 [distribution]. —Bowles, 1995:160 [checklist].

**Distribution.** Mexico.

***neblinensis*** Flint and Denning, 1989b:112 [Type locality: Venezuela, Amazonas Federal Territory, Cerro de la Neblina, basecamp (0°51'N, 66°10'W); NMNH; ♂]. —Bowles, 1995:160 [checklist].

**Distribution.** Venezuela.

***nielseni*** Flint and Denning, 1989b:120 [Type locality: Argentina, Rio Negro Province, Puerto Blest, Lago Nahuel Huapi; UZMC; ♂]. —Bowles, 1995:160 [checklist].

**Distribution.** Argentina.

***panamensis*** Flint, 1973a:136 [Type locality: Panama, Cerro Campana; NMNH; ♂]. —Flint and Denning, 1989b:112 [distribution; biology]. —Aguila, 1992:537 [distribution]. —Muñoz and Holzenthal, 1993:570 [♂; distribution]. —Bowles, 1995:160 [checklist]. —Chamorro-Lacayo et al., 2007:40 [distribution]. —Djernaes, 2011:12 [♂; ♀]. —Armitage et al., 2015b:4 [checklist]. —Armitage and Cornejo, 2015:192 [checklist].

**Distribution.** Costa Rica, Nicaragua, Panama.

***paraguayensis*** Flint, 1983a:22 [Type locality: Paraguay, Dpto. Paraguarí, Colonia Piraretá; NMNH; ♂]. —Flint and Denning, 1989b:117 [distribution; correction of original figure]. —Bowles, 1995:160 [checklist]. —Paprocki et al., 2004:5 [distribution]. —Paprocki and França, 2014:7 [checklist].

**Distribution.** Brazil, Paraguay.

***picada*** Flint, 1983a:22 [Type locality: Chile, Pcia. Chiloe, Huequetrumao, 22 km N Quellon; NMNH; ♂]. —Bowles, 1995:160 [checklist].

**Distribution.** Chile.

***prolixus*** Flint and Denning, 1989b:120 [Type locality: Brazil, Minas Gerais State, Chapéu do Sol, km 110, Serra do Cipó; MZUSP; ♂]. —Bowles, 1995:160 [checklist]. —Paprocki et al., 2004:5 [checklist]. —Dumas et al., 2009:357 [distribution]. —Dumas et al., 2010:7 [distribution]. —Calor, 2011:319 [checklist]. —Dumas and Nessimian, 2012:9 [checklist]. —Paprocki and França, 2014:8 [checklist].

**Distribution.** Brazil.

***quadrispina*** Schmid, 1958b:200 [Type locality: Chile, Contulmo (Palo Botado); NMNH; ♂]. —Flint, 1973a:140 [distribution]. —Flint, 1974e:87 [checklist]. —Bowles, 1995:160 [checklist].

**Distribution.** Chile.

***recta*** Schmid, 1964:322 [Type locality: Chile (Malleco) Rucanuco; NMNH; ♂]. —Flint, 1973a:140 [pupa; distribution]. —Flint, 1974e:87 [checklist]. —Flint and Denning, 1989b:109 [distribution]. —Bowles, 1995:160 [checklist].

**Distribution.** Argentina, Chile.

***recurvatus*** Flint, 1983a:25 [Type locality: Chile, Pcia. Maule, Alto Tregualemu, ca. 20 km SE Chovellén; NMNH; ♂]. —Bowles, 1995:160 [checklist].

**Distribution.** Chile.

***sedmani*** Flint, 1973a:140 [Type locality: Guatemala, Izabal, Las Escobas, near Matias de Galvez; NMNH; ♂; ♀]. —Holzenthal, 1988c:58 [distribution]. —Flint and Denning, 1989b:110 [distribution; original Belize record a misidentification of *freytagi*]. —Aguila, 1992:537 [distribution]. —Muñoz and Holzenthal, 1993:568 [/male; distribution]. —Bowles, 1995:160 [checklist]. —Armitage et al., 2015b:4 [checklist]. —Armitage and Cornejo, 2015:192 [checklist].

—*species* A McElravy et al., 1981:152 [Recorded from: Panama, Chiriqui Province, Fortuna Dam Site, 8°44'N, 82°16'W]. —Flint and Denning, 1989b:110 [to synonymy].

**Distribution.** Belize, Costa Rica, Guatemala, Panama.

***talcana*** (Navás), 1934a:165 [Type locality: Chile, Talca; MZBS; ♀; in *Tinodes*]. —Schmid, 1949a:340 [♀]. —Schmid 1955a:132 [to *Austrotinodes*]. —Flint, 1974e:87 [checklist]. —Flint and Denning, 1989b:109 [synonymy]. —Bowles, 1995:160 [checklist].

—*latior* Schmid, 1955a:132 [Type locality: Chile (ile de Chiloé) Aucar; NMNH; ♂]. —Flint, 1974e:87 [checklist]. —Flint and Denning, 1989b:109 [to synonymy]. —Cohen, 2004:74 [distribution].

**Distribution.** Argentina, Chile.

***taquaralis*** Thomson and Holzenthal, 2010:47 [Type locality: Brazil, Rio de Janeiro, Parque Nacional Itatiaia, Rio Taquaral, 22°27.252'S, 44°36.570'W, el. 1300 m; MZUSP; ♂]. —Dumas and Nessimian, 2012:9 [checklist]. —Paprocki and França, 2014:8 [checklist].

**Distribution.** Brazil.

***triangularis*** Schmid, 1958b:202 [Type locality: Chile, Pichinahuel, (Arauco); NMNH; ♂]. —Flint, 1974e:87 [checklist]. —Bowles, 1995:160 [checklist].

**Distribution.** Chile.

***tuxtlensis*** Flint and Denning, 1989b:114 [Type locality: Mexico, Veracruz State, Los Tuxtlas area, seeps at “Las Cabanas”; NMNH; ♂]. —Bowles, 1995:160 [checklist].

**Distribution.** Mexico.

***uruguayensis*** Angrisano, 1994:130 [Type locality: Uruguay, Depto. Paysandú, Sta. Rita; FHCU; ♂; ♀; wings]. —Blahnik et al., 2004:4 [distribution]. —Paprocki et al., 2004:5 [distribution]. —Paprocki and França, 2014:8 [checklist].

**Distribution.** Brazil, Uruguay.

#### Genus *Chilocentropus* Navás [1]

*Chilocentropus* Navás, 1934a:167 [Type species: *Chilocentropus disparilis* Navás, 1934a, original designation]. —Flint et al., 1999a:73 [to Ecnomidae].

This genus is likely a synonym of *Austrotinodes*, but having no evidence Flint et al., (1999a) made no formal taxonomic changes and we here follow that decision.

***disparilis*** Navás, 1934a:167 [Type locality: Chile, Marga-Marga; collection Navás, now lost?; sex unknown]. —Flint, 1974e:87 [checklist]. —Flint et al., 1999a:73 [status].

**Distribution.** Chile.

### Family Glossosomatidae

This cosmopolitan family of approximately 700 described species is represented in the Neotropics only by members of the subfamily Protoptilinae (save for one species of Glossosomatinae). The 269 Neotropical species, including 5 fossils in amber, are distributed among 11 genera, all endemic to the Neotropics, except for *Culoptila*, with species also in the southwestern U.S., *Protoptila*, with species widely distributed in North and South America, and a single, northern Mexican *Glossosoma* species. One of the Neotropical genera (*Cariboptila*) is endemic to the Greater Antilles, while 4 others (*Canoptila*, *Mastigoptila*, *Scotiotrichia*, *Tolhuaca*) are endemic to the Chilean Subregion and/or southeastern Brazil.

In their world revision of Glossosomatidae, Protoptilinae, Robertson and Holzenthal (2013) synonymized *Campsiphora* Flint, 1964 and *Cubanoptila* Sykora, 1973 with *Cariboptila* Flint, 1964. Their study included detailed diagnoses, descriptions, and phylogenetic hypotheses based on morphological and molecular sequence data to also justify the synonymization of several Asian and North American genera with *Padunia* Martynov, 1910, Malicky’s (2014) opinion notwithstanding.

Six of the 10 Neotropical genera are known in the immature stages. In general, the tropical species tolerate warmer and more slowly flowing waters than the northern species, but feed in the same manner, i.e., by scraping periphyton and associated detritus from the upper surfaces of rocks. The Neotropical species build typical tortoise-cases, often with dorsal respiratory openings resembling chimneys (Flint 1963a, Blahnik and Holzenthal 2006).

#### Genus *Canoptila* Mosely [2]

*Canoptila* Mosely, 1939a:218 [Type species: *Canoptila bifida* Mosely, 1939a, original designation]. —Robertson and Holzenthal, 2006:45 [revision, distribution; phylogeneitc position]. —Robertson and Holzenthal, 2013:27 [review of genus, phylogeny, diagnosis; key to Protoptilinae genera].

Two species are known in the genus, both endemic to the Atlantic forests of southeastern Brazil (Robertson and Holzenthal 2006). The immature stages and biology are unknown.

***bifida*** Mosely, 1939a:218 [Type locality: Brazil, Santa Catarina, Nova Teutonia; BMNH; ♂]. —Angrisano, 1999:30 [checklist]. —Paprocki et al., 2004:6 [checklist]. —Robertson and Holzenthal, 2006:51 [♂; redescription]. —Robertson and Holzenthal, 2013:51 [♂]. —Paprocki and França, 2014:8 [checklist].

**Distribution.** Brazil.

***williami*** Robertson and Holzenthal, 2006:53 [Type locality: Brazil, São Paulo, Parque Estadual Intervales, Riacho at Poços Altos, 24°18'20"S, 48°20'52"W, 830 m; MZUSP; ♂; ♀]. —Calor, 2011:320 [checklist]. —Paprocki and França, 2014:8 [checklist].

**Distribution.** Brazil.

#### Genus *Cariboptila* Flint [21 + †4]

*Cariboptila* Flint, 1964a:17 [Type species: *Cariboptila orophila* Flint 1964a, original designation]. —Wichard, 2007a:7 [key to fossil species, as *Cubanoptila*]. —Robertson and Holzenthal, 2013:1, 28 [review of genus, phylogeny, diagnosis; key to Protoptilinae genera].

*Campsiophora* Flint, 1964a:14 [Type species: *Campsiophora pedophila* Flint 1964a, original designation]. —Robertson and Holzenthal, 2013:1, 28 [review of genus, phylogeny, to synonymy].

*Cubanoptila* Sykora, in Botosaneanu and Sykora, 1973:383 [Type species: *Cubanoptila cubana* Sykora, 1973, original designation]. —Robertson and Holzenthal, 2013:1, 28 [new, subsequent designation of type species *Cubanoptila cubana* by virtual tautonomy; review of genus, phylogeny, to synonymy].

*Muangpaipsyche* Malicky and Silalom, 2012:22 [Type species: *Muangpaipsyche areopagita* Malicky and Silalom, 2012, by monotypy]. —Malicky 2013:42 [as synonym of *Campsiophora*]. —Robertson and Holzenthal, 2013:28 [to synonymy].

*Cariboptila* is endemic to the islands of the Greater Antilles. The immatures stages of *Cariboptila orophila* and *Cariboptila jamaicensis* were described by Flint (1964a, 1968a, respectively). The immature stages of *Cariboptila cubana* and *Cariboptila purpurea* were described by Sykora (in Botosaneanu and Sykora 1973), and details were mentioned for *Cariboptila guajira* and *Cariboptila poquita* by Botosaneanu (1994b). Flint (1964a) also described the immature stages of *Cariboptila pedophila* (as *Campsiophora*) and noted that the larvae occur by the hundreds on the surfaces of rocks in riffles of clear, lowland streams. Four fossil species (in amber) are known from the Dominican Republic.

***arawak*** (Flint), 1968a:13 [Type locality: Jamaica, St. Andrew, Yallahs River, Chestervale; NMNH; ♂; in *Campsiophora*]. —Flint, 1968b:80 [checklist]. —Botosaneanu and Hyslop, 1998:7 [distribution]. —Botosaneanu, 2002:80 [distribution]. —Robertson and Holzenthal, 2013:29 [to *Cariboptila*].

**Distribution.** Jamaica.

***aurulenta*** Flint, 1974a:7 [Type locality: Dominican Republic, Convento, 12 km S of Constanza; NMNH; ♂]. —Botosaneanu, 1996:8 [distribution]. —Flint and Pérez-Gelabert, 1999:36 [checklist]. —Botosaneanu, 2002:80 [distribution]. —Flint and Sykora, 2004:5 [distribution]. —Pérez-Gelabert, 2008:298 [checklist].

**Distribution.** Dominican Republic.

***botosaneanui*** (Kumanski), 1987:4 [Type locality: Cuba, Pinar del Río, ca. 15 km SE from La Palma, spring region of Río El Caimito; NMSB; ♂; ♀; in *Cubanoptila*]. —Flint, 1996c:15 [checklist]. —Botosaneanu, 2002:81 [distribution]. —Naranjo López and González Lazo, 2005:149 [checklist]. —Robertson and Holzenthal, 2013:29 [to *Cariboptila*].

**Distribution.** Cuba.

***caab*** Botosaneanu, 1996:8 [Type locality: Dominican Republic, springbrooks in La Descubierta, north shore of Lake Enriquillo; ZMUA; ♂; ♀]. —Flint and Pérez-Gelabert, 1999:36 [checklist]. —Botosaneanu, 2002:80 [distribution]. —Flint and Sykora, 2004:5 [distribution]. —Pérez-Gelabert, 2008:299 [checklist].

**Distribution.** Dominican Republic.

***calcigena*** Flint, 1974a:8 [Type locality: Dominican Republic, La Palma, 12 km E of El Río; NMNH; ♂]. —Flint and Pérez-Gelabert, 1999:36 [checklist]. —Botosaneanu, 2002:81 [distribution]. —Flint and Sykora, 2004:6 [distribution]. —Pérez-Gelabert, 2008:299 [checklist].

**Distribution.** Dominican Republic.

***cubana*** (Sykora), in Botosaneanu and Sykora, 1973:384 [Type locality: Cuba, Mogotes de Viñales (Pinar del Río); IZAC; ♂; ♀; larva; pupa; in *Cubanoptila*]. —Botosaneanu, 1979:43 [distribution]. —Botosaneanu, 1980:93 [distribution]. —Flint, 1996c:15 [checklist]. —Botosaneanu, 2002:81 [distribution; correction of figure]. —López del Castillo et al., 2004:229 [distribution]. —Naranjo López and González Lazo, 2005:149 [checklist]. —Robertson and Holzenthal, 2013:29 [♂; to *Cariboptila*].

**Distribution.** Cuba.

† ***grimaldii*** (Wichard), 1995a:160 [Type locality: Dominican Republic; AMNH; ♂; in amber; in *Cubanoptila*]. —Flint and Pérez-Gelabert, 1999:36 [checklist]. —Botosaneanu, 2002:81 [checklist]. —Pérez-Gelabert, 2008:299 [checklist]. —Robertson and Holzenthal, 2013:28 [to *Cariboptila*, by synonymy of *Cubanoptila*].

**Distribution.** Dominican Republic.

***guajira*** Botosaneanu, 1977:244 [Type locality: Cuba, Oriente, Massif de Gran Piedra, Arroyos de la Idalia; ZMUA; ♂]. —Botosaneanu, 1979:43 [distribution]. —Botosaneanu, 1994b:453 [larva]. —Flint, 1996c:15 [checklist]. —Botosaneanu, 2002:81 [distribution]. —Naranjo López and González Lazo, 2005:149 [checklist].

**Distribution.** Cuba.

***hispaniolica*** Flint, 1974a:8 [Type locality: Dominican Republic, La Palma, 12 km E of El Río; NMNH; ♂]. —Botosaneanu, 1996:8 [distribution]. —Flint and Pérez-Gelabert, 1999:36 [checklist]. —Botosaneanu, 2002:81 [distribution]. —Flint and Sykora, 2004:6 [distribution]. —Pérez-Gelabert, 2008:299 [checklist].

**Distribution.** Dominican Republic.

***jamaicensis*** Flint, 1968a:16 [Type locality: Jamaica, St. Andrew, Hope River near Newcastle at milepost 16.5; NMNH; ♂]. —Flint, 1968b:79 [checklist]. —Botosaneanu and Hyslop, 1998:7 [distribution]. —Botosaneanu, 2002:81 [distribution].

**Distribution.** Jamaica.

† ***longiscapa*** (Wichard), 2007a:7 [Type locality: Dominican Republic; SMNS; ♂; in amber; in *Cubanoptila*]. —Robertson and Holzenthal, 2013:28 [to *Cariboptila*, by synonymy of *Cubanoptila*].

**Distribution.** Dominican Republic.

***madremia*** (Botosaneanu), 1977:239 [Type locality: Cuba, Oriente, Baracoa, Rio Yumuri; ZMUA; ♂; ♀; in *Cubanoptila*]. —Botosaneanu, 1979:43 [distribution]. —Flint, 1996c:15 [checklist]. —Botosaneanu, 2002:81 [distribution]. —Naranjo López and González Lazo, 2005:149 [checklist]. —Robertson and Holzenthal, 2013:29 [to *Cariboptila*].

**Distribution.** Cuba.

***mathisi*** Flint and Sykora, 2004:6 [Type locality: Dominican Republic, Monseñor Nouel Province, 8.7 km W Bonao [jct. Carretera Duarte and rt. 12], 19°01.8'N, 70°29.4'W, 890 m; NMNH; ♂]. —Pérez-Gelabert, 2008:299 [checklist].

**Distribution.** Dominican Republic.

† ***mederi*** (Wichard), 1989:93 [Type locality: Dominican Republic; NMNH; ♂; in amber; in *Cubanoptila*]. —Flint and Pérez-Gelabert, 1999:36 [checklist]. —Botosaneanu, 2002:81 [checklist]. —Wichard, 2007a:8 [♂]. —Pérez-Gelabert, 2008:299 [checklist]. —Robertson and Holzenthal, 2013:28 [to *Cariboptila*, by synonymy of *Cubanoptila*].

**Distribution.** Dominican Republic.

***mulata*** (Botosaneanu), 1977:248 [Type locality: Cuba, Oriente, Baire, Rio Mogote; ZMUA; ♂; in *Campsiophora*]. —Botosaneanu, 1979:44 [distribution]. —Botosaneanu, 1994b:453 [larva]. —Flint, 1996c:15 [checklist]. —Botosaneanu, 2002:80 [distribution]. —López del Castillo et al., 2004:229 [distribution]. —Naranjo López and González Lazo, 2005:149 [checklist]. —López del Castillo et al., 2007:171 [distribution; seasonal abundance]. —Robertson and Holzenthal, 2013:29 [to *Cariboptila*].

**Distribution.** Cuba.

***muybonita*** (Botosaneanu), 1977:237 [Type locality: Cuba, Oriente, Baracoa, Rio Sabanilla; ZMUA; ♂; ♀; in *Cubanoptila*]. —Botosaneanu, 1979:43 [distribution]. —Botosaneanu, 1994b:453 [larva]. —Flint, 1996c:15 [checklist]. —Botosaneanu, 2002:81 [distribution]. —Naranjo López and González Lazo, 2005:149 [checklist]. —Robertson and Holzenthal, 2013:29 [to *Cariboptila*].

**Distribution.** Cuba.

***orophila*** Flint, 1964a:17 [Type locality: Puerto Rico, El Semil, near Villalba; NMNH; ♂]. —Flint, 1968b:79 [checklist]. —Flint and Masteller, 1993:140 [biology]. —Botosaneanu, 2002:81 [distribution].

**Distribution.** Puerto Rico.

***paradoxa*** Flint and Sykora, 2004:6 [Type locality: Dominican Republic, Independencia Province, La Descubierta, 18°34.1'N, 71°43.8, 0 m; NMNH; ♂]. —Pérez-Gelabert, 2008:299 [checklist].

**Distribution.** Dominican Republic.

***pedophila*** (Flint), 1964a:15 [Type locality: Puerto Rico, Maricao; NMNH; ♂; in *Campsiophora*]. —Flint, 1968b:80 [checklist]. —Botosaneanu, 1991a:116 [distrubution]. —Botosaneanu, 1996:6 [distribution]. —Flint and Pérez-Gelabert, 1999:36 [checklist]. —Botosaneanu, 2002:80 [distribution]. —Flint and Sykora, 2004:5 [distribution]. —Pérez-Gelabert, 2008:298 [checklist]. —Robertson and Holzenthal, 2013:29 [♂; to *Cariboptila*].

—*areopagita* (Malicky and Silalom), 2012:22 [Type locality: Thailand, Prov. Mae Hong Son, Muang Pai Resort, 19°23'N, 98°23'E, 730 m; Collection Malicky; ♂; in *Muangpaipsyche*]. —Robertson and Holzenthal, 2013:29 [to synonymy, distribution in question]. —Malicky, 2014:45 [good species].

**Distribution.** Dominican Republic, Haiti, Puerto Rico, Thailand [?].

† ***poinari*** (Wichard), 1989:92 [Type locality: Dominican Republic; UCB; ♂; in amber; in *Cubanoptila*]. —Flint and Pérez-Gelabert, 1999:36 [checklist]. —Botosaneanu, 2002:81 [checklist]. —Wichard, 2007a:8 [♂]. —Pérez-Gelabert, 2008:299 [checklist]. —Robertson and Holzenthal, 2013:28 [to *Cariboptila*, by synonymy of *Cubanoptila*].

**Distribution.** Dominican Republic.

***poquita*** Botosaneanu, 1977:242 [Type locality: Cuba, Oriente, Baracoa, Rio Yumuri; ZMUA; ♂]. —Botosaneanu, 1979:43 [distribution]. —Botosaneanu, 1980:92 [distribution]. —Botosaneanu, 1994b:453 [larva]. —Flint, 1996c:15 [checklist]. —Botosaneanu, 2002:81 [distribution]. —Naranjo López and González Lazo, 2005:149 [checklist].

**Distribution.** Cuba.

***purpurea*** (Sykora), in Botosaneanu and Sykora, 1973:387 [Type locality: Cuba, Río Cañas, Río Frio, El Cobre (Oriente); IESHC; ♂; ♀; larva; pupa; case; in *Cubanoptila*]. —Botosaneanu, 1979:43 [distribution]. —Botosaneanu, 1980:91 [distribution]. —Kumanski 1987:5 [♀; distribution]. —Flint, 1996c:15 [checklist]. —Botosaneanu, 2002:81 [distribution; correction of figure]. —López del Castillo et al., 2004:229 [distribution]. —Naranjo López and González Lazo, 2005:149 [checklist]. —López del Castillo et al., 2007:171 [distribution; seasonal abundance]. —Robertson and Holzenthal, 2013:29 [to *Cariboptila*].

**Distribution.** Cuba.

***soltera*** Botosaneanu, 1977:246 [Type locality: Cuba, Pinar del Rio, Soroa, Rio Manatiales; ZMUA; ♂]. —Botosaneanu, 1979:43 [distribution]. —Botosaneanu, 1980:96 [♀; distribution]. —Kumanski 1987:6 [distribution]. —Flint, 1996c:15 [checklist]. —Botosaneanu, 2002:81 [distribution]. —Naranjo López and González Lazo, 2005:149 [checklist].

**Distribution.** Cuba.

***tridens*** (Botosaneanu), in Botosaneanu and Hyslop, 1998:7 [Type locality: Jamaica, Rio Munho in its upper reach at Grantham, a few km. W. of Frankfield, Clarendon; ZMUA; ♂; /female; in *Cubanoptila*]. —Botosaneanu and Hyslop, 1999:325 [♂; distribution]. —Botosaneanu, 2002:81 [distribution; population differences]. —Robertson and Holzenthal, 2013:29 [to *Cariboptila*].

**Distribution.** Jamaica.

***trispinata*** Flint, 1992c:380 [Type locality: Puerto Rico, El Verde Field Station, Quebrada Prieta; NMNH; ♂]. —Flint and Masteller, 1993:140 [biology]. —Botosaneanu, 2002:81 [distribution].

**Distribution.** Puerto Rico.

#### Genus *Culoptila* Mosely [23 + †1]

*Culoptila* Mosely, 1954:336 [Type species: *Culoptila aluca* Mosely, 1954, original designation]. —Blahnik and Holzenthal, 2006:1 [revision, key to species]. —Robertson and Holzenthal, 2013:1, 30 [review of genus, phylogeny, diagnosis; key to Protoptilinae genera].

The genus was revised by Blahnik and Holzenthal (2006). Twenty-six species are known, 3 exclusively from north of Mexico - *Culoptila cantha* (Ross), *Culoptila kimminsi* Denning, *Culoptila plummerensis* Blahnik and Holzenthal) - and 23 from southwestern US and Mexico through Central America to Costa Rica and Panama. A fossil species was described from Mexican amber (Wichard et al., 2006), one of the first caddisflies described from these mid-Miocene inclusions. Wiggins (1996) described the larva of *Culoptila moselyi* from Arizona, and Houghton and Stewart (1998b) described all 5 larval instars, the pupa, and the case of *Culoptila cantha* from Texas. Larvae seem to prefer larger rivers (Wiggins 1996). Blahnik and Holzenthal (2006) figured a larva and cases from Costa Rica. Houghton and Stewart (1998a) provided a detailed life history study and larval descriptions of *Culoptila cantha*, from Texas; some of the Mexican species may have similar life histories and habitat preferences.

***acaena*** Bueno-Soria and Santiago-Fragoso, 1996a:448 [Type locality: Mexico, Guerrero, carretera 130, 80 km NW from Zihuatanejo; IBUNAM; ♂]. —Blahnik and Holzenthal, 2006:11 [♂; redescription].

**Distribution.** Mexico.

† ***aguilerai*** Wichard, Solórzano-Kraemer and Luer, 2006:39 [Type locality: Chiapas, Simojovel de Allende, approximately 50 km from Tuxtla Gutiérrez, 17°08'19"N, 92°42'00"W, 600 m; IHNEC; ♂; in amber].

**Distribution.** Mexico.

***aluca*** Mosely, 1954:337 [Type locality: Mexico, Caracuaro; BMNH; ♂]. —Bueno-Soria and Flint, 1978:192 [distribution]. —Blahnik and Holzenthal, 2006:13 [♂; redescription].

**Distribution.** Mexico.

***amberia*** Mosely, 1954:338 [Type locality: Mexico, Liquidamber; BMNH; ♂]. —Bueno-Soria and Flint, 1978:192 [distribution]. —Blahnik and Holzenthal, 2006:14 [♂; redescription]. —Bueno-Soria and Barba-Álvarez, 2011:354 [checklist].

**Distribution.** Mexico.

***azulae*** Bueno-Soria and Santiago-Fragoso, 1996a:451 [Type locality: Mexico, Chiapas, Reserva Montes Azules; IBUNAM; ♂]. —Blahnik and Holzenthal, 2006:15 [♂; redescription]. —Bueno-Soria and Barba-Álvarez, 2011:354 [checklist].

**Distribution.** Mexico.

***barrerai*** Bueno-Soria and Santiago-Fragoso, 1996a:448 [Type locality: Mexico, Oaxaca, Pochutla, Finca Progreso; IBUNAM; ♂]. —Blahnik and Holzenthal, 2006:17 [♂; redescription].

**Distribution.** Mexico.

***bidentata*** Blahnik and Holzenthal, 2006:17 [Type locality: Costa Rica, Alajuela, Río Pizote, ca. 5 km N Dos Rios, 10°56'53"N, 85°17'28"W, 470 m; UMSP; ♂].

**Distribution.** Costa Rica.

***buenoi*** Blahnik and Holzenthal, 2006:19 [Type locality: Mexico, Puebla, 26 km N Xicotepec de Juárez, km 93, Tra 130; UNAM; ♂].

**Distribution.** Mexico.

***cascada*** Blahnik and Holzenthal, 2006:22 [Type locality: Costa Rica, Cartago, Reserva Tapantí, Quebrada Palmitos & falls, ca. 9 km (road) NW tunnel, 9°43'12"N, 83°46'48"W, 1400 m; UMSP; ♂].

**Distribution.** Costa Rica.

***costaricensis*** Flint, 1974a:9 [Type locality: Costa Rica, Cartago, Ojo de Agua, route 2, km 75; NMNH; ♂]. —Holzenthal, 1988c:54 [distribution]. —Blahnik and Holzenthal, 2006:23 [♂; redescription].

**Distribution.** Costa Rica.

***denningi*** Bueno-Soria and Santiago-Fragoso, 1996a:451 [Type locality: Mexico, Guerrero, Ruta 130, 80 km NW from Zihuatanejo; IBUNAM; ♂]. —Blahnik and Holzenthal, 2006:24 [♂; redescription]. —Bueno-Soria et al., 2007:32 [distribution].

**Distribution.** Mexico.

***hamata*** Blahnik and Holzenthal, 2006:26 [Type locality: Costa Rica, Alahuela, Río Toro, 3.0 km (road) SE Bajos del Toro, 10°12'14"N, 85°18'58"W, 1530 m; UMSP; ♂].

**Distribution.** Costa Rica.

***jamapa*** Bueno-Soria and Santiago-Fragoso, 1996a:446 [Type locality: Mexico, Veracruz, Río Jamapa; NMNH; ♂]. —Rojas-Ascencio, et al., 2002:377 [distribution]. —Blahnik and Holzenthal, 2006:28 [♂; redescription].

**Distribution.** Mexico.

***montanensis*** Flint, 1967b:2 [Type locality: Guatemala, El Progreso, Finca La Cajeta; NMNH; ♂]. —Blahnik and Holzenthal, 2006:30 [♂; redescription].

**Distribution.** Guatemala.

***moselyi*** Denning, 1965c:269 [Type locality: U.S.A., Arizona, Apache County, Greer, White Mountains (near Springerville), 8,00 ft.; CAS; ♂; female]. —Houghton, 2001:90 [distribution]. —Blinn and Ruiter, 2006:332 [biology]. —Blahnik and Holzenthal, 2006:31 [♂; redescription]. —Bueno-Soria et al., 2007:32 [distribution]. —Blinn and Ruiter, 2009b:185 [phenology, distribution].

**Distribution.** Mexico, U.S.A.

***nahuatl*** Flint, 1974a:8 [Type locality: Mexico, Veracruz, Fortín de las Flores; NMNH; ♂]. —Bueno-Soria and Flint, 1978:192 [distribution]. —Blahnik and Holzenthal, 2006:32 [♂; redescription].

**Distribution.** Mexico.

***pararusia*** Blahnik and Holzenthal, 2006:34 [Type locality: Mexico, Chiapas, trib. to Rio de Teapa on Mex. 195, 1.5 mi. N Ixhuatan; NMNH; ♂]. —Bueno-Soria and Barba-Álvarez, 2011:354 [checklist].

**Distribution.** Mexico.

***rusia*** Mosely, 1954:341 [Type locality: Mexico, La Prusia; BMNH; ♂]. —Bueno-Soria and Flint, 1978:192 [distribution]. —Blahnik and Holzenthal, 2006:38 [♂; redescription]. —Bueno-Soria and Barba-Álvarez, 2011:354 [checklist].

**Distribution.** Guatemala, Mexico.

***saltena*** Mosely, 1954:342 [Type locality: Mexico, Huixtla; BMNH; ♂]. —Bueno-Soria and Flint, 1978:192 [distribution]. —Maes, 1999:1192 [checklist]. —Blahnik and Holzenthal, 2006:39 [♂; redescription]. —Chamorro-Lacayo et al., 2007:40 [checklist]. —Bueno-Soria and Barba-Álvarez, 2011:354 [checklist].

**Distribution.** Guatemala, Honduras, Mexico, Nicaragua.

***tapanti*** Blahnik and Holzenthal, 2006:40 [Type locality: Costa Rica, Cartago, Reserva Tapantí, Río Grande de Orosí, 9°41'10"N, 83°45'22"W, 1650 m; UMSP; ♂].

**Distribution.** Costa Rica.

***tarascanica*** Flint, 1974a:9 [Type locality: Mexico, Michoacan, Carapan, route 15, km 431; NMNH; ♂]. —Blahnik and Holzenthal, 2006:42 [♂; redescription].

**Distribution.** Mexico.

***thoracica*** (Ross), 1938b:114 [Type locality: U.S.A.: Boulder, Wyoming, along tributary of Big Piney River; INHS; ♂; in *Protoptila*]. —Blahnik and Holzenthal, 2006:42 [♂; redescription; distribution]. —Blinn and Ruiter, 2006:329, 332 [ecology].

**Distribution.** Mexico, U.S.A.

***unispina*** Blahnik and Holzenthal, 2006:44 [Type locality: Costa Rica, Puntarenas, Río Bellavista, ca 1.5 km NW Las Alturas, 8°57'04"N, 82°50'46"W, 1400 m; UMSP; ♂; larval case]. —Armitage et al., 2015b:6 [checklist]. —Armitage and Cornejo, 2015:194 [checklist].

**Distribution.** Costa Rica, Panama.

***vexillifera*** Blahnik and Holzenthal, 2006:45 [Type locality: Guatemala, Chimaltenango; NMNH; ♂].

**Distribution.** Guatemala.

#### Genus *Glossosoma* Curtis [1]

*Glossosoma* Curtis, 1834:216 [Type species: *Glossosoma boltoni* Curtis, 1834, monotype]. —Ross, 1956a:127 [revision].

This is a large genus of primarily Holarctic and Oriental species. One western North American species, *Glossosoma ventrale* Banks, extends southward into northern Mexico. Larvae of the genus have been described a number of times (Wiggins 1996). They feed on periphyton and detritus, which they scrape from the surfaces of rocks and other substrates in rapid, cool streams.

***ventrale*** Banks, 1904a:109 [Type locality: United States, New Mexico, East Las Vegas; MCZ; ♂]. —Ross, 1956a:153 [redescription]. —Flint, 1967d:165 [distribution]. —Bueno-Soria and Flint, 1978:192 [distribution]. —Blinn and Ruiter, 2009a:302 [biology]. —Blinn and Ruiter, 2009b:185 [phenology, distribution].

**Distribution.** Mexico, U.S.A.

#### Genus *Itauara* Müller [22]

*Itauara* Müller, 1888:275 [Type species: *Antoptila brasiliana* Mosely, 1939a, subsequent selection of Flint et al., 1999a]. —Robertson and Holzenthal, 2011:46 [revision, key to species]. —Robertson and Holzenthal, 2013:1, 31 [review of genus, phylogeny, diagnosis; key to Protoptilinae genera].

*Antoptila* Mosely, 1939a:219 [Type species: *Antoptila brasiliana* Mosely, 1939a, original designation]. —Angrisano, 1993:59 [larval and adult characterization]. —Flint et al., 1999a:74 [to synonymy].

Robertson and Holzenthal (2011) revised this exclusively South American genus, adding 18 new species to the 4 previously known. Species are now known from Venezuela, Guyana, Peru, Brazil, Argentina, and Uruguay. The immature stages of the type species were described by Angrisano (1993). They were found in a sandy bottom stream with scarce vegetation, mostly of Characeae algae on whose stems the pupae were fixed.

***alexanderi*** Robertson and Holzenthal, 2011:50 [Type locality: Brazil, Nova Friburgo, 22°16'00"S, 042°31'59” W, 950 m; NMNH; ♂]. —Paprocki and França, 2014:9 [checklist].

**Distribution.** Brazil.

***amazonica*** (Flint), 1971c:13 [Type locality: Brazil [Edo. Amazonas], Rio Marauiá, Endstation langer Cachoeira, Fluss tritt hier aus dem Gebirge mit starkem Gefálle; NMNH; ♂; in *Antoptila*]. —Angrisano, 1999:29 [checklist]. —Flint et al., 1999a:74 [to *Itauara*]. —Paprocki et al., 2004:6 [checklist]. —Robertson and Holzenthal, 2011:52 [♂; redescription]. —Paprocki and França, 2014:9 [checklist].

**Distribution.** Brazil.

***bidentata*** Robertson and Holzenthal, 2011:54 [Type locality: Guyana, Kumu, 25 km SE Lethem, 3°15'54"N, 59°43'36” W; NMNH; ♂].

**Distribution.** Guyana.

***blahniki*** Robertson and Holzenthal, 2011:56 [Type locality: Brazil, São Paulo, Estação Biológica Boraceia, Rio Guaratuba, 23°40'02"S, 45°53'46"W, 775 m; MZUSP; ♂]. —Paprocki and França, 2014:9 [checklist].

**Distribution.** Brazil.

***brasiliana*** (Mosely), 1939a:220 [Type locality: Brazil, Santa Catarina, Nova Teutonia; BMNH; ♂; in *Antoptila*]. —Angrisano, 1993:59 [♀; larva; pupa; case; distribution]. —Angrisano, 1997b:58 [distribution]. —Angrisano, 1999:29 [checklist]. —Flint et al., 1999a:74 [to *Itauara*]. —Paprocki et al., 2004:6 [checklist]. —Angrisano and Sganga, 2007:22 [♂; distribution]. —Robertson and Holzenthal, 2011:58 [♂; redescription]. —Manzo et al., 2014:166 [distribution]. —Paprocki and França, 2014:9 [checklist].

**Distribution.** Argentina, Brazil, Uruguay.

***charlotta*** Robertson and Holzenthal, 2011:60 [Type locality: Brazil, Minas Gerais, Serra do Cipó, Cardeal Mota, Cachoeira Veu da Noiva, 19°18'55"S, 43°36'16"W, 800 m; MZUSP; ♂]. —Paprocki and França, 2014:9 [checklist].

**Distribution.** Brazil.

***emilia*** Robertson and Holzenthal, 2011:62 [Type locality: Brazil, São Paulo, Estação Biológica Boraceia, Rio Coruja, 23°40'06"S, 45°53'57"W, 850 m; MZUSP; ♂]. —Paprocki and França, 2014:9 [checklist].

**Distribution.** Brazil.

***flinti*** Robertson and Holzenthal, 2011:65 [Type locality: Brazil, São Paulo, Parque Estadual de Campos do Jordão, Rio Galharada, 22°41'40"S, 45°27'47"W, 1530 m; MZUSP; ♂]. —Paprocki and França, 2014:9 [checklist].

**Distribution.** Brazil.

***guarani*** (Angrisano), 1993:57 [Type locality: Argentina, Misiones, Dto. Belgrano, río Urugua-í; MACN; ♂; ♀; in *Antoptila*]. —Angrisano, 1999:29 [checklist]. —Flint et al., 1999a:74 [to *Itauara*]. —Robertson and Holzenthal, 2011:67 [♂; redescription].

**Distribution.** Argentina.

***guyanensis*** Robertson and Holzenthal, 2011:69 [Type locality: Guyana, Dubulay Ranch, Warniabo Cr., 5°39'48"N, 57°53'24"W [sic E]; NMNH; ♂].

**Distribution.** Guyana.

***jamesii*** Robertson and Holzenthal, 2011:71 [Type locality: Brazil, Minas Gerais, trib. to Rio do Salto, Ibitipoca, Fazenda Engenho, 21°44'06"S, 43°53'56"W, 875 m; MZUSP; ♂]. —Paprocki and França, 2014:9 [checklist].

**Distribution.** Brazil.

***julia*** Robertson and Holzenthal, 2011:73 [Type locality: Brazil, Rio de Janeiro, Parque Nacional do Itatiaia, Rio Campo Belo, trail to Veu da Noiva, 22°25'42"S, 44°37'10"W, 1310 m; MZUSP; ♂; adult habitus]. —Dumas and Nessimian, 2012:9 [distribution]. —Paprocki and França, 2014:10 [checklist].

**Distribution.** Brazil.

***lucinda*** Robertson and Holzenthal, 2011:75 [Type locality: Brazil, Minas Gerais, Parque Nacional do Caparaó, small trib. to Rio Caparaó, Vale Verde, 20°25'02"S, 41°, 50'46"W, 1350 m; MZUSP; ♂]. —Paprocki and França, 2014:10 [checklist].

**Distribution.** Brazil.

***ovis*** Robertson and Holzenthal, 2011:77 [Type locality: Guyana, Kanuku Mountains, Kumu River & Falls, 3°15'54"N, 59°43'30"W; NMNH; ♂].

**Distribution.** Guyana, Venezuela.

***peruensis*** Robertson and Holzenthal, 2011:79 [Type locality: Peru, Madre de Dios, Manu Biosphere Reserve, Pakitza Biological Station, Trail 2, 1st stream, 12°07'00"S, 70°58'00"W, 250 m; NMNH; ♂].

**Distribution.** Peru.

***plaumanni*** (Flint), 1974a:7 [Type locality: Brazil, Santa Catarina, Nova Teutonia; NMNH; ♂; in *Antoptila*]. —Angrisano, 1993:59 [♀; distribution]. —Angrisano, 1997b:58 [distribution]. —Angrisano, 1999:30 [checklist]. —Flint et al., 1999a:74 [to *Itauara*]. —Paprocki et al., 2004:6 [checklist]. —Robertson and Holzenthal, 2011:81 [♂; redescription]. —Paprocki and França, 2014:10 [checklist].

**Distribution.** Argentina, Brazil, Uruguay.

***rodmani*** Robertson and Holzenthal, 2011:83 [Type locality: Brazil, Minas Gerais, Corrego das Aguas Pretas & tribs., ca. 15 km S Aiuruoca, 22°03'42"S, 44°38'14"W, 1386 m; MZUSP; ♂]. —Paprocki and França, 2014:10 [checklist].

**Distribution.** Brazil.

***simplex*** Robertson and Holzenthal, 2011:86 [Type locality: Brazil, São Paulo, Parque Nacional da Serra da Bocaina, Cachoeira dos Posses, 22°46'26"S, 44°36'15"W, 1250 m; MZUSP; ♂]. —Paprocki and França, 2014:10 [checklist].

**Distribution.** Brazil.

***spiralis*** Robertson and Holzenthal, 2011:88 [Type locality: Guyana, Paramakatoi, 4°42'00"N, 59°42'48"W; NMNH; ♂].

**Distribution.** Guyana.

***stella*** Robertson and Holzenthal, 2011:90 [Type locality: Brazil, São Paulo, Estação Biológica Boraceia, Rio Venerando & tribs, 23°39'11"S, 45°53'25"W, 850 m; UZUSP; ♂]. —Paprocki and França, 2014:10 [checklist].

**Distribution.** Brazil.

***tusci*** Robertson and Holzenthal, 2011:92 [Type locality: Brazil, Rio de Janeiro, Rio das Flores, Macaé de Cima, 10 km SE Mury, 1000 m; MZUSP; ♂]. —Paprocki and França, 2014:10 [checklist].

**Distribution.** Brazil.

***unidentata*** Robertson and Holzenthal, 2011:94 [Type locality: Guyana, Kanuku Mountains, Kumu River & Falls, 3°15'54"N, 59°43'30"W; NMNH; ♂].

**Distribution.** Guyana.

#### Genus *Mastigoptila* Flint [9]

*Mastigoptila* Flint, 1967a:49 [Type species: *Mastigoptila curvicornuta* Flint, 1967a, original designation]. —Schmid, 1958b:191 [diagnosis of Chilean species, as *Antoptila*]. —Robertson and Holzenthal, 2013:1, 33 [review of genus, phylogeny, diagnosis; key to Protoptilinae genera].

The 9 species of *Mastigoptila* are known only from the Chilean Subregion of the Neotropics. Valverde and Miserendino (1998) fully described the larva and pupa of *Mastigoptila longicornuta*, and presented data on its population densities and physical characteristics of the stream. Adults are commonly taken at light near fast-flowing rivers and streams.

***bicornuta*** (Schmid), 1958b:192 [Type locality: Chile, Ñuble, Tregualemu; NMNH; ♂; in *Antoptila*]. —Flint 1967a:50 [to *Mastigoptila*]. —Flint, 1974e:86 [checklist]. —Angrisano, 1999:30 [checklist].

**Distribution.** Chile.

***brevicornuta*** (Schmid), 1958b:192 [Type locality: Chile, Chiloé, Aulen; NMNH; ♂; in *Antoptila*]. —Flint 1967a:50 [to *Mastigoptila*, distribution]. —Flint, 1974e:86 [checklist]. —Angrisano, 1999:30 [checklist].

**Distribution.** Chile.

***complicornuta*** Holzenthal, 2004:111 [Type locality: Chile, VIII Región del Bío-Bío, Bío-Bío, small trib. to Río Queco, 5 km E Ralco, 37°51.619'S, 71°36.257'W, el. 500 m; UMSP; ♂].

**Distribution.** Chile.

***curvicornuta*** Flint, 1967a:50 [Type locality: Chile, Ñuble, Río Ñuble about 80 km. north of Los Angeles; NMNH; ♂]. —Flint, 1974e:86 [checklist]. —Angrisano, 1999:30 [checklist].

**Distribution.** Chile.

***ecornuta*** Flint, 1974a:9 [Type locality: Chile, Arauco, Caramavida; NMNH; ♂]. —Angrisano, 1999:30 [checklist]. —Flint, 1974e:86 [checklist].

**Distribution.** Chile.

***elae*** Holzenthal, 2004:113 [Type locality: Chile, VII Región del Araucania, Cautín, nr. Pucon; NMNH; ♂].

**Distribution.** Chile.

***longicornuta*** (Schmid), 1958b:193 [Type locality: Chile, Ñuble, Los Pellines; NMNH; ♂; in *Antoptila*]. —Flint 1967a:50, 51 [to *Mastigoptila*, distribution]. —Flint, 1974e:86 [checklist]. —Valverde and Miserendino, 1998:49 [larva; pupa; biology]. —Angrisano, 1999:30 [checklist]. —Miserendino and Brand, 2007:312 [biology]. —Brand and Miserendino, 2011a:29 [biology]. —Brand and Miserendino, 2014:6 [community ecology].

**Distribution.** Argentina, Chile.

***ruizi*** (Navás), 1933c:110 [Type locality: Chile, Bío-Bío; DEI; ♂; in *Mortoniella*]. —Flint, 1974e:83, 86 [to *Mastigoptila*, distribution]. —Flint, 1990:116 [♂ lectotype].

—*duplicicornuta* (Schmid), 1964:311 [Type locality: Chile, Liucura, Malleco; NMNH; ♂; in *Antoptila*]. —Flint 1967a:50 [to *Mastigoptila*, distribution]. —Flint, 1974e:86 [checklist]. —Flint, 1990:116 [to synonymy]. —Angrisano, 1999:30 [checklist].

**Distribution.** Argentina, Chile.

***ventricornuta*** Flint, 1967a:51 [Type locality: Chile, Valdivia, Río Llancahue; NMNH; ♂]. —Flint, 1974e:86 [checklist]. —Angrisano, 1999:30 [checklist].

**Distribution.** Chile.

#### Genus *Merionoptila* Schmid [1]

*Merionoptila* Schmid, 1959:477 [Type species: *Merionoptila wygodzinskyi* Schmid, 1959, original designation].

The immature stages are not known for this monotypic genus, but the biology of the adult was described by Schmid (1959). The small, brachypterous adults live on the surface of the water of small streams flowing through high elevation, xerophytic areas.

***wygodzinskyi*** Schmid, 1959:482 [Type locality: Argentina, Tucumán, Quebrada de Amaicha; NMNH; ♂]. —Angrisano, 1999:30 [checklist]. —Rueda Martín and Gibon, 2008:222 [checklist, distribution]. —Isa Miranda and Rueda Martín, 2014:199 [distribution].

**Distribution.** Argentina.

#### Genus *Mortoniella* Ulmer [97]

*Mortoniella* Ulmer, 1906:95 [Type species: *Mortoniella bilineata* Ulmer, 1906, by monotypy]. —Flint 1963a:465 [description], —Flint, 1996b:381 [key]. —Sykora 1999:377 [review]. —Blahnik and Holzenthal, 2008:1 [revision of Central American species]. —Blahnik and Holzenthal, 2011:1 [revision of austral South American species]. —Robertson and Holzenthal, 2013:1, 34 [review of genus, phylogeny, diagnosis; key to Protoptilinae genera].

*Mexitrichia* Mosely, 1937:158 [Type species: *Mexitrichia leroda* Mosely, 1937, original designation]. —Blahnik and Holzenthal, 2008:9 [to synonymy].

*Paraprotoptila* Jacquemart, 1963:342 [Type species: *Paraprotoptila armata* Jacquemart, 1963, by monotypy]. —Flint et al., 1999a:74 [to synonymy].

The 97 described species of *Mortoniella* are known from Mexico, Central, and South America as far south as Argentina and southeastern Brazil. Flint (1963a) and Botosaneanu and Alkins-Koo (1993) described the immature stages. They are commonly found in fast-flowing rivers and streams on stones and boulders; the adults come readily to lights placed near these habitats.

***acauda*** Blahnik and Holzenthal, 2011:8 [Type locality: Brazil, Santa Catarina, Urubici, Cachoeira Avencal, 28°02'50"S, 049°37'00"W, 1260 m; MZUSP; ♂; *leroda* species group, *albolineata* subgroup]. —Paprocki and França, 2014:11 [checklist].

**Distribution.** Brazil.

***aequalis*** (Flint), 1963a:472 [Type locality: Peru, Río Pichis, Puerto Bermudez; CU; ♂; in *Mexitrichia*]. —Blahnik and Holzenthal, 2008:70 [to *Mortoniella*; *ormina* species group].

**Distribution.** Peru.

***agosta*** Blahnik and Holzenthal, 2011:8 [Type locality, Brazil, Rio de Janeiro, Rio Macaé, Macaé de Cima, 22°23'41"S, 042°30'08"W, 1000 m; MZUSP; ♂; *leroda* species group, *albolineata* subgroup]. —Paprocki and França, 2014:11 [checklist].

**Distribution.** Brazil.

***akantha*** Blahnik and Holzenthal, 2008:11 [Type locality: Costa Rica, San José: Parque Nacional Braulio Carrillo, Quebrada Sanguijuela, 10°09'36"N, 83°57'47"W, 800 m; UMSP; ♂; *leroda* species group].

**Distribution.** Costa Rica.

***albolineata*** Ulmer, 1907a:44 [Type locality: Brazil, Sta. Catharina [sic]; PAN; ♀; ♂ unnoticed in series]. —Mosely 1939a:218 [from *Mortoniella* to *Antoptila*?, distribution]. —Flint 1963a:465. —Flint, 1966a:2 [to *Mexitrichia*, ♂ lectotype, *teutonia* erroneously to synonymy]. —Flint, 1972b:225 [distribution]. —Angrisano, 1999:30 [checklist]. —Paprocki et al., 2004:6 [checklist]. —Blahnik et al., 2004:4 [distribution]. —Rueda Martín and Gibon, 2008:223 [checklist, distribution]. —Blahnik and Holzenthal, 2008:69 [*leroda* species group]. —Calor, 2011:320 [checklist]. —Blahnik and Holzenthal, 2011:10 [♂; redescription; distribution; *albolineata* subgroup]. —Manzo et al., 2014:167 [distribution]. —Paprocki and França, 2014:11 [checklist].

**Distribution.** Argentina, Brazil, Uruguay.

***alicula*** Blahnik and Holzenthal, 2011:47 [Type locality: Brazil, Rio de Janeiro, Rio Macaé, Macaé de Cima, 22°23'41"S, 042°30'08"W, 1000 m; MZUSP; ♂; *ormina* species group]. —Paprocki and França, 2014:11 [checklist].

**Distribution.** Brazil.

***anakantha*** Blahnik and Holzenthal, 2008:14 [Type locality: Costa Rica, Puntarenas, Río Bellavista trib., Las Alturas, road to quarry, 08°57'07"N, 82°50'53"W, 1480 m; UMSP; ♂; *leroda* species group]. —Armitage et al., 2015b:6 [checklist]. —Armitage and Cornejo, 2015:194 [checklist].

**Distribution.** Costa Rica, Panama.

***angulata*** Flint, 1963a:468 [Type locality: Ecuador, stream 11 miles west of Pujilí; NMNH; ♂; larva; pupa]. —Sykora, 1999:386 [*bilineata* group]. —Blahnik and Holzenthal, 2008:70 [*bilineata* species group].

**Distribution.** Ecuador.

***apiculata*** Flint, 1963a:466 [Type locality: Ecuador, 1 mile east of Papallacta; NMNH; ♂; larva; pupa]. —Knutson and Flint, 1979:32 [Empididae predators in pupal cocoons]. —Sykora, 1999:386 [*bilineata* group]. —Blahnik and Holzenthal, 2008:70 [*bilineata* species group].

**Distribution.** Ecuador.

***argentinica*** Flint, 1974a:13 [Type locality: Argentina, Catamarca, N. Aconquija; NMNH; ♂]. —Angrisano, 1999:30 [checklist]. —Sykora, 1999:386 [*argentinica* group]. —Rueda Martín and Gibon, 2008:224 [checklist, distribution]. —Blahnik and Holzenthal, 2008:70 [*bilineata* species group]. —Blahnik and Holzenthal, 2011:67 [♂; redescription; unplaced]. —Isa Miranda and Rueda Martín, 2014:199 [distribution].

**Distribution.** Argentina.

***aries*** (Flint), 1963a:470 [Type locality: Ecuador, Napo-Pastaza Province, Río Chingual, 8 miles east of El Pun; NMNH; ♂; immature larva; pupa; in *Mexitrichia*]. —Blahnik and Holzenthal, 2008:70 [to *Mortoniella*; *ormina* species group].

**Distribution.** Ecuador, Peru.

***armata*** (Jacquemart), 1963:342 [Type locality: Argentina [San Juan Province], Rio Sasso; IRSNB; ♂; in *Paraprotoptila*]. —Angrisano, 1999:30 [checklist]. —Flint et al., 1999a:74 [to *Mexitrichia*, no specimen on type slide]. —Blahnik and Holzenthal, 2008:69 [to *Mortoniella*, *leroda* species group]. —Manzo et al., 2014:167 [distribution].

**Distribution.** Argentina.

***asymmetris*** Blahnik and Holzenthal, 2011:12 [Type locality: Paraguay, Amambay, Cerro Cora, Río Aquidaban; NMNH; ♂; *leroda* species group, *albolineata* subgroup]. —Souza et al., 2013a:3 [distribution]. —Paprocki and França, 2014:11 [checklist].

**Distribution.** Brazil, Paraguay.

***atenuata*** (Flint), 1963a:473 [Type locality: Peru, Río Pichis, Puerto Bermudez; CU; ♂; in *Mexitrichia*]. —Flint, 1996b:382 [discussion, distribution]. —Blahnik and Holzenthal, 2008:69 [to *Mortoniella*; *leroda* species group].

**Distribution.** Peru.

***aviceps*** Blahnik and Holzenthal, 2008:16 [Type locality: Costa Rica, Alajuela, Reserva Forestal San Ramón, Río San Lorencito & tribs., 10°12'58"N, 84°36'25"W, 980 m; UMSP; ♂; *leroda* species group]. —Armitage et al., 2015b:6 [checklist]. —Armitage and Cornejo, 2015:194 [checklist].

**Distribution.** Costa Rica, Panama.

***bifurcata*** Sykora, 1999:382 [Type locality: Venezuela, Yacambu, 1200 m; NMNH; ♂; *flinti* group]. —Blahnik and Holzenthal, 2008:70 [*bilineata* species group].

**Distribution.** Venezuela.

***bilineata*** Ulmer, 1906:97 [Type locality: Ecuador, Chimbo; RNH; ♂]. —Flint 1963a:466 [♂]. —Flint, 1991:22 [♂; distribution]. —Sykora 1999:386 [*bilineata* group]. —Muñoz-Quesada, 2000:275 [checklist]. —Blahnik and Holzenthal, 2008:70 [*bilineata* species group]. —Blahnik and Holzenthal, 2011:61 [female].

**Distribution.** Colombia, Ecuador.

***bocaina*** Blahnik and Holzenthal, 2011:55 [Type locality: Brazil, São Paulo, Parque Nacional da Serra da Bocaina, Cachoeira dos Posses, 22°46'26"S, 044°36'15"W, 1250 m; ♂; ♀; MZUSP; *velasquezi* species group]. —Paprocki and França, 2014:11 [checklist].

**Distribution.** Brazil.

***bolivica*** (Schmid), 1958b:193 [Type locality: Bolivia, Coroico; NMNH; ♂; in *Mexitrichia*]. —Angrisano, 1999:30 [checklist]. —Rueda Martín and Gibon, 2008:223 [checklist, distribution]. —Blahnik and Holzenthal, 2008:69 [to *Mortoniella*; *leroda* species group].

**Distribution.** Bolivia.

***brachyrhachos*** Blahnik and Holzenthal, 2008:18 [Type locality: Mexico, Oaxaca, Loxicha, Pluma Hidalgo, 450 m; ♂; IBUNAM; *leroda* species group].

**Distribution.** Mexico.

***buenoi*** Blahnik and Holzenthal, 2008:19 [Type locality: Mexico, Oaxaca, Loxicha, Pluma Hidalgo, 450 m; ♂; IBUNAM; *leroda* species group].

**Distribution.** Mexico.

***carinula*** Blahnik and Holzenthal, 2008:22 [Type locality: Costa Rica, Cartago, Reserva Tapantí, Quebrada Segunda @ administration building, 9°45'40"N, 83°47'13"W, 1250 m; UMSP; ♂; *leroda* species group].

**Distribution.** Costa Rica.

***catarinensis*** (Flint), 1974a:12 [Type locality: Brazil, Santa Catarina, Nova Teutonia; NMNH; ♂; in *Mexitrichia*]. —Angrisano, 1999:30 [checklist]. —Paprocki et al., 2004:6 [checklist]. —Blahnik and Holzenthal, 2008:70 [to *Mortoniella*; *ormina* species group]. —Blahnik and Holzenthal, 2011:49 [♂; redescription]. —Paprocki and França, 2014:11 [checklist].

**Distribution.** Brazil.

***caudicula*** Blahnik and Holzenthal, 2008:24 [Type locality: Costa Rica, Alajuela, Río Pizote, ca. 5 km (air) S Brasilia, 10°58'19"N, 85°20'42"W, 390 m; UMSP; ♂; *leroda* species group].

**Distribution.** Costa Rica.

***chicana*** Sykora, 1999:378 [Type locality: Eduador, Chin. [sic], Rio Chicana, 880 m, 5 km N Yanzantza; NMNH; ♂; *bilineata* group]. —Blahnik and Holzenthal, 2008:70 [*bilineata* species group].

**Distribution.** Ecuador.

***collegarum*** (Rueda Martín and Gibon), 2008:216 [Type locality: Bolivia, Tarija: O´Connor, Saladito Ríver, 21°18'28"S, 64°7'2.8"W, 900 m; ♂; IML; in *Mexitrichia*]. —Blahnik and Holzenthal, 2011:51 [♂; redescription; to *Mortoniella*, *ormina* species group].

**Distribution.** Argentina, Bolivia, Chile.

***crescentis*** Blahnik and Holzenthal, 2011:14 [Type locality: Brazil, Rio de Janeiro, Rio Campo Belo, trail to Veu da Noiva, 22°25'42"S, 044°37'10"W, 1310 m; MZUSP; ♂; *leroda* species group, *albolineata* subgroup]. —Dumas and Nessimian, 2012:10 [distribution]. —Paprocki and França, 2014:12 [checklist].

**Distribution.** Brazil.

***denticulata*** Sykora, 1999:382 [Type locality: Venezuela, Me. [sic], Rt. 4, 27 km W Merida; NMNH; ♂; *flinti* group]. —Blahnik and Holzenthal, 2008:70 [*bilineata* species group].

**Distribution.** Venezuela.

***dolonis*** Blahnik and Holzenthal, 2011:16 [Type locality: Brazil, São Paulo, Pedregulho, Riberão São Pedro, 20°09'07"S, 047°30'38"W, 617 m; MZUSP; ♂; *leroda* species group, *albolineata* subgroup]. —Paprocki and França, 2014:12 [checklist].

**Distribution.** Brazil.

***eduardoi*** (Rueda Martín and Gibon), 2008:216 [Type locality: Bolivia, La Paz, Yara River near Caranavi; ♂; IML; in *Mexitrichia*]. —Blahnik and Holzenthal, 2011:53 [to *Mortoniella*, *velasquezi* species group].

**Distribution.** Bolivia.

***elongata*** (Flint), 1963a:474 [Type locality: Colombia, Valle, Tablones, Finca la Florida; NMNH; ♂; in *Mexitrichia*]. —Flint, 1991:20 [♂; distribution]. —Muñoz-Quesada, 2000:275 [checklist]. —Blahnik and Holzenthal, 2008:69 [to *Mortoniella*; *leroda* species group].

**Distribution.** Colombia.

***enchrysa*** Flint, 1991:24 [Type locality: Colombia, Dpto. Risaralda, Termales de Santa Rosa de Cabal; NMNH; ♂]. —Sykora, 1999:386 [*enchrysa* group]. —Muñoz-Quesada, 2000:275 [checklist]. —Blahnik and Holzenthal, 2008:70 [*bilineata* species group].

**Distribution.** Colombia.

***falcicula*** Blahnik and Holzenthal, 2008:24 [Type locality: Mexico, Oaxaca, Puente Angel, Rt. 175, 1420 m; ♂; IBUNAM; *leroda* species group].

**Distribution.** Mexico.

***flinti*** Sykora, 1999:382 [Type locality: Venezuela, Est. Exp. Cataurito, ca. 32 km E Villa; NMNH; ♂; *flinti* group]. —Blahnik and Holzenthal, 2008:70 [*bilineata* species group].

**Distribution.** Venezuela.

***florica*** (Flint), 1974a:10 [Type locality: Mexico, Veracruz, Río Tacolapan, route 180, km 551; NMNH; ♂; in *Mexitrichia*]. —Bueno-Soria and Flint, 1978:192 [distribution]. —Maes, 1999:1192 [checklist]. —Bueno-Soria et al., 2005:75 [distribution]. —Chamorro-Lacayo et al., 2007:40 [checklist]. —Blahnik and Holzenthal, 2008:27, 69 [♂; redescription; distribution; to *Mortoniella*; *leroda* species group].

**Distribution.** Mexico, Nicaragua.

***foersteri*** (Schmid), 1964:311 [Type locality: Columbia, Cundinamarca, Monterredondo; NMNH; ♂; in *Mexitrichia*]. —Sykora, 1999:386 [to *Mortoniella*; *bilineata* group]. —Muñoz-Quesada, 2000:275 [checklist]. —Blahnik and Holzenthal, 2008:70 [*bilineata* species group].

**Distribution.** Colombia.

***froehlichi*** Blahnik and Holzenthal, 2011:55 [Type locality: Brazil, Rio de Janeiro, Parati, Riacho Perequê-açu, Sitio Cachoeira Grande, 23°13'14"S, 044°47'24"W, 120 m; ♂; ♀; MZUSP; *velasquezi* species group]. —Paprocki and França, 2014:12 [checklist].

**Distribution.** Brazil.

***guahybae*** Blahnik and Holzenthal, 2011: 19 [Type locality: Brazil, São Paulo, Parque Estadual de Campos do Jordão, Rio Galharada, 22°41'40"S, 045°27'47"W, 1530 m; MZUSP; ♂; *leroda* species group, *albolineata* subgroup]. —Paprocki and França, 2014:12 [checklist].

**Distribution.** Brazil.

***guairica*** (Flint), 1974a:12 [Type locality: Paraguay, Salto de Guaira; NMNH; ♂; in *Mexitrichia*]. —Angrisano, 1999:30 [checklist]. —Blahnik and Holzenthal, 2008:70 [to *Mortoniella*; *incertae sedis*]. —Blahnik and Holzenthal, 2011:67 [♂; redescription; unplaced].

**Distribution.** Paraguay.

***hodgesi*** Flint, 1963a:470 [Type locality: Ecuador, stream 5 miles south of Antisana; NMNH; ♂; larva; pupa]. —Sykora 1999:386 [*bilineata* group]. —Blahnik and Holzenthal, 2008:70 [*bilineata* species group].

**Distribution.** Ecuador.

***hystricosa*** Blahnik and Holzenthal, 2011:19 [Type locality: Brazil, Santa Catarina, Parque Ecológica Spitzkopf, confl. Rio Ouro & Rio Caeté, 27°00'21"S, 49°06'42"W, 140 m; MZUSP; ♂; *leroda* species group, *albolineata* subgroup]. —Paprocki and França, 2014:12 [checklist].

**Distribution.** Brazil.

***intervales*** Blahnik and Holzenthal, 2011:21 [Type locality: Brazil, São Paulo, Parque Estadual Intervales, Rio do Carmo, 24°18'59"S, 48°25'15"W, 560 m; MZUSP; ♂; *leroda* species group, *albolineata* subgroup]. —Paprocki and França, 2014:12 [checklist].

**Distribution.** Brazil.

***iridescens*** Flint, 1991:24 [Type locality: Colombia, Dpto. Antioquia, 12 km N Fredonia (road to Medellín); NMNH; ♂]. —Sykora 1999:386 [*bilineata* group]. —Muñoz-Quesada, 2000:275 [checklist]. —Blahnik and Holzenthal, 2008:70 [*bilineata* species group].

**Distribution.** Colombia.

***latispina*** Blahnik and Holzenthal, 2011:24 [Type locality: Brazil, Rio de Janeiro, Parque Nacional do Itatiaia, Rio Campo Belo, trail to Veu da Noiva, 22°25'42"S, 44°37'10"W, 1310 m; MZUSP; ♂; *leroda* species group, *albolineata* subgroup]. —Dumas and Nessimian, 2012:10 [distribution]. —Paprocki and França, 2014:12 [checklist].

**Distribution.** Brazil.

***leei*** (Flint), 1974a:12 [Type locality: Colombia, Valle, Río Raposo; NMNH; ♂; in *Mexitrichia*]. —Flint, 1991:21 [♂; distribution]. —Muñoz-Quesada, 2000:275 [checklist]. —Blahnik and Holzenthal, 2008:69 [to *Mortoniella*; *leroda* species group].

**Distribution.** Colombia.

***leroda*** (Mosely), 1937:158 [Type locality: Mexico, Chiapas, Barranca Honda; BMNH; ♂; in *Mexitrichia*]. —Bueno-Soria and Flint, 1978:193 [distribution]. —Maes, 1999:1192 [checklist]. —Chamorro-Lacayo et al., 2007:40 [checklist]. —Blahnik and Holzenthal, 2008:29, 69 [♂; redescription; to *Mortoniella*; *leroda* species group]. —Bueno-Soria and Barba-Álvarez, 2011:354 [checklist].

**Distribution.** Honduras, Mexico, Nicaragua.

***limona*** (Flint), 1981a:9 [Type locality: Venezuela, Aragua, Maracay, Río Limón, Estación Piscicultura; NMNH; ♂; in *Mexitrichia*]. —Blahnik and Holzenthal, 2008:69 [to *Mortoniella*; *leroda* species group].

**Distribution.** Venezuela.

***longispina*** Blahnik and Holzenthal, 2011:24 [Type locality: Brazil Santa Catarina, Urubici, Rio Canoas, road to Campo dos Padres, 28°00'15"S, 049°22'24"W, 1100 m; MZUSP; ♂; *leroda* species group, *albolineata* subgroup]. —Paprocki and França, 2014:12 [checklist].

**Distribution.** Brazil.

***macarenica*** (Flint), 1974a:11 [Type locality: Colombia, Meta, Refugio Macarena; NMNH; ♂; in *Mexitrichia*]. —Muñoz-Quesada, 2000:275 [checklist]. —Blahnik and Holzenthal, 2008:70 [to *Mortoniella*; *ormina* species group].

**Distribution.** Colombia.

***macuta*** (Botosaneanu), 1998:460 [Type locality: Venezuela, Macuto, Rio del Teleferico, about 12 km N from the northern limits of Caracas; ZMUA; ♂; in *Mexitrichia*]. —Blahnik and Holzenthal, 2008:69 [to *Mortoniella*; *leroda* species group].

**Distribution.** Venezuela.

***marini*** (Rueda Martín and Gibon), 2008:219 [Type locality: Bolivia, La Paz, small triburaty of the Unduavi River at Puente Villa, 16°24'03"S, 67°38'30"W; ♂; IML; in *Mexitrichia*]. —Blahnik and Holzenthal, 2011:45 [to *Mortoniella*, *leroda* species group, *punensis* subgroup].

**Distribution.** Bolivia.

***meloi*** Blahnik and Holzenthal, 2011:69 [Type locality: Brazil, São Paulo, Parque Estadual Intervales, Rio do Carmo, 24°18'59"S, 048°25'15"W, 560 m; MZUSP; ♂; unplaced]. —Paprocki and França, 2014:13 [checklist].

**Distribution.** Brazil.

***meralda*** (Mosely), 1954:342 [Type locality: Mexico, Huixtla; BMNH; ♂; in *Mexitrichia*]. —Bueno-Soria and Flint, 1978:193 [distribution]. —Holzenthal, 1988c:54 [distribution]. —Maes, 1999:1192 [checklist]. —Rojas-Ascencio, et al., 2002:377 [distribution]. —Bueno-Soria et al., 2007:32 [distribution]. —Chamorro-Lacayo et al., 2007:40 [checklist]. —Blahnik and Holzenthal, 2008:31 [♂; redescription; to *Mortoniella*; *leroda* species group]. —Bueno-Soria and Barba-Álvarez, 2011:354 [checklist].

**Distribution.** Costa Rica, Guatemala, Honduras, Mexico, Nicaragua.

***mexicana*** Blahnik and Holzenthal, 2008:34 [Type locality: Mexico, Puebla, Patla; NMNH; ♂; *leroda* species group].

**Distribution.** Mexico.

***munozi*** Blahnik and Holzenthal, 2008:34 [Type locality: Costa Rica, Cartago, Río Chitaría, rt 10, 10 km NW Río Reventazón, 9°55'12"N, 83°36'14"W, 740 m; UMSP; ♂; *leroda* species group]. —Armitage et al., 2015b:6 [checklist]. —Armitage and Cornejo, 2015:194 [checklist].

**Distribution.** Costa Rica, Panama.

***opinionis*** Blahnik and Holzenthal, 2008:37 [Type locality: Costa Rica, Cartago, Reserva Tapantí, Quebrada Palmitos & falls, ca. 9 km (road) NW tunnel, 9°43'12"N, 83°46'48"W, 1400 m; UMSP; ♂; *leroda* species group].

**Distribution.** Costa Rica.

***ormina*** (Mosely), 1939a:222 [Type locality: Brazil, Santa Catharina [sic], Nova Teutonia; BMNH; ♂; in *Mexitrichia*]. —Angrisano, 1999:30 [checklist]. —Paprocki et al., 2004:6 [checklist]. —Blahnik and Holzenthal, 2008:70 [to *Mortoniella*; *ormina* species group]. —Blahnik and Holzenthal, 2011:53 [♂; redescription]. —Paprocki and França, 2014:13 [checklist].

**Distribution.** Brazil.

***pacuara*** (Flint), 1974a:11 [Type locality: Costa Rica, San Jose, Río General, Pacuare (10 miles S of San Isidro); NMNH; ♂; in *Mexitrichia*]. —Holzenthal, 1988c:54 [distribution]. —Muñoz-Quesada, 2000:275 [checklist]. —Blahnik and Holzenthal, 2008:39, 70 [♂; redescription; to *Mortoniella*; *ormina* species group].

**Distribution.** Colombia, Costa Rica.

***panamensis*** Blahnik and Holzenthal, 2008:42 [Type locality: Panama, San Blas, Río Carti Grande, 2 km W Nusagandi; NMNH; ♂; *leroda* species group]. —Armitage et al., 2015b:6 [checklist]. —Armitage and Cornejo, 2015:194 [checklist].

**Distribution.** Panama.

***papillata*** Blahnik and Holzenthal, 2008:44 [Type locality: Costa Rica, Guanacaste, Parque Nacional Guanacaste, ca. 0.7 km N Est. Maritza, 10°57'36"N, 85°30'00"E, 550 m; UMSP; ♂; *leroda* species group].

**Distribution.** Costa Rica.

***paraenchrysa*** Sykora, 1999:381 [Type locality: Bolivia, La Paz, Coroico, 2200 m; NMNH; ♂; *enchrysa* group]. —Rueda Martín and Gibon, 2008:224 [checklist, distribution]. —Blahnik and Holzenthal, 2008:70 [*bilineata* species group].

**Distribution.** Bolivia.

***paraguaiensis*** Blahnik and Holzenthal, 2011:26 [Type locality: Paraguay, Alto Parana, SE Naranja, ca. 20 km S. Pto. Stroessner; NMNH; ♂; *leroda* species group, *albolineata* subgroup].

**Distribution.** Paraguay.

***paralineata*** Sykora, 1999:378 [Type locality: Eduador, Zamb.-Chin. [sic], Rio Jamboe, 1,340 m, 21 km S Zamora; NMNH; ♂; *bilineata* group]. —Blahnik and Holzenthal, 2008:70 [*bilineata* species group].

**Distribution.** Ecuador.

***parauna*** Blahnik and Holzenthal, 2011:28 [Type locality: Brazil, Minas Gerais, Rio Paraúna, 3 km S Santana do Riacho, 19°10'59"S, 043°43'29"W, 650 m; MZUSP; ♂; *leroda* species group, *albolineata* subgroup]. —Paprocki and França, 2014:13 [checklist].

**Distribution.** Brazil.

***paraunota*** Blahnik and Holzenthal, 2011:31 [Type locality: Brazil, Santa Catarina, Seara (Nova Teutônia), 27°11'S, 052°23'W, 300-500 m; MZUSP; ♂; *leroda* species group, *albolineata* subgroup]. —Paprocki and França, 2014:13 [checklist].

**Distribution.** Argentina, Brazil.

***pectinella*** Blahnik and Holzenthal, 2008:46 [Type locality: Panama, Chiriquí, Fortuna Dam Site nr. Hornitos, 1050 m; NMNH; ♂; *leroda* species group]. —Armitage et al., 2015b:6 [checklist]. —Armitage and Cornejo, 2015:194 [checklist].

**Distribution.** Panama.

***pocita*** (Flint), 1983a:8 [Type locality: Argentina, Pcia. Salta, Río Pescado, W Orán; NMNH; ♂; in *Mexitrichia*]. —Angrisano, 1999:30 [checklist]. —Rueda Martín and Gibon, 2008:223 [checklist, distribution]. —Blahnik and Holzenthal, 2008:69 [to *Mortoniella*; *leroda* species group]. —Blahnik and Holzenthal, 2011:39 [♂; redescription; *pocita* subgroup].

**Distribution.** Argentina, Bolivia.

***propinqua*** Blahnik and Holzenthal, 2008:46 [Type locality: Costa Rica, San José, Parque Nacional Braulio Carrillo, Quebrada Sanguijuela, 10°09'36"N, 83°57'47"W, 800 m; UMSP; ♂; *leroda* species group].

**Distribution.** Costa Rica.

***pumila*** Blahnik and Holzenthal, 2011:41 [Type locality: Brazil, Rio de Janeiro, Encontro dos Rios (Macaé/Bonito), 6 km S Lumiar, 22°23'29"S, 042°18'42"W, 600 m; MZUSP; ♂; *leroda* species group, *pumila* subgroup]. —Paprocki and França, 2014:13 [checklist].

**Distribution.** Brazil.

***punensis*** (Flint), 1983a:9 [Type locality: Argentina, Pcia. Tucumán, Rt. 307, La Angostura; NMNH; ♂; in *Mexitrichia*]. —Angrisano, 1999:30 [checklist]. —Rueda Martín and Gibon, 2008:223 [checklist, distribution]. —Blahnik and Holzenthal, 2008:69 [to *Mortoniella*; *leroda* species group]. —Blahnik and Holzenthal, 2011:45 [♂; redescription; *punensis* subgroup]. —Isa Miranda and Rueda Martín, 2014:199 [distribution].

**Distribution.** Argentina, Bolivia.

***pusilla*** Blahnik and Holzenthal, 2011:43 [Type locality: Brazil, Minas Gerais, Corrego Pitanga, upstream of confl. with Rio Santo Antônio, 19°05'40"S, 042°39'54"W, 238 m; MZUSP; ♂; *leroda* species group, *pumila* subgroup]. —Paprocki and França, 2014:13 [checklist].

**Distribution.** Brazil.

***quinuas*** Harper and Turcotte, 1985:137 [Type locality: Ecuador, Quinuas Valley near Cuenca, small affluents of Río Matadero, (79°09'W, 02°48'S); UMQ; ♂]. —Sykora 1999:384 [♂; *flinti* group]. —Blahnik and Holzenthal, 2008:70 [*bilineata* species group].

**Distribution.** Ecuador.

***rancura*** (Mosely), 1954:345 [Type locality: Mexico, Barranca Honda; BMNH; ♂; in *Mexitrichia*]. —Bueno-Soria and Flint, 1978:193 [distribution]. —Blahnik and Holzenthal, 2008:49, 69 [♂; redescription; to *Mortoniella*; *leroda* species group]. —Bueno-Soria and Barba-Álvarez, 2011:354 [checklist].

**Distribution.** Mexico.

***redunca*** Blahnik and Holzenthal, 2008:49 [Type locality: Costa Rica, Alajuela, Río Toro, 3.0 km (road) SW Bajos del Toro, 10°12'14"N, 84°18'58"W, 1530 m; UMSP; ♂; *leroda* species group]. —Armitage et al., 2015a:4 [distribution]. —Armitage et al., 2015b:6 [checklist]. —Armitage and Cornejo, 2015:194 [checklist].

**Distribution.** Costa Rica, Panama.

***rodmani*** Blahnik and Holzenthal, 2008:52 [Type locality: Costa Rica, Guanacaste, Parque Nacional Rincón de la Vieja, Quebrada Zopilote, 10°45'54"N, 85°18'32"W, 785 m; ♂; UMSP]. —Blahnik and Holzenthal, 2008:70 [*Mortoniella*, *incertae sedis*].

**Distribution.** Costa Rica.

***roldani*** Flint, 1991:23 [Type locality: Colombia, Dpto. Antioquia, Río Aurrá at km 50, E San Jerónimo; NMNH; ♂]. —Sykora 1999:386 [*bilineata* group]. —Muñoz-Quesada, 2000:275 [checklist]. —Blahnik and Holzenthal, 2008:70 [*bilineata* species group].

**Distribution.** Colombia.

***rovira*** (Flint), 1974a:10 [Type locality: Panama, Rovira, Chiriquí, David; NMNH; ♂; in *Mexitrichia*]. —Aguila, 1992:533 [distribution]. —Blahnik and Holzenthal, 2008:54 [♂; redescription; distribution; to *Mortoniella*, *leroda* species group]. —Armitage et al., 2015b:6 [checklist]. —Armitage and Cornejo, 2015:194 [checklist].

**Distribution.** Costa Rica, Panama.

***santiaga*** Sykora, 1999:383 [Type locality: Ecuador, Morone, Santiago, 34 km SE Gualaceo, Rio Culebrillas, 2200 m; CMNH; ♂; *flinti* group]. —Blahnik and Holzenthal, 2008:70 [*bilineata* species group].

**Distribution.** Ecuador.

***sicula*** Blahnik and Holzenthal, 2008:56 [Type locality: Costa Rica, Guanacaste, Parque Nacional Guanacaste, Río Tempisquito, Estación Maritza, 10°57'29"N, 85°29'49"W, 550 m; UMSP; ♂; *leroda* species group].

**Distribution.** Costa Rica.

***similis*** Sykora, 1999:380 [Type locality: Eduador, Pich. [sic], Sto. Domingo do los Colorados, 14 km E; NMNH; ♂; *bilineata* group]. —Blahnik and Holzenthal, 2008:70 [*bilineata* species group].

**Distribution.** Ecuador.

***simla*** (Flint), 1974a:11 [Type locality: Trinidad, Simla; NMNH; ♂; in *Mexitrichia*]. —Botosaneanu and Sakal, 1992:201 [distribution; ecology]. —Botosaneanu and Alkins-Koo, 1993:7 [♀; larva; distribution]. —Flint, 1996a:70 [distribution]. —Botosaneanu, 2002:81 [distribution]. —Rueda Martín and Gibon, 2008:224 [checklist, distribution]. —Blahnik and Holzenthal, 2008:69 [to *Mortoniella*; *leroda* species group].

**Distribution.** Bolivia, Trinidad, Venezuela.

***spinulata*** (Flint), 1991:22 [Type locality: Colombia, Dpto. Antioquia, Quebrada Espadera, 7 km E Medellín (on road to Sta. Elena); NMNH; ♂; in *Mexitrichia*]. —Muñoz-Quesada, 2000:275 [checklist]. —Blahnik and Holzenthal, 2008:69 [to *Mortoniella*; *leroda* species group].

**Distribution.** Colombia.

***squamata*** Sykora, 1999:379 [Type locality: Eduador, Napo, 5 kms S Baeza, 1900 m; NMNH; ♂; *bilineata* group]. —Blahnik and Holzenthal, 2008:70 [*bilineata* species group].

**Distribution.** Ecuador.

***stilula*** Blahnik and Holzenthal, 2008:58 [Type locality: Costa Rica, Heredia, Río Bijagual, on road to Magsasay, 10°24'29"N, 84°04'34"W, 140 m; UMSP; ♂; *leroda* species group].

**Distribution.** Costa Rica.

***tapanti*** Blahnik and Holzenthal, 2008:60 [Type locality: Costa Rica, Cartago, Reserva [Parque Nacional] Tapantí, Quebrada Palmitos & falls, ca. 9 km (road) NW tunnel, 9°43'12"N, 83°46'48"W, 1400 m; UMSP; ♂; *leroda* species group]. —Armitage et al., 2015b:6 [checklist]. —Armitage and Cornejo, 2015:194 [checklist].

**Distribution.** Costa Rica, Panama.

***taurina*** Blahnik and Holzenthal, 2008:62 [Type locality: Costa Rica, Cartago, Quebrada Platanillo, ca. 5 km E Moravia de Chirripó, 9°49'16"N, 83°24'25"W, 1130 m; UMSP; ♂; *leroda* species group]. —Armitage et al., 2015b:6 [checklist]. —Armitage and Cornejo, 2015:194 [checklist].

**Distribution.** Costa Rica, Panama.

***teutona*** (Mosely), 1939a:223 [Type locality: Brazil, Santa Catarina, Nova Teutonia; BMNH; ♂; in *Mexitrichia*]. —Flint 1963a:474 [distribution]. —Flint, 1966a:2 [erroneously to synonymy with *albolineata*]. —Flint, 1972b:226 [resurrected, distribution]. —Angrisano, 1997b:58 [distribution; as *teutonia*]. —Angrisano, 1999:30 [checklist]. —Flint et al., 1999b:27 [as *teutonia*]. —Paprocki et al., 2004:6 [checklist]. —Blahnik et al., 2004:4 [distribution]. —Blahnik and Holzenthal, 2008:69 [to *Mortoniella*; *leroda* species group]. —Calor, 2011:320 [checklist]. —Dumas et al., 2009:364 [distribution]. —Blahnik and Holzenthal, 2011:31 [♂; redescription; distribution; *albolineata* subgroup]. —Dumas and Nessimian, 2012:10 [distribution]. —Manzo et al., 2014:166 [distribution]. —Paprocki and França, 2014:13 [checklist].

**Distribution.** Argentina, Brazil, Uruguay.

***tranquilla*** Martynov, 1912:38 [Type locality: Peru, Callanga; ASL; ♀]. —Flint 1963a:465 [unidentifiable]. —Sykora, 1999:385 [unknown group]. —Blahnik and Holzenthal, 2008:70 [*bilineata* species group].

**Distribution.** Peru.

***tripuiensis*** Blahnik and Holzenthal, 2011:58 [Type locality: Brazil, Minas Gerais, Estação Ecológica do Tripuí, Córrego Tripuí, 20°23'22"S, 043°32'32"W, 1070 m; ♂; ♀; MZUSP; *velasquezi* species group]. —Paprocki and França, 2014:13 [checklist].

**Distribution.** Brazil.

***truncata*** Blahnik and Holzenthal, 2011:34 [Type locality: Brazil, Minas Gerais, spring trib to Rio Macauba, near Pandeiros, 15°28'38"S, 044°44'38"W, 525 m; MZUSP; ♂; *leroda* species group, *albolineata* subgroup]. —Paprocki and França, 2014:14 [checklist].

**Distribution.** Brazil.

***umbonata*** Blahnik and Holzenthal, 2008:64 [Type locality: Panama, Chiriquí, Guadalupe Arriba, 8°52'26"N, 82°33'13"W; NMNH; ♂; *leroda* species group]. —Armitage et al., 2015b:6 [checklist]. —Armitage and Cornejo, 2015:194 [checklist].

**Distribution.** Panama.

***unilineata*** Sykora, 1999:385 [Type locality: Venezuela, Me [sic], 11 km SE Apartaderos; NMNH; ♂; *argentinica* group]. —Blahnik and Holzenthal, 2008:70 [*bilineata* species group].

**Distribution.** Venezuela.

***unota*** (Mosely), 1939a:223 [Type locality: Brazil, Santa Catharina [sic], Nova Teutonia; BMNH; ♂; in *Mexitrichia*]. —Angrisano, 1997b:58 [distribution]. —Angrisano, 1999:30 [checklist]. —Paprocki et al., 2004:6 [checklist]. —Blahnik and Holzenthal, 2008:69 [to *Mortoniella*; *leroda* species group]. —Blahnik and Holzenthal, 2011:37 [♂; redescription; distribution; *albolineata* subgroup]. —Manzo et al., 2014:167 [distribution]. —Paprocki and França, 2014:14 [checklist].

**Distribution.** Argentina, Brazil.

***uruguaiensis*** Blahnik and Holzenthal, 2011:37 [Type locality: Uruguay, Artigas, San Gregorio, 30°33'S, 057°52'W; NMNH; ♂; *leroda* species group, *albolineata* subgroup]. —Paprocki and França, 2014:14 [checklist].

**Distribution.** Brazil, Uruguay.

***usseglioi*** (Rueda Martín and Gibon), 2008:219 [Type locality: Bolivia, Rio Tumusla (not very far from Tumusla and the crossing with the road Potosi-Tupiza); ♂; IML; in *Mexitrichia*]. **NEW COMBINATION**

**Distribution.** Bolivia.

***velasquezi*** (Flint), 1991:22 [Type locality: Colombia, Dpto. Antioquia, Río Aurrá at km 50, E San Jerónimo; NMNH; ♂; in *Mexitrichia*]. —Muñoz-Quesada, 2000:275 [checklist]. —Blahnik and Holzenthal, 2008:70 [to *Mortoniella*; *velasquezi* species group].

**Distribution.** Colombia.

***wygodzinskii*** (Schmid), 1958b:194 [Type locality: Argentina, Tucumán, Quebrada Los Sosa; NMNH; ♂; in *Mexitrichia*]. —Flint 1963a:465 [possibly a species of *Mortoniella*]. —Knutson and Flint, 1979:32 [Empididae predators in pupal cocoons]. —Angrisano, 1999:30 [checklist]. —Sykora, 1999:385 [to *Mortoniella*, *wygodzinskii* group; distribution]. —Rueda Martín and Gibon, 2008:224 [checklist, distribution]. —Blahnik and Holzenthal, 2008:70 [*bilineata* species group]. —Blahnik and Holzenthal, 2011:63 [♂; redescription]. —Isa Miranda and Rueda Martín, 2014:199 [distribution].

**Distribution.** Argentina, Bolivia, Ecuador, Venezuela.

#### Genus *Protoptila* Banks [87]

*Protoptila* Banks, 1904c:215 [Type species: *Beraea ? maculata* Hagen, 1861, original designation]. —Mosely, 1937:152 [♀; description]. —Mosely, 1954:318 [description]. —Flint, 1971c:13 [key]. —Flint 1996b:381 [key]. —Holzenthal and Blahnik, 2006:1 [revision of Costa Rican species]. —Robertson and Holzenthal, 2013:1, 38 [review of genus, phylogeny, diagnosis; key to Protoptilinae genera].

Eighty-seven species and subspecies are presently known from Mexico, Central, and South America, including the Lesser Antilles, but many undescribed species undoubtedly occur in nature. The larvae have been described a number of times (e.g., Ross 1944, Wiggins 1996, Valverde and Abelando 2006) for both North and South American species. They are found on rocks in flowing waters. Gut contents were of fine organic particles with some diatoms (Wiggins 1996). Adults fly in large numbers to lights placed near rivers.

***alexanderi*** Ross, 1941:48 [Type locality: United States, Texas, San Antonio, San Antonio River; INHS; ♂]. —Mosely 1954:327 [♂; ♀; distribution]. —Flint, 1963a:464 [larval diagnosis]. —Bueno-Soria and Flint, 1978:193 [distribution]. —Baumgardner and Bowles, 2005:7, 11 [distribution]. —Bowles et al., 2007:21 [distribution; biology].

**Distribution.** Mexico, U.S.A.

***altura*** Holzenthal and Blahnik, 2006:4 [Type locality: Costa Rica, Puntarenas, Río Cotón, in Las Alturas, 8°56'17"N, 82°49'34"W, el. 1360 m; ♂; UMSP]. —Armitage et al., 2015a:4 [distribution]. —Armitage et al., 2015b:6 [checklist]. —Armitage and Cornejo, 2015:194 [checklist].

**Distribution.** Costa Rica, Panama.

***alumnorum*** Rueda Martín and Gibon, 2008:219 [Type locality: Bolivia, Rio Yara, at the crossing with the road Caranavi/Quiquibey; ♂; IML].

**Distribution.** Bolivia.

***bicornuta*** Flint, 1963a:475 [Type locality: Honduras, Lancetilla, August; MCZ; ♂]. —Bueno-Soria and Flint, 1978:193 [distribution]. —Holzenthal 1988c:54 [distribution]. —Holzenthal and Blahnik, 2006:7 [♂; diagnosis; distribution]. —Chamorro-Lacayo et al., 2007:40 [checklist]. —Bueno-Soria and Barba-Álvarez, 2011:354 [checklist].

**Distribution.** Belize, Costa Rica, Guatemala, Honduras, Mexico.

***boruca*** Flint, 1974a:18 [Type locality: Costa Rica, San José, Río General, Pacuare; NMNH; ♂]. —Holzenthal, 1988c:54 [distribution]. —Holzenthal and Blahnik, 2006:7 [♂; diagnosis; distribution].

**Distribution.** Costa Rica.

***bribri*** Holzenthal and Blahnik, 2006:9 [Type locality: Costa Rica, Alajuela, Río Pizote, ca. 5 km (air) S Brasilia, 10°58'19"N, 85°20'42"W, el. 390 m; ♂; UMSP].

**Distribution.** Costa Rica.

***burica*** Flint, 1974a:17 [Type locality: Costa Rica, Puntarenas, 2.8 miles E of Golfito; NMNH; ♂]. —Holzenthal, 1988c:54 [distribution]. —Holzenthal and Blahnik, 2006:12 [♂; diagnosis; distribution].

**Distribution.** Costa Rica.

***cana*** Flint, 1974a:18 [Type locality: Costa Rica, Guanacaste, Río Corobici, Las Canas; NMNH; ♂]. —Aguila, 1992:534 [distribution]. —Holzenthal, 1988c:54 [distribution]. —Holzenthal and Blahnik, 2006:12 [♂; diagnosis; distribution]. —Armitage et al., 2015b:6 [checklist]. —Armitage and Cornejo, 2015:195 [checklist].

**Distribution.** Costa Rica, Panama.

***cardela*** Mosely, 1954:336 [Type locality: Mexico, Vera Cruz, Cardel; BMNH; ♂]. —Bueno-Soria and Flint, 1978:193 [distribution].

**Distribution.** Mexico.

***chitaria*** Holzenthal and Blahnik, 2006:13 [Type locality: Costa Rica, Cartago, Río Chitaría, rt 10, 10 km NW Río Reventazón, 9°55'12"N, 83°36'14"W, el. 740 m; ♂; UMSP]. —Armitage et al., 2015a:4 [distribution]. —Armitage et al., 2015b:6 [checklist]. —Armitage and Cornejo, 2015:195 [checklist].

**Distribution.** Costa Rica, Panama.

***choluteca*** Flint, 1974a:16 [Type locality: Honduras, Valle, Nacaome; NMNH; ♂]. —Maes, 1999:1192 [checklist]. —Chamorro-Lacayo et al., 2007:40 [checklist].

**Distribution.** Honduras, Nicaragua.

***chontala*** Flint, 1974a:16 [Type locality: Mexico, Tabasco, Río Puyacatengo, E of Teapa; NMNH; ♂]. —Bueno-Soria and Flint, 1978:193 [distribution].

**Distribution.** Mexico.

***colombiensis*** Flint, 1974a:14 [Type locality: Colombia, Valle, Río Raposo; NMNH; ♂]. —Muñoz-Quesada, 2000:275 [checklist].

**Distribution.** Colombia.

***condylifera*** Flint, 1971c:16 [Type locality: Brazil [Edo. Amazonas], Río Marauiá, Endstation vor langer Cachoeira, fluss tritt hier aus dem Gebirge mit starkem Gefälle; NMNH; ♂]. —Angrisano, 1999:30 [checklist]. —Paprocki et al., 2004:6 [checklist]. —Paprocki and França, 2014:14 [checklist].

**Distribution.** Brazil.

***cora*** Flint, 1983a:9 [Type locality: Paraguay, Dpto. Aquidabán, Cerro Corá; NMNH; ♂]. —Angrisano, 1999:30 [checklist]. —Paprocki et al., 2004:6 [checklist]. —Blahnik et al., 2004:4 [distribution]. —Paprocki and França, 2014:14 [checklist].

**Distribution.** Paraguay.

***cristata*** Flint, 1967b:4 [Type locality: Mexico, Veracruz, Cuitlahuac; NMNH; ♂]. —Bueno-Soria and Flint, 1978:193 [distribution]. —Bueno-Soria et al., 2005:75 [distribution].

**Distribution.** Mexico.

***cristula*** Holzenthal and Blahnik, 2006:18 [Type locality: Costa Rica, Alajuela, Río Pizote, ca. 5 km (air) S Brasilia, 10°58'19"N, 085°20'42"W, el. 390 m; ♂; UMSP]. —Chamorro-Lacayo et al., 2007:40 [checklist].

**Distribution.** Costa Rica, Nicaragua.

***ctenacantha*** Flint, 1974c:13 [Type locality: Suriname, Coeroeni-eiland; RNH; ♂].

**Distribution.** Suriname.

***ctilopsis*** Flint, 1974c:15 [Type locality: Suriname, Brownsberg, mountain creek near Golddiggers camp; RNH; ♂].

**Distribution.** Suriname.

***curiosa*** Flint, 1974a:15 [Type locality: Colombia, Valle, Río Raposo; NMNH; ♂]. —Muñoz-Quesada, 2000:275 [checklist].

**Distribution.** Colombia.

***delaca*** Mosely, 1954:333 [Type locality: Mexico, Vera Cruz, Cardel; BMNH; ♂]. —Bueno-Soria and Flint, 1978:193 [distribution].

**Distribution.** Mexico.

***diablita*** Robertson and Holzenthal, 2008:466 [Type locality: Bolivia, La Paz, ANMI (Área Natural de Manejo Integrado) Madidi, Raya Mayo river at Wabacuro trail, Chalalan Ecolodge, 14°26'33"S, 67°54'39"W, 351 m; ♂; UASC; discussion of scales on this species].

**Distribution.** Bolivia.

***disticha*** Flint, 1971c:18 [Type locality: Brazil [Edo. Amazonas], Rio Solimões, bei der Mündung Ipixuna; NMNH; ♂]. —Angrisano, 1999:30 [checklist]. —Paprocki et al., 2004:6 [checklist]. —Paprocki and França, 2014:14 [checklist].

**Distribution.** Brazil.

***dominicensis*** Flint, 1968b:7 [Type locality: Dominica, Morne Nicholls; NMNH; ♂; larva; pupa]. —Malicky, 1983:264 [distribution]. —Flint and Sykora, 1993:49 [checklist]. —Botosaneanu, 1994a:35 [distribution]. —Botosaneanu, 2000:256 [distribution]. —Botosaneanu, 2002:81 [distribution]. —Botosaneanu and Thomas, 2005:55 [checklist].

**Distribution.** Dominica, Guadeloupe.

***dubitans*** Mosely, 1939a:221 [Type locality: Brazil, Santa Catarina, Nova Teutonia; BMNH; ♂]. —Mangeaud, 1996:154 [distribution]. —Angrisano, 1999:30 [checklist]. —Cohen, 2004:76 [distribution]. —Paprocki et al., 2004:6 [checklist]. —Valverde and Abelando, 2006:12 [larva; pupa; distribution]. —Rueda Martín and Gibon, 2008:224 [checklist, distribution]. —Angrisano and Sganga, 2007:23 [♂; distribution]. —Robertson and Holzenthal, 2008:470 [distribution]. —Paprocki and França, 2014:14 [checklist].

**Distribution.** Argentina, Bolivia, Brazil, Uruguay.

***ensifera*** Flint, 1971c:16 [Type locality: Brazil [Edo. Amazonas], Rio Cuieiras, Cachoeira da Traira; NMNH; ♂]. —Flint, 1974c:12 [♂; distribution]. —Flint, 1992d:64 [distribution]. —Angrisano, 1999:30 [checklist]. —Paprocki et al., 2004:6 [checklist]. —Paprocki and França, 2014:15 [checklist].

**Distribution.** Brazil, Suriname.

***erotica*** Ross, 1938b:113 [Type locality: U.S.A., Wyoming, along North Platte River; INHS; ♂; ♀]. —Houghton, 2001:90 [distribution]. —Blinn and Ruiter, 2006:332 [biology]. —Bueno-Soria et al., 2007:32 [distribution]. —Blinn and Ruiter, 2009b:185 [phenology, distribution].

**Distribution.** Mexico, U.S.A.

***fimbriata*** Flint, 1981a:8 [Type locality: Venezuela, Aragua, Maracay, Río Limón, Estación Piscicultura; NMNH; ♂]. —Flint, 1991:24 [♂; distribution]. —Muñoz-Quesada, 2000:275 [checklist].

**Distribution.** Colombia, Venezuela.

***flexispina*** Flint, 1971c:18 [Type locality: Brazil [Edo. Amazonas], Rio Solimões, Igarapé Uarini, 20 km oberhalb; NMNH; ♂]. —Angrisano, 1999:30 [checklist]. —Paprocki et al., 2004:6 [checklist]. —Paprocki and França, 2014:15 [checklist].

**Distribution.** Brazil.

***goitiai*** Rueda Martín and Gibon, 2008:220 [Type locality: Bolivia, Cochabamba, from a small tributary of the Rio Espiritu Santo (Chipiriri near Villa Tunari), 16°50'45"S, 65°25'33W; IML; ♂].

**Distribution.** Bolivia.

***guarani*** Flint, 1974a:18 [Type locality: Paraguay, Salto de Guaira; NMNH; ♂]. —Angrisano, 1999:30 [checklist].

**Distribution.** Paraguay.

***guata*** Mosely, 1954:331 [Type locality: Mexico, Sinaloa, Badiraguata; BMNH; ♂]. —Bueno-Soria et al., 2007:32 [distribution].

**Distribution.** Mexico.

***huasteca*** Flint, 1967b:4 [Type locality: Mexico, San Luis Potosí, 25 miles north of Tamazunchale; NMNH; ♂]. —Bueno-Soria and Flint, 1978:193 [distribution].

**Distribution.** Mexico.

***huava*** Flint, 1974a:15 [Type locality: Mexico, Oaxaca, Jaltepec, Isthmus of Tehuantepec; NMNH; ♂]. —Bueno-Soria and Flint, 1978:193 [distribution]. —Bueno-Soria and Barba-Álvarez, 2011:354 [checklist].

**Distribution.** Mexico.

***ignera*** Flint, 1974a:16 [Type locality: Trinidad, Simla; NMNH; ♂]. —Botosaneanu and Sakal, 1992:201 [biology]. —Botosaneanu and Alkins-Koo, 1993:7 [♀; larva; distribution]. —Flint, 1996a:69 [distribution]. —Botosaneanu, 2002:81 [distribution].

**Distribution.** Tobago, Trinidad.

***ixtala*** Mosely, 1937:156 [Type locality: Mexico, Huixtla; BMNH; ♂]. —Bueno-Soria and Flint, 1978:193 [distribution]. —Holzenthal, 1988c:55 [distribution]. —Maes, 1999:1192 [checklist]. —Holzenthal and Blahnik, 2006:19 [♂; diagnosis; distribution]. —Chamorro-Lacayo et al., 2007:40 [checklist]. —Bueno-Soria and Barba-Álvarez, 2011:354 [checklist].

**Distribution.** Costa Rica, Guatemala, Honduras, Mexico, Nicaragua.

***jolandae*** Holzenthal and Blahnik, 2006:19 [Type locality: Costa Rica, Alajuela, Reserva Forestal San Ramón, Río San Lorencito & tribs., 10°12'58"N, 84°36'25"W, el. 980 m; ♂; UMSP].

**Distribution.** Costa Rica.

***julieta*** Robertson and Holzenthal, 2008:470 [Type locality: Bolivia, Cochabamba, Paracticito, R. San Rafaél, Pte. “Panchito,” nr. P.N. Carrasco station, 17°03'39"S, 65°28'58"W, 438 m; ♂; UASC]. —Isa Miranda and Rueda Martín, 2014:199 [distribution].

**Distribution.** Argentina, Bolivia, Peru.

***kjeri*** Holzenthal and Blahnik, 2006:22 [Type locality: Costa Rica, Alajuela, Río Pizote, ca. 5 km (air) S Brasilia, 10°58'19"N, 85°20'42"W, el. 390 m; ♂; UMSP]. —Chamorro-Lacayo et al., 2007:40 [checklist].

**Distribution.** Costa Rica.

***laterospina*** Flint, 1967b:3 [Type locality: Costa Rica, La Lola; NMNH; ♂]. —Holzenthal, 1988c:55 [distribution]. —Aguila, 1992:534 [distribution]. —Holzenthal and Blahnik, 2006:25 [♂; diagnosis; distribution]. —Armitage et al., 2015b:6 [checklist]. —Armitage and Cornejo, 2015:195 [checklist].

**Distribution.** Costa Rica, Panama.

***leonilae*** Bueno-Soria and Santiago-Fragoso, 1995:88 [Type locality: Mexico, Oaxaca, Totoltepec; IBUNAM; ♂].

**Distribution.** Mexico.

***liqua*** Mosely, 1954:327 [Type locality: Mexico, Liquidamber; BMNH; ♂]. —Bueno-Soria and Flint, 1978:193 [distribution]. —Bueno-Soria and Barba-Álvarez, 2011:354 [checklist].

**Distribution.** Mexico.

***locula*** Mosely, 1954:322 [Type locality: Mexico, Caracuara; BMNH; ♂]. —Bueno-Soria and Flint, 1978:193 [distribution].

**Distribution.** Mexico.

***longispinata*** Santos and Nessimian, 2009c:724 [Type locality: Brazil, Amazonas, Manaus, tributary to Rio Branquinho, 2°31'24.6"S, 60°20'05.3"W; INPA; ♂]. —Paprocki and França, 2014:15 [checklist].

**Distribution.** Brazil.

***lorada*** Mosely, 1954:333 [Type locality: Mexico, Guerrera, Tierra Colorada; BMNH; ♂]. —Bueno-Soria and Flint, 1978:193 [distribution].

**Distribution.** Mexico.

***lucia*** Flint, 1974c:12 [Type locality: Suriname, Lucie River, Camp, Wilhelmina Mountains Expedition; RNH; ♂].

**Distribution.** Suriname.

***macilenta*** Flint, 1971c:19 [Type locality: Brazil [Edo. Pará], Rio Tocantins, im Hause des Ingenieurs von Rio Impex; NMNH; ♂]. —Angrisano, 1999:30 [checklist]. —Paprocki et al., 2004:6 [checklist]. —Paprocki and França, 2014:15 [checklist].

**Distribution.** Brazil.

***malica*** Mosely, 1954:324 [Type locality: Mexico, Colima; BMNH; ♂]. —Bueno-Soria and Flint, 1978:193 [distribution].

**Distribution.** Mexico.

***mara*** Flint, 1971c:16 [Type locality: Brazil [Edo. Amazonas], Rio Marauiá, Endstation vor langer Cachoeira, fluss tritt hier aus dem Gebirge mit starkem Gefälle; NMNH; ♂]. —Angrisano, 1999:31 [checklist]. —Paprocki et al., 2004:6 [checklist]. —Paprocki and França, 2014:15 [checklist].

**Distribution.** Brazil.

***marqua*** Flint, 1967b:3 [Type locality: Mexico, Las Cruces National Park, La Marquesa; NMNH; ♂]. —Bueno-Soria and Flint, 1978:193 [distribution]. —Bueno-Soria et al., 2007:32 [distribution].

**Distribution.** Mexico.

***mayana*** Flint, 1974a:17 [Type locality: Belize [British Honduras], Cayo, Blancaneaux lodge; NMNH; ♂].

**Distribution.** Belize.

***mina*** Flint, 1974c:13 [Type locality: Suriname, Wilhelmina Mountains, Linker Coppename River, Zuid Creek; RNH; ♂].

**Distribution.** Suriname.

***misionensis*** Flint, 1972b:226 [Type locality: Argentina, Misiones Province, Mbopicua, near Puerto Rico; NMNH; ♂]. —Angrisano, 1999:31 [checklist]. —Valverde and Abelando, 2006:14 [larva; pupa; distribution]. —Rueda Martín and Gibon, 2008:224 [checklist, distribution]. —Isa Miranda and Rueda Martín, 2014:199 [distribution].

**Distribution.** Argentina, Bolivia.

***mixteca mixteca*** Flint, 1974a:17 [Type locality: Mexico, Oaxaca, Tamazulapan; NMNH; ♂]. —Bueno-Soria and Flint, 1978:193 [distribution].

**Distribution.** Mexico.

***mixteca veracruzensis*** Flint, 1974a:17 [Type locality: Mexico, Veracruz, Fortín de las Flores; NMNH; ♂]. —Bueno-Soria and Flint, 1978:194 [distribution].

**Distribution.** Mexico.

***myriamae*** Rueda Martín and Gibon, 2008:220 [Type locality: Bolivia, Rio Ilenez in Versalles, 12°39'39"S, 63°22'22"W; IML; ♂].

**Distribution.** Bolivia.

***olvidada*** Bueno-Soria, Santiago-Fragoso and Barba-Álvarez, 2004:479 [Type locality: Mexico, Oaxaca, Loxicha, Pluma Hidalgo, el. 450 m; CNIN; ♂].

**Distribution.** Mexico.

***orotina orotina*** Flint, 1974a:13 [Type locality: Costa Rica, Puntarenas, 9 miles NW of Esparta; NMNH; ♂]. —Holzenthal, 1988c:55 [distribution]. —Aguila, 1992:534 [distribution]. —Holzenthal and Blahnik, 2006:25 [♂; diagnosis; distribution]. —Armitage et al., 2015b:6 [checklist]. —Armitage and Cornejo, 2015:195 [checklist].

**Distribution.** Costa Rica, Panama.

***orotina raposa*** Flint, 1974a:13 [Type locality: Colombia, Valle, Río Raposo; NMNH; ♂]. —Flint and Reyes, 1991:478 [distribution]. —Muñoz-Quesada, 2000:275 [checklist].

**Distribution.** Colombia, Peru.

***perdida*** Bueno-Soria, Santiago-Fragoso and Barba-Álvarez, 2004:480 [Type locality: Panama, Canal Zone, Pipeline Road; CAS; ♂]. —Armitage et al., 2015b:6 [checklist]. —Armitage and Cornejo, 2015:195 [checklist].

**Distribution.** Panama.

***phyllisae*** Bueno-Soria, 1983b:450 [Type locality: Mexico, Chiapas, Agua Azul, 59 km southwest from Palenque; NMNH; ♂]. —Bueno-Soria and Barba-Álvarez, 2011:354 [checklist].

**Distribution.** Mexico.

***piacha*** Mosely, 1954:322 [Type locality: Mexico, Tierra Colorada; BMNH; ♂]. —Bueno-Soria and Flint, 1978:194 [distribution]. —Bueno-Soria and Barba-Álvarez, 2011:354 [checklist].

**Distribution.** Mexico.

***primerana*** (Weyenbergh), 1881:133 [Type locality: Argentina, Córdoba, Primero brook; type depository unknown; larva; in *Rhiacophila* (sic)]. —Flint et al., 1999a:74 [systematic position, to *Protoptila*].

**Distribution.** Argentina.

***pseudopiacha*** Bueno-Soria, 1984b:393 [Type locality: Mexico, Oaxaca, Guelatao; NMNH; ♂].

**Distribution.** Mexico.

***quicha*** Flint, 1974a:15 [Type locality: Guatemala, Chimaltenango, Tecpán; NMNH; ♂].

**Distribution.** Guatemala.

***quinoi*** Bueno-Soria and Santiago-Fragoso, 1979:477 [Type locality: Mexico, Veracruz, Balzapote; NMNH; ♂].

**Distribution.** Mexico.

***resolda*** Mosely, 1937:157 [Type locality: Mexico, Chiapas, Dolores; BMNH; ♂]. —Maes, 1999:1192 [checklist]. —Bueno-Soria and Flint, 1978:194 [distribution]. —Bueno-Soria et al., 2007:32 [distribution]. —Chamorro-Lacayo et al., 2007:40 [checklist]. —Bueno-Soria and Barba-Álvarez, 2011:354 [checklist].

**Distribution.** Mexico, Nicaragua.

***rota*** Mosely, 1937:152 [Type locality: Mexico, Chiapas, Dolores; BMNH; ♂]. —Maes, 1999:1192 [checklist]. —Bueno-Soria and Flint, 1978:194 [distribution]. —Chamorro-Lacayo et al., 2007:40 [checklist]. —Bueno-Soria and Barba-Álvarez, 2011:354 [checklist].

**Distribution.** Mexico, Nicaragua.

***salta*** Mosely, 1937:154 [Type locality: Mexico, Chiapas, Dolores; BMNH; ♂]. —Flint 1963a:475 [distribution]. —Bueno-Soria and Flint, 1978:194 [distribution]. —Maes, 1999:1193 [checklist]. —Rojas-Ascencio, et al., 2002:377 [distribution]. —Chamorro-Lacayo et al., 2007:40 [checklist]. —Bueno-Soria and Barba-Álvarez, 2011:354 [checklist].

**Distribution.** Guatemala, Mexico, Nicaragua.

***simplex*** Flint, 1971c:15 [Type locality: Brazil [Edo. Pará], Rio Tocantins, im Hause des Ingenieurs von Rio Impex; NMNH; ♂]. —Flint, 1974c:11 [♂; distribution]. —Angrisano, 1999:31 [checklist]. —Paprocki et al., 2004:6 [checklist]. —Paprocki and França, 2014:15 [checklist].

**Distribution.** Brazil, Suriname.

***spangleri*** Flint, 1967b:5 [Type locality: Mexico, Veracruz, Cuitlahuac; NMNH; ♂]. —Bueno-Soria and Flint, 1978:194 [distribution]. —Bueno-Soria et al., 2005:75 [distribution].

**Distribution.** Mexico.

***spirifera*** Flint, 1974a:14 [Type locality: Costa Rica, Cartago, Ojo de Agua, route 2, km 75; NMNH; ♂]. —Holzenthal, 1988c:55 [distribution]. —Holzenthal and Blahnik, 2006:28 [♂; diagnosis; distribution].

**Distribution.** Costa Rica.

***strepsicera*** Holzenthal and Blahnik, 2006:28 [Type locality: Costa Rica, Limón, Reserva Biológica Hitoy-Cerere, Río Cerere, Est. Miramar, 9°40'16"N, 83°01'41"W, el. 90 m; ♂; UMSP].

**Distribution.** Costa Rica.

***talamanca*** Flint 1974a:14 [Type locality: Costa Rica, Cartago, 3 miles W of Turrialba; NMNH; ♂]. —Holzenthal, 1988c:55 [distribution]. —Holzenthal and Blahnik, 2006:30 [♂; diagnosis; distribution].

**Distribution.** Costa Rica.

***tarahumara*** Bueno-Soria, 2010:23 [Type locality: Mexico, Chihuahua, Sierra Tarahumara, Urique, Río Urique, 27°13'51"N, 107°53'05"W, el. 575 m; CNIN; ♂].

**Distribution.** Mexico.

***techila*** Mosely, 1954:324 [Type locality: Mexico, Oaxaca, Río Chiltepec; BMNH; ♂]. —Bueno-Soria and Flint, 1978:194 [distribution].

**Distribution.** Mexico.

***ternatia*** Flint, 1971c:17 [Type locality: Brazil [Edo. Amazonas], Rio Solimões, Igarapé Uarini, 20 km oberhalb; NMNH; ♂]. —Angrisano, 1999:31 [checklist]. —Paprocki et al., 2004:6 [checklist]. —Paprocki and França, 2014:15 [checklist].

**Distribution.** Brazil.

***tetravittata*** Flint, 1971c:17 [Type locality: Brazil [Edo. Amazonas], Rio Cuieiras, Igarapé Cachoeira, bei dem Wasserfall Pedra dos Indios; NMNH; ♂]. —Angrisano, 1999:31 [checklist]. —Paprocki et al., 2004:6 [checklist]. —Paprocki and França, 2014:15 [checklist].

**Distribution.** Brazil.

***tica*** Bueno-Soria, 1984b:392 [Type locality: Costa Rica, Corcovado, Estación Sirena; IBUNAM; ♂]. —Holzenthal, 1988c:55 [distribution]. —Holzenthal and Blahnik, 2006:30 [♂; diagnosis; distribution].

**Distribution.** Costa Rica.

***ticumanensis*** Bueno-Soria, 1984b:392 [Type locality: Mexico, Morelos, Ticuman; IBUNAM; ♂]. —Bueno-Soria et al., 2007:32 [distribution].

**Distribution.** Mexico.

***tojana*** Mosely, 1954:331 [Type locality: Mexico, Chiapas, Jonata; BMNH; ♂]. —Flint 1963a:476 [distribution]. —Bueno-Soria and Flint, 1978:194 [distribution]. —Maes and Flint 1988:2 [distribution]. —Holzenthal, 1988c:55 [distribution]. —Flint and Reyes, 1991:478 [distribution]. —Maes, 1999:1193 [checklist]. —Holzenthal and Blahnik, 2006:32 [♂; diagnosis; distribution]. —Chamorro-Lacayo et al., 2007:40 [checklist]. —Bueno-Soria and Barba-Álvarez, 2011:354 [checklist]. —Armitage et al., 2015b:6 [checklist]. —Armitage and Cornejo, 2015:195 [checklist].

**Distribution.** Costa Rica, Honduras, Mexico, Nicaragua, Panama, Peru.

***trichoglossa*** Holzenthal and Blahnik, 2006:34 [Type locality: Costa Rica, Puntarenas, Río Bellavista, ca. 1.5 km NW Las Alturas, 8°57'04"N, 82°50'46"W, el. 1400 m; ♂; UMSP]. —Armitage et al., 2015a:4 [distribution]. —Armitage et al., 2015b:6 [checklist]. —Armitage and Cornejo, 2015:195 [checklist].

**Distribution.** Costa Rica, Panama.

***trispicata*** Flint, 1971c:17 [Type locality: Brazil [Edo. Amazonas], Cachoeira do Gigante; NMNH; ♂]. —Angrisano, 1999:31 [checklist]. —Paprocki et al., 2004:6 [checklist]. —Paprocki and França, 2014:16 [checklist].

**Distribution.** Brazil.

***truncata*** Flint, 1983a:10 [Type locality: Argentina, Pcia. Misiones, Puerto Libertad; NMNH; ♂]. —Angrisano, 1999:31 [checklist].

**Distribution.** Argentina.

***uruguayensis*** Angrisano, 1997b:56 [Type locality: Uruguay, Salto, Salto Grande, en la cascada; FHCU; ♂; ♀]. —Angrisano, 1999:31 [checklist].

**Distribution.** Uruguay.

***voluta*** Flint, 1991:25 [Type locality: Colombia, Dpto. Antioquia, Quebrada La Jiménez, Sopetrán (trap C); NMNH; ♂]. —Muñoz-Quesada, 2000:275 [checklist].

**Distribution.** Colombia.

***yurumanga*** Flint, 1974a:18 [Type locality: Colombia, Valle, Río Raposo; NMNH; ♂]. —Muñoz-Quesada, 2000:275 [checklist].

**Distribution.** Colombia.

#### Genus *Scotiotrichia* Mosely [1]

*Scotiotrichia* Mosely, 1934b:160 [Type species: *Scotiotrichia ocreata* Mosely, 1934b, original designation]. —Robertson and Holzenthal, 2013:1, 39 [review of genus, phylogeny, diagnosis; key to Protoptilinae genera].

A single species is known in the genus, endemic to the Chilean Subregion. Its immature stages have not been described.

***ocreata*** Mosely, 1934b:160 [Type locality: Argentina, Terr. Rio Negro, Bariloche; BMNH; ♂]. —Schmid, 1958b:194 [as *acreata*, misspelling of *ocreata*]. —Flint 1963a:477 [description; distribution]. —Flint, 1974e:86 [checklist]. —Angrisano, 1999:31 [checklist]. —Robertson and Holzenthal, 2013:39 [♂]. —Brand and Miserendino, 2014:6 [community ecology].

**Distribution.** Argentina, Chile.

#### Genus *Tolhuaca* Schmid [2]

*Tolhuaca* Schmid, 1964:336 [Type species: *Tolhuaca cupulifera* Schmid, 1964, original designation]. —Robertson and Holzenthal, 2005:53 [revision, phylogeny, biogeography]. —Robertson and Holzenthal, 2013:1, 40 [review of genus, phylogeny, diagnosis; key to Protoptilinae genera].

The genus now contains 2 disjunct species, one in southern Chile and one in southeastern Brazil. Nothing is known of the immature stages or their biology. Robertson and Holzenthal (2005) discussed the biogeography of the genus and speculated that the disjunct occurrence of the 2 species represents relicts of a more widespread southern Gondwanan ancestor.

***brasiliensis*** Robertson and Holzenthal, 2005:62 [Type locality: Brazil, São Paulo, Parque Estadual Campos do Jordão, 1st order trib. to Rio Galharada, 22°41'40"S, 45°27'47"W, 1530 m; MZUSP; ♂; female]. —Spies and Froehlich, 2009:215 [distribution]. —Calor, 2011:320 [checklist]. —Paprocki and França, 2014:16 [checklist].

**Distribution.** Brazil.

***cupulifera*** Schmid, 1964:337 [Type locality: Chile, Pichinahuel, Arauca; NMNH; ♂; in Sericostomatidae]. —Flint 1967a:52 [rectification of transposition of wing figures in original description, to Glossosomatidae, Protoptilinae, distribution]. —Flint, 1974e:86 [checklist]. —Angrisano, 1999:31 [checklist]. —Robertson and Holzenthal, 2005:57 [♂; ♀; redescription; distribution].

**Distribution.** Chile.

### Family Helicophidae

The 43 extant species in this family occur on both sides of the southern Pacific Ocean. Four genera and 27 species are known from Australia, New Caledonia, and New Zealand and five genera and 16 species are found in southern Chile and Argentina. Johanson and Keijsner (2008) reviewed the family and provided a hypothesis of its phylogeny.

The immature stages of several Australian-New Zealand genera are known (Cowley 1978, Dean et al. 2004) as are those of the Patagonian genera *Eosericostoma* and *Austrocentrus* (Flint 1992e, 1997). Larvae build cases of sand grains or plant material and live in clear streams in forested areas.

#### Genus *Alloecentrellodes* Flint [2]

*Alloecentrellodes* Flint, 1979:646 [Type species: *Alloecentrellodes obliquus* Flint, 1979, original designation].

The two species known in this genus are associated with small, clear, cold, fast-flowing streams in forested areas (Flint 1979). The immature stages are unknown.

***elongatus*** Flint, 1979:649 [Type locality: Chile, Prov. Ñuble, Recinto; NMNH; ♂].

**Distribution.** Chile.

***obliquus*** Flint, 1979:646 [Type locality: Chile, Prov. Malleco, Parque Nacional Contulmo (near boundary of Prov. Arauco); NMNH; ♂].

**Distribution.** Chile.

#### Genus *Austrocentrus* Schmid [3]

*Austrocentrus* Schmid, 1964:337 [Type species: *Austrocentrus griseus* Schmid, 1964, original designation; in Sericostomatidae]. —Flint 1979:646 [to Helicophidae]. —Flint, 1997:99 [revision, immature stages].

Flint’s (1997) revision of this genus raised the number of species in *Austrocentrus* to three. He also described the larvae, pupae, and cases of *Austrocentrus valgiformis* and discussed its biology. Larvae are found in small rocky streams with clear water and moderate flow in forested regions, and are usually associated with aquatic moss (Flint 1997, Brand 2009).

***bifidus*** Flint, 1997:106 [Type locality: Chile, Capt. Prat, 25 km S Cochrane; NMNH; ♂].

**Distribution.** Chile.

***griseus*** Schmid, 1964:338 [Type locality: Chile, Arauco, Pichinahuel; NMNH; ♂]. —Flint, 1974e:91 [checklist]. —Flint 1997:104 [♂; distribution].

**Distribution.** Chile.

***valgiformis*** Flint, 1997:103 [Type locality: Chile, Malleco, [West of Paso] Pino Hachado; NMNH; ♂; ♀; larva; pupa; case]. —Brand, 2009:223 [distribution]. —Brand and Miserendino, 2011a:35 [biology; habitat]. —Brand and Miserendino, 2014:6 [community ecology].

**Distribution.** Argentina, Chile.

#### Genus *Eosericostoma* Schmid [2]

*Eosericostoma* Schmid, 1955a:156 [Type species: *Eosericostoma inaequispinum* Schmid, 1955a, original designation; in Sericostomatidae]. —Flint, 1992e:494 [revision, immature stages, to Helicophidae].

Two species are known in *Eosericostoma*. Both are common and widespread in central Chile. The larval stages of *Eosericostoma inaequispina* have been described (Flint 1992e). The larval case is broad and flat, like those of the European genus *Thremma*, and constructed of sand grains and some plant fragments. Brand and Miserendino (2011b) studied the ecology and life history of *Eosericostoma aequispina* in Argentina and found the species to be univoltive, with a larval development time of 11 months; larvae were scrapers. Adult emergence was synchronous and occurred in February.

***aequispina*** Schmid, 1955a:157 [Type locality: Chile, Chiloé, Aucár; NMNH; ♂]. —Flint, 1974e:91 [checklist]. —Flint, 1992e:504 [♂; ♀; redescription; distribution]. —Brand and Miserendino, 2011b:142 [life history]. —Brand and Miserendino, 2014:6 [community ecology].

**Distribution.** Argentina, Chile.

***inaequispina*** Schmid, 1955a:156 [Type locality: Chile, Santiago [now Prov. Cordillera], El Manzano; NMNH; ♂]. —Flint, 1974e:91 [checklist]. —Flint, 1992e:500 [♂; ♀; larva; pupa; redescription; distribution].

—*aequispina* Schmid, 1957:394 [misidentification, in part - series from Tregualemu]. —Schmid, 1964:339 [misidentification, in part - series from Curacautín, and series from Pillim-Pilli mixed]. —Flint, 1992e:500 [reidentification].

**Distribution.** Argentina, Chile.

#### Genus *Microthremma* Schmid [8]

*Microthremma* Schmid, 1955a:148 [Type species: *Microthremma crassifimbriatum* Schmid, 1955a, original designation; in Thremmidae]. —Flint, 1979:646 [to Helicophidae]. —Flint, 2002b:225 [revision, key to males].

With eight species, *Microthremma* is the largest genus in the family in the Western Hemisphere. The immature stages have not been described. Adults have been collected near small streams and spring runs (Flint 1983a).

***angulatum*** Flint, 2002b:226 [Type locality: Chile, Pcia. Ñuble, Recinto; NMNH; ♂; *crassifimbriatum* complex].

**Distribution.** Chile.

***bipartitum*** Flint, 1983a:90 [Type locality: Chile, Pcia. Chiloé, Dalcahue; NMNH; ♂]. —Flint, 2002b:230 [distribution; *griseum* complex].

**Distribution.** Chile.

***caudatum*** Flint, 1969b:511 [Type locality: Chile, Prov. Concepción, Fundo Pinares, near Concepción; NMNH; ♂; in Sericostomatidae]. —Flint, 1974e:91 [checklist]. —Flint, 2002b:231 [distribution; *griseum* complex].

**Distribution.** Chile.

***crassifimbriatum*** Schmid, 1955a:149 [Type locality: Chile, Ñuble, Recinto; NMNH; ♂]. —Flint, 1974e:91 [checklist]. —Flint, 2002b:226 [♂; redescription; distribution; *crassifimbriatum* complex].

**Distribution.** Chile.

***griseum*** Schmid, 1957:394 [Type locality: Chile, Arauco, Butamalal; NMNH; ♂]. —Flint, 1974e:91 [checklist]. —Flint, 2002b:231 [distribution; *griseum* complex].

**Distribution.** Chile.

***lobatum*** Flint, 2002b:231 [Type locality: Chile, Pcia. Valdivia, Las Trancas, W. La Union; NMNH; ♂; *griseum* complex].

**Distribution.** Chile.

***patagonicum*** Flint, 2002b:228 [Type locality: Argentina, Pcia. Neuquén, 3 km W. Estación Forestal Purará; NMNH; ♂; *crassifimbriatum* complex].

**Distribution.** Argentina, Chile.

***villosum*** Schmid, 1957:395 [Type locality: Chile, Arauco, Caramavida; NMNH; ♂]. —Flint, 1974e:91 [checklist]. —Flint, 2002b:230 [distribution; *crassifimbriatum* complex].

**Distribution.** Chile.

#### Genus *Pseudosericostoma* Schmid [1]

*Pseudosericostoma* Schmid, 1957:395 [Type species: *Pseudosericostoma simplississimum* Schmid, 1957, original designation; in Sericostomatidae]. —Flint 1983a:90 [to Helicophidae].

The single species, *Pseudosericostoma simplississimum*, is only known from the single adult type. The larva and its biology are not known.

***simplississimum*** Schmid, 1957:396 [Type locality: Chile, Arauco, Pichinahuel; NMNH; ♂]. —Flint, 1974e:92 [checklist].

**Distribution.** Chile.

### Family Helicopsychidae

Well over 250 species are currently recognized in this small, primarily tropical family, which has been cataloged (Johanson 1995) and assessed phylogenetically (Johanson 1998). All of the species, except one, belong in the genus *Helicopsyche*, following the synonymization of 4 previosuly recognized genera. The only other genus in the family recongnized by Johanson (1998), *Rakiura*, is endemic to New Zealand and contains a single species. *Helicopsyche* is cosmopolitan in distribution, and comprises over 120 species in the Neotropics, including three fossil species.

All known larvae build cases in the general form of a snail shell, but there is great diversity in the height of the case, number and tightness of whorls, size, nature, and degree of minerals and silk incorporated, etc. (Cowley 1978, Flint 1968a, Wiggins 1996). Larvae appear to be periphyton scrapers on rocks in moderate to slow flowing sections of streams or wave-washed shores (Resh et al., 1984).

#### Genus *Helicopsyche* Siebold [121 + †3]

*Cochliopsyche* Müller, 1885:201 [Type species: *Tetanonema clarum* Ulmer, 1905a, subsequent selection of Ulmer, 1955]. —Flint, 1986:213 [review]. —Monson et al., 1988:158 [diagnosis]. —Schmid, 1993:97 [diagnosis]. —Johanson, 1995:107 [catalog]. —Johanson, 1998:128 [as subgenus of *Helicopsyche*]. —Johanson, 2003d:384 [review]. —Wichard, 2007a:35 [diagnosis].

*Tetanonema* Ulmer 1905a:17 [Type species: *Tetanonema clarum* Ulmer 1905a, by monotypy]. —Ulmer, 1955:589 [as synonym of *Cochliopsyche*].

*Helicopsyche* Siebold, 1856:38 [Type species: *Helicopsyche shuttleworthi* Siebold, 1856, subsequent selection of Flint, 1964a]. —Schmid, 1993:67 [diagnosis]. —Johanson, 1995:101 [catalog]. —Johanson, 1998 [revision; phylogeny; biogeography].

*Feropsyche* Johanson, 1998:131 [Type species: *Notidobia borealis* Hagen, 1861, original designation, as subgenus, phylogeny]. —Johanson, 2002:16 [revision; key; new species]. —Wichard, 2007a:37 [diagnosis].

The genus has been recorded from all biogeographical regions, except Antarctica (Johanson 1998), but by far the greatest species diversity occurs in the tropics of Asia, including Australia, and the Americas, between 5-30°N and 15-45°S (Johanson 1997, 1998, Schmid 1993, Williams et al. 1983). Johanson (1998) revised the world fauna and placed all the Neotropical species in two subgenera, *Feropsyche* and *Cochliopsyche*. The subgenus *Feropsyche*, endemic to the Western Hemisphere, was reviewed by Johanson (2002). Johanson (2003d) rrevised the subgenus *Cochliopsyche*, endemic to Central and South America, redescribed the 4 previously known species, and added 12 new species. In addition, another ca. 30 species in the subgenus *Feropsyche* have been described by Johanson and collaborators from throughout the Neotropics (e.g., Johanson and Holzenthal 2004, 2010; Johanson and Malm 2006). In total, *Helicopsyche* comprises 123 species in the region, 16 in *Cochliopsyche* and 102 extant and 3 fossil species in *Feropsyche*. The three known fossil species are members of *Feropsyche* (Johanson and Wichard 1996).

Larvae construct helical cases of sand grains closely resembling the shells of snails. In fact, some of the American species were originally described as molluscs. Cases vary greatly among species in structure and composition (Flint 1968a, Botosaneanu and Sykora 1973, Ross 1975). Larvae, especially those of Helicopsyche (Feropsyche) borealis, have been described a number of times (Marlier 1964b, Wiggins 1996, Roldán-Pérez 1988). The immature stages of Helicopsyche (Cochliopysche) vazquezae were described in detail by Monson et al. (1988), who also discussed some aspects of the biology of the species. Larvae of *Feropsyche* are found in springs, streams, and rivers, and, in temperate areas, on the shores of lakes (Wiggins 1996); those of *Cochliopysche* seem to prefer medium to large rivers. The biology of *Helicopsyche borealis* is well known in comparison to other species (Resh et al., 1984, Vaughn 1985, Williams et al. 1983), although Maharaj and Alkins-Koo (1997) studied the population dynamics of *Helicopsyche margaritensis* (now *Helicopsyche vergelana*) on Trinidad. Adults are often attracted to lights in large numbers.

***alajuela*** (*Feropsyche*) Johanson and Holzenthal, 2010:38 [Type locality: Costa Rica, Alajuela, Reserva Forestal San Ramón, Río San Lorencito and tribs. 10.216°N, 84.607°W, el. 980 m; NMNH; ♂].

**Distribution.** Costa Rica.

***altercoma*** (*Feropsyche*) Botosaneanu and Flint, 1991b:178 [Type locality: Dominican Republic, Dajabón Province, Río Massacre, Balneario El Salto, Loma de Cabrera; NMNH; ♂; ♀]. —Botosaneanu, 1991b:66 [biology]. —Johanson, 1995:108 [catalog]. —Botosaneanu, 1996:22 [distribution]. —Johanson, 1998:131 [phylogeny]. —Flint and Pérez-Gelabert, 1999:36 [checklist]. —Botosaneanu, 2002:99 [ckecklist]. —Johanson, 2002:16 [♂; distribution]. —Flint and Sykora, 2004:8 [distribution]. —Pérez-Gelabert, 2008:299 [checklist].

**Distribution.** Dominican Republic.

***amazona*** (*Cochliopsyche*) Johanson, 2003d:409 [Type locality: Brazil, Amazonas, Manaus area, Rio Branquinho, Lager Tapiri; USNM; ♂]. —Paprocki and França, 2014:16 [checklist].

**Distribution.** Brazil.

***amica*** (*Cochliopsyche*) Johanson, 2003d:395 [Type locality: Venezuela, TFA, Puerto Ayacucho; MVC; ♂; ♀]. —Paprocki and França, 2014:16 [checklist].

**Distribution.** Brazil, Guyana, Venezuela.

***angeloi*** (*Feropsyche*) Holzenthal, Blahnik and Calor, 2016:346 [Type locality: Brazil, Minas Gerais, Córrego das Águas Pretas & tribs., ca. 15 km S Aiuruoca, 22°03.704'S, 44°38.241'W, el. 1386 m; MZUSP; ♂; ♀].

**Distribution.** Brazil.

***angulata*** (*Feropsyche*) Flint, 1981a:37 [Type locality: Venezuela, Aragua, Maracay, Río Limón, Estación Piscicultura; NMNH; ♂; ♀]. —Flint, 1991:103 [♂; distribution]. —Johanson, 1995:108 [catalog]. —Johanson, 1998:131 [phylogeny]. —Muñoz-Quesada, 2000:275 [checklist]. —Johanson, 2002:119 [♂; distribution].—Johanson and Holzenthal, 2004:29 [distribution].

**Distribution.** Colombia, Ecuador, Venezuela.

***apicauda*** (*Feropsyche*) Flint, 1968b:77 [Type locality: Dominica, Pont Casse, 0.5 miles south; NMNH; ♂; ♀; larva; pupa; case]. —Flint and Sykora, 1993:50 [checklist]. —Botosaneanu, 1994a:52 [distribution]. —Johanson, 1998:131 [phylogeny]. —Botosaneanu, 2000:256 [checklist]. —Botosaneanu, 2002:99 [ckecklist]. —Johanson, 2002:99 [♂; distribution]. —Botosaneanu and Thomas, 2005:56 [checklist].

**Distribution.** Dominica, Guadeloupe.

***auroa*** (*Feropsyche*) Johanson and Holzenthal, 2004:11 [Type locality: Venezuela, Lara, P. N. [Parque Nacional] Terepaima, Río Auro near Sabana Alta, 9°44.740'N, 69°16.614'W, el. 480 m; UMSP; ♂].

**Distribution.** Venezuela.

***blahniki*** (*Cochliopsyche*) Johanson, 2003d:401 [Type locality: Venezuela, Guarico, Hato Masuguaral, 45 km S Calabozo, 8.57°N, 67.58°W, el. 75 m; UMSP; ♂; ♀]. —Paprocki and França, 2014:16 [checklist].

**Distribution.** Brazil, Colombia, Ecuador, Guyana, Peru, Venezuela.

***blancasi*** (*Feropsyche*) Schmid, 1958b:209 [Type locality: Peru, Rio Zapatilla; NMNH; ♂]. —Johanson, 1995:109 [catalog]. —Johanson, 2002:55 [♂; distribution].

**Distribution.** Peru.

***blantoni*** (*Feropsyche*) Johanson and Malm, 2006:4 [Type locality: Panama, Cerro Campana, R. Panama; NMNH; ♂]. —Armitage et al., 2015b:9 [checklist]. —Armitage and Cornejo, 2015:198 [checklist].

**Distribution.** Panama.

***borealis*** (*Feropsyche*) (Hagen), 1861:271 [Type locality: Canada, St. Lawrence River; MCZ; ♂; in *Notidobia*]. —Hagen, 1866:253 [to *Helicopsyche*]. —Ross, 1938a:42 [♂; lectotype]. —Ross, 1944:266, 288 [♂; ♀; larva]. —Ross, 1951a:74 [distribution]. —Denning, 1964:134 [checklist]. —Bueno-Soria and Flint, 1978:214 [distribution]. —Williams et al., 1983:2290 [biology; distribution]. —Holzenthal, 1988c:76 [distribution]. —Aguila, 1992:545 [distribution]. —Johanson, 1995:109 [catalog]. —Wiggins, 1996:90 [larva]. —Johanson, 1998:131 [phylogeny]. —Rojas-Ascencio, et al., 2002:377 [distribution]. —Johanson, 2002:30 [♂; distribution]. —Baumgardner and Bowles, 2005:11 [distribution]. —Blinn and Ruiter, 2006:332 [biology]. —Bowles et al., 2007:23 [distribution; biology]. —Bueno-Soria et al., 2007:32 [distribution]. —Chamorro-Lacayo et al., 2007:41 [checklist]. —Blinn and Ruiter, 2009a:302 [biology]. —Blinn and Ruiter, 2009b:185 [phenology, distribution].—Johanson and Holzenthal, 2010:44 [distribution]. —Bueno-Soria and Barba-Álvarez, 2011:355 [checklist]. —Djernaes, 2011:52 [♂; ♀]. —Flint, 2011:103 [distribution]. —Armitage et al., 2015b:9 [checklist]. —Armitage and Cornejo, 2015:198 [checklist].

—*lustrica* Say, 1821:75 [Type locality: U.S.A., Cayuga Lake, as *Paludina*, mollusc]. —Baker, 1961:146 [suppressed name].

—*arenifera* Lea, 1834:104 [Type locality: U.S.A., Tensessee, Cumverland River, as *Valvata*, mollusc]. —Johanson, 2002:32 [to synonymy].

—*glabra* Hagen, 1864a:130, 237 [Type locality: North America; no type nor type depository designated; case]. —Hagen, 1866:253 [to synonymy].

—*californica* Banks, 1899:210 [Type locality: United States, California; MCZ; ♂]. —Ross, 1944:266, 303 [to synonymy].

—*annulicornis* Banks, 1904c:212 [Type locality: United States, Plummer’s Island, Maryland; MCZ; ♂]. —Betten, 1934:416 [to synonymy].

**Distribution.** Canada, Costa Rica, Guatemala, Mexico, Nicaragua, Panama, U.S.A.

***brazilia*** (*Cochliopsyche*) Johanson, 2003d:410 [Type locality: Brazil, Minas Gerais, confluence Rio Peixe & Rio Preto do Itambe, 19°17.525'S, 43°15.457'W, el. 500 m; MZUSP; ♂; ♀]. —Paprocki and França, 2014:16 [checklist].

**Distribution.** Brazil.

***braziliensis*** (*Feropsyche*) (Swainson), 1840:353 [Type locality: Brazil; no type nor type depository designated; case only; in *Thelidomus*, as mollusc]. —Hagen, 1864b:886 [synonymizes, erroneously, species with *Helicopsyche arenifera* Lea, thereby transferring species to *Helicopsyche*]. —Johanson, 2002:144 [taxonomic remarks]. —Paprocki et al., 2004:6 [checklist]. —Paprocki and França, 2014:17 [checklist].

**Distribution.** Brazil.

***breviterga*** (*Feropsyche*) Flint, 1991:105 [Type locality: Colombia, Dpto. Antioquia, Boquerón, source of Quebrada Potreros, W La Fé; NMNH; ♂]. —Johanson, 1995:109 [catalog]. —Johanson, 1998:131 [phylogeny]. —Muñoz-Quesada, 2000:275 [checklist]. —Johanson, 2002:63 [♂; distribution]. —Johanson and Holzenthal, 2004:30 [distribution].

**Distribution.** Colombia, Venezuela.

***caligata*** (*Feropsyche*) Flint, 1967a:67 [Type locality: Chile, Prov. Valdivia, brook at Fundo Walper, near Valdivia; NMNH; ♂]. —Flint, 1974e:92 [checklist]. —Johanson, 1995:109 [catalog]. —Johanson, 1998:131 [phylogeny]. —Johanson, 2002:127 [♂; distribution].

**Distribution.** Chile.

***camuriensis*** (*Feropsyche*) Johanson and Holzenthal, 2004:9 [Type locality: Venezuela, Dist. Fed. [Distrito Federal], Río Camuri Grande, 1 km S Camuri (nucleo U.S.B.), 10.616°N, 66.715°W, el. 30 m; UMSP; ♂].

**Distribution.** Venezuela.

***centrocubana*** (*Feropsyche*) Botosaneanu and Flint, 1991a:212 [Type locality: Cuba, Province Matanzas, Cuabales de Galindo, Valle de Yumuri; ZMUA; ♂]. —Botosaneanu, 1994b:472 [larva]. —Johanson, 1995:109 [catalog]. —Flint, 1996c:15 [checklist]. —Johanson, 1998:131 [phylogeny]. —Botosaneanu, 2002:99 [ckecklist]. —Johanson, 2002:46 [♂; distribution]. —Naranjo López and González Lazo, 2005:150 [checklist].

**Distribution.** Cuba.

***chilensis*** (*Feropsyche*) Flint, 1983a:91 [Type locality: Chile, Pcia. Bío-Bío, Estero Huequecura, 25 km E Santa Bárbara; NMNH; ♂]. —Johanson, 1995:110 [catalog]. —Johanson, 1998:131 [phylogeny]. —Johanson, 2002:37 [♂; distribution].

**Distribution.** Chile.

***chiriquensis*** (*Feropsyche*) Johanson and Malm, 2006:8 [Type locality: Panama, Chiriqui, Fortuna Dam Site, nr. Hornitos, 8°55'N, 82°16'W, el. 1050 m; NMNH; ♂; ♀]. —Johanson and Holzenthal, 2010:44 [distribution]. —Armitage et al., 2015b:9 [checklist]. —Armitage and Cornejo, 2015:198 [checklist].

**Distribution.** Costa Rica, Panama.

***chocoensis*** (*Cochliopsyche*) Johanson, 2003d:401 [Type locality: Colombia, Choco, Rio Atrato, Yuto; USNM; ♂; /females/].

**Distribution.** Colombia.

***cipoensis*** (*Feropsyche*) Johanson and Malm, 2006:17 [Type locality: Brazil, Minas Gerais, Serra do Cipo; NMNH; ♂]. —Paprocki and França, 2014:17 [checklist].

**Distribution.** Brazil.

***circulata*** (*Feropsyche*) Johanson and Holzenthal, 2004:25 [Type locality: Venezuela, Aragua, 1 km E Estación Biológica Rancho Grande, 10.352°N, 67.680°W, el. 1100 m; UMSP; ♂].

**Distribution.** Venezuela.

***clara*** (*Cochliopsyche*) (Ulmer), 1905a:18 [Type locality: Brazil, Santa Catarina; PAN; ♂; in *Tetanonema*]. —Flint, 1966a:12 [♂; lectotype]. —Johanson, 1995:107 [catalog]. —Johanson, 1998:128 [status; phylogeny]. —Johanson, 2003d:388 [♂; ♀; redescription; distribution]. —Blahnik et al., 2004:4 [distribution]. —Paprocki et al., 2004:6 [checklist]. —Calor, 2011:320 [checklist]. —Souza et al., 2013a:3 [distribution]. —Paprocki and França, 2014:17 [checklist].

**Distribution.** Argentina, Brazil, Ecuador.

***cochleara*** (*Feropsyche*) Johanson, 1999:128 [Type locality: Ecuador, Past. Puyo (27 km N), Est. Fluv. Metrica; NMHH; ♂]. —Johanson, 2002:134 [♂; distribution].

**Distribution.** Ecuador.

***colombiensis*** (*Feropsyche*) Siebold, 1856:144 [Type locality: Colombia, Puerto Cabello; no type nor type depository designated; case only]. —Hagen, 1864a:127 [case]. —Muñoz-Quesada, 2000:275 [checklist]. —Johanson, 2002:139 [taxonomic remarks].

**Distribution.** Colombia, Venezuela.

***comosa*** (*Feropsyche*) Kingsolver, 1964:259 [Type locality: Cuba, Aspiro-Rangel, Pinar del Rio Province; INHS; ♂]. —Flint, 1968b:83 [checklist]. —Botosaneanu, 1979:53 [distribution]. —Botosaneanu and Flint, 1991b:176 [♂; ♀; redescription]. —Johanson, 1995:110 [catalog]. —Flint, 1996c:15 [checklist]. —Johanson, 1998:131 [phylogeny]. —Botosaneanu, 2002:99 [ckecklist]. —Johanson, 2002:19 [♂; distribution]. —López del Castillo et al., 2004:229 [sp. near *comosa*, distribution]. —González Lazo et al., 2005:260 [c.f. *comosa*, distribution]. —Naranjo López and González Lazo, 2005:150 [checklist].

**Distribution.** Cuba.

***cotopaxi*** (*Feropsyche*) Botosaneanu and Flint, 1982:25 [Type locality: Ecuador, Volcan Cotopaxi; NMNH, ♂; ♀; case]. —Johanson, 1995:110 [catalog]. —Johanson, 1998:131 [phylogeny]. —Johanson, 2002:129 [♂; distribution].

**Distribution.** Ecuador.

***cubana*** (*Feropsyche*) Kingsolver, 1964:259 [Type locality: Cuba, Moa, Oriente Province; INHS; ♂]. —Flint, 1968a:64 [distribution; redescription; ♂; ♀; larva; pupa; case]. —Flint, 1968b:83 [checklist]. —Botosaneanu, 1979:53 [distribution]. —Botosaneanu and Flint, 1991a:207 [as *cubana cubana*; redescription]. —Botosaneanu, 1994b:468 [as *cubana*; larva]. —Johanson, 1995:110 [catalog]. —Flint, 1996c:15 [checklist]. —Johanson, 1998:131 [phylogeny]. —Botosaneanu, 2002:99 [ckecklist]. —Johanson, 2002:44 [♂; distribution]. —Naranjo López and González Lazo, 2005:150 [checklist].

**Distribution.** Cuba, Jamaica.

***curvipalpia*** (*Feropsyche*) Johanson and Malm, 2006:3 [Type locality: Mexico, Chih. Hwy 127, 27.7 mi SW La Junta, 0.5 mi N Sierra Alta Tarahumara, 6900 ft; INHS; ♂; ♀].

**Distribution.** Mexico.

***dampfi*** (*Feropsyche*) Ross, 1956b:398 [Type locality: Mexico, Chiapas, Finca Germania; INHS; ♂]. —Bueno-Soria and Flint, 1978:215 [distribution]. —Maes and Flint, 1988:7 [distribution]. —Johanson, 1995:110 [catalog]. —Johanson, 1998:131 [phylogeny]. —Maes, 1999:1195 [checklist]. —Johanson, 2002:121 [♂; distribution]. —Chamorro-Lacayo et al., 2007:41 [checklist]. —Johanson and Holzenthal, 2010:44 [distribution]. —Bueno-Soria and Barba-Álvarez, 2011:355 [checklist].

**Distribution.** Costa Rica, Guatemala, Mexico, Nicaragua.

***disjuncta*** (*Feropsyche*) Johanson and Holzenthal, 2004:5 [Type locality: Venezuela, Sucre, Parque Nacional Peninsula de Paria, Uquire, Río La Viuda, 10°42.830'N, 61°57.661'W, el. 15 m; NMNH; ♂]

**Distribution.** Venezuela.

***dominicana*** (*Feropsyche*) Botosaneanu and Flint, 1991a:200 [Type locality: Dominican Republic, La Vega Province, 12 km S. of Constanza; NMNH; ♂; ♀]. —Johanson, 1995:110 [catalog]. —Botosaneanu, 1996:22 [distribution]. —Johanson, 1998:131 [phylogeny]. —Flint and Pérez-Gelabert, 1999:36 [checklist]. —Botosaneanu, 2002:99 [ckecklist]. —Johanson, 2002:118 [♂; distribution]. —Flint and Sykora, 2004:8 [distribution]. —Pérez-Gelabert, 2008:299 [checklist].

**Distribution.** Dominican Republic.

***dorsocurvata*** (*Feropsyche*) Johanson and Holzenthal, 2010:41 [Type locality: Costa Rica, Cartago, Reserva Tapanti, Quebrada Palmitos and falls, 9.72°N, 83.78°W, el. 1400 m; UMSP; ♂].

**Distribution.** Costa Rica.

† ***electra*** (*Feropsyche*) Johanson and Wichard, 1996:199 [Type locality: Dominican Republic; ♂; collection Wichard; in amber]. —Flint and Pérez-Gelabert, 1999:36 [checklist]. —Botosaneanu, 2002:99 [ckecklist]. —Wichard, 2007a:38 [♂; key]. —Pérez-Gelabert, 2008:299 [checklist].

**Distribution.** Dominican Republic.

***extensa*** (*Feropsyche*) Ross, 1956b:397 [Type locality: Peru, Department of Cusco, Valley of the Cosnipata, Santa Isabel; INHS; ♂]. —Flint, 1996b:429 [distribution]. —Johanson, 1995:111 [catalog]. —Johanson, 1998:131 [phylogeny]. —Johanson, 2002:123 [♂; distribution]. —Johanson and Holzenthal, 2004:30 [distribution].

**Distribution.** Peru, Venezuela.

***falcigona*** (*Feropsyche*) Botosaneanu and Flint, 1991a:210 [Type locality: Cuba, Isla de Pinos, Santa Fé, Arroyo La Talega; ZMUA; ♂]. —Botosaneanu, 1994b:472 [larva]. —Botosaneanu, 2002:100 [ckecklist; as subspecies of *Helicopsyche ochthephila*]. —Flint, 1996c:15 [checklist]. —Botosaneanu and Hyslop, 1998:23 [as subspecies of *ochthephila*]. —Johanson, 1995:111 [catalog]. —Johanson, 1998:131 [phylogeny]. —Johanson, 2002:77 [to species; ♂; distribution]. —Naranjo López and González Lazo, 2005:150 [checklist; as subspecies of *ochthephila*].

**Distribution.** Cuba.

***fistulata*** (*Feropsyche*) Flint, 1991:105 [Type locality: Colombia, Dpto. Antioquia, Quebrada La Mosca, 1 km W Guarne; NMNH; ♂]. —Johanson, 1995:111 [catalog]. —Johanson, 1998:131 [phylogeny]. —Muñoz-Quesada, 2000:275 [checklist]. —Johanson, 2002:68 [♂; distribution]. —Johanson and Holzenthal, 2004:30 [distribution].

**Distribution.** Colombia, Venezuela.

***flinti*** (*Feropsyche*) Johanson, 1999:127 [Type locality: Brazil, [Santa Catarina], Nova Teutonia, 27°11'B[S], 52°23'L[W]; BMNH; ♂]. —Johanson, 2002:111 [♂; distribution]. —Paprocki et al., 2004:6 [checklist]. —Paprocki and França, 2014:17 [checklist].

**Distribution.** Brazil.

***fridae*** (*Feropsyche*) Johanson, 2003a:5 [Type locality: Panama, Province of Panama, Barro Colorado Island; NMNH; ♂; ♀; key]. —Armitage et al., 2015b:9 [checklist]. —Armitage and Cornejo, 2015:198 [checklist].

**Distribution.** Panama.

***golfitoensis*** (*Feropsyche*) Johanson and Holzenthal, 2010:39 [Type locality: Costa Rica, [Puntarenas], 2.8 mi E of Golfito; NMNH; ♂].

**Distribution.** Costa Rica.

***granpiedrana*** (*Feropsyche*) Botosaneanu and Sykora, 1973:402 [Type locality: Cuba, La Gran Piedra; ZMA; ♂]. —Botosaneanu, 1979:52 [distribution]. —Johanson, 1995:111 [catalog]. —Flint, 1996c:15 [checklist]. —Botosaneanu, 2002:99 [ckecklist]. —Johanson, 2002:116 [♂; distribution]. —Naranjo López and González Lazo, 2005:150 [checklist].

**Distribution.** Cuba.

***grenadensis*** (*Feropsyche*) Flint and Sykora, 1993:61 [Type locality: Grenada, Parish St. Andrews, Clabony; FSCA; ♂]. —Johanson, 1995:111 [catalog]. —Johanson, 1998:131 [phylogeny]. —Botosaneanu, 2002:99 [ckecklist]. —Johanson, 2002:111 [♂; distribution]. —Johanson and Holzenthal, 2004:31 [distribution].

**Distribution.** Grenada, Venezuela.

***guadeloupensis*** (*Feropsyche*) Malicky, 1980:222 [Type locality: Guadaloupe, mittellauf des flusses Lezard bei Chemin de Diane; collection Malicky; ♂]. —Malicky, 1983:264 [distribution]. —Botosaneanu, 1988:227 [♀; larva]. —Flint and Sykora, 1993:60 [distribution; synonymy]. —Botosaneanu, 1994a:51 {distribution]. —Johanson, 1995:111 [catalog]. —Johanson, 1998:131 [phylogeny]. —Botosaneanu, 2000:256 [distribution]. —Botosaneanu, 2002:99 [ckecklist]. —Johanson, 2002:81 [♂; distribution]. —Botosaneanu and Thomas, 2005:56 [checklist].

—*species* 1 Flint, 1968b:78 [♀]. —Flint and Sykora, 1993:60 [to synonymy].

—*species* 2 Flint, 1968b:79 [larva]. —Flint and Sykora, 1993:60 [probable synonym].

**Distribution.** Dominica, Guadeloupe, Martinique, St. Lucia.

***guara*** (*Feropsyche*) Holzenthal, Blahnik and Calor, 2016:348 [Type locality: Brazil, Santa Catarina, Rio Caeté, at entrance to Parque Ecológico Spitzkopf, 27°00.350'S, 49°06.650'W, el. 92 m; MZUSP; ♂; ♀].

**Distribution.** Brazil.

***hageni*** (*Feropsyche*) Banks, 1938:296 [Type locality: Cuba, Oriente, north side of Pico Turquino; MCZ; ♂]. —Flint, 1967c:24 [♂; lectotype]. —Flint, 1968b:83 [checklist]. —Botosaneanu, 1979:53 [distribution].—Botosaneanu and Flint, 1991a:199 [redescription]. —Botosaneanu, 1994b:468 [probable larva]. —Johanson, 1995:111 [catalog]. —Botosaneanu, 1996:21 [♀; misidentification of *Helicopsyche parahageni* according to Flint and Sykora, 2004]. —Flint, 1996c:15 [checklist]. —Johanson, 1998:131 [phylogeny]. —Flint and Pérez-Gelabert, 1999:37 [checklist]. —Botosaneanu, 2002:99 [ckecklist]. —Johanson, 2002:79 [♂; distribution].—López del Castillo et al., 2004:229 [cf *hageni*, distribution]. —Naranjo López and González Lazo, 2005:150 [checklist]. —López del Castillo et al., 2007:171 [distribution; seasonal abundance]. —Pérez-Gelabert, 2008:299 [checklist].

**Distribution.** Cuba, Dominican Republic.

***haitiensis*** (*Feropsyche*) Banks, 1938:296 [Type locality: Haiti, La Vesite, La Selle Range; MCZ; ♂]. —Flint, 1967c:24 [♂; lectotype]. —Flint, 1968b:83 [checklist]. —Botosaneanu and Flint, 1991a:203 [redescription]. —Johanson, 1995:111 [catalog]. —Johanson, 1998:131 [phylogeny]. —Flint and Pérez-Gelabert, 1999:37 [checklist]. —Botosaneanu, 2002:99 [ckecklist]. —Johanson, 2002:69 [♂; distribution]. —Flint and Sykora, 2004:9 [distribution]. —Pérez-Gelabert, 2008:299 [checklist].

—*haitiense* Banks, 1938. —Botosaneanu, 1991a:134 [distribution]. —Botosaneanu and Flint, 1991a:203 [♂; invalid emendation].

**Distribution.** Haiti.

***helicoidella*** (*Feropsyche*) (Vallot), 1855:XII [Type locality: [Brazil], Bahia; no type nor type depository designated; case; in *Phryganea*]. —Hagen, 1864a:131 [to *Helicopsyche*]. —Johanson, 2002:139 [taxonomic remarks]. —Paprocki et al., 2004:6 [checklist]. —Paprocki and França, 2014:18 [checklist].

**Distribution.** Brazil.

***holzenthali*** (*Cochliopsyche*) Johanson, 2003d:403 [Type locality: Venezuela, Barinas, Rio Singüis in Cano Grande, 8°24.00'N, 70°46.45'W, el. 520 m; UMSP; ♂; ♀].

**Distribution.** Venezuela.

***incisa*** (*Feropsyche*) Ross, 1956b:398 [Type locality: Mexico, Chiapas, Finca Esperanza; INHS; ♂]. —Bueno-Soria and Flint, 1978:215 [distribution]. —Aguila, 1992:545 [distribution]. —Johanson, 1995:112 [catalog]. —Johanson, 1998:131 [phylogeny]. —Johanson, 2002:105 [♂; distribution]. —Johanson, 2003a:8 [distribution; key]. —Bueno-Soria et al., 2005:75 [distribution]. —Johanson and Malm, 2006:22 [distribution]. —Chamorro-Lacayo et al., 2007:41 [checklist]. —Johanson and Holzenthal, 2010:45 [distribution]. —Bueno-Soria and Barba-Álvarez, 2011:355 [checklist]. —Armitage et al., 2015b:9 [checklist]. —Armitage and Cornejo, 2015:198 [checklist].

**Distribution.** Costa Rica, Mexico, Nicaragua, Panama.

***kalaom*** (*Feropsyche*) Botosaneanu, 1996:22 [Type locality: Dominican Republic, springbrook, 150 m from Salto Agua Blanca, road to Convento; ZMUA; ♂]. —Flint and Pérez-Gelabert, 1999:37 [checklist]. —Botosaneanu, 2002:99 [ckecklist]. —Johanson, 2002:103 [♂; distribution]. —Flint and Sykora, 2004:9 [distribution]. —Pérez-Gelabert, 2008:299 [checklist].

**Distribution.** Dominican Republic.

***kingstona*** (*Feropsyche*) Johanson, 2003c:33 [Type locality: Jamaica, 5 mi directly W of Kingston; UCD; ♂; relationships].

**Distribution.** Jamaica.

***lambda*** (*Feropsyche*) Flint, 1983a:93 [Type locality: Argentina, Pcia Misiones, Arroyo Piray Mini, Rt. 17 W Dos Hermanas; NMNH; ♂]. —Johanson, 1995:112 [catalog]. —Johanson, 1998:131 [phylogeny]. —Johanson, 2002:101 [♂; distribution]. —Manzo et al., 2014:167 [distribution].

**Distribution.** Argentina.

***laneblina*** (*Feropsyche*) Johanson and Holzenthal, 2004:21 [Type locality: Venezuela, T.F.A. [Territorio Federal Amazonas = Estado Amazonas], Camp VII, Cerro de la Neblina, 0°51'N, 65°58'W, el. 1800 m; NMNH; ♂].

**Distribution.** Venezuela.

***lara*** (*Feropsyche*) Johanson and Holzenthal, 2004:19 [Type locality: Venezuela, Lara, Parque Nacional Dinira, Quebrada Buenos Aires, 9°36.407'N, 70°04.178'W, el. 1850 m; UMSP; ♂].

**Distribution.** Venezuela.

***lazzariae*** (*Feropsyche*) Holzenthal, Blahnik and Calor, 2016:348 [Type locality: Brazil, Paraná, Voçoroca, along main road to Joinville, 25°50.332'S, 49°03.332'W, el. 650 m; MZUSP; ♂; ♀].

**Distribution.** Brazil.

***lewalleni*** (*Feropsyche*) Denning and Blickle, 1979:27 [Type locality: El Salvador, La Libertad, west bank Rio Chilama; CAS; ♂; ♀]. —Johanson, 1995:112 [catalog]. —Johanson, 1998:131 [phylogeny]. —Johanson, 2002:28 [♂; distribution]. —Johanson and Holzenthal, 2010:45 [distribution].

**Distribution.** Costa Rica, El Salvador.

***linabena*** (*Feropsyche*) Johanson and Holzenthal, 2004:23 [Type locality: Venezuela, T.F.A. [Territorio Federal Amazonas = Estado Amazonas], Camp IV, Cerro de la Neblina, 0°58'N, 65°57'W, el. 760 m; NMNH; ♂].

**Distribution.** Venezuela.

***linguata*** (*Feropsyche*) Johanson and Malm, 2006:14 [Type locality: Panama, Chiriqui, Fortuna Dam Site, nr. Hornitos, 8°55'N, 82°16'W, el. 1050 m; NMNH; ♂]. —Armitage et al., 2015b:9 [checklist]. —Armitage and Cornejo, 2015:198 [checklist].

**Distribution.** Panama.

***lobata*** (*Cochliopsyche*) Flint, 1983a:95 [Type locality: Argentina, Pcia. Misiones, Arroyo Piray Guazú, N San Pedro; NMNH; ♂]. —Johanson, 1995:107 [catalog]. —Johanson, 1998:128 [status; phylogeny]. —Johanson, 2003d:391 [♂; redescription; distribution]. —Blahnik et al., 2004:4 [distribution]. —Paprocki et al., 2004:6 [checklist]. —Manzo et al., 2014:167 [distribution]. —Paprocki and França, 2014:17 [checklist].

— n. sp. 1 Flint, 1996b: 429 [♂]. —Johanson, 2003d:391 [to *lobata*].

**Distribution.** Argentina, Brazil, Peru.

***lutea*** (*Feropsyche*) (Hagen), 1861:271 [Type locality: Santo Domingo; MCZ; ♀; in *Notidobia*]. —Hagen, 1866:254 [to *Helicopsyche*]. —Ross 1952:35 [♀; lectotype]. —Flint 1967c:24 [taxonomic remarks]. —Flint, 1968b:83 [checklist]. —Botosaneanu and Flint, 1991b:181 [♀; redescription]. —Johanson, 1995:112 [catalog]. —Johanson, 1998:131 [phylogeny]. —Flint and Pérez-Gelabert, 1999:37 [checklist]. —Botosaneanu, 2002:99 [ckecklist]. —Johanson, 2002:138 [♀; distribution]. —Flint and Sykora, 2004:9 [distribution]. —Pérez-Gelabert, 2008:299 [checklist].

**Distribution.** Dominican Republic.

***maculisternum*** (*Feropsyche*) Botosaneanu, in Botosaneanu and Alkins-Koo, 1993:40 [Type locality: Trinidad, the stream just below Maracas waterfall; ZMUA; ♂; ♀; larva; case]. —Botosaneanu and Sakal, 1992:203 [distribution; ecology]. —Johanson, 1995:113 [catalog]. —Flint, 1996a:108 [distribution]. —Johanson, 1998:131 [phylogeny]. —Botosaneanu, 2002:99 [ckecklist]. —Johanson, 2002:59 [♂; distribution]. —Johanson and Holzenthal, 2004:31 [distribution].

—*agglutinans* Lechmere-Guppy, 1864:245 [Type locality: Trinidad; as *Valvata*, mollusc]. —Johanson, 2002:59 [to synonymy].

**Distribution.** Trinidad, Venezuela.

***melanochaeta*** (*Feropsyche*) Flint and Sykora, 2004:11 [Type locality: Dominican Republic, Pedernales Province, Río Mulito, 21 km N Pedernales, 18°09.3'N, 71°45.6W, el. 280 m; NMNH; ♂; ♀]. —Botosaneanu, 2002:100 [ckecklist]. —Pérez-Gelabert, 2008:299 [checklist].

—sp. indet. ex “gr. *comosa*” Botosaneanu, 1996:22 [Dominican Republic, Pedernales Province, Río Mulito, 2 km from the village Mencia de Pedernales and about 6-7 km from its springs, el. 250 m; ZMUA; ♀]. —Botosaneanu, 2002:100 [referred to *melanochaeta*].

**Distribution.** Dominican Republic.

***merida*** (*Feropsyche*) Botosaneanu and Flint, 1982:24 [Type locality: Venezuela, Edo. Mérida, 11 km southeast of Apartaderos; NMNH; ♂; case]. —Johanson, 1995:113 [catalog]. —Johanson, 1998:132 [phylogeny]. —Johanson, 2002:65 [♂; distribution]. —Johanson and Holzenthal, 2004:31 [distribution].

**Distribution.** Venezuela.

***mexicana*** (*Feropsyche*) Banks, 1901:368 [Type locality: Mexico, Cuernavaca; MCZ; ♂]. —Ross, 1944:289, 303 [♂; figured as *arizonensis*]. —Flint, 1967d:176 [distribution]. —Bueno-Soria and Flint, 1978:215 [distribution]. —Denning and Blickle, 1979:32 [redescription; ♀; distribution]. —Johanson, 1995:113 [catalog]. —Johanson, 1998:132 [phylogeny]. —Rojas-Ascencio, et al., 2002:377 [distribution]. —Johanson, 2002:38 [♂; distribution]. —Miller et al., 2002:1663 [biology]. —Blinn and Ruiter, 2006:332 [biology]. —Bueno-Soria et al., 2007:32 [distribution]. —Blinn and Ruiter, 2009a:302 [biology]. —Blinn and Ruiter, 2009b:185 [phenology, distribution]. —Johanson and Holzenthal, 2010:45 [distribution].

—*arizonensis* Banks, 1907a:125 [Type locality: United States, Arizona, Nogales; MCZ; ♀]. —Ross, 1944:303 [to synonymy].

**Distribution.** Costa Rica, Mexico, U.S.A.

***minima*** (*Feropsyche*) Siebold 1856:38 [Type locality: Puerto Rico, Sierra de Luquillo (according to Flint 1964a); no type nor type depository designated; case only]. —Flint, 1964a:71 [redescription; ♂; ♀; larva; pupa; case]. —Flint, 1968b:83 [checklist]. —Botosaneanu and Flint, 1991a:213 [♂; ♀; redescription]. —Johanson, 1995:113 [catalog]. —Johanson, 1998:132 [phylogeny]. —Maes, 1999:1195 [checklist]. —Johanson, 2002:53 [♂; distribution].—Botosaneanu, 2002:100 [ckecklist]. —Chamorro-Lacayo et al., 2007:41 [checklist].

—*nigra* Bremi-Wolf, 1848:125 [Type locality: [U.S.A.] Puerto Rico, Aus einem Backe der Hochebene der Sierra de Suevilla]. —Johanson, 1995:121 [to synonymy].

**Distribution.** Nicaragua, Puerto Rico.

***minuscula*** (*Feropsyche*) Martynov, 1912:3 [Type locality: Peru, Callanga; PAN; /female.]. —Betten 1934:46 [venation]. —Johanson, 1995:113 [catalog]. —Johanson, 2002:137 [♂; distribution].

**Distribution.** Peru.

***molesta*** (*Feropsyche*) Botosaneanu, in Botosaneau and Hyslop, 1998:24 [Type locality: Jamaica, streamlet tributary of East Lucea River at ca. 2 km. upstream from its mouth, Hanover (Lucea); ZMUA; ♂; as subspecies of *occidentale*]. —Botosaneanu, 2002:100 [ckecklist; as subspecies of *occidentale*]. —Johanson, 2002:44, 93 [to species; ♂; distribution].

**Distribution.** Jamaica.

***monda*** (*Feropsyche*) Flint, 1983a:93 [Type locality: Paraguay, Depto. Alto Paraná, Salto del Monday, near Puerto Presidente Franco; NMNH; ♂]. —Johanson, 1995:113 [catalog]. —Johanson, 1998:132 [phylogeny]. —Johanson, 2002:92 [♂; distribution]. —Blahnik et al., 2004:4 [distribution]. —Paprocki et al., 2004:6 [checklist]. —Johanson and Holzenthal, 2004:31 [distribution]. —Dumas et al., 2009:371 [distribution]. —Calor, 2011:320 [checklist]. —Dumas and Nessimian, 2012:10 [checklist]. —Paprocki and França, 2014:18 [checklist].

**Distribution.** Argentina, Brazil, Paraguay, Venezuela.

***montana*** (*Feropsyche*) Felber, 1912:46 [Type locality: Mexico; not stated, perhaps NMB; larva; pupa; case]. —Bueno-Soria and Flint, 1978:215 [distribution]. —Johanson, 1995:113 [catalog]. —Johanson, 2002:141 [taxonomic remarks].

**Distribution.** Mexico.

***muelleri*** (*Feropsyche*) Banks, 1920:348 [Type locality: Brazil, Santa Catharina [sic]; MCZ; ♂; as *mulleri*]. —Flint, 1967c:25 [lectotype]. —Johanson, 1995:113 [catalog]. —Johanson, 1998:132 [phylogeny]. —Johanson, 2002:48 [♂; distribution]. —Cohen, 2004:77 [distribution]. —Paprocki et al., 2004:6 [checklist]. —Paprocki and França, 2014:18 [checklist]. —Isa Miranda and Rueda Martín, 2014:200 [distribution].

—*angelae* Marlier, 1964a:9 [Type locality: Peru, Río Huallaga, tributary to upper Marañon; IRSNB; ♂; larva; pupa; case]. —Flint, 1967c:25 [to synonymy].

**Distribution.** Argentina, Brazil, Peru.

***napoa*** (*Cochliopsyche*) Johanson, 2003d:398 [Type locality: Ecuador, Napo, Lago Agrio; USNM; ♂; ♀].

**Distribution.** Ecuador.

***neblinensis*** (*Feropsyche*) Johanson and Holzenthal, 2004:15 [Type locality: Venezuela, T.F.A. [Territorio Federal Amazonas = Estado Amazonas]: Cerro de la Neblina, Basecamp, 0°51'N, 66°10'W, el. 140 m; NMNH; ♂].

**Distribution.** Venezuela.

***nigrisensilla*** (*Feropsyche*) Botosaneanu and Flint, 1991a:210 [Type locality: Dominican Republic, La Vega Province, 20 km S. of Constanza; NMNH; ♂; ♀]. —Johanson, 1995:114 [catalog]. —Botosaneanu, 1996:22 [distribution]. —Johanson, 1998:132 [phylogeny]. —Flint and Pérez-Gelabert, 1999:37 [checklist]. —Botosaneanu, 2002:100 [ckecklist]. —Johanson, 2002:109 [♂; distribution]. —Flint and Sykora, 2004:10 [distribution]. —Pérez-Gelabert, 2008:299 [checklist].

**Distribution.** Dominican Republic.

***obscura*** (*Feropsyche*) Rueda Martín and Isa Miranda, 2015:205 [Type locality: Argentina, Tucumán, Anfama, 2645'08.9 S, 06531'22 W, 1169 m; IBN; ♂; larva; pupa; biology].

**Distribution.** Argentina.

***occidentale*** (*Feropsyche*) Botosaneanu and Flint, 1991a:207 [Type locality: Cuba, Pinar de Rio Province, Soroa, Río El Manantiales; ZMUA; ♂; as subspecies of *cubana*]. —Botosaneanu, 1994b:471 [larva; as valid species]. —Johanson, 1995:114 [catalog]. —Flint, 1996c:15 [checklist]. —Botosaneanu and Hyslop, 1998:24 [as subspecies]. —Johanson, 1998:132 [phylogeny]. —Botosaneanu, 2002:100 [checklist]. —Johanson, 2002:42 [♂; distribution]. —Naranjo López and González Lazo, 2005:150 [checklist; as *Helicopsyche occidentalis*].

**Distribution.** Cuba.

***ochthephila*** (*Feropsyche*) Flint, 1968a:65 [Type locality: Jamaica, St. Andrew, Hardwar Gap, Dicks Pond Trail; NMNH; ♂; ♀; larva; pupa; case]. —Flint, 1968b:83 [checklist]. —Johanson, 1995:114 [catalog]. —Johanson, 1998:132 [phylogeny]. —Botosaneanu and Hyslop, 1998:23 [♂; larva; case]. —Botosaneanu, 2002:100 [ckecklist]. —Johanson, 2002:75 [♂; distribution].

**Distribution.** Jamaica.

***ocosingua*** (*Cochliopsyche*) Johanson, 2003d:405 [Type locality: Mexico, Chiapas, Ocosingo Valley, Monte Finca Libano; INHS; ♂; ♀].

**Distribution.** Mexico.

***opalescens*** (*Cochliopsyche*) Flint, 1972b:245 [Type locality: Argentina, Misiones, Puerto Rico; NMNH; ♂]. —Flint, 1974c:145 [♂; distribution]. —Flint, 1992d:81 [distribution]. —Johanson, 1995:107 [catalog]. —Flint, 1996b:428 [distribution]. —Johanson, 1998:128 [status, phylogeny]. —Johanson, 2003d:393 [♂; redescription; distribution]. —Blahnik et al., 2004:4 [distribution]. —Cohen, 2004:77 [distribution]. —Paprocki et al., 2004:6 [checklist]. —Dumas et al., 2009:372 [distribution]. —Calor, 2011:320 [checklist]. —Paprocki and França, 2014:17 [checklist].

**Distribution.** Argentina, Brazil, Ecuador, Guyana, Paraguay, Peru, Suriname, Uruguay, Venezuela.

***pandeirosa*** (*Cochliopsyche*) Johanson, 2003d:407 [Type locality: Brazil, Minas Gerais, Rio Pandeirosa in Pandeiros, ca. 50 km W Januária, 15°30.727'S, 44°30.255'W, el. 495 m; MZUSP; ♂; ♀]. —Paprocki and França, 2014:17 [checklist].

**Distribution.** Brazil.

***paprockii*** (*Feropsyche*) Johanson and Malm, 2006:11 [Type locality: Brazil, Minas Gerais, Serra do Cipo; NMNH; ♂]. —Paprocki and França, 2014:18 [checklist].

**Distribution.** Brazil.

***paraguaiensis*** (*Cochliopsyche*) Johanson, 2003d:413 [Type locality: Paraguay; Rio Aquidaban, Cerro Cora; NMNH; ♂].

**Distribution.** Paraguay.

***parahageni*** (*Feropsyche*) Flint and Sykora, 2004:9 [Type locality: Dominican Republic [Barahona Province], Ran Rafael, 8.3 km S of Baoruco, 18°01.9'N, 71°08.4'W, el. 30 m ; NMNH; ♂; ♀]. —Botosaneanu, 1996 [♀; as *hageni*]. —Pérez-Gelabert, 2008:299 [checklist].

**Distribution.** Dominican Republic.

***paucispina*** (*Feropsyche*) Botosaneanu and Flint, 1991a:213 [Type locality: Cuba, Pinar del Rio Province, Viñales, San Vicente, Arroyo del Aqueducto; ZMUA; ♂]. —Flint, 1996c:15 [checklist]. —Johanson, 1995:114 [catalog]. —Johanson, 1998:132 [phylogeny]. —Botosaneanu, 2002:100 [ckecklist]. —Johanson, 2002:97 [♂; distribution]. —Naranjo López and González Lazo, 2005:150 [checklist].

**Distribution.** Cuba.

***perija*** (*Feropsyche*) Johanson and Holzenthal, 2004:27 [Type locality: Venezuela, Táchira, trib. to Río El Valle, 3.8 km SE El Zumbador, 7°57.411'N, 72°04.394'W, el. 2730 m; UMSP; ♂].

**Distribution.** Venezuela.

***peruana*** (*Feropsyche*) Banks, 1920:349 [Type locality: Peru, Natucana; MCZ; ♂]. —Flint, 1967c:25 [relationships]. —Johanson, 1995:114 [catalog]. —Johanson, 1998:132 [phylogeny]. —Johanson, 2002:61 [♂; distribution]. —Johanson, 2003b:1 [♂; redescription].

**Distribution.** Peru.

***pietia*** (*Feropsyche*) Denning, 1964:132 [Type locality: Mexico, Baja California, 3 miles southwest of Mission San Javier southwest of Loreto; CAS; ♂; ♀]. —Bueno-Soria and Flint, 1978:215 [distribution]. —Johanson, 1995:115 [catalog]. —Johanson, 1998:132 [phylogeny]. —Johanson, 2002:61 [♂; distribution].

**Distribution.** Mexico.

***piroa*** (*Feropsyche*) Ross, 1944:289 [Type locality: United States, Texas, San Antonio, along San Antonio River; INHS; ♂]. —Ross, 1956b:400-401 [diagnosis]. —Johanson, 1995:115 [catalog]. —Johanson, 1998:132 [phylogeny]. —Johanson, 2002:24 [♂; distribution]. —Bowles et al., 2007:23 [distribution; biology]. —Chamorro-Lacayo et al., 2007:41 [checklist]. —Bueno-Soria and Barba-Álvarez, 2011:355 [checklist].

**Distribution.** Mexico, Nicaragua, U.S.A.

***planata*** (*Feropsyche*) Ross, 1956b:400 [Type locality: Mexico, Chiapas, San Cristóbal; INHS; ♂]. —Bueno-Soria and Flint, 1978:215 [distribution]. —Johanson, 1995:115 [catalog]. —Johanson, 1998:132 [phylogeny]. —Maes, 1999:1196 [checklist]. —Johanson, 2002:88 [♂; distribution]. —Bueno-Soria et al., 2007:32 [distribution]. —Chamorro-Lacayo et al., 2007:41 [checklist]. —Bueno-Soria and Barba-Álvarez, 2011:355 [checklist].

**Distribution.** Mexico, Nicaragua.

***planorboides*** (*Feropsyche*) Machado, 1957:193 [Type locality: Brazil, Minas Gerais, Tarumirim, Rio Doce valley; type depository not designated, but now in DZRJ (A.P.M. Santos, personal communication); ♂; ♀; larva; pupa; case]. —Johanson, 1995:115 [catalog]. —Johanson, 2002:135 [distribution]. —Paprocki et al., 2004:6 [checklist]. —Paprocki and França, 2014:18 [checklist].

**Distribution.** Brazil.

***poliochaeta*** (*Feropsyche*) Flint and Sykora, 2004:11 [Type locality: Dominican Republic, Pedernales Province, Río Mulito, 21 km N Pedernales, 18°09.3'N, 71°45.6W, el. 280 m; NMNH; ♂; ♀]. —Botosaneanu, 2002:100 [ckecklist]. —Pérez-Gelabert, 2008:299 [checklist].

**Distribution.** Dominican Republic.

***propinqua*** (*Feropsyche*) Botosaneanu and Flint, 1991a:215 [Type locality: Puerto Rico, Toro Negro Forest, Doña Juana creek; NMNH; ♂]. —Johanson, 1995:115 [catalog]. —Johanson, 1998:132 [phylogeny]. Botosaneanu, 2002:100 [ckecklist]. —Johanson, 2002:83 [♂; distribution]. —

**Distribution.** Puerto Rico.

***puyoa*** (*Cochliopsyche*) Johanson, 2003d:406 [Type locality: Ecuador: Past. Puyo (22 km W); USNM; ♂; ♀].

**Distribution.** Ecuador.

***quadrosa*** (*Feropsyche*) Ross, 1956b:400 [Type locality: Mexico, Chiapas, Finca Victoria; INHS; ♂]. —Bueno-Soria and Flint, 1978:215 [distribution]. —Johanson, 1995:115 [catalog]. —Johanson, 1998:132 [phylogeny]. —Johanson, 2002:81 [♂; distribution]. —Bueno-Soria et al., 2005:75 [distribution; as *cuadrata*, mispelling]. —Bueno-Soria and Barba-Álvarez, 2011:355 [checklist].

**Distribution.** Mexico.

***ramosi*** (*Feropsyche*) Flint, 1964a:72 [Type locality: Puerto Rico, Yauco-Lares Rd, Km. 22; NMNH; ♂; ♀; larva; pupa; case]. —Flint, 1968b:83 [checklist]. —Botosaneanu and Flint 1991a:205 [♂; ♀; redescription]. —Johanson, 1995:115 [catalog]. —Johanson, 1998:132 [phylogeny]. —Botosaneanu, 2002:100 [ckecklist]. —Johanson, 2002:95 [♂; distribution].

**Distribution.** Puerto Rico.

***rentzi*** (*Feropsyche*) Denning and Blickle, 1979:31 [Type locality: Costa Rica, Guanacaste, Finca La Pacifica, 10 miles north Cañas; CAS; ♂; ♀]. —Holzenthal, 1988c:76 [distribution]. —Johanson, 1995:115 [catalog]. —Johanson, 1998:132 [phylogeny]. —Johanson, 2002:115 [♂; distribution]. —Johanson and Holzenthal, 2010:46 [distribution].

**Distribution.** Costa Rica.

***sanblasensis*** (*Feropsyche*) Johanson and Malm, 2006:19 [Type locality: Panama, San Blas, 2 km S. Nusagandi; NMNH; ♂]. —Armitage et al., 2015b:9 [checklist]. —Armitage and Cornejo, 2015:198 [checklist].

**Distribution.** Panama.

***scalaris*** (*Feropsyche*) Hagen, 1864a:128 [Type locality: Venezuela, Rio lego, 7000 feet above the sea; no type nor type depository designated; case]. —Johanson, 2002:140 [taxonomic remarks].

**Distribution.** Venezuela.

† ***scaloida*** (*Feropsyche*) Johanson and Wichard, 1996:197 [Type locality: Dominican Republic; ♂; GPIMH; in amber]. —Flint and Pérez-Gelabert, 1999:37 [checklist]. —Botosaneanu, 2002:100 [ckecklist]. —Wichard, 2007a:38 [♂; key]. —Pérez-Gelabert, 2008:299 [checklist].

**Distribution.** Dominican Republic.

***selanderi*** (*Feropsyche*) Ross, 1956b:400 [Type locality: Mexico, Michoacán, 20 miles west of Morelia; INHS; ♂]. —Bueno-Soria and Flint, 1978:215 [distribution]. —Johanson, 1995:115 [catalog]. —Johanson, 1998:132 [phylogeny]. —Johanson, 2002:130 [♂; distribution]. —Johanson and Holzenthal, 2004:32 [distribution]. —Johanson and Holzenthal, 2010:46 [distribution].

**Distribution.** Costa Rica, Mexico, Venezuela.

***septifera*** (*Feropsyche*) Flint and Sykora, 2004:13 [Type locality: Dominican Republic, Pedernales Province, Río Mulito, 21 km N Pedernales, 18°09.3'N, 71°45.6'W, el. 280 m; NMNH; ♂; ♀]. —Botosaneanu, 2002:100 [ckecklist]. —Pérez-Gelabert, 2008:299 [checklist].

—cf. *minima* Von Siebold *in* Botosaneanu, 1996:22 [Dominican Republic, Pedernales Province, Río Mulito, 2 km from the village Mencia de Pedernales and about 6-7 km from its springs, el. 250 m; ♀]. —Botosaneanu, 2002:100 [referred to *septifera*].

**Distribution.** Dominican Republic.

***sigillata*** (*Feropsyche*) Botosaneanu and Flint, 1991b:182 [Type locality: Cuba, Oriente Province, Baracoa, Monte Iberia; ZMUA; ♂; ♀]. —Johanson, 1995:116 [catalog]. —Flint, 1996c:16 [checklist]. —Johanson, 1998:132 [phylogeny]. —Botosaneanu, 2002:100 [ckecklist]. —Johanson, 2002:20 [♂; distribution]. —Naranjo López and González Lazo, 2005:150 [checklist].

**Distribution.** Cuba.

***singulare*** (*Feropsyche*) Botosaneanu and Flint, 1991a:199 [Type locality: Puerto Rico, El Verde Field Station, Quebrada Prieta; NMNH; ♂]. —Johanson, 1995:116 [catalog]. —Johanson, 1998:132 [phylogeny]. —Johanson, 2002:131 [♂; distribution]. —Botosaneanu, 2002:100 [ckecklist].

**Distribution.** Puerto Rico.

***sinuata*** (*Feropsyche*) Denning and Blickle, 1979:31 [Type locality: [U.S.A.] California, San Bernardino County, Sheep Creek Canyon; UCD; ♂]. —Johanson, 1995:116 [catalog]. —Johanson, 1998:132 [phylogeny]. —Johanson, 2002:125 [♂; distribution]. —Johanson and Malm, 2006:23 [distribution].

**Distribution.** Mexico, U.S.A.

***succincta*** (*Feropsyche*) Johanson and Holzenthal, 2004:7 [Type locality: Venezuela, T.F.A. [Territorio Federal Amazonas = Estado Amazonas], Camp XII, el. 1950 m, near Pico Phelps; NMNH; ♂].

**Distribution.** Venezuela.

***sucrensis*** (*Feropsyche*) Johanson and Holzenthal, 2004:3 [Type locality: Venezuela, Sucre, Peninsula de Paria, Puerto Viejo, “Rio el Pozo”, 10°43.073'N, 62°28.569'W, el. 20 m; UMSP; ♂].

**Distribution.** Venezuela.

***tachira*** (*Feropsyche*) Johanson and Holzenthal, 2004:13 [Type locality: Venezuela, Táchira, trib. to Río El Valle, 3.8 km SE El Zumbador, 7°57.411'N, 72°4.394'W, el. 2730 m; UMSP; ♂].

**Distribution.** Venezuela.

***tapadas*** (*Feropsyche*) Denning, 1966:238 [Type locality: Mexico, Nayarit, Arroyo Santiago, 3 miles northwest of Jesus Maria; CAS; ♂]. —Bueno-Soria and Flint, 1978:215 [distribution]. —Johanson, 1995:116 [catalog]. —Johanson, 1998:132 [phylogeny]. —Johanson, 2002:26 [♂; distribution].

**Distribution.** Mexico.

***temora*** (*Feropsyche*) Denning and Blickle, 1979:30 [Type locality: Mexico, Chihuahua, 6.4 km (4 mi) SW of Temoris; UCD; ♂]. —Johanson, 1995:116 [catalog]. —Johanson, 1998:132 [phylogeny]. —Johanson, 2002:30 [♂; distribution].

**Distribution.** Mexico.

***thelidomus*** (*Feropsyche*) Hagen, 1864a:127 [Type locality: Venezuela, Rio lego 7000 feet above the sea; no type nor type depository designated; case]. —Johanson, 2002:139 [taxonomic remarks].

**Distribution.** Venezuela.

***timbira*** (*Feropsyche*) Silva, Santos and Nessimian, 2014:436 [Type locality: Brazil, Rio de Janeiro, Parque Nacional da Serra dos Órgãos, Rio Beija Flor, 22°26'50.9"S, 43°00'19.4"W, el. 1187 m; DZRJ; ♂]. —Paprocki and França, 2014:19 [checklist].

**Distribution.** Brazil.

***truncata*** (*Feropsyche*) Ross, 1956b:398 [Type locality: Mexico, Chiapas, Finca Vergel; INHS; ♂]. —Bueno-Soria and Flint, 1978:215 [distribution]. —Aguila, 1992:545 [distribution]. —Johanson, 1995:117 [catalog]. —Johanson, 1998:132 [phylogeny]. —Johanson, 2002:71 [♂; distribution]. —Johanson and Holzenthal, 2010:46 [distribution]. —Bueno-Soria and Barba-Álvarez, 2011:355 [checklist]. —Armitage et al., 2015b:9 [checklist]. —Armitage and Cornejo, 2015:198 [checklist].

**Distribution.** Costa Rica, Mexico, Panama.

***turbida*** (*Feropsyche*) Navás, 1923a:200 [Type locality: Argentina, Alta Gracia; MZBS; ♀]. —Schmid, 1949a:419 [/male; ♀; redescription]. —Flint, 1967c:25 [as synonym of *muelleri*]. —Flint, 1982c:63 [as valid species]. —Johanson, 1995:117 [catalog]. —Mangeaud, 1996:154 [distribution]. —Johanson, 2002:30 [♂; distribution]. —Isa Miranda and Rueda Martín, 2014:200 [distribution]. —Rueda Martín and Isa Miranda, 2015:208 [redescription; ♂; larva; pupa; biology; distribution].

**Distribution.** Argentina.

***tuxtlensis*** (*Feropsyche*) Bueno-Soria, 1983b:455 [Type locality: Mexico, Veracruz, Balzapote, 3 km north from Estacion de Biologia “Los Tuxtlas”; IBUNAM; ♂]. —Johanson, 1995:117 [catalog]. —Johanson, 1998:132 [phylogeny]. —Johanson, 2002:86 [♂; distribution]. —Johanson, 2003a:8 [distribution; key]. —Armitage et al., 2015b:9 [checklist]. —Armitage and Cornejo, 2015:198 [checklist].

**Distribution.** Guatemala, Mexico, Panama.

***umbonata*** (*Feropsyche*) Hagen, 1864a:128 [Type locality: Jamaica, Chitty, Paines Town; no type nor type depository designated; case]. —Flint, 1968a:62 [distribution; redescription; ♂; ♀; larva; pupa; case]. —Flint, 1968b:83 [checklist]. —Johanson, 1995:117 [catalog]. —Johanson, 1998:132 [phylogeny]. —Malicky, 1999:117, 118 [distribution]. —Johanson, 2002:73 [♂; distribution]. —Botosaneanu, 2002:100 [ckecklist].

**Distribution.** Jamaica.

***valligera*** (*Feropsyche*) Flint, 1983a:93 [Type locality: Argentina, Pcia. Misiones, Arroyo Coatí, 15 mi E San José; NMNH; ♂]. —Johanson, 1995:117 [catalog]. —Johanson, 1998:132 [phylogeny]. —Johanson, 2002:90 [♂; distribution]. —Paprocki et al., 2004:6 [checklist]. —Paprocki and França, 2014:18 [checklist].

**Distribution.** Argentina, Brazil.

***vazquezae*** (*Cochliopsyche*) Flint, 1986:214 [Type locality: Mexico, Chiapas, Río Tulijá, 48 km south of Palenque; NMNH; ♂]. —Holzenthal, 1988c:75 [distribution]. —Monson et al., 1988:154 [larva; pupa; biology; distribution]. —Johanson, 1995:107 [catalog]. —Johanson, 1998:129 [status, phylogeny]. —Muñoz-Quesada, 2000:275 [checklist]. —Johanson, 2003d:392 [♂; redescription; distribution]. —Bueno-Soria and Barba-Álvarez, 2011:354 [checklist].

**Distribution.** Bolivia, Colombia, Costa Rica, Ecuador, Honduras, Mexico, Venezuela.

***venezuelensis*** (*Feropsyche*) Johanson and Holzenthal, 2004:17 [Type locality: Venezuela, Miranda, Río Caruao, 1.6 km S Caruao, 10.597°N, 66.346°W, 5 m; UMSP; ♂].

**Distribution.** Venezuela.

***vergelana*** (*Feropsyche*) Ross, 1956b:400 [Type locality: Mexico, Chiapas, Finca Vergel; INHS; ♂]. —Flint, 1974c:144 [♂; distribution]. —Bueno-Soria and Flint, 1978:215 [distribution]. —Flint, 1981a:37 [♂; distribution]. —Holzenthal, 1988c:76 [distribution]. —Flint, 1991:103 [♂; distribution]. —Flint and Reyes, 1991:490 [distribution]. —Aguila, 1992:545 [distribution]. —Johanson, 1995:117 [catalog]. —Johanson, 1998:132 [phylogeny]. —Maes, 1999:1196 [checklist]. —Muñoz-Quesada, 2000:275 [checklist]. —Johanson, 2002:21 [♂; distribution]. —Johanson, 2003a:8 [distribution; key]. —Paprocki et al., 2004:6 [checklist]. —Bueno-Soria et al., 2005:75 [distribution]. —Chamorro-Lacayo et al., 2007:41 [checklist]. —Johanson and Holzenthal, 2010:46 [distribution]. —Bueno-Soria and Barba-Álvarez, 2011:355 [checklist]. —Souza et al., 2013a:3 [distribution]. —Paprocki and França, 2014:18 [checklist]. —Armitage et al., 2015b:9 [checklist]. —Armitage and Cornejo, 2015:198 [checklist].

—*margaritensis* (*Feropsyche*) Botosaneanu, 1959:61 [Type locality: Venezuela, Isla de Margarita, Toma de Agua de Encañado; ZMUA; ♂; /female; larva; pupa; case]. —Flint, 1981a:37 [♂; ♀; misidentified as *vergelana*]. —Botosaneanu and Sakal, 1992:204 [distribution; ecology]. —Botosaneanu and Alkins-Koo, 1993:39 [larva; distribution]. —Flint and Sykora, 1993:60 [as valid species; distribution]. —Flint, 1996a:107 [distribution]. —Maharaj and Alkins-Koo, 1997:277 [biology]. —Johanson, 1995:113 [catalog]. —Johanson, 1998:132 [phylogeny]. —Botosaneanu, 2002:99 [ckecklist]. —Botosaneanu and Viloria, 2002:110 [distribution]. —Johanson, 2002:24 [to synonymy].

**Distribution.** Belize, Brazil, Costa Rica, Grenada, Guatemala, Honduras, Mexico, Nicaragua, Panama, Paraguay, Peru, Suriname, Tobago, Trinidad, Venezuela.

***villegasi*** (*Feropsyche*) Denning and Blickle, 1979:29 [Type locality: Mexico, Zacatecuas [sic], 4 km W of Nochistlan, Río de Nochistlan, at dam named Presade, “Los Tuzas”; UCD; ♂]. —Johanson, 1995:117 [catalog]. —Johanson, 1998:132 [phylogeny]. —Rojas-Ascencio, et al., 2002:377 [distribution]. —Johanson, 2002:107 [♂; distribution]. —Johanson, 2003c:35 [relationships]. —Bueno-Soria et al., 2007:32 [distribution].

**Distribution.** Mexico.

† ***voigti*** (*Feropsyche*) Johanson and Wichard, 1996:201 [Type locality: Dominican Republic; ♂; collection Wichard; in amber]. —Flint and Pérez-Gelabert, 1999:37 [checklist]. —Botosaneanu, 2002:100 [ckecklist]. —Wichard, 2007a:40 [♂; key]. —Pérez-Gelabert, 2008:299 [checklist].

**Distribution.** Dominican Republic.

***woldai*** (*Feropsyche*) Johanson, 2003a:2 [Type locality: Panama, Province of Panama, Barro Colorado Island; NMNH; ♂; key]. —Johanson and Malm, 2006:22 [distribution]. —Armitage et al., 2015b:9 [checklist]. —Armitage and Cornejo, 2015:198 [checklist].

**Distribution.** Panama.

***woytkowskii*** (*Feropsyche*) Ross, 1956b:398 [Type locality: Peru, Department of Cusco, Valley of the Cosnipata, Santa Isabel; INHS; ♂]. —Flint, 1967c:25 [to synonymy with *peruana*]. —Johanson, 1995:117 [catalog]. —Flint, 1996b:428 [resurrected; distribution]. —Johanson, 2002:57 [♂; distribution]. —Johanson and Holzenthal, 2004:89 [distribution].

**Distribution.** Peru, Venezuela.

***xinguensis*** (*Cochliopsyche*) Johanson, 2003d:397 [Type locality: Brazil, Pará, Rio Xingu Camp, 3°39'S, 52°22'W, ca. 60 km S Altamira; MZUSP; ♂; ♀]. —Paprocki and França, 2014:17 [checklist].

**Distribution.** Brazil.

### Family Hydrobiosidae

This family was originally established by Ulmer (1905d) as a subfamily of the Rhyacophilidae, but has long been considered a distinct family (Neboiss 1986, Schmid 1989). It appears to be the southern hemisphere equivalent of the northern Rhyacophildae. Although no examples are known from southern Africa, it is widespread in the Australasian Region, as far north as the Himalayas and eastern Russia in the Oriental Region, and in the Neotropical Region it occurs northward into the southwestern United States. Fifty genera are known and were extensivly reviewed by Schmid in his 1989 world revision. However, he did not recognize the two subfamilies, Apsilochoreminae and Hydrobiosinae, of Neboiss (1986). Ward et al. (2004) presented a phylogeny of the genera and Strandberg and Johanson (2011) inferred the phylogeny of species of *Apsilochorema* and included other genera as outgroups. The 22 New World genera are almost entirely endemic to patagonian Chile and Argentina, save for *Atopsyche*, widely distributed across South and Central America and including the Antilles and the southwestern US, and *Cailloma*, occurring in the Andes as far north as Ecuador.

As in Rhyacophilidae, the larvae of the hydrobiosids are free-living, and as far as known, predators. They greatly resemble the larvae of gill-less *Rhyacophila*, but the forelegs are modified for grasping their prey (Wiggins 1996). Bravo and Agrisano, in a number of papers (e.g., 2001, 2003, 2004a, b, c, 2005), described the larvae and pupae of many of the Neotropical genera, such that 17 of the 22 genera are known in the immature stages; only *Androchorema*, *Australobiosis*, *Heterochorema*, and *Isochorema* are known from adults only (the genus *Nolganema* is known only from an illustration of its wing venation). Rueda Martín (2006c) presented methods for collecting, rearing, and associating immatures and adults of *Atopsyche* species; these techniques would also apply to other genera. Angrisano and Sganga (2009) provided a key to the larvae and adults of the genera.

#### Genus *Amphichorema* Schmid [3]

*Amphichorema* Schmid, 1989:51 [Type species: *Amphichorema monicae* Schmid, 1989, original designation].

The genus contains three species, all restricted to the Chilean Subregion. The immature stages *Amphichorema costiferum* are described (Bravo and Angrisano, 2004b). Adults are taken at light or swept from bushes near small, fast-flowing streams or larger rivers, usually in forested areas but sometimes in open country.

***costiferum*** (Flint), 1969b:501 [Type locality: Chile, Prov. Cautin, near Pucon; NMNH; ♂; in *Parachorema*]. —Flint, 1974e:85 [checklist]. —Schmid, 1989:143 [♂; ♀]. —Angrisano, 1999:26 [checklist]. —Bravo and Angrisano, 2004b:21 [larva; pupa].

**Distribution.** Argentina, Chile.

***monicae*** Schmid, 1989:116 [Type locality: Chile, Prov. Maule, Paso Garcia; NMNH; ♂]. —Angrisano, 1999:26 [checklist].

**Distribution.** Chile.

***zotheculum*** (Flint), 1969b:503 [Type locality: Chile, Prov. Ñuble, Rio Teno; NMNH; ♂; in *Parachorema*]. —Flint, 1974e:86 [checklist]. —Schmid, 1989:143 [♂]. —Angrisano, 1999:26 [checklist]. —Bravo and Angrisano, 2004b:23 [larva similar to *Amphichorema costiferum*]. —Miserendino and Brand, 2007:312 [biology; distribution].

**Distribution.** Argentina, Chile.

#### Genus *Androchorema* Flint [1]

*Androchorema* Flint, 1979:643 [Type species: *Androchorema chilense* Flint, 1979, original designation]. —Schmid, 1989:86 [redescription].

The genus still contains only a single species, described from Chile. Its immature stages are unknown. Only a very few adults ever have been taken at light near small, fast-flowing streams in forested areas.

***chilense*** Flint, 1979:644 [Type locality: Chile, Prov. Osorno, Pucatrihue; NMNH; ♂]. —Schmid, 1989:147 [♂]. —Angrisano, 1999:26 [checklist].

**Distribution.** Chile.

#### Genus *Apatanodes* Navás [2]

*Apatanodes* Navás, 1934a:177 [Type species: *Apatanodes sociata* Navás, 1934a, original designation]. —Schmid, 1955b:224 [transferred to Hydrobiosinae].

*Australochorema* Schmid, 1955a:129 [Type species: *Australochorema rectispina* Schmid, 1955a, original designation]. —Schmid, 1989:88 [redescription]. —Angisano, 1997a:15 [immatures stages]. —Flint et al., 1999a:75 [to synonymy].

This is a small genus of two species, restricted to the Chilean Subregion. Their immature stages have been described (Angrisano 1997a). Adults are taken at light or by sweeping near small streams in forested areas.

***brachyterga*** (Flint), 1974a:6 [Type locality: Chile, Prov. Chiloe, Río Butalcura; NMNH; ♂; as *Australochorema brachytergum*]. —Flint, 1974e:84 [checklist]. —Angrisano, 1999:27 [checklist]. —Flint et al., 1999a:75 [to *Apatanodes*].

**Distribution.** Chile.

***sociata*** Navás, 1934a:177 [Type locality: Chile, Bio-Bio and Marga-Marga; type depository unknown; ♂]. —Flint, 1974e:84 [checklist]. —Angrisano, 1999:27 [checklist].

—*rectispinum* Schmid, 1955a:130 [Type locality: Chile, Prov. Santiago, Penalolen; NMNH; ♂; as *Australochorema rectispina*]. —Flint, 1974e:84 [checklist]. —Schmid 1989:147 [♂; ♀]. —Angrisano, 1997a:15 [larva; pupa; as *Australochorema rectispinum*]. —Flint et al., 1999a:75 [to synonymy].

**Distribution.** Argentina, Chile.

#### Genus *Atopsyche* Banks [132 + †1]

*Atopsyche* Banks, 1905:17 [Type species: *Atopsyche tripunctata* Banks 1905, original designation]. —Ross and King, 1952:177 [revision; biogeography]. —Schmid, 1989:58 [redescription]. —Gomes and Calor, 2016:51 [review of Brazilian species; key to males].

*Dolochorema* Banks, 1913a:240 [Type species: *Dolochorema irregularis* Banks, 1913a, by monotypy]. —Ross, 1956a:125 [redescription]. —Schmid, 1989:60 [redescription; to subgenus].

*Harpax* Müller, 1921:524 [Type species: no species ever included. Preoccupied by Parkinson, 1811 and 3 others]. —Ulmer, 1957:142 [bibliography; placement; to synonymy].

*Ventrarma* Navás, 1924c:76 [Type species: *Ventrarma implexa* Navás, 1924c, original designation]. —Ross, 1947:127 [to synonmy]. —Ross and King, 1952:177 [as subgenus]. —Ross, 1953:288 [to full synonymy].

*Atopsychodes* Mosely, 1949:37 [Type species: *Atopsychodes cira* Mosely, 1949, original designation]. —Ross and King, 1952:177 [to synonymy].

*Atopsaura* Ross, 1953:292 [Type species: *Atopsyche hamata* Ross and King, 1952, original designation, as a subgenus].

This is by far the largest genus in the family, with 132 extant and 1 fossil species. It has the widest distribution of the New World genera. Species are known from the southwestern tier of states in the United States, south throughout Central and South America, including the Greater Antilles, but stopping short of the Chilean Subregion. Many locally endemic species occur in the tropical Andes.

The larvae and pupae of a few species in the genus have been described (Flint 1963a, Wiggins 1996). They are all free-living predators, found in rapidly flowing water, from small streams to rivers or even in hygropetric habitats. Reynaga and Rueda Martín (2010) found little niche overlap between *Atopsyche yunguensis* and *Atopsyche spinosa*. The former fed mostly on Trichoptera and the latter on Diptera, capturing prey with its chelate forelegs.

***acahuana*** (*Atopsaura*) Schmid, 1989:117 [Type locality: Brazil, Edo. ES [Espirito Santo], 15 km SE Santa Teresa, Fazenda Santa Clara; MZUSP; ♂]. —Angrisano, 1999:26 [checklist]. —Paprocki et al., 2004:7 [checklist]. —Blahnik et al., 2004:4 [distribution]. —Dumas et al., 2009:364 [distribution]. —Dumas and Nessimian, 2012:25 [distribution]. —Paprocki and França, 2014:19 [checklist]. —Gomes and Calor, 2016:53 [redescription; distribution].

**Distribution.** Brazil.

***allani*** (*Atopsyche*) Holzenthal and Cressa, 2002:136 [Type locality: Venezuela, Estado Mérida, Río Sinigüis at ‘El Molino’, 8°34.76'N, 70°58.33'W, el. 2340 m; NMNH; ♂].

**Distribution.** Venezuela.

***antisuya*** (*Atopsaura*) Schmid, 1989:117 [Type locality: Brazil, Edo. MG [Minas Gerais], Serra do Cipó, km 116, Rio Brauninha; MZUSP; ♂]. —Angrisano, 1999:26 [checklist]. —Paprocki et al., 2004:7 [checklist]. —Paprocki and França, 2014:19 [checklist]. —Gomes and Calor, 2016:54 [redescription; distribution].

**Distribution.** Brazil.

***aplita*** (*Atopsyche*) Ross and King, 1952:192 [Type locality: Mexico, Rio Frio; INHS; ♂]. —Bueno-Soria and Flint, 1978:191 [distribution].

**Distribution.** Mexico.

***apurimac*** (*Atopsaura*) Schmid, 1989:118 [Type locality: Brazil, Edo. RJ [Rio de Janeiro], km 18, 18 km S of Teresopolis; MZUSP; ♂]. —Angrisano, 1999:26 [checklist]. —Paprocki et al., 2004:7 [checklist]. —Dumas et al., 2009:364 [distribution]. —Paprocki and França, 2014:19 [checklist]. —Gomes and Calor, 2016:54 [redescription; distribution].

**Distribution.** Brazil.

***asancaru*** (*Atopsaura*) Schmid, 1989:118 [Type locality: Venezuela, Edo. Bolivar, 10 km S of km 88, at Piedra de Virgen; NMNH; ♂]. —Holzenthal and Cressa, 2002:134 [checklist].

**Distribution.** Venezuela.

***atahuallpa*** (*Atopsaura*) Schmid, 1989:118 [Type locality: Venezuela, Edo. Bolivar, Ptari-tepui, 30 mi N Kavanayen; NMNH; ♂]. —Holzenthal and Cressa, 2002:134 [checklist].

**Distribution.** Venezuela.

***ayacucho*** (*Atopsaura*) Schmid, 1989:119 [Type locality: Venezuela, T.F.A. [Territorio Federal Amazonas], Cerro d.l. Neblina, camp II, 0°49'N, 65°59'W; NMNH; ♂]. —Holzenthal and Cressa, 2002:134 [checklist].

**Distribution.** Venezuela.

***ayahuaca*** (*Atopsaura*) Schmid, 1989:119 [Type locality: Venezuela, T.F.A. [Territorio Federal Amazonas], Cerro d.l. Neblina, camp II, 0°49'N, 65°59'W; NMNH; ♂]. —Holzenthal and Cressa, 2002:134 [checklist].

**Distribution.** Venezuela.

***banksi*** (*Atopsyche*) Ross, 1953:290 [Type locality: Colombia, San Antonio; MCZ; ♂]. —Sykora, 1991:250 [distribution]. —Muñoz-Quesada, 2000:275 [checklist].

**Distribution.** Colombia, Ecuador.

***batesi*** (*Atopsaura*) Banks, 1938:304 [Type locality: Haiti, La Vesite, La Selle Range; MCZ; ♂]. —Ross and King, 1952:198 [♂]. —Flint, 1967c:2 [♂ lectotype]. —Flint, 1968b:79 [checklist]. —Flint and Pérez-Gelabert, 1999:37 [checklist]. —Botosaneanu, 2002:80 [checklist]. —Flint and Sykora, 2004:14 [distribution]. —Pérez-Gelabert, 2008:299 [checklist].

**Distribution.** Haiti.

***bicolorata*** (unplaced) Schmid, 1958b:190 [Type locality: Bolivia, Dpto. Cochabamba, Incachaca; NMNH; ♂]. —Angrisano, 1999:27 [checklist]. —Cohen, 2004:76 [distribution].

**Distribution.** Bolivia, Peru.

***bispinosa*** (*Dolochorema*) Schmid, 1989:120 [Type locality: Bolivia, Dpto. La Paz, Unduavi-Coroico; NMNH; ♂]. —Angrisano, 1999:27 [checklist].

**Distribution.** Bolivia.

***blahniki*** (*Atopsaura*) Santos and Holzenthal, 2012:66 [Type locality: Brazil, Rio de Janeiro, Cachoeiras de Macacu, Rio Souza, 16°26.567'S, 42°37.957'W, 150 m; MZUSP; ♂]. —Paprocki and França, 2014:19 [checklist]. —Gomes and Calor, 2016:55 [redescription; distribution].

**Distribution.** Brazil.

***bolivari*** (*Atopsyche*) Banks, 1924:443 [Type locality: Colombia, Dpto. Tolima, Monte Socorro, Tochecito, Quindini; MCZ; ♂]. —Ross and King, 1952:195 [♂]. —Flint, 1967c:2 [♂ lectotype]. —Muñoz-Quesada, 2000:275 [checklist].

**Distribution.** Colombia.

***boneti*** (*Atopsyche*) Ross and King, 1952:194 [Type locality: Mexico, Edo. Morelos, Cuernavaca; INHS; ♂]. —Flint, 1967d:163 [distribution]. —Bueno-Soria and Flint, 1978:191 [distribution].

**Distribution.** Mexico.

***brachycerca*** (*Atopsaura*) Flint, 1968a:10 [Type locality: Jamaica, Portland Parish, Hardwar Gap, “Green Hills”; NMNH; ♂; ♀; larva; pupa]. —Flint, 1968b:79 [checklist]. —Botosaneanu, 2002:80 [checklist].

**Distribution.** Jamaica.

***cajas*** (unplaced) Harper and Turcotte, 1985:134 [Type locality: Ecuador, small stream, outlet of Laguna Verde Cocha, near its junction with Rio Matadero, Chirimachay, Quinuas Valley; UMQ; ♂].

**Distribution.** Ecuador.

***calahuaya*** (*Atopsaura*) Schmid, 1989:120 [Type locality: Venezuela, Edo. Bolivar, Ptari-tepui, 30 mi N Kavanayen; NMNH; ♂]. —Holzenthal and Cressa, 2002:134 [checklist].

**Distribution.** Venezuela.

***callosa*** (*Atopsaura*) (Navás), 1924c:78 [Type locality: Costa Rica; MNHNP; ♂; in *Ventrarma*]. —Flint, 1975:566 [synonymy]. —McElravy et al., 1981:152 [distribution]. —Holzenthal, 1988c:53 [distribution]. —Sykora, 1991:250 [distribution]. —Flint, 1991:18 [♂; distribution]. —Aguila, 1992:533 [distribution]. —Flint, 1996b:379 [distribution]. —Angrisano, 1999:26 [checklist]. —Muñoz-Quesada, 2000:275 [checklist]. —Holzenthal and Cressa, 2002:134 [distribution]. —Rueda Martín, 2006a:58 [larva; pupa; distribution]. —Isa Miranda and Rueda Martín, 2014:199 [distribution]. —Armitage et al., 2015b:8 [checklist]. —Armitage and Cornejo, 2015:197 [checklist].

—*alconura* (*Atopsaura*) Ross, 1953:288 [Type locality: Peru, Dpto. Cuzco, Pcia. Paucartambo, Cosnipata Valley; INHS; ♂]. —Flint, 1975:566 [to synonymy].

—*schmidi* (*Atopsaura*) Denning, 1965c:267 [Type locality: Costa Rica, San Jose; CAS; ♂]. —Flint, 1975:566 [to synonymy].

**Distribution.** Argentina, Bolivia, Colombia, Costa Rica, Ecuador, Panama, Peru, Venezuela.

***calopta*** (*Atopsyche*) Ross and King, 1952:188 [Type locality: Mexico, Edo. Morelos, Cuernavaca; INHS; ♂]. —Flint, 1967d:163 [distribution]. —Bueno-Soria and Flint, 1978:191 [distribution]. —Rojas-Ascencio, et al., 2002:377 [distribution]. —Bueno-Soria et al., 2007:32 [distribution]. —Bueno-Soria and Barba-Álvarez, 2011:355 [checklist].

**Distribution.** Mexico.

***caquetia*** (*Atopsyche*) Flint, 1974a:2 [Type locality: Venezuela, Edo. Aragua, Rancho Grande; NMNH; ♂]. —Flint, 1981a:7 [♂; distribution]. —Holzenthal and Cressa, 2002:135 [distribution].

**Distribution.** Venezuela.

***catherinae*** (*Atopsyche*) Harper and Turcotte, 1985:136 [Type locality: Ecuador, small stream, outlet of Laguna Verde Cocha, near its junction with Rio Matadero, Chirimachay, Quinuas Valley; UMQ; ♂].

**Distribution.** Ecuador.

***chimpuocllo*** (*Atopsyche*) Schmid, 1989:120 [Type locality: Costa Rica, Prov. Cartago, Tapantí; SDNHM, on deposit NMNH; ♂]. —Armitage et al., 2015a:6 [distribution]. —Armitage et al., 2015b:8 [checklist]. —Armitage and Cornejo, 2015:197 [checklist].

**Distribution.** Costa Rica, Panama.

***chimuru*** (*Atopsaura*) Schmid, 1989:121 [Type locality: Venezuela, T.F.A. [Territorio Federal Amazonas], Cerro d.l. Neblina, camp II, 0°49'N, 65°59'W; NMNH; ♂]. —Holzenthal and Cressa, 2002:135 [checklist].

**Distribution.** Venezuela.

***chinchacamac*** (*Atopsaura*) Schmid, 1989:121 [Type locality: Venezuela, T.F.A. [Territorio Federal Amazonas], Cerro d.l. Neblina, camp II, 0°49'N, 65°59'W; NMNH; ♂]. —Holzenthal and Cressa, 2002:135 [checklist].

**Distribution.** Venezuela.

***chirihuana*** (*Atopsyche*) Schmid, 1989:121 [Type locality: Ecuador, Prov. Pichincha, Santo Domingo (47 km S); NMNH; ♂]. —Paprocki et al., 2004:7 [checklist]. —Blahnik et al., 2004:4 [distribution]. —Paprocki and França, 2014:21 [checklist]. —Gomes and Calor, 2016:55 [redescription; distribution].

**Distribution.** Brazil, Ecuador.

***chirimachaya*** (unplaced) Harper and Turcotte, 1985:135 [Type locality: Ecuador, small stream, outlet of Laguna Verde Cocha, near its junction with Rio Matadero, Chirimachay, Quinuas Valley; UMQ; ♂].

**Distribution.** Ecuador.

***choronica*** (*Atopsaura*) Flint, 1974a:3 [Type locality: Venezuela, Edo. Aragua, Choroni Pass; NMNH; ♂]. —Flint, 1981a:7 [♂; distribution]. —Holzenthal and Cressa, 2002:135 [checklist].

**Distribution.** Venezuela.

***cira*** (*Atopsyche*) (Mosely), 1949:37 [Type locality: Costa Rica, Irazu; BMNH; ♂; in *Atopsychodes*]. —Ross and King, 1952:190 [♂; to *Atopsyche*]. —Holzenthal, 1988c:53 [distribution]. —Armitage et al., 2015a:7 [distribution]. —Armitage et al., 2015b:8 [checklist]. —Armitage and Cornejo, 2015:197 [checklist].

**Distribution.** Costa Rica, Panama.

***clarkei*** (*Atopsaura*) Flint, 1963a:456 [Type locality: Colombia, Dpto. Cundinamarca, Chicó; NMNH; ♂]. —Sykora, 1991:250 [distribution]. —Muñoz-Quesada, 2000:275 [checklist].

**Distribution.** Colombia, Ecuador.

***conventica*** (*Atopsaura*) Flint, 1974a:4 [Type locality: Dominican Republic, Convento, 12 km S Constanza; NMNH; ♂]. —Flint and Pérez-Gelabert, 1999:37 [checklist]. —Botosaneanu, 2002:80 [checklist]. —Flint and Sykora, 2004:14 [distribution]. —Pérez-Gelabert, 2008:299 [checklist].

**Distribution.** Dominican Republic.

***copayapu*** (*Atopsyche*) Schmid, 1989:122 [Type locality: Ecuador, Prov. Pichincha, Santo Domingo de los Colorados, 14 km E; NMNH; ♂].

**Distribution.** Ecuador.

***cordoba*** (*Atopsyche*) Denning, 1968a:17 [Type locality: Mexico, Edo. Veracruz, Cordoba; CAS; ♂]. —Bueno-Soria and Flint, 1978:191 [distribution]. —Chamorro-Lacayo et al., 2007:41 [checklist].

**Distribution.** Mexico, Nicaragua.

***cubana*** (*Atopsaura*) Flint, 1968c:151 [Type locality: Cuba; MCZ; ♂]. —Flint, 1968b:79 [checklist]. —Botosaneanu, 1979:43 [distribution]. —Kumanski, 1987:3 [distribution]. —Flint, 1996c:16 [checklist]. —Botosaneanu, 2002:80 [checklist]. —Naranjo López and González Lazo, 2005:149 [checklist].

**Distribution.** Cuba.

***dampfi*** (*Atopsyche*) Ross and King, 1952:194 [Type locality: Mexico, Edo. Chiapas, Finca Vergel; INHS; ♂]. —Bueno-Soria and Flint, 1978:191 [distribution]. —McElravy et al., 1981:152 [distribution]. —Holzenthal, 1988c:53 [distribution]. —Aguila, 1992:533 [distribution]. —Maes, 1999:1195 [checklist]. —Rojas-Ascencio, et al., 2002:377 [distribution]. —Chamorro-Lacayo et al., 2007:41 [checklist]. —Bueno-Soria and Barba-Álvarez, 2011:355 [checklist]. —Armitage et al., 2015b:8 [checklist]. —Armitage and Cornejo, 2015:197 [checklist].

**Distribution.** Costa Rica, Guatemala, Honduras, Mexico, Nicaragua, Panama.

***davidsoni*** (unplaced) Sykora, 1991:246 [Type locality: Ecuador, Prov. Bolivar, 16 km NNE Guaranda; CMNH; ♂].

**Distribution.** Ecuador.

***davisorum*** (*Atopsaura*) Flint, 1974a:4 [Type locality: Dominican Republic, 4 km SE of Rio Limpio; NMNH; ♂]. —Flint and Pérez-Gelabert, 1999:37 [checklist]. —Botosaneanu, 2002:80 [checklist]. —Flint and Sykora, 2004:14 [distribution]. —Pérez-Gelabert, 2008:299 [checklist].

**Distribution.** Dominican Republic.

***diamantina*** (*Atopsaura*) Flint, 1974a:4 [Type locality: Dominican Republic, 4 km SE of Rio (*Atopsaura*) Gomes and Calor, 2016:66 [Type locality: Brazil, Bahia, Mucugê, Chapada Diamantina, Parque Municipal Sempre Viva, Rio Piabinha, 12°59'34"S, 41°20'27"W, el. 921 m; MZUSP; ♂].

**Distribution.** Brazil.

***erigia*** (*Atopsyche*) Ross, 1947:129 [Type locality: Mexico, Edo. Tamaulipas, Hacienda Santa Engracia; INHS; ♂]. —Ross and King, 1952:188 [♂; distribution]. —Edwards and Arnold, 1961:401 [larva; distribution]. —Bueno-Soria and Flint, 1978:191 [distribution]. —Maes and Flint, 1988:2 [distribution]. —Holzenthal, 1988c:53 [distribution]. —Aguila, 1992:533 [distribution]. —Maes, 1999:1195 [checklist]. —Paprocki et al., 2004:7 [checklist]. —Blahnik et al., 2004:4 [distribution]. —Bowles et al., 2007:21 [distribution; biology]. —Chamorro-Lacayo et al., 2007:41 [checklist]. —Bueno-Soria and Barba-Álvarez, 2011:355 [checklist]. —Calor, 2011:320 [checklist]. —Paprocki and França, 2014:21 [checklist]. —Armitage et al., 2015b:8 [checklist]. —Armitage and Cornejo, 2015:197 [checklist]. —Gomes and Calor, 2016:55 [redescription; distribution].

**Distribution.** Brazil, Costa Rica, Guatemala, Nicaragua, Mexico, Panama, U.S.A.

***espala*** (*Atopsyche*) Ross and King, 1952:190 [Type locality: Mexico, Edo. Chiapas, Tecpatan; INHS; ♂]. —Bueno-Soria and Flint, 1978:191 [distribution]. —Bueno-Soria and Barba-Álvarez, 2011:355 [checklist].

**Distribution.** Mexico.

***explanata*** (*Atopsyche*) Ross, 1953:288 [Type locality: Peru, Dpto. Cuzco, Pcia. Paucartambo, Cosnipata Valley; INHS; ♂].

**Distribution.** Peru.

***flinti*** (unplaced) Sykora, 1991:244 [Type locality: Ecuador, Prov. Chimborazo, 21 km ESE Licto, stream above Río Alao; CMNH; ♂].

**Distribution.** Ecuador.

***galharada*** (*Atopsaura*) Santos and Holzenthal, 2012:71 [Type locality: Brazil, São Paulo, Campos do Jordão, Parque Estadual de Campos do Jordão, Rio Galharada, 22°41.662'S, 45°27.783'W, 1530 m; MZUSP; ♂]. —Paprocki and França, 2014:19 [checklist]. —Gomes and Calor, 2016:56 [redescription; distribution].

**Distribution.** Brazil.

***hamata*** (*Atopsaura*) Ross and King, 1952:202 [Type locality: Brazil, Summit of Mt. Roraima; AMNH; ♂]. —Angrisano, 1999:26 [checklist]. —Paprocki et al., 2004:7 [checklist]. —Paprocki and França, 2014:20 [checklist]. —Gomes and Calor, 2016:56 [redescription; distribution].

**Distribution.** Brazil.

***hatunpuna*** (*Atopsaura*) Schmid, 1989:122 [Type locality: Brazil, Edo. SP [Sao Paulo], Casa Grande, Ribera Curuja; MZUSP; ♂]. —Angrisano, 1999:26 [checklist]. —Paprocki et al., 2004:7 [checklist]. —Dumas et al., 2010:7 [distribution]. —Calor, 2011:320 [checklist]. —Dumas and Nessimian, 2012:10 [distribution]. —Paprocki and França, 2014:20 [checklist]. —Gomes and Calor, 2016:57 [diagnosis; distribution].

**Distribution.** Brazil.

***hidalgoi*** (*Atopsyche*) Flint, 1967b:2 [Type locality: Mexico, Edo. Mexico, Las Cruces National Park, La Marquesa; NMNH; ♂]. —Bueno-Soria and Flint, 1978:191 [distribution].

**Distribution.** Mexico.

***hinnulus*** (unplaced) Flint and Sykora, 2004:14 [Type locality: Dominican Republic, Pedernales Province, Río Mulito, 21 km N Pedernales, 18°09.3'N, 71°45.6'W, 270 m; NMNH; ♂; unplaced to subgenus, but referred to *batesi* Group of subgenus *Atopsaura*]. —Pérez-Gelabert, 2008:299 [checklist].

**Distribution.** Dominican Republic.

***hintoni*** (*Atopsyche*) Denning, 1964:129 [Type locality: Mexico, Edo. Baja California, Arroyo San Bernadino, about 3 miles northwest Miraflores; CAS; ♂; larva; pupa]. —Bueno-Soria and Flint, 1978:191 [distribution].

**Distribution.** Mexico.

***hispida*** (*Atopsyche*) Denning, 1965c:266 [Type locality: Mexico, Edo. Veracruz, Fortin de los Flores; CAS; ♂]. —Bueno-Soria and Flint, 1978:191 [distribution].

**Distribution.** Mexico.

***huacachaca*** (*Atopsaura*) Schmid, 1989:122 [Type locality: Brazil, Edo. Rio Jan. [Rio de Janeiro], Itatiaia, Registro Pass; MZUSP; ♂]. —Angrisano, 1999:26 [checklist]. —Paprocki et al., 2004:7 [checklist]. —Dumas et al., 2009:365 [distribution]. —Paprocki and França, 2014:20 [checklist]. —Gomes and Calor, 2016:57 [redescription; distribution].

**Distribution.** Brazil.

***huacapuncu*** (*Atopsaura*) Schmid, 1989:123 [Type locality: Venezuela, Edo. Merida, S Bolivar N.P., near La Aguada; NMNH; ♂]. —Holzenthal and Cressa, 2002:135 [distribution].

**Distribution.** Venezuela.

***huachacuyac*** (*Atopsaura*) Schmid, 1989:123 [Type locality: Venezuela, Edo. Merida, Mucuy Fish Hatchery, 7 km E Tabay; NMNH; ♂]. —Holzenthal and Cressa, 2002:135 [distribution].

**Distribution.** Venezuela.

***huainacapac*** (*Atopsaura*) Schmid, 1989:123 [Type locality: Costa Rica, Prov. Alajuela, Cerro Campana, ca. 6 km (air) NW Dos Rios, 10.9°N, 85.4°W; NMNH; ♂]. —Armitage et al., 2015a:7 [distribution]. —Armitage et al., 2015b:8 [checklist]. —Armitage and Cornejo, 2015:197 [checklist].

**Distribution.** Costa Rica, Panama.

***huallaripa*** (*Atopsaura*) Schmid, 1989:124 [Type locality: Venezuela, T.F.A. [Territorio Federal Amazonas], Cerro d.l. Neblina, camp II, 0°49'N, 65°59'W; NMNH; ♂]. —Holzenthal and Cressa, 2002:135 [checklist].

**Distribution.** Venezuela.

***huamachucu*** (*Atopsaura*) Schmid, 1989:124 [Type locality: Brazil, Edo. RJ [Rio de Janeiro], km 17, 18 km S of Teresopolis; MZUSP; ♂]. —Angrisano, 1999:26 [checklist]. —Paprocki et al., 2004:7 [checklist]. —Dumas et al., 2009:365 [distribution]. —Dumas and Nessimian, 2012:11 [distribution]. —Paprocki and França, 2014:20 [checklist]. —Gomes and Calor, 2016:58 [redescription; distribution].

**Distribution.** Brazil.

***huanapu*** (*Atopsaura*) Schmid, 1989:124 [Type locality: Brazil, Edo. SP [São Paulo], E.B. Boraceia, Paredo da Pedreira; MZUSP; ♂]. —Angrisano, 1999:26 [checklist]. —Paprocki et al., 2004:7 [checklist]. —Blahnik et al., 2004:4 [distribution]. —Dumas et al., 2009:365 [distribution]. —Calor, 2011:320 [checklist]. —Dumas and Nessimian, 2012:11 [distribution]. —Paprocki and França, 2014:20 [checklist]. —Gomes and Calor, 2016:58 [redescription; distribution].

**Distribution.** Brazil.

***huanucu*** (*Atopsaura*) Schmid, 1989:125 [Type locality: Venezuela, Edo. Lara, Yacambu National Park, 13 km SE Sanare; NMNH; ♂]. —Holzenthal and Cressa, 2002:135 [distribution].

**Distribution.** Venezuela.

***huarcu*** (*Atopsaura*) Schmid, 1989:125 [Type locality: Brazil, Edo. Minas Gerais, Nova Lima; MZUSP; ♂]. —Angrisano, 1999:26 [checklist]. —Paprocki et al., 2004:7 [checklist]. —Blahnik et al., 2004:4 [distribution]. —Dumas et al., 2009:365 [distribution]. —Calor, 2011:320 [checklist]. —Dumas and Nessimian, 2012:11 [distribution]. —Paprocki and França, 2014:20 [checklist]. —Gomes and Calor, 2016:58 [redescription; distribution].

**Distribution.** Brazil.

***huenga*** (*Atopsyche*) Flint, 1974a:2 [Type locality: Guatemala, Dpto. Huehuetenango, 20 mi NW of Huehuetenango; NMNH; ♂]. —Bueno-Soria and Flint, 1978:191 [distribution]. —Maes, 1999:1195 [checklist]. —Chamorro-Lacayo et al., 2007:41 [checklist]. —Bueno-Soria and Barba-Álvarez, 2011:355 [checklist].

**Distribution.** Guatemala, Mexico, Nicaragua.

***iana*** (*Atopsaura*) Mosely, 1949:40 [replacement name for *Atopsyche spinosa* Mosely, 1931:545, preoccupied by *Ventrarma spinosa* Navás, 1930c:131]. [Type locality: British Guiana, Kaieteur; BMNH; ♂]. —Ross and King, 1952:200 [♂]. —Angrisano, 1999:26 [checklist].

**Distribution.** Guyana.

***ikonnikovi*** (*Atopsyche*) Martynov, 1912:34 [Type locality: Peru, La Merced; type depository unknown; ♂]. —Ross and King, 1952:195 [♂]. —Angrisano, 1999:27 [checklist].

**Distribution.** Peru.

***implexa*** (*Atopsyche*) (Navás), 1924c:77 [Type locality: Costa Rica, La Caja; MNHNP; ♂; in *Ventrarma*]. —Ross and King, 1952:197. —Ross, 1953: 291 [♂]. —McElravy et al., 1981:152 [distribution]. —Holzenthal, 1988c:53 [distribution]. —Aguila, 1992:533 [distribution]. —Chamorro-Lacayo et al., 2007:41 [checklist]. —Armitage et al., 2015b:8 [checklist]. —Armitage and Cornejo, 2015:197 [checklist].

**Distribution.** Costa Rica, Nicaragua, Panama.

***incatupac*** (*Atopsyche*) Schmid, 1989:125 [Type locality: Ecuador, Prov. Cotopaxi, 113 km W Latacunga; NMNH; ♂].

**Distribution.** Ecuador.

***irregularis*** (*Dolochorema*) (Banks), 1913a:240 [Type locality: Peru, S.E., Cuzco; MCZ; ♂; in *Dolochorema*]. —Ross, 1956a:125 [♂]. —Schmid, 1989:60 [to *Atopsyche*].

**Distribution.** Peru.

***jaba*** (unplaced) Blahnik and Gottschalk, 1997:162 [Type locality: Costa Rica, Puntarenas, Rio Jaba at rock quarry, 1.4 km (air) W Las Cruces, 8.79°N, 82.97°W; NMNH; ♂]. —Armitage et al., 2015a:7 [distribution]. —Armitage et al., 2015b:8 [checklist]. —Armitage and Cornejo, 2015:197 [checklist].

**Distribution.** Costa Rica, Panama.

***janethae*** (*Atopsyche*) Harper and Turcotte, 1985:134 [Type locality: Ecuador, small stream, outlet of Laguna Verde Cocha, near its junction with Rio Matadero, Chirimachay, Quinuas Valley; UMQ; ♂].

**Distribution.** Ecuador.

***japoda*** (*Atopsaura*) Ross and King, 1952:202 [Type locality: Mexico; INHS; ♂]. —Bueno-Soria and Flint, 1978:191 [distribution]. —Chamorro-Lacayo et al., 2007:41 [checklist].

**Distribution.** Mexico, Nicaragua.

***kamakan*** (*Atopsaura*) Gomes and Calor, 2016:68 [Type locality: Brazil, Bahia, Camacan, Reserva Particular do Patrimônio Natural Serra Bonita, 15°23'02"S, 39°34'10"W; MZUSP; ♂].

**Distribution.** Brazil.

***kamesa*** (*Atopsyche*) Ross and King, 1952:196 [Type locality: Bolivia, Incachaca; CMNH; ♂]. —Angrisano, 1999:27 [checklist]. —Rueda Martín, 2006a:60 [distribution].

**Distribution.** Argentina, Bolivia.

***kingi*** (*Atopsyche*) Ross, 1953:289 [Type locality: Peru, Dpto. Cuzco, Pcia. Paucartambo, Cosnipata Valley; INHS; ♂]. —Flint, 1996b:380 [distribution].

**Distribution.** Peru.

***lilicae*** (*Atopsaura*) Botosaneanu, 1991a:114 [Type locality: Haiti, Dpartement de l’Ouest, Vile Bonheur (Ville Saut d’Eau); ZMUA; ♂]. —Flint and Pérez-Gelabert, 1999:37 [checklist]. —Botosaneanu, 2002:80 [checklist]. —Flint and Sykora, 2004:14 [distribution]. —Pérez-Gelabert, 2008:299 [checklist].

**Distribution.** Haiti.

***lobosa*** (*Atopsaura*) Ross and King, 1952:200 [Type locality: Bolivia, Incachaca; CMNH; ♂]. —Flint, 1996b:380 [distribution]. —Angrisano, 1999:26 [checklist].

**Distribution.** Bolivia, Peru.

***longipennis*** (*Atopsaura*) (Ulmer), 1905a:110 [Type locality: not given; ZSZMH; ♂; as *Psilochorema longipenne*]. —Ulmer 1909b:73 [to *Atopsyche*, as *Atopsyche brasiliana*, a lapsus for *longipennis*, see Ulmer 1913:384]. —Ross and King, 1952:202 [type from Brazil, Santa Catharina [sic]; ♂]. —Flint, 1972b:225 [distribution]. —Angrisano, 1999:27 [checklist]. —Paprocki et al., 2004:7 [checklist]. —Paprocki and França, 2014:20 [checklist]. —Gomes and Calor, 2016:59 [redescription; distribution].

**Distribution.** Argentina, Brazil.

***macrocerca*** (*Atopsaura*) Flint, 1968a:12 [Type locality: Jamaica, St. Andrew Parish, Hope River near Newcastle at mile post 16.5; NMNH; ♂; ♀; larva; pupa]. —Flint, 1968b:79 [checklist]. —Botosaneanu, 2002:80 [checklist].

**Distribution.** Jamaica.

***maitacapac*** (*Atopsyche*) Schmid, 1989:126 [Type locality: Ecuador, Prov. Napo, Lago Agrio (30 km E) via a Tarapoa; NMNH; ♂].

**Distribution.** Ecuador.

***majada*** (*Atopsaura*) Ross, 1947:129 [Type locality: Mexico, Edo. Michoacan, La Majada, Apatazingan; INHS; ♂]. —Ross and King, 1952:197 [♂]. —Bueno-Soria and Flint, 1978:191 [distribution]. —McElravy et al., 1981:152 [distribution]. —Maes and Flint, 1988:2 [distribution]. —Holzenthal, 1988c:53 [distribution]. —Aguila, 1992:533 [distribution]. —Maes, 1999:1195 [checklist]. —Rojas-Ascencio, et al., 2002:377 [distribution]. —Chamorro-Lacayo et al., 2007:41 [checklist]. —Bueno-Soria and Barba-Álvarez, 2011:355 [checklist]. —Armitage et al., 2015b:8 [checklist]. —Armitage and Cornejo, 2015:197 [checklist].

**Distribution.** Belize, Costa Rica, Guatemala, Honduras, Nicaragua, Mexico, Panama.

***major*** (*Dolochorema*) Schmid, 1989:126 [Type locality: Bolivia, Prov. La Paz, Rio Zongo; NMNH; ♂]. —Angrisano, 1999:27 [checklist].

**Distribution.** Bolivia.

***mancocapac*** (*Atopsyche*) Schmid, 1989:126 [Type locality: Ecuador, Prov. Pastaza, Puyo (3 km North); NMNH; ♂]. —Flint, 1996b:380 [distribution].

**Distribution.** Ecuador, Peru.

***maxi*** (*Atopsyche*) Rueda Martín, 2005:294 [Type locality: Argentina, Tucumán Prov., Trancas, Gonzalo; IML; ♂; larva; pupa]. —Isa Miranda and Rueda Martín, 2014:199 [distribution].

**Distribution.** Argentina.

***mayucapac*** (*Atopsaura*) Schmid, 1989:127 [Type locality: Venezuela, Edo. Merida, El Valle; IZAM; ♂]. —Holzenthal and Cressa, 2002:135 [distribution].

**Distribution.** Venezuela.

***mexicana*** (unplaced) (Banks), 1901:370 [Type locality: Mexico, Edo. Veracruz, Jalapa; MCZ; ♂; in *Philopotamus*]. —Ross, 1953:293 [to *Atopsyche*, lacks abdomen]. —Flint, 1967c:2 [abdomen missing]. —Bueno-Soria and Flint, 1978:192 [distribution]. —Schmid 1989:144 [*nomen dubium*].

**Distribution.** Mexico.

***milenae*** (unplaced) Sykora, 1991:248 [Type locality: Ecuador, Prov. Bolivar, 16 km NNE Guaranda; CMNH; ♂].

**Distribution.** Ecuador.

***minimajada*** (unplaced) Blahnik and Gottschalk, 1997:164 [Type locality: Costa Rica, Guanacaste, Estación Pitilla, Río Orosi, 10.931°N, 85.428°W; NMNH; ♂].

**Distribution.** Costa Rica.

***misionensis*** (*Atopsaura*) Flint, 1983a:5 [Type locality: Argentina, Pcia Misiones, Arroyo Piray Mini, Rt. 17 W Dos Hermanas; NMNH; ♂]. —Angrisano, 1999:27 [checklist].

**Distribution.** Argentina.

***muelleri*** (*Atopsaura*) Gomes and Calor, 2016:70 [Type locality: Brazil, Goiás, Chapada dos Veadeiros, Ribeirão Água Fria; MZUSP; ♂].

**Distribution.** Brazil.

***neolobosa*** (*Atopsaura*) Flint, 1963a:456 [Type locality: Ecuador, Papallacta; NMNH; ♂].

**Distribution.** Ecuador.

***neotropicalis*** (*Atopsaura*) Schmid, 1989:127 [Type locality: Peru, Dpto. Cuzco, Quincemil; CNC; ♂]. —Flint, 1996b:379 [distribution].

**Distribution.** Peru.

***onorei*** (unplaced) Sykora, in Flint et al., 1999a:75 [replacement name for *Atopsyche schmidi* Sykora, 1991:246, a junior homonym of *Atopsyche schmidi* Denning, 1965c:267]. [Type locality: Ecuador, Prov. Loja, Cordillera Cordoncillo, 11 km S Saraguro; CMNH; ♂].

**Distribution.** Ecuador.

***orientalis*** (unplaced) Flint and Sykora, 2004:15 [Type locality: Dominican Republic, Seibo Province, 15 km S Miches, ca. 500 m; NMNH; ♀; unplaced to subgenus]. —Pérez-Gelabert, 2008:299 [checklist].

**Distribution.** Dominican Republic.

***pachacamac*** (*Atopsyche*) Schmid, 1989:127 [Type locality: Costa Rica, Prov. San Jose, Rio Chirripo Pacifico, 9.5 km NE Rivas, 9.470°N, 83.591°W; NMNH; ♂].

**Distribution.** Costa Rica.

***pachacutec*** (*Atopsyche*) Schmid, 1989:127 [Type locality: Ecuador, Prov. Cotopaxi, 113 km W Latacunga; NMNH; ♂].

**Distribution.** Ecuador.

***pacharurac*** (*Atopsyche*) Schmid, 1989:127 [Type locality: Venezuela, Edo. Merida, La Campana, 12 km SE Santo Domingo; NMNH; ♂]. —Holzenthal and Cressa, 2002:135 [distribution].

**Distribution.** Venezuela.

***parauna*** (*Atopsyche*) Santos and Holzenthal, 2012:69 [Type locality: Brazil, Minas Gerais, Rio Paraúna, 3 km S Santana do Riacho, 19°10.986'S, 43°43.485'W, 650 m; MZUSP; ♂]. —Paprocki and França, 2014:22 [checklist]. —Gomes and Calor, 2016:59 [diagnosis; distribution].

**Distribution.** Brazil.

***parihuana*** (*Atopsaura*) Schmid, 1989:128 [Type locality: Venezuela, T.F.A. [Territorio Federal Amazonas], Cerro d.l. Neblina, camp IV, 0°58'N, 65°57'W; NMNH; ♂]. —Holzenthal and Cressa, 2002:136 [checklist].

**Distribution.** Venezuela.

***paucartampu*** (*Atopsyche*) Schmid, 1989:129 [Type locality: Costa Rica, Hwy. 2, Km 95, 9°36'N, 83°44'W; NMNH; ♂].

**Distribution.** Costa Rica.

***peravia*** (unplaced) Flint and Sykora, 2004:16 [Type locality: Dominican Republic, Peravia province, 3 km SW La Nuez, upper Río Las Cuevas, 18°39'N, 70°36'W, 1880 m, cloud forest on river; CMNH; ♂; unplaced to subgenus, but compared to Atopsyche (Atopsaura) batesi]. —Pérez-Gelabert, 2008:299 [checklist].

**Distribution.** Dominican Republic.

† ***perlucida*** (unplaced) Wichard, 2007a:4 [Type locality: Dominican Republic; SMNS; ♂; in amber; unplaced to subgenus, but said to be similar to Atopsyche (Atopsyche) rinconi].

**Distribution.** Dominican Republic.

***pilcomayo*** (*Atopsyche*) Schmid, 1989:129 [Type locality: Mexico, Prov. Oaxaca, 53 mi N.E. Guelatao; SDNHM, on indefinite loan to NMNH; ♂].

**Distribution.** Mexico.

***plancki*** (*Atopsaura*) Marlier, 1964a:2 [Type locality: Brazil, Edo. Sao Paulo, Boraceia; IRSNB; ♂]. —Angrisano, 1999:27 [checklist]. —Paprocki et al., 2004:7 [checklist]. —Blahnik et al., 2004:4 [distribution]. —Dumas et al., 2009:365 [distribution]. —Calor, 2011:320 [checklist]. —Santos and Holzenthal, 2012:77 [distribution]. —Paprocki and França, 2014:20 [checklist]. —Gomes and Calor, 2016:60 [redescription; distribution].

**Distribution.** Brazil.

***puharcocha*** (*Atopsaura*) Schmid, 1989:129 [Type locality: Bolivia, Prov. Cochabamba, Rio Ronquito, rd. to Villa Tunari, Chapare; NMNH; ♂]. —Sykora, 1991:250 [distribution]. —Flint, 1996b:380 [distribution]. —Angrisano, 1999:27 [checklist].

**Distribution.** Bolivia, Ecuador, Peru.

***rawlinsi*** (*Atopsaura*) Sykora, 1991:244 [Type locality: Ecuador, Prov. Loja, Cordillera Cordoncillo, 11 km S Saraguro; CMNH; ♂].

**Distribution.** Ecuador.

***rinconi*** (*Atopsyche*) Holzenthal and Cressa, 2002:138 [Type locality: Venezuela, Estado Sucre, Río Cocollar, 1.5 km SE Las Piedras de Cocollar, 10°09.617'N, 63°47.605'W, el. 810 m; NMNH; ♂]. —Gomes and Calor, 2016:60 [redescription; distribution].

**Distribution.** Brazil, Venezuela.

***sanctipauli*** (*Atopsaura*) Flint, 1974a:5 [Type locality: Brazil, Edo. Sao Paulo, Alto da Serra; NMW; ♂]. —Angrisano, 1999:27 [checklist]. —Blahnik et al., 2004:4 [distribution]. —Paprocki et al., 2004:7 [checklist]. —Dumas et al., 2009:365 [distribution]. —Calor, 2011:320 [checklist]. —Dumas and Nessimian, 2012:11 [distribution]. —Paprocki and França, 2014:21 [checklist]. —Gomes and Calor, 2016:61 [♂; redescription; distribution].

**Distribution.** Brazil.

***segninii*** (*Atopsaura*) Holzenthal and Cressa, 2002:140 [Type locality: Venezuela, Estado Mérida, Río Sinigüis, at ‘El Tambor’, Parque Nacional Sierra Nevada, 8°33.34'N, 70°57.87'W, el. 3200 m; NMNH; ♂].

**Distribution.** Venezuela.

***serica*** (*Atopsaura*) Ross, 1953:292 [Type locality: Brazil, Nova Teutonia, 27°11'S, 52°23'W; MCZ; ♂]. —Angrisano, 1999:27 [checklist]. —Paprocki et al., 2004:7 [checklist]. —Paprocki and França, 2014:21 [checklist]. —Gomes and Calor, 2016:62 [redescription; distribution].

**Distribution.** Brazil.

***sinchicurac*** (*Atopsaura*) Schmid, 1989:129 [Type locality: Ecuador, Prov. Zamora Chinchipe, Loja/Zamora; NMNH; ♂].

**Distribution.** Ecuador.

***siolii*** (*Atopsaura*) Flint, 1971c:12 [Type locality: Brazil, [Edo. Amazonas] Rio Marauia, Cachoeira Pora Comeschie, dicht oberhalb Endstation; NMNH; ♂; larva; pupa]. —Angrisano, 1999:27 [checklist]. —Paprocki et al., 2004:7 [checklist]. —Paprocki and França, 2014:21 [checklist]. —Gomes and Calor, 2016:63 [diagnosis; distribution].

**Distribution.** Brazil.

***socialis*** (*Atopsyche*) Flint, 1967d:165 [Type locality: Mexico, Edo. Durango, 10 miles west of El Salto; CNC; ♂]. —Bueno-Soria and Flint, 1978:192 [distribution].

**Distribution.** Mexico.

***sperryi*** (*Atopsyche*) Denning, 1949:88. [Type locality: United States, Arizona, Oak Creek Canyon, Todd’s Lodge; CAS; ♂]. —Ross and King, 1952:190 [♂]. —Flint, 1967d:163 [distribution]. —Bueno-Soria and Flint, 1978:192 [distribution]. —Blinn and Ruiter, 2006:332 [ecology]. —Blinn and Ruiter, 2009a:303 [biology]. —Blinn and Ruiter, 2009b:185 [phenology, distribution]. —Ruiter and Blinn, 2009:4 [♀].

**Distribution.** Mexico, U.S.A.

***spinosa*** (*Atopsaura*) (Navás), 1930c:131 [Type locality: Argentina, Prov. Buenos Aires, Palo Blanco; UNLP; ♂; in *Ventrarma*]. —Flint, 1982c:15 [distribution]. —Angrisano, 1999:27 [checklist]. —Cohen, 2004:76 [distribution]. —Rueda Martín, 2006a:56 [larva; pupa; distribution]. —Reynaga and Rueda Martín, 2010:61 [trophic biology]. —Isa Miranda and Rueda Martín, 2014:199 [distribution; as *espinosa*].

—*falina* (*Atopsaura*) Ross and King, 1952:198 [Type locality: Argentina, Prov. Tucuman, Tafi del Valle; IML; ♂]. —Flint et al., 1999a:75 [to synonymy]. —Cohen, 2004:74 [list of type material].

**Distribution.** Argentina, Bolivia.

***taina*** (*Atopsaura*) Flint, 1974a:4 [Type locality: Dominican Republic, Convento, 12 km S Constanza; NMNH; ♂]. —Flint and Pérez-Gelabert, 1999:37 [checklist]. —Botosaneanu, 2002:80 [checklist]. —Flint and Sykora, 2004:16 [distribution]. —Pérez-Gelabert, 2008:299 [checklist].

**Distribution.** Dominican Republic.

***talamanca*** (*Atopsyche*) Flint, 1974a:2 [Type locality: Costa Rica, Prov. Cartago, route 2, km 75; NMNH; ♂]. —Holzenthal, 1988c:54 [distribution].

**Distribution.** Costa Rica.

***tampurimac*** (*Atopsyche*) Schmid, 1989:130 [Type locality: Ecuador, Prov. Napo, San Franciso de Borja; NMNH; ♂]. —Flint, 1991:19 [♂; distribution]. —Muñoz-Quesada, 2000:275 [checklist]. —Medellín et al., 2004:201 [biology].

**Distribution.** Colombia, Ecuador.

***tapanti*** (unplaced) Blahnik and Gottschalk, 1997:167 [Type locality: Costa Rica, Cartago, Reserva Tapantí, Río Grande de Orosi, 9.686°N, 83.756W; NMNH; ♂].

**Distribution.** Costa Rica.

***thomasi*** (unplaced) Flint and Sykora, 2004:16 [Type locality: Haiti, Département de l’Ouest, saddle between Fe Noir and Enfer, 1700 m; FSCA; ♂; unplaced to subgenus, but compared to Atopsyche (Atopsaura) lilicae]. —Pérez-Gelabert, 2008:299 [checklist].

**Distribution.** Haiti.

***tincuracu*** (*Atopsaura*) Schmid, 1989:130 [Type locality: Bolivia, Prov. La Paz, Unduavi/Coroico; NMNH; ♂]. —Angrisano, 1999:27 [checklist].

**Distribution.** Bolivia.

***tlaloc*** (*Atopsyche*) Schmid, 1989:130 [Type locality: Ecuador, Cuenca/Gral.Plaza; NMNH; ♂].

**Distribution.** Ecuador.

***trifida*** (*Atopsaura*) Denning, 1948b:113 [Type locality: Puerto Rico, Luquillo; CAS; ♂; as *trifidus*]. —Ross and King, 1952:198 [♂]. —Flint, 1964a:13 [♂; ♀; larva; pupa]. —Flint, 1968b:79 [checklist]. —Schmid 1989:144 [Costa Rica, erroneous distribution; lapsus for Puerto Rico]. —Masteller and Flint, 1993:65 [biology]. —Flint and Masteller, 1993:140 [biology]. —Botosaneanu, 2002:80 [checklist].

**Distribution.** Puerto Rico.

***tripunctata*** (*Atopsyche*) Banks, 1905:17 [Type locality: United States, Arizona, Huachuca Mountains; MCZ; ♂]. —Ross, 1947: 128 [♂]. —Ross and King, 1952:190 [♂; distribution]. —Flint, 1967d:163 [distribution]. —Bueno-Soria and Flint, 1978:192 [distribution]. —Schmid, 1989:143 [♂; ♀]. —Blinn and Ruiter, 2006:332 [ecology]. —Blinn and Ruiter, 2009a:303 [biology]. —Blinn and Ruiter, 2009b:185 [phenology, distribution]. —Ruiter and Blinn, 2009:4 [♀].

**Distribution.** Mexico, U.S.A.

***ulmeri*** (*Atopsyche*) Ross, 1953:288 [Type locality: Peru, Dpto. Cuzco, Pcia. Paucartambo, Cosnipata Valley; INHS; ♂]. —Flint, 1996b:379 [distribution]. —Angrisano, 1999:27 [checklist].

**Distribution.** Bolivia, Peru.

***unicolorata*** (unplaced) Schmid, 1989:131 [Type locality: Bolivia, Prov. La Paz, Sorata; NMNH; ♂]. —Angrisano, 1999:27 [checklist].

**Distribution.** Bolivia.

***uruguayensis*** (*Atopsaura*) Angrisano, 1997b:56 [Type locality: Uruguay, Cerro Largo, Sierra de Acegué; FHCU; ♂]. —Angrisano, 1999:27 [checklist].

**Distribution.** Uruguay.

***urumarca*** (*Atopsyche*) Schmid, 1989:131 [Type locality: Brazil, Edo. M.♂. [Minas Gerais], Serra do Cipó, Rio Capivara; MZUSP; ♂]. —Angrisano, 1999:27 [checklist]. —Paprocki et al., 2004:7 [checklist]. —Santos and Holzenthal, 2012:75 [♂; variation, distribution]. —Paprocki and França, 2014:22 [checklist]. —Gomes and Calor, 2016:63 [redescription; distribution].

**Distribution.** Brazil.

***usingeri*** (*Atopsaura*) Denning and Sykora, 1968:172 [Type locality: Brazil, Edo. Rio de Janeiro, Teresopolis, Organ Mountains; CAS; ♂]. —Angrisano, 1999:27 [checklist]. —Paprocki et al., 2004:7 [checklist]. —Dumas et al., 2009:365 [distribution]. —Calor, 2011:320 [checklist]. —Paprocki and França, 2014:21 [checklist]. —Gomes and Calor, 2016:64 [redescription; distribution].

**Distribution.** Brazil.

***vatucra*** (*Atopsyche*) Ross, 1953:290 [Type locality: Peru, Dpto. Cuzco, Pcia. Paucartambo, Cosnipata Valley; INHS; ♂]. —Flint, 1996b:379 [distribution].

**Distribution.** Peru.

***vinai*** (*Atopsaura*) Sykora and Botosaneanu, in Botosaneanu and Sykora, 1973:382 [Type locality: Cuba, Prov. Oriente, Cabezadas de Río Indio, massif de Gran Piedra; ZMUA; ♂; larva]. —Botosaneanu, 1979:43 [distribution]. —Botosaneanu, 1994b:453 [larva]. —Flint, 1996c:16 [checklist]. —Botosaneanu, 2002:80 [checklist]. —López del Castillo et al., 2004:229 [distribution]. —Naranjo López and González Lazo, 2005:149 [checklist]. —González Lazo et al., 2005:260 [distribution]. —López del Castillo et al., 2007:171 [distribution; seasonal abundance].

**Distribution.** Cuba.

***viracocha*** (*Atopsyche*) Schmid, 1989:131 [Type locality: Venezuela, Edo. Lara, Yacambu National Park, 13 km SE Sanare; NMNH; ♂]. —Holzenthal and Cressa, 2002:136 [checklist].

**Distribution.** Venezuela.

***weibezahni*** (*Atopsyche*) Flint, 1974a:3 [Type locality: Venezuela, Edo. Merida, Rio Santo Domingo, outlet to Laguna Mucubaji; NMNH; ♂]. —Holzenthal and Cressa, 2002:136 [distribution].

**Distribution.** Venezuela.

***youngi*** (unplaced) Sykora, 1991:245 [Type locality: Ecuador, Prov. Azuay, Pass 8 km NE Girón; CMNH; ♂].

**Distribution.** Ecuador.

***yunguensis*** (*Atopsaura*) Rueda Martín, 2006a:52 [Type locality: Argentina, Salta, Santa Victoria, Lipeo, Río Los Naranjos, 22°25'47"S, 64°44'20"W, 1109 m; IML; ♂; larva; pupa]. —Reynaga and Rueda Martín, 2010:61 [trophic biology].

**Distribution.** Argentina, Bolivia.

***yupanqui*** (unplaced) Schmid, 1989:132 [Type locality: Venezuela, Edo. Merida, 8 km SE Apartaderos; NMNH; ♂]. —Holzenthal and Cressa, 2002:136 [distribution]. —Medellín et al., 2004:201 [biology; distribution].

**Distribution.** Colombia, Venezuela.

***zernyi*** (*Atopsaura*) Flint, 1974a:5 [Type locality: Brazil, Edo. Sao Paulo, Alto da Serra; NMW; ♂]. —Angrisano, 1999:27 [checklist]. —Paprocki et al., 2004:7 [checklist]. —Blahnik et al., 2004:4 [distribution]. —Dumas et al., 2009:365 [distribution]. —Dumas et al., 2010:7 [distribution]. —Calor, 2011:320 [checklist]. —Dumas and Nessimian, 2012:11 [distribution]. —Paprocki and França, 2014:21 [checklist]. —Gomes and Calor, 2016:64 [♂; redescription; distribution].

**Distribution.** Brazil.

#### Genus *Australobiosis* Schmid [3]

*Australobiosis* Schmid, 1958b:187 [Type species: *Australobiosis araucanica* Schmid, 1958b, original designation]. —Schmid 1989:54 [redescription].

This is a genus of only three species, all restricted to the Chilean Subregion. Their immature stages are undescribed. Adults are rarely taken, usually by net, near small streams and waterfalls in densely forested sites.

***araucanica*** Schmid, 1958b:188 [Type locality: Chile, Prov. Chiloé, Dalcahue; NMNH; ♂]. —Flint, 1974e:84 [checklist]. —Schmid 1989:116 [♂; ♀]. —Angrisano, 1999:27 [checklist].

**Distribution.** Chile.

***bidens*** Schmid, 1989:117 [Type locality: Chile, Prov. Ñuble, Recinto; NMNH; ♂]. —Angrisano, 1999:27 [checklist].

**Distribution.** Chile.

***gladiocincta*** Schmid, 1989:117 [Type locality: Argentina, Prov. Neuquén, 5 km NW Lago Lolog; NMNH; ♂]. —Angrisano, 1999:27 [checklist].

**Distribution.** Argentina.

#### Genus *Cailloma* Ross and King [4]

*Cailloma* Ross and King, 1951:507 [Type species: *Cailloma brunosa* Ross and King, 1951, original designation, a synonym of *lucidula* (Ulmer)]. —Knutson and Flint, 1971:315 [Empididae predators in pupal cocoons]. —Flint, 1974d:473 [revision of genus, larva; pupa]. —Schmid, 1989:45 [redescription].

Genus A Flint, 1963a:463 [based on larva]. —Flint, 1974d:474 [to synonymy].

This is one of the first described genera of Neotropical Hydrobiosidae and one with a wide distribution. It is the only hydrobiosid genus that is both widespread in the Chilean Subregion and northward along the Andes. It now contains four species.

The larvae and pupae of all four species have been described and can be differentiated (Flint 1974d, Molina Arzabe and Gibon 2009). The immature stages are found in fast to moderately flowing streams and glacial meltwaters, often exposed to the full sunlight and harsh environments of the high elevations of the Altiplano (3,800 m). The females of *Cailloma rubenmarini* are brachypterous and do not readily fly.

***lucidula*** (Ulmer), 1909b:73 [Type locality: [Argentina, Prov. Mendoza], Potrerillos; ZSZMH; ♂; in *Atopsyche*]. —Flint, 1974d:476 [synonyny, ♂; ♀; larva; pupa; distribution]. —Flint, 1974e:84 [checklist]. —Schmid, 1989:143 [♂; ♀]. —Sykora, 1991:250 [distribution]. —Mangeaud, 1996:154 [distribution]. —Angrisano, 1999:27 [checklist]. —Cohen, 2004:76 [distribution]. —Isa Miranda and Rueda Martín, 2014:199 [distribution].

—*brunosa* Ross and King, 1951:507 [Type locality: Peru, San Ignacio, Cailloma; FMNH; ♂]. —Flint, 1974d:477 [to synonymy].

—*angustipennis* Schmid, 1955a:122 [Type locality: Chile, Prov. Santiago, El Manzano; NMNH; ♂]. —Flint, 1974d:477 [to synonymy].

**Distribution.** Argentina, Bolivia, Chile, Ecuador, Peru.

***pumida*** Ross, 1956a:125 [Type locality: Chile, Coquimbo, 5 mi. west of La Junta; CAS; ♂]. —Flint, 1974d:477 [synonyny, ♂; ♀; larva; pupa; distribution]. —Flint, 1974e:84 [checklist]. —Angrisano, 1999:27 [checklist]. —Brand and Miserendino, 2011b:143 [biology]. —Brand et al., 2012:90 [biology]. —Brand and Miserendino, 2014:6 [community ecology].

—*erinaceus* Schmid, 1957:382 [Type locality: [Chile], Prov. O’Higgins, La Leonera; NMNH; ♂]. —Flint, 1974d:480 [to synonymy].

**Distribution.** Argentina, Chile.

***rotunda*** Flint, 1967a:46 [Type locality: Chile, Prov. Santiago, near Los Valdes; NMNH; ♂]. —Flint, 1974d:477 [♂; ♀; larva; pupa; distribution]. —Flint, 1974e:84 [checklist]. —Schmid, 1989:143 [♂]. —Angrisano, 1999:27 [checklist]. —Brand, 2009:224 [distribution].

**Distribution.** Argentina, Chile.

***rubemarini*** Molina Arzabe and Gibon, 2009:24 [Type locality: Bolivia, Kullu Kachi River (16°17'48"S, 68°27'17"W, few kilometers fro. Batalia, E Titicaca Lake; MNHN; ♂; ♀; larva; pupa; ecology, biology].

**Distribution.** Bolivia.

#### Genus *Clavichorema* Schmid [7]

*Clavichorema* Schmid, 1955a:119 [Type species: *Clavichorema trancasica* Schmid, 1955a, original designation]. —Schmid, 1989:60 [redescription].

The genus contains seven known species, all restricted to the Chilean Subregion. The immature stages of *Clavichorema trancasicum* were described by Bravo and Angrisano (2005). Adults are taken at light or by sweeping near small streams in forested areas.

***capillatum*** Schmid, 1955a:122 [Type locality: Chile, Prov. Ñuble, Las Vizcachas; NMNH; ♂; as *capillata*]. —Flint, 1974e:84 [checklist]. —Schmid, 1989:144 [♂]. —Angrisano, 1999:28 [checklist].

**Distribution.** Chile.

***chiloeanum*** Schmid, 1955a:121 [Type locality: Chile, Island of Chiloé, Aucar; NMNH; ♂; as *chiloeana*]. —Flint, 1974e:85 [checklist]. —Schmid, 1989:144 [♂]. —Angrisano, 1999:28 [checklist].

**Distribution.** Chile.

***complicatissimum*** Schmid, 1955a:122 [Type locality: Chile, Prov. Ñuble, Recinto; NMNH; ♂; as *complicatissima*]. —Flint, 1974e:85 [checklist]. —Schmid, 1989:144 [♂]. —Angrisano, 1999:28 [checklist].

**Distribution.** Chile.

***pescaderum*** Flint, 1983a:6 [Type locality: Chile, Prov. Osorno, Parque Nacional Puyehue, Río Pescadero; NMNH; ♂]. —Angrisano, 1999:28 [checklist].

**Distribution.** Chile.

***pillimpilli*** Schmid, 1957:380 [Type locality: [Chile], Prov. Arauco, Pichinahuel; NMNH; ♂]. —Flint, 1974e:85 [checklist]. —Schmid, 1989:144 [♂]. —Angrisano, 1999:28 [checklist].

**Distribution.** Chile.

***purgatorium*** Flint, 1969b:497 [Type locality: Chile, Prov. Ñuble, Cord. Chillan, El Purgatorio; NMNH; ♂; as *purgatoria*]. —Flint, 1974e:85 [checklist]. —Angrisano, 1999:28 [checklist].

**Distribution.** Chile.

***trancasicum*** Schmid, 1955a:121 [Type locality: Chile, Prov. Ñuble, Las Trancas; NMNH; ♂; as *trancasica*]. —Flint, 1974e:85 [checklist]. —Schmid, 1989:144 [♂; ♀]. —Angrisano, 1999:28 [checklist]. —Bravo and Angrisano, 2005:20 [larva; pupa; distribution].

**Distribution.** Argentina, Chile.

#### Genus *Heterochorema* Schmid [1]

*Heterochorema* Schmid, 1989:55 [Type species: *Neochorema paradoxicum* Flint, 1983a, original designation].

The genus contains a single species, known only from southern Chile. Its immature stages are unknown.

***paradoxicum*** (Flint), 1983a:7 [Type locality: Chile, Prov. Llanquihue, El Chingue, N Correntoso (S Volcán Calbuco); NMNH; ♂; in *Neochorema*]. —Schmid, 1989:143 [♂; ♀]. —Angrisano, 1999:28 [checklist].

**Distribution.** Chile.

#### Genus *Iguazu* Ross and King [2]

*Iguazu* Ross and King, 1951:505 [Type species: *Iguazu ulmeri* Ross and King, 1951, original designation]. —Schmid, 1989:80 [redescription].

This a small genus of only two species, both known from the Chilean Subregion. Angrisano (2001) described the immatures. Adults are infrequently taken at lights near small, fast-flowing streams in forested areas.

***flavofuscum*** Schmid, 1957:385 [Type locality: [Chile], Prov. Arauco, Pichinahuel; NMNH; ♂; *Igazu*, misspelling]. —Flint, 1974e:85 [checklist]. —Schmid 1989:144 [♂; ♀]. —Angrisano, 1999:28 [checklist]. —Angrisano, 2001:200 [larva; distribution].

**Distribution.** Chile.

***ulmeri*** Ross and King, 1951:505 [Type locality: Argentina, Prov. Misiones, Iguazú; IML; ♂; the type was assuredly mislabelled, no other specimen of this species or genus is known from NE Argentina]. —Angrisano, 1999:28 [checklist, probable erroneous locality]. —Cohen, 2004:74 [list of type material].

**Distribution.** Argentina [?].

#### Genus *Isochorema* Schmid [2]

*Isochorema* Schmid, 1989:89 [Type species: *Isochorema curvispinum* Schmid, 1989, original designation].

†*Isochorema* Wichard, 2013:34 [Type species: †*Isochorema secunda* Wichard, 2013, original designation; Baltic amber; junior homonym of *Isochorema* Schmid, 1989, replaced by *Electrochorema* Wichard, 2014:4].

The genus contains two species, both known from only a few specimens collected in Chile. Their immature stages are undescribed, and their biology is unknown.

***curvispinum*** Schmid, 1989:137 [Type locality: Chile, Prov. Ñuble, Alto Tregualemu, ca. 20 km SE Chovellen; NMNH; ♂]. —Angrisano, 1999:28 [checklist].

**Distribution.** Chile.

***flintorum*** Schmid, 1989:137 [Type locality: Chile, Prov. Valdivia, 36 km. W. La Union; NMNH; ♂; ♀]. —Angrisano, 1999:28 [checklist].

**Distribution.** Chile.

#### Genus *Metachorema* Schmid [2]

*Metachorema* Schmid, 1957:382 [Type species: *Metachorema gregarium* Schmid, 1957, original designation]. —Schmid, 1989:46 [redescription].

The genus still contains only two species restricted to the Chilean Subregion. The immature stages of *Australobiosis griseum* were described by Bravo and Angrisano (2003). Adults are commonly taken at light near small, fast-flowing streams in forested areas.

***gregarium*** Schmid, 1957:383 [Type locality: [Chile], Prov. Valdivia, Rio Chaquigua; NMNH; ♂]. —Flint, 1974e:85 [checklist]. —Schmid 1989:143 [♂]. —Angrisano, 1999:28 [checklist].

**Distribution.** Chile.

***griseum*** Schmid, 1957:383 [Type locality: [Chile], Prov. Valdivia, Neltume; NMNH; ♂]. —Flint, 1974e:85 [checklist]. —Schmid 1989:143 [♂; ♀]. —Angrisano, 1999:28 [checklist]. —Bravo and Angrisano, 2003:205 [larva; pupa; distribution]. —Brand and Miserendino, 2011a:35 [biology; habitat]. —Brand et al., 2012:90 [biology]. —Brand and Miserendino, 2014:6 [community ecology].

**Distribution.** Argentina, Chile.

#### Genus *Microchorema* Schmid [4]

*Microchorema* Schmid, 1955a:128 [Type species: *Microchorema recintoi* Schmid, 1955a, original designation]. —Schmid, 1989:63 [redescription].

This is a genus of four species, all wholly restricted to Chile. The immature stages of *Microchorema extensum* were described by Bravo and Angrisano (2004a) as were those of two unknown genera, Genus X and Genus Y. Adults are very rarely taken by sweeping near small, forested streams.

***extensum*** Schmid, 1964:308 [Type locality: Chile, Prov. Curico, El Coigo; NMNH; ♂]. —Flint, 1974e:85 [checklist]. —Schmid 1989:145 [♂; ♀]. —Angrisano, 1999:28 [checklist]. —Bravo and Angrisano, 2004a:98 [larva; pupa].

**Distribution.** Chile.

***larica*** Flint, 1969b:499 [Type locality: Chile, Prov. Cautin, 30 km. NE of Villarrica; NMNH; ♂]. —Flint, 1974e:85 [checklist]. —Schmid, 1989:145 [♂]. —Angrisano, 1999:28 [checklist].

**Distribution.** Chile.

***penai*** Schmid, 1958b:189 [Type locality: Chile, Prov. Chiloé, Dalcahue; NMNH; ♂]. —Flint, 1974e:85 [checklist]. —Schmid 1989:145 [♂]. —Angrisano, 1999:28 [checklist].

**Distribution.** Chile.

***recintoi*** Schmid, 1955a:128 [Type locality: Chile, Prov. Ñuble, Recinto; NMNH; ♂]. —Flint, 1974e:85 [checklist]. —Schmid 1989:145 [♂]. —Angrisano, 1999:28 [checklist].

**Distribution.** Chile.

#### Genus *Neoatopsyche* Schmid [5]

*Neoatopsyche* Schmid, 1955a:126 [Type species: *Neoatopsyche chilensis* Schmid, 1955a, original designation]. —Knutson and Flint, 1971:315 [Empididae predators in pupal cocoons]. —Schmid, 1989:57 [redescription]. —Angrisano, 1998:121 [immature stages].

The genus contains five known species, all restricted to the Chilean Subregion. The immature stages of all five species were recently described and diagnosed (Angrisano, 1998). Adults are commonly taken at light in large numbers near small streams to large rivers in either forested areas or open countryside.

***brevispina*** Schmid, 1957:381 [Type locality: [Chile], Prov. Arauco, Caramavida; NMNH; ♂]. —Denning, 1965b:697 [♂]. —Flint, 1974e:85 [checklist]. —Schmid, 1989:143 [♂; ♀]. —Angisano, 1998:122 [larva; distribution]. —Angrisano, 1999:28 [checklist]. —Brand and Miserendino, 2011a:35 [biology; habitat]. —Brand and Miserendino, 2011b:143 [biology]. —Brand et al., 2012:90 [biology]. —Brand and Miserendino, 2014:6 [community ecology].

**Distribution.** Argentina, Chile.

***chilensis*** Schmid, 1955a:126 [Type locality: Chile, Prov. Ñuble, Recinto; NMNH; ♂]. —Flint, 1974e:85 [checklist]. —Schmid 1989:143 [♂]. —Angrisano, 1999:28 [checklist]. —Angisano, 1998:123 [larva; distribution]. —Angrisano, 1999:28 [checklist]. —Miserendino and Brand, 2007:312 [biology].

**Distribution.** Argentina, Chile.

***obliqua*** Flint, 1969b:499 [Type locality: Chile, Prov. Aconcagua, SW Catapilco; NMNH; ♂]. —Flint, 1974e:85 [checklist]. —Knutson and Flint, 1979:32 [Empididae predators in pupal cocoons]. —Angisano, 1998:122 [larva; distribution]. —Angrisano, 1999:28 [checklist].

**Distribution.** Argentina, Chile.

***spinosella*** Schmid, 1955a:127 [Type locality: Chile, Prov. Santiago, El Manzano; NMNH; ♂]. —Flint, 1974e:85 [checklist]. —Schmid 1989:143 [♂]. —Angisano, 1998:122 [larva; distribtion]. —Angrisano, 1999:28 [checklist].

**Distribution.** Argentina, Chile.

***unispina*** Flint, 1967a:47 [Type locality: Argentina, Prov. Río Negro, El Bolson; NMNH; ♂]. —Flint, 1974e:85 [checklist]. —Angisano, 1998:122 [larva]. —Angrisano, 1999:28 [checklist]. —Brand and Miserendino, 2011a:35 [biology; habitat]. —Brand and Miserendino, 2011b:143 [biology]. —Brand et al., 2012:90 [biology]. —Brand and Miserendino, 2014:6 [community ecology].

**Distribution.** Argentina, Chile.

#### Genus *Neochorema* Schmid [4]

*Neochorema* Schmid, 1957:384 [Type species: *Neochorema dictynnum* Schmid, 1957, original designation]. —Schmid, 1989:62 [redescription].

This genus of four species is restricted to Argentina and Chile. The immature stages of *Neochorema sinuatum*, as well as those of two unknown genera, Genus X and Genus Y, were described by Bravo and Angrisano (2004a). Adults are very rarely taken at light near rivers and streams.

***dictynnum*** Schmid, 1957:385 [Type locality: [Chile], Prov. Coquimbo, Rivadavia; NMNH; ♂]. —Flint, 1974e:85 [checklist]. —Schmid 1989:145 [♂]. —Angrisano, 1999:29 [checklist].

**Distribution.** Chile.

***jaula*** Flint, 1969b:501 [Type locality: Chile, Prov. Curico, Estero La Jaula, Los Quenes; NMNH; ♂]. —Flint, 1974e:85 [checklist]. —Schmid, 1989:145 [♂]. —Angrisano, 1999:29 [checklist].

**Distribution.** Chile.

***lobiferum*** Flint, 1969b:500 [Type locality: Chile, Prov. Cautin, 30 km NE Villarrica; NMNH; ♂]. —Flint, 1974e:85 [checklist]. —Schmid, 1989:145 [♂]. —Angrisano, 1999:29 [checklist].

**Distribution.** Chile.

***sinuatum*** Schmid, 1964:307 [Type locality: Chile, Prov. Arauco, Pichinahuel; NMNH; ♂]. —Flint, 1974e:85 [checklist]. —Schmid 1989:145 [♂; ♀]. —Angrisano, 1999:29 [checklist]. —Bravo and Angrisano, 2004a:101 [larva; pupa; distribution].

**Distribution.** Chile.

#### Genus *Neopsilochorema* Schmid [1]

*Neopsilochorema* Schmid, 1955a:127 [Type species: *Neopsilochorema tricarinata* Schmid, 1955a, original designation]. —Schmid, 1989:87 [redescription]. —Angrisano,1997a:15 [immature stages].

The genus contains only a single species, restricted to the Chilean Subregion. Angrisano (1997a) described the immature stages. Adults are taken at light or by sweeping near small streams in forested areas.

***tricarinatum*** Schmid, 1955a:128 [Type locality: Chile, Island of Chiloé, Toi-Goi; NMNH; ♂; as *tricarinata*]. —Flint, 1974e:85 [checklist]. —Schmid 1989:147 [♂; ♀]. —Angrisano, 1997a:15 [larva; pupa; distribution]. —Angrisano, 1999:29 [checklist]. —Miserendino and Brand, 2007:312 [biology]. —Brand and Miserendino, 2011a:35 [biology; habitat]. —Brand and Miserendino, 2011b:143 [biology]. —Brand et al., 2012:90 [biology]. —Brand and Miserendino, 2014:6 [community ecology].

**Distribution.** Argentina, Chile.

#### Genus *Nolganema* Navás [1]

*Nolganema* Navás, 1934a:169 [Type species: *Nolganema chilense* Navás, 1934a, original designation]. —Flint, 1974e:83 [transferred to Hydrobiosinae]. —Schmid, 1989:64 [relationship].

This monotypic genus is only known from its original description which included a figure of its wing venation. It appears to be a member of the *Amphichorema* group of genera (Schmid 1989).

***chilense*** Navás, 1934a:169 [Type locality: Chile, Angol; type depository unknown; ♂]. —Flint, 1974e:85 [checklist]. —Angrisano, 1999:29 [checklist].

**Distribution.** Chile.

#### Genus *Parachorema* Schmid [1]

*Parachorema* Schmid, 1957:383 [Type species: *Parachorema bifidum* Schmid, 1957, original designation]. —Schmid, 1989:49 [redescription].

The immature stages of this monotypic genus were described by Bravo and Angrisano (2004b). Adults are commonly taken at light near larger rivers, often fully exposed to the sun.

***bifidum*** Schmid, 1957:384 [Type locality: [Chile], Prov. Ñuble, Atacalco; NMNH; ♂]. —Flint, 1974e:85 [checklist]. —Schmid 1989:143 [♂; ♀]. —Angrisano, 1999:29 [checklist]. —Bravo and Angrisano, 2004b:23 [larva; pupa].

**Distribution.** Argentina, Chile.

#### Genus *Pomphochorema* Flint [1]

*Pomphochorema* Flint, 1969b:503 [Type species: *Pomphochorema chilensis* Flint, 1969b, original designation]. —Schmid, 1989:61 [redescription].

The single species in the genus is known from Chile. Its immature stages are known (Bravo and Angrisano 2004c). Adults are infrequently taken at light near small, fast-flowing streams in forested areas.

***chilensis*** Flint, 1969b:504 [Type locality: Chile, Prov. Arauco, Caramavida; NMNH; ♂]. —Flint, 1974e:86 [checklist]. —Schmid, 1989:145 [♂; ♀]. —Angrisano, 1999:29 [checklist]. —Bravo and Angrisano, 2004c:38 [larva; pupa].

**Distribution.** Chile.

#### Genus *Pseudoradema* Schmid [1]

*Pseudoradema* Schmid, 1955a:124 [Type species: *Pseudoradema spinosissima* Schmid, 1955a, original designation]. —Schmid 1989:44 [redescription].

The immatures of this monotypic genus were described by Bravo and Angrisano (2005).

***spinosissimum*** Schmid, 1955a:125 [Type locality: Chile, Prov. Malleco, Curacautin; NMNH; ♂; as *spinosissima*]. —Flint, 1974e:86 [checklist]. —Schmid 1989:143 [♂; ♀]. —Angrisano, 1999:29 [checklist]. —Bravo and Angrisano, 2005:23 [larva; pupa].

**Distribution.** Chile.

#### Genus *Rheochorema* Schmid [4]

*Rheochorema* Schmid, 1955a:118 [Type species: *Rheochorema tenuispina* Schmid, 1955a, original designation]. —Schmid, 1989:79 [redescription]. —Angrisano, 2001:196 [larva].

The genus contains four described species, all restricted to the Chilean Subregion. The larva of all species are described (Flint1967a, Angrisano 2001). Adults are commonly taken at light in large numbers near small streams to large rivers in either forested areas or open countryside.

***lobuliferum*** Flint, 1967a:47 [Type locality: Chile, Prov. Magallanes, Río Penitente; NMNH; ♂; as *lobulifera*, larva]. —Flint, 1974e:86 [checklist]. —Schmid, 1989:146 [♂]. —Angrisano, 1999:29 [checklist]. —Angrisano, 2001:196 [larva; distribution]. —Brand et al., 2012:90 [biology]. —Brand and Miserendino, 2014:6 [community ecology].

**Distribution.** Chile.

***magellanicum*** Flint, 1974a:6 [Type locality: Argentina, Isla de los Estados, Puerto Cook; NMNH; ♂; as *magellanica*]. —Flint, 1974e:86 [checklist]. —Schmid, 1989:146 [♂]. —Angrisano, 1999:29 [checklist]. —Angrisano, 2001:199 [larva; distribution].

**Distribution.** Argentina, Chile.

***robustum*** Schmid, 1955a:119 [Type locality: Chile, Prov. Malleco, Rio Blanco; NMNH; ♂; as *robusta*]. —Flint, 1974e:86 [checklist]. —Schmid 1989:146 [♂; ♀]. —Angrisano, 1999:29 [checklist]. —Angrisano, 2001:197 [larva; distribution]. —Cohen, 2004:76 [distribution]. —Brand and Miserendino, 2011b:143 [biology; distribution]. —Brand et al., 2012:90 [biology]. —Brand and Miserendino, 2014:6 [community ecology].

**Distribution.** Argentina, Chile.

***tenuispinum*** Schmid, 1955a:118 [Type locality: Chile, Prov. Ñuble, Recinto; NMNH; ♂; as *tenuispina*]. —Flint, 1974e:86 [checklist]. —Schmid 1989:146 [♂]. —Angrisano, 1999:29 [checklist]. —Angrisano, 2001:197 [larva; distribution]. —Brand and Miserendino, 2011b:143 [biology; distribution]. —Brand et al., 2012:90 [biology]. —Brand and Miserendino, 2014:6 [community ecology].

**Distribution.** Argentina, Chile.

#### Genus *Schajovskoya* Flint [1]

*Schajovskoya* Flint, 1979:641 [Type species: *Schajovskoya neuquenensis* Flint, 1979, original designation]. —Schmid, 1989:50 [redescription].

The genus still contains only a single species, known from Patagonian Argentina and Chile. Its immature stages were described at the time of the description of the genus and figured by Angrisano (2000). Adults are infrequently taken at light or by sweeping near small, fast-flowing streams in forested areas.

***neuquenensis*** Flint, 1979:641 [Type locality: Argentina, Prov. Neuquen, Arroyo Culebra, 20 km S San Martin de los Andes; NMNH; ♂; larva]. —Schmid, 1989:143 [♂; ♀]. —Angrisano, 1999:29 [checklist]. —Angrisano, 2000:291 [larva; pupa; compared to *Stenochorema*, distribution].

**Distribution.** Argentina, Chile.

#### Genus *Stenochorema* Schmid [1]

*Stenochorema* Schmid, 1955a:123 [Type species: *Stenochorema crassicosta* Schmid, 1955a, original designation]. —Schmid 1989:48 [redescription].

The immature stages of this monotypic genus were described by Angrisano (2000). It has been taken near small forested streams in southern Chile.

***crassicostum*** Schmid, 1955a:124 [Type locality: Chile, Island of Chiloé, Aucar; NMNH; ♂; as *crassicosta*]. —Flint, 1974e:86 [checklist]. —Schmid 1989:143 [♂; ♀]. —Angrisano, 1999:29 [checklist]. —Angrisano, 2000:290 [larva; pupa; compared to *Schajovskoya*, distribution].

**Distribution.** Chile.

### Family Hydropsychidae

The family Hydropsychidae is found in flowing water habitats around the world. Larvae spin silken nets to filter the water of food material and are common and often abundant components of the bottom fauna. Five subfamilies are recognized: Arctopsychinae, Diplectroninae, Hydropsychinae, Smicrideinae, and Macronematinae. The monophyly and phylogenetic relationships among these subfamilies has been the subject of recent studies (Geraci et al. 2005, Schefter 1996, 2005). Only Arctopsychinae does not occur in the region covered by this catalog and Diplectroninae and Hydropsychinae are represented by only a few genera and species, although the latter contains Neotropical endemic genera. The greatest diversity of species and genera occur in the Macronematinae and Smicrideinae.

Diplectroninae are cosmopolitan, but not very rich in genera or species. Only a few species of *Diplectrona* occur in the mountains of Mexico and Central America. Similarly, the Neotropical members of Hydropsychinae are found only in the northern regions, from Mexico into Panama and the Greater Antilles. Two genera, *Cheumatopsyche* and *Hydropsyche* are North American taxa that extend into northern Mexico, while *Calosopsyche*, *Plectropsyche*, and *Streptospyche* are endemic to southern Mexico, Central America, or the Greater Antilles. The subfamily Smicrideinae in the New World only contains *Smicridea* with its two subgenera, *Smicridea* and *Rhyacophylax*, but it is the most diverse Neotropical genus of Hydropsychidae, with over 200 described species. Macronematinae is richly represented in the Neotropics in genera and species. Of the genera, *Blepharopus*, *Centromacronema*, *Macronema*, *Plectromacronema*, *Pseudomacronema*, and *Synoestropsis* are allendemic to the region, while *Leptonema* and *Macrostemum* also have representatives in the Old World.

#### Genus *Blepharopus* Kolenati [1]

*Blepharopus* Kolenati, 1859:242 [Type species: *Blepharopus diaphanus* Kolenati, 1859, by monotypy].

The genus contains only a single species endemic to tropical South America. The robust, hirsute adults frequently come to lights near medium to large sized rivers. Flint and Wallace (1980) described the immature stages. Larvae constructed nets attached to stones in rapidly flowing water. Morphology of larval legs and mouthparts and net structure suggested to these authors that larvae fed on fine particulate organic matter suspended in the water.

***diaphanus*** Kolenati, 1859:242 [Type locality: [Brazil] Brazilia [specific locality unknown, but the collector Beske lived and worked in the vicinity of Nova Friburgo, Rio de Janeiro state; NMW; ♂]. —Ulmer, 1907c:42 [♂; wings; head]. —Flint and Wallace, 1980:179 [distribution; larva; pupa]. —Flint, 1992d:69 [distribution]. —Marinoni and Almeida, 2000:286 [distribution; biology]. —Cohen, 2004:74 [distribution]. —Blahnik, et al., 2004:4 [distribution]. —Paprocki et al., 2004:7 [checklist]. —Dumas et al., 2009:357 [distribution]. —Calor, 2011:320 [checklist]. —Nogueira and Cabette, 2011:351 [distribution]. —Barcelos-Silva et al., 2012:1277 [distribution]. —Souza et al., 2013a:4 [distribution]. —Costa et al., 2014:218 [distribution]. —Paprocki and França, 2014:22 [checklist].

—*reticulatus* Ulmer, 1905a:52 [Type locality: [Brazil], Santa Catharina [sic]; PAN; ♂]. —Flint, 1978:395, 404 [♂; wings; to synonymy].

**Distribution.** Argentina, Brazil, Venezuela.

#### Genus *Calosopsyche* Ross and Unzicker [13 + †1]

*Calosopsyche* Ross and Unzicker, 1977:309 [Type species: *Hydropsyche calosa* Banks, 1938, original designation]. —Flint and Bueno-Soria, 1987:29 [revision]. —Schefter, 2005:149 [phylogeny]. —Wichard, 2007a:20 [diagnosis]. —Oláh and Johanson, 2008:55 [diagnosis]. —Geraci et al., 2010:925 [redefinition].

The genus contains 14 species, including one fossil species, placed in two species groups: the *calosa* group of 9 species is endemic to the Greater Antillean islands, while the *continentalis* group of 5 species is found in southern Central America. The *calosa* group is distinguished from the *continentalis* group by the shape of the apical segment of the inferior appendage, which is not bifid at the tip or bearing a lobe on one side (Wichard 2007a). After the proposal of a close relationship between *Calosopsyche* and *Streptopsyche* (Geraci et al. 2005), Oláh and Johanson (2008) proposed their synonymy based on the similarity of “primary generic characters” in both genera as well on the homoplastic origins of the “tertiary generic characters.” Additionally, these authors proposed a new species group within *Calosopsyche*, the *antilles* group, based on their diagnostic endothecal processes, to classify the species previously described as *Streptopsyche*. Geraci et al. (2010), in a comprehensive revision, resurrected *Streptopsyche* and we here follow this classification.

The larva and pupa of *Calosopsyche continentalis* were described by Flint and Bueno-Soria (1987) and the larva of *Calosopsyche cubana* by Botosaneanu (1994b). They are found in small streams to larger rivers, usually at higher elevations in forested sites. Nothing has yet been reported on their type of retreat or food preferences; it is quite probable that they will be found to be similar to the other genera in the subfamily.

***ardisia*** Flint and Bueno-Soria, 1987:36 [Type locality: Costa Rica, (Pcia. Alajuela), Turricares (sic., Turrucares); MCZ; ♂; ♀]. —Holzenthal, 1988c:65 [distribution]. —Oláh and Johanson, 2008:53 [catalog]. —Armitage et al., 2015a:3 [distribution]. —Armitage et al., 2015b:5 [checklist]. —Armitage and Cornejo, 2015:193 [checklist].

**Distribution.** Costa Rica, Panama.

***batesi*** (Flint), 1962a:25 [Type locality: Haiti, LaVisite and vicinity, La Selle Range; MCZ; ♀; in *Hydropsyche*]. —Flint, 1968b:81 [checklist]. —Ross and Unzicker, 1977:309 [to *Calosopsyche*]. —Flint and Bueno-Soria, 1987:33 [checklist]. —Flint and Pérez-Gelabert, 1999:37 [checklist]. —Botosaneanu, 2002:92 [checklist; as *Hydropsyche*]. —Flint and Sykora, 2004:18 [distribution]. —Oláh and Johanson, 2008:55 [catalog; taxonomic remarks]. —Pérez-Gelabert, 2008:299 [checklist].

**Distribution.** Haiti.

***bicuspis*** Flint and Bueno-Soria, 1987:36 [Type locality: Costa Rica, Pcia. Alajuela, Rio La Paz, route 9, 7.6 km N Vara Blanca (10.208°N, 84.166°W); NMNH; ♂; ♀]. —Holzenthal, 1988c:65 [distribution]. —Oláh and Johanson, 2008:51 [catalog]. —Armitage et al., 2015a:3 [distribution]. —Armitage et al., 2015b:5 [checklist]. —Armitage and Cornejo, 2015:193 [checklist].

**Distribution.** Costa Rica, Panama.

***bohio*** (Botosaneanu), 1991a:132 [Type locality: Haïti, Département de l’Ouest, Ville Bonheur (Ville Saut d’Eau): Le Saut d’Eau; ZMUA; ♀; in *Hydropsyche*]. —Flint et al., 1999a:75 [to *Calosopsyche*]. —Flint and Pérez-Gelabert, 1999:38 [checklist]. —Botosaneanu, 2002:92 [checklist; as *Hydropsyche*]. —Flint and Sykora, 2004:18 [distribution]. —Oláh and Johanson, 2008:55 [catalog, taxonomic remarks]. —Pérez-Gelabert, 2008:299 [checklist].

**Distribution.** Haiti.

***calosa*** (Banks), 1938:300 [Type locality: Cuba (probably Oriente) [recent advances in knowledge of Cuban Trichoptera leads to the realization that the “Cuba Ch. Wright” material actually must have come from Pinar del Rio Province, not Oriente]; MCZ; ♂; in *Hydropsyche*]. —Flint, 1962a:23 [♂; ♀]. —Flint, 1967c:12 [lectotype]. —Flint, 1968b:81 [checklist]. —Ross and Unzicker, 1977:309 [to *Calosopsyche*]. —Botosaneanu, 1979:46 [distribution]. —Flint, 1996c:16 [checklist]. —Botosaneanu, 2002:92 [checklist; as *Hydropsyche*]. —Naranjo López and González Lazo, 2005:150 [checklist; as *Hydropsyche*]. —Oláh and Johanson, 2008:51 [catalog].

**Distribution.** Cuba.

***carinifera*** (Flint), 1962a:27 [Type locality: Dominican Republic, foothills Cordillera Central, South of Santiago; MCZ; ♀; in *Hydropsyche*]. —Flint, 1968b:81 [checklist]. —Ross and Unzicker, 1977:309 [to *Calosopsyche*]. —Flint and Bueno-Soria, 1987:34 [♂]. —Botosaneanu, 1996:17 [distribution; taxonomic remarks; as *Hydropsyche*]. —Botosaneanu, 2002:93 [checklist; as *Hydropsyche*]. —Flint and Pérez-Gelabert, 1999:38 [checklist]. —Flint and Sykora, 2004:18 [distribution]. —Oláh and Johanson, 2008:51 [catalog]. —Pérez-Gelabert, 2008:299 [checklist].

**Distribution.** Dominican Republic.

***continentalis*** Flint and Bueno-Soria, 1987:34 [Type locality: Panama, Pcia. Cocle, El Valle; NMNH; ♂; ♀; larva; pupa]. —Holzenthal, 1988c:65 [distribution]. —Aguila, 1992:541 [distribution]. —Oláh and Johanson, 2008:53 [catalog; ♂]. —Armitage et al., 2015b:5 [checklist]. —Armitage and Cornejo, 2015:193 [checklist].

—Genus B, undescribed species A McElravy et al., 1981:153. —Flint and Bueno-Soria, 1987:34 [to synonymy].

—Genus A, undescribed species A McElravy et al., 1982:307. —Flint and Bueno-Soria, 1987:34 [to synonymy].

**Distribution.** Costa Rica, Panama.

***cubana*** (Flint), 1962a:24 [Type locality: Cuba, Sierra Maestra near Rio Yao; NMNH; ♀; in *Hydropsyche*]. —Flint, 1968b:81 [checklist]. —Botosaneanu, 1977:280 [♂]. —Ross and Unzicker, 1977:309 [to *Calosopsyche*]. —Botosaneanu, 1979:46 [distribution]. —Botosaneanu, 1994b:462 [larva; in *Hydropsyche*].—Flint, 1996c:16 [checklist]. —Botosaneanu, 2002:93 [checklist; as *Hydropsyche*]. —López del Castillo et al., 2004:229 [distribution]. —González Lazo et al., 2005:260 [distribution; as *Hydropsyche*]. —Naranjo López and González Lazo, 2005:150 [checklist; as *Hydropsyche*]. —López del Castillo et al., 2007:171 [distribution; seasonal abundance]. —Oláh and Johanson, 2008:52 [catalog].

**Distribution.** Cuba.

***darlingtoni*** (Flint), 1962a:23 [Type locality: Cuba, Hanabanilla Falls, Trinidad Mountains; MCZ; ♀; in *Hydropsyche*]. —Flint, 1968b:81 [checklist]. —Ross and Unzicker, 1977:309 [to *Calosopsyche*]. —Botosaneanu, 1979:47 [distribution]. —Flint, 1996c:16 [checklist]. —Botosaneanu, 2002:93 [checklist; as *Hydropsyche*]. —Naranjo López and González Lazo, 2005:150 [checklist; as *Hydropsyche*]. —Oláh and Johanson, 2008:56 [catalog; taxonomic remarks].

**Distribution.** Cuba.

***dearmasi*** (Botosaneanu), 1980:105 [Type locality: [Cuba], Monte Iberia, Nibujon, Baracoa, Prov. Oriente; ZMUA; ♂; in *Hydropsyche*]. —Botosaneanu, 1979:47 [*nomen nudum* (name included in checklist); distribution]. —Flint and Bueno-Soria, 1987:33 [to *Calosopsyche*]. —Flint, 1996c:16 [checklist]. —Botosaneanu, 2002:93 [checklist; as *Hydropsyche*]. —Naranjo López and González Lazo, 2005:150 [checklist; as *Hydropsyche*]. —Oláh and Johanson, 2008:52 [catalog].

**Distribution.** Cuba.

***domingensis*** (Banks), 1941:398 [Type locality: [Dominican Republic], Constanza to Jarabacoa; MCZ; ♂; ♀; in *Hydropsyche*]. —Flint, 1962a:24 [♀]. —Flint, 1967c:12 [lectotype]. —Flint, 1968b:81 [checklist]. —Ross and Unzicker, 1977:308 [to *Plectropsyche*]. —Botosaneanu, 1996:17 [♂; in *Hydropsyche*]. —Flint et al., 1999a:75 [to *Calosopsyche*]. —Flint and Pérez-Gelabert, 1999:38 [checklist]. —Botosaneanu, 2002:93 [checklist; as *Hydropsyche*]. —Flint and Sykora, 2004:19 [distribution; taxonomic remarks]. —Oláh and Johanson, 2008:52 [catalog; taxonomic remarks]. —Pérez-Gelabert, 2008:299 [checklist].

**Distribution.** Dominican Republic.

***elachista*** Flint and Bueno-Soria, 1987:36 [Type locality: Panama, [Pcia. Panama], Cerro Campana; NMNH; ♂; ♀]. —Holzenthal, 1988c:65 [distribution]. —Aguila, 1992:541 [distribution]. —Oláh and Johanson, 2008:53 [catalog; taxonomic remarks]. —Armitage et al., 2015b:5 [checklist]. —Armitage and Cornejo, 2015:193 [checklist].

—Genus A, undescribed species A McElravy et al., 1981:153. —Flint and Bueno-Soria, 1987:36 [to synonymy].

—Genus B, undescribed species A McElravy et al., 1982:307, 310. —Flint and Bueno-Soria, 1987:36 [to synonymy].

**Distribution.** Costa Rica, Panama.

† ***palaeoelegans*** Wichard, 2007a:20 [Type locality: Dominican Republic; SMNS; ♂; in amber].

**Distribution.** Dominican Republic.

***sandrae*** (Flint), 1967b:15 [Type locality: Costa Rica, Golfito; NMNH; ♂; in *Plectropsyche*]. —Flint and Bueno-Soria, 1987:33 [to *Calosopsyche*]. —Holzenthal, 1988c:65 [distribution]. —Oláh and Johanson, 2008:53 [catalog; taxonomic remarks].

**Distribution.** Costa Rica.

#### Genus *Centromacronema* Ulmer [17]

*Centromacronema* Ulmer, 1905b:86 [Type species: *Macronema auripenne* Rambur, 1842, subsequent designation of Fischer, 1963].

Adults of the species in this genus are relatively large, conspicuous, and often encountered on streamside vegetation during the day. Males often display by waving their long antennae or through rapid, jerking, crawling movements on large leaves dappled with sunlight. Because of this, they were easily and frequently collected and described by the early South American naturalists. While several new species have been described recently, the identity of many of the older names is not clear because their types are poorly known and inadequately described. There seems to be a great deal of variation in color within a species, even at one site, and the differences in the genitalia between putative species are very small. There is a great need for a careful revision of all the species, including a reexamination of types, including those of the synonyms. The immature stages are known, but not yet described. Bueno-Soria (in litt.) indicated that the larvae described by Roback (1966) as Hydropsychidae sp. 4 agrees with a species of *Centromacronema* he reared from Mexico.

***apicale*** (Walker), 185b2:72 [Type locality: Venezuela; BMNH; ♂]. —Betten and Mosely, 1940:205 [♂; redescription of type; venation]. —Holzenthal, 1988c:65 [distribution]. —Flint, 1991:80 [distribution; wings]. —Flint, 1996b:410 [distribution]. —Muñoz-Quesada, 2000:275 [checklist].

**Distribution.** Colombia, Costa Rica, Peru, Venezuela.

***auripenne*** (Rambur), 1842:507 [Type locality: Brésil (Brazil); IRSNB; ♂; in *Macronema*]. —Banks, 1901:371 [distribution]. —Ulmer, 1905b:87 [to *Centromacronema*]. —Ulmer, 1907b:63 [type]. —Ulmer, 1907c:112 [♂; synonymy; redescription]. —Betten and Mosely, 1940:209 [redescription of ♂ of Walker’s *cupreum*; venation]. —Bueno-Soria and Flint, 1978:208 [distribution]. —Maes and Flint, 1988:4 [distribution]. —Holzenthal, 1988c:65 [distribution]. —Aguila, 1992:539 [distribution]. —Flint, 1996b:411 [distribution]. —Maes, 1999:1184 [checklist]. —Muñoz-Quesada, 2000:276 [checklist]. —Paprocki et al., 2004:7 [checklist]. —Bueno-Soria et al., 2005:75 [distribution]. —Chamorro-Lacayo et al., 2007:41 [checklist]. —Dumas et al., 2009:357 [distribution]. —Bueno-Soria and Barba-Álvarez, 2011:355 [checklist]. —Dumas and Nessimian, 2012:11 [checklist]. —Oláh and Johanson, 2012:217 [distribution; taxonomic remarks]. —Paprocki and França, 2014:22 [checklist]. —Armitage et al., 2015b:5 [checklist]. —Armitage and Cornejo, 2015:193 [checklist].

—*cupreum* (Walker), 1852:76 [Type locality: Brazil; BMNH; ♂; in *Macronema*]. —Ulmer, 1907c:112 [to synonymy].

—*niveistigma* (Walker), 1860:176 [Type locality: Brazil; BMNH; ♀; in *Leptocerus*]. —Ulmer, 1907c:112 [to synonymy].

—*abjurans* (Walker), 1860:177 [Type locality: Brazil; BMNH; ♂; in *Leptocerus*]. —Ulmer, 1907c:112 [to synonymy].

—*quadrifurca* (Walker), 1860:177 [Type locality: Brazil; BMNH; ♂; in *Leptocerus*]. —Ulmer, 1907c:112 [to synonymy].

—*extensum* Banks, 1913a:238 [Type locality: Panama, Lino; MCZ; ♂]. —Flint, 1967c:7 [to synonymy].

**Distribution.** Bolivia, Brazil, Colombia, Costa Rica, El Salvador, French Guiana, Guatemala, Guyana, Honduras, Mexico, Nicaragua, Panama, Peru, Venezuela.

***dentatum*** Navás, 1924a:207 [Type locality: Columbia, Cauca Vall.; collection Navás, now lost?; ♂]. —Muñoz-Quesada, 2000:276 [checklist].

**Distribution.** Colombia.

***excisum*** (Ulmer), 1905a:85 [Type locality: Ecuador, Santa Inéz; PAN; ♀]. —Ulmer, 1905b:87 [to *Centromacronema*]. —Flint, 1966a:6 [wings holotype]. —Flint, 1991:80 [distribution; wings]. —Muñoz-Quesada, 2000:276 [checklist]. —Oláh and Johanson, 2012:217 [distribution].

**Distribution.** Bolivia, Colombia, Ecuador, Peru, Venezuela.

***facile*** (Navás), 1924c:81 [Type locality: Costa Rica, Sarapiqui, La Virgen; MNHNP; ♂; in *Leptonema*]. —Mosely, 1933:65 [to *Centromacronema*]. —Holzenthal, 1988c:65 [distribution].

**Distribution.** Costa Rica.

***felfele*** Oláh and Johanson, 2012:218 [Type locality: Peru, River Salvation, Mt Manu, Mother of Good [sic, Madre de Díos], 71°21'22"W, 12°50'16"S, el. 546 m; NRM; ♂].

**Distribution.** Peru.

***ferrugineum*** (Navás), 1924c:80 [Type locality: Costa Rica; MNHNP; ♂; in *Leptonema*]. —Mosely, 1933:65 [to *Centromacronema*]. —Holzenthal, 1988c:65 [distribution].

**Distribution.** Costa Rica.

***kanalas*** Oláh and Johanson, 2012:218 [Type locality: Peru, Dept. Pasco, Yanachaga Chemilien [sic, Chemillén] NP, INRENA Refugio El Cedro, 10°32.717S, 75°21.492W, el. 2460 m; HNHM; ♂].

**Distribution.** Peru.

***nigrifrons*** Banks, 1913a:238 [Type locality: Rio Negro, E. Colombia; MCZ; ♂]. —Flint, 1967c:8 [discussion of holotype]. —Muñoz-Quesada, 2000:276 [checklist].

**Distribution.** Colombia.

***nigripenne*** Flint, 1981a:18 [Type locality: Venezuela, Miranda, Parque Nacional Guatopo, Santa Cruz de Río Grande; NMNH; ♂].

**Distribution.** Venezuela.

***oaxacensis*** Bueno-Soria, in Flint et al., 1999a:76 [replacement name for *Centromacronema ferrugineum* Bueno-Soria, 1986:62, preoccupied by *Leptonema ferrugineum* (Navás), 1924c:80]. [Type locality: Mexico, Oaxaca, Metates, 5 km al S de Valle Nacional (Sierra de Jurez); IBUNAM; ♂].

**Distribution.** Mexico.

***obscurum*** (Ulmer), 1905a:83 [Type locality: Brazil, Alta da Serra bei Santos; BMNH; ♂; in *Macronema*]. —Ulmer, 1907c:112 [to synonymy of *auripenne*]. —Flint, 1996b:411 [resurrected, distribution]. —Blahnik et al., 2004:4 [distribution]. —Paprocki et al., 2004:7 [checklist]. —Calor, 2011:320 [checklist]. —Paprocki and França, 2014:22 [checklist].

**Distribution.** Bolivia, Brazil, Honduras, Peru.

***oculatum*** (Walker), 1852:75 [Type locality: Venezuela; BMNH; ♂; in *Macronema*]. —Ulmer, 1907d:166 [to *Centromacronema*]. —Betten and Mosely, 1940:207 [♂; lectotype; venation]. —Flint, 1981a:18 [♂; distribution; wings]. —Muñoz-Quesada, 2000:276 [checklist].

**Distribution.** Colombia, Venezuela.

***pioneira*** Dias and Calor, 2016:130 [Type locality: Brazil, Bahia, Santa Teresinha, Pedra Branca, Serra da Jiboia, Morro da Pioneira, Córrego das Torres, 12°51'01.6’’S, 39°28'48’’W, el. 679 m; MZUSP; ♂; ♀].

**Distribution.** Brazil.

***poyanawa*** Dias and Calor, 2016:133 [Type locality: Brazil, Acre, Mâncio Lima, Parque Nacional da Serra do Divisor, Igarapé Amor, 07°26'46.6’’S, 73°40'10.8’’W, el. 291 m; MZUSP; ♂].

**Distribution.** Brazil.

***pygmaeum*** Botosaneanu, in Botosaneanu and Alkins-Koo, 1993:33 [Type locality: Trinidad, Northern Coast Road W. from Maracas Bay, hygropetric habitat (Spring from rock); ZMUA; ♂]. —Botosaneanu and Sakal, 1992 [for 1993]:203 [*nomen nudum* (name included in checklist); distribution; ecology]. —Flint, 1996a:84 [distribution]. —Botosaneanu, 2002:92 [checklist].

**Distribution.** Trinidad, Venezuela.

***talan*** Oláh and Johanson, 2012:220 [Type locality: Peru, Huanuco, stream at Carpish, 76°09'W, 9°40'S, el. 2500 m; NRM; ♂].

**Distribution.** Peru.

#### Genus *Cheumatopsyche* Wallengren [6]

*Cheumatopsyche* Wallengren, 1891:138, 142 [Type species: *Hydropsyche lepida* Pictet 1834, by monotypy]. —Gordon, 1974:117 [revision, Nearctic species]. —Schefter, 2005:149 [phylogeny]. —Oláh and Johanson, 2008:176 [review of species groups]. —Oláh et al., 2008:1 [review of Oriental and Afrotripical species]. —Geraci et al., 2010:925 [classification; phylogeny].

This is a large genus of several hundred species found on all continents except South America. Several species occuring in the southwestern U.S.A. also occur in Nearctic northern Mexico and are listed below.

Immature stages of a number of species from many regions of the world have been described, (Lepneva 1970, Scott 1983, Wiggins 1996). The larvae spin retreats and silken nets to capture food from flowing waters.

***arizonensis*** (Ling), 1938:66 [Type locality: United States, Arizona, Chiricahua Mts.; CAS; ♂; in *Hydropsychodes*]. —Flint, 1967d:168 [distribution; as *zion* Ross]. —Gordon, 1974:124 [♂; ♀; redescription]. —Bueno-Soria and Flint, 1978:205 [distribution]. —Blinn and Ruiter, 2006:332 [biology]. —Bueno-Soria et al., 2007:33 [distribution]. —Blinn and Ruiter, 2009a:303 [biology]. —Blinn and Ruiter, 2009b:185 [phenology, distribution].

—*zion* Ross, 1947:141 [Type locality: United States, Utah, Zion National Park; INHS; ♂]. —Gordon, 1974:124 [to synonymy].

**Distribution.** Mexico, U.S.A.

***enonis*** Ross, 1938b:153 [Type locality: [United States], Wyoming, Parco, along North Platte River near town; MCZ; ♂; ♀]. —Ross, 1944:294 [♂]. —Gordon, 1974: 142 [review]. —Blinn and Ruiter, 2006:332 [biology]. —Bueno-Soria et al., 2007:33 [distribution]. —Blinn and Ruiter, 2009a:304 [biology].

—*geolca* Denning, 1952:21 [Type locality: [United States], Nevada, Humboldt River, near Carlin, el. 4900; CAS; ♂]. —Gordon, 1974:142 [to synonymy].

**Distribution.** Mexico, U.S.A.

***gelita*** Denning, 1952:20 [Type locality: United States, Arizona, Diamond Creek, White Mts.; SEMC; ♂]. —Flint, 1967d:168 [distribution]. —Gordon, 1974:143 [♂; ♀; redescription]. —Bueno-Soria and Flint, 1978:206 [distribution]. —Bueno-Soria et al., 2007:33 [distribution]. —Blinn and Ruiter, 2009a:303 [biology].

**Distribution.** Mexico, U.S.A.

***lasia*** Ross, 1938b:154 [Type locality: United States, Oklahoma, Davis; INHS; [♂; ♀]. —Gordon, 1974:132[♂; ♀; redescription]. —Bueno-Soria and Flint, 1978:206 [distribution]. —Baumgardner and Bowles, 2005:11 [distribution]. —Blinn and Ruiter, 2006:332 [biology]. —Bowles et al., 2007:22 [distribution; biology]. —Bueno-Soria et al., 2007:33 [distribution].

**Distribution.** Mexico, U.S.A.

***mickeli*** Denning, 1942:50 [Type locality: United States, California, Morgan Hill (Santa Clara County); UMSP; [♂; ♀]. —Ross, 1951a:71 [distribution]. —Denning, 1964:133 [checklist; as *micleli*]. —Gordon, 1974:132 [♂; ♀; redescription]. —Bueno-Soria and Flint, 1978:206 [distribution].

**Distribution.** Mexico, U.S.A.

***pinula*** Denning, 1952:21 [Type locality: United States, Arizona, Carrizo Creek, near Carrizo; SEMC; ♂]. —Gordon, 1974:133 [♂; ♀; redescription]. —Bueno-Soria and Flint, 1978:206 [distribution]. —Blinn and Ruiter, 2006:332 [biology]. —Blinn and Ruiter, 2009a:304 [biology]. —Blinn and Ruiter, 2009b:185 [phenology, distribution].

**Distribution.** Mexico, U.S.A.

#### Genus *Diplectrona* Westwood [2]

*Diplectrona* Westwood, 1839:49 [Type species: *Aphelocheira flavomaculata* Stephens, 1836, *nec* Pictet, synonym of *Diplectrona felix* McLachlan, original designation].

*Diplectrona*, with about 120 species, is found in all biogeographic regions, but concentrated in the Oriental and Australasian regions and absent from South America. In the New World it is exclusively northern with sparse representation in Mexico and Guatemala at higher elevations. Schefter (1996) provided evidence that neither the genus nor the subfamily may be monophyletic.

In Mesoamerica, the immature stages live in small streams at higher elevations, usually in the pine forest zone. They construct a typical hydropsychid capture net and retreat on solid objects in the substrate. The retreat is rather rough and bulky, constructed mostly of irregular pieces of plant matter (Flint, personal observation).

***chiapensis*** Flint, 1967b:14 [Type locality: Mexico (Chiapas), Dolores, route 190, km 1190; NMNH; ♂]. —Bueno-Soria and Flint, 1978:205 [distribution]. —Bueno-Soria and Barba-Álvarez, 2011:355 [checklist].

**Distribution.** Guatemala, Mexico.

***solitaria*** Bueno-Soria, 1986:55 [Type locality: Mexico, Oaxaca, Ruta 175 Miahuatlán-Pochutla; IBUNAM; ♂].

**Distribution.** Mexico.

#### Genus *Hydropsyche* Pictet [9]

*Hydropsyche* Pictet, 1834:23,139 [Type species: *Hydropsyche cinerea* Pictet, 1834, subsequent designation of Ross, 1944, generally considered a synonym of *instabilis* Curtis]. —Schefter, 2005:148 [revision; phylogeny]. —Oláh and Johanson, 2008:56 [revision]. —Geraci et al. 2010:925 [revision].

*Ceratopsyche* Ross and Unzicker, 1977:305 [Type species: *Hydropsyche bronta* Ross, 1938b, original designation, as subgenus of *Symphitopsyche* Ulmer, 1907a]. —Schefter et al., 1986:69 [as subgenus of *Hydropsyche*]. —Oláh and Johanson, 2008:56 [to synonymy]. —Geraci et al., 2010:926 [revision].

*Mexipsyche* Ross and Unzicker, 1977:305 [Type species: *Mexipsyche dampfi* Ross and Unzicker, 1977, original designation]. —Oláh and Johanson, 2008:56 [to synonymy]. —Geraci et al., 2010:926 [classification].

This genus of almost 600 species is cosmopilitan, but absent from South America and Australia. Like *Cheumatopsyche*, the species in Latin America are largely found in the northern half of Mexico, although those formerly placed in *Mexipsyche* occur in southern Mexico and Guatemala. *Hydropsyche* has been the subject a several taxonomic and phylogenetic assessments (Schefter et al., 1986, Schefter 2005, Oláh and Johanson 2008) and its once unsteady classification is nearing a stable concensus (Geraci et al., 2010).

The well-known larvae are common and abundant in flowing waters or along the waveswept shores of northern lakes (Ross 1944, Wallace and Merritt 1980, Schuster and Etnier 1978, Shefter and Wiggins 1986). Their nets have been the subjects of many studies (Merritt and Wallace 1981). The larva, pupa, retreat and habitat of *Hydropsyche toschiae* (as *Mexipsyche*) were described by Bueno-Soria (1984c).

***ancestralis*** (Ross and Unziker), 1977:306 [Type locality: Mexico, Oaxaca, Jaloa; INHS; ♂; as *Mexipsyche*]. —Flint et al., 1999b:69 [catalog, as *Mexipsyche*]. —Oláh and Johanson, 2008:56 [to *Hydropsyche*].

**Distribution.** Mexico.

***auricolor*** Ulmer, 1905c:33 [Type locality: Mexico; MNHNP; ♂]. —Bueno-Soria and Flint, 1978:206 [distribution]. —Blinn and Ruiter, 2006:332 [biology]. —Bueno-Soria et al., 2007:32 [distribution]. —Blinn and Ruiter, 2009a:304 [biology; distribution]. —Blinn and Ruiter, 2009b:185 [phenology, distribution].

—*solex* Ross, 1944:271 [Type locality: United States, Texas, Balmorhea, along stone irrigation flume; INHS; ♂]. —Flint, 1967d:168 [distribution]. —Bueno-Soria and Flint, 1978:190 [to synonymy].

**Distribution.** Mexico, U.S.A.

***dampfi*** (Ross and Unzicker), 1977:306 [Type locality: Mexico, Chiapas, Tecpatan; INHS; ♂; as *Mexipsyche*]. —Flint et al., 1999b:69 [catalog, as *Mexipsyche*]. —Oláh and Johanson, 2008:56 [to *Hydropsyche*]. —Bueno-Soria and Barba-Álvarez, 2011:355 [checklist].

**Distribution.** Mexico.

***delrio*** Ross, 1941:86 [Type locality: United States, Texas, near San Felipe Springs, Del Rio; INHS; ♂; ♀]. —Bueno-Soria and Flint, 1978:206 [distribution]. —Bowles et al., 2007:22 [distribution; biology]. —Bueno-Soria et al., 2007:32 [distribution].

**Distribution.** Mexico, U.S.A.

***occidentalis*** Banks, 1900:258 [Type locality: United States, Washington, Pullman; MCZ; ♂]. —Ross, 1938a:17 [♂; lectotype]. —Denning, 1964:133 [checklist]. —Flint, 1967d:169 [distribution]. —Bueno-Soria and Flint, 1978:206 [distribution]. —Blinn and Ruiter, 2006:332 [biology]. —Blinn and Ruiter, 2009a:303 [biology]. —Blinn and Ruiter, 2009b:185 [phenology, distribution].

—*novamexicana* Banks, 1904a:110 [Type locality: New Mexico, Roswell; MCZ; ♂]. —Milne, 1936:73 [to synonymy].

**Distribution.** Mexico, U.S.A.

***oslari*** Banks, 1905:13 [Type locality: United States, southwestern Colorado; MCZ; ♂; ♀]. —Ross, 1938a:18 [♂; lecotype]. —Denning, 1964:133 [checklist]. —Flint, 1967d:168 [distribution]. —Bueno-Soria and Flint, 1978:206 [distribution]. —Blinn and Ruiter, 2006:332 [biology]. —Blinn and Ruiter, 2009a:303 [biology]. —Blinn and Ruiter, 2009b:185 [phenology, distribution].

—*partita* Banks, 1914a:252 [Type locality: United States, California, San Gabriel Mts., Switzer’s Camp; MCZ; ♂]. —Milne, 1936:73 [to synonymy].

**Distribution.** Mexico, U.S.A.

***philo*** Ross, 1941:90 [Type locality: United States, California, Hastings Natural History Reservation, Monterey County; MCZ; ♂; ♀]. —Ross, 1951a:70 [distribution]. —Denning, 1964:133 [checklist]. —Bueno-Soria and Flint, 1978:206 [distribution].

**Distribution.** Mexico, U.S.A.

***toschiae*** Denning, 1965a:80 [Type locality: Mexico, Veracruz, Fortin de las Flores, near Córdoba; CAS; ♂; ♀]. —Ross and Unzicker, 1977:306 [♂; ♀; to *Mexipsyche*]. —Bueno-Soria and Flint, 1978:206 [distribution; in *Hydropsyche*]. —Bueno-Soria, 1984c:49 [biology; retreat; larva; pupa]. —Flint et al., 1999b:69 [catalog, as *Mexipsyche*]. —Rojas-Ascencio, et al., 2002:377 [distribution]. —Oláh and Johanson, 2008:56 [to *Hydropsyche*].

**Distribution.** Guatemala, Mexico.

***vespertina*** Flint, 1967b:14 [Type locality: Mexico, Michoacan, Tuxpan; NMNH; ♂]. —Bueno-Soria and Flint, 1978:206 [distribution]. —Rojas-Ascencio, et al., 2002:377 [distribution]. —Bueno-Soria et al., 2007:32 [distribution].

**Distribution.** Mexico.

#### Genus *Leptonema* Guérin-Méneville [125]

*Leptonema* Guérin-Méneville, 1843:396 [Type species: *Leptonema pallida* Guérin-Méneville, 1843, by monotypy]. —Ulmer 1907a:58 [catalog]. —Mosely, 1933:1 [revision]. —Flint et al., 1987:1 [revision]. —Muñoz-Quesada, 1999:963 [Costa Rican species; key].

*Neoleptonema* Ulmer, 1907b:61 [Type species: *Neoleptonema aspersum* Ulmer, 1907, by monotypy]. —Flint et al., 1987:3 [to synonymy].

Flint et al. (1987) provided a comprehensive review of the genus, but since then 34 new species have been described from the region. The genus also has species in Africa and Madagascar. The adults are much larger than most Neotropical caddisflies and the common and abundant larvae certainly contribute greatly to secondary production in streams.

Larvae live in a wide range of running water habitats, from small first order streams to large meandering rivers (Flint and Wallace 1980, Scott 1983, Wiggins 1996, Nessimian and Dumas 2010). Oliveira and Froehlich (1996) recorded diatoms as the most frequent food item of an unidentified Brazilian species, but green algae and insect fragments were also common in the gut. The food consumed by larvae of *Leptonema tridens* was reported to be plant fibers, insect parts, filamentous algae, fungal hyphae, and detrital matter (Nessimian and Dumas 2010), indicating that the species was an omnivorous filterer, likely typical of the genus as a whole.

***acutum*** Mosely, 1933:61 [Type locality: Guatemala, Baja Verapaz, Panimá; BMNH; ♂]. —Flint et al., 1987:51 [♂; distribution]. —Chamorro-Lacayo et al., 2007:41 [checklist].

**Distribution.** Guatemala, Mexico, Nicaragua.

***agraphum*** (Kolenati), 1859:148 [Type locality: Brazil; NMW; ♂; in *Macronema*]. —Flint et al., 1987:43 [♂; distribution]. —Paprocki et al., 2004:7 [checklist]. —Dumas et al., 2009:357 [distribution]. —Paprocki and França, 2014:23 [checklist].

—*trilobata* (Jacquemart), 1962:1 [Type locality: Brazil, Edo. Rio de Janeiro, Bomanca; IRSNB; ♂; in *Hydropsyche*]. —Flint et al., 1987:43 [to synonymy].

**Distribution.** Brazil.

***albovirens*** (Walker), 1852:76 [Type locality: Venezuela; BMNH; ♂; in *Macronema*]. —Flint, 1968b:31 [♂; ♀; larva; pupa]. —Flint, 1981a:19 [♂; distribution]. —Bueno-Soria and Flint, 1978:208 [distribution]. —Flint et al., 1987:65 [♂; distribution]. —Maes and Flint, 1988:4 [distribution]. —Holzenthal, 1988c:66 [distribution]. —Flint, 1991:83 [♂; distribution]. —Aguila, 1992:540 [distribution]. —Botosaneanu and Sakal, 1992:203 [distribution; ecology]. —Botosaneanu and Alkins-Koo, 1993:33 [distribution]. —Flint and Sykora, 1993:54 [distribution]. —Flint, 1996a:83 [distribution]. —Maes, 1999:1185 [checklist]. —Muñoz-Quesada, 1999:989 [♂; distribution]. —Muñoz-Quesada, 2000:276 [checklist]. —Botosaneanu, 2002:93 [checklist]. —Botosaneanu and Viloria, 2002:108 [distribution]. —Bueno-Soria et al., 2005:75 [distribution]. —Bueno-Soria et al., 2007:33 [distribution]. —Chamorro-Lacayo et al., 2007:41 [checklist]. —Bueno-Soria and Barba-Álvarez, 2011:355 [checklist]. —Djernaes, 2011:8 [♂; ♀]. —Armitage et al., 2015b:5 [checklist]. —Armitage and Cornejo, 2015:193 [checklist].

—*guatemalum* Banks, 1913b:89 [Type locality: Guatemala, Dpto. Solola, Olas de Moka; MCZ; ♂]. —Flint, 1967c:8 [to synonymy].

**Distribution.** Belize, Colombia, Costa Rica, Grenada, Guatemala, Honduras, Mexico, Nicaragua, Panama, St. Vincent, Tobago, Trinidad, U.S.A., Venezuela.

***album*** Mosely, 1933:49 [Type locality: Ecuador; ZSZMH; ♂]. —Flint et al., 1987:71 [♂; distribution]. —Oláh and Johanson, 2012:222 [distribution].

**Distribution.** Ecuador.

***alceatum*** Flint, McAlpine and Ross, 1987:65 [Type locality: Peru, Dpto. Cuzco, Santa Isabel, Cosipata Valley; NMNH; ♂]. —Flint, 1996b:414 [distribution].

**Distribution.** Bolivia, Peru.

***amazonense*** Flint, 1978:399 [Type locality: Brazil, Amazonas, Manaus, Reserva Ducke; INPA; ♂]. —Flint et al., 1987:39 [/male; distribution; wings]. —Paprocki et al., 2004:7 [checklist]. —Ribeiro et al., 2009:34 [list of types]. —Nogueira and Cabette, 2011:351 [distribution]. —Nogueira et al., 2011:176 [community ecology; distribution]. —Paprocki and França, 2014:23 [checklist].

**Distribution.** Brazil, Venezuela.

***amplifurcatum*** Dumas and Nessimian, 2009a:65 [Type locality: Brazil, Rio de Janeiro, Mangaratiba: Reserva Ecológica Rio das Pedras, Rio Grande, 22°59'31.20"S 44°06'18.00"W, el. 96 m; DZRJ; ♂]. —Paprocki and França, 2014:23 [checklist].

**Distribution.** Brazil.

***andinum*** Flint, 1991:83 [Type locality: Colombia, Dpto. Antioquia, Quebrada Espadera, 7 km E Medellín [road to Sta. Elena]; NMNH; ♂]. —Muñoz-Quesada, 2000:276 [checklist].

**Distribution.** Colombia.

***andrea*** Flint, McAlpine and Ross, 1987:51 [Type locality: Ecuador, Pcia. Pastaza, Estación Fluviometrico, 27 km N Puyo; NMNH; ♂].

**Distribution.** Ecuador.

***anomalum*** Flint, 2008:464 [Type locality: Peru, Depto. Cuzco, Pcia. Paucartambo, Puente San Pedro, [km 152], [44] km, NW Pilcopata, [13°03.30'S, 71°32.78'W, el. 1450 m]; MHNJP; ♂; ♀].

**Distribution.** Peru.

***araguense*** Flint, 1981a:20 [Type locality: Venezuela, Edo. Aragua, Choroni Pass; NMNH; ♂]. —Flint et al., 1987:52 [♂; distribution].

**Distribution.** Venezuela.

***archboldi*** Flint, 1968b:29 [Type locality: Dominica, 0.5 mi S Pont Casse; NMNH; ♂; larva]. —Flint et al., 1987:67 [♂; distribution]. —Botosaneanu, 1988:219 [distribution]. —Flint and Sykora, 1993:49 [checklist]. —Botosaneanu, 1994a:51 [distribution]. —Botosaneanu, 2002:93 [checklist]. —Botosaneanu and Thomas, 2005:56 [checklist].

**Distribution.** Dominica, Guadeloupe, Martinique.

***asclepium*** Flint, McAlpine and Ross, 1987:60 [Type locality: Costa Rica, Pcia. Cartago, Turrialba; NMNH; ♂]. —Holzenthal, 1988c:66 [distribution]. —Muñoz-Quesada, 1999:983 [♂; distribution]. —Chamorro-Lacayo et al., 2007:41 [checklist].

**Distribution.** Costa Rica, Nicaragua.

***aspersum*** (Ulmer), 1907c:61 [Type locality: Brazil, Santa Rita; NMW; ♀; in *Neoleptonema*]. —Flint, 1974c:103 [♂; distribution]. —Flint et al., 1987:29 [♂; to *Leptonema*; distribution; wings]. —Flint, 1992d:67 [distribution]. —Paprocki et al., 2004:7 [checklist]. —Nogueira and Cabette, 2011:351 [distribution]. —Paprocki and França, 2014:23 [checklist].

**Distribution.** Brazil, Guyana, Suriname, Venezuela.

***aterrimum*** Mosely, 1933:21 [Type locality: Brazil, Unter Amazon, Taperinha, bei Santarem; NMW; ♂]. —Flint et al., 1987:42 [♂; distribution; wings]. —Paprocki et al., 2004:7 [checklist]. —Paprocki and França, 2014:23 [checklist].

**Distribution.** Brazil.

***auriculatum*** Flint, McAlpine and Ross, 1987:47 [Type locality: Bolivia, Dpto. La Paz, quebradas del Río Zongo; NMNH; ♂].

**Distribution.** Bolivia.

***banksi*** Mosely, 1933:55 [Type locality: Colombia, Bogota; MCZ; ♂]. —Flint et al., 1987:72 [♂; distribution]. —Muñoz-Quesada, 2000:276 [checklist].

**Distribution.** Colombia.

***bifurcatodes*** Flint, 2008:462 [Type locality: Brazil, Rio de Janeiro, Parque Nacional Itatiaia, Rio Campo Belo, 22°27.033'N[S], 44°36.318'W, el. 1300 m; MZSP; ♂; ♀]. —Dumas et al., 2009:357 [distribution]. —Dumas and Nessimian, 2012:12 [checklist]. —Paprocki and França, 2014:23 [checklist].

**Distribution.** Brazil.

***bifurcatum*** Flint, McAlpine and Ross, 1987:44 [Type locality: Brazil, Edo. Espirito Santo; NMW; ♂]. —Blahnik et al., 2004:4 [distribution]. —Paprocki et al., 2004:7 [checklist]. —Barcelos-Silva et al., 2012:1277 [distribution]. —Paprocki and França, 2014:23 [checklist].

**Distribution.** Brazil.

***bilobatum*** Schmid, 1964:318 [Type locality: Colombia, Dpto. Cundinamarca, Monterredondo; CNC; ♂]. —Flint et al., 1987:53 [♂; distribution]. —Muñoz-Quesada, 2000:276 [checklist].

**Distribution.** Colombia.

***boliviense boliviense*** Mosely, 1933:36 [Type locality: Bolivia, Tipuani, Quellft d’Beni; ZMZMH; ♂; destroyed]. —Flint et al., 1987:47 [♂; distribution]. —Oláh and Johanson, 2012:222 [distribution]. —Isa Miranda and Rueda Martín, 2014:200 [distribution]. —Rueda Martín and Isa Miranda, 2015:211 [redescription; ♂; larva; pupa; biology; distribution].

**Distribution.** Argentina, Bolivia, Peru.

***boliviense plumosum*** Flint, McAlpine and Ross, 1987:47 [Type locality: Argentina, Pcia. Tucuman, Cumbre Tafecillo; NMNH; ♂]. —Isa Miranda and Rueda Martín, 2014:200 [distribution].

**Distribution.** Argentina.

***boraceia*** Flint, McAlpine and Ross, 1987:44 [Type locality: Brazil, Edo. São Paulo, Estação Biológica Boracéia, Mun. Salesópolis; MZUSP; ♂; wings]. —Paprocki et al., 2004:7 [checklist]. —Dumas et al., 2009:357 [distribution]. —Calor, 2011:320 [checklist]. —Paprocki and França, 2014:24 [checklist].

**Distribution.** Brazil.

***bunkok*** Oláh and Johanson, 2012:222 [Type locality: French Guiana, Approuaguekaw, Kaw Mt, 104 mao, 4°33.035'N, 52°11.661'W; NRM; ♂].

**Distribution.** French Guiana.

***campanum*** Flint, McAlpine and Ross, 1987:60 [Type locality: Panama, Pcia. Panama, Cerro Campana, near Chica; NMNH; ♂]. —Holzenthal, 1988c:66 [distribution]. —Muñoz-Quesada, 1999:983 [♂; distribution]. —Armitage et al., 2015b:5 [checklist]. —Armitage and Cornejo, 2015:193 [checklist].

**Distribution.** Costa Rica, Panama.

***championi*** Mosely, 1933:35 [Type locality: Guatemala, Baja Vera Paz, Cahabon; BMNH; ♂]. —Flint et al., 1987:61 [♂; distribution].

**Distribution.** Guatemala, Mexico.

***cheesmanae*** Mosely, 1933:51 [Type locality: Colombia, Gorgona Island; BMNH; ♂]. —Flint et al., 1987:72 [♂; distribution]. —Aguila, 1992:540 [distribution]. —Muñoz-Quesada, 1997:130 [♂; distribution]. —Muñoz-Quesada, 1999:991 [♂; distribution]. —Muñoz-Quesada, 2000:276 [checklist]. —Armitage et al., 2015b:5 [checklist]. —Armitage and Cornejo, 2015:193 [checklist].

**Distribution.** Colombia, Costa Rica, Panama.

***chiapense*** Flint, McAlpine and Ross, 1987:53 [Type locality: Mexico, Edo. Chiapas, Cascada Misolja, 20 km S Palenque; NMNH; ♂]. —Bueno-Soria and Barba-Álvarez, 2011:355 [checklist].

**Distribution.** Mexico.

***chila*** Flint, 1967b:15 [Type locality: Mexico, Edo. Guerrero, near Chilpancingo (route 95, km 297); NMNH; ♂]. —Bueno-Soria and Flint, 1978:209 [distribution]. —Flint et al., 1987:53 [♂; distribution].

**Distribution.** Mexico.

***chocoense*** Flint, McAlpine and Ross, 1987:40 [Type locality: Colombia, Dpto. Choco, km 130, 86 km E Quibdo; NMNH; ♂; wings]. —Muñoz-Quesada, 2000:276 [checklist].

**Distribution.** Colombia.

***cinctum*** Ulmer, 1905a:64 [Type locality: Ecuador, Balzapamba; PAN; ♂]. —Flint, 1966a:5 [holotype; ♂]. —Flint et al., 1987:17 [♂; distribution; wings]. —Flint, 1991:81 [♂; distribution]. —Muñoz-Quesada, 2000:276 [checklist].

**Distribution.** Colombia, Ecuador.

***clorito*** Muñoz-Quesada, 1997:117 [Type locality: Costa Rica, Alajuela, Cerro Campana, ca.6 km (air) NW Dos Ríos, 10.9°N, 85.4°W; NMNH; ♂]. —Muñoz-Quesada, 1999:993 [♂; distribution].

**Distribution.** Costa Rica.

***coheni*** Flint, McAlpine and Ross, 1987:53 [Type locality: Ecuador, Pcia. Cotopaxi, 113 km W Latacunga; NMNH; ♂].

**Distribution.** Ecuador.

***columbianum*** Ulmer, 1905a:61 [Type locality: Colombia; PAN; ♀]. —Flint, 1966a:5 [♀; lectotype]. —Flint, 1974c:101 [♂; distribution]. —Flint, 1982c:31 [distribution]. —Flint et al., 1987:33 [♂; distribution]. —Flint, 1991:81 [♂; distribution]. —Muñoz-Quesada, 2000:276 [checklist]. —Cohen, 2004:75 [distribution]. —Paprocki et al., 2004:7 [checklist]. —Calor, 2011:320 [checklist]. —Paprocki and França, 2014:24 [checklist]. —Moretto and Bispo, 2015:126 [distrtibution].

—*externum* Banks, 1913b:87 [Type locality: Brazil, camp 41, 360 km from Porto Velho; MCZ; ♀]. —Mosely, 1933:13 [to synonymy].

—*cellare* Navás, 1927a:41 [Type locality: Brazil, Minas Gerais; DEI; ♀]. —Flint, 1978:385 [to synonymy].

—*silvestrinum* Navás, 1934a:168 [Type locality: Brazil, Mato Grosso, Corumba; DEI; ♂]. —Flint et al., 1987:17 [to synonymy].

**Distribution.** Argentina, Bolivia, Brazil, Colombia, Guyana, Paraguay, Peru, Suriname, Venezuela.

***complexum*** Mosely, 1933:54 [Type locality: Panama, Bugaba; BMNH; ♂]. —Flint et al., 1987:72 [♂; distribution]. —Holzenthal, 1988c:66 [distribution]. —Aguila, 1992:540 [distribution]. —Muñoz-Quesada, 1999:993 [♂; distribution]. —Armitage et al., 2015b:5 [checklist]. —Armitage and Cornejo, 2015:193 [checklist].

**Distribution.** Costa Rica, Panama.

***coronatum*** Flint, 2008:461 [Type locality: Venezuela, T. F. [now Edo.] Amazonas, Río Agua Blanca, 29 km Puerto Ayacucho; NMNH; ♂].

**Distribution.** Venezuela.

***crassum*** Ulmer, 1905a:58 [Type locality: Brazil, Espirito Santo; ZSZMH; ♂; destroyed]. —Weidner, 1964:84 [type]. —Flint, 1972b:235 [distribution]. —Flint, 1978b:385 [type; distribution]. —Bueno-Soria and Flint, 1978:209 [distribution]. —Flint et al., 1987:34 [♂; distribution]. —Maes and Flint, 1988:4 [distribution]. —Holzenthal, 1988c: 66 [distribution]. —Aguila, 1992:540 [distribution]. —Flint, 1996b:415 [distribution]. —Maes, 1999:1185 [checklist]. —Muñoz-Quesada, 1999:966 [♂; distribution]. —Muñoz-Quesada, 2000:276 [checklist]. —Cohen, 2004:75 [distribution]. —Paprocki et al., 2004:7 [checklist]. —Chamorro-Lacayo et al., 2007:41 [checklist]. —Calor, 2011:320 [checklist]. —Nogueira and Cabette, 2011:351 [distribution]. —Barcelos-Silva et al., 2012:1277 [distribution]. —Paprocki and França, 2014:24 [checklist]. —Armitage et al., 2015b:5 [checklist]. —Moretto and Bispo, 2015:126 [distrtibution]. —Armitage and Cornejo, 2015:193 [checklist].

—*radiale* Navás, 1927a:42 [Type locality: Brazil, Minas Gerais; DEI; ♀]. —Flint, 1978:385 [to synonymy].

—*grisolinum* Navás, 1933b:312 [Type locality: Venezuela, Edo. Miranda, Caucagua; MNHNP; ♂]. —Flint et al., 1987:17 [to synonymy].

**Distribution.** Argentina, Brazil, Colombia, Costa Rica, Guatemala, Honduras, Mexico, Nicaragua, Panama, Paraguay, Peru, Venezuela.

***cressae*** Flint, 2008:468 [Type locality: Venezuela, Edo. Tachira, Queb. Mesa del Palmar, 5 km S El Cobre, 7°59.851'N, 72°03.759'W; UMSP; ♂; ♀].

**Distribution.** Venezuela.

***davisi*** Flint, McAlpine and Ross, 1987:41 [Type locality: Venezuela, T.F. Amazonas, Cerro de la Neblina, basecamp, 0°50'N, 66°10'W; NMNH; ♂; wings].

**Distribution.** Venezuela.

***divaricatum*** Flint, McAlpine and Ross, 1987:36 [Type locality: Ecuador, Pcia. Pichincha, 29 km W Santo Domingo de los Colorados; NMNH; ♂]. —Holzenthal, 1988c:67 [distribution]. —Flint, 1991:81 [♂; distribution]. —Muñoz-Quesada, 1999:971 [♂; distribution]. —Muñoz-Quesada, 2000:276 [checklist].

—*crassum* Mosely, 1933:12 [misidentification [Cachabé, Ecuador], *sensu* Flint et al., 1987]. —Fischer, 1947:313 [misidentification *sensu* Flint et al., 1987]. —Schmid, 1964:317 [misidentification *sensu* Flint et al., 1987]. —Flint, 1981a:20 [misidentification *sensu* Flint et al., 1987].

**Distribution.** Colombia, Costa Rica, Ecuador, Venezuela.

***dyeri*** Flint, McAlpine and Ross, 1987:61 [Type locality: Honduras, Tegucigalpa; NMNH; ♂]. —Maes and Flint, 1988:4 [distribution]. —Maes, 1999:1185 [checklist]. —Chamorro-Lacayo et al., 2007:41 [checklist].

**Distribution.** El Salvador, Guatemala, Honduras, Nicaragua.

***ekisi*** Flint, McAlpine and Ross, 1987:54 [Type locality: Panama, Pcia. Chiriqui, Bambito; NMNH; ♂]. —Holzenthal, 1988c:67 [distribution]. —Aguila, 1992:540 [distribution]. —Muñoz-Quesada, 1999:972 [♂; distribution]. —Armitage et al., 2015b:5 [checklist]. —Armitage and Cornejo, 2015:193 [checklist].

**Distribution.** Costa Rica, Panama.

***enikolah*** Oláh and Johanson, 2012:223 [Type locality: Colombia, Minca, el. 650 m; OPC; ♂].

**Distribution.** Colombia.

***eugnathum*** (Müller), 1921:536 [Type locality: Brazil, [Sta. Catarina], Itajahy; MCZ; ♂; in *Macronema*]. —Flint et al., 1987:45 [♂; distribution]. —Paprocki et al., 2004:7 [checklist]. —Paprocki and França, 2014:24 [checklist].

—*ochraceum* Mosely, 1933:28 [Type locality: Brazil, Sta. Catarina, Boiteuxburgo; ZSZMH; ♂; destroyed]. —Ulmer, 1957:341 [to synonymy].

**Distribution.** Brazil.

***ferelunatum*** Jardim, Dumas and Nessimian 2010:51 [Type locality: Brazil, Rio de Janeiro state, Nova Friburgo municipality, Lumiar, Córrego Boa Vista, Cachoeira Indiana Jones, 22°19'02.1"S, 42°17'28.5"W, el. 900 m; DZRJ; ♂]. —Paprocki and França, 2014:24 [checklist].

**Distribution.** Brazil.

***flintorum*** Muñoz-Quesada, 1997:119 [Type locality: Costa Rica, Puntarenas, Río Bellavista, ca. 1.5 km NW Las Alturas, 8.951°N, 82.846°W; NMNH; ♂]. —Muñoz-Quesada, 1999:972 [♂; distribution].

**Distribution.** Costa Rica.

***forficulum*** Mosely, 1933:52 [Type locality: Panama, Cabima; MCZ; ♂]. —Flint et al., 1987:73 [♂; distribution]. —Holzenthal, 1988c:67 [distribution]. —Aguila, 1992:540 [distribution]. —Muñoz-Quesada, 1999:998 [♂; distribution]. —Chamorro-Lacayo et al., 2007:41 [checklist]. —Armitage et al., 2015b:5 [checklist]. —Armitage and Cornejo, 2015:193 [checklist].

**Distribution.** Costa Rica, Nicaragua, Panama.

***fortunum*** Flint, McAlpine and Ross, 1987:54 [Type locality: Panama, Pcia. Chiriqui, Fortuna Dam Site; NMNH; ♂]. —McElravy et al., 1981:153 [as species A]. —Holzenthal, 1988c:67 [distribution]. —Aguila, 1992:540 [distribution]. —Muñoz-Quesada, 1999:976 [♂; distribution].—Armitage et al., 2015b:5 [checklist]. —Armitage and Cornejo, 2015:193 [checklist].

**Distribution.** Costa Rica, Panama.

***furciligerum*** Flint, McAlpine and Ross, 1987:73 [Type locality: Costa Rica, Pcia. Puntarenas, Golfito; NMNH; ♂]. —Holzenthal, 1988c:67 [distribution]. —Muñoz-Quesada, 1999:999 [♂; distribution].

**Distribution.** Costa Rica.

***gadzux*** Flint, McAlpine and Ross, 1987:43 [Type locality: Venezuela, T.F. Amazonas, San Carlos de Río Negro, 1°56'N, 67°03'W; NMNH; ♂].

**Distribution.** Venezuela.

***guyanense*** Flint, McAlpine and Ross, 1987:37 [Type locality: Venezuela, Edo Bolívar, Kanarakuni; IZAM; ♂].

**Distribution.** Venezuela.

***hamuli*** Flint, McAlpine and Ross, 1987:55 [Type locality: Panama, Canal Zone, Barro Colorado Island; NMNH; ♂]. —Holzenthal, 1988c:67 [distribution]. —Aguila, 1992:540 [distribution]. —Maes, 1999:1185 [checklist]. —Muñoz-Quesada, 1999:977 [♂; distribution]. —Chamorro-Lacayo et al., 2007:41 [checklist]. —Armitage et al., 2015b:5 [checklist]. —Armitage and Cornejo, 2015:193 [checklist].

**Distribution.** Costa Rica, Nicaragua, Panama.

***harpagum*** Flint, McAlpine and Ross, 1987:74 [Type locality: Peru, Dpto. Huanuco, Monson Valley, Tingo Maria; CAS; ♂]. —Oláh and Johanson, 2012:224 [distribution].

**Distribution.** Peru.

***heppneri*** Flint, McAlpine and Ross, 1987:55 [Type locality: Venezuela, Edo. Lara, Yacumbú National Park; NMNH; ♂].

**Distribution.** Venezuela.

***hirsutum*** Flint, 1974c:102 [Type locality: Suriname, Tapanahoni River, Granhoni Poeketi; RNH; ♂]. —Flint et al., 1987:38 [♂; distribution].

**Distribution.** Guyana, Suriname, Venezuela.

***huismanae*** Muñoz-Quesada, 1997:117 [Type locality: Costa Rica, Alajuela, Reserva Forestal San Ramón, Río San Lorencito and tribs., 10.216°N, 84.607°W; NMNH; ♂]. —Muñoz-Quesada, 1999:977 [♂; distribution].

**Distribution.** Costa Rica.

***inca*** Mosely, 1933:38 [Type locality: Peru, Pachitea; ZSZMH; ♂]. —Flint et al., 1987:74 [♂; distribution]. —Flint, 1996b:413 [distribution]. —Oláh and Johanson, 2012:224 [distribution].

**Distribution.** Bolivia, Peru.

***inspiratum*** Flint, McAlpine and Ross, 1987:56 [Type locality: Peru, Dpto. Puno, Río Inambari/Loromayu; CNC; ♂]. —Oláh and Johanson, 2012:225 [distribution].

**Distribution.** Peru.

***insulanum*** Banks, 1924:455 [Type locality: mislabelled as Puerto Rico, San Juan; MCZ; ♂]. —Flint, 1964a:36 [♂; larva; distribution; synonymy]. —Flint, 1968b:81 [Puerto Rico, in error]. —Flint, 1981a:20 [♂; distribution]. —Botosaneanu and Flint, 1982:16 [larva]. —Flint et al., 1987:45 [♂; distribution; not on Puerto Rico].

—*ulmeri* Mosely, 1933:39 [Type locality: Venezuela; BMNH; ♂]. —Flint, 1964a:36 [to synonymy].

**Distribution.** Venezuela.

***intermedium*** Mosely, 1933:48 [Type locality: Ecuador, Chimbo; ZSZMH; ♂]. —Weidner 1964:84 [type]. —Flint et al., 1987:74 [♂; distribution]. —Holzenthal, 1988c:67 [distribution]. —Aguila, 1992:540 [distribution]. —Muñoz-Quesada, 1999:999 [♂; distribution]. —Muñoz-Quesada, 2000:276 [checklist]. —Oláh and Johanson, 2012:225 [checklist]. —Armitage et al., 2015b:5 [checklist]. —Armitage and Cornejo, 2015:193 [checklist].

**Distribution.** Colombia, Costa Rica, Ecuador, Panama.

***irroratum*** Flint, 1974c:100 [Type locality: Suriname, Nassau Mountains, trail km 11.2, north valley near large falls; RNH; ♂]. —Flint et al., 1987:40 [♂; distribution; wings].

**Distribution.** Suriname, Venezuela.

***janolah*** Oláh and Johanson, 2012:225 [Type locality: Ecuador, Western Andean Slope, Alambi; OPC; ♂].

**Distribution.** Ecuador.

***ketos*** Oláh and Johanson, 2012:226 [Type locality: French Guiana, Approuaguekaw, Kaw Mt, 104 mao, 4°33.035'N, 52°11.661'W; NRM; ♂].

**Distribution.** French Guiana.

***kunbenorum*** Oláh and Johanson, 2012:227 [Type locality: Peru, dept. Pasco, Yanachaga Chemilien [sic, Chemillén] NP, INRENA Refugio El Cedro, 10°32.717S, 75°21.492W, el. 2460 m; HNHM; ♂].

**Distribution.** Peru.

***lacuniferum*** Flint, 1978:384 [Type locality: Brazil [Edo. Amazonas], Gebeit Endstation Rio Marauia, Bergbach II; NMNH; ♂]. —Flint et al., 1987:18 [♂; distribution]. —Paprocki et al., 2004:7 [checklist]. —Paprocki and França, 2014:24 [checklist].

**Distribution.** Brazil, Venezuela.

***lineaticorne*** Flint, 2008:458 [Type locality: Peru, Pcia. Madre de Dios, Manu, Pakitza, Biol. Sta., Quebrada Pakitza near intersection with Río Manu, 11°56.65'S, 71°16.98'W, el. 350 m; MHNJP; ♂; ♀]. —Flint, 1996: 415 [as n. sp. 2]. —Paprocki and França, 2014:24 [checklist].

**Distribution.** Brazil, Peru.

***lojaense*** Flint, McAlpine and Ross, 1987:18 [Type locality: Ecuador, Environs de Loja; MCZ; ♂; wings].

**Distribution.** Ecuador.

***lunatum*** Flint, McAlpine and Ross, 1987:38 [Type locality: Brazil, Edo. Santa Catarina, Corupa (Hansa Humboldt); AMNH; ♂]. —Paprocki et al., 2004:7 [checklist]. —Paprocki and França, 2014:24 [checklist].

**Distribution.** Brazil.

***macacu*** Flint, 2008:458 [Type locality: Brazil, Edo. Rio de Janeiro, Rio Macacú (2nd order), on RJ 116, Km 62, 22°23.201'S, 42°33.395'W, el. 840 m; MZSP; ♂]. —Dumas et al., 2009:357 [distribution]. —Paprocki and França, 2014:25 [checklist].

**Distribution.** Brazil.

***maculatum*** Mosely, 1933:20 [Type locality: Brazil, Unter Amazon, Taperinha, bei Santarem; NMW; ♂]. —Flint, 1974c:100 [♂; distribution]. —Flint et al., 1987:41 [♂; distribution; wings]. —Paprocki et al., 2004:7 [checklist]. —Nogueira and Cabette, 2011:351 [distribution]. —Nogueira et al., 2011:176 [community ecology, distribution]. —Oláh and Johanson, 2012:228 [distribution]. —Paprocki and França, 2014:25 [checklist].

**Distribution.** Brazil, French Guiana, Suriname.

***magas*** Oláh and Johanson, 2012:229 [Type locality: Peru, San Martin Prov., river crossing rd. Olmos-Tarapoto, 385 km (rd.) E Olmos Desv. Jaén, 5°40.055'S, 77°43.396'W; NRM; ♂].

**Distribution.** Peru.

***mandibulatum*** Flint, McAlpine and Ross, 1987:38 [Type locality: Peru, Dpto. Huanuco, Tingo Maria; NMNH; ♂]. —Flint, 1996b:415 [distribution]. —Oláh and Johanson, 2012:230 [distribution].

**Distribution.** Bolivia, Ecuador, Peru.

***masinca*** Oláh and Johanson, 2012:230 [Type locality: Peru, San Martin Prov., La Catarata de Ahuashiyascu, 6°27.544’ S, 76°18.192’ W; NRM; ♂].

**Distribution.** Peru.

***mastigion*** Flint, McAlpine and Ross, 1987:56 [Type locality: Ecuador, Pcia. Los Ríos, Río Palenque Biological Station; NMNH; ♂]. —Oláh and Johanson, 2012:231 [distribution].

**Distribution.** Ecuador.

***meginca*** Oláh and Johanson, 2012:232 [Type locality: Peru, San Martin Prov., creek crossing rd. Juan Guerra-Chazuta, 14 km (rd.) E Colombia Bridge, 6°35.594’ S, 76°13.172’ W; NRM; ♂].

**Distribution.** Peru.

***menkei*** Flint, McAlpine and Ross, 1987:18 [Type locality: Venezuela, Edo. Lara, Parque Nacional Yacambú; NMNH; ♂].

**Distribution.** Venezuela.

***michoacanense*** Flint, McAlpine and Ross, 1987:56 [Type locality: Mexico, Edo. Michoacán, San Lorenzo, rt. 15, km 206; NMNH; ♂]. —Rojas-Ascencio, et al., 2002:377 [distribution].

**Distribution.** Mexico.

***moselyi*** Flint, McAlpine and Ross, 1987:67 [Type locality: Mexico, Edo. Morelos, Xochitepec; NMNH; ♂]. —Rojas-Ascencio, et al., 2002:377 [distribution]. —Bueno-Soria et al., 2007:33 [distribution].

**Distribution.** Mexico.

***naevosum*** Navás, 1916b:66 [Type locality: Colombia, Coachí; collection Apollinaris, probably lost; ♀]. —Flint et al., 1987:76 [*nomen dubium*]. —Muñoz-Quesada, 2000:276 [checklist].

**Distribution.** Colombia.

***neadelphus*** Flint, McAlpine and Ross, 1987:49 [Type locality: Colombia, Dpto. Antioquia, 10 km E Medellín, road to La Palma; NMNH; ♂]. —Flint, 1991:81 [♂; distribution]. —Muñoz-Quesada, 2000:276 [checklist].

**Distribution.** Colombia, Venezuela.

***neblinense*** Flint, McAlpine and Ross, 1987:41 [Type locality: Venezuela, T.F. Amazonas, Cerro de la Neblina, Camp X, 0°54'N, 60°2'W; NMNH; ♂; wings].

**Distribution.** Venezuela.

***nubestre*** Flint, 2008:456 [Type locality: Colombia, Dept. Magdalena, Las Nubes, el. 1370 m; CMNH; ♂].

**Distribution.** Colombia.

***nygmosum*** Navás, 1916b:65 [Type locality: Colombia, Coachí; collection Navás, now lost?; ♂]. —Flint et al., 1987:76 [*nomen dubium*]. —Muñoz-Quesada, 2000:276 [checklist].

**Distribution.** Colombia.

***olmos*** Oláh and Johanson, 2012:233 [Type locality: Peru, Amazonas Prov., river crossing Olmos-Tarapoto rd., 71 km (rd.) E Olmos Desv. Jaén, 5°41.178'S, 77°46.421'W; NRM; ♂].

**Distribution.** Peru.

***pallidum*** Guérin-Méneville, 1843:396 [Type locality: Brazil; type depository unknown; sex unknown]. —Banks, 1901:370 [misidentification]. —Flint et al., 1987:68 [♂; distribution]. —Oliveira and Froehlich, 1996:757 [biology]. —Paprocki et al., 2004:7 [checklist]. —Dumas et al., 2009:357 [distribution]. —Calor, 2011:320 [checklist]. —Dumas and Nessimian, 2012:12 [checklist]. —Barcelos-Silva et al., 2012:1277 [distribution]. —Oláh and Johanson, 2012:234 [distribution]. —Costa et al., 2014:219 [distribution]. —Paprocki and França, 2014:25 [checklist]. —Moretto and Bispo, 2015:126 [distrtibution].

—*furcatum* Ulmer, 1905a:57 [Type locality: Brazil, Espírito Santo; PAN; ♂]. —Mosely, 1939b:310 [to synonymy].

—*flagellata* (Jacquemart), 1962:6 [Type locality: Brazil, Edo. de Rio de Janeiro, Bomanca; IRSNB; ♂; in *Hydropsyche*]. —Flint et al., 1987:68 [to synonymy].

**Distribution.** Argentina, Brazil, French Guiana.

***piliferum*** Schmid, 1964:318 [Type locality: Bolivia, Cochabamba, Alto Palmar; CNC; ♂]. —Flint et al., 1987:19 [♂; distribution].

**Distribution.** Bolivia.

***pinotepa*** Bueno-Soria, Santiago-Fragoso, and Barba-Álvarez, 2001:153 [Type locality: Mexico, Oaxaca, Metates, Sierra de Juárez, el. 1600 m; CNIN; ♂].

**Distribution.** Mexico.

***plicatum*** Mosely, 1933:58 [Type locality: Guatemala, Dptos. Sololá and Suchitepéquez, Volcan de Atitlán; BMNH; ♂]. —Bueno-Soria and Flint, 1978:209 [distribution]. —Flint et al., 1987:57 [♂; distribution]. —Oláh and Johanson, 2012:235 [checklist].

**Distribution.** Guatemala, Mexico.

***poeyi*** (Banks), 1938:299 [Type locality: Cuba, coast below Pico Turquino; MCZ; ♂; in *Macronema*]. —Flint, 1967c:8 [♂; lectotype]. —Flint, 1968b:81 [checklist]. —Botosaneanu, 1979:47 [distribution]. —Botosaneanu, 1980:108 [distribution]. —Flint et al., 1987:30 [♂; distribution]. —Botosaneanu, 1994b:464 [larva]. —Flint, 1996c:16 [checklist]. —Botosaneanu, 2002:93 [checklist]. —Naranjo López and González Lazo, 2005:150 [checklist]. —López del Castillo et al., 2007:171 [distribution; seasonal abundance].

**Distribution.** Cuba.

***pseudocinctum*** Flint, McAlpine and Ross, 1987:19 [Type locality: Ecuador, Pcia. Tungurahua, 39 km E Banos; NMNH; ♂].

**Distribution.** Ecuador.

***pseudostigmosum*** Flint, 1981a:20 [Type locality: Venezuela, Aragua, Rancho Grande; MCZ; ♂]. —Flint et al., 1987:19 [♂; distribution].

**Distribution.** Venezuela.

***rafita*** Muñoz-Quesada, 1997:124 [Type locality: Costa Rica, Alajuela, Río Peje and falls, ca. 1 km SE San Vicente, Ciudad Quesada, 10.277°N, 84.388°W; NMNH; ♂]. —Muñoz-Quesada, 1999:979 [♂; distribution]. —Armitage et al., 2015a:4 [distribution]. —Armitage et al., 2015b:5 [checklist]. —Armitage and Cornejo, 2015:193 [checklist].

**Distribution.** Costa Rica, Panama.

***ramosum*** Flint, McAlpine and Ross, 1987:68 [Type locality: Venezuela, Edo. Bolvar, 10 km S of km 88, Piedra de Virgen; NMNH; ♂].

**Distribution.** Guyana, Suriname, Venezuela.

***rosenbergi*** Mosely, 1933:47 [Type locality: Ecuador, Cachabé; ZSZMH; ♂]. —Flint et al., 1987:75 [♂; distribution]. —Muñoz-Quesada, 2000:276 [checklist]. —Oláh and Johanson, 2012:235 [distribution].

**Distribution.** Colombia, Ecuador.

***rostratum*** Flint, McAlpine and Ross, 1987:30 [Type locality: Argentina, Pcia. Entre Ríos, Salto Grande; NMNH; ♂]. —Flint, 1992d:67 [distribution]. —Paprocki et al., 2004:7 [checklist]. —Ribeiro et al., 2009:34 [list of types]. —Nogueira and Cabette, 2011:351 [distribution]. —Costa et al., 2014:219 [distribution]. —Paprocki and França, 2014:25 [checklist].

**Distribution.** Argentina, Brazil, Uruguay.

***salvini*** Mosely, 1933:57 [Type locality: Panama, Volcan de Chiriqui; BMNH; ♂]. —Flint et al., 1987:46 [♂; distribution]. —Holzenthal, 1988c:67 [distribution]. —Aguila, 1992:540 [distribution]. —Muñoz-Quesada, 1999:980 [♂; distribution]. —Armitage et al., 2015b:5 [checklist]. —Armitage and Cornejo, 2015:193 [checklist].

**Distribution.** Costa Rica, Panama.

***sancticaroli*** Flint, McAlpine and Ross, 1987:30 [Type locality: Venezuela, T.F. Amazonas, 2 km E San Carlos de Río Negro; NMNH; ♂]. —Dumas et al., 2010:7 [distribution]. —Paprocki and França, 2014:25 [checklist].

**Distribution.** Brazil, Venezuela.

***santosi*** Jardim, Dumas and Nessimian, 2010:56 [Type locality: Brazil, Rio de Janeiro state, Nova Friburgo municipality, Lumiar, Córrego Boa Vista, Sítio Dois Irmãos, 22°19'01.5"S, 42°17'23.3"W, el. 910 m; DZRJ; ♂]. —Paprocki and França, 2014:25 [checklist].

**Distribution.** Brazil.

***serranum*** Navás, 1933c:112 [Type locality: Brazil, Edo. São Paulo, Alto da Serra; DEI; ♀]. —Flint et al., 1987:45 [placement]. —Paprocki et al., 2004:8 [checklist]. —Calor, 2011:320 [checklist]. —Paprocki and França, 2014:25 [checklist]. —Moretto and Bispo, 2015:126 [distrtibution].

**Distribution.** Brazil.

***serratum*** Jardim, Dumas and Nessimian, 2010:57 [Type locality: Brazil, Rio de Janeiro state, Macaé municipality, Crubixais de Cima, 1st order tributary of Rio Crubixais, 22°11'38.4"S, 42°04'46.1"W, el. 576 m; DZRJ; ♂]. —Paprocki and França, 2014:26 [checklist].

**Distribution.** Brazil.

***simplex*** Mosely, 1933:57 [Type locality: Ecuador, Loja; BMNH; ♂]. —Flint et al., 1987:57 [♂; distribution].

**Distribution.** Ecuador.

***simulans mayanum*** Flint, McAlpine and Ross, 1987:62 [Type locality: Guatemala, Dpto. Huehuetenango, 20 mi NW Huehuetenango; NMNH; ♂]. —Bueno-Soria and Flint, 1978:209 [distribution; as nominotypical species]. —Maes and Flint, 1988:5 [distribution]. —Maes, 1999:1185 [checklist]. —Bueno-Soria et al., 2005:75 [distribution]. —Chamorro-Lacayo et al., 2007:41 [checklist]. —Bueno-Soria and Barba-Álvarez, 2011:356 [checklist].

**Distribution.** Guatemala, Mexico, Nicaragua.

***simulans simulans*** Mosely, 1933:62 [Type locality: Panama, V. de Chiriqui; BMNH; ♂]. —Weidner, 1964:84 [type]. —Flint et al., 1987:62 [♂; distribution]. —Holzenthal, 1988c:67 [distribution]. —Aguila, 1992:540 [distribution]. —Muñoz-Quesada, 1999:985 [♂; distribution]. —Armitage et al., 2015b:5 [checklist]. —Armitage and Cornejo, 2015:193 [checklist].

**Distribution.** Costa Rica, Panama.

***sinuatum*** Mosely, 1933:59 [Type locality: Colombia, Gorgona Island; BMNH; ♂]. —Flint et al., 1987:57 [♂; distribution]. —Holzenthal, 1988c:68 [distribution]. —Aguila, 1992:540 [distribution]. —Muñoz-Quesada, 1999:981 [♂; distribution]. —Muñoz-Quesada, 2000:276 [checklist]. —Armitage et al., 2015b:5 [checklist]. —Armitage and Cornejo, 2015:193 [checklist].

**Distribution.** Colombia, Costa Rica, Panama.

***sociale*** Flint, 2008:464 [Type locality: Ecuador, Prov. Orellana, Río Tiputini, [Tiputini Biodiversity Station, SE Puerto Orellana], 0°38.2'S, 76°08.9'W; NMNH; ♂; ♀]. —Flint, 1996:414 [as n. sp. 3, distribution].

**Distribution.** Ecuador, Peru.

***spangleri*** Flint, McAlpine and Ross, 1987:69 [Type locality: Venezuela, Edo. Barinas, Barinitas; NMNH; ♂].

**Distribution.** Venezuela.

***sparsum*** (Ulmer), 1905a:76 [Type locality: Brazil; ZIUH; ♂; in *Macronema* (*Leptonema*?)]. —Flint, 1974c:98 [♂; distribution]. —Flint et al., 1987:32 [♂; distribution; wings]. —Aguila, 1992:540 [distribution]. —Flint, 1992d:68 [distribution]. —Flint, 1996b:415 [distribution]. —Marinoni and Almeida, 2000:286 [distribution; biology]. —Blahnik et al., 2004:4 [distribution]. —Cohen, 2004:75 [distribution]. —Paprocki et al., 2004:8 [checklist]. —Dumas et al., 2009:358 [distribution]. —Calor, 2011:320 [checklist]. —Nogueira and Cabette, 2011:351 [distribution]. —Nogueira et al., 2011:176 [community ecology, distribution]. —Oláh and Johanson, 2012:235 [checklist]. —Costa et al., 2014:220 [distribution]. —Paprocki and França, 2014:26 [checklist]. —Armitage et al., 2015b:5 [checklist]. —Moretto and Bispo, 2015:126 [distrtibution]. —Armitage and Cornejo, 2015:193 [checklist].

**Distribution.** Argentina, Brazil, Ecuador, Guyana, Panama, Paraguay, Peru, Suriname, Venezuela.

***speciosum*** (Burmeister), 1839:916 [Type locality: Brazil; ZIUH; ♂; as *Macronemum speciosum*]. —Flint et al., 1987:45 [♂; distribution; wings]. —Paprocki et al., 2004:8 [checklist]. —Dumas et al., 2009:358 [distribution]. —Paprocki and França, 2014:26 [checklist].

**Distribution.** Brazil.

***spinulum*** Flint, McAlpine and Ross, 1987:63 [Type locality: Peru, Dept. Cuzco, Cosnipata Valley, Hacienda Maria; NMNH; ♂]. —Flint, 1996b:414 [distribution]. —Paprocki et al., 2004:8 [checklist]. —Dumas et al., 2010:8 [distribution]. —Nogueira and Cabette, 2011:351 [distribution]. —Paprocki and França, 2014:26 [checklist].

**Distribution.** Argentina, Brazil, Guyana, Peru, Venezuela.

***spirillum*** Flint, McAlpine and Ross, 1987:50 [Type locality: Peru, Dept. Cuzco, Paucartambo, Cosnipata Valley; NMNH; ♂]. —Flint, 1991:83 [♂; distribution]. —Flint, 1996b:413 [distribution]. —Muñoz-Quesada, 2000:276 [checklist]. —Oláh and Johanson, 2012:235 [distribution].

**Distribution.** Bolivia, Colombia, Ecuador, Peru, Venezuela.

***stigmaticum*** Navás, 1916a:30 [Type locality: Brazil, Nueva Friburgo; collection Navás, now lost?; ♀]. —Flint et al., 1987:46 [Neotype: Brazil, Edo. Rio de Janeiro, 26 km E Nova Friburgo; MZUSP; ♂; distribution]. —Paprocki et al., 2004:8 [checklist]. —Dumas et al., 2009:358 [distribution]. —Paprocki and França, 2014:26 [checklist].

**Distribution.** Brazil.

***stigmosum*** Ulmer, 1905a:60 [Type locality: Ecuador, Balzapamba; PAN; ♂]. —Flint, 1966a:6 [♂; lectotype]. —Flint et al., 1987:50 [♂;distribution]. —Flint, 1991:83 [♂; distribution]. —Muñoz-Quesada, 2000:276 [checklist]. —Medellín et al., 2004:201 [distribution].

**Distribution.** Colombia, Ecuador, Venezuela.

***tapanti*** Muñoz-Quesada, 1997:128 [Type locality: Costa Rica, Cartago, Reserva Tapantí, Quebrada Palmitos and falls, 9.72°N, 83.78°W; NMNH; ♂]. —Muñoz-Quesada, 1999:971 [♂; distribution]. —Armitage et al., 2015b:5 [checklist]. —Armitage and Cornejo, 2015:193 [checklist].

**Distribution.** Costa Rica, Panama.

***tholloni*** Navás, 1923c:48 [Type locality: Gabon [in error; actually Brazil sensu Flint et al., 1987:46]; MNHNP; ♂]. —Flint et al., 1987:46 [♂; distribution]. —Paprocki et al., 2004:8 [checklist]. —Dumas et al., 2009:358 [distribution]. —Paprocki and França, 2014:26 [checklist].

**Distribution.** Brazil.

***tica*** Muñoz-Quesada, 1999:987 [Type locality: Costa Rica, San José, Parque Nacional Braulio Carrillo, Estación Carrillo, Quebrada Sanguijuela, 10.160°N, 83.963°W, el. 800 m; NMNH; ♂].

**Distribution.** Costa Rica.

***tollas*** Oláh and Johanson, 2012:235 [Type locality: Peru, San Martin Prov., creek crossing rd. Juan Guerra-Chazuta, 14 km (rd.) E Colombia Bridge, 6°35.594’ S, 76°13.172’ W; NRM; ♂].

**Distribution.** Peru.

***tridens*** Mosely, 1933:17 [Type locality: Brazil, Paraná; BMNH; ♂]. —Flint et al., 1987:46 [♂; distribution]. —Blahnik et al., 2004:4 [distribution]. —Paprocki et al., 2004:8 [checklist]. —Dumas et al., 2009:358 [distribution]. —Nessimian and Dumas, 2010:466 [larva; pupa; biology]. —Calor, 2011:320 [checklist]. —Dumas and Nessimian, 2011:9 [checklist]. —Dumas and Nessimian, 2012:12. —Paprocki and França, 2014:26 [checklist]. —Moretto and Bispo, 2015:126 [distrtibution].

**Distribution.** Brazil, Paraguay [?].

***trifidum*** Flint, McAlpine and Ross, 1987:75 [Type locality: Ecuador, Pcia. Napo, Tena; NMNH; ♂]. —Flint, 1996b:413 [distribution]. —Oláh and Johanson, 2012:236 [distribution].

**Distribution.** Ecuador, Peru.

***tripartitum*** Flint, McAlpine and Ross, 1987:64 [Type locality: Colombia, Dpto. Antioquia, Quebrada Honda, 12 km SW Fredonia; NMNH; ♂]. —Muñoz-Quesada, 2000:276 [checklist].

**Distribution.** Colombia, Venezuela.

***trispicatum*** Flint, McAlpine and Ross, 1987:46 [Type locality: Brazil, Edo. Sao Paulo, Municipalidad de Iporanga; MZUSP; ♂]. —Blahnik et al., 2004:4 [distribution]. —Paprocki et al., 2004:8 [checklist]. —Calor, 2011:320 [checklist]. —Paprocki and França, 2014:27 [checklist]. —Moretto and Bispo, 2015:126 [distrtibution].

**Distribution.** Brazil.

***turrialbum*** Flint, McAlpine and Ross, 1987:58 [Type locality: Costa Rica, Pcia. Cartago, Turrialba; NMNH; ♂]. —Holzenthal, 1988c:68 [distribution]. —Muñoz-Quesada, 1999:981 [♂; distribution].

**Distribution.** Costa Rica.

***uncatum*** Mosely, 1933:41 [Type locality: Colombia, Sozonoco; MCZ; ♂]. —Flint et al., 1987:65 [♂; distribution]. —Muñoz-Quesada, 2000:276 [checklist].

**Distribution.** Colombia.

***viridianum*** Navás, 1916a:31 [Type locality: Brazil, Bahia; collection Navás, now lost?; ♀]. —Flint et al., 1987:70 [♂; distribution]. —Flint, 1996b:414 [distribution]. —Oliveira and Froehlich, 1996:757 [biology]. —Muñoz-Quesada, 2000:276 [checklist]. —Blahnik et al., 2004:4 [distribution]. —Paprocki et al., 2004:8 [checklist]. —Dumas et al., 2009:358 [distribution]. —Dumas et al., 2010:8 [distribution]. —Calor, 2011:321 [checklist]. —Dumas and Nessimian, 2012:12 [checklist]. —Barcelos-Silva et al., 2012:1277 [distribution]. —Oláh and Johanson, 2012:236 [distribution]. —Souza et al., 2013a:4 [distribution]. —Costa et al., 2014:220 [distribution]. —Paprocki and França, 2014:27 [checklist].

—*dissimile* Mosely 1933:43 [Type locality: Bolivia, Pcia. Sara; MCZ; ♂]. —Flint, 1974c:101 [♂; distribution]. —Flint, 1978:384 [to synonymy].

**Distribution.** Argentina, Bolivia, Brazil, Colombia, Ecuador, Guyana, Paraguay, Peru, Suriname, Venezuela.

***vitum*** Flint, McAlpine and Ross, 1987:58 [Type locality: Costa Rica, Pcia. Puntarenas, Las Cruces, near San Vito; NMNH; ♂]. —Holzenthal, 1988c:68 [distribution]. —Muñoz-Quesada, 1999:982 [♂; distribution].

**Distribution.** Costa Rica.

***woldianum*** Flint, McAlpine and Ross, 1987:59 [Type locality: Panama, Pcia. Chiriquí, Fortuna dam site; NMNH; ♂]. —McElravy, et al., 1981:153 [as species B]. —Holzenthal, 1988c:68 [distribution]. —Aguila, 1992:541 [distribution]. —Muñoz-Quesada, 1999:982 [♂; distribution]. —Armitage et al., 2015b:5 [checklist]. —Armitage and Cornejo, 2015:193 [checklist].

**Distribution.** Costa Rica, Panama.

#### Genus *Macronema* Pictet [33 + †1]

*Macronema* Pictet, 1836:400 [Type species: *Macronema lineatum* Pictet 1836, by monotypy]. —Ulmer, 1907c:62 [revision]. —Flint, 1978:386 [systematics]. —Flint and Bueno-Soria, 1979:524 [revision]. —Flint and Bueno-Soria, 1982:358 [redefinition]. —Wichard, 2007a:22 [diagnosis].

*Macronemum* Burmeister, 1839:915 [unjustified emendation of *Macronema*, apparently intended to replace *Macronema* Stephens, 1829 (Coleoptera), which was a *nomen nudum* for *Macronema*; see ICZN, 1962:80].

This genus of strikingly colored species is endemic to the Neotropics. Many of the species have the basal half or two-thirds of the forewing covered with emerald green scales, with the apex irrorate, in various manners, with gold, black, silver, or brown scales.

Flint and Bueno-Soria (1982) described the immature stages and retreat of a Mexican species. They construct tubular, silken retreats in tangles of plant roots or trailing plants in flowing water, but apparently without a definitive capture net. Small, cubic pieces of plant mater in the gut suggests that the larvae bite them off the surrounding vegetation. They pupate within a strengthened larval tube, closed by plugs of vegetation.

***amazonense*** Flint, 1978:403 [Type locality: Brazil, Amazonas, Manaus, Reserva Egler, Estrada Amazonas 010, km 64; INPA; ♂]. —Paprocki et al., 2004:8 [checklist]. —Ribeiro et al., 2009:34 [list of types]. —Paprocki and França, 2014:27 [checklist].

**Distribution.** Brazil.

***argentilineatum*** Ulmer, 1905a:77 [Type locality: [Brazil], Par [Pará], Amazonstrom; PAN; ♂]. —Flint, 1966a:6 [♂; holotype; wings]. —Flint, 1974c:109 [♂; distribution]. —Paprocki et al., 2004:8 [checklist]. —Paprocki and França, 2014:27 [checklist].

—*polygramma* Navás, 1927a:42 [Type locality: Brazil, Sao Paulo de Olivensa, Rio Solimoes; DEI; ♂]. —Flint, 1978:392, 402 [to synonymy].

**Distribution.** Brazil, Suriname.

***bicolor*** Ulmer, 1905a:75 [Type locality: Brasilien [Brazil], Santa Catharina [sic]; PAN; ♂]. —Flint, 1966a:6 [mlae/; lectotype; wings]. —Flint and Bueno-Soria, 1982:359 [♂; synonymy; wings]. —Paprocki et al., 2004:8 [checklist]. —Dumas et al., 2010:8 [distribution]. —Calor, 2011:321 [checklist]. —Dumas and Nessimian, 2012:13 [checklist]. —Paprocki and França, 2014:27 [checklist].

—*agnathum* Müller, 1921:530 [Type locality: none given, but presumably, Brazil, Santa Catarina; NMW; ♂]. —Flint and Bueno-Soria, 1982:359 [Neotype: Brazil, Santa Catarina; NMW; ♂; to synonymy].

—*apicale* (Navás), 1927a:40 [Type locality: Brazil, Minas Geraes; DEI; ♂; in *Leptonema*]. —Flint and Bueno-Soria, 1982:359 [to synonymy].

—*chloraemus* Müller, 1921:554 [Type locality: Brazil]. —Ulmer, 1957:340 [to synonymy].

**Distribution.** Brazil.

***bifidum*** Flint, 1974c:113 [Type locality: Suriname, Litani River, near Feti Creek; RNH; ♂]. —Oláh and Johanson, 2012:237 [distribution].

**Distribution.** French Guiana, Suriname.

***burmeisteri*** Banks, 1924:452 [Type locality: Peru, Yurimaguas; MCZ; ♀]. —Flint, 1967c:9 [lectotype]. —Flint, 1978:393, 402 [♂; distribution; wings]. —Flint and Bueno-Soria, 1979:527 [male/; distribution; wings]. —Maes and Flint, 1988:5 [distribution]. —Holzenthal, 1988c:68 [distribution]. —Aguila, 1992:541 [distribution]. —Maes, 1999:1185 [checklist]. —Paprocki et al., 2004:8 [checklist]. —Chamorro-Lacayo et al., 2007:42 [checklist]. —Oláh and Johanson, 2012:237 [checklist]. —Paprocki and França, 2014:27 [checklist]. —Armitage et al., 2015b:5 [checklist]. —Armitage and Cornejo, 2015:193 [checklist].

**Distribution.** Brazil, Costa Rica, Ecuador, Nicaragua, Panama, Peru.

***chalybeoides*** Ulmer, 1951:202 [replacement name for material from Mexico previously called *chalybeum* Hagen]. [Type locality: Mexico, Cuernavacca; NMW; ♂]. —Ulmer, 1905b:83 [as *chalybeum* Hag.; wings]. —Ulmer, 1907c:81 [as *chalybeum* Hag.; wings]. —Bueno-Soria and Flint, 1978:209 [distribution]. —Flint and Bueno-Soria, 1979:534 [♂; lectotype].

**Distribution.** Mexico.

***esterum*** Flint, 1983a:54 [Type locality: Argentina, Pcia. Corrientes, C. Pellegrini; MACN; ♂].

**Distribution.** Argentina.

***exophthalmum*** Flint, 1978:391, 401 [Type locality: Brazil, Edo. Amazonas, Cachoeira do Gigante; NMNH; ♂; wings]. —Paprocki et al., 2004:8 [checklist]. —Ribeiro et al., 2009:34 [list of types]. —Paprocki and França, 2014:27 [checklist].

**Distribution.** Brazil.

***fragile*** Banks, 1914b (1915):631 [Type locality: British Guiana, Bartica; MCZ; ♂; as *fragilis*]. —Flint, 1967c:10 [♂; holotype]. —Flint, 1974c:110 [♂; distribution]. —Flint, 1978:403 [♂; distribution; wings]. —Paprocki et al., 2004:8 [checklist]. —Oláh and Johanson, 2012:237 [distribution]. —Paprocki and França, 2014:28 [checklist].

**Distribution.** Brazil, French Guiana, Guyana, Suriname.

***fraternum*** Banks, 1910:159 [Type locality: Guiana; MCZ; ♀; as *fraterna*]. —Flint, 1967c:10 [♂; wings]. —Flint, 1974c; 114 [♂; distribution]. —Flint, 1996b:411 [distribution]. —Holzenthal, 1988c:68 [distribution]. —Oláh and Johanson, 2012:238 [distribution].

**Distribution.** Costa Rica, Ecuador, French Guiana, Guyana, Peru, Suriname.

***fulvum*** Ulmer, 1905a:79 [Type locality: Brazil, Ilha Grande; ZSZMH; ♂]. —Weidner, 1964:86 [holotype destroyed]. —Paprocki et al., 2004:8 [checklist]. —Dumas et al., 2009:358 [distribution]. —Calor, 2011:321 [checklist]. —Paprocki and França, 2014:28 [checklist].

**Distribution.** Brazil.

***gundlachi*** Banks, 1924:454 [Type locality: Cuba; MCZ; ♂]. —Flint, 1967c:10 [♂; lectotype]. —Flint, 1968b:81 [checklist]. —Botosaneanu, 1979:47 [distribution]. —Botosaneanu, 1994b:466 [probable larva]. —Flint, 1996c:16 [checklist]. —Botosaneanu, 2002:93 [checklist]. —Naranjo López and González Lazo, 2005:150 [checklist].

**Distribution.** Cuba.

***hageni*** Banks, 1924:452 [Type locality: Brazil, Tapajos; MCZ; ♂]. —Flint, 1967c:10 [♂; lectotype, wings]. —Flint, 1974c:112 [♂; distribution]. —Flint, 1978:392 [distribution]. —Flint, 1991:79 [♂; distribution; wings]. —Flint, 1992d:68 [distribution]. —Muñoz-Quesada, 2000:276 [checklist]. —Blahnik et al., 2004:4 [distribution]. —Paprocki et al., 2004:8 [checklist]. —Nogueira and Cabette, 2011:351 [distribution]. —Paprocki and França, 2014:28 [checklist].

**Distribution.** Argentina, Bolivia, Brazil, Colombia, Ecuador, Paraguay, Peru, Suriname, Venezuela.

† ***hispaniola*** Wichard, 2007a:22 [Type locality: Dominican Republic; SMNS; ♂; in amber].

**Distribution.** Domincan Republic.

***immaculatum*** Mosely, 1934a:139 [Type locality: [Brazil], Parana, Castro; BMNH; ♂; as *immaculata*]. —Oliveira and Froehlich, 1996:757 [biology]. —Paprocki et al., 2004:8 [checklist]. —Calor, 2011:321 [checklist]. —Paprocki and França, 2014:28 [checklist]. —Moretto and Bispo, 2015:126 [distrtibution].

**Distribution.** Argentina, Brazil.

***ketleben*** Oláh and Johanson, 2012:238 [Type locality: French Guiana, Approuaguekaw, Kaw Mt, 216 m, 4°33.257’ N, 52°11.920’ W; NRM; ♂].

**Distribution.** French Guiana.

***lachlani*** Banks, 1924:452 [Type locality: Brazil, Teffe; MCZ; ♂]. —Flint, 1967c:10 [♂; holotype; wings]. —Flint, 1978:392 [♂; wings]. —Paprocki et al., 2004:8 [checklist]. —Paprocki and França, 2014:28 [checklist].

**Distribution.** Brazil.

***lineatum*** Pictet, 1836:400 [Type locality: Brazil, Bahia, Caravellas; MHNG; ♂]. —Ulmer, 1907c:67 [wings]. —Botosaneanu and Schmid, 1973:233 [lectotype /male; without abdomen]. —Paprocki et al., 2004:8 [checklist]. —Paprocki and França, 2014:28 [checklist].

**Distribution.** Brazil.

***luteipenne*** Flint and Bueno-Soria, 1979:532 [Type locality: Panama, Canal Zone, Rio Agua Salud, Navy Reserve (pipeline Road); NMNH; ♂]. —Holzenthal, 1988c:68 [distribution]. —Aguila, 1992:541 [distribution]. —Bueno-Soria and Barba-Álvarez, 2011:356 [checklist]. —Armitage et al., 2015b:5 [checklist]. —Armitage and Cornejo, 2015:193 [checklist].

**Distribution.** Costa Rica, Mexico, Panama.

***matthewsi*** Flint, 1964a:39 [Type locality: Puerto Rico, El Yunque, Big Tree Trail; NMNH; ♂]. —Flint, 1968b:81 [checklist]. —Botosaneanu, 2002:93 [checklist].

**Distribution.** Puerto Rico.

***muelleri*** Banks, 1924:453 [Type locality: Brazil, Flores; MCZ; ♂; as *mülleri*]. —Flint, 1967c:11 [♂; lectotype]. —Flint, 1978:393, 402 [♂; distribution; wings]. —Paprocki et al., 2004:8 [checklist]. —Paprocki and França, 2014:28 [checklist].

**Distribution.** Brazil.

***paliferum*** Flint, 1974c:113 [Type locality: Suriname, Apisiké, southern boundary; RNH; ♂].

**Distribution.** Suriname.

***partitum*** Navás, 1932b:63 [Type locality: Brazil, Rio de Janeiro, Barao Homem de Mello; DEI; ♀]. —Paprocki et al., 2004:8 [checklist]. —Dumas et al., 2009:358 [distribution]. —Dumas and Nessimian, 2012:13 [checklist]. —Paprocki and França, 2014:28 [checklist].

**Distribution.** Brazil.

***parvum*** Ulmer, 1905a:73 [Type locality: “Südamerika”; PAN; ♂]. —Flint, 1966a:7 [♂; lectotype; wings]. —Flint, 1974c:112 [♂; distribution]. —Flint, 1978:393, 402 [♂; distribution; wings]. —Paprocki et al., 2004:8 [checklist]. —Paprocki and França, 2014:29 [checklist].

**Distribution.** Brazil, Suriname.

***pennyi*** Flint, 1978:401 [Type locality: Brazil, Amazonas, Manaus, Reserva Ducke; INPA; ♂; wings]. —Paprocki et al., 2004:8 [checklist]. —Ribeiro et al., 2009:34 [list of types]. —Paprocki and França, 2014:29 [checklist].

**Distribution.** Brazil.

***percitans*** Walker, 1860:177 [Type locality: [Brazil], Amazon Region; BMNH; ♂]. —Betten and Mosely, 1940:203 [♂; redescription holotype]. —Flint, 1974c:109 [♂; distribution]. —Flint, 1978:392, 402 [♂; distribution; wings]. —Flint, 1996b:411 [distribution]. —Paprocki et al., 2004:8 [checklist]. —Nogueira and Cabette, 2011:351 [distribution]. —Paprocki and França, 2014:29 [checklist].

**Distribution.** Brazil, Guyana, French Guiana, Peru, Suriname.

***pertyi*** Banks, 1924:451 [Type locality: Brazil, Tapajos; MCZ; ♀]. —Flint, 1967c:11 [holotype; wings]. —Flint, 1978:391, 401 [distribution; wings]. —Flint, 1996b:411 [distribution]. —Paprocki et al., 2004:8 [checklist]. —Nogueira and Cabette, 2011:351 [distribution]. —Paprocki and França, 2014:29 [checklist].

**Distribution.** Brazil, Peru.

***picteli*** Banks, 1915:631 [Type locality: British Guiana, Mallali; MCZ; ♂]. —Ulmer, 1907c:69 [*argentilineatum* (in part), example from Guyana only]. —Ulmer, 1913:395 [*percitans*; misidentification]. —Flint, 1967c:11 [as *picteti*; wings]. —Flint, 1974c:110 [♂; distribution].

**Distribution.** Guyana, Suriname.

***reinburgi*** Navás, 1933b:313 [Type locality: Perou, Iquitos (Haute Amazone); MNHNP; ♂]. —Flint, 1978:391 [/male; holotype; wings].

**Distribution.** Peru.

***rubiginosum*** Guérin-Méneville, 1843:395 [Type locality: Brésil [Brazil]; type depository unknown; ♂?]. —Ulmer, l907c:75 [redescription]. —Paprocki et al., 2004:8 [checklist]. —Paprocki and França, 2014:29 [checklist].

**Distribution.** Brazil.

***studiosorum*** Botosaneanu and Hyslop, 1999:327 [Type locality: Jamaica, St. Elizabeth, Y. S. Falls on Y. S. River; ZMA; ♂; ♀]. —Botosaneanu and Hyslop, 1998:21 [as sp., distribution]. —Botosaneanu, 2002:93 [checklist].

**Distribution.** Jamaica.

***toblet*** Oláh and Johanson, 2012:239 [Type locality: French Guiana, Approuaguekaw, Kaw Mt, 104 mao, 4°33.035’ N, 52°11.661’ W; NRM; ♂].

**Distribution.** French Guiana.

***tremenda*** Botosaneanu, 1980:107 [Type locality: Cuba, Prov. Oriente, Cupeyal, Yateras; ZMUA; ♂]. —Botosaneanu, 1979:47 [*nomen nudum* (name included in checklist); distribution]. —Flint, 1996c:16 [checklist]. —Botosaneanu, 2002:93 [checklist]. —Naranjo López and González Lazo, 2005:150 [checklist].

**Distribution.** Cuba.

***variipenne*** Flint and Bueno-Soria, 1979:528 [Type locality: Mexico, San Luis Potosi near Huichihuayan (Rt.85, km 399, 25 mi N of Tamazunchale); NMNH; ♂]. —Flint and —Bueno-Soria, 1982:362 [larva; pupa; biology]. —Ulmer, 1905a:80 [*fulvum* (in part), examples from Chiriqui only]. —Ulmer, 1907c:74 [*percitans* (in part), examples from Chiriqui only]. —Maes and Flint, 1988:5 [distribution]. —Holzenthal, 1988c:68 [distribution]. —Aguila, 1992:541 [distribution]. —Flint, 1996b:412 [distribution]. —Maes, 1999:1186 [checklist]. —Bueno-Soria et al., 2005:75 [distribution]. —Chamorro-Lacayo et al., 2007:42 [checklist]. —Bueno-Soria and Barba-Álvarez, 2011:356 [checklist]. —Armitage et al., 2015b:5 [checklist]. —Armitage and Cornejo, 2015:193 [checklist].

**Distribution.** Costa Rica, Ecuador, Mexico, Nicaragua, Panama, Peru [?].

#### Genus *Macrostemum* Kolenati [18]

*Macrostemum* Kolenati, 1859:239 [Type species: *Hydropsyche hyalina* Pictet, 1836, subsequent designation of Ulmer, 1957]. —Ulmer, 1907c:62 [revision, under *Macronema*]. —Ross, 1944:114 [characterization of *Macrostemum carolina*, *Macrostemum transversum*, *Macronema zebratum*, as *Macronema*]. —Ulmer, 1957:339 [type species selected: *Hydropsyche hyalina* Pictet]. —Flint and Bueno-Soria, 1982:358 [redefinition; resurrected]. —Nimmo, 1987:173 [characterization]. —França et al., 2013:304 [revision of Neotropical species].

In the Neotropics and elsewhere, this genus is noted for its strikingly patterned adults. The wings have dark marks contrased against light or clear membranous areas. Species occur in North America, Asia, and Africa. The immature stages make complex retreats with very fine-meshed capture nets in or on a solid substrate, especially submerged wood (Flint and Bueno-Soria 1982, Lepneva 1970, Ross 1944, Scott 1983, Wiggins 1996).

***arcuatum*** (Erichson), 1848:586 [Type locality: by inference, “Britisch-Guiana”; MCZ; ♂; as *Macronema arcuata*]. —Ulmer, 1907c:40 [♂; wings; in *Pseudomacronema*]. —Mosely, 1931:170 [distribution; in *Pseudomacronema*]. —Flint, 1974c:106 [♂; distribution]. —Flint, 1978:388, 400 [♂; distribution; wings; as *Macronema*]. —Flint and Bueno-Soria, 1982:358 [to *Macrostemum*]. —Flint, 1996b:412 [distribution]. —Paprocki et al., 2004:8 [checklist]. —Nogueira and Cabette, 2011:351 [distribution]. —França et al., 2013:309 [♂; wings]. —Paprocki and França, 2014:29 [checklist].

**Distribution.** Brazil, Guyana, Peru, Suriname.

***brasiliense*** (Fisher), 1970:242 [*nomen novum* for *Phryganea maculata* Perty, 1833, preoccupied in *Phryganea* by *Phryganea maculata* Donovan, 1813 which is now a synonym of *Hydropsyche instabilis* (Curtis, 1834)]. —França et al., 2013:309 [♂; wings; status]. —Paprocki and França, 2014:29 [checklist].

—*maculatum* (Perty), 1833:129 [Type locality: [Brazil], inter St. Pauli civitatem et Villam riccam; ZSM; ♂; as *Phryganea maculata*]. —Ulmer, 1907a:79 [wings; ♀; as *Macronema*]. —Ulmer, 1913:395 [♂; distribution; as *Macronema*]. —Fischer, 1963:190 [bibliography; as *Macronema*]. —Flint and Bueno-Soria, 1982:358 [to *Macrostemum*]. —Burmeister 1983:273 [type situation; as *Macronema*]. —Burmeister, 1989:259 [lectotype; ♂; as *Macronema*]. —Paprocki et al., 2004:8 [checklist]. —Calor, 2011:321 [checklist]. —Dumas and Nessimian, 2012:13 [checklist]. —Barcelos-Silva et al., 2012:1278 [distribution]. —França et al., 2013:309 [to synonymy].

—*tuberosum* Ulmer, 1905b:82 [Type locality: Brazil, Bahia, Brasilia; NMW; ♂; wings; in *Macronema*]. —Ulmer, 1907a:78 [♂; wings]. —Ulmer, 1907b:165 [distribution]. —Flint, 1966a:7 [lectotype; ♂; wings]. —Ulmer, 1913:408 [distribution]. —Fischer, 1963:199 [bibliography; checklist]. —Burmeister 1983: 273 [to synonymy of *maculatum*].

**Distribution.** Brazil.

***braueri*** (Banks), 1924:454 [Type locality: Brazil, Teffe; MCZ; ♀; wings; in *Macronema*]. —Flint, 1978:390, 401 [♂; distribution; wings]. —Flint and Bueno-Soria, 1982:358 [to *Macrostemum*]. —Paprocki et al., 2004:8 [checklist]. —França et al., 2013:314 [♂; wings]. —Paprocki and França, 2014:30 [checklist].

**Distribution.** Brazil.

***bravoi*** França, Paprocki and Calor, 2013:307 [Type locality: Brazil, Bahia, Barreiras, APA Rio de Janeiro, Cachoeira Acaba Vida, 11°53'S, 45°36'W, el. 705 m; MZUSP; ♂]. —Costa et al., 2014:220 [distribution]. —Paprocki and França, 2014:30 [checklist].

**Distribution.** Brazil.

***digramma*** (McLachlan), 1871:131 [Type locality: Brasilia [Brazil], Minas Gerais; BMNH; ♂; in *Macronema*]. —Ulmer, 1907c:80 [♂; wings]. —Flint and Bueno-Soria, 1982:358 [to *Macrostemum*]. —Paprocki et al., 2004:8 [checklist]. —Dumas et al., 2009:358 [distribution]. —Calor, 2011:321 [checklist]. —Barcelos-Silva et al., 2012:1277 [distribution]. —França et al., 2013:316 [♂; wings]. —Paprocki and França, 2014:30 [checklist].

**Distribution.** Brazil.

***erichsoni*** (Banks), 1920:356 [Type locality: French Guiana, Nouveau Chantier; MCZ; ♀; wings; in *Macronema*]. —Ulmer, 1913:395 [wings; as *Macrostemum hyalinum*, var.]. —Flint, 1967c:11 [holotype; wings]. —Flint, 1974c:108 [♂; distribution]. —Flint, 1978:389, 400 [♂; distribution; wings]. —Flint and Bueno-Soria, 1982:358 [to *Macrostemum*]. —Paprocki et al., 2004:8 [checklist]. —Barcelos-Silva et al., 2012:1277 [distribution]. —Oláh and Johanson, 2012:240 [checklist]. —França et al., 2013:316 [♂; wings]. —Paprocki and França, 2014:30 [checklist].

**Distribution.** Brazil, French Guiana, Guyana, Suriname.

***felker*** Oláh and Johanson, 2012:240 [Type locality: Peru, San Martin Prov., La Catarata de Ahuashiyascu, 6°27.544'S, 76°18.192'W; NRM; ♂].

**Distribution.** Peru.

***hyalinum*** (Pictet), 1836:401 [Type locality: Indes Orientales; type lost (Hollier, 2007);; sex unknown; as *Hydropsyche hyalina*]. —Burmeister, 1839:916 [type locality as Brazil; distribution; in *Macronema*]. —Ulmer 1905b:67 [♂; wings]. —Ulmer, 1907c:75 [♂; wings]. —Flint, 1978:389 [♂; wings]. —Flint and Bueno-Soria, 1982:358 [to *Macrostemum*]. —Flint, 1996b:412 [distribution]. —Marinoni and Almeida, 2000:286 [distribution; biology]. —Muñoz-Quesada, 2000:276 [checklist]. —Paprocki et al., 2004:8 [checklist]. —Hollier, 2007:53 [holotype situation]. —Dumas et al., 2009:358 [distribution]. —Calor, 2011:321 [checklist]. —Nogueira and Cabette, 2011:351 [distribution]. —Dumas and Nessimian, 2012:13 [checklist]. —Barcelos-Silva et al., 2012:1277 [distribution]. —Oláh and Johanson, 2012:240 [checklist]. —França et al., 2013:316 [♂; wings]. —Costa et al., 2014:221 [distribution]. —Paprocki and França, 2014:30 [checklist].

**Distribution.** Brazil, Colombia, Guyana, Peru, Venezuela.

***negrense*** (Flint), 1978:390, 400 [Type locality: Brazil [Edo. Amazonas], Rio Negro; AMNH; ♀; in *Macronema*]. —Flint and Bueno-Soria, 1982:358 [to *Macrostemum*]. —Paprocki et al., 2004:8 [checklist]. —França et al., 2013:323 [♂; wings]. —Paprocki and França, 2014:30 [checklist].

**Distribution.** Brazil.

***nigrum*** França, Paprocki and Calor, 2013:305 [Type locality: Brazil, Bahia, Wenceslau Guimarães, Estação Ecológica Estadual Wenceslau Guimarães, Riacho Serra Grande, cachoeira em cima, 13°35'34.3"S, 39°42'51.8"W, el. 482 m; MZUSP; ♂]. —Paprocki and França, 2014:30 [checklist].

**Distribution.** Brazil.

***par*** (Navás), 1930a:74 [Type locality: Brazil, [São Paulo], Ipiranga; DEI; ♀; in *Macronema*]. —Paprocki et al., 2004:8 [checklist]. —Calor, 2011:321 [checklist]. —França et al., 2013:323 [♂; wings]. —Paprocki and França, 2014:30 [checklist].

**Distribution.** Brazil.

***ramosum*** (Navás), 1916a:28 [Type locality: [Brazil], Nueva Friburgo; collection Navás, now lost?; sex unknown; as *Macronema tuberosum* Ulm. var. *ramosa*]. —Flint and Bueno-Soria, 1982:358 [to *Macrostemum*]. —Paprocki et al., 2004:8 [checklist]. —Dumas et al., 2009:359 [distribution]. —França et al., 2013:323 [♂; wings].

**Distribution.** Brazil.

***santaeritae*** (Ulmer), 1905b:85 [Type locality: Brazil, Rio Preto, zw. Boquerao und Sta. Rita; NMW; ♀; as *Macronema Santae Ritae*]. —Ulmer, 1907b:165 [distribution; as *Macronema santae ritae*]. —Ulmer, 1907c:79 [♀; wings]. —Ulmer, 1913:397, 408, 412 [distribution; as *Macronema Santae Ritae*]. —Flint, 1966a:7 [wings; holotype; as *Macronema santaeritae*]. —Flint, 1978:390, 400 [♂; distribution; wings]. —Flint and Bueno-Soria, 1982:358 [to *Macrostemum*]. —Paprocki et al., 2004:8 [checklist]. —Nogueira and Cabette, 2011:351 [distribution]. —França et al., 2013:325 [♂; wings]. —Costa et al., 2014:221 [distribution]. —Paprocki and França, 2014:31 [checklist].

**Distribution.** Argentina, Brazil.

***subaequale*** (Banks), 1920:355 [Type locality: Argentina, Misiones, Haute Parana, San Ignacio; MCZ; ♂; in *Macronema*]. —Flint, 1967c:11 [♂]. —Flint and Bueno-Soria, 1982:358 [to *Macrostemum*]. —França et al., 2013:327 [♂].

**Distribution.** Argentina.

***surinamense*** (Flint), 1974c:108 [Type locality: Suriname, Coppename River, Bakhuis Mountains, camp III; RNH; ♀; wings; in *Macronema*]. —Flint, 1978:389 [/male; near *surinamense*; distribution; wings]. —Flint and Bueno-Soria, 1982:358 [to *Macrostemum*]. —Paprocki et al., 2004:8 [checklist]. —França et al., 2013:327 [♂]. —Paprocki and França, 2014:31 [checklist].

**Distribution.** Brazil, Suriname.

***trigramma*** (Navás), 1916a:29 [Type locality: [Brazil], Nueva Friburgo; collection Navás, now lost?; ♀; in *Macronema*]. —Flint and Bueno-Soria, 1982:358 [to *Macrostemum*]. —Paprocki et al., 2004:8 [checklist]. —Dumas et al., 2009:359 [distribution]. —França et al., 2013:327 [to *nomen dubium*].

—*pullatum* (Navás), 1932b:64 [Type locality: Brazil, Barao Homem de Mello, Rio de Janeiro; DEI; ♀; in *Macronema*]. —Flint and Bueno-Soria, 1982:369 [to synonymy].

**Distribution.** Brazil.

***triste*** (Navás), 1916a:29 [Type locality: [Brazil], Nueva Friburgo; collection Navás, now lost?; ♀; in *Macronema*]. —Flint and Bueno-Soria, 1982:358 [to *Macrostemum*]. —Paprocki et al., 2004:8 [checklist]. —Dumas et al., 2009:359 [distribution]. —França et al., 2013:329 [to *nomen dubium*].

**Distribution.** Brazil.

***ulmeri*** (Banks), 1913a:237 [Type locality: Rio Negro, Colombia; MCZ; ♂; in *Macronema*]. —Ulmer, 1907c:76 [wings; as *Macrostemum hyalinum*, var.]. —Flint, 1967c:11 [♂; holotype; wings]. —Flint, 1974c:107 [♂; distribution]. —Flint, 1978:388 [♂; wings; distribution]. —Flint and Bueno-Soria, 1982:358 [to *Macrostemum*]. —Holzenthal, 1988c:69 [distribution]. —Flint, 1991:79 [♂; wings; distribution]. —Aguila, 1992:541 [distribution]. —Botosaneanu and Sakal, 1992:203 [distribution; ecology]. —Botosaneanu and Alkins-Koo, 1993:33 [distribution]. —Flint, 1996a:85 [distribution]. —Flint, 1996b:412 [distribution]. —Muñoz-Quesada, 2000:276 [checklist]. —Botosaneanu, 2002:94 [checklist]. —Blahnik et al., 2004:4 [distribution]. —Paprocki et al., 2004:8 [checklist]. —Nogueira and Cabette, 2011:351 [distribution]. —Oláh and Johanson, 2012:242 [distribution]. —França et al., 2013:330 [♂; wings]. —Souza et al., 2013a:4 [distribution]. —Paprocki and França, 2014:31 [checklist]. —Armitage et al., 2015b:5 [checklist]. —Armitage and Cornejo, 2015:194 [checklist].

—*siolii* (Marlier), 1964b:136 [Type locality: unspecified, by inference Brazil, São Paulo de Olivença, Émissaire du source, Igarapé-Jaratuba; IRSNB; ♂; larva; pupa; in *Macronema*]. —Flint, 1978:388 [to synonymy].

**Distribution.** Brazil, Colombia, Costa Rica, Ecuador, Honduras, Panama, Peru, Suriname, Trinidad.

#### Genus † *Palaehydropsyche* Wichard [†1]

† *Palaehydropsyche* Wichard, 1986:189 [Type species: † *Palaehydropsyche fossilis* Wichard, 1986, original designation]. —Geraci et al., 2010:925 [classification].

This fossil genus, containing a single species from Miocene-Oligocene Dominican amber, was included in the Hydropsychinae by Geraci et al (2010). When described it was said to be related to *Calosopsyche* and *Plectropsyche*.

† ***fossilis*** Wichard, 1986:192 [Type locality: Dominican Republic; BMNH; ♂; in amber]. —Flint and Pérez-Gelabert, 1999:38 [checklist]. —Botosaneanu, 2002:93 [checklist; as *Hydropsyche*]. —Pérez-Gelabert, 2008:299 [checklist]. —Geraci et al., 2010:925 [classification].

**Distribution.** Dominican Republic.

#### Genus *Plectromacronema* Ulmer [3]

*Plectromacronema* Ulmer, 1906:63 [Type species: *Plectromacronema comptum* Ulmer, 1906, by monotypy]. —Flint, 1983b:225 [revision, key to Neotropical Macronematinae larvae].

*Podomacronema* Banks, 1920:356 [Type species: *Podomacronema subfuscum* Banks, 1920, original designation]. —Flint, 1967c:11 [to synonymy].

*Plectromacronema*, one of several endemic Neotropical macronematines, contains three species distributed sporadically from southern Mexico to tropical Argentina. Flint (1983b) described the larvae, pupae, and biology of *Plectromacronema lisae* from Costa Rica and Mexico. The larvae are predaceous, inhabiting long sand and silk tubes on the bottoms of streams in gravel riffles. No capture net is built.

***comptum*** Ulmer, 1906:63 [Type locality: Brazilen, (Amazonas), Santarem; BMNH; ♂]; 1907c:41 [wings; head]. —Flint, 1974c:115 [♂; distribution]. —Flint, 1978:395, 403 [distribution]. —Flint, 1983b:226 [♂;, distribution; wings]. —Flint, 1992d:69 [distribution]. —Paprocki et al., 2004:9 [checklist]. —Oláh and Johanson, 2008:242 [distribution]. —Paprocki and França, 2014:31 [checklist].

**Distribution.** Brazil, French Guiana, Guyana, Suriname, Venezuela.

***lisae*** Flint, 1983b:228 [Type locality: Mexico, Edo. Chiapas, rt.185 km 35 [12 km north Arriaga]; NMNH; ♂; larva; pupa]. —Holzenthal, 1988c:69 [distribution]. —Bueno-Soria and Barba-Álvarez, 2011:356 [checklist].

**Distribution.** Costa Rica, Mexico.

***subfuscum*** (Banks), 1920:356 [Type locality: Argentina, Misiones; MCZ; ♂; in *Podomacronema*]. —Flint, 1983b:227 [♂; distribution; wings]. —Paprocki et al., 2004:9 [checklist]. —Paprocki and França, 2014:31 [checklist].

**Distribution.** Argentina, Brazil, Uruguay.

#### Genus *Plectropsyche* Ross [3 + †1]

*Plectropsyche* Ross, 1947:141 [Type species: *Plectrosyche hoogstraali* Ross, 1947, original designation]. —Ross and Unzicker, 1977:308 [revision]. —Schefter, 2005:149 [phylogeny]. —Oláh and Johanson, 2008:178 [synonym of *Cheumatopsyche*]. —Geraci et al., 2010:925 [resurrected; classification]. —Bueno-Soria and Barba-Álvarez, 2015:421 [revision, key to species].

The placement of the species in this taxon has been the subject of recent taxonomic uncertainty. Oláh and Johanson (2008) synonymized the genus with *Cheumatopsyche* and placed the included species in their *Cheumatopsyche pali* species group. Geraci et al. (2010) reinstated the genus and Bueno-Soria and Barba-Álvarez (2015) revised the species and described two new ones. Adults have been collected near small to large rivers at ultraviolet light, and larvae and male metamorphotypes of the Costa Rican species have taken at the same sites, but the larval stages have not yet been described. These immature stages are very similar to those of *Cheumatopsyche* (Flint and Holzenthal, personal observation).

† ***alvarezi*** Wichard, Solórzano-Kraemer and Luer, 2006:41 [Type locality: Mexico, Chiapas, Simojovel de Allende,; IHNEC; ♂; in amber]. —Bueno-Soria and Barba-Álvarez, 2015:430 [distribution].

**Distribution.** Mexico.

***hoogstraali*** Ross, 1947:142 [Type locality: Mexico, Michoacan, La Majada, Apatzingan; INHS; ♂; ♀]. —Ross and Unzicker, 1977:308 [♂]. —Bueno-Soria and Flint, 1978:207 [distribution]. —Holzenthal, 1988c:69 [distribution]. —Bueno-Soria et al., 2007:33 [distribution]. —Oláh and Johanson, 2008:178 [to *Cheumatopsyche*]. —Geraci et al., 2010:925 [returned to *Plectropsyche*]. —Bueno-Soria and Barba-Álvarez, 2015:423 [♂; ♀; distribution].

—*pitella* (Denning), 1968a:18 [Type locality: Mexico, Sinaloa, 20 miles east of Villa Union; CAS; ♂; ♀; in *Cheumatopsyche*]. —Gordon, 1974:143 [to *Plectropsyche*]. —Bueno-Soria et al., 2007:33 [distribution]. —Oláh and Johanson, 2008:178 [to *Cheumatopsyche*]. —Geraci et al., 2010:925 [returned to *Plectropsyche*]. —Bueno-Soria and Barba-Álvarez, 2015:423 [to synonymy].

**Distribution.** Guatemala, Honduras, Mexico, Nicaragua.

***velascoi*** Bueno-Soria and Barba-Álvarez, 2015:426 [Type locality: Mexico, Morelos, San Rafael Vicente Aranda, 15 km SE Tehuixtla; CNIN; ♂; female].

**Distribution.** Mexico.

***wallacei*** Bueno-Soria and Barba-Álvarez, 2015:428 [Type locality: Costa Rica, Limón, Río Barbilla, ca. 8 km W B-Line, 10.067°N, 83.369°W, elev. 30 m; UMSP; ♂; ♀]. —Armitage and Cornejo, 2015:194 [checklist].

**Distribution.** Costa Rica, Panama.

#### Genus *Pseudomacronema* Ulmer [1]

*Pseudomacronema* Ulmer, 1905a:86 [Type species *Pseudomacronema vittatum* Ulmer, 1905a, by monotypy].

This is another monotypic genus of Neotropical Macronematinae, similar in apperance to *Macronema*. A second species at one time placed in the genus, *Macronema arcuatum* Erichson, proved to be a *Macrostemum* lacking a small cross vein. Discovery of the immature stages of *Pseudomacronema vittatum* might prove that it is also a *Macrostemum* or closely related to that genus.

***vittatum*** Ulmer, 1905a:87 [Type locality: Colombien, Bogota; ZSZMH; ♂]. —Ulmer, 1907c:39 [♂; head; wings]. —Weidner, 1964:92 [lectotype]. —Flint, 1978:394, 403 [♂; distribution; wings]. —Muñoz-Quesada, 2000:276 [checklist]. —Cohen, 2004:75 [distribution]. —Paprocki et al., 2004:9 [checklist]. —Paprocki and França, 2014:31 [checklist].

**Distribution.** Argentina, Brazil, Colombia, Paraguay.

#### Genus *Smicridea* McLachlan [230]

*Smicridea* McLachlan, 1871:134 [Type species: *Smicridea fasciatella* McLachlan, 1871, subsequent designation of Milne 1936]. —Flint, 1974b:1 [revision of North and Central American species]. —Flint, 1989:1 [revision of Chilean species]. —Oláh and Johanson, 2012:243 [synopsis of species groups].

*Rhyacophylax* Müller, 1879b:140 [Type species: *Rhyacophylax brasilianus* Ulmer, 1905a, subsequent designation of Fischer 1963]. —Ross, 1947:144 [to synonymy]. —Flint 1974b:4 [as subgenus]

*Pellopsyche* Banks, 1903b:243 [Type species: *Pellopsyche signata* Banks, 1903b, by monotypy]. —Ulmer, 1907d:175 [to synonymy under *Rhyacopsyche*].

*Antarctopsyche* Ulmer, 1907a:30 [Type species: *Antarctopsyche annulicornis* Ulmer (not Blanchard), 1907a, by monotypy]. —Flint, 1974b:4 [to synonymy].

*Badallus* Navás, 1918d:21 [Type species: *Badallus argentinus* Navás, 1918d, original designation]. —Navás, 1920b:42 [treated as synonym].

This genus is ubiquitous across the entire Neotropical region, where it is very diverse and generally abundant. Species are found as far north as the southwestern United States, and over all the Antillean islands. All the species are distributed between two subgenera, *Smicridea* and *Rhyacophylax*, with 130 and 100 species, respectively, from Mexico, Central and South America, and the Caribbean. The species of this genus seem to be filling the niches in the Neotropics occupied by the genera *Hydropsyche* and *Cheumatopsyche* in the rest of the world.

All species are restricted to the vicinity of flowing water, where their immature stages are found. Their larvae and pupae have been described a number of times (Correa et al., 1981, Flint 1974b, 1989, Wiggins 1996, Albino et al. 2011). They construct typical hydropsychid capture nets and retreats, with reported mesh size of 60 x 68 micrometers (Oliveira and Froehlich 1996). These same authors reported gut contents to be largely diatoms, but also containing insect fragments and green algae. Larvae have also been reported from travertine streams and thermal waters (Paprocki et al. 2003, Rueda Martín and Sganga 2011).

***abrupta*** (*Rhyacophylax*) Flint, 1974c:93 [Type locality: Suriname, Coppename River, Raleigh Falls; RNH; ♂]. —Flint, 1978:380 [distribution]. —Flint, 1992d:66 [distribution]. —Paprocki et al., 2004:9 [checklist]. —Nogueira and Cabette, 2011:351 [distribution]. —Paprocki and França, 2014:32 [checklist].

**Distribution.** Brazil, Suriname.

***acuminata*** (*Rhyacophylax*) Flint, 1974b:37 [Type locality: Costa Rica, Cartago, Turrialba; NMNH; ♂; ♀]. —Flint, 1975:572 [distribution]. —Holzenthal, 1988c:69 [distribution]. —Flint, 1991:75 [♂; distribution]. —Flint, 1996b:406 [distribution]. —Muñoz-Quesada, 2000:276 [checklist].

**Distribution.** Colombia, Costa Rica, Peru.

***aequalis*** (*Smicridea*) Banks, 1920:358 [Type locality: British Guiana, Bartica; MCZ; ♂]. —Flint, 1967c:13 [♂; redescription]. —Flint, 1974c:87 [♂; distribution]. —Flint, 1978:376 [distribution]. —Paprocki et al., 2004:10 [checklist]. —Paprocki and França, 2014:37 [checklist].

**Distribution.** Brazil, Guyana, Suriname.

***albifrontalis*** (*Smicridea*) Martynov, 1912:25 [Type locality: Peru, Callanga; ASL; ♂].

**Distribution.** Peru.

***albosignata*** (*Smicridea*) Ulmer, 1907a:34 [Type locality: Brazil, Santos; ZSZMH; ♂]. —Weidner, 1964:97 [lectotype]. —Denning and Sykora, 1968:176 [♂; redescription]. —Marinoni and Almeida, 2000:286 [distribution; biology]. —Blahnik et al., 2004:4 [distribution]. —Paprocki et al., 2004:10 [checklist]. —Dumas et al., 2009:359 [distribution]. —Calor, 2011:321 [checklist]. —Dumas and Nessimian, 2012:14 [distribution]. —Paprocki and França, 2014:37 [checklist].

—*maculata* Banks, 1920:359 [Type locality: Brazil; MCZ; ♂]. —Flint, 1967c:13 [♂; redescription; to synonymy].

**Distribution.** Brazil.

***alticola*** (*Smicridea*) Flint, 1964a:40 [Type locality: Puerto Rico, Toro Negro Forest, Doña Juana Cr. at recreation area; NMNH; ♂; ♀; larva; pupa]. —Flint, 1968b:81 [checklist]. —Botosaneanu, 2002:94 [checklist].

**Distribution.** Puerto Rico.

***amplispina*** (*Smicridea*) Flint, 1981a:22 [Type locality: Venezuela, Aragua, Dos Riitos, 6 km N Rancho Grande; NMNH; ♂].

**Distribution.** Venezuela.

***anaticula*** (*Smicridea*) Flint, 1981a:22 [Type locality: Venezuela, Aragua, Dos Riitos, 6 km N Rancho Grande; NMNH; ♂].

**Distribution.** Venezuela.

***andicola*** (*Rhyacophylax*) Flint, 1991:77 [Type locality: Ecuador, Prov. Pastaza, Puyo; NMNH; ♂]. —Flint, 1996b:406 [distribution]. —Muñoz-Quesada, 2000:277 [checklist].

**Distribution.** Colombia, Ecuador, Peru.

***annulicornis*** (*Smicridea*) (Blanchard), 1851:140 [Type locality: Chile; MNHNP; ♂; in *Hydropscyhe*]. —Flint, 1974e:88 [checklist]. —Flint, 1989:9 [♂; ♀; redescription; distribution]. —Cohen, 2004:75 [distribution]. —Sganga, 2005:142 [distribution]. —Sganga and Fontanarrosa, 2006:3 [larva; pupa; distribution]. —Miserendino and Brand, 2007:312 [biology]. —Brand and Miserendino, 2011a:35 [biology; habitat]. —Brand and Miserendino, 2011b:143 [biology]. Sabando et al., 2011:1 [population genetic structure]. —Brand et al., 2012:90 [biology]. —Oláh and Johanson, 2012:242 [distribution]. —Brand and Miserendino, 2014:6 [community ecology].

—*chilensis* (Navás), 1923b:23 [Type locality Chile, Marga Marga; MZBS; ♀; in *Rhyacophylax*]. —Flint, 1974e:88 [checklist]. —Flint, 1989:9 [to synonymy].

**Distribution.** Argentina, Chile.

***anomala*** (*Smicridea*) Flint and Denning, 1989a:419 [Type locality: Trinidad Island, Simla Research Station; CAS; ♂]. —Botosaneanu and Sakal, 1992:203 [distribution; ecology]. —Botosaneanu and Alkins-Koo, 1993:34 [distribution]. —Flint, 1996a:83 [distribution]. —Botosaneanu, 2002:94 [checklist].

**Distribution.** Tobago, Trinidad, Venezuela.

***anticura*** (*Smicridea*) Flint, 1989:22 [Type locality: Chile, Pcia. Osorno, Parque Nacional Puyehue, Río Anticura; NMNH; ♂; ♀]. —Sganga, 2005:142 [distribution].

**Distribution.** Argentina, Chile.

***appendicula*** (*Smicridea*) Flint, 1974c:85 [Type locality: Suriname, Brownsberg, mountain creek near Golddiggers camp; RNH; ♂].

**Distribution.** Suriname.

***appendiculata*** (*Rhyacophylax*) Flint, 1972b:238 [Type locality: Argentina, Pcia. Santa Fe, Arroyo Saladillo, near Santa Fe; NMNH; ♂]. —Flint, 1978:377 [distribution]. —Blahnik et al., 2004:4 [distribution]. —Cohen, 2004:75 [distribution]. —Paprocki et al., 2004:9 [checklist]. —Sganga, 2005:142 [distribution]. —Albino et al., 2011:21 [♂ phallus, larva; pupa; biology; distribution]. —Nogueira and Cabette, 2011:351 [distribution]. —Paprocki and França, 2014:32 [checklist].

**Distribution.** Argentina, Brazil.

***araguaiense*** (*Rhyacophylax*) Albino, Pes and Hamada, 2011:3 [Type locality: Brazil, Mato Grosso, Nova Xavantina, Campus da Unemat; INPA; ♂]. —Paprocki and França, 2014:32 [checklist].

**Distribution.** Brazil.

***argentina*** (*Rhyacophylax*) (Navás), 1918d:21 [Type locality: Argentina, Santa Fe; MZBS; ♂; as *Badallus argentinus*]. —Navás, 1920b:42 [distribution; to *Rhyacophylax*]. —Schmid 1949a:341 [♂; lectotype, redescription]. —Flint, 1972b:237 [♂; to Smicridea (Rhyacophylax)]. —Flint, 1982c:27 [distribution]. —Sganga, 2005:142 [distribution].

**Distribution.** Argentina, Paraguay [?], Peru[?].

***aries*** (*Smicridea*) Blahnik, 1995:85 [Type locality: Costa Rica, Cartago, Reserva Tapantí, Quebrada Palmitos & falls, 9.72°N, 83.78°W; NMNH; ♂].

**Distribution.** Costa Rica.

***arizonensis*** (*Rhyacophylax*) Flint, 1974b:35 [Type locality: U.S.A., Arizona, Clear Creek Campground SE of Camp Verde; NMNH; ♂; ♀]. —Blinn and Ruiter, 2006:332 [biology]. —Bueno-Soria et al., 2007:33 [distribution]. —Blinn and Ruiter, 2009b:185 [phenology, distribution].

**Distribution.** Mexico, U.S.A.

***astarte*** (*Smicridea*) Malicky, 1980:221 [Type locality: Guadeloupe, Mittelauf des Flusses Lezard bei Chemin de Diane; collection Malicky; ♂]. —Malicky, 1983:264 [distribution]. —Flint and Sykora, 1993:49 [checklist]. —Botosaneanu, 1994a:49 [distribution]. —Blahnik, 1995:93 [♂; ♀; redescription]. —Botosaneanu, 2000:256 [distribution]. —Botosaneanu, 2002:94 [checklist]. —Botosaneanu and Thomas, 2005:56 [checklist].

**Distribution.** Guadeloupe.

***aterrima*** (*Smicridea*) Ulmer, 1911:19 [Type locality: Argentina, [Pcia.] Misiones, Bompland; ZSZMH; ♂]. —Weidner, 1964:97 [lectotype]. —Sganga, 2005:142 [distribution].

**Distribution.** Argentina.

***atmena*** (*Rhyacophylax*) Oláh and Johanson, 2012:244 [Type locality: Peru, Chontachaca, Kosnipata-Cusco, 13°01'25"S, 71°28'03"W, 700 m, humid subtropical forest; NRS; ♂].

**Distribution.** Peru.

***atrobasis*** (*Rhyacophylax*) Flint, 1983a:63 [Type locality: Argentina, Pcia. Entre Ríos, Salto Grande, Río Uruguay; NMNH; ♂]. —Paprocki et al., 2004:9 [checklist]. —Sganga, 2005:142 [distribution]. —Sganga and Angrisano, 2005:132 [♂; distribution]. —Rueda Martín and Sganga, 2011:2225 [♂; distribution]. —Paprocki and França, 2014:32 [checklist]. —Isa Miranda and Rueda Martín, 2014:200 [distribution].

**Distribution.** Argentina, Bolivia, Brazil, Uruguay.

***aurra*** (*Rhyacophylax*) Flint, 1991:71 [Type locality: Colombia, Dpto. Antioquia, Río Aurra, Km 50, E of San Jerónimo; NMNH; ♂]. —Muñoz-Quesada, 2000:277 [checklist].

**Distribution.** Colombia.

***banksi*** (*Smicridea*) Flint, 1967c:13 [replacement name for *Smicridea unicolor* Banks 1938:303, preoccupied by *Diplectrona unicolor* Banks 1901:370]. [Type locality: Haiti, La Visite & vic., La Selle Range; MCZ; ♂]. —Flint, 1967c:13 [♂; redescription; lectotype]. —Flint, 1968b:81 [checklist]. —Botosaneanu, 1996:15 [distribution]. —Flint and Pérez-Gelabert, 1999:38 [checklist]. —Botosaneanu, 2002:94 [checklist]. —Flint and Sykora, 2004:24 [♂; distribution]. —Pérez-Gelabert, 2008:300 [checklist].

**Distribution.** Dominican Republic, Haiti.

***begorba*** (*Rhyacophylax*) Oláh and Johanson, 2012:245 [Type locality: Ecuador, Amasonia [sic], Gareno Lodge, Amasonian Lowland; OPC; ♂].

**Distribution.** Ecuador.

***bicornuta*** (*Rhyacophylax*) Albino, Pes and Hamada, 2011:5 [Type locality: Brazil, Mato Grosso, Nova Xavantina municipality, Córrego da Mata (4th order stretch), S15°01'32", W52°26'29"; INPA; ♂]. —Paprocki and França, 2014:32 [checklist].

**Distribution.** Brazil.

***bidactyla*** (*Rhyacophylax*) Flint and Reyes, 1991:483 [Type locality: Ecuador, Prov. El Oro, 6 km E Pasajes; NMNH; ♂]. — Flint, 1996b:408 [distribution].

**Distribution.** Ecuador, Peru, Venezuela.

***bidentata*** (*Smicridea*) Martynov, 1912:24 [Type locality: Peru, Callanga; ASL; ♂]. —Flint, 1996b:405 [distribution]. —Oláh and Johanson, 2012:246 [♂; distribution].

**Distribution.** Peru.

***bifasciata*** (*Rhyacophylax*) Albino, Pes and Hamada, 2011:7 [Type locality: Brazil, Mato Grosso, Nova Xavantina municipality, Córrego da Mata (2nd order stretch), S14°59'18" W52°27'30"; INPA; ♂]. —Paprocki and França, 2014:32 [checklist].

**Distribution.** Brazil.

***bifida*** (*Rhyacophylax*) Rueda Martín and Sganga, 2011:2216 [Type locality: Argentina, Salta, El Rey National Park, A° La Sala; IML; ♂].

**Distribution.** Argentina.

***bifurcata*** (*Rhyacophylax*) Flint, 1974b:33 [Type locality: Costa Rica, Guanacaste, Río Corobici, Las Canas; NMNH; ♂; ♀]. —Holzenthal, 1988c:69 [distribution].

**Distribution.** Costa Rica, Honduras.

***biserrulata*** (*Rhyacophylax*) Flint, 1991:77 [Type locality: Colombia, Dpto. Antioquia, Quebrada Espadera, 7 km E Medellín [road to Sta. Elena]; NMNH; ♂]. —Muñoz-Quesada, 2000:277 [checklist].

**Distribution.** Colombia, Ecuador.

***bivittata*** (*Smicridea*) (Hagen), 1861:291 [Type locality: Panama; MCZ; ♀; in *Hydropsyche* ?]. —Ross, 1952:33 [lectotype]. —Flint, 1967c:13 [♂]. — Flint, 1974b:16 [♂; ♀; distribution; redescription; larva; pupa]. —Flint, 1974c:90 [♂; distribution]. —Bueno-Soria and Flint, 1978:207 [distribution]. —Flint, 1981a:22 [♂; distribution]. —Maes and Flint, 1988:5 [distribution]. —Holzenthal, 1988c:70 [distribution]. —Flint, 1991:63 [♂; distribution]. Flint and Reyes, 1991:481 [distribution]. —Botosaneanu, 1989b:205 [distribution]. —Aguila, 1992:541 [distribution]. —Botosaneanu and Sakal, 1992:203 [distribution; ecology]. —Botosaneanu and Alkins-Koo, 1993:34 [distribution]. —Blahnik, 1995:88 [♂; ♀; diagnosis; redescription; distribution]. —Flint, 1996a:82 [distribution]. —Maes, 1999:1186 [checklist]. —Muñoz-Quesada, 2000:277 [checklist]. —Botosaneanu, 2002:94 [checklist]. —Botosaneanu and Viloria, 2002:108 [distribution; Isla Margarita]. —Blahnik et al., 2004:4 [distribution]. —Paprocki et al., 2004:10 [checklist]. —Bueno-Soria et al., 2005:75 [distribution]. —Chamorro-Lacayo et al., 2007:42 [checklist]. —Albino et al., 2011:3 [distribution]. —Bueno-Soria and Barba-Álvarez, 2011:356 [checklist]. —Calor, 2011:321 [checklist]. —Oláh and Johanson, 2012:246 [distribution]. —Paprocki and França, 2014:37 [checklist]. —Armitage et al., 2015b:5 [checklist]. —Armitage and Cornejo, 2015:194 [checklist].

—*albata* (Navás), 1924c:75 [Type locality: Costa Rica, La Caja; MNHNP; ♀; in *Wormaldia*]. —Flint, 1974b:16 [to synonymy].

**Distribution.** Brazil, Costa Rica, Ecuador, El Salvador, Guatemala, Honduras, Mexico, Nicaragua, Panama, Peru, Suriname, Tobago, Trinidad, Venezuela.

***brasiliana*** (*Rhyacophylax*) (Ulmer), 1905a:107 [Type locality: Brazil, Santa Catharina [sic]; ZSZMH; ♂; as *Rhyacophylax brasilianus*]. —Weidner, 1964:97 [lectotype]. —Flint, 1966a:7 [invalid lectotype, misidentification]. —Flint, 1972b:238 [discussion of lectotype]. —Paprocki et al., 2004:9 [checklist]. —Paprocki and França, 2014:32 [checklist].

**Distribution.** Brazil.

***breviuncata*** (*Smicridea*) Flint, 1974b:18 [Type locality: Costa Rica, Cartago, Turrialba; NMNH; ♂; ♀]. —Holzenthal, 1988c:70 [distribution]. —Flint and Denning, 1989a:419 [pheromone glands]. —Flint, 1991:64 [♂; distribution]. —Aguila, 1992:541 [distribution]. —Muñoz-Quesada, 2000:277 [checklist]. —Armitage et al., 2015b:5 [checklist]. —Armitage and Cornejo, 2015:194 [checklist].

**Distribution.** Colombia, Costa Rica, Panama.

***brunnescens*** (*Smicridea*) Flint and Sykora, 2004:22 [Type locality: Dominican Republic, [La Vega Province, Río Jimenoa]: Jarabacoa; NMNH; ♂; female]. —Pérez-Gelabert, 2008:300 [checklist].

**Distribution.** Dominican Republic.

***bulara*** (*Smicridea*) Flint and Denning, 1989a:423 [Type locality: Trinidad Island, Simla Research Station; CAS; ♂]. —Botosaneanu and Sakal, 1992:203 [distribution; ecology]. —Flint, 1996a:82 [distribution]. —Botosaneanu, 2002:94 [checklist]. —Bueno-Soria et al., 2005:75 [distribution].

**Distribution.** Mexico, Trinidad.

***bulbosa*** (*Smicridea*) Flint, 1974c:88 [Type locality: Suriname, Moengo; RNH; ♂].

**Distribution.** Suriname.

***caldwelli*** (*Smicridea*) Ross, 1947:145 [Type locality: Mexico, Veracruz, Fortin; INHS; ♂]. —Flint, 1974b:19 [♂; ♀; distribution; redescription]. —Bueno-Soria and Flint, 1978:207 [distribution].

**Distribution.** Mexico.

***caligata*** (*Rhyacophylax*) Flint, 1974c:91 [Type locality: Suriname, Nickerie River, Blanche Marie, falls in creek; RNH; ♂]. —Flint, 1978: 377 [distribution]. —Paprocki et al., 2004:9 [checklist]. —Albino et al., 2011:3 [distribution]. —Paprocki and França, 2014:33 [checklist].

**Distribution.** Brazil, Suriname.

***calopa*** (*Smicridea*) Flint, 1974b:22 [Type locality: Mexico, Veracruz, Río Tacolopán, route 180, km 551; NMNH; ♂; ♀]. —Bueno-Soria and Flint, 1978:207 [distribution]. —Oláh and Johanson, 2012:247 [distribution].

**Distribution.** Mexico.

***campana*** (*Smicridea*) Flint, 1974b:26 [Type locality: Panama, Panama, Cerro Campana; NMNH; ♂]. —Aguila, 1992:541 [distribution]. —Armitage et al., 2015b:5 [checklist]. —Armitage and Cornejo, 2015:194 [checklist].

**Distribution.** Panama.

***cariba*** (*Smicridea*) Flint, 1968b:25 [Type locality: Dominica, Pont Casse, 2.2 miles east; NMNH; ♂; ♀; larva; pupa]. —Flint and Sykora, 1993:49 [checklist]. —Botosaneanu, 2002:94 [checklist, correction of mispelling]. —Bass, 2004:245 [larval drift behavior, voltinism]. —Botosaneanu and Thomas, 2005:56 [checklist].

**Distribution.** Dominica, Guadeloupe.

***cartiensis*** (*Smicridea*) Flint and Denning, 1989a:421 [Type locality: Panama, Intendency of San Blas, Río Cartí Grande, 2 km W. Nusagandi (90°20'N; 78°56'W); NMNH; ♂]. —Aguila, 1992:542 [distribution]. —Armitage et al., 2015b:5 [checklist]. —Armitage and Cornejo, 2015:194 [checklist].

**Distribution.** Panama.

***catherinae*** (*Smicridea*) Blahnik, 1995:94 [Type locality: Costa Rica, Guanacaste, Las Canas, Río Corrobici; NMNH; ♂; ♀]. —Oláh and Johanson, 2012:247 [distribution]. —Armitage et al., 2015b:5 [checklist]. —Armitage and Cornejo, 2015:194 [checklist].

**Distribution.** Costa Rica, El Salvador, Guatemala, Honduras, Mexico, Panama.

***chicoana*** (*Rhyacophylax*) Flint, 1983a:58 [Type locality: Argentina, Pcia. Salta, Chicoana, S. Salta; NMNH; ♂]. —Sganga, 2005:142 [distribution].

**Distribution.** Argentina.

***cholta*** (*Smicridea*) Flint, 1974b:21 [Type locality: Guatemala, Izabal, Matias de Galvez; NMNH; ♂; ♀]. —Chamorro-Lacayo et al., 2007:42 [checklist].

**Distribution.** Guatemala, Nicaragua.

***circinata*** (*Smicridea*) Flint and Denning, 1989a:429 [Type locality: Panama, Bocas del Toro Province, Miramar [9°N; 82°15'W]; UCD; ♂]. —Aguila, 1992:542 [distribution]. —Armitage et al., 2015b:5 [checklist]. —Armitage and Cornejo, 2015:194 [checklist].

**Distribution.** Panama.

***columbiana*** (*Rhyacophylax*) (Ulmer), 1905a:106 [Type locality: Colombia, [Hacienda] Pehlke; ZSZMH; ♂; as *Rhyacophylax columbianus*]. —Ulmer, 1909a:306 [distribution]. —Weidner, 1964:97 [lectotype]. —Flint, 1966a:7 [invalid lectotype, redescription]. —Flint, 1974c:96 [♂; distribution]. —Flint, 1978:398 [distribution]. —Flint, 1992d:66 [distribution]. —Muñoz-Quesada, 2000:277 [checklist]. —Paprocki et al., 2004:9 [checklist]. —Oláh and Johanson, 2012:248 [distribution]. —Paprocki and França, 2014:33 [checklist].

**Distribution.** Argentina, Brazil, Colombia, Suriname, Venezuela [?].

***comma*** (*Smicridea*) Banks, 1924:451 [Type locality: Cuba; MCZ; ♀]. —Flint, 1967c:14 [♂; lectotype, redescription]. —Flint, 1968b:81 [checklist]. —Botosaneanu, 1977:232 [distribution; probably *comma*]. —Botosaneanu, 1979:47 [distribution]. —Kumanski, 1987:12 [♂; ♀; distribution]. —Oláh, 1987:151 [♂; as *Smicridea jamaicensis*, misidentification of *Smicridea comma* according to Botosaneanu, 2002:94]. —Botosaneanu, 1991a:133 [distribution]. —Botosaneanu, 1994b:464 [larva]. —Botosaneanu, 1996:15 [distribution]. —Flint, 1996c:16 [checklist]. —Flint and Pérez-Gelabert, 1999:38 [checklist]. —Botosaneanu, 2002:94 [checklist]. —Flint and Sykora, 2004:23 [distribution]. —López del Castillo et al., 2004:229 [distribution]. —González Lazo et al., 2005:260 [distribution]. —Naranjo López and González Lazo, 2005:150 [checklist]. —López del Castillo et al., 2007:171 [distribution; seasonal abundance]. —Pérez-Gelabert, 2008:300 [checklist].

**Distribution.** Cuba, Dominican Republic, Haiti.

***completa*** (*Smicridea*) Banks, 1941:398 [Type locality: Santo Domingo [now Dominican Republic], Villa Altagracia; MCZ; ♂]. —Flint, 1967c:14 [♂; lectotype, redescription]. —Flint, 1968b:81 [checklist]. —Flint and Pérez-Gelabert, 1999:38 [checklist]. —Botosaneanu, 2002:94 [checklist]. —Flint and Sykora, 2004:24 [♂; distribution]. —Pérez-Gelabert, 2008:300 [checklist].

**Distribution.** Dominican Republic.

***complicatissima*** (*Smicridea*) Flint, 1989:31 [Type locality: Chile, Pcia. Malleco, Parque Nacional Contulmo; NMNH; ♂].

**Distribution.** Chile.

***compostella*** (*Smicridea*) Bueno-Soria, 1986:57 [Type locality: Mexico, Nayarit, Compostela; IBUNAM; ♂].

**Distribution.** Mexico.

***conjuncta*** (*Smicridea*) Flint, 1991:65 [Type locality: Colombia, Dpto. Antioquia, Quebrada La Iguaná, 17 km NW Medellín [road to San Jerónimo]; NMNH; ♂]. —Muñoz-Quesada, 2000:277 [checklist].

**Distribution.** Colombia.

***cornuta*** (*Smicridea*) Flint, 1974c:86 [Type locality: Suriname, Wilhelmina Mountins, trail I km 8, small stony creek; RNH; ♂].

**Distribution.** Suriname.

***coronata*** (*Rhyacophylax*) Flint, 1980b:138 [Type locality: Argentina, Pcia. Cordoba, Villa Anizacate; NMNH; ♂]. —Oliveira and Froehlich, 1996:757 [biology]. —Mangeaud, 1996:154 [distribution]. —Paprocki et al., 2004:9 [checklist]. —Sganga, 2005:142 [distribution]. —Sganga and Angrisano, 2005:133 [♂; ♀; distribution]. —Calor, 2011:321 [checklist]. —Albino et al., 2011:33 [♂; variation, distribution]. —Nogueira and Cabette, 2011:351 [distribution]. —Barcelos-Silva et al., 2012:1278 [distribution]. —Paprocki and França, 2014:33 [checklist]. —Moretto and Bispo, 2015:126 [distrtibution].

**Distribution.** Argentina, Brazil, Paraguay, Uruguay.

***corralita*** (*Smicridea*) Flint and Denning, 1989a:423 [Type locality: Mexico, Chiapas State, Corralito, 7 km W. Abasolo; NMNH; ♂]. —Bueno-Soria and Barba-Álvarez, 2011:356 [checklist].

**Distribution.** Mexico.

***cubana*** (*Smicridea*) Kumanski, 1987:14 [Type locality: Cuba, Province Las Villas, the massive of Guamuaya, Rio Nabujina (right tributary of Agabama) near El Piojillo village; NMSB; ♂]. —Botosaneanu, 1994b:464 [doubtful about distinction between *Smicridea minima* and *Smicridea cubana*]. —Flint, 1996c:16 [checklist]. —Botosaneanu, 2002:94 [checklist]. —Naranjo López and González Lazo, 2005:150 [checklist]. —González Lazo et al., 2005:260 [as c.f. *minima*, distribution]. —López del Castillo et al., 2007:171 [distribution; seasonal abundance].

**Distribution.** Cuba.

***cuna*** (*Smicridea*) Flint, 1974b:23 [Type locality: Panama, Canal Zone, Barro Colorado Island, Shannon Creek; NMNH; ♂; ♀]. —Flint and Denning, 1989a:419 [pheromone glands]. —Aguila, 1992:542 [distribution]. —Armitage et al., 2015b:5 [checklist]. —Armitage and Cornejo, 2015:194 [checklist].

**Distribution.** Panama.

***curvipenis*** (*Smicridea*) Flint, 1991:65 [Type locality: Colombia, Dpto. Antioquia, 12 km NW Medellín [road to San Pedro]; NMNH; ♂]. —Flint, 1996b:404 [distribution]. —Muñoz-Quesada, 2000:277 [checklist].

**Distribution.** Colombia, Ecuador, Peru.

***dampfi*** (*Smicridea*) Flint, 1974b:20 [Type locality: Mexico, Chiapas, Finca Vergel; INHS; ♂]. —Bueno-Soria and Flint, 1978:207 [distribution]. —Bueno-Soria et al., 2005:75 [distribution]. —Bueno-Soria and Barba-Álvarez, 2011:356 [checklist].

**Distribution.** Mexico.

***decora*** (*Smicridea*) (Navás), 1930b:362 [Type locality: Chile, Bio- Bio; MZBS; ♂; in *Rhyacophylax*]. —Flint, 1974e:88 [checklist]. —Flint, 1989:13 [♂; ♀; synonymy, redescription; distribution]. —Sganga, 2005:142 [distribution].

—*annulicornis* (Ulmer), 1907a:30 [Type locality: Chile, Bäder von Longavi, Parral; ZMHU; ♂; in *Antarctopsyche*, secondary homonym of *Smicridea annulicornis* (Blanchard)]. —Flint, 1989:13 [to synonymy].

—*albescens* (Navás), 1932c:118 [Type locality: Chile, Bio-Bio; DEI; ♂; in *Antarctopsyche*]. —Flint, 1974e:88 [checklist]. —Flint, 1989:15 [to synonymy].

**Distribution.** Argentina, Chile.

***dentifera*** (*Rhyacophylax*) Flint, 1983a:63 [Type locality: Argentina, Pcia. Misiones Río Iguazú, Camp Nañdu; NMNH; ♂]. —Marinoni and Almeida, 2000:286 [distribution; biology]. —Blahnik et al., 2004:4 [distribution]. —Paprocki et al., 2004:9 [checklist]. —Sganga, 2005:142 [distribution]. —Sganga and Angrisano, 2005:134 [♂; distribution]. —Angrisano and Sganga, 2007:13 [♂; distribution]. —Calor, 2011:321 [checklist, as *dendifera*, misspelling]. —Paprocki and França, 2014:33 [checklist]. —Moretto and Bispo, 2015:126 [distrtibution].

**Distribution.** Argentina, Brazil, Uruguay.

***dentisserrata*** (*Rhyacophylax*) Albino, Pes and Hamada, 2011:9 [Type locality: Brazil, Mato Grosso, Nova Xavantina municipality, Córrego da Mata (2nd order stretch), S 14°59'18” W 52°27'30”; INPA; ♂]. —Paprocki and França, 2014:33 [checklist].

**Distribution.** Brazil.

***discalis*** (*Rhyacophylax*) Flint, 1972b:237 [Type locality: Argentina, Pcia. Misiones, Puerto Rico; NMNH; ♂]. —Marinoni and Almeida, 2000:286 [distribution; biology]. —Blahnik et al., 2004:4 [distribution]. —Cohen, 2004:75 [distribution]. —Paprocki et al., 2004:9 [checklist]. —Sganga, 2005:142 [distribution]. —Sganga and Angrisano, 2005:134 [♂; distribution]. —Oláh and Johanson, 2012:248 [distribution]. —Paprocki and França, 2014:33 [checklist].

**Distribution.** Argentina, Brazil, Uruguay.

***dispar*** (*Rhyacophylax*) (Banks), 1905:16 [Type locality: United States, Arizona, Tucson; MCZ; ♀; in *Polycentropus*]. —Flint, 1974b:41 [♂; ♀; redescription; distribution; larva; pupa]. —Bueno-Soria and Flint, 1978:207 [distribution]. —Rojas-Ascencio, et al., 2002:377 [distribution]. —Blinn and Ruiter, 2006:332 [ecology]. —Blinn and Ruiter, 2009a:304 [biology]. —Blinn and Ruiter, 2009b:185 [phenology, distribution].

—*utico* Ross, 1947:144 [Type locality: United States, Utah, Colorado River near Moab; INHS; ♂]. —Flint, 1967d:168 [distribution]. —Flint, 1974b:40 [to synonymy].

**Distribution.** Mexico, U.S.A.

***dithyra*** (*Rhyacophylax*) Flint, 1974b:42 [Type locality: Mexico, Veracruz, near Huatusco; NMNH; ♂; ♀]. —Bueno-Soria and Flint, 1978:208 [distribution]. —Sganga and Fontanarrosa, 2006:8 [larva; pupa; distribution]. —Miserendino and Brand, 2007:312 [biology]. —Brand and Miserendino, 2011a:35 [biology; habitat]. —Bueno-Soria and Barba-Álvarez, 2011:356 [checklist]. —Rueda Martín and Sganga, 2011:2225 [♂; distribution]. —Oláh and Johanson, 2012:248 [distribution]. —Isa Miranda and Rueda Martín, 2014:200 [distribution].

**Distribution.** Argentina, Bolivia, Guatemala, Honduras, Mexico.

***dombora*** (*Smicridea*) Oláh and Johanson, 2012:248 [Type locality: French Guiana, Approuaguekaw, Kaw Mt, 104 mao, 4°33.035'N, 52°11.661'W; NRS; ♂].

**Distribution.** French Guiana.

***duarte*** (*Smicridea*) Flint and Sykora, 2004:24 [Type locality: Dominican Republic, [La Vega Province], Río Baiguate, 1-2 km S Jarabacoa, 19°06.9'N, 70°37.0'W, 520 m; NMNH; ♂; female]. —Pérez-Gelabert, 2008:300 [checklist].

**Distribution.** Dominican Republic, Haiti.

***egsera*** (*Smicridea*) Oláh and Johanson, 2012:249 [Type locality: Mexico, State of Veracruz, Tecomaluca, Sierra de agua, N 18°44.977’, W 097°14.576’, 1420 m; NRS; ♂].

**Distribution.** Mexico.

***elisae*** (*Smicridea*) Rueda Martín and Sganga, 2011:2218 [Type locality: Argentina, Salta, Chicoana, Rio Chicoana; IML; ♂].

**Distribution.** Argentina.

***ephippifer*** (*Rhyacophylax*) Flint, 1978:379 [Type locality: Brazil [Edo. Pará], Rio Paru, Malloca Apicó; NMNH; ♂]. —Paprocki et al., 2004:9 [checklist]. —Albino et al., 2011:3 [distribution]. —Nogueira and Cabette, 2011:351 [distribution]. —Oláh and Johanson, 2012:250 [distribution]. —Paprocki and França, 2014:33 [checklist].

**Distribution.** Brazil, French Guiana.

***erda*** (*Smicridea*) Oláh and Johanson, 2012:251 [Type locality: French Guiana, Approuaguekaw, Kaw Mt, 104 mao, 4°33.035'N, 52°11.661'W; NRS; ♂].

**Distribution.** French Guiana.

***erecta*** (*Smicridea*) Flint, 1974c:87 [Type locality: Suriname, Nassau Mountains, km 11.3, creek; RNH; ♂]. —Oláh and Johanson, 2012:252 [distribution].

**Distribution.** French Guiana, Suriname.

***fasciatella*** (*Smicridea*) McLachlan, 1871:136 [Type locality: United States, Texas; BMNH; ♂; ♀]. —Kimmins and Denning, 1951:116 [♂; ♀; lectotype]. —Flint, 1967d:168 [distribution]. —Flint, 1974b:11 [♂; ♀; distribution; redescription; larva; pupa]. —Bueno-Soria and Flint, 1978:207 [distribution]. —Blahnik, 1995:96 [♂; ♀; diagnosis; redescription; distribution]. —Baumgardner and Bowles, 2005:11 [distribution]. —Blinn and Ruiter, 2006:332 [ecology]. —Bowles et al., 2007:22 [distribution; biology]. —Blinn and Ruiter, 2009a:305 [biology]. —Blinn and Ruiter, 2009b:185 [phenology, distribution].

—*divisa* (Banks), 1903a:244 [Type locality: United States, Arizona, Salt River; MCZ; ♂; in *Hydropsyche*]. —Milne, 1936:73 [to synonymy].

**Distribution.** Costa Rica, El Salvador, Guatemala, Mexico, U.S.A.

***felsa*** (*Rhyacophylax*) Oláh and Johanson, 2012:252 [Type locality: Ecuador, Amasonian [sic] Lowland, Gareno, near Puerto Napo, 400 m; OPC; ♂].

**Distribution.** Ecuador.

***figueroai*** (*Smicridea*) Holzenthal, 2004:115 [Type locality: Chile, VIII Región del Bío-Bío, Bío-Bío, small trib. to Río Queco, 5 km E Ralco, 37°51.619'S, 71°36.257'W, el. 500 m; UMSP; ♂].

**Distribution.** Chile.

***filicata*** (*Smicridea*) Flint and Denning, 1989a:426 [Type locality: Costa Rica, Puntarenas Province, Rio Singri (9.05`N; 83.082°W), ca. 2 km (air) S. Finca Helechales; NMNH; ♂]. —McElravy et al., 1981:153 [as *Smicridea* B]. —Aguila, 1992:542 [distribution]. —Armitage et al., 2015b:5 [checklist]. —Armitage and Cornejo, 2015:194 [checklist].

**Distribution.** Costa Rica, Panama.

***flinti*** (*Rhyacophylax*) Albino, Pes and Hamada, 2011:11 [Type locality: Brazil, Amazonas, Presidente Figueiredo municipality, Balneário Sossego da Pantera, Igarapé da Onça; S 02°00'52” W 60°01'43”; INPA; ♂]. —Paprocki and França, 2014:34 [checklist].

**Distribution.** Brazil.

***florecita*** (*Smicridea*) Bueno-Soria, 2010:29 [Type locality: Mexico, Chiapas, Cascada “Cerro Las Flores,” 17°21'39"N, 93°37'29"W, el. 540 m; IBUNAM; ♂; female].

**Distribution.** Mexico.

***fogasa*** (*Rhyacophylax*) Oláh and Johanson, 2012:253 [Type locality: Ecuador, Wild Sumaco, near Pacto Sumaco; OPC; ♂].

**Distribution.** Ecuador.

***forcipata*** (*Rhyacophylax*) Flint, 1983a:61 [Type locality: Argentina, Pcia. Misiones, Arroyo Piray Mini, Rt.17 W Dos Hermanas; NMNH; ♂]. —Blahnik et al., 2004:4 [distribution]. —Paprocki et al., 2004:9 [checklist]. —Sganga, 2005:142 [distribution]. —Paprocki and França, 2014:34 [checklist].

**Distribution.** Argentina, Brazil.

***franciscana*** (*Smicridea*) Rocha, Dumas and Nessimian, 2016b:426 [Type locality: Brazil, Minas Gerais, São Roque de Minas, Parque Nacional da Serra da Canastra, afluente do Ribeirão das Posses (Córrego dos Pombos), 20°14'56.6"S, 46°38'04.9"W, el. 997 m; DZRJ; ♂].

**Distribution.** Brazil.

***frequens*** (*Smicridea*) (Navás), 1930b:362 [Type locality: [Chile], Talca; MZBS; ♂; ♀; in *Rhyacophylax*]. —Schmid, 1949a:347 [♂; to *Smicridea*, redescription]. —Flint, 1974e:88 [checklist]. —Flint, 1989:23 [♂; ♀; lectotype, distribution; redescription]. —Sganga, 2005:142 [distribution]. —Sganga and Fontanarrosa, 2006:4 [larva; pupa; distribution]. —Miserendino and Brand, 2007:312 [biology]. —Brand and Miserendino, 2011a:35 [biology; habitat]. —Brand and Miserendino, 2011b:143 [biology]. —Brand et al., 2012:90 [biology]. —Oláh and Johanson, 2012:254 [distribution]. —Brand and Miserendino, 2014:6 [community ecology].

**Distribution.** Argentina, Chile.

***froehlichi*** (*Rhyacophylax*) Almeida and Flint, 2002:768 [Type locality: Brazil, Rio de Janeiro, Km 17, 18 km S. of Teresópolis, 1180 m; MZUSP; ♂]. —Paprocki et al., 2004:9 [checklist]. —Dumas et al., 2009:359 [distribution]. —Dumas et al., 2010:8 [distribution]. —Calor, 2011:321 [checklist]. —Dumas and Nessimian, 2012:14 [distribution]. —Paprocki and França, 2014:34 [checklist]. —Moretto and Bispo, 2015:126 [distrtibution].

**Distribution.** Brazil.

***furesa*** (*Rhyacophylax*) Oláh and Johanson, 2012:254 [Type locality: Ecuador, Archidona, 1100 m; OPC; ♂].

**Distribution.** Ecuador, Peru.

***fuscifurca*** (*Smicridea*) Botosaneanu, in Botosaneanu and Hyslop, 1998:21 [Type locality: Jamaica, St. Elizabeth, Y.S. Falls on Y.S. River; ZMUA; ♂].

**Distribution.** Jamaica.

***gemina*** (*Smicridea*) Blahnik, 1995:90 [Type locality: Costa Rica, Alahuela, Reserva Forestal San Ramón, Río San Lorencito and tribs., 10.216°N, 84.607°W; NMNH; ♂; ♀]. —Muñoz-Quesada, 2000:277 [distribution]. —Chamorro-Lacayo et al., 2007:42 [checklist]. —Dumas et al., 2009:359 [distribution]. —Dumas and Nessimian, 2012:14 [distribution]. —Barcelos-Silva et al., 2012:1278 [distribution]. —Paprocki and França, 2014:37 [checklist]. —Armitage et al., 2015b:5 [checklist]. —Armitage and Cornejo, 2015:194 [checklist].

**Distribution.** Brazil, Colombia, Costa Rica, Ecuador, Nicaragua, Panama.

***gladiator*** (*Rhyacophylax*) Flint, 1978:379 [Type locality: Brazil [Edo. Amazonas], Rio Marauia, Igarape S. Antonio (Cachoeira); NMNH; ♂]. —Paprocki et al., 2004:9 [checklist]. —Ribeiro et al., 2009:34 [list of types]. —Albino et al., 2011:3 [distribution]. —Paprocki and França, 2014:34 [checklist].

**Distribution.** Brazil.

***gomezi*** (*Smicridea*) Blahnik, 1995:86 [Type locality: Costa Rica, Puntarenas, Jardín Botanico R & C Wilson, trib. along Sendero del Agua, 8.80°N, 83.95°W; NMNH; ♂].

**Distribution.** Costa Rica.

***gomphotheria*** (*Smicridea*) Blahnik, 1995:87 [Type locality: Costa Rica, Guanacaste, Parque Nacional Guanacaste, Estación Pitilla, Río Orosí, 10.991°N, 85.428°W; NMNH; ♂]. —Chamorro-Lacayo et al., 2007:42 [checklist].

**Distribution.** Costa Rica, Nicaragua.

***grandis*** (*Smicridea*) Flint, 1968a:27 [Type locality: Jamaica, St. Andrew, Hope River near Newcastle at mile post 16.5; NMNH; ♂; ♀; larva; pupa]. —Flint, 1968b:81 [checklist]. —Botosaneanu and Hyslop, 1998:19 [distribution]. —Botosaneanu, 2002:94 [checklist].

**Distribution.** Jamaica.

***grandisaccata*** (*Smicridea*) Flint, 1991:67 [Type locality: Colombia, Dpto. Antioquia, Quebrada Agua Mala, 35 km NW Medellín [road to San Jerónimo]; NMNH; ♂]. —Muñoz-Quesada, 2000:277 [checklist].

**Distribution.** Colombia.

***grenadensis*** (*Smicridea*) Flint, 1968b:28 [Type locality: Grenada, 2 miles west Lake Grand Etan♂; NMNH; ♂; ♀]. —Flint and Sykora, 1993:53 [distribution]. —Botosaneanu, 2002:94 [checklist].

**Distribution.** Grenada.

***hajla*** (*Rhyacophylax*) Oláh and Johanson, 2012:256 [Type locality: Ecuador, Amasonian [sic] Lowland, Gareno, near Puerto Napo; OPC; ♂].

**Distribution.** Ecuador.

***haraga*** (*Smicridea*) Oláh and Johanson, 2012:257 [Type locality: Peru, San Martin Prov., La Catarata de Ahuashiyascu, 6°27.544'S, 76°18.192'W; NRS; ♂].

**Distribution.** Peru.

***helenae*** (*Rhyacophylax*) Albino, Pes and Hamada, 2011:13 [Type locality: Brazil, Roraima, Caracaraí municipality, Rio Branco, Cachoeira do Bem Querer, N01°55'42" W61°00'09"; INPA; ♂; larva; pupa; biology]. —Paprocki and França, 2014:34 [checklist].

**Distribution.** Brazil.

***holzenthali*** (*Smicridea*) Flint and Denning, 1989a:431 [Type locality: Costa Rica, Guanacaste Province, Rio Tizate (10.773°N; 85.449°W), 7.2 km NE Canas Dulces; NMNH; ♂].

**Distribution.** Costa Rica.

***homora*** (*Rhyacophylax*) Oláh and Johanson, 2012:258 [Type locality: Ecuador, Amasonian [sic] Lowland, Gareno, near Puerto Napo, 400 m; OPC; ♂].

**Distribution.** Ecuador, Peru.

***horga*** (*Smicridea*) Oláh and Johanson, 2012:259 [Type locality: Ecuador, Tinalandia Nature Reserve, West Andean Slope, 600 m, 150 km near Quito; OPC; ♂].

**Distribution.** Ecuador.

***hybrida*** (*Smicridea*) Blahnik, 1995:98 [Type locality: Costa Rica, Puntarenas, Reserva Biológica Carara, Río Carara, 4.3 km (rd) E Costanera Sur, 9.810°N, 84.572°W; NMNH; ♂; ♀]. —Maes, 1999:1186 [checklist]. —Chamorro-Lacayo et al., 2007:42 [checklist]. —Armitage et al., 2015b:5 [checklist]. —Armitage and Cornejo, 2015:194 [checklist].

**Distribution.** Costa Rica, Guatemala, Honduras, Nicaragua, Panama.

***iguazu*** (*Rhyacophylax*) Flint, 1983a:60 [Type locality: Argentina, Pcia. Misiones, Río Iguazú, Camp Nañdu; NMNH; ♂]. —Marinoni and Almeida, 2000:286 [distribution; biology]. —Blahnik et al., 2004:4 [distribution]. —Paprocki et al., 2004:9 [checklist]. —Sganga, 2005:142 [distribution]. —Dumas et al., 2009:359 [distribution]. —Dumas and Nessimian, 2012:25 [distribution]. —Barcelos-Silva et al., 2012:1278 [distribution]. —Paprocki and França, 2014:34 [checklist]. —Moretto and Bispo, 2015:126 [distrtibution].

**Distribution.** Argentina, Brazil.

***inaequispina*** (*Smicridea*) Flint, 1974c:83 [Type locality: Suriname, Nassau Mountains, trail km 12.5, small creek in forest; RNH; ♂]. — Oláh and Johanson, 2012:260 [distribution].

**Distribution.** French Guiana, Suriname.

***inarmata*** (*Rhyacophylax*) Flint, 1974b:34 [Type locality: Mexico, Chiapas, Finca Esperanza; INHS; ♂]. —Bueno-Soria and Flint, 1978:208 [distribution]. —Maes, 1999:1186 [checklist]. —Chamorro-Lacayo et al., 2007:42 [checklist]. —Bueno-Soria and Barba-Álvarez, 2011:356 [checklist].

**Distribution.** Mexico, Nicaragua.

***jamaicensis*** (*Smicridea*) Flint, 1968a:28 [Type locality: Jamaica, St. Andrew, Chestervale, Yallahs River; NMNH; ♂; ♀; larva; pupa]. —Flint, 1968b:81 [checklist]. —Oláh, 1987:151 [♂; misidentification of *Smicridea coma* according to Botosaneanu, 2002:94]. —Botosaneanu and Hyslop, 1998:19 [distribution]. —Malicky, 1999:116, 117 [distribution]. —Botosaneanu, 2002:94 [checklist].

**Distribution.** Jamaica.

***jundiai*** (*Rhyacophylax*) Almeida and Flint, 2002:769 [Type locality: Brazil, Espirito Santo, 15 km SE. of Santa Teresa, Fazenda Santa Clara, 460 m; MZUSP; ♂]. —Paprocki et al., 2004:9 [checklist]. —Dumas et al., 2009:360 [distribution]. —Dumas et al., 2010:8 [distribution]. —Calor, 2011:321 [checklist]. —Dumas and Nessimian, 2012:14 [distribution]. —Barcelos-Silva et al., 2012:1278 [distribution]. —Paprocki and França, 2014:34 [checklist]. —Moretto and Bispo, 2015:126 [distrtibution].

**Distribution.** Brazil.

***kampoka*** (*Rhyacophylax*) Oláh and Johanson, 2012:261 [Type locality: Peru, San Martin Prov., creek crossing rd. Tarapoto-Yurimaguas, ca. 30 km (rd.) NE Tarapoto, 6°24.904'S, 76°18.756'W; NRS; ♂].

**Distribution.** Peru.

***kana*** (*Smicridea*) Oláh and Johanson, 2012:262 [Type locality: Peru, San Martin Prov., La Catarata de Ahuashiyascu, 6°27.544'S, 76°18.192'W; NRS; ♂].

**Distribution.** Peru.

***kapara*** (*Rhyacophylax*) Oláh and Johanson, 2012:263 [Type locality: Peru, Chontachaca, Kosnipata-Cusco, 13°01'25"S, 71°28'03"W, 700 m, humid subtropical forest; NRS; ♂].

**Distribution.** Peru.

***karukerae*** (*Smicridea*) Botosaneanu, 1994a:49 [Type locality: Guadeloupe, ruisseau sur le versant S de la Soufrière à l’aire de pique-nique de Beausoleil; ZMUA; ♂; ♀]. —Botosaneanu, 2000:259 [redescription; ♀]. —Botosaneanu, 2002:94 [checklist]. —Botosaneanu and Thomas, 2005:56 [checklist].

**Distribution.** Guadeloupe.

***kovera*** (*Smicridea*) Oláh and Johanson, 2012:264 [Type locality: Argentina, Jujuy, RN, 9, nr. Leon, 1693 m, 24°01.9565'S, 65°26.519'W; NRS; ♂].

**Distribution.** Argentina.

***lacanha*** (*Smicridea*) Bueno-Soria and Hamilton, 1986:304 [Type locality: Mexico, Chiapas, Río Lacanhá; IBUNAM; ♂]. —Blahnik, 1995:99 [♂; ♀; redescription; distribution; as *lacanja*, misspelling; ♂]. —Bueno-Soria and Barba-Álvarez, 2011:356 [checklist].

**Distribution.** Guatemala, Mexico.

***latipala*** (*Smicridea*) Flint and Denning, 1989a:431 [Type locality: Panama, Chiriqui Province, Guadalupe Arriba (8°52'26"N; 82°33'13"W); NMNH; ♂]. —Aguila, 1992:542 [distribution]. —Armitage et al., 2015b:5 [checklist]. —Armitage and Cornejo, 2015:194 [checklist].

**Distribution.** Panama.

***lebena*** (*Rhyacophylax*) Oláh and Johanson, 2012:265 [Type locality: Peru, Amazonas Prov., river crossing Olmos-Tarapoto rd., 371 km (rd.) E Olmos Desv. Jaén, 5°41.178'S, 77°46.421'W; NRS; ♂].

**Distribution.** Peru.

***legezoa*** (*Rhyacophylax*) Oláh and Johanson, 2012:267 [Type locality: Peru, San Martin Prov., Rio Negro, 37 km (rd.) W Moyobamba, near Olmos-Tarapoto rd., 6°00.278'S, 77°15.437'W; NRS; ♂].

**Distribution.** Peru.

***leloga*** (*Rhyacophylax*) Oláh and Johanson, 2012:268 [Type locality: Peru, San Martin Prov., Rio Mayo, 11 km (rd.) E Mayobamba, 6°04.989'S, 76°53.065'W; NRS; ♂].

**Distribution.** Peru.

***lemeza*** (*Smicridea*) Oláh and Johanson, 2012:269 [Type locality: Peru, San Martin Prov., stream crossing Juan Guerra-Chazuta rd., 10 km (rd.) W Chazuta, 6°37.157'S, 76°10.905'W; NRS; ♂].

**Distribution.** Peru.

***lobata*** (*Rhyacophylax*) (Ulmer), 1909a:305 [Type locality: Venezuela, Las Trinchéras; UZMC; ♂; as *Rhyacophylax lobatus*]. —Flint, 1974c:95 [♂; distribution; figures are of *pseudolobata*]. —Flint, 1978:377 [discussion of type]. —Rázuri-Gonzales and Holzenthal, 2016:21 [compared to *Smicridea signata*; ♂; distribution; synonymy].

—*islamarga* (*Rhyacophylax*) (Botosaneanu), in Botosaneanu and Viloria, 2002:108 [Type locality: Venezuela, Isla de Margarita, Rio San Juan at Fuentidueño; ZMUA; ♂; in *Leptonema*]. —Rázuri-Gonzales and Holzenthal, 2016:29 [to *Smicridea*, to synonymy].

—*repula* (*Rhyacophylax*) Oláh and Johanson, 2012:278 [Type locality: Mexico, State of Veracruz, Los Tuxtlas area, Rio la Palma, near to the Estacion the Biologia Los Tuxtlas, N 18°33.680’, W 095°02.943’, 30 mao; NRS; ♂]. —Rázuri-Gonzales and Holzenthal, 2016:29 [to synonymy].

**Distribution.** Costa Rica, El Salvador, Guatemala, Honduras, Mexico, Nicaragua, Panama, Venezuela.

***lourditae*** (*Smicridea*) Pauls, Blahnik and Holzenthal, in Pauls et al., 2010:1066 [Type locality: Chile, Region X - Los Lagos, Monumento Nacional Alerce Costero, unnamed tributary on trail to Alerce Milenario, 895 m, 40°11.874'S, 73°26.217'W; UMSP; ♂].

**Distribution.** Chile.

***magdalenae*** (*Rhyacophylax*) Flint, 1991:73 [Type locality: Colombia, Dpto. Tolima, Armero, near Guyabal; NMNH; ♂]. —Muñoz-Quesada, 2000:277 [checklist].

**Distribution.** Colombia.

***magnipinnata*** (*Rhyacophylax*) Flint, 1991:71 [Type locality: Colombia, Dpto. Antioquia, 10 km E Medellín [road to Las Palmas]; NMNH; ♂]. —Muñoz-Quesada, 2000:277 [checklist].

**Distribution.** Colombia.

***mangaratiba*** (*Rhyacophylax*) Almeida and Flint, 2002:770 [Type locality: Brazil, Rio de Janeiro, Mangaratiba, 150 m; MZUSP; ♂]. —Paprocki et al., 2004:9 [checklist]. —Dumas et al., 2009:360 [distribution]. —Paprocki and França, 2014:34 [checklist].

**Distribution.** Brazil.

***manzanara*** (*Smicridea*) Flint, 1989:16 [Type locality: Chile, Pcia. Malleco, Ro Manzanares [~10 km W Purén]; NMNH; ♂; ♀].

**Distribution.** Chile.

***marlieri*** (*Rhyacophylax*) Flint, 1978:378 [Type locality: Brazil, Para, Santarem (FAO); IRSNB; ♂]. —Paprocki et al., 2004:9 [checklist]. —Paprocki and França, 2014:35 [checklist].

**Distribution.** Brazil, Venezuela.

***martinica*** (*Smicridea*) Botosaneanu and Thomas, 2005:47 [Type locality: Martinique, premier affluent (sans nom) en rive gauche de la Riv. Blanch, sur la route en amont d’Alma, alt 490 m [14°42'N, 61°06'W]; ZMUA; ♂].

**Distribution.** Martinique.

***marua*** (*Rhyacophylax*) Flint, 1978:380 [Type locality: Brazil [Edo. Amazonas], Rio Marauia, Endstation vor langer Cachoeira, Fluss tritt hier aus dem Geberg mit starkem gefälle ber Granitblöcken; NMNH; ♂]. —Paprocki et al., 2004:9 [checklist]. —Paprocki and França, 2014:35 [checklist].

**Distribution.** Brazil.

***matagalpa*** (*Smicridea*) Flint, 1974b:24 [Type locality: Nicaragua, Matagalpa, 5.3 miles E of Matagalpa; NMNH; ♂; ♀]. —Maes and Flint, 1988:5 [distribution]. —Holzenthal, 1988c:70 [distribution]. —Aguila, 1992:542 [distribution]. —Maes, 1999:1187 [checklist]. —Chamorro-Lacayo et al., 2007:42 [checklist]. —Armitage et al., 2015b:5 [checklist]. —Armitage and Cornejo, 2015:194 [checklist].

**Distribution.** Costa Rica, Honduras, Nicaragua, Panama.

***matancilla*** (*Smicridea*) Flint, 1989:32 [Type locality: Chile, Pcia. Cachapoal, Cerro La Matancilla, Cordillera Coastal; NMNH; ♂].

**Distribution.** Chile.

***medena*** (*Rhyacophylax*) Oláh and Johanson, 2012:270 [Type locality: Colombia, Otun Quinbaya national Park, La Suiza, 1900 m; OPC; ♂].

**Distribution.** Colombia, Peru.

***meridensis*** (*Smicridea*) Botosaneanu and Flint, 1982:14 [Type locality: Venezuela, Edo. Mérida, 11 km southeast of Apartaderos; NMNH; ♂; ♀; larva; pupa].

**Distribution.** Venezuela.

***mesembrina*** (*Rhyacophylax*) (Navás), 1918a:502 [Type locality: Argentina, Provincia de Buenos Aires; UNLP; ♂; in *Rhyacophylax*]. —Schmid, 1949a:343 [♂; redescription]. —Flint, 1982c:28 [to Smicridea (Rhyacophylax), ♂; distribution]. —Mangeaud, 1996:154 [distribution]. —Cohen, 2004:75 [distribution]. —Sganga, 2005:142 [distribution]. —Sganga and Angrisano, 2005:134 [♂; distribution]. —Angrisano and Sganga, 2007:13 [♂; distribution]. —Albino et al., 2011:3 [distribution]. —Nogueira and Cabette, 2011:351 [distribution]. —Paprocki and França, 2014:35 [checklist]. —Manzo et al., 2014:166 [distribution]. —Isa Miranda and Rueda Martín, 2014:200 [distribution].

—*nivosa* (Navás), 1920b:65 [Type locality: Argentina, La Plata; MZBS; ♂; as *Rhyacophylax nivosus*]. —Schmid, 1949a:344 [to synonymy].

**Distribution.** Argentina, Bolivia, Brazil, Uruguay.

***microsaccata*** (*Smicridea*) Flint, 1991:67 [Type locality: Colombia, Dpto. Antioquia, Urrao; NMNH; ♂]. —Muñoz-Quesada, 2000:277 [checklist].

**Distribution.** Colombia.

***mincana*** (*Smicridea*) Oláh and Johanson, 2012:271 [Type locality: Colombia, Minca, 650 m; OPC; ♂].

**Distribution.** Colombia.

***minima*** (*Smicridea*) Flint, 1968a:27 [Type locality: Jamaica, St. Andrew, Hardwar Gap, Dicks Pond Trail; NMNH; ♂; ♀; larva; pupa]. —Flint, 1968b:81 [checklist]. —Botosaneanu, 2002:94 [checklist, doubtful about distinction between *Smicridea minima* and *Smicridea cubana*].

**Distribution.** Jamaica.

***minuscula*** (*Rhyacophylax*) Flint, 1973b:239 [replacement name for Smicridea (Rhyacophylax) minima Flint, 1972b:241, preoccupied by Smicridea (Smicridea) minima Flint 1968a:27]. [Type locality: Argentina, Prov. Misiones, Puerto Rico; NMNH; ♂]. —Sganga, 2005:142 [distribution].

**Distribution.** Argentina, Paraguay.

***mirama*** (*Smicridea*) Flint and Denning, 1989a:426 [Type locality: Panama, Bocas del Toro Province, Miramar [9°N; 82°15'W]; UCD; ♂]. —Aguila, 1992:542 [distribution]. —Maes, 1999:1187 [checklist]. —Chamorro-Lacayo et al., 2007:42 [checklist]. —Armitage et al., 2015b:5 [checklist]. —Armitage and Cornejo, 2015:194 [checklist].

**Distribution.** Nicaragua, Panama.

***mirnae*** (*Smicridea*) Almeida and Flint, 2002:774 [Type locality: Brazil, Parana, Jundiaí do Sul, Fazenda Monte Verde, 23°26'S, 50°16'W, 500 m; DZUP; ♂]. —Paprocki et al., 2004:10 [checklist]. —Paprocki and França, 2014:37 [checklist].

**Distribution.** Brazil.

***mucronata*** (*Smicridea*) Flint, 1989:26 [Type locality: Chile, Pcia. Chiloé, Dalcahue; NMNH; ♂; ♀]. —Sganga, 2005:142 [distribution]. —Oláh and Johanson, 2012:272 [distribution]. —Pfenninger et al., 2012:1 [intraspecific genetic diversity].

**Distribution.** Argentina, Chile.

***multidens*** (*Smicridea*) Flint and Denning, 1989a:423 [Type locality: Panama, Bocas del Toro Province, Miramar [9°N; 82°15'W]; UCD; ♂]. —Aguila, 1992:542 [distribution]. —Armitage et al., 2015b:5 [checklist]. —Armitage and Cornejo, 2015:194 [checklist].

**Distribution.** Panama.

***murina*** (*Rhyacophylax*) McLachlan, 1871:137 [Type locality: Chile; BMNH; ♂]. —Kimmins, 1957:106 [lectotype]. —Flint, 1974e:88 [checklist]. —Holzenthal, 1988c:70 [distribution; as *zanclophora*]. —Flint, 1989:33 [♂; ♀; distribution; synonymy]. —Flint, 1991:71 [♂; distribution]. —Flint and Reyes, 1991:483 [distribution]. —Aguila, 1992:542 [distribution; as *zanclophora*]. —Flint, 1996b:407 [distribution]. —Maes, 1999:1186 [checklist, as *magna*]. —Muñoz-Quesada, 2000:277 [checklist]. —Cohen, 2004:75 [distribution]. —Sganga, 2005:142 [distribution]. —Chamorro-Lacayo et al., 2007:42 [checklist]. —Oláh and Johanson, 2012:273 [♂; distribution]. —Armitage et al., 2015b:5 [checklist]. —Armitage and Cornejo, 2015:194 [checklist].

—*magna* (Ulmer), 1909b:120 [Type locality: Argentina, Mendoza; ZSZMH; ♂; as *Rhyacophylax magnus*, larva; pupa]. —Weidner, 1964:95 [lectotype]. —Flint, 1989:33 [to synonymy].

—*mendocensis* (Navás), 1920b:43 [Type locality: Argentina, Mendoza; collection Navás, now lost?; ♂; illustrations in figs. 7 and 9 are reversed, in *Rhyacophylax*]. —Flint, 1989:33 [to synonymy].

—*zanclophora* Flint, 1974b:39 [Type locality: Panama, Canal Zone, Pipeline Road, Río Agua Salud; NMNH; ♂; ♀]. —Maes and Flint, 1988:5 [distribution]. —Flint, 1989:33 [to synonymy].

**Distribution.** Argentina, Bolivia, Chile, Colombia, Costa Rica, Ecuador, Nicaragua, Panama, Peru, Venezuela.

***nahuatl*** (*Smicridea*) Flint, 1974b:20 [Type locality: Mexico, Veracruz, Puente Nacional; NMNH; ♂]. —Bueno-Soria and Flint, 1978:207 [distribution]. —Bueno-Soria and Barba-Álvarez, 2011:356 [checklist].

**Distribution.** Mexico.

***nanda*** (*Rhyacophylax*) Flint, 1983a:65 [Type locality: Argentina, Pcia. Misiones Río Iguazú, Camp Nañdu; NMNH; ♂]. —Sganga, 2005:142 [distribution].

**Distribution.** Argentina.

***necator*** (*Rhyacophylax*) Rocha, Dumas and Nessimian, 2016b:424 [Type locality: Brazil, Minas Gerais, Delfinópolis, surrounding area of the Parque Nacional da Serra da Canastra, afluente do Ribeirão Forquilha, 20°18'55.58"S, 46°49'59.04"W, el. 720 m; DZRJ; ♂].

**Distribution.** Brazil.

***nemorosa*** (*Rhyacophylax*) Holzenthal and Blahnik, 1995:214 [Type locality: Costa Rica, Alajuela, Reserva Forestal San Ramón, Río San Lorencito and tribs., 10.216°N, 84.607°W; NMNH; ♂; ♀].

**Distribution.** Costa Rica.

***nemtompa*** (*Rhyacophylax*) Oláh and Johanson, 2012:274 [Type locality: Ecuador, Amasonian [sic] Lowland, Gareno, near Puerto Napo, 400 m; OPC; ♂].

**Distribution.** Ecuador, Peru.

***nigerrima*** (*Smicridea*) Flint, 1983a:55 [Type locality: Argentina, Pcia. Tucumán, Horco Molle, near Tucumán; NMNH; ♂]. —Cohen, 2004:74, 75 [list of type material, distributiom]. —Sganga, 2005:142 [distribution]. —Isa Miranda and Rueda Martín, 2014:200 [distribution].

**Distribution.** Argentina.

***nigricans*** (*Smicridea*) Flint, 1991:67 [Type locality: Colombia, Dpto. Antioquia, 7 km E San Jerónimo [road to Medellín]; NMNH; ♂]. —Flint, 1996b:405 [distribution; identification not certain]. —Muñoz-Quesada, 2000:277 [checklist].

**Distribution.** Colombia, Ecuador, Peru [?].

***nigripennis*** (*Smicridea*) Banks, 1920:359 [Type locality: Colombia, Caldras [sic, recte Caldas]; MCZ; ♂]. —Flint,1967c:14 [♂; lectotype, redescription]. —Flint, 1981a:23 [♂; distribution]. —Flint, 1991:69 [♂; distribution]. —Muñoz-Quesada, 2000:277 [checklist].

**Distribution.** Colombia, Venezuela.

***obesa*** (*Smicridea*) Banks, 1938:303 [Type locality: Cuba, Oriente, Pico Turquino, summit; MCZ; ♂]. —Flint, 1967c:14 [♂; lectotype, redescription]. —Flint, 1968b:81 [checklist]. —Botosaneanu, 1979:47 [distribution]. —Flint, 1996c:16 [checklist]. —Botosaneanu, 2002:94 [checklist]. —Naranjo López and González Lazo, 2005:150 [checklist].

**Distribution.** Cuba.

***obliqua*** (*Smicridea*) Flint, 1974c:90 [Type locality: Suriname, Kaboeri Creek, first camp, near Winnana creek; RNH; ♂]. —Flint, 1978:398 [distribution]. —Paprocki et al., 2004:10 [checklist]. —Albino et al., 2011:25 [larva; pupa; biology; distribution]. —Paprocki and França, 2014:37 [checklist].

**Distribution.** Brazil, Suriname.

***octospina*** (*Smicridea*) Flint, 1974c:83 [Type locality: Suriname, Litani River, Waremapan, small spring creek; RNH; ♂].

**Distribution.** Suriname.

***olivacea*** (*Smicridea*) Flint, 1983a:57 [Type locality: Argentina, Pcia. Catamarca, Arroyo El Pintado, near La Viña; NMNH; ♂]. —Mangeaud, 1996:154 [distribution]. —Sganga, 2005:142 [distribution].

**Distribution.** Argentina.

***palifera*** (*Smicridea*) Flint, 1981a:23 [Type locality: Venezuela, Aragua, Maracay, El Limón; NMNH; ♂; ♀]. —Flint, 1992d:66 [distribution]. —Flint and Sykora, 1993:53 [distribution]. —Botosaneanu, 2002:94 [checklist]. —Botosaneanu and Viloria, 2002:108 [distribution; Isla Margarita]. —Blahnik et al., 2004:4 [distribution]. —Paprocki et al., 2004:10 [checklist]. —Dumas et al., 2009:360 [distribution]. —Albino et al., 2011:29 [larva; pupa; biology; distribution]. —Nogueira and Cabette, 2011:351 [distribution]. —Barcelos-Silva et al., 2012:1278 [distribution]. —Souza et al., 2013a:5 [distribution]. —Paprocki and França, 2014:37 [checklist].

**Distribution.** Brazil, Grenada, Venezuela.

***pallidivittata*** (*Rhyacophylax*) Flint, 1972b:239 [Type locality: Argentina, Prov. Misiones, Capioví; NMNH; ♂]. —Sganga, 2005:142 [distribution].

**Distribution.** Argentina.

***palmar*** (*Rhyacophylax*) Sganga, 2005:143 [Type locality: Argentina, Entre Ríos, arroyo El Palmar, Ruta Nacional 14; MACN; ♂; ♀]. —Angrisano and Sganga, 2007:13 [♂; ♀; distribution]. —Albino et al., 2011:3 [distribution]. —Nogueira and Cabette, 2011:351 [distribution]. —Barcelos-Silva et al., 2012:1278 [distribution]. —Souza et al., 2013a:5 [distribution]. —Paprocki and França, 2014:35 [checklist].

**Distribution.** Argentina, Brazil.

***pampeana*** (*Rhyacophylax*) Flint, 1980b:137 [Type locality: Argentina, Pcia. Buenos Aires, Río Sauce Grande, Sierra de la Ventana; NMNH; ♂; as *pampena*, misspelling]. —Flint, 1982c:29 [distribution]. —Sganga, 2005:142 [distribution]. —Sganga and Angrisano, 2005:135 [♂; distribution]. —Sganga and Fontanarrosa, 2006:9 [larva; pupa; distribution]. —Rueda Martín and Sganga, 2011:2226 [♂; distribution].

**Distribution.** Argentina, Bolivia, Uruguay.

***paranensis*** (*Smicridea*) Flint, 1983a:55 [Type locality: Argentina, Pcia. Misiones, 7 km E El Dorado; NMNH; ♂]. —Marinoni and Almeida, 2000:286 [distribution; biology]. —Blahnik et al., 2004:4 [distribution]. —Paprocki et al., 2004:10 [checklist]. —Sganga, 2005:142 [distribution]. —Dumas et al., 2009:360 [distribution]. —Calor, 2011:321 [checklist]. —Paprocki and França, 2014:38 [checklist].

**Distribution.** Argentina, Brazil, Paraguay.

***parany*** (*Rhyacophylax*) Oláh and Johanson, 2012:275 [Type locality: Peru, Amazonas Prov., Rio Utcabamba, Bajra Grande [sic, Utcubamba, Bagua Grande], at Rio Hotel, 5°45.824'S, 78°25.414'W; NRS; ♂].

**Distribution.** Peru.

***patinae*** (*Smicridea*) Pauls, Blahnik and Holzenthal, in Pauls et al., 2010:1067 [Type locality: Chile, Region X - Los Lagos, Monumento Nacional Alerce Costero, unnamed tributary on trail to Alerce Milenario, 895 m, 40°11.874'S, 73°26.217'W; UMSP; ♂].

**Distribution.** Chile.

***penai*** (*Smicridea*) Flint, 1989:17 [Type locality: Chile, Pcia. Osorno, Pucatrihue; NMNH; ♂; ♀]. —Oláh and Johanson, 2012:277 [distribution].

**Distribution.** Chile.

***peruana*** (*Rhyacophylax*) (Martynov), 1912:27 [Type locality: Peru, Callanga; ASL; ♂; as *Rhyacophylax peruanus*]. —Flint, 1975:572 [distribution]. —Flint, 1980a:214 [distribution; larva?]. —Flint, 1996b:406 [distribution]. —Cohen, 2004:75 [distribution]. —Sganga, 2005:142 [distribution]. —Rueda Martín and Sganga, 2011:2228 [♂; redescription; distribution]. —Oláh and Johanson, 2012:277 [distribution]. —Isa Miranda and Rueda Martín, 2014:200 [distribution].

**Distribution.** Argentina, Bolivia, Peru.

***petasata*** (*Rhyacophylax*) Flint, 1981a:23 [Type locality: Venezuela, Aragua, Maracay, Río Limón, Estación Piscicultura; NMNH; ♂].

**Distribution.** Ecuador, Venezuela.

***pipila*** (*Smicridea*) Flint, 1974b:26 [Type locality: Guatemala, Escuintla, Río Metapa, 10 km SE of Escuintla; NMNH; ♂; ♀]. —Bueno-Soria and Flint, 1978:208 [distribution].

**Distribution.** Guatemala, Mexico.

***piraya*** (*Rhyacophylax*) Flint, 1983a:58 [Type locality: Argentina, Pcia. Misiones, Arroyo Piray Guazú, N San Pedro; NMNH; ♂]. —Marinoni and Almeida, 2000:286 [distribution; biology]. —Blahnik et al., 2004:4 [distribution]. —Paprocki et al., 2004:9 [checklist]. —Sganga, 2005:142 [distribution]. —Calor, 2011:321 [checklist]. —Barcelos-Silva et al., 2012:1278 [distribution]. —Paprocki and França, 2014:35 [checklist]. —Moretto and Bispo, 2015:126 [distrtibution].

**Distribution.** Argentina, Brazil.

***pochutla*** (*Smicridea*) Bueno-Soria, Santiago-Fragoso, and Barba-Álvarez, 2001:150 [Mexico, Oaxaca, Pchutla, Finca Progreso; CNIN; ♂].

**Distribution.** Mexico.

***polyfasciata*** (*Smicridea*) Martynov, 1912:22 [Type locality: Peru, 11°3'S, 75°17'W; now lost?; ♂]. —Flint, 1991:69 [♂; distribution; redescription]. —Flint, 1996b:405 [distribution]. —Muñoz-Quesada, 2000:277 [checklist].

**Distribution.** Bolivia, Colombia, Ecuador, Peru.

***probolophora*** (*Rhyacophylax*) Flint, 1991:73 [Type locality: Colombia, Dpto. Antioquia, Quebrada Honda, Marsella [12 km SW Fredonia]; NMNH; ♂]. —Muñoz-Quesada, 2000:277 [checklist].

**Distribution.** Colombia, Venezuela.

***prorigera*** (*Smicridea*) Flint, 1991:65 [Type locality: Colombia, Dpto. Antioquia, Quebrada La Cebolla, Fizebad [W La Fe]; NMNH; ♂]. —Muñoz-Quesada, 2000:277 [checklist].

**Distribution.** Colombia.

***protera*** (*Smicridea*) (Denning), 1947a:658 [Type locality: Puerto Rico, Luquillo; CAS; ♂; ♀; in *Cheumatopsyche*]. —Flint, 1964a: 41 [♂; ♀; to *Smicridea*, distribution; larva; pupa]. —Flint, 1968b:81 [checklist]. —Flint and Masteller, 1993:141 [biology]. —Masteller and Flint, 1993:69 [biology]. —Botosaneanu, 2002:94 [checklist].

**Distribution.** Puerto Rico.

***pseudolobata*** (*Rhyacophylax*) Flint, 1978:377 [Type locality: Brazil, Amazonas, Rio Marauia, Endstation vor langer Cachoeira, Fluss tritt hier aus dem Geberg mit starkem gefälle; NMNH; ♂]. —Flint, 1974c:95 [misidentified as *lobata*, distribution]. —Paprocki et al., 2004:9 [checklist]. —Dumas et al., 2010:8 [distribution]. —Paprocki and França, 2014:35 [checklist].

**Distribution.** Brazil, Suriname.

***pseudoradula*** (*Rhyacophylax*) Flint, 1991:73 [Type locality: Colombia, Dpto. Antioquia, Río Aurra, km 50, E San Jerónimo; NMNH; ♂]. —Flint, 1996b:408 [distribution]. —Muñoz-Quesada, 2000:277 [checklist]. —Oláh and Johanson, 2012:277 [distribution].

**Distribution.** Colombia, Peru, Venezuela.

***pucara*** (*Smicridea*) Flint, 1989:19 [Type locality: Argentina, Pcia. Neuquén, Pantano, near Estación Forestal Pucará [near SW end Lago Lacar]; NMNH; ♂; ♀]. —Sganga, 2005:142 [distribution]. —Oláh and Johanson, 2012:277 [distribution].

**Distribution.** Argentina, Chile.

***radula*** (*Rhyacophylax*) Flint, 1974b:36 [Type locality: Costa Rica, San Jose, Río General, Pacuare; NMNH; ♂; ♀]. —Bueno-Soria and Flint, 1978:208 [distribution]. —Holzenthal, 1988c:69 [distribution]. —Aguila, 1992:542 [distribution]. —Maes, 1999:1186 [checklist]. —Blahnik et al., 2004:4 [distribution]. —Paprocki et al., 2004:9 [checklist]. —Chamorro-Lacayo et al., 2007:42 [checklist]. —Dumas et al., 2009:360 [distribution]. —Bueno-Soria and Barba-Álvarez, 2011:356 [checklist]. —Calor, 2011:321 [checklist]. —Dumas and Nessimian, 2012:25 [distribution]. —Barcelos-Silva et al., 2012:1278 [distribution]. —Paprocki and França, 2014:35 [checklist]. —Armitage et al., 2015b:6 [checklist]. —Moretto and Bispo, 2015:126 [distrtibution]. —Armitage and Cornejo, 2015:194 [checklist].

**Distribution.** Costa Rica, Brazil, El Salvador, Guatemala, Honduras, Mexico, Nicaragua, Panama.

***ralphi*** (*Rhyacophylax*) Almeida and Flint, 2002:772 [Type locality: Brazil, Rio de Janeiro, Km 54, 26 km E. of Nova Friburgo, 410 m; MZUSP; ♂]. —Paprocki et al., 2004:9 [checklist]. —Dumas et al., 2009:360 [distribution]. —Calor, 2011:321 [checklist]. —Barcelos-Silva et al., 2012:1278 [distribution]. —Paprocki and França, 2014:36 [checklist]. —Moretto and Bispo, 2015:126 [distrtibution].

**Distribution.** Brazil.

***rara*** (*Smicridea*) Bueno-Soria and Márquez-Mayaudón, 1979:481 [Type locality: Mexico, Veracruz, Balzapote, a un kilmetro de la Estación de Biologa Tropical “Los Tuxtlas”; IBUNAM; ♂; as *rarus*]. —Oláh and Johanson, 2012:277 [distribution; as *rarus*].

**Distribution.** Mexico.

***redunca*** (*Smicridea*) Flint, 1989:29 [Type locality: Chile, Pcia. Concepción, Fundo Pinares [about 10 km E Concepción on south side of Río Bío-Bío]; NMNH; ♂; ♀].

**Distribution.** Chile.

***reinerti*** (*Smicridea*) Flint, 1978:376 [Type locality: Brazil, Edo. Para, 164 km.w. of Altamira; MZUSP; ♂]. —Paprocki et al., 2004:10 [checklist]. —Paprocki and França, 2014:38 [checklist].

**Distribution.** Brazil.

***resela*** (*Rhyacophylax*) Oláh and Johanson, 2012:279 [Type locality: Peru, San Martin Prov., creek crossing rd. Tarapoto-Yurimaguas, ca. 30 km (rd.) NE Tarapoto, 6°24.905'S, 76°18.756'W; NRS; ♂].

**Distribution.** Peru.

***riita*** (*Smicridea*) Flint, 1981a:22 [Type locality: Venezuela, Aragua, Dos Riitos, 6 km N Rancho Grande; NMNH; ♂].

**Distribution.** Venezuela.

***roraimense*** (*Rhyacophylax*) Albino, Pes and Hamada, 2011:19 [Type locality: Brazil, Roraima, Caracaraí municipality, Rio Branco, Cachoeira do Bem Querer, N 01°55'42” W 61°00'09”; INPA; ♂]. —Barcelos-Silva et al., 2012:1278 [distribution]. —Souza et al., 2013a:5 [distribution]. —Paprocki and França, 2014:36 [checklist].

**Distribution.** Brazil.

***ruginasa*** (*Smicridea*) Flint, 1991:65 [Type locality: Colombia, Dpto. Antioquia, Quebrada Honda, Marsella [12 km SW Fredonia]; NMNH; ♂]. —Muñoz-Quesada, 2000:277 [checklist].

**Distribution.** Colombia.

***salta*** (*Rhyacophylax*) Flint, 1974b:35 [Type locality: Mexico, San Luis Potosi, El Salto, 26 miles W of Antiguo Morelos; NMNH; ♂; ♀]. —Bueno-Soria and Flint, 1978:208 [distribution].

**Distribution.** Mexico.

***sarkoska*** (*Rhyacophylax*) Oláh and Johanson, 2012:281 [Type locality: Ecuador, Mindo, Septimo Paraiso, 1600 m; OPC; ♂].

**Distribution.** Colombia, Ecuador.

***sarla*** (*Smicridea*) Oláh and Johanson, 2012:282 [Type locality: Peru, Huanuco, stream at Carpish, 2500 m, 76°09'W, 9°40'S; NRS; ♂].

**Distribution.** Peru.

***sarvaka*** (*Rhyacophylax*) Oláh and Johanson, 2012:283 [Type locality: Peru, San Martin Prov., Rio Negro, 37 km (rd.) W Moyobamba, near Olmos-Tarapoto rd., 6°00.278'S, 77°15.437'W; NRS; ♂].

**Distribution.** Peru.

***sattleri*** (*Smicridea*) Denning and Sykora, 1968:175 [Type locality: Brazil, Sao Paulo, waterfall on Iporanga Beach, Guaruja Island near Santos; CAS; ♂]. —Paprocki et al., 2004:10 [checklist]. —Calor, 2011:321 [checklist]. —Paprocki and França, 2014:38 [checklist]. —Rocha et al., 2016b:429 [distribution].

**Distribution.** Brazil.

***saucia*** (*Smicridea*) McLachlan, 1871:137 [Type locality: Peru, probably in the vicinity of Lima; BMNH; ♂]. —Kimmins, 1957:106 [lectotype]. —Flint and Reyes, 1991:481 [♂; redescription; distribution].

**Distribution.** Peru.

***scutellaris*** (*Rhyacophylax*) Flint, 1974c:96 [Type locality: Suriname, Coeroeni-eiland; RNH; ♂]. —Flint, 1978:381 [distribution]. —Flint, 1992d:67 [distribution]. —Blahnik et al., 2004:4 [distribution]. —Paprocki et al., 2004:9 [checklist]. —Oláh and Johanson, 2012:284 [distribution; as *scutellaria*]. —Paprocki and França, 2014:36 [checklist].

**Distribution.** Brazil, French Guiana, Suriname.

***sepala*** (*Smicridea*) Rocha, Dumas and Nessimian, 2016b:427 [Type locality: Brazil, Minas Gerais, Delfinópolis, surrounding area of the Parque Nacional da Serra da Canastra, confluência do Ribeirão Grande e Córrego Mata do Engenho, 20°31'20.20"S, 46°30'37.57"W, el. 661 m; DZRJ; ♂].

**Distribution.** Brazil.

***sexspinosa*** (*Smicridea*) Flint, 1978:376 [Type locality: Brazil [Edo. Amazonas], Gebeit Endstation Rio Marauia, Bergbach II, schattig, starkes Gefalle ber Granitblocken; NMNH; ♂]. —Paprocki et al., 2004:10 [checklist]. —Albino et al., 2011:3 [distribution]. —Paprocki and França, 2014:38 [checklist].

**Distribution.** Brazil.

***signata*** (*Rhyacophylax*) (Banks), 1903b:243 [Type locality: United States, Colorado; MCZ; ♀; in *Pellopsyche*]. —Ross, 1944:294 [to *Smicridea*]. —Flint, 1974b:29 [♂; ♀; redescription; distribution; larva; pupa]. —Bueno-Soria and Flint, 1978:208 [distribution]. —Maes and Flint, 1988:5 [distribution]. —Holzenthal, 1988c:69 [distribution]. —Aguila, 1992:542 [distribution]. —Maes, 1999:1186 [checklist]. —Baumgardner and Bowles, 2005:11 [distribution]. —Bueno-Soria et al., 2005:75 [distribution]. —Blinn and Ruiter, 2006:332 [ecology]. —Chamorro-Lacayo et al., 2007:42 [checklist]. —Blinn and Ruiter, 2009a:304 [biology]. —Bueno-Soria and Barba-Álvarez, 2011:356 [checklist]. —Armitage et al., 2015b:6 [checklist]. —Armitage and Cornejo, 2015:194 [checklist]. —Rázuri-Gonzales and Holzenthal, 2016:21 [compared to *Smicridea lobata*; distribution; ♂].

**Distribution.** Guatemala, Mexico, U.S.A.

***simmonsi*** (*Smicridea*) Flint, 1968b:28 [Type locality: St. Lucia, Vergallier River, near Marquis; NMNH; ♂; larva; pupa]. —Botosaneanu, 1990a:40 [♂; ♀; synonymy, redescription]. —Flint and Sykora, 1993:53 [distribution]. —Botosaneanu, 2002:94 [checklist, not from Guadaloupe]. —Botosaneanu and Thomas, 2005:50 [distribution].

—*therezieni* (*Smicridea*) Malicky, 1987a:84 [Type locality: Martinique, Rav. Pirogue; collection Malicky; ♂]. — Botosaneanu, 1988:216 [♂; ♀; redescription; larva; pupa]. —Botosaneanu, 1990a:40 [to synonymy].

—*aurimacula* (*Smicridea*) Flint and Denning, 1989a:421 [Type locality: St. Vincent, Hermitage; NMNH; ♂]. —Botosaneanu, 1990a:40 [to synonymy].

**Distribution.** Martinique, St. Lucia, St. Vincent.

***singri*** (*Rhyacophylax*) Holzenthal and Blahnik, 1995:216 [Type locality: Costa Rica, Puntarenas, Río Singri, ca. 2 km (air) S Finca Helechales, 9.057°N, 83.082°W; NMNH; ♂; ♀].

**Distribution.** Costa Rica.

***sirena*** (*Smicridea*) Bueno-Soria, 1986:59 [Type locality: Costa Rica, Estación Sirena, Cocovado; IBUNAM; ♂]. —Holzenthal, 1988c:70 [distribution].

**Distribution.** Costa Rica.

***smilodon*** (*Smicridea*) Flint, 1989:29 [Type locality: Chile, Pcia. Ñuble, Recinto; NMNH; ♂; ♀].

**Distribution.** Chile.

***soyatepecana*** (*Smicridea*) Bueno-Soria, 1986:61 [Type locality: Mexico, Guerrero, Soyatepec, 7 kms al poniente del Ocotito; IBUNAM; ♂]. —Bueno-Soria et al., 2005:75 [distribution].

**Distribution.** Mexico.

***spinulosa*** (*Rhyacophylax*) Flint, 1972b:242 [Type locality: Argentina, Prov. Misiones, Puerto Rico; NMNH; ♂]. —Marinoni and Almeida, 2000:286 [distribution; biology]. —Blahnik et al., 2004:4 [distribution]. —Paprocki et al., 2004:9 [checklist]. —Sganga, 2005:142 [distribution]. —Sganga and Angrisano, 2005:135 [♂; distribution]. —Sganga and Fontanarrosa, 2006:11 [larva; pupa; distribution]. —Angrisano and Sganga, 2007:14 [♂; distribution]. —Calor, 2011:321 [checklist]. —Paprocki and França, 2014:36 [checklist]. —Manzo et al., 2014:166 [distribution]. —Moretto and Bispo, 2015:126 [distrtibution].

**Distribution.** Argentina, Brazil, Uruguay.

***sudara*** (*Rhyacophylax*) Oláh and Johanson, 2012:285 [Type locality: Ecuador, Western Andean Slope, Alambi; OPC; ♂].

**Distribution.** Ecuador, Peru.

***talamanca*** (*Rhyacophylax*) Flint, 1974b:39 [Type locality: Costa Rica, Cartago, Chitaria; NMNH; ♂; ♀]. —Holzenthal, 1988c:70 [distribution]. —Aguila, 1992:542 [distribution]. —Armitage et al., 2015b:6 [checklist]. —Armitage and Cornejo, 2015:194 [checklist].

**Distribution.** Costa Rica, Panama.

***tapanti*** (*Rhyacophylax*) Holzenthal and Blahnik, 1995:218 [Type locality: Costa Rica, Cartago, Reserva Tapantí, Quebrada Palmitos and falls, 9.686°N, 83.78°W; NMNH; ♂; ♀].

**Distribution.** Costa Rica.

***tarasca*** (*Smicridea*) Flint, 1974b:25 [Type locality: Mexico, Michoacan, San Lorenzo, route 15, km 206; NMNH; ♂; ♀]. —Bueno-Soria and Flint, 1978:207 [distribution].

**Distribution.** Mexico.

***tavola*** (*Rhyacophylax*) Oláh and Johanson, 2012:286 [Type locality: Ecuador, Amasonian [sic] Lowland, Gareno, near Puerto Napo, 400 m; OPC; ♂].

**Distribution.** Ecuador.

***thermophila*** (*Rhyacophylax*) Rueda Martín and Sganga, 2011:2219 [Type locality: Argentina, Jujuy province, Ledesma, Río Aguas Calientes, 23°44'46"S, 64°31'29"W, 100 m; IML; ♂; larva; pupa; biology].

**Distribution.** Argentina.

***tina*** (*Rhyacophylax*) Oláh and Johanson, 2012:287 [Type locality: Ecuador, Tinalandia Nature Reserve, West Andean Slope, 600 m, 150 km near Quito; OPC; ♂].

**Distribution.** Ecuador.

***titschacki*** (*Rhyacophylax*) Flint, 1975:570 [Type locality: Sud- Peru, Sivia; ZSZMH; ♂]. —Flint, 1996b:407 [distribution; as *titschki*, mispelling]. —Oláh and Johanson, 2012:288 [distribution].

**Distribution.** Peru.

***tobada*** (*Smicridea*) Flint and Denning, 1989a:419 [Type locality: Tobago, St. John Province, Charlotteville; NMNH; ♂]. —Botosaneanu and Sakal, 1992:203 [distribution; ecology]. —Botosaneanu and Alkins-Koo, 1993:34 [distribution]. —Flint, 1996a:83 [distribution]. —Botosaneanu, 2002:94 [checklist].

**Distribution.** Tobago, Trinidad, Venezuela.

***torpa*** (*Smicridea*) Oláh and Johanson, 2012:289 [Type locality: French Guiana, Approuaguekaw, Kaw Mt, 104 mao, 4°33.035'N, 52°11.661'W; NRS; ♂].

**Distribution.** French Guiana.

***travertinera*** (*Smicridea*) Paprocki, Holzenthal and Cressa, 2003:404 [Type locality: Vene­zuela, Falcón, Quebrada El Charo at cataratas, 10°46.771'N, 69°12.174'W, el. 425 m; UMSP; /male; larva; biology]. —Angrisano and Sganga, 2007:14 [♂; distribution].

**Distribution.** Argentina, Venezuela.

***tregala*** (*Smicridea*) Flint, 1989:20 [Type locality: Chile, Pcia. Arauco, Puente Trongol [~12 km S Curanilahue]; NMNH; ♂; ♀].

**Distribution.** Chile.

***truncata*** (*Smicridea*) Flint, 1974c:91 [Type locality: Suriname, Kaboeri Creek, first camp; RNH; ♂]. —Flint, 1978:381 [distribution]. —Paprocki et al., 2004:10 [checklist]. —Pes et al., 2008:55 [larva; pupa; biology]. —Dumas et al., 2009:360 [distribution]. —Nogueira and Cabette, 2011:351 [distribution]. —Nogueira et al., 2011:176 [community ecology, distribution]. —Paprocki and França, 2014:38 [checklist].

**Distribution.** Brazil, Suriname.

***turgida*** (*Smicridea*) Flint, 1989:28 [Type locality: Chile, Pcia. Arauco, Caramávida; NMNH; ♂; ♀].

**Distribution.** Chile.

***turrialbana*** (*Smicridea*) Flint, 1974b:22 [Type locality: Costa Rica, Cartago, 3 miles W of Turrialba; NMNH; ♂; ♀]. —Holzenthal, 1988c:70 [distribution]. —Flint and Denning, 1989a:431 [variation, distribution]. —Armitage et al., 2015b:6 [checklist]. —Armitage and Cornejo, 2015:194 [checklist].

**Distribution.** Costa Rica, Panama.

***ulva*** (*Smicridea*) Flint, 1974b:23 [Type locality: Nicaragua, Chontales, Puente Quinama, E of Villa Somoza; NMNH; ♂]. —Maes and Flint, 1988:5 [distribution]. —Holzenthal, 1988c:70 [distribution]. —Maes, 1999:1187 [checklist]. —Chamorro-Lacayo et al., 2007:42 [checklist].

**Distribution.** Costa Rica, Nicaragua.

***unguiculata*** (*Rhyacophylax*) Flint, 1983a:65 [Type locality: Argentina, Pcia. Misiones, Río Iguazú, Camp Nañdu; NMNH; ♂]. —Marinoni and Almeida, 2000:286 [distribution; biology]. —Blahnik et al., 2004:4 [distribution]. —Paprocki et al., 2004:9 [checklist]. —Sganga, 2005:142 [distribution]. —Calor, 2011:321 [checklist]. —Paprocki and França, 2014:36 [checklist].

**Distribution.** Argentina, Brazil, Paraguay.

***unicolor*** (*Smicridea*) (Banks), 1901:370 [Type locality: Mexico, Cuernavaca; MCZ; ♂; in *Diplectrona*]. —Ross, 1947:144 [to *Smicridea*]. —Flint, 1967c:15 [♂; lectotype, redescription]. —Flint, 1974b:17 [♂; distribution; redescription]. —Bueno-Soria and Flint, 1978:207 [distribution].

**Distribution.** Mexico.

***urra*** (*Smicridea*) Flint, 1991:63 [Type locality: Colombia, Dpto. Antioquia, Urrao; NMNH; ♂]. —Muñoz-Quesada, 2000:277 [checklist].

**Distribution.** Colombia.

***vagotta*** (*Smicridea*) Oláh and Johanson, 2012:290 [Type locality: French Guiana, Approuaguekaw, Kaw Mt, 216 m, 4°33.257'N, 52°11.920'W; NRS; ♂].

**Distribution.** French Guiana.

***vakara*** (*Rhyacophylax*) Oláh and Johanson, 2012:291 [Type locality: Peru, San Martin Prov., Rio Huallaga, at Pumarihi Huallaga Lodge, between Juan Guerra and Chazuta, 14 km (rd) W Chazuta, 6°36.643'S, 76°12.555'W; NRS; ♂].

**Distribution.** Peru.

***valeni*** (*Rhyacophylax*) Rueda Martín and Sganga, 2011:2223 [Type locality: Argentina, Tucumán, El Siambón, Río el Siambón; IML; ♂]. —Isa Miranda and Rueda Martín, 2014:200 [distribution].

**Distribution.** Argentina.

***varia*** (*Smicridea*) (Banks), 1913a:239 [Type locality: Costa Rica, Turricares [sic, Turrúcares]; MCZ; ♂; in *Rhyacophylax*]. —Flint, 1967c:15 [♂; to *Smicridea*]. —Flint, 1974b:17 [♂; ♀; distribution; redescription; larva; pupa]. —Bueno-Soria and Flint, 1978:207 [distribution]. —Maes and Flint, 1988:5 [distribution]. —Holzenthal, 1988c:71 [distribution]. —Aguila, 1992:542 [distribution]. —Blahnik, 1995:100 [♂; ♀; diagnosis; redescription; distribution]. —Maes, 1999:1187 [checklist]. —Chamorro-Lacayo et al., 2007:42 [checklist]. —Bueno-Soria and Barba-Álvarez, 2011:356 [checklist]. —Armitage et al., 2015b:6 [checklist]. —Armitage and Cornejo, 2015:194 [checklist].

**Distribution.** Costa Rica, Ecuador, Guatemala, Mexico, Nicaragua, Panama.

***vaskosa*** (*Smicridea*) Oláh and Johanson, 2012:292 [Type locality: French Guiana, Approuaguekaw, Kaw Mt, 4°33.235'N, 52°11.988'W, 225 mao; NRS; ♂].

**Distribution.** French Guiana.

***vekona*** (*Rhyacophylax*) Oláh and Johanson, 2012:293 [Type locality: Argentina, Jujuy, PN, Calilegua, 13 km NW, visitor center, RP 83, 1263 m, 23°41.923'S, 64°52.342'W; NRS; ♂].

**Distribution.** Argentina.

***ventridenticulata*** (*Rhyacophylax*) Flint, 1991:75 [Type locality: Colombia, Dpto. Antioquia, Quebrada La Agudelo, 2 km E El Retiro; NMNH; ♂]. —Muñoz-Quesada, 2000:277 [checklist]. —Oláh and Johanson, 2012:295 [distribution].

**Distribution.** Colombia, Ecuador, Venezuela.

***veracruzensis*** (*Rhyacophylax*) Flint, 1974b:43 [Type locality: Mexico, Veracruz, Cordoba; NMNH; ♂; ♀]. —Bueno-Soria and Flint, 1978:208 [distribution]. —Bueno-Soria and Barba-Álvarez, 2011:356 [checklist].

**Distribution.** Guatemala, Mexico.

***vermiculata*** (*Rhyacophylax*) Flint, 1978:381 [Type locality: Argentina, Prov. Misiones, Arroyo Saura, 9 km. north of L.N. Alem; NMNH; ♂]. —Marinoni and Almeida, 2000:286 [distribution; biology]. —Blahnik et al., 2004:4 [distribution]. —Paprocki et al., 2004:9 [checklist]. —Sganga, 2005:142 [distribution]. —Calor, 2011:321 [checklist]. —Paprocki and França, 2014:36 [checklist].

**Distribution.** Argentina, Brazil, Paraguay.

***vilela*** (*Rhyacophylax*) Flint, 1978:382 [Type locality: Argentina, Rcho. Barranqueras, Puerto Vilelas; NMNH; ♂]. —Flint, 1982c:29 [distribution]. —Paprocki et al., 2004:9 [checklist]. —Sganga, 2005:142 [distribution]. —Paprocki and França, 2014:36 [checklist].

**Distribution.** Argentina, Brazil.

***villa*** (*Rhyacophylax*) Oláh and Johanson, 2012:295 [Type locality: Peru, Chontachaca, Kosnipata-Cusco, 13°01'25"S, 71°28'03"W, 700 m, humid subtropical forest; NRS; ♂].

**Distribution.** Peru.

***villarricensis*** (*Rhyacophylax*) Flint, 1983a:61 [Type locality: Paraguay, Dpto. Guairá, 3.9 km S Villarrica; NMNH; ♂].

**Distribution.** Paraguay.

***voluta*** (*Rhyacophylax*) Flint, 1978:378 [Type locality: Brazil [Edo. Amazonas], Rio Solimoes, Bereich von Favonio; NMNH; ♂]. —Flint, 1982c:30 [distribution]. —Flint, 1996b:409 [distribution]. —Paprocki et al., 2004:9 [checklist]. —Sganga, 2005:142 [distribution]. —Ribeiro et al., 2009:34 [list of types]. —Paprocki and França, 2014:36 [checklist].

**Distribution.** Argentina, Brazil, Peru.

***weidneri*** (*Rhyacophylax*) Flint, 1972b:238 [Type locality: Argentina, Prov. Misiones, Capioví; NMNH; ♂]. —Flint, 1966a:8 [as *brasilianus*, invalid lectotype, distribution]. —Marinoni and Almeida, 2000:286 [distribution; biology]. —Paprocki et al., 2004:9 [checklist]. —Sganga, 2005:142 [distribution]. —Paprocki and França, 2014:37 [checklist]. —Manzo et al., 2014:166 [distribution].

**Distribution.** Argentina, Brazil.

#### Genus *Streptopsyche* Ross and Unzicker [5]

*Streptopsyche* Ross and Unzicker, 1977:307 [Type species: *Hydropsyche antilles* Ross and Palmer, 1946, original designation]. —Schefter, 2005:149 [phylogeny]. —Oláh and Johanson, 2008:55 [synonym of *Calosopsyche*]. —Geraci et al., 2010:925 [resurrected].

This small genus of five species, known only from Hispaniola, has recently gone into and out of synonymy with *Calosopsyche* (Oláh and Johanson 2008, Geraci et al. 2010). The immature stages are unknown and little is known of its biology, other than the collection of adults near small streams in mountainous areas.

***antilles*** (Ross and Palmer), 1946:184 [Type locality: Santo Domingo [now Hispaniola], [Dominican Republic], Trujillo City [now Santo Domingo]; INHS; ♂; ♀; biology; in *Hydropsyche*]. —Flint, 1962a:25 [♀; distribution]. —Flint, 1968b:81 [checklist]. —Ross and Unzicker, 1977:307 [♂; to *Streptopsyche*]. —Flint and Pérez-Gelabert, 1999:38 [checklist]. —Botosaneanu, 2002:92 [checklist]. —Flint and Sykora, 2004:20 [distribution]. —Oláh and Johanson, 2008:54 [to *Calosopsyche*]. —Pérez-Gelabert, 2008:300 [checklist]. —Geraci et al., 2010:925 [returned to *Streptopsyche*].

**Distribution.** Dominican Republic, Haiti.

***davisorum*** Ross and Unzicker, 1977:308 [Type locality: Dominican Republic, La Estrellita Prov., 4 km SE Rio Limpio; NMNH; ♂]. —Botosaneanu, 1996:18 [♂; ♀; distribution; taxonomic remarks; in *Hydropsyche*]. —Flint and Pérez-Gelabert, 1999:38 [checklist]. —Botosaneanu, 2002:93 [checklist; as *Hydropsyche*]. —Flint, 2002:409 [larva; pupa; similar to *Streptopsyche parander*]. —Flint and Sykora, 2004:20 [distribution]. —Oláh and Johanson, 2008:54 [to *Calosopsyche*]. —Pérez-Gelabert, 2008:300 [checklist]. —Geraci et al., 2010:925 [returned to *Streptopsyche*].

**Distribution.** Dominican Republic.

***parander*** (Botosaneanu), 1996:19 [Type locality: Dominican Republic, Arroyo San Rafael, S from Barahona; ZMUA; ♂; ♀; in *Hydropsyche*]. —Flint et al., 1999a:76 [to *Streptopsyche*]. —Flint and Pérez-Gelabert, 1999:38 [checklist]. —Botosaneanu, 2002:93 [checklist; as *Hydropsyche*]. —Flint and Sykora, 2004:20 [distribution]. —Oláh and Johanson, 2008:55 [to *Calosopsyche*]. —Pérez-Gelabert, 2008:300 [checklist]. —Geraci et al., 2010:925 [returned to *Streptopsyche*].

**Distribution.** Dominican Republic.

***praecipua*** Flint and Sykora, 2004:22 [Type locality: Haiti, Departement de l’Ouest, Manneville, about 60 ft alt [about el. 18 m]; AMNH; ♂]. —Oláh and Johanson, 2008:54 [to *Calosopsyche*]. —Pérez-Gelabert, 2008:300 [checklist]. —Geraci et al., 2010:925 [returned to *Streptopsyche*].

**Distribution.** Haiti.

***rawlinsi*** Flint and Sykora, 2004:20 [Type locality: Haiti, Departement du Sud, S slope Morne Formon, Ville Formon, 31 km NW Les Cayes, Massif de la Hotte, 18°20'N, 74°01'W, el. 1405 m; CMNH; ♂; ♀]. —Oláh and Johanson, 2008:54 [to *Calosopsyche*]. —Pérez-Gelabert, 2008:300 [checklist]. —Geraci et al., 2010:925 [returned to *Streptopsyche*].

**Distribution.** Haiti.

#### Genus *Synoestropsis* Ulmer [10]

*Synoestropsis* Ulmer, 1905a:43 [Type species: *Synoestropsis pedicillata* Ulmer, 1905a, subsequent designation Fischer 1963]. —Roback, 1966:243 [probable larva]. —Bentes et al., 2008:580 [biology]. —Calor, 2008a:320 [larva].

*Chiasmoda* Navás, 1920b:40 [Type species: *Chiasmoda ecliptica* Navás, 1920b, original designation]. —Flint et al., 1999a:75 [to synonymy].

This is the only member of the macronematine tribe Polymorphanisini to occcur in the Neotropics, where its ten species are found from central Mexico to northern Argentina, but not on the Antillean islands.

The larva of an unknown species, long suspected to be *Synoestropsis*, was ilustrated by Roback (1966) as Hydropsychidae sp. 1, but is was not until Calor (2008) described the larva of *Synoestropsis furcata* that the immatures have been positively associated. Both Calor (2008) and Flint (personal communication) collected larvae from a gravel riffle of a large stream. Larvae were found several centimeters down in the gravel or on rocky substrate. No clearly recognizable silken retreat or net was found, but these might have been destroyed in digging through the gravel. Bentes et al. (2008) examined the stomach contents of 48 larvae and, based on the high frequency of animal items, inferred that the larvae were predators. Adults, almost always females, come to bright lights near large lowland rivers. Flint did observe males of *Synoestropsis obliqua* swarming near dusk along the margin of the same river in which he collected larvae. They were flying up and down from 3 to 10 m in elevation in a large, but not very dense, swarm next to the marginal forest.

***ecliptica*** (Navás), 1920b:41 [Type locality: Republica Argentina, Rosario; collection Navás, now lost?; ♂; in *Chiasmoda*; the figures over the legends for figs. 7 and 9 have clearly been reversed, thus the figure that should have been over the legend for Fig. 7 appears over Fig. 9, and vice versa]. —Flint et al., 1999a:76 [to *Synoestropsis*].

**Distribution.** Argentina.

***euryphlebia*** Navás, 1934b:90 [Type locality: Guyane fraç.se, S.t Jean de Maroni; MNHNP; ♀; now lacks abdomen].

**Distribution.** French Guiana.

***furcata*** Flint, 1974c:117 [Type locality: Guyana, Esseq., 6 miles south of Wineperu, Picrewana Island; NMNH; ♂]. —Flint, 1978:396 [♂; distribution; wings]. —Botosaneanu and Flint, 1982:16 [♀]. —Flint, 1992d:67 [distribution]. —Paprocki et al., 2004:10 [checklist]. —Calor, 2008a:320 [larva]. —Nogueira and Cabette, 2011:351 [distribution]. —Paprocki and França, 2014:38 [checklist].

**Distribution.** Brazil, Guyana, Suriname, Venezuela.

***grisoli*** Navás, 1924b:252 [Type locality: Venezuela, Arismondi; MNHNP; ♀]. —Flint, 1974c:118 [♂; distribution]. —Flint, 1978:396, 404 [♂; distribution; wings]. —Botosaneanu and Flint, 1982:17 [♀]. —Flint, 1992d:67 [distribution]. —Flint, 1996b:412 [distribution]. —Blahnik et al., 2004:4 [distribution]. —Paprocki et al., 2004:10 [checklist]. —Nogueira and Cabette, 2011:351 [distribution]. —Costa et al., 2014:221 [distribution]. —Paprocki and França, 2014:38 [checklist].

**Distribution.** Brazil, Guyana, Peru, Suriname, Venezuela.

***manicata*** (Navás), 1920b:42 [Type locality: R. Argentina, Paso Patria; collection Návas, now lost?; ♀; in *Chiasmoda*]. —Flint et al., 1999a:76 [to *Synoestropsis*].

**Distribution.** Argentina.

***obliqua*** Ulmer, 1905a:45 [Type locality: Brasilien, Rio Grande do Sul; PAN; ♂]; 1907c:28 [wings]. —Flint, 1966a:8 [♂; wings; lectotype]. —Paprocki et al., 2004:10 [checklist]. —Paprocki and França, 2014:39 [checklist].

**Distribution.** Brazil.

***pedicillata*** Ulmer, 1905a:43 [Type locality: [Brazil], Sta. Catharina [sic]; PAN; ♂]. —Ulmer, 1907c:26 [♂; wings]. —Flint, 1966a:8 [♂; wings; lectotype]. —Flint, 1972b:235 [distribution]. —Blahnik et al., 2004:4 [distribution]. —Paprocki et al., 2004:10 [checklist]. —Calor, 2011:321 [checklist]. —Barcelos-Silva et al., 2012:1278 [distribution]. —Souza et al., 2013a:6 [tentative, unconfirmed identification]. —Paprocki and França, 2014:39 [checklist].

**Distribution.** Argentina, Brazil.

***punctipennis*** Ulmer, 1905a:47 [Type locality: Colombien, Bogota; ZSZMH; ♀]. —Ulmer, 1907c:26 [wings]. —Weidner, 1964:99 [type destroyed]. —Flint, 1978:397 [distribution; wings]. —Bueno-Soria and Flint, 1978:209 [distribution]. —Holzenthal, 1988c:71 [distribution]. —Maes and Flint, 1988:5 [distribution]. —Flint, 1996b:413 [distribution]. —Maes, 1999:1187 [checklist]. —Muñoz-Quesada, 2000:277 [checklist]. —Paprocki et al., 2004:10 [checklist]. —Chamorro-Lacayo et al., 2007:42 [checklist]. —Paprocki and França, 2014:39 [checklist].

**Distribution.** Brazil, Colombia, Costa Rica, Ecuador, Guatemala, Guyana, Honduras, Mexico, Nicaragua, Peru.

***stictonota*** Navás, 1932a:83 [Type locality: Brazil, Blumenau; collection Navás, now lost?; ♀; the figures over the legends for figs. 72 and 73 have clearly been reversed, thus the figure that should have been over the legend for Fig. 72 appears over Fig. 73, and vice versa]. —Paprocki et al., 2004:10 [checklist]. —Paprocki and França, 2014:39 [checklist].

**Distribution.** Brazil.

***vitrea*** Navás, 1920a:133 [Type locality: [Argentina], Santa Fe; collection Navás, now lost?; ♀].

**Distribution.** Argentina.

### Family Hydroptilidae

Hydroptilidae, or microcaddisflies, are found around the globe. The family is among the most species rich in all of Trichoptera. The adults of most of the species are usually no more than 5 mm in total length, and some much smaller. Marshall (1979) provided the last comprehensive review and recorded 46 genera and 616 species worldwide. Almost 30 years later, Holzenthal et al. (2007b) listed a world fauna of ca. 70 genera and 2,000 species. Although 922 species, inlcuding 7 fossil species, placed in 36 genera are here recorded from the Neotropics, many more undescribed species are known to occur in the region. The Neotropical fauna includes a number of endemic genera, especially in the Leucotrichiinae, but several very species rich cosmopolitan gerera also occur in the region, e.g., *Hydroptila* and *Oxyethira*. One genus, *Eutonella*, formerly included in the family was removed and placed as *incertae sedis* within the order by Santos et al., (2016).

The higher classification proposed by Marshall (1979) has remained relatively stable, especially of the tribes (now subfamilies), except for a few changes. Malicky (2001, 2008) elevated the subfamily Ptilocolepinae to family status, thus restricting the family to include only the subfamily Hydroptilinae as defined by Marshall (1979). Consequently, all of the tribes listed by Marshall are now considered subfamilies. Ptilocolepidae and its two included genera are restricted to the Northern Hemisphere and are not treated here. The Hydroptilinae are of wide distribution and consist of six subfamilies, all of which are represented in the Neotropics:

Hydroptilinae: *Hydroptila*, *Oxyethira*, *Tricholeiochiton*

Leucotrichiinae: *Acostatrichia*, *Alisotrichia*, *Anchitrichia*, *Ascotrichia*, *Betrichia*, *Byrso­pteryx*, *Ceratotrichia*, *Celaenotrichia*, *Cerasmatrichia*, *Costatrichia*, *Leucotrichia*, *Mejicanotrichia*, *Peltopsyche*, *Scelobotrichia*, *Tupini­quintrichia*, *Zumatrichia*

Neotrichiinae: *Kumanskiella*, *Mayatrichia*, *Neotrichia*, *Taraxitrichia*

Ochrotrichiinae: *Angrisanoia*, *Metrichia*, *Nothotrichia*, *Ochrotrichia*, *Ragatrichia*, *Rhyacopsyche*

Orthotrichiinae: *Ithytrichia*, *Orthotrichia*

Stactobiinae: *Bredinia*, *Flintiella*, *Orinocotrichia*, *Tizatetrichia*

*Incertae Sedis*: *Dicaminus*

Recently, Santos et al. (2016) revised the phylogeny and classification of Leucotrichiinae, based on an analysis of morphological and molecular data. Their results supported the monophyly of Leucotrichinae and suggested that the included genera belong to two monophyletic tribes, the newly established Alisotrichiini (*Alisotrichia*, *Byrsopteryx*, *Celaenotrichia*, *Cerasmatrichia*, *Mejicanotrichia*, *Scelobotrichia*), and a revised definition of Leucotrichiini Flint (*Acostatrichia*, *Anchitrichia*, *Ascotrichia*, *Betrichia*, *Ceratotrichia*, *Costatrichia*, *Leucotrichia*, *Peltopsyche*, *Tupiniquintrichia*, *Zumatrichia*). They also proposed several nomenclatural changes to reflect their phylogenetic results, including the synonymy of *Abtrichia* with *Peltopsyche*, the transfer of *Betrichia hamulifera* to *Costatrichia*, *Betrichia alibrachia* and *Costatrichia falsa* to *Leucotrichia*, and *Costatrichia fluminensis* to *Acostatrichia*. They also established a new genus, *Tupiniquintrichia*, to include *Peltopsyche maclachlani* and *Leucotrichia procera*. Earlier, Oláh and Johanson (2011) and Oláh and Flint (2012) described many new species in several genera, including a new one, *Ragatrichia*, from across the Neotropics and assigned these to genus clusters based on overall similarity. Finally, Thomson and Holzenthal (2015) provided a revision of *Leucotrichia* and described about a dozen new species. Many other new species, as indicated below, have been described in the last 10 years, especially by South American workers (e.g., Angrisano, Rueda Martín, Santos, Souza).

The larvae of microcaddisflies are highly diverse in form, habitat, and feeding behavior. Although most construct cases of silk or sand, some construct shelters covering only the exposed side and are firmly attached to the substrate, and others remain free-living prior to pupation. Several genera occurring in the Neotropics remain unknown in the larval stage.

#### Genus *Acostatrichia* Mosely [20]

*Acostatrichia* Mosely, 1939a:228 [Type species: *Acostatrichia plaumanni* Mosely 1939a, original designation]. —Angrisano and Sganga, 2010:56 [immatures; biology]. —Santos et al., 2016a:470 [phylogeny].

This genus of Leucotrichiinae, Leucotrichiini, is widespread over much of South America and Panama, but is not known from the rest of Central America or the Antilles. The monophyly of the genus is equivocal and the taxonomy and phylogeny of this and several others in the tribe (e.g., *Betrichia*, *Costatrichia*) require further carefull study (Santos et al., 2016a).

The immature stages of *Acostatrichia simulans* were described by Angrisano and Sganga (2010) and appear to be typical of the tribe. The larvae were collected in a sunny section of the stream where the canopy was partially open. Cases were abundant and attached to the substrate in shallow areas of the study section (Angrisano and Sganga 2010).

***brevipenis*** Flint, 1974c:54 [Type locality: Suriname, Lawa River, Anapaike; RNH; ♂]. —Flint, 1992d:69 [distribution]. —Angrisano, 1999:31 [checklist]. —Paprocki et al., 2004:10 [checklist]. —Oláh and Johanson, 2011:156 [distribution]. —Paprocki and França, 2014:40 [checklist].

**Distribution.** Brazil, French Guiana, Suriname.

***buborektala*** Oláh and Johanson, 2011:155 [Type locality: Peru, San Martin Prov., Rio Huallaga, at Pumarihri Huallaga Lodge, between Juan Guerra and Chazuta, 14 km (rd.) W Chazuta, 6°36.643'S, 76°12.555'W; NHRS; ♂]. —Oláh and Flint, 2012:143 [distribution].

**Distribution.** Brazil, Peru.

***cerna*** Oláh and Flint, 2012:143 [Type locality: Ecuador, Los Rios Province. Quevedo (56 km North), Rio Palenque Biological Station, el. 250 m; NMNH; ♂].

**Distribution.** Ecuador.

***darda*** Oláh and Flint, 2012:145 [Type locality: Peru, Cusco Department, Pilcopata, premontane moist forest, el. 600 m; NMNH; ♂].

**Distribution.** Ecuador, Peru.

***digitata*** Thomson and Holzenthal, 2012:21 [Type locality: Venezuela, Bolívar, E Tumeremo, W Bochinche, Río Botonamo, 07°25.462'N, 61°14.318'W, el. 150 m; UMSP; ♂].

**Distribution.** Venezuela.

***elvesta*** Oláh and Flint, 2012:146 [Type locality: Brazil, Rondonia State, creek, 8 km South Cacaulandia; NMNH; ♂].

**Distribution.** Brazil.

***fimbriata*** Flint, 1974c:54 [Type locality: Suriname, Coppename River, Raleigh Falls; RNH; ♂].

**Distribution.** Suriname.

***fluminensis*** (Santos and Nessimian), 2010b:840 [Type locality: Brazil, Rio de Janeiro, Mangarativa, Reserva Ecológica Rio das Pedras, 22°59'29.4"S, 44°06'02.6"W; DZRJ; ♂; in *Costatrichia*]. —Paprocki and França, 2014:43 [checklist]. —Santos et al., 2016a:472 [to *Acostatrichia*].

**Distribution.** Brazil.

***hosulaba*** Oláh and Flint, 2012:147 [Type locality: Ecuador, Pastaza Province, Puyo (1.5 km South); NMNH; ♂].

**Distribution.** Ecuador.

***ketvilla*** Oláh and Flint, 2012:149 [Type locality: Brazil, Pará State, Rio Xingu Camp, circa. 60 km South Altamira, 52°22'W, 3°39'S; MZUSP; ♂].

**Distribution.** Brazil.

***kihara*** Oláh and Flint, 2012:150 [Type locality: Ecuador, Napo Province, Pano, at stream, el. 580 m; NMNH; ♂].

**Distribution.** Ecuador, Venezuela.

***pika*** Oláh and Flint, 2012:151 [Type locality: Ecuador, Pichincha Province, Santo Domingo de los Colorados, 14 km East; NMNH; ♂].

**Distribution.** Ecuador.

***plaumanni*** Mosely, 1939a:228 [Type locality: Brazil, Edo. Santa Catarina, Nova Teutonia; BMNH; ♂]. —Angrisano, 1995b:505 [distribution]. —Angrisano, 1999:31 [checklist]. —Paprocki et al., 2004:10 [checklist]. —Manzo et al., 2014:166 [distribution]. —Paprocki and França, 2014:40 [checklist]. —Santos et al., 2016a:466 [♂ head].

**Distribution.** Argentina, Brazil, Uruguay.

***rovidka*** Oláh and Flint, 2012:153 [Type locality: Guyana, Moco-Moco, 30 km East Lethem, 3°18.2'N, 59°39.0'W; NMNH; ♂].

**Distribution.** Guyana.

***simulans*** Mosely, 1939a:229 [Type locality: Brazil, Edo. Santa Catarina, Nova Teutonia; BMNH; ♂]. —Angrisano, 1995b:505 [distribution]. —Angrisano, 1999:31 [checklist]. —Paprocki et al., 2004:10 [checklist]. —Angrisano and Sganga, 2010:56 [larva; pupa; case; biology]. —Paprocki and França, 2014:40 [checklist].

**Distribution.** Brazil, Uruguay.

***spinifera*** Flint, 1974c:53 [Type locality: Suriname, Nickerie River, Lombok Falls; RNH; ♂].

**Distribution.** Suriname.

***tapada*** Oláh and Flint, 2012:154 [Type locality: Venezuela, Bolivar State, Rio Caroni at Paso Caruachi; NMNH; ♂].

**Distribution.** Venezuela.

***topora*** Oláh and Flint, 2012:156 [Type locality: Panama, Barro Colorado Island, Snyder-Molino trail; NMNH; ♂]. —Armitage et al., 2015b:6 [checklist]. —Armitage and Cornejo, 2015:195 [checklist].

**Distribution.** Panama.

***tuskera*** Oláh and Flint, 2012:157 [Type locality: Brazil, São Paulo State, Piracicaba; NMNH; ♂].

**Distribution.** Brazil.

***ujasa*** Oláh and Flint, 2012:158 [Type locality: Ecuador, Pastaza Province, Puyo (27 km North), Estacion Fluviometrica; NMNH; ♂].

**Distribution.** Ecuador.

#### Genus *Alisotrichia* Flint [58 + †1]

*Alisotrichia* Flint, 1964a:46 [Type species: *Alisotrichia hirudopsis* Flint, 1964a, original designation]. —Flint, 1970:24 [revision; in Leucotrichiinae]. —Marshall 1979:183 [diagnosis; in Hydroptilinae, Leucotrichiini]. —Harris and Holzenthal, 1993:155 [phylogeny; to Hydroptilinae: Stactobiini]. —Bowles et al. 1999:51 [to Hydroptilinae, Leucotrichiini]. —Santos et al., 2016a:471 [type genus of tribe Alisotrichiini].

*Rioptila* Blickle and Denning, 1977:300 [Type species: *Rioptila arizonica* Blickle and Denning, 1977, original designation]. —Harris and Holzenthal 1993 [to synonymy].

The genus was placed in the Leucotrichiinae by Flint (1964a) and Marshall (1979), but was moved to the Stactobiinae by Harris and Holzenthal (1993). A study, especially of the larvae, by Bowles et al. (1999) returned *Alisotrichia* and related genera back to the Leucotrichiinae. Santos et al. (2016) corroborated its its monophyly and placement in the Leucotrichinae, Alisotrichiini, based on phylogenetic inference using molecular and morphological characters. Harris and Holzenthal (1993) divided the genus into eight species groups on the basis of such adult characters as spur count, antennal structure, and structure of the male genitalia. The three basal groups have now been raised to generic level as *Cerasmatrichia* (the *dominicensis* group), *Scelobotrichia* (the *quemada* group), and *Mejicanotrichia* (the *blantoni* group). The genus occurs from the southwestern United States, through Mexico south to Venezuela and throughout the Antilles. A single species is known from Dominican amber.

Larvae of the type species, *Alisotrichia hirudopsis*, were described by Flint (1964a) and larvae of other Antillean species have been described since then (Botosaneanu 1990a, 1994b, Flint 1968a, 1970). The case-less larvae are typically found on rocks in splash zones around falls and rapids or on moist rocks just above or below the water line. They undoubtedly feed on organic matter scraped from the substrate.

***aglae*** Botosaneanu, 1991a:118 [Type locality: Haiti, Département de l’Ouest, Ville Bonheur (Ville Saut d’Eau); ZMUA; ♂]. —Botosaneanu, 2002:81 [checklist]. —Flint and Pérez-Gelabert, 1999:39 [checklist]. —Flint and Sykora, 2004:26 [distribution]. —Pérez-Gelabert, 2008:300 [checklist].

**Distribution.** Dominican Republic, Haiti.

***alayoana*** Botosaneanu, 1977:256 [Type locality: Cuba, Oriente, Baire, Rio Mogote; NMNH; ♂]. —Botosaneanu, 1979:48 [distribution]. —Botosaneanu, 1994b: 455 [larva]. —Flint, 1996c:16 [checklist]. —Botosaneanu, 2002:81 [checklist]. —López del Castillo et al., 2004:229 [distribution]. —Naranjo López and González Lazo, 2005:149 [checklist].

**Distribution.** Cuba.

***aquaecadentis*** Botosaneanu, 1991a:116 [Type locality: Haiti, Département du Sud, Saut Mathurine, Rivière du Cavaillon; ZMUA; ♂; ♀]. —Botosaneanu, 2002:81 [checklist]. —Flint and Pérez-Gelabert, 1999:39 [checklist]. —Flint and Sykora, 2004:27 [distribution]. —Pérez-Gelabert, 2008:300 [checklist].

**Distribution.** Dominican Republic, Haiti.

***arcana*** Botosaneanu, 1991a:124 [Type locality: Haiti, Département du Sud, près de Camp Perrin, Résurgence du Moreau; ZMUA; ♂]. —Flint and Pérez-Gelabert, 1999:39 [checklist]. —Flint and Sykora, 2004:27 [distribution]. —Pérez-Gelabert, 2008:300 [checklist].

**Distribution.** Haiti.

***argentilinea*** Flint, 1968a:34 [Type locality: Jamaica, St. Andrew, Chestervale, Yallahs River; NMNH; ♂; ♀; larva; case]. —Flint, 1968b:81 [checklist]. —Botosaneanu, 2002:81 [checklist].

**Distribution.** Jamaica.

† ***arizela*** Wells and Wichard, 1989:43 [Type locality: Dominican Republic; collection Wichard; ♂; in amber]. —Flint and Pérez-Gelabert, 1999:39 [checklist]. —Botosaneanu, 2002:81 [checklist]. —Wichard, 2007a:48 [checklist]. —Pérez-Gelabert, 2008:300 [checklist].

**Distribution.** Dominican Republic.

***asta*** Harris and Flint, 2002:207 [Type locality: Panama, Barro Colorado Island, Snyder-Molino trail, marker 3; NMNH; ♂]. —Armitage et al., 2015b:6 [checklist]. —Armitage and Cornejo, 2015:195 [checklist].

**Distribution.** Panama.

***befoga*** Oláh and Flint, 2012:159 [Type locality: Peru, Huanuco Province, Tingo Maria, el. 672 m, premontane rain forest; NMNH; ♂].

**Distribution.** Peru.

***benji*** Rueda Martín, 2011:2 [Type locality: Argentina, Jujuy, A8 Yuto, Parque Nacional Calilegua, 23°38'40.2"S, 64°35'53.7"W, el. 505 m; IML; ♂].

**Distribution.** Argentina.

***bisetosa*** Flint and Sykora, 2004:27 [Type locality: Dominican Republic, Independence Province, Río Guyabal, 4.5 km N Poster Río, 18°34.7'N, 7137.7'W, el. 150 m; NMNH; ♂]. —Pérez-Gelabert, 2008:300 [checklist].

**Distribution.** Dominican Republic.

***cacaulandia*** Harris and Flint, 2002:200 [Type locality: Brazil, Rondonia, creek 8 km S Cacaulandia; NMNH; ♂]. —Paprocki et al., 2004:10 [checklist]. —Paprocki and França, 2014:40 [checklist].

**Distribution.** Brazil.

***cainguas*** Angrisano and Sganga, 2009:58 [Type locality: Argentina, Misiones: Parque Provincial Salto Encantado, tributary of Arroyo Cuñá-Pirú; MACN; ♂].

**Distribution.** Argentina.

***chihuahua*** Bueno-Soria and Harris, 1993:54 [Type locality: Mexico, Chihuahua, Río Concheno, ruta 16 cerca de Basaseachic; NMNH; ♂; ♀]. —Bueno-Soria et al., 2007:33 [distribution].

**Distribution.** Mexico.

***chiquitica*** Botosaneanu, 1977:258 [Type locality: Cuba, Oriente, Baracoa, Rio Jojo; NMNH; ♂; ♀]. —Botosaneanu, 1979:48 [distribution]. —Flint, 1996c:16 [checklist]. —Botosaneanu, 2002:81 [checklist]. —López del Castillo et al., 2004:229 [distribution]. —Naranjo López and González Lazo, 2005:149 [checklist].

**Distribution.** Cuba.

***chorra*** Flint, 1970:27 [Type locality: Mexico, Chiapas, El Chorreadero, 6.4 mi S. Chiapa de Corzo; NMNH; ♂]. —Bueno-Soria and Flint, 1978:200 [distribution]. —Bueno-Soria and Barba-Álvarez, 2011:356 [checklist].

**Distribution.** Mexico.

***cimarrona*** Botosaneanu, 1977:254 [Type locality: Cuba, Pinar del Rio, Soroa, Rio Manantiales; NMNH; ♂; ♀]. —Botosaneanu, 1979:48 [distribution]. —Flint, 1996c:16 [checklist]. —Botosaneanu, 2002:82 [checklist]. —Naranjo López and González Lazo, 2005:149 [checklist].

**Distribution.** Cuba.

***circinata*** Flint, 1992c:383 [Type locality: Puerto Rico, El Verde Field Station, Quebrada Prieta; NMNH; ♂]. —Botosaneanu, 2002:82 [checklist].

**Distribution.** Puerto Rico.

***cornicula*** Bueno-Soria and Harris, 1993:52 [Type locality: Mexico, Guerrero, Soyatepec; IBUNAM; ♂].

**Distribution.** Mexico.

***cuernita*** Harris and Flint, 2002:207 [Type locality: Panama, Barro Colorado Island, Snyder-Molino trail, marker 3; NMNH; ♂]. —Armitage et al., 2015b:6 [checklist]. —Armitage and Cornejo, 2015:195 [checklist].

**Distribution.** Panama.

***cyanolenos*** Flint, 1996a:91 [Type locality: Trinidad, Blue Basin Waterfall, 10°44'N, 61°32'W; NMNH; ♂]. —Botosaneanu, 2002:82 [checklist].

**Distribution.** Trinidad, Venezuela.

***euphrosyne*** Botosaneanu, 1991a:118 [Type locality: Haiti, Département de l’Ouest, Ville Bonheur (Ville Saut d’Eau); ZMUA; ♂]. —Flint and Pérez-Gelabert, 1999:39 [checklist]. —Botosaneanu, 2002:82 [checklist]. —Flint and Sykora, 2004:27 [distribution]. —Pérez-Gelabert, 2008:300 [checklist].

**Distribution.** Dominican Republic, Haiti.

***flintiana*** Botosaneanu, 1977:253 [Type locality: Cuba, Oriente, Baire, Rio Mogote; NMNH; ♂]. —Botosaneanu, 1979:48 [distribution]. —Kumanski, 1987:15 [♀]. —Botosaneanu, 1994b:455 [larva]. —Flint, 1996c:16 [checklist]. —Botosaneanu, 2002:82 [checklist].—Naranjo López and González Lazo, 2005:149 [checklist].

**Distribution.** Cuba.

***fundorai*** (Botosaneanu and Sykora), 1973:397 [Type locality: Cuba, Petit affluent du Rio Caburny, Sierra Escambray, près Topes de Collantes; NMNH; ♂; in *Oxyethira*]. —Botosaneanu, 1979:40 [♂; to *Alisotrichia*]. —Kumanski, 1987:15 [♀]. —Flint, 1996c:16 [checklist]. —Botosaneanu, 2002:82 [checklist].—Naranjo López and González Lazo, 2005:149 [checklist].

**Distribution.** Cuba.

***gabriel*** Angrisano and Burgos, 2002:108 [Type locality: Argentina, Misiones, Bernardo de Irigoyen, Cuenca del arroyo Urugua-í, Establecimento Intercontinental; MACN; ♂].

**Distribution.** Argentina

***giampaolina*** Botosaneanu, in Botosaneanu and Hyslop, 1998:10 [Type locality: Jamaica, St. Ann, Ocho Rios, Shaw Park Gardens; ZMUA; ♂; ♀]. —Botosaneanu, 2002:82 [checklist].

**Distribution.** Jamaica.

***hirudopsis aitija*** Botosaneanu, 1995:22 [Type locality: Dominican Republic, Arroyo los Guineos, on road San Francisco de Macoris to Loma; ZMUA; ♂]. —Flint and Pérez-Gelabert, 1999:39 [checklist]. —Flint and Sykora, 2004:27 [distribution]. —Pérez-Gelabert, 2008:300 [checklist].

**Distribution.** Dominican Republic.

***hirudopsis hirudopsis*** Flint, 1964a:47 [Type locality: Puerto Rico, El Yunque, stream crossing road 191 at km 6.4; NMNH; ♂; ♀; larva; pupa; case]. —Flint, 1968b:81 [checklist]. —Botosaneanu, 2002:82 [checklist]. —Santos et al., 2016a:466 [♂].

**Distribution.** Puerto Rico.

***hispaniolina*** Botosaneanu, 1991a:116 [Type locality: Haiti, Département de l’Ouest, Rivière Tombe à Mirebalais; ZMUA; ♂; ♀]. —Botosaneanu, 1995:23 [distribution]. —Flint and Pérez-Gelabert, 1999:39 [checklist]. —Botosaneanu, 2002:82 [checklist].—Flint and Sykora, 2004:27 [distribution]. —Pérez-Gelabert, 2008:300 [checklist].

**Distribution.** Dominican Republic, Haiti.

***holzenthali*** Santos, 2011:60 [Type locality: Brazil, Minas Gerais State, Santana do Riacho municipality, Cardeal Mota, Rio Cipó, Cachoeira Grande, 19°20'46.7"S, 43°38'09.7"W; DZRJ; ♂; ♀]. —Paprocki and França, 2014:40 [checklist].

**Distribution.** Brazil.

***kantala*** Oláh and Johanson, 2011:143 [Type locality: Peru, San Martin Prov., La Catarata de Ahuashiyascu, 6°27.544'S, 76°18.192'W; NHRS; ♂].

**Distribution.** Peru.

***kanukua*** Harris and Flint, 2002:200 [Type locality: Guyana, Kanuku Mountains, Moco River, 3°18.2'N, 59°38.9'W; NMNH; ♂; ♀]. —Oláh and Johanson, 2011:144 [distribution].

**Distribution.** French Guiana, Guyana.

***kevera*** Oláh and Johanson, 2011:144 [Type locality: French Guiana, Approuaguekaw, Kaw Mt, 4°33.257'N, 52°11.920'W, el. 216 m; NHRS; ♂].

**Distribution.** French Guiana.

***latipalpis*** Flint, 1991:44 [Type locality: Colombia, Dpto. Antioquia, Quebrada La Jiménez, Sopetrán; NMNH; ♂]. —Muñoz-Quesada, 2000:277 [checklist].

**Distribution.** Colombia.

***linterna*** Harris and Flint, 2002:198 [Type locality: Panama, Barro Colorado Island, Snyder-Molino trail, marker 3; NMNH; ♂]. —Armitage et al., 2015b:6 [checklist]. —Armitage and Cornejo, 2015:195 [checklist].

**Distribution.** Panama.

***lobata*** Flint, 1968b:43 [Type locality: Dominica, Clarke Hall; NMNH; ♂; ♀]. —Flint, 1968b:81 [checklist]. —Flint and Sykora, 1993:49 [checklist]. —Botosaneanu, 2002:82 [checklist]. —Botosaneanu and Thomas, 2005:55 [checklist].

**Distribution.** Dominica.

***macae*** Santos, 2011:65 [Type locality: Brazil, Rio de Janeiro State, Macaé Municipality, Rio São Pedro, 22°13'47.6"S, 42°08'04.7"W, el. 470 m; DZRJ; ♂]. —Paprocki and França, 2014:40 [checklist].

**Distribution.** Brazil.

***mathisi*** Harris and Flnt, 2002:202 [Type locality: Jamaica, St. Andrew, Mavis Bank (1.7 km E), Yal-lahs River, l8°2.4'N, 77°39.5'W, el. 575 m; ♂; ♀].

**Distribution.** Jamaica.

***muellita*** Harris and Flint, 2002:197 [Type locality: Peru, Madre de Dios, Manu, Pakitza, 11°56’ S, 71°18’ W, el. 250 m; NMNH; ♂].

**Distribution.** Peru.

***neblina*** Harris and Flint, 2002:205 [Type locality: Venezuela, Territorio Federal Amazonas, Cerro de la Neblina, basecamp, 0°50'N, 66°10'W, el. 140 m; NMNH; ♂; ♀].

**Distribution.** Venezuela.

***nessimiani*** Santos, 2011:66 [Type locality: Brazil, Rio de Janeiro State, Nova Friburgo municipality, Cascata, tributary to Rio Macaé, 22°21'54.9"S, 42°15'20.5"W, el. 391 m; DZRJ; ♂; ♀]. —Paprocki and França, 2014:41 [checklist].

**Distribution.** Brazil.

***orophila guadeloupea*** Botosaneanu, 1994a:35 [Type locality: Guadeloupe, Rivère du Grand Carbet: ZMUA; ♂]. —Botosaneanu, 2000:256 [distribution]. —Botosaneanu, 2002:82 [checklist]. —Botosaneanu and Thomas, 2005:55 [checklist].

**Distribution.** Guadeloupe.

***orophila orophila*** Flint, 1968b:41 [Type locality: Dominica, Dleau Gommier; NMNH; ♂; larva; pupa; case]. —Flint, 1968b:81 [checklist]. —Botosaneanu, 1989a:97 [distribution]. —Botosaneanu, 1990a:44 [distribution]. —Flint and Sykora, 1993:49 [checklist]. —Botosaneanu, 2002:82 [checklist]. —Botosaneanu and Thomas, 2005:37 [distribution].

**Distribution.** Dominica, Martinique.

***panamensis*** Harris and Flint, 2002:195 [Type locality: Panama, Barra Colorado Island, Canal Zone; NMNH; ♂]. —Armitage et al., 2015b:6 [checklist]. —Armitage and Cornejo, 2015:195 [checklist].

**Distribution.** Panama.

***paxilla*** Harris and Flint, 2002:204 [Type locality: Jamaica, St. Elizabeth, Elim, l8°7.1'N, 77°40.5'W; NMNH; ♂].

**Distribution.** Jamaica.

***rugoka*** Oláh and Johanson, 2011:146 [Type locality: French Guiana, Approuaguekaw, Kaw Mt, 4°33.035'N, 52°11.661'W, el. 104 m; NHRS; ♂].

**Distribution.** French Guiana.

***schmidi*** Kumanski, 1987:16 [Type locality: Cuba, Province Las Villas, massive of Guamuaya, Rio Nabujina, near El Piojillo village; NMSB; ♂; ♀]. —Flint, 1996c:16 [checklist]. —Botosaneanu, 2002:82 [checklist]. —Naranjo López and González Lazo, 2005:149 [checklist].

**Distribution.** Cuba.

***setigera*** Flint, 1992c:383 [Type locality: Puerto Rico, El Verde Field Station, Quebrada Prieta; NMNH; ♂]. —Botosaneanu, 2002:82 [checklist].

**Distribution.** Puerto Rico.

***sonora*** Bueno-Soria and Harris, 1993:51 [Type locality: Mexico, Sonora, Maycoba River, west of Maycoba; NMNH; ♂]. —Bueno-Soria et al., 2007:33 [distribution].

**Distribution.** Mexico.

***tenuivirga*** Botosaneanu, in Botosaneanu and Hyslop, 1998:10 [Type locality: Jamaica, Buff Bay River in Green Hill at “Regele”, Blue Mountains, Portland; ZMUA; ♂]. —Botosaneanu, 2002:82 [checklist].

**Distribution.** Jamaica.

***tetraespinosa*** Bueno-Soria and Harris, 1993:53 [Type locality: Mexico, Guerrero, ruta 130, 80 km N. Zihuatanejo; IBUNAM; ♂].

**Distribution.** Mexico.

***thalia*** Botosaneanu, 1991a:120 [Type locality: Haiti, Département de l’Ouest, Ville Bonhour (Ville Saut d’Eau); ZMUA; ♂]. —Flint and Pérez-Gelabert, 1999:39 [checklist]. —Botosaneanu, 2002:82 [checklist].—Flint and Sykora, 2004:29 [distribution]. —Pérez-Gelabert, 2008:300 [checklist].

**Distribution.** Dominican Republic, Haiti.

***timouchela*** Botosaneanu, 1989a:98 [Type locality: Martinique, Rivière Coco (Morne-Vert); ZMUA; ♂; p. 96; ♀; as *Bredinia* sp.]; —Botosaneanu, 1990a:44 [larva; pupa; case; synonymy of *Bredinia* sp.; distribution]. —Flint and Sykora, 1993:49 [checklist]. —Botosaneanu, 2002:82 [checklist]. —Harris and Flint, 2002:210 [distribution]. —Botosaneanu and Thomas, 2005:38 [distribution].

**Distribution.** Martinique, St. Vincent, Venezuela.

***tiza*** Harris and Holzenthal, 1993:157 [Type locality: Costa Rica, Guanacaste, Río Tizate, 7.2 km NE Cañas Dulces; NMNH; ♂].

**Distribution.** Costa Rica.

***ubatuba*** Santos, 2011:62 [Type locality: Brazil, São Paulo State, Ubatuba municipality, Rio Canoas, 23°20'18,7"S, 44°50'16,8"W, el. 475 m; DZRJ; ♂; ♀]. —Paprocki and França, 2014:41 [checklist].

**Distribution.** Brazil.

***ultima*** Flint and Sykora, 2004:29 [Type locality: Dominican Republic, Azua Province, Río Las Cuevas, 8 km NE Padre Las Casas, 18°46'N, 70°53'W, el. 580 m; CMNH; ♂]. —Pérez-Gelabert, 2008:300 [checklist].

**Distribution.** Dominican Republic.

***ventricosa*** Flint, 1991:44 [Type locality: Colombia, Dpto. Antioquia, Quebrada La Jiménez, Sopetrán; NMNH; ♂]. —Muñoz-Quesada, 2000:277 [checklist].

**Distribution.** Colombia.

***viuda*** Harris and Flint, 2002:205 [Type locality: Venezuela, Sucre, Parque Nacional Peninsula de Paria, Uquire, Rio La Viuda, 10°42.83'N, 61°57.66'W, el. 15 m; NMNH; ♂].

**Distribution.** Venezuela.

***woldai*** Harris and Flint, 2002:198 [Type locality: Panama, Barro Colorado Island, Snyder-Molino trail, marker 3; NMNH; ♂]. —Armitage et al., 2015b:6 [checklist]. —Armitage and Cornejo, 2015:195 [checklist].

**Distribution.** Panama.

***woodruffi*** Flint and Sykora, 2004:29 [Type locality: Dominican Republic, Monseñor Nouel Province [not La Vega as labeled], 6 km [not mi. as labeled] NW Rt. 1 on road to Constanza; FSCA; ♂]. —Pérez-Gelabert, 2008:300 [checklist].

**Distribution.** Dominican Republic.

#### Genus *Anchitrichia* Flint [8]

*Anchitrichia* Flint, 1970:14 [Type species: *Anchitrichia spangleri* Flint, 1970, original designation].

Among the Neotropical Leucotrichiinae, *Anchitrichia* species can be rather large, often exceeding 5 mm. The monophyly of the genus was corroborated in a recent study (Santos et al. 2016). One species is common throughout Central America and most others occur in the Andes, although some extend to lower elevations in Brazil and Paraguay. Larvae of *Anchitrichia spangleri* were described by Flint (1970) and those of *Anchitrichia duplifurcata* by Guahyba (1991). Adults occur near small to medium-sized rivers that have reaches of fast flowing water and a rocky substrate; they can also be seen swarming on streamside vegetation during the day.

***agaboga*** Oláh and Flint, 2012:161 [Type locality: Ecuador, Cotopaxi Province, Latacunga, 133 km West, el. 1080 m; NMNH; ♂].

**Distribution.** Ecuador.

***carolae*** Oláh and Flint, 2012:163 [Type locality: Venezuela, Barinas State, Rio Santo Domingo, Barinas; NMNH; ♂].

**Distribution.** Venezuela.

***duplifurcata*** Flint, 1983a:36 [Type locality: Paraguay, Dpto. Amambay, 2 km S Cerro Cora; NMNH; ♂]. —Guahyba, 1991:121 [larva; pupa; case]. —Angrisano, 1999:31 [checklist]. —Blahnik et al., 2004:4 [distribution]. —Paprocki et al., 2004:10 [checklist]. —Dumas et al., 2009:365 [distribution]. —Paprocki and França, 2014:41 [checklist]. —Santos et al., 2016a:464 [larva; pupa].

**Distribution.** Brazil, Paraguay.

***harrisi*** Oláh and Flint, 2012:164 [Type locality: Venezuela, Zulia State, El Tucuco, Sierra de Perija, montane forest; NMNH; ♂].

**Distribution.** Colombia, Venezuela.

***holzenthali*** Oláh and Flint, 2012:166 [Type locality: Ecuador, Napo Province, Rio Jondachi, 950 m, 30 km North Tena; NMNH; ♂].

**Distribution.** Ecuador.

***palmatiloba*** Flint, 1991:38 [Type locality: Colombia, Dpto. Antioquia, Río Aurrá, km 50, E San Jerónimo; NMNH; ♂]. —Muñoz-Quesada, 2000:277 [checklist].

**Distribution.** Colombia, Ecuador, Venezuela.

***spangleri*** Flint, 1970:14 [Type locality: Mexico, Chiapas, Arriaga; NMNH; ♂; larva; case]. —Bueno-Soria and Flint, 1978:200 [distribution]. —Holzenthal, 1988c:60 [distribution]. —Aguila, 1992:537 [distribution]. —Chamorro-Lacayo et al., 2007:42 [checklist]. —Bueno-Soria and Barba-Álvarez, 2011:356 [checklist]. —Armitage et al., 2015b:6 [checklist]. —Armitage and Cornejo, 2015:195 [checklist].

**Distribution.** Costa Rica, Guatemala, Honduras, Mexico, Nicaragua, Panama.

***trifurcata*** Angrisano, 1984:4 [Type locality: Argentina, Salta, Parque Nacional Baritú; MACN; ♂]. —Angrisano, 1999:31 [checklist]. —Oláh and Johanson, 2011:156 [distribution]. —Oláh and Flint, 2012:167 [distribution].

**Distribution.** Argentina, Peru.

#### Genus *Angrisanoia* Özdikmen [5]

*Paratrichia* Angrisano, 1995b:507 [Type species: Ochrotrichia (Paratrichia) cebollati, Angrisano, 1995b, original designation; as subgenus of *Ochrotrichia*]. —Flint et al., 1999b:106 [as subgenus of *Ochrotrichia*]. —Angrisano, 2002:405 [to genus status]. ­—Özdikmen, 2008:615 [preoccupied by *Paratrichia* Kelsey, 1969:320, in Diptera, Scenopinidae].

*Angrisanoia* Özdikmen, 2008:615 [Type species: Ochrotrichia (Paratrichia) cebollati Angrisano, 1995b; replacement name for *Paratrichia* Angrisano, 1995b]. —Oláh and Johanson, 2011:235 [redescription].

A small genus of Ochrotrichiinae, first established as the subgenus *Paratrichia* of *Ochrotrichia*, and later elevated to genus *Paratrichia* (Angrisano 1995b, 2002, respectively). Özdikmen (2008) transferred the type species, *Paratrichia cebolatii*, to a new genus, *Angrisanoia*, because *Paratrichia* was a junior homonym of a Diptera genus within Scenopinidae (Kelsey 1969). Oláh and Johanson (2011) added some morphological characters to the genus diagnosis. *Angrisanoia* occurs from Venezuela and French Guyana to Argentina. Larvae are unknown.

***acuti*** (Angrisano and Sganga), 2009:62 [Type locality: Argentina, Misiones, Parque Provincial Salto Encantado, Salto Acutí; MACN; ♂; in *Paratrichia*]. —Oláh and Johanson, 2011:236 [to *Angrisanoia*]. —Manzo et al., 2014:166 [distribution].

**Distribution.** Argentina.

***agazoka*** Oláh and Johanson, 2011:236 [Type locality: French Guiana, Approuaguekaw, Kaw Mt, 4°32.833'N, 52°11.452'W, el. 77 m; NHRS; ♂].

**Distribution.** French Guiana.

***cebollati*** (Angrisano), 1995b:509 [Type locality: Uruguay, Lavalleja, Rio Cebollati, Picada de Rodriguez; FHCU; ♂; in Ochrotrichia (Paratrichia)]. —Angrisano, 1999:34 [checklist]. —Angrisano, 2002:405 [to *Paratrichia*]. —Özdikmen, 2008:615 [to *Angrisanoia*]. —Oláh and Johanson, 2011:238 [checklist].

**Distribution.** Uruguay.

***lemeza*** Oláh and Johanson, 2011:238 [Type locality: French Guiana, Approuaguekaw, Kaw Mt, 4°33.035'N, 52°11.661'W, el. 104 m; NHRS; ♂].

**Distribution.** French Guiana.

***otarosa*** (Wasmund and Holzenthal), 2007:17 [Type locality: Venezuela, T. F. A. [Amazonas], Camp IV, Cerro d. l. Neblina, 0°58'N, 65°57'W, el. 760 m; NMNH; ♂; in *Rhyacopsyche*]. —Oláh and Johanson, 2011:239 [to *Angrisanoia*].

**Distribution.** Venezuela.

#### Genus *Ascotrichia* Flint [3]

*Ascotrichia* Flint, 1983a:35 [Type species: *Ascotrichia frontalis* Flint, 1983a, original designation].

This Leucotrichiinae genus, established by Flint (1983a), now contains three species from eastern South America. Larvae are unknown. Adults have been taken at light near swift flowing, larger, lowland rivers. The genus was consistently monophyletic under all assumptions in the analyses by Santos et al. (2016).

***frontalis*** Flint, 1983a:36 [Type locality: Paraguay, Dpto. Alto Paraná, Salto del Monday, near Puerto Presidente Franco; NMNH; ♂]. —Angrisano, 1995b:505 [distribution]. —Angrisano, 1999:31 [checklist]. —Paprocki et al., 2004:10 [checklist]. —Dumas et al., 2009:366 [distribution]. —Oláh and Johanson, 2011:157 [distribution]. —Paprocki and França, 2014:41 [checklist]. —Santos et al., 2016a:465 [adult photograph].

**Distribution.** Brazil, Paraguay, Uruguay.

***spangleri*** Oláh and Flint, 2012:167 [Type locality: Venezuela, Amazonas Federal Territory, Puerto Ayacucho (40km South), El Tobogan, Cano Coromoto; NMNH; ♂].

**Distribution.** Venezuela.

***surinamensis*** (Flint), 1974c:57 [Type locality: Suriname, Nickerie River, Blanche Marie; RNH; ♂; in *Betrichia*]. —Flint, 1983a:36 [to *Ascotrichia*]. —Oláh and Johanson, 2011:157 [distribution].

**Distribution.** French Guiana, Suriname.

#### Genus *Betrichia* Mosely [10]

*Betrichia* Mosely, 1939a:230 [Type species: *Betrichia zilbra* Mosely, 1939a, original designation]. —Santos et al., 2016a:472 [phylogenetic position, larva photograph]

This is a Leucotrichiine genus of ten described species, all known from eastern South America. There are no precise diagnostic characters to separate *Betrichia* from similar leucotrichiine genera (Marshall 1979). Santos et al. (2016) corroborated the placement of the genus within the Leucotrichiini clade of Leucotrichiinae, but did not support its monophyly and echoed Marshall’s observation of a lack of clear morphological delineation of the genus. Larvae of the genus are known, but not yet described. Adults commonly come to light, usually near larger, lowland rivers.

***argentinica*** Flint, 1972b:232 [Type locality: Argentina, Prov. Misiones, Capiovi; NMNH; ♂]. —Angrisano, 1995b:505 [distribution]. —Angrisano, 1999:32 [checklist]. —Thomson, 2012:2 [checklist].

**Distribution.** Argentina, Uruguay.

***bispinosa*** Flint, 1974c:59 [Type locality: Suriname, Lawa River, Anapaike; RNH; ♂]. —Thomson, 2012:2 [checklist]. —Souza et al., 2016a:295 [distribution].

**Distribution.** Brazil, Suriname.

***kagyla*** Oláh and Flint, 2012:170 [Type locality: Brazil, Amazonas State, Igarape Tarumanzinho, near Manaus; MZUSP; ♂].

**Distribution.** Brazil.

***longistyla*** Flint, 1983a:38 [Type locality: Brazil, Edo. Santa Catarina, Nova Teutonia; NMNH; ♂]. —Angrisano, 1999:32 [checklist]. —Paprocki et al., 2004:10 [checklist]. —Thomson, 2012:2 [checklist]. —Paprocki and França, 2014:42 [checklist].

**Distribution.** Brazil.

***nhundiaquara*** Souza, Santos and Takiya, 2016a:291 [Type locality: Brazil, Paraná, Morretes, Rio Nhundiaquara, 25°25'25"S, 48°54'0"W, el. 89 m; DZRJ; ♂].

**Distribution.** Brazil.

***occidentalis*** Flint, 1974c:60 [Type locality: Suriname, Blanche Marie, falls in creek; RNH; ♂]. —Oláh and Johanson, 2011:159 [distribution]. —Thomson, 2012:2 [checklist].

**Distribution.** French Guiana, Suriname.

***rovatka*** Oláh and Johanson, 2011:159 [Type locality: French Guiana, Roura, Cacao, 4°33.639'N, 52°24.629'W el. 66 m; NHRS; ♂]. —Oláh and Flint, 2012:171 [distribution]. —Thomson, 2012:2 [checklist].

**Distribution.** Ecuador, French Guiana.

***uruguayensis*** Angrisano, 1995b:505 [Type locality: Uruguay, Paysandu, Sta. Rita, Puerto Pepeaji; FHCU; ♂]. —Angrisano, 1999:32 [checklist]. —Oláh and Flint, 2012:171 [distribution]. —Thomson, 2012:2 [checklist].

**Distribution.** Brazil, Uruguay.

***varratlana*** Oláh and Flint, 2012:171 [Type locality: Brazil, Rondonia State, creek 8 km South Cacaulandia; NMNH; ♂].

**Distribution.** Brazil, Guyana.

***zilbra*** Mosely, 1939a:231 [Type locality: Brazil, Edo. Santa Catarina, Nova, Teutonia; BMNH; ♂]. —Angrisano, 1995b:505 [distribution]. —Angrisano, 1999:32 [checklist]. —Paprocki et al., 2004:10 [checklist]. —Oláh and Flint, 2012:173 [distribution]. —Thomson, 2012:2 [checklist]. —Paprocki and França, 2014:42 [checklist]. —Souza et al., 2016a:293 [♂; distribution].

**Distribution.** Argentina, Brazil, Guyana, Uruguay.

#### Genus *Bredinia* Flint [16]

*Bredinia* Flint, 1968b:50 [Type species: *Bredinia dominicensis* Flint, 1968b, original designation]. — Angrisano, 2002:398 [larva]. —Harris et al., 2002c:14 [revision].

*Bredinia* is a genus currently restricted to the Neotropics and placed in the Stactobiinae. Members of the genus are minute in size and gray in coloration. Adults are attracted to lights along larger rivers. Immature stages were described by Angrisano (2002). The larvae and cases show no distinctive characters and they are of the “primitive form” among the Hydroptilidae (Angrisano, 2002).

***alza*** Harris, Holzenthal and Flint, 2002c:35 [Type locality: Paraguay, Concepción, Concepción; NMNH; ♂]. —Angrisano and Sganga, 2007:28 [♂; larva; distribution].

**Distribution.** Argentina, Paraguay.

***appendiculata*** Flint and Sykora, 1993:56 [Type locality: Grenada, Parish St. Andrews, Balthazar Estate; FSCA; ♂]. —Flint and Sykora, 1993:49 [checklist]. —Botosaneanu, 2002:82 [checklist]. —Harris et al., 2002c:22 [♂; ♀; redescription; distribution].

**Distribution.** Grenada, Peru, Venezuela.

***costaricensis*** (Flint), 1967b:13 [Type locality: Costa Rica, La Lola near Martina; NMNH; ♂; in *Neotrichia*]. —Holzenthal, 1988c:62 [distribution]. —Flint et al., 1999a:76 [to *Bredinia*]. —Harris et al., 2002c:24 [♂; ♀; redescription; distribution]. —Armitage et al., 2015b:6 [checklist]. —Armitage and Cornejo, 2015:195 [checklist].

**Distribution.** Costa Rica, Panama.

***davenporti*** Harris, Holzenthal and Flint, 2002c:24 [Type locality: Peru, Loreto, Río Sucusari at Explornapo Camp; NMNH; ♂].

**Distribution.** Peru.

***dominicensis*** Flint, 1968b:51 [Type locality: Dominica, Hodges River mouth, swamp forest; NMNH; ♂; ♀]. —Flint and Sykora, 1993:49 [checklist]. —Flint, 1996a:90 [distribution]. —Botosaneanu, 2002:82 [checklist]. —Harris et al., 2002c:15 [♂; ♀; redescription; distribution]. —Botosaneanu and Thomas, 2005:38 [distribution]. —Armitage et al., 2015b:6 [checklist]. —Armitage and Cornejo, 2015:195 [checklist].

**Distribution.** Costa Rica, Dominica, Ecuador, Martinique, Panama, Trinidad.

***emarginata*** Harris, Holzenthal and Flint, 2002c:37 [Type locality: Costa Rica, Alajuela, Río Pizote, ca 5 km N Dos Ríos, 10.948°N, 85.291°W; NMNH; ♂].

**Distribution.** Costa Rica.

***espinosa*** Harris, Holzenthal and Flint, 2002c:20 [Type locality: Ecuador, Los Ríos, Quevedo (56 km N), Río Palenque Biological Station; NMNH; ♂; ♀]. —Paprocki et al., 2004:11 [checklist]. —Nogueira and Cabette, 2011:351 [distribution]. —Oláh and Johanson, 2011:248 [distribution]. —Paprocki and França, 2014:42 [checklist].

**Distribution.** Brazil, Ecuador, French Guiana, Venezuela.

***guanacasteca*** Harris, Holzenthal and Flint, 2002c:17 [Type locality: Costa Rica, Guanacaste, Río Tempisquito, ca 3 km S route 1, 10.790°N, 85.552°W, el. 75 m; NMNH; ♂].

**Distribution.** Costa Rica.

***manabiensis*** Harris, Holzenthal and Flint, 2002c:27 [Type locality: Ecuador, Manabi, 29 km W Santo Domingo, Rancho Ronald; NMNH; ♂].

**Distribution.** Ecuador.

***mexicana*** Harris, Holzenthal and Flint, 2002c:35 [Type locality: Mexico, Tamaulipas, Río Frio at La Poza Azul near Gómez Farias; NMNH; /male; ♀].

**Distribution.** Mexico.

***pilcopata*** Harris, Holzenthal and Flint, 2002c:32 [Type locality: Peru, Cuzco, Pilcopata, el. 600 m; NMNH; ♂; ♀]. —Oláh and Johanson, 2011:248 [distribution].

**Distribution.** Peru.

***selva*** Harris, Holzenthal and Flint, 2002c:19 [Type locality: Costa Rica, Heredia, Estación Biológica La Selva; NMNH; ♂].

**Distribution.** Costa Rica.

***spangleri*** Harris, Holzenthal and Flint, 2002c:34 [Type locality: Ecuador, Pastaza, Puyo (16 km W); NMNH; ♂].

**Distribution.** Ecuador.

***sucrensis*** Harris, Holzenthal and Flint, 2002c:37 [Type locality: Venezuela, Sucre, Parque Nacional Peninsula de Paria, Uquire, Río La Viuda, 10°42.830'N, 61°57.661'W, el. 15 m; NMNH; /male; ♀].

**Distribution.** Venezuela.

***venezuelensis*** Harris, Holzenthal and Flint, 2002c:29 [Type locality: Venezuela, Zulia, Perija El Tucuco, Mission El Tucuco, Río El Tucuco, 11 km from church; NMNH; ♂; ♀]. —Oláh and Johanson, 2011:248 [distribution].

**Distribution.** Ecuador, Peru, Venezuela.

***zulia*** Harris, Holzenthal and Flint, 2002c:39 [Type locality: Venezuela, Zulia, El Tucuco, Sierra de Perija; NMNH; ♂].

**Distribution.** Venezuela.

#### Genus *Byrsopteryx* Flint [16]

*Byrsopteryx* Flint, 1981a:27 [Type species: *Byrsopteryx mirifica* Flint, 1981a, original designation]. —Harris and Holzenthal, 1994:154 [revision, placement]. —Bowles et al., 1999:45 [placement]. —Santos et al., 2016a:471 [phylogenetic placement].

The genus *Byrsopteryx* was originally placed in the Leucotrichiinae, transferred to the Stactobiinae (Harris and Holzenthal 1994), and returned to the Leucotrichiinae (Bowles et al. 1999). Santos et al. (2016) established the monophyly of the genus and refined its position even further as a member of the Leucotrichiinae, tribe Alisotrichiini. The adults of the genus are small, but easily recognized by the bright white or greenish-white spots on the dark body and forewings. All known species are found in southern Central America, South America, and the Lesser Antilles.

Adults are day-active, running about over streamside rocks, boulders, and low vegetation. As they rarely fly to collecting lights at night, they are often poorly represented in collections. Larvae of *Byrsopteryx mirifica* and *Byrsopteryx carioca* were described by Holzenthal and Harris (1991) and Santos and Nessimian (2010c), respectively. These authors also described the immatures of *Byrsopteryx abrelata* and *Byrsopteryx espinhosa*. Larvae build portable cases and are madicolous, occurring in the spray and splash zones of small waterfalls and mountain streams, where they apparently feed by scraping diatoms and associated periphyton from the substrate.

***abrelata*** Harris and Holzenthal, 1994:157 [Type locality: Brazil, Rio de Janeiro, Nova Friburgo, municipal water supply; MZUSP; ♂; ♀]. —Blahnik et al., 2004:4 [distribution]. —Paprocki et al., 2004:11 [checklist]. —Dumas et al., 2009:366 [distribution]. —Santos and Nessimian, 2010c:52 [larva; pupa; case]. —Dumas and Nessimian, 2012:15 [checklist]. —Paprocki and França, 2014:42 [checklist]. —Santos et al., 2016a:465 [adult photograph]

**Distribution.** Brazil.

***bipartiterga*** Botosaneanu, 2000:252 [Type locality: Guadeloupe, Parc National de la Guadeloupe, La Deuxieme Chute du Carbet, el. 580 m; ZMUA; /female]. —Botosaneanu, 2002:82 [checklist].

**Distribution.** Guadeloupe.

***carioca*** Santos and Nessimian, 2010c:45 [Type locality: Brazil, Rio de Janeiro State, Rio de Janeiro, Floresta da Tijuca: Parque Nacional da Tijuca, Rio Humaitá, 22°57'30.1"S 43°17'21.4"W, el. 475 m; DZRJ; ♂; ♀; larva; pupa; biology]. —Paprocki and França, 2014:42 [checklist]. —Santos et al., 2016a:466 [♂].

**Distribution.** Brazil.

***chaconi*** Harris and Holzenthal, 1994:160 [Type locality: Costa Rica, Puntarenas, roadside seep, route 2, just W km 234, 8.976°N, 83.299°W; NMNH; ♂; ♀].

**Distribution.** Costa Rica.

***cuchilla*** Harris and Holzenthal, 1994:164 [Type locality: Costa Rica, Cartago, Chitaria; NMNH; ♂; ♀].

**Distribution.** Costa Rica.

***esparta*** Harris and Holzenthal, 1994:163 [Type locality: Costa Rica, Puntarenas, 14.1 mi SE Esparta; NMNH; ♂].

**Distribution.** Costa Rica.

***espinhosa*** Harris and Holzenthal, 1994:164 [Type locality: Brazil, Rio de Janeiro, km 17, 18 km S Teresopolis; MZUSP; ♂]. —Paprocki et al., 2004:11 [checklist]. —Dumas et al., 2009:366 [distribution]. —Santos and Nessimian, 2010c:52 [♀; larva; pupa; case]. —Paprocki and França, 2014:43 [checklist]. —Santos et al., 2016a:464 [larva photograph].

**Distribution.** Brazil.

***gomezi*** Harris and Holzenthal, 1994:164 [Type locality: Costa Rica, Puntarenas, Río Bellavista, ca. 1.5 km NW las Alturas, 8.951°N, 82.846°W; NMNH; ♂; ♀].

**Distribution.** Costa Rica.

***loja*** Harris and Holzenthal, 1994:167 [Type locality: Ecuador, Zamora-Chinchipe, 30 km E Loja; NMNH; ♂; ♀].

**Distribution.** Ecuador.

***mirifica*** Flint, 1981a:27 [Type locality: Venezuela, Aragua, Maracay, Río Limón, Estación Piscicultura; NMNH; ♂; ♀; larva; case]. —Holzenthal and Harris, 1991:405 [♂; ♀ larva; case]. —Harris and Holzenthal, 1994:170 [♂; ♀].

**Distribution.** Venezuela.

***rayada*** Harris and Holzenthal, 1994:172 [Type locality: Ecuador, Cañar, Río Chauchas, 3km N Zhud; NMNH; ♂; ♀].

**Distribution.** Ecuador.

***septempunctata*** (Flint), 1968b:46 [Type locality: Dominica, Pont Casse, 2.2mi E; NMNH; ♂; in *Alisotrichia*]. —Flint, 1981a:27 [to *Byrsopteryx*]. —Flint and Sykora, 1993:49 [checklist]. —Harris and Holzenthal, 1994:172 [♂]. —Botosaneanu, 2000:254 [♂; ♀]. —Botosaneanu, 2002:82 [checklist]. —Botosaneanu and Thomas, 2005:55 [checklist].

**Distribution.** Dominica, Guadaloupe.

***solisi*** Harris and Holzenthal, 1994:175 [Type locality: Costa Rica, Puntarenas, Río Singrí, 2 km (air) S Finca Helechales, 9.057°N, 83.082°W; NMNH; ♂; ♀].

**Distribution.** Costa Rica.

***tabasquensis*** Bueno-Soria, Santiago-Fragoso, and Barba-Álvarez, 2001:146 [Type locality: Mexico, Tabasco, Municipio de Huimanguillo, Arroyo las Flores, Villa de Guadalupe 2a sección Los Chimalapas, km 5 Ruta Malpasito–Carlos A. Madrazo, 17°22'05"N, 93°36'25"W; CNIN; ♂]. —Bueno-Soria et al., 2005:75 [distribution].

**Distribution.** Mexico.

***tapanti*** Harris and Holzenthal, 1994:177 [Type locality: Costa Rica, Cartago, Res. Tapantí, Quebrada Palmitos and falls, 9.72°N, 83.78°W; NMNH; ♂; ♀].

**Distribution.** Costa Rica.

***tica*** Harris and Holzenthal, 1994:179 [Type locality: Costa Rica, Res. Tapantí, unnamed tributary, ca. 8 km (rd.) S headquarters, 9.76°N, 83.78°W; NMNH; ♂; ♀].

**Distribution.** Costa Rica.

#### Genus *Celaenotrichia* Mosely [1]

*Celaenotrichia* Mosely, 1934b:158 [Type species: *Celaenotrichia edwardsi* Mosely 1934b, original designation]. —Harris and Flint, 1993:101 [redescription; placement]. —Santos et al., 2016a:471 [phylogenetic position].

Mosely (1934b) established the monotypic genus *Celaenotrichia* for *Celaenotrichia edwardsi*, collected on Chiloe Island in southern Chile. The species has since been collected at several other localities in Chile as well as in Argentina. The genus was placed in the Leucotrichiinae by Marshall (1979), transferred to the Stactobiinae (Harris and Flint 1993), and returned to the Leucotrichiinae (Bowles et al. 1999). Its placement has since been further defined to Leucotrichiinae, Alisotrichiini (Santos et al. 2016a).

Adults are day active, occurring on rocks adjacent to swift flowing streams. Larvae of *Celaenotrichia edwardsi* were described by Harris and Flint (1993). They build portable, purse cases and are found on the rocky substrate of swift flowing streams where they apparently feed as scrapers or grazers.

***edwardsi*** Mosely, 1934b:158 [Type locality: Chile, Chiloe Island, Castro; BMNH; ♂]. —Flint, 1974e:87 [checklist]. —Harris and Flint, 1993:101 [♂; ♀; larva; case; distribution]. —Angrisano, 1999:32 [checklist].

**Distribution.** Argentina, Chile.

#### Genus *Cerasmatrichia* Flint, Harris and Botosaneanu [8]

*Cerasmatrichia* Flint, Harris and Botosaneanu, 1994:360 [Type species: *Cerasmatrichia trinitatis* Flint, Harris and Botosaneanu 1994, original designation]. —Santos et al., 2016a:464 [larva photograph].

This genus was established for the *dominicensis* group, once included in *Alisotrichia*. The genus was originally placed in the tribe Stactobiinae, but has now been transferred to the Leucotrichiinae, Alisotrichiini and its monophyly has been established (Bowles et al. 1999, Santos et al. 2016a). *Cerasmatrichia* is known to occur from Costa Rica south to Peru, east to Trinidad and throughout the Lesser Antilles.

Larvae have been described for *Cerasmatrichia spinosa* and are known for several additional species in the genus (Flint et al. 1994). The free-living larvae are found on rocks and boulders in splash zones or at the water’s edge, where they remain damp. They occur in small to medium sized rivers, but the adults are seldom attracted to light.

***adunca*** (Flint), 1991:44 [Type locality: Colombia, Dpto. Antioquia 10 km E Medellín, road to Guarne; NMNH; ♂; in *Alisotrichia*]. —Flint et al., 1994:377 [♂; ♀; to *Cerasmatrichia*]. —Muñoz-Quesada, 2000:277 [checklist]. —Oláh and Johanson, 2011:148 [distribution; in synonymy as *Rioptila*].

**Distribution.** Colombia, Peru.

***argylensis*** Flint, Harris and Botosaneanu, 1994:370 [Type locality: Tobago, St. Paul Parish, Argyle River at Argyle Waterfall; ZMUA; ♂; ♀]. —Botosaneanu, 2002:82 [checklist].

—Hydroptilid genus, sp. 2, Botosaneanu and Sakal, 1992:201. —Botosaneanu and Alkins-Koo, 1993:14 [distribution]. —Flint et al., 1994:370 [to synonymy].

**Distribution.** Tobago, Trinidad.

***dominicensis*** (Flint), 1968b:44 [Type locality: Dominica, 2.2 mi E Pont Casse; NMNH; ♂; in *Alisotrichia*]. —Flint, 1968b:81 [checklist]. —Botosaneanu, 1989a:97 [distribution]. —Flint and Sykora, 1993:49 [checklist]. —Flint et al., 1994:369 [♂; ♀; to *Cerasmatrichia*]. —Botosaneanu, 2000:256 [distribution]. —Botosaneanu, 2002:83 [checklist]. —Botosaneanu and Thomas, 2005:38 [probable, distribution].

—Ochtrotrichia (Ochtrotrichia) species, Flint and Sykora, 1993:58 [misidentification]. —Flint et al., 1994:369 [to synonymy].

**Distribution.** Dominica, Guadeloupe, Martinique [?].

***fulika*** Oláh and Johanson, 2011:150 [Type locality: French Guiana, Approuaguekaw, Kaw Mt, 4°33.035'N, 52°11.661'W, el. 104 m; NHRS; ♂].

**Distribution.** French Guiana.

***hidala*** Oláh and Johanson, 2011:148 [Type locality: Peru, San Martin Prov., creek crossing rd. Tarapoto-Yurimaguas, ca. 30 km (rd.) NE Tarapoto, 6°24.904'S, 76°18.756'W; NHRS; ♂].

**Distribution.** Peru.

***spinosa*** Flint, Harris and Botosaneanu, 1994:368 [Type locality: Venezuela, Edo. Aragua, Rio El Limón, fish hatchery, Maracay; NMNH; ♂; ♀; larva; case]. —Flint, 1981a:26 [in part, misidentification of *Alisotrichia wirthi* material from Río El Limón].

**Distribution.** Venezuela.

***trinitatis*** Flint, Harris and Botosaneanu, 1994:374 [Type locality Trinidad, St. George County, Northern Range, Maracas Waterfall; ZMUA; ♂; ♀]. —Flint, 1996a:90 [distribution]. —Botosaneanu, 2002:83 [checklist].

—Hydroptilid genus, sp. 1, Botosaneanu and Sakal, 1992:201. —Botosaneanu and Alkins-Koo, 1993:14 [distribution]. —Flint et al., 1994:374 [to synonymy].

**Distribution.** Trinidad, Venezuela.

***wirthi*** (Flint), 1968b:46 [Type locality: Dominica, Fond Figues River; NMNH; ♂; in *Alisotrichia*]. —Flint et al., 1994:374 [♂; ♀; to *Cerasmatrichia*]. —Botosaneanu, 2002:83 [checklist]. —Botosaneanu and Thomas, 2005:38 [probable, distribution].

**Distribution.** Dominica, Guadeloupe, Martinique [?], Venezuela.

#### Genus *Ceratotrichia* Flint [5]

*Ceratotrichia* Flint, 1992b:527 [Type species: *Ceratotrichia fairchildi* Flint 1992b, original designation]. —Pes and Hamada, 2004:32 [larva; pupa; taxonomic remarks; distribution].

This genus was established for two unusual species, one from Panama (*Ceratotrichia fairchildi*) the other northern South America (*Ceratotrichia flavicoma*). Later, another three species were described from Bolivia and Ecuador. As typical for other Leucotrichiinae genera, it is defined primarily on male secondary sexual features, but its monophyly has been confirmed (Santos et al. 2016a). Adults have been taken at lights near fast flowing small to large rivers usually in densely forested regions.

Pes and Hamada (2004) described the immature stages of an unidentified species and recorded the genus for the first time from Brazil. The larvae were more common in the turbulent flow of rapids and waterfalls, and were found in association with species of *Zumatrichia*, *Alisotrichia*, and *Leucotrichia* in these situations (Pes and Hamada 2004).

***balra*** Oláh and Johanson, 2011:157 [Type locality: Bolivia, Hung. Soil. Zool. Exp. II, S. Amer. No.B-B: No. 493, Alcoche (La Paz), surroundings of Hotel, el. 600 m; HNHM; ♂].

**Distribution.** Bolivia.

***fairchildi*** Flint, 1992b:528 [Type locality: Panama, Comarca of San Blas, Quebrada Pingandi, 9 km N Nusagandi; NMNH; ♂]. —Aguila, 1992:537 [distribution]. —Armitage et al., 2015b:6 [checklist]. —Armitage and Cornejo, 2015:195 [checklist].

**Distribution.** Panama.

***felgorba*** Oláh and Flint, 2012:173 [Type locality: Ecuador, Napo Province, Pano, el. 580 m; NMNH; ♂].

**Distribution.** Ecuador.

***flavicoma*** Flint, 1992b:529 [Type locality: Venezuela, State of Barinas, Puente Parangula, 8 km S Barinitas; NMNH; ♂]. —Flint, 1996b:396 [distribution].

**Distribution.** Ecuador, Peru, Venezuela.

***jobbra*** Oláh and Flint, 2012:174 [Type locality: Ecuador, Manabi Province, 29 km West Santo Domingo, Rancho Ronald; NMNH; ♂].

**Distribution.** Ecuador.

#### Genus *Costatrichia* Mosely [14]

*Costatrichia* Mosely, 1937:166 [Type species: *Costatrichia lodora* Mosely 1937, original designation]. —Flint, 1970:11 [revision]. —Holzenthal and Harris, 1999:540 [revision; key to species].

The leucotrichiine genus *Costatrichia* was originally erected for a single Mexican species and now contains 14 species. Distribution is from Mexico through Central America, south to Argentina, Paraguay and Uruguay. Santos et al. (2016) concluded that the genus could not be unequivocally defined by morphological characters and it was not recovered as monophyletic in their phylogenetic analyses.

The immature stages are unknown. Adults have been taken, but sparsely, at lights placed near flowing water, although *Costatrichia simplex* has also been taken near a large lake.

***bipartita*** Flint, 1970:12 [Type locality: Nicaragua, Chontales, Puente Quinama, near Villa Somoza; NMNH; ♂]. —Maes and Flint, 1988:4 [distribution]. —Holzenthal and Harris, 1999:564 [♂]. —Maes, 1999:1193 [checklist]. —Chamorro-Lacayo et al., 2007:42 [checklist].

**Distribution.** Nicaragua.

***carara*** Holzenthal and Harris, 1999:552 [Type locality: Costa Rica, San José, Reserva Bio­lógica Carara, Río del Sur, 1.5 km (rd) S Carara, 9.769°N, 84.531°W; NMNH; ♂; ♀].

**Distribution.** Costa Rica.

***cressae*** Holzenthal and Harris, 1999:555 [Type locality: Venezuela, Distrito Federal, Río Camuri Grande, 1 km S Camuri (nucleo U.S.B.), 10.616°N, 66.175°W; NMNH; ♂; ♀].

**Distribution.** Venezuela.

***flinti*** Holzenthal and Harris, 1999:545 [Type locality: Costa Rica, Puntarenas, Río Singrí, ca. 2 km (air) S Finca Helechales, 9.057°N, 83.082°W; NMNH; ♂].

**Distribution.** Costa Rica.

***hamulifera*** (Flint), 1983a:38 [Type locality: Argentina, Pcia. Entre Rios, Rio Uruguay, Salto Grande; NMNH; ♂; in *Betrichia*]. —Angrisano, 1995b:505 [distribution]. —Angrisano, 1999:32 [checklist]. —Angrisano and Sganga, 2007:30 [♂; distribution]. —Calor, 2011:321 [checklist]. —Oláh and Johanson, 2011:159 [distribution]. —Oláh and Flint, 2012:170 [distribution]. —Thomson, 2012:2 [checklist]. —Souza et al., 2013b:585 [distribution]. —Paprocki and França, 2014:42 [checklist]. —Santos et al., 2016a:472 [to *Costatrichia*].

**Distribution.** Argentina, Brazil, French Guiana, Paraguay, Uruguay.

***ipixuna*** Santos, Takiya and Nessimian, 2013:448 [Type locality: Brazil, Amazonas, Ipixuna, Rio Liberdade, Comunidade São Vicente, 07°21'47"S, 71°52'07"W, el. 175 m; INPA; ♂; ♀]. —Paprocki and França, 2014:43 [checklist]. —Santos et al., 2016a:466 ♂ antennae, ♀].

**Distribution.** Brazil.

***lodora*** Mosely, 1937:168 [Type locality: Mexico, Chiapas, Dolores; BMNH; ♂]. —Flint, 1970:12 [♂; distribution]. —Bueno-Soria and Flint, 1978:200 [distribution]. —Holzenthal, 1988c:60 [distribution]. —Holzenthal and Harris, 1999:541 [♂; distribution]. —Bueno-Soria et al., 2005:75 [distribution]. —Chamorro-Lacayo et al., 2007:42 [checklist]. —Bueno-Soria and Barba-Álvarez, 2011:356 [checklist].

**Distribution.** Costa Rica, Mexico, Nicaragua.

***nelsonferreirai*** Santos and Nessimian, 2010b:838 [Type locality: Brazil, Pará, Canaã dos Carajás (Floresta Nacional – FLONA – de Carajás, lagoa Redonda, 06°21'20.7"S, 50°23'26.7"W, el. 705 m; DZRJ; ♂; ♀]. —Paprocki and França, 2014:43 [checklist].

**Distribution.** Brazil.

***noite*** Angrisano, 1995b:507 [Type locality: Uruguay, Tacuarembo, Ao. Laureles, Rincón de la Vasoura; FHCU; ♂]. —Angrisano, 1999:32 [checklist]. —Holzenthal and Harris, 1999:540, 564 [♂; distribution]. —Oláh and Flint, 2012:176 [distribution]. —Santos et al., 2013:450 [distribution]. —Paprocki and França, 2014:43 [checklist].

**Distribution.** Brazil, Ecuador, Paraguay, Peru, Uruguay.

***panamensis*** Flint, 1967b:11 [Type locality: Panama, Canal Zone, Río Agua Salud; NMNH; ♂]. —Flint, 1970:12 [♂]. —Aguila, 1992:538 [distribution]. —Holzenthal and Harris, 1999:568 [♂]. —Armitage et al., 2015b:6 [checklist]. —Armitage and Cornejo, 2015:195 [checklist].

**Distribution.** Panama.

***simplex*** Flint, 1970:13 [Type locality: El Salvador, San Salvador, Lake Ilopango, near Apulo; NMNH; ♂]. —Bueno-Soria and Flint, 1978:200 [distribution]. —Holzenthal, 1988c:61 [distribution]. —Holzenthal and Harris, 1999:545 [♂; ♀; distribution]. —Chamorro-Lacayo et al., 2007:42 [checklist]. —Bueno-Soria and Barba-Álvarez, 2011:356 [checklist].

**Distribution.** Costa Rica, El Salvador, Honduras, Mexico, Nicaragua.

***spinifera*** Flint, 1970:13 [Type locality: Panama, Canal Zone, Río Agua Salud, Pipeline Road; NMNH; ♂]. —Aguila, 1992:538 [distribution]. —Holzenthal and Harris, 1999:558 [♂; distribution]. —Armitage et al., 2015b:6 [checklist]. —Armitage and Cornejo, 2015:195 [checklist].

**Distribution.** Costa Rica, Panama.

***tripartita*** Flint, 1970:13 [Type locality: Panama, Canal Zone, Río Aqua Salud, Pipeline Road; NMNH; ♂]. —Aguila, 1992:538 [distribution]. —Holzenthal and Harris, 1999:549 [♂; ♀; distribution]. —Armitage et al., 2015b:6 [checklist]. —Armitage and Cornejo, 2015:195 [checklist].

**Distribution.** Costa Rica, Panama.

***venezuelensis*** Flint, 1981a:25 [Type locality: Venezuela, Aragua, Maracay, Río Limón, Estación Piscicultura; NMNH; ♂; as subspecies of *tripartita*]. —Holzenthal and Harris, 1999:558 [new status; diagnosis; ♂; distribution]. —Oláh and Flint, 2012:176 [distribution].

**Distribution.** Costa Rica, Venezuela.

#### Genus *Dicaminus* Müller [1]

*Dicaminus* Müller, 1879a:39 [Type species: no included species, but *Dicaminus* replacing *Diaulus* gets its type species, *ladislavii*.]. —Ulmer, 1957:172 [references].

*Diaulus* Müller 1879b:142 [Type species: *Diaulus ladislavii* Müller 1879b, by monotypy]. —Ulmer, 1957:173 [to synonymy].

Müller (1879a) described a number of unusual larval cases with small dorsal chimneys from Brazil, under the generic name of *Dicaminus*, although neither a description of the larvae or a species name was given. In a subsequent paper, Müller (1879b) refers to the same cases as *Diaulus ladislavii*. Ulmer (1957) synonymized *Diaulus* with *Dicaminus*, which has priority. Botosaneanu and Flint (1982) examined a number of cases having two chimneys from the dorsal surface from Panama, Ecuador, Bolivia, Argentina, and Venezuela, some of which contained male metamorphotypes of species of *Metrichia*. As these cases match those described by Müller, *Dicaminus* is possibly synonymous with *Metrichia* or at least closely related, although such a placement is, as yet, unproven.

***ladislavii*** Müller, 1879b:142 [Type locality: South Brazil; MNRJ; case]. —Müller, 1880a:118 [case; figures, type locality: [Brazil], Santa Catarina, Ribeirão dos Bugres, tributary of Itajahy]. —Ulmer, 1957:172 [complete references]. —Angrisano, 1999:32 [checklist]. —Paprocki et al., 2004:11 [checklist]. —Paprocki and França, 2014:44 [checklist].

**Distribution.** Brazil.

#### Genus *Flintiella* Angrisano [15]

*Flintiella* Angrisano, 1995b:502 [Type species: *Flintiella andreae* Angrisano, 1995b, original designation]. —Harris et al., 2002b:66 [revision].

The genus *Flintiella*, placed in the Stactobiinae, was established for a species from Uruguay and Argentina. Since then, additional species have been described throughout the tropical regions of the Neotropics. Both females and larvae were described by Angrisano (1995b). They come to lights placed near small to large rivers.

***alajuela*** Harris, Flint and Holzenthal, 2002b:66 [Type locality: Costa Rica, Alajuela, Rio Pizote, ca. 5 km N Dos Rios, 10.948°N, 85.291°W, el. 40 m; NMNH; ♂].

**Distribution.** Costa Rica.

***andreae*** Angrisano, 1995b:503 [Type locality: Uruguay, Artigas, Ao. de la Invernada; FHCU; ♂; ♀; larva; case]. —Angrisano, 1999:32 [checklist]. —Harris et al., 2002b:75 [♂; ♀; redescription]. —Angrisano and Sganga, 2007:28 [♂; ♀; larva; pupa; distribution]. —Souza et al., 2013b:585 [distribution]. —Paprocki and França, 2014:44 [checklist].

**Distribution.** Argentina, Brazil, Uruguay.

***astilla*** Harris, Flint and Holzenthal, 2002b:69 [Type locality: Venezuela, Amazonas, Rio Cataniapo, 10 km S Puerto Ayacucho; NMNH; ♂; ♀]. —Calor, 2011:321 [checklist]. —Nogueira and Cabette, 2011:351 [distribution]. —Paprocki and França, 2014:44 [checklist].

**Distribution.** Brazil, Costa Rica, Ecuador, Paraguay, Peru, Venezuela.

***boraceia*** Harris, Flint and Holzenthal, 2002b:69 [Type locality: Brazil, São Paulo, Estacion Biologica Boracéia; MZUSP; ♂]. —Paprocki et al., 2004:11 [checklist]. —Calor, 2011:321 [checklist]. —Paprocki and França, 2014:44 [checklist].

**Distribution.** Brazil.

***carajas*** Santos, Jardim and Nessimian, 2011:813 [Type locality: Brazil, Pará, Parauapebas (Floresta Nacional de Carajás, small stream, 06°04'57"S, 50°08'05"W, el. 642 m); DZRJ; ♂; ♀]. —Paprocki and França, 2014:44 [checklist].

**Distribution.** Brazil.

***harma*** Oláh and Johanson, 2011:248 [Type locality: French Guiana, Approuaguekaw, Kaw Mt, 4°32.833'N, 52° 11.452'W, el. 77 m; NHRS; ♂].

**Distribution.** French Guiana.

***harrisi*** Souza, Santos and Takiya, 2016b:341 [Type locality: Brazil, Piauí, Piracuruca, Parque Nacional de Sete Cidades, Riacho Piedade, Pennsylvania trap, 04°06'34"S, 41°4'39"W, el. 169 m; CZMA; ♂].

**Distribution.** Brazil.

***heredia*** Harris, Flint and Holzenthal, 2002b:77 [Type locality: Costa Rica, Heredia, Rio Bijagual on road to Magsasay, 10.408°N, 84.076°W, el. 140 m; NMNH; ♂; ♀].

**Distribution.** Costa Rica, Ecuador, Peru.

***leloga*** Oláh and Johanson, 2011:250 [Type locality: French Guiana, Approuaguekaw, Kaw Mt, 4°33.035'N, 52°11.661'W, el. 104 m; NHRS; ♂].

**Distribution.** French Guiana.

***manauara*** Santos and Nessimian, 2009a:65 [Type locality: Brazil, Amazonas, Manaus, tributary to Rio Branquinho, 02°31'24.6"S 60°20'05.3"W; INPA; ♂; ♀]. —Paprocki and França, 2014:45 [checklist].

**Distribution.** Brazil.

***pallida*** Souza, Santos and Takiya, 2016b:341 [Type locality: Brazil, Maranhão, Carolina, Parque Nacional da Chapada das Mesas, Riacho Cancela, Malaise trap, 07°06'43.4"S, 47°17'16.6"W, el. 186 m; CZMA; ♂].

**Distribution.** Brazil.

***panamensis*** Harris, Flint and Holzenthal, 2002b:79 [Type locality: Panama, Panama, Barro Colorado Island, Snyder-Molino trail; NMNH; ♂]. —Armitage et al., 2015b:6 [checklist]. —Armitage and Cornejo, 2015:195 [checklist].

**Distribution.** Panama.

***pizotensis*** Harris, Flint and Holzenthal, 2002b:73 [Type locality: Costa Rica, Limon, Rio Telire and small tributaries SE Suretka, 9.554°N, 82.892°W, el. 48 m; NMNH; ♂; ♀]. —Dumas et al., 2010:8 [distribution]. —Paprocki and França, 2014:45 [checklist]. —Armitage et al., 2015b:6 [checklist]. —Armitage and Cornejo, 2015:195 [checklist].

**Distribution.** Brazil, Colombia, Costa Rica, Ecuador, Mexico, Nicaragua, Panama, Peru.

***tamaulipasa*** Harris, Flint and Holzenthal, 2002b:79 [Type locality: Mexico, Tamaulipas, Rio Frio at La Poza Azul, near Gomez Farias; NMNH; ♂; ♀].

**Distribution.** Mexico.

***yanamona*** Harris, Flint and Holzenthal, 2002b:79 [Type locality: Peru, Loreto, small stream near Explorama Lodge; NMNH; ♂].

**Distribution.** Peru.

#### Genus *Hydroptila* Dalman [72]

*Hydroptila* Dalman, 1819:125 [Type species: *Hydroptila tineoides* Dalman, 1819, by monotypy]. —Bueno-Soria, 1984a:83 [revision of Mexican and Central American species]. —Harris and Holzenthal, 1999:16 [key to Central American species].

The genus *Hydroptila* (Hydroptilinae) is found around the world and contains more species than any other hydroptilid genus. Marshall (1979) divided it into 13 species groups based on distribution and structure of the male genitalia.

Larvae are well known although very few have been associated with species (Lepneva 1970, Roldán Pérez 1988, Wiggins 1996). The case consists of two silken valves covered with sand. Larvae live in both lotic and lentic waters and, according to Nielsen (1948), feed on filamentous algae.

***acuminata*** Bueno-Soria, 1984a:88 [Type locality: Mexico, Tamaulipas, 40 km S Ciudad Victoria, Río Purificación; IBUNAM; ♂]. —Moulton and Stewart, 1997b:350 [distribution]. —Bowles et al., 2007:21 [distribution; biology].

**Distribution.** Mexico, U.S.A.

***ajax*** Ross, 1938b:127 [Type locality: United States, Illinois, Oakwood, along Salt Fork River; INHS; ♂]. —Ross, 1944:153 [♂; ♀; larva]. —Bueno-Soria, 1984a:109 [♂; distribution]. —Blinn and Ruiter, 2006:332 [biology]. —Bowles et al., 2007:21 [distribution; biology]. —Chamorro-Lacayo et al., 2007:42 [checklist]. —Blinn and Ruiter, 2009a:304 [biology]. —Blinn and Ruiter, 2009b:186 [phenology, distribution].

**Distribution.** Mexico, Nicaragua, U.S.A.

***aldricki*** Bueno-Soria, 1984a:108 [Type locality: Mexico, Guerrero, Cocula; BMNH; ♂].

**Distribution.** Mexico.

***ancistrion*** Flint, 1968a:48 [Type locality: Jamaica, Portland, Rio Grande, at Fellowship; NMNH; ♂; ♀]. —Flint, 1968b:82 [checklist]. —Botosaneanu and Hyslop, 1998:15 [distribution]. —Botosaneanu, 2002:83 [checklist].

**Distribution.** Jamaica.

***angusta*** Ross, 1938b:130 [Type locality: United States, Illinois, Muncie, along Stony Creek; INHS; ♂]. —Ross, 1944:152 [♂; ♀; larva]. —Bueno-Soria and Flint, 1978:201 [distribution]. —Bueno-Soria, 1984a:122 [♂; distribution]. —Houghton and Stewart, 1998c:105 [biology]. —Baumgardner and Bowles, 2005:11 [distribution]. —Bowles et al., 2007:21 [distribution; biology]. —Bueno-Soria and Barba-Álvarez, 2011:356 [checklist].

**Distribution.** Mexico, U.S.A.

***antilliarum*** Flint, 1968b:57 [Type locality: Dominica, Pont Casse, 1.6 mi W; NMNH; ♂; ♀]. —Malicky, 1983:264 [distribution]. —Botosaneanu, 1989a:100 [♂; scent organ; distribution]. —Flint and Sykora, 1993:50 [checklist]. —Botosaneanu, 1994a:40 [distribution]. —Botosaneanu, 2002:83 [checklist]. —Botosaneanu and Thomas, 2005:40 [distribution].

**Distribution.** Dominica, Guadeloupe, Martinique, St. Lucia.

***arctia*** Ross, 1938b:129 [Type locality: United States, Idaho, Bear River Narrows; INHS; ♂]. —Bueno-Soria, 1984a:97 [♂; distribution]. —Flint et al., 2003:31 [distribution; does not occur in Hawaii, misidentification of *Hydroptila potosina*]. —Baumgardner and Bowles, 2005:11 [distribution]. —Blinn and Ruiter, 2006:332 [biology]. —Bueno-Soria et al., 2007:33 [distribution]. —Blinn and Ruiter, 2009a:303 [biology]. —Blinn and Ruiter, 2009b:186 [phenology, distribution].

**Distribution.** Mexico, U.S.A.

***argentinica*** Flint, 1983a:43 [Type locality: Argentina, Pcia. Tucumán, S Concepción; NMNH; ♂; ♀]. —Angrisano, 1995b:509 [distribution]. —Angrisano, 1999:32 [checklist]. —Blahnik et al., 2004:5 [distribution]. —Paprocki et al., 2004:11 [checklist]. —Angrisano and Sganga, 2007:32 [♂; ♀; distribution]. —Dumas et al., 2009:366 [distribution]. —Calor, 2011:321 [checklist]. —Rueda Martín, 2011:6 [♂; distribution]. —Dumas and Nessimian, 2012:15 [checklist].—Paprocki and França, 2014:45 [checklist]. —Isa Miranda and Rueda Martín, 2014:199 [distribution].

**Distribution.** Argentina, Bolivia, Brazil, Uruguay.

***bidens*** Flint, 1983a:45 [Type locality: Argentina, Pcia. Jujuy, Aguas Calientes; NMNH; ♂; ♀]. —Angrisano, 1999:32 [checklist]. —Rueda Martín, 2011:7 [♂; distribution]. —Isa Miranda and Rueda Martín, 2014:199 [distribution].

**Distribution.** Argentina, Bolivia.

***brailovskyi*** Bueno-Soria, 1984a:122 [Type locality: Mexico, Veracruz, Chicontepec; IBUNAM; ♂]. —Harris and Holzenthal, 1999:34 [♂; distribution].

**Distribution.** Costa Rica, Mexico.

***carara*** Harris and Holzenthal, 1999:19 [Type locality: Costa Rica, San José, Reserva Biológica Carara, Quebrada Bonita, 9.775°N, 84.605°W; NMNH; ♂].

**Distribution.** Costa Rica.

***catamarcensis*** Flint, 1983a:45 [Type locality: Argentina, Pcia. Catamarca, Arroyo El Pintado, near La Viña; NMNH; ♂; ♀]. —Angrisano, 1999:32 [checklist].

**Distribution.** Argentina.

***constricta*** Bueno-Soria, 1984a:99 [Type locality: Mexico, Chiapas, La Prusia; BMNH; ♂]. —Flint, 1991:47 [♂ ♀ distribution]. —Flint and Reyes, 1991:484 [distribution]. —Harris and Holzenthal, 1999:34 [♂; distribution]. —Muñoz-Quesada, 2000:277 [checklist]. —Bueno-Soria and Barba-Álvarez, 2011:356 [checklist].

**Distribution.** Belize, Colombia, Costa Rica, Honduras, Mexico, Peru.

***coscaroni*** Flint, 1983a:46 [Type locality: Argentina, Pcia. Salta, 5 km S Oran; NMNH; ♂]. —Angrisano, 1999:32 [checklist].

**Distribution.** Argentina.

***cressae*** Thomson and Holzenthal, 2012:23 [Type locality: Venezuela, Bolívar, Gran Sabana, E. Pauji, “Río Curvita”, 04°31.237'N, 61°31.591'W, el. 869 m; UMSP; ♂].

**Distribution.** Venezuela.

***cubana*** Kumanski, 1987:30 [Type locality: Cuba, Province Las Villas, the massive of Guamuaya, Rio Nabujina near El Piojillo village; NMSB; ♂; ♀]. —Flint, 1996c:16 [checklist]. —Botosaneanu, 2002:83 [checklist]. —Naranjo López and González Lazo, 2005:149 [checklist].

—*pseudomeralda* Botosaneanu 1979:51 [*nomen nudum*, attributed to Sykora]. —Kumanski, 1987:30 [to synonymy].

**Distribution.** Cuba.

***curvata*** Bueno-Soria, 1984a:123 [Type locality: Honduras, El Zamorano; NMNH; ♂]. —Harris and Holzenthal, 1999:38 [♂; distribution].

**Distribution.** Costa Rica, Honduras.

***denza*** Ross, 1948a:204 [Type locality: Mexico, Tamaulipas, Hacienda Santa Engracia; INHS; ♂]. —Bueno-Soria and Flint, 1978:201 [distribution]. —Bueno-Soria, 1984a:114 [♂; distribution]. —Holzenthal, 1988c:61 [distribution]. —Maes, 1999:1193 [checklist]. —Chamorro-Lacayo et al., 2007:42 [checklist].

**Distribution.** Costa Rica, Mexico, Nicaragua.

***ditalea*** Flint, 1968a:46 [Type locality: Jamaica, St. Andrew, Fresh River, Ferry; NMNH; ♂; ♀]. —Flint, 1968b:82 [checklist]. —Bueno-Soria, 1984a:119 [♂; distribution]. —Flint and Reyes, 1991:484 [distribution]. —Botosaneanu, 1995:27 [distribution]. —Botosaneanu and Hyslop, 1998:16 [distribution].—Flint and Pérez-Gelabert, 1999:39 [checklist]. —Botosaneanu, 2002:83 [checklist]. —Flint and Sykora, 2004:31 [distribution]. —Pérez-Gelabert, 2008:300 [checklist].

**Distribution.** Dominican Republic, Ecuador, Jamaica, Mexico, Peru.

***dominicana*** Botosaneanu, 1995:27 [Type locality: Dominican Republic, La Descubierta, north shore Lago Enriquillo, south from Sierra de Neiba; ZMUA; ♂; ♀]. —Botosaneanu, 2002:83 [checklist]. —Flint and Pérez-Gelabert, 1999:39 [checklist]. —Flint and Sykora, 2004:31 [distribution]. —Pérez-Gelabert, 2008:300 [checklist].

**Distribution.** Cuba, Dominican Republic.

***felfela*** Oláh and Johanson, 2011:119 [Type locality: Mexico, State of Veracruz, Los Manantioles, Tlilapan, 18°47.944'N, 097°06.270'W, el. 1171 m; NHRS; ♂].

**Distribution.** Mexico.

***flinti*** Bueno-Soria, 1984a:107 [Type locality: Costa Rica, Turrialba; NMNH; ♂]. —Holzenthal, 1988c:61 [distribution]. —Harris and Holzenthal, 1999:38 [♂; distribution]. —Armitage et al., 2015a:5 [distribution]. —Armitage et al., 2015b:6 [checklist]. —Armitage and Cornejo, 2015:195 [checklist].

**Distribution.** Costa Rica, Panama.

***florestani*** Souza, Santos and Takiya, 2014b:640 [Type locality: Brazil, Piauí, Parque Nacional de Sete Cidades, Riacho Piedade, 04°06'34"S, 41°43'39"W, el. 169 m; CZMA; ♂].

**Distribution.** Brazil.

***furtiva*** Bueno-Soria, 1984a:104 [Type locality: Mexico, Oaxaca, Puerta de Uxpanapa; IBUNAM; ♂].

**Distribution.** Mexico.

***grenadensis*** Flint, 1968b:58 [Type locality: Grenada, 2 mi W Grand Etang; NMNH; ♂; ♀]. —Flint and Reyes, 1991:484 [distribution]. —Flint and Sykora, 1993:57 [distribution]. —Flint, 1996a:97 [distribution]. —Harris and Holzenthal, 1999:27 [♂]. —Maes, 1999:1193 [checklist]. —Muñoz-Quesada, 2000:277 [checklist]. —Botosaneanu, 2002:83 [checklist]. —Chamorro-Lacayo et al., 2007:43 [checklist]. —Oláh and Johanson, 2011:120 [distribution]. —Armitage et al., 2015b:6 [checklist]. —Armitage and Cornejo, 2015:195 [checklist].

—*acutissima* Botosaneanu, in Botosaneanu and Alkins-Koo, 1993:24 [Type locality: Trinidad, upper course of River Guanapo; ZMUA; ♂; ♀]. —Botosaneanu and Sakal, 1992:202 [*nomen nudum* (name included in checklist); distribution; ecology]. —Flint, 1996a:97 [to synonymy].

**Distribution.** Colombia, Ecuador, Grenada, Nicaragua, Panama, Peru, Tobago, Trinidad, Venezuela.

***hamata*** Morton, 1905:67 [Type locality: United States, New York, Ithaca; type depository unknown, CU?; ♂]. —Ross, 1944:149 [♂; ♀; larva; distribution]. —Bueno-Soria and Flint, 1978:201 [distribution]. —Bueno-Soria, 1984a:89 [distribution]. —Blinn and Ruiter, 2006:332 [biology]. —Bowles et al., 2007:21 [distribution; biology]. —Bueno-Soria et al., 2007:33 [distribution]. —Blinn and Ruiter, 2009a:303 [biology]. —Blinn and Ruiter, 2009b:186 [phenology, distribution].

**Distribution.** Canada, Mexico, U.S.A.

***helicina*** Flint, 1991:49 [Type locality: Colombia, Dpto. Antioquia, Quebrada Espadera, 7 km E Medellín, road to Sta. Elena; NMNH; ♂; ♀]. —Muñoz-Quesada, 2000:277 [checklist].

**Distribution.** Colombia.

***hoffmannae*** Bueno-Soria and Santiago-Fragoso, 1996b:345 [Type locality: Mexico, Veracruz, Los Tuxtlas, Arroyo Tebanca, 15 km SE La Estación de Biología Los Tuxtlas; IBUNAM; ♂].

**Distribution.** Mexico.

***hossa*** Oláh and Johanson, 2011:121 [Type locality: Peru, San Martin Prov., stream crossing Juan Guerra-Chazuta rd., 10 km (rd.) W Chazuta, 6°37.157'S, 76°10.905'W; NHRS; ♂].

**Distribution.** Peru.

***icona*** Mosely, 1937:161 [Type locality: Mexico, Chiapas, Dolores, BMNH; ♂]. —Ross, 1944:154 [♂; distribution]. —Bueno-Soria and Flint, 1978:201 [distribution]. —Bueno-Soria, 1984a:110 [♂; distribution]. —Houghton and Stewart, 1998c: 105 [biology]. —Harris and Holzenthal, 1999:38 [♂; distribution]. —Maes, 1999:1193 [checklist]. —Flint et al., 2003:33 [♀; distribution; introduced to Hawaii]. —Baumgardner and Bowles, 2005:11 [distribution]. —Blinn and Ruiter, 2006:332 [biology]. —Bowles et al., 2007:21 [distribution; biology]. —Bueno-Soria et al., 2007:33 [distribution]. —Chamorro-Lacayo et al., 2007:43 [checklist]. —Blinn and Ruiter, 2009a:305 [biology]. —Blinn and Ruiter, 2009b:186 [phenology, distribution]. —Bueno-Soria and Barba-Álvarez, 2011:357 [checklist].

**Distribution.** Costa Rica, Honduras, Mexico, Nicaragua, U.S.A.

***inornata*** Flint, 1991:47 [Type locality: Colombia, Dpto. Antioquia, Quebrada Espadera, 7 km E Medellín, road to Sta. Elena; NMNH; ♂; ♀]. —Muñoz-Quesada, 2000:277 [checklist].

**Distribution.** Colombia.

***karikatla*** Oláh and Johanson, 2011:123 [Type locality: Peru, San Martin Prov., creek crossing rd. Tarapoto-Yurimaguas, ca. 30 km (rd.) NE Tarapoto, 6°24.904'S, 76°18.756'W; NHRS; ♂].

**Distribution.** Peru.

***karima*** Oláh and Johanson, 2011:123 [Type locality: Peru, Amazonas Prov., river crossing Olmos-Tarapoto rd., 371 km (rd.) E Olmos Desv. Jaén, 5°41.178'S, 77°46.421'W; NHRS; ♂].

**Distribution.** Peru.

***lacandona*** Bueno-Soria, 1984a:118 [Type locality: Mexico, Chiapas, 10 km from Bonampak; IBUNAM; ♂]. —Bueno-Soria and Barba-Álvarez, 2011:357 [checklist].

**Distribution.** Mexico.

***longissima*** Bueno-Soria, 1984a:97 [Type locality: Mexico, Guerrero, Acahuizotla; IBUNAM; ♂; as *longissimus*].

**Distribution.** Honduras, Mexico.

***marighellai*** Souza, Santos and Takiya, 2014b:640 [Type locality: Brazil, Ceará, Parque Nacional de Ubajara, Rio das Minas próximo ao teleférico, 03°48'58"S, 40°53'53"W, el. 420 m; CZMA; ♂].

**Distribution.** Brazil.

***maritza*** Harris and Holzenthal, 1999:21 [Type locality: Costa Rica, Guanacaste, Parque Nacional Guanacaste, Maritza, Río Tempisquito, 10.958°N, 85.497°W; NMNH; ♂].

**Distribution.** Costa Rica.

***martorelli*** Flint, 1964a:52 [Type locality: Puerto Rico, Maricao, at fish hatchery; NMNH; ♂; ♀; larva; case]. —Flint, 1968b:82 [checklist]. —Malicky, 1983:264 [distribution]. —Flint and Sykora, 1993:50 [checklist]. —Botosaneanu, 1994a:41 [distribution]. —Botosaneanu, 2000:256 [distribution]. —Botosaneanu, 2002:83 [checklist]. —Botosaneanu and Thomas, 2005:55 [checklist].

**Distribution.** Guadeloupe, Puerto Rico.

***maza*** Harris and Holzenthal, 1999:29 [Type locality: Costa Rica, San José, Reserva Biológica Carara, Río de Sur, 1.5 km (rd) S Carara, 9.769°N, 84.531°W; NMNH; ♂].

**Distribution.** Costa Rica.

***medinai*** Flint, 1964a:54 [Type locality: Puerto Rico, Maricao, at fish hatchery; NMNH; ♂; ♀]. —Flint, 1968b:82 [checklist]. —Botosaneanu, 1977:271 [♂; variation, distribution]. —Botosaneanu, 1979:51 [distribution]. —Kumanski, 1987:30 [distribution]. —Botosaneanu, 1991a:130 [distribution]. —Flint, 1996c:16 [checklist]. —Flint and Pérez-Gelabert, 1999:39 [checklist]. —Botosaneanu, 2002:83 [checklist].—Flint and Sykora, 2004:31 [distribution]. —Naranjo López and González Lazo, 2005:149 [checklist]. —Pérez-Gelabert, 2008:300 [checklist].

**Distribution.** Cuba, Dominican Republic, Haiti, Puerto Rico.

***meralda*** Mosely, 1937:162 [Type locality: Mexico, Chiapas, Esmeralda; BMNH; ♂]. —Bueno-Soria and Flint, 1978:201 [distribution]. —Bueno-Soria, 1984a:128 [♂]. —Harris and Holzenthal, 1999:42 [♂; distribution]. —Chamorro-Lacayo et al., 2007:43 [checklist]. —Bueno-Soria and Barba-Álvarez, 2011:357 [checklist].

**Distribution.** Costa Rica, Mexico, Nicaragua.

***mexicana*** Mosely, 1937:160 [Type locality: Mexico, Chiapas, Dolores; BMNH; ♂]. —Bueno-Soria and Flint, 1978:201 [distribution]. —Bueno-Soria, 1984a:109 [♂; distribution]. —Harris and Holzenthal, 1999:42 [♂; distribution]. —Maes, 1999:1193 [checklist]. —Bueno-Soria et al., 2005:75 [distribution]. —Chamorro-Lacayo et al., 2007:43 [checklist]. —Bueno-Soria and Barba-Álvarez, 2011:357 [checklist].

**Distribution.** Costa Rica, Honduras, Mexico, Nicaragua.

***misolha*** Bueno-Soria, 1984a:127 [Type locality: Mexico, Chiapas, Cascada de Misolha; IBUNAM; ♂]. —Maes and Flint, 1988:4 [distribution]. —Harris and Holzenthal, 1999:45 [♂; distribution]. —Maes, 1999:1193 [checklist]. —Bueno-Soria et al., 2005:75 [distribution]. —Chamorro-Lacayo et al., 2007:43 [checklist]. —Bueno-Soria and Barba-Álvarez, 2011:357 [checklist].

**Distribution.** Belize, Costa Rica, Honduras, Mexico, Nicaragua.

***modica*** Mosely, 1937:163 [Type locality: Mexico, Chiapas, Dolores, BMNH; ♂]. —Bueno-Soria and Flint, 1978:202 [distribution]. —Bueno-Soria, 1984a:90 [♂; distribution]. —Moulton and Stewart, 1997b:350 [distribution]. —Bowles et al., 2007:21 [distribution; biology]. —Bueno-Soria et al., 2007:33 [distribution]. —Bueno-Soria and Barba-Álvarez, 2011:357 [checklist].

**Distribution.** Mexico, U.S.A.

***narifer*** Flint, 1991:47 [Type locality: Colombia, Dpto. Antioquia, Quebrada La Jiménez, Sopetrán; NMNH; ♂; ♀]. —Muñoz-Quesada, 2000:278 [checklist].

**Distribution.** Colombia.

***neoleonensis*** Bueno-Soria, 1984a:113 [Type locality: Mexico, Nuevo Leon, Linares; NMNH; ♂].

**Distribution.** Mexico.

***nusagandia*** Harris and Holzenthal, 1999:29 [Type locality: Panama, San Blas, Quebrada Pingad, 9 km N Nusagandi; NMNH; ♂]. —Armitage et al., 2015b:6 [checklist]. —Armitage and Cornejo, 2015:195 [checklist].

**Distribution.** Panama.

***osa*** Harris and Holzenthal, 1999:21 [Type locality: Costa Rica, Puntarenas, Quebrada Pita, ca 3 km (air) W Golfito, 8.642°N, 83.193°W; NMNH; ♂].

**Distribution.** Costa Rica.

***paradenza*** Harris and Holzenthal, 1999:25 [Type locality: Costa Rica, Limón, E.A.R.T.H., Río Destierra, Poza Azul, 10.208°N, 83.574°W; NMNH; ♂]. —Chamorro-Lacayo et al., 2007:43 [checklist].

**Distribution.** Costa Rica, Nicaragua, Mexico.

***parhuzam*** Oláh and Johanson, 2011:125 [Type locality: Peru, Pasco Reg., Yanachaga-Chemillen NP., side river to Rio Huancabamba, N end of park, along Oxabamba-Pozuzo rd., 10°11.133'S, 75°34.106'W; NHRS; ♂].

**Distribution.** Peru.

***paschia*** Mosely, 1937:164 [Type locality: Mexico, Chiapas, Dolores; BMNH; ♂]. —Bueno-Soria and Flint, 1978:202 [distribution]. —Bueno-Soria, 1984a:102 [♂; distribution]. —Holzenthal, 1988c:61 [distribution]. —Harris and Holzenthal, 1999:45 [♂; distribution]. —Maes, 1999:1193 [checklist]. —Bueno-Soria et al., 2007:33 [distribution]. —Chamorro-Lacayo et al., 2007:43 [checklist]. —Bueno-Soria and Barba-Álvarez, 2011:357 [checklist].

**Distribution.** Costa Rica, Mexico, Nicaragua.

***potosina*** Bueno-Soria, 1984a:95 [Type locality: Mexico, San Luis Potosi, Palitla; NMNH; ♂]. —Moulton and Stewart, 1997b:350 [distribution]. —Flint et al., 2003:31 [♂; ♀; distribution; introduced to Hawaii]. —Bowles et al., 2007:21 [distribution; biology].

**Distribution.** Mexico, U.S.A.

***producta*** Mosely, 1939a:236 [Type locality: Brazil, Edo. Santa Catarina, Nova Teutonia; BMNH; ♂]. —Angrisano, 1995b:509 [distribution]. —Angrisano, 1999:32 [checklist]. —Paprocki et al., 2004:11 [checklist]. —Dumas et al., 2009:366 [distribution]. —Dumas and Nessimian, 2012:15 [checklist]. —Paprocki and França, 2014:45 [checklist].

**Distribution.** Brazil, Uruguay.

***pulestoni*** Flint, 1980b:138 [Type locality: Argentina, Pcia. Buenos Aires, Estancia Delta, near Balneario Monte Hermosa; NMNH; ♂; ♀]. —Flint, 1982c:35 [distribution]. —Angrisano, 1995b:509 [distribution]. —Mangeaud, 1996:154 [distribution]. —Angrisano, 1999:32 [checklist]. —Muzón et al., 2005: 57 [distribution]. —Angrisano and Sganga, 2007:32 [♂; distribution]. —Oláh and Johanson, 2011:126 [distribution]. —Isa Miranda and Rueda Martín, 2014:199 [distribution].

**Distribution.** Argentina, Chile, Uruguay.

***rastrilla*** Harris and Holzenthal, 1999:32 [Type locality: Limón, Reserva Biológica Barbilla, Río Dantas, 15 km (rd) S Pacuarito, 9.994°N, 83.443°W; NMNH; ♂]. —Armitage et al., 2015b:6 [checklist]. —Armitage and Cornejo, 2015:195 [checklist].

**Distribution.** Costa Rica, Panama.

***rono*** Ross, 1941:66 [Type locality: United States, Utah, Huntsville; INHS; ♂; ♀]. —Bueno-Soria and Flint, 1978:202 [distribution]. —Bueno-Soria, 1984a:93 [♂; distribution]. —Blinn and Ruiter, 2006:332 [biology]. —Blinn and Ruiter, 2009b:186 [phenology, distribution].

**Distribution.** Mexico, U.S.A.

***sarkos*** Oláh and Johanson, 2011:126 [Type locality: Peru, San Martin Prov., creek crossing rd. Tarapoto-Yurimaguas, ca. 30 km (rd.) NE Tarapoto, 6°24.904'S, 76°18.756'W; NHRS; ♂].

**Distribution.** Peru.

***sauca*** Flint, 1980b:141 [Type locality: Argentina, Pcia. Buenos Aires, Rio Sauce Grande, Sierra de la Ventana; NMNH; ♂; ♀]. —Flint, 1982c:35 [distribution]. —Angrisano, 1995b:509 [distribution]. —Angrisano, 1999:32 [checklist]. —Angrisano and Sganga, 2007:32 [♂; distribution].

**Distribution.** Argentina, Uruguay.

***selvatica*** Botosaneanu, 1977:269 [Type locality: Cuba, Oriente, Baire, petit ruisseau, affluent de Rio Brazo Seco, a Matias; NMNH; ♂]. —Botosaneanu, 1979:51 [distribution]. —Flint, 1996c:16 [checklist]. —Botosaneanu, 2002:83 [checklist]. —Naranjo López and González Lazo, 2005:149 [checklist].

**Distribution.** Cuba.

***sicilicula*** Flint and Reyes, 1991:484 [Type locality: Peru, Dept. Lambayeque, Río Saña, Saña near ruins of Corbacho; NMNH; ♂].

**Distribution.** Peru.

***singri*** Harris and Holzenthal, 1999:34 [Type locality: Costa Rica, Puntarenas, Río Singrí, ca 2 km (air) S Finca Helechales, 9.057°N, 83.082°W; NMNH; ♂].

**Distribution.** Costa Rica.

***spada*** Flint, 1991:47 [Type locality: Colombia, Dpto. Antioquia, Quebrada Espadera, 7 km E Medellín, road to Sta. Elena; NMNH; ♂]. —Muñoz-Quesada, 2000:278 [checklist].

**Distribution.** Colombia.

***spangleri*** Bueno-Soria, 1984a:113 [Type locality: Guatemala, Matias de Galvez; NMNH; ♂].

**Distribution.** Guatemala.

***spirula*** Bueno-Soria, 1984a:121 [Type locality: Mexico, Michoacán, Carácuaro; BMNH; ♂].

**Distribution.** Mexico.

***surinamensis*** Flint, 1974c:64 [Type locality: Suriname, Blanche Marie, falls behind camp; RNH; ♂].

**Distribution.** Suriname.

***tobaga*** Botosaneanu, in Botosaneanu and Alkins-Koo, 1993:27 [Type locality: Tobago, streamlet, cut by road Roxborough- Parlatuvier, near summit; ZMUA; ♂; ♀]. —Botosaneanu and Sakal, 1992:202 [*nomen nudum* (name included in checklist); distribution; ecology]. —Flint, 1996a:98 [distribution]. —Botosaneanu, 2002:83 [checklist].

**Distribution.** Tobago.

***tulipa*** Oláh and Johanson, 2011:127 [Type locality: Peru, Dep. Lima, Pacaran, Province Canete, River Chillon Obrajillo, 12°52'05"S, 76°02'60"W, el. 877 m; NHRS; ♂].

**Distribution.** Peru.

***unicuspis*** Flint, 1991:49 [Type locality: Colombia, Dpto. Antioquia, Quebrada La Cebolla, El Retiro; NMNH; ♂; ♀]. —Muñoz-Quesada, 2000:278 [checklist].

**Distribution.** Colombia.

***vazquezae*** Bueno-Soria, 1984a:105 [Type locality: Mexico, Chiapas, Santa Elena, 50 km S Montebello; IBUNAM; ♂]. —Bueno-Soria and Barba-Álvarez, 2011:357 [checklist].

**Distribution.** Mexico.

***venezuelensis*** Flint, 1981a:29 [Type locality: Venezuela, Aragua, Maracay, Río Limón, Estacion Piscicultura; NMNH; ♂; ♀]. —Botosaneanu, 2002:83 [checklist]. —Oláh and Johanson, 2011:128 [distribution].

**Distribution.** Ecuador, Venezuela.

***veracruzensis*** Flint, 1967b:13 [Type locality: Mexico, Vera Cruz, Cuitlahuac; NMNH; ♂]. —Bueno-Soria, 1984a:116 [♂; distribution]. —Bueno-Soria and Flint, 1978:202 [distribution]. —Botosaneanu and Alkins-Koo, 1993:24 [♂; ♀; distribution]. —Flint, 1996a:97 [distribution]. —Harris and Holzenthal, 1999:45 [♂; distribution]. —Maes, 1999:1193 [checklist]. —Botosaneanu and Viloria, 2002:106 [distribution]. —Bueno-Soria et al., 2005:75 [distribution]. —Chamorro-Lacayo et al., 2007:43 [checklist]. —Bueno-Soria and Barba-Álvarez, 2011:357 [checklist]. —Armitage et al., 2015a:5 [distribution]. —Armitage et al., 2015b:6 [checklist]. —Armitage and Cornejo, 2015:195 [checklist].

**Distribution.** Costa Rica, Mexico, Nicaragua, Panama, Trinidad, Venezuela.

***zerbinae*** Souza, Santos and Takiya, 2014b:641 [Type locality: Brazil, Pernambuco, Vicência Cachoeira do Engenho Embú, 07°37'22"S, 35°22'51"W, el. 186 m; DZRJ; ♂].

**Distribution.** Brazil.

#### Genus *Ithytrichia* Eaton [2]

*Ithytrichia* Eaton, 1873:139 [Type species: *Ithytrichia lamellaris* Eaton, 1873, original designation]. —Ross, 1944:123 [revision North American species]. —Moulton et al., 1999:233 [distribution; review of North American species]. —Rueda Martín, 2006b:252 [distribution].

*Ithytrichia* is a genus of seven species placed in the Orthotrichiinae and occurring in Europe, North America, and South America. Moulton et al. (1999) provided a review and illustrated key to the three North American species, including the single species known from south of the U.S. border.

Larvae of several European and North American species are known (Nielsen 1948, Wiggins 1996). They live on the surface of rocks or on moss in lotic sites and feed on diatoms and other periphyton that they scrape off the surface (Nielsen 1948). Rueda Martín (2006b) described and illustrated *Ithytrichia ferni*, including adults, larvae, and pupae, and recorded the genus from South America for the first time.

***ferni*** Rueda Martín, 2006b:252 [Type locality: Argentina, Tucumán Prov., Tafí Viejo, Río Tafí, 26°43'25"S, 64°17'26"W, el. 827 m; FML (Rueda Martín, personal com.); ♂; larva; pupa; case; biology]. —Isa Miranda and Rueda Martín, 2014:199 [distribution].

**Distribution.** Argentina.

***mexicana*** Harris and Contreras-Ramos, 1989:176 [Type locality: Mexico, Tamaulipas, Rio Frio, 6 km S Gomez Farias; NMNH; ♂]. —Moulton et al., 1999:236 [♂; ♀; distribution]. —Blinn and Ruiter, 2006:332 [biology]. —Blinn and Ruiter, 2009a:303 [biology]. —Blinn and Ruiter, 2009b:186 [phenology, distribution].

**Distribution.** Mexico, U.S.A.

#### Genus *Kumanskiella* Harris and Flint [2]

*Kumanskiella* Harris and Flint, 1992:581 [Type species: *Kumanskiella karenae* Harris and Flint 1992, original designation]. —Oláh and Johanson, 2011: 167 [in Neotrichiini].

*Kumanskiella*, endemic to the Greater Antilles and belonging to the Neotrichiinae, was established for a previously described species from Cuba and an undescribed species from Puerto Rico. The genus is still only known from these species and these islands.

Larvae are typical members of the Neotrichiinae and are distinguished on the basis of case structure and thoracic setation (Harris and Flint 1992). They are found on and under rocks and boulders in small, tumbling, mountain streams. Adults were taken primarily in emergence and Malaise traps, although a few came to light.

***aliena*** (Kumanski), 1987:24 [Type locality: Cuba, Province Las Villas, Sierra de Trinidad, road Trinidad-Topes de Collantes; NMSB; ♂; in *Mayatrichia*]. —Harris and Flint, 1992:587 [♂; to *Kumanskiella*]. —Flint, 1996c:16 [checklist]. —Botosaneanu, 2002:83 [checklist]. —Naranjo López and González Lazo, 2005:149 [checklist].

**Distribution.** Cuba.

***karenae*** Harris and Flint, 1992:582 [Type locality: Puerto Rico, El Verde Field Station, Quebrada Prieta; NMNH; ♂; ♀; larva; case]. —Botosaneanu, 2002:83 [checklist].

**Distribution.** Puerto Rico.

#### Genus *Leucotrichia* Mosely [44 + †1]

*Leucotrichia* Mosely, 1934b:157 [Type species: *Leucotrichia melleopicta* Mosely, 1934b, original designation]. —Flint, 1970:3 [revision]. —Marshall, 1979:178 [revision]. —Oláh and Johanson 2011:152 [discussion of *Leucotrichia* genus cluster]. —Thomson and Holzenthal, 2015:7 [revision; key]. —Santos et al., 2016a:475 [assessment of monophyly].

*Leucotrichia* is a moderately large genus, the type genus of the subfamily Leucotrichiinae. Species are found over most of the United States, Central and northern South America, the Greater Antilles, and the southernmost Lesser Antilles. A single species is known from Dominican amber, *Leucotrichia adela*. The genus was recovered as monophyletic by Santos et al., (2016) after both including and removing a few enigmatic species from and to other genera. Thomson and Holzenthal (2015) revised the species taxonomy of the more broadly defined genus.

Larvae and cases have been described for a number of species (Flint 1970, Wiggins 1996). They construct flattened, oval cases with a circular opening at each end, and are tightly attached to a rock or boulder in fast flowing streams. Adults do come to light, but are most often taken from marginal foliage by net during the day. Many species have bright green patches of hairs on the forewings and body, contrasting with darker colored areas. Adult males commonly display by crawling rapidly and erratically on large riparian leaves, especially those dappled in sunlight.

† ***adela*** Wells and Wichard, 1989:42 [Type locality: Dominican Republic; NMNH; ♂; in amber]. —Flint and Pérez-Gelabert, 1999:39 [checklist]. —Botosaneanu, 2002:84 [checklist]. —Wichard, 2007a:48 [checklist]. —Pérez-Gelabert, 2008:300 [checklist]. —Thomson and Holzenthal, 2015:9 [♂].

**Distribution.** Dominican Republic.

***alibrachia*** (Thomson), 2012:4 [Type locality: Brazil: Rio de Janeiro, Resende, Ribeirás do Palmital, 22°25'26.2"S, 44°44'21.6"W, el. 969 m; DZRJ; ♂; in *Betrichia*]. —Paprocki and França, 2014:41 [checklist]. —Santos et al., 2016a:472 [to *Leucotrichia*]

**Distribution.** Brazil.

***alisensis*** Rueda Martín, 2011:4 [Type locality: Argentina, Tucumán, Parque Nacional Campo de Los Alisos, Río de las Pavas, 27°12'39"S, 65°55'39"W, el. 1655 m; IML; ♂; morphotype; larva; pupa]. —Thomson, 2012:2 [checklist]. —Thomson and Holzenthal, 2015:10 [♂]. —Isa Miranda and Rueda Martín, 2014:199 [distribution].

**Distribution.** Argentina.

***angelinae*** Thomson and Holzenthal, 2015:11 [Type locality: Venezuela, Mérida, Cacuta, 10 km E Tabay; NMNH; ♂].

**Distribution.** Venezuela.

***ayura*** Flint, 1991:41 [Type locality: Colombia, Dpto. Antioquia, 12km NW Medellín, road to San Pedro; NMNH; ♂]. —Muñoz-Quesada, 2000:278 [checklist]. —Thomson, 2012:2 [checklist]. —Thomson and Holzenthal, 2015:11 [♂].

**Distribution.** Colombia.

***bicornuta*** Thomson, 2012:4 [Type locality: Brazil: Rio de Janeiro, Panedo [sic], Rio das Pedras, Três Bacias, 22°24'32.2"S, 44°33'06.5"W, el. 735 m; DZRJ; ♂]. —Paprocki and França, 2014:45 [checklist]. —Thomson and Holzenthal, 2015:12 [♂].

**Distribution.** Brazil.

***botosaneanui*** Flint, 1996a:86 [Type locality: Tobago, big waterfall 4km S Charlotteville, 11°19'N, 60°33'W; NMNH; ♂]. —Botosaneanu and Sakal, 1992:201 [distribution; ecology; as *limpia*]. —Botosaneanu and Alkins-Koo, 1993:10 [larva; as *limpia*]. —Botosaneanu, 2002:84 [checklist]. —Thomson, 2012:2 [checklist]. —Thomson and Holzenthal, 2015:13 [♂].

**Distribution.** Tobago, Trinidad.

***brasiliana*** Sattler and Sykora, 1977:239 [Type locality: Brazil, Amazonas Staat, bereich des Rio Marauía, bei Tapuruquara, oberer Rio Negro; type depository unknown; ♂; larva; pupa; case]. —Angrisano, 1999:32 [checklist]. —Paprocki et al., 2004:11 [checklist]. —Thomson, 2012:2 [checklist]. —Paprocki and França, 2014:45 [checklist]. —Thomson and Holzenthal, 2015:14 [♂ topotype].

**Distribution.** Brazil.

***brochophora*** Flint, 1991:41 [Type locality: Colombia, Dpto. Antioquia, Quebrada Espadera, 7km E Medellín, road to Sta. Elena; NMNH; ♂]. —Muñoz-Quesada, 2000:278 [checklist]. —Thomson, 2012:2 [checklist]. —Thomson and Holzenthal, 2015:15 [♂].

**Distribution.** Colombia.

***chiriquiensis*** Flint, 1970:6 [Type locality: Panama, Chiriqui, Alto Lino above Boquete; NMNH; ♂; larva; case]. —Aguila, 1992:538 [distribution]. —Thomson, 2012:2 [checklist]. —Thomson and Holzenthal, 2015:16 [♂]. —Armitage et al., 2015b:6 [checklist]. —Armitage and Cornejo, 2015:195 [checklist].

**Distribution.** Panama.

***denticulata*** Thomson and Holzenthal, 2015:17 [Type locality: Mexico, Nuevo Leon, Municipio de Santiago, Arroyo San Juan on road to Laguna de Sanchez, 3.5 km W La Cienegra, 25°24'N, 10°17'W, el. 1400 m; UMSP; ♂].

**Distribution.** Mexico.

***dianeae*** Thomson and Holzenthal, 2015:17 [Type locality: Costa Rica, Cartago, Reserva Tapantí, waterfall, ca. 1 km (road) NW tunnel, 9.69°N, 83.76°W, el. 1600 m; UMSP; ♂].

**Distribution.** Costa Rica.

***dinamica*** Bueno-Soria, 2010:23 [Type locality: Mexico, D. F., Delegación Magdalena-Contreras, Parque ‘‘Los Dinamos,” el. 3091 m; CNIN; ♂]. —Thomson, 2012:2 [checklist]. —Thomson and Holzenthal, 2015:18 [♂].

**Distribution.** Mexico.

***extraordinaria*** Bueno-Soria, Santiago-Fragoso, and Barba-Álvarez, 2001:145 [Type locality: Mexico, Tabasco, Municipio de Huimanguillo, Arroyo las Flores, Villa de Guadalupe 2a sección Los Chimalapas, km 5 Ruta Malpasito–Carlos A. Madrazo, 17°22'05"N, 93°36'25"W; CNIN; ♂]. —Bueno-Soria et al., 2005:75 [distribution]. —Thomson, 2012:2 [checklist]. —Thomson and Holzenthal, 2015:19 [♂].

**Distribution.** Mexico.

***fairchildi*** Flint, 1970:10 [Type locality: Panama, Cocle, El Valle; MCZ; ♂]; 1968b:38 [♂; ♀; Grenada, but misidentified as *sarita*]. —Flint, 1981a:25 [♂; distribution]. —Flint, 1991:39 [♂; distribution]. —Aguila, 1992:538 [distribution]. —Botosaneanu and Sakal, 1992:201 [distribution; ecology]. —Flint and Sykora, 1993:54 [Grenada, but misidentified as *sarita*]. —Botosaneanu and Alkins-Koo, 1993:7 [larva; case]. —Flint, 1996a:86 [distribution]. —Muñoz-Quesada, 2000:278 [checklist]. —Botosaneanu, 2002:84 [checklist]. —Botosaneanu and Viloria, 2002:106 [distribution]. —Thomson, 2012:2 [checklist]. —Thomson and Holzenthal, 2015:20 [♂; distribution]. —Armitage et al., 2015b:6 [checklist]. —Armitage and Cornejo, 2015:195 [checklist].

—Leucotrichiini, case 2 Botosaneanu and Alkins-Koo, 1993:14 [♀]. —Flint 1996a:86 [to synonymy].

**Distribution.** Colombia, Costa Rica, Ecuador, El Salvador, Grenada, Panama, Tobago, Trinidad, Venezuela.

***falsa*** (Santos, Takiya and Nessimian), 2013:448 [Type locality: Costa Rica, Puntarenas, La Gamba, Esquinas Lodge, river at waterfall trail, 08°41'05"N, 83°12'17"W, el. 70 m; INBIO; ♂; ♀; in *Costatrichia*]. —Santos et al., 2016a:472 [to *Leucotrichia*].

**Distribution.** Costa Rica.

***forrota*** Oláh and Johanson, 2011:160 [Type locality: Peru, San Martin Prov., Rio Huallaga tributary, small river passing Chazuta, 6°34.665'S, 76°08.209'W; NHRS; ♂]. —Oláh and Flint, 2012:176 [distribution]. —Thomson, 2012:2 [checklist]. —Thomson and Holzenthal, 2015:22 [♂].

**Distribution.** Ecuador, Peru.

***fulminea*** Thomson and Holzenthal, 2015:23 [Type locality: Ecuador, Cañar, Río Chauchas, 2910m, 3 km N Zhud; NMNH; ♂].

**Distribution.** Ecuador.

***gomezi*** Flint, 1970:7 [Type locality: Dominican Republic, La Palma, 12km E. El Rio; NMNH; ♂; larva; case]. —Flint and Pérez-Gelabert, 1999:39 [checklist]. —Botosaneanu, 2002:84 [checklist]. —Flint and Sykora, 2004:32 [distribution]. —Pérez-Gelabert, 2008:300 [checklist]. —Thomson, 2012:2 [checklist]. —Thomson and Holzenthal, 2015:24 [♂].

**Distribution.** Dominican Republic.

***hispida*** Thomson and Holzenthal, 2015:25 [Type locality: Costa Rica, San José, Río Savegre, 9°33.9'N, 83°48'W, el. 2270 m; NMNH; ♂].

**Distribution.** Costa Rica.

***imitator*** Flint, 1970:8 [Type locality: Mexico, Vera Cruz, Plan del Rio Ver, Rt. 140, km 368; NMNH; ♂; larva; case]. —Bueno-Soria and Flint, 1978:200 [distribution]. —Holzenthal, 1988c:61 [distribution]. —Bueno-Soria et al., 2007:33 [distribution]. —Thomson, 2012:2 [checklist]. —Thomson and Holzenthal, 2015:26 [♂].

**Distribution.** Costa Rica, Guatemala, Mexico.

***inflaticornis*** Botosaneanu, in Botosaneanu and Alkins-Koo, 1993:10 [Type locality: Trinidad, 2nd. order stream at “La Laja”, catchment of Rio Guanapo; ZMUA; male/; larva; case]. —Botosaneanu and Sakal, 1992:201 [*nomen nudum* (name included in checklist); distribution; ecology]. —Flint, 1996a:89 [distribution]. —Botosaneanu, 2002:84 [checklist]. —Thomson, 2012:2 [checklist]. —Thomson and Holzenthal, 2015:27 [♂].

**Distribution.** Trinidad.

***inops*** Flint, 1991:43 [Type locality: Colombia, Dpto. Antioquia, 12km E Medellín, road to Sta. Elena; NMNH; ♂]. —Muñoz-Quesada, 2000:278 [checklist]. —Thomson, 2012:2 [checklist]. —Thomson and Holzenthal, 2015:27 [♂; distribution].

**Distribution.** Colombia, Ecuador.

***interrupta*** Flint, 1991:41 [Type locality: Colombia, Dpto. Antioquia, Quebrada Espadera, 7km E Medellín, on road to Sta. Elena; NMNH; ♂]. —Muñoz-Quesada, 2000:278 [checklist]. —Thomson, 2012:2 [checklist]. —Thomson and Holzenthal, 2015:28 [♂].

**Distribution.** Colombia.

***kateae*** Thomson and Holzenthal, 2015:29 [Type locality: Venezuela, Aragua, 1 km E Estacíon Biológica Rancho Grande, 10.352°N, 67.680°W, el. 1100 m; UMSP; ♂].

**Distribution.** Venezuela.

***laposka*** Oláh and Johanson, 2011:162 [Type locality: Peru, San Martin Prov., creek crossing rd. Juan Guerra-Chazuta, 14 km (rd.) E Colombia Bridge, 6°35.594'S, 76°13.172'W; NHRS; ♂]. —Thomson, 2012:2 [checklist]. —Thomson and Holzenthal, 2015:30 [♂].

**Distribution.** Peru.

***lerma*** Angrisano and Burgos, 2002:106 [Type locality: Argentina, Salta, río Lesser, 18 km NW Salta; IML; ♂]. —Thomson, 2012:2 [checklist]. —Isa Miranda and Rueda Martín, 2014:196 [larva; pupa; case; distribution]. —Thomson and Holzenthal, 2015:31 [♂].

**Distribution.** Argentina.

***limpia*** Ross, 1944:273 [Type locality: United States, Texas, Fort Davis, Limpia Creek; INHS; ♂]. —Flint, 1970:6 [♂]. —Bueno-Soria and Flint, 1978:200 [distribution]. —Holzenthal, 1988c:61 [distribution]. —Flint, 1996a:86 [correction of errors in 1970 paper]. —Baumgardner and Bowles, 2005:11 [distribution]. —Bowles et al., 2007:21 [distribution; biology]. —Bueno-Soria et al., 2007:33 [distribution]. —Blinn and Ruiter, 2009b:186 [phenology, distribution]. —Bueno-Soria and Barba-Álvarez, 2011:357 [checklist]. —Thomson, 2012:2 [checklist]. —Thomson and Holzenthal, 2015:31 [♂; distribution].

**Distribution.** Costa Rica, Mexico, U.S.A.

***melleopicta*** Mosely, 1934b:157 [Type locality: Mexico, Tabasco, Teapa; BMNH; ♂]. —Flint, 1970:5 [♂]. —Bueno-Soria and Flint, 1978:200 [distribution]. —Flint, 1981a:25 [♂; distribution]. —Thomson, 2012:2 [checklist]. —Thomson and Holzenthal, 2015:9 [♂].

**Distribution.** Mexico, Venezuela.

***mutica*** Flint, 1991:39 [Type locality: Colombia, Dpto. Antioquia, Quebrada Honda, Marsella, 12km SW Fredonia; NMNH; ♂]. —Muñoz-Quesada, 2000:278 [checklist]. —Thomson, 2012:2 [checklist]. —Thomson and Holzenthal, 2015:32 [♂].

**Distribution.** Colombia.

***padera*** Flint, 1991:41 [Type locality: Colombia, Dpto. Antioquia, Quebrada Espadera, 7km E Medellín, road to Sta. Elena; NMNH; ♂]. —Muñoz-Quesada, 2000:278 [checklist]. —Thomson, 2012:2 [checklist]. —Thomson and Holzenthal, 2015:33 [♂].

**Distribution.** Colombia.

***pectinata*** Thomson and Holzenthal, 2015:34 [Type locality: Ecuador, Tungurahua, 13 km E Baños, el. 1550 m; NMNH; ♂].

**Distribution.** Ecuador.

***pictipes*** (Banks), 1911:359 [Type locality: United States, New York, Johnstown, Hales Creek; MCZ; male; in *Orthotrichia*; ♂]. —Ross, 1938:10 [as *Stactobia pictipes* (Banks)]. —Ross, 1944:120 [to *Leucotrichia*]. —Nielsen, 1948:11 [misidentified as *Ithytrichia confusa* Morton]. —Flint, 1970:10 [♂; distribu­tion]. —McAuliffe, 1982:1557 [biology, life history]. —Blinn and Ruiter, 2006:332 [biology]. —Bueno-Soria et al., 2007:33 [distribution]. —Thomson, 2012:2 [checklist]. —Thomson and Holzenthal, 2015:35 [♂].

**Distribution.** Mexico, U.S.A.

***repanda*** Thomson and Holzenthal, 2015:37 [Type locality: Venezuela, Sucre, Península de Paria, Santa Isabel, Río Sta. Isabel, 10°44.294'N, 62°38.954'W, el. 20 m; UMSP; ♂].

**Distribution.** Venezuela.

***rhomba*** Thomson and Holzenthal, 2015:38 [Type locality: Costa Rica, Puntarenas, Río Jaba at rock quarry, 1.4 km (air) W Las Cruces, 8.79°N, 82.97°W, el. 1150 m; UMSP; ♂].

**Distribution.** Costa Rica.

***riostoumae*** Thomson and Holzenthal, 2015:39 [Type locality: Ecuador, Imbabura, Reserva los Cedros, Río de la Plata, 00.32495°N, 78.78084°W, el. 1587 m; UMSP; ♂].

**Distribution.** Ecuador.

***sarita*** Ross, 1944:274 [Type locality: United States, Texas, Balmorhea, along stone irrigation flume; INHS; ♂]. —Flint, 1968b:38 [♂; ♀; larva; pupa; distribution]. —Flint, 1970:9 [/male; larva; case; distribution]. —Bueno-Soria and Flint, 1978:200 [distribution]. —Holzenthal, 1988c:61 [distribution]. —Flint and Sykora, 1993:54 [distribution]. —Bowles et al., 2007:21 [distribution; biology]. —Bueno-Soria et al., 2007:33 [distribution]. —Chamorro-Lacayo et al., 2007:43 [checklist]. —Bueno-Soria and Barba-Álvarez, 2011:357 [checklist]. —Thomson, 2012:2 [checklist]. —Thomson and Holzenthal, 2015:40 [♂].

**Distribution.** Costa Rica, El Salvador, Grenada, Guatemala, Mexico, Nicaragua, U.S.A.

***sidneyi*** Thomson and Holzenthal, 2015:42 [Type locality: Venezuela, T. F. A., Camp IV, 0°58'N, 65°57'W, Cerro d. l. Neblina, el. 760 m; NMNH; ♂].

**Distribution.** Venezuela.

***tapantia*** Thomson and Holzenthal, 2015:42 [Type locality: Costa Rica, Cartago, Reserva Tapantí, waterfall, ca. 1 km (road) NW tunnel, 9.69°N, 83.76°W, el. 1600 m; UMSP; ♂].

**Distribution.** Costa Rica.

***termitiformis*** Botosaneanu, in Botosaneanu and Alkins-Koo, 1993:13 [Type locality: Trinidad, stream below Maracas waterfall; ZMUA; ♂; larva]. —Botosaneanu and Sakal, 1992:201 [*nomen nudum* (name included in checklist); distribution; ecology]. —Flint, 1996a:89 [distribution]. —Botosaneanu, 2002:84 [checklist]. —Thomson, 2012:2 [checklist]. —Thomson and Holzenthal, 2015:43 [♂].

**Distribution.** Trinidad.

***tritoven*** Flint, 1996a:89 [Type locality: Trinidad, streamlet, Lalaja Road, 10°43'N, 61°17'W; NMNH; ♂]. —Botosaneanu, 2002:84 [checklist]. —Thomson, 2012:2 [checklist]. —Thomson and Holzenthal, 2015:44 [♂; distribution].

**Distribution.** Guyana, Tobago, Trinidad, Venezuela.

***tubifex*** Flint, 1964a:44 [Type locality: Puerto Rico, Maricao, at fish hatchery; NMNH; ♂; /female; larva; pupa; case]. —Flint, 1968a:33 [♂; ♀; larva; pupa; distribution]. —Flint, 1968b:81 [checklist]. —Flint, 1970:7 [/male; larva; case; distribution]. —Botosaneanu, 1991a:116 [distribution]. —Botosaneanu, 1995:22 [distribution]. —Botosaneanu and Bolland, 1997:71 [parasitized by mite, genus *Leptus*]. —Botosaneanu and Hyslop, 1998:7 [distribution]. —Flint and Pérez-Gelabert, 1999:39 [checklist]. —Botosaneanu, 2002:84 [checklist]. —Flint and Sykora, 2004:32 [distribution]. —Pérez-Gelabert, 2008:300 [checklist]. —Thomson, 2012:2 [checklist]. —Thomson and Holzenthal, 2015:45 [♂].

**Distribution.** Dominican Republic, Haiti, Jamaica, Puerto Rico.

***viridis*** Flint, 1967b:10 [Type locality: Guatemala, Izabal, Las Escobas near Matias de Galvez; NMNH; ♂]. —Flint, 1970:5 [♂; distribution]. —Bueno-Soria and Flint, 1978:201 [distribution]. —Aguila, 1992:538 [distribution]. —Bueno-Soria and Barba-Álvarez, 2011:357 [checklist]. —Thomson, 2012:2 [checklist]. —Thomson and Holzenthal, 2015:46 [♂]. —Armitage et al., 2015b:6 [checklist]. —Armitage and Cornejo, 2015:195 [checklist].

**Distribution.** El Salvador, Guatemala, Mexico, Panama.

***yungarum*** Angrisano and Burgos, 2002:105 [Type locality: Argentina, Salta, Finca, Jakúlika, 22°41'01"S, 64°30'40"W, el. 630 m; IML; ♂]. —Thomson, 2012:2 [checklist]. —Thomson and Holzenthal, 2015:47 [♂].

**Distribution.** Argentina.

***zopilote*** (Holzenthal and Harris), 1999:561 [Type locality: Costa Rica, Guanacaste, Parque Nacional Rincón de la Vieja, Guebrada Zopilote, 10.765°N, 85.309°W; NMNH; ♂; ♀; in *Costatrichia*]. —Santos et al., 2016a:472 [to *Leucotrichia*].

**Distribution.** Costa Rica.

#### Genus *Mayatrichia* Mosely [4]

*Mayatrichia* Mosely, 1937:182 [Type species: *Mayatrichia ayama* Mosely, 1937, original designation]. —Ross, 1944:160, 278 [revision]. —Harris and Holzenthal, 1990:453 [revision, key].

This genus of neotrichiine hydroptilids is widespread across North and Central America. Three of the seven known species occur in the Neotropics, with one reaching as far south as Ecuador. Larvae and their cases were first described by Ross (1944) and have been redescribed since (Wiggins 1996). They are recognized easily by their silken cases with reinforced ridges. They are found in rapidly flowing reaches of rivers and streams. Adults are frequently taken at light at night.

***ayama*** Mosely, 1937:182 [Type locality: Mexico, Guerrero, Cocula; BMNH; ♂]. —Ross, 1944:160, 279 [♂; /female; larva; case; distribution]. —Bueno-Soria and Flint, 1978:202 [distribution]. —Holzenthal, 1988c:61 [distribution]. —Harris and Holzenthal, 1990:458 [♀; distribution]. —Maes, 1999:1194 [checklist]. —Baumgardner and Bowles, 2005:11 [distribution]. —Blinn and Ruiter, 2006:333 [biology]. —Blinn and Ruiter, 2009a:305 [biology]. —Bowles et al., 2007:21 [distribution; biology]. —Chamorro-Lacayo et al., 2007:43 [checklist]. —Flint, 2011:104 [checklist].

**Distribution.** Canada, Costa Rica, Honduras, Mexico, Nicaragua, U.S.A.

***illobia*** Harris and Holzenthal, 1990:456 [Type locality: Costa Rica, Puntarenas, Río Guineal, ca. 1 km (air) E Finca Helechales, 9.076°N, 83.092°W; NMNH; ♂; ♀].

**Distribution.** Costa Rica, Ecuador.

***rualda*** Mosely, 1937:183 [Type locality: Mexico, Chiapas, Barranca Honda; BMNH; ♂]. —Bueno-Soria and Flint, 1978:202 [distribution]. —Holzenthal, 1988c:62 [distribution]. —Harris and Holzenthal, 1990:456, 459 [♀; distribution]. —Chamorro-Lacayo et al., 2007:43 [checklist]. —Bueno-Soria and Barba-Álvarez, 2011:357 [checklist].

**Distribution.** Costa Rica, Mexico, Nicaragua.

***tuscaloosa*** Harris and Sykora, 1996:23 [Type locality: U.S.A., Tuscaloosa County, Big Sandy Creek at 4.5 mi south of Coaling on an unnumbered county road; NMNH; ♂]. —Harris and Flint, 2016:7 [distribution].

**Distribution.** Mexico, U.S.A.

#### Genus *Mejicanotrichia* Harris and Holzenthal [7]

*Mejicanotrichia* Harris and Holzenthal, 1997:129 [Type species: *Alisotrichia blantoni* Flint, 1970, original designation]. —Santos et al., 2016a:471 [phylogenetic placement].

This genus was established for a distinctive segregate of species from the polytypic genus *Alisotrichia*, originally noted by Harris and Holzenthal (1993). They were confirmed to be members of Leucotrichiinae, Alisotrichiini by Santos et al. (2016). Adults are generally day active and are found on the vegetation and substrate along waterfalls, cascades, and turbulent streams. The very distinctive larva was illustrated and described by Wiggins (1996) in the genus *Alisotrichia* (Bowles et al. 1999). They occur on the surfaces of rocks and do not build a case, although just prior to pupation the larva constructs a simple, silken enclosure; this free-living behavior is typical of other members of the Alisotrichiini (Santos et al. 2016).

***blantoni*** (Flint), 1970:28 [Type locality: Mexico, San Luis Potosi, Rancho Quemado, 3.5 mi S Tamazunchale; NMNH; ♂; in *Alisotrichia*]. —Bueno-Soria and Flint, 1978:200 [distribution]. —Harris and Holzenthal 1997:131 [♂; ♀; redescription; to *Mejicanotrichia*]. —Bowles et al., 1999:46 [larva].

**Distribution.** Mexico.

***estaquillosa*** Harris and Holzenthal, 1997:135 [Type locality: Mexico, Nuevo Leon, Mpio. de Santiago, Cola de Caballo below falls, 3 km SW Cienquilla; NMNH; ♂; ♀;]. —Bowles et al., 1999:47 [larva].

—*Alisotrichia* species Wiggins, 1996:80 [larva]. —Harris and Holzenthal, 1997:136 [to synonymy].

**Distribution.** Mexico.

***harrisi*** Bueno-Soria and Barba-Álvarez, 1999a:118 [Type locality: Mexico, Guerrero, Municipio de Taxco, Teusisapan, Río Temascalapa, 18°25.083'N, 99°41.490'W; IBUNAM; ♂].

**Distribution.** Mexico.

***rara*** Bueno-Soria and Barba-Álvarez, 1999a:118 [Type locality: Mexico, Guerrero, Municipio de Taxco, Teusisapan, Río Temascalapa, 18°25.083'N, 99°41.490'W; IBUNAM; ♂].

**Distribution.** Mexico.

***tamaza*** (Flint), 1970:28 [Type locality: Mexico, Oaxaca, Tamazulapan; NMNH; ♂; in *Alisotrichia*]. —Bueno-Soria and Flint, 1978:200 [distribution]. —Harris and Holzenthal, 1997:133 [♂; ♀; redescription; to *Mejicanotrichia*].

**Distribution.** Mexico.

***tridentata*** (Bueno-Soria and Hamilton), 1986:301 [Type locality: Mexico, Chiapas, tributario del Rio Teapa, 3 km N Ixhuatan; NMNH; ♂; in *Alisotrichia*]. —Harris and Holzenthal, 1997:134 [♂; ♀; redescription; to *Mejicanotrichia*]. —Bueno-Soria and Barba-Álvarez, 2011:357 [checklist].

**Distribution.** Mexico.

***trifida*** (Flint), 1970:29. [Type locality: Guatemala, Izabal, Las Escobas near Matias de Galvez; NMNH; ♂; in *Alisotrichia*]. —Harris and Holzenthal, 1997:134 [♂; redescription; to *Mejicanotrichia*].

**Distribution.** Guatemala.

#### Genus *Metrichia* Ross [129]

*Metrichia* Ross, 1938a:9 [Type species: *Orthotrichia nigritta* Banks, 1907b, original designation]. —Flint, 1968a:48 [to status of subgenus in *Ochrotrichia*]. —Bueno-Soria and Flint, 1978:204 [as subgenus of *Ochrotrichia*; catalog; distribution]. —Wiggins, 1996:92 [returned to full generic status]. —Flint and Bueno-Soria, 1998:489 [checklist; bibliography]. —Bueno-Soria, 2002:224 [revision; Mexican species]. —Angrisano and Sganga, 2005:114 [Argentinian species; key]. —Santos et al., 2016b:1 [new species; integrative taxonomy].

*Argentitrichia* Jacquemart, 1963:339 [Type species: *Argentitrichia bulbosa* Jacquemart, 1963, by monotypy]. —Marshall, 1979:186 [to synonymy].

The genus *Metrichia*, placed in the Ochrotrichiinae, is among the most species diverse genera in the Hydroptilidae and also among all Neotropical caddisflies. A few species are known from the southwestern United States, but many more are recorded from Central and South America, and both the Greater and Lesser Antilles.

Larvae have been described for a number of species (Edwards and Arnold 1961, Flint 1964a, Wiggins 1996, Angrisano and Sganga 2005). They are found in streams and spring runs, often in tangles of filamentous algae (Wiggins 1996). They generally construct a purse shaped case of silk and plant fragments, but often add two “chimneys” to the dorsal margin (Botosaneanu and Flint 1982, Angrisano and Sganga 2005).

***aberrans*** (Flint), 1972a:14 [Type locality: Mexico, Veracruz, Fortin de las Flores; NMNH; ♂; in Ochrotrichia (Metrichia)]. —Bueno-Soria and Flint, 1978:204 [distribution]. —Bueno-Soria, 2002:240 [checklist].

**Distribution.** Mexico.

***acicula*** Bueno-Soria and Holzenthal, 2003:175 [Type locality: Costa Rica, Guanacaste, Río Mena, 4.2km W Santa Cecilia, 11.059°N, 85.448°W, el. 260 m; UMSP; ♂].

**Distribution.** Costa Rica.

***acuminata*** Santos, Takiya and Nessimian, 2016b:8 [Type locality: Brazil, Ceará, Ubajara, Parque Nacional de Ubajara, Cachoeira do Gameleira, 03°50'21"S, 40°54'23"W, el. 880 m; CZMA; ♂].

**Distribution.** Brazil.

***adamsae*** Flint and Bueno-Soria, 1998:493 [Type locality: Peru, Madre de Dios, Pakitza, 12°7'S, 70°58'W; NMNH; ♂].

**Distribution.** Peru

***alajuela*** Bueno-Soria and Holzenthal, 2003:177 [Type locality: Costa Rica, Alajuela, Reserva Forestal San Ramón, Río San Lorencito and tributaries, 10.216°N, 84.607°W, el. 980 m; UMSP; ♂].

**Distribution.** Costa Rica.

***alhoma*** Oláh and Johanson, 2011:204 [Type locality: Peru, San Martin Prov., La Catarata de Ahuashiyascu, 6°27.544'S, 76°18.192'W; NHRS; ♂].

**Distribution.** Peru.

***amplitudinis*** Bueno-Soria and Holzenthal, 2003:177 [Type locality: Costa Rica, Cartago, Reserva Tapantí, Río Grande de Orosí, 9.686°N, 83.756°W, el. 1650 m; UMSP; ♂].

**Distribution.** Costa Rica.

***ancora*** Bueno-Soria and Holzenthal, 2003:177 [Type locality: Costa Rica, Guanacaste, Río Góngora (Sulfur mine), 4 km (air) NE Quebrada Grande, 10.887°N, 85.470°W, el. 590 m; UMSP; ♂].

**Distribution.** Costa Rica.

***angulosa*** Bueno-Soria and Holzenthal, 2003:179 [Type locality: Costa Rica, Puntarenas, Río Cotón in Las Alturas, 8.938°N, 82.826°W, el. 1360 m; UMSP; ♂].

**Distribution.** Costa Rica.

***anisoscola*** (Flint), 1991:58 [Type locality: Colombia, Dpto. Antioquia, Quebrada La Ayura, Envigado (trap B); NMNH; ♂; in Ochrotrichia (Metrichia)]. —Muñoz-Quesada, 2000:278 [checklist].

**Distribution.** Colombia.

***araguensis*** (Flint), 1981a:29 [Type locality: Venezuela, Aragua, Dos Riitos, 6km N Rancho Grande; NMNH; ♂; in Ochrotrichia (Metrichia)].

**Distribution.** Venezuela.

***arenifera*** (Flint), 1980a:214 [Type locality: Peru, Dept. del Cuzco, Rio Vilcanota above P’Isaq; NMNH; ♂; in Ochrotrichia (Metrichia)].

**Distribution.** Peru.

***argentinica*** Schmid, 1958b:195 [Type locality: Argentina, Siambon, Tucuman; NMNH; ♂]. —Flint, 1974e:87 [as Ochrotrichia (Metrichia)]. —Flint and Bueno-Soria 1998:490 [as *Metrichia*]. —Angrisano, 1999:33 [checklist]. —Angrisano and Sanga, 2005:116 [larva; distribution]. —Isa Miranda and Rueda Martín, 2014:199 [distribution].

**Distribution.** Argentina, Chile, Peru.

***arizonensis*** (Flint), 1972:12 [Type locality: U.S.A., Arizona, Santa Cruz Co., Sycamore Canyon, Atascosa Mts.; NMNH; ♂; in Ochrotrichia (Metrichia)]. —Bueno-Soria et al., 2007:33 [distribution].

**Distribution.** Mexico, U.S.A.

***avon*** (Bueno-Soria), 1983a:82 [Type locality: Mexico, Chiapas, Cascada de Misolha, 20km SE Palenque; IBUNAM; ♂; in Ochrotrichia (Metrichia)]. —Bueno-Soria, 2002:240 [checklist]. —Bueno-Soria and Holzenthal, 2003:196 [distribution]. —Bueno-Soria et al., 2005:75 [distribution].

**Distribution.** Costa Rica, Mexico.

***azul*** Santos, Takiya and Nessimian, 2016b:13 [Type locality: Brazil, Paraná, Céu Azul, Parque Nacional do Iguaçu, Rio Azul, 25°09;21"S, 53°47;44"W, el. 510 m; DZRJ; ♂].

**Distribution.** Brazil.

***bidentata*** (Flint), 1983a:41 [Type locality: Argentina, Pcia. Neuquen, 13km E Quila Quina; NMNH; ♂; in Ochrotrichia (Metrichia)]. —Flint and Bueno-Soria 1998:490 [as *Metrichia*]. —Angrisano, 1999:33 [checklist]. —Angrisano and Sanga, 2005:116 [larva; distribution].

**Distribution.** Argentina, Chile.

***biungulata*** (Flint), 1972a:13 [Type locality: Panama, Cerro Campana; NMNH; ♂; in Ochrotrichia (Metrichia)]. —Aguila, 1992:539 [distribution]. —Bueno-Soria and Santiago-Fragoso, 2002:252 [checklist]. —Bueno-Soria and Holzenthal, 2003:196 [distribution]. —Armitage et al., 2015b:7 [checklist]. —Armitage and Cornejo, 2015:195 [checklist].

**Distribution.** Costa Rica, Panama.

***bola*** (Flint), 1991:58 [Type locality: Colombia, Dpto. Antioquia, Quebrada La Cebolla, El Retiro (trap A); NMNH; ♂; in Ochrotrichia (Metrichia)]. —Muñoz-Quesada, 2000:278 [checklist].

**Distribution.** Colombia.

***bonita*** Santos, Takiya and Nessimian, 2016b:15 [Type locality: Brazil, Mato Grosso do Sul, Bonito, Rio Formosinho, 21°1'16"S, 56°26'47"W, el. 275 m; DZRJ; ♂; larva; biology].

**Distribution.** Brazil.

***bostrychion*** Thomson and Holzenthal, 2012:23 [Type locality: Venezuela, Monagas, Guachero Cave National Park at La Paila waterfall, 10°10.322'N, 63°33.315'W, el. 1110 m; UMSP; ♂].

**Distribution.** Venezuela.

***bracui*** Santos, Takiya and Nessimian, 2016b:19 [Type locality: Brazil, Rio de Janeiro, Angra dos Reis, Rio Bracuí, 23°0'23"S, 44°29'15"W, el. 75 m; DZRJ; ♂].

**Distribution.** Brazil.

***brevitas*** Bueno-Soria and Santiago-Fragoso, 2002:252 [Type locality: Panama, Chiriqui, Guadalupe, Arriba 8°52'26"N, 82°33'13"W; NMNH; ♂]. —Armitage et al., 2015b:7 [checklist]. —Armitage and Cornejo, 2015:195 [checklist].

**Distribution.** Panama.

***bulbosa*** (Jacquemart), 1963:339 [Type locality: Argentina, [San Juan], Rio Sasso; IRSNB; ♂; in *Argentitrichia*]. —Mangeaud, 1996:154 [distribution]. —Angrisano, 1999:33 [checklist]. —Angrisano and Sanga, 2005:117 [♀; larva; distribution]. —Isa Miranda and Rueda Martín, 2014:199 [distribution].

**Distribution.** Argentina.

***cafetalera*** Botosaneanu, 1980:110 [Type locality: Cuba, Prov. Las Villas, Cafetal Gavina, La Sierrita; ZMUA; ♂]. —Botosaneanu, 1979:49 [*nomen nudum* (name included in checklist); distribution]. —Botosaneanu, 1995:26 [♂; ♀; distribution]. —Flint, 1996c:16 [checklist]. —Flint and Pérez-Gelabert, 1999:40 [checklist]. —Botosaneanu, 2002:84 [checklist]. —Flint and Sykora, 2004:32 [distribution]. —Naranjo López and González Lazo, 2005:149 [checklist]. —Pérez-Gelabert, 2008:300 [checklist].

**Distribution.** Cuba, Dominican Republic.

***campana*** (Flint), 1968b:62 [Type locality: Dominica, Dleau Gommier; NMNH; ♂; in Ochrotrichia (Metrichia)]. —Flint and Sykora, 1993:50 [checklist]. —Botosaneanu, 2002:84 [checklist]. —Botosaneanu and Thomas, 2005:55 [checklist].

**Distribution.** Dominica, Guadeloupe.

***caraca*** Santos, Takiya and Nessimian, 2016b:20 [Type locality: Brazil, Minas Gerais, Catas Altas, RPPN Santuário do Caraça, Ribeirão, Caraça; DZRJ; ♂].

**Distribution.** Brazil.

***carbetina*** (Botosaneanu), 1994a:38 [Type locality: Guadeloupe, Chute du Carbet, 580m; ZMUA; ♂; in Ochrotrichia (Metrichia)]. —Botosaneanu, 2002:84 [checklist]. —Botosaneanu and Thomas, 2005:40 [distribution].

**Distribution.** Guadeloupe, Martinique.

***ceer*** (Flint), 1992c:387 [Type locality: Puerto Rico, El Verde Field Station, Quebrada Prieta; NMNH; ♂; in Ochrotrichia (Metrichia)]. —Botosaneanu, 2002:84 [checklist].

**Distribution.** Puerto Rico.

***circulatrix*** Bueno-Soria, 2002:224 [Type locality: Tabasco, Municipio de Huimanguillo, Arroyo las Flores, Villa de Guadalupe, 2a sección Los Chimalapas, km 5+920, Ruta Malpasito-Carlos A. Madrazo, 17°22'05"N, 93°36'25"W; CNIN; ♂].

**Distribution.** Mexico.

***circuliforme*** Santos, Takiya and Nessimian, 2016b: 22 [Type locality: Brazil, Rio de Janeiro, Itatiaia, Rio das Pedras, Cachoeira de Deus,. 22°25'0” S, 44°32'50"W, el. 689 m; DZRJ; ♂].

**Distribution.** Brazil.

***continentalis*** (Flint), 1972a:14 [Type locality: Panama, Canal Zone, Barro Colorado Island; NMNH; ♂; in Ochrotrichia (Metrichia)]. —Aguila, 1992:539 [distribution]. —Bueno-Soria and Santiago-Fragoso, 2002:252 [checklist]. —Armitage et al., 2015b:7 [checklist]. —Armitage and Cornejo, 2015:195 [checklist].

**Distribution.** Panama.

***crenula*** Bueno-Soria, 2002:227 [Type locality: Mexico, Morelos, Huautla Estación CEAMISH, 2.5 km N 4 km W, 18°27'.871N, 99°02'.475W, el. 940 m; CNIN; ♂].

**Distribution.** Mexico.

***cuenca*** (Harper and Turcotte), 1985:139 [Type locality: Ecuador, small stream outlet of Laguna Verde Cocha, near junction with Rio Matadero, Chirimachay, Quinuas Valley; UMQ; ♂; in Ochrotrichia (Metrichia)].

**Distribution.** Ecuador.

***cuniapiru*** Angrisano, in Angrisano and Sanga 2005:114 [Type locality: Argentina, Misiones, Cuñá Pirú Provincial Park, Cuñá Pirú Stream; UNLP; ♂; ♀].

**Distribution.** Argentina.

***curta*** Santos, Takiya and Nessimian, 2016b:24 [Type locality: Brazil, Rio de Janeiro, Itatiaia, Rio das Pedras, 22°24'33"S, 44°33'08"W, el. 706 m; DZRJ; ♂].

**Distribution.** Brazil.

***cuspidata*** (Flint), 1991:57 [Type locality: Colombia, Dpto. Antioquia, 10 km E Medellin, road to Las Palmas; NMNH; ♂; in Ochrotrichia (Metrichia)]. —Muñoz-Quesada, 2000:278 [checklist]. —Oláh and Johanson, 2011:205 [distribution].

**Distribution.** Colombia, Mexico.

***decora*** Bueno-Soria and Holzenthal, 2003:179 [Type locality: Costa Rica, Heredia, Río Sarapiquí, 7 km W Puerto Viejo, 10.452°N, 84.067°W, el. 50 m; UMSP; ♂].

**Distribution.** Costa Rica.

***difusa*** Bueno-Soria and Santiago-Fragoso, 2002:246 [Type locality: Panama, Barro Colorado, Island Snyder Molino Makers 3; NMNH; ♂]. —Armitage et al., 2015b:7 [checklist]. —Armitage and Cornejo, 2015:195 [checklist].

**Distribution.** Panama.

***diosa*** Flint and Bueno-Soria, 1998:491 [Type locality: Peru, Madre de Dios, Pakitza, 12°7'S, 70°58'W; NMNH; ♂].

**Distribution.** Peru.

***disparilis*** (Flint), 1983a:41 [Type locality: Argentina, Pcia. Tucuman, Rt. 307, 33.7km W Acheral; NMNH; ♂; in Ochrotrichia (Metrichia)]. —Angrisano, 1999:33 [checklist]. —Angrisano and Sanga, 2005:119 [larva; distribution]. —Isa Miranda and Rueda Martín, 2014:199 [distribution].

**Distribution.** Argentina.

***eltera*** Oláh and Johanson, 2011:205 [Type locality: French Guiana, Approuaguekaw, Kaw Mt, 4°33.035'N, 52°11.661'W, el. 104 m; NHRS; ♂].

**Distribution.** French Guiana.

***enigmatica*** Bueno-Soria and Santiago-Fragoso, 2002:249 [Type locality: Panama, San Blas, Río Carti Grande, 2 km W Nusagandi; NMNH; ♂]. —Armitage et al., 2015b:7 [checklist]. —Armitage and Cornejo, 2015:195 [checklist].

**Distribution.** Panama.

***espera*** Botosaneanu, 1977:265 [Type locality: Cuba, Pinar del Rio, Soroa, Rio Manantiales; NMNH; ♂]. —Botosaneanu, 1979:49 [distribution]. —Botosaneanu, 1980:111 [♀]. —Flint, 1996c:16 [checklist]. —Botosaneanu, 2002:84 [checklist]. —Naranjo López and González Lazo, 2005:149 [checklist].

**Distribution.** Cuba.

***excisa*** (Kumanski), 1987:20 [Type locality: Cuba, Province Las Villas, Sierra de Trinidad, small torrent on road Trinidad—Topes de Colantes; NMSB; ♂; ♀; in Ochrotrichia (Metrichia)]. —Flint, 1996c:16 [checklist]. —Botosaneanu, 2002:84 [checklist]. —Naranjo López and González Lazo, 2005:149 [checklist].

**Distribution.** Cuba.

***exclamationis*** (Flint), 1968b:64 [Type locality: Dominica, Clarke Hall, cocoa trail; NMNH; ♂; in Ochrotrichia (Metrichia)]. —Flint and Sykora, 1993:50 [checklist]. —Botosaneanu, 1994a:38 [distribution]. —Botosaneanu, 2002:84 [checklist]. —Botosaneanu and Thomas, 2005:55 [checklist].

**Distribution.** Dominica, Guadeloupe.

***extragma*** Bueno-Soria and Barba-Álvarez, 1999b:30 [Type locality: Mexico, Guerrero, Taxco, Totoapan, río Temascalapa, 8 km NW Ahuehuepan, Rd. 51, 18°22.70'N, 99°39.77'W, el. 900 m; CNIN; ♂]. —Bueno-Soria, 2002:240 [checklist].

**Distribution.** Mexico.

***farofa*** Santos, Takiya and Nessimian, 2016b:24 [Type locality: Brazil, Minas Gerais, Jaboticatubas, Parque Nacional da Serra do Cipó, Cachoeira da Farofa, 19°22'47"S, 43°34'36"W, el. 811 m; DZRJ; ♂].

**Distribution.** Brazil.

***favus*** (Botosaneanu), in Botosaneanu and Alkins-Koo, 1993:18 [Type locality: Trinidad, two 1st order streams, La Laja catchment of Rio Guanapo; ZMUA; ♂; in Ochrotrichia (Metrichia)]. —Botosaneanu and Sakal, 1992:202 [*nomen nudum* (name included in checklist); distribution; ecology]. —Flint, 1996a:96 [distribution]. —Botosaneanu, 2002:84 [checklist].

**Distribution.** Trinidad.

***florecita*** Bueno-Soria, 2002:228 [Type locality: Mexico, Tabasco, Municipio de Huimanguillo Ejido, Villa de Guadalupe, 1a sección, Cascada Cerro de Las Flores, 17°21'39"N, 93°37'29"W, Rta. Malpasito-Carlos A. Madrazo; CNIN; ♂].

**Distribution.** Mexico.

***fontismoreaui*** (Botosaneanu), 1991a:125 [Type locality: Haiti, Departement du Sud, pres de Camp Perrin, Resurgence du Moreau; ZMUA; ♂; in Ochrotrichia (Metrichia)]. —Botosaneanu, 1995:27 [♀; distribution]. —Flint and Pérez-Gelabert, 1999:40 [checklist]. —Botosaneanu, 2002:85 [checklist]. —Flint and Sykora, 2004:32 [distribution]. —Pérez-Gelabert, 2008:300 [checklist].

**Distribution.** Dominican Republic, Haiti.

***forceps*** Santos, Takiya and Nessimian, 2016b:27 [Type locality: Brazil, Paraná, Céu Azul, Parque Nacional do Iguaçu, Rio Azul, 25°09'21"S, 53°47'44"W, el. 510 m; DZRJ; ♂].

**Distribution.** Brazil.

***formosinha*** Santos, Takiya and Nessimian, 2016b:28 [Type locality: Brazil, Mato Grosso do Sul, Bonito, Rio Formosinho, 21°10'16"S, 56'26”47"W, el. 275 m; DZRJ; ♂].

**Distribution.** Brazil.

***fugga*** Oláh and Johanson, 2011:207 [Type locality: Peru, San Martin Prov., La Catarata de Ahuashiyascu, 6°27.544'S, 76°18.192'W; NHRS; ♂].

**Distribution.** Peru.

***geminata*** (Flint), 1996a:96 [Type locality: Trinidad, streamlet, Lalaja Rd., 520m, 10°43'N, 61°17'W; NMNH; ♂; in Ochrotrichia (Metrichia)]. —Botosaneanu, 2002:85 [checklist].

**Distribution.** Tobago, Trinidad.

***goiana*** Santos, Takiya and Nessimian, 2016b:30 [Type locality: Brazil, Goiás, Alto Paraíso de Goiás, Rio Bartolomeu tributary, 14°07'25"S, 47°30'30"W, el. 1,165 m; DZRJ; ♂].

**Distribution.** Brazil.

***gomboska*** Oláh and Johanson, 2011:208 [Type locality: Peru, Huanuco, Tomayquichua Distr. River Tomayquichua, humid subtropical forest 10°04'27"S, 76°12'36'W, el. 2041 m; NHRS; ♂].

**Distribution.** Peru.

***gordita*** Bueno-Soria and Holzenthal, 2003:183 [Type locality: Costa Rica, Puntarenas, Río Singri, ca. 2 km (air) S Finca Helechales, 9.057°N, 83.082°W, el. 720 m; UMSP; ♂].

**Distribution.** Costa Rica.

***haranga*** Oláh and Johanson, 2011:210 [Type locality: Peru, San Martin Prov., La Catarata de Ahuashiyascu, 6°27.544'S, 76°18.192'W; NHRS; ♂].

**Distribution.** Peru.

***helenae*** Flint and Bueno-Soria, 1998:495 [Type locality: Peru, Madre de Dios, Pakitza, 12°7'S, 70°58'W; NMNH; ♂].

**Distribution.** Peru.

***itabaiana*** Santos, Takiya and Nessimian, 2016b:32 [Type locality: Brazil, Sergipe, Areia Branca, Parque Nacional da Serra de Itabaiana, Rio dos Negros, 10°44'51"S, 37°20'24"W, el. 208 m; DZRJ; ♂].

**Distribution.** Brazil.

***jorobada*** Bueno-Soria, 2002:228 [Type locality: Mexico, Tabasco, Municipio de Huimanguillo, Arroyo las Flores, Villa de Guadalupe, 2a sección Los Chimalapas, km 5+920, Ruta Malpasito-Carlos A. Madrazo, 17°22'05"N, 93°36'25"W; CNIN; ♂].

**Distribution.** Mexico.

***juana*** (Flint), 1964a:60 [Type locality: Puerto Rico, Toro Negro Forest, Dona Juana Creek; NMNH; ♂; ♀; larva; in *Ochrotrichia*]. —Flint, 1968b:82 [checklist]. —Botosaneanu, 2002:85 [checklist].

**Distribution.** Puerto Rico.

***kocka*** Oláh and Johanson, 2011:211 [Type locality: Peru, San Martin Prov., La Catarata de Ahuashiyascu, 6°27.544'S, 76°18.192'W; NHRS; ♂].

**Distribution.** Peru.

***kumanskii jamaicae*** Botosaneanu, in Botosaneanu and Hyslop, 1998:13 [Type locality: Jamaica, Buff Bay River in Green Hill at “Regale”, Blue Mountains, Portland; ZMUA; ♂]. —Botosaneanu, 2002:85 [checklist].

**Distribution.** Jamaica.

***kumanskii kumanskii*** (Botosaneanu), 1991a:128 [Type locality: Haiti, Departement de l’ Quest, Ville Bonheur, Le Saut d’Eau; ZMUA; ♂; in Ochrotrichia (Metrichia)]. —Flint and Pérez-Gelabert, 1999:40 [checklist]. —Botosaneanu, 2002:85 [checklist]. —Flint and Sykora, 2004:33 [distribution]. —Pérez-Gelabert, 2008:300 [checklist].

**Distribution.** Dominican Republic, Haiti.

***lacuna*** (Bueno-Soria), 1983a:79 [Type locality: Mexico, Chiapas, Cascada de Misolha, 20km SE Palenque; IBUNAM; ♂; in Ochrotrichia (Metrichia)]. —Bueno-Soria, 2002:240 [checklist]. —Bueno-Soria and Barba-Álvarez, 2011:357 [checklist].

**Distribution.** Mexico.

***lemniscata*** (Flint), 1972a:14 [Type locality: Panama, Chiriqui, David, Rovira; NMNH; ♂; in Ochrotrichia (Metrichia)]. —Aguila, 1992:539 [distribution]. —Bueno-Soria and Santiago-Fragoso, 2002:252 [checklist]. —Bueno-Soria and Holzenthal, 2003:196 [distribution]. —Armitage et al., 2015b:7 [checklist]. —Armitage and Cornejo, 2015:195 [checklist].

**Distribution.** Costa Rica, Panama.

***lenophora*** (Flint), 1991:54 [Type locality: Colombia, Dpto. Antioquia, 10km E. Medellin, road to Guarne; NMNH; ♂; in Ochrotrichia (Metrichia)]. —Muñoz-Quesada, 2000:278 [checklist].

**Distribution.** Colombia.

***longispina*** Flint and Sykora, 2004:33 [Type locality: Dominican Republic, [La Vega Province], Convento, 12 km S Constanza; NMNH; ♂; ♀]. —Pérez-Gelabert, 2008:300 [checklist].

**Distribution.** Dominican Republic.

***longissima*** Santos, Takiya and Nessimian, 2016b:34 [Type locality: Brazil, Rio de Janeiro, Itatiaia, Rio Palmital, 22°25'34"S, 44°32'52"W, el. 637 m; DZRJ; ♂].

**Distribution.** Brazil.

***longitudinis*** Bueno-Soria, 2002:231 [Type locality: Mexico, Tabasco, Municipio de Huimanguillo Ejido Villa de Guadalupe, 1a sección, Cascada Cerro de Las Flores, 17°21'39"N, 93°37'29"W, Rta. Malpasito-Carlos A. Madrazo; CNIN; ♂].

**Distribution.** Mexico.

***luna*** Bueno-Soria and Holzenthal, 2003:183 [Type locality: Costa Rica, Alajuela, Reserva Forestal San Ramón, Río San Lorencito and tribs., 10.216°N, 84.607°W, el. 980 m; UMSP; ♂].

**Distribution.** Costa Rica.

***macrophallata*** (Flint), 1991:57 [Type locality: Colombia, Dpto. Antioquia, Quebrada Honda, Marsella, 12km SW Fredonia; NMNH; ♂; in Ochrotrichia (Metrichia)]. —Muñoz-Quesada, 2000:278 [checklist].

**Distribution.** Colombia.

***madicola*** (Botosaneanu), 1994a:39 [Type locality: Guadeloupe, Chute du Carpet, 580m; ZMUA; ♂; in Ochrotrichia (Metrichia)]. —Botosaneanu, 2002:85 [checklist]. —Botosaneanu and Thomas, 2005:40 [distribution].

**Distribution.** Guadeloupe, Martinique.

***madre*** Flint and Bueno-Soria, 1998:493 [Type locality: Peru, Madre de Dios, Pakitza, 12°7'S, 70°58'W; NMNH; ♂].

**Distribution.** Peru

***magna*** Bueno-Soria and Holzenthal, 2003:185 [Type locality: Costa Rica, Puntarenas, roadside seep, route 2, just W km 234, 8.976°N, 83.299°W, el. 100 m; UMSP; ♂].

**Distribution.** Costa Rica.

***malada*** (Flint), 1991:55 [Type locality: Colombia, Dpto. Antioquia, Quebrada Agua Mala, 34km NW Medellin, road to San Jeronimo; NMNH; ♂; in Ochrotrichia (Metrichia)]. —Flint and Reyes, 1991:487 [distribution]. —Muñoz-Quesada, 2000:278 [checklist].

**Distribution.** Colombia, Peru.

***mechuda*** Bueno-Soria and Holzenthal, 2003:185 [Type locality: Costa Rica, San José, Río Savegra, ca. San Gerardo de Dota, 9.33°N, 83.48°W, el. 2200 m; CMNH; ♂].

**Distribution.** Costa Rica.

***meta*** Bueno-Soria and Holzenthal, 2003:185 [Type locality: Costa Rica, Guanacaste, Parque Nacional Rincón de la Vieja, Quebrada Zopilote, 10.765°N, 85.309°W, el. 785 m; UMSP; ♂].

**Distribution.** Costa Rica.

***minera*** Bueno-Soria, 2002:231 [Type locality: Mexico, Veracruz, Las Minas; CNIN; ♂].

**Distribution.** Mexico.

***munieca*** Botosaneanu, 1977:264 [Type locality: Cuba, Oriente, Gran Piedra, Arroyos de la Idalia; NMNH; ♂]. —Botosaneanu, 1979:49 [distribution]. —Flint, 1996c:16 [checklist]. —Botosaneanu, 2002:85 [checklist]. —Naranjo López and González Lazo, 2005:149 [checklist].

**Distribution.** Cuba.

***necopina*** Botosaneanu and Thomas, 2005:40 [Type locality: Martinique, Riv Lezarde au départ de la route forestiere de Palourde, el. 250 m; ZMUA; ♂].

**Distribution.** Martinique.

***neotropicalis*** Schmid, 1958b:195 [Type locality: Argentina, Siamba, Tucuman; NMNH; ♂]. —Flint, 1967a:56 [distribution; misidentified as *argentinica*]. —Flint, 1974e:88 [checklist]. —Flint, 1980a:216 [correction of switched captions in Schmid, 1958b]. —Flint, 1990:118 [distribution]. —Mangeaud, 1996:154 [distribution]. —Angrisano, 1999:33 [checklist]. —Angrisano and Sanga, 2005:117 [♀; larva; pupa; distribution]. —Muzón et al., 2005: 57 [distribution]. —Miserendino and Brand, 2007:312 [biology]. —Brand and Miserendino, 2011a:35 [biology]. —Brand and Miserendino, 2011b:143 [biology]. —Brand and Miserendino, 2014:6 [community ecology]. —Isa Miranda and Rueda Martín, 2014:199 [distribution].

**Distribution.** Argentina, Chile, Peru.

***nigritta*** (Banks), 1907b:163 [Type locality: United States, Texas, Austin; MCZ; ♂; in *Orthotrichia*]. —Ross, 1938a:9 [type species of *Metrichia*; lectotype; ♂]. —Edwards and Arnold, 1961:411 [larva]. —Flint, 1968:48 [to subgenus; in Ochrotrichia (Metrichia)]. —Flint, 1972a:12 [♂; distribution]. —Bueno-Soria and Flint, 1978:204 [distribution]. —Wiggins, 1996:92 [larva]. —Bueno-Soria, 2002:240 [checklist]. —Bueno-Soria and Santiago-Fragoso, 2002:252 [distribution]. —Rojas-Ascencio, et al., 2002:377 [distribution]. —Blinn and Ruiter, 2006:333 [biology]. —Bowles et al., 2007:22 [distribution; biology]. —Blinn and Ruiter, 2009b:186 [phenology, distribution]. —Oláh and Johanson, 2011:212 [distribution]. —Armitage et al., 2015b:7 [checklist]. —Armitage and Cornejo, 2015:195 [checklist].

**Distribution.** El Salvador, Mexico, Panama, U.S.A.

***nowaczyki*** Harris and Armitage, 2015:8 [Type locality: Panama, Chiriquí Province, Cuenca 108, Quebrada Grande, Boquete, Valle Escondido, below Sabor Restaurant, 8.77970°N, 82.44016°W, el. 1122 m; MIUP; ♂]. —Armitage et al., 2015b:7 [checklist]. —Armitage and Cornejo, 2015:195 [checklist].

**Distribution.** Panama.

***pakitza*** Flint and Bueno-Soria, 1998:491 [Type locality: Peru, Madre de Dios, Pakitza, 12°7'S, 70°58'W; NMNH; ♂].

**Distribution.** Peru

***palida*** Bueno-Soria and Santiago-Fragoso, 2002:249 [Type locality: Panama, Chiriqui, Guadalupe, Arriba 8°52'26"N, 82°33'13"W; NMNH; ♂]. —Armitage et al., 2015b:7 [checklist]. —Armitage and Cornejo, 2015:195 [checklist].

**Distribution.** Panama.

***patagonica*** (Flint), 1983a:41 [Type locality: Argentina, Pcia. Rio Negro, 5 km 5 Rio Villegas; NMNH; ♂; in Ochrotrichia (Metrichia)]. —Mangeaud, 1996:154 [distribution]. —Angrisano, 1999:33 [checklist]. —Angrisano and Sanga, 2005:121 [♀; larva; case; distribution]. —Brand and Miserendino, 2011a:35 [biology]. —Brand and Miserendino, 2011b:143 [biology]. —Oláh and Johanson, 2011:212 [distribution]. —Brand and Miserendino, 2014:6 [community ecology].

**Distribution.** Argentina, Chile, Peru.

***peluda*** Santos, Takiya and Nessimian, 2016b:35 [Type locality: Brazil, Rio de Janeiro, Itatiaia, 1st order tributary of Rio Palmital, 22°25'40"S, 44°32'46"W, el. 584 m; DZRJ; ♂].

**Distribution.** Brazil.

***penicillata*** (Flint), 1972a:13 [Type locality: Guatemala, Escuintla, Grutas de San Pedro Martir; NMNH; ♂; in Ochrotrichia (Metrichia)]. —Bueno-Soria, 2002:240 [checklist]. —Bueno-Soria and Santiago-Fragoso, 2002:253 [distribution]. —Bueno-Soria and Holzenthal, 2003:196 [distribution]. —Chamorro-Lacayo et al., 2007:43 [checklist]. —Armitage et al., 2015b:7 [checklist]. —Armitage and Cornejo, 2015:195 [checklist].

**Distribution.** Costa Rica, Guatemala, Nicaragua, Panama.

***pernambucana*** Souza and Santos, in Souza, et al.,, 2013b:584 [Type locality: Brazil, Pernambuco State, Tamandaré, Reserva Biológica de Saltinho, Riacho Mamucabas, 35°11'14.0"W, 08°43'21.6"S; DZRJ; ♂]. —Paprocki and França, 2014:46 [checklist].

**Distribution.** Brazil.

***picuda*** Bueno-Soria and Holzenthal, 2003:187 [Type locality: Costa Rica, Alajuela, Reserva Forestal San Ramón, Río San Lorencito and tribs., 10.216°N, 84.607°W, el. 980 m; UMSP; ♂].

**Distribution.** Costa Rica.

***pitu*** Angrisano and Sganga, 2009:60 [Type locality: Argentina, Misiones, Departamento de Cainguás, Parque Provincial Salto Encantado; MACN; ♂].

**Distribution.** Argentina.

***platigona*** (Botosaneanu), in Botosaneanu and Alkins-Koo, 1993:18 [Type locality: Tobago, Argyll River below Argyll waterfall; ZMUA; ♂; in Ochrotrichia (Metrichia)]. —Botosaneanu and Sakal, 1992:202 [*nomen nudum* (name included in checklist); distribution; ecology]. —Flint, 1996a:95 [distribution]. —Botosaneanu, 2002:85 [checklist].

**Distribution.** Tobago, Trinidad, Venezuela.

***potosina*** Bueno-Soria, 2002:232 [Type locality: Mexico, San Luis Potosí, La Cascada del Tamasopo; NMNH; ♂].

**Distribution.** Mexico.

***prolata*** Bueno-Soria and Holzenthal, 2003:187 [Type locality: Costa Rica, Alajuela, Reserva Forestal San Ramón, Río San Lorencito and tribs., 10.216°N, 84.607°W, el. 980 m; UMSP; ♂].

**Distribution.** Costa Rica.

***prolixa*** Bueno-Soria, 2002:233 [Type locality: Mexico, Tabasco, Mpio. Huimanguillo, Villa de Guadalupe, 2a sección los Chimalapas, km 5.92, Rta. Malpasito-Carlos A. Madraz, 17°22'05"N, 93°36'25"W, el. 335 m; CNIN; ♂].

**Distribution.** Mexico.

***protrudens*** (Flint), 1991:57 [Type locality: Colombia, Dpto. Antioquia, 12km N Fredonia, road to Medellin; NMNH; ♂; in Ochrotrichia (Metrichia)]. —Muñoz-Quesada, 2000:278 [checklist].

**Distribution.** Colombia.

***pseudopatagonica*** Bueno-Soria and Holzenthal, 2003:187 [Type locality: Costa Rica, Limón, E.A.R.T.H., forest reserve arroyo, 7.5 km (air) NW Pocora, 10.23°N, 83.56°W, el. 10 m; UMSP; ♂]. —Bueno-Soria and Santiago-Fragoso, 2002:253 [*nomen nudum* (name included in checklist); as *seudopatagonica*; distribution]. —Armitage et al., 2015b:7 [checklist]. —Armitage and Cornejo, 2015:195 [checklist].

**Distribution.** Costa Rica, Panama.

***quadrata*** (Flint), 1972a:14 [Type locality: Mexico, Veracruz, Rio Jamapa, north of Coscomatepec; NMNH; ♂; in Ochrotrichia (Metrichia)]. —Bueno-Soria and Flint, 1978:204 [distribution]. —Bueno-Soria, 2002:241 [checklist]. —Bueno-Soria and Holzenthal, 2003:196 [distribution].

**Distribution.** Costa Rica, Mexico.

***rafaeli*** Santos, Takiya and Nessimian, 2016b:37 [Type locality: Brazil, Ceará, Ubajara, Parque Nacional de Ubajara, Rio das Minas, 03°50'03"S, 40°54'18"W, el. 524; DZRJ; ♂].

**Distribution.** Brazil.

***rawlinsi*** (Flint and Sykora), 1993:58 [Type locality: Dominica, Parish St. Paul, Springfield Estate; CMNH; ♂; in Ochrotrichia (Metrichia)]. —Botosaneanu, 2002:85 [checklist]. —Botosaneanu and Thomas, 2005:42 [distribution].

**Distribution.** Dominica, Martinique.

***riva*** (Bueno-Soria), 1983a:79 [Type locality: Mexico, Chiapas, Cascada de Misolha, 20 km SE Palenque; IBUNAM; ♂; in Ochrotrichia (Metrichia)]. —Bueno-Soria, 2002:241 [checklist]. —Bueno-Soria and Holzenthal, 2003:196 [distribution]. —Bueno-Soria et al., 2005:75 [distribution]. —Bueno-Soria and Barba-Álvarez, 2011:357 [checklist].

**Distribution.** Costa Rica, Mexico.

***rona*** (Flint), 1991:55 [Type locality: Colombia, Dpto. Antioquia, 7km E. San Jerónimo; NMNH; ♂; in Ochrotrichia (Metrichia)]. —Muñoz-Quesada, 2000:278 [checklist].

**Distribution.** Colombia.

***sacculifera*** (Flint), 1991:55 [Type locality: Colombia, Dpto. Antioquia, Quebrada Honda, Marsella, 12km SW Fredonia; NMNH; ♂; in Ochrotrichia (Metrichia)]. —Muñoz-Quesada, 2000:278 [checklist].

**Distribution.** Colombia.

***savegra*** Bueno-Soria and Holzenthal, 2003:191 [Type locality: Costa Rica, San José, Río Savegra, ca. San Gerardo de Dota 9.33°N, 83.48°W, el. 2200 m; CMNH; ♂].

**Distribution.** Costa Rica.

***sencilla*** Harris and Armitage, 2015:8 [Type locality: Panama, Chiriquí Province, Cuenca 108, Quebrada Grande, Boquete, Valle Escondido, below Sabor Restaurant, 8.77970°N, 82.44016°W, el. 1122 m; MIUP; ♂]. —Armitage et al., 2015b:7 [checklist]. —Armitage and Cornejo, 2015:196 [checklist].

**Distribution.** Panama.

***separata*** Bueno-Soria and Holzenthal, 2003:191 [Type locality: Costa Rica, Alajuela, Río Agrio, ca. 3.5 km NE Bajos del Toro, 10.243°N, 84.279°W, el. 1290 m; UMSP; ♂].

**Distribution.** Costa Rica.

***sesquipedalis*** Bueno-Soria and Holzenthal, 2003:191 [Type locality: Costa Rica, San Jose, Río Savegra ca. San Gerardo de Dota 9.33°N, 83.48°W, el. 200 m; CMNH; ♂]. —Bueno-Soria and Santiago-Fragoso, 2002:253 [*nomen nudum* (name included in checklist); distribution]. —Armitage et al., 2015b:7 [checklist]. —Armitage and Cornejo, 2015:196 [checklist].

**Distribution.** Costa Rica, Panama.

***similis*** (Flint), 1968b:62 [Type locality: Dominica, Boiling Lake; NMNH; ♂; in Ochrotrichia (Metrichia)]. —Flint and Sykora, 1993:50 [checklist]. —Botosaneanu, 2002:85 [checklist]. —Botosaneanu and Thomas, 2005:55 [checklist].

**Distribution.** Dominica, Guadeloupe.

***simples*** Santos, Takiya and Nessimian, 2016b:38 [Type locality: Brazil, Paraná, Céu Azul, Parque Nacional do Iguaçu, Rio Azul, 25°09'21"S, 53°47'44"W, el. 510 m; DZRJ; ♂].

**Distribution.** Brazil.

***sonora*** Bueno-Soria, 2002:236 [Type locality: Mexico, Sonora, Cajón Bonito, 38 miles E de A. P. Waters Falls; CAS; ♂].

**Distribution.** Mexico.

***spica*** Bueno-Soria and Holzenthal, 2003:195 [Type locality: Costa Rica, Alajuela, Reserva Forestal San Ramón, Río San Lorencito and tribs., 10.216°N, 84.607°W, el. 980 m; UMSP; ♂].

**Distribution.** Costa Rica.

***squamigera*** (Flint), 1992c:385 [Type locality: Puerto Rico, El Verde Field Station, Quebrada Prieta; NMNH; ♂; in Ochrotrichia (Metrichia)]. —Botosaneanu, 2002:85 [checklist]. —Flint and Sykora, 2004:33 [distribution]. —Pérez-Gelabert, 2008:300 [checklist].

**Distribution.** Dominican Republic, Puerto Rico.

***talhada*** Santos, Takiya and Nessimian, 2016b:40 [Type locality: Brazil, Alagoas, Quebrangulo, Reserva Biológica de Pedra Talhada, Rio Caranguejo, 09°15'26"S, 36°25'08"W, el. 550 m; DZRJ; ♂].

**Distribution.** Brazil.

***temascalapensis*** Bueno-Soria and Barba-Álvarez, 1999b:30 [Type locality: Mexico, Guerrero, Taxco, Teucisapan, Río Temascalapa, 12 km NW Ahuehuepan, Rd. 51, 18°25.56'N, 99°42.5’ W, el. 1052 m; CNIN; ♂]. —Bueno-Soria, 2002:241 [checklist].

**Distribution.** Mexico.

***tere*** Santos, Takiya and Nessimian, 2016b:42 [Type locality: Brazil, Rio de Janeiro, Teresópolis, Parque Nacional da Serra dos Órgãos, Rio Paquequer, 22°27'25"S, 42°59'52"W, el. 1,100 m; DZRJ; ♂].

**Distribution.** Brazil.

***thirysae*** Jacquemart, 1980a:303 [Type locality: Chile, Arica, Vallee d’Azapa, Quebrada Azapa; IRSNB; ♂]. —Flint, 1990:117 [redescription; ♂]. —Angrisano, 1999:33 [checklist].

**Distribution.** Chile.

***triangula*** Bueno-Soria and Santiago-Fragoso, 2002:246 [Type locality: Panama, Barro Colorado; NMNH; ♂]. —Armitage et al., 2015b:7 [checklist]. —Armitage and Cornejo, 2015:196 [checklist].

**Distribution.** Panama.

***trigonella*** (Flint) 1972a:13 [Type locality: Mexico, Veracruz, Fortin de las Flores; NMNH; ♂; in Ochrotrichia (Metrichia)]. —Bueno-Soria and Flint, 1978:204 [distribution]. —Bueno-Soria, 2002:241 [checklist].

**Distribution.** Honduras, Mexico.

***triquetra*** Bueno-Soria and Holzenthal, 2003:195 [Type locality: Costa Rica, San José, Río Savegra, ca. San Gerardo de Dota, 9.33°N, 83.48°W, el. 2200 m; CMNH; ♂]. —Bueno-Soria and Santiago-Fragoso, 2002:253 [distribution]. —Armitage et al., 2015b:7 [checklist]. —Armitage et al., 2015b:7 [checklist]. —Armitage and Cornejo, 2015:196 [checklist].

**Distribution.** Costa Rica, Panama.

***trispinosa*** (Bueno-Soria), 1977:142 [Type locality: Mexico, Veracruz, Eyipantla; IBUNAM; ♂; in Ochrotrichia (Metrichia)]. —Bueno-Soria, 2002:241 [checklist].

**Distribution.** Mexico.

***truncata*** Bueno-Soria and Holzenthal, 2003:195 [Type locality: Costa Rica, Alajuela, Río Pizote, ca. 5 km (air) S Brasilia, 10.972°N, 84.345°W, el. 390 m; UMSP; ♂]. —Bueno-Soria and Santiago-Fragoso, 2002:253 [*nomen nudum* (name included in checklist); distribution].

**Distribution.** Costa Rica.

***ubajara*** Santos, Takiya and Nessimian, 2016b:43 [Type locality: Brazil, Ceará, Ubajara, Parque Nacional de Ubajara, Rio das Minas, 03°49'58"S, 40°53'53"W, el. 420 m; DZRJ; ♂].

**Distribution.** Brazil.

***vulgaris*** Santos, Takiya and Nessimian, 2016b:45 [Type locality: Brazil, Rio de Janeiro, Itatiaia, Rio Palmital, 22°25'34"S, 44°32'52"W, el. 637 m; DZRJ; ♂].

**Distribution.** Brazil.

***warema*** (Flint), 1974c:61 [Type locality: Suriname, Litani River, Waremapan Rapids; RNH; ♂; in Ochrotrichia (Metrichia)].

**Distribution.** Suriname.

***yalla*** (Flint), 1968a:50 [Type locality: Jamaica, St. Andrew, Chestervale, Yallahs River; NMNH; ♂; in Ochrotrichia (Metrichia)]. —Flint, 1968b:82 [checklist]. —Botosaneanu, 2002:85 [checklist].

**Distribution.** Jamaica.

***yavesia*** Bueno-Soria, 2002:239 [Type locality: Mexico, Oaxaca, Santa María de Yavesia (Planta embotelladora de agua), 17°13'36"N, 96°25'35"W, el. 1930 m; CNIN; ♂].

**Distribution.** Mexico.

#### Genus *Neotrichia* Morton [144]

*Cyllene* Chambers, 1873:124 [Type species: *Cyllene minutisimella* Chambers, 1873, by monotypy. Preoccupied several times, *vide* Fischer, 1961].

*Neotrichia* Morton, 1905:72 [Type species: *Neotrichia collata* Morton, 1905, by monotypy]. ­­—Keth et al., 2015:5 [revision North American and Caribbean species, key].

*Microsiphon* Müller, 1921:525 [Type species: no species ever included. Preoccupied by Del Guercio, 1907]. —Flint et al., 1999a:77 [to synonymy].

*Exitrichia* Mosely, 1937:170 [Type species: *Exitrichia anahua* Mosely, 1937, original designation]. —Ross, 1944:154 [to synonymy].

*Dolotrichia* Mosely, 1937:177 [Type species: *Dolotrichia canixa* Mosely, 1937, original designation]. —Ross, 1944:154 [to synonymy].

*Guerrotrichia* Mosely, 1937:179 [Type species: *Guerrotrichia caxima* Mosely, 1937, original designation]. —Ross, 1944:154 [to synonymy].

*Lorotrichia* Mosely, 1937:181 [Type species: *Lorotrichia hiaspa* Mosely, 1937, original designation]. —Ross, 1944:154 [to synonymy].

*Neotrichia* of the Neotrichiinae is second only to *Ochrotrichia* in numbers of species among the Neotropical hydroptilids. In addition to being diverse in species, the genus is widely distributed across North, Central, and South America, and the West Indies. Marshall (1979) divided *Neotrichia* into six species groups, roughly corresponding to the genera erected by Mosely (1937), and later synonymized by Ross (1944). In recent years, the number of described *Neotrichia* has increased rapidly, making the species groupings of Marshall less clearly defined. Keth et al. (2015) provided a recent revision of the North American and Caribbean species, and divided this fauna into six species groups. It is likely that some of the species described or recorded only from Arizona, Texas and other southwestern USA states will also be discovered in adjacent northern Mexico.

Larvae were first described by Ross (1944), but few other larvae of the many described species have been associated (Botosaneanu 1994b, Flint 1964a, Wiggins 1996). Those that are known construct short, tapered cases of small sand grains. They live in rapidly flowing streams and rivers on and under rocks and boulders. Adults are commonly taken in large numbers at light.

***abbreviata*** Flint, 1983a:48 [Type locality: Brazil, Edo. Santa Catarina, Nova Teutonia; NMNH; ♂]. —Angrisano, 1995b:511 [distribution]. —Angrisano, 1999:32 [checklist]. —Paprocki et al., 2004:11 [checklist]. —Paprocki and França, 2014:46 [checklist].

**Distribution.** Brazil, Uruguay

***abbreviatoides*** Angrisano, 1995b:513 [Type locality: Uruguay, Artigas, Ao. de la Invernada; FHCU; ♂]. —Angrisano, 1999:32 [checklist].

**Distribution.** Uruguay.

***aequispina*** Angrisano, 1995b:515 [Type locality: Uruguay, Tacuarembo, Tbo. Chico; FHCU; ♂]. —Angrisano, 1999:32 [checklist]. —Keth, 2004:172 [♂].

**Distribution.** Uruguay.

***alata*** Flint, 1968a:37 [Type locality: Jamaica, Portland, Rio Grande at Fellowship; NMNH; ♂; ♀]. —Flint, 1968b:81 [checklist]. —Botosaneanu, 1979:51 [distribution]. —Flint, 1996c:16 [checklist]. —Botosaneanu, 2002:85 [checklist]. —Naranjo López and González Lazo, 2005:149 [checklist]. —Keth et al., 2015:56 [♂].

**Distribution.** Cuba, Jamaica.

***alsa*** Oláh and Johanson, 2011:169 [Type locality: Peru, San Martin Prov., Rio Negro, 37 km (rd.) W Moyobamba, near Olmos-Tarapoto rd., 6°00.278'S, 77°15.437'W; NHRS; ♂].

**Distribution.** Peru.

***alyshae*** Keth, in Keth, Harris and Armitage, 2015:57 [Type locality: Mexico, Sonora, Rio Aros at Arroyo El Pavo; NMNH; ♂].

**Distribution.** Mexico.

***amplector*** Keth, 2004:165 [Type locality: Mexico, Tabasco, Teapa, Grutas de Colona, Rio Puyacatengo; NMNH; ♂]. —Keth et al., 2015:58 [♂].

**Distribution.** Mexico.

***amplio*** Keth, 2004:164 [Type locality: Belize, Orange Walk District. New River Lagoon, dock area at Lamanai Ruins; NMNH; ♂].

**Distribution.** Belize.

***anahua*** (Mosely), 1937:170 [Type locality: Mexico, Chiapas, Dolores; BMNH; ♂; in *Exitrichia*]. —Bueno-Soria and Flint, 1978:202 [distribution]. —Bueno-Soria et al., 2007:33 [distribution]. —Bueno-Soria and Barba-Álvarez, 2011:357 [checklist]. —Keth et al., 2015:59 [♂].

**Distribution.** Mexico.

***angulata*** Flint, 1983a:48 [Type locality: Uruguay, Dpto. Artigas, Arroyo de la Invernada; NMNH; ♂]. —Angrisano, 1995b:511 [distribution]. —Angrisano, 1999:32 [checklist].

**Distribution.** Uruguay.

***arista*** Harris, 1990:251 [Type locality: Venezuela, Territorio Federal Amazonas, Río Cataniapo, 10 km S Puerto Ayacucho; NMNH; ♂].

**Distribution.** Venezuela.

***armata*** Botosaneanu, in Botosaneanu and Alkins-Koo, 1993:20 [Type locality: Tobago, Argyll River below Argyll waterfall; ZMUA; ♂]. —Botosaneanu and Sakal, 1992:202 [*nomen nudum* (name included in checklist); distribution; ecology]. —Flint, 1996a:100 [distribution]. —Botosaneanu, 2002:85 [checklist]. —Keth et al., 2015:95 [♂].

—*species* B Botosaneanu and Alkins-Koo, 1993:23 [♀]. —Flint, 1996a:100 [distribution; to synonymy].

**Distribution.** Tobago, Trinidad.

***baritu*** Angrisano, 1984:4 [Type locality: Argentina, Salta, Parque Nacional Baritú; MACN; ♂; ♀]. —Angrisano, 1999:32 [checklist]. —Isa Miranda and Rueda Martín, 2014:199 [distribution].

**Distribution.** Argentina.

***bellini*** Santos and Nessimian, 2009d:760 [Type locality: Brazil, Amazonas, Rio Preto da Eva, (tributary to Rio Preto da Eva, 02°36'45.5"S, 59°43'59.1"W); INPA; ♂]. —Paprocki and França, 2014:46 [checklist].

**Distribution.** Brazil.

***bifida*** Flint, 1974c:77 [Type locality: Suriname, Lawa River, Anapaike; RNH; ♂]. —Oláh and Johanson, 2011:170 [distribution].

**Distribution.** French Guiana, Suriname.

***bifurcata*** Harris, in Flint and Sykora, 2004:34 Type locality: Dominican Republic, Peder­nales Province, Rio Mulito, 13 km N Pedernales, 18°09'N, 71°46'W, el. 230 m; CMNH; ♂; ♀]. —Pérez-Gelabert, 2008:300 [checklist]. —Keth et al., 2015:104 [♂].

**Distribution.** Dominican Republic.

***bika*** Oláh and Johanson, 2011:170 [Type locality: French Guiana, Approuaguekaw, Kaw Mt, 4°33.035'N, 52°11.661'W, el. 104 m ; NHRS; ♂].

**Distribution.** French Guiana.

***biuncifera*** Flint, 1974c:72 [Type locality: Suriname, Käyser Airstrip; RNH; ♂].

**Distribution.** Suriname.

***botka*** Oláh and Johanson, 2011:172 [Type locality: French Guiana, Approuaguekaw, Kaw Mt, 4°33.035'N, 52°11.661'W, el. 104 m; NHRS; ♂].

**Distribution.** French Guiana.

***botonia*** Harris, 1990:254 [Type locality: Venezuela, Territorio Federal Amazonas, San Carlos de Río Negro; NMNH; ♂].

**Distribution.** Venezuela.

***brevispina*** Flint, 1983a:51 [Type locality: Argentina, Pcia. Misiones, Arroyo Coatí, 13km E San José; NMNH; ♂]. —Angrisano, 1995b:511 [distribution]. —Angrisano, 1999:32 [checklist]. —Angrisano and Sganga, 2007:38 [♂; distribution].

**Distribution.** Argentina, Uruguay.

***browni*** Harris 1990:248 [Type locality: Venezuela, Territorio Federal Amazonas, San Carlos de Río Negro; NMNH; ♂]. —Santos and Nessimian, 2009d:766 [distribution]. —Paprocki and França, 2014:46 [checklist].

**Distribution.** Brazil, Venezuela.

***buenoi*** Harris and Flint, 2016:2 [Type locality: Mexico, Veracruz, Las Tuxtlas, Rio Palma above La Palma; NMNH; ♂].

**Distribution.** Mexico.

***bullata*** Flint, 1974c:71 [Type locality: Suriname, Käyser Airstrip; RNH; ♂]. —Angrisano, 1995b:511 [distribution]. —Angrisano, 1999:32 [checklist]. —Oláh and Johanson, 2011:173 [distribution].

**Distribution.** French Guiana, Suriname, Uruguay.

***cameria*** (Mosely), 1937:180 [Type locality: Mexico, Guerrero, Cocula; BMNH; ♂; in *Guerrotrichia*]. —Bueno-Soria and Flint, 1978:202 [distribution]. —Keth et al., 2015:42 [♂].

**Distribution.** Mexico.

***canixa*** (Mosely), 1937:177 [Type locality: Mexico, Chiapas, Dolores; BMNH; ♂; in *Dolotrichia*]. —Bueno-Soria and Flint, 1978:202 [distribution]. —Bueno-Soria and Barba-Álvarez, 2011:357 [checklist]. —Armitage et al., 2015a:5 [distribution]. —Armitage et al., 2015b:7 [checklist]. —Keth et al., 2015:26 [♂]. —Armitage and Cornejo, 2015:196 [checklist].

**Distribution.** Mexico, Panama, U.S.A.

***caxima*** (Mosely), 1937:179 [Type locality: Mexico, Guerrero, Cocula; BMNH; ♂; in *Guerrotrichia*]. —Bueno-Soria and Flint, 1978:202 [distribution]. —Keth et al., 2015:43 [♂].

**Distribution.** Mexico.

***cayada*** Harris, in Harris and Davenport, 1992:455 [Type locality: Venezuela, Territorio Federal Amazonas, Cerro de Neblina, basecamp near Rio Baria; NMNH; ♂].

**Distribution.** Venezuela.

***chana*** Angrisano, 1995b:513 [Type locality: Uruguay, Tacuarembo, Tbo. Chico; FHCU; ♂]. —Angrisano, 1999:32 [checklist].

**Distribution.** Uruguay.

***charrua*** Angrisano, 1984:1 [Type locality: Argentina, Entre Rios, Parque Nacional el Palmar; MACN; ♂; ♀]. —Angrisano, 1999:32 [checklist]. —Angrisano and Sganga, 2007:40 [♂; ♀; distribution].

**Distribution.** Argentina.

***chihuahua*** Harris and Flint, 2016:2 [Type locality: Mexico, Chihuahua, Rio Concheno at Highway 16 near Basaseachic; NMNH; ♂].

**Distribution.** Mexico.

***chilensis*** Flint, 1983a:53 [Type locality: Chile, Pcia. Linares, Puente Malcho, Río Longavi; NMNH; ♂]. —Angrisano, 1999:33 [checklist].

**Distribution.** Argentina, Chile.

***colmillosa*** Harris 1990:246 [Type locality: Venezuela, Territorio Federal Amazonas, Cerro de Neblina basecamp; NMNH; ♂]. —Santos and Nessimian, 2009d:766 [distribution]. —Paprocki and França, 2014:46 [checklist].

**Distribution.** Brazil, Venezuela.

***colombiensis*** Harris, 1990:257 [Type locality: Colombia, Antioquia Department, Quebrada la Jiménez, Sopetrán; NMNH; ♂]. —Flint, 1991:28 [♂; distribution]. —Muñoz-Quesada, 2000:278 [checklist].

**Distribution.** Colombia.

***connori*** Keth, in Keth, Harris and Armitage, 2015:77 [Type locality: Mexico, Nuevo Leon, Municipio de Sanchez, Arroyo San Juan on road to Laguna de Sanchez, 3.5 km west of La Cienegra, 25.4°N, 100.2833°W; PSUC; ♂].

**Distribution.** Mexico.

***contrerasi*** Harris and Flint, 2016:3 [Type locality: Mexico, Nuevo Leon, Municipio de Santiago, Rio Ramos at Los Adjuntas, 4.5 km southeast Puerto Genovevo, N25°18', W100°8'; NMNH; ♂].

**Distribution.** Mexico.

***corniculans*** Flint, 1968b:50 [Type locality: Dominica, Dleau Gommier; NMNH; ♂]. —Flint, 1968b:81 [checklist]. —Flint, 1974c:76 [♂; distribution]. —Flint and Sykora, 1993:49 [checklist]. —Harris and Tiemann, 1993:292 [♂]. —Botosaneanu, 2002:85 [checklist]. —Keth et al., 2015:27 [♂].

**Distribution.** Dominica, Suriname.

***cuernuda*** Harris, 1990:248 [Type locality: Venezuela, Territorio Federal Amazonas, Agua Blanca, Cerro de la Neblina; NMNH; ♂].

**Distribution.** Venezuela.

***delgadeza*** Harris, in Harris and Davenport, 1992:458 [Type locality: Ecuador, Pastaza, Tzapino; NMNH; ♂].

**Distribution.** Ecuador.

***didii*** Santos and Nessimian, 2009d:763 [Type locality: Brazil, Amazonas, Rio Preto da Eva (tributary to Rio Preto da Eva, 02°38'14.6"S, 59°44'09.9"W); INPA; ♂]. —Paprocki and França, 2014:47 [checklist].

**Distribution.** Brazil.

***dientera*** Harris, 1990:251 [Type locality: Venezuela, Territorio Federal Amazonas, San Carlos de Río Negro; NMNH; ♂].

**Distribution.** Venezuela.

***digitata*** (Mosely), 1937:171 [Type locality: Mexico, Guerrero, Cocula; BMNH; ♂; in *Exitrichia*]. —Bueno-Soria and Flint, 1978:202 [distribution]. —Keth et al., 2015:62 [♂].

**Distribution.** Mexico.

***dikeros*** Flint, 1983a:48 [Type locality: Argentina, Pcia. Entre Ríos, Arroyo P. Verne, 4km N Villa San José; NMNH; ♂]. —Angrisano, 1995b:511 [distribution]. —Angrisano, 1999:33 [checklist].

**Distribution.** Argentina, Uruguay.

***djalmasantosi*** Santos and Nessimian, 2009d:759 [Type locality: Brazil, Amazonas, Rio Preto da Eva (tributary to Rio Preto da Eva, 02°36'45.5"S, 59°43'59.1"W); INPA; ♂]. —Paprocki and França, 2014:47 [checklist].

**Distribution.** Brazil.

***dubitans*** (Mosely), 1939a:235 [Type locality: Brazil, Edo. Santa Catharina [sic], Nova Teutonia; BMNH; ♂; in *Dolotrichia* ?]. —Angrisano, 1999:33 [checklist]. —Paprocki et al., 2004:11 [checklist]. —Dumas et al., 2009:366 [distribution]. —Dumas and Nessimian, 2012:15 [checklist]. —Paprocki and França, 2014:47 [checklist].

**Distribution.** Brazil.

***durior*** Flint, 1983a:49 [Type locality: Brazil, Edo Santa Catarina, Nova Teutonia; NMNH; ♂]. —Angrisano, 1995b:511 [distribution]. —Angrisano, 1999:33 [checklist]. —Paprocki et al., 2004:11 [checklist]. —Paprocki and França, 2014:47 [checklist].

**Distribution.** Brazil, Uruguay.

***elongata*** Flint, 1983a:53 [Type locality: Argentina, Pcia. Salta, Cañada la Gotera, Rt. 59, km 23.5; NMNH; ♂]. —Angrisano, 1999:33 [checklist]. —Muzón et al., 2005: 57 [distribution]. —Rueda Martín, 2011:7 [♂; distribution].

**Distribution.** Argentina.

***eroga*** (Mosely), 1937:172 [Type locality: Mexico, Guerrero, Cocula; BMNH; ♂; in *Exitrichia*]. —Bueno-Soria and Flint, 1978:202 [distribution]. —Keth et al., 2015:80 [♂].

**Distribution.** Mexico.

***esmalda*** (Mosely), 1937:173 [Type locality: Mexico, Chiapas, Esmeralda; BMNH; ♂; in *Exitrichia*]. —Bueno-Soria and Flint, 1978:202 [distribution]. —Holzenthal, 1988c:62 [distribution]. —Maes, 1999:1194 [checklist]. —Bueno-Soria et al., 2005:75 [distribution]. —Chamorro-Lacayo et al., 2007:43 [checklist]. —Bueno-Soria and Barba-Álvarez, 2011:357 [checklist]. —Keth et al., 2015:72 [♂].

**Distribution.** Costa Rica, Mexico, Nicaragua.

***exicoma*** (Mosely), 1937:174 [Type locality: Mexico, Chiapas, Dolores; BMNH; ♂; in *Exitrichia*]. —Bueno-Soria and Flint, 1978:203 [distribution]. —Bueno-Soria and Barba-Álvarez, 2011:357 [checklist]. —Keth et al., 2015:81 [♂].

**Distribution.** Mexico.

***falcifera*** Flint, 1974c:75 [Type locality: Suriname, Nickerie River, Blanche Marie Falls; RNH; ♂]. —Angrisano, 1999:33 [checklist].

**Distribution.** Suriname.

***farkoska*** Oláh and Johanson, 2011:173 [Type locality: French Guiana, Approuaguekaw, Kaw Mt, 4°33.035'N, 52°11.661'W, el. 104 m; NHRS; ♂].

**Distribution.** French Guiana.

***felkurta*** Oláh and Johanson, 2011:175 [Type locality: French Guiana, Sinnamary, Petit Saut, 05°03.853'N, 053°02.814'W, el. 9 m; NHRS; ♂].

**Distribution.** French Guiana.

***feolai*** Santos and Nessimian, 2009d:766 [Type locality: Brazil, Amazonas, Rio Preto da Eva (tributary to Rio Preto da Eva, 02°38'14.6"S, 59°44'09.9"W); INPA; ♂]. —Thomson and Holzenthal, 2012:25 [redescription; distribution]. —Souza et al., 2013b:586 [distribution]. —Paprocki and França, 2014:47 [checklist].

**Distribution.** Brazil, Venezuela.

***filifera*** Flint, 1983a:46 [Type locality: Uruguay, Dpto. Lavalleja, Río Cebollati, Picada de Rodriguez; NMNH; ♂]. —Harris and Davenport, 1992:461 [redescription]. —Angrisano, 1995b:511 [distribution]. —Angrisano, 1999:33 [checklist]. —Blahnik et al., 2004:5 [distribution]. —Paprocki et al., 2004:11 [checklist]. —Souza et al., 2013b:586 [distribution]. —Paprocki and França, 2014:47 [checklist].

**Distribution.** Brazil, Uruguay.

***flowersi*** Harris, 1990:257 [Type locality: Panama, Bocas del Toro Province, Quebrada Canza at pipeline road; NMNH; ♂]. —Armitage et al., 2015b:7 [checklist]. —Armitage and Cornejo, 2015:196 [checklist].

**Distribution.** Panama.

***fogaka*** Oláh and Johanson, 2011:176 [Type locality: French Guiana, Approuaguekaw, Kaw Mt, 4°33.235'N, 52°11.988'W, el. 225 m; NHRS; ♂].

**Distribution.** French Guiana.

***garra*** Keth, 2004:170 [Type locality: Belize, Orange Walk District. New River Lagoon, dock area at Lamanai Ruins; NMNH; ♂].

**Distribution.** Belize.

***garrinchai*** Santos and Nessimian, 2009d:764 [Type locality: Brazil, Amazonas, Manaus (Igarapé Arumã, tributary to Rio Cuieiras, 02°30'55.2"S, 60°15'44.4"W); INPA; ♂]. —Paprocki and França, 2014:47 [checklist].

**Distribution.** Brazil.

***gilmari*** Santos and Nessimian, 2009d:759 [Type locality: Brazil, Amazonas, Rio Preto da Eva (tributary to Rio Urubu, 02°31'01.3"S, 59°43'13.7"W); INPA; ♂]. —Paprocki and França, 2014:47 [checklist].

**Distribution.** Brazil.

***gotera*** Flint, 1983a:51 [Type locality: Argentina, Pcia.Salta, Cañada la Gotera, Rt. 59, km 23.5; NMNH; ♂]. —Flint and Reyes, 1991:487 [♂; distribution]. —Angrisano, 1999:33 [checklist]. —Rueda Martín, 2011:8 [♂; distribution]. —Isa Miranda and Rueda Martín, 2014:199 [distribution].

**Distribution.** Argentina, Bolivia, Peru.

***hajla*** Oláh and Johanson, 2011:177 [Type locality: French Guiana, Approuaguekaw, Kaw Mt, 4°33.035'N, 52°11.661'W, el. 104 m; NHRS; ♂].

**Distribution.** French Guiana.

***heleios*** Flint, 1968a:38 [Type locality: Jamaica, St. Catherine, Bog Walk; MCZ; ♂; ♀]. —Flint, 1968b:81 [checklist]. —Botosaneanu, 2002:85 [checklist]. —Keth et al., 2015:96 [♂].

**Distribution.** Jamaica.

***hiaspa*** (Mosely), 1937:181 [Type locality: Mexico, Chiapas, Dolores; BMNH; ♂; in *Lorotrichia*]. —Ross, 1944:154 [to synonymy]. —Bueno-Soria and Flint, 1978:203 [distribution]. —Chamorro-Lacayo et al., 2007:43 [checklist]. —Bueno-Soria and Barba-Álvarez, 2011:357 [checklist]. —Oláh and Johanson, 2011:179 [checklist]. —Keth et al., 2015:67 [♂].

**Distribution.** Mexico, Nicaragua.

***horgoska*** Oláh and Johanson, 2011:179 [Type locality: French Guiana, Maripasoula, Lawa River, Gzaan Dayé, 4°01.130'N, 54°19.015'W, el. 74 m; NHRS; ♂].

**Distribution.** French Guiana.

***interrupta*** Flint, 1974c:77 [Type locality: Suriname, Lucie River camp, Wilhelmina Mountains; RNH; ♂].

**Distribution.** Suriname.

***iridescens*** Flint, 1964a:51 [Type locality: Puerto Rico, Maricao, at fish hatchery; NMNH; ♂; ♀; larva; case]. —Flint, 1968a:37 [♂; ♀; larva; distribution]. —Flint, 1968b:48 [♂; ♀; larva; distribution]. —Botosaneanu, 1979:51 [distribution]. —Malicky, 1983:264 [distribution]. —Kumanski, 1987:23 [distribution]. —Botosaneanu, 1989a:99 [distribution]. —Botosaneanu, 1991a:128 [distribution]. —Flint and Sykora, 1993:49 [checklist]. —Botosaneanu, 1994a:43 [distribution]. —Botosaneanu, 1995:32 [distribution]. —Flint, 1996c:16 [checklist]. —Botosaneanu and Hyslop, 1998:18 [distribution]. —Flint and Pérez-Gelabert, 1999:40 [checklist]. —Botosaneanu, 2000:256 [distribution]. —Botosaneanu, 2002:85 [checklist]. —Flint and Sykora, 2004:34 [distribution]. —Botosaneanu and Thomas, 2005:42 [distribution]. —Naranjo López and González Lazo, 2005:149 [checklist]. —Pérez-Gelabert, 2008:300 [checklist]. —Keth et al., 2015:97 [♂].

**Distribution.** Cuba, Dominica, Dominican Republic, Guadeloupe, Haiti, Jamaica, Martinique, Puerto Rico, St. Lucia.

***ismetla*** Oláh and Johanson, 2011:180 [Type locality: French Guiana, Approuaguekaw, Kaw Mt, 4°33.257'N, 52°11.920'W, el. 216 m; NHRS; ♂].

**Distribution.** French Guiana.

***jarochita*** Bueno-Soria, 1999:114 [Type locality: Mexico, Veracruz, Estación de Biología Los Tuxtlas, UNAM, Arroyo del Zoológico; IBUNAM; ♂]. —Oláh and Johanson, 2011:181 [distribution]. —Keth et al., 2015:28 [♂].

**Distribution.** Mexico.

***juntada*** Harris, in Harris and Davenport, 1992:465 [Type locality: Peru, Loreto, tributary to Rio Yanomono at Explorama Lodge; NMNH; ♂].

**Distribution.** Peru, Venezuela.

***kampa*** Oláh and Johanson, 2011:182 [Type locality: Peru, San Martin Prov., Rio Mayo, 11 km (rd.) E Mayobamba, 6°04.989'S, 76°53.065'W; NHRS; ♂].

**Distribution.** Peru.

***kampoka*** Oláh and Johanson, 2011:183 [Type locality: Peru, San Martin Prov., Rio Mayo, 37 km (rd.) W Moyobamba, near Olmos-Tarapoto rd., 6°00.278'S, 77°15.437'W; NHRS; ♂].

**Distribution.** Peru.

***kehelia*** Oláh and Johanson, 2011:184 [Type locality: Peru, San Martin Prov., La Catarata de Ahuashiyascu, 6°27.544'S, 76°18.192'W; NHRS; ♂].

**Distribution.** Peru.

***ketaguka*** Oláh and Johanson, 2011:187 [Type locality: French Guiana, Approuaguekaw, Kaw Mt, 4°33.035'N, 52°11.661'W, el. 104 m; NHRS; ♂].

**Distribution.** French Guiana.

***kurta*** Oláh and Johanson, 2011:187 [Type locality: Peru, San Martin Prov., Rio Huallaga tributary, small river passing Chazuta, 6°34.665'S, 76°08.209'W; NHRS; ♂].

**Distribution.** Peru.

***kurtika*** Oláh and Johanson, 2011:188 [Type locality: French Guiana, Maripasoula: Lawa River: Gzaan Dayé, 4°01.130'N, 54°19.015'W, el. 74 m; NHRS; ♂].

**Distribution.** French Guiana.

***kurtitva*** Oláh and Johanson, 2011:190 [Type locality: Peru, San Martin Prov., Rio Huallaga, at Pumarihri Huallaga Lodge, between Juan Guerra and Chazuta, 14 km (rd.) W Chazuta, 6°36.643'S, 76°12.555'W; NHRS; ♂].

**Distribution.** Peru.

***labios*** Keth, in Keth, Harris and Armitage, 2015:98 [Type locality: Mexico, Sonora, Rio Arros at Arroyo El Pavo; NMNH; ♂].

**Distribution.** Mexico.

***lacertina*** Botosaneanu, 1994a:43 [Type locality: Guadeloupe, River Lézarde, Saut de la Lézarde; ZMUA; ♂]. —Botosaneanu, 2000:256, 259 [♂; ♀; distribution]. —Botosaneanu, 2002:85 [checklist]. —Botosaneanu and Thomas, 2005:42 [distribution]. —Keth et al., 2015:68 [♂].

**Distribution.** Guadeloupe, Martinique.

***lefela*** Oláh and Johanson, 2011:191 [Type locality: French Guiana, Approuaguekaw, Kaw Mt, 4°33.035'N, 52°11.661'W, el. 104 m; NHRS; ♂].

**Distribution.** French Guiana.

***leonensis*** Keth, in Keth, Harris and Armitage, 2015:85 [Type locality: Mexico, Nuevo Leon, Municipio de Santiago, Arroyo San Juan on road to Laguna de Sanchez, 3.5 km west of La Cienegra, 25°24'N, 100°17'W; PSUC; ♂].

**Distribution.** Mexico.

***lobata*** Flint, 1974c:79 [Type locality: Suriname, Lucie River camp, Wilhelmina Mountains; RNH; ♂].

**Distribution.** Suriname.

***longissima*** Flint, 1983a:49 [Type locality: Brazil, Edo. Santa Catarina, Nova Teutonia; NMNH; ♂]. —Angrisano, 1999:33 [checklist]. —Paprocki et al., 2004:11 [checklist]. —Paprocki and França, 2014:48 [checklist].

**Distribution.** Brazil.

***lucrecia*** Angrisano, 1995b:513 [Type locality: Uruguay, Artigas, Potrero Sucio, Ao. Tres Cruces; FHCU; ♂]. —Angrisano, 1999:33 [checklist].

**Distribution.** Uruguay.

***malickyi*** Harris, in Harris and Tiemann, 1993:288 [Type locality: Panama, Barro Colorado Island, Lutz; NMNH; ♂]. —Armitage et al., 2015b:7 [checklist]. —Armitage and Cornejo, 2015:196 [checklist].

**Distribution.** Panama.

***manopla*** Keth, in Keth, Harris and Armitage, 2015:46 [Type locality: Mexico, Sonora, Cajon Bonito, Losojas Ranch, 31.27854°N, 109.00196°W; NMNH; ♂].

**Distribution.** Mexico.

***margaritena*** Botosaneanu, in Botosaneanu and Viloria, 2002:106 [Type locality: Venezuela, Isla de Margarita, Rio San Juan at fuentidueno; ZMUA; ♂; ♀]. —Botosaneanu, 2002:85 [checklist].

**Distribution.** Venezuela.

***maria*** Bueno-Soria and Hamilton, 1986:302 [Type locality: Mexico, Oaxaca, 7km NE Huautla de Jimenez; NMNH; ♂]. —Keth, 2004:177 [♂]. —Keth et al., 2015:31 [♂].

**Distribution.** Mexico.

***mathisi*** Keth, 2004:170 [Type locality: Belize, Orange Walk District. New River Lagoon, dock area at Lamanai Ruins; NMNH; ♂].

**Distribution.** Belize.

***maya*** Harris and Flint, 2016:3 [Type locality: Belize, Stann Creek District, Cockscomb Wildlife Preserve, Cockscomb A, B4, Maya Mountains, el.200 m, N16-80, W88-55; NMNH; ♂].

**Distribution.** Belize.

***mobilensis*** Harris, 1985:252 [Type locality: U.S.A., Alabama, Mobile County, Mobile River at Mt. Vernon, T2N, R1W; NMNH; ♂.] —Harris and Flint, 2016:7 [distribution].

**Distribution.** Mexico, U.S.A.

***napoensis*** Harris, in Harris and Davenport, 1992:461 [Type locality: Ecuador, Napo, 7km N Lago Agrio; NMNH; ♂].

**Distribution.** Ecuador.

***negroensis*** Harris, 1990:254 [Type locality: Venezuela, Territorio Federal Amazonas, San Carlos de Río Negro; NMNH; ♂].

**Distribution.** Venezuela.

***nesiotes*** Flint and Sykora, 1993:55 [Type locality: Grenada, Parish St. Andrews, Balthazar Estate; FSCA; ♂]. —Botosaneanu and Alkins-Koo, 1993:20 [♂; distribution]. —Flint, 1996a:100 [distribution]. —Botosaneanu, 2002:85 [checklist]. —Keth et al., 2015:50 [♂].

—*intortigona* Botosaneanu and Sakal, 1992:202 [*nomen nudum*; distribution; ecology]. —Flint et al., 1999b:104 [to synonymy; is *nesiotes*].

**Distribution.** Grenada, Trinidad.

***niltonsantosi*** Santos and Nessimian, 2009d:762 [Type locality: Brazil, Amazonas, Manaus (tributary to Igarapé da Cachoeira, 02°41'45.4"S, 60°17'42.7"W); INPA; ♂]. —Paprocki and França, 2014:48 [checklist].

**Distribution.** Brazil.

***noteuna*** (Mosely), 1939a:232 [Type locality: Brazil, Edo. Santa Catharina [sic], Nova Teutonia; BMNH; ♂; in *Exitrichia*]. —Angrisano, 1995b:511 [distribution]. —Angrisano, 1999:33 [checklist]. —Paprocki et al., 2004:11 [checklist]. —Paprocki and França, 2014:48 [checklist].

**Distribution.** Brazil, Uruguay.

***novara*** (Mosely), 1939a:232 [Type locality: Brazil, Edo. Santa Catharina [sic], Nova Teutonia; BMNH; ♂; in *Exitrichia*]. —Angrisano, 1995b:511 [distribution]. —Angrisano, 1999:33 [checklist]. —Paprocki et al., 2004:11 [checklist]. —Angrisano and Sganga, 2007:40 [♂; distribution]. —Manzo et al., 2014:166 [distribution]. —Paprocki and França, 2014:48 [checklist].

**Distribution.** Argentina, Brazil, Uruguay.

***oldalia*** Oláh and Johanson, 2011:192 [Type locality: Peru, San Martin Prov., Rio Huallaga, at Pumarihri Huallaga Lodge, between Juan Guerra and Chazuta, 14 km (rd.) W Chazuta, 6°36.643'S, 76°12.555'W; NHRS; ♂].

**Distribution.** Peru.

***olorina*** (Mosely), 1937:175 [Type locality: Mexico, Guerrero, Cocula; BMNH; ♂; in *Exitrichia*]. —Bueno-Soria and Flint, 1978:203 [distribution]. —Blinn and Ruiter, 2006:333 [biology; distribution]. —Blinn and Ruiter, 2009a:305 [biology]. —Keth et al., 2015:73 [♂].

**Distribution.** Mexico, U.S.A.

***orejona*** Harris and Davenport, 1999:26 [Type locality: Peru, Loreto, edge of Rio Sucusari backwater, adjoining Explornapo Camp; NMNH; ♂].

**Distribution.** Peru.

***orlandoi*** Santos and Nessimian, 2009d:761 [Type locality: Brazil, Amazonas, Manaus (tributary to Rio Branquinho, 02°31'24.6"S, 60°20'05.3"W); INPA; ♂]. —Paprocki and França, 2014:48 [checklist].

**Distribution.** Brazil.

***ovona*** (Mosely), 1939a:233 [Type locality: Brazil, Edo. Santa Catharina [sic], Nova Teutonia; BMNH; ♂; in *Exitrichia*]. —Angrisano, 1999:33 [checklist]. —Paprocki et al., 2004:11 [checklist]. —Paprocki and França, 2014:48 [checklist].

**Distribution.** Brazil.

***oxima*** (Mosely), 1937:176 [Type locality: Mexico, Guerrero, Cocula; BMNH; ♂; in *Exitrichia*]. —Bueno-Soria and Flint, 1978:203 [distribution]. —Keth et al., 2015:89 [♂].

**Distribution.** Mexico.

***palitla*** Harris and Flint, 2016:4 [Type locality: Mexico, San Luis Potosi, Palitla; NMNH; ♂].

**Distribution.** Mexico.

***palma*** Flint, 1982a:45 [Type locality: Argentina, Pcia. Buenos Aires, Río Parana de las Palmas; NMNH; ♂; ♀]. —Flint, 1982c:38 [distribution]. —Angrisano, 1995b:511 [distribution]. —Angrisano, 1999:33 [checklist]. —Angrisano and Sganga, 2007:40 [♂; distribution].

**Distribution.** Argentina, Paraguay, Uruguay.

***pamelae*** Harris and Armitage, 2015:5 [Type locality: Panama, Chiriquí Province, Cuenca 108, tributary of Quebrada Grande, at waterfall, Boquete, Valle Escondido, 8.78291°N, 82.44579°W, el. 1253 m; MIUP; ♂]. —Armitage et al., 2015b:7 [checklist]. —Armitage and Cornejo, 2015:196 [checklist].

**Distribution.** Panama.

***parabullata*** Harris and Armitage, 2015:6 [Type locality: Panama, Panama Canal Zone, Cuenca 115, Isla Abogada, 9.19903°N, 79.85980°W; NMNH; ♂]. —Armitage et al., 2015b:7 [checklist]. —Armitage and Cornejo, 2015:196 [checklist].

**Distribution.** Panama.

***parany*** Oláh and Johanson, 2011:193 [Type locality: Peru, Amazonas Prov., Rio Utcabamba, Bajra Grande, at Rio Hotel, 5°45.824'S, 78°25.414'W; NHRS; ♂].

**Distribution.** Peru.

***pelei*** Santos and Nessimian, 2009d:766 [Type locality: Brazil, Amazonas, Rio Preto da Eva (tributary to Rio Preto da Eva, 02°32'09.4"S, 59°49'59.3"W); INPA; ♂]. —Paprocki and França, 2014:48 [checklist].

**Distribution.** Brazil.

***pequenita*** Botosaneanu, 1977:277 [Type locality: Cuba, Oriente, Baracoa, Rio Sabanilla; NMNH; ♂]. —Botosaneanu, 1979:51 [distribution]. —Kumanski, 1987:23 [distribution]. —Botosaneanu, 1990a:46 [♂; ♀; distribution]. —Botosaneanu, 1991a:128 [distrubution]. —Botosaneanu and Sakal, 1992:202 [distribution; ecology]. —Botosaneanu and Alkins-Koo, 1993:18 [distribution]. —Flint and Sykora, 1993:49 [checklist]. —Botosaneanu, 1994b:458 [larva]. —Flint, 1996a:101 [distribution]. —Flint, 1996c:16 [checklist]. —Botosaneanu and Hyslop, 1998:18 [distribution]. —Flint and Pérez-Gelabert, 1999:40 [checklist]. —Botosaneanu, 2002:85 [checklist]. —Flint and Sykora, 2004:36 [distribution]. —Naranjo López and González Lazo, 2005:149 [checklist]. —Pérez-Gelabert, 2008:300 [checklist]. —Keth et al., 2015:51 [♂].

—*Neotrichia* sp. 1 Kumanski, 1987:23 [♀]. —Flint and Sykora, 2004:36 [to synonymy].

**Distribution.** Barbados, Dominican Republic, Cuba, Haiti, Jamaica, Trinidad.

***picada*** Flint, 1983a:53 [Type locality: Uruguay, Dpto. Lavalleja, Rio Cebollati, Picada de Rodriguez; NMNH; ♂]. —Angrisano, 1995b:511 [distribution]. —Angrisano, 1999:33 [checklist].

**Distribution.** Uruguay.

***pinarenia*** Botosaneanu, 1980:113 [Type locality: Cuba, Prov. Pinar del Rio, Arroyo del Pinar de Viñales; ZMUA; ♂]. —Botosaneanu, 1979:51 [*nomen nudum* (name included in checklist); distribution]. —Flint, 1996c:16 [checklist]. —Botosaneanu, 2002:86 [checklist]. —Naranjo López and González Lazo, 2005:149 [checklist]. —Keth et al., 2015:32 [♂].

**Distribution.** Cuba.

***proboscidea*** Flint, 1974c:73 [Type locality: Suriname, Nickerie River, Lombok falls; RNH; ♂].

**Distribution.** Suriname.

***pulgara*** Keth, 2004:174 [Type locality: Belize, Orange Walk District. New River Lagoon, dock area at Lamanai Ruins; NMNH; ♂].

**Distribution.** Belize.

***riparia*** Flint and Reyes, 1991:486 [Type locality: Peru, Dept. La Libertad, Prov. Trujillo, Dist. Simbal, Río Lucumar, Simbal; NMNH; ♂].

**Distribution.** Peru.

***rotundata*** Flint, 1974c:76 [Type locality: Suriname, Käyser Airstrip; RNH; ♂]; —Flint, 1992d:69 [distribution; as near *rotunda*]. —Angrisano, 1999:33 [checklist]. —Paprocki et al., 2004:11 [checklist]. —Paprocki and França, 2014:48 [checklist].

**Distribution.** Brazil, Suriname.

***ruiteri*** Keth, in Keth, Harris and Armitage, 2015:54 [Type locality: Mexico, Sonora, Canon Alacran; NMNH; ♂].

**Distribution.** Mexico.

***sala*** Angrisano, 1984:1 [Type locality: Argentina, Salta, Parque Nacional el Rey, Rio La Sala; MACN; ♂]. —Angrisano, 1999:33 [checklist].

**Distribution.** Argentina.

***salada*** Flint, 1982a:43 [Type locality: Argentina, Pcia. Buenos Aires, Rio Salado, Rt. 3, S San Miguel del Monte; NMNH; ♂; ♀]. —Flint, 1982c:39 [distribution]. —Angrisano, 1995b:513 [distribution]. —Angrisano, 1999:33 [checklist].

**Distribution.** Argentina, Paraguay, Uruguay.

***sicilicula*** Flint, 1983a:51 [Type locality: Brazil, Edo. Santa Catarina, Nova Teutonia; NMNH; ♂]. —Angrisano, 1995b:513 [distribution]. —Angrisano, 1999:33 [checklist]. —Paprocki et al., 2004:11 [checklist]. —Paprocki and França, 2014:49 [checklist].

**Distribution.** Brazil, Uruguay.

***sokaga*** Oláh and Johanson, 2011:194 [Type locality: Peru, San Martin Prov., creek crossing rd. Juan Guerra-Chazuta, 14 km (rd.) E Colombia Bridge, 6°35.594'S, 76°13.172'W; NHRS; ♂].

**Distribution.** Peru.

***soleaferrea*** , Botosaneanu, in Botosaneanu and Hyslop, 1998:18 [Type locality: Jamaica, St. Elizabeth, Black River in its upper course at Windsor; ZMUA; ♂; ♀]. —Botosaneanu, 2002:86 [checklist]. —Keth et al., 2015:100 [♂].

**Distribution.** Jamaica.

***sucusaria*** Harris and Davenport, 1992:458 [Type locality: Peru, Loreto, Rio Sucusari just up stream from Explornapo Camp; NMNH; ♂].

**Distribution.** Peru.

***tauricornis*** Malicky, 1980:220 [Type locality: Guadeloupe, Bras de David beim Forsthaus, Zufluss des Flusses Goyaves; Coll. Malicky; ♂]. —Malicky, 1983:264 [checklist]. —Botosaneanu, 1989a:99 [♀; distribution]. —Flint, 1991:28 [♂; distribution]. —Botosaneanu and Sakal, 1992:202 [distribution; ecology]. —Flint and Sykora, 1993:56 [distribution]. —Harris and Tiemann, 1993:288 [♂; distribution]. —Botosaneanu and Alkins-Koo, 1993:21 [distribution]. —Botosaneanu, 1994a:43 [distribution]. —Flint, 1996a:101 [distribution]. —Muñoz-Quesada, 2000:278 [checklist]. —Botosaneanu, 2000:256 [distribution]. —Botosaneanu, 2002:86 [checklist]. —Botosaneanu and Thomas, 2005:44 [distribution]. —Armitage et al., 2015b:7 [checklist]. —Keth et al., 2015:35 [♂]. —Armitage and Cornejo, 2015:196 [checklist].

**Distribution.** Colombia, Grenada, Guadeloupe, Martinique, Panama, St. Lucia, Tobago, Trinidad.

***tertia*** (Mosely), 1939a:235 [Type locality: Brazil, Edo. Santa Catharina [sic], Nova Teutonia; BMNH; ♂; in *Exitrichia*]. —Angrisano, 1995b:513 [distribution]. —Angrisano, 1999:33 [checklist]. —Paprocki et al., 2004:11 [checklist]. —Paprocki and França, 2014:49 [checklist].

**Distribution.** Brazil, Uruguay.

***teutonia*** Flint, 1983a:49 [Type locality: Brazil, Edo Santa Catarina, Nova Teutonia: NMNH; ♂]. —Angrisano, 1999:33 [checklist]. —Paprocki et al., 2004:11 [checklist]. —Paprocki and França, 2014:49 [checklist].

**Distribution.** Brazil.

***tirabuzona*** Harris and Davenport, 1999:27 [Type locality: Peru, Loreto, edge of Rio Sucusari backwater, adjoining Explornapo Camp, NMNH; ♂].

**Distribution.** Peru.

***tompa*** Oláh and Johanson, 2011:196 [Type locality: French Guiana, Approuaguekaw, Kaw Mt, 4°33.035'N, 52°11.661'W, el. 104 m; NHRS; ♂].

**Distribution.** French Guiana.

***tubulifera*** Flint, 1980b:141 [Type locality: Argentina, Pcia. Entre Rios, Salto Grande, Rio Uruguay; NMNH; ♂]. —Flint, 1982c:40 [distribution]. —Angrisano, 1995b:513 [distribution]. —Angrisano, 1999:33 [checklist]. —Angrisano and Sganga, 2007:40 [♂; distribution].

**Distribution.** Argentina, Uruguay.

***tuxtla*** Bueno-Soria, 1999:113 [Type locality: Mexico, Veracruz, Estación de Biología Los Tuxtlas, UNAM, Arroyo del Zoológico; IBUNAM; ♂]. —Keth et al., 2015:69 [♂].

**Distribution.** Mexico.

***unamas*** Botosaneanu, in Botosaneanu and Alkins-Koo, 1993:22 [Type locality: Tobago, Argyll River below Argyll waterfall; ZMUA; ♂]. —Botosaneanu and Sakal, 1992:202 [*nomen nudum* (name included in checklist); distribution; ecology]. —Flint, 1996a:101 [distribution]. —Botosaneanu, 2002:86 [checklist]. —Keth et al., 2015:36 [♂].

—*species* A Botosaneanu and Alkins-Koo, 1993:23 [♀]. —Flint, 1996a:101 [distribution; to synonymy].

**Distribution.** Tobago, Trinidad, Venezuela.

***unispina*** Flint, 1974c:72 [Type locality: Suriname, Lucie River camp, Wilhelmina Mountains; RNH; ♂]. —Flint, 1996b:402 [distribution].

**Distribution.** Peru, Suriname.

***vavai*** Santos and Nessimian, 2009d:763 [Type locality: Brazil, Amazonas, Manaus (tributary to Rio Branquinho, 02°31'24.6"S, 60°20'05.3"W); INPA; ♂]. —Paprocki and França, 2014:49 [checklist].

**Distribution.** Brazil.

***vekonyka*** Oláh and Johanson, 2011:197 [Type locality: French Guiana, Approuaguekaw, Kaw Mt, 4°33.035'N, 52°11.661'W, el. 104 m; NHRS; ♂].

**Distribution.** French Guiana.

***vibrans*** Ross, 1938b:119 [Type locality: U.S.A., Illinois, Oakwood, Middle Fork River; INHS; ♂]. —Bueno-Soria and Flint, 1978:203 [distribution]. —Bowles et al., 2007:21 [distribution; biology]. —Keth et al., 2015:101 [♂].

—*ranea* (Denning), 1947c:20 [Type locality: U.S.A., Florida, Miami; CAS; ♂]. —Ross, 1948a:205 [to synonymy].

**Distribution.** Mexico, U.S.A.

***villa*** Oláh and Johanson, 2011:199 [Type locality: French Guiana, Approuaguekaw, Kaw Mt, 4°33.035'N, 52°11.661'W, el. 104 m; NHRS; ♂].

**Distribution.** French Guiana.

***vissa*** Oláh and Johanson, 2011:200 [Type locality: Peru, San Martin Prov., La Catarata de Ahuashiyascu, 6°27.544'S, 76°18.192'W; NHRS; ♂].

**Distribution.** Peru.

***vonza*** Oláh and Johanson, 2011:201 [Type locality: French Guiana, Maripasoula, Maroni River, Damason campo, Village, 4°35.112'N, 54°24.799'W, 38 m; NHRS; ♂].

**Distribution.** French Guiana.

***xicana*** (Mosely), 1937:178 [Type locality: Mexico, Chiapas, Dolores; BMNH; ♂; in *Dolotrichia*]. —Bueno-Soria and Flint, 1978:203 [distribution]. —Maes, 1999:1194 [checklist]. —Bueno-Soria et al., 2005:75 [distribution]. —Bueno-Soria et al., 2007:33 [distribution]. —Chamorro-Lacayo et al., 2007:43 [checklist]. —Bueno-Soria and Barba-Álvarez, 2011:357 [checklist]. —Oláh and Johanson, 2011:202 [distribution]. —Keth et al., 2015:37 [male].

**Distribution.** Mexico, Nicaragua.

***yagua*** Harris and Davenport, 1992:463 [Type locality: Peru, Loreto, Rio Sucusari just upstream from Explornapo Camp; NMNH; ♂].

**Distribution.** Peru.

***yanomonoa*** Harris and Davenport, 1992:454 [Type locality: Peru, Loreto, small tributary to Rio Yanomono at Explorama Lodge; NMNH; ♂].

**Distribution.** Peru.

***yavesia*** Bueno-Soria, 2010:29 [Type locality: Mexico, Oaxaca, Santa María de Yavesia, 17°14'04.76”6N, 96°25'35.06"W, el. 2058 m; CNIN; ♂]. —Keth et al., 2015:102 [male].

**Distribution.** Mexico.

***zagalloi*** Santos and Nessimian, 2009d:765 [Type locality: Brazil, Amazonas, Rio Preto da Eva (Igarapé Jangada, tributary to Rio Urubu, 02°26'32.5"S, 59°32'46.2"W); INPA; ♂]. —Paprocki and França, 2014:49 [checklist].

**Distribution.** Brazil.

***zitoi*** Santos and Nessimian, 2009d:762 [Type locality: Brazil, Amazonas, Manaus (Igarapé Arumã, tributary to Rio Cuieiras, 02°30'55.2"S, 60°15'44.4"W); INPA; ♂]. —Paprocki and França, 2014:49 [checklist].

**Distribution.** Brazil.

#### Genus *Nothotrichia* Flint [5]

*Nothotrichia* Flint, 1967a:56 [Type species: *Nothotrichia illiesi* Flint, 1967a, original designation]. —Harris and Armitage, 1997:123 [redescription; placement]. —Parys and Harris, 2013:590 [larva; taxonomic remarks].

Marshall (1979) was unable to place the genus in a tribe and left it as *incertae sedis* within Hydroptilinae, as then defined, but Harris and Armitage (1997) and Parys and Harris (2013) placed it in the Ochrotrichiinae. It was perhaps erroneously included in the Orthotrichiinae in the *Trichoptera World Checklist*, and this error was transcribed by Holzenthal et al., (2007b). The genus was thought to be amphitropical in distribution, occurring in the United States (California, one species) and Chile (two species), but three additional species have now been discovered in Costa Rica, Panama, and Brazil.

The larva of *Nothotrichia shasta* from California was described by Parys and Harris (2013). Larvae of this species were collected from filamantous green algae in high elevation, fast-flowing streams; gut contents were composed of strands of algae.

***cautinensis*** Flint 1983a:40 [Type locality: Chile, Pcia. Cautín, Río Cautín, Cajón; NMNH; ♂]. —Harris and Armitage, 1997:125 [♂; ♀; redescription]. —Angrisano, 1999:33 [checklist]. —Oláh and Johanson, 2011:213 [distribution].

**Distribution.** Chile.

***illiesi*** Flint, 1967a:56 [Type locality: Chile, Prov. Cautín, brook on Lago Villarica; NMNH; ♂]. —Flint, 1974e:87 [checklist]. —Harris and Armitage, 1997:124 [♂; ♀; redescription]. —Angrisano, 1999:33 [checklist].

**Distribution.** Chile.

***munozi*** Holzenthal and Harris, 2002:106 [Type locality: Costa Rica, Guanacaste, Area de Conservación Guanacaste, Parque Nacional Guanacaste, Estación Maritza, Rio Tempisquito, 10.958°N, 85.497°W, el. 550 m; UMSP; ♂].

**Distribution.** Costa Rica.

***panama*** Harris and Armitage, 2015:11 [Type locality: Panama, Chiriquí Province, Cuenca 108, Tributary of Quebrada Grande, at waterfall, Boquete, Valle Escondido, 8.78291°N, 82.44579°W, el. 1253 m; MIUP; ♂]. —Armitage et al., 2015b:7 [checklist]. —Armitage and Cornejo, 2015:196 [checklist].

**Distribution.** Panama.

***tupi*** Holzenthal and Harris, 2002:109 [Type locality: Brazil, Minas Gerais, Parque Estadual Itacolomi, Rio Belchior, 20°25.041'S, 43°25.633'W, el. 725 m; MZUSP; ♂]. —Paprocki et al., 2004:11 [checklist]. —Paprocki and França, 2014:49 [checklist].

**Distribution.** Brazil.

#### Genus *Ochrotrichia* Mosely [159 + †5]

*Polytrichia* Sibley, 1926:102 [Type species: *Ithytrichia confusa* Morton 1905, by monotypy; preoccupied]. —Ross, 1944:125, 126 [preoccupied].

*Ochrotrichia* Mosely, 1934b:162 [Type species: *Ochrotrichia insularis* Mosely, 1934b, original designation; synonymized with *Polytrichia* by Mosely (1937). —Ross, 1944: 125, 126 [recognized *Polytrichia* as preoccupied, and resurrected *Ochrotrichia*]. —Bueno-Soria, 2009:60 [revision].

This nominotypical genus of the Ochrotrichiinae is found across North, Central and South America, and the West Indies. Many new species have been described during the last decade or so, especially by J. Bueno-Soria and colleagues. The genus is the largest of the Neotropical Hydroptilidae, with 164 species, including five species known from Dominican amber (Wells and Wichard 1989).

Larvae have been associated for only a very few species, given the size of the genus (Roldán Pérez 1988, Ross 1944, Wiggins 1996). They frequent flowing water of all types, from larger rivers down to small streams, springs and temporary streams, and also madicolous habitats where they feed, at least in part, on diatoms scraped from the substrate (Wiggins 1996). Luhman et al. (1999) reported the pupae of a Costa Rican species parasitized by a ceraphronid wasp.

***abrelata*** Harris and Armitage, 2015:11 [Type locality: Chiriquí Province, Cuenca 108, tributary of Quebrada Grande, at waterfall, Boquete, Valle Escondido, 8.78291°N, 82.44579°W, el. 1253 m; MIUP; ♂]. —Armitage et al., 2015b:7 [checklist]. —Armitage and Cornejo, 2015:196 [checklist].

**Distribution.** Panama.

***affinis*** Bueno-Soria and Holzenthal, 2004:251 [Type locality: Mexico, Tabasco, Municipio de Huimanguillo, Ejido Villa de Guadalupe, 1a sección Cascada Cerro de Las Flores, 17°21'39"N, 93°37'29"W, el. 540 m; CNIN; ♂]. —Bueno-Soria, 2009:133 [♂].

**Distribution.** Mexico.

***alargada*** Bueno-Soria and Holzenthal, 2004:246 [Type locality: Mexico, Guerrero, Municipio de Taxco: Teusisapan, Rio Temazcalapa, 12 km. NW Ahuehuepan, Rta 51, 18°25.56'N, 99°42.5'W, el. 1052 m; CNIN; ♂]. —Bueno-Soria, 2009:112 [♂].

**Distribution.** Mexico.

***aldama*** (Mosely) 1937:185 [Type locality: Mexico, Chiapas, Esmeralda; BMNH; ♂; in *Polytrichia*]. —Bueno-Soria and Flint, 1978:203 [distribution]. —Wells and Wichard, 1989:46 [in Dominican amber]. —Flint and Pérez-Gelabert, 1999:40 [checklist]. —Botosaneanu, 2002:86 [checklist]. —Bueno-Soria and Holzenthal, 2004:246 [distribution]. —Wichard, 2007a:48 [checklist; in amber]. —Bueno-Soria and Holzenthal, 2008:48 [distribution]. —Eskov et al., 2008: [checklist; in amber]. —Pérez-Gelabert, 2008:300 [checklist]. —Bueno-Soria, 2009:110 [♂; distribution]. —Bueno-Soria and Barba-Álvarez, 2011:357 [checklist].

**Distribution.** Costa Rica, Dominican Republic (in amber), Mexico, Panama.

† ***aliceae*** Wichard, 2000:242 [Type locality: Dominican Republic; NMNH; in amber]. —Wichard, 2007a:48 [checklist]. —Pérez-Gelabert, 2008:300 [checklist].

**Distribution.** Dominican Republic.

***amorfa*** Bueno-Soria and Holzenthal, 2004:252 [Type locality: Mexico, Tabasco, Municipio de Huimanguillo, Arroyo las Flores, Villa de Guadalupe, 2a sección, Los Chimalapas km 5 Ruta Malpasito-Carlos A. Madrazo, 17°22'05"N, 93°36'25", el. 540 m; CNIN; ♂]. —Bueno-Soria, 2009:133 [♂].

**Distribution.** Mexico.

***angularis*** Bueno-Soria, 2009:131 [Type locality: Mexico, Morelos, Huautla, Reserva de la Biosfera de Huautla, 18°20'10"N, 98°51'20"W, el. 900 m; CNIN; ♂].

**Distribution.** Mexico.

***anomala*** Bueno-Soria and Santiago-Fragoso, 1997:365 [Type locality: Panama, Barro Colorado Island, Snyder-Molino Trail, Marker 3; NMNH; ♂]. —Bueno-Soria, 2009:68 [♂]. —Armitage et al., 2015b:7 [checklist]. —Armitage and Cornejo, 2015:196 [checklist].

**Distribution.** Panama.

***argentea*** Flint and Blickle, in Denning and Blickle, 1972:150 [Type locality: United States, N[ew] Mex[ico], Near Silver City, Cherry Creek Rec. Area; NMNH; ♂]. —Bueno-Soria, 2009:147 [♂]. —Ruiter and Harris, 2015:330 [distribution].

**Distribution.** Mexico, U.S.A.

***arranca*** (Mosely) 1937:185 [Type locality: Mexico, Chiapas, Barranca Honda; BMNH; ♂; in *Polytrichia*]. —Flint, 1972a:7 [redescription; ♂]. —Bueno-Soria and Flint, 1978:203 [distribution]. —Rojas-Ascencio, et al., 2002:377 [distribution]. —Bueno-Soria et al., 2005:75 [distribution]. —Bueno-Soria and Holzenthal, 2008:48 [distribution]. —Bueno-Soria, 2009:122 [♂]. —Bueno-Soria and Barba-Álvarez, 2011:357 [checklist].

**Distribution.** Costa Rica, Mexico.

***arriba*** Bueno-Soria and Santiago-Fragoso, 1997:361 [Type locality: Panama, Chiriqui, Guadalupe Arriba, 8°52'26"N, 82°33'13"W; NMNH; ♂]. —Bueno-Soria, 2009:114 [♂]. —Armitage et al., 2015b:7 [checklist]. —Armitage and Cornejo, 2015:196 [checklist].

**Distribution.** Panama.

***assita*** Bueno-Soria and Holzenthal, 2004:251 [Type locality: Panama, Chiriqui, Fortuna Dam site near Hornitos, 8°55'N, 82°16'W, el. 1050 m; NMNH; ♂]. —Bueno-Soria and Holzenthal, 2008:48 [distribution]. —Bueno-Soria, 2009:114 [♂]. —Armitage et al., 2015b:7 [checklist]. —Armitage and Cornejo, 2015:196 [checklist].

**Distribution.** Costa Rica, Panama.

***atezcae*** Bueno-Soria and Santiago-Fragoso, 1981:384 [Type locality: Mexico, Hidalgo, Laguna de Atezca, 3 km de Molango; IBUNAM; ♂]. —Bueno-Soria, 2009:132 [♂].

**Distribution.** Mexico.

***attenuata*** Flint, 1972a:11 [Type locality: Guatemala, Huehuetenango, 32 km NW Huehuetenango; NMNH; ♂]. —Bueno-Soria, 2009:154 [♂].

**Distribution.** Guatemala.

***avicula*** Bueno-Soria and Holzenthal, 2008:42 [Type locality: Costa Rica, Puntarenas, Río Jaba at rock quarry, 1.4 km (air) W Las Cruces, 8.79°N, 82.97°W, el. 1150 m; UMSP; ♂].

**Distribution.** Costa Rica.

***avis*** Bueno-Soria and Holzenthal, 1998:606 [Type locality: Costa Rica, Alajuela, Reserva Forestal San Rámon, Río San Lorencito and tribs, 10°12'96"N, 84°36'42"W; NMNH; ♂]. —Bueno-Soria and Holzenthal, 2008:48 [distribution]. —Bueno-Soria, 2009:115 [♂].

**Distribution.** Costa Rica.

***ayaya*** Botosaneanu, 1977:260 [Type locality: Cuba, Oriente, Baracoa, Rio Sabanilla; NMNH; ♂; as subspecies of *Ochrotrichia insularis*]. —Botosaneanu, 1979:49 [distribution]. —Flint, 1996c:16 [checklist]. —Botosaneanu, in Botosaneanu and Hyslop, 1998:13 [as distinct species]. —Botosaneanu, 2002:86 [checklist]. —Naranjo López and González Lazo, 2005:149 [checklist].

**Distribution.** Cuba.

***balra*** Oláh and Johanson, 2011:214 [Type locality: French Guiana, Approuaguekaw, Kaw Mt, 4°33.035'N, 52°11.661'W, el. 104 m; NHRS; ♂].

**Distribution.** French Guiana.

***baorucoensis*** Flint and Sykora, 2004:36 [Type locality: Dominican Republic, Barahoma Province, San Rafael, 8.3 km S of Baoruco, 18°01.9'N, 71°8.4'W, el. 30 m, NMNH; ♂]. —Pérez-Gelabert, 2008:300 [checklist].

**Distribution.** Dominican Republic.

***bicaudata*** Bueno-Soria and Santiago-Fragoso, 1997:367 [Type locality: Panama, Barro Colorado Island, Snyder-Molino Trail, Marker 3; NMNH; ♂]. —Bueno-Soria, 2009:115 [♂]. —Armitage et al., 2015b:7 [checklist]. —Armitage and Cornejo, 2015:196 [checklist].

**Distribution.** Panama.

***bipartita*** Flint and Bueno-Soria, 1999:732 [Type locality: Peru, Department Cuzco, Province Paucartambo, stream, 50 m E Quitacalzón; MHNJP; ♂; ♀].

**Distribution.** Peru.

***blanca*** Bueno-Soria and Santiago-Fragoso, 1997:363 [Type locality: Belize, Cayo District, Rio Privassion, Blancaneaux Lodge; NMNH; ♂]. —Bueno-Soria, 2009:129 [♂].

**Distribution.** Belize.

***boquillas*** Moulton and Harris, 1997:496 [Type locality: United States of America, Texas, Brewster Co, Glenn Spring, Big Bend National Park; NMNH; ♂]. —Baumgardner and Bowles, 2005:11 [distribution]. —Bowles et al., 2007:22 [distribution; biology]. —Bueno-Soria et al., 2007:33 [distribution].

**Distribution.** Mexico, U.S.A.

***bractea*** Bueno-Soria and Holzenthal, 2004:252 [Type locality: Mexico, Morelos, Municipio de Huautla, Reserva de la Biosfera de Huautla, 18°20'10”–18°34'20"N, 98°51'20”–99°08'15"W, el. 900 m; CNIN; ♂]. —Bueno-Soria, 2009:130 [♂].

**Distribution.** Mexico.

***brayi*** Flint, 1968b:61 [Type locality: Dominica, Freshwater Lake, NMNH; ♂]. —Flint and Sykora, 1993:50 [checklist]. —Botosaneanu, 2000:250 [♀]. —Botosaneanu, 2002:86 [checklist]. —Botosaneanu and Thomas, 2005:55 [checklist].

**Distribution.** Dominica, Guadaloupe.

† ***brodzinskyi*** Wells and Wichard, 1989:45 [Type locality: Dominican Republic; collection Wichard; ♂; in amber]. —Flint and Pérez-Gelabert, 1999:40 [checklist]. —Botosaneanu, 2002:86 [checklist]. —Wichard, 2007a:48 [checklist]. —Pérez-Gelabert, 2008:300 [checklist].

**Distribution.** Dominican Republic.

***caatinga*** Souza, Santos and Takiya, 2014a:274 [Type locality: Brazil, Ceará, Ubajara, Parque Nacional de Ubajara, Rio Cafundó, acima da cachoeira, 3°50'13"S, 40°54'19"W, el. 874 m; CZMA; ♂]. —Paprocki and França, 2014:50 [checklist].

**Distribution.** Brazil.

***cachonera*** Botosaneanu, 1995:23 [Type locality: Dominican Republic, springs near La Descubierta, S of Sierra de Neiba; ZMUA; ♂]. —Flint and Pérez-Gelabert, 1999:40 [checklist]. —Botosaneanu, 2002:86 [checklist]. —Flint and Sykora, 2004:36 [distribution]. —Pérez-Gelabert, 2008:300 [checklist].

**Distribution.** Dominican Republic.

***caimita*** Flint, 1972a:6 [Type locality: Panama, Chiriqui, Rio Caimito, 16 km NW David; NMNH; ♂]. —Aguila, 1992:538 [distribution]. —Bueno-Soria and Holzenthal, 2008:49 [distribution]. —Bueno-Soria, 2009:134 [♂]. —Armitage et al., 2015b:7 [checklist]. —Armitage and Cornejo, 2015:196 [checklist].

**Distribution.** Costa Rica, Panama.

***calcarata*** Flint and Bueno-Soria, 1999:733 [Type locality: Peru, Department Cuzco, Province Paucartambo, Puente San Pedro at km 152, 44 km (road) W of Pilcopata, 13°03.30'S, 71°32.78'W; MHNJP; ♂].

—Ochrotrichia (Ochrotrichia) n. sp. 3 Flint, 1996b:398. —Flint and Bueno-Soria, 1999:733 [to synonymy].

**Distribution.** Peru.

***caligula*** Flint, 1968a:49 [Type locality: Jamaica, St. Andrew, Hope River near New castle at mile post 16.5; NMNH; ♂]. —Flint, 1968b:82 [checklist]. —Botosaneanu, 2002:86 [checklist].

**Distribution.** Jamaica.

***campanilla*** Flint and Bueno-Soria, 1999:735 [Type locality: Peru, Department Madre de Dios, Province Manu, Pakitza, trail 1, 1st stream; MHNJP; ♂].

—Ochrotrichia (Ochrotrichia) n. sp. 4 Flint, 1996b:398. —Flint and Bueno-Soria, 1999:735 [to synonymy].

**Distribution.** Peru.

***canicula*** Bueno-Soria, 2009:148 [Type locality: Mexico, Estado de Mexico, Tetesontle, 19°05'37"N, 98°36'22"W, el. 3350 m; CNIN; ♂].

**Distribution.** Mexico.

***caramba*** Botosaneanu, 1977:262 [Type locality: Cuba, Oriente, Gran Piedra, Arroyos de la Idalia; NMNH; ♂]. —Botosaneanu, 1979:48 [distribution]. —Flint, 1996c:16 [checklist]. —Botosaneanu, 2002:86 [checklist]. —Naranjo López and González Lazo, 2005:149 [checklist].

**Distribution.** Cuba.

***catarina*** Bueno-Soria and Holzenthal, 2004:249 [Type locality: Mexico, Oaxaca, Santa Catarina La Chatao, 17°15'58"N, 96°28'15W, el. 2160 m; CNIN; ♂]. —Bueno-Soria, 2009:116 [♂].

**Distribution.** Mexico.

***cavitectum*** Botosaneanu, in Botosaneanu and Hyslop, 1998:13 [Type locality: Jamaica, St. Ann, Roaring River W. from Ocho Rios; ZMUA; ♂]. —Botosaneanu, 2002:86 [checklist].

**Distribution.** Jamaica.

† ***chaulioda*** Wells and Wichard, 1989:46 [Type locality: Dominican Republic; NMNH; ♂; in amber]. —Flint and Pérez-Gelabert, 1999:40 [checklist]. —Botosaneanu, 2002:86 [checklist]. —Wichard, 2007a:48 [checklist]. —Pérez-Gelabert, 2008:300 [checklist].

**Distribution.** Dominican Republic.

***chiapa*** Denning and Blickle, 1972:147 [Type locality: Mexico, Chiapas, 6 miles south of Puebla Nueva; CAS; ♂]. —Bueno-Soria and Flint, 1978:203 [distribution]. —Bueno-Soria, 2009:148 [♂]. —Bueno-Soria and Barba-Álvarez, 2011:358 [checklist].

**Distribution.** Mexico.

***cieneguilla*** Harris, in Harris and Moulton, 1993:545 [Type locality: Mexico, Nuevo León, Municipio de Santiago, Cola de Caballo, down stream falls, 3 km SW Cieneguilla; NMNH; ♂]. —Bueno-Soria, 2009:123 [♂].

**Distribution.** Mexico.

***citra*** Bueno-Soria and Holzenthal, 2004:247 [Type locality: Mexico, Tabasco, Municipio de Huimanguillo Rta. Malpasito-Carlos A. Madrazo, Ejido Villa de Guadalupe, 1a seccíon Cascada Cerro de las Flores, 17°21'39"N, 93°37'29"W, el. 540 m; CNIN; ♂]. —Bueno-Soria, 2009:117 [♂].

**Distribution.** Mexico.

***compacta*** Bueno-Soria and Holzenthal, 2004:247 [Type locality: Mexico, Tabasco, Municipio de Huimanguillo Rta. Malpasito-Carlos A. Madrazo, Ejido Villa de Guadalupe, 1a seccíon Cascada Cerro de las Flores, 17°21'39"N, 93°37'29"W, el. 540 m; CNIN; ♂]. —Bueno-Soria, 2009:117 [♂].

**Distribution.** Mexico.

***concha*** Bueno-Soria and Santiago-Fragoso, 1992:446 [Type locality: Brazil, Amazonas State, AM010, km 246, ca. 20 km W Itacoatiara; NMNH; ♂]. —Angrisano, 1999:34 [checklist]. —Paprocki et al., 2004:11 [checklist]. —Paprocki and França, 2014:50 [checklist].

**Distribution.** Brazil.

***conformalis*** Bueno-Soria and Holzenthal, 2008:45 [Type locality: Costa Rica, Alajuela, Reserva Forestal San Ramón Río San Lorencito and tribs., 10.216°N, 84.607°W, el. 980 m; UMSP; ♂].

**Distribution.** Costa Rica.

***confusa*** (Morton), 1905:69 [Type locality: USA, New York, Ithaca; type depository not stated, likely BMNH or CU; in *Ithytrichia*]. —Ross, 1944:133 [larva; to *Ochrotrichia*]. —Denning and Blickle, 1972:142 [checklist]. —Bueno-Soria et al., 2007:33 [distribution]. —Bueno-Soria, 2009:124 [♂].

**Distribution.** Mexico, U.S.A.

***constricta*** Souza, Santos and Takiya, 2014a:278 [Type locality: Brazil, Igrapiúna, Reserva Ecológica Michelin, Mata da Vila 5, 13°49'22.9"S, 39°12'6.5"W, el. 87 m; DZRJ; ♂]. —Paprocki and França, 2014:50 [checklist].

**Distribution.** Brazil.

***contrerasi*** Harris, in Harris and Moulton, 1993:545 [Type locality: Mexico, Tamaulipas, Municipio de Gómez Farias, Río Frio at La Poza Azul, 6 km S Gómez Farias; NMNH; ♂]. —Bueno-Soria, 2009:149 [♂].

**Distribution.** Mexico.

***corneolus*** Bueno-Soria and Santiago-Fragoso, 1997:365 [Type locality: Panama, Barro Colorado Island, Snyder-Molino Trail, Marker 3; NMNH; ♂]. —Bueno-Soria, 2009:135 [♂]. —Armitage et al., 2015b:7 [checklist]. —Armitage and Cornejo, 2015:196 [checklist].

**Distribution.** Panama.

***crucecita*** Bueno-Soria and Santiago-Fragoso, 1997:360 [Type locality: Panama, Chiriqui, Guadalupe Arriba, 8°52'26"N, 82°33'13"W; NMNH; ♂]. —Bueno-Soria, 2009:144 [♂]. —Armitage et al., 2015b:7 [checklist]. —Armitage and Cornejo, 2015:196 [checklist].

**Distribution.** Panama.

***cruces*** Flint, 1967b:12 [Type locality: Mexico, Las Cruces National Park, La Marquesa; NMNH; ♂]. —Bueno-Soria and Flint, 1978:203 [distribution]. —Bueno-Soria, 2009:145 [♂].

**Distribution.** Mexico.

***csiga*** Oláh and Johanson, 2011:215 [Type locality: Peru, San Martin Prov., creek crossing rd. Juan Guerra-Chazuta, 14 km (rd.) E Colombia Bridge, 6°35.594'S, 76°13.172'W; NHRS; ♂].

**Distribution.** Peru.

***curvata*** Bueno-Soria and Holzenthal, 2004:250 [Type locality: Panama, Chiriqui: Fortuna Dam site near Hornitos, 8°55'N, 82°16'W, el. 1050 m; NMNH; ♂]. —Bueno-Soria, 2009:135 [♂]. —Armitage et al., 2015b:7 [checklist]. —Armitage and Cornejo, 2015:196 [checklist].

**Distribution.** Panama.

***cuspidatus*** Bueno-Soria and Holzenthal, 2004:254 [Type locality: Mexico, Guerrero, Municipio Taxco, Teusisapan. Río Temascalapa, 12 km. NW Ahuehuepan Rta. 51, 18°25.56'N, 99°42.5'W, el. 1052 m; CNIN; ♂]. —Bueno-Soria, 2009:149 [♂].

**Distribution.** Mexico.

***dactylophora*** Flint, 1965:171 [Type locality: USA, Arizona, coconi County, West Fork, 16 miles Southwest of Flagstaff, 6500 ft; ♂]. —Denning and Blickle, 1972:142 [checklist]. —Baumgardner and Bowles, 2005:11 [distribution]. —Blinn and Ruiter, 2006:333 [biology]. —Bueno-Soria et al., 2007:33 [distribution]. —Blinn and Ruiter, 2009a:303 [biology]. —Blinn and Ruiter, 2009b:186 [phenology, distribution]. —Bueno-Soria, 2009:125 [♂; distribution].

**Distribution.** Mexico, U.S.A.

***delgada*** Bueno-Soria and Holzenthal, 2004:253 [Type locality: Mexico, Tabasco, Municipio de Huimanguillo, km 5 Ruta Malpasito-Carlos A. Madrazo, Arroyo Las Flores, Villa de Guadalupe 2a sección Los Chimalapas, 17°22'05"N, 93°36'25"W, el. 545 m; CNIN; ♂]. —Bueno-Soria, 2009:150 [♂].

**Distribution.** Mexico.

† ***denaia*** Wells and Wichard, 1989:47 [Type locality: Dominican Republic; NMNH; ♂; in amber]. —Flint and Pérez-Gelabert, 1999:40 [checklist]. —Botosaneanu, 2002:86 [checklist]. —Wichard, 2007a:48 [checklist]. —Pérez-Gelabert, 2008:301 [checklist].

**Distribution.** Dominican Republic.

† ***doehleri*** Wichard, 1981:161 [Type locality: Dominican Republic; collection Wichard; ♂; in amber]. —Wells and Wichard, 1989:48 [♂; redescription]. —Flint and Pérez-Gelabert, 1999:40 [checklist]. —Botosaneanu, 2002:86 [checklist]. —Wichard, 2007a:48 [checklist]. —Pérez-Gelabert, 2008:301 [checklist].

**Distribution.** Dominican Republic.

***dulce*** Bueno-Soria and Holzenthal, 1998:608 [Type locality: Costa Rica, Guanacaste, Río Tizate, 7.2 km N.E. Cañas Dulces, 10°43'98"N, 66°[sic, should be 85°]26'94"W; NMNH; ♂]. —Bueno-Soria and Holzenthal, 2008:49 [distribution]. —Bueno-Soria, 2009:140 [♂].

**Distribution.** Costa Rica.

***ecuatoriana*** Bueno-Soria and Santiago-Fragoso, 1992:440 [Type locality: Ecuador, Pastaza Prov., Puyo; NMNH; ♂]. —Muñoz-Quesada, 2000:278 [checklist]. —Oláh and Johanson, 2011:218 [distribution].

**Distribution.** Columbia, Ecuador, Peru.

***escoba*** Flint, 1972a:9 [Type locality: Guatemala, Izabal, Las Escobas, near Matias de Galvez; NMNH; ♂]. —Bueno-Soria, 2009:141 [♂]. —Bueno-Soria and Barba-Álvarez, 2011:358 [checklist].

**Distribution.** Guatemala.

***eyipantla*** Bueno-Soria and Santiago-Fragoso 1997:363 [Type locality: Mexico, Veracruz, Salto de Eyipantla, Eyipantla River; IBUNAM; ♂]. —Bueno-Soria et al., 2005:75 [distribution]. —Bueno-Soria, 2009:130 [♂].

**Distribution.** Mexico.

***felipe*** Ross, 1944:275 [Type locality: United States, Texas, San Felipe Springs, Del Rio; INHS; ♂]. —Flint, 1972a:9 [♂; distribution]. —Bueno-Soria and Flint, 1978:203 [distribution]. —Bueno-Soria, 2009:141 [♂]. —Bowles et al., 2007:22 [distribution; biology].

**Distribution.** Mexico, U.S.A.

***filiforma*** Flint, 1972a:9 [Type locality: Costa Rica, Cartago, Chitaria; NMNH; ♂]. —Holzenthal, 1988c:62 [distribution]. —Bueno-Soria and Holzenthal, 2008:49 [distribution]. —Bueno-Soria, 2009:142 [♂].

**Distribution.** Costa Rica.

***fioka*** Oláh and Johanson, 2011:217 [Type locality: Peru, San Martin Prov., La Catarata de Ahuashiyascu, 6°27.544'S, 76°18.192'W; NHRS; ♂].

**Distribution.** Peru.

***flagellata*** Flint, 1972a:5 [Type locality: Panama Canal Zone, Barro Colorado Island; NMNH; ♂]. —Aguila, 1992:538 [distribution]. —Bueno-Soria, 2009:110 [♂; distribution]. —Armitage et al., 2015b:7 [checklist]. —Armitage and Cornejo, 2015:196 [checklist].

**Distribution.** Panama, Mexico.

***flexura*** Flint and Bueno-Soria, 1999:732 [Type locality: Peru, Department Madre de Dios, Province Manu, Pakitza, trail 2, marker 15, Quebrada Trompetero; MHNJP; ♂].

—Ochrotrichia (Ochrotrichia) n. sp. 8 Flint, 1996b:399. —Flint and Bueno-Soria, 1999:732 [to synonymy].

**Distribution.** Peru.

***flintiana*** Kumanski, 1987:18 [Type locality: Cuba, Province las Villas, S. foothills of Sierra de Trinidad, Rio San Juan de la Juyua; NMSB; ♂]. —Flint, 1996c:16 [checklist]. —Botosaneanu, 2002:86 [checklist]. —Naranjo López and González Lazo, 2005:149 [checklist].

**Distribution.** Cuba.

***glabra*** Bueno-Soria and Santiago-Fragoso, 1997:364 [Type locality: Panama, Chiriqui, Guadalupe Arriba, 8°52'26"N, 82°33'13"W; NMNH; ♂]. —Bueno-Soria and Holzenthal, 2008:49 [distribution]. —Bueno-Soria, 2009:110 [♂]. —Armitage et al., 2015b:7 [checklist]. —Armitage and Cornejo, 2015:196 [checklist].

**Distribution.** Costa Rica, Panama.

***gretae*** Bueno-Soria, 2009:113 [Type locality: Mexico, Chihuahua, Mpio. Guachochi, km 114.5 Route 25 Creel-Guachochi, 27°35'05"N, 107°32'46"W, el. 2150 m; CNIN; ♂].

**Distribution.** Mexico.

***gurneyi*** Flint, 1964a: 60 [Type locality: Puerto Rico, El Yunque, cabins at La Mina; NMNH; ♂]. —Flint, 1968b:82 [checklist]. —Botosaneanu, 2002:86 [checklist].

**Distribution.** Puerto Rico.

***hamatilis*** Flint and Bueno-Soria, 1999:733 [Type locality: Peru, Department Madre de Dios, Province Manu, Pakitza, trail 2, first stream; MHNJP; ♂; ♀].

—Ochrotrichia (Ochrotrichia) n. sp. 6 Flint, 1996b:398. —Flint and Bueno-Soria, 1999:733 [to synonymy].

**Distribution.** Peru.

***harmas*** Oláh and Johanson, 2011:218 [Type locality: Peru, San Martin Prov., creek crossing rd. Juan Guerra-Chazuta, 14 km (rd.) E Colombia Bridge, 6°35.594'S, 76°13.172'W; NHRS; ♂].

**Distribution.** Peru.

***hata*** Oláh and Johanson, 2011:220 [Type locality: French Guiana, Approuaguekaw, Kaw Mt, 4°33.072'N, 52°12.462'W, el. 270 m; NHRS; ♂].

**Distribution.** French Guiana.

***hondurenia*** Bueno-Soria and Santiago-Fragoso, 1997:364 [Type locality: Belize, Cayo District, Mountain Pine Ridge; NMNH; ♂]. —Bueno-Soria and Holzenthal, 2008:49 [distribution]. —Bueno-Soria, 2009:111 [♂].

**Distribution.** Belize, Costa Rica.

***igrapiuna*** Souza, Santos and Takiya, 2014a:279 [Type locality: Brazil, Igrapiúna, Reserva Ecológica Michelin, Mata da Vila 5, 13°49'22.9"S, 39°12'6.5"W, el. 87 m; DZRJ; ♂]. —Paprocki and França, 2014:50 [checklist].

**Distribution.** Brazil.

***ildria*** Denning and Blickle, 1972:145 [Type locality: United States of America, Arizona, Oak Creek Canyon; CAS; ♂]. —Blinn and Ruiter, 2006:333 [biology]. —Bueno-Soria et al., 2007:33 [distribution]. —Bueno-Soria, 2009:150 [♂]. —Blinn and Ruiter, 2009a:306 [biology]. —Blinn and Ruiter, 2009b:186 [phenology, distribution].

**Distribution.** Mexico, U.S.A.

***indefinida*** Bueno-Soria and Holzenthal, 2004:248 [Type locality: Mexico, Tabasco, Municipio de km 5 Ruta Malpasito-Carlos A. Madrazo, Arroyo las Flores Villa de Guadalupe 2da sección Los Chimalapas, 17°22'05"N, 93°36'25"W, el. 545 m; CNIN; ♂]. —Bueno-Soria, 2009:136 [♂].

**Distribution.** Mexico.

***ingloria*** Botosaneanu, 1995:25 [Type locality: Dominican Republic, springs near La Descubierta, S of Sierra de Neiba; ZMUA; ♂]. —Flint and Pérez-Gelabert, 1999:40 [checklist]. —Botosaneanu, 2002:86 [checklist]. —Flint and Sykora, 2004:37 [distribution]. —Pérez-Gelabert, 2008:301 [checklist].

**Distribution.** Dominican Republic.

***insularis*** Mosely, 1934b:163 [Type locality: Jamaica, Runnaway Bay; BMNH; ♂]. —Flint, 1968a:49 [♂; ♀]. —Flint, 1968b:82 [checklist]. —Botosaneanu and Hyslop, 1998:12 [♂; “enantiomorphic” morphotypes]. —Botosaneanu, 2002:86 [checklist].

**Distribution.** Jamaica.

***intermedia*** Flint, 1972a:10 [Type locality: Guatemala, Chimaltenango, Tecpan Guatemala; NMNH; ♂]. —Bueno-Soria, 2009:142 [♂].

**Distribution.** Guatemala.

***intortilis*** Flint and Bueno-Soria, 1999:730 [Type locality: Peru, Department Cuzco, Province Paucartambo, Puente San Pedro at km 152, 44 km (road) W of Pilcopata, 13°03.30'S, 71°32.78'W; MHNJP; ♂; ♀].

—Ochrotrichia (Ochrotrichia) n. sp. 1 Flint, 1996b:398. —Flint and Bueno-Soria, 1999:730 [to synonymy].

**Distribution.** Peru.

***involuta*** Bueno-Soria and Holzenthal, 2004:249 [Type locality: Mexico, Guerrero, km 7 Route Taxco-Ixcateopan; CNIN; ♂]. —Bueno-Soria, 2009:143 [♂].

**Distribution.** Mexico.

***islena*** Botosaneanu, 1977:261 [Type locality: Cuba, Isla de Pinos, Santa Fé, Arroyo La Talega; NMNH; ♂]. —Botosaneanu, 1979:49 [distribution]. —Kumanski, 1987:17 [distribution; as *Ochrotrichia islenia*]. —Flint, 1996c:16 [checklist, as *Ochrotrichia islenia*]. —Botosaneanu, 2002:86 [checklist]. —Naranjo López and González Lazo, 2005:149 [checklist; as *Ochrotrichia islenia*].

**Distribution.** Cuba.

***ixcateopana*** Bueno-Soria and Santiago-Fragoso, 1997:360 [Type locality: Mexico, Guerrero, 7 km Route Taxco-Ixcateopan; IBUNAM; ♂]. —Rojas-Ascencio, et al., 2002:377 [distribution]. —Bueno-Soria, 2009:125 [♂].

**Distribution.** Mexico.

***ixtlahuaca*** Bueno-Soria and Holzenthal, 2004:253 [Type locality: Mexico, Hidalgo, Ixtlahuaco, Ruta 105, Hotel Campestre Conchita, 20°53.04'N, 98°41'W, el. 1420 m; CNIN; ♂]. —Bueno-Soria, 2009:151 [♂].

**Distribution.** Mexico.

***jolandae*** Bueno-Soria and Holzenthal, 2008:43 [Type locality: Costa Rica, Alajuela, Reserva Forestal San Ramón Río San Lorencito and tribs., 10.216°N, 84.607°W, el. 980 m; UMSP; ♂].

**Distribution.** Costa Rica.

***jonssoni*** Oláh and Johanson, 2011:221 [Type locality: French Guiana, Approuaguekaw, Kaw Mt, 4°33.133'N, 52°12.205'W, el. 263 m; NHRS; ♂].

**Distribution.** French Guiana.

***ketaga*** Oláh and Johanson, 2011:222 [Type locality: Peru, San Martin Prov., creek crossing rd. Tarapoto-Yurimaguas, ca. 30 km (rd.) NE Tarapoto, 6°24.904'S, 76°18.756'W; NHRS; ♂].

**Distribution.** Peru.

***ketarca*** Oláh and Johanson, 2011:224 [Type locality: Peru, Chontachaca, Kosnipata-Cusco, humid subtropical forest, 13°01'25"S, 71°28'03'W, el. 700 m; NHRS; ♂].

**Distribution.** Peru.

***kettes*** Oláh and Johanson, 2011:225 [Type locality: Peru, San Martin Prov., creek crossing rd. Juan Guerra-Chazuta, 14 km (rd.) E Colombia Bridge, 6°35.594'S, 76°13.172'W; NHRS; ♂].

**Distribution.** Peru.

***labafura*** Oláh and Johanson, 2011:227 [Type locality: French Guiana, Approuaguekaw, Kaw Mt, 4°33.035'N, 52°11.661'W, el. 104 m; NHRS; ♂].

**Distribution.** French Guiana.

***larimar*** Flint and Sykora, 2004:38 [Type locality: Dominican Republic, Barahona Pro­vince, Larimar Mine nr Filipinas; FSCA; ♂; ♀]. —Pérez-Gelabert, 2008:301 [checklist].

**Distribution.** Dominican Republic.

***legeza*** Oláh and Johanson, 2011:228 [Type locality: Peru, San Martin Prov., La Catarata de Ahuashiyascu, 6°27.544'S, 76°18.192'W; NHRS; ♂].

**Distribution.** Peru.

***leona*** Bueno-Soria and Holzenthal, 2004:257 [Type locality: Mexico, Distrito Federal, Desierto de Los Leones, Arroyo San Borja, 19°18.140'N, 99°18.648'W, el. 2650 m; CNIN; ♂]. —Bueno-Soria, 2009:126 [♂].

**Distribution.** Mexico.

***limeirai*** Souza, Santos and Takiya, 2014a:277 [Type locality: Brazil, Ceará, Ubajara, Parque Nacional de Ubajara, 3°50'31.7"S, 40°53'55"W; CZMA; ♂]. —Paprocki and França, 2014:50 [checklist].

**Distribution.** Brazil.

***limonensis*** Flint, 1981a:29 [Type locality: Venezuela, Aragua, Dos Riitos, 6 km N Rancho Grande; NMNH; ♂].

**Distribution.** Venezuela.

***lobifera*** Flint, 1968a:50 [Type locality: Jamaica, St. Andrew, Hope River near Newcastle at mile post 16.5; NMNH; ♂]. —Flint, 1968b:82 [checklist]. —Botosaneanu, 2002:86 [checklist].

**Distribution.** Jamaica.

***logana*** (Ross), 1941:54 [Type locality: [United States], Utah, Logan Canyon; INHS; ♂; ♀; in *Polytrichia*]. —Ross, 1944:295 [to *Ochrotrichia*]. —Denning and Blickle, 1972:142 [checklist]. —Blinn and Ruiter, 2006:333 [biology]. —Blinn and Ruiter, 2009a:305 [biology]. —Bueno-Soria, 2009:152 [♂; distribution]. —Bueno-Soria and Barba-Álvarez, 2011:358 [checklist].

**Distribution.** Mexico, U.S.A.

***longispina*** Bueno-Soria and Holzenthal, 2004:250 [Type locality: Panama, Chiriqui: Fortuna Dam site near Hornitos, 8°55'N, 82°16'W, el. 1050 m; NMNH; ♂]. —Bueno-Soria and Holzenthal, 2008:50 [distribution]. —Bueno-Soria, 2009:118 [♂]. —Oláh and Johanson, 2011:229 [distribution]. —Armitage et al., 2015b:7 [checklist]. —Armitage and Cornejo, 2015:196 [checklist].

**Distribution.** Costa Rica, Panama, Peru.

***lupita*** Bueno-Soria and Santiago-Fragoso, 1997:368 [Type locality: Panama, Chiriqui, Guadalupe Arriba, 8°52'26"N, 82°33'13"W; NMNH; ♂]. —Bueno-Soria, 2009:118 [♂]. —Armitage et al., 2015b:7 [checklist]. —Armitage and Cornejo, 2015:196 [checklist].

**Distribution.** Panama.

***machiguenga*** Flint and Bueno-Soria, 1999:733 [Type locality: Peru, Department Madre de Dios, Province Manu, Pakitza, trail 1, marker 14 (1st stream); MHNJP; ♂].

—Ochrotrichia (Ochrotrichia) n. sp. 7 Flint, 1996b:398. —Flint and Bueno-Soria, 1999:733 [to synonymy].

**Distribution.** Peru.

***maga*** Oláh and Johanson, 2011:229 [Type locality: Peru, San Martin Prov., La Catarata de Ahuashiyascu, 6°27.544'S, 76°18.192'W; NHRS; ♂].

**Distribution.** Peru.

***manuensis*** Flint and Bueno-Soria, 1999:735 [Type locality: Peru, Department Madre de Dios, Province Manu, Pakitza, trail 2, first stream; MHNJP; ♂; ♀]. —Souza et al., 2014a:281 [distribution]. —Paprocki and França, 2014:50 [checklist].

—Ochrotrichia (Ochrotrichia) n. sp. 5 Flint, 1996b:398. —Flint and Bueno-Soria, 1999:735 [to synonymy].

**Distribution.** Brazil, Peru.

***marica*** Flint, 1964a:60 [Type locality: Puerto Rico, Maricao Forest, at stone house; NMNH; ♂]. —Flint, 1968b:82 [checklist]. —Botosaneanu, 2002:86 [checklist].

**Distribution.** Puerto Rico.

***maya*** Bueno-Soria and Santiago-Fragoso, 1997:369 [Type locality: Mexico, Chiapas, Cascada de Misolja, 20 km S from Palenque; NMNH; ♂]. —Bueno-Soria, 2009:137 [♂]. —Bueno-Soria and Barba-Álvarez, 2011:358 [checklist].

**Distribution.** Mexico.

***maycoba*** Bueno-Soria and Santiago-Fragoso 1997:363 [Type locality: Mexico, Sonora, Maycoba River, west of Maycoba; NMNH; ♂]. —Bueno-Soria, 2009:131 [♂].

**Distribution.** Mexico.

***membrana*** Bueno-Soria and Holzenthal, 1998:604 [Type locality: Costa Rica, Alajuela, Reserva Forestal San Ramón, Río San Lorencito and tribs, 10°12'96"N, 84°36'42"W; NMNH; ♂]. —Bueno-Soria and Holzenthal, 2008:50 [distribution]. —Bueno-Soria, 2009:119 [♂].

**Distribution.** Costa Rica.

***moselyi*** Flint, 1972a:7 [Type locality: Mexico, Veracruz, Rio Tacolapan, route 180, km 551; NMNH; ♂]. —Bueno-Soria and Flint, 1978:203 [distribution]. —Luhman et al., 1999:126 [pupae parasitized by Hymenoptera: Ceraphronidae; distribution]. —Bueno-Soria et al., 2005:75 [distribution]. —Bueno-Soria et al., 2007:33 [distribution]. —Bueno-Soria and Holzenthal, 2008:50 [distribution]. —Bueno-Soria, 2009:126 [♂]. —Bueno-Soria and Barba-Álvarez, 2011:358 [checklist].

**Distribution.** Costa Rica, Guatemala, Mexico.

***nicaragua*** Bueno-Soria, 2009:153 [Type locality: Nicaragua, Zelaya, Cerro Saslaya, 13°44'N, 85°01'W, el. 700 m; NMNH; ♂].

**Distribution.** Nicaragua.

***nimmoi*** Harris and Armitage, 2015:13 [Type locality: Chiriquí Province, Cuenca 108, tributary of Quebrada Grande, at waterfall, Boquete, Valle Escondido, 8.78291°N, 82.44579°W, 1253 m; MIUP; ♂]. —Armitage et al., 2015b:7 [checklist]. —Armitage and Cornejo, 2015:196 [checklist].

**Distribution.** Panama.

***oblongata*** Bueno-Soria and Santiago-Fragoso, 1992:443 [Type locality: Trinidad, Arima cascade; NMNH; ♂]. —Botosaneanu and Sakal, 1992:202 [distribution; ecology, as Ochrotrichia (Ochrotrichia)]. —Botosaneanu and Alkins-Koo, 1993:17 [♂; /female]. —Flint, 1996a:93 [distribution]. —Botosaneanu, 2002:86 [checklist].

**Distribution.** Trinidad, Venezuela.

***obovata*** Flint and Sykora, 2004:38 [Type locality: Dominican Republic, [La Vega Province] 20 km S Constanza; NMNH; ♂]. —Pérez-Gelabert, 2008:301 [checklist].

**Distribution.** Dominican Republic.

***obtecta*** Flint and Bueno-Soria, 1999:730 [Type locality: Peru, Department Cuzco, Province Paucartambo, Puente San Pedro at km 152, 44 km (road) W of Pilcopata, 13°03.30'S, 71°32.78'W; MHNJP; ♂; ♀].

—Ochrotrichia (Ochrotrichia) n. sp. 2 Flint, 1996b:398. —Flint and Bueno-Soria, 1999:730 [to synonymy].

**Distribution.** Peru.

***okanoganensis*** Flint, 1965:171 [Type locality: [United States], Washington, Okanogan County, Winthrop; NMNH; ♂]. —Bueno-Soria et al., 2007:33 [distribution; as *Ochrotrichia okanogensis*].

**Distribution.** Mexico, U.S.A.

***oldala*** Oláh and Johanson, 2011:231 [Type locality: French Guiana, Approuaguekaw, Kaw Mt, 4°33.035'N, 52°11.661'W, el. 104 m; NHRS; ♂].

**Distribution.** French Guiana.

***ostoroska*** Oláh and Johanson, 2011:232 [Type locality: Peru, San Martin Prov., La Catarata de Ahuashiyascu, 6°27.544'S, 76°18.192'W; NHRS; ♂].

**Distribution.** Peru.

***pacifica*** Flint, 1972a:6 [Type locality: Panama, Chiriqui, Rio Caimito, 16km NW David; NMNH; ♂]. —Bueno-Soria and Flint, 1978:204 [distribution]. —Holzenthal, 1988c:62 [distribution]. —Aguila, 1992:538 [distribution]. —Bueno-Soria et al., 2005:75 [distribution]. —Bueno-Soria and Holzenthal, 2008:50 [checklist]. —Bueno-Soria, 2009:137 [♂]. —Bueno-Soria and Barba-Álvarez, 2011:358 [checklist]. —Armitage et al., 2015b:7 [checklist]. —Armitage and Cornejo, 2015:196 [checklist].

**Distribution.** Costa Rica, Mexico, Panama.

***palitla*** Flint, 1972a:9 [Type locality: Mexico, San Luis Potosi, Palitla; NMNH; ♂]. —Bueno-Soria and Flint, 1978:204 [distribution]. —Bueno-Soria, 2009:143 [♂].

**Distribution.** Mexico.

***palmata*** Bueno-Soria and Santiago-Fragoso, 1997:369 [Type locality: Mexico, Estado de Mexico, Temascaltepec; IBUNAM; ♂]. —Bueno-Soria, 2009:138 [♂].

**Distribution.** Mexico.

***panamensis*** Flint, 1972a:10 [Type locality: Panama, Chiriqui, Rovira, David; NMNH; ♂]. —Aguila, 1992:539 [distribution]. —Chamorro-Lacayo et al., 2007:43 [checklist]. —Bueno-Soria and Holzenthal, 2008:51 [distribution]. —Bueno-Soria, 2009:153 [♂]. —Armitage et al., 2015b:7 [checklist]. —Armitage and Cornejo, 2015:196 [checklist].

**Distribution.** Costa Rica, Nicaragua, Panama.

***paraldama*** Bueno-Soria, 2009:113 [Type locality: Panama, San Blas, Río Cartí Grande, 2 km. Nusagandi; NMNH; ♂]. —Armitage et al., 2015b:7 [checklist]. —Armitage and Cornejo, 2015:196 [checklist].

**Distribution.** Panama.

***patulosa*** (Wasmund and Holzenthal), 2007:18 [Type locality: Brazil, Rio de Janeiro, Parque Nacional da Serra dos Órgãos, Rio Beija-flor, 22°27'04"S, 43°00'04"W, el. 1125 m; MZUSP; ♂; ♀; in *Rhyacopsyche*]. —Dumas et al., 2009:367 [distribution]. —Oláh and Johanson, 2011:234 [to *Ochrotrichia*]. —Souza et al., 2014a:281 [distribution]. —Paprocki and França, 2014:50 [checklist].

**Distribution.** Brazil.

***pectinata*** Flint, 1972a:5 [Type locality: Mexico, Veracruz, Rio Jamapa, north of Coscomatepec; NMNH; ♂]. —Bueno-Soria and Flint, 1978:204 [distribution]. —Bueno-Soria, 2009:111 [♂].

**Distribution.** Mexico.

***pectinifera*** Flint, 1972a:7 [Type locality: Mexico, Veracruz, Fortin de las Flores; NMNH; ♂]. —Bueno-Soria and Flint, 1978:204 [distribution]. —Bueno-Soria, 2009:127 [♂].

**Distribution.** Mexico.

***poblana*** Bueno-Soria and Santiago-Fragoso, 1997:370 [Type locality: Mexico, Puebla, km 30, route Zacapoaxtla-Zacatlán; IBUNAM; ♂]. —Bueno-Soria, 2009:154 [♂].

**Distribution.** Mexico.

***ponta*** Flint, 1968b:61 [Type locality: Dominica, Pont Casse, 0.4 mi E; NMNH; ♂]. —Flint and Sykora, 1993:58 [distribution]. —Botosaneanu, 1994a:37 [distribution]. —Botosaneanu, 2002:86 [checklist]. —Botosaneanu, and Thomas, 2005:44 [distribution].

**Distribution.** Dominica, Grenada, Guadeloupe, Martinique, St. Vincent.

***pora*** Angrisano and Sganga, 2009:62 [Type locality: Argentina, Misiones, Parque Provincial Salto Encantado, Salto Acutí; MACN; ♂].

**Distribution.** Argentina.

***priapo*** Souza, Santos and Takiya, 2014a:275 [Type locality: Brazil, Bahia, Igrapiúna, Reserva Ecológica da Michelin, Mata da Vila 5, 13°49'22.6"S, 39°12'6.5"W, el. 87 m; DZRJ; ♂]. —Paprocki and França, 2014:51 [checklist].

**Distribution.** Brazil.

***pulgara*** Harris and Armitage, 2015:13 [Type locality: Chiriquí Province, Cuenca 108, tributary of Quebrada Grande, at waterfall, Boquete, Valle Escondido, 8.78291°N, 82.44579°W, el. 1253 m; MIUP; ♂]. —Armitage et al., 2015b:7 [checklist]. —Armitage and Cornejo, 2015:196 [checklist].

**Distribution.** Panama.

***puposa*** Oláh and Johanson, 2011:234 [Type locality: Peru, San Martin Prov., La Catarata de Ahuashiyascu, 6°27.544'S, 76°18.192'W; NHRS; ♂].

**Distribution.** Peru.

***puyana*** Bueno-Soria and Santiago-Fragoso, 1992:440 [Type locality: Ecuador, Pastaza Prov., Puyo; NMNH; ♂].

**Distribution.** Ecuador.

***quasi*** Bueno-Soria and Holzenthal, 2008:46 [Type locality: Costa Rica, San José, Río Savegra, San Gerardo de Dota, 9.33°N, 83.48°W, el. 2200 m; CMC; ♂].

**Distribution.** Costa Rica.

***quebrada*** Bueno-Soria and Holzenthal, 1998:607 [Type locality: Costa Rica, Guanacaste, P.N. Rincón de la Vieja, Quebrada Zopilote, 10°45'9"N, 83°18'54"W; NMNH; ♂]. —Bueno-Soria and Holzenthal, 2008:51 [distribution]. —Bueno-Soria, 2009:119 [♂].

**Distribution.** Costa Rica.

***quinealensis*** Bueno-Soria and Holzenthal, 1998:611 [Type locality: Costa Rica, Puntarenas, Río Guineal, ca. 1 km (air) E Finca Helechales, 9°4'56"N, 83°5'52"W; NMNH; ♂]. —Bueno-Soria and Holzenthal, 2008:51 [distribution]. —Bueno-Soria, 2009:138 [♂].

**Distribution.** Costa Rica.

***ramona*** Bueno-Soria and Holzenthal, 1998:610 [Type locality: Costa Rica, Alajuela, Reserva Forestal San Ramón, Río San Lorencito and tribs, 10°12'96"N, 84°36'42"W; NMNH; ♂]. —Bueno-Soria and Holzenthal, 2008:52 [distribution]. —Bueno-Soria, 2009:146 [♂].

**Distribution.** Costa Rica.

***raposa*** Bueno-Soria and Santiago-Fragoso, 1992:440 [Type locality: Colombia, Dept. Valle del Cauca, Río Raposo; NMNH; ♂]. —Muñoz-Quesada, 2000:278 [checklist].

**Distribution.** Colombia, Ecuador.

***regina*** Bueno-Soria and Santiago-Fragoso, 1997:368 [Type locality: Panama, Barro Colorado Island, Snyder-Molino Trail, Marker 3; NMNH; ♂]. —Bueno-Soria, 2009:120 [♂]. —Armitage et al., 2015b:7 [checklist]. —Armitage and Cornejo, 2015:196 [checklist].

**Distribution.** Panama.

***regiomontana*** Bueno-Soria and Holzenthal, 2004:255 [Type locality: Mexico, Nuevo Leon, Municipio Santiago, Potrero Redondo; CNIN; ♂]. —Bueno-Soria, 2009:155 [♂].

**Distribution.** Mexico.

***rothi*** Denning and Blickle, 1972:149 [Type locality: [United States], Ariz[ona], Southwest Research Station, Cochise County, Portal; CAS; ♂; ♀]. —Baumgardner and Bowles, 2005:11 [distribution]. —Bueno-Soria et al., 2007:33 [distribution]. —Bueno-Soria, 2009:152 [♂].

**Distribution.** Mexico, U.S.A.

***seiba*** Flint and Sykora, 2004:38 [Type locality: Dominican Republic, El Seibo Province, Pedro Sanchez, small stream; FSCA; ♂; ♀]. —Pérez-Gelabert, 2008:301 [checklist].

**Distribution.** Dominican Republic.

***serra*** Botosaneanu, 1991a:125 [Type locality: Haiti, Département de l’Ouest, Ville Bonheur, Le Saut d’Eau; ZMUA; ♂]. —Flint and Pérez-Gelabert, 1999:41 [checklist]. —Botosaneanu, 2002:87 [checklist]. —Flint and Sykora, 2004:39 [distribution]. —Pérez-Gelabert, 2008:301 [checklist].

**Distribution.** Haiti.

***serrana*** Bueno-Soria and Santiago-Fragoso, 1997:370 [Type locality: Mexico, Guerrero, Acahuizotla, 10 km E of Chilpancingo; IBUNAM; ♂]. —Bueno-Soria, 2009:156 [♂].

**Distribution.** Mexico.

***silva*** Bueno-Soria and Holzenthal, 1998:606 [Type locality: Costa Rica, Alajuela, Reserva Forestal San Ramón, Río San Lorencito and tribs, 10°12'96"N, 84°36'42"W; NMNH; ♂]. —Bueno-Soria and Holzenthal, 2008:52 [distribution]. —Bueno-Soria, 2009:121 [♂].

**Distribution.** Costa Rica.

***spina*** Bueno-Soria and Holzenthal, 2004:254 [Type locality: Mexico, Veracruz, Puente Río Jamapa, Route Coscomatepec-Huatusco, el. 1320 m; CNIN; ♂]. —Bueno-Soria, 2009:156 [♂].

**Distribution.** Mexico.

***spinosissima*** Flint, 1964a:58 [Type locality: Puerto Rico, Toro Negro Forest, near Dona Juana area; NMNH; ♂]. —Flint, 1968b:59 [♂; ♀; distribution]. —Flint and Sykora, 1993:50 [checklist]. —Botosaneanu, 2002:87 [checklist]. —Botosaneanu and Thomas, 2005:44 [distribution].

**Distribution.** Dominica, Martinique, Puerto Rico.

***spinula*** Bueno-Soria and Holzenthal, 2004:255 [Type locality: Mexico, Chiapas: Reserva de Ia Biosfera El Triunfo; CNIN; ♂]. —Bueno-Soria, 2009:157 [♂]. —Bueno-Soria and Barba-Álvarez, 2011:358 [checklist].

**Distribution.** Mexico.

***spinulata*** Denning and Blickle, 1972:149 [Type locality: [United States], Ariz[ona], Portal, Cochise County; CAS; ♂]. —Baumgardner and Bowles, 2005:11 [distribution]. —Blinn and Ruiter, 2006:333 [biology]. —Bueno-Soria et al., 2007:33 [distribution]. —Bueno-Soria, 2009:146 [♂].

**Distribution.** Mexico, U.S.A.

***spinulosa*** Bueno-Soria, 2009:124 [Type locality: Mexico, Estado de México,Temascaltepec Real de Arriba, 19°02'24"N, 100°02'47” W, el. 1720 m; CNIN; ♂].

**Distribution.** Mexico.

***spira*** Thomson and Holzenthal, 2012:27 [Type locality: Venezuela, Monagas, Guachero Cave National Park, 10°10.322'N, 63°33.315'W, el. 1110 m; UMSP; ♂].

**Distribution.** Venezuela.

***stylata*** (Ross), 1938b:120 [Type locality: United States, Wyoming, Farson, Little Sandy Creek; INHS; ♂; in *Polytrichia*]. —Flint, 1972a:5 [♂; distribution]. —Bueno-Soria and Flint, 1978:204 [distribution]. —Blinn and Ruiter, 2006:333 [biology]. —Bowles et al., 2007:22 [distribution; biology]. —Bueno-Soria et al., 2007:33 [distribution]. —Bueno-Soria, 2009:139 [♂]. —Blinn and Ruiter, 2009a:303 [biology]. —Blinn and Ruiter, 2009b:186 [phenology, distribution].

**Distribution.** Guatemala, Mexico, U.S.A.

***tagala*** Flint, 1972a:8 [Type locality: Guatemala, Huehuetenango, 32 km NW Huehuetenango; NMNH; ♂]. —Maes and Flint, 1988:4 [distribution]. —Maes, 1999:1194 [checklist]. —Bueno-Soria et al., 2005:75 [distribution]. —Chamorro-Lacayo et al., 2007:43 [checklist]. —Bueno-Soria and Holzenthal, 2008:52 [distribution]. —Bueno-Soria, 2009:128 [♂; distribution].

**Distribution.** Costa Rica, Guatemala, Mexico, Nicaragua.

***tarsalis*** (Hagen), 1861:275 [Type locality: Canada, St. Lawrence River; MCZ; ♂; in *Hydroptila*]. —Ross, 1938a:10 [lectotype, in *Polytrichia*]. —Ross, 1944:130 [♂; ♀; in *Ochrotrichia*, distribution]. —Flint, 1972a:6 [♂; distribution]. —Bueno-Soria and Flint, 1978:204 [distribution]. —Baumgardner and Bowles, 2005:11 [distribution]. —Bueno-Soria et al., 2005:75 [distribution]. —Blinn and Ruiter, 2006:333 [biology]. —Bowles et al., 2007:22 [distribution; biology]. —Blinn and Ruiter, 2009a:304 [biology]. —Blinn and Ruiter, 2009b:186 [phenology, distribution]. —Bueno-Soria, 2009:139 [♂].

**Distribution.** Canada, Mexico, U.S.A.

***tenanga*** (Mosely), 1937:185 [Type locality: Mexico, Chiapas, Saltenango de la Paz; BMNH; ♂; in *Polytrichia*]. —Ross, 1944:276 [♂; in *Ochrotrichia*]. —Flint, 1972a:8 [♂; distribution]. —Bueno-Soria and Flint, 1978:204 [distribution]. —Flint, 1981a:29 [♂; distribution]. —Holzenthal, 1988c:62 [distribution]. —Flint and Reyes, 1991:487 [distribution]. —Aguila, 1992:538 [distribution]. —Bueno-Soria and Holzenthal, 2008:52 [distribution]. —Bueno-Soria, 2009:144 [♂]. —Bueno-Soria and Barba-Álvarez, 2011:358 [checklist]. —Armitage et al., 2015b:7 [checklist]. —Armitage and Cornejo, 2015:196 [checklist].

**Distribution.** Costa Rica, Guatemala, Honduras, Mexico, Panama, Peru, Venezuela.

***trinitatis*** Flint, 1996a:93 [Type locality: Trinidad, streamlet below Maracas Waterfall, 250m, 10°44'N, 61°24'W; NMNH; ♂]. —Botosaneanu, 1997:45 [♂; as undescribed species]. —Botosaneanu, 2002:87 [checklist].

**Distribution.** Trinidad.

***unica*** Bueno-Soria and Santiago-Fragoso, 1992:443 [Type locality: Colombia, Dept. Valle del Cauca, Rio Raposo; NMNH; ♂]. —Muñoz-Quesada, 2000:278 [checklist].

**Distribution.** Colombia.

***unicornia*** Bueno-Soria and Holzenthal, 2004:256 [Type locality: Mexico, Oaxaca, Santa Maria de Yavesia (water plant), 17°13'36"N, 96°25'35"W, el. 1930 m; CNIN; ♂]. —Bueno-Soria, 2009:122 [♂].

**Distribution.** Mexico.

***velascoi*** Bueno-Soria and Santiago-Fragoso, 1997:371 [Type locality: Mexico, Guerrero, route 134, 102 km N.W. Zihuatanejo; IBUNAM; ♂]. —Bueno-Soria, 2009:157 [♂].

**Distribution.** Mexico.

***verda*** Flint, 1968c:153 [Type locality: Puerto Rico, el Verde; NMNH; ♂]. —Botosaneanu, 2002:87 [checklist]. —Flint, 1968b:82 [checklist]. —Flint and Sykora, 2004:40 [distribution]. —Pérez-Gelabert, 2008:301 [checklist].

**Distribution.** Dominican Republic, Puerto Rico.

***vieja*** Bueno-Soria and Holzenthal, 1998:608 [Type locality: Costa Rica, Guanacaste, P.N. Rincón de la Vieja, Quebrada Provisión, 10°46'14"N, 85°16'86"W; NMNH; ♂]. —Bueno-Soria and Holzenthal, 2008:53 [distribution]. —Bueno-Soria, 2009:147 [♂].

**Distribution.** Costa Rica.

***villarenia*** Botosaneanu, 1980:108 [Type locality: Cuba, Prov. Las Villas, Cafetal << Gavina>>, La Sierrita; ZMUA; ♂]. —Kumanski, 1987:18 [distribution]. —Botosaneanu, 1979:48 [*nomen nudum* (name included in checklist); distribution]. —Flint, 1996c:16 [checklist]. —Botosaneanu, 2002:87 [checklist]. —Naranjo López and González Lazo, 2005:149 [checklist].

**Distribution.** Cuba.

***yanayacuana*** Bueno-Soria and Santiago-Fragoso, 1992:443 [Type locality: Ecuador, Tungurahua Prov., Yanayacu; NMNH; ♂].

**Distribution.** Ecuador.

***yavesia*** Bueno-Soria and Holzenthal, 2004:256 [Type locality: Mexico, Oaxaca, Santa Maria de Yavesia (water plant), 17°13'36"N, 96°25'35"W, el. 1930 m; CNIN; ♂]. —Bueno-Soria, 2009:158 [♂].

**Distribution.** Mexico.

***yepachica*** Harris, in Harris and Moulton, 1993:548 [Type locality: Mexico, Chihuahua, Río Concheno at Hwy 16, 12 km SW Yepachic; NMNH; ♂]. —Bueno-Soria, 2009:128 [♂].

**Distribution.** Mexico.

***yetla*** Bueno-Soria, 2009:129 [Type locality: Mexico, Oaxaca, San Mateo Yetla, 17°.75N, 96°.4W, el. 840 m; CNIN; ♂].

**Distribution.** Mexico.

***zihuaquia*** Bueno-Soria and Santiago-Fragoso, 1997:362 [Type locality: Mexico, Guerrero, route 134, 102 km N.W. Zihuatanejo; IBUNAM; ♂]. —Bueno-Soria, 2009:132 [♂].

**Distribution.** Mexico.

#### Genus *Orinocotrichia* Harris, Flint and Holzenthal [3]

*Orinocotrichia* Harris, Flint and Holzenthal, 2002a:50 [Type species: *Orinocotrichia calcariga* Harris, Flint and Holzenthal, 2002a, original designation].

*Orinocotrichia* was erected for a single species from the Río Cataniapo, Venezuela. More than a decade later, Oláh and Johanson (2011) described a second species from French Guiana. A third species was recently described from Brazil. *Orinocotrichia* is likely closely related to *Flintiella*, also a member of the Stactobiinae, according to Harris et al. (2002a). Immature stages are unkown.

***angelus*** Souza, Santos and Takiya, 2016b:338 [Type locality: Brazil, Maranhão, Carolina, Parque Nacional da Chapada das Mesas, Riacho Cancela, Malaise trap, 07°06'43.4"S, 47°17'16.6"W, el. 186 m; CZMA; ♂].

**Distribution.** Brazil.

***calcariga*** Harris, Flint and Holzenthal, 2002a:51 [Type locality: Venezuela, T. F. Amazonas, Río Cataniapo, 10 km S Puerto Ayacucho; NMNH; ♂; ♀].

**Distribution.** Venezuela.

***tagola*** Oláh and Johanson, 2011:251 [Type locality: French Guiana, Approuaguekaw, Kaw Mt, 4°32.833'N, 52°11.452'W, el. 77 m; NHRS; ♂].

**Distribution.** French Guiana.

#### Genus *Orthotrichia* Eaton [3]

*Orthotrichia* Eaton, 1873:141 [Type species: *Hydroptila angustella* McLachlan, 1865, original designation]. —Ross, 1944:139 [revision of North American species]. —Kingsolver and Ross, 1961:28 [revision of North American species].

In the Neotropics, this otherwise large, cosmopolitan genus is represented by only three species from the Greater Antilles, Nicaragua, and Peru. Otherwise, the genus is diverse in southeast Asia, Australia, and Africa.

Several species from outside the Neotropics are known in the larval stages (Nielsen 1948, Wells 1985, Wiggins 1996). They are typically found in aquatic vegetation and filamentous algae in standing waters, but Wells (1985) recorded some Australian species from rocks in flowing waters, unassociated with filamentous algae. An Australian species is parasitic within the pupal cocoons of some Hydropsychidae (Wells 1992), the only such behavior known in Trichoptera.

***aegerfasciella*** (Chambers), 1873:114 [Type locality: United States, Kentucky; type depository unknown; sex unknown; in *Clymene*]. —Ross, 1944:140 [♂; ♀; distribution; as *americana*]. —Bueno-Soria and Flint, 1978:201 [distribution]. —Botosaneanu, 1979:49 [distribution]. —Botosaneanu, 1991a:132 [distribution]. —Flint, 1996c:16 [checklist]. —Flint and Pérez-Gelabert, 1999:41 [checklist]. —Maes, 1999:1194 [checklist]. —Botosaneanu, 2002:87 [checklist]. —Flint and Sykora, 2004:40 [distribution]. —Naranjo López and González Lazo, 2005:149 [checklist]. —Bowles et al., 2007:22 [distribution; biology]. —Chamorro-Lacayo et al., 2007:43 [checklist]. —Pérez-Gelabert, 2008:301 [checklist]. —Flint, 2011:104 [checklist].

—*americana* Banks, 1904b:116 [Type locality: United States, Washington, D.C.; MCZ; ♂]. —Flint, 1966b:135 [to synonymy].

—*dorsalis* (Banks), 1904c:216 [Type locality: United States, Washington, D.C.; MCZ; ♀; in *Oxyethira*]. —Ross, 1944:140 [as synonym of *americana*].

—*brachiata* Morton, 1905:70 [Type locality: United States, New York, Ithaca; type depository unknown, CU?; ♂]. —Ross, 1938a:9 [as synonym of *americana*].

**Distribution.** Canada, Cuba, Dominican Republic, Haiti, Mexico, Nicaragua, U.S.A.

***cristata*** Morton, 1905:75 [Type locality: United States, Lake Forest, Illinois; type depository unknown, CU?; ♂]. —Ross, 1944:141 [♂; ♀; distribution]. —Flint, 1968a:45 [♂; ♀; distribution]. —Flint, 1968b:82 [checklist]. —Botosaneanu, 1979:49 [distribution]. —Angrisano, 1995b:509 [distribution]. —Flint, 1996c:16 [checklist]. —Botosaneanu and Hyslop, 1998:12 [distribution]. —Houghton and Stewart, 1998c:105 [biology]. —Angrisano, 1999:34 [checklist]. —Botosaneanu, 2002:87 [checklist]. —Flint and Sykora, 2004:40 [distribution]. —Naranjo López and González Lazo, 2005:149 [checklist]. —Bowles et al., 2007:22 [distribution; biology]. —Pérez-Gelabert, 2008:301 [checklist].

**Distribution.** Canada, Cuba, Dominican Republic, Jamaica, Uruguay, U.S.A.

***shimigaya*** Harris and Davenport, 1999:29 [Type locality: Peru, Loreto, small stream just outside grounds of Explorama Inn; NMNH; ♂].

**Distribution.** Peru.

#### Genus *Oxyethira* Eaton [94]

*Oxyethira* Eaton, 1873:143 [Type species: *Hydroptila costalis* Curtis 1834, original designation, a species of *Orthotrichia* according to Neboiss (1963). *Oxyethira costalis* (Curtis) sensu Eaton, 1873, is probably *Oxyethira flavicornis* (Pictet), 1834]. —Kelley, 1983:41 [new Neotropical species]. —Kelley, 1984:435 [revision]. —Holzenthal and Harris, 1992:155 [Costa Rican species].

*Lagenopsyche* Müller, 1879a:39 [Type species: *Lagenopsyche spirogyrae* Müller 1879a, subsequent selection of Fischer 1961:112]. —Müller, 1887b:338 [withdrawn in favor of *Oxyethira*]. —Kelley, 1984:436 [to synonymy].

*Argyrobothrus* Barnard, 1934:392 [Type species: *Argyrobothrus velocipes* Barnard 1934, by monotypy]. —Ross, 1948:202 [to synonymy]. —Kelley, 1984:438 [as subgenus].

*Loxotrichia* Mosely, 1937:165 [Type species: *Loxotrichia azteca* Mosely 1937, original designation]. —Ross, 1944:133 [to synonymy]. —Kelley, 1984:442 [as subgenus].

*Dampfitrichia* Mosely, 1937:169 [Type species: *Dampfitrichia ulmeri* Mosely 1937, by monotypy]. —Ross, 1944:133 [to synonymy]. —Kelley, 1984:438 [as subgenus].

*Oxytrichia* Mosely, 1939c:289 [Type species: *Oxyethira mirabilis* Morton 1904, original designation]. —Kimmins, 1966:114 [type species returned to *Oxyethira*, thus synonymizing genus]. —Kelley, 1984:440 [review, as subgenus].

*Mesotrichia* Kelley, 1984:458 [Type species: *Oxyethira jamaicensis* Flint 1968a, original designation, as subgenus]. —Özdikmen, 2007:444 [preoccupied by *Mesotrichia* Westwood, 1838, in Hymenoptera, Apidae].

*Tanytrichia* Kelley 1984:459 [Type species: *Oxyethira longissima* Flint 1974c, original designation, as subgenus].

*Dactylotrichia* Kelley, 1984:459 [Type species: *Oxyethira santiagensis* Flint 1982a, original designation, as subgenus].

*Kelleyella* Özdikmen, 2007:444 [Type species: *Oxyethira jamaicensis* Flint, 1968a; replacement name for *Mesotrichia* Kelley, 1984; as subgenus].

This cosmopolitan and diverse genus of Hydroptilinae contains almost 100 species in the Neotropics alone. In the New World, *Oxyethira* species occur in North, Central and South America (including the Chilean Subregion), and the Greater and Lesser Antilles. Kelley (1984) reviewed the genus and placed its included species in several subgenera, although some of the Neotropical species are unplaced to subgenus.

The distinctive larvae and their silken bottle-shaped cases are well known, but only for a few of the Neotropical species (Nielsen 1948, Wiggins 1996, Angrisano, 1995a). Larvae of the North American and European species are usually found in lentic waters among aquatic vascular plants and filamentous algae (Nielsen 1948, Wiggins 1996), but elsewhere they are also known from lotic waters (Wells 1985). They feed by puncturing the cells of filamentous algae and feeding on the contents, but diatoms and entire algal filaments have been found in the gut (Wiggins 1996).

***absona*** (unplaced) Flint, 1991:51 [Type locality: Colombia, Dpto. Antioquia, Quebrada La Cebolla, El Retiro; NMNH; ♂]. —Muñoz-Quesada, 2000:278 [checklist].

**Distribution.** Colombia.

***acegua*** (*Dactylotrichia*) Angrisano, 1995b:510 [Type locality; Uruguay, Cerro Largo, Sa. de Acegua; FHCU; ♂]. —Angrisano, 1999:34 [checklist].

**Distribution.** Uruguay.

***aculea*** (*Dampfitrichia*) Ross, 1941:53 [Type locality; United States, Oklahoma, Honey Creek, Turner Falls State Park; INHS; ♂]. —Bueno-Soria and Flint, 1978:204 [distribution]. —Kelley and Morse, 1982:266 [♀]. —Baumgardner and Bowles, 2005:11 [distribution]. —Blinn and Ruiter, 2006:333 [biology]. —Bowles et al., 2007:22 [distribution; biology]. —Bueno-Soria et al., 2007:33 [distribution].

**Distribution.** Mexico, U.S.A.

***alaluz*** (*Dampfitrichia*) Botosaneanu, 1980:112 [Type locality: Cuba, Jardin Botanique de Soledad, Cienfuegos, Prov. Las Villas; ZMUA; ♂]. —Botosaneanu, 1979:50 [*nomen nudum* (name included in checklist); distribution]. —Flint, 1996c:16 [checklist]. —Botosaneanu, 2002:87 [checklist]. —Naranjo López and González Lazo, 2005:149 [checklist].

**Distribution.** Cuba.

***albaeaquae*** (*Kelleyella*) Botosaneanu, 1995:30 [Type locality: Dominican Republic, Salto Aqua Blanca, Rio Grande, 3km from Convento; ZMUA; ♂; ♀]. —Flint and Pérez-Gelabert, 1999:41 [checklist]. —Botosaneanu, 2002:87 [checklist]. —Flint and Sykora, 2004:41 [distribution; to *Mesotrichia*]. —Pérez-Gelabert, 2008:301 [checklist].

**Distribution.** Dominican Republic.

***andina*** (*Oxytrichia*) Kelley, 1983:52 [Type locality: Argentina, Rio Negro Prov., Rio Guillelmo, Villa Mascardi; NMNH; ♂]. —Angrisano, 1999:34 [checklist].

**Distribution.** Argentina, Chile.

***apinolada*** (*Oxytrichia*) Holzenthal and Harris, 1992:157 [Type locality: Costa Rica, Guanacaste, Parque Nacional Rincón de la Vieja, Quebrada Agua Apinolada, 10.759°N, 85.292°W; NMNH; ♂].

**Distribution.** Costa Rica.

***arantala*** (*Oxytrichia*) Oláh and Johanson, 2011:130 [Type locality: Peru, San Martin Prov., creek crossing rd. Juan Guerra-Chazuta, 14 km (rd.) E Colombia Bridge, 6°35.594'S, 76°13.172'W; NHRS; ♂].

**Distribution.** Peru.

***arctodactyla*** (*Dactylotrichia*) Kelley, 1983:43 [Type locality: Venezuela, Merida State, Mucujun Valley, 19 km NE Merida; NMNH; ♂].

**Distribution.** Venezuela.

***argentinensis*** (*incertae sedis*) Flint, 1982a:45 [Type locality: Argentina, Pcia. Buenos Aires, Arroyo Pescado, Rt. 11, 15km E La Plata; NMNH; ♂]. —Flint, 1982c:42 [distribution]. —Kelley, 1984:442 [incertae sedis]. —Angrisano, 1995a:34 [larva]. —Angrisano, 1995b:510 [distribution]. —Mangeaud, 1996:154 [distribution]. —Angrisano, 1999:34 [checklist]. —Angrisano and Sganga, 2007:36 [♂; distribution; as *Oxyethira argentiniensis*].

**Distribution.** Argentina, Uruguay.

***arizona*** (*Dampfitrichia*) Ross, 1948a:202 [Type locality: United States, Arizona, Pinal County, Superior, in Boyce Thompson Arboretum; INHS; ♂]. —Bueno-Soria and Flint, 1978:204 [distribution]. —Kelley and Morse, 1982:263 [♀]. —Holzenthal, 1988c:62 [distribution; as *arizonica*]. —Botosaneanu, 1989a:101 [distribution]. —Holzenthal and Harris, 1992:172 [distribution]. —Flint and Sykora, 1993:49 [checklist, as *arizonensis*]. —Keiper and Walton, 1999:214 [larva; biology]. —Maes, 1999:1194 [checklist]. —Botosaneanu, 2002:87 [checklist]. —Botosaneanu and Thomas, 2005:44 [distribution]. —Naranjo López and González Lazo, 2005:149 [checklist]. —Blinn and Ruiter, 2006:333 [biology]. —Blinn and Ruiter, 2009a:305 [biology]. —Chamorro-Lacayo et al., 2007:43 [checklist]. —Armitage et al., 2015a:5 [distribution]. —Armitage et al., 2015b:7 [checklist]. —Armitage and Cornejo, 2015:196 [checklist].

**Distribution.** Costa Rica, Cuba, Dominica, Jamaica, Martinique, Mexico, Nicaragua, Panama, Puerto Rico, U.S.A.

***azteca*** (*Loxotrichia*) (Mosely), 1937:165 [Type locality: Mexico, Chiapas, Dolores; BMNH; ♂; in *Loxotrichia*]. —Flint, 1968b:55 [♂; ♀; distribution]. —Flint, 1974c:66 [♂; distribution]. —Bueno-Soria and Flint, 1978:205 [distribution]. —Flint, 1981a:30 [♂; distribution]. —Holzenthal, 1988c:62 [distribution]. —Flint and Reyes, 1991:488 [♂; female/; distribution]. —Holzenthal and Harris, 1992:172 [distribution]. —Aguila, 1992:539 [distribution]. —Botosaneanu and Sakal, 1992:202 [distribution]. —Botosaneanu and Alkins-Koo, 1993:27 [distribution]. —Flint and Sykora, 1993:57 [distribution]. —Flint, 1996a:99 [distribution]. —Flint, 1996b:401 [distribution]. —Muñoz-Quesada, 2000:278 [checklist]. —Botosaneanu, 2002:88 [checklist]. —Baumgardner and Bowles, 2005:11 [distribution]. —Bowles et al., 2007:22 [distribution; biology]. —Bueno-Soria et al., 2007:33 [distribution]. —Chamorro-Lacayo et al., 2007:43 [checklist]. —Bueno-Soria and Barba-Álvarez, 2011:358 [checklist]. —Oláh and Johanson, 2011:131 [distribution]. —Armitage et al., 2015b:7 [checklist]. —Armitage and Cornejo, 2015:196 [checklist].

**Distribution.** Belize, Colombia, Costa Rica, Ecuador, French Guiana, Grenada, Guatemala, Mexico, Nicaragua, Panama, Peru, Suriname, Tobago, Trinidad, Venezuela, U.S.A.

***baritu*** (*Dactylotrichia*) Angrisano, 1995a:30 [Type locality: Argentina, Salta, Parque Nacional Baritú; MACN; ♂]. —Angrisano, 1999:34 [checklist].

**Distribution.** Argentina.

***bettyae*** (*Tanytrichia*) Thomson and Holzenthal, 2012:29 [Type locality: Venezuela, Guárico, UCV San Nicolasito Field Station, 08°8.296'N, 66°24.459'W, el. 62 m; UMSP; ♂]. —Souza et al., 2013b:586 [distribution]. —Paprocki and França, 2014:51 [checklist].

**Distribution.** Brazil, Venezuela.

***bicornuta*** (*Tanytrichia*) Kelley, 1983:45 [Type locality: Brazil, Amazonas State, Igarape do Mendu, nr. Manaus; NMNH; ♂]. —Angrisano, 1999:34 [checklist]. —Paprocki et al., 2004:11 [checklist]. —Santos et al., 2009:36 [checklist]. —Paprocki and França, 2014:51 [checklist].

**Distribution.** Brazil.

***bidentata*** (*Oxytrichia*) Mosely, 1934b:155 [Type locality: Argentina, Lake Correntoso, Terr. Rio Negro; BMNH; ♂]. —Mosely, 1939c:289 [to *Oxytrichia*]. —Flint, 1974e:88 [checklist, to *Oxyethira*]. —Kelley, 1984:440 [to subgenus *Oxytrichia*]. —Angrisano, 1999:34 [checklist]. —Muzón et al., 2005: 57 [distribution]. —Miserendino and Brand, 2007:312 [biology]. —Brand and Miserendino, 2011a:35 [biology]. —Oláh and Johanson, 2011:132 [distribution]. —Brand et al., 2012:90 [biology]. —Brand and Miserendino, 2014:6 [community ecology].

**Distribution.** Argentina, Chile.

***brasiliensis*** (*incertae sedis*) Kelley, 1983:49 [Type locality: Brazil, Para State, Rio Cururu, area of Missao Cururu; NMNH; ♂]. —Angrisano, 1999:35 [checklist]. —Paprocki et al., 2004:11 [checklist]. —Santos et al., 2009:36 [checklist]. —Paprocki and França, 2014:51 [checklist].

**Distribution.** Brazil.

***campesina*** (*Dampfitrichia*) Botosaneanu, 1977:275 [Type locality: Cuba, Oriente, Baire, Rio Mogote; NMNH; ♂]. —Kumanski, 1987:26 [distribution]. —Botosaneanu, 1979:51 [distribution]. —Flint, 1996c:16 [checklist]. —Botosaneanu, 2002:87 [checklist]. —Naranjo López and González Lazo, 2005:149 [checklist].

**Distribution.** Cuba.

***circaverna*** (*Dampfitrichia*) Kelley, 1983:50 [Type locality: Panama, Canal Zone, Madden Dam; NMNH; ♂]. —Flint, 1992a:174 [distribution]. —Aguila, 1992:539 [distribution]. —Angrisano, 1995a:30 [larva; case; distribution]. —Angrisano, 1995b:510 [distribution]. —Angrisano, 1999:34 [checklist]. —Botosaneanu, 2002:87 [checklist]. —Angrisano and Sganga, 2007:34 [♂; distribution]. —Santos et al., 2009:42 [distribution]. —Manzo et al., 2014:166 [distribution]. —Paprocki and França, 2014:51 [checklist]. —Armitage et al., 2015b:7 [checklist]. —Armitage and Cornejo, 2015:196 [checklist].

**Distribution.** Argentina, Brazil, Curacao, Ecuador, Panama, Uruguay.

***cirrifera*** (*Dampfitrichia*) Flint, 1964a:57 [Type locality: Puerto Rico, Maricao, at fish hatchery; NMNH; ♂; ♀]. —Flint, 1968a:42 [♂; ♀; distribution]. —Flint, 1968b:38 [♂; ♀; distribution]. —Botosaneanu, 1979:50 [distribution]. —Kelley and Morse, 1982:258 [to synonymy of *Oxyethira arizona*]. —Kumanski, 1987:26 [♀; distribution]. —Botosaneanu, 1991a:130 [distribution]. —Flint, 1996c:16 [checklist]. —Botosaneanu and Hyslop, 1998:16 [resurrected]. —Flint and Pérez-Gelabert, 1999:41 [checklist]. —Flint and Sykora, 2004:43 [distribution]. —Naranjo López and González Lazo, 2005:149 [distribution; as synonym of *Oxyethira arizona*]. —Pérez-Gelabert, 2008:301 [checklist].

**Distribution.** Cuba, Dominica, Dominican Republic, Haiti, Puerto Rico.

***colombiensis*** (*Tanytrichia*) Kelley, 1983:44 [Type locality: Colombia, Valle Dept., Rio Raposo; NMNH; ♂]. —Muñoz-Quesada, 2000:278 [checklist].

**Distribution.** Colombia, Ecuador.

***copina*** (*Loxotrichia*) Angrisano, 1995a:32 [Type locality: Argentina, Cordoba, Copina; MACN; ♂; larva; case]. —Angrisano, 1999:34 [checklist].

**Distribution.** Argentina.

***costaricensis*** (*Dactylotrichia*) Kelley, 1983:44 [Type locality: Costa Rica, Heredia Prov., Los Cartagos; NMNH; ♂]. —Holzenthal, 1988c:62 [distribution].

**Distribution.** Costa Rica.

***cuernuda*** (*Tanytrichia*) Holzenthal and Harris, 1992:157 [Type locality: Costa Rica, Alajuela, Río Pizote, 5 km (air) S Brasilia, 10.972°N, 85.345°W; NMNH; ♂].

**Distribution.** Costa Rica.

***culebra*** (*Oxytrichia*) Holzenthal and Harris, 1992:160 [Type locality: Costa Rica, Alajuela, Río Pizote, 5 km (air) S Brasilia, 10.972°N, 85.345°W; NMNH; ♂].

**Distribution.** Costa Rica.

***dactylonedys*** (*Dactylotrichia*) Kelley, 1983:42 [Type locality: Paraguay, Amambay Dept., Rio Aquidaban, Cerro Cora; NMNH; ♂]. —Angrisano, 1999:34 [checklist].

**Distribution.** Paraguay.

***dalmeria*** (*Loxotrichia*) (Mosely), 1937:166 [Type locality: Mexico, Chiapas, Esmeralda; BMNH; ♂; in *Loxotrichia*]. —Bueno-Soria and Flint, 1978:205 [distribution]. —Bueno-Soria and Barba-Álvarez, 2011:358 [checklist].

**Distribution.** Mexico.

***desadorna*** (*Oxytrichia*) Moulton and Harris, 1997:499 [Type locality: Mexico, Nuevo Leon, Municipio de Santiago, spring along road above Cola de Caballo; NMNH; ♂]. —Bueno-Soria et al., 2007:33 [distribution].

**Distribution.** Mexico.

***discaelata*** (*Dampfitrichia*) Kelley, 1983:48 [Type locality: Venezuela, Bolivar State, Morichal Tauca, 22km E Rio Caura; NMNH; ♂]. —Angrisano, 1999:34 [checklist]. —Paprocki et al., 2004:11 [checklist]. —Santos et al., 2009:36 [checklist]. —Paprocki and França, 2014:51 [checklist].

**Distribution.** Brazil, Venezuela.

***espinada*** (*Tanytrichia*) Holzenthal and Harris, 1992:160 [Type locality: Costa Rica, Alajuela, Río Pizote, 5 km N Dos Ríos, 10.948°N, 85.291°W; NMNH; ♂]. —Blahnik et al., 2004:5 [distribution]. —Paprocki et al., 2004:12 [checklist]. —Santos et al., 2009:36 [checklist]. —Paprocki and França, 2014:51 [checklist].

**Distribution.** Brazil, Costa Rica.

***florida*** (*Dampfitrichia*) Denning, 1947c:12 [Type locality: United States, Florida, Miami; CAS; ♂]. —Botosaneanu, 1979:40, 51 [♂; ♀; distribution]. —Kelley and Morse, 1982:266 [♀]. —Flint, 1996c:16 [checklist]. —Botosaneanu, 2002:87 [checklist]. —Naranjo López and González Lazo, 2005:149 [checklist].

**Distribution.** Cuba, U.S.A.

***garifosa*** (*Dampfitrichia*) Moulton and Harris, 1997:496 [Type locality: Mexico, Tamaulipas, Municipio de Ciudad Victoria, Arroyo los Troncones, Ejido La Libertad, ca. 10 km NW Victoria; NMNH; ♂].

**Distribution.** Mexico.

***geminata*** (*Kelleyella*) Flint and Sykora, 2004:41 [Type locality: Dominican Republic, La Vega Province, 11.5 km S of Constanza (1 km N El Convento), 18°51.7'N, 70°41.0'W, el. 1410 m; NMNH; ♂; ♀]. —Pérez-Gelabert, 2008:301 [checklist].

**Distribution.** Dominican Republic.

***glasa*** (*Argyrobothrus*) (Ross), 1941:70 [Type locality: United States, Oklahoma, Honey Creek, Turner Falls State Park; INHS; ♂; in *Loxotrichia*]. —Botosaneanu, 1979:40, 50 [♂; distribution]. —Kelley and Morse, 1982:264 [♀]. —Holzenthal, 1988c:63 [distribution]. —Holzenthal and Harris, 1992:173 [distribution]. —Flint, 1996c:16 [checklist]. —Maes, 1999:1194 [checklist]. —Botosaneanu, 2002:87 [checklist]. —Naranjo López and González Lazo, 2005:149 [checklist]. —Chamorro-Lacayo et al., 2007:43 [checklist]. —Armitage et al., 2015a:5 [distribution]. —Armitage et al., 2015b:7 [checklist]. —Armitage and Cornejo, 2015:196 [checklist].

**Distribution.** Costa Rica, Cuba, Nicaragua, Panama, U.S.A.

***hilosa*** (*Tanytrichia*) Holzenthal and Harris, 1992:163 [Type locality: Costa Rica, Alajuela, Río Pizote, 5 km (air) S Brasilia, 10.972°N, 85.345°W; NMNH; ♂]. —Bueno-Soria, 1999:117 [distribution]. —Chamorro-Lacayo et al., 2007:43 [checklist]. —Armitage et al., 2015b:7 [checklist]. —Armitage and Cornejo, 2015:196 [checklist].

**Distribution.** Costa Rica, Mexico, Nicaragua, Panama.

***hozosa*** (unplaced) Harris and Davenport, 1999:35 [Type locality: Peru, Loreto, Rio Yanamono just below Explorama Lodge; NMNH; ♂].

**Distribution.** Peru.

***hyalina*** (*incertae sedis*) Müller, 1879b:143 [Type locality: Brazil, Santa Catarina; MNRJ; larval case; in *Lagenopsyche*]. —Ulmer 1905d:74 [to *Oxyethira*]. —Ulmer, 1957:172 [bibliography]. —Kelley, 1984:442 [incertae sedis]. —Angrisano, 1999:35 [checklist]. —Paprocki et al., 2004:12 [checklist]. —Santos et al., 2009:36 [checklist]. —Paprocki and França, 2014:52 [checklist].

**Distribution.** Brazil.

***inaequispina*** (*Oxytrichia*) Flint, 1990:118 [Type locality: Chile, Prov. El Loa, brook of Toconao; IRSNB; ♂]. —Angrisano, 1999:34 [checklist].

**Distribution.** Chile.

***jamaicensis*** (*Kelleyella*) Flint, 1968a:44 [Type locality: Jamaica, St. Andrew, Hope River near Newcastle at mile post 16.5; NMNH; ♂; ♀]. —Flint, 1968b:82 [checklist]. —Botosaneanu, 2002:89 [checklist].

**Distribution.** Jamaica.

***janella*** (*Loxotrichia*) Denning, 1948a:397 [Type locality: United States, Florida, Winter Park; CAS; ♂]. —Flint, 1968a:42 [♂; ♀; distribution]. —Flint, 1968b:52 [♂; ♀; distribution]. —Bueno-Soria and Flint, 1978:205 [distribution]. —Botosaneanu, 1979:50 [distribution]. —Kelley and Morse, 1982:261 [♀]. —Malicky, 1983:264 [distribution]. —Kumanski, 1987:27 [distribution]. —Holzenthal, 1988c:63 [distribution]. —Botosaneanu, 1989a:101 [distribution]. —Botosaneanu, 1990a:47 [distribution]. —Botosaneanu, 1991a:132 [distribution]. —Holzenthal and Harris, 1992:173 [distribution]. —Aguila, 1992:539 [distribution]. —Flint and Sykora, 1993:57 [distribution]. —Botosaneanu, 1994a:42 [distribution]. —Botosaneanu, 1995:32 [distribution]. —Flint, 1996a:98 [distribution]. —Flint, 1996c:16 [checklist]. —Botosaneanu and Hyslop, 1998:16 [distribution]. —Flint and Pérez-Gelabert, 1999:41 [checklist]. —Botosaneanu, 2000:256 [distribution]. —Botosaneanu, 2002:88 [checklist]. —Flint and Sykora, 2004:43 [distribution]. —Botosaneanu and Thomas, 2005:55 [checklist]. —Naranjo López and González Lazo, 2005:149 [checklist]. —Bowles et al., 2007:22 [distribution; biology]. —Pérez-Gelabert, 2008:301 [checklist].

—*neglecta* (*Loxotrichia*) Flint, 1964a:57 [Type locality: Puerto Rico, Maricao, fish hatchery; NMNH; ♂; ♀]. —Flint, 1968a:42 [to synonymy].

**Distribution.** Barbados [?], Costa Rica [?], Cuba, Dominica, Dominican Republic, Grenada, Guadeloupe, Haiti, Jamaica, Martinique [?], Mexico, Panama [?], Puerto Rico, St. Lucia, St. Vincent, U.S.A. [records from Central America likely *Oxyethira tica*].

***kerek*** (*Dactylotrichia*) Oláh and Johanson, 2011:132 [Type locality: Peru, San Martin Prov., Rio Negro, 37 km (rd.) W Moyobamba, near Olmos-Tarapoto rd., 6°00.278'S, 77°15.437'W; NHRS; ♂].

**Distribution.** Peru.

***lagunita*** (*Dampfitrichia*) Flint, 1980b:142 [Type locality: Argentina, Pcia. Entre Rios, Arroyo P. Verne, 4 km N Villa San José; NMNH; ♂]. —Flint, 1982c:42 [distribution]. —Kelley, 1984:440 [distribution]. —Angrisano, 1995b:510 [distribution]. —Mangeaud, 1996:154 [distribution]. —Angrisano, 1999:34 [checklist]. —Paprocki et al., 2004:12 [checklist]. —Angrisano and Sganga, 2007:36 [♂; distribution]. —Santos et al., 2009:36 [checklist]. —Paprocki and França, 2014:52 [checklist].

**Distribution.** Argentina, Brazil, Uruguay.

***longipenis*** (unplaced) Santos, Henriques-Oliveira and Nessimian, 2009:40 [Type locality: Brazil, Amazonas, Manaus, tributary to Rio Cuieiras, 02°42'25.1"S, 60°22'28.2"W; INPA; ♂; not placed in subgenus, but compared with species in Oxyethira (Oxitrichia)]. —Paprocki and França, 2014:52 [checklist].

**Distribution.** Brazil.

***longispinosa*** (*Dampfitrichia*) Kumanski, 1987:29 [Type locality: Cuba, Province Pinar del Rio, Rio El Ballio near Isabel Rubio village, or Rio Esmeralda near Vinales; NMSB, ♂]. —Flint, 1996c:16 [checklist]. —Botosaneanu and Hyslop, 1998:16 [distribution; as *mirebalina* or *longispinosa*]. —Botosaneanu, 2002:87 [checklist].

**Distribution.** Cuba, Jamaica[?].

***longissima*** (*Tanytrichia*) Flint, 1974c:66 [Type locality: Suriname, Republiek; RNH; ♂]. —Santos et al. 2009:42 [distribution]. —Paprocki and França, 2014:52 [checklist].

**Distribution.** Brazil, Suriname.

***luanae*** (*Tanytrichia*) Santos, Henriques-Oliveira and Nessimian, 2009:37 [Type locality: Brazil, Amazonas, Manaus, tributary to Igarapé da Cachoeira, basin of Rio Cuieiras, 02°41'46.0"S, 60°17'42.7"W; INPA; ♂]. —Paprocki and França, 2014:52 [checklist].

**Distribution.** Brazil.

***macrosterna*** (*Tanytrichia*) Flint, 1974c:67 [Type locality: Suriname, Nickerie River, Blanche Marie, falls in creek; RNH; ♂]. —Angrisano, 1999:34 [checklist]. —Santos et al., 2009:42 [distribution]. —Oláh and Johanson, 2011:134 [distribution]. —Paprocki and França, 2014:52 [checklist].

**Distribution.** Brazil, French Guiana, Suriname.

***maryae*** (*Oxytrichia*) Kelley, 1983:53 [Type locality: Colombia, Meta Dept, Refugio Macarena; NMNH; ♂]. —Muñoz-Quesada, 2000:278 [checklist].

**Distribution.** Colombia.

***matadero*** (*Dactylotrichia*) Harper and Turcotte, 1985:138 [Type locality: Ecuador, small stream, outlet of Laguna Verde Cocha, near junction with Rio Matadero, Chirimachay, Quinuas Valley; UMQ; ♂].

**Distribution.** Ecuador.

***maya*** (*Dampfitrichia*) Denning, 1947c:16 [Type locality: United States, Georgia, Macon; CAS; ♂]. —Kelley and Morse, 1982:263 [♀]. —Kelley, 1984:440 [distribution]. —Flint et al., 2003:34 [♀; distribution; introduced to Hawaii]. —Bowles et al., 2007:22 [distribution; biology]. —Armitage et al., 2015a:6 [distribution]. —Armitage et al., 2015b:7 [checklist]. —Armitage and Cornejo, 2015:196 [checklist].

**Distribution.** Mexico, Panama, U.S.A.

***merga*** (*Tanytrichia*) Kelley, 1983:45 [Type locality: Venezuela, Bolivar State, Rio Cuyuni, El Dorado; NMNH; ♂]. —Flint, 1992d:70 [distribution]. —Angrisano, 1999:34 [checklist]. —Paprocki et al., 2004:12 [checklist]. —Santos et al., 2009:36 [checklist]. —Paprocki and França, 2014:52 [checklist].

**Distribution.** Brazil, Venezuela.

***mirebalina*** (*Dampfitrichia*) Botosaneanu, 1991a:130 [Type locality: Haiti, Département de l’Ouest, Grande rivière d l’Artibonite à Mirebalais; ZMUA; ♂; ♀]. —Botosaneanu, 1995:29 [distribution]. —Botosaneanu and Hyslop, 1998:16 [distribution; as *mirebalina* or *longispinosa*]. —Flint and Pérez-Gelabert, 1999:41 [checklist]. —Botosaneanu, 2002:87 [checklist]. —Flint and Sykora, 2004:41 [distribution; ♀ allotype is actually *Oxyethira simulatrix*]. —Pérez-Gelabert, 2008:301 [checklist].

**Distribution.** Dominican Republic, Haiti, Jamaica [?].

***misionensis*** (*Dactylotrichia*) Angrisano, 1995a:30 [Type locality: Argentina, Misiones, Posadas; MACN; ♂]. —Angrisano, 1999:34 [checklist].

**Distribution.** Argentina.

***mocoi*** (unplaced) Angrisano, 1995a:34 [Type locality: Argentina, Entre Rios, Parque Nacional El Palmar; MACN; ♂]. —Angrisano, 1999:35 [checklist]. —Angrisano and Sganga, 2007:38 [♂; distribution].

**Distribution.** Argentina.

***nyultka*** (*Tanytrichia*) Oláh and Johanson, 2011:134 [Type locality: French Guiana, Approuaguekaw, Kaw Mt, 4°32.833'N, 52° 11.452'W; NHRS; ♂].

**Distribution.** French Guiana.

***obscura*** (*Oxytrichia*) Flint, 1974c:69 [Type locality: Suriname, Suriname River, Botopasie; RNH; ♂]. —Angrisano, 1995b:510 [distribution]. —Angrisano, 1999:34 [checklist].

**Distribution.** Suriname, Uruguay.

***orellanai*** (*Tanytrichia*) Harris and Davenport, 1992:465 [Type locality: Peru, Loreto, Rio Sucusari just up stream from Explornapo Camp; NMNH; ♂].

**Distribution.** Peru.

***ortizorum*** (*Kelleyella*) Botosaneanu, 1995:29 [Type locality: Dominican Republic, Arroyo el Dulce, sección Manavao-Los Dajaos of Jarabacoa; ZMUA; ♂]. —Botosaneanu, 2002:88 [checklist]. —Flint and Pérez-Gelabert, 1999:41 [checklist]. —Flint and Sykora, 2004:41 [distribution; to subgenus *Mesotrichia*]. —Pérez-Gelabert, 2008:301 [checklist].

**Distribution.** Dominican Republic.

***parazteca*** (*Loxotrichia*) Kelley, 1983:53 [Type locality: Ecuador, Cotopaxi Prov., 133km W Latacunga; NMNH; ♂]. —Holzenthal, 1988c:63 [distribution]. —Holzenthal and Harris, 1992:173 [distribution].

**Distribution.** Costa Rica, Ecuador.

***parce*** (*Loxotrichia*) (Edwards and Arnold), 1961:405 [Type locality: United States, Texas, Caldwell Co., San Marcos River; type destroyed; ♂; in *Protoptila*]. —Flint, 1991:51 [♂; distribution]. —Flint and Reyes, 1991:487 [♂; ♀; distribution]. —Holzenthal and Harris, 1992:173 [distribution]. —Flint, 1996a:99 [distribution]. —Mangeaud, 1996:154 [distribution]. —Angrisano, 1999:34 [checklist]. —Muñoz-Quesada, 2000:278 [checklist]. —Botosaneanu, 2002:88 [checklist]. —Botosaneanu and Viloria, 2002:108 [distribution]. —Blahnik et al. 2004:5 [distribution]. —Paprocki et al., 2004:12 [checklist]. —Muzón et al., 2005: 57 [distribution]. —Santos et al., 2009:36 [checklist]. —Rueda Martín, 2011:9 [♂; distribution]. —Paprocki and França, 2014:52 [checklist]. —Isa Miranda and Rueda Martín, 2014:199 [distribution]. —Armitage et al., 2015b:7 [checklist]. —Armitage and Cornejo, 2015:196 [checklist].

**Distribution.** Argentina, Bolivia, Brazil, Colombia, Costa Rica, Ecuador, Guyana, Mexico, Panama, Peru, Trinidad, Venezuela, U.S.A.

***paritentacula*** (*Tanytrichia*) Kelley, 1983:45 [Type locality: Belize, Cayo Dist., Rio Privassion, Blancaneaux Lodge; NMNH; ♂].

**Distribution.** Belize.

***peruviana*** (unplaced) Harris and Davenport, 1999:33 [Type locality: Peru, Loreto, tributary to Rio Yanamono at Explorama Lodge; NMNH; ♂]. —Santos et al., 2009:43 [distribution]. —Paprocki and França, 2014:53 [checklist].

**Distribution.** Brazil, Peru.

***petei*** (*Oxytrichia*) Angrisano, 1995a:29 [Type locality: Argentina, Entre Rios, Parque Nacional el Palmar; MACN; ♂]. —Angrisano, 1999:34 [checklist]. —Angrisano and Sganga, 2007:36 [♂; distribution].

**Distribution.** Argentina.

***picita*** (unplaced) Harris and Davenport, 1999:35 [Type locality: Peru, Loreto, edge of Rio Sucusari backwater, adjoining Explornapo Camp; NMNH; ♂]. —Santos et al., 2009:43 [distribution]. —Thomson and Holzenthal, 2012:29 [distribution]. —Paprocki and França, 2014:53 [checklist].

**Distribution.** Brazil, Peru, Venezuela.

***poapi*** (unplaced) Angrisano and Sganga, 2009:67 [Type locality: Argentina, Misiones, Parque Provincial Salto Encantado; MACN; ♂].

**Distribution.** Argentina.

***presilla*** (unplaced) Harris and Davenport, 1999:29 [Type locality: Peru, Loreto, Yanamono Creek at jungle’s edge, near Explorama Lodge; NMNH; ♂]. —Santos et al., 2009:43 [distribution]. —Paprocki and França, 2014:53 [checklist].

**Distribution.** Brazil, Peru.

***puertoricensis*** (*Loxotrichia*) Flint, 1964a:55 [Type locality: Puerto Rico, Maricao, at fish hatchery; NMNH; ♂; ♀; larva; case]. —Flint, 1968a:40 [distribution]. —Flint, 1968b:82 [checklist]. —Botosaneanu, 1991a:132 [distrubution]. —Botosaneanu, 1995:32 [distribution]. —Flint, 1996c:16 [checklist, as *quelinda*]. —Botosaneanu and Hyslop, 1998:17 [distribution]. —Flint and Pérez-Gelabert, 1999:41 [checklist]. —Botosaneanu, 2002:88 [checklist]. —Flint and Sykora, 2004:44 [distribution]. —Naranjo López and González Lazo, 2005:149 [checklist]. —Pérez-Gelabert, 2008:301 [checklist].

—*quelinda* (*Loxotrichia*) (Botosaneanu), 1977:267 [Type locality: Cuba, Oriente, Baracoa, Rio Sabanilla; NMNH; ♂; in *Loxotrichia*]. —Botosaneanu, 1979:50 [distribution]. —Botosaneanu, 1995:32 [to synonymy].

**Distribution.** Cuba, Dominican Republic, Haiti, Jamaica, Puerto Rico.

***quinquaginta*** (*incertae sedis*) Kelley, 1983:54 [Type locality: Ecuador, Pastaza Prov., Puyo; NMNH; ♂].

**Distribution.** Ecuador.

***quiramae*** (*Dactylotrichia*) Thomson and Holzenthal, 2012:31 [Type locality: Venezuela, Guárico, UCV San Nicolasito Field Station, 08°8.296'N, 66°24.459'W, el. 62 m; UMSP; ♂].

**Distribution.** Venezuela.

***rareza*** (unplaced) Holzenthal and Harris, 1992:163 [Type locality Costa Rica, Alajuela, Río Pizote, 5 km N Dos Ríos, 10.948°N, 85.291°W; NMNH; ♂].

**Distribution.** Costa Rica.

***redunca*** (unplaced) Thomson and Holzenthal, 2012:33 [Type locality: Venezuela, Bolívar, Gran Sabana, E. Pauji, “Río Curvita”, 4°31.237'N, 61°31.591'W, el. 869 m; UMSP; ♂].

**Distribution.** Venezuela.

***ritae*** (*Loxotrichia*) Angrisano, 1995b:510 [Type locality: Uruguay, Paysandu, Sta. Rita; FHCU; ♂]. —Angrisano, 1999:34 [checklist].

**Distribution.** Uruguay.

***santiagensis*** (*Dactylotrichia*) Flint, 1982a:46 [Type locality: Argentina, Pcia. Buenos Aires, Rio Santiago, Palo Blanco, Berisso; NMNH; ♂]. —Flint, 1982c:43 [distribution]. —Kelley, 1984:442 [distribution]. —Angrisano, 1995b:511 [distribution]. —Angrisano, 1999:34 [checklist]. —Paprocki et al., 2004:12 [checklist]. —Santos et al., 2009:37 [checklist]. —Paprocki and França, 2014:53 [checklist].

**Distribution.** Argentina, Brazil, Uruguay.

***scaeodactyla*** (*Dactylotrichia*) Kelley, 1983:42 [Type locality: Ecuador, Pastaza Prov., Puyo; NMNH; ♂].

**Distribution.** Ecuador.

***scopulina*** (*Kelleyella*) Flint and Sykora, 2004:43 [Type locality: Dominican Republic, Peravia Province, 3 km SW La Nuez, upper Rio Las Cuevas, 18°40'N, 70°36'W, el. 1850 m; CMNH; ♂; ♀]. —Pérez-Gelabert, 2008:301 [checklist].

**Distribution.** Dominican Republic.

***sencilla*** (*Tanytrichia*) Holzenthal and Harris, 1992:165 [Type locality: Costa Rica, Alajuela, Río Pizote, 5 km N Dos Ríos, 10.948°N, 85.291°W; NMNH; ♂].

**Distribution.** Costa Rica.

***sierruca*** (unplaced) Holzenthal and Harris, 1992:165 [Type locality: Costa Rica, Guanacaste, Quebrada Garcia, 10.6 km ENE Quebrada Grande, 10.862°N, 85.428°W; NMNH; ♂]. —Armitage et al., 2015a:6 [distribution]. —Armitage et al., 2015b:7 [checklist]. —Armitage and Cornejo, 2015:196 [checklist].

**Distribution.** Costa Rica, Panama.

***simanka*** (unplaced) Oláh and Johanson, 2011:135 [Type locality: Ecuador, Wild Sumaco, near Pacto Sumaco; OPC; ♂].

**Distribution.** Ecuador.

***simulatrix*** (*Dampfitrichia*) Flint, 1968a:43 [Type locality: Jamaica, St. Andrew, Fresh River, Ferry; NMNH; ♂; ♀]. —Flint, 1968b:82 [checklist]. —Holzenthal, 1988c:63 [distribution]. —Holzenthal and Harris, 1992:174 [distribution]. —Flint, 1996c:16 [checklist]. —Botosaneanu and Hyslop, 1998:16 [distribution]. —Maes, 1999:1194 [checklist]. —Botosaneanu, 2002:88 [checklist]. —Flint and Sykora, 2004:44 [distribution taxonomic, remarks]. —Chamorro-Lacayo et al., 2007:44 [checklist]. —Armitage et al., 2015a:6 [distribution]. —Armitage et al., 2015b:7 [checklist]. —Armitage and Cornejo, 2015:196 [checklist].

—*simulatrix cubana* Kumanski, 1987:27 [Type locality: Cuba, Province Pinar del Rio, Rio El Ballio, near Isabel Rubio village, or Rio Esmeralda in the vicinity of Viñales; NMSB; ♂; ♀; as *Orthotrichia* sp.]. —Botosaneanu, 1991a:130 [distribution; ♀ attributed is undoubtedly *tega*]. —Botosaneanu and Hyslop, 1998:16 [distribution]. —Flint and Pérez-Gelabert, 1999:41 [checklist]. —Botosaneanu, 2002:88 [checklist]. —Flint and Sykora, 2004:44 [to synonymy]. —Botosaneanu and Thomas, 2005:55 [checklist]. —Naranjo López and González Lazo, 2005:149 [checklist]. —Pérez-Gelabert, 2008:301 [checklist].

—*mirebalina* Botosaneanu, 1991a:130, part [Type locality: Haiti; ♀ allotype, misidentification of *Oxyethira simulatrix* sensu Flint and Sykora, 2004].

**Distribution.** Costa Rica, Cuba, Dominican Republic, Guadeloupe, Haiti, Jamaica, Mexico, Nicaragua, Panama, U.S.A.

***sinistra*** (unplaced) Santos, Henriques-Oliveira and Nessimian, 2009:40 [Type locality: Brazil, Amazonas, Manaus, Igarapé Arumã, tributary to Rio Cuieiras, 02°30'55.2"S, 60°15'44.4"W; INPA; ♂]. —Paprocki and França, 2014:53 [checklist].

**Distribution.** Brazil.

***spirogyrae*** (*incertae sedis*) Müller, 1879b:143 [Type locality: Brazil, Santa Catarina; MNRJ; case; larva; in *Lagenopsyche*]. —Müller, 1887b:339 [to *Oxyethira*]. —Ulmer, 1957:172 [bibliography]. —Kelley, 1984:442 [*incertae sedis*]. —Angrisano, 1999:35 [checklist]. —Paprocki et al., 2004:12 [checklist]. —Santos et al., 2009:37 [checklist]. —Paprocki and França, 2014:53 [checklist].

**Distribution.** Brazil.

***spissa*** (*incertae sedis*) Kelley, 1983:48 [Type locality: Brazil, Pará State, Rio Cururu, area of Missao Cururu; NMNH; ♂]. —Angrisano, 1999:35 [checklist]. —Paprocki et al., 2004:12 [checklist]. —Santos et al., 2009:37 [checklist]. —Paprocki and França, 2014:53 [checklist].

**Distribution.** Brazil.

***tamandua*** (unplaced) Angrisano and Sganga, 2009:65 [Type locality: Argentina, Misiones, Parque Provincial Salto Encantado; MACN; ♂].

**Distribution.** Argentina.

***tega antillularum*** (*Dampfitrichia*) Botosaneanu, 1994a:41 [Type locality: Guadeloupe, rivière St-Louis dans les Hauts du Matouba: ZMUA; ♂]. —Flint and Sykora, 1993:57 [distribution; as *tega*]. —Botosaneanu, 2002:88 [checklist]. —Botosaneanu and Thomas, 2005:55 [checklist].

—*Orthotrichia* sp. Malicky, 1983:265 [♀; misidentification of *tega*, *vide* Flint and Sykora, 1993:57].

**Distribution.** Dominica, Guadeloupe.

***tega tega*** (*Dampfitrichia*) Flint, 1968a:44 [Type locality: Jamaica, Trelawny, Martha Brae, near Falmouth; NMNH; ♂; ♀]. —Flint, 1968b:56 [distribution]. —Botosaneanu, 1977:273 [variability; distribution]. —Botosaneanu, 1979:50 [distribution]. —Malicky, 1983:264 [distribution]. —Flint, 1996c:16 [checklist]. —Botosaneanu and Hyslop, 1998:16 [distribution]. —Botosaneanu, 2002:88 [checklist]. —Flint and Sykora, 2004:45 [distribution; taxonomic remarks]. —Naranjo López and González Lazo, 2005:149 [checklist]. —Pérez-Gelabert, 2008:301 [checklist].

**Distribution.** Cuba, Jamaica, Dominica, Haiti, Hispaniola.

***teixeirai*** (*Tanytrichia*) Harris and Davenport, 1992:470 [Type locality: Peru, Loreto, small tributary to the Rio Sucusari at Explornapo Camp; NMNH; ♂].

**Distribution.** Peru.

***tica*** (*Loxotrichia*) Holzenthal and Harris, 1992:168 [Type locality: Costa Rica, Guanacaste, Parque Nacional Santa Rosa, Quebrada El Duende near La Casona, 10.838°N, 85.614°W; NMNH; ♂; ♀]. —Botosaneanu and Sakal, 1992:202 [distribution; ecology]. —Botosaneanu and Alkins-Koo, 1993:27 [♂; distribution]. —Botosaneanu, 1994a:43 [distribution]. —Flint, 1996a:98 [distribution]. —Botosaneanu, 2000:256 [distribution]. —Botosaneanu, 2002:88 [checklist]. —Blahnik et al., 2004:5 [distribution]. —Paprocki et al., 2004:12 [checklist]. —Botosaneanu and Thomas, 2005:44 [distribution]. —Chamorro-Lacayo et al., 2007:44 [checklist]. —Dumas et al., 2009:366 [distribution]. —Santos et al., 2009:43 [distribution]. —Oláh and Johanson, 2011:137 [distribution]. —Dumas and Nessimian, 2012:15 [checklist]. —Paprocki and França, 2014:53 [checklist]. —Armitage et al., 2015b:7 [checklist]. —Armitage and Cornejo, 2015:196 [checklist].

**Distribution.** Brazil, Costa Rica, Dominica, Ecuador, French Guiana, Grenada, Guadeloupe, Honduras, Martinique, Mexico, Nicaragua, Panama, St. Lucia, St. Vincent, Trinidad, Venezuela.

***torza*** (unplaced) Oláh and Johanson, 2011:137 [Type locality: French Guiana, Roura, Cacao, 4°33.639'N, 52°24.629'W, el. 66 m; NHRS; ♂].

**Distribution.** French Guiana.

***tuveva*** (*Tanytrichia*) Oláh and Johanson, 2011:138 [Type locality: French Guiana, Approuaguekaw, Kaw Mt, 4°32.833'N, 52°11.452'W, el. 77 m; NHRS; ♂].

**Distribution.** French Guiana.

***ulmeri*** (*Dampfitrichia*) (Mosely), 1937:169 [Type locality: Mexico, Chiapas, Dolores; BMNH; ♂; in *Dampfitrichia*]. —Bueno-Soria and Flint, 1978:205 [distribution]. —Kelley and Morse, 1982:267 [♀]. —Angrisano, 1995b:510 [distribution]. —Angrisano, 1999:34 [distribution—Bowles et al., 2007:22 [distribution; biology]. —Bueno-Soria et al., 2007:33 [distribution]. —Bueno-Soria and Barba-Álvarez, 2011:358 [checklist]. —Rueda Martín, 2011:9 [♂; distribution]. —Isa Miranda and Rueda Martín, 2014:199 [distribution].

**Distribution.** Argentina, Mexico, Uruguay, U.S.A.

***unispina*** (*Oxytrichia*) Flint, 1974c:67 [Type locality: Suriname, Republiek; RNH; ♂]. —Oláh and Johanson, 2011:140 [taxonomic remarks; under sugenus *Dampfitrichia*].

**Distribution.** Suriname.

***vaina*** (unplaced) Harris and Davenport, 1999:33 [Type locality: Peru, Loreto, edge of Rio Sucusari backwater, adjoining Explornapo Camp; NMNH; ♂].

**Distribution.** Peru.

***vaza*** (*Dampfitrichia*) Oláh and Johanson, 2011:140 [Type locality: French Guiana, Roura, Cacao, 4°33.639'N, 52°24.629'W, el. 66 m; NHRS; ♂].

**Distribution.** French Guiana.

***vipera*** (*Oxytrichia*) Kelley, 1983:50 [Type locality: Chile, Valdavia Prov., S of Valdavia; NMNH; ♂]. —Angrisano, 1999:34 [checklist]. —Oláh and Johanson, 2011:141 [distribution].

**Distribution.** Chile.

***zilaba*** (*Loxotrichia*) (Mosely), 1939a:238 [Type locality: Brazil, Edo. Santa Catarina, Nova Teutonia; BMNH; ♂; in *Loxotrichia*]. —Angrisano, 1995b:510 [distribution]. —Angrisano, 1999:34 [checklist]. —Blahnik et al., 2004:5 [distribution]. —Paprocki et al., 2004:12 [checklist]. —Angrisano and Sganga, 2007:36 [♂; distribution]. —Santos et al. 2009:37 [checklist]. —Calor, 2011:321 [checklist]. —Paprocki and França, 2014:54 [checklist].

**Distribution.** Argentina, Brazil, Paraguay, Uruguay.

#### Genus *Peltopsyche* Müller [6]

*Peltopsyche* Müller, 1879b:144 [Type species: *Peltopsyche sieboldii* Müller, 1879b, subsequent selection of Fischer, 1961]. —Ulmer, 1957:172 [bibliography, discussion]. —Flint et al., 1999b:118 [discussion].

*Abtrichia* Mosely, 1939a:224 [Type species: *Abtrichia antennata* Mosely 1939, original designation]. —Santos et al., 2016a:472 [to synonymy].

The genus was established on the basis of two species collected by Müller from the Province of Santa Catarina in southern Brazil. Only a few larval features and basal antennal segments of the males were figured. Santos et al. (2016) synonymized *Abtrichia* with *Peltopsyche* based on larvae, pupae, and pharate adults and matching the figures and features provided by Müller.

Larvae of *Peltopsyche antennata* (as *Abtrichia*) have been described by Flint (1972b) and are distinguished by the papillae on the head. They are found in small and medium sized, fast-flowing streams and rivers, on rocks and boulders. They probably feed on organic matter they scrape from the substrate adjacent to their cases. Angrisano (2002) described the pupa of *Peltopsyche antennata* (as *Abtrichia antennata*).

***antennata*** (Mosely), 1939a:227 [Type locality: Brazil, Edo. Santa Catarina, Nova Teutonia; BMNH; ♂; in *Abtrichia*]. —Flint, 1972b:233 [♂; larva; distribution; in *Abtrichia*]. —Angrisano, 1995b:505 [distribution]. —Angrisano, 1999:31 [checklist]. —Angrisano, 2002:406 [pupa]. —Blahnik et al., 2004:4 [distribution]. —Paprocki et al., 2004:10 [checklist]. —Angrisano and Sganga, 2007:29 [♂; larva; pupa; distribution]. —Dumas et al., 2010:8 [distribution]. —Oláh and Flint, 2012:138 [distribution]. —Souza et al., 2013b:585 [distribution]. —Paprocki and França, 2014:39 [checklist]. —Santos et al., 2016a:472 [to *Peltopsyche*].

**Distribution.** Argentina, Brazil, Uruguay.

***epara*** (Oláh and Flint), 2012:139 [Type locality: Argentina, Tucumán Province, South of Concepción; NMNH; /male; in *Abtrichia*]. —Santos et al., 2016a:472 [to *Peltopsyche*].

**Distribution.** Argentina.

***sieboldii*** Müller, 1879b:144 [Type locality: Brazil, Santa Catarina, Garcia, Encano, and Warnow Rivers, tributaries of the Itajahy River; MNRJ (cases only); case; ♂; antenna; larva]. —Müller, 1880a:133 [larval case]. —Müller, 1880b:83 [larval case]. —Müller, 1921:386 [♂, antenna]. —Ulmer, 1957:172 [bibliography; possibly *Abtrichia antennata*]. —Angrisano, 1999:35 [checklist]. —Paprocki et al., 2004:12 [checklist]. —Paprocki and França, 2014:54 [checklist].

**Distribution.** Brazil.

***squamosa*** (Mosely), 1939a:226 [Type locality: Brazil, Edo. Santa Catarina, Nova Teutonia; BMNH; ♂; in *Abtrichia*]. —Angrisano, 1999:31 [checklist]. —Blahnik et al., 2004:4 [distribution]. —Paprocki et al., 2004:10 [checklist]. —Dumas et al., 2009:365 [distribution]. —Dumas and Nessimian, 2012:14 [checklist]. —Oláh and Flint, 2012:140 [distribution]. —Paprocki and França, 2014:39 [checklist]. —Santos et al., 2016a:472 [to *Peltopsyche*].

**Distribution.** Argentina, Brazil.

***vegosa*** (Oláh and Flint), 2012:140 [Type locality: Paraguay, 2 km South, Cerro Cora; NMNH; ♂; in *Abtrichia*]. —Santos et al., 2016a:472 [to *Peltopsyche*].

**Distribution.** Brazil, Paraguay.

***veva*** (Oláh and Johanson), 2011:153 [Type locality: French Guiana, Maripasoula, Lawa River: Maripasoula, 3°37.959'N, 54°1.426'W, el. 83 m; NHRS; ♂; in *Abtrichia*]. —Oláh and Flint, 2012:140 [distribution]. —Santos et al., 2016a:472 [to *Peltopsyche*].

**Distribution.** French Guiana, Guyana.

#### Genus *Ragatrichia* Oláh and Johanson [5]

*Ragatrichia* Oláh and Johanson, 2011:239 [Type species: *Ragatrichia ragada* Oláh and Johanson, 2011, original designation].

This is a recently described genus containing five species, two newly described and three others transferred from different genera: *Metrichia dietzi* Flint, 1974; *Rhyacopsyche garuhape* Angrisano and Sganga, 2009; and *Rhyacopsyche yatay* Angrisano, 1989. Immatures and cases were described for *Ragatrichia yatay* (Angrisano, 2002) (as *Rhyacopsyche yatay*).

***angrisanae*** Oláh and Johanson, 2011:240 [Type locality: French Guiana, Approuaguekaw, Kaw Mt, 4°33.035'N, 52°11.661'W, el. 104 m; OPC; ♂].

**Distribution.** French Guiana.

***dietzi*** (Flint), 1974c:63 [Type locality: Guyana, Rockstone, Essequibo River; NMNH; ♂; in Ochrotrichia (Metrichia)]. —Oláh and Johanson, 2011:240 [to *Ragatrichia*; distribution].

**Distribution.** French Guiana, Guyana, Suriname.

***garuhape*** (Angrisano and Sganga), 2009:63 [Type locality: Argentina, Misiones: Parque Provincial Salto Encantado; MACN; in *Rhyacopsyche*]. —Oláh and Johanson, 2011:243 [to *Ragatrichia*].

**Distribution.** Argentina.

***ragada*** Oláh and Johanson, 2011:242 [Type locality: French Guiana, Approuaguekaw, Kaw Mt, 4°33.035'N, 52°11.661'W, el. 104 m; NHRM; ♂].

**Distribution.** French Guiana.

***yatay*** (Angrisano), 1989:157 [Type locality: Argentina, Entre Rios, Parque Nacional el Palmar; MACN; ♂; ♀; in *Rhyacopsyche*]. —Angrisano, 1999:35 [checklist]. —Angrisano, 2002:402 [larva; pupa; case; biology; taxonomic remarks]. —Angrisano and Sganga, 2007:31 [♂; larva; distribution]. —Wasmund and Holzenthal, 2007:21 [♂; illustrations after Angrisano, 1989]. —Oláh and Johanson, 2011:242 [to *Ragatrichia*].

**Distribution.** Argentina.

#### Genus *Rhyacopsyche* Müller [28]

*Rhyacopsyche* Müller, 1879a:40 [*nomen nudum*]. —Müller, 1879b:143 [Type species: *Rhyacopsyche hagenii* Müller, 1879b; by monotypy]. —Müller, 1880a:121. —Müller, 1880b:72. —Flint, 1971a:516 [definition; revision]. —Angrisano, 2002:403 [taxonomic remarks]. —Wasmund and Holzenthal, 2007:5 [revision, key to species].

Müller (1879a) mentioned the generic name *Rhyacopsyche* for a Brazilian species, which was subsequently (1879b) named *hagenii*, and described from larval cases only. The first description of the adults and larvae was later published by Thienemann (1905a), and a detailed description of the genus was given by Flint (1971a) and again by Wasmund and Holzenthal (2007). *Rhyacopsyche* species are primarily Central and South American in distribution, and are placed in the Ochrotrichiini.

Larvae of three species have been described: *Rhyacopsyche hagenii* by Thienemann (1905a), *Rhyacopsyche mexicana* by Flint (1971a), and *Rhyacopsyche mutisi* by Mey and Joost (1990). They construct silken cases attached by silken stalk to a solid substrate in fast flowing water. The attached cases, either singly or in clusters, float freely in the current. At pupation the stalk is shortened and thickened so that the case stands out stiffly from the substrate (Wasmund and Holzenthal 2007). The larval food is unknown.

***andina*** Flint, 1991:61 [Type locality: Colombia, Dpto. Antioquia, Quebrada la Agudelo, 2km E El Retiro; NMNH; ♂]. —Flint, 1996b:397 [distribution]. —Muñoz-Quesada, 2000:278 [checklist]. —Wasmund and Holzenthal, 2007:7 [♂; distribution].

**Distribution.** Colombia, Peru, Venezuela.

***angra*** Santos, Jardim and Nessimian, 2011:817 [Type locality: Brazil, Rio de Janeiro, Angra dos Reis, 23°00'23"S, 44°29'15"W, el. 40 m; DZRJ; ♂; ♀]. —Paprocki and França, 2014:54 [checklist].

**Distribution.** Brazil.

***benwa*** Wasmund and Holzenthal, 2007:8 [Type locality: Peru, Madre de Dios, Manu, Pakitza, el. 250 m; MHNJP; ♂].

**Distribution.** Bolivia, Ecuador, Peru.

***bulbosa*** Wasmund and Holzenthal, 2007:8 [Type locality: Brazil, Rio de Janeiro, Nova Friburgo, municipal water supply, el. 950 m; MZUSP; ♂]. —Dumas et al., 2009:366 [distribution]. —Calor, 2011:321 [checklist]. —Dumas and Nessimian, 2012:15 [checklist]. —Paprocki and França, 2014:54 [checklist].

**Distribution.** Brazil.

***bunkotala*** Oláh and Johanson, 2011:244 [Type locality: Ecuador, Wild Sumaco, near Pacto Sumaco; OPC; ♂].

**Distribution.** Ecuador.

***chichotla*** Bueno-Soria and Hamilton, 1986:303 [Type locality: Mexico, Oaxaca, 7km NE Huautla de Jimenez; NMNH; ♂]. —Wasmund and Holzenthal, 2007:9 [♂; redescription].

**Distribution.** Mexico.

***colei*** Wasmund and Holzenthal, 2007:9 [Type locality: Venezuela, Lara, Parque Nacional Dinira, Quebrada Buenos Aires, 09°36'24"N, 70°04'11"W, el. 1850 m; UMSP; ♂].

**Distribution.** Venezuela.

***colombiana*** Wasmund and Holzenthal, 2007:10 [Type locality: Colombia, Valle Del Cauca, Municipio El Cerrito, Rio Cerrito 7.1 km E. Hacienda “El Paraiso”, 03°38'59"N, 76°09'10"W, el. 1950 m; UMSP; ♂].

**Distribution.** Colombia.

***colubrinosa*** Wasmund and Holzenthal, 2007:11 [Type locality: Peru, Cuzco, Paucartambo to Pilcopata Rd. streamlet 50 m E Quiacalzón, 13°01.57'S, 71°29.97'W, el. 1050 m; MHNJP; ♂; ♀]. —Oláh and Johanson, 2011:244 [checklist].

**Distribution.** Ecuador, Peru.

***diacantha*** Santos, Jardim and Nessimian, 2011:815 [Type locality: Brazil, Pará, Parauapebas (Área de Proteção Ambiental do Igarapé Gelado, Barragem do Gelado, 05°57'56"S, 50°13'00"W, el. 224 m; DZRJ; ♂; ♀]. —Paprocki and França, 2014:54 [checklist].

**Distribution.** Brazil.

***dikrosa*** Wasmund and Holzenthal, 2007:11 [Type locality: Brazil, São Paulo, Pedregulho, 140 km NE Ribeirão Preto; MZUSP; ♂; ♀]. —Dumas et al., 2009:366 [distribution]. —Calor, 2011:321 [checklist]. —Dumas and Nessimian, 2012:16 [checklist]. —Paprocki and França, 2014:55 [checklist].

**Distribution.** Brazil.

***duplicispina*** Flint, 1996a:91 [Type locality: Tobago, Bridge B1/5, 6.5 km N Roxborough; NMNH; ♂]. —Botosaneanu, 2002:89 [checklist]. —Wasmund and Holzenthal, 2007:12 [♂; redescription].

**Distribution.** Tobago.

***flinti*** Wasmund and Holzenthal, 2007:13 [Type locality: Venezuela, Guárico, Parque Nacional Guatopo, Queb. Guatopo, 0.5 km N Est. La Colina, 10°0'50"N, 66°21'47"W, el. 600 m; UMSP; ♂; ♀].

**Distribution.** Venezuela.

***hagenii*** Müller, 1879b:143 [Type locality: Brazil; MNRJ; larval and pupal cases]. —Thienemann, 1905a:287 [larva; ♂]. —Müller, 1921:525 [larva]. —Ulmer, 1957:172, 187 [literature; key to larval genus]. —Angrisano, 1995b:509 [distribution]. —Angrisano, 1999:35 [checklist]. —Paprocki et al., 2004:12 [checklist]. —Wasmund and Holzenthal, 2007:6 [♂; ♀; distribution]. —Dumas et al., 2009:367 [distribution]. —Calor, 2011:321 [checklist]. —Dumas and Nessimian, 2012:16 [checklist]. —Paprocki and França, 2014:55 [checklist].

**Distribution.** Argentina; Brazil, Uruguay.

***hajtoka*** Oláh and Johanson, 2011:245 [Type locality: Ecuador, Alambi; OPC; ♂].

**Distribution.** Ecuador.

***hasta*** Wasmund and Holzenthal, 2007:13 [Type locality: Peru, Cuzco, Paucartambo to Pilcopata rd., streamlet 50 m E Quiacalzón, 13°01.57’ S, 71°29.97’ W, el. 1050 m; MHNJP; ♂]. —Oláh and Johanson, 2011:247 [checklist].

**Distribution.** Peru.

***intraspira*** Wasmund and Holzenthal, 2007:14 [Type locality: Peru, Cuzco, Paucartambo to Pilcopata rd., Rio San Pedro at Puente San Pedro, 13°03.30'S, 71°32.78'W, el. 1445 m; MHNJP; ♂].

**Distribution.** Peru.

***jimena*** Flint, 1991:59 [Type locality: Colombia, Dpto. Antioquia, Quebrada la Jimenez, Sopetran; NMNH; ♂]. —Muñoz-Quesada, 2000:278 [checklist]. —Wasmund and Holzenthal, 2007:14 [♂; redescription].

**Distribution.** Colombia.

***matthiasi*** Flint, 1991:61 [Type locality: Colombia, Dpto. Antioquia, Urrao; NMNH; ♂]. —Muñoz-Quesada, 2000:278 [checklist]. —Wasmund and Holzenthal, 2007:15 [♂; redescription].

**Distribution.** Colombia.

***mexicana*** (Flint), 1967b:12 [Type locality: Mexico, Vera Cruz, Rio Tacolapan; NMNH; ♂; in *Metrichia*]. —Flint, 1971a:519 [♂; ♀; larva; case; distribution; to *Rhyacopsyche*]. —Bueno-Soria and Flint, 1978:205 [distribution]. —Maes, 1999:1195 [checklist]. —Bueno-Soria et al., 2005:75 [distribution]. —Chamorro-Lacayo et al., 2007:44 [checklist]. —Wasmund and Holzenthal, 2007:15 [♂; redescription; distribution].

**Distribution.** Costa Rica, Guatemala, Mexico, Nicaragua.

***mutisi*** Mey and Joost, 1990:134 [Type locality: Colombia, Dept. Tolima, Mariquita, Rio Medina; KMUL; ♂; ♀; larva; case]. —Muñoz-Quesada, 2000:278 [checklist]. —Wasmund and Holzenthal, 2007:16 [♂; redescription].

**Distribution.** Colombia.

***obliqua*** Flint, 1971a:523 [Type locality: Mexico, Veracruz, Fortin de las Flores; NMNH; ♂; ♀]. —Bueno-Soria and Flint, 1978:205 [distribution]. —Wasmund and Holzenthal, 2007:16 [♂; redescription].

**Distribution.** Mexico.

***peruviana*** Flint, 1975:568 [Type locality: South-Peru, Sivia; ZSZMH; ♂; ♀]. —Wasmund and Holzenthal, 2007:18 [♂; redescription; distribution].

**Distribution.** Ecuador, Peru.

***rhamphisa*** Wasmund and Holzenthal, 2007:19 [Type locality: Colombia, Valle Del Cauca, Municipio El Cerrito, Rio Cerrito 7.1 kms E. Hacienda “El Paraiso”, 03°38'59"N, 076°09'10"W, el. 1950 m; UMSP; ♂; ♀]. —Oláh and Johanson, 2011:248 [distribution].

**Distribution.** Colombia, Costa Rica, Peru.

***shorti*** Thomson and Holzenthal, 2012:36 [Type locality: Venezuela, Bolívar, Gran Sabana, E. Pauji, “Río Curvita”, 4°31.237'N, 61°31.591'W, el. 869 m; UMSP; ♂].

**Distribution.** Venezuela.

***tanylobosa*** Wasmund and Holzenthal, 2007:19 [Type locality: Venezuela, Barinas, Parque Nacional Sierra Nevada, Queb. San Juan in Sta. Rosa, 08°27.87'N, 070°50.92'W, el. 1000 m; UMSP; ♂; ♀].

**Distribution.** Ecuador, Peru, Venezuela.

***torulosa*** Flint, 1971a:521 [Type locality: Guatemala, Escuintla, Rio Metapa, 10km SE Escuintla; NMNH; ♂; ♀]. —Holzenthal, 1988c:63 [distribution]. —Wasmund and Holzenthal, 2007:20 [♂; redescription].

**Distribution.** Costa Rica, Guatemala.

***turrialbae*** Flint, 1971a:523 [Type locality: Costa Rica, Cartago, Chitaria; NMNH; ♂; ♀]. —Holzenthal, 1988c:63 [distribution]. —Wasmund and Holzenthal, 2007:21 [♂; redescription].

**Distribution.** Costa Rica.

#### Genus *Scelobotrichia* Harris and Bueno-Soria [3]

*Scelobotrichia* Harris and Bueno-Soria, 1993:75 [Type species: *Scelobotrichia contrerasi* Harris and Bueno-Soria, 1993, original designation]. —Bowles et al., 1999:47 [larva; taxonomic remarks]. —Oláh and Johanson, 2011:142 [taxonomic remarks].

The genus *Scelobotrichia* was erected for a small group of species from Mexico, similar to a species then considered to be an aberrant member of *Alisotrichia*. It was originally placed in the Stactobiinae (Harris and Bueno-Soria 1993), but recent study has shown that it, and related genera, are better placed in the Leucotrichiinae, tribe Alisotrichiini (Bowles et al. 1999; Santos et al. 2016a). Adults are typically collected on rocks or vegetation at the sides of waterfalls or turbulent streams. The larvae of *Scelobotrichia contrerasi* and *Scelobotrichia profunda* have been described (Bowles et al. 1999). They do not build cases.

***contrerasi*** Harris and Bueno-Soria, 1993:77 [Type locality: Mexico, Nuevo Leon, Municipio de Santiago, roadside waterfall near Cola de Caballo, 3 km SW Cieneguilla; NMNH; ♂; ♀]. —Bowles et al., 1999:47 [larva].

**Distribution.** Mexico.

***profunda*** Harris and Bueno-Soria, 1993:78 [Type locality: Mexico, Guerrero, Rio en Barranca, Ruta Taxco-Teloloapan; IBUNAM; male/; ♀]. —Bowles et al., 1999:48 [larva].

**Distribution.** Mexico.

***quemada*** (Flint), 1970:28 [Type locality: Mexico, San Luis Potosi, Rancho Quemado, Rt. 85, 6 km S Tamazunchale; NMNH; ♂; in *Alisotrichia*]. —Bueno-Soria and Flint, 1978:200 [distribution]. —Harris and Bueno-Soria, 1993:80 [♂; to *Scelobotrichia*].

**Distribution.** Mexico.

#### Genus *Taraxitrichia* Flint and Harris [1]

*Taraxitrichia* Flint and Harris, 1991:411 [Type species: *Taraxitrichia amazonensis* Flint and Harris, 1991, original designation]. —Pes and Hamanda, 2003:2 [larva; cases; biology; distribution].

The genus *Taraxitrichia* was established for a single species of microcaddisfly collected near Puerto Ayacucho, Venezuela. The genus is one of four genera placed in the exclusively New World subfamily Neotrichiinae. The adults were taken at lights placed near clear, fast-flowing streams.

Pes and Hamanda (2003) described the immatures of an unknown species collected in streams located in an open area where the forest had been cleared for road construction near Manaus, Brazil. Larvae and pupae were only collected in association with freshwater sponges (genera *Metania* and *Spongilla*), and the sponge spicules were incorporated and could be identified in the larval cases (Pes and Hamanda 2003).

***amazonensis*** Flint and Harris, 1991:411 [Type locality: Venezuela, Territorio Federal Amazonas, Rio Cataniapo, 10 km S Puerto Ayacucho; NMNH; ♂; ♀].

**Distribution.** Brazil (tentative), Venezuela.

#### Genus *Tizatetrichia* Harris, Flint and Holzenthal [1]

*Tizatetrichia* Harris, Flint and Holzenthal, 2002a:55 [Type species: *Tizatetrichia costaricensis*, original designation].

*Tizatetrichia* was established for a single species from the Río Tizate, Costa Rica. The genus was placed in the Stactobiinae and is most closely related to *Bredinia* (Harris et al. 2002a). Female and immature stages are unknown.

***costaricensis*** Harris, Flint and Holzenthal, 2002a:51 [Type locality: Costa Rica, Guanacaste, Río Tizate, 7.2 km NE Canãs Dulces, 10.773°N, 85.449°W, el. 275 m; NMNH; ♂].

**Distribution.** Costa Rica.

#### Genus *Tricholeiochiton* Kloet and Hincks [1]

*Leiochiton* Guinard, 1879:139 [Type species *Leiochiton fagesii* Guinard, 1879, by monotypy; preoccupied by *Leiochiton* Curtis, 1831, in Coleoptera]

*Tricholeiochiton* Kloet and Hincks, 1944:97 [Type species: *Leiochiton fagesii* Guinard 1879, by monotypy; replacement name for *Leiochiton* Guinard 1879]. —Wells, 1982:251 [revision, Australian species].

This member of the subfamily Hydroptilinae occurs in Europe and southeast Asia (Marshall 1979), with its greatest diversity in Australia. A single species from Brazil is the only known representative of *Tricholeiochiton* in the New World.

Larvae of *Tricholeiochiton fagesii* and several of the Australian species have been described (Lepneva 1970, Wells 1985). They are found in stagnant or slowly flowing water bodies in plant masses.

***neotropicalis*** Flint, 1992d:70 [Type locality: Brazil, Estado Roraima, Ilha Maraca, Rio Urariocoera; INPA; ♂; ♀]. —Angrisano, 1999:35 [checklist]. —Paprocki et al., 2004:12 [checklist]. —Ribeiro et al., 2009:34 [list of types]. —Paprocki and França, 2014:55 [checklist].

**Distribution.** Brazil.

#### Genus *Tupiniquintrichia* Santos, Nessimian and Takiya [2]

*Tupiniquintrichia* Santos, Nessimian and Takiya, 2016a:475 [Type species: *Peltopsyche maclachlani* Müller, 1879b, original designation].

*Tupiniquintrichia* was proposed for two species, *Tupiniquintrichia maclachlani* and *Tupiniquintrichia procera*, transfferred from *Peltopsyche* and *Leucotrichia*, respectively. The genus has all the typical features of Leucotrichiinae, and is defined by several morphological synapomorphies as well as molecular data (Santos et al., 2016a).

***maclachlani*** (Müller), 1879b:144 [Type locality: Brazil, Santa Catarina, Warnow River, tributary of Itajahy River; MNRJ; case; larva; in *Peltopsyche*]. —Müller, 1880a:133 [larval case]. —Müller, 1880b:83 [larval case]. —Müller, 1921:386 [♂; antenna]. —Ulmer, 1957:172 [bibliography]. —Paprocki et al., 2004:12 [checklist]. —Paprocki and França, 2014:54 [checklist]. —Santos et al., 2016a:476 [to *Tupiniquintrichia*; type status].

**Distribution.** Brazil.

***procera*** (Thomson and Holzenthal), 2015:37 [Type locality: Brazil, Minas Gerais, Córrego da Serra de Ouro Fino, Vale do Tropeiro, 20°12.371'S, 43°38.581'W, el. 1000 m; MZUSP; ♂; in *Leucotrichia*]. —Santos et al., 2016a:476 [to *Tupiniquintrichia*].

**Distribution.** Brazil.

#### Genus *Zumatrichia* Mosely [50]

*Zumatrichia* Mosely, 1937:187 [Type species: *Zumatrichia filosa* Mosely, 1937, original designation]. —Flint, 1970:16 [revision].

*Zumatrichia* is a fairly large monophyletic genus of Leucotrichiinae with 50 species now known in the Neotropics (Santos et al., 2016a). A single species, *Zumatrichia notosa* (Ross), occurs in the United States; the others occur from Mexico, through Central America, to northern South America, with a couple of species in the Lesser Antilles.

Larvae have been described for *Zumatrichia antilliensis*, *Zumatrichia anomaloptera*, *Zumatrichia multisetosa*, and *Zumatrichia notosa* (Flint 1968b, 1970, Wiggins 1996). They construct typical leucotrichiine flattened, silken cases with circular openings at the anterior and posterior ends, attached to rocks in fast flowing water. They feed on periphyton reached by extending the head and thorax through one opening or the other in the retreat.

***alarca*** Oláh and Johanson, 2011:164 [Type locality: Peru, San Martin Prov., Rio Huallaga tributary, small river passing Chazuta, 6°34.665'S, 76°08.209'W; NHRS; ♂].

**Distribution.** Peru.

***angulata*** Flint, 1970:21 [Type locality: Panama, Chiriqui, Rovira, David; NMNH; ♂]. —Aguila, 1992:538 [distribution]. —Armitage et al., 2015b:7 [checklist]. —Armitage and Cornejo, 2015:196 [checklist].

**Distribution.** Panama.

***anomaloptera*** Flint, 1968b:37 [Type locality: Grenada, Balthazar, NMNH; ♂; ♀]. —Malicky, 1983:264 [distribution]. —Botosaneanu, 1988:221 [♂; ♀; distribution]. —Botosaneanu and Sakal, 1992:201 [distribution; ecology]. —Botosaneanu and Alkins-Koo, 1993:7 [distribution]. —Flint and Sykora, 1993:55 [distribution]. —Botosaneanu, 1994a:37 [distribution]. —Flint, 1996a:85 [distribution]. —Botosaneanu, 2002:89 [checklist]. —Botosaneanu and Thomas, 2005:45 [distribution].

**Distribution.** Dominica, Grenada, Guadeloupe, Martinique, St. Lucia, St. Vincent, Tobago, Trinidad.

***antilliensis*** Flint, 1968b:34 [Type locality: Dominica, Clarke Hall; NMNH; ♂; ♀; larva; pupa; case]. —Malicky, 1983:264 [distribution]. —Botosaneanu, 1988:221 [♂; ♀; distribution]. —Flint and Sykora, 1993:54 [distribution]. —Botosaneanu, 1994a:37 [distribution]. —Flint, 1996a:85 [distribution]. —Botosaneanu, 2000:256 [distribution]. —Botosaneanu, 2002:89 [checklist]. —Botosaneanu and Thomas, 2005:45 [distribution]. —Oláh and Flint, 2012:177 [distribution]. —Armitage et al., 2015b:7 [checklist]. —Armitage and Cornejo, 2015:196 [checklist].

**Distribution.** Colombia, Dominica, Ecuador, Grenada, Guadeloupe, Martinique, Panama, St. Lucia, St. Vincent, Venezuela.

***atmena*** Oláh and Flint, 2012:177 [Type locality: Venezuela, Aragua State, Cuyagua, Rio Grande; NMNH; ♂].

**Distribution.** Venezuela.

***attenuata*** Flint, 1970:22 [Type locality: Costa Rica, Cartago, Quebrada Relleno, La Cruzada, E Turrialba; NMNH; ♂]. —Holzenthal, 1988c:63 [distribution]. —Armitage et al., 2015a:6 [distribution]. —Armitage et al., 2015b:7 [checklist]. —Armitage and Cornejo, 2015:196 [checklist].

**Distribution.** Costa Rica, Panama.

***befela*** Oláh and Flint, 2012:179 [Type locality: Venezuela, Barinas State, Rio Santo Domingo, Barinas; NMNH; ♂].

**Distribution.** Venezuela.

***bevagota*** Oláh and Flint, 2012:180 [Type locality: Ecuador, Cotopaxi Province, Quevedo (36 km Northeast), el. 1100 m; NMNH; ♂].

**Distribution.** Ecuador.

***bifida*** Flint, 1970:21 [Type locality: Costa Rica, San Jose, Rio General, Pacuare; NMNH; ♂]. —Holzenthal, 1988c:63 [distribution]. —Aguila, 1992:538 [distribution]. —Armitage et al., 2015b:8 [checklist]. —Armitage and Cornejo, 2015:196 [checklist].

**Distribution.** Costa Rica, Panama.

***caudifera*** Flint, 1970:23 [Type locality: Panama, Chiriqui, Dolega; NMNH; ♂]. —Holzenthal, 1988c:63 [distribution]. —Aguila, 1992:538 [distribution]. —Chamorro-Lacayo et al., 2007:44 [checklist]. —Armitage et al., 2015b:8 [checklist]. —Armitage and Cornejo, 2015:197 [checklist].

**Distribution.** Costa Rica, Nicaragua, Panama.

***chiriquiensis*** Flint, 1970:20 [Type locality: Panama, Chiriqui, Dolega; NMNH; ♂]. —Holzenthal, 1988c:64 [distribution]. —Aguila, 1992:538 [distribution]. —Armitage et al., 2015b:8 [checklist]. —Armitage and Cornejo, 2015:197 [checklist].

**Distribution.** Costa Rica, Panama.

***corosa*** Oláh and Flint, 2012:181 [Type locality: Ecuador, Cotopaxi Province, Quevedo (36 km Northeast), el. 1100 m; NMNH; ♂].

**Distribution.** Ecuador.

***dereka*** Oláh and Flint, 2012:183 [Type locality: Panama, San Blas Province, Rio Carti Grande, 2 km West Nusagandi; NMNH; ♂]. —Armitage et al., 2015b:8 [checklist]. —Armitage and Cornejo, 2015:197 [checklist].

**Distribution.** Panama.

***diamphidia*** Flint, 1970:21 [Type locality: Costa Rica, Puntarenas, 2.8 miles E Golfito; NMNH; ♂]. —Holzenthal, 1988c:64 [distribution].

**Distribution.** Costa Rica.

***echinata*** Flint, 1967b:11 [Type locality: Guatemala, El Progreso, San Agustin Acasaguastlan; NMNH; ♂]. —Flint, 1970:18 [distribution]. —Chamorro-Lacayo et al., 2007:44 [checklist].

**Distribution.** Guatemala, Honduras, Nicaragua.

***felfesa*** Oláh and Flint, 2012:184 [Type locality: Venezuela, Zulia State, Perijo El Tucuco, Mission el Tucuco, Rio El Tucuco, 0.5 km from Church; NMNH; ♂].

**Distribution.** Venezuela.

***fesuka*** Oláh and Flint, 2012:185 [Type locality: Ecuador, Napo Province, Pano, el. 580 m; NMNH; ♂].

**Distribution.** Ecuador.

***filosa*** Mosely, 1937:187 [Type locality: Mexico, Chiapas, Saltenango de la Paz; BMNH; ♂]. —Flint, 1970:23 [♂; distribution]. —Bueno-Soria and Flint, 1978:201 [distribution]. —Holzenthal, 1988c:64 [distribution]. —Maes and Flint, 1988:4 [distribution]. —Maes, 1999:1195 [checklist]. —Chamorro-Lacayo et al., 2007:44 [checklist]. —Bueno-Soria and Barba-Álvarez, 2011:358 [checklist].

**Distribution.** Costa Rica, Guatemala, Mexico, Nicaragua.

***galtena*** Mosely, 1937:188 [Type locality: Mexico, Chiapas, Saltenango de la Paz; BMNH; ♂]. —Flint, 1970:19 [♂; distribution]. —Bueno-Soria and Flint, 1978:201 [distribution]. —Holzenthal, 1988c:64 [distribution]. —Chamorro-Lacayo et al., 2007:44 [checklist]. —Bueno-Soria and Barba-Álvarez, 2011:358 [checklist]. —Armitage et al., 2015a:6 [distribution]. —Armitage et al., 2015b:8 [checklist]. —Armitage and Cornejo, 2015:197 [checklist].

**Distribution.** Costa Rica, Honduras, Mexico, Nicaragua, Panama.

***gorba*** Oláh and Flint, 2012:187 [Type locality: Ecuador, Zamora-Chinchipe Province, Rio Chicana, 9 km North Yanzatza, el. 880 m; NMNH; ♂].

**Distribution.** Ecuador.

***gula*** Oláh and Flint, 2012:188 [Type locality: Venezuela, Barinas State, Rio Santo Domingo, Barinas; NMNH; ♂].

**Distribution.** Venezuela.

***haroma*** Oláh and Flint, 2012:189 [Type locality: Venezuela, Barinas State, Puente Parangula, 8 km South Barinitas; NMNH; ♂].

**Distribution.** Venezuela.

***kerekeda*** Oláh and Flint, 2012:191 [Type locality: Colombia, Rio Raposo; NMNH; ♂].

**Distribution.** Colombia, Ecuador.

***kisgula*** Oláh and Flint, 2012:192 [Type locality: Ecuador, Napo Province, Lago Agrio (48 km West), Rio Aguarico; NMNH; ♂].

**Distribution.** Ecuador.

***kislaba*** Oláh and Flint, 2012:194 [Type locality: Ecuador, Pastaza Province, Puyo (3 km West); NMNH; ♂].

**Distribution.** Ecuador.

***koztesa*** Oláh and Flint, 2012:195 [Type locality: Venezuela, Aragua State, Parque Nacional Henri Pittier, Rio La Trilla, 22.5 km North of Rancho Grande on Road; NMNH; ♂].

**Distribution.** Venezuela.

***lapa*** Oláh and Flint, 2012:196 [Type locality: Ecuador, Pastaza Province, Puyo (27 km North), Estacion Fluviometrica; NMNH; ♂].

**Distribution.** Ecuador.

***lezarda*** Malicky, 1980:220 [Type locality: Guadeloupe, Mittellauf de Flusses Lezard bei Chemin de Diane; Coll. Malicky; ♂]. —Malicky, 1983:264 [checklist]. —Flint and Sykora, 1993:49 [checklist]. —Botosaneanu, 2002:89 [checklist]. —Botosaneanu and Thomas, 2005:55 [checklist].

**Distribution.** Guadeloupe.

***longispina*** Bueno-Soria, 1983b:454 [Type locality: Mexico, Veracruz, Los Tuxtlas area, Rio La Palma; NMNH; ♂].

**Distribution.** Mexico.

***marica*** Flint, 1981a:26 [Type locality: Venezuela, Aragua, Maracay, Río Limón, Estacion Piscicultura; NMNH; ♂].

**Distribution.** Venezuela.

***masa*** Oláh and Flint, 2012:198 [Type locality: Ecuador, Pastaza Province, Puyo; NMNH; ♂].

**Distribution.** Ecuador.

***maskara*** Oláh and Flint, 2012:199 [Type locality: Panama, San Blas Province, Rio Carti Grande, 2 km West Nusagandi; NMNH; ♂]. —Armitage et al., 2015b:8 [checklist]. —Armitage and Cornejo, 2015:197 [checklist].

**Distribution.** Panama.

***maskoska*** Oláh and Flint, 2012:201 [Type locality: Venezuela, Zula State, Perijo El Tucuco, Mission El Tucuco, Rio El Tucuco, 0.5 km from Church; NMNH; ♂].

**Distribution.** Venezuela.

***multisetosa*** Flint, 1970:17 [Type locality: Guatemala, Suchitepequez, Cuyotenango; NMNH; ♂; larva; case]. —Bueno-Soria and Flint, 1978:201 [distribution]. —Holzenthal, 1988c:64 [distribution]. —Bueno-Soria and Barba-Álvarez, 2011:358 [checklist].

**Distribution.** Costa Rica, Guatemala, Honduras, Mexico.

***nelkula*** Oláh and Flint, 2012:202 [Type locality: Panama, San Blas Province, Rio Carti Grande, 2 km West Nusagandi; NMNH; ♂]. —Armitage et al., 2015b:8 [checklist]. —Armitage and Cornejo, 2015:197 [checklist].

**Distribution.** Panama.

***palmara*** Flint, 1970:22 [Type locality: El Salvador, La Libertad, Rio El Palmar, 15 miles N La Libertad; NMNH; ♂]. —Holzenthal, 1988c:64 [distribution]. —Flint and Reyes, 1991:484 [distribution]. —Chamorro-Lacayo et al., 2007:44 [checklist]. —Armitage et al., 2015b:8 [checklist]. —Armitage and Cornejo, 2015:197 [checklist].

**Distribution.** Costa Rica, Ecuador, El Salvador, Nicaragua, Panama, Peru.

***picigula*** Oláh and Flint, 2012:203 [Type locality: Ecuador, Napo Province, Rio Jondachi, el. 950 m, 30 km North Tena; NMNH; ♂].

**Distribution.** Ecuador.

***rhamphoides*** Flint, 1970:24 [Type locality: Costa Rica, Puntarenas, Rio La Vieja, near Lagarto; NMNH; ♂]. —Holzenthal, 1988c:64 [distribution]. —Aguila, 1992:538 [distribution]. —Armitage et al., 2015b:8 [checklist]. —Armitage and Cornejo, 2015:197 [checklist].

**Distribution.** Costa Rica, Panama.

***saluda*** Flint, 1970:19 [Type locality: Panama, Canal Zone, pipeline road, Rio Agua Salud; NMNH; ♂]. —Aguila, 1992:538 [distribution]. —Armitage et al., 2015b:8 [checklist]. —Armitage and Cornejo, 2015:197 [checklist].

**Distribution.** Panama.

***sima*** Oláh and Flint, 2012:205 [Type locality: Ecuador, Pichincha Province, Santo Domingo de los Colorados, 14 km East; NMNH; ♂].

**Distribution.** Ecuador.

***sortetla*** Oláh and Flint, 2012:206 [Type locality: Panama, Darien Province, Rio Tuira at Rio Pucuro; NMNH; ♂]. —Armitage et al., 2015b:8 [checklist]. —Armitage and Cornejo, 2015:197 [checklist].

**Distribution.** Panama.

***strobilina*** Flint, 1970:20 [Type locality: Costa Rica, Cartago, 3 miles W Turrialba; NMNH; ♂]. —Holzenthal, 1988c:64 [distribution].

**Distribution.** Costa Rica.

***teapa*** Flint, 1970:24 [Type locality: Mexico, Tabasco, Rio Puyacatengo, E Teapa; NMNH; ♂]. —Bueno-Soria and Flint, 1978:201 [distribution].

**Distribution.** Mexico.

***teribe*** Harris and Armitage, 2015:2 [Type locality: Panama, Chiriquí Province, Cuenca 108, Quebrada Grande, Boquete, Valle Escondido, below Sabor Restaurant, 8.77970°N, 82.44016°W, el .1122 m; MIUP; ♂]. —Armitage et al., 2015b:8 [checklist]. —Armitage and Cornejo, 2015:197 [checklist].

**Distribution.** Panama.

***tompagula*** Oláh and Flint, 2012:207 [Type locality: Colombia, Meta Department, Quebrada Blanca, 3 km West Restrepo; NMNH; ♂].

**Distribution.** Colombia.

***turuda*** Oláh and Flint, 2012:208 [Type locality: Panama, San Blas Province, Quebrada Pingadi, 9 km North Nusagandi; NMNH; ♂]. —Armitage et al., 2015b:8 [checklist]. —Armitage and Cornejo, 2015:197 [checklist].

**Distribution.** Panama.

***tusa*** Oláh and Flint, 2012:210 [Type locality: Colombia, Choco Department, Rio Atrato, Yuto; NMNH; ♂].

**Distribution.** Colombia, Venezuela.

***varrata*** Oláh and Johanson, 2011:165 [Type locality: Peru, San Martin Prov., Rio Huallaga tributary, small river passing Chazuta, 6°34.665'S, 76°08.209'W; NHRS; ♂].

**Distribution.** Peru.

***vieja*** Flint, 1970:19 [Type locality: Costa Rica, Puntarenas, Rio La Vieja, near Lagarto, E Palmar Norte; NMNH; ♂]. —Holzenthal, 1988c:64 [distribution].

**Distribution.** Costa Rica.

***zegla*** Harris and Armitage, 2015:4 [Type locality: Panama, Bocas del Toro Province, Cuenca 91, Río Teribe at Zegla; NMNH; ♂]. —Armitage et al., 2015b:8 [checklist]. —Armitage and Cornejo, 2015:197 [checklist].

**Distribution.** Panama.

### Family Kokiriidae

This is a small family of 6 genera and 15 species occurring in the Australasian and Neotropical regions. Two New World species, including the immature stages of one, are known. Members of the family in Australia and New Zealand construct tubular cases of sand grains, which are somewhat dorsoventrally flattened (McFarlane 1964, Neboiss 1991). These larvae live on sandy substrates of streams and lakes, or burried just below the sandy bottom. The Chilean species, *Pangulia nea*, has similar habits and case morphology (Rojas 2007).

#### Genus *Pangullia* Navás [2]

*Pangullia* Navás, 1934a:178 [Type species: *Pangullia faziana* Navás, 1934a, original designation; in Limnephilidae]. —Schmid, 1955b:224 [not a Limnephilidae]. —Flint, 1974e:85 [as unidentified, probably a Hydrobiosinae]. —Flint et al., 1999a:77 [to Kokiriidae]. —Rojas, 2005:27 [review of genus].

*Rhynchopsyche* Schmid, 1955a:151 [Type species: *Rhyncopsyche fusca* Schmid, 1955, original designation; in Brachycentridae]. —Flint, 1974e:90 [to Kokiriidae]. —Flint et al., 1999a:77 [to synonymy].

The two species in this genus, described 75 years apart, are limited to Chile. The immature stages of the newly described species are known (Rojas 2007).

***faziana*** Navás, 1934a:178 [Type locality: Chile, Panguipulli; DEI; ♂]. —Flint, 1974e:85 [checklist]. —Rojas, 2005:29 [♀].

—*fusca* (Schmid) 1955a:151 [Type locality: Chile, Isla de Chiloé, Aucár; NMNH; ♂; in *Rhyncopsyche*]. —Flint, 1974e:90 [checklist]. —Flint et al., 1999a:77 [to synonymy].

**Distribution.** Chile.

***nea*** Rojas, 2005:29 [Type locality: Chile, VII Región, Parque Nacional Los Ruiles, 35°50'32"S, 72°30'24"W; MNHNS; ♂; reared from pupa]. —Rojas, 2007:26 [larva; pupa].

**Distribution.** Chile.

### Family Lepidostomatidae

This family of about 500 described species is predominately Holarctic, Afrotropical, and Oriental in distribution. Weaver (1988) provided a review of the North and Central American species, and a generic synonymy and catalog of world species (Weaver 2002). In the latter work, he synonymized about two dozen genera or subgenera with *Lepidostoma*. Malicky (2005) and Ivanov (2011) expressed objections to this act. Myers and Sperling (2002) inferred the phylogeny of some of these former genus-group taxa based on gene sequence data and found that the monophly of some was doubtful. Only two genera occur in the New World, *Theliopsyche* and *Lepidostoma*, with only the latter extending into the Neotropics, as far south as northwestern Panama.

Larvae feed on decaying organic matter and are more often than not associated with springs and quiet waters of small streams in mountainous area. Wiggins (1996) and Weaver (1988) provided descriptions of the larvae of several North American species.

#### Genus *Lepidostoma* Rambur [28]

*Lepidostoma* Rambur, 1842:493 [Type species: *Lepidostoma squamulosum* Rambur, 1842 = *Phryganea hirta* Fabricius 1775, subsequent selection of Ross 1944]. —Ross, 1944:258 [synonymy]. —Ross, 1946:266 [revision]. —Weaver, 1988:15 [revision]. —Weaver, 2002:173 [supression of subgenera names, generic synonymy, checklist of world species].

*Nosopus* McLachlan, 1871:114 [Type species: *Nosopus podager* McLachlan, 1871, by monotypy]. —Ross, 1944:258 [to synonymy]. —Weaver, 1988:19 [as subgenus]. —Weaver, 2002:173, 174 [supression of subgenus name, all former subgenus *Nosopus* species to *Lepidostoma podagrum* branch].

*Olemira* Banks, 1897:29 [Type species: *Olemira americana* Banks, 1897, by monotypy]. —Ross, 1944:258 [to synonymy]. —Weaver, 1988:19 [as synonym of *Nosopus*].

*Eremopsyche* Banks, 1901:367 [Type species: *Eremopsyche frontalis* Banks, 1901, original designation]. —Ross, 1946:290 [placement]. —Flint, 1967c:23 [to synonymy].

*Atomyiodes* Ulmer, 1911:23 [Type species: *Atomyiodes bispinosus* Ulmer, 1911, by monotypy]. —Ross 1946:290 [placement]. —Denning, 1962a:39 [to synonymy].

*Phanopsyche* Banks 1911:357 [Type species: *Phanopsyche grisea* Banks, 1911, by monotypy]. —Ross, 1944:258 [to synonymy].

*Neodinarthrum* Weaver, 1988:75 [Type species: *Olemira pluvialis* Milne, 1936, original designation, as subgenus]. —Weaver, 2002:174 [supression of subgenus name].

*Lepidostoma* is one of the two currently recognized lepidostomatid genera in the Western Hemisphere, and the sole genus with Neotropical representation. Most of the approximately 80 New World species occur in the western and eastern mountains of the United States and Canada. Twenty-eight species are now known from Mexico and Central America.

The Neotropical species are confined to small forest streams generally above 1000 m, and seem to be locally endemic. Additional collecting will likely reveal more new species. They frequent the quiet shallows where leaf litter accumulates and are presumed to be detritivorous. As reported by Weaver (1988), larvae construct cylindrical, sand grain cases as well as cases of plant fragments, which are often square in cross section.

***aztecum*** Flint and Bueno-Soria, 1977:378 [Type locality: Mexico, Morelos, Lagunas Zempoala; NMNH; ♂]. —Bueno-Soria and Flint, 1978:214 [distribution]. —Weaver 1988:47 [♂; pupa; subgenus *Nosopus*, *Mexicanum* Group]. —Rojas-Ascencio, et al., 2002:377 [distribution].

**Distribution.** Mexico.

***bakeri*** Flint, 1965:175 [Type locality: United States, Arizona, Portal, Southwest Research Station; NMNH; ♂]. —Flint, 1967d:175 [distribution]. —Denning 1968b:68 [♂; ♀; redescription; distribution]. —Flint and Bueno-Soria, 1977:378 [diagnosis; distribution]. —Bueno-Soria and Flint, 1978:214 [distribution]. —Weaver, 1988:40 [♂; ♀; larva; *Unicolor* Group]. —Bueno-Soria and Barba-Álvarez, 2011:358 [checklist].

**Distribution.** Guatemala, Mexico, U.S.A.

***catarina*** Bueno-Soria, Santiago-Fragoso, and Barba-Álvarez, 2001:153 [Type locality: Mexico, Oacaxa, Sta Catarina La Chatao, 17°15'58"N, 96°28'15"W, el. 2160 m; CNIN; ♂; in subgenus *Nosopus*].

**Distribution.** Mexico.

***chiriquiense*** Holzenthal and Strand, 1992:490 [Type locality: Panama, Chiriquí, Guadaloupe Arriba, 8°52'26"N, 82°33'13"W; NMNH; ♂; subgenus *Nosopus*, *Mexicanum* Group, as *chiriquiensis*]. —Armitage et al., 2015b:9 [checklist]. —Armitage and Cornejo, 2015:198 [checklist].

**Distribution.** Panama.

***dafila*** Bueno-Soria and Contreras, 1986:209 [Type locality: Mexico, Oaxaca, Finca Pacifica, Mpio. de Pluma Hidalgo; IBUNAM; ♂]. —Weaver 1988:48 [♂; subgenus *Nosopus*, *Mexicanum* Group].

**Distribution.** Mexico.

***delongi*** Ross, 1946:283 [Type locality: Mexico, Zitacuara; INHS; ♂]. —Bueno-Soria and Padilla, 1981:391 [distribution]. —Bueno-Soria and Flint, 1978:214 [distribution]. —Weaver, 1988:48 [♂; subgenus *Nosopus*, *Mexicanum* Group].

**Distribution.** Mexico.

***denningi*** Weaver, 1988:49 [Type locality: Mexico, Chiapas, San Cristobal; NMNH; ♂; subgenus *Nosopus*, *Mexicanum* Group]. —Bueno-Soria and Barba-Álvarez, 2011:358 [checklist].

**Distribution.** Mexico.

***ectopium*** Holzenthal and Strand, 1992:491 [Type locality: Costa Rica, Puntarenas, Río Bellavista, ca. 1.5 km NW Las Alturas, 8.951°N, 82.846°W; NMNH; ♂; subgenus *Nosopus*, *Mexicanum* Group]. —Armitage et al., 2015b:9 [checklist]. —Armitage and Cornejo, 2015:198 [checklist].

**Distribution.** Costa Rica, Panama.

***frontale*** (Banks), 1901:367 [Type locality: Mexico, Veracruz, Jalapa; MCZ; ♂; in *Eremopsyche*]. —Flint, 1967c:24 [♂; to *Lepidostoma*]. —Bueno-Soria and Padilla, 1981:391 [distribution]. —Bueno-Soria and Flint, 1978:214 [distribution]. —Weaver, 1988:49 [♂; subgenus *Nosopus*, *Mexicanum* Group]. —Rojas-Ascencio, et al., 2002:377 [distribution].

**Distribution.** Mexico.

***griseum*** (Banks), 1911:357 [Type locality: USA, New York, Fulton County, Woodworth’s Lake; MCZ; ♂; in *Phanopsyche*]. —Armitage et al., 2015a:8 [distribution]. —Armitage et al., 2015b:9 [checklist]. —Armitage and Cornejo, 2015:198 [checklist].

**Distribution.** Canada, Panama, U.S.A.

***heveli*** Flint and Bueno-Soria, 1977:381 [Type locality: Guatemala, Quiche, El Quiche, 7.3 km S Chichicastenango, 14°54'N, 91°07'W; NMNH; ♂]. —Weaver 1988:49 [♂; subgenus *Nosopus*, *Mexicanum* Group].

**Distribution.** Guatemala.

***ibarrai*** Bueno-Soria, Santiago-Fragoso, and Barba-Álvarez, 2004:481 [Type locality: Mexico, Oaxaca, Santa María Yavesia, 17°13'36"N, 96°25'35"W, el. 1920 m; CNIN; ♂].

**Distribution.** Mexico.

***ixtlahuaca*** Bueno-Soria, Santiago-Fragoso, and Barba-Álvarez, 2001:156 [Type locality: Mexico, Hidalgo, Ixtlahuaco, el. 1320 m; CNIN; ♂; in subgenus *Nosopus*].

**Distribution.** Mexico.

***knulli*** Ross, 1946:280 [Type locality: United States, Arizona, Oak Creek Canyon; INHS; ♂]. —Flint, 1967d:175 [distribution]. —Flint and Bueno-Soria, 1977:381 [diagnosis; distribution]. —Bueno-Soria and Flint, 1978:214 [distribution]. —Weaver, 1988:44 [♂; ♀; larva; *Unicolor* Group]. —Rojas-Ascencio, et al., 2002:377 [distribution]. —Bueno-Soria et al., 2007:33 [distribution].

—*leechi* Denning, 1962a:37 [Type locality: Mexico, 1 mile west of La Marquesa (34 kilometers west of Mexico, D.F.); CAS; ♂]. —Flint and Bueno-Soria, 1977:381 [to synonymy].

**Distribution.** Mexico, U.S.A.

***lacinatum*** Flint, 1967d:175 [Type locality: Mexico, Durango, 10 miles west of El Salto; CNC; ♂]. —Denning 1973:142 [♂ variation, distribution]. —Flint and Bueno-Soria, 1977:382 [diagnosis; distribution]. —Bueno-Soria and Flint, 1978:214 [distribution]. —Weaver, 1988:50 [♂; ♀; subgenus *Nosopus*, *Mexicanum* Group].

**Distribution.** Mexico, U.S.A.

***leonilae*** Bueno-Soria and Contreras, 1986:209 [Type locality: Mexico, Nuevo León, Santiago, Potrero Redondo; IBUNAM; ♂]. —Weaver, 1988:50 [♂; subgenus *Nosopus*, *Mexicanum* Group].

**Distribution.** Mexico.

***mexicanum*** (Banks), 1901:367 [Type locality: Mexico, Tacubaya; MCZ; ♀; in *Olemira*]. —Ross, 1946:288 [to *Lepidostoma*]. —Flint, 1967c:24 [♀]. —Flint and Bueno-Soria, 1977:382 [diagnosis; distribution]. —Bueno-Soria and Flint, 1978:214 [distribution]. —Weaver, 1988:51 [♂; ♀ lectotype, larva; subgenus *Nosopus*, *Mexicanum* Group]. —Holzenthal, 1988c:75 [distribution]. —Aguila, 1992:545 [distribution]. —Armitage et al., 2015b:9 [checklist]. —Armitage and Cornejo, 2015:198 [checklist].

—*bispinosa* (Ulmer), 1911:25 [Type locality: Costa Rica, San José; ZMHU; ♂; in *Atomyiodes*]. —Denning, 1962a:39 [♂; redescription; to *Lepidostoma*]. —Flint, 1967d:175 [variation, distribution]. —Flint and Bueno-Soria, 1977:382 [to synonymy].

—*alexanderi* Denning, 1962a:37 [Type locality: United States, Arizona, Cochise County, Southwestern Research Station, Chiricahua Mountains; CAS; ♂]. —Flint, 1967d:175 [as synonym of *bispinosa*].

**Distribution.** Costa Rica, Guatemala, Mexico, Panama, U.S.A.

***oaxacense*** Bueno-Soria and Contreras, 1986:208 [Type locality: Mexico, Oaxaca, Carretera No. 175, Portillo del Rayo; IBUNAM; ♂]. —Weaver, 1988:52 [♂; subgenus *Nosopus*, *Mexicanum* Group].

**Distribution.** Mexico.

***pluviale*** (Milne), 1936:117 [Type locality: United States, Colorado, Creede; MCZ; ♂; in *Olemira*]. —Weaver 1988:81 [♂; ♀; larva; distribution]. —Weaver, 2002: 182 [*Lepidostoma ferox* branch]

—*rhino* Ross, 1946:276 [Type locality: Mexico, Baja California, R. Santo Domingo, Rancho San Antonio; INHS; ♂]. —Ross, 1951a:75 [distribution]. —Denning, 1964:134 [checklist]. —Bueno-Soria and Flint, 1978:214 [distribution]. —Weaver, 1988:81 [to synonymy].

—*veleda* Denning, 1948c:20 [Type locality: United States, Wyoming, Albany County, Snowy Range, near Centennial; CAS; ♂]. —Weaver, 1988:81 [to synonymy].

**Distribution.** Canada, Mexico, U.S.A.

***polylepidum*** Holzenthal and Strand, 1992:494 [Type locality: Costa Rica, San José, Río Parrita Chiquito, rt 12, 6.5 km SW jct rt 2, 9.703°N, 83.970°W; NMNH; ♂; subgenus *Nosopus*, *Mexicanum* Group]. —Armitage et al., 2015a:8 [distribution]. —Armitage et al., 2015b:9 [checklist]. —Armitage and Cornejo, 2015:199 [checklist].

**Distribution.** Costa Rica, Panama.

***quila*** Bueno-Soria and Padilla-Ramírez, 1981:390 [Type locality: Mexico, Mexico, Lagunas de Zempoala; IBUNAM; ♂]. —Weaver, 1988:52 [♂; subgenus *Nosopus*, *Mexicanum* Group].

**Distribution.** Mexico.

***rectangulare*** Flint, 1967d:176 [Type locality: Mexico, Durango, 10 miles west of El Salto; CNC; ♂]. —Bueno-Soria and Flint, 1978:214 [distribution]. —Weaver, 1988:53 [♂; subgenus *Nosopus*, *Mexicanum* Group].

**Distribution.** Mexico.

***reimoseri*** Flint and Bueno-Soria, 1977:383 [Type locality: Costa Rica, Cartago, Volcan Irazu; NMW; ♂]. —Weaver, 1988:53 [♂; subgenus *Nosopus*, *Mexicanum* Group]. —Holzenthal, 1988c:75 [distribution]. —Armitage et al., 2015a:8 [distribution]. —Armitage et al., 2015b:9 [checklist]. —Armitage and Cornejo, 2015:199 [checklist].

**Distribution.** Costa Rica, Panama.

***steinhauseri*** Flint and Bueno-Soria, 1977:384 [Type locality: El Salvador, Santa Ana, Cerro Miramundo; NMNH; ♂]. —Weaver, 1988:53 [♂; subgenus *Nosopus*, *Mexicanum* Group]. —Chamorro-Lacayo et al., 2007:44 [checklist].

**Distribution.** El Salvador, Nicaragua.

***talamancense*** Flint and Bueno-Soria, 1977:386 [Type locality: Costa Rica, Cartago, Ojo de Agua, route 2 km 75; NMNH; ♂]. —Weaver, 1988:54 [♂; larva; subgenus *Nosopus*, *Mexicanum* Group]. —Holzenthal, 1988c:75 [distribution].

**Distribution.** Costa Rica.

***tapanti*** Holzenthal and Strand, 1992:496 [Type locality: Costa Rica, Cartago, Reserva Tapantí, Río Dos Amigos and falls, ca. 6 km (rd) NW tunnel, 9.704°N, 83.783°W; NMNH; ♂; subgenus *Nosopus*, *Mexicanum* Group]. —Armitage et al., 2015b:9 [checklist]. —Armitage and Cornejo, 2015:199 [checklist].

**Distribution.** Costa Rica, Panama.

***textor*** Bueno-Soria, Santiago-Fragoso, and Barba-Álvarez, 2006:249 [replacement name for *Lepidostoma weaveri* Bueno-Soria, Santiago-Fragoso, and Barba-Álvarez, 2004:481, preoccupied by *Lepidostoma weaveri* Harris, 1986:36]. [Type locality: Mexico, Estado de Mexico, Temascaltepec, Real de Arriba, el. 1740 m; CNIN; ♂].

**Distribution.** Mexico.

***xolotl*** Holzenthal and Strand, 1992:497 [Type locality: Mexico, Nayarit, 49.4 mi E Venado; NMNH; ♂; subgenus *Nosopus*, *Mexicanum* Group].

**Distribution.** Mexico.

### Family Leptoceridae

The Leptoceridae, or long-horned caddisflies, are cosmopolitan in distribution and many of the approximately 1,800 species and 47 genera are found in warmer regions. The family is now divided into four subfamilies (Malm and Johanson 2011), of which three occur in the Neotropics: Triplectidinae (*Hudsonema*, *Notalina*, *Triplectides*), Grumichellinae (*Amazonatolica*, *Atanatolica*, *Grumichella*, *Osflintia*), and Leptocerinae (*Achoropsyche*, *Amphoropsyche*, *Brachysetodes*, *Nectopsyche*, *Neoathripsodes*, *Oecetis*, *Setodes*, *Triaenodes*). *Mystacides*, a primarily Northern Hemisphere leptocerine, is known from Baja California, Mexico, and is thus incuded in this catalog, as are a few species of Nearctic *Triaenodes*. Another leptocerine genus, *Setodes*, in known from the Neotropics from two fossil species in Donimican amber and a recently described extant species from the Domincan Republic; otherwise, it has a few species in eastern North America and is hyper diverse in the Oriental Region (Schmid, 1987). Morse (1981) presented the first cladistic phylogeny of the family-group taxa of Leptoceridae. Morse’s classification was largely corroborated by the comprehensive study by Malm and Johanson (2011) using gene sequence data; their classification is adopted here. Two hundred twenty-one extant and two fossil species are known from the Neotropics, where many new species, especially in *Nectopsyche* and *Oecetis*, undoubtedly occur.

Larvae of many genera are well known (Hickin 1967, Lepneva 1971, Wiggins 1996), including most of the Neotropical genera (Holzenthal 1986a, 1986b, 1986d, 1988a, 1988b). They are found in a wide variety of habitats, including large and small rivers, cascades, and even semi-terrestrial situations, from both lowland and highland areas. Larvae construct tubular cases from a wide variety of plant and mineral materials or entirely from silk. A few utilize the discarded cases of other Trichoptera. They feed generally as detritivores or omnivores, but some are specialized as scrapers of periphyton or as predators of other arthropods.

#### Genus *Achoropsyche* Holzenthal [1]

*Achoropsyche* Holzenthal, 1984:181 [Type species: *Setodes Aunctata* Navás, 1916a, by monotypy].

This monotypic genus, endemic to the Neotropics, was erected for a single, widespread South American species (Holzenthal 1984). Larvae of the species are unknown. Nothing can be said of the adult biology except that they are usually collected at lights along large, lowland rivers.

***duodecimpunctata*** (Navás), 1916a:33 [Type locality: Brazil, Nova Friburgo; collection Navás, now lost?; ♂; in *Setodes*]. —Flint, 1972b:244 [to *Brachysetodes*]. —Flint, 1974c:120 [♂]. —Flint, 1982c:47 [distribution]. —Holzenthal, 1984:182 [♂; ♀; distribution; to *Achoropsyche*]. —Flint 1992d:71 [distribution]. —Flint, 1996b:418 [distribution]. —Almeida and Marinoni, 2000:349 [distribution; biology]. —Muñoz-Quesada, 2000:278 [checklist]. —Cohen, 2004:77 [distribution]. —Blahnik et al., 2004:5 [distribution]. —Paprocki et al., 2004:12 [checklist]. —Angrisano and Sganga, 2007:44 [♂; distribution]. —Dumas et al., 2009:368 [distribution]. —Calor, 2011:321 [checklist]. —Nogueira and Cabette, 2011:351 [distribution]. —Nogueira et al., 2011:176 [community ecology, distribution]. —Manzo et al., 2014:167 [distribution]. —Paprocki and França, 2014:56 [checklist]. —Quinteiro et al., 2014:233 [distribution]. —Moretto and Bispo, 2015:126 [distrtibution].

**Distribution.** Argentina, Bolivia, Brazil, Colombia, Ecuador, Guyana, Paraguay, Peru, Suriname, Uruguay, Venezuela.

#### Genus *Amazonatolica* Holzenthal and Pes [1]

*Amazonatolica* Holzenthal and Pes, 2004:3 [Type species: *Amazonatolica hamadae* Holzenthal and Pes, 2004, original designation].

This is another monotypic genus endemic to the Neotropics. Its single species, *Amazonatolica hamadae*, is known only from around the region of Manaus, Brazil, but it is likely more widespread. Holzenthal and Pes (2004) described all of the life history stages and biology. The larvae live in clear, fast flowing, black water streams, where they cling to long leaves of aquatic macrophytes waving in the current; their cases are made entirely of semi-transparent silk.

***hamadae*** Holzenthal and Pes, 2004:6 [Type locality: Brazil, Amazonas, Manaus, Reserva Florestal Adolpho Ducke, 02°57'S, 59°57'W, Igarapé Barro Branco (sede); INPA; ♂; ♀; larva; pupa; habitat, biology]. —Ribeiro et al., 2009:34 [list of types]. —Nogueira and Cabette, 2011:351 [distribution]. —Paprocki and França, 2014:56 [checklist].

**Distribution.** Brazil.

#### Genus *Amphoropsyche* Holzenthal [16]

*Amphoropsyche* Holzenthal, 1985:255 [Type species: *Brachysetodes insularis* Flint, 1968b, original designation]. —Holzenthal, 1985:255 [revision]. —Holzenthal and Rázuri-Gonzales, 2011:63 [key to species].

The 16 species and subspecies of the endemic South American genus *Amphoropsyche*, established for the Dominican species *Brachysetodes insularis* Flint by Holzenthal (1985), are very rare and local in occurrence in the northern Andes and on several islands of the Lesser Antilles. The described species, known from only a very few individuals, probably represent a small fraction of the actual diversity.

Adults and larvae frequent small forest streams in mountainous areas. Little is known of the larval biology. Flint (1968b) found them scattered in the sand bottom of a pool of a small stream. Larvae build cases of sand grains. Adults do not fly to lights readily and are best collected by beating riparian vegetation or with Malaise traps. Adult males contain interesting pheromone dispersing structures on the genitalia (Botosaneanu 1991b).

***aragua*** Holzenthal, 1985:261 [Type locality: Venezuela, Aragua, road between Maracay and Choroni; UCV; ♂].

**Distribution.** Venezuela.

***ayura*** Holzenthal, 1985:264 [Type locality: Colombia, Antioquia, Quebrada la Ayurá, above Envigado, S of Medellín; NMNH; ♂; ♀]. —Flint, 1991:90 [♂; distribution]. —Muñoz-Quesada, 2000:279 [checklist].

**Distribution.** Colombia.

***cauca*** Holzenthal, 1985:268 [Type locality: Colombia, Antioquia, 12 km N of Fredonia; NMNH; ♂]. —Flint, 1991:90 [♂; distribution]. —Muñoz-Quesada, 2000:279 [checklist].

**Distribution.** Colombia.

***choco*** Holzenthal, 1985:264 [Type locality: Colombia, Chocó, km 130, 86 E Quibdo; NMNH; ♂]. —Muñoz-Quesada, 2000:279 [checklist].

**Distribution.** Colombia.

***flinti*** Holzenthal, 1985:258 [Type locality: Colombia, Antioquia, 27 km W of Medellín, road to San Jerónimo; NMNH; ♂]. —Flint, 1991:90 [♂; distribution]. —Muñoz-Quesada, 2000:279 [checklist].

**Distribution.** Colombia.

***insularis*** (Flint), 1968b:69 [Type locality: Dominica, Pont Casse, 1.6 miles west; NMNH; ♂; ♀; larva; pupa; in *Brachysetodes*]. —Holzenthal, 1985:255 [♂; ♀; to *Amphoropsyche*]. —Holzenthal, 1986b:255 [larva; pupa]. —Flint and Sykora, 1993:50 [checklist]. —Botosaneanu 1994a:51 [distribution]. —Botosaneanu and Thomas, 2005:53 [distribution].

**Distribution.** Dominica, Guadalupe, Martinique.

***janstockiana*** Botosaneanu, 1990b:319 [Type locality: Saint Vincent; BMNH; ♂; ♀?]. —Botosaneanu, 1991b:66 [biology]. —Flint and Sykora, 1993:50 [checklist].

**Distribution.** Saint Vincent, Mustique [?].

***napo*** Holzenthal, 1985:258 [Type locality: Ecuador, Napo, Sebundoy, 0°30'N, 77°30'W; NMNH; ♂].

**Distribution.** Ecuador.

***quebrada*** Holzenthal, 1985:266 [Type locality: Colombia, Antioquia, Quebrada del Cebolla, W of La Fe, 25 E of Medellín; NMNH; ♂; ♀]. —Flint, 1991:90 [♂; distribution]. —Muñoz-Quesada, 2000:279 [checklist].

**Distribution.** Colombia.

***real*** Holzenthal and Ríos-Touma, 2016:62 [Type locality: Ecuador, Morona-Santiago, Macas, small gravel stream (Wallace/Real property), 02.20299°S, 078.08539°W, el. 1076 m; UMSP; /male/].

**Distribution.** Ecuador

***refugia*** Holzenthal, 1985:260 [Type locality: Venezuela, Distrito Federal, Estación Experimental Bajo Seco, ca. 15 km NE of Colonia Tovar; NMNH; ♂; ♀].

**Distribution.** Venezuela.

***spinifera*** Holzenthal, 1986b:251 [Type locality: Bolivia, Yungas La Paz, Pte. Mururata to Cusilloni; NMNH; ♂; ♀]. —Flint, 1996b:418 [distribution].

**Distribution.** Bolivia, Peru.

***stellata*** Holzenthal, 1985:260 [Type locality: Colombia, Risaralda, Termales de Santa Rosa de Cabal; NMNH; ♂]. —Muñoz-Quesada, 2000:279 [checklist].

**Distribution.** Colombia.

***tandayapa*** Holzenthal and Rázuri-Gonzales, 2011:61 [Type locality: Ecuador, Pichincha, 2.3 km S Tandayapa, 1800 m; NMNH; ♂].

**Distribution.** Ecuador.

***woodruffi multispinosa*** Botosaneanu, in Botosaneanu and Alkins-Koo, 1993:36 [Type locality: Trinidad, two first order streams at “La laja”, catchment of Rio Guanapo; ZMUA; ♂; ♀]. —Botosaneanu and Sakal, 1992:204 [distribution; ecology]. —Flint, 1996a:102 [distribution; to subspecific status].

**Distribution.** Trinidad.

***woodruffi woodruffi*** Flint and Sykora, 1993:59 [Type locality: Grenada, Parish St. Johns, Concord Falls; FSCA; ♂]. —Flint and Sykora, 1993:50 [checklist]. —Flint, 1996a:102 [distribution].

**Distribution.** Grenada, Venezuela.

#### Genus *Atanatolica* Mosely [20]

*Atanatolica* Mosely, 1936a:123 [Type species: *Mystacides brasilianus* Brauer, 1865, original designation]. —Holzenthal, 1988b:74 [♂; ♀; larva; pupa; revision; phylogeny; distribution].

This is yet another endemic Neotropical leptocerid genus, one of four endemic genera in the subfamily Grumichellinae endemic to tropical America. Most of the 20 Neotropical species in the genus were described by Holzenthal (1988b). Two species groups are known from the Neotropics, the *dominicana* Group from Central America, northern South America, and the Lesser Antilles and the *brasiliana* Group from southeastern Brazil (Holzenthal 1988b).

Holzenthal (1988b) discussed the biology of a Costa Rican species, which appears to be typical for the genus. Larvae frequent waterfalls, cascades, and the fast flowing areas of boulder-strewn streams in forested, mountainous areas. Cases, constructed of mineral fragments or entirely of darkened silk, are long, slender, and tapering. In a few species, mineral or plant fragments are placed laterally to form wide flanges (Botosaneanu 1974, Holzenthal 1988b). Larvae are semi-terrestrial and live in the spray and splash zones of their habitats. Morphology of the mandibles and other mouth parts suggest that they feed by scraping the abundant periphyton growing in the habitat. Adults are diurnal and form swarms above the larval habitat. They are best collected by passing a long handled net through the swarm or by netting a mated pair that has landed on nearby vegetation. Costa and Calor (2014) collected abundant specimens with Malaise traps and noticed distinct peaks of activity over 4 years of sampling. Abundance of indivuduals was greatest during the dry season (May-October) in NE Brazil.

***acuminata*** Holzenthal, 1988b:77 [Type locality: Ecuador, Zamora-Chinchipe, Zamora (30 km W); NMNH; ♂].

**Distribution.** Ecuador.

***aurea*** Holzenthal, 1988b:77 [Type locality: Colombia, Antioquia, 24 km NW Medellín, rd. to San Jerónimo; NMNH; ♂; ♀]. —Flint, 1991:92 [♂; distribution]. —Muñoz-Quesada, 2000:279 [checklist].

**Distribution.** Colombia.

***bonita*** Costa and Calor, 2014:195 [Type locality: Brazil, Bahia, Camacan, RPPN Serra Bonita, stream 3, 15°23'02"S, 39°34'10"W, 806 m; MZUSP; ♂; ♀; biology]. —Dias et al., 2015:376 [distribution].

**Distribution.** Brazil.

***botosaneanui*** Flint, 1981a:31 [Type locality: Venezuela, Aragua, Dos Riitos, 6 km N Rancho Grande; NMNH; ♂; ♀]. —Botosaneanu and Flint, 1982:21 [larva; case]. —Holzenthal, 1988b:78 [♂; ♀; larva; case].

**Distribution.** Venezuela.

***brasiliana*** (Brauer), 1865:418 [Type locality: Brazil, Rio de Janeiro; NMW; ♂; in *Mystacides*]. —Ulmer, 1905b:72 [to *Notanatolica*]. —Mosely, 1936a:123 [to *Atanatolica*]. —Flint, 1966a:10 [♂ lectotype]. —Holzenthal, 1988b:78 [redescription]. —Paprocki et al., 2004:12 [checklist]. —Dumas et al., 2009:368 [distribution]. —Calor, 2011:321 [checklist]. —Paprocki and França, 2014:56 [checklist].

**Distribution.** Brazil.

***caldas*** Holzenthal, 1988b:79 [Type locality: Colombia, Caldas, 5 km W Termales de Ruiz; NMNH; ♂]. —Muñoz-Quesada, 2000:279 [checklist].

**Distribution.** Colombia.

***choco*** Holzenthal, 1988b:80 [Type locality: Colombia, Choco, km 130, 86 E Quibdo; NMNH; ♂; ♀]. —Muñoz-Quesada, 2000:279 [checklist].

**Distribution.** Colombia.

***cotopaxi*** Holzenthal, 1988b:80 [Type locality: Ecuador, Cotopaxi, Pujili (34 km W); NMNH; ♂].

**Distribution.** Ecuador.

***dominicana*** Flint, 1968b:71 [Type locality: Dominica, Pont Casse, 0.4 E; NMNH; ♂; ♀; larva; pupa]. —Malicky, 1983:264 [distribution]. —Holzenthal, 1988b:81 [♂; ♀; larva; case]. —Flint and Sykora, 1993:50 [checklist]. —Botosaneanu, 1994a:51 [distribution]. —Botosaneanu and Thomas, 2005:53 [distribution].

**Distribution.** Dominica, Guadelupe, Martinique.

***flinti*** Holzenthal, 1988b:81 [Type locality: Brazil, Rio de Janeiro, Nova Friburgo, Serra; MZSP; ♂; ♀]. —Paprocki et al., 2004:12 [checklist]. —Dumas et al., 2009:368 [distribution]. —Paprocki and França, 2014:56 [checklist].

**Distribution.** Brazil.

***manabi*** Holzenthal, 1988b:83 [Type locality: Ecuador, Manabi, Santo Domingo de los Colorados (79 km W); NMNH; ♂].

**Distribution.** Ecuador.

***moselyi*** Denning and Holzenthal, in Holzenthal, 1988b:83 [Type locality: Costa Rica, Puntarenas, Río Jaba, Las Cruces; UCD; ♂].

**Distribution.** Costa Rica.

***muyupampa*** Holzenthal, 1988b:84 [Type locality: Bolivia, Chuquisaca, Incahuasi, ♀ Muyupampa; NMNH; ♂; ♀].

**Distribution.** Bolivia.

***nigra*** Holzenthal, 1988b:84 [Type locality: Colombia, Caldas, 3.7 E Termales de Ruiz; NMNH; ♂; ♀]. —Muñoz-Quesada, 2000:279 [checklist].

**Distribution.** Colombia.

***nivea*** Holzenthal, 1988b:85 [Type locality: Colombia, Risaralda, Termales de Santa Rosa de Cabal; NMNH; ♂; ♀]. —Muñoz-Quesada, 2000:279 [checklist].

**Distribution.** Colombia.

***nordestina*** Henriques-Oliveira and Santos, 2014:538 [Type locality: Brazil, Ceará, Ubajara, Parque Nacional de Ubajara, trilha Araticum, Rio das Minas, 03°49'58"S, 40°53'53"W, el. 420 m; CZMA; ♂; ♀; larva; pupa; distribution; biology].

**Distribution.** Brazil.

***panamensis*** Holzenthal, 1988b:85 [Type locality: Panama, Canal Zone, Pipeline Rd, Río Agua Salud; NMNH; ♂]. —Aguila, 1992:544 [distribution]. —Armitage et al., 2015b:8 [checklist]. —Armitage and Cornejo, 2015:197 [checklist].

**Distribution.** Panama.

***penai*** Holzenthal, 1988b:86 [Type locality: Bolivia, Cochabamba, Río Ronquito, rd. to Villa Tunari, Chapare; NMNH; ♂; ♀].

**Distribution.** Bolivia.

***quechua*** Henriques-Oliveira and Santos, 2014:543 [Type locality: Peru, Cuzco, Puente Inambari, 13°10'53"S, 70°23'06"W, el. 365 m; MHNJP; ♂; ♀; larva].

**Distribution.** Peru.

***zongo*** Holzenthal, 1988b:86 [Type locality: Bolivia, La Paz, quebradas del Río Zongo; NMNH; ♂; ♀].

**Distribution.** Bolivia.

#### Genus *Brachysetodes* Schmid [10]

*Brachysetodes* Schmid, 1955a:134 [Type species: *Brachysetodes trifidus* Schmid, 1955a, original designation]. —Holzenthal, 1986a:409 [♂; ♀; larva; pupa; revision, phylogeny, distribution].

This is yet another endemic Neotropical genus of 10 species, but the only one restricted to the Chilean Subregion and the only leptocerine widely occurring in that Subregion. Holzenthal (1986a) described the immature stages, but little is known of their biology except that they frequent small to moderate sized, cool, clear, gravel-bottomed streams. They construct slightly curved and tapered cases of sand grains.

***bifidus*** Schmid, 1955a:135 [Type locality: Chile, Santiago, El Manzano; NMNH; ♂]. —Flint 1974e:90 [checklist]. —Holzenthal, 1986a:417 [♂; ♀; distribution].

**Distribution.** Chile.

***bifurcatus*** Flint, 1983a:69 [Type locality: Chile, Cautín, Fundo el Coigüe, 27 km NE Villarrica; NMNH; ♂]. —Holzenthal, 1986a: 414 [♂; distribution].

**Distribution.** Chile.

***extensus*** Schmid, 1958b:204 [Type locality: Chile, Arauco, Pichinahuel; NMNH; ♂]. —Flint 1974e:90 [checklist]. —Holzenthal, 1986a:414 [♂; ♀; distribution].

**Distribution.** Argentina, Chile.

***forcipatus*** Schmid, 1964:334 [Type locality: Chile, Bio-Bio, Santa Barbara, Los Angeles; NMNH; ♂]. —Flint 1974e:90 [checklist]. —Holzenthal, 1986a:415 [♂; ♀; distribution].

**Distribution.** Argentina, Chile.

***major*** Schmid, 1958b:204 [Type locality: Chile, Linares, Hacienda San-Manuel; NMNH; ♂]. —Flint 1974e:90 [checklist]. —Holzenthal, 1986a:417 [♂; ♀; distribution]. —Miserendino and Brand, 2007:312 [biology].

**Distribution.** Argentina, Chile.

***nublensis*** Flint, 1969b:511 [Type locality: Chile, Ñuble, Recinto; NMNH; ♂]. —Flint 1974e:90 [checklist]. —Holzenthal, 1986a:415 [♂; ♀; distribution].

**Distribution.** Chile.

***quadrifidus*** Schmid, 1955a:136 [Type locality: Chile, Isla de Chilóe, Aucar; NMNH; ♂]. —Flint 1974e:90 [checklist]. —Holzenthal, 1986a:415 [♂; ♀; distribution]. —Brand and Miserendino, 2011b:143 [biology]. —Brand et al., 2012:90 [biology]. —Brand and Miserendino, 2014:6 [community ecology].

**Distribution.** Argentina, Chile.

***spinosus*** Schmid, 1958b:203 [Type locality: Chile, Arauco, Pichinahuel; NMNH; ♂]. —Flint 1974e:90 [checklist]. —Holzenthal, 1986a:416 [♂; ♀; distribution].

**Distribution.** Chile.

***trifidus*** Schmid, 1955a:135 [Type locality: Santiago, El Manzano; NMNH; ♂]. —Flint 1974e:90 [checklist]. —Holzenthal, 1986a:416 [♂; ♀; distribution].

**Distribution.** Chile.

***tripartitus*** Schmid, 1964:333 [Type locality: Chile, O’Higgins, Graneros; NMNH; ♂]. —Flint 1974e:90 [checklist]. —Holzenthal, 1986a:416 [♂; ♀; distribution].

**Distribution.** Chile.

#### Genus *Grumichella* Müller [13]

*Grumichella* Müller, 1879a:407 [Type species: *Grumichella rostrata* Thienemann, 1905b, first included species]. —Holzenthal, 1988b:88 [♂; ♀; larva; pupa; revision, phylogeny, distribution]. —Calor et al., 2016:137 [revision, phylogeny].

*Leptocellodes* Ulmer, 1911:21 [Type species: *Leptocellodes flaveola* Ulmer, 1911, original designation]. —Ulmer, 1955:499 [to synonymy].

*Grumichella* is another member of the subfamily Grumichellinae endemic to and widely distributed over tropical South America (Morse 1981). Holzenthal (1988b) illustrated cases of several unassociated species, and suggested that the genus was likely more species rich than previously known. This supposition was recently corroborated by Calor et al., (2016) who decribed 9 new species from Brazil, Peru, and Venezuela, bringing the genus total to 13 species.

Müller (1879a, p. 40) introduced the term “*grumichinha*” for a leptocerid for which he later proposed the new generic name *Grumichella*. Flint et al. (1999a) surpressed “*grumichinha*” as an unavailable vernacular name.

Larvae live on rocks in fast flowing streams in montane areas and have various behavioral and morphological adaptations for maintaining their hold in currents (Holzenthal 1988b). Larvae of most species construct cases entirely of silk, but some others incorporate substantial mineral material.

***aequiunguis*** Flint, 1983a:68 [Type locality: Argentina, Pcia. Misiones, Arroyo Piray Mini, rt. 17, W Dos Hermanas; NMNH; ♂]. —Holzenthal, 1988b:91 [♂; ♀; larva; pupa; case; distribution]. —Blahnik et al., 2004:5 [distribution]. —Paprocki et al., 2004:12 [checklist]. —Paprocki and França, 2014:57 [checklist]. —Calor et al., 2016:140 [redescription].

**Distribution.** Argentina, Brazil, Paraguay.

***blahniki*** Calor and Holzenthal, in Calor et al., 2016:147 [Type locality: Peru, Pauractambo, Pte. San Pedro, c. 50 km NW Pilcopata; NMNH; ♂; female].

**Distribution.** Peru.

***boraceia*** Calor and Holzenthal, in Calor et al., 2016:148 [Type locality: Brazil, São Paulo, Salesópolis, Rio Claro, 23°38'08"S, 45°49'55"W, el. 800 m; MZUSP; ♂; ♀, larva; case; pupa].

**Distribution.** Brazil.

***cressae*** Calor and Holzenthal, in Calor et al., 2016:149 [Type locality: Venezuela, Lara, P.N. Dinira, Quebrada Buenos Aires, 9°36'24"N, 70°04'11"W, el. 1850 m; UMSP; ♂; female].

**Distribution.** Venezuela.

***flaveola*** (Ulmer), 1911:22 [Type locality: Bolivia, Yungas, Bogota; ZMHU; ♂; in *Leptocellodes*]. —Mosely, 1949:41 [redescription]. —Holzenthal, 1988b:91 [♂; ♀; larva; pupa; case; distribution]. —Flint, 1991:93 [♂; distribution]. —Flint, 1996b:417 [distribution]. —Muñoz-Quesada, 2000:279 [checklist]. —Medellín et al., 2004:201 [distribution; biology]. —Calor et al., 2016:142 [redescription].

—*poujadi* (Navás), 1927b:73 [Type locality: Ecuador, Loja; MNHNP; ♂; in *Notanatolica*]. —Mosely, 1936a:107 [to synonymy].

**Distribution.** Argentina, Bolivia, Colombia, Ecuador, Peru, Venezuela.

***jureia*** Calor and Holzenthal, in Calor et al., 2016:150 [Type locality: Brazil, São Paulo, Estação Ecológica Juréia-Itatins, Córrego próximo à sede; MZUSP; ♂; ♀; case; pupa].

**Distribution.** Brazil.

***leccii*** Calor and Holzenthal, in Calor et al., 2016:152 [Type locality: Brazil, São Paulo, Jundiai, Serra do Japi, P.S. 11, stream before reservoir, 23°14'30"S, 46°57'16"W; MZUSP; ♂; ♀; larva; larval case; pupa; pupal case].

**Distribution.** Brazil.

***muelleri*** Calor and Holzenthal, in Calor et al., 2016:153 [Type locality: Brazil, Santa Catarina, Parque Ecológico Spitzkopf, confl. Rio Ouro & Rio Caeté, 27°00'21"S, 49°06'42"W; MZUSP; ♂; female].

**Distribution.** Brazil.

***paprockii*** Calor and Holzenthal, in Calor et al., 2016:155 [Type locality: Brazil, Minas Gerais, Córrego da Serra de Ouro Fino, Vale do Tropeiro, 20°12'22"S, 43°38'35"W, el. 1000 m; MZUSP; ♂; female].

**Distribution.** Brazil.

***parati*** Calor and Holzenthal, in Calor et al., 2016:156 [Type locality: Brazil, Rio de Janeiro, Parati, Riacho Perequê-açu, Sitio Cachoeira Grande, 23°13'14"S, 44°47'24"W, el. 120 m; MZUSP; ♂; female].

**Distribution.** Brazil.

***pulchella*** (Banks), 1910:160 [Type locality: Colombia, Cañon del Monte Tolima; MCZ; ♂; in *Leptocella*]. —Ulmer, 1955:499 [in *Leptocellodes*]. —Flint, 1967c:22 [♂]. —Holzenthal, 1988b:93 [to *Grumichella*]. —Muñoz-Quesada, 2000:279 [checklist]. —Calor et al., 2016:144 [redescription].

**Distribution.** Colombia.

***rostrata*** Thienemann 1905b:537 [Type locality: no type nor type depository designated, but name based on material probably from Brazil, Santa Catarina, Gruta dos Macacos, near Blumenau according to Holzenthal 1988b; case; pupa]. —Thienemann, 1909:41, 42, 125 [larva; pupa]. —Holzenthal 1988b:93 [♂; ♀; larva; pupa]. —Blahnik et al., 2004:5 [distribution]. —Paprocki et al., 2004:12 [checklist]. —Dumas et al., 2009:368 [distribution]. —Calor, 2011:322 [checklist]. —Dumas and Nessimian, 2012:16 [distribution]. —Paprocki & França, 2014:57 [distribution]. —Paprocki and França, 2014:57 [checklist]. —Dias et al., 2015:376 [distribution]. —Moretto and Bispo, 2015:126 [distrtibution]. —Calor et al., 2016:145 [redescription].

**Distribution.** Brazil.

***trujilloi*** Calor and Holzenthal, in Calor et al., 2016:158 [Type locality: Venezuela, Trujillo, Quebrada Potrerito, 7.5 km NE Bocono, 09°16'26"N, 70°13'06"W, el. 1530 m; UMSP; ♂; female].

**Distribution.** Venezuela.

#### Genus *Hudsonema* Mosely [1]

*Hudsonema* Mosely, 1936a:110 [Type species: *Tetracentron amabile* McLachlan, 1868, original designation]. —Holzenthal, 1986d:268 [phylogeny, biogeography].

*Condocerus* Neboiss, 1977:140 [Type species: *Condocerus paludosis* Neboiss, 1977, original designation]. —Malm and Johanson, 2011: 16 [to synonymy].

Five species are now known in this genus, two from Australia, two from New Zealand, and one, *Hudsonema flaminii*, from Chilean South America. Holzenthal (1986d) hypothesized that this distribution conformed to a New Zealand-South American trans-Antarctic biogeographical track. Immature stages of the South American species were described by Holzenthal (1986d), but nothing is known of their biology; larvae can be very abundant in shallow, still areas of streams.

***flaminii*** (Navás), 1926:335 [Type locality: Chile, Lonquimay; MZBS; ♂; in *Triplectides*]. —Mosely, 1936a:111 [♂; to *Hudsonema*]. —Schmid, 1949a:361 [♀; redescription]. —Flint 1974e:90 [checklist]. —Holzenthal, 1986d:269 [♂; ♀; larva; pupa; redescription; distribution; biogeography]. —Cohen, 2004:78 [distribution]. —Brand and Miserendino, 2011a:35 [biology; habitat]. —Brand et al., 2012:90 [biology]. —Brand and Miserendino, 2014:6 [community ecology].

—*discolor* (Navás), 1932d:83 [Type locality: Chile, Marga-Marga; type depository not stated; ♂; in *Triplectides*]. —Mosely, 1936a:114 [to *Hudsonema*]. —Schmid, 1949a:362 [♀; redescription]. —Flint, 1974e:84 [to synonymy].

—*fazi* (Navás), 1932a:84 [Type locality: Chile, Bío-Bío; DEI; ♀; in *Triplectides*]. —Flint 1974e:90 [checklist]. —Holzenthal, 1986d:269 [to synonymy].

—*pirioni* (Navás) 1935:374 [Type locality: Chile, Aysén; type missing from MNHNS, Camousseight, pers. com.; ♀; in *Triplectides*]. —Flint et al., 1999a:78 [to synonymy].

**Distribution.** Argentina, Chile.

#### Genus *Mystacides* Berthold [1]

*Mystacides* Berthold, 1827:437 [Type species: *Phryganea nigra* Linnaeus 1758, by monotypy].

*Mystacides* occurs in the Holarctic and Oriental regions. The three Nearctic species are well known in both their adult and immature stages (Yamamoto and Wiggins 1964, Wiggins 1996) and one of these species, *Mystacides alafimbriata*, occurs in Baja California.

Larvae are found in shallow marginal areas of lakes and ponds or in the slowly moving parts of streams. They are omnivorous, feeding on plant detritus as well as other arthropods (Wiggins 1996). Their cases are made of mineral fragments with long pieces of plant material incorporated. Adults engage in crepuscular swarming activity, especially at higher latitudes.

***alafimbriata*** Hill-Griffin, 1912:19 [Type locality: United States, Oregon, Mt. Jefferson, Permelia Lake; CU; ♂]. —Denning, 1964:134 [distribution]. —Yamamoto and Wiggins, 1964:1117 [♂; ♀; larva; pupa; redescription; distribution]. —Bueno-Soria and Flint, 1978:212 [distribution].

**Distribution.** Mexico, U.S.A.

#### Genus *Nectopsyche* Müller [49]

*Nectopsyche* Müller, 1879a:38 [Type species: *Setodes gemma* Müller, 1880a, first included species].

*Leptocella* Banks, 1899:214 [Type species: *Mystacides uwarowii* Kolenati, 1859, original designation]. —Flint, 1974c:127 [to synonymy].

*Brethesella* Navás, 1920b:71 [Type species: *Brethesella decorata* Navás, 1920b, by monotypy]. —Flint, 1982c:57 [to synonymy].

This genus of some 56 described species is restricted to the New World. Species occur from Canada south through the United States, Mexico, and Central and South America, to Argentina and Chile. Many new species await description and many more certainly occur in nature. A revision of the entire genus is sorely needed, the only one being that of Haddock (1977) for the Nearctic species. Flint, in a number of papers, clarified the identity of many of the older names, especially those described by Navás, and placed many in synonymy, and Holzenthal (1995) reviewed the *Gemma* group as it occurs in Costa Rica. Identity of species is based largely on color pattern of the body and forewings, male genitalia being rather uniform throughout the genus. It is therefore essential to have pinned, unrubbed material for identification.

Larvae are well known for both the Nearctic (Ross 1944, Haddock 1977, Wiggins 1996) and Neotropical faunas (Marlier 1964b, Roback 1966, Flint 1968a, Botosaneanu and Flint 1982, Roldán Pérez 1988, Glover and Floyd 2004). They generally build long, slender, tubular cases of sand grains or plant material or both. They inhabit small and large lentic and lotic habitats and are classified trophically as shredders-herbivores and collectors-gatherers (Morse and Holzenthal 2008). Larvae of some species have rows of long setae on the hind legs, enabling them to swim.

***acutiloba*** Flint, 1974c:133 [Type locality: Suriname, Kaboeri Creek, first camp; RNH; ♂]. —Blahnik et al., 2004:5 [distribution]. —Paprocki et al., 2004:12 [checklist]. —Paprocki and França, 2014:57 [checklist].

**Distribution.** Brazil, Guyana, Suriname.

***adusta*** Flint, 1983a:74 [Type locality: Argentina, Pcia. Entre Ríos, Salto Grande, Río Uruguay; NMNH; ♂]. —Angrisano and Sganga, 2007:46 [distribution]. —Calor, 2011:322 [checklist]. —Paprocki and França, 2014:57 [checklist].

**Distribution.** Argentina, Brazil.

***argentata*** Flint, 1991:94 [Type locality: Colombia, Dpto. Antioquia, Quebrada Honda, Marsella [12 km SW Fredonia]; NMNH; ♂]. —Holzenthal 1995:66 [♂; redescription; distribution; corrections]. —Muñoz-Quesada, 2000:279 [checklist]. —Bueno-Soria and Barba-Álvarez, 2011:358 [checklist].

**Distribution.** Colombia, Costa Rica, Mexico, Peru, Venezuela.

***aureofasciata*** Flint, 1981a:34 [Type locality: Venezuela, Aragua, Maracay, El Limón; NMNH; ♂].

**Distribution.** Venezuela.

***aureovittata*** Flint, 1983a:74 [Type locality: Argentina, Pcia. Misiones, Río Iguazú, Camp Nañdu; NMNH; ♂]. —Almeida and Marinoni, 2000:349 [distribution; biology]. —Blahnik et al., 2004:5 [distribution]. —Paprocki et al., 2004:12 [checklist]. —Dumas et al., 2009:368 [distribution]. —Calor, 2011:322 [checklist]. —Dumas and Nessimian, 2012:16 [distribution]. —Paprocki and França, 2014:57 [checklist]. —Moretto and Bispo, 2015:126 [distrtibution].

**Distribution.** Argentina, Brazil, Paraguay.

***bella*** (Müller), 1921:538 [Locality: Brazil, Santa Catarina; no type nor type depository designated, topotypic material collected by Müller in MNRJ (A.P.M. Santos, personal communication); pupa; in *Setodes*]. —Paprocki et al., 2004:12 [checklist]. —Paprocki and França, 2014:57 [checklist].

**Distribution.** Brazil.

***brethesi*** (Navás), 1920b:40, 66 [Type locality: Uruguay, Banda Oriental; MZBS; ♂; ♀; distribution; in *Leptocella*]. —Schmid 1949a:386 [no ♂ in Navás collection].

**Distribution.** Argentina, Uruguay.

***bruchi*** (Navás), 1920b:66 [Type locality: Argentina, Monte Veloz, estancia Barreto; MACN; ♂; in *Leptocella*]. —Flint 1972b:243 [diagnosis; distribution]. —Flint, 1982c:55 [redescription; distribution]. —Cohen, 2004:77 [distribution]. —Blahnik et al., 2004:5 [distribution]. —Paprocki et al., 2004:12 [checklist]. —Angrisano and Sganga, 2007:46 [distribution]. —Dumas et al., 2009:368 [distribution]. —Dumas and Nessimian, 2012:16 [distribution]. —Paprocki and França, 2014:57 [checklist].

**Distribution.** Argentina, Brazil, Paraguay.

***brunneofascia*** Flint, 1983a:71 [Type locality: Argentina, Pcia. Misiones, Puerto Libertad; NMNH; ♂]. —Blahnik et al., 2004:5 [distribution]. —Paprocki et al., 2004:12 [checklist]. —Calor, 2011:322 [checklist]. —Paprocki and França, 2014:58 [checklist].

**Distribution.** Argentina, Brazil.

***cana*** (Navás), 1924b:254 [Type locality: Venezuela, Arismondi; MNHNP; ♀; in *Leptocella*].

**Distribution.** Venezuela.

***candida*** (Hagan), 1861:280 [Type locality: U.S.A., Florida; MCZ; ♂; ♀; in *Setodes*]. —Ross, 1938a:22 [lectotype ♂]. —Haddock, 1977:396 [♂; larva; distribution]. —Glover and Floyd, 2004:538 [larva; distribution]. —Bueno-Soria et al., 2007:33 [distribution].

**Distribution.** Canada, Mexico, U.S.A.

***cubana*** (Banks), 1938:299 [Type locality: Cuba, Guines (sic); MCZ; ♂; in *Leptocella*]. —Flint, 1967c:21 [♂ lectotype]. —Flint, 1968a:54 [♂; ♀; distribution]. —Flint, 1968b:82 [checklist]. —Botosaneanu, 1979:52 [distribution]. —Kumanski, 1987:33 [distribution]. —Flint, 1992c:387 [♀; possibly *cubana* on Puerto Rico]. —Botosaneanu, 1994b:468 [larva]. —Botosaneanu, 1996:19 [distribution]. —Flint, 1996c:16 [checklist]. —Botosaneanu and Hyslop, 1998:21 [probable distribution]. —Flint and Pérez-Gelabert, 1999:41 [checklist]. —López del Castillo et al., 2004:229 [distribution]. —Flint and Sykora, 2004:45 [distribution]. —Naranjo López and González Lazo, 2005:150 [checklist]. —López del Castillo et al., 2007:171 [distribution; seasonal abundance]. —Pérez-Gelabert, 2008:301 [checklist].

**Distribution.** Cuba, Dominican Republic, Jamaica, Puerto Rico [?].

***diminuta*** (Banks), 1920:353 [Type locality: British Guiana, Bartica; MCZ; ♂; in *Leptocella*]. —Flint, 1967c:21 [♂ lectotype]. —Flint, 1974c:133 [♂ redescription; distribution]. —Flint, 1992d:71 [distribution]. —Paprocki et al., 2004:12 [checklist]. —Paprocki and França, 2014:58 [checklist].

**Distribution.** Brazil, Guyana, Suriname.

***dorsalis*** (Banks), 1901:368 [Type locality: Mexico, Veracruz, Jalapa; MCZ; sex not stated; in *Leptocella*]. —Flint, 1967c:21 [type is ♀; topotypic ♂ illustrated]. —Flint, 1967d:174 [distribution]. —Haddock, 1977:408 [♂; larva; distribution]. —Bueno-Soria and Flint, 1978:212 [distribution]. —Flint, 1981a:34 [♂; distribution]. —Maes and Flint, 1988:6 [distribution]. —Holzenthal, 1988c:73 [distribution]. —Flint, 1991:93 [♂; distribution]. —Aguila, 1992:543 [distribution]. —Maes, 1999:1197 [checklist]. —Muñoz-Quesada, 2000:279 [checklist]. —Blinn and Ruiter, 2006:333 [biology; distribution]. —Chamorro-Lacayo et al., 2007:44 [checklist]. —Blinn and Ruiter, 2009a:305 [biology]. —Blinn and Ruiter, 2009b:186 [phenology, distribution]. —Bueno-Soria and Barba-Álvarez, 2011:358 [checklist]. —Armitage et al., 2015b:8 [checklist]. —Armitage and Cornejo, 2015:197 [checklist].

—*serrei* (Navás), 1924c:83 [Type locality: Costa Rica, Sarapiquí, La Virgen; MNHNP; ♂; in *Leptocella*]. —Flint, 1981a:34 [to synonymy].

**Distribution.** Colombia, Costa Rica, El Salvador, Guatemala, Honduras, Mexico, Nicaragua, Panama, U.S.A, Venezuela.

***exophthalma*** Holzenthal, 1995:68 [Type locality: Costa Rica, Alajuela, Río Agrio, ca. 3.5 km NE Bajo del Toro, 10.243°N, 84.279°W; NMNH; ♂].

**Distribution.** Costa Rica.

***flavofasciata*** (Ulmer), 1907a:18 [Type locality: Brazil, Santa Catarina; PAN; ♂; in *Leptocella*]. —Flint, 1966a:9 [♂; redescription]. —Flint, 1972b:242 [distribution]. —Cohen, 2004:77 [distribution]. —Paprocki et al., 2004:12 [checklist]. —Calor, 2011:322 [checklist]. —Paprocki and França, 2014:58 [checklist]. —Isa Miranda and Rueda Martín, 2014:200 [distribution].

—*decorata* (Navás), 1920b:71 [Type locality: Argentina, Entre Ríos; MZBS; ♀; in *Brethesella*]. —Flint, 1982c:56 [to synonymy]. —Blahnik et al., 2004:5 [distribution].

—*sparsa* (Banks) 1920:353 [Type locality: Argentina, Misiones; MCZ; ♂; in *Leptocella*]. —Flint, 1966a:9 [to synonymy]. —Flint, 1967c:21 [♂ lectotype].

—*ditata* (Navás), 1933a:118 [Type locality: Argentina, Santa Fe, Piquete; ISMA; ♀; in *Leptocella*]. —Flint, 1982c:56 [to synonymy].

**Distribution.** Argentina, Bolivia, Brazil, Peru.

***fulva*** (Navás), 1930b:363 [Type locality: Chile, Perales; type depository not designated; ♀; in *Leptocella*]. —Flint 1974e:90 [checklist]. —Schmid, 1949a:386 [no ♂ in Navás collection].

**Distribution.** Chile.

***fuscomaculata*** Flint, 1983a:73 [Type locality: Argentina, Pcia. Misiones, Arroyo Liso, 8 km W General Güemes; NMNH; ♂]. —Flint, 1992d:72 [possibly *fuscomaculata*]. —Almeida and Marinoni, 2000:349 [distribution; biology]. —Blahnik et al., 2004:5 [distribution]. —Paprocki et al., 2004:12 [checklist]. —Dumas et al., 2009:368 [distribution]. —Calor, 2011:322 [checklist]. —Dumas and Nessimian, 2012:16 [distribution]. —Souza et al, 2013a:6 [distribution]. —Paprocki and França, 2014:58 [checklist]. —Dias et al., 2015:376 [distribution].

**Distribution.** Argentina, Brazil, Paraguay.

***gemma*** (Müller), 1880a:110, 130 [Type locality: Brazil, Santa Catarina; MNRJ; cases; in *Setodes*]. —Müller, 1880b:59, 80 [case]. —Ulmer, 1905b:74 [♂; to *Leptocella*]. —Müller, 1921:539 [pupa; in *Nectopsyche*; figs. 186e, 189d in *Setodes*]. —Flint, 1974c:129 [♂; distribution]. —Flint, 1991:95 [Neotype: Brazil, Sta. Catarina, Itajahy; MCZ; ♂; species figured in Flint 1974c as *gemma*, is *ortizi* Holzenthal 1995]. —Muñoz-Quesada, 2000:279 [checklist]. —Paprocki et al., 2004:12 [checklist]. —Paprocki and França, 2014:58 [checklist]. —Armitage et al., 2015b:8 [checklist]. —Armitage and Cornejo, 2015:197 [checklist].

—*festiva* (Navás), 1913:76 [Type locality: Ecuador, Loja; MNHNP; sex not stated; in *Leptocella* ?]. —Flint, 1991:95 [to synonymy].

—*genuosa* (Navás), 1920b:39. [Bolivia]. —Fischer, 1966:56 [probably misprint for *gemma*].

**Distribution.** Argentina, Bolivia, Brazil, Colombia, Ecuador, Guatemala, Mexico, Panama, Paraguay, Peru, Suriname, Venezuela [records from Mexico, Central America, and northern South America likely *Nectopsyche gemmoides* or *Nectopsyche ortizi*].

***gemmoides*** Flint, 1981a:35 [Type locality: Venezuela, Aragua, Maracay, Río Limón, Estación Pisicultura; NMNH; ♂]. —Botosaneanu and Flint, 1982:19 [larva; pupa]. —Maes and Flint, 1988:6 [distribution]. —Holzenthal, 1988c:73 [distribution]. —Flint, 1992d:73 [possibly *gemmoides*]. —Aguila, 1992:543 [distribution]. —Botosaneanu and Alkins-Koo, 1993:35 [distribution]. —Holzenthal 1995:69 [♂; ♀; larva; redescription; distribution]. —Flint, 1996a:104 [distribution]. —Maes, 1999:1197 [checklist]. —Muñoz-Quesada, 2000:279 [checklist]. —Rojas-Ascencio, et al., 2002:377 [distribution]. —Chamorro-Lacayo et al., 2007:44 [checklist]. —Bueno-Soria and Barba-Álvarez, 2011:358 [checklist]. —Calor, 2011:322 [checklist]. —Paprocki and França, 2014:58 [checklist]. —Armitage et al., 2015b:8 [checklist]. —Armitage and Cornejo, 2015:197 [checklist].

—*cupreosquamosa* Botosaneanu, in Botosaneanu and Alkins-Koo, 1993:35 [Type locality: Trinidad, lower course of River Guanapo near WASA pump station; ZMUA; ♂]. —Botosaneanu and Sakal, 1992:204 [distribution; ecology]. —Flint 1996a: 104 [to synonymy].

**Distribution.** Brazil, Colombia, Costa Rica, Ecuador, Guatemala, Guyana, Mexico, Nicaragua, Panama, Paraguay, Peru, Trinidad, Venezuela.

***globigona*** Botosaneanu, in Botosaneanu and Hyslop, 1998:21 [Type locality: Jamaica, Rio Minho in its upper reach at Grantham, a few km. W of Frankfield, Clarendon; ZMUA; ♂; larva]. —Botosaneanu and Hyslop, 1999:329 [female].

**Distribution.** Jamaica.

***gracilis*** (Banks), 1901:369 [Type locality: Mexico, Morelos, Cuernavaca; MCZ; ♂; in *Leptocella*]. —Fischer, 1966:51 [as synonym of *albida*]. —Flint, 1967c:21 [♂ holotype]. —Flint, 1967d:175 [distribution]. —Haddock, 1977:403 [♂; ♀; larva; distribution]. —Bueno-Soria and Flint, 1978:212 [distribution]. —Wiggins, 1996:170-171 [larva; case; as *intervena*]. —Holzenthal, 1988c:73 [distribution]. —Baumgardner and Bowles, 2005:11 [distribution]. —Blinn and Ruiter, 2006:333 [biology; distribution]. —Bowles et al., 2007:24 [distribution; biology]. —Bueno-Soria et al., 2007:33 [distribution].

—*exilis* (Banks), 1905:19 [replacement name for *Leptocella gracilis* Banks 1904a:110, preoccupied by *Leptocella gracilis* Banks, 1901:369]. [Type locality: United States, New Mexico, Gallinas Canyon; MCZ; ♂; in *Leptocella*]. —Fischer, 1966:54 [*gracilis* Banks 1904 as synonym of *exilis*]. —Haddock 1977:403 [to synonymy].

—*intervena* (Banks) 1914a:262 [Type locality: United States, Texas, Zavalla Co., Nueces River; NMNH; ♂; in *Leptocella*]. —Haddock, 1977:403 [to synonymy]. —Blinn and Ruiter, 2009a:304 [biology].

**Distribution.** Canada, Costa Rica, El Salvador, Guatemala, Mexico, U.S.A.

***jenseni*** (Ulmer), 1905b:75 [Type locality: Argentina, Mendoza, Santa Rosa; ZSZMH; ♂; ♀; in *Leptocella*]. —Banks, 1913b:87 [distribution]. —Mangeaud, 1996:154 [distribution]. —Cohen, 2004:78 [distribution]. —Paprocki et al., 2004:13 [checklist]. —Paprocki and França, 2014:58 [checklist].

—*mixta* (Navás), 1920b:67 [Type locality: Argentina, Córdoba, Alta Gracia; MZBS; ♂; in *Leptocella*]. —Schmid, 1949a:386 [♂; redescription]. —Flint, 1966a:9 [to synonymy with *Nectopsyche punctata*]. —Flint et al., 1999a:77 [to synonymy with *Nectopsyche jenseni*].

—*lucipeta* (Navás), 1923a:200 [Type locality: Argentina, Alta Gracia; MACN & collection Navás, now lost?; ♀; in *Leptocella*]. —Schmid, 1949a:386 [no ♂ in Navás collection]. —Flint et al., 1999a:77 [to synonymy].

**Distribution.** Argentina, Brazil.

***lahontanensis*** Haddock, 1977:412 [Type locality: United States, Utah, Jaub Co., .5 mile W Mills, on Sevier River; MCZ; ♂; larva].

**Distribution.** Canada, Mexico, U.S.A.

***lewisi*** (Flint), 1968a:55 [Type locality: Jamaica, St. Andrew, Hardwar Gap, Dicks Pond Trail; NMNH; ♂; ♀; larva; pupa; in *Leptocella*]. —Flint, 1968b:82 [checklist].

**Distribution.** Jamaica.

***maculipennis*** Flint, 1983a:75 [Type locality: Paraguay, Dpto. Amambay, 2 km S Cerro Corá; NMNH; ♂]. —Flint, 1996b:419 [distribution].

**Distribution.** Paraguay, Peru.

***modesta*** (Müller), 1921:535, fig. 186 f [Type locality: Brazil; type depository unknown; pupal abdominal segment 5; in *Setodes*]. —Ulmer, 1955:497 [bibliography]. —Flint et al., 1999a:78 [identity, to *Nectopsyche*, *nomen dubium*]. —Paprocki et al., 2004:13 [checklist]. —Paprocki and França, 2014:59 [checklist].

**Distribution.** Brazil.

***monticola*** Holzenthal, 1995:71 [Type locality: Costa Rica, San José, Quebrada Muerte, Rt. 2, 3.5 km (air) W Villa Mills, 9.562°N, 83.743°W; NMNH; ♂].

**Distribution.** Costa Rica.

***muelleri*** (Ulmer), 1905a:29 [Type locality: Brazil, Santa Catarina; PAN; ♂; in *Leptocella*]. —Flint 1966a:9 [♂]. —Paprocki et al., 2004:13 [checklist]. —Angrisano and Sganga, 2007:46 [distribution]. —Paprocki and França, 2014:59 [checklist].

**Distribution.** Argentina, Brazil.

***muhni*** (Navás), 1916b:68 [Type locality: Argentina, Santa Fe; MZBS; ♀; in *Leptocella*]. —Schmid, 1949a:388 [♂; type is ♀]. —Flint, 1974c:132 [♂; distribution]. —Flint, 1982c:58 [distribution]. —Flint, 1992d:71 [distribution]. —Almeida and Marinoni, 2000:349 [distribution; biology]. —Blahnik et al., 2004:5 [distribution]. —Cohen, 2004:78 [distribution]. —Paprocki et al., 2004:13 [checklist]. —Dumas et al., 2009:368 [distribution]. —Calor, 2011:322 [checklist]. —Dumas and Nessimian, 2012:17 [distribution]. —Paprocki and França, 2014:59 [checklist].

—*fulvocapilla* (Navás), 1922a:399 [Type locality: Argentina, LaPlata, Palo Blanco; MACN; ♂; in *Leptocella*]. —Flint, 1972b:243 [to synonymy].

—*pretiosella* (Banks), 1924:447 [Type locality: Peru, Yurimaguas; MCZ; ♀; in *Setodes*]. —Flint, 1982c:58 [to synonymy].

—*bridarollia* (Navás), 1930a:75 [Type locality: Argentina, Santa Fe, Piquete; ISMA; ♀; in *Leptocella*]. —Flint 1982c:58 [to synonymy].

**Distribution.** Argentina, Bolivia, Brazil, Ecuador, Guyana, Paraguay, Peru, Suriname, Venezuela.

***multilineata*** Flint, 1983a:73 [Type locality: Argentina, Pcia. Entre Ríos, Río Uruguay, Salto Grande; NMNH; ♂]. —Flint, 1992d:72 [distribution]. —Paprocki and França, 2014:59 [checklist].

**Distribution.** Argentina, Brazil, Venezuela.

***navasi*** Holzenthal, in Flint et al., 1999a:77 [replacement name for *Leptocella candida* Navás, 1923b:23, preoccupied by *Setodes candida* Hagen, 1861:280; lectotype]. [Type locality: Chile, Marga-Marga; MNHNP; ♀]. —Flint 1974e:90 [checklist]. —Blahnik et al., 2004:5 [distribution]. —Paprocki et al., 2004:13 [checklist]. —Paprocki and França, 2014:59 [checklist].

**Distribution.** Brazil, Chile.

***nigricapilla*** (Navás), 1920b:68 [Type locality: Paraguay, Río Paraguay; MZBS; ♂; in *Leptocella*]. —Schmid, 1949a:389 [♂]. —Flint, 1972b:243 [distribution; synonymy]. —Muñoz-Quesada, 2000:279 [checklist]. —Nogueira and Cabette, 2011:352 [distribution]. —Paprocki and França, 2014:59 [checklist].

—*ornata* (Navás), 1933a:119 [Type locality: Argentina, Santa Fe; type depository not given; ♂; in *Leptocella*]. —Flint, 1972b:243 [to synonymy].

**Distribution.** Argentina, Brazil, Colombia, Paraguay.

***onyx*** Holzenthal, 1995:71 [Type locality: Costa Rica, Puntarenas, Parque Nacional Corcovado, Piedra el Arco, 8.582°N, 83.709°W; NMNH; ♂].

**Distribution.** Costa Rica.

***ortizi*** Holzenthal, 1995:73 [Type locality: Costa Rica, Limón, Parque Nacional Tortuguero, Río Tortuguero, 3.5 km S Tortuguero, 10.509°N, 83.504°W; NMNH; ♂]. —Flint 1974c:129 [♂; as *Nectopsyche gemma*, nec Müller 1880a]. —Almeida and Marinoni, 2000:349 [distribution; biology]. —Blahnik et al., 2004:5 [distribution]. —Paprocki et al., 2004:13 [checklist]. —Dumas et al., 2009:369 [distribution]. —Bueno-Soria and Barba-Álvarez, 2011:358 [checklist]. —Calor, 2011:322 [checklist]. —Dumas and Nessimian, 2012:17 [distribution]. —Paprocki and França, 2014:59 [checklist]. —Armitage et al., 2015b:8 [checklist]. —Moretto and Bispo, 2015:126 [distrtibution]. —Armitage and Cornejo, 2015:197 [checklist].

**Distribution.** Argentina, Brazil, Costa Rica, Guyana, Mexico, Panama, Paraguay, Peru, Suriname, Venezuela.

***padrenavasi*** Holzenthal, in Flint et al., 1999a:78 [replacement name for *Leptocella nivea* Navás, 1920b:39, preoccupied by *Setodes nivea* Hagen, 1861:281; lectotype ♂]. [Type locality: Bolivia; MACN; sex not stated, but fig. is ♀].

**Distribution.** Bolivia.

***pantosticta*** Flint, 1983a:71 [Type locality: Argentina, Pcia. Misiones, Arroyo Coatí, 15 E San José; NMNH; ♂]. —Blahnik et al., 2004:5 [distribution]. —Paprocki et al., 2004:13 [checklist]. —Dumas et al., 2009:369 [distribution]. —Calor, 2011:322 [checklist]. —Dumas and Nessimian, 2012:25 [distribution]. —Paprocki and França, 2014:59 [checklist].

**Distribution.** Argentina, Brazil.

***pavida*** (Hagen), 1861:282 [Type locality: United States, Washington, D.C.; MCZ; sex not stated; in *Setodes*]. —Haddock, 1977:401 [♀ lectotype; ♂; larva; distribution]. —Bueno-Soria and Flint, 1978:212 [distribution]. —Maes and Flint, 1988:6 [distribution]. —Holzenthal, 1988c:73 [distribution]. —Aguila, 1992:543 [distribution]. —Moulton and Stewart, 1996:147 [distribution]. —Maes, 1999:1197 [checklist]. —Glover and Floyd, 2004:533 [larva; distribution]. —Bowles et al., 2007:24 [distribution; biology]. —Bueno-Soria et al., 2007:33 [distribution]. —Chamorro-Lacayo et al., 2007:44 [checklist]. —Bueno-Soria and Barba-Álvarez, 2011:359 [checklist]. —Armitage et al., 2015b:8 [checklist]. —Armitage and Cornejo, 2015:197 [checklist].

**Distribution.** Canada, Costa Rica, Guatemala, Mexico, Nicaragua, Panama, U.S.A.

***punctata*** (Ulmer), 1905b:75 [Type locality: Brazil, Santa Rita, Boquero, Rio Preto; NHMW; ♂; in *Leptocella*]. —Flint, 1966a:9 [♂ lectotype]. —Flint, 1974c:131 [♂; distribution]. —Bueno-Soria and Flint, 1978:212 [distribution]. —Flint, 1981a:34 [♂; distribution]. —Holzenthal, 1988c:74 [distribution]. —Flint, 1991:94 [distribution]. —Flint and Reyes, 1991:488 [distribution]. —Flint, 1992d:72 [distribution]. —Aguila, 1992:544 [distribution]. —Flint, 1996b:419 [distribution]. —Mangeaud, 1996:154 [distribution]. —Muñoz-Quesada, 2000:279 [checklist]. —Cohen, 2004:78 [distribution]. —Blahnik et al., 2004:5 [distribution]. —Paprocki et al., 2004:13 [checklist]. —Dumas et al., 2009:369 [distribution]. —Calor, 2011:322 [checklist]. —Dumas and Nessimian, 2012:17 [distribution]. —Paprocki and França, 2014:60 [checklist]. —Isa Miranda and Rueda Martín, 2014:200 [distribution]. —Armitage et al., 2015b:8 [checklist]. —Armitage and Cornejo, 2015:197 [checklist].

—*fenestrata* (Banks), 1913a:237 [Type locality: Panama, Lino; MCZ; ♂; in *Leptocella*]. —Flint, 1966a:9 [to synonymy].

—*spegazzinia* (Navás), 1920b:69 [Type locality: Paraguay, Río Paraguay; MZBS; ♂; in *Leptocella*]. —Flint, 1981a:34 [to synonymy].

—*ambitiosa* (Navás), 1933a:118 [Type locality: Argentina, Santa Fe; MZBS; ♂; in *Leptocella*]. —Schmid, 1949a:386 [to synonymy with *Nectopsyche mixta*]. —Flint, 1966a:9 [to synonymy].

**Distribution.** Argentina, Bolivia, Brazil, Colombia, Costa Rica, Ecuador, Guyana, Mexico, Panama, Paraguay, Peru, Suriname, Venezuela.

***quatuorguttata*** (Navás), 1922c:61 [Type locality: Bolivia; collection Navás, now lost?; ♀; in *Leptocella*]. —Schmid, 1949a:386 [no ♂ in Navás collection]. —Flint, 1974c:131 [♂; distribution]. —Flint, 1996b:419 [distribution]. —Cohen, 2004:78 [distribution]. —Nogueira and Cabette, 2011:352 [distribution]. —Paprocki and França, 2014:60 [checklist].

**Distribution.** Bolivia, Brazil, Guyana, Suriname, Paraguay, Peru.

***separata*** (Banks), 1920:353 [Type locality: Brazil, Santa Catarina; MCZ; ♂; in *Leptocella*]. —Flint, 1967c:22 [♂ lectotype]. —Flint, 1972b:242 [distribution]. —Almeida and Marinoni, 2000:349 [distribution; biology]. —Blahnik et al., 2004:5 [distribution]. —Paprocki et al., 2004:13 [checklist]. —Angrisano and Sganga, 2007:46 [distribution]. —Dumas et al., 2009:369 [distribution]. —Calor, 2011:322 [checklist]. —Dumas and Nessimian, 2012:18 [distribution]. —Paprocki and França, 2014:60 [checklist]. —Moretto and Bispo, 2015:126 [distrtibution].

—*graphica* (Navás), 1932b:65 [Type locality: Brazil, Rio de Janeiro, Barao Homen de Mello; DEI; ♂; in *Leptocella*]. —Flint, 1982c:59 [distribution; to synonymy].

**Distribution.** Argentina, Brazil, Paraguay.

***spiloma*** (Ross), 1944:219 [Type locality: United States, Kansas, Junction City; INHS; ♂; in *Leptocella*, ♀; larva]. —Haddock, 1977:392 [♂; larva; distribution]. —Flint and Reyes, 1991:488 [distribution]. —Aguila, 1992:544 [distribution]. —Moulton and Stewart, 1996:148 [distribution]. —Glover and Floyd, 2004:538 [larva; distribution]. —Chamorro-Lacayo et al., 2007:44 [checklist]. —Armitage et al., 2015b:8 [checklist]. —Armitage and Cornejo, 2015:197 [checklist].

**Distribution.** Costa Rica, Ecuador, Guatemala, Honduras, Mexico, Nicaragua, Panama, Peru, U.S.A.

***splendida*** (Navás), 1917a:403 [Type locality: Argentina, Santa Fe; collection Navás, now lost?; ♂; in *Leptocella*]. —Schmid, 1949a: 386 [no ♂ in Navás collection]. —Flint, 1982c:60 [redescription; distribution]. —Flint, 1992d:72 [distribution]. —Flint, 1996b:418 [distribution]. —Almeida and Marinoni, 2000:349 [distribution; biology]. —Muñoz-Quesada, 2000:279 [checklist]. —Paprocki et al., 2004:13 [checklist]. —Paprocki and França, 2014:60 [checklist]. —Quinteiro et al., 2014:233 [distribution].

**Distribution.** Argentina, Bolivia, Brazil, Colombia, Ecuador, Guyana, Paraguay, Peru, Venezuela.

***stigmatica*** (Banks), 1914a:262 [Type locality: United States, New Mexico, Jemez Mts.; MCZ; ♂; in *Leptocella*]. —Haddock, 1977:410 [♂; ♀; larva; distribution]. —Bueno-Soria and Flint, 1978:212 [distribution]. —Blinn and Ruiter, 2006:333 [biology; distribution]. —Bueno-Soria et al., 2007:33 [distribution]. —Blinn and Ruiter, 2009b:186 [phenology, distribution].

—*aeola* (Denning), 1948c:16 [Type locality: United States, Wyoming, Sybille River, near Wheatland; CAS; ♂; in *Leptocella*]. —Haddock, 1977:410 [to synonymy].

—*vakaca* (Schmid), 1949a:391 [replacement name for *Leptocerus stigmaticus* Navás 1917b:8, preoccupied by *Leptocella stigmatica* Banks, 1914a:262]. [Type locality: United States, New Mexico; MZBS; ♂]. —Haddock, 1977:410 [to synonymy].

**Distribution.** Mexico, U.S.A.

***taleola*** Flint, 1974c:134 [Type locality: Suriname, Litani River, Waremapan rapids; RNH; ♂]. —Flint, 1996b:419 [distribution].

**Distribution.** Peru, Suriname.

***tapanti*** Holzenthal, 1995:75 [Type locality: Costa Rica, Cartago, Reserva Tapantí, Quebrada Palmitos and falls, 9.72°N, 83.78°W; NMNH; ♂].

**Distribution.** Costa Rica.

***thallina*** (Navás), 1922b:264 [Type locality: Paraguay, Río Paraguay; collection Navás, now lost?; ♀; in *Leptocella*].

**Distribution.** Paraguay.

***tuanis*** Holzenthal, 1995:76 [Type locality: Costa Rica, Heredia, Parque nacional Braulio Carrillo, Est. El Ceibo, Río Peje, 10.327°N, 84.078°W; NMNH; ♂]. —Maes, 1999:1197 [checklist]. —Chamorro-Lacayo et al., 2007:44 [checklist].

**Distribution.** Costa Rica, Nicaragua.

***utleyorum*** Holzenthal, 1995:80 [Type locality: Costa Rica, Puntarenas, Reserva Bosque Nubosa Monteverde, Quebrada Cuecha, 10.31°N, 84.79°W; NMNH; ♂].

**Distribution.** Costa Rica.

#### Genus *Neoathripsodes* Holzenthal [2]

*Neoathripsodes* Holzenthal, 1989:31 [Type species: *Neoathripsodes anomalus* Holzenthal, 1989, original designation].

The genus now contains two species, both endemic to the Atlantic forest of southeastern Brazil. The immature stages are unknown and nothing is known of the biology of the species. Holzenthal (1989) suggested that the genus has African biogeographic affinities, but this has not been substantiated.

***anomalus*** Holzenthal, 1989:31 [Type locality: Brazil, Rio de Jaineiro, km 17, 18 km S Teresopolis; MZUSP; ♂]. —Paprocki et al., 2004:13 [checklist]. —Dumas et al., 2009:369 [distribution]. —Calor, 2011:322 [checklist]. —Dumas and Nessimian, 2012:18 [distribution]. —Paprocki and França, 2014:60 [checklist].

**Distribution.** Brazil.

***holzenthali*** Dias, Quinteiro, and Calor, 2015:372 [Type locality: Brazil, Bahia, Camacan, Reserva Particular do Patrimônio Natural Serra Bonita, stream after the dam supply, 15°23'26.6"S, 039°33'57.2"W, el. 828 m; MZUSP; ♂; female].

**Distribution.** Brazil.

#### Genus *Notalina* Mosely [11]

*Notalina* Mosely, 1936a:114 [Type species: *Notalina parkeri* Mosely, 1936a, original designation].

*Neonotalina* Holzenthal, 1986c:62 [Type species: *Notalina brasiliana* Holzenthal, 1986c, original designation, subgenus of *Notalina*]. —Calor et al., 2006:33 [phylogeny, biogeography]. —Calor and Froehlich, 2008:45 [larva; pupa; key to Neotropical Leptoceridae larvae]. —Henriques-Oliveira et al., 2012:132 [key to Neotropical species].

Holzenthal (1986c) placed the Neotropical species of this genus in a new subgenus, *Neonotalina*, establishing the presence of the genus in the Neotropics. It was previously known from Australia and Tasmania. The immature stages of the South American species were recenty described (Calor and Froehlich 2008) as were those of the Australian species (St. Clair 1991). The larvae of *Notalina morsei* were found in slowly flowing parts of streams and were associted with aquatic plants and plant detritus. Abandoned cases of some larvae were inhabited by *Triplectides gracilis* larvae. The phylogenetic analysis of Calor et al. (2006) confirmed Holzenthal’s (1986) placement of the Neotropical species into two species groups, one occuring in northern South America and the other in southeastern Brazil.

***brasiliana*** Holzenthal, 1986c:63 [Type locality: Brazil, Minas Gerais, Serra do Caraça; MZUSP; ♂; ♀]. —Paprocki et al., 2004:13 [checklist]. —Calor et al., 2006:41 [distribution]. —Paprocki and França, 2014:60 [checklist].

**Distribution.** Brazil.

***cipo*** Holzenthal, 1986c:67 [Type locality: Brazil, Minas Gerais, Serra do Cipó; MZUSP; ♂]. —Paprocki et al., 2004:13 [checklist]. —Calor et al., 2006:41 [distribution]. —Paprocki and França, 2014:61 [checklist]. —Dias et al., 2015:376 [distribution]. —Moretto and Bispo, 2015:126 [distrtibution].

**Distribution.** Brazil.

***froehlichi*** Calor and Holzenthal, in Calor et al., 2006:37 [Type locality: Brazil, Minas Gerais, Rio Caraça, near Santa Barbara, 20°01'22"S, 043°28'45"W, el. 728 m; MZUSP; ♂]. —Paprocki and França, 2014:61 [checklist].

**Distribution.** Brazil.

***goianensis*** Calor, 2008b:176 [Type locality: Brazil, Goiás, Chapada dos Veadeiros National Park, Alto Paraíso, Ribeirão Água Fria, 14°05'26.3"S, 47°29'38.5"W, el. 1225 m; MZUSP; ♂; ♀]. —Paprocki and França, 2014:61 [checklist].

**Distribution.** Brazil.

***hamiltoni*** Holzenthal, 1986c:67 [Type locality: Brazil, São Paulo, Estación Biol. Boraceia; MZUSP; ♂]. —Paprocki et al., 2004:13 [checklist]. —Calor et al., 2006:41 [distribution]. —Dumas et al., 2009:370 [distribution]. —Dumas et al., 2010:8 [distribution]. —Calor, 2011:322 [checklist]. —Dumas and Nessimian, 2012:18 [distribution]. —Paprocki and França, 2014:61 [checklist]. —Moretto and Bispo, 2015:126 [distrtibution].

**Distribution.** Brazil.

***jordanensis*** Henriques-Oliveira, Speis, and Dumas, 2012:131 [Type locality: Brazil, São Paulo, Campos do Jordão, Parque Estadual de Campos do Jordão, afluente do Córrego Galharada, 22°41'30"S, 45°27'36"W, el. 1600 m; MZUSP; ♂]. —Paprocki and França, 2014:61 [checklist].

**Distribution.** Brazil.

***matthiasi*** Holzenthal, 1986c:70 [Type locality: Colombia, Antioquia, Quebrada de Cebolla, W of La Fe, 25 E Medellín; NMNH; ♂; ♀]. —Flint, 1991:90 [♂; distribution]. —Muñoz-Quesada, 2000:279 [checklist].

**Distribution.** Colombia.

***morsei*** Holzenthal, 1986c:63 [Type locality: Brazil, Minas Gerais, Serra do Cipó; MZUSP; ♂; ♀]. —Paprocki et al., 2004:13 [checklist]. —Calor et al., 2006:41 [distribution]. —Calor and Froehlich, 2008:46 [larva; pupa; biology; distribution]. —Dumas et al., 2009:370 [distribution]. —Calor, 2011:322 [checklist]. —Dumas and Nessimian, 2012:18 [distribution]. —Paprocki and França, 2014:61 [checklist]. —Moretto and Bispo, 2015:126 [distrtibution].

**Distribution.** Brazil.

***nanay*** Holzenthal, 1986c:70 [Type locality: Peru, Loreto, Callicebus Res. Station Mishana, Río Nanay, 25 km SW Iquitos; NMNH; ♂].

**Distribution.** Peru.

***paulista*** Calor and Holzenthal, in Calor, Holzenthal, and Amorim, 2006:39 [Type locality: Brazil, São Paulo, Cachoeira do Paredão, Lajeado, Serra da Bocaina, 22°43'32"S, 044°37'16"W, el. 1550 m; MZUSP; ♂]. —Calor, 2011:322 [checklist]. —Paprocki and França, 2014:61 [checklist]. —Moretto and Bispo, 2015:126 [distrtibution].

**Distribution.** Brazil.

***roraima*** Holzenthal, 1986c:67 [Type locality: Venezuela, Bolivar, Cumbre Roraima, Cerra Hitogeographica; IZAM; ♂]. —Zamora-Muñoz et al., 2013:450 [♂; redescription; variation, larva; distribution].

**Distribution.** Venezuela.

#### Genus *Oecetis* McLachlan [55]

*Oecetis* McLachlan, 1877:294, 329 [Type species: *Leptocerus ochraceus* Curtis, 1825, subsequent selection of Ross 1944]. —Rueda Martín et al., 2011:19 [review of Neotropical species, key to species]. —Blahnik and Holzenthal, 2014:1 [review of *avara* Group, key to species]. —Quinteiro and Calor, 2015:1 [discussion of forewing venation, key to Brazilian species].

*Oecetina* Banks, 1899:213 [Type species: *Setodes incerta* Provancher, 1877, nec Walker, 1852 = *Oecetis inconspicua* (Walker) 1852, original designation]. —Ulmer, 1907d:142 [to synonymy].

*Pseudosetodes* Ulmer, 1905b:76 [Type species: *Pseudosetodes punctipennis* Ulmer, 1905b, by monotypy]. —Flint, 1966a:10 [to synonymy].

*Oecetinella* Ulmer, 1907d:133 [Type species: *Oecetis confluens* Ulmer, 1906, by monotypy]. —Schmid, 1958a:140 [to synonymy].

This is a large cosmopolitan genus of over 500 described species. The 55 described Neotropical species occur throughout the Brazilian Subregion, but the genus is absent from the Chilean Subregion. Species level taxonomy of the genus generally is well founded and based upon distinct differences in male genitalia. However, in certain species groups, for example the *avara* group, new, cryptic species have been discoverd and described (e.g., Blahnik and Holzenthal, 2014) and likely occur in other groups (e.g., *inconspicua* group, *punctata* group). In spite of the recent surge in species descriptions (Rueda-Martin et al. 2011, Blahnik and Holzenthal 2014, Quinteiro and Calor 2015) many undescribed species certainly occur in South America, where the Amazon basin is a major center of diversity.

Two species previously recorded from the area covered by this catalog no longer have confirmed records in the region. As discussed by Blahnik and Holzenthal (2014) *Oecetis avara* is restricted in distrubution to eastern North America, exclusive of Mexico. Previous records from Mexico, Central, and South America now pertain to a number of new species described and diagnosed by Blahnik and Holzenthal (2014). Likewise, *Oecetis disjuncta* is now restricted to the west coast of the United States (California, Oregon); literature records from Mexico pertain to *Oecetis sordida* according to Blahnik and Holzenthal (2014).

Larvae of the North American species are well known (Ross 1944, Floyd 1995, Wiggins 1996). Larvae of the Neotropical species have been described by Flint (1968a), Marlier (1964b), Roback (1966), and Roldán Pérez (1988). Larvae live on the bottoms of lakes and rivers where they prey on small arthropods.

***acanthostema*** Quinteiro and Calor, 2015:3 [Type locality: Brazil, Bahia, Senhor do Bonfim, Serra Santana; MZUSP; ♂; *Quaria* species group].

**Distribution.** Brazil.

***acciptrina*** Blahnik and Holzenthal, 2014:10 [Type locality: Ecuador, Pichincha, Santo Domingo (47 km S), Río Palenque Biol. Station, el. 750 m; NMNH; ♂]. —Armitage et al., 2015b:8 [checklist]. —Armitage and Cornejo, 2015:198 [checklist].

**Distribution.** Costa Rica, Ecuador, Panama.

***agosta*** Blahnik and Holzenthal, 2014:11 [Type locality: Mexico, San Luis Potosí, El Salto Falls; NMNH; ♂].

**Distribution.** Mexico.

***amazonica*** (Banks), 1924:447 [Type locality: Brazil, Manáos; MCZ; ♂; in *Oecetina*]. —Flint, 1967c:22 [♂ lectotype]. —Flint, 1982c:49 [♂; distribution]. —Paprocki et al., 2004:13 [checklist]. —Rueda Martín et al., 2011:21 [♂; redescription; distribution]. —Paprocki and França, 2014:62 [checklist]. —Henriques-Oliveira et al., 2014:279 [distribution]. —Quinteiro and Calor, 2015:15 [♂, redescription; distribution].

**Distribution.** Argentina, Bolivia, Brazil, Peru, Venezuela.

***angelae*** Henriques-Oliveira, Dumas, and Nessimian, 2014:274 [Type locality: Brazil, Mato Grosso do Sul, Ladário, Pantanal, Rio Paraguai floodplain, lake near Pousada Porto Vitória Régia, 19°01'10.00"S, 57°33'02.10"W, el. 91 m; DZRJ; ♂]. —Paprocki and França, 2014:62 [checklist].

**Distribution.** Brazil.

***angularis*** Blahnik and Holzenthal, 2014:13 [Type locality: Costa Rica, Puntarenas, Río Bellavista, ca. 1.5 km NW Las Alturas, 8°57.060'N, 82°50.760'W, el. 1400 m; UMSP; ♂].

**Distribution.** Costa Rica, Ecuador, Guatemala.

***arizonica*** Denning, 1951:159 [Type locality: United States, Arizona, Cochise Co., Huachuca, Gordon Canyon; CAS; ♂]. —Flint, 1967d:174 [distribution]. —Bueno-Soria and Flint, 1978:213 [distribution]. —Blinn and Ruiter, 2006:333 [biology; distribution]. —Blinn and Ruiter, 2009b:186 [phenology, distribution].

**Distribution.** Mexico, U.S.A.

***bilobosa*** Flint, 1974c:126 [Type locality: Suriname, Nassau Mountains, trail km 11; RNH; ♂].

**Distribution.** Suriname.

***campana*** Blahnik and Holzenthal, 2014:23 [Type locality: Ecuador, Zamora-Chinchipe, Río Chicaña, 9 km N Yanzatza, el. 880 m; NMNH; ♂; species illustrated as *avara* from Bolivia likely this species]. —Rueda Martín et al., 2011:21 [♂; distribution; nec *avara*].

**Distribution.** Bolivia, Ecuador.

***carlibanezae*** Rueda Martín, Gibon, and Molina, 2011:23 [Type locality: Bolivia, Béni, Lake Belen, near Trinidad, 14°27'29"S, 64°51'41"W; IML; ♂].

**Distribution.** Bolivia.

***chipiriri*** Rueda Martín, Gibon, and Molina, 2011:23 [Type locality: Bolivia: Cochabamba: Small tributary of the Río Espiritu Santo, Chipiriri, near Villa Tunari, 16°50'45"S, 65°25'33"W; IML; ♂].

**Distribution.** Bolivia.

***cinerascens*** (Hagen), 1861:282 [Type locality: United States, Washington, D.C.; MCZ; ♂; in *Setodes*]. —Ross, 1938a:24 [♂ lectotype]. —Ross, 1944:241 [♂; ♀; larva; distribution]. —Bueno-Soria and Flint, 1978:213 [distribution]. —Moulton and Stewart, 1996:150 [distribution]. —Bowles et al., 2007:24 [distribution; biology].

—*fumosa* (Banks), 1899:216 [Type locality: United States, Washington, D.C.; MCZ; ♀; in *Oecetina*]. —Ross, 1938a:25 [to synonymy].

—*floridana* (Banks), 1899:216 [Type locality: United States, Florida, Biscayne Bay; MCZ; ♂; in *Oecetina*]. —Milne, 1934:19; according to 1935 errata [to synonymy, as *florida*]. —Ross, 1938a:24 [as synonym of *inconspicua*]. —Fischer, 1966:155 [Ross’s referral to *inconspicua* must be a mistake].

**Distribution.** Canada, Mexico, U.S.A.

***clavicornia*** Quinteiro and Calor, 2015:8 [Type locality: Brazil, Bahia, Wenceslau Guimarães, Rio Patioba, 13°34'50.3"S, 39°42'17"W, el. 432 m; MZUSP; ♂].

**Distribution.** Brazil.

***connata*** Flint, 1974c:122 [Type locality: Suriname, Coeroeni River Expedition, Zuid River, in Lucie River; RNH; ♂]. —Dumas et al., 2010:8 [distribution]. —Quinteiro et al., 2014:233 [distribution]. —Paprocki and França, 2014:62 [checklist]. —Henriques-Oliveira et al., 2014:279 [distribution]. —Quinteiro and Calor, 2015:17 [♂, redescription; distribution].

**Distribution.** Brazil, Guyana, Suriname.

***constricta*** Blahnik and Holzenthal, 2014:25 [Type locality: Costa Rica, San José, Res. Biol. Carara, Río Carara in Carara, 9°46.680'N, 84°31.860'W, el. 200 m; UMSP; ♂]. —Armitage et al., 2015b:8 [checklist]. —Armitage and Cornejo, 2015:198 [checklist].

**Distribution.** Colombia, Costa Rica, Ecuador, Guatemala, Guyana, Honduras, Mexico, Nicaragua, Panama, Trinidad, Venezuela.

***danielae*** Henriques-Oliveira, Dumas, and Nessimian, 2014:276 [Type locality: Brazil, Amazonas, Barcelos, Rio Aracá, Comunidade Bacuquara, 00°00'55.11"N, 63°10'38.75"W, el. 52 m; DZRJ; ♂]. —Paprocki and França, 2014:62 [checklist].

**Distribution.** Brazil.

***doesburgi*** Flint, 1974c:126 [Type locality: Suriname, Boven Para, near Berlijn; RNH; ♂]. —Henriques-Oliveira et al., 2014:279 [distribution].

**Distribution.** Brazil, Suriname.

***dominguezi*** Rueda Martín, Gibon, and Molina, 2011:25 [Type locality: Bolivia, Béni, Lake Colorada, near Trinidad, 14°48'21"S, 64°58'41"W; IML; ♂]. —Henriques-Oliveira et al., 2014:280 [distribution].

**Distribution.** Bolivia, Brazil.

***elata*** Denning and Sykora, 1966:1225 [Type locality: Mexico, Veracruz, Almilinta; CAS; ♂; ♀; as *elatus*]. —Bueno-Soria and Flint, 1978:213 [distribution]. —Blahnik and Holzenthal, 2014:31 [♂; redescription].

**Distribution.** Mexico.

***excisa*** Ulmer, 1907a:15 [Type locality: Argentina, Chaco de Santa Fé, Las Garzas, Río Las Garzas, 25 km W Ocampo; MNHNP; ♂]. —Flint, 1982c:50 [♂; distribution]. —Cohen, 2004:78 [distribution]. —Paprocki et al., 2004:13 [checklist]. —Angrisano and Sganga, 2007:47 [♂; distribution]. —Rueda Martín et al., 2011:25 [♂; redescription; distribution]. —Calor, 2011:322 [checklist]. —Souza et al, 2013a:6 [distribution]. —Quinteiro et al., 2014:233 [distribution]. —Paprocki and França, 2014:62 [checklist]. —Henriques-Oliveira et al., 2014:280 [distribution]. —Quinteiro and Calor, 2015:19 [♂; redescription; distribution]. —Moretto and Bispo, 2015:126 [distrtibution].

—*mutila* Navás, 1918d:22 [Type locality: Argentina, Santa Fe; MZBS; ♂]. —Schmid, 1949a:382 [to synonymy].

—*castilleja* Navás, 1920a:134 [Type locality: Argentina, Santa Fe; collection Navás, now lost?; ♀]. —Schmid, 1949a:381 [possible synonym of *excisa*]. —Flint, 1972b:244 [to synonymy].

—*muhnia* Navás, 1920c:28 [Type locality: Argentina, Santa Fe; collection Navás, now lost?; ♂]. —Flint, 1972b:244 [to synonymy].

—*apicata* Navás, 1931a:323 [Type locality: Argentina, Chaco de Santiago del Estero, env. d’Icaño; MNHNP; ♀]. —Flint, 1982c:50 [to synonymy].

**Distribution.** Argentina, Bolivia, Brazil, Mexico, Paraguay, Venezuela.

***falicia*** Denning, in Denning and Sykora 1966:1225 [Type locality: Panama, Canal Zone, Barro Colorado Island; CAS; ♂; ♀]. —Aguila, 1992:544 [distribution]. —Armitage et al., 2015b:8 [checklist]. —Armitage and Cornejo, 2015:198 [checklist].

**Distribution.** Panama.

***fibra*** Chen and Morse, 2012, in Quinteiro and Calor, 2012:54 [Type locality: Brazil, São Paulo, Estação Biológica Boracéia, Córrego Venerando; MZUSP; ♂]. —Henriques-Oliveira et al., 2014:280 [distribution]. —Paprocki and França, 2014:62 [checklist].

**Distribution.** Brazil.

***froehlichi*** Quinteiro and Calor, 2015:12 [Type locality: Brazil, Bahia, Wenceslau Guimarães, Estação Ecológica Estadual Wenceslau Guimarães, Riacho Serra Grande; MZUSP; ♂].

**Distribution.** Brazil.

***furcata*** Quinteiro and Calor, 2015:10 [Type locality: Brazil, Bahia, Camacan, Reserva Particular do Patrimônio Natural Serra Bonita, stream 1; MZUSP; ♂]. —Dias et al., 2015:377 [distribution].

**Distribution.** Brazil.

***haitises*** Flint and Sykora, 2004:46 [Type locality: Dominican Republic, Hato Mayor Province, Parque Los Haitises, 3 km W Cueva de Arena, 19°04'N, 69°29'W, el. 20 m; CMNH; ♀; ♂ unknown]. —Pérez-Gelabert, 2008:301 [checklist].

**Distribution.** Dominican Republic.

***iara*** Henriques-Oliveira, Dumas, and Nessimian, 2014:277 [Type locality: Brazil, Paraná, Foz do Iguaçu, Parque Nacional do Iguaçu, Rio São João, 25°37'13.50"S, 54°28'35.90"W, el. 178 m; DZRJ; ♂]. —Paprocki and França, 2014:62 [checklist].

**Distribution.** Brazil.

***iguazu*** Flint, 1983a:70 [Type locality: Argentina, Pcia. Misiones, Río Iguazú, Camp Nañdu; NMNH; ♂]. —Blahnik et al., 2004:5 [distribution]. —Paprocki et al., 2004:13 [checklist]. —Dumas et al., 2009:370 [distribution]. —Calor, 2011:322 [checklist]. —Quinteiro et al., 2014:234 [distribution]. —Paprocki and França, 2014:63 [checklist]. —Henriques-Oliveira et al., 2014:280 [distribution]. —Quinteiro and Calor, 2015:22 [♂; redescription; distribution]. —Moretto and Bispo, 2015:126 [distrtibution].

**Distribution.** Argentina, Brazil, Paraguay.

***inconspicua*** (Walker), 1852:71 [Type locality: United States, Georgia; BMNH; ♂; in *Leptocerus*]. —Betten and Mosely, 1940:67 [♂; redescription]. —Ross, 1944:242 [♂; ♀; larva; pupa; synonymy; distribution]. —Ross, 1951a:73 [distribution]. —Denning, 1964:134 [checklist]. —Flint, 1964a:64 [♂; distribution]. —Flint, 1967d:174 [distribution]. —Flint, 1968a:54 [♂; ♀; distribution]. —Flint, 1968b:82 [checklist]. —Bueno-Soria and Flint, 1978:213 [distribution]. —Botosaneanu, 1979:52 [distribution]. —Flint, 1981a:32 [♂; distribution]. —Maes and Flint, 1988:6 [distribution]. —Holzenthal, 1988c:74 [distribution]. —Botosaneanu, 1991a:134 [distrubution]. —Flint, 1991:97 [♂; distribution]. —Flint, 1992d:74 [possibly *inconspicua*]. —Aguila, 1992:544 [distribution]. —Flint, 1996a:105 [distribution]. —Flint, 1996b: 421 [distribution]. —Flint, 1996c:16 [checklist]. —Moulton and Stewart, 1996:151 [distribution]. —Maes, 1999:1197 [checklist]. —Flint and Pérez-Gelabert, 1999:41 [checklist]. —Muñoz-Quesada, 2000:279 [checklist]. —Flint and Sykora, 2004:46 [distribution]. —Blahnik et al., 2004:5 [distribution]. —Paprocki et al., 2004:13 [checklist]. —Flint and Sykora, 2004:46 [distribution]. —Naranjo López and González Lazo, 2005:150 [checklist]. —Blinn and Ruiter, 2006:333 [biology; distribution]. —Bowles et al., 2007:24 [distribution; biology]. —Chamorro-Lacayo et al., 2007:44 [checklist]. —Pérez-Gelabert, 2008:301 [checklist]. —Blinn and Ruiter, 2009a:305 [biology]. —Bueno-Soria and Barba-Álvarez, 2011:359 [checklist]. —Calor, 2011:322 [checklist]. —Rueda Martín et al., 2011:27 [♂; redescription; distribution]. —Quinteiro et al., 2014:234 [distribution]. —Paprocki and França, 2014:62 [checklist]. —Henriques-Oliveira et al., 2014:281 [distribution]. —Quinteiro and Calor, 2015:24 [♂; redescription; distribution]. —Armitage et al., 2015b:8 [checklist]. —Armitage and Cornejo, 2015:198 [checklist].

—*flaveolata* (Hagen), 1861:282 [Type locality: United States, Louisiana, New Orleans; MCZ; ♂; ♀; in *Setodes*]. —Milne 1934:19 [to synonymy].

—*micans* (Hagen), 1861:283 [Type locality: United States, Washington, D.C. and Mexico; MCZ; ♂; ♀; in *Setodes*]. —Milne 1934:19 [to synonymy].

—*sagitta* (Hagen), 1861:284 [Type locality: United States, Florida; MCZ; ♀; in *Setodes*]. —Milne 1934:19 [to synonymy].

—*parvula* (Banks), 1899:215 [Type locality: United States, Washington, D.C.; MCZ; ♀; in *Oecetina*]. —Milne 1934:19 [to synonymy].

—*flavida* (Banks), 1899:216 [Type locality: United States, Florida, Kissimmee; MCZ; sex not stated; in *Oecetina*]. —Milne 1934:19 [to synonymy].

—*inornata* (Banks), 1907a:128 [Type locality: United States, Arizona, Douglas; MCZ; sex not stated; in *Oecetina*]. —Milne 1934:19 [to synonymy].

—*apicalis* (Banks), 1907a:129 [Type locality: United States, Texas, Brownsville; MCZ; ♂; in *Oecetina*]. —Milne 1934:19 [to synonymy].

—*antillana* (Banks), 1938:298 [Type locality: Cuba, Soledad, near Cienfuegos; MCZ; ♂; in *Oecetina*]. —Flint, 1967c:23 [to synonymy].

**Distribution.** Bahamas, Bolivia, Brazil, Canada, Colombia, Costa Rica, Cuba, Dominican Republic, El Salvador, Guatemala, Haiti, Honduras, Jamaica, Mexico, Nicaragua, Panama, Peru, Puerto Rico, U.S.A., Venezuela.

***inflata*** Flint, 1974c:125 [Type locality: Suriname, Wilhelmina Mountains, trail II km 5.7, creek; RNHL; ♂].

**Distribution.** Suriname.

***knutsoni*** Flint, 1981a:32 [Type locality: Venezuela, Aragua, Ocumare [de la Costa]]; NMNH; ♂]. —Malicky, 1983:264, 266 [distribution]. —Flint, 1991:97 [♂; distribution]. —Flint and Sykora, 1993:50 [checklist]. —Botosaneanu 1994a:51, [distribution]. —Flint, 1996b:422 [distribution]. —Muñoz-Quesada, 2000:279 [checklist]. —Medellín et al., 2004:201 [distribution; biology]. —Botosaneanu and Thomas, 2005:56 [checklist]. —Rueda Martín et al., 2011:27 [♂; redescription; distribution]. —Henriques-Oliveira et al., 2014:281 [distribution]. —Isa Miranda and Rueda Martín, 2014:200 [distribution]. —Armitage et al., 2015a:7 [distribution]. —Armitage et al., 2015b:8 [checklist]. —Armitage and Cornejo, 2015:198 [checklist].

**Distribution.** Bolivia, Brazil, Colombia, Guadeloupe, Panama, Peru, Venezuela.

***maritza*** Blahnik and Holzenthal, 2014:40 [Type locality: Costa Rica, Guanacaste, Parque Nacional Guanacaste, Estación Maritza, Río Tempisquito, 10°57.480'N, 85°29.820'W, el. 550 m; UMSP; ♂].

**Distribution.** Costa Rica.

***marquesi*** Bueno-Soria, 1981:113 [Type locality: Mexico, Nuevo León, Monterrey, Potrero Redondo; IBUNAM; ♂]. —Blahnik and Holzenthal, 2014:67 [♂; redescription].

**Distribution.** Mexico.

***martinae*** Quinteiro and Calor, 2015:6 [Type locality: Brazil, Bahia, Wenceslau Guimarães, Estação Ecológica Estadual Wenceslau Guimarães, Riacho Serra Grande, near Station headquarters, 13°35'43"S, 38°43'12"W; MZUSP; ♂]. —Dias et al., 2015:377 [distribution].

**Distribution.** Brazil.

***maspeluda*** Botosaneanu, 1977:280 [Type locality: Cuba, Isla de Pinos, Santa Fé, Arroyo La Talega; NMNH; ♂]. —Botosaneanu, 1979:52 [distribution]. —Flint, 1996c:16 [checklist]. —Naranjo López and González Lazo, 2005:150 [checklist].

**Distribution.** Cuba.

***metlacensis*** Bueno-Soria, 1981:109 [Type locality: Mexico, Veracruz, 5 km Puente Villa Unión; IBUNAM; ♂]. —Houghton 2001:91 [distrobution]. —Blinn and Ruiter, 2006:333 [likely *Oecetis apache*, biology; distribution]. —Blahnik and Holzenthal, 2014:42 [♂; redescription; distribution; record by Houghton, 2001, from Arizona is *Oecetis apache*].

**Distribution.** Costa Rica, Mexico.

***mexicana*** Blahnik and Holzenthal, 2014:45 [Type locality: Mexico, Chiapas, Río Tulija, 48 km S Palenque; NMNH; ♂]. —Armitage et al., 2015b:8 [checklist]. —Armitage and Cornejo, 2015:198 [checklist].

**Distribution.** Costa Rica, Ecuador, Honduras, Mexico, Panama, Venezuela.

***oberdorffi*** Rueda Martín, Gibon, and Molina, 2011:29 [Type locality: Bolivia, Bella Vista, Lake Granja, 13°15'50"S, 63°42'33"W; IML; ♂].

**Distribution.** Bolivia.

***paranensis*** Flint, 1982a:46 [Type locality: Argentina, Chaco, Riacho. Barranqueras, Puerto Vilelas; NMNH; ♂]. —Flint, 1982c:52 [distribution]. —Flint, 1996b:421 [distribution]. —Paprocki et al., 2004:13 [checklist]. —Rueda Martín et al., 2011:29 [♂; redescription; distribution]. —Souza et al, 2013a:6 [distribution]. —Quinteiro et al., 2014:235 [distribution]. —Paprocki and França, 2014:63 [checklist]. —Henriques-Oliveira et al., 2014:281 [distribution]. —Quinteiro and Calor, 2015:27 [♂; redescription; distribution].

**Distribution.** Argentina, Bolivia, Brazil, Paraguay, Peru.

***patula*** Blahnik and Holzenthal, 2014:49 [Type locality: Guatemala, Baja Verapaz, Rt 5, Km 156, Puente Las Burras; NMNH; ♂].

**Distribution.** Guatemala, Nicaragua.

***peruviana*** (Banks), 1924:446 [Type locality: Peru, Iquitos; MCZ; ♂; in *Oecetina*]. —Flint, 1967c:23 [♂ lectotype].

**Distribution.** Peru.

***pratti*** Denning, 1947a:656 [Type locality: Puerto Rico, El Yunque; CAS; ♂]. —Flint, 1964a:62 [♂; distribution]. —Flint, 1968b:67 [♂; ♀; larva]. —Flint and Sykora, 1993:59 [distribution].

**Distribution.** Grenada, Dominica, Puerto Rico.

***prolongata*** Flint, 1981a:33 [Type locality: Venezuela, Aragua, Maracay, Río Limón, Estación Piscicultura; NMNH; ♂].

**Distribution.** Venezuela.

***protrusa*** Blahnik and Holzenthal, 2014:51 [Type locality: Costa Rica, Heredia, Parque Nacional Braulio Carrillo, Est. Magsasay, Río Peje, 10°24.120'N, 84°3.000'W, el. 130 m; UMSP; ♂]. —Armitage et al., 2015b:8 [checklist]. —Armitage and Cornejo, 2015:198 [checklist].

**Distribution.** Colombia, Costa Rica, Ecuador, Guatemala, Mexico, Nicaragua, Panama.

***pseudoamazonica*** Rueda Martín, Gibon, and Molina, 2011:31 [Type locality: Bolivia, Lake Colorada, near Trinidad, 14°48'21"S, 64°58'41"W; IML; ♂].

**Distribution.** Bolivia, Venezuela.

***pseudoinconspicua*** Bueno-Soria, 1981:106 [Type locality: Mexico, Oaxaca, Cd. Alemán; IBUNAM; ♂]. —Holzenthal, 1988c:74 [distribution]. —Aguila, 1992:544 [distribution]. —Armitage et al., 2015b:8 [checklist]. —Armitage and Cornejo, 2015:198 [checklist].

**Distribution.** Costa Rica, Mexico, Panama.

***punctata*** (Navás), 1924c:85 [Type locality: Costa Rica; MNHNP; ♀; in *Oecetinella*]. —Holzenthal, 1988c:74 [distribution]. —Bueno-Soria et al., 2005:75 [distribution].

**Distribution.** Costa Rica, Mexico.

***punctipennis*** (Ulmer), 1905b:77 [Type locality: Brazil, Sta. Rita; NHMW; ♀; in *Pseudosetodes*]. —Flint, 1966a:10 [♀ lectotype; ♂]. —Flint, 1974c:123 [♂; distribution]. —Flint, 1982c:53 [distribution]. —Holzenthal, 1988c:74 [distribution]. —Maes and Flint, 1988:6 [distribution]. —Flint, 1992d:73 [distribution]. —Flint, 1996b:421 [distribution]. —Aguila, 1992:544 [distribution]. —Maes, 1999:1198 [checklist]. —Muñoz-Quesada, 2000:279 [checklist]. —Paprocki et al., 2004:13 [checklist]. —Angrisano and Sganga, 2007:47 [♂; distribution]. —Chamorro-Lacayo et al., 2007:44 [checklist]. —Rueda Martín et al., 2011:31 [♂; redescription; distribution]. —Calor, 2011:322 [checklist]. —Souza et al, 2013a:7 [distribution]. —Quinteiro et al., 2014:235 [distribution]. —Paprocki and França, 2014:63 [checklist]. —Quinteiro and Calor, 2015:29 [♂; redescription; distribution]. —Armitage et al., 2015b:8 [checklist]. —Armitage and Cornejo, 2015:198 [checklist].

—*parishi* (Banks), 1914b (1915):631 [Type locality: Guyana; MCZ; ♂; in *Oecetina*]. —Flint, 1966a:10 [to synonymy].

—*bridarollina* Navás, 1933a:116 [Type locality: Argentina, Santa Fe; ISMA; ♂]. —Flint, 1972b:245 [to synonymy].

**Distribution.** Argentina, Bolivia, Brazil, Costa Rica, Ecuador, Guyana, Nicaragua, Panama, Peru, Suriname, Venezuela.

***rafaeli*** Flint, 1992d:74 [Type locality: Brazil, Estado Roraima, Ilha Maraca, Rio Uraricoera; INPA; ♂]. —Paprocki et al., 2004:13 [checklist]. —Ribeiro et al., 2009:34 [list of types]. —Rueda Martín et al., 2011:21 [♂; redescription; distribution]. —Paprocki and França, 2014:63 [checklist].

**Distribution.** Bolivia, Brazil.

***scoparia*** Flint, 1974c:123 [Type locality: Suriname, Nassau Mountains, km 11.3, creek; RNH; ♂].

**Distribution.** Suriname.

***silviae*** Bueno-Soria, 1981:115 [Type locality: Mexico, Veracruz, Las Minas; IBUNAM; ♂].

**Distribution.** Mexico.

***sordida*** Blahnik and Holzenthal, 2014:56 [Type locality: USA, South Dakota, Lawrence Co., Black Hills National Forest, Boxelder Creek, 1.8 mi W Nemo, 44°11.846'N, 103°32.024'W, el. 1470 m; UMSP; ♂].

—*disjuncta* Smith and Lehmkuhl 1980: 638 *nec* Banks [larval description and distribution, at least in part]. —Floyd 1995:29 [ditto]. —Blahnik and Holzenthal, 2014:56 [some material of authors likely *sordida*].

**Distribution.** Mexico, U.S.A.

***traini*** Rueda Martín, Gibon, and Molina, 2011:33 [Type locality: Bolivia, Lake Bay, Río Manuripi basin; IML; ♂].

**Distribution.** Bolivia.

***tumida*** Blahnik and Holzenthal, 2014:59 [Type locality: Costa Rica, Limón, Res. Biol. Barbilla, Río Dantas, 15 km (road) S Pacuarito, 9°59.640'N, 83°26.580'W, el. 300 m; UMSP; ♂].

**Distribution.** Costa Rica.

***uncata*** Blahnik and Holzenthal, 2014:61 [Type locality: Costa Rica, Cartago, Reserva Tapantí, Río Dos Amigos & falls, ca. 6 km (rd) NW tunnel, 9°42.240'N, 83°46.980'W, el. 1500 m; UMSP; ♂].

**Distribution.** Costa Rica.

***verrucula*** Blahnik and Holzenthal, 2014:63 [Type locality: Costa Rica, Guanacaste, Hda. Tempisquito (Pelón de la Altura), 1 km NE km 265, rt. 1, 10°50.400'N, 85°33.600'W, el. 100 m; UMSP; ♂].

**Distribution.** Costa Rica, El Salvador, Guatemala, Honduras, Mexico, Nicaragua.

#### Genus *Osflintia* Calor and Holzenthal [1]

*Osflintia* Calor and Holzenthal, 2008:249 [Type species: *Osflintia manu*, Calor and Holzenthal, 2008, original designation].

The genus is known only from the male holotype from Peru, but a second male specimen was collected recently in the country (L.E. Rázuri Gonzales, personal communication). While the other life history stages are unknown, it is a typical member of the subfamily Grumichellinae. The genus was sister to *Atanatolica* in the phylogeny proposed by Calor and Holzenthal (2008) when both adult and larval characters where included in their analysis, but was recovered as sister to *Amazonatolica* with only adult characters considered.

***manu*** Calor and Holzenthal, 2008:250 [Type locality: Peru, Cuzco, Puente San Pedro, 50 km NW Pilcopata, el. 1600 m; MHNJP, currently housed in NMNH; ♂]. —Flint, 1996b:417 [as probable new genus, n. sp.].

**Distribution.** Peru.

#### Genus *Setodes* Rambur [1 + †2]

*Setodes* Rambur, 1842:515 [Type species: *Setodes punctella* Rambur, 1842, synonym of *Phryganea viridis* Fourcroy 1785, subsequent designation of Milne 1934].

This is a large genus found on all continents except South and Central America and from west of the Great Plains in North America. Schmid (1987) provided a world revision. In the Neotropics, two fossil species have been described from Dominican amber and one living species occurs on Hispaniola. This distribution is anomalous and it is unknown whether the Domincan species are more closely related to eastern North American species, African species, or those from elsewhere.

The immature stages are found in cool, running water where they burrow in sand, often in the lee of large rocks in the stream. The larvae are omnivorous, feeding on both filamentous algae and small animals that wander within capturing distance (Merrill and Wiggins 1971).

***anomalus*** Flint and Sykora, 2004:47 [Type locality: Dominican Republic, Peravia Province, 3 km SW La Nuez, tributary to Río Las Cuevas, 18°40'N , 70°36'W, 1870 m; CMNH; ♂; ♀]. —Pérez-Gelabert, 2008:301 [checklist].

**Distribution.** Dominican Republic.

† ***aureoinclusa*** Wichard, 1995b:170 [Type locality: Dominican Republic; NMNH; ♂; in amber]. —Pérez-Gelabert, 2008:301 [checklist]. —Flint and Pérez-Gelabert, 1999:42 [checklist].

**Distribution.** Dominican Republic.

† ***resinacapta*** Wichard, 1995b:168 [Type locality: Dominican Republic; collection Wichard; ♂; in amber]. —Pérez-Gelabert, 2008:301 [checklist]. —Flint and Pérez-Gelabert, 1999:42 [checklist].

**Distribution.** Dominican Republic.

#### Genus *Triaenodes* McLachlan [26]

*Triaenodes* McLachlan, 1865:110 [replacement name for *Triaena* McLachlan] [Type species: *Leptocerus bicolor* Curtis, 1834, subsequent selection of Ross 1944]. —Holzenthal and Andersen, 2004:1 [revision of Neotropical species, key]. —Manuel, 2010:1 [revision of North American species, key, distribution].

The genus *Triaenodes* is a large, cosmopolitan group of leptocerine caddisflies that is especially diverse in the Old World tropics. In the Neotropics, 26 species occur from southern Mexico to Peru. All except one, *Triaenodes frontalis* Banks a Nearctic species reaching northern Baja California, belong to a morphologically distinct subgroup within the genus. Of the Latin American species, only *Triaenodes columbicus* Ulmer strays from the group pattern as its genitalia resemble those of Old World species. Its type, with the abdomen now lost, was probably mislabelled. *Triaenodes tardus* Milne was listed in the checklist by Rasmussen and Morse (2014) as “Mexico (Hagen, 1861),” but since the species was not described in 1861 this must be a *lapsus calami*. Also, this species was not listed from Mexico in the extensive review of the genus by Manuel (2010) nor in any checklist of Mexican caddisflies.

The larval and pupal stages of the Costa Rican species *Triaenodes tico* were described by Holzenthal and Andersen (2004) and closely resemble immatures of the North American species in both morphology and case architecture. These larvae were collected from mats of riparian tree rootlets submerged along the edge of a small mountain stream, a habitat similar to that of the North American species *Triaenodes taenia* Ross (Manuel and Braatz 1984).

***abruptus*** Flint, 1991:96 [Type locality: Colombia, Dpto. Antioquia, Quebrada La Ayurá, Envigado (trap B); NMNH; ♂]. —Muñoz-Quesada, 2000:279 [checklist]. —Holzenthal and Andersen, 2004:14 [♂; redescription].

**Distribution.** Colombia.

***acanthus*** Holzenthal and Andersen, 2004:15 [Type locality: Mexico, Veracruz, Río Jamapa, 6 km. N Coscomatepec; NMNH; ♂].

**Distribution.** Mexico.

***anomalus*** Flint, 1967b:16 [Type locality: Mexico, Guerrero, near Chilpancingo, route 95, km 297; NMNH; ♂]. /]. —Holzenthal and Andersen, 2004:16 [♂; ♀; redescription; distribution]. —Chamorro-Lacayo et al., 2007:44 [checklist]. —Armitage et al., 2015a:7 [distribution]. —Armitage et al., 2015b:9 [checklist]. —Armitage and Cornejo, 2015:198 [checklist].

**Distribution.** Mexico, Nicaragua, Panama.

***chirripo*** Holzenthal and Andersen, 2004:17 [Type locality: Costa Rica, Cartago, Quebrada Platanillo, ca. 5 km E Moravia de Chirripó, 09°49'16"N, 083°24'25"W, 1130 m; UMSP; ♂; ♀].

**Distribution.** Costa Rica.

***clauseni*** Holzenthal and Andersen, 2004:18 [Type locality: Costa Rica, Alajuela, Cerro Campana, Río Bochinche trib. 6 km (air) NW Dos Rios, 10°56'42"N, 085°24'47"W, el. 600 m; UMSP; ♂; female]. —Chamorro-Lacayo et al., 2007:44 [checklist]. —Armitage et al., 2015b:9 [checklist]. —Armitage and Cornejo, 2015:198 [checklist].

**Distribution.** Costa Rica, Nicaragua, Panama.

***columbicus*** Ulmer, 1909c:141 [Type locality: Colombia; PAN; ♂]. —Flint, 1966a:10 [type abdomen lost]. —Muñoz-Quesada, 2000:279 [checklist]. —Holzenthal and Andersen, 2004:5 [discussion of type].

**Distribution.** Colombia.

***cuyotenango*** Holzenthal and Andersen, 2004:19 [Type locality: Guatemala, Suchitepquez, Cuyotenango; NMNH; ♂].

**Distribution.** Guatemala.

***delicatus*** Navás, 1924c:84 [Type locality: Costa Rica, La Caja; MNHNP; ♂]. —Holzenthal, 1988c:75 [distribution]. —Holzenthal and Andersen, 2004:20 [♂; ♀; redescription; variation, distribution]. —Armitage et al., 2015b:9 [checklist]. —Armitage and Cornejo, 2015:198 [checklist].

**Distribution.** Costa Rica, Panama.

***flintorum*** Holzenthal and Andersen, 2004:23 [Type locality: Mexico, Oaxaca, 8 km S Valle Nacional; NMNH; ♂].

**Distribution.** Mexico.

***frontalis*** Banks, 1907a: 127 [Type locality: USA, Colorado, Fort Collins; MCZ; ♂]. —Manuel, 2010:101 [♂; female/, review, distribution (Baja California)]

**Distribution.** Canada, Mexico, U.S.A.

***guadaloupe*** Holzenthal and Andersen, 2004:24 [Type locality: Panama, Chiriqui, Guadalupe Arriba, 08°52'26"N, 082°33'13"W; NMNH; ♂]. —Armitage et al., 2015b:9 [checklist]. —Armitage and Cornejo, 2015:198 [checklist].

**Distribution.** Panama.

***hodgesi*** Holzenthal and Andersen, 2004:24 [Type locality: Ecuador, Pichincha, Sto. Domingo de los Colorados, 14 km E; NMNH; ♂; ♀].

**Distribution.** Ecuador.

***hornitos*** Holzenthal and Andersen, 2004:25 [Type locality: Panama, Chiriqui, Fortuna Dam Site nr. Hornitos, 08°55'00"N, 082°16'00"W, 1050 m; NMNH; ♂]. —Armitage et al., 2015b:9 [checklist]. —Armitage and Cornejo, 2015:198 [checklist].

**Distribution.** Panama.

***kilambe*** Holzenthal and Andersen, 2004:26 [Type locality: Nicaragua, Jinotega, Cerro Kilambé, 13°34'00"N, 085°43'00"W, el. 1520 m; UMSP; ♂]. —Chamorro-Lacayo et al., 2007:44 [checklist].

**Distribution.** Nicaragua.

***mexicanus*** Holzenthal and Andersen, 2004:27 [Type locality: Mexico, Morélos, Cuernavaca, vi.1911; BMNH; ♂].

**Distribution.** Mexico.

***moncho*** Holzenthal and Andersen, 2004:28 [Type locality: Costa Rica, Alajuela, Reserva Forestal San Ramón, Río San Lorencito & tribs., 10°12'58"N, 084°36'25"W, el. 980 m; UMSP; ♂].

**Distribution.** Costa Rica.

***morai*** Holzenthal and Andersen, 2004:29 [Type locality: Costa Rica, Alajuela, Reserva Forestal San Ramón, Río San Lorencito & tribs., 10°12'58"N, 084°36'25"W, el. 980 m; UMSP; ♂]. —Chamorro-Lacayo et al., 2007:44 [checklist].

**Distribution.** Costa Rica, Nicaragua.

***nicaraguensis*** Holzenthal and Andersen, 2004:29 [Type locality: Nicaragua, Matagalpa, 50 km E Matagalpa, El Coyolar, 85°50'00"N, 013°07'00"W; NMNH; ♂]. —Chamorro-Lacayo et al., 2007:44 [checklist].

**Distribution.** Nicaragua.

***oaxacensis*** Holzenthal and Andersen, 2004:30 [Type locality: Mexico, Oaxaca, 1 mi. NE of Ixtlan de Juarez; NMNH; ♂].

**Distribution.** Mexico.

***peruanus*** Flint and Reyes, 1991:488 [Type locality: Peru, Dept. La Libertad, Prov. Trujillo, Dist. Simbal, Río Lucumar, Simbal; NMNH; ♂]. —Holzenthal and Andersen, 2004:31 [♂; ♀; redescription; distribution].

**Distribution.** Colombia, Ecuador, Peru.

***tajo*** Holzenthal and Andersen, 2004:32 [Type locality: Costa Rica, Puntarenas, Río Jaba, rock quarry, 1.4 km (air) W Las Cruces, 08°47'24"N, 082°58'12"W, el. 1150 m; UMSP; ♂].

**Distribution.** Costa Rica.

***talamanca*** Holzenthal and Andersen, 2004:33 [Type locality: Costa Rica, Puntarenas, Río Guineal, ca 1 km (air) E Finca Helechales, 09°04'34"N, 083°05'31"W, el. 840 m; UMSP; ♂]. —Armitage et al., 2015a:8 [distribution]. —Armitage et al., 2015b:9 [checklist]. —Armitage and Cornejo, 2015:198 [checklist].

**Distribution.** Costa Rica, Panama.

***tapanti*** Holzenthal and Andersen, 2004:34 [Type locality: Costa Rica, Cartago, Reserva Tapantí, unnamed tribs [QuebradaPalmitos & falls], ca. 9 km (road) NW tunnel, 09°43'12"N, 083°46'48"W, el. 1400 m; UMSP; ♂]. —Armitage et al., 2015b:9 [checklist]. —Armitage and Cornejo, 2015:198 [checklist].

**Distribution.** Costa Rica, Panama.

***tico*** Holzenthal and Andersen, 2004:35 [Type locality: Costa Rica, San José, Río Parrita Chiquito, rt. 12, 6.5 km SW jct. rt. 2, 09°42'11"N, 083°58'12"W, el. 1990 m; UMSP; ♂; ♀; larva; pupa; biology]. —Armitage et al., 2015b:9 [checklist]. —Armitage and Cornejo, 2015:198 [checklist].

**Distribution.** Costa Rica, Panama.

***tuxtlensis*** Holzenthal and Andersen, 2004:37 [Type locality: Mexico, Veracruz, Los Tuxtlas Biological Station, Los Tuxtlas area, Río Palma, above La Palma; NMNH; ♂].

**Distribution.** Mexico.

***woldai*** Holzenthal and Andersen, 2004:38 [Type locality: Panama, Bocas del Toro, Miramar, 09°00'00"N, 082°15'00"W; NMNH; ♂]. —Armitage et al., 2015b:9 [checklist]. —Armitage and Cornejo, 2015:198 [checklist].

**Distribution.** Panama.

#### Genus *Triplectides* Kolenati [16]

*Triplectides* Kolenati, 1859:247 [Type species: *Mystacides gracilis* Burmeister, 1839, subsequent designation of Mosely, 1936a]. —Mosely, 1936a:91 [revision]. —Holzenthal, 1988a:187 [revision].

*Tetracentron* Brauer 1865:418 [Type species: *Tetracentron sarothrops* Brauer, 1865, by monotypy]. —Mosely, 1936a:92 [to synonymy].

This primarily southern hemisphere genus contains about 80 species, making it the largest in its subfamily. Morse and Neboiss (1982) reviewed the Australian species and Holzenthal (1988a) reviewed the Neotropical species. Sixteen species are known from the Neotropics and occur from southern Mexico to Patagonia.

The larvae are easily recognized by their cases, which are often constructed of a hollowed-out twig or a discarded case of another trichopteran, and have been described a number of times (Holzenthal 1988a, Crisci-Bisbo et al. 2004, Camargos and Pes 2011, Sganga et al. 2013). Larvae are found in pools of small streams where they undoubtledly feed as detrital shredders.

***chilensis*** Holzenthal, 1988a:191 [Type locality: Chile, Osorno, Parque Nacional Puyehue, Aguas Calientes; NMNH; ♂; ♀].

**Distribution.** Argentina, Chile.

***cipo*** Henriques-Oliveira and Dumas, 2015:67 [Type locality: Brazil, Minas Gerais, Jaboticatubas, Parque Nacional da Serra do Cipó, Córrego das Pedras, 19°22'16.7"S, 48°36'2.8"W, el. 766 m; DZRJ; ♂; ♀].

**Distribution.** Brazil.

***colombicus*** Navás, 1916b:67 [Type locality: Colombia, Choachí; type depository not designated; stated as ♀; but ♂ according to Holzenthal, 1988a:193]. —Mosely, 1936a:126 [as *Triplectides* ?]. —Holzenthal, 1988a:193 [*nomen dubium*]. —Muñoz-Quesada, 2000:279 [checklist].

**Distribution.** Colombia.

***egleri*** Sattler, 1963c:20 [Type locality: Brazil, Pará, Belém; type depository not designated; ♂; ♀; larva; pupa]. —Flint, 1974c:137 [♂; redescription; distribution]. —Holzenthal, 1988a:193 [redescription; distribution]. —Paprocki et al., 2004:13 [checklist]. —Sganga et al., 2013:27 [larva; differentiation from other species]. —Paprocki and França, 2014:63 [checklist]. —Gomes de Brito et al., 2015:332 [ecology].

**Distribution.** Brazil, Guyana, Suriname.

***flintorum*** Holzenthal, 1988a:193 [Type locality: Costa Rica, Guanacaste, 2.5 mi. W Tilarán; NMNH; ♂; ♀].—Ulmer, 1907b:41 [misidentification as *gracilis*, in part]. —Mosely, 1936a:96 [misidentification as *gracilis*, in part]. —Flint, 1974c:137 [as *gracilis*, in part]. —Bueno-Soria and Flint, 1978:212 [misidentification as *gracilis*, in part]. —Holzenthal, 1988c:75 [distribution]. —Maes and Flint, 1988:7 [misidentification as *gracilis*, in part]. —Flint, 1991:87 [♂; distribution]. —Aguila, 1992:544 [distribution]. —Flint, 1996b: 418 [distribution]. —Maes, 1999:1198 [checklist]. —Muñoz-Quesada, 2000:279 [checklist]. —Medellín et al., 2004:201 [distribution; biology]. —Chamorro-Lacayo et al., 2007:45 [checklist]. —Bueno-Soria and Barba-Álvarez, 2011:359 [checklist]. —Armitage et al., 2015b:9 [checklist]. —Armitage and Cornejo, 2015:198 [checklist].

**Distribution.** Colombia, Costa Rica, Ecuador, Guatemala, Honduras, Mexico, Nicaragua, Panama, Peru, Suriname.

***gracilis*** (Burmeister), 1839:921 [Type locality: Brazil, Nova Friburgo; ZIUH (destroyed); ♂; in *Mystacides*]. —Ulmer, 1905a:27 [redescription ♂ type]. —Mosely, 1936a:96 [♂]. —Holzenthal, 1988a:195 [Neotype: Brazil, Rio de Janeiro, Nova Friburgo, municipal water supply; NMNH; ♂; ♀; redescription; distribution]. —Almeida and Marinoni, 2000:349 [distribution; biology]. —Paprocki et al., 2004:13 [checklist]. —Angrisano and Sganga, 2007:46 [♂; distribution]. —Dumas et al., 2009:370 [distribution]. —Calor, 2011:322 [checklist]. —Dumas and Nessimian, 2012:18 [distribution]. —Sganga et al., 2013:26 [larva; pupa; distribution]. —Manzo et al., 2014:167 [distribution]. —Paprocki and França, 2014:64 [checklist]. —Quinteiro et al., 2014:235 [distribution]. —Dias et al., 2015:377 [distribution]. —Moretto and Bispo, 2015:126 [distrtibution].

—*princeps* (Burmeister), 1839:921 [Type locality: Brazil, Nova Friburgo; ZIUH (destroyed); ♂; in *Mystacides*]. —Ulmer, 1905a:27 [to synonymy].

—*ramulorus* (Müller), 1921:541, 543, figs. 189bb, 194bd, 234 [Type locality: Brazil, Santa Catarina; no type nor type depository designated; larva; pupa; in *Tetracentron*]. —Holzenthal, 1988a:195 [to synonymy].

**Distribution.** Argentina, Brazil, Paraguay, Suriname.

***itatiaia*** Dumas and Nessimian, 2010b:949 [Type locality: Brazil, Rio de Janeiro, Itatiaia, Parque Nacional do Itatiaia, Rio Tapera, 22°26'59.64"S, 44°36'19.39"W, el. 794 m; DZRJ; ♂]. —Dumas and Nessimian, 2012:19 [distribution]. —Paprocki and França, 2014:64 [checklist].

**Distribution.** Brazil.

***jaffuelli*** Navás, 1918d:9 [Type locality: Chile, Marga-Marga; MZBS; ♂]. —Mosely, 1936a:126 [redescription]. —Schmid, 1949a:352 [♂]. —Flint 1974e:90 [checklist]. —Holzenthal, 1988a:198 [♂; ♀]. —Flint, 1990:119 [distribution]. —Cohen, 2004:78 [distribution]. —Sganga et al., 2013:27 [larva; differentiation from other species].

—*monotona* Navás, 1918c:225 [Type locality: Chile, Marga-Marga, Los Perales; MZBS; ♂]. —Schmid, 1949a:358 [to synonymy].

—*robustus* Schmid, 1955a:136 [Type locality: Chile, Isla de Chilóe, Ancud; NMNH; ♂]. —Flint 1974e:90 [checklist]. —Holzenthal, 1988a:198 [to synonymy].

**Distribution.** Argentina, Chile.

***misionensis*** Holzenthal, 1988a:198 [Type locality: Argentina, Misiones, Arroyo Piray Guazú, N San Pedro, NMNH; ♂; ♀]. —Blahnik et al., 2004:5 [distribution]. —Paprocki et al., 2004:13 [checklist]. —Dumas et al., 2009:371 [distribution]. —Dumas et al., 2010:8 [distribution]. —Calor, 2011:322 [checklist]. —Dumas and Nessimian, 2012:19 [distribution]. —Sganga et al., 2013:24 [larva; pupa; distribution]. —Paprocki and França, 2014:64 [checklist].

**Distribution.** Brazil, Argentina.

***neblinus*** Holzenthal, 1988a:199 [Type locality: Venezuela, Territorio Federal Amazonas, basecamp, 0°51'N, 66°10'W, Cerro de la Neblina; NMNH; ♂].

**Distribution.** Venezuela.

***neotropicus*** Holzenthal, 1988a:200 [Type locality: Venezuela, Territorio Federal Amazonas, camp IV, 0°58'N, 65°57'W, Cerro de la Neblina; NMNH; ♂]. —Blahnik et al., 2004:5 [distribution]. —Paprocki et al., 2004:13 [checklist]. —Dumas et al., 2009:371 [distribution]. —Dumas et al., 2010:8 [distribution]. —Calor, 2011:322 [checklist]. —Dumas and Nessimian, 2012:19 [distribution]. —Paprocki and França, 2014:64 [checklist].

**Distribution.** Brazil, Venezuela.

***nevadus*** Holzenthal, 1988a:202 [Type locality: Venezuela, Territorio Federal Amazonas, 2 E San Carlos de Río Negro; NMNH; ♂; ♀].

**Distribution.** Peru, Venezuela.

***nigripennis*** Mosely, 1936a:97 [Type locality: Argentina, Terr. Río Negro, Bariloche; BMNH; ♂; ♀]. —Flint 1974e:90 [checklist]. —Holzenthal, 1988a:202 [redescription]. —Cohen, 2004:74, 78 [list of type material, distribution].

—*multipunctatus* Schmid, 1955a:136 [Type locality: Chile, Isla de Chilóe, Ancud; NMNH; ♂]. —Flint 1974e:90 [checklist]. —Holzenthal, 1988a:203 [to synonymy].

**Distribution.** Argentina, Chile.

***qosqo*** Henriques-Oliveira and Dumas, 2015:67 [Type locality: Peru, Cuzco, 19 rd km W Quincemil, Río Araza tributary, 12°20'10"S, 70°50'5"W, el. 874 m; MHNJP; ♂].

**Distribution.** Peru.

***tepui*** Holzenthal, 1988a:203 [Type locality: Venezuela, Territorio Federal Amazonas, camp II, 0°49'N, 65°59'W, Cerro de la Neblina; NMNH; ♂; ♀].

**Distribution.** Venezuela.

***ultimus*** Holzenthal, 1988a:205 [Type locality: Brazil, Rio de Janeiro, Itatiaia; MZSP; ♂; ♀]. —Paprocki et al., 2004:13 [checklist]. —Dumas et al., 2009:371 [distribution]. —Dumas et al., 2010:8 [distribution]. —Dumas and Nessimian, 2012:19 [distribution]. —Paprocki and França, 2014:64 [checklist].

**Distribution.** Brazil.

### Family Limnephilidae

This large family of some 1000 species is one of the dominant families in the northern regions and high elevations of North America and Eurasia. None are knowv from tropical Africa, and no more than a couple from Australia. In Mexico and the mountains of Central America, as far south as Panama, several species occur, but these are clearly part of a temperate, western North American cordilleran fauna. In South America several endemic genera and species occur, all members of the subfamily Dicosmoecinae, but most of these are restricted to the high Andes of Boliva, Colombia, Ecuador, and Peru or to Patagonia, where several dozen species occur. None occur in the topical lowlands. These species are perhaps persistent representatives of an ancient trans-Antarctic fauna, a distributional pattern typical of the austral South American caddisfly fauna as a whole. The occurrence of a species in southeastern Brazil is notable. In total, 10 genera and 51 species are currently known from Mexico and the Neotropics and all but 3 genera and 19 species are primarily restricted to the southern cone of the South American landmass.

#### Genus *Anomalocosmoecus* Schmid [4]

*Anomalocosmoecus* Schmid 1957:390 [Type species: *Anomalocosmoecus blancasi* Schmid, 1957, original designation]. —Flint, 1982b:3 [larva].

This South American endemic genus of four species was originally established for *Anomalocosmoecus blancasi* Schmid from Lake Titicaca. A second species, *Anomalocosmoecus illiesi* (Marlier), is known from high elevations in the Andes of Peru, Ecuador, and southern Colombia. The third and fourth species, *Anomalocosmoecus argentinica* Flint and *Anomalocosmoecus subtropicalis* (Schmid), are known from Argentina and Peru, respectively, although the identity of the latter species in questionable.

Immature stages of *Anomalocosmoecus illiesi* were described by Marlier (1963) and larvae of *Anomalocosmoecus blancasi* by Flint (1982b). Larvae of *Anomalocosmoecus illiesi* were reported from small mountain streams in the páramo and upper yungan areas whereas those of *Anomalocosmoecus blancasi* occurred among *Elodea* and *Scirpus* beds in the littoral zone of Lake Titicaca, as well as on the bottom, 5 m below the surface (Flint 1982b).

***argentinicus*** Flint, 1983a:66 [Type locality: Argentina, Pcia. Salta, Malcante, rt. 59, km 32, W Chicoana; NMNH; pharate ♂; ♀; few larval characters]. —Isa Miranda and Rueda Martín, 2014:200 [distribution].

**Distribution.** Argentina.

***blancasi*** Schmid, 1957:390 [Type locality: Peru, Lake Titicaca, Pomata; ROM; ♂]. —Flint, 1980a:216 [distribution]. —Flint, 1982b:3 [larva].

**Distribution.** Bolivia/Peru [Lake Titicaca].

***illiesi*** (Marlier), 1962b:5 [Type locality: Peru, Río Huallaga; IRSNB; ♂; in *Magellomyia*]. —Marlier 1963:243 [larva; pupa]. —Flint, 1982b:3 [to *Anomalocosmoecus*]. —Botosaneanu and Flint, 1982:19 [distribution]. —Flint, 1991:85 [distribution]. —Muñoz-Quesada, 2000:279 [checklist].

**Distribution.** Colombia, Ecuador, Peru.

***subtropicalis*** (Schmid), 1955a:146 [Type locality: Peru, Cuzco, Hacienda Urco, near Calca; FMNH, type now missing according to Flint, 1982b; ♀; in *Magellomyia*]. —Flint, 1982b:3 [to *Anomalocosmoecus*].

**Distribution.** Peru.

#### Genus *Antarctoecia* Ulmer [2]

*Antarctoecia* Ulmer, 1907d:61 [Type species: *Dicosmoecus nordenskioeldii* Ulmer, 1905b, by monotypy]. —Schmid, 1955b:43 [diagnosis].

This genus, now of two species, is known with certainly only from elevations of 4500–5000 meters in the provinces of Jujuy and Catamarca, Argentina, and from southeastern Brazil.

Flint (1982b) attributed larvae from Cochabamba, Bolivia, to the genus, but cautioned that they might represent an undescribed species or genus. These larvae built circular, slightly tapered and curved, “shaggy” cases of transversely or obliquely placed plant material. They occurred in the puna grassland in meadow pools along small, spring-fed creeks. The larva described from Brazil lived on the rocky bottom, in the fast flowing area of a third order stream; gut contents of algae indicated that the larvae fed as scrapers (Huamantinco and Nessimian 2003). Cases were made of mineral fragments.

***brasiliensis*** Huamantinco and Nessimian, 2003:226 [Type locality: Brazil, Minas Gerais, Itamonte, Rio Aiuruoca, 1860 m, 22°20'9.28"S, 44°4'6.06"W; DZRJ; ♂; ♀]. —Huamantinco and Nessimiana, 2004a:2 [larva; pupa]. —Paprocki et al., 2004:13 [checklist]. —Dumas and Nessimian, 2012:20 [distribution]. —Paprocki and França, 2014:64 [checklist].

**Distribution.** Brazil.

***nordenskioeldii*** (Ulmer), 1905b:65 [Type locality: Argentina, Chani, Puna de Jujuy; NRS; ♂; in *Dicosmoecus*]. —Ulmer 1907d:61 [to *Antarctoecia*]. —Schmid, 1949b:594 [redescription; ♂; ♀]. —Flint, 1982b:4 [tentative larva].

**Distribution.** Argentina, Bolivia [?].

#### Genus *Austrocosmoecus* Schmid [1]

*Austrocosmoecus* Schmid, 1955b:54 [Type species: *Austrocosmoecus hirsutus* Schmid, 1955b, by monotypy]. —Flint, 1982b:6 [larva].

The single species in this genus is widespread and common in small mountain streams in the Andes from the Straits of Magellan north to south-central Chile and adjacent Argentina.

Larval cases are variable, but usually made of plant matter (Flint 1982b). Larvae most commonly are associated with the *Nothofagus* forest, where they crawl in leaf-litter piles in small, clear, cool, fast flowing streams.

***hirsutus*** Schmid, 1955b:55 [Type locality: Chile, Malleco, Río Blanco; ROM; ♂; ♀]. —Ulmer, 1904:14 [in part, larvae only, as *Stenophylax hyadesi* (Mabille), 1888]. —Flint, 1974e:84, 88 [larva described by Ulmer as *Stenophylax hyadesi* is this species, checklist]. —Flint, 1982b:6 [larva; distribution]. —Cohen, 2004:78 [distribution]. —Brand and Miserendino, 2011a:35 [biology; habitat].

**Distribution.** Argentina, Chile.

#### Genus *Clistoronia* Banks [1]

*Clistoronia* Banks, 1916:119 [Type species: *Halesus magnus* Banks, 1899 [recte *magnificus*], by monotypy]. —Ross and Merkley, 1952:436 [as synonym of *Limnephilus*]. —Schmid, 1955b:154 [redescription].

This genus contains four species distributed over the western North American cordillera, from Alaska to Durango, Mexico. Larvae of *Clistoronia magnifica* were described by Wiggins (1996) and its biology was studied by Winterbourn (1971). Larvae build cases of small pieces of irregularly arranged wood and, at least in *Clistoronia magnifica*, live in small lakes and ponds at high elevations. Anderson and Belnavis (1991) gave an account of rearing the species in laboratory culture for 30 generations.

***graniculata*** (Denning), in Denning and Sykora, 1966:1223 [Type locality: Mexico, Durango, ten miles west of El Salto; CAS; ♂; ♀; in *Limnephilus*]. —Flint, 1967d:171 [to *Clistoronia*; distribution]. —Bueno-Soria and Flint, 1978:209 [distribution].

**Distribution.** Mexico.

#### Genus *Hesperophylax* Banks [2]

*Hesperophylax* Banks, 1916:118 [Type species: *Platyphylax occidentalis* Banks, 1908, by monotypy]. —Parker and Wiggins, 1985:2444 [revision].

Parker and Wiggins (1985) recognized seven species in this genus. Species occur across the northern part of the North American continent, throughout the western mountains, and in the Mexican transvolcanic belt. Several species found in the mountains of southern Arizona undoubtedly also occur in adjacent Mexico.

Larvae build slightly curved and tapered cases of rock fragments, and are usually associated with depositional areas in running waters. They seem to be broadly omnivorous, feeding on both plant and animal material. The larvae of *Hesperophylax mexicanus* occured among aquatic plants in a small spring-fed stream at 2,820 m elevation in the mountains south of Mexico City (Bueno-Soria et al., 1981). Gut contents of larvae contained small pieces of vascular plant tissue as well as detritus (Parker and Wiggins 1985).

***magnus*** Banks, 1918:20 [Type locality: Arizona, Cochise Co., Palmerlee; MCZ; ♂]. —Ross, 1938a:32 [♂ lectotype]. —Flint, 1967d:171 [distribution]. —Bueno-Soria and Flint, 1978:209 [distribution]. —Parker and Wiggins, 1985:2461 [♂; ♀; larva]. —Blinn and Ruiter, 2006:333 [distribution; biology]. —Blinn and Ruiter, 2009a:303 [biology]. —Blinn and Ruiter, 2009b:186 [phenology, distribution].

**Distribution.** Mexico, U.S.A.

***mexico*** Parker and Wiggins, 1985:2464 [Type locality: Mexico, Morelos, P.N. Lagunas de Zempoala; NMNH; ♂; ♀; larva].

**Distribution.** Mexico.

#### Genus *Limnephilus* Leach [13]

*Limnephilus* Leach, 1815:136 [Type species: *Phryganea rhombica* Linnaeus, 1758, by monotypy]. —Fischer, 1968:1 [synonymy, catalog]. —Ruiter, 1995:1 [revision North American species].

This large genus of over 150 species occurs throughout the Holarctic Region, with outliers extending southwardly in the higher elevation of Mexico and Central America to central Costa Rica. The approximately 100 North American species were reviewed by Ross and Merkley (1952) and by Ruiter (1995), where all species are illustrated as well. Vshivkova et al. (2007) reviewed the higher-level phylogeny and classification of *Limnephilus* and related genera.

The larvae of many species have been described from Europe, northern Asia, and North America (Hickin 1967, Lepneva 1971, Wiggins 1996). They make a diverse variety of cases, incorporating mineral matter, plant fragments, and sometimes small molluscan shells, crustaceans and caddisfly cases. In general they occupy lentic sites, including small vernal pools, marshes, tarns, ponds, and lakes; some are also known from slower reaches of rivers and streams, or even cold springs. They generally feed on plant matter, although they will feed opportunistically on dead animal matter.

***baja*** Ruiter, 1995:24 [Type locality: Mexico, Baja California Norte, 10.1 miles west of Catavina; NMNH; ♂; ♀].

**Distribution.** Mexico.

***biparta*** Denning, in Denning and Sykora, 1966:1223 [Type locality: Mexico, Durango, ten miles west of El Salto; CAS; ♂]. —Flint, 1967d:170 [distribution]. —Bueno-Soria and Flint, 1978:210 [distribution]. —Ruiter, 1995:26 [♂; ♀; redescription].

**Distribution.** Mexico.

***ctenifer*** Flint, 1967d:170 [Type locality: Mexico, Durango, 10 miles west of El Salto; CNC; ♂; ♀]. —Bueno-Soria and Flint, 1978:210 [distribution]. —Ruiter, 1995:26 [♂; ♀; redescription].

**Distribution.** Mexico.

***discolor*** (Banks), 1901:367 [Type locality: Mexico, D.F., Tacubaya; MCZ; ♂; in *Platyphylax*]. —Flint, 1963b:211 [♂; redescription]. —Denning and Sykora, 1966:1222 [♀; distribution]. —Bueno-Soria and Flint, 1978:210 [distribution]. —Ruiter, 1995:28 [♂; ♀; redescription].

**Distribution.** Mexico.

***frijole*** Ross, 1944:282 [Type locality: Texas, Frijole, Manzaneta Spring; INHS; ♂; ♀]. —Denning, 1964:134 [checklist]. —Bueno-Soria and Flint, 1978:210 [distribution]. —Ruiter 1995:30 [♂; ♀; redescription; distribution]. —Blinn and Ruiter, 2006:333 [distribution; biology].

**Distribution.** Mexico, U.S.A.

***hamifer*** Flint, 1963b:212 [Type locality: Costa Rica, Mount Poas; NMNH; ♂]. —Holzenthal, 1988c:71 [distribution]. —Ruiter, 1995:30 [♂; ♀; redescription]. —Armitage et al., 2015a:8 [distribution]. —Armitage et al., 2015b:9 [checklist]. —Armitage and Cornejo, 2015:198 [checklist].

**Distribution.** Costa Rica, Panama.

***lithus*** (Milne), 1935:40 [Type locality: Colo., Boulder; MCZ; ♂; in *Anabolina*]. —Ross, 1944:298 [to *Limnephilus*]. —Ross and Merkley, 1952:450 [♂]. —Ruiter, 1995:13 [♂; ♀; redescription; distribution]. —Blinn and Ruiter, 2006:333 [distribution; biology]. —Blinn and Ruiter, 2009a:303 [biology]. —Blinn and Ruiter, 2009b:186 [phenology, distribution].

**Distribution.** Mexico, U.S.A.

***maya*** Flint, 1967b:16 [Type locality: Guatemala, El Progreso, Finca la Cajeta; NMNH; ♂]. —Bueno-Soria and Flint, 1978:210 [distribution]. —Holzenthal, 1988c:71 [distribution; record doubtful]. —Ruiter 1995:31 [♂; ♀; redescription; distribution]. —Bueno-Soria and Barba-Álvarez, 2011:359 [checklist].

**Distribution.** Costa Rica[?], Guatemala, Honduras, Mexico.

***mexicanus*** Flint, 1967d:171 [Type locality: Mexico, Durango, 10 miles west of El Salto; CNC; ♂; ♀]. —Bueno-Soria and Flint, 1978:210 [distribution]. —Ruiter, 1995:31 [♂; ♀; redescription].

**Distribution.** Mexico.

***pollux*** Flint, 1967d:169 [Type locality: Mexico, Durango, 10 miles west of El Salto; CNC; ♂; ♀]. —Bueno-Soria and Flint, 1978:210 [distribution]. —Ruiter, 1995:33 [♂; ♀; redescription].

**Distribution.** Mexico.

***rothi*** Denning, 1966:235 [Type locality: Arizona, Cochise County, Southwestern Research Station, 5 miles west of Portal; CAS; ♂; ♀]. —Flint, 1967d:169 [distribution]. —Bueno-Soria and Flint, 1978:210 [distribution]. —Ruiter, 1995:28 [♂; ♀; redescription; distribution]. —Blinn and Ruiter, 2006:333 [distribution; biology].

**Distribution.** Mexico, U.S.A.

***solidus*** (Hagen), 1861:267 [Type locality: Mexico; ZMHU; ♀; in *Hallesus*]. —Bueno-Soria and Flint, 1978:210 [distribution]. —Ruiter, 1995:34 [♂; ♀; redescription].

—*toussianti* Banks, 1924:439 [Type locality: Haiti, Port au Prince; MCZ; ♂]. —Flint, 1963b:211 [♂; redescription; type locality in error?; not Haiti, emended to *toussainti*]. —Flint, 1967d:169 [distribution]. —Ruiter, 1995:34 [to synoymy].

**Distribution.** Haiti[?], Mexico.

***tulatus*** Denning, 1962a:36 [Type locality: Arizona, Santa Cruz County, Sycamore Creek, near Ruby; CAS; ♂]. —Bueno-Soria and Flint, 1978:210 [distribution]. —Ruiter, 1995:13 [♂; ♀; redescription; distribution]. —Blinn and Ruiter, 2006:333 [distribution; biology]. —Bueno-Soria et al., 2007:33 [distribution].

**Distribution.** Mexico, U.S.A.

#### Genus *Metacosmoecus* Schmid [1]

*Metacosmoecus* Schmid, 1955b:39 [Type species: *Metacosmoecus nigrofasciatus* Schmid, 1955b, by monotypy]. —Flint, 1982b:10 [larva].

The single species in this genus is known from central Chile where it is infrequently encountered. Larvae burrow in sandy deposits in small streams, but are also known to burrow into accumulations of sand and silt beneath moss growing on vertical rock surfaces. Cases are made of small rock fragments (Flint 1982b).

***nigrofasciatus*** Schmid, 1955b:40 [Type locality: Chile, Isla de Chiloé, Aulen; ROM; ♂]. —Schmid, 1957:387 [variation, distribution]. —Flint, 1974e:89 [checklist]. —Flint, 1982b:10 [larva].

**Distribution.** Chile.

#### Genus *Monocosmoecus* Ulmer [6]

*Monocosmoecus* Ulmer, 1906:13 [Type species: *Monocosmoecus vanderweeli* Ulmer, 1906, subsequent selection of Schmid 1955b]. —Schmid, 1955b:62 [diagnosis]. —Flint, 1982b:11 [larva].

*Isocentropus* Navás, 1918a:497 [Type species: *Isocentropus lutzinus* Navás, 1918a = *Halesus hyadesi* Mabille 1888, by monotypy]. —Schmid, 1955b:62 [to synonymy].

*Nolga* Navás, 1929a:333 [Type species: *Nolga truncata* Navás, 1929a = *Monocosmoecus vanderweeli* Ulmer 1906, by monotypy]. —Schmid, 1949a:409 [to synonymy].

*Monocosmoecus* contains six species. Species occur widely from Tierra del Fuego through Patagonia to the province of Coquimbo, Chile. Larvae of three species have been described: *Monocosmoecus obtusus* by Flint (1967a), *Monocosmoecus hyadesi* by Ulmer (1904), and *Monocosmoecus vanderweeli* by Flint (1982b). Species occur in a variety of aquatic habitats, including very small mountain brooks, large clear rivers, lakes, and even artificial watercourses (Flint 1982b). Cases are made of mineral fragments with wood incorporated posteriorly.

***aberrans*** Flint, 1969b:508 [Type locality: Argentina, Prov. Río Negro, Puerto Blest; NMNH; ♂].

**Distribution.** Argentina.

***hyadesi*** (Mabille), 1888:7 [Type locality: [Chile, Isla Hoste] Terre de Feu, baie Orange; MNHNP; ♂; in *Halesus*]. —Ulmer, 1906:19 [♂]. —Flint, 1974e:84, 89 [distribution].

—*branchiatus* (Ulmer), 1904:17 [Type locality: Süd Patagonien, Punta Arenas; lacking in ZSZMH; larva; in *Stenophylax*]. —Flint, 1974e:84 [to synonymy].

—*lutzinus* (Navás), 1918a:498 [Type locality: Argentina, Santa Cruz, Valle Túnel; MACN; ♂; in *Isocentropus*]. —Schmid, 1955b:64 [to synonymy].

**Distribution.** Argentina, Chile.

***minor*** Schmid, 1955a:147 [Type locality: Chile, Malleco, Río Blanco; ROM; ♂]. —Flint, 1974e:89 [checklist].

**Distribution.** Chile.

***obtusus*** Schmid, 1957:386 [Type locality: Chile, Ñuble, Las Trancas; ROM; ♂]. —Flint, 1967a:58 [larva; pupa; distribution]. —Flint, 1974e:89 [checklist]. —Cohen, 2004:79 [distribution].

**Distribution.** Argentina, Chile.

***pulcher*** Ulmer, 1906:16 [Type locality: Argentina[?], Tierra de Fuego, “Rio Mc. Clelland”; BMNH; ♂]. —Cohen, 2004:79 [distribution].

—*olens* Döhler, 1915:409 [Type locality: Chile; ZMHU; ♀]. —Flint, 1974e:89 [checklist]. —Flint et al., 1999a:79 [to synonymy].

—*pulcherrimus* Schmid, 1955a:146 [Type locality: Argentina, Neuquen, Nantuc; ROM; ♂]. —Flint, 1974e:89 [checklist]. —Flint et al., 1999a:80 [to synonymy]. —Cohen, 2004:79 [distribution].

**Distribution.** Argentina, Chile.

***vanderweeli*** Ulmer, 1906:13 [Type locality: Argentina, Chubut; ZSZMH; ♂]. —Schmid, 1949a:409 [♂]. —Flint, 1974e:89 [checklist]. —Schmid 1955b:64 [type species of genus]. —Flint, 1982b:12 [larva].

—*truncatus* (Navás), 1929a:333 [Type locality: Chile, Angol; collection Navás, lost; ♂; in *Nolga*]. —Schmid, 1949a:409 [Neotype: Chile, Angol; MZBS?; ♂; to *Monocosmoecus*]. —Schmid 1964:326 [to synonymy].

—*calceatus* (Navás), 1930b:364 [Type locality: Chile, Pilahueque, Dumo; type depository not given; ♀; in *Nolga*]. —Schmid, 1955b:64 [to synonymy of *truncatus*].

**Distribution.** Argentina, Chile.

#### Genus *Platycosmoecus* Schmid [1]

*Beaumontia* Schmid, 1958 [Type species: *Beaumontia beaumonti* Schmid, 1958b:206, original designation; preoccupied].

*Platycosmoecus* Schmid, 1964:326 [replacement name for *Beaumontia*, preoccupied. Type species: *Beaumontia beaumonti* Schmid, 1958b:207]. —Flint, 1982b:13 [larva].

This is yet another monotypic genus known only from the Chilean Subregion. The single species is large and distinctive and known from Ñuble south to Magallanes in Chile and adjacent Argentina. The larval stage occurs in small, fast-flowing mountain streams in the *Nothofagus* forest. They live among growths of mosses from which they build their cases (Flint 1982b).

***beaumonti*** (Schmid), 1958b:207 [Type locality: Chile, Chiloé, Río Carihueco; ROM; ♂; as *Beaumontia beaumontia*, lapsus for *beaumonti*]. —Schmid 1964:326 [to *Platycosmoecus*]. —Flint, 1974e:89 [distribution] . —Flint, 1982b:13 [larva].

**Distribution.** Argentina, Chile.

#### Genus *Verger* Navás [20]

*Verger* Navás, 1918b:362 [Type species: *Halesus porteri* Navás, 1907, by monotypy]. —Schmid, 1955b:50, 53 [as synonyn of *Magellomyia*].

*Nostrafilla* Navás, 1918a:499 [Type species: *Nostrafilla lutzi* Navás, 1918a, original designation]. —Schmid, 1955b:50 [possible synonym of *Magellomyia*]. —Flint et al., 1999a:79 [to synonymy].

*Magellomyia* Banks, 1920:348 [Type species: *Magellomyia moesta* Banks, 1920 = *Stenophylax appendiculatus* Ulmer, 1904, original designation]. —Flint, 1982b:8 [larva]. —Flint et al., 1999a:79 [to synonymy].

*Australomyia* Schmid, 1949b:600 [Type species: *Limnephilus meridionalis* Ulmer, 1905c = *Stenophylax appendiculatus* Ulmer, 1904, original designation]. —Schmid, 1955b:50, 52 [as synonym of *Magellomyia*].

This is the largest dicosmoecine genus in the Neotropics, containing 20 described species. They are distributed from Tierra del Fuego, north into north central Chile and Argentina.

The larvae are generally found in lentic waters or backwaters of rivers and streams. Some species are known to inhabit vernal pools (Flint 1971b). They employ a variety of material in their cases, both plant and mineral particles being used, depending in part on the species. They seem to be mostly detritivores, feeding on course particulate organic matter and assimilating the microorganisms growing on leaf letter (Diáz Villanueva and Trochine 2005)

***affinis*** (Schmid), 1955a:144 [Type locality: Chile; ROM; ♂; in *Magellomyia*]. —Schmid, 1964:324 [diagnosis; distribution]. —Flint, 1974e:88 [checklist]. —Flint et al., 1999a:79 [to *Verger*]. —Brand, 2009:224 [distribution].

**Distribution.** Argentina, Chile.

***appendiculatus*** (Ulmer), 1904:19 [Type locality: [Chile] Feuerländischer Archipel, Isla Picton, Süsswassersee; ZSZMH; ♂; in *Stenophylax*]. —Schmid, 1949b:600 [to *Australomyia*]. —Flint, 1967a:57 [♂; distribution]. —Flint, 1974e:88 [checklist]. —Flint, 1982b:8 [larva]. —Angrisano, 1983:325 [larva; pupa]. —Flint et al., 1999a:79 [to *Verger*]. —Cohen, 2004:79 [distribution]. —Brand, 2009:224 [distribution].

—*patagonica* (Ulmer), 1904:9 [Type locality: Süd Patagonien, Agua Fresca, südlich von Punta Arenas; ZSZMH; larva; in *Limnophilus*]. —Flint, 1974e:84, 89 [to *Magellomyia*; unidentified larva]. —Flint, 1982b:8 [to synonymy].

—*setipes* (Ulmer), 1904:10 [Type locality: Süd Patagonien, Agua Fresca, südlich von Punta Arenas; ZSZMH; larva; in *Limnophilus*]. —Flint, 1974e:84, 89 [to *Magellomyia*; unidentified larva]. —Flint, 1982b:8 [to synonymy].

—*meridionalis* (Ulmer), 1905c:18 [Type locality: Argentina, Patagonia, Santa Cruz; MNHNP; ♀; in *Limnophilus*]. —Ulmer, 1906:9 [♂; ♀]. —Schmid, 1949b:600 [to synonymy].

—*stigmata* Navás, 1918a:500 [Type locality: Argentina, Santa Cruz, Valle Túnel; MACN; ♂]. —Schmid, 1955b:54 [to *Magellomyia*, incertae sedis]. —Flint et al., 1999a:80 [to synonymy].

—*chilensis* (Banks), 1920:347 [Type locality: Chile, Corral; MCZ; ♀; in *Algonquina*]. —Schmid, 1955b:54 [to *Magellomyia*, incertae sedis]. —Flint, 1967c:16 [to synonymy].

—*moesta* (Banks), 1920:348 [Type locality: Strait of Magellan, near the Hassler Glacier; MCZ; ♂; in *Magellomyia*]. —Schmid, 1955b:54 [to synonymy].

—*extrema* (Navás), 1932d:85 [Type locality: Tierra del Fuego, Punta Arenas; type depository not given; ♂; in *Limnophilus*]. —Schmid, 1955b:54 [to *Magellomyia*, incertae sedis]. —Flint, 1974e:88 [checklist]. —Flint et al., 1999a:78 [to synonymy].

—*lonquimaya* (Navás), 1932d:86 [Type locality: Chile, Lonquimay; MNHNP; ♀; in *Limnephilus*]. —Schmid, 1955b:54 [to *Magellomyia*, *incertae sedis*]. —Flint, 1974e:89 [checklist]. —Flint et al., 1999a:79 [to synonymy].

**Distribution.** Argentina, Chile.

***armatus*** (Ulmer), 1904:8 [Type locality: Süd-Patagonien, Agua Fresca, südlich von Punta Arenas; ZSZMH; larva; in *Limnophilus*]. —Flint, 1974e:84, 88 [to *Magellomyia*; unidentified larva]. —Flint et al., 1999a:79 [to *Verger*].

**Distribution.** Chile.

***bispinus*** (Schmid), 1957:389 [Type locality: Chile, Arauco, Caramavida; ROM; ♂; in *Magellomyia*]. —Schmid, 1958b:206 [variation; distribution]. —Flint, 1974e:88 [checklist]. —Flint et al., 1999a:79 [to *Verger*]. —Brand, 2009:225 [distribution].

**Distribution.** Argentina, Chile.

***bruchinus*** (Navás), 1918a:501 [Type locality: Argentina, Provincia de Buenos Aires; UNLP; ♂; in *Nostrafilla*]. —Schmid, 1955b:54 [to *Magellomyia*, *incertae sedis*]. —Flint, 1982c:45 [♂]. — Angrisano, 1986:1 [♀; larva]. —Flint et al., 1999a:79 [to *Verger*]. —Cohen, 2004:79 [distribution].

**Distribution.** Argentina.

***capillatus*** (Ulmer), 1906:11 [Type locality: Argentina, Chubut; ZSZMH; ♂; in *Limnophilus*]. —Schmid, 1949b:600 [to *Australomyia*]. Schmid, 1955b:53 [to *Magellomyia*]. —Flint, 1974e:88 [checklist]. —Flint et al., 1999a:79 [to *Verger*].

—*australis* (Banks) 1920:347 [Type locality: Chile; MCZ; ♂; in *Ironoquia*]. —Schmid 1955b:53 [to synonymy]. —Flint, 1967c:16 [♂ lectotype].

**Distribution.** Argentina, Chile.

***curtior*** (Schmid), 1955a:142 [Type locality: Chile, Talca, El Radal; ROM; ♂; in *Magellomyia*]. —Flint, 1974e:88 [checklist]. —Flint et al., 1999a:79 [to *Verger*].

**Distribution.** Chile.

***fuscovittatus*** (Schmid), 1955a:142 [Type locality: Chile, Marga-Marga; ROM; ♂; in *Magellomyia*]. —Flint, 1974e:88 [checklist]. —Flint et al., 1999a:79 [to *Verger*].

**Distribution.** Chile.

***impluviatus*** Blanchard, 1851:141 [Type locality: Chile, Province Valdivia; lost, not in NMHNP; sex unknown; as *Phryganea impluviata*]. —Ulmer, 1913:408 [to *Limnophilus* or *Psilopsyche*]. —Flint, 1974e:88 [unidentified, possibly a Limnephilidae]. —Flint et al., 1999a:78 [identity, to *Verger*, *nomen dubium*].

**Distribution.** Chile.

***kuscheli*** (Schmid), 1955a:138 [Type locality; Chile, “Cord. Chillan”; ROM; ♂; in *Magellomyia*]. —Flint, 1974e:89 [checklist]. —Flint et al., 1999a:79 [to *Verger*].

**Distribution.** Argentina, Chile.

***lutzi*** (Navás), 1918a:499 [Type locality: Argentina, Santa Cruz, Valle Túnel; MACN; ♀; in *Nostrafilla*]. —Schmid, 1955b:54 [to *Magellomyia*, incertae sedis]. —Cohen, 2004:79 [distribution]. —Brand, 2009:225 [distribution].

—*pirioni* (Navás), 1929a:332 [Type locality: Chile, Pailahueque; MZBS; ♀; in *Psilopsyche*]. —Schmid, 1949a:403 [♂; ♀; to *Australomyia*]. —Schmid, 1955b:53 [to *Magellomyia*]. —Flint, 1974e:89 [checklist]. —Flint et al., 1999a:80 [to synonymy].

—*latchani* (Navás), 1935:374 [Type locality: Chile, Aysén; MNHNS, not present, Camousseight, pers. com.; ♀; in *Psilopsyche*]. —Flint et al., 1999a:79 [to synonymy].

**Distribution.** Argentina, Chile.

***masafuera*** (Schmid), 1952:31 [Type locality: Chile, Islas Juan Fernandez, Masafuera, Quebrada de las Casas; UChS; ♂; in *Australomyia*]. —Flint, 1974e:89 [checklist]. —Jacquemart, 1980b:10 [larva]. —Flint et al., 1999a:79 [to *Verger*].

**Distribution.** Chile.

***michaelseni*** (Ulmer), 1904:7 [Type locality: Argentina, Süd- Feuerland, Harberton Harbour (Puerto Bridges); ZSZMH; ♂; in *Limnophilus*]. —Schmid, 1949b:600 [to *Australomyia*]. —Schmid, 1955a:142 [to *Magellomyia*; ♂]. —Flint, 1974e:89 [checklist]. —Flint et al., 1999a:79 [to *Verger*]. —Cohen, 2004:79 [distribution].

**Distribution.** Argentina, Chile.

***modestus*** (Schmid), 1955a:141 [Type locality: Chile, Région de Magellan; ROM; ♂; in *Magellomyia*]. —Flint, 1974e:89 [checklist]. —Flint et al., 1999a:79 [to *Verger*].

**Distribution.** Chile.

***obliquus*** (Schmid), 1955a:145 [Type locality: Chile, Isla de Chiloé, Aucar; ROM; ♂; in *Magellomyia*]. —Flint, 1974e:89 [checklist]. —Flint et al., 1999a:79 [to *Verger*]. —Brand, 2009:225 [distribution].

**Distribution.** Argentina, Chile.

***porteri*** (Navás), 1907:397 [Type locality: Chile, Valparaiso [incorrect]; MZBS; ♀; in *Halesus*]. —Navás, 1918b:362 [to *Verger*]. —Schmid, 1949a:406 [redescripton, ♀]. —Schmid, 1952:29 [distribution]. —Schmid, 1955b:54 [♂; ♀; to *Magellomyia*]. —Flint, 1968d:61 [larva; pupa]. —Flint, 1974e:89 [checklist]. —Jacquemart, 1980b:4 [larva; pupa]. —Flint et al., 1999a:79 [to *Verger*].

—*masatierra* (Schmid), 1952:29 [Type locality: Chile, Islas Juan Fernandez, Masatierra, Plazoleta del Yunque; UChS; ♂; in *Australomyia*]. —Flint, 1968d:61 [to synonymy].

**Distribution.** Chile.

***quadrispinus*** (Schmid), 1955a:137 [Type locality: Chile, Santiago, El Quisco; ROM; ♂; in *Magellomyia*]. —Flint, 1974e:89 [checklist]. —Flint et al., 1999a:79 [to *Verger*].

**Distribution.** Chile.

***spinosus*** (Ulmer), 1904:11 [Type locality: Argentina, Süd- Feuerland, Uschuaia, Süsswassersee auf der Halbinsel; ZSZMH; larva; in *Anabolia*]. —Flint, 1974e:84, 89 [to *Magellomyia*; unidentified larva]. —Flint et al., 1999a:79 [to *Verger*].

**Distribution.** Argentina, Chile.

***stenopterus*** (Schmid), 1955a:143 [Type locality: Argentina, Lago Cardiel; ROM; ♂; in *Magellomyia*]. —Flint, 1974e:89 [checklist]. —Flint, 1990:119 [distribution]. —Flint et al., 1999a:79 [to *Verger*].

**Distribution.** Argentina, Chile.

***vespersus*** (Navás), 1932d:84 [Type locality: Chile, Lonquimay; MNHNP; ♀; in *Limnophilus*]. —Schmid, 1955b:54 [to *Magellomyia*, incertae sedis]. —Flint et al., 1999a:79 [to *Verger*].

—*limnophilus* (Schmid), 1955a:139 [Type locality: Chile, Isla de Chiloé; ROM; ♂; in *Magellomyia*]. —Flint, 1974e:89 [checklist]. —Angrisano, 1983:325 [larva; pupa]. —Flint et al., 1999a:79 [to synonymy].

**Distribution.** Argentina, Chile.

### Family Odontoceridae

This is a small family of approximately 14 genera and about 120 species species. Species occur in all faunal regions except the Afrotropical, but there is no great diversity of species in any region, expect perhaps of *Marilia* in South America. Only three genera occur in the Neotropics, the well-known *Marilia*, with 43 species, and the monotypic genera *Anastomoneura* and *Barypenthus* from Brazil.

Larvae are found in springs and small to medium sized streams. Shallow marginal pools or areas of moderate flow seem to be the preferred microhabitat. A few are associated with waterfalls. Larvae of some genera are known to burrow into sandy substrates. Larvae are omnivorous, feeding on vascular plants, algae, and other arthropods. Cases are constructed of sand grains or other mineral fragments and are heavily reinforced with silken mortar.

#### Genus *Anastomoneura* Huamantinco and Nessimian [1]

*Anastomoneura* Huamantinco and Nessimian, 2004b:282 [Type species: *Anastomoneura guahybae* Huamantinco and Nessimian, 2004b, original designation]. —Dumas and Nessimian, 2006:45 [larva; pupa; case; biology].

A single species, *Anastomoneura guahybae*, was described from material collected at high elevation (1860–1900 m) in the Itatiaia mountain range in the Serra da Mantiqueira, Minas Gerais State, Brazil. The genus can be distinguished from other odontocerine genera by characters of the wing venation and genitalia (Huamantinco and Nessimian 2004b). Immature stages were described by Dumas and Nessimian (2006).

***guahybae*** Huamantinco and Nessimian, 2004b:282 [Type locality: Brazil, Minas Gerais, Itamonte, Rio Aiuruoca, 22°20'9.28"S, 44°41'6.06"W, el. 1860 m; DZRJ; ♂; ♀]. —Dumas and Nessimian, 2006:45 [larva; pupa; case; biology]. —Dumas and Nessimian, 2012:20 [checklist]. —Paprocki and França, 2014:65 [checklist].

**Distribution.** Brazil.

#### Genus *Barypenthus* Burmeister [1]

*Barypenthus* Burmeister, 1839:928 [Type species: *Barypenthus concolor* Burmeister, 1839, subsequent selection of Fischer, 1965]. —Paprocki and Holzenthal, 2002:224 [revision]. —Moretti and Loyola, 2005:337 [larva; biology].

*Musarna* Walker, 1860:178 [Type species: *Musarna aperiens* Walker, 1860 = *Barypenthus concolor* Burmeister 1839, subsequent selection of Fischer, 1965].

*Barypenthus* is another endemic monotypic Neotropical genus from southeastern Brazil. Paprocki and Holzenthal (2002) reviewed the genus and synonymized all previously described species with the type species, *Barypenthus concolor*.

The immature stages were described by Flint (1969a). They construct typical odontocerid cases of sand grains and probably exhibit the typical habitat preferences and mode of living. The larvae of *Barypenthus concolor* do not select specific particle sizes for case building (Moretti and Loyola, 2005).

***concolor*** Burmeister, 1839:929 [Type locality: Brazil, Nova Friburgo; ZIUH; ♂; destroyed]. —Ulmer 1905a:22 [♂]. —Paprocki and Holzenthal, 2002:224 [revision; biology]. —Paprocki et al., 2004:14 [checklist]. —Moretti and Loyola, 2005:337 [larva; biology]. —Dumas et al., 2009:371 [distribution]. —Amato et al., 2011:50 [host of *Temnocephala*, Platyhelminthes]. —Calor, 2011:322 [checklist]. —Dumas and Nessimian, 2012:20 [checklist]. —Paprocki and França, 2014:65 [checklist]. —Quinteiro et al., 2014:236 [distribution].

—*aperiens* (Walker), 1860:178 [Type locality: South America; BMNH; ♀; in *Musarna*]. —Ulmer, 1905a:23 [to synonymy]. —Betten and Mosely, 1940:225 [type is ♀; redescription].

—*chysopus* Navás, 1934a:172 [Type locality: Brazil, Rio de Janeiro, Barão Homem de Mello; DEI; ♂]. —Paprocki and Holzenthal, 2002:225 [to synonymy].

—*claudens* (Walker), 1860:179 [Type locality: Brazil; BMNH; ♂; in *Musarna*]. —Betten and Mosely, 1940:222 [♂; redescription]. —Flint, 1969a:24 [larva; pupa; distribution]. —Paprocki and Holzenthal, 2002:225 [to synonymy].

—*ferrugineus* Navás, 1934a:171 [Type locality: Brazil, Rio de Janeiro, Barao Homem de Mello; type depository not stated, DEI?; ♂]. —Paprocki and Holzenthal, 2002:225 [to synonymy].

—*interclusus* (Walker), 1860:178 [Type locality: Brazil; BMNH; E; in *Musarna*]. —Betten and Mosely, 1940:226 [♀; redescription]. —Paprocki and Holzenthal, 2002:225 [to synonymy].

—*rufipes* Burmeister, 1839:929 [Type locality: Brazil, Nova Friburgo; ZIUH; ♂; destroyed]. —Ulmer, 1905a:20 [♂]. —Paprocki and Holzenthal, 2002:225 [to synonymy].

**Distribution.** Brazil.

#### Genus *Marilia* Müller [43]

*Marilia* Müller, 1880a:127 [Type species: *Marilia major* Müller, 1880a, subsequent selection of Mosely and Kimmins, 1953]. — Bueno-Soria and Rojas-Ascencio, 2004:679 [Mexican and Central American species, revision]. —Camargos and Pes, 2011:354 [larval biology].

Most of the species diversity in *Marilia* occurs in the Neotropics, including the Greater Antilles, but species are also known from North America, the Orient, and Australia. Species appear to be rather local in occurrence, but this may reflect inadequate collecting, rather than a natural phenomenon.

Larvae have been described a number of times (Flint 1968a, Wiggins 1996, Roldán Pérez 1988, Rueda Martín 2008, Camargos and Pes, 2011). They construct tubular cases of sand grains and live in tranquil areas of small creeks and rivers with sand and gravel bottoms. They feed primarily on invertebrates, but also consume algae and vascular plants.

***aiuruoca*** Dumas and Nessimian, 2009b:344 [Type locality: Brazil, Minas Gerais, Itamonte, Rio Aiuruoca, 22°20'56.9"S 44°41'06.6"W, el. 1860 m; DZRJ; ♂; ♀]. —Dumas and Nessimian, 2012:21 [checklist]. —Paprocki and França, 2014:65 [checklist].

**Distribution.** Brazil.

***alata*** Flint, 1974c:143 [Type locality: Suriname, Coeroeni-eiland; RNH; ♂]. —Angrisano and Sganga, 2007:43 [♂; distribution]. —Souza et al., 2013a:7 [distribution]. —Paprocki and França, 2014:65 [checklist].

**Distribution.** Argentina, Brazil, Suriname.

***albicornis*** (Burmeister), 1839:918 [Type locality: Brazil; ZIUH?; ♂; in *Mystacides*]. —Ulmer, 1905a:24 [♂]. —Paprocki et al., 2004:14 [checklist]. —Calor, 2011:322 [checklist]. —Paprocki and França, 2014:65 [checklist].

**Distribution.** Brazil.

***amnicola*** Flint, 1968a:60 [Type locality: Jamaica, St. Andrew, Hardwar Gap, Dicks Pond Trail; NMNH; ♂ (pharate); ♀; larva; pupa]. —Flint, 1968b:83 [checklist]. —Botosaneanu, 2002:98 [checklist].

**Distribution.** Jamaica.

***baumanni*** Bueno-Soria and Rojas-Ascencio, 2004:679 [Type locality: Mexico, Oaxaca, Chiltepec; CNIN; ♂]. —Bueno-Soria and Barba-Álvarez, 2011:359 [checklist].

**Distribution.** Mexico.

***biloba*** Flint, 1974c:141 [Type locality: Suriname, Suriname River, Botopasie; RNH; ♂].

**Distribution.** Suriname.

***cinerea*** Navás, 1931a:323 [Type locality: Argentina, Alta Gracia; type depository not stated; ♂]. —Mangeaud, 1996:154 [distribution]. —Cohen, 2004:79 [distribution]. —Rueda Martín, 2008:13 [♂; larva; pupa; case; biology; distribution]. — Reynaga and Rueda Martín, 2014:545 [biology]. —Isa Miranda and Rueda Martín, 2014:200 [distribution].

**Distribution.** Argentina, Bolivia.

***crea*** Mosely, 1949:40 [Type locality: Costa Rica, San José; BMNH; ♂]. —Holzenthal, 1988c:72 [distribution]. —Bueno-Soria and Rojas-Ascencio, 2004:681 [♂; redescription].

**Distribution.** Costa Rica.

***eleutheria*** Flint, 1983a:80 [Type locality: Argentina, Pcia. Misiones, Río Iguazú, Camp Nañdu; NMNH; ♂].

**Distribution.** Argentina, Paraguay, Uruguay.

***elongata*** Martynov, 1912:14 [Type locality: Peru, 11°8'S, 75°17'W; collection Martynov, now lost?; ♂]. —Flint, 1996b:426 [distribution]. —Blahnik et al., 2004:5 [distribution]. —Paprocki et al., 2004:14 [checklist]. —Rueda Martín, 2008:16 [♂; larva; pupa; case; biology; distribution]. — Reynaga and Rueda Martín, 2014:545 [biology]. —Paprocki and França, 2014:66 [checklist]. —Isa Miranda and Rueda Martín, 2014:200 [distribution].

**Distribution.** Argentina, Brazil, Peru.

***fasiculata*** Banks, 1913b:86 [Type locality: Brazil, Mato Grosso [Rondônia], Madeira Mamoré River; MCZ; ♂]. —Flint, 1967c:19 [♂; as *fasciculata*]. —Maes, 1999:1198 [checklist]. —Paprocki et al., 2004:14 [checklist]. —Chamorro-Lacayo et al., 2007:45 [checklist]. —Souza et al., 2013a:7 [distribution; as *Marilia fasciculata*]. —Paprocki and França, 2014:66 [checklist; as *Marilia fasciculata*].

**Distribution.** Brazil, Nicaragua.

***flexuosa*** Ulmer, 1905b:70 [Type locality: United States, Texas; NMW; ♀]. —Martynov, 1912:17 [♂]. —Ross, 1951a:71 [restricts lectotype locality to Texas; distribution]. —Denning, 1956b:263 [♂]. —Denning, 1964:134 [checklist]. —Flint, 1967d:173 [distribution]. —Bueno-Soria and Flint, 1978:211 [distribution]. —Holzenthal, 1988c:72 [distribution]. —Flint, 1991:100 [♂; distribution]. —Aguila, 1992:543 [distribution]. —Flint, 1996b:426 [distribution]. —Wiggins, 1996:362 [larva]. —Muñoz-Quesada, 2000:280 [checklist]. —Bueno-Soria and Rojas-Ascencio, 2004:681 [male; distribution]. —Paprocki et al., 2004:14 [checklist]. —Baumgardner and Bowles, 2005:11 [distribution]. —Blinn and Ruiter, 2006:334 [biology]. —Bowles et al., 2007:24 [distribution; biology]. —Bueno-Soria et al., 2007:33 [distribution]. —Chamorro-Lacayo et al., 2007:45 [checklist]. —Rueda Martín, 2008:19 [distribution]. —Blinn and Ruiter, 2009a:307 [biology]. —Blinn and Ruiter, 2009b:186 [phenology, distribution]. —Ruiter and Blinn, 2009:5 [checklist]. —Bueno-Soria and Barba-Álvarez, 2011:359 [checklist]. —Djernaes, 2011:42 [♂; ♀]. —Reynaga and Rueda Martín, 2014:545 [biology]. —Paprocki and França, 2014:66 [checklist]. —Isa Miranda and Rueda Martín, 2014:200 [distribution]. —Armitage et al., 2015b:9 [checklist]. —Armitage and Cornejo, 2015:198 [checklist].

—*fusca* (Banks) 1905:19 [Type locality: United States, Arizona, Phoenix; MCZ; sex not stated; in *Anisocentropus*]. —Betten, 1934:242 [to synonymy].

**Distribution.** Argentina, Brazil, Canada, Colombia, Costa Rica, Guatemala, Mexico, Nicaragua, Panama, Peru, U.S.A.

***flinti*** Bueno-Soria and Rojas-Ascencio, 2004:684 [Type locality: Mexico, San Luis Potosi, 25 mi. N Tamazunchale, 400’; NMNH; ♂].

**Distribution.** Mexico.

***furthi*** Bueno-Soria and Rojas-Ascencio, 2004:684 [Type locality: Mexico, Tabasco, Municipio Huimanguillo, Carlos A Madrazo, 17°23.759'N, 93°39.757'W, Rio Pueblo Viejo; CNIN; ♂].

**Distribution.** Mexico.

***gigas*** Flint, 1991:102 [Type locality: Ecuador, Pcia. Pastaza, 3 km N Puyo; NMNH; ♂]. —Muñoz-Quesada, 2000:280 [checklist].

**Distribution.** Colombia, Ecuador.

***gracilis*** Banks, 1938:297 [Type locality: Haiti, La Visite, La Selle Range; MCZ; ♂]. —Flint, 1967c:19 [♂; lectotype]. —Flint, 1968b:83 [checklist]. —Flint and Pérez-Gelabert, 1999:42 [checklist]. —Botosaneanu, 2002:98 [checklist]. —Flint and Sykora, 2004:49 [distribution]. —Pérez-Gelabert, 2008:301 [checklist].

**Distribution.** Haiti.

***guaira*** Flint, 1983a:82 [Type locality: Paraguay, Dpto. Alto Paraná, Salto de Guaira; NMNH; ♂]. —Flint, 1992d:81 [distribution]. —Muñoz-Quesada, 2000:280 [checklist]. —Paprocki et al., 2004:14 [checklist]. —Paprocki and França, 2014:66 [checklist].

**Distribution.** Bolivia, Brazil, Colombia, Paraguay, Venezuela.

***holzenthali*** Bueno-Soria and Rojas-Ascencio, 2004:686 [Type locality: Belize, Rio Privassion, Blancaneaux Lodge; NMNH; ♂].

**Distribution.** Belize.

***huamantincoae*** Dumas and Nessimian, 2009b:345 [Type locality: Brazil, Rio de Janeiro, Itatiaia, Maromba, Escorrega do Maromba, Rio Preto, 22°19'48.81"S 44°36'53.94"W, el. 1357 m; DZRJ; ♂; ♀]. —Dumas and Nessimian, 2012:21 [checklist]. —Paprocki and França, 2014:66 [checklist].

**Distribution.** Brazil.

***humerosa*** Flint, 1983a:85 [Type locality: Argentina, Pcia. Misiones, Río Iguazú, Camp Nañdu; NMNH; ♂].

**Distribution.** Argentina.

***infundibulum*** Flint, 1983a:82 [Type locality: Brazil, Edo. Santa Catarina, Nova Teutonia (27°11'S, 52°23'W); NMNH; ♂]. —Paprocki et al., 2004:14 [checklist]. —Paprocki and França, 2014:66 [checklist].

**Distribution.** Argentina, Brazil.

***kingsolveri*** Bueno-Soria and Rojas-Ascencio, 2004:688 [Type locality: Costa Rica, Puntarenas, Rio Bellavista, ca. 1.5 km NW Las Alturas, 8.951°N, 82.846°W, el. 1400m; UMSP; ♂].

**Distribution.** Costa Rica.

***lateralis*** Flint, 1983a:85 [Type locality: Paraguay, Dpto. San Pedro, Arroyo Tapiracuay, San Estanislao; NMNH; ♂]. —Muñoz-Quesada, 2000:280 [checklist]. —Paprocki et al., 2004:14 [checklist]. —Paprocki and França, 2014:66 [checklist].

**Distribution.** Brazil, Colombia, Paraguay, Uruguay.

***major*** Müller, 1880a:127 [Type locality: Brazil, Santa Catarina; MNRJ; case]. —Ulmer, 1905a:25 [♂]. —Blahnik et al., 2004:5 [distribution]. —Paprocki et al., 2004:14 [checklist]. —Dumas and Nessimian, 2009:347 [♀]. —Dumas and Nessimian, 2012:21 [checklist]. —Paprocki and França, 2014:66 [checklist].

**Distribution.** Brazil.

***mathisi*** Bueno-Soria and Rojas-Ascencio, 2004:689 [Type locality: Mexico, Durango, 5 mi. SW El Salto; NMNH; ♂].

**Distribution.** Costa Rica.

***mexicana*** (Banks), 1901:368 [Type locality: Mexico, Morelos, Cuernavaca; MCZ; ♀; in *Leptocerus*]. —Fischer, 1965:211 [catalog; in *Athripsodes*]. —Flint, 1967c:20 [♀; to *Marilia*]. —Bueno-Soria and Flint, 1978:211 [distribution]. —Bueno-Soria and Rojas-Ascencio, 2004:681 [checklist].

**Distribution.** Mexico.

***microps*** Flint, 1991:102 [Type locality: Colombia, Dpto. Chocó, km 114 [road to Quibdù], 6 km E El Siete; NMNH; ♂]. —Muñoz-Quesada, 2000:280 [checklist].

**Distribution.** Colombia.

***minor*** Müller, 1880a:127 [Type locality: Brazil, Santa Catarina; MNRJ; case]. —Ulmer, 1907a:9 [♂; distribution]. —Blahnik et al., 2004:5 [distribution]. —Paprocki et al., 2004:14 [checklist]. —Dumas et al., 2009:371 [distribution]. —Paprocki and França, 2014:67 [checklist].

**Distribution.** Brazil.

***misionensis*** Flint, 1983a:83 [Type locality: Argentina, Pcia. Misiones, Arroyo Piray Mini, Rt. 17 W Dos Hermanas; NMNH; ♂]. —Manzo et al., 2014:167 [distribution].

**Distribution.** Argentina.

***modesta*** Banks, 1913a:235 [Type locality: [E.] Colombia, Villavicencio; MCZ; ♂]. —Flint, 1967c:20 [♂]. —Muñoz-Quesada, 2000:280 [checklist].

**Distribution.** Colombia.

***morsei*** Bueno-Soria and Rojas-Ascencio, 2004:689 [Type locality: Guatemala, Dpto. El Progreso, Finca La Cajeta; NMNH; ♂].

**Distribution.** Guatemala.

***nigrescens*** Banks, 1941:397 [Type locality: Dominican Republic, Santo Domingo, Valle Nuevo, southeast of Constanza; MCZ; ♂]. —Flint, 1967c:20 [♂; lectotype]. —Flint et al., 1999b:146 [as subspecies of *Marilia gracilis*]. —Flint and Pérez-Gelabert, 1999:42 [checklist, as subspecies of *Marilia gracilis*]. —Botosaneanu, 2002:98 [checklist; as *Marilia gracilis* “var.” *nigrescens*]. —Flint and Sykora, 2004:49 [distribution; as species, new status]. —Pérez-Gelabert, 2008:301 [checklist].

**Distribution.** Dominican Republic.

***nobsca*** Milne, 1936:79 [Type locality: United States, Texas, Jeff Davis Co., Ft. Davis, Davis Mts.; MCZ; ♀]. —Ross, 1951a:71 [distribution]. —Denning, 1964:134 [checklist]. —Bueno-Soria and Flint, 1978:211 [distribution]. —Bueno-Soria and Rojas-Ascencio, 2004:681 [♂; distribution]. —Baumgardner and Bowles, 2005:11 [distribution]. —Blinn and Ruiter, 2006:334 [biology]. —Blinn and Ruiter, 2009b:186 [phenology, distribution]. —Ruiter and Blinn, 2009:5 [♀]. —Bueno-Soria and Barba-Álvarez, 2011:359 [checklist].

**Distribution.** Guatemala, Mexico; U.S.A.

***salta*** Flint, 1983a:85 [Type locality: Argentina, Pcia. Entre Ríos, Río Uruguay, Salto Grande; NMNH; ♂].

**Distribution.** Argentina.

***scudderi*** Banks, 1924:446 [Type locality: Cuba, Isle of Pines; MCZ; ♂]. —Flint 1967c:20 [♂]. —Flint, 1968b:83 [checklist]. —Botosaneanu, 1979:52 [distribution]. —Botosaneanu, 1994b:466 [larva]. —Flint, 1996c:16 [checklist]. —Botosaneanu, 2002:98 [checklist]. —López del Castillo et al., 2004:229 [distribution]. —Naranjo López and González Lazo, 2005:150 [checklist]. — López del Castillo et al., 2007:172 [checklist].

**Distribution.** Cuba.

***sioli*** Marlier, 1964b:97 [Type locality: Brazil, Amazon region; IRSNB; ♂]. —Paprocki et al., 2004:14 [checklist]. —Paprocki and França, 2014:67 [checklist].

**Distribution.** Brazil.

***spangleri*** Bueno-Soria and Rojas-Ascencio, 2004:693 [Type locality: Mexico, Oaxaca, Sierra de Juarez, Cerro Pelon, km 110 carr. Tuxtepec-Oaxaca; CNIN; ♂]. —Bueno-Soria and Barba-Álvarez, 2011:359 [checklist].

**Distribution.** Guatemala.

***spinosula*** Flint, 1996a:105 [Type locality: Trinidad, Tacariqua River, Caura Recreation Area, 10°43'N, 61°17'W; NMNH; ♂]. —Botosaneanu, 2002:98 [checklist].

**Distribution.** Trinidad.

***triangularis*** Flint, 1983a:82 [Type locality: Paraguay, Dpto. Amambay, 2 km S Cerro Corá; NMNH; ♂].

**Distribution.** Paraguay.

***truncata*** Flint, 1983a:80 [Type locality: Paraguay, Dpto. Amambay, Río Aquidabán, Cerro Corá; NMNH; ♂]. —Blahnik et al., 2004:5 [distribution]. —Paprocki et al., 2004:14 [checklist]. —Paprocki and França, 2014:67 [checklist].

**Distribution.** Brazil, Paraguay.

***valga*** Flint and Sykora, 2004:49 [Type locality: Dominican Republic, Peravia Province, 3 km SW La Nuez, upper Río Las Cuevas, 18°40'N, 70°36'W, el. 1850 m; CMNH; ♂; ♀]. —Pérez-Gelabert, 2008:301 [checklist].

**Distribution.** Dominican Republic.

***williammerrilli*** Bueno-Soria and Rojas-Ascencio, 2004:693 [Type locality: Costa Rica, Alajuela, Reserva forestal San Ramon, Rio San Josecilo y tributarios, 10.216°N. 84.607°W, el. l. 980 m; UMSP; ♂].

**Distribution.** Costa Rica.

***wrighti*** Banks, 1924:446 [Type locality: Cuba; MCZ; ♂]. —Flint, 1967c:20 [♂; lectotype]. —Flint, 1968b:83 [checklist]. —Botosaneanu, 1979:52 [distribution]. —Flint, 1996c:16 [checklist]. —Botosaneanu, 2002:98 [checklist]. —Naranjo López and González Lazo, 2005:150 [checklist].

**Distribution.** Cuba.

### Family Philopotamidae

This cosmopolitan family is rich in species, especially in tropical regions, where many new species have been described. The generic classification has undergone several reassessments such that 19 are now recognized, in three subfamilies, Chimarrinae, Philopotaminae, and Rossodinae (Blahnik 2005). The genus *Chimarra*, found in all faunal regions, is the largest in all of Trichoptera with almost 800 described species (Kjer et al. 2014).

Larvae of a number of genera are well known from many parts of the world (e.g., Wiggins 2004). They construct long, tubular nets of fine silken mesh, usually attached to the undersurface of objects on the substrate.

#### Genus *Alterosa* Blahnik [39]

*Alterosa* Blahnik, 2005:10 [Type species: *Alterosa bocainae* Blahnik, 2005, original designation]. —Dumas et al., 2013:10 [key to species]. —Dumas and Nessimian, 2013:26 [review].

This genus of 39 species represents a remarkable radiation of diversity in the mountains of southeastern and southern Brazil. Each small mountain valley seems to hold an endemic species. Blahnik (2005) established the genus for two species formerly placed in *Dolophilodes* subgenus *Sortosa*. The larval stages are not described.

***affinis*** Dumas and Nessimian, 2013:27 [Type locality: Brazil, Minas Gerais, Alto Caparaó, Parque Nacional do Caparaó, Rio Caparaó, Vale Verde, 20°25'11.6"S, 41°50'44.8"W, el. 1306 m; DZRJ; ♂]. —Paprocki and França, 2014:67 [checklist].

**Distribution.** Brazil.

***amadoi*** Dumas, Calor and Nessimian, 2013:3 [Type locality: Brazil, Bahia, Camacan, RPPN Serra Bonita, riacho 1, trilha nova, 15°23'35"S, 39°33'50"W, ca 770 m; MZUSP; ♂]. —Paprocki and França, 2014:67 [checklist].

**Distribution.** Brazil.

***bandeira*** Dumas and Nessimian, 2013:29 [Type locality: Brazil, Minas Gerais, Alto Caparaó, Parque Nacional do Caparaó, Rio Caparaó, Vale Verde, 20°25'11.6"S, 41°50'44.8"W, el. 1306 m; DZRJ; ♂]. —Paprocki and França, 2014:67 [checklist].

**Distribution.** Brazil.

***beckeri*** Blahnik, 2005:14 [Type locality: Brazil, Rio de Janeiro, Itatiaia, 2100 m; MZUSP; ♂]. —Dumas et al., 2009:361 [distribution]. —Dumas and Nessimian, 2012:21 [distribution]. —Paprocki and França, 2014:68 [checklist].

**Distribution.** Brazil.

***bilanceolata*** Dumas and Nessimian, 2013:31 [Type locality: Brazil, Paraná, Guaraqueçaba, Ribeirão do Engenho, 25°10'31.0"S, 48°22'16.2"W, el. 25 m; DZRJ; ♂]. —Paprocki and França, 2014:68 [checklist].

**Distribution.** Brazil.

***bocainae*** Blahnik, 2005:15 [Type locality: Brazil, São Paulo, Lajeado, Serra da Bocaina, Cachoeira do Lajeado, 22°43.208'S, 44°37.782'W, el 1590 m; MZUSP; ♂; ♀]. —Calor, 2011:322 [checklist]. —Paprocki and França, 2014:68 [checklist].

**Distribution.** Brazil.

***boraceiae*** Blahnik, 2005:17 [Type locality: Brazil, São Paulo, Estação Biológica Boraceia, Rio Venerando & tribs., 23°39.185'S, 45°53.414'W, el 850 m; MZUSP; ♂]. —Calor, 2011:322 [checklist]. —Paprocki and França, 2014:68 [checklist].

**Distribution.** Brazil.

***caissara*** Dumas and Nessimian, 2013:31 [Type locality: Brazil, São Paulo, Ubatuba, Parque Estadual da Serra do Mar, Núcleo Picinguaba, Km 2 of BR 101 road, small tributary, near Cachoeira da Escada, 23°21'14.5"S, 44°46'03.8"W, el. 296 m; DZRJ; ♂]. —Paprocki and França, 2014:68 [checklist].

**Distribution.** Brazil.

***caparaonensis*** Blahnik, 2005:19 [Type locality: Brazil, Minas Gerais, Parque Nacional do Caparaó, small trib. to Rio Caparaó, Vale Verde, 20°25.029'S, 41°50.767'W, el 1350 m; MZUSP; ♂]. —Dumas and Nessimian, 2013:47 [distribution]. —Paprocki and França, 2014:68 [checklist].

**Distribution.** Brazil.

***capixaba*** Dumas and Nessimian, 2013:34 [Type locality: Brazil, Espírito Santo, Santa Teresa, Estação Biológica de Santa Lúcia, Córrego Bonito, above Cachoeira Heloísa Torres, 19°58'25.9"S, 40°31'46.3"W, el. 685 m; DZRJ; ♂]. —Paprocki and França, 2014:68 [checklist].

**Distribution.** Brazil.

***castroalvesi*** Dumas, Calor and Nessimian, 2013:5 [Type locality: Brazil, Bahia, Camacan, Serra Bonita, Córrego das Torres, 15°23'01"S, 39°34'19"W, ca 860 m; MZUSP; ♂]. —Paprocki and França, 2014:68 [checklist].

**Distribution.** Brazil.

***catarinae*** Dumas and Nessimian, 2013:36 [Type locality: Brazil, Santa Catarina, Blumenau, Parque Ecológico Spitzkopf, Ribeirão do Caeté, 27°00'22.6"S, 49°06'50.4"W, el. 130 m; DZRJ; ♂]. —Paprocki and França, 2014:69 [checklist].

**Distribution.** Brazil.

***caymmii*** Dumas, Calor and Nessimian, 2013:7 [Type locality: Brazil, Bahia, Varzedo, Serra da Jibóia, Reserva Jequitibá, 12°52'21.5"S, 39°28'56.5"W, ca 400 m; MZUSP; ♂]. —Costa et al., 2014:222 [distribution]. —Paprocki and França, 2014:69 [checklist].

**Distribution.** Brazil.

***escova*** Blahnik, 2005:21 [Type locality: Brazil, São Paulo, small stream on São Paulo Route 247, 11 km SE Bananal, 22°45.684'S, 44°23.190'W, el 675 m; MZUSP; ♂]. —Dumas et al., 2009:361 [distribution]. —Calor, 2011:322 [checklist]. —Dumas and Nessimian, 2012:21 [distribution]. —Paprocki and França, 2014:69 [checklist].

**Distribution.** Brazil.

***falcata*** Blahnik, 2005:22 [Type locality: Brazil, Minas Gerais, Ibitipoca, sitio of Anestis Papadopoulos, cachoeira, 21°43.441'S, 43°54.537'W, el 1125 m; MZUSP; ♂]. —Dumas et al., 2009:361 [distribution]. —Calor, 2011:322 [checklist]. —Dumas and Nessimian, 2012:21 [distribution]. —Paprocki and França, 2014:69 [checklist].

**Distribution.** Brazil.

***fimbriata*** Blahnik, 2005:25 [Type locality: Brazil, Rio de Janeiro, Parque Nacional da Serra dos Órgãos, Rio Paquequer, 22°26.992'S, 42°59.899'W, el 1000 m; MZUSP; ♂]. —Paprocki and França, 2014:69 [checklist].

**Distribution.** Brazil.

***flinti*** Blahnik, 2005:26 [Type locality: Brazil, Espirito Santo, 24 km SE Santa Teresa, el 280 m; MZUSP; ♂]. —Dumas et al., 2009:361 [distribution]. —Dumas and Nessimian, 2012:21 [distribution]. —Dumas and Nessimian, 2013:47 [distribution]. —Paprocki and França, 2014:69 [checklist].

**Distribution.** Brazil.

***fluminensis*** Blahnik, 2005:28 [Type locality: Brazil, Rio de Janeiro, Rio Sousa in Cachoeiras de Macacú, 26°26.567'S, 42°37.957'W, el 150 m: MZUSP; ♂]. —Dumas et al., 2009:361 [distribution]. —Paprocki and França, 2014:69 [checklist].

**Distribution.** Brazil.

***graciosa*** Dumas and Nessimian, 2013:36 [Type locality: Brazil, Paraná, Morretes, Serra da Graciosa, Rio Grota Funda, 25°20'26.1"S, 48°53'41.2"W, el. 657 m; DZRJ; ♂]. —Paprocki and França, 2014:70 [checklist].

**Distribution.** Brazil.

***guapimirim*** Blahnik, 2005:30 [Type locality: Brazil, Rio de Janeiro, Parque Nacional da Serra dos Orgãos, Guapimirim, Trilha das Ruinas, 22°29.679'S, 42°59.729'W, el 940 m; MZUSP; ♂]. —Dumas et al., 2009:362 [distribution]. —Paprocki and França, 2014:70 [checklist].

**Distribution.** Brazil.

***holzenthali*** Blahnik, 2005:32 [Type locality: Brazil, Santa Catarina, Parque Ecológica Spitzkopf, confluence Rio Ouro & Rio Caeté, 27°00.352'S, 49°06.693'W, el 140 m; MZUSP; ♂]. —Paprocki and França, 2014:70 [checklist].

**Distribution.** Brazil.

***inappendiculata*** Dumas and Nessimian, 2013:38 [Type locality: Brazil, Paraná, Guaraqueçaba, Reserva Natural de Salto Morato, small tributary of Rio Morato, 25°09'53.9"S, 48°17'54.3"W, el. 55 m; DZRJ; ♂]. —Paprocki and França, 2014:70 [checklist].

**Distribution.** Brazil.

***intervales*** Blahnik, 2005:33 [Type locality: Brazil, São Paulo, Parque Estadual Intervales, small trib. to Agua Comprida, 24°16.531'S, 48°25.042'W, el 800 m; MZUSP; ♂]. —Calor, 2011:322 [checklist]. —Dumas and Nessimian, 2013:47 [distribution]. —Paprocki and França, 2014:70 [checklist].

**Distribution.** Brazil.

***itatiaiae*** Blahnik, 2005:35 [Type locality: Brazil, Rio de Janeiro, Parque Nacional Itatiaia, Rio Campo Belo, trail to Veu da Noiva, 22°25.706'S, 44°37.171'W, el 1310 m; MZUSP; ♂]. —Dumas and Nessimian, 2012:21 [distribution]. —Paprocki and França, 2014:70 [checklist].

**Distribution.** Brazil.

***jordaensis*** Blahnik, 2005:37 [Type locality: Brazil, São Paulo, Parque Estadual de Campos do Jordão, Rio Galharada, 22°41.662'S, 45°27.783'W, el 1530 m; MZUSP; ♂]. —Calor, 2011:322 [checklist]. —Paprocki and França, 2014:70 [checklist].

**Distribution.** Brazil.

***marinonii*** (Almeida and Duarte), 2003:967 [Type locality: Brazil, Paraná, Antonina, Reserva de Sapitanduva, 25°28'S, 48°50'W, el 60 m; DZUP; ♂; in Dolophilodes (Sortosa)]. —Blahnik, 2005:39 [♂; redescription; to *Alterosa*]. —Dumas and Nessimian, 2013:47 [distribution]. —Paprocki and França, 2014:70 [checklist].

**Distribution.** Brazil.

***morato*** Dumas and Nessimian, 2013:38 [Type locality: Brazil, Paraná, Guaraqueçaba, Reserva Natural de Salto Morato, tributary of Córrego Morato, 25°09'53.9"S, 48°17'54.3"W, el. 55 m; DZRJ; ♂]. —Paprocki and França, 2014:71 [checklist].

**Distribution.** Brazil.

***nessimiani*** Jardim and Dumas, 2012:578 [Type locality: Brazil, Rio de Janeiro, Nova Friburgo, Cascata, Rio Macaé, Cachoeira da Fumaça, 22°21'56.1” S - 42°15'13.1” W, 368 m; DZRJ; ♂]. —Paprocki and França, 2014:71 [checklist].

**Distribution.** Brazil.

***orgaosensis*** Blahnik, 2005:41 [Type locality: Brazil, Rio de Janeiro, Parque Nacional da Serra dos Orgãos, Rio Beija-flor, 22°27.063'S, 43°00.065'W, el 1125 m; MZUSP; ♂]. —Dumas et al., 2009:362 [distribution]. —Paprocki and França, 2014:71 [checklist].

**Distribution.** Brazil.

***paprockii*** Blahnik, 2005:42 [Type locality: Brazil, Minas Gerais, Cachoeira do Abacaxi, Vale do Tropeiro, 20°12.270'S, 43°38.163'W, el 1120 m; MZUSP; ♂]. —Paprocki and França, 2014:71 [checklist].

**Distribution.** Brazil.

***paranaensis*** Dumas and Nessimian, 2013:42 [Type locality: Brazil, Paraná, Guaratuba, Castelhanos road, small tributary on main road, 25°49'56.2"S, 48°55'45.1"W, el. 594 m; DZRJ; ♂]. —Paprocki and França, 2014:71 [checklist].

**Distribution.** Brazil.

***polyacinata*** Barcelos-Silva, Dumas and Pes, 2015:596 [Type locality: Brazil, Espírito Santo, Fundão, Hotel Fazenda Monte Sião, 19°56'02.0"S 40°24'45.0"W; DZRJ; ♂].

**Distribution.** Brazil.

***ruschii*** Dumas and Nessimian, 2013:42 [Type locality: Brazil, Espírito Santo, Santa Teresa, Estação Biológica de Santa Lúcia, Córrego Bonito, above Cachoeira Heloísa Torres, 19°58'25.9"S, 40°31'46.3"W, el. 685 m; DZRJ; ♂]. —Paprocki and França, 2014:71 [checklist]. —Paprocki and França, 2014:71 [checklist].

**Distribution.** Brazil.

***sanctaeteresae*** Blahnik, 2005:44 [Type locality: Brazil, Espirito Santo, 24 km SE of Santa Teresa, el 280 m; MZUSP; ♂]. —Paprocki and França, 2014:71 [checklist].

**Distribution.** Brazil.

***sanctipauli*** (Flint), 1971c:20 [Type locality: Brazil, São Paulo; NMNH; ♂; in Dolophilodes (Sortosa)]. —Paprocki et al., 2004:15 [checklist]. —Blahnik, 2005:46 [♂; redescription; to *Alterosa*]. —Calor, 2011:322 [checklist]. —Paprocki and França, 2014:71 [checklist].

**Distribution.** Brazil.

***schadrackorum*** Blahnik, 2005:48 [Type locality: Brazil, Santa Catarina, Parque Ecológica Spitzkopf, Rio Caeté above 1st falls, 27°00.35'S, 49°06.70'W, el 170 m; MZUSP; ♂]. —Paprocki and França, 2014:72 [checklist].

**Distribution.** Brazil.

***spiesae*** Dumas and Nessimian, 2013:45 [Type locality: Brazil, São Paulo, Campos do Jordão, Parque Estadual de Campos do Jordão, tributary of Córrego Serrote, 22°39'25.0"S, 45°26'34.0"W, el. 1567 m; MZUSP; ♂]. —Paprocki and França, 2014:72 [checklist].

**Distribution.** Brazil.

***tripuiensis*** Blahnik, 2005:50 [Type locality: Brazil, Minas Gerais, Estação Ecológica do Tripuí, CórregoTripuí, 20°23.364'S, 43°32.541'W, el 1070 m; MZUSP; ♂]. —Paprocki and França, 2014:72 [checklist].

**Distribution.** Brazil.

***truncata*** Blahnik, 2005:51 [Type locality: Brazil, Minas Gerais, Estação Ecológica de Peti, Córrego Brucutu, 19°52.995'S, 43°22.452'W; MZUSP; ♂]. —Dumas et al., 2009:362 [distribution]. —Calor, 2011:322 [checklist]. —Dumas and Nessimian, 2012:22 [distribution]. —Barcelos-Silva et al., 2012:1278 [distribution]. —Paprocki and França, 2014:72 [checklist].

**Distribution.** Brazil.

#### Genus *Chimarra* Stephens [256 + †6]

*Chimarra* Stephens, 1829:318 [Type species: *Phryganea marginata* Linnaeus, 1767, by monotypy]. —*Chimarrha* Burmeister, 1839:910 [invalid subsequent emendation]. —Blahnik, 1998:1 [revision of New World species]. —Wichard, 2007a:11 [diagnosis]. —Wahlberg and Johanson, 2014:4 [phylogeny]. —Kjer et al., 2014:348 [phylogeny].

*Curgia* Walker, 1860:179 [Type species: *Curgia braconoides* Walker, 1860, by monotypy]. —Milne, 1936:81 [to subgenus of *Chimarrha*]. —Flint, 1998:1 [revision].

*Chimarrhodes* Müller, 1887a:290 [Type species: *Chimarrha morio* Burmeister, 1839, subsequent designation of Fischer, 1961]. —Fischer, 1961:53 [as synonym of *Chimarra*].

*Chimarrita* Blahnik, 1997:201 [Type species: *Chimarra simpliciforma* Flint, 1971, original designation; as subgenus of *Chimarra*; revision]. —Wichard, 2007a:13 [diagnosis]. —Vilarino and Calor, 2015b:119 [Brazilian species, key, phylogeny].

*Otarrha* Blahnik, 2002:68 [Type species: *Chimarra patosa* Ross, 1956, original designation; as subgenus of *Chimarra*; revision; phylogeny].

Four subgenera are known from the Netropics, *Chimarra*, *Curgia*, *Chimarrita*, and *Otarrha*, the latter three being endemic to the region, although *Curgia* has species that extend into the southwestern United States. The phylogenetic relationships among its species have been investigated by Kjer et al. (2014) and Wahlberg and Johanson (2014).

The larval stage has been described for species from most regions of the world (Flint 1964a, Marlier 1943, Ross 1944, Wiggins 1996). In the Neotropics, the described larvae are almost all from the Caribbian islands (e.g., Botosaneanu 1989b, Flint 1964a, 1968a, 1968b); few of the mainland larvae have been associated. They construct tubular, silken retreats attached beneath or between rocks or other large substrate in streams and rivers. Some have been found attached directly to vertical rock faces where the net is kept distended by the thin sheet of water flowing through it (Sattler 1962).

***acinaciformis*** (*Curgia*) Flint, 1998:62 [Type locality: Ecuador, Pcia. Pastaza, Estación Fluviometrica, Puyo (27 km N); NMNH; ♂; in *banksi* group].

**Distribution.** Ecuador.

***acula*** (*Curgia*) Flint, 1998:28 [Type locality: Peru, Dept. Cusco, Pcia. Paucartambo, Puente San Pedro, km 152 (13°03.3'S, 71°32.8'W), 44 km NW Pilcopata; NMNH; ♂; in *margaritae* group].

**Distribution.** Peru.

***acuta*** (*Chimarra*) Ross, 1959:171 [Type locality: Mexico, Mor[elos], Cuernavaca; INHS; ♂]. —Bueno-Soria and Flint, 1978:195 [distribution]. —Blahnik, 1998:39 [♂; ♀; to *beameri* group, synonymy, distribution]. —Rojas-Ascencio, et al., 2002:377 [distribution]. —Bueno-Soria et al., 2007:33 [distribution]. —Maes, 1999:1190 [checklist, as *boneti*]. —Chamorro-Lacayo et al., 2007:45 [checklist]. —Bueno-Soria and Barba-Álvarez, 2011:359 [checklist].

—*boneti* (*Chimarra*) Ross, 1959:171 [Type locality: Mexico, Chis.[Chiapas], Ocosingo Valley, Finca El Real, Rio Sta. Cruz; INHS; ♂]. —Maes and Flint, 1988:2 [distribution]. —Blahnik, 1998:39 [to synonymy].

**Distribution.** Guatemala, Honduras, Mexico, Nicaragua.

***adamsae*** (*Chimarra*) Blahnik, 1998:106 [Type locality: Peru, Madre de Dios, Manu, Pakitza, 12°7'S, 70°58'W; MHNJP; ♂; ♀; in *poolei* group]. —Blahnik et al., 2004:5 [distribution]. —Paprocki et al., 2004:14 [checklist]. —Calor, 2011:323 [checklist]. —Paprocki and França, 2014:72 [checklist].

**Distribution.** Brazil, Peru.

***adella*** (*Chimarra*) Denning, 1952:17 [Type locality: United States, Arizona, Cochise Co., Chiricahua Mts.; SEMC; ♂]. —Blahnik, 1998:127 [♂; ♀; to *utahensis* group, distribution]. —Baumgardner and Bowles, 2005:11 [distribution]. —Bueno-Soria et al., 2007:33 [distribution]. —Ruiter and Blinn, 2009:5 [♀].

**Distribution.** Mexico, U.S.A.

***adelphe*** (*Chimarra*) Blahnik, 1998:53 [Type locality: Costa Rica, Puntarenas, Río Jaba at rock quarry, 1.4 km (air) W Las Cruces, 8.79°N, 82.97°W; NMNH; ♂; ♀; in *bicolor* group]. —Chamorro-Lacayo et al., 2007:45 [checklist]. —Armitage et al., 2015b:3 [checklist]. —Armitage and Cornejo, 2015:191 [checklist].

**Distribution.** Costa Rica, Nicaragua, Panama.

***akantha*** (*Chimarrita*) Blahnik, 1997:219 [Type locality: Brazil, Amazonas, Res. Ducke, 26 km E Manaus; MZUSP; ♂; ♀; in *simpliciforma* group]. —Paprocki et al., 2004:14 [checklist]. —Paprocki and França, 2014:73 [checklist].

**Distribution.** Brazil.

***alata*** (*Chimarra*) Bueno-Soria, 1983b:451 [Type locality: Mexico, Chiapas, Bonampak; IBUNAM; ♂]. —Maes and Flint, 1988:2 [distribution]. —Holzenthal, 1988c:55 [distribution]. —Blahnik, 1998:70 [♂; ♀; to *dentosa* group]. —Maes, 1999:1190 [checklist]. —Chamorro-Lacayo et al., 2007:45 [checklist]. —Bueno-Soria and Barba-Álvarez, 2011:359 [checklist].

**Distribution.** Costa Rica, Mexico, Nicaragua.

***albomaculata*** (*Curgia*) Kolbe, 1888:175 [Type locality: Puerto Rico; ZMHU; sex unknown; in *Chimarrha*]. —Palmer, 1938:69 [♂; ♀; larva; pupa; biology]. —Flint, 1964a:21 [♂; ♀; larva; pupa; distribution; synonymy]. —Flint, 1968b:80 [checklist]. —Flint, 1998:55 [♂; redescription; to *braconoides* group]. —Botosaneanu, 2002:90 [checklist].

—*luquillo* Denning, 1947a:657 [Type locality: Puerto Rico, Luquillo; CAS; ♂]. —Flint, 1964a:21 [to synonymy].

**Distribution.** Puerto Rico.

***altmani*** (*Chimarra*) Blahnik, 1998:92 [Type locality: Panama, San Blas, Río Carti Grande, 2 km W Nusagandi; NMNH; ♂; ♀; in *picea* group]. —Armitage et al., 2015b:3 [checklist]. —Armitage and Cornejo, 2015:191 [checklist].

**Distribution.** Panama.

***amazonia*** (*Otarrha*) Blahnik, 2002:71 [Type locality: Peru, Madre de Dios, Amazonia Lodge, Toma del Agua (stream), 12°52.22'S, 71°22.56'W, el. 415 m; JMP; ♂; ♀].

**Distribution.** Peru.

***amica*** (*Chimarra*) Blahnik and Holzenthal, 1992b:412 [Type locality: Costa Rica, Guanacaste, Estación Maritza, Río Tempisquito, 10.958°N, 85.497°W; NMNH; ♂]. —Blahnik, 1998:30 [♂; to *amica* group]. —Chamorro-Lacayo et al., 2007:45 [checklist].

**Distribution.** Costa Rica, Nicaragua.

***angularis*** (*incertae sedis*) Blahnik, 2002:113 [Type locality: Venezuela, T. F. Amazonas, Cerro de la Neblina, basecamp (rainforest clearing near Rio Baria), 0°50'N, 66°10'W, el. 140 m; NMNH; ♂; ♀].

**Distribution.** Guyana, Venezuela.

***angustipennis*** (*Chimarra*) Banks, 1903a:242 [Type locality: United States, Arizona], Hot Springs; NMNH; ♂; in *Chimarrha*]. —Ross, 1944:51 [♂; distribution]. —Ross, 1951a:68 [distribution]. —Denning, 1964: 133 [checklist]. —Bueno-Soria and Flint, 1978:195 [distribution]. —Blahnik, 1998:32 [♂; ♀; to *angustipennis* group, distribution]. —Maes, 1999:1190 [checklist]. —Muñoz-Quesada, 2000:280 [checklist]. —Baumgardner and Bowles, 2005:11 [distribution]. —Blinn and Ruiter, 2006:334 [biology]. —Bowles et al., 2007:23 [distribution; biology]. —Bueno-Soria et al., 2007:33 [distribution]. —Chamorro-Lacayo et al., 2007:45 [checklist]. —Ruiter and Blinn, 2009:5 [♀]. —Bueno-Soria and Barba-Álvarez, 2011:359 [checklist].

—*siva* Denning, 1949:115. —Blahnik, 1998:32 [to synonymy].

**Distribution.** Colombia, Costa Rica, Guatemala, El Salvador, Honduras, Mexico, Nicaragua, U.S.A., Venezuela.

***anticheira*** (*Chimarrita*) Vilarino and Calor, 2015b:127 [Type locality: Brazil, Bahia, Varzedo, Fazenda Baixa Grande, Riacho Cai Camarão, Propriedade do Sr. Getúlio Rodrigues Leal, 12°57'45.1"S, 39°27'12.1"W, el. 280 m; MZUSP; ♂; /♀; *simpliciforma* Group].

**Distribution.** Brazil.

***antigua*** (*Chimarra*) Flint, 1967b:6 [Type locality: Mexico, San Luis Potosi, El Salto, about 25 miles west of Antiguo Morelos; NMNH; ♂]. —Bueno-Soria and Flint, 1978:195 [distribution]. —Blahnik, 1998:117 [♂; ♀; to *primula* group].

**Distribution.** Mexico.

***antilliana*** (*Chimarra*) Flint, 1968b:13 [Type locality: Dominica, Mannett Gutter, near Clark Hall; NMNH; ♂; ♀; larva; pupa]. —Flint, 1968b:80 [checklist]. —Botosaneanu, 1988:221 [distribution]. —Flint and Sykora, 1993:49 [checklist]. —Blahnik, 1998:57 [♂; ♀; to *bidens* group; distribution]. —Botosaneanu, 2002:89 [checklist]. —Botosaneanu and Thomas, 2005:46 [distribution].

**Distribution.** Dominica, Guadeloupe, Martinique, St. Lucia.

***argentella*** (*Curgia*) Ulmer, 1906:92 [Type locality: Jamaica, Constant Springs; BMNH; ♂; in *Chimarrha*]. —Flint, 1968a:17 [♂; ♀; larva; distribution]. —Flint, 1968b:80 [checklist]. —Flint, 1998:55 [♂; redescription; to *braconoides* group]. —Malicky, 1999:115, 117 [distribution]. —Botosaneanu, 2002:90 [checklist].

**Distribution.** Jamaica.

***argentinica*** (*Chimarra*) Ulmer, 1909b:74 [Type locality: Argentina, Pedregal; ZSZMH; ♂; in *Chimarrha*]. —Weidner, 1964:70 [♂; lectotype]. —Mangeaud, 1996:154 [distribution]. —Blahnik, 1998:38 [♂; ♀; to *argentinica* group; distribution]. —Cohen, 2004:75 [distribution]. —Isa Miranda and Rueda Martín, 2014:200 [distribution].

—*armata* Navás, 1920b:38 [Type locality: Argentina, Mendoza; MZBS; ♂; in *Chimarrha*]. —Schmid, 1949a:318 [to synonymy].

—*canosa* Navás, 1918a:503 [Type locality: Argentina, Mendoza; MACN; ♂; in *Chimarrha*]. —Schmid, 1949a:320 [to synonymy, as *canossa*].

**Distribution.** Argentina, Bolivia, Peru.

***arima*** (*Chimarra*) Blahnik, 1998:108 [Type locality: Trinidad, Mount St. Benedict, 10°39'N, 61°24'W; NMNH; ♂; ♀; in *poolei* group]. —Botosaneanu and Alkins-Koo, 1993:30 [as Chimarra (Chimarra) spangleri, misidentification]. —Botosaneanu, 2002:89 [checklist].

**Distribution.** Trinidad.

***atilanoi*** (*Chimarra*) Blahnik, 1998:128 [Type locality: Mexico, San Luis Potosí, El Salto Falls, 26 mi W Antiguo Morelos; NMNH; ♂; ♀; in *utahensis* group].

**Distribution.** Mexico.

***aurantibasis*** (*Curgia*) Flint, 1998:53 [Type locality: Cuba, Ote., Piloto, Moa; INHS; ♂; in *braconoides* group]. —Flint, 1996c:16 [checklist]. —Botosaneanu, 2002:90 [checklist]. —Naranjo López and González Lazo, 2005:149 [checklist].

**Distribution.** Cuba.

***aureopunctata*** (*Curgia*) Flint, 1967b:7 [Type locality: Costa Rica, Turrialba; NMNH; ♂]. —Holzenthal, 1988c:57 [distribution]. —Maes and Flint, 1988:2 [distribution]. —Aguila, 1992:534 [distribution]. —Flint, 1998:63 [♂; redescription; distribution; to *banksi* group]. —Maes, 1999:1191 [checklist]. —Chamorro-Lacayo et al., 2007:45 [checklist]. —Armitage et al., 2015b:4 [checklist]. —Armitage and Cornejo, 2015:191 [checklist].

**Distribution.** Costa Rica, Nicaragua, Panama.

***aurivittata*** (*Curgia*) Flint, 1971c:22 [Type locality: Guyana, Essequibo, Mazaruni River, 39 miles southwest of Wineperu; NMNH; ♂]. —Flint, 1998:22 [♂; redescription; distribution; to *aurivittata* group]. —Paprocki et al., 2004:14 [checklist]. —Paprocki and França, 2014:74 [checklist].

**Distribution.** Brazil, Guyana, Venezuela.

***aviceps*** (*Curgia*) Flint, 1998:42 [Type locality: Peru, Depto. Cusco, Pcia. Paucartambo, stream 3 km E Puente San Pedro (13°03.3'S, 71°32.8'W), 41 km NW Pilcopata; NMNH; ♂; in *distermina* group].

**Distribution.** Peru.

***banksi*** (*Curgia*) (Ulmer), 1907d:198 [replacement name for *Wormaldia mediana* Banks, 1905:18, preoccupied by *Wormaldia mediana* McLachlan 1878:391]. [Type locality: Nicaragua, Chinandega; MCZ; ♂]. —Flint, 1967c:3 [♂; redescription]. —Holzenthal, 1988c:57 [distribution]. —Maes and Flint, 1988:3 [distribution]. —Flint, 1998:58 [♂;redescription; distribution; to *banksi* group]. —Maes, 1999:1191 [checklist]. —Chamorro-Lacayo et al., 2007:45 [checklist]. —Armitage et al., 2015b:4 [checklist]. —Armitage and Cornejo, 2015:191 [checklist].

**Distribution.** Costa Rica, Honduras, Nicaragua, Panama.

***barinas*** (*Otarrha*) Blahnik, 2002:72 [Type locality: Venezuela, Barinas, 22 km NW Barinitas; NMNH; ♂; in *Chimarra sensillata* species complex].

**Distribution.** Venezuela.

***barinita*** (*Curgia*) Flint, 1998:31 [Type locality: Venezuela, Edo. Barinas, 22 km NW Barinitas; NMNH; ♂; in *fernandezi* group]. —Muñoz-Quesada, 2000:280 [checklist].

**Distribution.** Colombia, Venezuela.

***barrettae*** (*Curgia*) (Banks), 1900:259 [Type locality: Mexico, Vera Cruz, Jalapa; MCZ; ♀; in *Philopotamus*]. —Banks, 1901:370 [distribution]. —Flint, 1967c:3 [mistakenly reduced to synonymy of *mexicana* (Banks)]. —Flint, 1998:44 [♂; redescription; distribution; resurrected to *mexicana* group]. —Maes, 1999:1191 [checklist]. —Chamorro-Lacayo et al., 2007:45 [checklist]. —Armitage et al., 2015b:4 [checklist]. —Armitage and Cornejo, 2015:191 [checklist].

**Distribution.** Costa Rica, Guatemala, Mexico, Nicaragua, Panama.

***beameri*** (*Chimarra*) Denning, 1950:101 [Type locality: United States, Texas, Uvalde Co.,Rio Frio River, Concan; SEMC; ♂]. —Bueno-Soria and Flint, 1978:196 [distribution]. —Blahnik, 1998:41 [♂; ♀; to *beameri* group, distribution]. —Bowles et al., 2007:23 [distribution; biology]. —Bueno-Soria et al., 2007:33 [distribution]. —Bueno-Soria and Barba-Álvarez, 2011:359 [checklist].

—*calva* Ross, 1959:174 [Type locality: Mexico, Chis.[Chiapas], Tecpatan; INHS; ♂]. —Bueno-Soria and Flint, 1978:190, 195 [to synonymy].

**Distribution.** Belize, Mexico, U.S.A.

***beckeri*** (*Curgia*) Flint, 1998:19 [Type locality: Brazil, Edo. Rio de Janeiro, Mangaratiba; MZUSP; ♂; in *morio* group]. —Paprocki et al., 2004:14 [checklist]. —Dumas et al., 2009:362 [distribution]. —Dumas et al., 2010:8 [distribution]. —Calor, 2011:323 [checklist]. —Dumas and Nessimian, 2012:22 [checklist]. —Barcelos-Silva et al., 2012:1278 [distribution]. —Paprocki and França, 2014:74 [checklist].

**Distribution.** Brazil.

***belizensis*** (*Chimarra*) Blahnik, 1998:42 [Type locality: Belize, Cayo Dist., just E of Roaring Creek at landing strip; NMNH; ♂; ♀; in *beameri* group].

**Distribution.** Belize.

***bicolor*** (*Chimarra*) (Banks), 1901:370 [Type locality: Mexico, Morelos, Cuernavaca; MCZ; ♂; in *Philopotamus*]. —Flint, 1967c:3 [♂; synonymy]. —Flint, 1967d:165 [distribution]. —Bueno-Soria and Flint, 1978:195 [distribution]. —Holzenthal, 1988c:55 [distribution]. —Maes and Flint, 1988:2 [distribution]. —Aguila, 1992:535 [distribution]. —Blahnik, 1998:55 [♂; ♀; to *bicolor* group; distribution]. —Maes, 1999:1190 [checklist]. —Bueno-Soria et al., 2005:75 [distribution]. —Bueno-Soria et al., 2007:33 [distribution]. —Chamorro-Lacayo et al., 2007:45 [checklist]. —Bueno-Soria and Barba-Álvarez, 2011:359 [checklist].

—*xesta* Denning, 1952:17 [Type locality: Mexico, Morelos, Cuernavaca; CAS; ♂]. —Flint, 1967c:3 [to synonymy].

**Distribution.** Costa Rica, Guatemala, Honduras, Mexico, Nicaragua.

***bicoloroides*** (*Chimarra*) Flint, 1967d:166 [Type locality: Mexico, Durango, 10 miles west of El Salto; CNC; ♂]. —Bueno-Soria and Flint, 1978:195 [distribution]. —Blahnik, 1998:56 [♂; to *bicoloroides* group].

**Distribution.** Mexico.

***bidens*** (*Chimarra*) Ulmer, 1909a:307 [Type locality: Venezuela, Caracas; UZMC; ♂; in *Chimarrha*]. —Flint, 1981a:13 [♂; synonymy]. —Flint, 1996a:72 [distribution]. —Blahnik, 1998:58 [♂; ♀; to *bidens* group; distribution].

—*caribea surinamensis* Flint, 1974c:30 [Type locality: Suriname, Brownsberg, mountain creek near Golddiggers camp; RNH; ♂]. —Flint, 1981a:13 [to synonymy].

**Distribution.** Suriname, Trinidad, Venezuela.

***bidentata*** (*Chimarra*) Blahnik, 1998:63 [Type locality: Peru, Pilcopata; NMNH; ♂; in *bidentata* group].

**Distribution.** Peru.

***bisectilis*** (*Curgia*) Flint, 1998:47 [Type locality: Costa Rica, Pcia. San José, Río Carara, Reserva Biologica Carara, 9.778°N, 84.531°W; NMNH; ♂; in *mexicana* group]. —Armitage et al., 2015b:4 [checklist]. —Armitage and Cornejo, 2015:191 [checklist].

**Distribution.** Costa Rica, Panama.

***blepharophera*** (*Curgia*) Flint, 1998:70 [Type locality: Mexico, Edo. Oaxaca, 7.4 mi [11.9 km] N Putla, Hwy 125; NMNH; ♂; in *banksi* group].

**Distribution.** Mexico.

***boraceia*** (*Curgia*) Flint, 1998:18 [Type locality: Brazil, Edo. São Paulo, Reserva Casa Grande, Corrego da Pedreira; MZUSP; ♂; in *morio* group]. —Paprocki et al., 2004:14 [checklist]. —Calor, 2011:323 [checklist]. —Paprocki and França, 2014:74 [checklist].

**Distribution.** Brazil.

***braconoides*** (*Curgia*) (Walker), 1860:179 [Type locality: St. Domingo [Dominican Republic]; BMNH; ♂; in *Curgia*]. —Betten and Mosely, 1940:15 [♂; holotype redescription]. —Flint, 1968b:80 [checklist]. —Flint, 1998:52 [♂; redescription; distribution; to *braconoides* group]. —Flint and Pérez-Gelabert, 1999:42 [checklist]. —Botosaneanu, 2002:90 [checklist]. —Flint and Sykora, 2004:50 [distribution]. —Pérez-Gelabert, 2008:301 [checklist].

**Distribution.** Dominican Republic, Haiti.

***brasiliana*** (*Curgia*) Ulmer, 1905a:96 [Type locality: Brazil, Santa Catharina [sic]; PAN; ♂; in *Chimarrha*]. —Flint, 1966a:3 [♂; lectotype; synonymy]. —Angrisano, 1997b:56 [distribution]. —Flint, 1998:72 [♂; redescription; distribution; to *banksi* group]. —Almeida and Marinoni, 2001:973 [♀]. —Paprocki et al., 2004:14 [checklist]. —Manzo et al., 2014:166 [distribution]. —Paprocki and França, 2014:74 [checklist].

—*parva* (Ulmer), 1905b:90 [Type locality: Brazil, Blumenau; NMW; ♂; in *Wormaldia*]. —Flint, 1966a:3 [lectotype; to synonymy].

**Distribution.** Argentina, Brazil, Paraguay, Uruguay.

***burmeisteri*** (*Curgia*) Flint, 1998:20 [Type locality: Brazil, Edo. Rio de Janeiro, municipal water supply, Nova Friburgo; MZUSP; ♂; in *morio* group]. —Paprocki et al., 2004:14 [checklist]. —Dumas et al., 2009:362 [distribution]. —Paprocki and França, 2014:75 [checklist].

**Distribution.** Brazil.

***butleri*** (*Chimarra*) Denning, 1962a:33 [Type locality: United States, California, Kings Canyon National Park, Sheep Creek Campground; CAS; ♂]. —Blahnik, 1998:129 [♂; ♀; to *utahensis* group, distribution]. —Bueno-Soria et al., 2007:33 [distribution].

**Distribution.** Mexico, U.S.A.

***calori*** (*Chimarra*) Blahnik and Holzenthal, 2012:3 [Type locality: Brazil, São Paulo, Altinópolis, Cachoeira Dos Macacos, 20°55.380'S, 47°22.758'W, el. 759 m; MZUSP; ♂; ♀; in *ortiziana* group]. —Paprocki and França, 2014:72 [checklist].

**Distribution.** Brazil.

***camella*** (*Chimarrita*) Blahnik, 1997:219 [Type locality: Brazil, Minas Gerais, Serra do Cipó, km 116; MZUSP; ♂; in *simpliciforma* group]. —Blahnik et al., 2004:5 [distribution]. —Paprocki et al., 2004:14 [checklist]. —Dumas et al., 2009:362 [distribution]. —Calor, 2011:323 [checklist]. —Blahnik and Holzenthal, 2012:24 [♀]. —Dumas and Nessimian, 2012:22 [checklist]. —Paprocki and França, 2014:73 [checklist].

**Distribution.** Brazil.

***camposae*** (*Curgia*) Flint, 1998:77 [Type locality: Brazil, Edo. Minas Gerais, Corrego Marumbe, Municipio Nova Lima, 20°03'31"S, 43°53'29"W; MZUSP; ♂; in *banksi* group]. —Paprocki et al., 2004:14 [checklist]. —Paprocki and França, 2014:75 [checklist].

**Distribution.** Brazil.

***camura*** (*Chimarrita*) Blahnik, 1997:222 [Type locality: Brazil, Rio de Janeiro, km 54, 26 km E Nova Friburgo; MZUSP; ♂; ♀; in *simpliciforma* group]. —Blahnik et al., 2004:5 [distribution]. —Paprocki et al., 2004:14 [checklist]. —Dumas et al., 2009:362 [distribution]. —Calor, 2011:323 [checklist]. —Dumas and Nessimian, 2012:22 [checklist]. —Paprocki and França, 2014:73 [checklist].

**Distribution.** Brazil.

***canoaba*** (*Curgia*) Flint, 1998:33 [Type locality: Venezuela, Edo. Carabobo, near Canoabo; NMNH; ♂; in *canoaba* group].

**Distribution.** Venezuela.

***caribea caribea*** (*Chimarra*) Flint, 1968b:14 [Type locality: Grenada, 2 miles west of Lake Grand Etang; NMNH; ♂; ♀]. —Flint, 1968b:80 [checklist]. —Malicky, 1983:264 [distribution]. —Botosaneanu, 1989b:205 [distribution; larva]. —Flint, 1990a:47 [distribution]. —Flint and Sykora, 1993:51 [distribution]. —Blahnik, 1998:59 [♂; ♀; *bidens* group; distribution]. —Botosaneanu, 2002:89 [checklist]. —Botosaneanu and Viloria, 2002:108 [distribution]. —Chamorro-Lacayo et al., 2007:45 [checklist]. —Armitage et al., 2015b:3 [checklist]. —Armitage and Cornejo, 2015:191 [checklist].

**Distribution.** Grenada, Mustique, Nicaragua, Panama, Suriname, Trinidad, Venezuela (Isla Margarita).

***caribea tobaga*** Botosaneanu, 1990a:48 [Type locality: Tobago, Scarborough; BMNH; ♂]. —Botosaneanu and Sakal, 1992:202 [distribution]. —Botosaneanu and Alkins-Koo, 1993:30 [to synonymy]. —Flint, 1996a:72 [resurrected as subspecies; distribution].

**Distribution.** Tobago.

***carolae*** (*Curgia*) Flint, 1998:40 [Type locality: Venezuela, Edo. Bolivar, La Escalera, 108 km S Río Cuyuni; NMNH; ♂; in *medioloba* group].

**Distribution.** Venezuela.

***cascada*** (*Chimarra*) Blahnik, 1998:132 [Type locality: Costa Rica, Alajuela, Río Peje and falls, ca. 1 km SE San Vicente, 10.277°N, 84.388°W; NMNH; ♂; ♀; in *virgencita* group].

**Distribution.** Costa Rica.

***cauca*** (*Chimarra*) Blahnik and Holzenthal, 2012:16 [Type locality: Colombia, Cauca, Municipio de Inz., Quebrada San Andrés, ca. 500 m, W Restaurante “La Portada”, San Andrés de Pisimbalá, 2°34'56"N, 76°2'36"W, el. 1750 m,; UMSP; ♂; ♀; in *poolei* group].

**Distribution.** Colombia.

***centralis*** (*Curgia*) Ross, 1959:178 [Type locality: Panama, Potrerillos; INHS; ♂]. —Holzenthal, 1988c:57 [distribution]. —Aguila, 1992:534 [distribution]. —Flint, 1998:61 [mlae/; redescription; distribution; to *banksi* group]. —Chamorro-Lacayo et al., 2007:45 [checklist]. —Armitage et al., 2015b:4 [checklist]. —Armitage and Cornejo, 2015:191 [checklist].

**Distribution.** Costa Rica, Ecuador, Nicaragua, Panama.

***centrispina*** (*Curgia*) Flint, 1998:21 [Type locality: Brazil, Edo. Minas Gerais, Rio Cipó, Yaboticatubas; INHS; ♂; in *morio* group]. —Paprocki et al., 2004:14 [checklist]. —Paprocki and França, 2014:75 [checklist].

**Distribution.** Brazil.

***chela*** (*Chimarrita*) Blahnik, 1997:212 [Type locality: Venezuela, T.F. Amazonas, Cerro de la Neblina, Basecamp; NMNH; ♂; ♀; in *rosalesi* group]. —Santos and Nessimian, 2009b:23 [distribution]. —Paprocki and França, 2014:73 [checklist].

**Distribution.** Brazil, Venezuela.

***chimalapa*** (*Chimarra*) Bueno-Soria, Santiago-Fragoso, and Barba-Álvarez, 2001:149 [Type locality: Mexico, Tabasco, Municipio de Huimanguillo, Arroyo las Flores, Villa de Guadalupe 2a Sección Los Chimalapas, km 5 Ruta Malpasito-Carlos A. Madrazo, 17°22'05"N, 93°36'25"W; CNIN; ♂; in *primula* group]. —Bueno-Soria et al., 2005:75 [distribution].

**Distribution.** Mexico.

***chocoensis*** (*Chimarra*) Blahnik, 1998:109 [Type locality: Colombia, Chocó, km 130, 86 km E Quibdo; NMNH; ♂; in *poolei* group]. —Muñoz-Quesada, 2000:280 [checklist].

**Distribution.** Colombia.

***chrysosoma*** (*Curgia*) Flint, 1998:27 [Type locality: Bolivia, Pcia. La Paz, Yungas La Paz, Circuata to Cajuata; NMNH; ♂; in *margaritae* group].

**Distribution.** Bolivia, Peru.

***cipoensis*** (*Curgia*) Flint, 1998:18 [Type locality: Brazil, Edo. Minas Gerais, Serra do Cipó, km 110 [on road to Conceição do Mato Dentro]; MZUSP; ♂; in *morio* group]. —Paprocki et al., 2004:14 [checklist]. —Paprocki and França, 2014:75 [checklist].

**Distribution.** Brazil.

***cirrifera*** (*Curgia*) Flint, 1998:37 [Type locality: Venezuela, Territorio Federal Amazonas, Cerro de la Neblina, Basecamp; NMNH; ♂; in *medioloba* group].

**Distribution.** Venezuela.

***claviloba*** (*Curgia*) Flint, 1974c:22 [Type locality: Suriname, Nassau Mountains, km. 11.2; RNH; ♂]. —Flint, 1998:36 [♂; redescription; to *medioloba* group].

**Distribution.** Suriname.

***coheni*** (*Chimarra*) Blahnik, 1998:43 [Type locality: Ecuador, Pichincha, Santo Domingo de los Colorados (29 km W); NMNH; ♂; in *beameri* group].

**Distribution.** Ecuador.

***colmillo*** (*Chimarra*) Blahnik and Holzenthal, 1992b:412 [Type locality: Costa Rica, Guanacaste, Parque Nacional Guanacaste, Río San Josecito, Estación Mengo, 10.922°N, 85.470°W; NMNH; ♂]. —Blahnik, 1998:78 [♂; ♀; to *ortiziana* group]. —Armitage et al., 2015a:2 [distribution]. —Armitage et al., 2015b:3 [checklist]. —Armitage and Cornejo, 2015:191 [checklist].

**Distribution.** Costa Rica, Panama.

***conica*** (*Curgia*) Flint, 1983a:20 [Type locality: Argentina, Pcia. Misiones, Arroyo Piray Mini, Rt.17 W Dos Hermanas; NMNH; ♂]. —Flint, 1998:16 [♂; redescription; distribution; variation; to *morio* group]. —Blahnik et al., 2004:5 [distribution]. —Paprocki et al., 2004:14 [checklist]. —Dumas et al., 2009:363 [distribution]. —Nogueira and Cabette, 2011:352 [distribution]. —Barcelos-Silva et al., 2012:1278 [distribution]. —Paprocki and França, 2014:75 [checklist].

**Distribution.** Argentina, Brazil.

***consimilis*** (*Curgia*) Martynov, 1912:33 [Type locality: Peru, Callanga; ASL ?; ♀; in *Chimarrha*].

**Distribution.** Peru.

***cornuta*** (*Chimarra*) Ross, 1959:175 [Type locality: Mexico, Chis.[Chiapas], Finca Vergel; INHS; ♂]. —Bueno-Soria and Flint, 1978:195 [distribution]. —Blahnik, 1998:69 [♂; to *cornuta* group]. —Bueno-Soria and Barba-Álvarez, 2011:359 [checklist].

**Distribution.** Mexico.

***costaricensis*** (*Curgia*) Flint, 1998:32 [Type locality: Costa Rica, Pcia. Guanacaste, Río Negro, Parque Nacional Rincón de la Vieja (10.765°N, 85.313°W; NMNH; ♂; in *fernandezi* group]. —Armitage et al., 2015a:2 [distribution]. —Armitage et al., 2015b:4 [checklist]. —Armitage and Cornejo, 2015:191 [checklist].

**Distribution.** Costa Rica, Panama.

***creagra*** (*Chimarra*) Flint, 1981a:14 [Type locality: Venezuela, Aragua, Maracay, Río Limón, Estación Pisicultura; NMNH; ♂; ♀]. —Blahnik, 1998:93 [♂; ♀; to *picea* group].

**Distribution.** Ecuador, Venezuela.

***crena*** (*Chimarra*) Bueno-Soria, 1983b:453 [Type locality: Mexico, Veracruz, Rio Jamapa, 6 km east from Coscomatepec; IBUNAM; ♂]. —Blahnik, 1998:118 [♂; ♀; to *primula* group].

**Distribution.** Mexico.

***cressae*** (*Chimarra*) Blahnik, 1998:110 [Type locality: Venezuela, Miranda, P. N. Guatopo, Quebrada Macanilla at La Macanilla, 10.113°N, 66.516°W; NMNH; ♂; in *poolei* group].

**Distribution.** Venezuela.

***cubanorum*** (*Otarrha*) Botosaneanu, 1980:69, 98 [Type locality: Cuba, Prov. Oriente, Cupeyal; ZMUA; ♂]. —Botosaneanu, 1979:45 [*nomen nudum* (name included in checklist); distribution]. —Flint, 1996c:16 [checklist]. —Botosaneanu, 2002:90 [checklist]. —Blahnik, 2002:73 [♂; to *Otarrha*]. —Naranjo López and González Lazo, 2005:149 [checklist].

**Distribution.** Cuba.

***cultellata*** (*Curgia*) Flint, 1983a:15 [Type locality: Brazil, Edo. Santa Catarina, Nova Teutonia (27°11'S, 52°23'W); NMNH; ♂]. —Flint, 1998:73 [♂; redescription; distribution; to *banksi* group]. —Paprocki et al., 2004:14 [checklist]. —Dumas et al., 2009:363 [distribution]. —Dumas et al., 2010:8 [distribution]. —Paprocki and França, 2014:75 [checklist].

**Distribution.** Argentina, Brazil, Venezuela.

***curfmani*** (*Chimarra*) Ross, 1959:174 [Type locality: Mexico, Chis.[Chiapas], Ocosingo Valley, Finca Monte Libano; INHS; ♂]. —Bueno-Soria and Flint, 1978:195 [distribution]. —Blahnik, 1998:44 [♂; *beameri* group, distribution]. —Bueno-Soria and Barba-Álvarez, 2011:359 [checklist].

**Distribution.** Mexico.

***curvipenis*** (*Chimarra*) Blahnik and Holzenthal, 2012:22 [Type locality: Brazil, Minas Gerais, Serra do Cipó, Capão da Mata, 19°19.347'S, 43°32.249'W, el. 1170 m; MZUSP; ♂; ♀; in *simpliciforma* group]. —Paprocki and França, 2014:73 [checklist].

**Distribution.** Brazil.

***darlingtoni*** (*Otarrha*) Blahnik, 2002:73 [Type locality: Cuba, coast below Pico Turquino; MCZ; ♂]. —Botosaneanu, 2002:90 [checklist]. —Naranjo López and González Lazo, 2005:149 [checklist].

**Distribution.** Cuba.

***decimlobata*** (*Chimarra*) Flint, 1991:30 [Type locality: Colombia, Dpto. Antioquia, Quebrada Espadera, 7 km NW Medellín [road to Sta. Elena]; NMNH; ♂]. —Blahnik, 1998:94 [♂; ♀; to *picea* group]. —Muñoz-Quesada, 2000:280 [checklist].

**Distribution.** Colombia.

***dentosa*** (*Chimarra*) Ross, 1948b:25 [Type locality: Mexico, Michoacan, Apatzingan; INHS; ♂]. —Bueno-Soria and Flint, 1978:195 [distribution]. —Maes and Flint, 1988:2 [distribution]. —Blahnik, 1998:71 [♂; ♀; to *dentosa* group, distribution]. —Maes, 1999:1190 [checklist]. —Chamorro-Lacayo et al., 2007:45 [checklist]. —Bueno-Soria and Barba-Álvarez, 2011:359 [checklist]. —Armitage et al., 2015b:3 [checklist]. —Armitage and Cornejo, 2015:191 [checklist].

**Distribution.** Costa Rica, Guatemala, Mexico, Nicaragua, Panama.

***desirae*** (*Chimarra*) Blahnik and Holzenthal, 2012:18 [Type locality: Bolivia, La Paz, AMNI Madidi, San Migual de Bala, Arroyo Bacuatra Grande, 14°30.737'S, 67°31.385'W, el. 280 m; UMSP; ♂; in *poolei* group].

**Distribution.** Colombia.

***diakis*** (*Otarrha*) Flint, 1971c:23 [Type locality: Brazil [Edo. Amazonas], Gebeit Endstation, Rio Marauia, Bergbach II, etwa 350 m, tiber dem Meeresspiegel, schattig, starkes Gefälle tiber Granitblöcke; NMNH; ♂]. —Blahnik, 2002:75 [♂; to *Otarrha*]. —Paprocki et al., 2004:15 [checklist]. —Paprocki and França, 2014:78 [checklist].

**Distribution.** Brazil.

***diannae*** (*Otarrha*) Flint and Sykora, 1993:48 [Type locality: St. Lucia, Quarter of Soufriere, Fond St. Jacques (13°50'N, 61°02'W); NMNH; ♂]. —Blahnik, 2002:75 [to *Otarrha*]. —Botosaneanu, 2002:90 [checklist].

**Distribution.** St. Lucia.

***diaphora*** (*Otarrha*) Blahnik, 2002:75 [Type locality: Venezuela, T. F. Amazonas, Agua Blanca, 0°49'N, 66°08'W, Cerro de la Neblina, el. 160 m; NMNH; ♂; ♀].

**Distribution.** Venezuela.

***didyma*** (*Curgia*) Flint, 1998:69 [Type locality: Panama, Pcia. Panamá, Cerro Azul; NMNH; ♂; variation, in *banksi* group]. —Armitage et al., 2015b:4 [checklist]. —Armitage and Cornejo, 2015:191 [checklist].

**Distribution.** Ecuador, Panama, Venezuela.

***distermina*** (*Curgia*) Flint, 1998:41 [Type locality: Bolivia, Dpto. La Paz, quebradas del Río Zongo; NMNH; ♂; in *distermina* group].

**Distribution.** Bolivia.

***dolabrifera*** (*Chimarra*) Flint and Reyes, 1991:478 [Type locality: Ecuador, Prov. Pichincha, Río Palenque Biological Station, 47 km S Santo Domingo de los Colorados]; NMNH; ♂]. —Blahnik, 1998:79 [♂; ♀; to *ortiziana* group; distribution]. —Muñoz-Quesada, 2000:280 [checklist]. —Armitage et al., 2015b:3 [checklist]. —Armitage and Cornejo, 2015:191 [checklist].

**Distribution.** Colombia, Ecuador, Panama, Peru.

† ***dommeli*** (*incertae sedis*) Wichard, 1983a:142 [Type locality: Dominican Republic; collection Wichard; ♂; in amber; in *Chimarrita*]. —Flint and Pérez-Gelabert, 1999:42 [checklist]. —Botosaneanu, 2002:91 [checklist]. —Wichard, 2007a:14 [♂; to *Chimarrita*; key]. —Pérez-Gelabert, 2008:301 [checklist]. —Kjer, et al., 2014:352 [*incertae sedis*].

**Distribution.** Dominican Republic.

***dominicana*** (*Otarrha*) Flint, 1968b:10 [Type locality: Dominica, .4 miles east Pont Casse; NMNH; ♂; ♀; larva; pupa]. —Flint, 1968b:80 [checklist]. —Malicky, 1983:264 [distribution]. —Botosaneanu, 1988:221 [distribution]. —Flint and Sykora, 1993:49 [checklist]. —Botosaneanu, 2002:90 [checklist]. —Blahnik, 2002:77 [♂; ♀; to *Otarrha*]. —Botosaneanu and Thomas, 2005:46 [distribution].

**Distribution.** Dominica, Guadeloupe, Martinique.

***donamariae*** (*Curgia*) Denning and Sykora, 1968:173 [Type locality: Brazil, Pará, Santa Izabel; CAS; ♂; ♀]. —Sattler, 1962:125 [biology; larva; pupa; as *Chimarrha* species]. —Flint, 1998:26 [♂; redescription; distribution; to *ensifera* group]. —Paprocki et al., 2004:14 [checklist]. —Paprocki and França, 2014:75 [checklist].

**Distribution.** Brazil, Peru.

***duckworthi*** (*Chimarra*) Flint, 1967b:5 [Type locality: Costa Rica, Turrialba; NMNH; ♂]. —Holzenthal, 1988c:56 [distribution]. —Flint, 1991:28 [♂; distribution]. —Aguila, 1992:535 [distribution]. —Blahnik, 1998:60 [♂; ♀; *bidens* group; distribution]. —Muñoz-Quesada, 2000:280 [checklist]. —Chamorro-Lacayo et al., 2007:45 [checklist]. —Bueno-Soria and Barba-Álvarez, 2011:359 [checklist]. —Armitage et al., 2015b:3 [checklist]. —Armitage and Cornejo, 2015:191 [checklist].

**Distribution.** Colombia, Costa Rica, Ecuador, Guatemala, Honduras, Mexico, Nicaragua, Panama, Venezuela.

***dudosa*** (*Chimarra*) Blahnik, 1998:45 [Type locality: Panama, San Blas, Río Carti Grande, 2 km W Nusagandi; NMNH; ♂; ♀; in *beameri* group]. —Armitage et al., 2015b:3 [checklist]. —Armitage and Cornejo, 2015:191 [checklist].

**Distribution.** Panama.

***elia*** (*Chimarra*) Ross, 1944:269 [Type locality: United States, Texas, spring-fed stream west of Brackettville; INHS; ♂]. —Ross, 1951a:68 [distribution]. —Flint, 1958:24 [phallus ♂]. —Denning, 1964:133 [checklist]. —Bueno-Soria and Flint, 1978:195 [distribution]. —Holzenthal, 1988c:56 [distribution]. —Maes and Flint, 1988:2 [distribution]. —Blahnik, 1998:72 [♂; ♀; to *elia* group]. —Maes, 1999:1190 [checklist]. —Bowles et al., 2007:23 [distribution; biology]. —Bueno-Soria et al., 2007:33 [distribution]. —Chamorro-Lacayo et al., 2007:45 [checklist]. —Bueno-Soria and Barba-Álvarez, 2011:360 [checklist].

—*barranca* Denning, 1962b:402 [Type locality: Mexico, Baja California, Sierra San Pedro Martir, La Grulla; CAS; ♂]. —Denning, 1964: 133 [checklist]. —Bueno-Soria and Flint, 1978:190, 195 [to synonymy].

**Distribution.** Costa Rica, Guatemala, Mexico, Nicaragua, U.S.A.

***embia*** (*Chimarra*) Ross, 1959:170 [Type locality: Mexico, Chis.[Chiapas], Tonala; INHS; ♂]. —Flint, 1967d:166 [distribution]. —Bueno-Soria and Flint, 1978:196 [distribution]. —Maes and Flint, 1988:2 [distribution]. —Blahnik, 1998:73 [♂; ♀; to *elia* group, distribution]. —Blahnik et al., 2007:13 [♂]. —Chamorro-Lacayo et al., 2007:45 [checklist]. —Bueno-Soria and Barba-Álvarez, 2011:360 [checklist].

—*rizona* Denning, 1962b:403 [Type locality: Mexico, Nayarit, Rio de las Canyas, 8 miles NW. Acaponeta; CAS; ♂]. —Bueno-Soria and Flint, 1978:190, 196 [to synonymy].

—*spicula* Denning, 1962b:404 [Type locality: Mexico, Veracruz, Alazan, approximately 75 miles south of Tampico; CAS; ♂]. —Bueno-Soria and Flint, 1978:190, 196 [to synonymy].

—*stellula* Denning, 1962b:404 [Type locality: Mexico, Veracruz, Alazan; CAS; ♂]. —Bueno-Soria and Flint, 1978:190, 196 [to synonymy].

**Distribution.** El Salvador, Honduras, Mexico, Nicaragua.

***emima*** (*Chimarra*) Ross, 1959:172 [Type locality: [Panama], Canal Zone, Madden Dam; INHS; ♂]. —Holzenthal, 1988c:56 [distribution]. —Flint, 1991:29 [♂; distribution]. —Flint and Reyes, 1991:480 [distribution]. —Aguila, 1992:535 [distribution]. —Blahnik, 1998:95 [♂; ♀; to *picea* group, distribution]. —Muñoz-Quesada, 2000:280 [checklist]. —Chamorro-Lacayo et al., 2007:45 [checklist]. —Armitage et al., 2015b:3 [checklist]. —Armitage and Cornejo, 2015:191 [checklist].

**Distribution.** Colombia, Costa Rica, Ecuador, Nicaragua, Panama, Peru, Venezuela.

***ensifera*** (*Curgia*) Flint, 1998:26 [Type locality: Venezuela, Territorio Federal Amazonas, Cerro de la Neblina, Camp IV, 0°58'N, 65°57'W; NMNH; ♂; in *ensifera* group].

**Distribution.** Venezuela.

***erectiloba*** (*Curgia*) Flint, 1998:78 [Type locality: Peru, Dpto. Cusco, Pcia, Paucartambo, Puente San Pedro at km 152 (13°03.3'S, 71°32.8'W), 44 km NW Pilcopata; NMNH; ♂; in *banksi* group].

**Distribution.** Peru.

***espinosa*** (*Chimarra*) Blahnik, 1998:33 [Type locality: Mexico, San Luis Potosí, El Salto; NMNH; ♂; ♀; in *angustipennis* group].

**Distribution.** Mexico.

***fernandezi*** (*Curgia*) Flint, 1981a:11 [Type locality: Venezuela, Aragua, El Limon; IZAM; ♂]. —Flint, 1998:31 [♂; redescription; to *fernandezi* group].

**Distribution.** Venezuela.

***fimbriata*** (*Curgia*) Flint, 1974c:22 [Type locality: Suriname, Nassau Mountains, trail south from km. 7; RNH; ♂]. —Flint, 1998:38 [♂; redescription; to *medioloba* group].

**Distribution.** Suriname.

***fittkaui*** (*Curgia*) Flint, 1971c:22 [Type locality: Brazil [Edo. Amazonas], Rio Marauia, Endstation; NMNH; ♂]. —Flint, 1998:74 [♂; redescription; distribution; to *banksi* group]. —Paprocki et al., 2004:14 [checklist]. —Paprocki and França, 2014:76 [checklist].

**Distribution.** Brazil, Guyana.

***flinti*** (*Chimarra*) Bueno-Soria, 1985:15 [Type locality: Mexico, Oaxaca, Uxpanapa; IBUNAM; ♂]. —Holzenthal, 1988c:56 [distribution]. —Aguila, 1992:535 [distribution]. —Botosaneanu and Sakal, 1992:202 [distribution]. —Botosaneanu and Alkins-Koo, 1993:29 [larva]. —Flint, 1996a:71 [distribution]. —Blahnik, 1998:46 [♂; ♀; *beameri* group; distribution]. —Maes, 1999:1190 [checklist]. —Muñoz-Quesada, 2000:280 [checklist]. —Botosaneanu, 2002:90 [checklist]. —Chamorro-Lacayo et al., 2007:45 [checklist]. —Bueno-Soria and Barba-Álvarez, 2011:360 [checklist]. —Armitage et al., 2015b:3 [checklist]. —Armitage and Cornejo, 2015:191 [checklist].

**Distribution.** Belize, Brazil, Colombia, Costa Rica, Honduras, Mexico, Nicaragua, Panama, Tobago, Trinidad, Venezuela.

***forcipata*** (*Chimarrita*) Blahnik, 1997:213 [Type locality: Venezuela, T.F. Amazonas, Puerto Ayacucho (40 km S), El Tobogán; NMNH; ♂; ♀; in *rosalesi* group].

**Distribution.** Venezuela.

***froehlichi*** (*Curgia*) Flint, 1998:16 [Type locality: Brazil, Edo. Rio de Janeiro, km 54, 26 km E Nova Friburgo; MZUSP; ♂; in *morio* group]. —Paprocki et al., 2004:14 [checklist]. —Dumas et al., 2009:363 [distribution]. —Calor, 2011:323 [checklist]. —Barcelos-Silva et al., 2012:1278 [distribution]. —Dumas and Nessimian, 2012:22 [checklist]. —Paprocki and França, 2014:76 [checklist].

**Distribution.** Brazil.

***garciai*** (*Otarrha*) Botosaneanu, 1980:99 [Type locality: Cuba, Prov. Oriente, Cupeyal; ZMUA; ♂]. —Botosaneanu, 1979:45 [*nomen nudum* (name included in checklist); distribution]. —Flint, 1996c:16 [checklist]. —Botosaneanu, 2002:91 [checklist]. —Blahnik, 2002:77 [♂; to *Otarrha*]. —Naranjo López and González Lazo, 2005:149 [checklist].

**Distribution.** Cuba.

***geranoides*** (*Curgia*) Flint, 1998:71 [Type locality: Ecuador, Pcia. Pichincha, Río Umachaca, Forestry Station Maquipucuna, ca.5 km E Nanegal; NMNH; ♂; in *banksi* group]. —Muñoz-Quesada, 2000:280 [checklist].

**Distribution.** Colombia, Ecuador.

***gibba*** (*Chimarra*) Blahnik, 1998:75 [Type locality: Costa Rica, Guanacaste, Río Tempisquito, ca. 3 km S Rt. 1, 10.790°N, 85.522°W; NMNH; ♂; ♀; in *elia* group]. —Chamorro-Lacayo et al., 2007:45 [checklist]. —Bueno-Soria and Barba-Álvarez, 2011:360 [checklist].

**Distribution.** Costa Rica, Mexico, Nicaragua.

***gilvimacula*** (*Curgia*) Flint, 1998:52 [Type locality: Dominican Republic, La Vega Prov., Río Camú, 19 km NE Jarabacoa; NMNH; ♂; in *braconoides* group]. —Botosaneanu, 1996:12 [♀; distribution]. —Flint and Pérez-Gelabert, 1999:42 [checklist]. —Botosaneanu, 2002:90 [checklist]. —Flint and Sykora, 2004:50 [distribution]. —Pérez-Gelabert, 2008:301 [checklist].

**Distribution.** Dominican Republic, Haiti.

***gondela*** (*Chimarra*) Flint, 1974c:27 [Type locality: Suriname, Brownsberg, mountain creek near Golddiggers camp; RNH; ♂]. —Blahnik, 1998:80 [♂; ♀; to *ortiziana* group, distribution].

**Distribution.** Suriname, Venezuela.

***guanacasteca*** (*Chimarra*) Blahnik and Holzenthal, 1992b:415 [Type locality: Costa Rica, Alajuela, Río Pizote, ca.5 km N Dos Ríos, 10.948°N, 85.291°W; NMNH; ♂]. —Blahnik, 1998:48 [♂; ♀; *beameri* group, distribution].

**Distribution.** Costa Rica.

***guapa*** (*Otarrha*) Botosaneanu, 1977:251 [Type locality: Cuba, Prov. Oriente, Rio Yumuri, Baracoa; NMNH; ♂; ♀]. —Kumanski, 1987:7 [distribution]. —Botosaneanu, 1979:45 [distribution]. —Botosaneanu, 1994b:459 [larva]. —Flint, 1996c:16 [checklist]. —Botosaneanu, 2002:91 [checklist]. —Blahnik, 2002:77 [♂; ♀; to *Otarrha*]. —López del Castillo et al., 2004:229 [distribution]. —Naranjo López and González Lazo, 2005:149 [checklist]. — López del Castillo et al., 2007:171 [checklist].

**Distribution.** Cuba.

***guatemalensis*** (*Chimarra*) Blahnik, 1998:119 [Type locality: Guatemala, El, Progresso, Finca la Cajeta; NMNH; ♂; in *primula* group]. —Maes, 1999:1190 [checklist]. —Chamorro-Lacayo et al., 2007:45 [checklist].

**Distribution.** Guatemala, Nicaragua.

***guyanensis*** (*Curgia*) Flint, 1998:41 [Type locality: Guyana, Kumu Stream, 25 km SE Lethem, 3°15.9'N, 59°43.6'W; NMNH; ♂; in *medioloba* group].

**Distribution.** Guyana.

***haesitationis*** (*Chimarra*) Botosaneanu, 1994a:47 [Type locality: Guadeloupe, rivière St-Louis dans les Hauts du Matouba; ZMUA; ♂; ♀]. —Blahnik, 1998:61 [♂; ♀; to *bidens* group]. —Botosaneanu, 2000:256 [distribution]. —Botosaneanu, 2002:90 [checklist]. —Botosaneanu and Thomas, 2005:55 [checklist].

**Distribution.** Guadeloupe.

***hairouna*** (*Chimarra*) Botosaneanu, 1990a:43 [Type locality: Saint Vincent, forest stream at Montreal (Mariaqua Valley, Mesopotamia); ZMUA; ♂; larva; pupa]. —Flint and Sykora, 1993:49 [checklist]. —Blahnik, 1998:49 [♂; to *beameri* group]. —Botosaneanu, 2002:90 [checklist].

**Distribution.** St. Vincent.

***heligma*** (*Chimarrita*) Blahnik, 1997:222 [Type locality: Brazil, Minas Gerais, Serra do Cipó; MZUSP; ♂; ♀; in *simpliciforma* group]. —Paprocki et al., 2004:14 [checklist]. —Paprocki and França, 2014:73 [checklist].

**Distribution.** Brazil.

***heppneri*** (*Chimarrita*) Blahnik, 1997:225 [Type locality: Peru, Loreto, Callicebus Res. Station, Mishana, Río Nanay, 25 km SW Iquitos; NMNH; ♂; ♀; in *simpliciforma* group].

**Distribution.** Peru.

***hyoeides*** (*Curgia*) Flint, 1983a:17 [Type locality: Argentina, Pcia. Misiones, Río Iguazú, Camp Nañdu; NMNH; ♂]. —Angrisano, 1997b:56 [distribution]. —Flint, 1998:50 [♂; redescription; to *mexicana* group]. —Paprocki et al., 2004:14 [checklist]. —Calor, 2011:323 [checklist]. —Barcelos-Silva et al., 2012:1278 [distribution]. —Souza et al., 2013a:8 [distribution]. —Paprocki and França, 2014:76 [checklist].

**Distribution.** Argentina, Brazil, Paraguay, Uruguay.

***immaculata*** (*Curgia*) Ulmer, 1911:15 [Type locality: Bolivia; ZMHU; ♂; in *Chimarrha*]. —Flint, 1981a:12 [♂; distribution]. —Flint, 1998:82 [♂; redescription; distribution; to *immaculata* group]. —Muñoz-Quesada, 2000:280 [checklist].

**Distribution.** Bolivia, Colombia, Ecuador, Peru, Venezuela.

***inchoata*** (*Chimarra*) Blahnik and Holzenthal, 2012:9 [Type locality: Venezuela, Sucre, Península de Paria, Puerto Viejo, Río Puerto Viejo, 10°43.137'N, 62°28.743'S, el. 15 m; UMSP; ♂; ♀; in *picea* group].

**Distribution.** Venezuela.

***incipiens*** (*Otarrha*) Blahnik, 2002:81 [Type locality: Venezuela, Distrito Federal, Bajo Seco, Est. Exp. Bajo Seco, c. 15 km NE Colonia Tovar, el. 2000 m; NMNH; ♂; in *Chimarra sensillata* species complex].

**Distribution.** Venezuela.

***inflata*** (*Chimarra*) Blahnik, 1998:110 [Type locality: Ecuador, Napo, Lago Agrio (5 km N); NMNH; ♂; ♀; in *poolei* group].

**Distribution.** Ecuador.

***irwini*** (*Curgia*) Flint, 1998:34 [Type locality: Venezuela, Edo. Aragua, Rancho Grande; CAS; ♂; in *canoaba* group].

**Distribution.** Venezuela.

***izabala*** (*Chimarra*) Blahnik, 1998:34 [Type locality: Guatemala, Izabal, nr. Matias de Galvez; NMNH; ♂; ♀; in *angustipennis* group].

**Distribution.** Belize, Guatemala.

***jamaicensis*** (*Otarrha*) Flint, 1968a:18 [Type locality: Jamacia, St. Andrew, Hardwar Gap, Dicks Pond trail; NMNH; ♂; ♀; larva; pupa]. —Flint, 1968b:80 [checklist]. —Blahnik, 2002:82 [♂; ♀; to *Otarrha*]. —Botosaneanu, 2002:91 [checklist].

**Distribution.** Jamaica.

***janzeni*** (*Chimarra*) Blahnik and Holzenthal, 1992b:417 [Type locality: Costa Rica, Alajuela, Cerro Campana, Río Bochinche trib., 6 km (air) NW Dos Ríos, 10.945°N, 85.413°W; NMNH; ♂]. —Blahnik, 1998:120 [♂; ♀; to *primula* group].

**Distribution.** Costa Rica.

***jemima*** (*Chimarra*) Blahnik and Holzenthal, 1992b:418 [Type locality: Costa Rica, Alajuela, Cerro Campana, Río Toro, 3.0 km (road) SW Bajos del Toro, 10.204°N, 84.316°W; NMNH; ♂]. —Blahnik, 1998:97 [♂; ♀; to *picea* group]. —Armitage et al., 2015a:3 [distribution]. —Armitage et al., 2015b:3 [checklist]. —Armitage and Cornejo, 2015:191 [checklist].

**Distribution.** Costa Rica, Panama.

***jugescens*** (*Curgia*) Flint, 1998:24 [Type locality: Brazil, E.D.O. Pará, stream at Caverna do Tatjuba, ~22 km SE Altamira; NMNH; ♂]. —Santos and Nessimian, 2009b:23 [distribution]. —Paprocki and França, 2014:76 [checklist].

**Distribution.** Brazil.

***juliae*** (*Curgia*) Flint, 1998:40 [Type locality: Venezuela, E.D.O. Bolivar, Piedra de Virgem, 10 km S of km 88 (at base of La Escalera); NMNH; ♂; ♀].

**Distribution.** Venezuela.

***koki*** (*Otarrha*) Botosaneanu, 1996:11 [Type locality: Dominican Republic, Parque Nacional Armando Bermudez, Arroyo Manuel Estrella nr. Cienaga entrance to park; ZMUA; ♂; ♀]. —Flint and Pérez-Gelabert, 1999:42 [checklist]. —Botosaneanu, 2002:91 [checklist]. —Blahnik, 2002:85 [to *Otarrha*]. —Flint and Sykora, 2004:51 [distribution]. —Pérez-Gelabert, 2008:301 [checklist].

**Distribution.** Dominican Republic.

***kontilos*** (*Chimarrita*) Blahnik, 1997:227 [Type locality: Brazil, Espirito Santo, Caixa d’Agua, Santa Teresa; MZUSP; ♂; ♀; in *simpliciforma* group]. —Blahnik et al., 2004:5 [distribution]. —Paprocki et al., 2004:14 [checklist]. —Dumas et al., 2009:363 [distribution]. —Calor, 2011:323 [checklist]. —Dumas and Nessimian, 2012:22 [checklist]. —Barcelos-Silva et al., 2012:1278 [distribution]. —Paprocki and França, 2014:73 [checklist]. —Vilarino and Calor, 2015b:133 [distribution].

**Distribution.** Brazil.

***laguna*** (*Curgia*) Ross, 1951a:68 [Type locality: Mexico, Lower California [Baja California Sur], Agua Caliente, Cape Region; CAS; ♂]. —Denning, 1964:133 [checklist]. —Bueno-Soria and Flint, 1978:196 [distribution; as *brustia*]. —Holzenthal, 1988c:57 [distribution; as *brustia*]. —Flint, 1998:79 [♂; redescription; distribution; variation; to *laguna* group]. —Chamorro-Lacayo et al., 2007:45 [checklist]. —Bueno-Soria and Barba-Álvarez, 2011:360 [checklist].

—*brustia* Ross, 1959:176 [Type locality: Mexico, Guerrero, Cocula; INHS; ♂]. —Flint, 1998:79 [to synonymy].

—*alamosa* Denning, 1962b:406 [Type locality: Mexico, Sonora, seven miles south of Alamosa; CAS; ♂]. —Bueno-Soria and Flint, 1978:190, 196 [distribution; to synonymy].

**Distribution.** Belize, Costa Rica, Guatemala, Honduras, Mexico, Nicaragua.

***langleyae*** (*Chimarra*) Blahnik, 1998:64 [Type locality: Ecuador, Napo, Lago Agrio; NMNH; ♂; in *bidentata* group].

**Distribution.** Ecuador.

***lata*** (*Chimarra*) Blahnik and Holzenthal, 1992b:421 [Type locality: Costa Rica, Guanacaste, Parque Nacional Guanacaste, ca. 0.7 km N Estación Maritza, 10.96°N, 85.50°W; NMNH; ♂]. —Blahnik, 1998:50 [♂; to *beameri* group]. —Chamorro-Lacayo et al., 2007:45 [checklist].

**Distribution.** Costa Rica, Nicaragua.

***latiforceps*** (*Chimarra*) Blahnik and Holzenthal, 2012:24 [Type locality: Brazil, São Paulo, Parque Estadual de Campos do Jordão, Rio Galharda, 22°41.662'S, 45°27.783'W, el. 1530 m; MZUSP; ♂; ♀; in *simpliciforma* group]. —Paprocki and França, 2014:73 [checklist].

**Distribution.** Brazil.

***limon*** (*Chimarra*) Blahnik, 1998:99 [Type locality: Costa Rica, Limón, Río Barbilla, ca. 8 km W B-Line, 10.067°N, 83.369°W; NMNH; ♂; ♂; in *picea* group].

**Distribution.** Costa Rica.

***lobata*** (*Curgia*) Flint, 1967b:7 [Type locality: Panama, Canal Zone, Rio Agua Salud; NMNH; ♂]. —Aguila, 1992:534 [distribution]. —Flint, 1998:46 [♂; redescription; to *mexicana* group]. —Armitage et al., 2015b:4 [checklist]. —Armitage and Cornejo, 2015:191 [checklist].

**Distribution.** Panama.

***lojaensis*** (*Curgia*) Flint, 1998:28 [Type locality: Ecuador, Pcia. Zamora-Chinchipe, 30 km E Loja; NMNH; ♂; in *margaritae* group].

**Distribution.** Ecuador.

***longiterga*** (*Chimarra*) Blahnik and Holzenthal, 1992b:421 [Type locality: Costa Rica, Puntarenas, Parque Nacional Corcovado, Piedra del Arco, 8.582°N, 83.709°W; NMNH; ♂]. —Blahnik, 1998:30 [♂; ♀; to *amica* group; distribution]. —Armitage et al., 2015b:3 [checklist]. —Armitage and Cornejo, 2015:191 [checklist].

**Distribution.** Costa Rica, Ecuador, Panama.

***macara*** (*Curgia*) Flint, 1998:61 [Type locality: Ecuador, Pcia. Loja, Macará to Catacocha; NMNH; ♂; in *banksi* group].

**Distribution.** Ecuador.

***machadoi*** (*Otarrha*) Camargos, 2016:334 [Type locality: Brazil, Goiás, Pirenópolis, Santuário Vagafogo, Vagafogo stream, 15.824685° S, 48.995421° W, el. 794 m; MZUSP; ♂].

**Distribution.** Brazil.

***machaerophora*** (*Otarrha*) Flint, 1968a:20 [Type locality: Jamaica, St. Andrew, Chestervale, Yallahs River; NMNH; ♂; ♀]. —Flint, 1968b:80 [checklist]. —Blahnik, 2002:85 [♂; ♀; to *Otarrha*]. —Botosaneanu, 2002:91 [checklist].

**Distribution.** Jamaica.

***majuscula*** (*Chimarrita*) Blahnik, 1997:227 [Type locality: Brazil, Rio de Janeiro, Nova Friburgo; MZUSP; ♂; ♀; in *simpliciforma* group]. —Blahnik et al., 2004:5 [distribution]. —Paprocki et al., 2004:14 [checklist]. —Dumas et al., 2009:363 [distribution]. —Calor, 2011:323 [checklist]. —Paprocki and França, 2014:74 [checklist].

**Distribution.** Brazil.

***maldonadoi*** (*incertae sedis*) Flint, 1964a:23 [Type locality: Puerto Rico, Maricao, fish hatchery; NMNH; ♂; ♀]. —Flint, 1968b:80 [checklist]. —Blahnik, 1997:205 [♂; ♀; to *Chimarrita maldonadoi* group]. —Botosaneanu, 2002:90 [checklist]. —Kjer, et al., 2014:352 [*incertae sedis*].

**Distribution.** Puerto Rico.

***margaritae*** (*Curgia*) Flint, 1991:26 [Type locality: Colombia, Dpto. Antioquia, 12 km NW Medellín [road to San Pedro]; NMNH; ♂]. —Flint, 1998:27 [♂; redescription; distribution; to *margaritae* group]. —Muñoz-Quesada, 2000:280 [checklist].

**Distribution.** Colombia, Ecuador.

***maritza*** (*Curgia*) Flint, 1998:65 [Type locality: Costa Rica, Pcia. Guanacaste, Parque Nacional Guanacaste, Rio Tempisquito, Maritza, 10.958°N, 85.497°W; NMNH; ♂; in *banksi* group].

**Distribution.** Costa Rica.

***medioloba*** (*Curgia*) Flint, 1971c:22 [Type locality: Brazil [Edo. Amazonas], Gebeit Endstation Rio Marauia, Bergbach II; NMNH; ♂]. —Flint, 1998:37 [♂; redescription; distribution; to *medioloba* group]. —Paprocki et al., 2004:14 [checklist]. —Paprocki and França, 2014:76 [checklist].

**Distribution.** Brazil, Venezuela.

***merengue*** (*incertae sedis*) Blahnik, 1997:210 [Type locality: Dominican Republic, Dajabon, 1.3 Km S Loma de Cabrera; NMNH; ♂; ♀; in *Chimarrita maldonadoi* group]. —Flint and Pérez-Gelabert, 1999:42 [checklist]. —Botosaneanu, 2002:90 [checklist]. —Flint and Sykora, 2004:51 [distribution]. —Pérez-Gelabert, 2008:301 [checklist]. —Kjer, et al., 2014:352 [*incertae sedis*].

**Distribution.** Dominican Republic.

***mesodonta*** (*Chimarrita*) Vilaniro and Calor, 2015b:123 [Type locality: Brazil, Bahia, Santa Teresinha, Pedra Branca, Serra da Jibóia, 12°51'016"S, 39°28'48"W, el. 679 m; ♂; ♀; *rosalesi* Group].

**Distribution.** Brazil.

***mexicana*** (*Curgia*) (Banks), 1900:259 [Type locality: Mexico, Vera Cruz, Xico; MCZ; ♂; in *Rhyacophila*]. —Banks, 1901:371 [distribution]. —Flint, 1967c:3 [♂; redescription; synonymy]. —Flint, 1967d:166 [distribution]. —Bueno-Soria and Flint, 1978:197 [distribution]. —Aguila, 1992:534 [distribution]. —Flint, 1998:43 [♂; redescription; distribution; to *mexicana* group]. —Bueno-Soria et al., 2007:33 [distribution]. —Bueno-Soria and Barba-Álvarez, 2011:360 [checklist].

—*mexicana* (Ulmer), 1905b:89 [Type locality: Mexico; NMW; ♂; in *Wormaldia*]. —Flint, 1966a:3 [lectotype]. —Flint, 1967c:3 [to synonymy].

**Distribution.** Guatemala, Mexico.

***minca*** (*Curgia*) Flint, 1998:72 [Type locality: Venezuela, Edo. Barinas, 15 km SW Barinitas; NMNH; ♂; in *banksi* group]. —Muñoz-Quesada, 2000:280 [checklist].

**Distribution.** Colombia, Venezuela.

***minga*** (*Curgia*) Flint, 1998:28 [Type locality: Venezuela, Edo. Barinas, 30 km NE Barinitas; NMNH; ♂; in *margaritae* group].

**Distribution.** Venezuela.

***moesta*** (*Curgia*) Banks, 1924:449 [Type locality: Cuba; MCZ; ♂; in *Chimarrha*]. —Flint, 1967c:3 [illustration of erroneously associated ♀]. —Flint, 1968b:80 [checklist]. —Botosaneanu, 1979:44 [distribution]. —Flint, 1996c:16 [checklist]. —Flint, 1998:54 [♂; redescription; correction of erroneously associated abdomen; to *braconoides* group]. —Botosaneanu, 2002:90 [checklist]. —González Lazo et al., 2005:260 [distribution]. —Naranjo López and González Lazo, 2005:149 [checklist].

—*alayoi* Botosaneanu, 1980:96 [Type locality: Cuba, Prov. Pinar del Rio, Arroyo del pinar de Viñales; ZMUA; ♂; ♀]. —Botosaneanu, 1979:44 [*nomen nudum* (name included in checklist); distribution]. —Botosaneanu, 1994b:459 [larva]. —Flint, 1998:54 [to synonymy].

**Distribution.** Cuba.

***morio*** (*Curgia*) Burmeister, 1839:911 [Type locality: Brasilien; ZIUH, now lost; ♀; in *Chimarrha*]. —Fischer, 1961:67 [bibliography]. —Flint, 1998:14 [♂; redescription; variation; distribution; to *morio* group]. —Paprocki et al., 2004:14 [checklist]. —Dumas et al., 2009:363 [distribution]. —Calor, 2011:323 [checklist]. —Dumas and Nessimian, 2012:23 [checklist]. —Paprocki and França, 2014:76 [checklist]. —Vilarino and Calor, 2015b:130 [♂; variation, distribution].

—*martinmoselyi* Botosaneanu, 1980:98 [replacement name for *Chimarra moselyi* Ross, 1956a:50, 71, preoccupied by *Chimarra moselyi* Denning, 1947b:251]. [Type locality: Argentina [sic, recte: Brazil], Petropolis, Rio de Janeiro; BMNH; ♂]. —Flint, 1998:14 [to synonymy].

**Distribution.** Brazil.

***munozi*** (*Chimarra*) Blahnik and Holzenthal, 1992b:424 [Type locality: Costa Rica, Heredia, Parque Nacional Braulio Carillo, Estación Magsasay, Río Peje, 10.402°N, 84.050°W; NMNH; ♂]. —Blahnik, 1998:51 [♂; ♀; to *beameri* group; distribution]. —Armitage et al., 2015b:3 [checklist]. —Armitage and Cornejo, 2015:191 [checklist].

**Distribution.** Costa Rica, Panama.

***mycterophora*** (*Curgia*) Flint, 1998:77 [Type locality: Bolivia, Dpto. La Paz, quebradas del Río Zongo; NMNH; ♂; in *banksi* group].

**Distribution.** Bolivia, Peru.

***nasuta*** (*Curgia*) Flint, 1998:70 [Type locality: Mexico, Edo. Veracruz, Los Tuxtlas area, near Balzapote; NMNH; ♂; in *banksi* group].

**Distribution.** Mexico.

***neblina*** (*Chimarrita*) Blahnik, 1997:213 [Type locality: Venezuela, T. F. Amazonas, Cerro de la Neblina, Camp III; NMNH; ♂; ♀; in *rosalesi* group].

**Distribution.** Venezuela.

***neofimbriata*** (*Curgia*) Flint, 1974c:23 [Type locality: Suriname, Wilhelmina Mountains, trail II km. 12; RNH; ♂]. —Flint, 1998:39 [♂; redescription; distribution; to *medioloba* group].

**Distribution.** Guyana, Suriname.

***nicehuh*** (*Chimarra*) Blahnik and Holzenthal, 2012:11 [Type locality: Venezuela, Trujillo, Quebrada Potrerito, 7.5 km NE Boconó, 9°16.435'N, 70°13.102'W, el. 1530 m; UMSP; ♂; ♀; in *picea* group].

**Distribution.** Venezuela.

***oaxaca*** (*Chimarra*) Blahnik, 1998:121 [Type locality: Mexico, Oaxaca, 8 km S Valle Nacional; NMNH; ♂; in *primula* group].

**Distribution.** Mexico.

***obscura*** (*Chimarra*) (Walker), 1852:121 [Type locality: [Canada], St. Martin’s Falls, Albany River, Hudson’s Bay; BMNH; ♂; in *Beraea* ?]. —Betten and Mosely, 1940:17 [♂; lectotype; in *Chimarrha*]. —Ross, 1944:51 [♂; ♀; larva; to *Chimarra*]. —Blahnik, 1998:76 [♂; ♀; to *obscura* group, distribution]. —Bowles et al., 2007:23 [distribution; biology].

—*plutonis* (Banks), 1911:358 [Type locality: United States, New Jersey-Pennsylvania, Delaware Water Gap; MCZ; ♂; in *Wormaldia*]. —Betten and Mosely, 1940:17, 19 [to synonymy].

—*lucia* Betten, 1934:175 [Type locality: United States, New York; CU; ♂]. —Ross, 1938a:7 [as synonym of *plutonis*].

**Distribution.** Canada, Mexico, U.S.A.

***odonta*** (*Otarrha*) Blahnik, 2002:85 [Type locality: Brazil, São Paulo, Est. Biol. Boraceia, el. 850 m; MZUSP; ♂; ♀]. —Paprocki et al., 2004:14 [checklist]. —Dumas et al., 2009:364 [distribution]. —Dumas et al., 2010:8 [distribution]. —Calor, 2011:323 [checklist]. —Dumas and Nessimian, 2012:23 [checklist]. —Barcelos-Silva et al., 2012:1279 [distribution]. —Paprocki and França, 2014:78 [checklist]. —Vilarino and Calor, 2015b:133 [distribution].

**Distribution.** Brazil.

***onchyrhina*** (*Chimarra*) Blahnik and Holzenthal, 2012:6 [Type locality: Venezuela, Sucre, Península de Paria, Puerto Viejo, “Río el Pozo”, 10°43.073'N, 62°28.569'S, el. 20 m; UMSP; ♂; ♀; in *ortiziana* group].

**Distribution.** Venezuela.

***onima*** (*Chimarra*) Flint, 1991:29 [Type locality: Colombia, Dpto. Antioquia, Quebrada Agua Mala, 34 km NW Medellín [road to San Jerónimo]; NMNH; ♂]. —Blahnik, 1998:100 [♂; ♀; to *picea* group]. —Muñoz-Quesada, 2000:280 [checklist].

**Distribution.** Colombia.

***ortiziana*** (*Chimarra*) Flint, 1967b:6 [Type locality: Mexico, Veracruz, near Huatusco; NMNH; ♂]. —Bueno-Soria and Flint, 1978:196 [distribution]. —Holzenthal, 1988c:56 [distribution]. —Blahnik, 1998:81 [♂; ♀; to *ortiziana* group; distribution]. —Bueno-Soria et al., 2005:75 [distribution]. —Bueno-Soria and Barba-Álvarez, 2011:360 [checklist].

**Distribution.** Belize, Costa Rica, Guatemala, Mexico.

***otuzcoensis*** (*Curgia*) Flint and Reyes, 1991:480 [Type locality: Peru, Dept. La Libertad, Prov. Otuzco, Dist. Sinsicap, Río Sinsicap, Sinsicap; NMNH; ♂]. —Flint, 1998:30 [♂; redescription; distribution; to *otuzcoensis* group].

**Distribution.** Ecuador, Peru.

***ovalis*** (*Chimarra*) Ross, 1959:170 [Type locality: Mexico, Chis.[Chiapas], Salto de Agua; INHS; ♂]. —Bueno-Soria and Flint, 1978:196 [distribution]. —Blahnik, 1998:122 [♂; ♀; to *primula* group]. —Bueno-Soria and Barba-Álvarez, 2011:360 [checklist].

**Distribution.** Guatemala, Mexico.

***pablito*** (*Curgia*) Flint, 1998:48 [Type locality: Costa Rica, Pcia. Cartago, Turrialba; NMNH; ♂; in *mexicana* group]. —Maes, 1999:1191 [checklist]. —Armitage et al., 2015b:4 [checklist]. —Armitage and Cornejo, 2015:191 [checklist].

—*spangleri* Trivette ms., McElravy et al., 1981:152 [distribution]. —Flint, 1998:48 [to synonymy].

**Distribution.** Costa Rica, Ecuador, Nicaragua, Panama.

† ***palaedominicana*** (*incertae sedis*) Wichard, 1983a:142 [Type locality: Dominican Republic; collection Wichard; ♂; in amber]. —Flint and Pérez-Gelabert, 1999:42 [checklist]. —Botosaneanu, 2002:91 [checklist]. —Pérez-Gelabert, 2008:302 [checklist]. —Wichard, 2007a:14 [♂; to *Chimarrita*; key]. —Kjer, et al., 2014:352 [*incertae sedis*].

**Distribution.** Dominican Republic.

† ***palaenova*** (*incertae sedis*) Wichard, 2007a:17 [Type locality: Dominican Republic; SMNS; ♂; in amber; in *Chimarrita*]. —Kjer, et al., 2014:352 [*incertae sedis*].

**Distribution.** Dominican Republic.

***paracreagra*** (*Chimarra*) Blahnik, 1998:101 [Type locality: Ecuador, Pastaza, Puyo (22 km W); NMNH; ♂; ♀; in *picea* group].

**Distribution.** Ecuador.

***parana*** (*Curgia*) Flint, 1972b:227 [Type locality: Argentina, Prov. Misiones, Puerto Rico; NMNH; ♂]. —Flint, 1998:73 [♂;redescription; distribution; to *banksi* group]. —Paprocki et al., 2004:15 [checklist]. —Dumas et al., 2009:364 [distribution]. —Calor, 2011:323 [checklist]. —Barcelos-Silva et al., 2012:1278 [distribution]. —Souza et al., 2013a:8 [distribution]. —Paprocki and França, 2014:76 [checklist].

—*punctulata* (*Curgia*) Flint, 1983a:16 [Type locality: Brazil, Edo. Santa Catarina, Nova Teutonia (27°11'S, 52°23'W); NMNH; ♂]. —Flint, 1998:73 [to synonymy].

**Distribution.** Argentina, Brazil.

***paraortiziana*** (*Chimarra*) Blahnik and Holzenthal, 1992b:426 [Type locality: Costa Rica, Heredia, Estación Biológica La Selva, Quebrada El Salto, 10.427°N, 84.005°W; NMNH; ♂]. —Blahnik, 1998:82 [♂; ♀ to *ortiziana* group; distribution]. —Maes, 1999:1190 [checklist]. —Chamorro-Lacayo et al., 2007:46 [checklist].

**Distribution.** Costa Rica, Nicaragua.

***parene*** (*Otarrha*) Blahnik, 2002:87 [Type locality: Peru, Parene, El Campamiento; CU; ♂].

**Distribution.** Peru.

***paria*** (*Curgia*) Flint, 1998:34 [Type locality: Venezuela, Edo. Sucre, Río la Viuda, Uquire, Peninsula de Paria, 10°42.830'N, 61°57.661'W; NMNH; ♂; in *canoaba* group].

**Distribution.** Venezuela.

***parilis*** (*Otarrha*) Blahnik, 2002:87 [Type locality: Peru, Madre de Dios, Amazonia Lodge, Toma del Agua (stream), l2°52.22'S, 7l°22.56'W, el. 414 m; MJP; ♂; ♀].

**Distribution.** Peru.

***particeps*** (*Otarrha*) Blahnik, 2002:89 [Type locality: Peru, Huanuco, Tingo Maria, el. 672 m; NMNH; ♂; ♀].

**Distribution.** Peru.

***patosa*** (*Otarrha*) Ross, 1956a:71 [Type locality: Peru, [Dpto.] Cuzco, [Prov.] Paucartambo, Cosnipata Valley; INHS; ♂]. —Flint, 1996b:388 [distribution]. —Blahnik, 2002:89 [♂; ♀; to *Otarrha*].

**Distribution.** Peru.

***paucispina*** (*Curgia*) Santos and Nessimian, 2009b:23 [Type locality: Brazil, Amazonas State, Manaus, Tributary of the Rio Cuieiras, 2°33'46.4”5, 60°19'03.4"W; INPA; ♂]. —Paprocki and França, 2014:76 [checklist].

**Distribution.** Brazil.

***peineta*** (*Chimarra*) Blahnik and Holzenthal, 1992b:428 [Type locality: Costa Rica, Guanacaste, Parque Nacional Guanacaste, El Hacha, Quebrada Alcornoque, 10.009°N, 85.577°W; NMNH; ♂]. —Blahnik, 1998:84 [♂; ♀; to *ortiziana* group; distribution]. —Maes, 1999:1191 [checklist]. —Chamorro-Lacayo et al., 2007:46 [checklist]. —Armitage et al., 2015b:3 [checklist]. —Armitage and Cornejo, 2015:191 [checklist].

**Distribution.** Costa Rica, Ecuador, Nicaragua, Panama.

***pelaezi*** (*Chimarra*) Bueno-Soria, 1985:19 [Type locality: Mexico, Guerrero, Acahuizotla, 17 km S de Chilpancingo; IBUNAM; ♂]. —Blahnik, 1998:123 [♂; to *primula* group].

**Distribution.** Mexico.

***persimilis*** (*Curgia*) Banks, 1920:360 [Type locality: Ecuador, Quevedo; MCZ; ♂; in *Chimarrha*]. —Flint, 1967c:4 [♂; lectotype]. —Maes and Flint, 1988:3 [distribution]. —Holzenthal, 1988c:57 [distribution]. —Aguila, 1992:534 [distribution]. —Flint, 1998:84 [♂; redescription; distribution; to *immaculata* group]. —Maes, 1999:1191 [checklist]. —Chamorro-Lacayo et al., 2007:46 [checklist]. —Armitage et al., 2015b:4 [checklist]. —Armitage and Cornejo, 2015:191 [checklist].

**Distribution.** Costa Rica, Ecuador, Honduras, Nicaragua, Panama, Peru.

***peruana*** (*Otarrha*) Blahnik, 2002:91 [Type locality: Peru, Aina, am back gestreift, el. 1400 m; ZMUH; ♂].

**Distribution.** Peru.

***peruviana*** (*Curgia*) Flint, 1998:71 [Type locality: Ecuador, Pcia. Napo, Río Jandachi, 30 km N Tena; NMNH; ♂; in *banksi* group].

**Distribution.** Ecuador, Peru.

***petersorum*** (*Curgia*) Flint, 1998:21 [Type locality: Brazil, Edo. Paraná, Rio Marumbi, Marumbi; MZUSP; ♂; in *morio* group]. —Paprocki et al., 2004:15 [checklist]. —Paprocki and França, 2014:77 [checklist].

**Distribution.** Brazil.

***petricola*** (*Curgia*) Flint, 1998:21 [Type locality: Brazil, Edo. Rio de Janeiro, Petrópolis; BMNH; ♂; in *morio* group]. —Paprocki et al., 2004:15 [checklist]. —Dumas et al., 2009:364 [distribution]. —Paprocki and França, 2014:77 [checklist].

**Distribution.** Brazil.

***peytoni*** (*Curgia*) Flint, 1998:85 [Type locality: Venezuela, Edo. Barinas, Puente Parangula, 8 km S Barinitas; NMNH; ♂; in *immaculata* group]. —Muñoz-Quesada, 2000:280 [checklist].

**Distribution.** Colombia, Venezuela.

***phthanorossi*** (*Otarrha*) Blahnik, 2002:91 [Type locality: Colombia, Chocó, km 130, 86 km E Quibdo; ZMUH; ♂; ♀].

**Distribution.** Colombia.

***picea*** (*Chimarra*) Navás, 1924c:79 [Type locality: Costa Rica; MNHNP; ♀; in *Chimarrha*]. —Holzenthal, 1988c:56 [distribution]. —Blahnik, 1998:102 [♂; ♀; to *picea* group; distribution]. —Chamorro-Lacayo et al., 2007:46 [checklist].

**Distribution.** Costa Rica, Nicaragua.

***piliferosa*** (*Curgia*) Flint, 1998:63 [Type locality: Bolivia, Yungas de la Paz, Río San Pedro; NMNH; ♂; in *banksi* group].

**Distribution.** Bolivia, Peru.

***piraya*** (*Curgia*) Flint, 1983a:15 [Type locality: Argentina, Pcia. Misiones, Arroyo Piray Mini, Rt.17 W Dos Hermanas; NMNH; ♂]. —Flint, 1998:72 [♂; redescription; to *banksi* group].

**Distribution.** Argentina.

***platyrhina*** (*Chimarra*) Flint, 1981a:13 [Type locality: Venezuela, Aragua, Maracay, Río Limón, Estación Pisicultura; NMNH; ♂; ♀]. —Flint, 1991:28 [♂; distribution]. —Blahnik, 1998:85 [♂; ♀; to *ortiziana* group; distribution]. —Muñoz-Quesada, 2000:280 [checklist].

**Distribution.** Venezuela.

***plaumanni*** (*Curgia*) Flint, 1983a:19 [Type locality: Brazil, Edo. Santa Catarina, Nova Teutonia (27°11'S, 52°23'W); NMNH; ♂]. —Angrisano, 1997b:56 [distribution]. —Flint, 1998:18 [♂; redescription; variation; to *morio* group]. —Paprocki et al., 2004:15 [checklist]. —Barcelos-Silva et al., 2012:1278 [distribution]. —Manzo et al., 2014:166 [distribution]. —Paprocki and França, 2014:77 [checklist].

**Distribution.** Argentina, Brazil, Uruguay.

***pollex*** (*Chimarra*) Blahnik and Holzenthal, 1992b:430 [Type locality: Costa Rica, Alajuela, Reserva Forestal San Ramón, Río San Lorencito & tribs, 10.216°N, 84.607°W; NMNH; ♂]. —Blahnik, 1998:86 [♂; ♀ to *ortiziana* group]. —Maes, 1999:1191 [checklist]. —Chamorro-Lacayo et al., 2007:46 [checklist]. —Armitage et al., 2015b:3 [checklist]. —Armitage and Cornejo, 2015:191 [checklist].

**Distribution.** Costa Rica, Nicaragua, Panama.

***poolei*** (*Chimarra*) Flint, 1981a:13 [Type locality: Venezuela, Aragua, Dos Riitos, 6 km N Rancho Grande; NMNH; ♂]. —Blahnik, 1998:111 [♂; ♀; to *poolei* group].

**Distribution.** Venezuela.

***potosi*** (*Chimarra*) Blahnik, 1998:124 [Type locality: Mexico, San Luis Potosí, 4 mi S Tamazunchale; NMNH; ♂; in *primula* group].

**Distribution.** Mexico.

***primula*** (*Chimarra*) Denning, 1950:100 [Type locality: United States, Arizona, Oak Creek Canyon; CAS; ♂]. —Bueno-Soria and Flint, 1978:196 [distribution; as *volenta*]. —Blahnik, 1998:125 [♂; ♀; to *primula* group, distribution]. —Blinn and Ruiter, 2006:334 [biology]. —Bueno-Soria et al., 2007:33 [distribution]. —Blinn and Ruiter, 2009b:187 [phenology, distribution]. —Ruiter and Blinn, 2009:5 [♀].

—*volenta* (*Chimarra*) Ross, 1959:170 [Type locality: presumably collected in Mexico; INHS; ♂]. —Blahnik, 1998:125 [to synonymy].

**Distribution.** Mexico, U.S.A.

***prolata*** (*Chimarrita*) Blahnik, 1997:214 [Type locality: Ecuador, Pastaza, Puyo (27 km N), Estación Fluviometrica; NMNH; ♂; ♀; in *rosalesi* group].

**Distribution.** Ecuador.

***protuberans*** (*Chimarra*) Blahnik, 1998:64 [Type locality: Peru, Madre de Dios, Manu, Erika (near Salvacion); NMNH; ♂; ♀; in *bidentata* group].

**Distribution.** Peru.

***puertoricensis*** (*Otarrha*) Flint, 1964a:23 [Type locality: Puerto Rico, Maricao, fish hatchery; NMNH; ♂; ♂; larva]. —Flint, 1968b:80 [checklist]. —Blahnik, 2002:95 [♂; ♀; to *Otarrha*]. —Botosaneanu, 2002:91 [checklist].

**Distribution.** Puerto Rico.

***pulchra*** (*Curgia*) Hagen, 1861:298 [Type locality: Cuba; MCZ; ♂; in *Chimarrha*]. —Ross, 1952:32 [♀; lectotype]. —Flint, 1967c:3 [♂; synonymy]. —Flint, 1968b:80 [checklist]. —Botosaneanu, 1979:44 [distribution]. —Kumanski, 1987:7 [♂; ♀; distribution]. —Botosaneanu, 1994b:459 [larva]. —Flint, 1996c:16 [checklist]. —Flint, 1998:56 [♂; redescription; variation; to *pulchra* group]. —Botosaneanu, 2002:90 [checklist]. —Naranjo López and González Lazo, 2005:149 [checklist]. —López del Castillo et al., 2007:171 [distribution; seasonal abundance].

—*fraterna* Banks, 1924:449 [Type locality: Cuba; MCZ; ♂; in *Chimarrha*]. —Flint, 1967c:4 [lectotype; to synonymy].

**Distribution.** Cuba.

***pumila*** (*Chimarra*) (Banks), 1920:359 [Type locality: Ecuador, Quevedo; MCZ; ♀; in *Chimarrha*]. —Flint, 1967c:4 [♀; lectotype]. —Blahnik, 1998:134 [♀; incertae sedis].

**Distribution.** Ecuador.

***purisca*** (*Curgia*) Flint, 1998:65 [Type locality: Costa Rica, Pcia. San José, P. N. Braulio Carillo, 6.2 km NE adm. Build., 10.09°N, 83.97°; NMNH; ♂; in *banksi* group]. —Armitage et al., 2015a:3 [distribution]. —Armitage et al., 2015b:4 [checklist]. —Armitage and Cornejo, 2015:191 [checklist].

**Distribution.** Costa Rica, Panama.

***pusilla*** (*Chimarrita*) Blahnik, 1997:213 [Type locality: Venezuela, T. F. Amazonas, Puerto Ayacucho (40 km S), El Tobogán; NMNH; ♂; ♀; in *rosalesi* group].

**Distribution.** Venezuela.

***puya*** (*Curgia*) Flint, 1998:31 [Type locality: Ecuador, Pcia. Pastaza, 27 km N Puyo, Estación Fluviometrico; NMNH; ♂; in *fernandezi* group].

**Distribution.** Ecuador.

***pylaea*** (*Chimarra*) Denning, 1941:84 [Type locality: Mexico, [Nuevo Leon], Monterey; UMSP; ♂; in *Chimarrha*]. —Bueno-Soria and Flint, 1978:196 [distribution]. —Blahnik, 1998:35 [♂; ♀; to *angustipennis* group; distribution].

**Distribution.** Mexico.

***quadratiterga*** (*Chimarra*) Blahnik, 1998:104 [Type locality: Ecuador, Zamora-Chinchipe, 6 km E Zumbi; NMNH; ♂; in *picea* group].

**Distribution.** Ecuador.

***quadrifurcata*** (*Otarrha*) Botosaneanu, 1994a:43 [Type locality: Guadeloupe, rivière de la Grande Anse, à “Moscou”; ZMUA; ♂; ♀]. —Botosaneanu, 2000:256 [distribution]. —Botosaneanu, 2002:91 [checklist]. —Blahnik, 2002:95 [♂; ♀; to *Otarrha*]. —Botosaneanu and Thomas, 2005:55 [checklist].

**Distribution.** Guadeloupe.

***quaternaria*** (*Curgia*) Flint, 1971c:23 [Type locality: Brazil [Edo. Amazonas], Gebeit Endstation Rio Marauia, Bergbach II; NMNH; ♂]. —Flint, 1998:40 [♂; redescription; to *medioloba* group]. —Paprocki et al., 2004:15 [checklist]. —Paprocki and França, 2014:77 [checklist].

**Distribution.** Brazil.

***quina*** (*Curgia*) Flint, 1998:54 [Type locality: Cuba, Holguín Province, Pinares de Mayari; NMNH; ♂; in *braconoides* group]. —Flint, 1996c:16 [checklist, as “in press”]. —Botosaneanu, 2002:90 [checklist]. —Naranjo López and González Lazo, 2005:149 [checklist].

**Distribution.** Cuba.

***quitacalzon*** (*Chimarra*) Blahnik, 1998:65 [Type locality: Peru, Cuzco, Paucartambo to Pilcopata rd., Quebrada Quitacalzón at Puente Quitacalzón; NMNH; ♂; ♂; in *bidentata* group].

**Distribution.** Peru.

***rafita*** (*Chimarra*) Blahnik, 1998:66 [Type locality: Ecuador, Pastaza, Puyo (27 km N), Estación Fluviometrico; NMNH; ♂; ♀; in *bidentata* group].

**Distribution.** Ecuador.

***redonda*** (*Otarrha*) Blahnik, 2002:98 [Type locality: Dominican Republic, La Palma, 12 km E of El Río; NMNH; ♂]. —Botosaneanu, 1996:11 [as undescribed species]. —Flint and Pérez-Gelabert, 1999:42 [checklist]. —Botosaneanu, 2002:91 [checklist]. —Flint and Sykora, 2004:51 [distribution]. —Pérez-Gelabert, 2008:301 [checklist].

**Distribution.** Dominican Republic.

† ***resinae*** (*incertae sedis*) Wichard, 1983a:141 [Type locality: Dominican Republic; collection Wichard; ♂; in amber]. —Flint and Pérez-Gelabert, 1999:42 [checklist]. —Botosaneanu, 2002:91 [checklist]. —Wichard, 2007a:14 [♂; to *Chimarrita*; key]. —Pérez-Gelabert, 2008:302 [checklist]. —Kjer, et al., 2014:352 [*incertae sedis*].

**Distribution.** Dominican Republic.

***retrorsa*** (*Otarrha*) Flint, 1974c:26 [Type locality: Suriname, Brownsberg, top; RNH; ♂]. —Blahnik, 2002:98 [♂; to *Otarrha*].

**Distribution.** Suriname.

***rhamphodes*** (*Chimarra*) Blahnik, 1998:67 [Type locality: Peru, Madre de Dios, Manu, Erika (near Salvación); NMNH; ♂; in *bidentata* group].

**Distribution.** Peru.

***ridleyi*** (*Chimarra*) Denning, 1941:83 [Type locality: Mexico, Nuevo Leon, Villa Allende; UMSP; ♂; in *Chimarrha*]. —Denning, 1962b:406 [clasper ♂]. —Bueno-Soria and Flint, 1978:196 [distribution]. —Holzenthal, 1988c:56 [distribution]. —Blahnik, 1998:36 [♂; ♀ *angustipennis* group; distribution]. —Baumgardner and Bowles, 2005:11 [distribution]. —Bueno-Soria et al., 2005:75 [distribution]. —Blinn and Ruiter, 2006:334 [biology]. —Bueno-Soria et al., 2007:33 [distribution]. —Chamorro-Lacayo et al., 2007:46 [checklist]. —Blinn and Ruiter, 2009a:305 [biology]. —Blinn and Ruiter, 2009b:187 [phenology, distribution]. —Ruiter and Blinn, 2009:5 [♀]. —Bueno-Soria and Barba-Álvarez, 2011:360 [checklist].

**Distribution.** Costa Rica, El Salvador, Guatemala, Honduras, Mexico, Nicaragua, U.S.A.

***rosalesi*** (*Chimarrita*) Flint, 1981a:12 [Type locality: Venezuela, Aragua, Dos Riitos, 6 km N Rancho Grande; NMNH; ♂]. —Blahnik, 1997:217 [♂; ♀; distribution; to *rosalesi* group].

**Distribution.** Venezuela.

***rossi*** (*Otarrha*) Bueno-Soria, 1985:22 [Type locality: Costa Rica, Corcovado, Estacion Sirena; IBUNAM; ♂]. —Holzenthal, 1988c:56 [distribution]. —Blahnik, 2002:99 [♂; ♀; distribution; to *Otarrha*]. —Chamorro-Lacayo et al., 2007:46 [checklist]. —Armitage et al., 2015b:4 [checklist]. —Armitage and Cornejo, 2015:192 [checklist].

**Distribution.** Costa Rica, Nicaragua, Panama.

***sarophora*** (*Curgia*) Flint, 1998:60 [Type locality: Panama, Pcia. Colón, Canal Zone, Río Agua Salud, Pipeline Road; NMNH; ♂; in *banksi* group]. —Chamorro-Lacayo et al., 2007:46 [checklist]. —Armitage et al., 2015b:4 [checklist]. —Armitage and Cornejo, 2015:191 [checklist].

**Distribution.** Nicaragua, Panama.

***schiza*** (*Chimarra*) Ross, 1959:172 [Type locality: Mexico, Oax[aca], Huajuapan; INHS; ♂]. —Bueno-Soria and Flint, 1978:196 [distribution]. —Blahnik, 1998:130 [♂; ♀ to *utahensis* group]. —Ruiter and Blinn, 2009:5 [♀; distribution].

**Distribution.** Mexico, U.S.A.

***scopula*** (*Curgia*) Flint, 1974c:18 [Type locality: Suriname, Saramacca River, Wedeboh Rapids; RNH; ♂]. —Flint, 1998:74 [♂; redescription; distribution; to *banksi* group].

**Distribution.** Suriname, Venezuela.

***scopuloides*** (*Curgia*) Flint, 1974c:19 [Type locality: Suriname, Tapanahoni River, Gwé Rapids; RNH; ♂]. —Flint, 1992d:65 [distribution]. —Flint, 1998:76 [♂; redescription; distribution; to *banksi* group]. —Paprocki et al., 2004:15 [checklist]. —Paprocki and França, 2014:77 [checklist].

—*catarinensis* (*Curgia*) Flint, 1983a:19 [Type locality: Brazil, Edo. Santa Catarina, Nova Teutonia (27°11'S, 52°23'W); NMNH; ♂]. —Flint, 1998:76 [to synonymy].

**Distribution.** Argentina, Brazil, Suriname.

***securigera*** (*Curgia*) Flint, 1998:86 [Type locality: Venezuela, Edo. Barinas, Río Santo Domingo, Barinas; NMNH; ♂; in *immcaculata* group].

**Distribution.** Venezuela.

***sensillata*** (*Otarrha*) Flint, 1981a:12 [Type locality: Venezuela, Aragua, 4 km S Rancho Grande; NMNH; ♂]. —Blahnik, 2002:102 [♂; to *Otarrha*; in *Chimarra sensillata* species complex].

**Distribution.** Venezuela.

***septemlobata*** (*Otarrha*) Flint, 1991:28 [Type locality: Colombia, Dpto. Antioquia, Quebrada La Iguana, 17 km NW Medellín [road to San Pedro]; NMNH; ♂]. —Muñoz-Quesada, 2000:280 [checklist]. —Blahnik, 2002:102 [♂; ♀; to *Otarrha*].

**Distribution.** Colombia.

***septifera*** (*Otarrha*) Flint, 1974c:25 [Type locality: Suriname, Brownsberg, mountain creek near Golddigers camp; RNH; ♂]. —Blahnik, 2002:102 [♂; to *Otarrha*].

**Distribution.** Suriname.

***setosa*** (*Chimarra*) Ross, 1959:175 [Type locality: Mexico, Chis.[Chiapas], Finca Vergel; INHS; ♂]. —Bueno-Soria and Flint, 1978:196 [distribution]. —Blahnik, 1998:126 [♂; ♀; to *setosa* group; distribution]. —Maes, 1999:1191 [checklist]. —Bueno-Soria et al., 2005:75 [distribution]. —Bueno-Soria et al., 2007:33 [distribution]. —Chamorro-Lacayo et al., 2007:46 [checklist]. —Bueno-Soria and Barba-Álvarez, 2011:360 [checklist].

**Distribution.** Guatemala, Mexico, Nicaragua.

***simpliciforma*** (*Chimarrita*) Flint, 1971c:23 [Type locality: Brazil [Edo. Amazonas], Reserva Ducke, Manaus; IRSNB; ♂]. —Flint, 1974c:29 [♂; distribution]. —Blahnik, 1997:230 [♂; ♀; distribution; to *simpliciforma* group]. —Paprocki et al., 2004:14 [checklist]. —Paprocki and França, 2014:74 [checklist].

**Distribution.** Brazil, Guyana, Suriname, Venezuela.

***solisi*** (*Chimarra*) Blahnik and Holzenthal, 1992b:433 [Type locality: Costa Rica, Heredia, Rara Avis Biol. Station, Quebrada Chiquiza, 10.229°N, 84.032°W; NMNH; ♂]. —Blahnik, 1998:88 [♂; ♀; to *ortiziana* group]. —Maes, 1999:1191 [checklist]. —Chamorro-Lacayo et al., 2007:46 [checklist].

**Distribution.** Costa Rica, Nicaragua.

***soroa*** (*Otarrha*) Blahnik and Holzenthal, 2012:29 [Type locality: Cuba, Pinar del Río, La Caridad, 2 km NW Soroa, 22°48.6'N, 83°01.2'W, el. 220 m; NMNH; ♂; ♀].

**Distribution.** Cuba.

***spangleri*** (*Chimarra*) Bueno-Soria, 1985:16 [Type locality: Costa Rica, Corcovado, Estacion Sirena; IBUNAM; ♂]. —Holzenthal, 1988c:56 [distribution]. —Botosaneanu and Sakal, 1992:202 [distribution]. —Botosaneanu and Alkins-Koo, 1993:30 [♂; distribution]. —Blahnik, 1998:112 [♂; ♀ to *poolei* group; distribution]. —Armitage et al., 2015b:3 [checklist]. —Armitage and Cornejo, 2015:191 [checklist].

**Distribution.** Costa Rica, Panama, Trinidad.

***spatulata*** (*Curgia*) Ross, 1959:176 [Type locality: Mexico, Chis. [Chiapas], Finca Vergel; INHS; ♂]. —Bueno-Soria and Flint, 1978:197 [distribution]. —Maes and Flint, 1988:3 [distribution]. —Holzenthal, 1988c:57 [distribution]. —Aguila, 1992:534 [distribution]. —Flint, 1998:68 [♂; redescription; distribution; to *banksi* group]. —Maes, 1999:1192 [checklist]. —Bueno-Soria et al., 2005:75 [distribution]. —Chamorro-Lacayo et al., 2007:46 [checklist]. —Bueno-Soria and Barba-Álvarez, 2011:360 [checklist]. —Armitage et al., 2015b:4 [checklist]. —Armitage and Cornejo, 2015:191 [checklist].

**Distribution.** Costa Rica, Mexico, Nicaragua, Panama.

***spinulifera baoruco*** (*Otarrha*) Flint and Sykora, 2004:51 [Type locality: Dominican Republic, Barahona Province, San Rafael, 8.3 km S Baoruco, 18°01.9'N, 71°08.4'W, el. 30 m; NMNH; ♂; ♀]. —Botosaneanu, 2002:91 [checklist, as unpublished species]. —Pérez-Gelabert, 2008:301 [checklist].

**Distribution.** Dominican Republic.

***spinulifera galalcha*** (*Otarrha*) Botosaneanu, 1996:11 [Type locality: Dominican Republic, Cordillera Septentrional, Arroyo Los Guineos, tributary of Rio Nagua; ZMUA; ♂; ♀]. —Botosaneanu, 2002:91 [checklist]. —Flint and Pérez-Gelabert, 1999:42 [checklist]. —Blahnik, 2002:107 [♂; ♀; to *Otarrha*]. —Flint and Sykora, 2004:51 [distribution]. —Pérez-Gelabert, 2008:301 [checklist].

**Distribution.** Dominican Republic.

***spinulifera spinulifera*** (*Otarrha*) Flint, 1968c:151 [Type locality: Haiti, Roche Croix, Mt. La Hotte; MCZ; ♂]. —Flint, 1968b:80 [checklist]. —Botosaneanu, 1996:12 [distribution]. —Flint and Pérez-Gelabert, 1999:42 [checklist]. —Blahnik, 2002:107 [♂; to *Otarrha*]. —Botosaneanu, 2002:91 [checklist]. —Flint and Sykora, 2004:51 [distribution]. —Pérez-Gelabert, 2008:301 [checklist].

**Distribution.** Haiti.

***straminea*** (*Curgia*) Flint, 1998:33 [Type locality: Venezuela, Edo. Mérida, 10 km E Santo Domingo; NMNH; ♂; in *canoaba* group].

**Distribution.** Venezuela.

***strongyla*** (*Chimarra*) Blahnik, 1998:68 [Type locality: Ecuador, Pichincha, Nanegal; NMNH; ♂; in *bidentata* group].

**Distribution.** Ecuador.

† ***succini*** (*incertae sedis*) Wichard, 1983b:4 [Type locality: Dominican Republic; SMNS; ♂; in amber]. —Flint and Pérez-Gelabert, 1999:43 [checklist]. —Botosaneanu, 2002:91 [checklist]. —Wichard, 2007a:15 [♂; to *Chimarrita*; key]. —Pérez-Gelabert, 2008:302 [checklist]. —Kjer, et al., 2014:352 [*incertae sedis*].

**Distribution.** Dominican Republic.

***sunima*** (*Chimarra*) Blahnik and Holzenthal, 2012:13 [Type locality: Colombia Valle, Municipio de Buenaventura, Río Escalerete, frente a casa de “Acua Valle”, ca. 15 km SE Cordoba, 3°49'38"N, 76°52'15"W, el. 200 m; UMSP; ♂; ♀; in *picea* group].

**Distribution.** Colombia.

***tachuela*** (*Otarrha*) Blahnik, 2002:108 [Type locality: Venezuela, Merida, La Campana, 12 km SE Santo Domingo; NMNH; ♂; ♀; in *Chimarra sensillata* species complex].

**Distribution.** Venezuela.

***tamba*** (*Curgia*) Flint, 1998:76 [Type locality: Peru, Dpto. Cusco, Pcia. Paucartambo, streamlet 50 m E Quitacalzón at km 164 (13°01.6'S, 71°30.0'W), 32 km NW Pilcopata; NMNH; ♂; in *banksi* group].

**Distribution.** Peru.

***tapanti*** (*Chimarra*) Blahnik, 1998:105 [Type locality: Costa Rica, Cartago, Reserva Tapantí, Quebrada Palmitos & falls, 9.72°N, 83.78°W; NMNH; ♂; ♀; in *picea* group].

**Distribution.** Costa Rica.

***teresae*** (*Curgia*) Flint, 1998:77 [Type locality: Brazil, Edo. Espírito Santo, Fazenda Santa Clara, 15 km SE Santa Teresa; MZUSP; ♂; in *banksi* group]. —Blahnik et al., 2004:5 [distribution]. —Paprocki et al., 2004:15 [checklist]. —Dumas et al., 2009:364 [distribution]. —Calor, 2011:323 [checklist]. —Barcelos-Silva et al., 2012:1279 [distribution]. —Paprocki and França, 2014:77 [checklist].

**Distribution.** Brazil.

***texana*** (*Curgia*) Banks, 1920:360 [Type locality: United States, Texas, San Antonio; MCZ; ♀; in *Chimarrha*]. —Bueno-Soria and Flint, 1978:197 [distribution]. —Flint, 1998:81 [♂; redescription; distribution; to *laguna* group]. —Bowles et al., 2007:23 [distribution; biology]. —Ruiter and Blinn, 2009:5 [♀].

—*betteni* Denning, 1941:82 [Type locality: Mexico, Nuevo Leon, Villa Allende; UMSP; ♂; in *Chimarrha*]. —Edwards and Arnold, 1961:406 [larva; pupa]. —Bueno-Soria and Flint, 1978:190, 197 [to synonymy].

**Distribution.** Mexico, U.S.A.

***tortuosa*** (*Chimarrita*) Blahnik, 1997:232 [Type locality: Brazil, Amazonas, Am. 010 km 246, 20 km W Itacoatiara; MZUSP; ♂; ♂; in *simpliciforma* group]. —Paprocki et al., 2004:14 [checklist]. —Paprocki and França, 2014:74 [checklist].

**Distribution.** Brazil.

***truncatiloba*** (*Curgia*) Flint, 1974c:21 [Type locality: Suriname, Nassau Mountains, km. 16.4; RNH; ♂]. —Flint, 1998:37 [♂; redescription; to *medioloba* group].

**Distribution.** Suriname.

***tucuna*** (*Curgia*) Flint, 1998:24 [Type locality: Brazil, Edo. Amazonas, Igarapé Tucunaré, 75 km W Itacoatiara; MZUSP; ♂; in *tucuna* group]. —Paprocki et al., 2004:15 [checklist]. —Paprocki and França, 2014:77 [checklist].

**Distribution.** Brazil.

***uara*** (*Chimarra*) Flint, 1971c:24 [Type locality: Brazil [Edo. Amazonas], Rio Marauia, Endstation; NMNH; ♂]. —Flint, 1974c:27 [♂; distribution]. —Blahnik, 1998:114 [♂; ♀ to *poolei* group; distribution]. —Blahnik et al., 2004:5 [distribution]. —Paprocki et al., 2004:14 [checklist]. —Dumas et al., 2010:8 [distribution]. —Souza et al., 2013a:7 [distribution]. —Paprocki and França, 2014:72 [checklist].

**Distribution.** Brazil, Guyana, Suriname, Venezuela.

***usitatissima*** (*incertae sedis*) Flint, 1971c:24 [Type locality: Brazil [Edo. Amazonas], Rio Branquinho, bei Cachoeira; NMNH; ♂]. — Flint, 1974c:29 [♂; distribution]. —Blahnik, 2002:119 [♂; ♀]. —Paprocki et al., 2004:15 [checklist]. —Paprocki and França, 2014:78 [checklist].

**Distribution.** Brazil, Suriname.

***utahensis*** (*Chimarra*) Ross, 1938:134 [Type locality: United States, Utah, Gandy; INHS; ♂]. —Ross, 1951a:67 [distribution]. —Denning, 1964:133 [checklist]. —Flint, 1967d:166 [distribution]. —Bueno-Soria and Flint, 1978:196 [distribution]. —Blahnik, 1998:131 [♂; ♀ to *utahensis* group; distribution]. —Blahnik, 2002:119 [to *incertae sedis*]. —Baumgardner and Bowles, 2005:11 [distribution]. —Blinn and Ruiter, 2006:334 [biology]. —Blinn and Ruiter, 2009a:303 [biology]. —Blinn and Ruiter, 2009b:187 [phenology, distribution]. —Ruiter and Blinn, 2009:5 [♀].

**Distribution.** Mexico, U.S.A.

***utra*** (*Chimarra*) Blahnik, 1998:89 [Type locality: Ecuador, Pastaza, Puyo (5 km E); NMNH; ♂; ♀; in *ortiziana* group].

**Distribution.** Ecuador.

***villalobosi*** (*Chimarra*) Bueno-Soria, 1985:17 [Type locality: Costa Rica, Corcovado, Estacion Sirena; IBUNAM; ♂]. —Holzenthal, 1988c:57 [distribution]. —Blahnik, 1998:90 [♂; ♀; to *ortiziana* group; distribution]. —Maes, 1999:1191 [checklist]. —Chamorro-Lacayo et al., 2007:46 [checklist]. —Armitage et al., 2015b:3 [checklist]. —Armitage and Cornejo, 2015:191 [checklist].

**Distribution.** Costa Rica, Nicaragua, Panama.

***virgencita*** (*Chimarra*) Blahnik and Holzenthal, 1992b:433 [Type locality: Costa Rica, Alajuela, Quebrada Virgencita, 10.2 km S Bajos del Toro, 10.168°N, 84.326°W; NMNH; ♂]. —Blahnik, 1998:133 [♂; ♀; to *virgencita* group].

**Distribution.** Costa Rica.

† ***weitschati*** (*incertae sedis*) Wichard, 1983a:139 [Type locality: Dominican Republic; collection Wichard; ♂; in amber—Flint and Pérez-Gelabert, 1999:43 [checklist]. —Botosaneanu, 2002:91 [checklist]. —Wichard, 2007a:14 [♂; to *Chimarrita*; key]. —Pérez-Gelabert, 2008:302 [checklist]. —Kjer, et al., 2014:352 [*incertae sedis*].

**Distribution.** Dominican Republic.

***wilcuma*** (*Chimarra*) Blahnik, 1998:91 [Type locality: Venezuela, Zulia, Parque Nacional Perija, Río Negro in Toromo; NMNH; ♂; ♀; in *ortiziana* group]. —Muñoz-Quesada, 2000:280 [checklist].

**Distribution.** Colombia, Venezuela.

***wilsoni*** (*Curgia*) Flint, 1967b:8 [Type locality: Costa Rica, Las Cruces near San Vito; NMNH; ♂]. —Holzenthal, 1988c:57 [distribution]. —Aguila, 1992:534 [distribution]. —Flint, 1998:46 [♂; redescription; distribution; to *mexicana* group]. —Armitage et al., 2015b:4 [checklist]. —Armitage and Cornejo, 2015:192 [checklist].

**Distribution.** Costa Rica, Panama.

***woldai*** (*Chimarra*) Blahnik, 1998:62 [Type locality: Panama, Panama, Barro Colorado Island, Snyder-Molino trail, marker 3; NMNH; ♂; in *bidens* group]. —Armitage et al., 2015b:4 [checklist]. —Armitage and Cornejo, 2015:191 [checklist].

**Distribution.** Panama.

***xingu*** (*Chimarrita*) Blahnik, 1997:232 [Type locality: Brazil, Pará, Rio Xingu Camp, 52°22'W, 3°39'S, ca. 60 km S Altamira, Igarape Jabuti; MZUSP; ♂; ♀; in *simpliciforma* group]. —Paprocki et al., 2004:14 [checklist]. —Paprocki and França, 2014:74 [checklist].

**Distribution.** Brazil.

***xus*** (*Chimarra*) Blahnik, 1998:115 [Type locality: Ecuador, Pastaza, Puyo (27 km N), Estación Fluviometrico; NMNH; ♂; in *poolei* group]. —Muñoz-Quesada, 2000:280 [checklist].

**Distribution.** Colombia, Ecuador.

***yanura*** (*Chimarra*) Blahnik and Holzenthal, 1992b:436 [Type locality: Costa Rica, Limón, Parque Nacional Braulio Carillo, Quebrada González, 10.160°N, 83.939°W; NMNH; ♂]. —Blahnik, 1998:52 [♂; ♀ to *beameri* group].

**Distribution.** Costa Rica.

***ypsilon*** (*Curgia*) Flint, 1983a:17 [Type locality: Argentina, Pcia. Misiones, Puerto Libertad; NMNH; ♂]. —Flint, 1998: [♂; redescription; to *mexicana* group]. —Almeida and Marinoni, 2001:974 [♀]. —Paprocki et al., 2004:15 [checklist]. —Dumas et al., 2009:364 [distribution]. —Paprocki and França, 2014:78 [checklist].

**Distribution.** Argentina, Brazil, Paraguay.

***zamora*** (*Chimarra*) Blahnik, 1998:116 [Type locality: Ecuador, Zamora-Chinchipe, Zamora; NMNH; ♂; ♀; in *poolei* group].

**Distribution.** Ecuador, Peru.

#### Genus *Chimarrhodella* Lestage [12]

*Chimarrhodella* Lestage, 1925:37 [Type species: *Chimarrha galeata* Martynov, 1912, original designation]. —Blahnik and Holzenthal, 1992a:109 [revision].

*Protarra* Ross, 1956a:68 [Type Species: *Protarra peruviana* Ross, 1956a, original designation]. —Flint, 1971c:20 [to synonymy].

*Loxinum* Navás, 1934a:175 [Type species: *Loxinum aequatorium* Navás, 1934a, original designation]. —Flint1983a:77 [as synonym of *Banyallarga*]. —Flint et al., 1999b:15 [as synonym of *Banyallarga*]. —Prather, 2004:9, 10 [to Philopotamidae, as synonym of *Chimarrhodella*].

This genus is limited to the Neotropical Region, where it is found along the western mountains from Peru to Costa Rica, and eastward into Venezuela and the island of Tobago. Twelve species have been discovered up to now. The immature stages are unknown. Adults are generally taken by net in the day, but a few will also come sparingly to light at night. They are found near small mountain streams in heavily forested areas.

***aequatoria*** (Navás), 1934a:176 [Type locality: Ecuador, Loja; MNHNP; ♀; in *Loxinum*]. —Flint, 1983a:77 [type is missing; to *Banyallarga*]. — Flint et al., 1999b:16 [in *Banyallarga*]. —Prather, 2004:9, 10 [to *Chimarrhodella*].

**Distribution.** Ecuador.

***costaricensis*** Blahnik and Holzenthal, 1992a:116 [Type locality: Costa Rica, Alajuela, Quebrada Latas, 8.9 km NE Bajos del Toro, 10.269°N, 84.260°W; NMNH; ♂; ♀].

**Distribution.** Costa Rica.

***flinti*** Blahnik and Holzenthal, 1992a:117 [Type locality: Venezuela, Barinas, Pte. Parangula, 8 km S Barinitas; NMNH; ♂; ♀].

**Distribution.** Venezuela.

***galeata*** (Martynov), 1912:30 [Type locality: Peru, Callanga; ASL; ♂; in *Chimarrha*]. —Flint, 1975:568 [synonymy]. —Blahnik and Holzenthal, 1992a:117 [♂; ♀; distribution].

—*sagittoides* (Ross), 1956a:69 [Type locality: Bolivia, Mapiri; ZSZMH; ♂; in *Protarra*]. —Flint, 1975:568 [to synonymy].

**Distribution.** Bolivia, Peru.

***nigra*** Flint, 1981:10 [Type locality: Venezuela, Aragua, Dos Riitos, 6 km N Rancho Grande; NMNH; ♂]. —Blahnik and Holzenthal, 1992a:121 [♂].

**Distribution.** Venezuela.

***ornata*** Blahnik, 2004:2 [Type locality: Ecuador, Tungurahua, Río Verde, 1600 m; NMNH; ♂; phylogenetic placement of species].

**Distribution.** Ecuador.

***paria*** Blahnik, 2004:4 [Type locality: Venezuela, Sucre, Peninsula de Paria, Santa Isabel, Río Santa Isabel, 10°44.294'N, 62°38.954'W, el 20 m; UMSP; ♂; phylogenetic placement of species].

**Distribution.** Venezuela.

***peruviana*** (Ross), 1956a:69 [Type locality: Peru, Cusco, Paucartambo, Cosnipata Valley; INHS; ♂; in *Protarra*]. —Flint, 1991:25 [♂; distribution]. —Blahnik and Holzenthal, 1992a:121 [♂; ♀; distribution]. —Flint, 1996b:385 [distribution]. —Muñoz-Quesada, 2000:280 [checklist].

**Distribution.** Colombia, Peru, Venezuela.

***pilcopata*** Blahnik and Holzenthal, 1992a:118 [Type locality: Peru, Cuzco, Pilcopata; NMNH; ♂].

**Distribution.** Peru.

***tapanti*** Blahnik and Holzenthal, 1992a:118 [Type locality: Costa Rica, Cartago, Reserva Tapantí, Quebrada Palmitos and falls, 9.72°N, 83.38°W; NMNH; ♂; ♀].

**Distribution.** Costa Rica.

***tobagoensis*** Blahnik and Holzenthal, 1992a:123 [Type locality: Tobago, St. John, Hermitage River Bridge, Charlotteville; NMNH; ♂; ♀]. —Botosaneanu and Sakal, 1992:202 [distribution]. —Botosaneanu and Alkins-Koo, 1993:29 [distribution]. —Flint, 1996a:71 [distribution].

**Distribution.** Tobago, Trinidad, Venezuela.

***ulmeri*** (Ross), 1956a:69 [Type locality: Peru, Alna [recte, Aina]; ZSZMH; ♂; in *Protarra*]. —Flint, 1981a:10 [♂; distribution]. —Aguila, 1992:535 [distribution]. —Blahnik and Holzenthal, 1992a:125 [♂; ♀; distribution]. —Flint, 1996b:386 [distribution]. —Muñoz-Quesada, 2000:280 [checklist]. —Armitage et al., 2015b:4 [checklist]. —Armitage and Cornejo, 2015:192 [checklist].

**Distribution.** Bolivia, Colombia, Costa Rica, Ecuador, Panama, Peru, Venezuela.

#### Genus *Sortosa* Navás [20]

*Sortosa* Navás, 1918c:227 [Type species: *Sortosa fusca* Navás, 1918c, by monotypy]. —Ross, 1956a:29 [revised as subgenus, but treated as a senior synonym of *Dolophilodes*]. —Schmid, 1964:312 [as subgenus of *Dolophilodes*]. —Blahnik, 2005:5 [elevated to genus].

Blahnik (2005) elevated this genus and several others, formerly treated as subgenera or synonyms of *Dolophilodes* by Ross (1956a) and other authors, to full generic status. As currently defined, the genus is restricted to southern Chile and adjacent regions of Argentina. It is infrequently collected and never in great numbers.

The larvae of a number of the former subgenera of *Dolophilodes* have been described (Weaver et al. 1981, Wiggins 1996). They all inhabit slender, finger-like silken nets in flowing water. Their nets contain a very fine inner silken mesh to filter food from the water (Wallace and Malas 1976). It is likely that the as yet unknown larvae of *Sortosa* have similar nets and habits.

***angulata*** (Schmid), 1964:314 [Type locality: Chile, (O’Higgins), La Leonera; NMNH; ♂; in *Dolophilodes*]. —Flint, 1974e:86 [checklist].

**Distribution.** Chile.

***appendiculata*** (Flint), 1967a:53 [Type locality: Chile, Pcia. Valdivia, Punucapa; NMNH; ♂; in *Dolophilodes*]. —Flint, 1974e:86 [checklist].

**Distribution.** Chile.

***bifida*** (Flint), 1969b:507 [Type locality: Chile, Prov. Concepcion, Quebrada Pinares, near Concepcion; NMNH; ♂; in *Dolophilodes*]. —Flint, 1974e:86 [checklist].

**Distribution.** Chile.

***bispinosa*** (Flint), 1967a:54 [Type locality: Chile, Pcia. Valdivia, Punucapa; NMNH; ♂; in *Dolophilodes*]. —Flint, 1974e:86 [checklist].

**Distribution.** Chile.

***chilensis*** (Navás), 1918d:10 [Type locality: Chile, Marga Marga; MZBS; ♂; in *Dolophilus*]. —Schmid, 1949a:316 [synonymy]. —Ross, 1956a:29 [♂]. —Flint, 1974e:86 [checklist]. —Cohen, 2004:76 [distribution].

—*fusca* Navás, 1918c:228 [Type locality: Chile, Los Perales, Marga-Marga; MZBS; ♂; in *Sortosa*]. —Schmid, 1949a:316 [to synonymy, ♂; ♀].

**Distribution.** Argentina, Chile.

***duplex*** (Schmid), 1964:315 [Type locality: Chile, [Area Metropolitana], Maipu, Rinconada; NMNH; ♂; in *Dolophilodes*]. —Flint, 1974e:87 [checklist].

**Distribution.** Chile.

***dupliplex*** (Flint), 1983a:13 [Type locality: Chile, Pcia. Maule, Alto Tregualemu, ca. 20 km SE Chovellén; NMNH; ♂; in *Dolophilodes*].

**Distribution.** Chile.

***edwardi*** Ross, 1956a:29, 56 [Type locality: Chile, Ñuble, 50 km. east of San Carlos; CAS; ♂]. —Flint, 1974e:87 [checklist].

**Distribution.** Chile.

***elongata*** (Schmid), 1964:315 [Type locality: Chile, (Arauco), Pichinahuel; NMNH; ♂; in *Dolophilodes*]. —Flint, 1974e:87 [checklist].

**Distribution.** Chile.

***elongatoides*** (Flint), 1967a:53 [Type locality: Chile, Pcia. Valdivia, brook at Fundo Walper near Valdivia; NMNH; ♂; in *Dolophilodes*]. —Flint, 1974e:87 [checklist].

**Distribution.** Chile.

***flavipunctata*** Schmid, 1955a:130 [Type locality: Chile, (ile de Chiloé) Aucar; NMNH; ♂; 1964:314 [♂]. —Flint, 1974e:87 [checklist].

**Distribution.** Chile.

***michelbacheri*** Ross, 1956a:29, 56 [Type locality: Chile, Llanquihue, Los Muermos; CAS; ♂]. —Flint, 1974e:87 [checklist].

**Distribution.** Chile.

***paxillifera*** (Flint), 1969b:505 [Type locality: Chile, Prov. Malleco, Rio Blanco, Curacautin; NMNH; ♂; in *Dolophilodes*]. —Flint, 1974e:87 [checklist].

**Distribution.** Chile.

***pectinifera*** Schmid, 1958b:198 [Type locality: Chile, (Linares), Estero de Lleiva; NMNH; ♂]. —Flint, 1974e:87 [checklist].

**Distribution.** Chile.

***prolixa*** (Flint), 1983a:13 [Type locality: Chile, Pcia. Maule, Alto Tregualemu, ca. 20 km SE Chovellén; NMNH; ♂; in *Dolophilodes*].

**Distribution.** Chile.

***scopula*** (Flint), 1983a:14 [Type locality: Chile, Pcia. Maule, Alto Tregualemu, ca. 20 km SE Chovellén; NMNH; ♂; in *Dolophilodes*].

**Distribution.** Chile.

***spectabilis*** (Flint), 1983a:11 [Type locality: Chile, Pcia. Malleco, Parque Nacional Contulmo; NMNH; ♂; in *Dolophilodes*].

**Distribution.** Chile.

***spinifera*** Schmid, 1958b:197 [Type locality: Chile, (Arauco), Pichinahuel; NMNH; ♂]. —Flint, 1974e:87 [checklist].

**Distribution.** Chile.

***spinosella*** (Flint), 1969b:505 [Type locality: Chile, Prov. Curico, Estero la Jaula, Los Quenes; NMNH; ♂; in *Dolophilodes*]. —Flint, 1974e:87 [checklist].

**Distribution.** Chile.

***ventricosta*** (Flint), 1983a:12 [Type locality: Chile, Pcia. Ñuble, Recinto; NMNH; ♂; in *Dolophilodes*].

**Distribution.** Chile.

#### Genus *Wormaldia* McLachlan [50]

*Wormaldia* McLachlan, 1865:140 [Type species: *Hydropsyche occipitalis* Pictet, 1834, subsequent designation of Ross 1949b]. —Ross, 1956a:38 [revision]. —Muñoz-Quesada and Holzenthal, 2008:1 [revision of Nearctic species]. —Muñoz-Quesada and Holzenthal, 2015:1 [revision of Neotropical species, key to males].

The genus is widespread over the Northern Hemisphere, and in the New World occurs in the eastern and western mountains of North America, south through Mexico into Peru and Bolivia, and including some of the Lesser Antillean islands; it is also represented in Baltic amber. In total the genus has close to 200 species; it is especially diverse in Asia and South America.

Larvae of several species from Europe and North America have been described (Wiggins 1996, 2004). As with all the other genera of the family, they construct a tubular retreat of silk that is lined with a very fine silken screen for filtering their food from flowing water. The larvae of the South American representatives have yet to be described.

***alicia*** Bueno-Soria, Santiago-Fragoso and Barba-Álvarez, 2004:483 [Type locality: Mexico, Tabasco, Municipio de Huimanguillo Arroyo las Flores Villa de Guadalupe 2nd seccíon Los Chimalapas, km 5 Ruta Malpasito-Carlos A. Madrazo, 17°22'05"N, 93°36'25"W; IBUNAM; ♂]. —Muñoz-Quesada and Holzenthal, 2015:9 [diagnosis].

**Distribution.** Mexico.

***andrea*** Muñoz-Quesada and Holzenthal, 2015:9 [Type locality: Ecuador, Tungurahua, 13 km E Baños, [1°23'S, 78°25'W], el. 1550 m; NMNH; ♂].

**Distribution.** Ecuador.

***anhelitus*** Muñoz-Quesada and Holzenthal, 2015:10 [Type locality: Costa Rica, Alajuela, Reserva Forestal San Ramón, Río San Lorencito and tribs., 10.216°N, 84.607°W, el. 980 m; NMNH; ♂]. —Armitage et al., 2015b:4 [checklist]. —Armitage and Cornejo, 2015:192 [checklist].

**Distribution.** Costa Rica, Guatemala, Nicaragua, Panama.

***araujoi*** Muñoz-Quesada and Holzenthal, 2015:12 [Type locality: Ecuador, Napo, 5.2 km SW Pano, [1°3'S, 77°52'W], el. 640 m; NMNH; ♂].

**Distribution.** Ecuador.

***arizonensis*** (Ling), 1938:63 [Type locality: United States, Arizona; CAS; ♂; in *Dolophilus*]. —Ross, 1941:51 [distribution]. —Ross, 1944:292 [checklist]. —Ross, 1949b:154 [♂; to *Wormaldia*, *Arizonensis* group]. —Ross, 1956a:38 [♂]. —Denning, 1956a:79 [key] —Flint, 1967d: 165 [distribution]. —Bueno-Soria and Flint, 1978:194 [distribution]. —Armitage, 1996:[work not paginated] [diagnosis; checklist]. —Rojas-Ascencio, et al., 2002:377 [distribution]. —Baumgardner and Bowles, 2005:11 [distribution]. —Bueno-Soria et al., 2007:33 [distribution]. —Muñoz-Quesada and Holzenthal, 2008:16 [♂; redescription; distribution]. —Blinn and Ruiter, 2009b:187 [phenology, distribution]. —Ruiter and Blinn, 2009:5 [♀]. —Muñoz-Quesada and Holzenthal, 2015:13 [♂; redescription; distribution].

**Distribution.** Mexico, U.S.A.

***aymara*** Muñoz-Quesada and Holzenthal, 2015:14 [Type locality: Bolivia, La Paz, Yungas, Circuata to Cajuata, [16°37'S, 67°15'W], el. 2400 m; NMNH;♂].

**Distribution.** Bolivia.

***barbai*** Muñoz-Quesada and Holzenthal, 2015:15 [Type locality: Mexico, Veracruz, near Huatusco, [19°9'N, 96°57'W]; NMNH; ♂].

**Distribution.** Mexico.

***bolivari*** Muñoz-Quesada and Holzenthal, 2015:16 [Type locality: Venezuela, Barinas, 22 km NW Barinitas, [8°45'N, 70°25'W]; NMNH; ♂].

**Distribution.** Venezuela.

***boteroi*** Muñoz-Quesada and Holzenthal, 2015:17 [Type locality: Colombia, Valle del Cauca, Río Raposo, [3°43'N, 77°8'W]; NMNH; ♂].

**Distribution.** Colombia.

***buenorum*** Muñoz-Quesada and Holzenthal, 2015:18 [Type locality: Mexico, Chiapas, Finca Vergel; INHS; ♂]. —Bueno-Soria and Barba-Álvarez, 2011:360 [*nomen nudum*, checklist].

**Distribution.** Mexico.

***calderonae*** Muñoz-Quesada and Holzenthal, 2015:19 [Type locality: Mexico, Chiapas, El Aguacero, Ruta 190 Tuxtla Gutiérrez-Ocosocuautla a 15 km, [16°4'N, 93°33'W]; IBUNAM; ♂]. —Bueno-Soria and Barba-Álvarez, 2011:360 [*nomen nudum*, checklist].

**Distribution.** Mexico.

***chrismark*** Muñoz-Quesada and Holzenthal, 2015:20 [Type locality: Panama, Chiriqui, Fortuna Dam Site, near Hornitos, 8°44'N, 82°16'W, el. 1050 m; NMNH; ♂]. —Armitage et al., 2015b:4 [checklist]. —Armitage and Cornejo, 2015:192 [checklist].

**Distribution.** Panama.

***contrerasi*** Muñoz-Quesada and Holzenthal, 2015:21 [Type locality: Panama, Panama, Cerro Campana, [8°40'60"N, 79°55'W]; NMNH; ♂]. —Armitage et al., 2015b:4 [checklist]. —Armitage and Cornejo, 2015:192 [checklist].

**Distribution.** Panama.

***cornuta*** Bueno-Soria and Holzenthal, 1986:138 [Type locality: Mexico, Chiapas, tributario del Río Teapa sobre la ruta Mex.195, a 3 km al norte de Ixhuatán; IBUNAM; ♂]. —Bueno-Soria and Barba-Álvarez, 2011:360 [checklist]. —Muñoz-Quesada and Holzenthal, 2015:22 [♂; redescription; distribution].

**Distribution.** Mexico.

***dachiardiorum*** Muñoz-Quesada and Holzenthal, 2015:23 [Type locality: Colombia, Magdalena, Parque Nacional Sierra Nevada de Santa Marta, Estación Experimental San Lorenzo, Quebrada Segunda, 11°61'46"N, 74°38'W, el. 2100 m; NMNH; ♂].

**Distribution.** Colombia.

***dampfi*** Ross and King, in Ross, 1956a:39, 62 [Type locality: Mexico, Chiapas, San Cristobal; INHS; ♂]. —Bueno-Soria and Flint, 1978:194 [distribution]. —Chamorro-Lacayo et al., 2007:46 [checklist]. —Bueno-Soria and Barba-Álvarez, 2011:360 [checklist]. —Muñoz-Quesada and Holzenthal, 2015:24 [♂; redescription; distribution].

**Distribution.** Mexico, Nicaragua.

***dorsata*** Ross and King, in Ross, 1956a:39, 62 [Type locality: Mexico, Chiapas, Finca Vergel; INHS; ♂]. —Bueno-Soria and Flint, 1978:194 [distribution]. —Bueno-Soria and Barba-Álvarez, 2011:360 [checklist]. —Muñoz-Quesada and Holzenthal, 2015:25 [♂; redescription; distribution].

**Distribution.** Mexico.

***eberhardi*** Muñoz-Quesada and Holzenthal, 2015:26 [Type locality: Panama, Panama, Canal Zone, Gamboa, Pipeline Rd., [9°7'N, 79°43'W]; NMNH; ♂]. —Armitage et al., 2015b:4 [checklist]. —Armitage and Cornejo, 2015:192 [checklist].

**Distribution.** Panama.

***endonima*** Ross and King, in Ross, 1956a:39, 62 [Type locality: Mexico, Chiapas, Finca Germania; INHS; ♂]. —Bueno-Soria and Flint, 1978:194 [distribution]. —Bueno-Soria and Barba-Álvarez, 2011:360 [checklist]. —Muñoz-Quesada and Holzenthal, 2015:27 [♂; redescription; distribution].

**Distribution.** Mexico.

***esperonis*** Ross and King, in Ross, 1956a:41, 63 [Type locality: Mexico, Chiapas, Finca Esperanza; INHS; ♂]. —Bueno-Soria and Flint, 1978:194 [distribution]. —Bueno-Soria and Barba-Álvarez, 2011:360 [checklist]. —Muñoz-Quesada and Holzenthal, 2015:28 [♂; redescription; distribution].

**Distribution.** Guatemala, Mexico.

***flinti*** Muñoz-Quesada and Holzenthal, 2015:28 [Type locality: Panama, Chiriqui, Guadalupe Arriba, 8°52'26"N, 82°33'13"W; NMNH; ♂]. —Armitage et al., 2015b:4 [checklist]. —Armitage and Cornejo, 2015:192 [checklist].

**Distribution.** Bolivia, Panama.

***francovilla*** Muñoz-Quesada and Holzenthal, 2015:30 [Type locality: Panama, Panama, Barro Colorado Island, [9°8'59"N, 79°50'59"W]; NMNH;♂]. —Armitage et al., 2015b:4 [checklist]. —Armitage and Cornejo, 2015:192 [checklist].

**Distribution.** Panama.

***fredycarol*** Muñoz-Quesada and Holzenthal, 2015:30 [Type locality: Costa Rica, San José, trib. to Quebrada Caraigres, 3.6 km (road) SW La Legua, 9.728°N, 84.125°W, el. 1650 m; UMSP; ♂]. —Armitage et al., 2015b:4 [checklist]. —Armitage and Cornejo, 2015:192 [checklist].

**Distribution.** Costa Rica, Panama.

***gallardoi*** Muñoz-Quesada and Holzenthal, 2015:31 [Type locality: Costa Rica, Guanacaste, Parque Nacional Guanacaste, Río San Josecito, 10.922°N, 85.470°W, el. 960 m; UMSP; ♂]. —Armitage et al., 2015b:4 [checklist]. —Armitage and Cornejo, 2015:192 [checklist].

**Distribution.** Costa Rica, Panama.

***gonzalezae*** Muñoz-Quesada and Holzenthal, 2015:34 [Type locality: Venezuela, Aragua, 1 km S Rancho Grande; NMNH; ♂].

**Distribution.** Venezuela.

***hedamafera*** Muñoz-Quesada and Holzenthal, 2015:34 [Type locality: Costa Rica, Alajuela, Río Toro, 3 km (road) SW Bajos del Toro, 10.204°N, 84.316°W, el. 1530 m; UMSP; ♂].

**Distribution.** Costa Rica, Nicaragua.

***imberti*** Muñoz-Quesada and Holzenthal, 2015:36 [Type locality: Costa Rica, Puntarenas, Península de Osa, Corcovado, Estación Sirena, [8°29'N, 83°35'W]; NMNH; ♂].

**Distribution.** Costa Rica.

***inca*** Muñoz-Quesada and Holzenthal, 2015:37 [Type locality: Peru, Huánuco, Tingo María, [9°18'S, 75°59'W], el. 672 m; NMNH; ♂].

**Distribution.** Peru.

***insignis*** (Martynov), 1912:29 [Type locality: Peru, Callanga; ASL; ♂; in *Dolophilus*]. —Ross, 1956a:62 [checklist]. —Flint, 1975:568 [synonymy]. —Flint, 1996b:385 [distribution]. —Muñoz-Quesada and Holzenthal, 2015:38 [♂; redescription; distribution].

—*ostina* Ross, 1956a:64 [Type locality: Peru, Department of Cuzco, Santa Isabel, Valley of Cosnipata; INHS; ♂]. —Flint, 1975:568 [to synonymy].

**Distribution.** Peru.

***isela*** Muñoz-Quesada and Holzenthal, 2015:39 [Type locality: Mexico, Nuevo León, Santiago, Potrero Redondo; IBUNAM; ♂].

**Distribution.** Mexico.

***juarox*** Muñoz-Quesada and Holzenthal, 2015:40 [Type locality: Costa Rica, Cartago, Reserva Tapanti, Quebrada Segunda @ administrative building, and falls, 9.761°N, 83.787°W, el. 1250 m; NMNH; ♂].

**Distribution.** Costa Rica.

***lauglo*** Muñoz-Quesada and Holzenthal, 2015:41 [Type locality: Panama, Chiriqui, Fortuna Dam Site, near Hornitos, 8°44'N, 82°6'W, el. 1050 m; NMNH; ♂]. —Armitage et al., 2015b:4 [checklist]. —Armitage and Cornejo, 2015:192 [checklist].

**Distribution.** Panama.

***luma*** Bueno-Soria and Holzenthal, 1986:140 [Type locality: Mexico, Oaxaca, La Esperanza, km 50, Ruta 175; IBUNAM; ♂]. —Muñoz-Quesada and Holzenthal, 2015:42 [♂; redescription; distribution].

**Distribution.** Mexico.

***machadorum*** Muñoz-Quesada and Holzenthal, 2015:43 [Type locality: Costa Rica, Alajuela, Reserva Forestal San Ramón, Río San Lorencito and tribs., 10.216°N, 84.607°W, el. 980 m; UMSP; ♂]. —Armitage et al., 2015b:4 [checklist]. —Armitage and Cornejo, 2015:192 [checklist].

**Distribution.** Costa Rica, Panama.

***maesi*** Muñoz-Quesada and Holzenthal, 2015:44 [Type locality: Nicaragua, Zelaya, Cerro Saslaya, 13°44'N, 85°01'W, el. 700 m; NMNH; ♂].

**Distribution.** Nicaragua.

***matagalpa*** Flint, 1995:8 [Type locality: Nicaragua, Department of Matagalpa, on the road Maragalpa-Jinotega, Fuente Pura, 13°01'N, 85°55'W; NMNH; ♂]. —Maes, 1999:1192 [checklist]. —Chamorro-Lacayo et al., 2007:46 [checklist]. —Muñoz-Quesada and Holzenthal, 2015:44 [♂; redescription; distribution].

**Distribution.** Costa Rica, Guatemala, Nicaragua.

***menchuae*** Muñoz-Quesada and Holzenthal, 2015:45 [Type locality: Guatemala, Izabal, Matías de Gálvez, [15°41'60"N, 88°37'W]; NMNH; ♂].

**Distribution.** Guatemala.

***monsonorum*** Muñoz-Quesada and Holzenthal, 2015:46 [Type locality: Costa Rica, Cartago, Turrialba, Río Chitaría, route 10, 10 km NW Río Reventazón, 9.920°N, 83.604°W, el. 740 m; UMSP; ♂].

**Distribution.** Costa Rica.

***navarroae*** Muñoz-Quesada and Holzenthal, 2015:47 [Type locality: Mexico, Oaxaca, km 11, carretera Teotitlán-Huautla, [18°15'N, 97°02'W]; IBUNAM; ♂].

**Distribution.** Mexico.

***palma*** Flint, 1991:31 [Type locality: Colombia, Dpto. Antioquia, 10 km E Medellín [road to Las Palmas]; NMNH; ♂]. —Muñoz-Quesada, 2000:280 [checklist]. —Muñoz-Quesada and Holzenthal, 2015:48 [♂; redescription; distribution].

**Distribution.** Colombia.

***paprockevi*** Muñoz-Quesada and Holzenthal, 2015:49 [Type locality: Costa Rica, Puntarenas, Río Jaba and rock quarry 1.4 km (air) W Las Cruces [San Vito de Jaba], 8.79°N, 82.97°W, el. 1150 m; UMSP; ♂].

**Distribution.** Costa Rica.

***planae*** Ross and King, in Ross, 1956a:41, 64 [Type locality: Mexico, Chiapas, Finca Vergel; INHS; ♂]. —Flint, 1968b:9 [♂; distribution]. —Bueno-Soria and Flint, 1978:194 [distribution]. —Flint, 1981a:10 [♂; ♀; distribution]. —Holzenthal, 1988c:58 [distribution]. —Botosaneanu, 1990a:43 [distribution]. —Flint, 1991:31 [♂; distribution]. —Aguila, 1992:535 [distribution]. —Botosaneanu and Sakal, 1992:202 [distribution; ecology]. —Botosaneanu and Alkins-Koo, 1993:29 [distribution]. —Flint and Sykora, 1993:48 [distribution]. —Flint, 1995:8 [distribution]. —Flint, 1996a:70 [distribution]. —Maes, 1999:1192 [checklist]. —Muñoz-Quesada, 2000:280 [checklist]. —Botosaneanu and Viloria, 2002:108 [distribution]. —Paprocki et al., 2004:15 [checklist]. —Botosaneanu and Thomas, 2005:47 [distribution]. —Bueno-Soria et al., 2005:75 [distribution]. —Chamorro-Lacayo et al., 2007:46 [checklist]. —Muñoz-Quesada and Holzenthal, 2008:53 [♂; redescription; distribution]. —Bueno-Soria and Barba-Álvarez, 2011:360 [checklist]. —Paprocki and França, 2014:78 [checklist]. —Muñoz-Quesada and Holzenthal, 2015:50 [♂; redescription; distribution]. —Armitage et al., 2015b:4 [checklist]. —Armitage and Cornejo, 2015:192 [checklist].

—*arcopa* Denning, in Denning and Sykora, 1966:1219 [Type locality: Panama, [Panama], Canal Zone, Barro Colorado Island; CAS; ♂]. —Aguila, 1992:535 [distribution]. —Muñoz-Quesada and Holzenthal, 2015:50 [to synonymy].

**Distribution.** Brazil, Colombia, Costa Rica, Dominica, Ecuador, Grenada, Guatemala, Guyana, Martinique, Mexico, Nicaragua, Panama, St. Vincent, Tobago, Trinidad, Venezuela, U.S.A.

***prolixa*** Flint, 1991:31 [Type locality: Colombia, Dpto. Antioquia, 12 km E Medellín [road to Sta. Elena]; NMNH; ♂]. —Muñoz-Quesada, 2000:280 [checklist]. —Muñoz-Quesada and Holzenthal, 2015:54 [♂; redescription; distribution].

**Distribution.** Colombia.

***saboriorum*** Muñoz-Quesada and Holzenthal, 2015:55 [Type locality: Panama, Chiriqui, Guadalupe Arriba, 8°52'26"N, 82°33'13"W; NMNH; ♂]. —Armitage et al., 2015b:4 [checklist]. —Armitage and Cornejo, 2015:192 [checklist].

**Distribution.** Panama.

***tarasca*** Bueno-Soria and Holzenthal, 1986:139 [Type locality: Mexico, Estado de Michoacán, Coalcomán (La Nieve); IBUNAM; ♂]. —Rojas-Ascencio, et al., 2002:377 [distribution]. —Muñoz-Quesada and Holzenthal, 2015:56 [♂; redescription; distribution].

**Distribution.** Mexico.

***tocajoma*** Muñoz-Quesada and Holzenthal, 2015:57 [Type locality: Costa Rica, Cartago, Reserva Tapanti, Quebrada Segunda @ administrative building, and falls, 9.761°N, 83.787°W, el. 1250 m; UMSP; ♂].

**Distribution.** Costa Rica.

***trondi*** Muñoz-Quesada and Holzenthal, 2015:58 [Type locality: Costa Rica, Alajuela, Reserva Forestal San Ramón, Río San Lorencito and tribs., 10.216°N, 84.607°W, el. 980 m; UMSP; ♂]. —Armitage et al., 2015b:4 [checklist]. —Armitage and Cornejo, 2015:192 [checklist].

**Distribution.** Costa Rica, Panama.

***tupacamara*** Muñoz-Quesada and Holzenthal, 2015:59 [Type locality: Bolivia, La Paz, Yungas, Puente Mururata to Cusilloni, [16°8'S, 67°44'W], el. 1600 m; NMNH; ♂].

**Distribution.** Bolivia.

***zunigae*** Muñoz-Quesada and Holzenthal, 2015:60 [Type locality: Colombia, Risaralda, Termales de Santa Rosa de Cabal, [4°52'N, 75°38'W]; NMNH; ♂].

**Distribution.** Colombia.

***zunigarceorum*** Muñoz-Quesada and Holzenthal, 2015:61 [Type locality: Costa Rica, Cartago, 5 km W. of Turrialba, [9°54'N, 83°40'W], el. 638 m; NMNH; ♂]. —Armitage et al., 2015b:4 [checklist]. —Armitage and Cornejo, 2015:192 [checklist].

**Distribution.** Costa Rica, Panama.

### Family Philorheithridae

The family is represented in the New World by two genera and six species endemic to southern Chile and adjacent Argentina. Seven other genera and 24 species are found on Australia, Tasmania, and New Zealand in the Australasian region, and from Madagascar, representing a Gondwanian distributional pattern. However, the discovery of a fossil philorheithrid from Transbaikalia in Russia suggests a former global distribution of the family (Sukatsheva and Vassilenko 2011).

Larvae of the Australian forms build stout cases of mineral matter and are predatory (Neboiss 1991). Larvae of the Chilean species, although collected, have not yet been described. They live in rivers and small streams, often in the sandy bottom.

#### Genus *Mystacopsyche* Schmid [2]

*Mystacopsyche* Schmid, 1955a:133 [Type species: *Mystacopsyche ochracea* Schmid, 1955a, original designation]. —Schmid 1964:328 [redescription].

Two species of this endemic Chilean genus are known. Adults are commonly swept from vegetation beside second and third order streams and also come to lights at night. No information is published on their immature stages.

***longipilosa*** Schmid, 1964:329 [Type locality: Chile, Malleco, Angol; NMNH; ♂]. —Flint, 1974e:91 [checklist]. —Flint, 1990:120 [distribution].

**Distribution.** Argentina, Chile.

***ochracea*** Schmid, 1955a:134 [Type locality: Chile, Malleco, Rio Blanco; NMNH; ♂]. —Schmid, 1964:329 [redescription]. —Flint, 1974e:91 [checklist].

**Distribution.** Chile.

#### Genus *Psilopsyche* Ulmer [4]

*Psilopsyche* Ulmer, 1907a:7 [Type species: *Psilopsyche kolbiana* Ulmer, 1907a, by monotypy].

The genus now contains four species, endemic to southern Chile and adjacent Argentina. The adults come commonly to lights placed close to most types of flowing water. The immature stages are undescribed.

***chillana*** Navás, 1934a:170 [Type locality: Chile, Chillán; collection Navás, now lost?; ♂]. —Flint, 1974e:91 [checklist].

**Distribution.** Chile.

***granda*** Oláh and Johanson, 2010:122 [Type locality: Chile, Region del Araucania (IX), PN Nahuelbuta, camp site, Estero Cabreria, crosspoint between streams draining Mts Pichimanquemáhuida and Pichinahuel, 37°49.647'S, 73°00.691'W, 1100 m; NRS; ♂].

**Distribution.** Chile.

***kolbiana*** Ulmer, 1907a:8 [Type locality: Chile, Linares, Longavi; ZMHU; ♂]. —Schmid, 1949a:396 [♂]. —Flint, 1974e:91 [checklist]. —Flint, 1990:120 [distribution]. —Flint et al., 1999a:80 [synonymy]. —Oláh and Johanson, 2010:121 [distribution].

—*ruiziana* Navás, 1926:332 [Type locality: Chile, Lonquimay; MZBS; ♂]. —Flint, 1974e:91 [checklist]. —Flint et al., 1999a:80 [to synonymy].

—*blanchardi* Navás, 1926:333 [Type locality: Chile, Lonquimay; collection Navás, now lost?; ♀]. —Navás, 1928:128 [to synonymy].

**Distribution.** Argentina, Chile.

***molinai*** Navás, 1926:334 [Type locality: Chile, Lonquimay; MZBS; ♂]. —Navás, 1928:128 [♀]. —Schmid, 1949a:395 [♂; redescription]. —Flint, 1974e:91 [checklist]. —Flint et al., 1999a:80 [synonymy]. —Cohen, 2004:79 [distribution]. —Brand, 2009:225 [distribution]. —Oláh and Johanson, 2010:122 [distribution]. —Brand and Miserendino, 2011a:35 [biology; habitat]. —Brand and Miserendino, 2011b:143 [biology]. —Brand et al., 2012:90 [biology]. —Brand and Miserendino, 2014:6 [community ecology].

—*macqueeni* Navás, 1935:373 [Type locality: Chile, Aysén; MNHNS, now lost; ♂; as *Mac-Queeni*]. —Flint et al., 1999a:80 [to synonymy].

**Distribution.** Argentina, Chile.

### Family Polycentropodidae

This is a large and diverse family well represented in all biogeographic regions of the world. The family has undergone two recent major phylogenetic assessments and changes to classification (Chamorro and Holzenthal 2011, Johanson et al. 2012). For the Neotropical fauna, this most directly involved the establishment of the family Pseudoneureclipsidae for the genera *Pseudoneureclipsis* and *Antillopsyche*, the later endemic to the Greater Antilles, and both formerly included in the Polycentropodidae. Accordingly, the family includes only five genera in the Neotropics and adjacent Nearctic Mexico: *Cernotina*, *Cyrnellus*, *Nyctiophylax*, *Polycentropus*, and *Polyplectropus*. The first two are endimic to the New World, but the others are more or less cosmopolitan and not unequivocally monophyletic.

The larvae all construct silken retreats, usually open at both ends and with a surrounding trap net of silken strands (Wiggins 1996), although these may not be present in some genera. They appear to be very strongly predaceous, but also ingest plant detritus (Townsend and Hildrew 1978, Wiggins 1996).

#### Genus *Cernotina* Ross [68 + †1]

*Cernotina* Ross, 1938b:136 [Type species: *Cernotina calcea* Ross, 1938b, original designation]. —Flint, 1971c:33 [key, Amazonian species]. —Wichard, 2007a:32 [diagnosis].

The genus is found only in the Nearctic and Neotropical regions, but it is in South America where the greatest diversity of species occurs. The larvae of a North American species were associated and described by Hudson et al. (1981). They are more common in lentic or slowly flowing lotic waters, but some species are found in small, fast streams (Flint 1964a, Hudson et al. 1981). Their silken retreats seemed to cover small depressions in the substrate, with one or both ends flared out into regular flaps of silken threads, similar to those of *Cyrnellus* (Hudson et al. 1981). The guts of *Cyrnellus spicata* were found filled with chironomid larvae and microcrustaceans (Hudson et al. 1981).

***abbreviata*** Flint, 1971c:40 [Type locality: Brazil [Edo. Pará, headwaters of Rio Paru], Igarapé Aepuku Äku; NMNH; ♂]. —Paprocki et al., 2004:15 [checklist]. —Paprocki and França, 2014:79 [checklist].

**Distribution.** Brazil.

***acalyptra*** Flint, 1971c:34 [Type locality: Brazil [Edo. Amazonas], Rio Marauiá, Cachoeira Rio Irapirapí; NMNH; ♂]. —Flint, 1974c:43 [♂; distribution]. —Paprocki et al., 2004:15 [checklist]. —Paprocki and França, 2014:79 [checklist].

**Distribution.** Brazil, Suriname.

***aestheticella*** Sykora, 1998:99 [Type locality: Peru, Departemento Loreto, bank of Yanomono Creek just below Explorama Lodge; CMNH; ♂].

**Distribution.** Peru.

***anhanguera*** Camargos, Barcelos-Silva and Pes *in* Barcelos-Silva et al., 2013:117 [Type locality: Brazil, Goiás, Niquelândia, Pires stream, Anglo American/Codemin, 14°11'0.59"S, 48°21'4.40"W; INPA; ♂]. —Paprocki and França, 2014:79 [checklist].

**Distribution.** Brazil.

***antonina*** Holzenthal and Almeida, 2003:23 [Type locality: Brazil, Paraná, Atonina, Reserva de Sapitanduva, 25°28'S, 48°50'W, el. 60 m; DZUP; ♂]. —Paprocki et al., 2004:15 [checklist]. —Barcelos-Silva et al., 2012:1279 [distribution]. —Souza et al., 2013a:8 [distribution]. —Paprocki and França, 2014:79 [checklist].

**Distribution.** Brazil.

***artiguensis*** Angrisano, 1994:135 [Type locality: Uruguay, Depto. Artigas, Sepulturas, Picada del Negro Muerto, orilla río Cuareim; FHCU; ♂].

**Distribution.** Uruguay.

***aruma*** Santos and Nessimian, 2008:30 [Type locality: Brazil, Amazonas, Manaus, Igarapé Arumã, tributary to Rio Cuieiras, 02°30'55.2"S, 60°15'44.4"W; INPA; ♂]. —Paprocki and França, 2014:79 [checklist].

**Distribution.** Brazil.

***astera*** Ross, 1941:76 [Type locality: United States, Texas, San Felipe Springs, Del Rio; INHS; ♂]. —Bueno-Soria and Flint, 1978:198 [distribution]. —Maes and Flint, 1988:3 [distribution]. —Maes, 1999:1188 [checklist]. —Bowles et al., 2007:23 [distribution; biology]. —Chamorro-Lacayo et al., 2007:46 [checklist].

**Distribution.** Mexico, Nicaragua, U.S.A.

***attenuata*** Flint, 1971c:36 [Type locality: Brazil [Edo. Amazonas], Igarapé, Barro branco; NMNH; ♂]. —Paprocki et al., 2004:15 [checklist]. —Paprocki and França, 2014:79 [checklist].

**Distribution.** Brazil.

***bibrachiata*** Flint, 1971c:37 [Type locality: Brazil [Edo. Amazonas], Manaus, Cachoeira do Gigante; NMNH; ♂]. —Sykora, 1998:102 [distribution]. —Paprocki et al., 2004:15 [checklist]. —Paprocki and França, 2014:80 [checklist].

**Distribution.** Brazil, Peru.

***bispicata*** Camargos, Barcelos-Silva and Pes *in* Barcelos-Silva et al., 2013:120 [Type locality: Brazil, Goiás, Niquelândia, Fazenda Horto Aranha, Anglo American/Codemin, 14°25'12.00"S, 48°44'9.00"W; INPA; ♂]. —Paprocki and França, 2014:80 [checklist].

**Distribution.** Brazil.

***cacha*** Flint, 1971c:35 [Type locality: Brazil [Edo. Amazonas], Rio Marauiá, Endstation vor larger Cachoeira; NMNH; ♂]. —Blahnik et al., 2004:5 [distribution]. —Paprocki et al., 2004:15 [checklist]. —Angrisano and Sganga, 2007:15 [♂; distribution]. —Paprocki and França, 2014:80 [checklist].

**Distribution.** Argentina, Brazil.

***cadeti*** Flint, 1968b:20 [Type locality: St. Lucia, Vergallier R, Marquis; NMNH; ♂]. —Flint and Sykora, 1993:49 [checklist]. —Botosaneanu, 2002:95 [checklist].

**Distribution.** St. Lucia.

***calcea*** Ross, 1938b:137 [Type locality: United States, Illinois, Kankakee; INHS; ♂]. —Bueno-Soria and Flint, 1978:198 [distribution]. —Maes and Flint, 1988:3 [distribution]. —Maes, 1999:1188 [checklist]. —Bowles et al., 2007:23 [distribution; biology]. —Chamorro-Lacayo et al., 2007:46 [checklist].

**Distribution.** Mexico, Nicaragua, U.S.A.

***caliginosa*** Flint, 1968a:24 [Type locality: Jamaica, St. Andrew, Hardwar Gap, Dicks Pond Trail; NMNH; ♂]. —Flint, 1968b:80 [checklist]. —Botosaneanu, 2002:95 [checklist].

**Distribution.** Jamaica.

***carbonelli*** Flint, 1983a:32 [Type locality: Uruguay, Dpto. Artigas, Río Cuareim, Sepulturas; NMNH; ♂]. —Angrisano, 1994:137 [distribution].

**Distribution.** Uruguay.

***chelifera*** Flint, 1972b:231 [Type locality: Argentina, Prov. Misiones, Capioví; NMNH; ♂].

**Distribution.** Argentina.

***chiapaneca*** Bueno-Soria, 2010:30 [Type locality: Mexico, Chiapas, Colón El Lagartero, 15°50'303.47"N, 91°52'32.78"W, el. 640 m; CNIN; ♂].

**Distribution.** Mexico.

***cingulata*** Flint, 1971c:41 [Type locality: Brazil [Edo. Amazonas], Rio Branquinho, Lager Tapirí; NMNH; ♂]. —Paprocki et al., 2004:15 [checklist]. —Paprocki and França, 2014:80 [checklist].

**Distribution.** Brazil.

***compressa*** Flint, 1971c:39 [Type locality: Brazil [Edo. Amazonas], Rio Marauiá, eine Tagesreise unterhalb der Mission S. Antonio; NMNH; ♂]. —Paprocki et al., 2004:15 [checklist]. —Paprocki and França, 2014:80 [checklist].

**Distribution.** Brazil.

***cygnea*** Flint, 1971c:37 [Type locality: Brazil [Edo. Amazonas], Rio Solimões, Ilha Juçara; NMNH; ♂]. —Sykora, 1998:102 [distribution]. —Paprocki et al., 2004:15 [checklist]. —Paprocki and França, 2014:80 [checklist].

**Distribution.** Brazil, Peru.

***cystophora*** Flint, 1971c:35 [Type locality: Brazil [Edo. Amazonas], Rio Branquinho, etwa 2 1/2 Stunden oberhalb Tapirí-Lager; NMNH; ♂]. —Paprocki et al., 2004:15 [checklist]. —Paprocki and França, 2014:80 [checklist].

**Distribution.** Brazil.

***danieli*** Flint and Sykora, 2004:52 [Type locality: Dominican Republic, Pedernales Province, Río Mulito, 13 km N Pedernales, 18°09'N, 71°46'W, el. 230 m; ♂; ♀]. —Pérez-Gelabert, 2008:302 [checklist].

—*Cernotina* sp. Flint and Pérez-Gelabert, 1999:43 [erroneously listed as ♂; recte ♀].

**Distribution.** Dominican Republic.

***declinata*** Flint, 1971c:36 [Type locality: Brazil [Edo. Para], Rio Paru, Mission Tiriyós; NMNH; ♂]. —Flint, 1974c:48 [♂; distribution]. —Paprocki et al., 2004:15 [checklist]. —Paprocki and França, 2014:80 [checklist].

**Distribution.** Brazil, Suriname.

***decumbens*** Flint, 1971c:37 [Type locality: Brazil [Edo. Amazonas], Rio Aripuana, Beneficente; NMNH; ♂; as *decembens*, lapsus calami]. —Paprocki et al., 2004:15 [checklist]. —Paprocki and França, 2014:81 [checklist].

**Distribution.** Brazil.

***depressa*** Flint, 1974c:49 [Type locality: Suriname, Lawa River, Anapaike; RNH; ♂].

**Distribution.** Suriname.

***ecotura*** Sykora, 1998:99 [Type locality: Brazil, Estado Roraima, Boa Vista, Rio Branco; CMNH; ♂]. —Paprocki et al., 2004:15 [checklist]. —Paprocki and França, 2014:81 [checklist].

**Distribution.** Brazil.

***encrypta*** Flint, 1971c:35 [Type locality: Brazil [Edo. Amazonas], Rio Negro, Ponta Negra; NMNH; ♂]. —Paprocki et al., 2004:15 [checklist]. —Paprocki and França, 2014:81 [checklist].

**Distribution.** Brazil.

***falcata*** Camargos, Barcelos-Silva and Pes *in* Barcelos-Silva et al., 2013:120 [Type locality: Brazil, Goiás, Niquelândia, Fazenda Horto Aranha, Anglo American/Codemin, 14°24'8.28"S, 48°43'40.19"W; INPA; ♂]. —Paprocki and França, 2014:81 [checklist].

**Distribution.** Brazil.

***fallaciosa*** Flint, 1983a:30 [Type locality: Argentina, Pcia. Misiones, Arroyo Coatí, 15 km E San José; NMNH; ♂]. —Angrisano, 1994:137 [distribution]. —Angrisano and Sganga, 2007:15 [♂; distribution].

**Distribution.** Argentina, Uruguay.

***filiformis*** Flint, 1971c:39 [Type locality: Brazil [Edo. Amazonas], Rio Branquinho, Lager Tapirí; NMNH; ♂; as *Cernotino filiformiss* on p. 39, a printers error]. —Flint, 1974c:48 [♂; distribution]. —Paprocki et al., 2004:15 [checklist]. —Paprocki and França, 2014:81 [checklist].

**Distribution.** Brazil, Suriname.

***flexuosa*** Santos and Nessimian, 2008:31 [Type locality: Brazil, Amazonas, Rio Preto da Eva municipality, tributary to Rio Preto da Eva, 02°41'28.7"S, 59°42'01.3"W; INPA; ♂]. —Paprocki and França, 2014:81 [checklist].

**Distribution.** Brazil.

***harrisi*** Sykora, 1998:96 [Type locality: Peru, Departemento Loreto, bank of Río Yanomono just belwo Explorama Lodge; CMNH; ♂].

**Distribution.** Peru.

***hastilis*** Flint, 1996a:75 [Type locality: Tobago, Bridge B1/5, 6.5 km N Roxborough, 11°17'N, 60°35'W; NMNH; ♂]. —Botosaneanu, 2002:95 [checklist].

**Distribution.** Tobago.

***intersecta*** Flint, 1974c:48 [Type locality: Suriname, Wilhelmina Mountains, trail I km 8, small stony creek; RNH; ♂].

**Distribution.** Suriname.

***lanceolata*** Barcelos-Silva, Camargos and Pes *in* Barcelos-Silva et al., 2013:122 [Type locality: Brazil, Espírito Santo, Linhares, Praia do Minotauro, 19°19'05.8"S, 40°05'11.9"W; CZNC; ♂]. —Barcelos-Silva et al., 2012:1279 [as *Cernotina* sp. 3]. —Paprocki and França, 2014:81 [checklist].

**Distribution.** Brazil.

***laticula*** Ross, 1951b:348 [Type locality: Mexico, Campeche, Salto Grande; INHS; ♂]. —Bueno-Soria and Flint, 1978:198 [distribution].

**Distribution.** Mexico.

***lazzarii*** Holzenthal and Almeida, 2003:24 [Type locality: Brazil, Paraná, município de Corbélia, Rio Novo headwaters, 24°53.886'S, 53°14.895'W, el. 700 m; MZUSP; ♂; ♀]. —Paprocki et al., 2004:15 [checklist]. —Paprocki and França, 2014:81 [checklist].

**Distribution.** Brazil.

***lobisomem*** Santos and Nessimian, 2008:27 [Type locality: Brazil, Amazonas, Manaus, tributary to Igarapé do Lobisomem, basin of Rio Cuieiras, 2°33'46.4"S, 60°19'03.4"W; INPA; ♂]. —Paprocki and França, 2014:82 [checklist].

**Distribution.** Brazil.

***longispina*** Barcelos-Silva, Camargos and Pes *in* Barcelos-Silva et al., 2013:124 [Type locality: Brazil, Espírito Santo, Pinheiros, stream Água Limpa, 18°22'04.1"S, 40°08'23.8"W; CZNC; ♂]. —Barcelos-Silva et al., 2012:1279 [as *Cernotina* sp. 1]. —Paprocki and França, 2014:82 [checklist].

**Distribution.** Brazil.

***longissima*** Flint, 1974c:46 [Type locality: Suriname, Brownsberg, mountain creek near Golddiggers camp; RNH; ♂].

**Distribution.** Suriname.

***lutea*** Flint, 1968b:19 [Type locality: Dominica, Pont Casse, 1.3 miles E; NMNH; ♂; ♀]. —Flint and Sykora, 1993:49 [checklist]. —Botosaneanu, 1994a:51 [distribution]. —Botosaneanu, 2002:95 [checklist]. —Botosaneanu and Thomas, 2005:51 [probable distribution].

**Distribution.** Dominica, Guadeloupe, Martinique.

***mandeba*** Flint, 1974c:45 [Type locality: Suriname, Nickerie River, Blanche Marie, falls in creek; RNH; ♂]. —Botosaneanu and Alkins-Koo, 1993:31 [♂; distribution]. —Flint, 1996a:74 [distribution]. —Botosaneanu, 2002:95 [checklist].

**Distribution.** Suriname, Tobago, Trinidad.

***mastelleri*** Flint, 1992c:382 [Type locality: Puerto Rico, El Verde Field Station, Quebrada Prieta; NMNH; ♂; ♀]. —Botosaneanu, 2002:95 [checklist].

—Subfamily Polycentropodinae species Flint, 1964a:34 [larva; pupa]. —Flint, 1992c:382 [to synonymy].

**Distribution.** Puerto Rico.

***medioloba*** Flint, 1972b:231 [Type locality: Argentina, Pcia. Santa Fe, Arroyo Saladillo, near Santa Fé; NMNH; ♂].

**Distribution.** Argentina.

***nigridentata*** Sykora, 1998:96 [Type locality: Peru, Departmento Loreto, banks of Yanomono Creek just below Explorama Lodge; CMNH; ♂].

**Distribution.** Peru.

***obliqua*** Flint, 1971c:40 [Type locality: Brazil [Edo. Amazonas], Rio Branquinho, bei der Mündung des Rio Cuieiras; NMNH; ♂]. —Paprocki et al., 2004:15 [checklist]. —Paprocki and França, 2014:82 [checklist].

**Distribution.** Brazil.

***odonta*** Santos and Nessimian, 2008:26 [Type locality: Brazil, Amazonas, Manaus, tributary to Igarapé do Lobisomem, basin of Rio Cuieiras, 2°33'46.4"S, 60°19'03.4"W; INPA; ♂]. —Paprocki and França, 2014:82 [checklist].

**Distribution.** Brazil.

***pallida*** Banks, 1904c:214 [Type locality: [U.S.A.], Washington, D.C.; Banks collection; as *Cyrnus pallidus*]. —Maes, 1999:1188 [checklist]. —Chamorro-Lacayo et al., 2007:46 [checklist].

**Distribution.** Nicaragua, U.S.A.

***perpendicularis*** Flint, 1971c:40 [Type locality: Brazil [Edo. Amazonas], Rio Negro, etwa 20-30 km. oberhalb von A-31 [A-31=80 km above Manaus]. NMNH; ♂]. —Flint, 1974c:49 [♂; distribution]. —Angrisano, 1994:137 [distribution]. —Blahnik et al., 2004:5 [distribution]. —Paprocki et al., 2004:15 [checklist]. —Angrisano and Sganga, 2007:16 [♂; distribution]. —Paprocki and França, 2014:82 [checklist].

**Distribution.** Argentina, Brazil, Suriname, Uruguay.

***pesae*** Santos and Nessimian, 2008:28 [Type locality: Brazil, Amazonas, Manaus, tributary to Igarapé Cachoeira, basin of Rio Cuieiras, 02°41'45.4"S 60°17'42.7"W; INPA; ♂]. —Paprocki and França, 2014:82 [checklist].

**Distribution.** Brazil.

† ***pulchra*** Wichard, 2007a:32 [Type locality: Dominican Republic; SMNS; ♂; in amber].

**Distribution.** Dominican Republic.

***puri*** Dumas and Nessimian, 2011:32 [Type locality: Brazil, Rio de Janeiro, Itatiaia (Penedo, tributary of Rio Palmital, 22°25'40.0"S, 44°32'46.0"W, el. 584 m; DZRJ; ♂; ♀]. —Dumas and Nessimian, 2012:23 [checklist]. —Paprocki and França, 2014:82 [checklist].

**Distribution.** Brazil.

***riosanjuanensis*** Chamorro-Lacayo, 2003:485 [Type locality: Nicaragua, Río San Juan, Refugio Bartola, small creek, 300 m NW of station, 10°58'N, 84°21'W, el. 35 m; UMSP; ♂]. —Chamorro-Lacayo et al., 2007:46 [checklist].

**Distribution.** Nicaragua.

***sexspinosa*** Flint, 1983a:32 [Type locality: Brazil, Edo. Santa Catarina, Nova Teutonia (27°11'S, 52°23'W); NMNH; ♂]. —Angrisano, 1994:137 [distribution]. —Paprocki et al., 2004:15 [checklist]. —Paprocki and França, 2014:82 [checklist].

**Distribution.** Brazil, Uruguay.

***sinosa*** Ross, 1951b:346 [Type locality: Mexico, Chiapas, Salto de Agua; INHS; ♂]. —Bueno-Soria and Flint, 1978:198 [distribution]. —Bueno-Soria and Barba-Álvarez, 2011:360 [checklist].

**Distribution.** Mexico.

***sinuosa*** Barcelos-Silva, Camargos and Pes *in* Barcelos-Silva et al., 2013:124 [Type locality: Brazil, Espírito Santo, Fundão, Hotel Fazenda Lua Nova; 19°56'02.0"S, 40°24'45.0"W; CZNC; ♂]. —Barcelos-Silva et al., 2012:1279 [as *Cernotina* sp. 2]. —Paprocki and França, 2014:83 [checklist].

**Distribution.** Brazil.

***spinigera*** Flint, 1971c:38 [Type locality: Brazil [Edo. Pará], Tapajós, dicht unterhalb des Zusammenflusses von Rio Juruena mit Rio São Manuel; NMNH; ♂]. —Angrisano, 1994:137 [distribution]. —Sykora, 1998:102 [♂; distribution]. —Paprocki et al., 2004:15 [checklist]. —Barcelos-Silva et al., 2013:124 [distribution]. —Paprocki and França, 2014:83 [checklist].

**Distribution.** Brazil, Uruguay.

***spinosior*** Flint, 1992d:65 [Type locality: Venezuela, Bolivar State, Rio Cuyuni, El Dorado; NMNH; ♂]. —Paprocki et al., 2004:15 [checklist]. —Paprocki and França, 2014:83 [checklist].

**Distribution.** Brazil, Venezuela.

***stannardi*** Ross, 1951b:343 [Type locality: Mexico, Chiapas, Ocosingo Valley, along Rio Santa Cruz, Finca el Real; INHS; ♂]. —Bueno-Soria and Flint, 1978:198 [distribution]. —Bueno-Soria and Barba-Álvarez, 2011:360 [checklist].

**Distribution.** Mexico.

***subapicalis*** Flint, 1971c:35 [Type locality: Brazil [Edo. Amazonas], Rio Marauiá, Endstation vor larger Cachoeira; NMNH; ♂]. —Flint, 1974c:43 [♂; distribution]. —Paprocki et al., 2004:15 [checklist]. —Paprocki and França, 2014:83 [checklist].

**Distribution.** Brazil, Suriname.

***taeniata*** Ross, 1951b:344 [Type locality: Mexico, Chiapas, Huehuetan; INHS; ♂]. —Bueno-Soria and Flint, 1978:198 [distribution]. —Holzenthal, 1988c:58 [distribution]. —Maes, 1999:1188 [checklist]. —Bueno-Soria et al., 2005:75 [distribution]. —Chamorro-Lacayo et al., 2007:46 [checklist]. —Bueno-Soria and Barba-Álvarez, 2011:360 [checklist].

**Distribution.** Costa Rica, Guatemala, Mexico, Nicaragua.

***trispina*** Flint, 1971c:38 [Type locality: Brazil [Edo. Amazonas], Rio Marauiá, Cachoeira, Rio Iripirapí; NMNH; ♂]. —Paprocki et al., 2004:15 [checklist]. —Paprocki and França, 2014:83 [checklist].

**Distribution.** Brazil.

***uara*** Flint, 1971c:36 [Type locality: Brazil [Edo. Amazonas], Rio Marauiá, eine Tagesreise oberhalb A-490 [A-490: 2 days journey above Mission S. Antônio]; NMNH; ♂]. —Flint, 1974c:43 [♂; distribution]. —Paprocki et al., 2004:15 [checklist]. —Paprocki and França, 2014:83 [checklist].

**Distribution.** Brazil, Suriname.

***uncifera*** Ross, 1951b:348 [Type locality: Mexico, Chiapas, Huehuetan; INHS; ♂]. —Bueno-Soria and Flint, 1978:198 [distribution]. —Aguila, 1992:536 [distribution]. —Chamorro-Lacayo et al., 2007:46 [checklist]. —Bueno-Soria and Barba-Álvarez, 2011:360 [checklist]. —Armitage et al., 2015b:4 [checklist]. —Armitage and Cornejo, 2015:192 [checklist].

**Distribution.** Mexico, Nicaragua, Panama.

***unguiculata*** Flint, 1971c:41 [Type locality: Brazil [Edo. Pará], Gebäude der Mission Cururú; NMNH; ♂]. —Paprocki et al., 2004:15 [checklist]. —Paprocki and França, 2014:83 [checklist].

**Distribution.** Brazil.

***verna*** Flint, 1983a:30 [Type locality: Argentina, Pcia. Entre Ríos, Arroyo P. Verne, 4 km N Villa San José; NMNH; ♂]. —Angrisano, 1994:137 [distribution].

**Distribution.** Argentina, Uruguay.

***verticalis*** Flint, 1971c:39 [Type locality: Brazil [Edo. Amazonas], Gebeit Endstation Rio Marauiá, Bergbach II; NMNH; ♂]. —Paprocki et al., 2004:15 [checklist]. —Paprocki and França, 2014:83 [checklist].

**Distribution.** Brazil.

***zanclana*** Ross, 1951b:344 [Type locality: Mexico, Oaxaca, Rancho Monter; INHS; ♂]. —Bueno-Soria and Flint, 1978:198 [distribution]. —Bueno-Soria, 2010:30 [♂].

**Distribution.** Belize, Mexico.

#### Genus *Cyrnellus* Banks [10]

*Cyrnellus* Banks, 1913b:88 [Type species: *Cyrnellus minimus* Banks 1913b, original designation]. —Flint, 1971c:28 [key, Amazonian species].

Like *Cernotina*, *Cyrnellus* is distributed widely across North, Central, and South America, but it is lacking in the Antilles. One species, *Cyrnellus fraternus* (Banks), is known from the northern United States to Argentina making it one of the most widely distributed New World caddisflies.

The larvae of *Cyrnellus fraternus* were described by Flint (1964b) and Wiggins (1996), among others. They construct a silken shelter, open at both ends and covered with fine debris, that is attached to large wood or rock substrate in either lakes or large rivers. Larval gut contents were found to be fine organic particles, rarely with arthropod parts (Wiggins 1996).

***arotron*** Flint, 1971c:32 [Type locality: Brazil [Edo. Pará], Rio Tocantins im hause des Ingenieurs von Rio Impex; NMNH; ♂]. —Flint, 1982c:19 [distribution]. —Angrisano, 1994:138 [distribution]. —Paprocki et al., 2004:16 [checklist]. —Angrisano and Sganga, 2007:18 [♂; distribution]. —Dumas et al., 2010:8 [distribution]. —Paprocki and França, 2014:84 [checklist].

**Distribution.** Argentina, Brazil, Uruguay.

***bifidus*** Flint, 1971c:32 [Type locality: Brazil [Edo. Amazonas], Paraná do Careiro, Divininopolis; NMNH; ♂]. —Flint, 1982c:20 [distribution]. —Paprocki et al., 2004:16 [checklist]. —Dumas et al., 2010:9 [distribution]. —Paprocki and França, 2014:84 [checklist].

**Distribution.** Argentina, Brazil.

***collaris*** Flint, 1971c:31 [Type locality: Brazil [Edo. Amazonas], Rio Solimões, bei Mission S. Rita; NMNH; ♂]. —Angrisano, 1994:139 [distribution]. —Flint, 1996b:391 [distribution]. —Paprocki et al., 2004:16 [checklist]. —Angrisano and Sganga, 2007:18 [♂; distribution]. —Paprocki and França, 2014:84 [checklist].

**Distribution.** Argentina, Brazil, Peru, Uruguay.

***fraternus*** (Banks), 1905:17 [Type locality: United States, Maryland, Plummer’s Island; MCZ; ♀; in *Cyrnus*]. —Flint, 1964b:469 [to *Cyrnellus*; larva; biology]. —Flint, 1971c:29 [♂; synonymy; distribution]. —Flint, 1974c:40 [♂; distribution]. —Bueno-Soria and Flint, 1978:198 [distribution]. —Flint, 1982c:21 [distribution]. —Holzenthal, 1988c:59 [distribution]. —Maes and Flint, 1988:3 [distribution]. —Aguila, 1992:536 [distribution]. —Angrisano, 1994:138 [distribution]. —Johnson et al., 1998:641 [biology]. —Maes, 1999:1188 [checklist]. —Blahnik et al., 2004:5 [distribution]. —Cohen, 2004:76 [distribution]. —Paprocki et al., 2004:16 [checklist]. —Bowles et al., 2007:23 [distribution; biology]. —Chamorro-Lacayo et al., 2007:46 [checklist]. —Dumas et al., 2009:360 [distribution]. —Dumas et al., 2010:9 [distribution]. —Stocks, 2010:165 [wing coupling structure and function]. —Djernaes, 2011:45 [♂; ♀]. —Flint, 2011:106 [checklist]. —Nogueira and Cabette, 2011:352 [distribution]. —Barcelos-Silva et al., 2012:1279 [distribution]. —Manzo et al., 2014:166 [distribution]. —Paprocki and França, 2014:84 [checklist]. —Armitage et al., 2015b:4 [checklist]. —Armitage and Cornejo, 2015:192 [checklist].

—*minimus* Banks, 1913b:88 [Type locality: Brazil Camp 41, 360 Kilometers from Porto Velho, Brazil; MCZ; ♂]. —Flint, 1967c:5 [♂; lectotype]. —Flint, 1971c:29 [to synonymy].

—*marginalis* (Banks), 1930:231 [Type locality: United States, Ohio, Put-in Bay; MCZ; [♂; in *Nyctiophylax*]. —Ross, 1938a:12 [[♂; lectotype]. —Flint, 1964b:469 [to synonymy].

—*zernyi* Mosely, 1934b:142 [Type locality: Brazil, Unt. Amazon, Taperinha b. Santarem; NMW; [♂]. —Ross, 1938a:13 [as synonym of *marginalis*].

**Distribution.** Argentina, Brazil, Costa Rica, Ecuador, El Salvador, Mexico, Nicaragua, Panama, Paraguay, Suriname, Uruguay, U.S.A., Venezuela.

***mammillatus*** Flint, 1971c:30 [Type locality: Brazil [Edo. Amazonas], Lago des Rio Luna am oberen Teil; NMNH; [♂]. —Flint, 1982c:21 [distribution]. —Angrisano, 1994:138 [distribution]. —Flint, 1996b:391 [distribution]. —Blahnik et al., 2004:5 [distribution]. —Cohen, 2004:76 [distribution]. —Paprocki et al., 2004:16 [checklist]. —Angrisano and Sganga, 2007:18 [♂; distribution]. —Calor, 2011:323 [checklist]. —Dumas et al., 2010:9 [distribution]. —Souza et al., 2013a:9 [distribution; as *mammilatus*]. —Paprocki and França, 2014:84 [checklist].

**Distribution.** Argentina, Brazil, Ecuador, Paraguay, Peru, Uruguay.

***misionensis*** Flint, 1983a:33 [Type locality: Argentina, Pcia. Misiones, Arroyo Piray Mini, Rt. 17 W Dos Hermanas; NMNH; [♂].

**Distribution.** Argentina.

***rianus*** Flint, 1983a:33 [Type locality: Argentina, Pcia. Entre Ros, Arroyo P. Verne, 4 km N Villa San José; NMNH; [♂]. —Angrisano, 1994:138 [distribution]. —Angrisano and Sganga, 2007:18 [♂; distribution].

**Distribution.** Argentina, Uruguay.

***risi*** (Ulmer), 1907a:40 [Type locality: Buenos Aires; ZSZMH; [♂; in *Cyrnus*]. —Banks, 1913b:88 [to *Cyrnellus*.]. —Flint, 1971c:31 [[♂; lectotype; distribution]. —Flint, 1974c:41 [♂; distribution]. —Flint, 1982c:22 [distribution]. —Angrisano, 1994:138 [distribution]. —Blahnik et al., 2004:5 [distribution]. —Cohen, 2004:76 [distribution]. —Paprocki et al., 2004:16 [checklist]. —Angrisano and Sganga, 2007:18 [♂; distribution]. —Barcelos-Silva et al., 2012:1279 [distribution]. —Paprocki and França, 2014:84 [checklist].

**Distribution.** Argentina, Brazil, Paraguay, Suriname, Uruguay.

***ulmeri*** Flint, 1971c:32 [Type locality: Brazil [Edo. Pará], Rio Tocantins im hause des Ingenieurs von Rio Impex; NMNH; ♂]. —Flint, 1982c:22 [distribution]. —Flint, 1996b:391 [distribution]. —Angrisano, 1994:138 [distribution]. —Paprocki et al., 2004:16 [checklist]. —Angrisano and Sganga, 2007:19 [♂; distribution]. —Paprocki and França, 2014:85 [checklist]. —Isa Miranda and Rueda Martín, 2014:200 [distribution].

**Distribution.** Argentina, Brazil, Peru, Uruguay.

***zapateriensis*** Chamorro-Lacayo, 2003:485 [Type locality: Nicaragua, Granada, Isla Zapatera, El Bambú, Frente a Lago de Nicaragua, 11°45.829'N, 85°51.991'W, el. 42 m; UMSP; ♂]. — Chamorro-Lacayo et al., 2007:46 [checklist].

**Distribution.** Nicaragua.

#### Genus *Nyctiophylax* Brauer [4]

*Nyctiophylax* Brauer, 1865:419 [Type species: *Nyctiophylax sinensis* Brauer, 1865, original designation]. —Neboiss, 1993:107 [redefinition].

This is a rather heterogenous assemblage of species occuring in most regions of the World, but it is unlikely that these are all congeneric, considering the great differences in genitalic morphology seen among the species. In fact, the monophyly of the genus was not supported in a recent phylogenetic analysis of morphological characters (Chamorro and Holzenthal 2011). However, lacking a world revision, the Neotropical species were retained in *Nyctiophylax*.

The larvae of several North American species have been described (Flint 1964b, Wiggins 1996). Their retreats are similar to those of *Cyrnellus*, a silken shelter, open at both ends and covered with fine debris. It is attached to a large rock, usually in slower flowing reaches in larger streams and rivers. As typical for the family, larvae are mostly carnivorous (Wiggins 1996).

***elongatus*** Flint, 1974c:39 [Type locality: Suriname, Brownsberg, mountain creek near Goldiggers Camp; RNH; ♂].

**Distribution.** Suriname.

***muhnianus*** Navás, 1920c:18 [Type locality: R. Argentina, Santa Fe; collection Navás, now lost?; ♀; quite probably a *Cyrnellus*]. —Navás, 1929b:225 [as *Nyctiophylax argentinus*, undoubtedly a lapsus for *muhnianus*].

**Distribution.** Argentina.

***neotropicalis*** Flint, 1971c:28 [Type locality: Colombia, Cundinamarca, Rio Sumapaz Gorge, east of Melgar; NMNH; ♂]. —Flint, 1974c:39 [♂; distribution]. —Angrisano, 1994:135 [distribution]. —Muñoz-Quesada, 2000:280 [checklist]. —Blahnik et al., 2004:5 [distribution]. —Paprocki et al., 2004:16 [checklist]. —Dumas et al., 2009:360 [distribution]. —Dumas et al., 2010:9 [distribution]. —Dumas and Nessimian, 2012:23 [checklist]. —Barcelos-Silva et al., 2012:1279 [distribution]. —Souza et al., 2013a:9 [distribution]. —Paprocki and França, 2014:85 [checklist].

**Distribution.** Argentina, Brazil, Colombia, Suriname, Uruguay.

***tacuarembo*** Angrisano, 1994:135 [Type locality: Uruguay, Depto. Treinta y Tres, Quebrada de los Cuervos, orilla Yerbal Chico; FHCU; ♂; wings].

**Distribution.** Uruguay.

#### Genus *Polycentropus* Curtis [105]

*Polycentropus* Curtis, 1835:pl. 544 and text [Type species: *Polycentropus irroratus* Curtis, 1835, original designation]. —Flint, 1976:233 [systematics of the *nigriceps* group]. —Flint, 1981b:148 [systematics of the *insularis* and *gertschi* groups]. —Hamilton, 1987:145 [*insularis* group phylogeny]. —Hamilton, 1988:153 [groups of Neotropical species and systematics of *nigriceps* group]. —Hamilton and Holzenthal, 2011:1 [review of Brazilian species].

*Placocentropus* Schmid, 1955a:131 [Type species: *Placocentropus obtusus* Schmid, 1955a, original designation]. — Schmid, 1964:319 [to synonymy].

Until recently, North American and European workers classified this cosmopolitan genus differently. Chamorro and Holzenthal (2011) rectifed the situation by placing several Nearctic species in *Holocentropus* and *Plectrocnemia*, genera long recognized in Europe, but included within a broad definition of *Polycentropus* by Ross (1944). However, the generic limits of *Polycentropus* are still not well circumscribed and the genus is not monophyletic (Chamorro and Holzenthal 2011, Johanson et al. 2012). For the time being, the Neotropical species are retained in *Polycentropus*.

The larvae of many species from Europe, North America and the West Indies have been described (Flint 1964a, Lepneva 1970, Wiggins 1996). The immature stages inhabit a variety of aquatic sites, but mostly slowly flowing backwaters, bottoms of pools in streams, or in lentic habitats. They construct a variety of nets and retreats, most of which collapse into an unrecognizable tangle of silk upon removal from water. The larvae are reported to be strongly predaceous eating chironomids, stoneflies, microcrustacea, and terrestrial litter mites that fall into the water (Townsend and Hildrew 1978). However, Gil et al. (2006) found that gut contents of *Polycentropus joergenseni* in Argentina also included unicellular algae and amorphous matter leading then to conclude that this species also engaged in filtering.

***acanthogaster*** Flint, 1981b:157 [Type locality: Panama, Prov. Chiriqui, Fortuna; NMNH; ♂]. —Aguila, 1992:536 [distribution]. —Armitage et al., 2015b:4 [checklist]. —Armitage and Cornejo, 2015:192 [checklist].

**Distribution.** Panama.

***acinaciformis*** Hamilton and Holzenthal, 2011:40 [Type locality: Brazil, Minas Gerais, Serra do Cipó, Capão da Mata, 19°19.347'S, 43°32.249'W, el. 1170 m; MZUSP; ♂]. —Paprocki and França, 2014:85 [checklist].

**Distribution.** Brazil.

***aguyje*** Angrisano and Sganga, 2009:272 [Type locality: Argentina, Misiones Province, Salto Encantado Provincial Park; MACN; ♂].

**Distribution.** Argentina.

***alatus*** Flint, 1981b:160 [Type locality: Mexico, Edo. Chiapas, Colon, Lagartero, 30 km. northeast of Ciudad Cuauhtemoc; NMNH; ♂]. —Bueno-Soria and Barba-Álvarez, 2011:361 [checklist].

**Distribution.** Mexico.

***aliciae*** Barba-Álvarez and Bueno-Soria, 2005:663 [Type locality: Mexico, Veracruz, Río Jamapa, 5 krn NE Coscomatepec; CNIN; ♂]. —Bueno-Soria and Barba-Álvarez, 2011:361 [checklist].

**Distribution.** Mexico.

***altmani*** Yamamoto, 1967:130 [Type locality: Panama, Fort Kobbe, Canal Zone; INHS; ♂]. —Flint, 1981a:15 [♂; distribution]. —Maes and Flint, 1988:3 [distribution]. —Holzenthal, 1988c:59 [distribution]. —Aguila, 1992:536 [distribution]. —Flint, 1996a:73 [distribution]. —Maes, 1999:1188 [checklist]. —Botosaneanu, 2002:95 [checklist]. —Bueno-Soria and Barba-Álvarez, 2011:361 [checklist]. —Armitage et al., 2015b:4 [checklist]. —Armitage and Cornejo, 2015:192 [checklist].

—*macrostylus* (Flint), 1967b:8 [Type locality: Costa Rica, Golfito; NMNH; ♂; in *Polyplectropus*]. —Flint, 1981a:15 [distribution; to synonymy]. —Chamorro-Lacayo et al., 2007:46 [checklist].

**Distribution.** Costa Rica, Ecuador, Honduras, Nicaragua, Panama, Tobago, Trinidad, Venezuela.

***amphirhamphus*** Hamilton and Holzenthal, 2011:44 [Type locality: Brazil, Rio de Janeiro, Nova Friburgo, municipal water supply, el. 950 m; NMNH; ♂]. —Paprocki and França, 2014:85 [checklist].

**Distribution.** Brazil.

***ancistrus*** Hamilton and Holzenthal, 2011:12 [Type locality: Brazil, São Paulo, Res. Casa Grande, Rib[eirão] Coruja; MZUSP; ♂]. —Paprocki and França, 2014:85 [checklist].

**Distribution.** Brazil.

***ariensis*** Denning and Sykora, 1966:1220 [Type locality: Mexico, Guerrero, Chilapa; CAS; ♂]. —Bueno-Soria and Flint, 1978:199 [distribution]. —Rojas-Ascencio, et al., 2002:377 [distribution].

**Distribution.** Mexico.

***arizonensis*** Banks, 1905:16 [Type locality: United States, Arizona, Huachuca Mountains; MCZ; ♂]. —Ross, 1938a:13 [♂; lectotype]. —Flint, 1967d:166 [distribution]. —Bueno-Soria and Flint, 1978:199 [distribution]. —Blinn and Ruiter, 2006:334 [biology]. —Bueno-Soria et al., 2007:33 [distribution]. —Blinn and Ruiter, 2009a:303 [biology]. —Blinn and Ruiter, 2009b:187 [phenology, distribution]. —Ruiter and Blinn, 2009:5 [♀].

**Distribution.** Mexico, U.S.A.

***aspinosus*** Schmid, 1964:320 [Type locality: Chile (Arauco) Caramavida; NMNH; ♂]. —Flint, 1974e:87 [distribution; as *espinosa*].

**Distribution.** Chile.

***aztecus*** Flint, 1967b:9 [Type locality: Mexico, (Michoacan), Insurgente Morelos National Park, east of Morelia; NMNH; ♂]. —Flint, 1967d:167 [distribution]. —Bueno-Soria and Flint, 1978:199 [distribution]. —Rojas-Ascencio, et al., 2002:377 [distribution]. —Blinn and Ruiter, 2006:334 [biology; distribution]. —Bueno-Soria et al., 2007:33 [distribution]. —Ruiter and Blinn, 2009:5 [checklist]. —Bueno-Soria and Barba-Álvarez, 2011:361 [checklist].

**Distribution.** Mexico, U.S.A.

***azulus*** Flint, 1981b:151 [Type locality: Mexico, Edo. Chiapas, Agua Azul; NMNH; ♂]. —Bueno-Soria and Barba-Álvarez, 2011:361 [checklist].

**Distribution.** Mexico.

***bartolus*** Denning, 1962b:407 [Type locality: Mexico, Baja California, 1.5 miles NW of San Bartolo; CAS; ♂]. —Denning, 1964:133 [checklist]. —Bueno-Soria and Flint, 1978:199 [distribution]. —Bueno-Soria and Barba-Álvarez, 2011:361 [checklist].

**Distribution.** Mexico.

***bellus*** Flint, 1981b:155 [Type locality: Mexico, Edo. Chiapas, Santa Elena, Rio Santo Domingo, 39 km. east of Lagunas Montebello; NMNH; ♂]. —Bueno-Soria and Barba-Álvarez, 2011:361 [checklist].

**Distribution.** Mexico.

***biappendiculatus*** Flint, 1974c:36 [Type locality: Suriname, Tafelberg Expedition, Kappelsavanne; RNH; ♂]. —Hamilton, 1988:177 [distribution].

**Distribution.** Suriname, Venezuela.

***bonus*** Flint, 1981b:157 [Type locality: Belize, Cayo Dist., Río Privassion, Blancaneaux Lodge; NMNH; ♂]. —Bueno-Soria and Barba-Álvarez, 2011:361 [checklist].

**Distribution.** Belize, Mexico.

***boraceia*** Hamilton and Holzenthal, 2011:4 [Type locality: Brazil, São Paulo, Estação Biológica Boracéia, Riberão Coruja; NMNH; ♂]. —Paprocki and França, 2014:85 [checklist].

**Distribution.** Brazil.

***brevicornutus*** Vilarino and Calor, 2015c:114 [Type locality: Brazil, Bahia, Elísio Medrado, Reserva Jequitibá, GAMBA, Córrego Caranguejo, 12°52'12.7’’S, 39°28'32.4’’W, el. 519 m; MZUSP; ♂].

**Distribution.** Brazil.

***caaete*** Hamilton and Holzenthal, 2011:28 [Type locality: Brazil, Santa Catarina, Parque Ecológica Spitzkopf, Rio Caeté above 1st falls, 27°00.35'S, 49°06.70'W, el. 170 m; MZUSP; ♂]. —Paprocki and França, 2014:86 [checklist].

**Distribution.** Brazil.

***cachoeira*** Hamilton and Holzenthal, 2011:46 [Type locality: Brazil, Santa Catarina, Urubici, Cachoeira Avencal, 28°02.839'S, 49°36.997'W, el. 1260 m; MZUSP; ♂]. —Paprocki and França, 2014:86 [checklist].

**Distribution.** Brazil.

***carioca*** Hamilton and Holzenthal, 2011:14 [Type locality: Brazil, Rio de Janeiro, Parque Nacional da Serra dos Órgãos, Rio Beija-flor, 22°27.063'S, 43°00.065'W, el. 1125 m; MZUSP; ♂]. —Paprocki and França, 2014:86 [checklist].

**Distribution.** Brazil.

***carolae*** Hamilton and Holzenthal, 2011:26 [Type locality: Brazil, Rio de Janeiro, km 54, 26 km E Nova Friburgo, el. 410 m; NMNH; ♂]. —Paprocki and França, 2014:86 [checklist].

**Distribution.** Brazil.

***casicus*** Denning, Denning and Sykora, 1966:1222 [Type locality: Mexico, Twenty-four miles west of La Ciudad, Durango; CAS; ♂]. —Flint, 1967d:167 [distribution]. —Bueno-Soria and Flint, 1978:199 [distribution]. —Rojas-Ascencio, et al., 2002:377 [distribution]. —Bueno-Soria and Barba-Álvarez, 2011:361 [checklist].

**Distribution.** Mexico.

***ceciliae*** Flint, 1991:35 [Type locality: Colombia, Dpto. Antioquia, Quebrada Honda, 12 km SW of Fredonia; NMNH; ♂]. —Muñoz-Quesada, 2000:280 [checklist].

**Distribution.** Colombia.

***cheliceratus*** Hamilton and Holzenthal, 2011:22 [Type locality: Brazil, Rio de Janeiro, km 17, 18 km S of Teresópolis, el. 1180 m; NMNH; ♂]. —Paprocki and França, 2014:86 [checklist].

**Distribution.** Brazil.

***chilensis*** Yamamoto, 1966:911 [Type locality: Chile, Valdivia, 8 miles east of Rio Bueno-Soria; CAS; ♂]. —Flint, 1974e:87 [checklist].

**Distribution.** Chile.

***cipoensis*** Hamilton and Holzenthal, 2011:36 [Type locality: Brazil, Minas Gerais, Serra do Cipó, caminho da usina; MZUSP; ♂]. —Paprocki and França, 2014:86 [checklist].

**Distribution.** Brazil.

***clarus*** Flint, 1981b:159 [Type locality: Mexico, Edo. Veracruz, Arroyo Claro, Sierra Sta. Marta, Los Tuxtlas; NMNH; ♂].

**Distribution.** Mexico.

***connatus*** Flint, 1981a:15 [Type locality: Venezuela, Aragua, Rancho Grande; NMNH; ♂].

**Distribution.** Venezuela.

***costaricensis*** Flint, 1967b:8 [Type locality: Costa Rica, Mount Poas; NMNH; ♂]. —Holzenthal, 1988c:59 [distribution]. —Holzenthal and Hamilton, 1988:342 [status; distribution].

**Distribution.** Costa Rica.

***cressae*** Hamilton and Holzenthal, 2005:3 [Type locality: Venezuela, Falcón, P[arque] N[acional] Sierra de San Luis, Río Negro, 11°11.750'N, 69°41.454'W, el. 1371 m; UMSP; ♂; ♀].

**Distribution.** Venezuela.

***criollo*** Botosaneanu, 1980:101 [Type locality: Cuba, Prov. Oriente, Pinares de Mayari; ZMUA; ♂]. —Botosaneanu, 1979:46 [*nomen nudum* (name included in checklist); distribution]. —Hamilton, 1988:167 [♂; distribution; phylogeny]. —Flint, 1996c:16 [checklist]. —Botosaneanu, 2002:95 [checklist]. —Naranjo López and González Lazo, 2005:150 [checklist].

**Distribution.** Cuba.

***cuspidatus*** Flint, 1981b:149 [Type locality: Ecuador, Prov. Pastaza, 16 kms. west of Puyo; NMNH; ♂]. —Flint, 1996b:391 [distribution].

**Distribution.** Ecuador, Peru.

***dentoides*** Yamamoto, 1967:132 [Type locality: Panama, Goofy Lake, Canal Zone; INHS; ♂]. —Holzenthal, 1988c:59 [distribution]. —Aguila, 1992:536 [distribution]. —Armitage et al., 2015b:4 [checklist]. —Armitage and Cornejo, 2015:192 [checklist].

**Distribution.** Costa Rica, Panama.

***dianae*** Barba-Álvarez and Bueno-Soria, 2005:666 [Type locality: Mexico, Nuevo León, Mpio. Zaragoza, El Salto; CNIN; ♂].

**Distribution.** Mexico.

***digitus*** Yamamoto, 1967:131 [Type locality: Panama, Cerro Punta; INHS; ♂]. —Holzenthal, 1988c:59 [distribution]. —Holzenthal and Hamilton, 1988:342 [distribution]. —Aguila, 1992:536 [distribution]. —Armitage et al., 2015b:4 [checklist]. —Armitage and Cornejo, 2015:192 [checklist].

**Distribution.** Costa Rica, Panama.

***domingensis*** Banks, 1941:399 [Type locality: Santo Domingo [Dominican Republic], Loma Rucilla; MCZ; ♂]. —Flint, 1967c:6 [♂; lectotype]. —Flint, 1968b:80 [checklist]. —Flint, 1976:237 [♂; distribution]. —Hamilton, 1988:175 [♂; distribution; phylogeny]. —Flint and Pérez-Gelabert, 1999:43 [checklist]. —Botosaneanu, 2002:95 [checklist]. —Flint and Sykora, 2004:54 [distribution]. —Pérez-Gelabert, 2008:302 [checklist].

**Distribution.** Dominican Republic.

***doronca*** Denning and Sykora, 1966:1220 [Type locality: Mexico, Almilinta; CAS; ♂]. —Bueno-Soria and Flint, 1978:199 [distribution].

**Distribution.** Mexico.

***encera*** Denning and Sykora, in Denning, 1971:205 [Type locality: Mexico, Veracruz, El Encero; CAS; ♂; ♀]. —Bueno-Soria and Flint, 1978:199 [distribution]. —Bueno-Soria and Barba-Álvarez, 2011:361 [checklist].

**Distribution.** Mexico.

***exsertus*** Flint, 1981b:155 [Type locality: Ecuador, Prov. Pastaza, 16 kms. west of Puyo; NMNH; ♂].

**Distribution.** Ecuador.

***fasthi*** Holzenthal and Hamilton, 1988:332 [Type locality: Costa Rica, Guanacaste, Parque Nacional Rincón de la Vieja, Río Negro, 10.765°N, 85.313°W; NMNH; ♂].

**Distribution.** Costa Rica.

***fluminensis*** Hamilton and Holzenthal, 2011:16 [Type locality: Brazil, Rio de Janeiro, km 17, 18 km S of Teresópolis, el. 1180 m; NMNH; ♂]. —Dumas and Nessimian, 2012:23 [checklist]. —Barcelos-Silva et al., 2012:1279 [distribution]. —Paprocki and França, 2014:86 [checklist].

**Distribution.** Brazil.

***fortispinus*** Holzenthal and Hamilton, 1988:335 [Type locality: Costa Rica, Guanacaste, Parque Nacional Rincón de la Vieja, Río Negro, 10.765°N, 85.313°W; NMNH; ♂]. —Maes, 1999:1188 [checklist]. —Chamorro-Lacayo et al., 2007:46 [checklist].

**Distribution.** Costa Rica, Nicaragua.

***fortunus*** Flint, 1981b:155 [Type locality: Panama, Prov. Chiriqui, Fortuna; NMNH; ♂]. —Holzenthal, 1988c:59 [distribution]. —Holzenthal and Hamilton, 1988:342 [distribution]. —Aguila, 1992:536 [distribution]. —Armitage et al., 2015b:4 [checklist]. —Armitage and Cornejo, 2015:192 [checklist].

**Distribution.** Costa Rica, Panama.

***froehlichi*** Hamilton and Holzenthal, 2011:10 [Type locality: Brazil, Santa Catarina, Morro da Igreja, Cachoeira Veu da Noiva, 28°04.595'S, 49°31.090'W, el. 1300 m; MZUSP; ♂]. —Paprocki and França, 2014:86 [checklist].

**Distribution.** Brazil.

***galharada*** Hamilton and Holzenthal, 2011:6 [Type locality: Brazil, São Paulo, Estação Biológica Boracéia, Rio Galharada, 22°41.662'S, 45°27.783'W, el. 1530 m; MZUSP; ♂]. —Paprocki and França, 2014:87 [checklist].

**Distribution.** Brazil.

***garfio*** Chamorro-Lacayo, 2003:488 [Type locality: Costa Rica, Alajuela, Río Agrio, ca. 3.5 km NE Bajos del Toro, 10.243°N, 84.279°W, el. 1290 m; UMSP; ♂].

**Distribution.** Costa Rica.

***gertschi*** Denning, 1950:100 [Type locality: [United States], Arizona, Oak Creek Canyon; AMNH; ♂]. —Blinn and Ruiter, 2006:334 [biology]. —Bueno-Soria et al., 2007:33 [distribution]. —Blinn and Ruiter, 2009a:303 [biology]. —Blinn and Ruiter, 2009b:187 [phenology, distribution]. —Ruiter and Blinn, 2009:5 [♀].

**Distribution.** Mexico, U.S.A.

***giovannae*** Barba-Álvarez and Bueno-Soria, 2005:668 [Type locality: Mexico, Oaxaca, route 175, La Esperanza, ca Valle Nacional; CNIN; ♂].

**Distribution.** Mexico.

***graciosa*** Hamilton and Holzenthal, 2011:12 [Type locality: Brazil, Paraná, Rio Cascata, Graciosa, road to Morretes, 25°20.214'S, 48°53.971'W, el. 750 m; MZUSP; ♂]. —Paprocki and França, 2014:87 [checklist].

**Distribution.** Brazil.

***guatemalensis*** Flint, 1967b:9 [Type locality: Guatemala, (Suchitepequez), Finca Mocá; NMNH; ♂]. —Bueno-Soria and Flint, 1978:199 [distribution]. —Maes and Flint, 1988:3 [distribution]. —Maes, 1999:1189 [checklist]. —Chamorro-Lacayo et al., 2007:47 [checklist]. —Bueno-Soria and Barba-Álvarez, 2011:361 [checklist].

**Distribution.** Guatemala, Mexico, Nicaragua.

***halidus*** Milne, 1936:86 [Type locality: United States, N. Mex., Hot Spngs; MCZ; ♂]. —Flint, 1967d:167 [distribution]. —Bueno-Soria and Flint, 1978:199 [distribution]. —Baumgardner and Bowles, 2005:11 [distribution]. —Bueno-Soria et al., 2007:33 [distribution]. —Blinn and Ruiter, 2009a:305 [biology]. —Blinn and Ruiter, 2009b:187 [phenology, distribution]. —Ruiter and Blinn, 2009:5 [checklist].

**Distribution.** Mexico, U.S.A.

***hamiferus*** Flint, 1981b:153 [Type locality: El Salvador, Dept. Santa Ana, north of Metapan, Cerro Miramundo; NMNH; ♂].

**Distribution.** El Salvador.

***hamiltoni*** Chamorro-Lacayo, 2003:488 [Type locality: Nicaragua, Jinotega, Cerro Mazu, l4°33'N, 85°07'W, el. 220 m; UMSP; ♂]. —Chamorro-Lacayo et al., 2007:47 [checklist].

**Distribution.** Nicaragua.

***holzenthali*** Bueno-Soria and Hamilton, 1986:300 [Type locality: Mexico, Chiapas, Tributario del Río Teapa situado en el carretera 195 a 3 km al N. de Ixhuatán; NMNH; ♂]. —Maes, 1999:1189 [checklist]. —Chamorro-Lacayo et al., 2007:47 [checklist]. —Bueno-Soria and Barba-Álvarez, 2011:361 [checklist].

**Distribution.** Nicaragua, Mexico.

***ibarrai*** Barba-Álvarez and Bueno-Soria, 2005:666 [Type locality: Mexico, Hidalgo, Hixtlahuaco, Hotel Campestre Conchita, 20°53.025'N, 98°42.140'W, el. 1400 m; CNIN; ♂].

**Distribution.** Mexico.

***insularis*** Banks, 1938:302 [Type locality: Grenada, Grand Etang; MCZ; ♂]. —Flint, 1967c:6 [♂; lectotype]. —Flint, 1968b:24 [♂; ♀; larva; distribution; biology]. —Hamilton, 1988:179 [♂; distribution; phylogeny]. —Flint and Sykora, 1993:53 [distribution]. —Botosaneanu, 2002:96 [checklist]. —Botosaneanu and Thomas, 2005:51 [distribution].

**Distribution.** Dominica, Grenada, Martinique.

***inusitatus*** Hamilton and Holzenthal, 2011:48 [Type locality: Brazil, Rio de Janeiro, Brejo da Lapa, Itatiaia; NMNH; ♂]. —Dumas and Nessimian, 2012:23 [checklist]. —Paprocki and França, 2014:87 [checklist].

**Distribution.** Brazil.

***itatiaia*** Hamilton and Holzenthal, 2011:30 [Type locality: Brazil, Rio de Janeiro, Parque Nacional do Itatiaia, trib. to Rio Taquaral, 22°26.688'S, 44°36.464'W, el. 1320 m; MZUSP; ♂]. —Dumas and Nessimian, 2012:24 [checklist]. —Paprocki and França, 2014:87 [checklist].

**Distribution.** Brazil.

***jamaicensis*** Flint, 1968a:25 [Type locality: Jamaica, St. Andrew, Hope River near Newcastle at mile post 16.5; NMNH; ♂; ♀; larva; pupa; biology]. —Flint, 1968b:80 [checklist]. —Hamilton, 1988:171 [♂; distribution; phylogeny]. —Botosaneanu, 2002:96 [checklist].

**Distribution.** Jamaica.

***jeldesi*** Flint, 1976:237 [Type locality: Dominican Republic, Convento, 12 km south of Constanza; NMNH; ♂]. —Hamilton, 1988:173 [♂; distribution; phylogeny]. —Flint and Pérez-Gelabert, 1999:43 [checklist]. —Botosaneanu, 2002:96 [checklist]. —Flint and Sykora, 2004:54 [distribution]. —Pérez-Gelabert, 2008:302 [checklist].

—*species* 2 Flint, 1976:239 [Locality: Dominican Republic, Convento, 12 km south of Constanza; NMNH; as possible ♀ of *jeldesi*]. —Flint and Sykora, 2004:54 [to synonymy].

—“sp. A” Botosaneanu, 1996:15 [♀]. —Flint and Sykora, 2004:54 [to synonymy].

**Distribution.** Dominican Republic.

***joergenseni*** Ulmer, 1909b:75 [Type locality: Argentina, Mendoza, Pedregal; ZSZMH; ♂; as *Jörgenseni*]. —Weidner, 1964:91 [lectotype]. —Flint and Reyes, 1991:480 [distribution; synonymy]. —Flint, 1996b:391 [distribution]. —Mangeaud, 1996:154 [distribution]. —Valverde, 1996:65 [♂; ♀; larva; pupa; biology]. —Muñoz-Quesada, 2000:281 [checklist]. —Cohen, 2004:76 [distribution]. —Gil et al., 2006:207 [biology]. —Isa Miranda and Rueda Martín, 2014:200 [distribution].

—*colombiensis* Banks, 1910:160 [Type locality: Cañon del Monte Tolima, Colombia; MCZ; ♀]. —Flint, 1967c:160 [♀]. —Flint and Reyes, 1991:480 [to synonymy].

—*anomalus* Navás, 1923a:201 [Type locality: Republica Argentina, Cordoba, Alta Gracia; MACN; ♂]. —Flint and Reyes, 1991:481 [to synonymy].

**Distribution.** Argentina, Bolivia, Colombia, Ecuador, Peru, Venezuela.

***lingulatus*** Flint, 1981b:151 [Type locality: Panama, Prov. Chiriqui, Fortuna; NMNH; ♂]. —Aguila, 1992:536 [distribution]. —Armitage et al., 2015b:4 [checklist]. —Armitage and Cornejo, 2015:192 [checklist].

**Distribution.** Panama.

***longispinosus*** Schmid, 1958b:199 [Type locality: Bolivie, (Cochab.), Incachaca; NMNH; ♂].

**Distribution.** Bolivia.

***marcanoi*** Flint, 1976:238 [Type locality: Dominican Republic, Dajabon Prov., 13 km south of Loma de Cabrere; NMNH; ♂; ♀]. —Hamilton, 1988:171 [♂; distribution; phylogeny]. —Flint and Pérez-Gelabert, 1999:43 [checklist]. —Botosaneanu, 2002:96 [checklist]. —Flint and Sykora, 2004:54 [distribution]. —Pérez-Gelabert, 2008:302 [checklist].

**Distribution.** Dominican Republic.

***mathisi*** Hamilton, 1986:731 [Type locality: Cuba, Pinar del Rio Prov., Soroa; NMNH; ♂]. —Hamilton, 1988:170 [♂; distribution; phylogeny]. —Botosaneanu, 2002:96 [checklist]. —Naranjo López and González Lazo, 2005:150 [checklist].

**Distribution.** Cuba.

***mayanus*** Flint, 1981b:151 [Type locality: Mexico, Edo. Chiapas, Río Chacamax, Palenque; NMNH; ♂]. —Holzenthal, 1988c:59 [distribution]. —Holzenthal and Hamilton, 1988:343 [distribution]. —Maes, 1999:1189 [checklist]. —Bueno-Soria et al., 2005:75 [distribution]. —Chamorro-Lacayo et al., 2007:47 [checklist]. —Bueno-Soria and Barba-Álvarez, 2011:361 [checklist].

**Distribution.** Costa Rica, Mexico, Nicaragua.

***meridiensis*** Flint, 1981b:153 [Type locality: Venezuela, Edo. Merida, 4 km. south of Santo Domingo; NMNH; ♂].

**Distribution.** Venezuela

***mexicanus*** (Banks), 1901:369 [Type locality: Mexico, D. F., Tacubaya; MCZ; ♂; as *Hydropsyche mexicana*]. —Flint, 1967c:6 [♂; to *Polycentropus*]. —Bueno-Soria and Flint, 1978:199 [distribution]. —Bueno-Soria and Barba-Álvarez, 2011:361 [checklist].

**Distribution.** Mexico.

***minero*** Hamilton and Holzenthal, 2011:24 [Type locality: Brazil, Minas Gerais: Serra do Cipo, Rio Capivara; NMNH; ♂]. —Paprocki and França, 2014:87 [checklist].

**Distribution.** Brazil.

***mixteco*** Barba-Álvarez and Bueno-Soria, 2005:665 [Type locality: Mexico, Oaxaca, La Esperanza, route 175, ca. Valle Nacional; CNIN; ♂].

**Distribution.** Mexico.

***neblinensis*** Hamilton and Holzenthal, 2005:5 [Type locality: Venezuela, Amazonas, Cerro de la Neblina, Camp II, 00°49'N, 65°59'W, el. 2100 m; UMSP; ♂; ♀].

**Distribution.** Venezuela.

***nebulosus*** Holzenthal and Hamilton, 1988:336 [Type locality: Costa Rica, Puntarenas, Río Bellavista, ca. 1.5 km NW Las Alturas, 8.951°N, 82.846°W; NMNH; ♂].

**Distribution.** Costa Rica.

***nigriceps*** Banks, 1938:301 [Type locality: Cuba, Soledad near Cienfuegos; MCZ; ♂]. —Flint, 1967c:6 [♂; lectotype]. —Flint, 1968b:80 [checklist]. —Flint, 1976:235 [♂; distribution]. —Botosaneanu, 1979:46 [distribution]. —Kumanski, 1987:11 [distribution]. —Hamilton, 1988:168 [♂; distribution; phylogeny]. —Flint, 1996c:16 [checklist]. —Botosaneanu, 2002:96 [checklist]. —Naranjo López and González Lazo, 2005:150 [checklist].

—*rosarius* Kingsolver, 1964:257 [Type locality: Cuba, Pinar del Rio Province, Rancho Mundito, Sierra del Rosario; INHS; ♂]. —Flint, 1976:235 [to synonymy].

—*species* 1 Flint, 1976:239 [Locality: Cuba, Pinar del Rio, S. Cajalbana; INHS; as probable ♀ of *nigriceps*].

**Distribution.** Cuba.

***obtusus*** (Schmid), 1955a:133 [Type locality: Chile (Prov. de Talca), Tonlemo; NMNH; ♂; in *Placocentropus*]. —Schmid, 1964:319 [♂; to *Polycentropus*]. —Flint, 1974e:87 [checklist].

**Distribution.** Chile.

***palmitus*** Flint, 1967d:167 [Type locality: Mexico, (Sinaloa), 8 miles west of El Palmito; CNC; ♂]. —Bueno-Soria and Flint, 1978:199 [distribution]. —Bueno-Soria and Barba-Álvarez, 2011:361 [checklist].

**Distribution.** Mexico.

***paprockii*** Hamilton and Holzenthal, 2011:49 [Type locality: Brazil, Minas Gerais, Parque Estadual do Rio Preto, Rio Preto, 18°06.993'S, 43°20.373'W, el. 650 m; MZUSP; ♂]. —Paprocki and França, 2014:87 [checklist].

**Distribution.** Brazil.

***pedernales*** Flint and Sykora, 2004:54 [Type locality: Dominican Republic, Pedernales Province, along Rio Mulito, 13 km N Pedemales, 18°09'N, 71°46'W, el. 230 m; CMNH; ♂; ♀]. —Botosaneanu, 2002:96 [checklist; as unpublished species]. —Pérez-Gelabert, 2008:302 [checklist].

—sp. B Botosaneanu, 1996:15 [♀]. —Flint and Sykora, 2004:54 [to synonymy].

**Distribution.** Dominican Republic.

***phraterus*** Chamorro-Lacayo, 2003:491 [Type locality: Costa Rica, Alajuela: Reserva Forestal San Ramón, Río San Lorencito and tributaries, 10.216°N, 84.606°W, el. 980 m; UMSP; ♂].

**Distribution.** Costa Rica.

***picana*** Ross, 1947:136 [Type locality: Mexico, Tamaulipas, Hacienda Santa Engracia; INHS; ♂]. —Flint, 1967d:167 [distribution]. —Bueno-Soria and Flint, 1978:199 [distribution]. —Bowles et al., 2007:23 [distribution; biology].

**Distribution.** Mexico, U.S.A.

***quadriappendiculatus*** Schmid, 1964:321 [Type locality: Chile (Aysen) Puerto Cisnes; NMNH; ♂]. —Flint, 1974e:87 [checklist].

**Distribution.** Chile.

***quadricuspidis*** Hamilton and Holzenthal, 2005:7 [Type locality: Ecuador, Zamora-Chinchipe, 30 km E Loja, el. 2000 m; NMNH; ♂; ♀].

**Distribution.** Ecuador.

***quadrispinosus*** Schmid, 1964:321 [Type locality: Chile (Malleco) Thermas de Tolhuaca; NMNH; ♂]. —Flint, 1974e:87 [checklist].

**Distribution.** Chile.

***rosalysae*** Hamilton and Holzenthal, 2011:42 [Type locality: Brazil, São Paulo, Parque Estadual de Campos do Jordão, Rio Galharada, 22°41.662'S, 45°27.783'W, el. 1530 m; MZUSP; ♂]. —Dumas and Nessimian, 2012:24 [checklist]. —Paprocki and França, 2014:87 [checklist].

**Distribution.** Brazil.

***santateresae*** Hamilton and Holzenthal, 2011:32 [Type locality: Brazil, Espírito Santo, 15 km SE Santa Teresa, Fazenda Santa Clara, el. 460 m; NMNH; ♂]. —Barcelos-Silva et al., 2012:1279 [distribution]. —Paprocki and França, 2014:88 [checklist].

**Distribution.** Brazil.

***sarandi*** Angrisano, 1994:132 [Type locality: Uruguay, Depto. Cerro Largo, Sarandí del Quebracho; FHCU; ♂; wings].

**Distribution.** Uruguay.

***silex*** Hamilton and Holzenthal, 2005:9 [Type locality: Ecuador, Pichincha, ca. 84 km SW Quito, Chiriboga Rd., el. 1400 m; NMNH; ♂; ♀].

**Distribution.** Ecuador.

***soniae*** Hamilton and Holzenthal, 2011:20 [Type locality: Brazil, Paraná, Rio Mãe Catira, 10 km N Porto de Cima, 25°21.821'S, 48°52.473'W, el. 200 m; MZUSP; ♂]. —Paprocki and França, 2014:88 [checklist].

**Distribution.** Brazil.

***spicatus*** Yamamoto, 1967:131 [Type locality: Panama, Cerro Punta; INHS; ♂]. —Holzenthal, 1988c:60 [distribution]. —Holzenthal and Hamilton, 1988:343 [distribution]. —Aguila, 1992:536 [distribution]. —Armitage et al., 2015b:4 [checklist]. —Armitage and Cornejo, 2015:192 [checklist].

**Distribution.** Costa Rica, Panama.

***surinamensis*** Flint, 1974c:37 [Type locality: Suriname, Nassau Mountains, km 11.2; RNH; ♂].

**Distribution.** Suriname.

***tripui*** Hamilton and Holzenthal, 2011:18 [Type locality: Brazil, Minas Gerais, Estação Ecológica de Tripuí, Córrego Tiririca, 20°23.009'S, 43°33.237'W; MZUSP; ♂]. —Paprocki and França, 2014:88 [checklist].

**Distribution.** Brazil.

***tuberculatus*** Flint, 1983a:26 [Type locality: Chile, Pcia. Maule, Alto Tregualemu, ~20 km SE Chovelln; NMNH; ♂].

**Distribution.** Chile.

***turquino*** Botosaneanu, 1980:102 [Type locality: Cuba, Prov. Oriente, Pico Cuba, Massif Turquino, Sierra Maestra; ZMUA; ♂]. —Botosaneanu, 1979:46 [*nomen nudum* (name included in checklist); distribution]. —Hamilton, 1988:174 [♂; distribution; phylogeny]. —Flint, 1996c:16 [checklist]. —Botosaneanu, 2002:96 [checklist]. —Naranjo López and González Lazo, 2005:150 [checklist]. —López del Castillo et al., 2007:171 [distribution; seasonal abundance].

**Distribution.** Cuba.

***unispinus*** Flint, 1991:35 [Type locality: Colombia, Dpto. Antioquia, Quebrada Espadera, 7 km E Medellín, road to Santa Elena; NMNH; ♂]. —Muñoz-Quesada, 2000:281 [checklist].

**Distribution.** Colombia.

***urubici*** Holzenthal and Almeida, 2003:26 [Type locality: Brazil, Paraná, Telêmaco Borba, Reserva Samuel Klabin, 24°17'S, 50°37'W, el. 750 m; UFPR; ♂]. —Paprocki et al., 2004:16 [checklist]. —Dumas et al., 2010:9 [distribution]. —Dumas and Nessimian, 2012:24 [checklist]. —Paprocki and França, 2014:88 [checklist].

**Distribution.** Brazil.

***valdiviensis*** Flint, 1983a:25 [Type locality: Chile, Pcia. Valdivia, S. Valdivia; NMNH; ♂].

**Distribution.** Chile.

***vanderpooli*** Flint, 1976:237 [Type locality: Dominican Republic, Convento, 12 km south of Constanza; NMNH; ♂]. —Hamilton, 1988:176 [♂; distribution; phylogeny]. —Botosaneanu, 1996:15 [distribution]. —Flint and Pérez-Gelabert, 1999:43 [checklist]. —Botosaneanu, 2002:96 [checklist]. —Flint and Sykora, 2004:55 [distribution]. —Pérez-Gelabert, 2008:302 [checklist].

**Distribution.** Dominican Republic.

***veracruzensis*** Flint, 1981b:151 [Type locality: Mexico, Edo. Veracruz, near Huatusco; NMNH; ♂].

**Distribution.** Mexico.

***verruculus*** Hamilton and Holzenthal, 2011:38 [Type locality: Brazil, Minas Gerais, Rio Guanhães, downstream from Salto Grande dam, 19°06.289'S, 42°42.635'W; MZUSP; ♂]. —Paprocki and França, 2014:88 [checklist].

**Distribution.** Brazil.

***virginiae*** Hamilton and Holzenthal, 2011:34 [Type locality: Brazil, Minas Gerais, Córrego da Serra de Ouro Fino, Vale do Tropeiro, 20°12.371'S, 43°38.581'W, el. 1000 m; MZUSP; ♂]. —Paprocki and França, 2014:88 [checklist].

**Distribution.** Brazil.

***volcanus*** Holzenthal and Hamilton, 1988:338 [Type locality: Costa Rica, San José, Parque Nacional Braulio Carillo, 6.2 km NE administration building, 10.09°N, 83.97°W; NMNH; ♂].

**Distribution.** Costa Rica.

***zanclus*** Flint, 1981b:155 [Type locality: Guatemala, Dept. El Quiche, 7.3 km. south of Chichicastenago; NMNH; ♂].]. —Chamorro-Lacayo et al., 2007:47 [checklist]. —Bueno-Soria and Barba-Álvarez, 2011:361 [checklist].

**Distribution.** Guatemala, Nicaragua.

***zaneta*** Denning, 1947a:660 [Type locality: Puerto Rico, Luquillo; CAS; ♂; ♀]. —Flint, 1964a:32 [♂; ♀; larva; pupa; distribution; biology]. —Flint, 1968b:80 [checklist]. —Hamilton, 1988:169 [♂; distribution; phylogeny]. —Botosaneanu, 2002:96 [checklist].

**Distribution.** Puerto Rico.

***zurqui*** Holzenthal and Hamilton, 1988:340 [Type locality: Costa Rica, San José, Parque Nacional Braulio Carillo, park headquarters [Estación Zurquí], 10.059°N, 84.01°W; NMNH; ♂].

**Distribution.** Costa Rica.

#### Genus *Polyplectropus* Ulmer [96]

*Polyplectropus* Ulmer, 1905a:103 [Type species: *Polyplectropus flavicornis* Ulmer, 1905a, by monotypy]. —Flint, 1968b:20 [systematics of the genus]. —Bueno-Soria, 1990:357 [revision of Mexican and Central American species]. —Chamorro and Holzenthal, 2010:44 [revision of New World species; phylogeny].

*Ecnomodes* Ulmer, 1911:17 [Type species: *Ecnomodes buchwaldi* Ulmer, 1911, by monotypy, preoccupied]. —Flint, 1968b:21 [to synonymy].

*Cordillopsyche* Banks, 1913a:238 [Type species: *Cordillopsyche costalis* Banks, 1913a, by monotypy]. —Flint, 1967c:6 [to synonymy].

*Ecnomodellina* Ulmer, 1962:5 [Type species: *Ecnomodes buchwaldi* Ulmer, 1911, replacement name for *Ecnomodes*]. —Flint, 1968b:21 [to synonymy].

As currently defined, this genus has representatives in Africa, the Orient, and as far south as New Zealand as well as the American tropics. Chamorro and Holzenthal (2010) reviewed the New World species and proposed a new classification, recognizing 10 species groups. The distribution of New World species is largely Neotropical, with most restricted to the Mexican and Brazilian subregions (Chamorro and Holzenthal 2010). In a phylogenetic assessment of the genera of Polycentropodidae, *Polyplectropus* was not recovered as globally monophyletic, although the New World species did form a monophyletic clade within the family (Chamorro and Holzenthal 2011).

The larvae of a species, probably *Polyplectropus charlesi* (described as Genus C, Flint 1964b), was the first described in the genus, and subsequently the larva of the Lesser Antillean *Polyplectropus bredini* was made known (Flint 1968b). The silken shelters, open at both ends, are attached to rocks under water. The larvae are predators (Flint 1964b, Wiggins 1996).

***adamsae*** Chamorro and Holzenthal, 2010:77 [Type locality: Peru, Madre de Dios, Manú [Biosphere Reserve], Pakitza Bio[logical] Sta[tion], Quebrada Trompetero, 11°56'39"S, 71°16'59"W, el. 350 m; NMNH; ♂].

**Distribution.** Peru.

***alatespinus*** Chamorro and Holzenthal, 2010:52 [Type locality: Brazil, Minas Gerais, Parque Estadual do Ibitipoca, Córrego dos Macacos, 21°42'33"S, 44°53'36"W, el. 1360 m; MZUSP; ♂; ♀]. —Calor, 2011:323 [checklist]. —Dumas and Nessimian, 2012:24 [checklist]. —Paprocki and França, 2014:88 [checklist].

**Distribution.** Brazil.

***alienus*** Bueno-Soria, 1990:373 [Type locality: Mexico, Chiapas, Ocosingo, Finca El Real, Río Santa Cruz; INHS; ♂]. —Chamorro and Holzenthal, 2010:47 [diagnosis; ♂; ♀; distribution]. —Bueno-Soria and Barba-Álvarez, 2011:361 [checklist].

**Distribution.** Bolivia, Mexico.

***alleni*** (Yamamoto), 1967:127 [Type locality: Costa Rica, Puerto Viejo; INHS; ♂; in *Polycentropus*]. —Flint, 1968b:21 [to *Polyplectropus*]. —Holzenthal, 1988c:60 [distribution]. —Bueno-Soria, 1990:394 [♂; distribution]. —Blahnik et al., 2004:5 [distribution]. —Paprocki et al., 2004:16 [checklist]. —Chamorro and Holzenthal, 2010:146 [diagnosis; ♂]. —Paprocki and França, 2014:89 [checklist].

**Distribution.** Brazil, Costa Rica.

***amazonicus*** Chamorro and Holzenthal, 2010:54 [Type locality: Venezuela, Amazonas, Cerro de la Neblina, Camp III, 00°56'10"N, 66°03'53"W, el. 1820 m; NMNH; ♂].

**Distribution.** Venezuela.

***anchorus*** Vilarino and Calor, 2015c:116 [Type locality: Brazil, Bahia, Varzedo, Fazenda Baixa Grande, Riacho Cai Camarão, 12°57'38.7"S, 39°26'54.2"W, el. 254 m; MZUSP; ♂].

**Distribution.** Brazil.

***andinensis*** Chamorro and Holzenthal, 2010:148 [Type locality: Bolivia, Chuquis[aca], Monteagudo, el. 1300 m; NMNH; ♂].

**Distribution.** Argentina, Bolivia.

***annulicornis*** Ulmer, 1905b:91 [Type locality: Brazil, Rio Gr.[Grande] do Sul; NMW; ♀]. —Flint, 1966a:4 [lectotype; ♂; ♀]. —Paprocki et al., 2004:16 [checklist]. —Chamorro and Holzenthal, 2010:56 [diagnosis; ♂; ♀; distribution]. —Dumas and Nessimian, 2012:24 [checklist]. —Paprocki and França, 2014:89 [checklist].

**Distribution.** Brazil.

***auriplicatus*** Vilarino and Calor, 2015c:119 [Type locality: Brazil, Bahia, Santa Teresinha, Pedra Branca, Córrego das torres, 12°51'00.3"S, 39°28'46.8"W, el. 754 m; MZUSP; ♂].

**Distribution.** Brazil.

***banksianus*** Flint, 1971c:27 [Type locality: Brazil [Edo. Amazonas], Manaus; MCZ; ♂]. —Banks, 1913b:88 [as *Ecnomodes buchwaldi*, misidentification]. —Paprocki et al., 2004:16 [checklist]. —Chamorro and Holzenthal, 2010:149 [diagnosis; ♂]. —Paprocki and França, 2014:89 [checklist].

**Distribution.** Brazil.

***beccus*** Hamilton and Holzenthal, 2005:11 [Type locality: Venezuela, T. F. Amazonas [=Estado Amazonas], Puerto Ayacucho (40 km S), El Tobogan, Caño Coromoto; NMNH; ♂]. —Chamorro and Holzenthal, 2010:224 [diagnosis; ♂].

**Distribution.** Venezuela.

***beutelspacheri*** Bueno-Soria, 1990:378 [Type locality: Mexico, Guerrero, Zihuaquio, km 95, Carretera 94, Coyuca-Zihuatanejo; IBUNAM; ♂]. —Chamorro and Holzenthal, 2010:226 [diagnosis; ♂].

**Distribution.** Mexico.

***blahniki*** Chamorro and Holzenthal, 2010:193 [Type locality: Venezuela, Falcón, P. N. Cueva de la Quebrada del Toro, 10°49'35"N, 69°07'59"W, el. 530 m; UMSP; ♂; ♀]. —Chamorro and Holzenthal, 2010:193 [misidentification of *recurvatus* (Yamamoto) in Flint, 1981:16].

**Distribution.** Venezuela.

***bolivianus*** Chamorro and Holzenthal, 2010:151 [Type locality: Bolivia, La Paz, Río Coroico, el. 1200 m; NMNH; ♂].

**Distribution.** Bolivia.

***brachyscolus*** Flint, 1971c:27 [Type locality: Brazil [Edo. Amazonas], Rio Marauiá, Endstation vor langer Cachoeira; NMNH; ♂]. —Flint, 1974c:33 [♂; distribution]. —Paprocki et al., 2004:16 [checklist]. —Chamorro and Holzenthal, 2010:153 [diagnosis; ♂; distribution]. —Paprocki and França, 2014:89 [checklist].

**Distribution.** Brazil, Guyana, Suriname.

***brasilensis*** Chamorro and Holzenthal, 2010:78 [Type locality: Brazil, Rio de Janeiro, Nova Friburgo, mun[icipal] water supply, el. 950 m; MZUSP; ♂; ♀]. —Calor, 2011:323 [checklist]. —Dumas and Nessimian, 2012:24 [checklist]. —Paprocki and França, 2014:89 [checklist].

**Distribution.** Brazil.

***bravoae*** Bueno-Soria, 1990:396 [Type locality: Costa Rica, Guanacaste, Río Tempisquito, ca. 3 km S de Ruta 1, 10.790°N, 89.552°W; NMNH; ♂]. —Maes, 1999:1189 [checklist]. —Chamorro-Lacayo et al., 2007:47 [checklist]. —Chamorro and Holzenthal, 2010:154 [diagnosis; ♂; distribution].

**Distribution.** Belize, Costa Rica, Honduras, Mexico, Nicaragua.

***brborichorum*** Chamorro and Holzenthal, 2010:157 [Type locality: Ecuador, Past[aza], Est[ación] Fluv[ial] Métrica, Puyo (27 km N); NMNH; ♂; ♀].

**Distribution.** Ecuador.

***bredini*** Flint, 1968b:21 [Type locality: Dominica, Pont Casse, 1.3 miles east; NMNH; ♂; ♂; biology; larva]. —Malicky, 1983:264 [distribution]. —Botosaneanu, 1988:219 [distribution]. —Flint and Sykora, 1993:49 [checklist]. —Botosaneanu, 2002:96 [checklist]. —Botosaneanu and Thomas, 2005:51 [distribution]. —Chamorro and Holzenthal, 2010:80 [diagnosis; ♂; ♀].

**Distribution.** Dominica, Grenada, Guadeloupe, Martinique, St. Lucia.

***buchwaldi*** (Ulmer), 1911:18 [Type locality: Ecuador; ZSZMH; ♂; in *Ecnomodes*, abdomen lost]. —Ulmer, 1962:5 [to *Ecnomodellina*]. —Weidner, 1964:74 [♂; lectotype]. —Flint, 1968b:21 [to *Polyplectropus*]. —Chamorro and Holzenthal, 2010:158 [*nomen dubium*].

**Distribution.** Ecuador.

***canastra*** Rocha, Dumas, and Nessimian, 2016a:392 [Type locality: Brazil, Minas Gerais, São Roque de Minas, Parque Nacional da Serra da Canastra, Cachoeira Capão Forro (confluência dos rios do Peixe e Rolador), 20°15'10.0"S, 46°24'24.0"W, el. 936 m; DZRJ; ♂].

**Distribution.** Brazil.

***carolae*** Bueno-Soria, 1990:364 [Type locality: Mexico, Veracruz, a 5 km de la Estación de Biologa Los Tuxtlas UNAM; NMNH; ♂]. —Chamorro and Holzenthal, 2010:196 [diagnosis; ♂].

**Distribution.** Mexico.

***charlesi*** (Ross), 1941:74 [Type locality: United States, Texas, San Felipe Springs, Del Rio; INHS; ♂; in *Polycentropus*]. —Flint, 1968b:21 [to *Polyplectropus*]. —Bueno-Soria and Flint, 1978:199 [distribution]. —Bueno-Soria, 1990:379 [♂; distribution]. —Maes, 1999:1189 [checklist]. —Bueno-Soria et al., 2005:75 [distribution]. —Bowles et al., 2007:23 [distribution; biology]. —Chamorro-Lacayo et al., 2007:47 [checklist]. —Ruiter and Blinn, 2009:5 [♀]. —Chamorro and Holzenthal, 2010:113 [diagnosis; ♂]. —Bueno-Soria and Barba-Álvarez, 2011:361 [checklist]. —Armitage et al., 2015b:4 [checklist]. —Armitage and Cornejo, 2015:192 [checklist].

**Distribution.** Mexico, Nicaragua, Panama, U.S.A.

***clauseni*** Chamorro-Lacayo and Holzenthal, 2004:202 [Type locality: Costa Rica, Alajuela, Cerro Campana, Río Bochinche, tributary, 6 km (air) NW Dos Ríos, 10.945°N, 85.413°W, el. 600 m; UMSP; ♂]. —Chamorro and Holzenthal, 2010:80 [diagnosis; ♂; ♀; distribution]. —Armitage et al., 2015b:4 [checklist]. —Armitage and Cornejo, 2015:192 [checklist].

**Distribution.** Costa Rica, Panama.

***colombianus*** Chamorro and Holzenthal, 2010:85 [Type locality: Colombia, Meta, Quebrada Blanca, 3 km W. Restrepo, [4°15'00"N, 73°34'00"W]. NMNH; ♂].

**Distribution.** Colombia.

***corniculatus*** Chamorro and Holzenthal, 2010:87 [Type locality: Peru, Madre de Dios, Manú [Biosphere Reserve], Pakitza Bio[logical] Sta[tion], Quebrada Paujil-Picoflor, 11°56'39"S, 71°16'59"W, el. 350 m; NMNH; ♂].

**Distribution.** Peru.

***costalis*** (Banks), 1913a:238 [Type locality: Colombia, Tolima, Cañon del Norte; MCZ; ♀; in *Cordillopsyche*]. —Flint, 1967c:7 [to *Polyplectropus*; ♀]. —Muñoz-Quesada, 2000:281 [checklist]. —Chamorro and Holzenthal, 2010:158 [diagnosis; ♀].

**Distribution.** Colombia.

***cressae*** Chamorro and Holzenthal, 2010:198 [Type locality: Venezuela, Lara, Parque Nacional Terepaima, Quebrada San Antonio, 9°51'45"N, 69°13'06"W, el. 631 m; UMSP; ♂; ♀].

**Distribution.** Venezuela.

***cuzcoensis*** Chamorro and Holzenthal, 2010:200 [Type locality: Peru, Cuzco, Paucartambo to Pilcopata rd., river at Puente Unión, 13°04'13"S, 71°34'00"W, el. 1670 m; NMNH; ♂].

**Distribution.** Peru.

***deltoides*** (Yamamoto), 1967:130 [Type locality: Panama, Cerro Punta; INHS; ♂; in *Polycentropus*]. —Flint, 1968b:21 [to *Polyplectropus*]. —Holzenthal, 1988c:60 [distribution]. —Bueno-Soria, 1990:364 [♂; distribution]. —Aguila, 1992:536 [distribution]. —Chamorro and Holzenthal, 2010:202 [diagnosis; ♂; ♀]. —Armitage et al., 2015b:4 [checklist]. —Armitage and Cornejo, 2015:192 [checklist].

**Distribution.** Costa Rica, Panama.

***denticulus*** Bueno-Soria, 1990:369 [Type locality: Costa Rica, Pacuare, Río General; NMNH; ♂]. —Maes, 1999:1189 [checklist]. —Chamorro-Lacayo et al., 2007:47 [checklist]. —Chamorro and Holzenthal, 2010:204 [diagnosis; ♂].

**Distribution.** Costa Rica, Mexico, Nicaragua.

***dubitatus*** Flint, 1983a:28 [Type locality: Argentina, Pcia. Misiones, Río Iguazú, Camp Nañdu; NMNH; ♂]. —Angrisano, 1994:134 [distribution]. —Paprocki et al., 2004:16 [checklist]. —Angrisano and Sganga, 2007:19 [♂; ♀; distribution]. —Chamorro and Holzenthal, 2010:160 [diagnosis; ♂]. —Paprocki and França, 2014:89 [checklist].

**Distribution.** Argentina, Brazil, Uruguay.

***ecuadoriensis*** Chamorro and Holzenthal, 2010:162 [Type locality: Ecuador, Sucumbios, Lago Agrio (5km N), 00°05'20"N, 76°52'09"W; NMNH; ♂].

**Distribution.** Bolivia, Ecuador.

***elongatus*** (Yamamoto), 1966:909 [Type locality: Argentina, Misiones, Iguazu; IML; ♂; in *Polycentropus*]. —Flint, 1968b:21 [to *Polyplectropus*]. —Flint, 1972b:228 [distribution]. —Chamorro and Holzenthal, 2010:164 [diagnosis; ♂; distribution; type at INHS]. —Paprocki and França, 2014:89 [checklist].

**Distribution.** Argentina, Brazil.

***exilis*** Chamorro-Lacayo and Holzenthal, 2004:204 [Type locality: Costa Rica, Cartago, Reserva Tapanti, Quebrada Segunda at administration building, 9.761°N, 83.78°W, el. 1250 m; UMSP; ♂]. —Chamorro and Holzenthal, 2010:116 [diagnosis; ♂].

**Distribution.** Costa Rica.

***flavicornis*** Ulmer, 1905a:103 [Type locality: Brazil, Santa Catharina [sic]; PAN; ♂]. —Flint, 1966a:4 [lectotype; ♂; ♀]. —Angrisano, 1994:134 [distribution]. —Paprocki et al., 2004:16 [checklist]. —Chamorro and Holzenthal, 2010:166 [diagnosis; ♂; taxonomic remarks]. —Paprocki and França, 2014:89 [checklist].

**Distribution.** Brazil, Uruguay.

***flintorum*** Chamorro and Holzenthal, 2010:89 [Type locality: Venezuela, Territorio Federal Amazonas [Estado Amazonas], Camp II, Cerro de la Neblina, 00°50'00"N, 065°59'00"W, el. 2100 m; NMNH; ♂].

**Distribution.** Venezuela.

***fuscatus*** Flint, 1983a:28 [Type locality: Argentina, Pcia. Misiones, Arroyo Piray Guazú, N San Pedro; NMNH; ♂]. —Angrisano, 1994:134 [distribution]. —Paprocki et al., 2004:16 [checklist]. —Chamorro and Holzenthal, 2010:121 [diagnosis; ♂]. —Paprocki and França, 2014:90 [checklist].

**Distribution.** Argentina, Brazil, Uruguay.

***gaesum*** Chamorro and Holzenthal, 2010:58 [Type locality: Brazil, Minas Gerais, Serra do Cipó, trib[utary] to Rio Capivara, 19°14'24"S, 43°34'56"W, el. 1000 m; MZUSP; ♂; ♀]. —Paprocki and França, 2014:90 [checklist].

**Distribution.** Brazil.

***guyanae*** Chamorro and Holzenthal, 2010:127 [Type locality: Guyana, Dubulay Ranch, Aramatani Cr[eek], 6°33'00"N, 57°48'00"W; NMNH; ♂].

**Distribution.** Guyana, Venezuela.

***hamatus*** Bueno-Soria, 1990:388 [Type locality: Mexico, Chiapas, Agua Azul, a 50 km SE de Palenque; IBUNAM; ♂]. —Chamorro and Holzenthal, 2010:168 [diagnosis; ♂]. —Bueno-Soria and Barba-Álvarez, 2011:361 [checklist].

**Distribution.** Belize, Mexico.

***hamulus*** Flint, 1972b:228 [Type locality: Argentina, Prov. Misiones, Puerto Rico; NMNH; ♂]. —Chamorro and Holzenthal, 2010:170 [diagnosis; ♂].

**Distribution.** Argentina.

***herrerai*** Bueno-Soria and Hamilton, in Bueno-Soria, 1990:386 [Type locality: Mexico, Chiapas, Cascada de Misolja, a 20 km al SE de Palenque; IBUNAM; ♂; ♀]. —Chamorro and Holzenthal, 2010:172 [diagnosis; ♂]. —Bueno-Soria and Barba-Álvarez, 2011:361 [checklist].

**Distribution.** Mexico.

***hollyae*** Chamorro and Holzenthal, 2010:136 [Type locality: Brazil, Minas Gerais, Cachoeira do Abacaxi, Vale do Tropeiro, 20°12'16"S, 43°38'10"W, el. 1120 m; MZUSP; ♂]. —Paprocki and França, 2014:90 [checklist].

**Distribution.** Brazil.

***holzenthali*** Chamorro-Lacayo, 2003:497 [Type locality: Nicaragua, Jinotega, Cerro Kilambe, 13°34'N, 85°43'W, el. 1520 m; UMSP; ♂].

**Distribution.** Nicaragua.

***hymenochilus*** Chamorro-Lacayo and Holzenthal, 2004:206 [Type locality: Costa Rica, Guanacaste, Parque Nacional Guanacaste, El Hacha, Quebrada Alcornoque, 11.009°N, 85.577°W, el. 250 m; UMSP; ♂]. —Chamorro and Holzenthal, 2010:91 [diagnosis; ♂].

**Distribution.** Costa Rica.

***hystricosus*** Chamorro and Holzenthal, 2010:60 [Type locality: Brazil, Minas Gerais, Parque Nacional do Caparaó, Rio Caparaó, Vale Verde, 20°25'02"S, 41°50'46"W, el. 1350 m; MZUSP; ♂]. —Dumas and Nessimian, 2012:24 [checklist]. —Paprocki and França, 2014:90 [checklist].

**Distribution.** Brazil.

***inarmatus*** Flint, 1971c:26 [Type locality: Brazil [Edo. Amazonas], Rio Marauiá, Endstation vor langer Cachoeira; NMNH; ♂]. ­—Flint, 1974c:33 [♂; distribution]. —Paprocki et al., 2004:16 [checklist]. —Chamorro and Holzenthal, 2010:49 [diagnosis; ♂; distribution]. —Paprocki and França, 2014:90 [checklist].

**Distribution.** Brazil, Ecuador, Peru, Suriname.

***insularis*** Chamorro and Holzenthal, 2010:206 [Type locality: Panama, Panama, Barro Colorado Island, Snyder-Molino Trail, marker 3; NMNH; ♂]. —Armitage et al., 2015b:4 [checklist]. —Armitage and Cornejo, 2015:192 [checklist].

**Distribution.** Panama.

***juliae*** Chamorro and Holzenthal, 2010:138 [Type locality: Brazil, Minas Gerais, Parque Estadual do Rio Preto, Rio Preto, 18°07'10"S, 43°20'28"W, el. 830 m; MZUSP; ♂]. —Paprocki and França, 2014:90 [checklist].

**Distribution.** Brazil.

***kanukarum*** Chamorro and Holzenthal, 2010:228 [Type locality: Guyana, [Upper Takutu-Upper Essequibo], Kanuku M[oun]t[ain]s, Moco Moco River, [3°18'02"N, 59°38'54"W]. NMNH; ♂].

**Distribution.** Guyana.

***kingsolveri*** Bueno-Soria, 1990:375 [Type locality: Mexico, Chiapas, Palenque; IBUNAM; ♂]. —Chamorro and Holzenthal, 2010:92 [diagnosis; ♂; ♀]. —Bueno-Soria and Barba-Álvarez, 2011:361 [checklist].

**Distribution.** Belize, Guatemala, Mexico.

***kylistos*** Chamorro-Lacayo and Holzenthal, 2004:208 [Type locality: Costa Rica, Guanacaste, Parque Nacional Guanacaste, Estación Pitilla, Río Orosí, 10.99l°N, 85.428°W, el. 700 m; UMSP; ♂]. —Chamorro and Holzenthal, 2010:118 [diagnosis; ♂].

**Distribution.** Costa Rica.

***laminatus*** (Yamamoto), 1966:909 [Type locality: Ecuador, El Oro, 9 miles South of Santa Rosa; INHS, not CAS; ♂; in *Polycentropus*]. —Flint, 1968b:21 [to *Polyplectropus*]. —Maes and Flint, 1988:3 [distribution]. —Flint, 1991:35 [♂; distribution]. —Bueno-Soria, 1990:397 [♂; distribution]. —Maes, 1999:1189 [checklist]. —Muñoz-Quesada, 2000:281 [checklist]. —Chamorro-Lacayo et al., 2007:47 [checklist]. —Chamorro and Holzenthal, 2010:174 [diagnosis; ♂; type at INHS; distribution]. —Armitage et al., 2015b:4 [checklist]. —Armitage and Cornejo, 2015:192 [checklist].

**Distribution.** Colombia, Costa Rica, Ecuador, Honduras, Nicaragua, Panama, Venezuela.

***maculatus*** Chamorro and Holzenthal, 2010:176 [Type locality: Venezuela, Territorio Federal Amazonas [Estado Amazonas], Cerro de la Neblina, Basecamp, 00°51'N, 66°10'W, el. 140 m; NMNH; ♂].

**Distribution.** Venezuela.

***maesi*** Chamorro-Lacayo, 2003:493 [Type locality: Nicaragua, Zelaya, Río Las Latas, l4°04'N, 88°33'W, el. 220 m; UMSP; ♂]. —Chamorro-Lacayo et al., 2007:47 [checklist]. —Chamorro and Holzenthal, 2010:94 [diagnosis; ♂; distribution]. —Armitage et al., 2015b:4 [checklist]. —Armitage and Cornejo, 2015:192 [checklist].

**Distribution.** Nicaragua, Panama.

***manuensis*** Chamorro and Holzenthal, 2010:131 [Type locality: Peru, Madre de Dios, Amazonia Lodge, Toma del Agua (stream), 12°52'13"S, 71°22'34"W, el. 415 m; NMNH; ♂].

**Distribution.** Peru.

***matatlanticus*** Chamorro and Holzenthal, 2010:63 [Type locality: [Brazil], São Paulo, Parque Estadual de Campos do Jordão, Rio Galharada, 22°41'40"S, 45°27'47"W, el. 1530 m; MZUSP; ♂; ♀]. —Calor, 2011:323 [checklist]. —Paprocki and França, 2014:90 [checklist].

**Distribution.** Brazil.

***mathisi*** Bueno-Soria, 1990:377 [Type locality: Panama, Chiriqui, Fortuna; NMNH; ♂]. —Chamorro and Holzenthal, 2010:96 [diagnosis; ♂]. —Armitage et al., 2015b:4 [checklist]. —Armitage and Cornejo, 2015:192 [checklist].

**Distribution.** Panama.

***mignonae*** Bueno-Soria, 1990:376 [Type locality: Nicaragua, Puente Quinama, E. Villa Somoza; NMNH; ♂]. —Maes, 1999:1189 [checklist]. —Chamorro-Lacayo et al., 2007:47 [checklist]. —Chamorro and Holzenthal, 2010:97 [diagnosis; ♂; ♀].

**Distribution.** Costa Rica, Nicaragua.

***minensium*** Chamorro and Holzenthal, 2010:140 [Type locality: Brazil, Minas Gerais, Parque Estadual do Itacolomi, trib[utary] to Rio Belchior, 20°25'18"S, 43°25'42"W, el. 700 m; MZUSP; ♂]. —Paprocki and França, 2014:91 [checklist].

**Distribution.** Brazil.

***misolja*** Bueno-Soria, 1990:374 [Type locality: Mexico, Chiapas, Cascada de Misolja a 20 km. al S. de Palenque; NMNH; ♂]. —Moulton and Stewart, 1997b:351 [distribution]. —Chamorro and Holzenthal, 2010:99 [diagnosis; ♂]. —Bueno-Soria and Barba-Álvarez, 2011:361 [checklist].

**Distribution.** Mexico, U.S.A.

***narifer*** Flint, 1974c:35 [Type locality: Suriname, Brownsberg, mountain creek near Golddiggers camp; RNH; ♂]. —Chamorro and Holzenthal, 2010:142 [diagnosis; ♂].

**Distribution.** Suriname.

***nayaritensis*** Bueno-Soria, 1990:362 [Type locality: Mexico, Nayarit, Compostela; IBUNAM; ♂]. —Chamorro and Holzenthal, 2010:230 [diagnosis; ♂].

**Distribution.** Mexico.

***nicaraguensis*** Chamorro-Lacayo, 2003:493 [Type locality: Nicaragua, Zelaya, Río Las Latas, l4°04'N, 88°33'W, el. 220 m; UMSP; ♂]. —Chamorro-Lacayo et al., 2007:47 [checklist]. —Chamorro and Holzenthal, 2010:101 [diagnosis; ♂].

**Distribution.** Nicaragua.

***novafriburgensis*** Chamorro and Holzenthal, 2010:66 [Type locality: Brazil, Rio de Janeiro, Nova Friburgo, mun[icipal] water supply, el. 950 m; MZUSP; ♂]. —Paprocki and França, 2014:91 [checklist].

**Distribution.** Brazil.

***oaxaquensis*** Bueno-Soria, 1990:395 [Type locality: Mexico, Oaxaca, Laguna superior de Juchitán; INHS; ♂]. —Chamorro and Holzenthal, 2010:178 [diagnosis; ♂; distribution].

**Distribution.** Mexico, Peru.

***panamensis*** Bueno-Soria, 1990:363 [Type locality: Panama, Barro Colorado Island, Zona del Canal; NMNH; ♂]. —Chamorro and Holzenthal, 2010:103 [diagnosis; ♂; distribution]. —Armitage et al., 2015b:5 [checklist]. —Armitage and Cornejo, 2015:192 [checklist].

**Distribution.** Costa Rica, Panama.

***paradelphae*** Chamorro-Lacayo and Holzenthal, 2004:210 [Type locality: Costa Rica, Limon, Reserva Biológica Hitoy-Cerere, Río Cerere, 9.671°N, 83.028°W, el. 90 m; UMSP; ♂]. —Chamorro and Holzenthal, 2010:207 [diagnosis; ♂].

**Distribution.** Costa Rica.

***paysandu*** Angrisano, 1994:134 [Type locality: Uruguay, Depto. Paysandú, Sta. Rita; FHCU; ♂; wings]. —Chamorro and Holzenthal, 2010:68 [diagnosis; ♂].

**Distribution.** Uruguay.

***perpendicularis*** Chamorro-Lacayo and Holzenthal, 2004:210 [Type locality: Costa Rica, Puntarenas, Tributary to Rio Bellavista in Las Alturas (road to quary), 8.952°N, 82.848°W, el. 1480 m; UMSP; ♂]. —Chamorro and Holzenthal, 2010:104 [diagnosis; ♂].

**Distribution.** Costa Rica.

***peruvianus*** Chamorro and Holzenthal, 2010:129 [Type locality: Peru, Loreto, Callicebus Research Station, Mishana, Río Nanay, 25 km SW Iquitos, [3°44'53"S, 73°14'50"W], el. 120 m; NMNH; ♂].

**Distribution.** Peru.

***petrae*** Chamorro and Holzenthal, 2010:106 [Type locality: Brazil, Minas Gerais: Estação Ecológica do Tripuí, Córrego Tripuí, 20°23'22"S, 43°32'32"W, el. 1070 m; MZUSP; ♂]. —Paprocki and França, 2014:91 [checklist].

**Distribution.** Brazil.

***pratherae*** Chamorro and Holzenthal, 2010:69 [Type locality: Brazil, Minas Gerais, Parque Nacional do Caparaó, Rio Caparaó, Vale Verde, 20°25'02"S, 41°50'46"W, el. 1350 m; MZUSP; ♂]. —Paprocki and França, 2014:91 [checklist].

**Distribution.** Brazil.

***profaupar*** Holzenthal and Almeida, 2003:26 [Type locality: Brazil, Santa Catarina, Morro da lgreja, Urubici, Cachoeira Véu da Noiva, 28°04.595'S, 49°3l.090'W, el. 1300 m; MZUSP; ♂; ♀]. —Paprocki et al., 2004:16 [checklist]. —Dumas et al., 2009:360 [distribution]. —Dumas et al., 2010:9 [distribution]. —Chamorro and Holzenthal, 2010:71 [diagnosis; ♂; ♀; distribution]. —Paprocki and França, 2014:91 [checklist].

**Distribution.** Brazil.

***pugiunculatus*** Botosaneanu, in Botosaneanu and Alkin-Koo, 1993:31 [Type locality: Trinidad, Northern Range, stream just below Blue Basin Waterfall; ZMUA; ♂; ♀]. —Botosaneanu and Sakal, 1992:203 [*nomen nudum* (name included in checklist); distribution; ecology]. —Flint, 1996a:74 [distribution]. —Botosaneanu, 2002:96 [checklist]. —Chamorro and Holzenthal, 2010:209 [diagnosis; ♂].

**Distribution.** Tobago, Trinidad.

***puyoensis*** Chamorro and Holzenthal, 2010:211 [Type locality: Ecuador, Pastaza, Puyo (16 kms. W); NMNH; ♂; ♀].

**Distribution.** Ecuador.

***recurvatus*** (Yamamoto), 1966:912 [Type locality: Colombia, Tolima, 18 miles west of Honda; CAS; ♂; in *Polycentropus*]. —Flint, 1968b:21 [to *Polyplectropus*]. —Flint, 1981a:16 [♂; distribution]. —Muñoz-Quesada, 2000:281 [checklist]. —Chamorro and Holzenthal, 2010:213 [diagnosis; ♂; ♀; distribution].

**Distribution.** Colombia, Ecuador, Venezuela.

***robacki*** (Yamamoto), 1966:911 [Type locality: Peru, Huallaga River, Tingo Maria; INHS; ♂; in *Polycentropus*]. —Flint, 1968b:21 [to *Polyplectropus*]. —Chamorro and Holzenthal, 2010:180 [diagnosis; ♂].

**Distribution.** Peru.

***robertsonae*** Chamorro and Holzenthal, 2010:132 [Type locality: Bolivia, Santa Cruz, P[arque] N[acional] & A[rea] N[atural] [de] M[anejo] I[ntegrado] Amboró, Guarda Parque Mataracú, Confluence of Quebrada Verde Uno y Dos, 17°33'11"S, 63°52'09"W, el. 371 m; UASC; ♂].

**Distribution.** Bolivia.

***rodmani*** Chamorro and Holzenthal, 2010:73 [Type locality: Brazil, São Paulo, Estação Biológica Boracéia, Rio Coruja, 23°40'06"S, 45°53'57"W, el. 850 m; MZUSP; ♂; ♀]. —Calor, 2011:323 [checklist]. —Paprocki and França, 2014:91 [checklist].

**Distribution.** Brazil.

***rondoniensis*** Chamorro and Holzenthal, 2010:122 [Type locality: Brazil, Rondônia, creek 8 km S of Cacaulandia; MZUSP; ♂; ♀]. —Paprocki and França, 2014:91 [checklist].

**Distribution.** Brazil.

***santiago*** (Ross), 1947:136 [Type locality: Mexico, Nuevo León, Villa Santiago; INHS; ♂; in *Polycentropus*]. —Flint, 1968b:21 [to *Polyplectropus*]. —Bueno-Soria and Flint, 1978:199 [distribution]. —Maes and Flint, 1988:3 [distribution]. —Holzenthal, 1988c:60 [distribution]. —Bueno-Soria, 1990:396 [♂; distribution]. —Aguila, 1992:536 [distribution]. —Maes, 1999:1189 [checklist]. —Bowles et al., 2007:23 [distribution; biology]. —Chamorro-Lacayo et al., 2007:47 [checklist]. —Chamorro and Holzenthal, 2010:181 [diagnosis; ♂]. —Armitage et al., 2015b:5 [checklist]. —Armitage and Cornejo, 2015:192 [checklist].

—*proditus* Edwards, 1973:502 [Type locality: United States, Texas, Frio River, Barksdale, Edwards Co. (erroneous, see Moulton, 1996); type destroyed]. —Moulton, 1996:273 [Neotype: United States, Texas, Bandera Co., Winans Creek @ TX Hwy 16, 4 mi N Bandera; INHS; ♂; to synonymy].

**Distribution.** Costa Rica, Honduras, Mexico, Nicaragua, Panama, U.S.A.

***spiculifer*** Flint, 1971c:27 [Type locality: Brazil [Edo. Amazonas], Rio Marauiá, Endstation vor langer Cachoeira; NMNH; ♂]. —Paprocki et al., 2004:16 [checklist]. —Chamorro and Holzenthal, 2010:183 [diagnosis; ♂]. —Paprocki and França, 2014:91 [checklist].

**Distribution.** Brazil.

***squalus*** Bueno-Soria, 1990:385 [Type locality: Mexico, Chiapas, Chajul, 90°30’ y 91°E, y 16°00’y 16°30'N; IBUNAM; ♂]. —Chamorro and Holzenthal, 2010:185 [diagnosis; ♂]. —Bueno-Soria and Barba-Álvarez, 2011:361 [checklist].

**Distribution.** Mexico.

***thilus*** (Denning), 1962b:407 [Type locality: Mexico, Jalisco, 17 miles south of Mazamitla; CAS; ♂; in *Polycentropus*]. —Flint, 1968b:21 [to *Polyplectropus*]. —Bueno-Soria and Flint, 1978:200 [distribution]. —Bueno-Soria, 1990:372 [♂; distribution]. —Chamorro and Holzenthal, 2010:216 [diagnosis; ♂; distribution]. —Bueno-Soria and Barba-Álvarez, 2011:361 [checklist].

**Distribution.** Mexico, Nicaragua.

***tragularius*** Chamorro and Holzenthal, 2010:134 [Type locality: Brazil, Minas Gerais, Parque Nacional Peruaçu, Rio Peruaçu, 15°06'40"S, 44°14'29"W, el. 590 m; MZUSP; ♂]. —Calor, 2011:323 [checklist]. —Barcelos-Silva et al., 2012:1279 [distribution]. —Paprocki and França, 2014:92 [checklist].

**Distribution.** Brazil.

***trilobatus*** Flint, 1981a:16 [Type locality: Venezuela, Aragua, Maracay, Río Limón, Estación Piscicultura; NMNH; ♂]. —Chamorro and Holzenthal, 2010:108 [diagnosis; ♂].

**Distribution.** Venezuela.

***tripunctatum*** Chamorro and Holzenthal, 2010:134 [Type locality: Peru, Cuzco, Paucartambo to Pilcopata rd., Quebrada Quitacalzón at Puente Quitacalzón, 13°01'34"S, 71°29'58"W, el. 1050 m; NMNH; ♂].

**Distribution.** Peru.

***ulmeriana*** Flint, 1983a:27 [Type locality: Argentina, Pcia. Misiones, Arroyo Piray Mini, Rt. 17 W Dos Hermanas; NMNH; ♂]. —Paprocki et al., 2004:16 [checklist]. —Chamorro and Holzenthal, 2010:143 [diagnosis; ♂]. —Paprocki and França, 2014:92 [checklist].

**Distribution.** Argentina, Brazil, Paraguay.

***venezolanus*** Chamorro and Holzenthal, 2010:218 [Type locality: Venezuela, Sucre, Rio Cocollar, 1.5 km SE Las Piedras de Cocollar, 10°09'37"N, 63°47'36"W, el. 810 m; UMSP; ♂].

**Distribution.** Venezuela.

***woldai*** Chamorro and Holzenthal, 2010:110 [Type locality: Panama, Chiriquí, Fortuna Dam Site nr. Hornitos, 8°55'00"N, 82°16'00"W, el. 1050 m; NMNH; ♂]. —Armitage et al., 2015b:5 [checklist]. —Armitage and Cornejo, 2015:192 [checklist].

**Distribution.** Panama.

***yolandae*** Chamorro-Lacayo and Holzenthal, 2004:213 [Type locality: Costa Rica, Guanacaste, Parque Nacional Guanacaste, Estacion Maritza, Rio Tempisquito, 10.958°N, 85.497°W, el. 550 m; UMSP; ♂]. —Chamorro-Lacayo et al., 2007:47 [checklist]. —Chamorro and Holzenthal, 2010:220 [diagnosis; ♂; ♀; distribution]. —Armitage et al., 2015b:5 [checklist].

**Distribution.** Costa Rica, Nicaragua, Panama.

***zamoranoensis*** Chamorro and Holzenthal, 2010:187 [Type locality: Honduras, El Zamorano; NMNH; ♂; ♀].

**Distribution.** Honduras.

***zaragozai*** Bueno-Soria, 1990:387 [Type locality: Mexico, Hidalgo, carretera 105, a 60 kms al NO de Pachuca en el Río Tulancingo, Puente Venados; IBUNAM; ♂]. —Chamorro and Holzenthal, 2010:188 [diagnosis; ♂].

**Distribution.** Guatemala, Honduras, Mexico.

***zuliae*** Chamorro and Holzenthal, 2010:222 [Type locality: Venezuela, Zulia, Los Angeles del Tucuco; NMNH; ♂; ♀].

**Distribution.** Venezuela.

### Family Pseudoneureclipsidae

The status of this family and its two included genera, *Antillopsyche* and *Pseudoneureclipsis*, has been equivocal. It was long considered a subfamily of Polycentropodidae, but Li et al. (2001) considered the nominotypical genus to be a member of Dipseudopsidae. After a rigorous phylogenetic analysis, Chamorro and Holzenthal (2011) concluded the genera were neither dipseudopsids nor polycentropodids and elevated the subfamily to family status, Pseudoneureclipsidae, probably sister to a clade containing Polycentropodidae and Ecnomidae (Johanson et al. 2012).

#### Genus *Antillopsyche* Banks [4 + †4]

*Antillopsyche* Banks, 1941:400 [Type species: *Antillopsyche wrighti* Banks, 1941, original designation]. —Flint, 1964a:29 [systematic position]. —Li et al., 2001:111 [to Dipseudopsidae, indirectly]. —Wichard, 2007a:26 [diagnosis; distribution; key, in Dipseudopsidae]. —Chamorro and Holzenthal, 2011:226 [to Pseudoneureclipsidae].

The genus is known from the Greater Antillean islands of Cuba, Hispaniola, and Puerto Rico, and is well represented in amber from the Dominican Republic and Mexico (Wichard 2007a).

The larvae and pupae of *Antillopsyche tubicola* were described by Flint (1964a). The larvae construct silken tubes attached to rocks below the soil-water interface. They were found in a small, clear stream, near the end of a pool.

† ***auricula*** Wichard, 2007a:27 [Type locality: Dominican Republic; SMNS (ex collection Wichard); ♂; in amber].

**Distribution.** Dominican Republic.

***aycara*** Botosaneanu, 1980:104 [Type locality: Cuba, Arroyo Blanco, Sabanas de San Felipe, Jatibonico, Prov. Las Villas; ZMUA; ♂; ♀]. —Botosaneanu, 1979:45 [*nomen nudum* (name included in checklist); distribution]. —Flint, 1996c:16 [checklist]. —Botosaneanu, 2002:95 [checklist]. —Naranjo López and González Lazo, 2005:150 [checklist].

**Distribution.** Cuba.

***demma*** Botosaneanu, 1996:13 [Type locality: Dominican Republic, Cordillera Central, Salto Agua Blanca, ca. 3 km from Convento; ZMUA; ♂]. —Flint and Pérez-Gelabert, 1999:43 [checklist]. —Botosaneanu, 2002:95 [checklist]. —Flint and Sykora, 2004:52 [distribution]. —Pérez-Gelabert, 2008:302 [checklist].

**Distribution.** Dominican Republic.

† ***digitus*** Wichard, 2007a:30 [Type locality: Dominican Republic; SMNS (ex collection Wichard); ♂; in amber].

**Distribution.** Dominican Republic.

† ***mexicana*** Wichard, Solórzano-Kraemer and Luer, 2006:43 [Type locality: Mexico; IHNEC; ♂; in amber].

**Distribution.** Mexico.

† ***oliveri*** Wichard, 1985:118 [Type locality: Dominican Republic; collection Wichard; ♂; in amber]. —Flint and Pérez-Gelabert, 1999:43 [checklist]. —Botosaneanu, 2002:95 [checklist]. —Wichard 2007a:27 [♂; diagnosis]. —Pérez-Gelabert, 2008:302 [checklist].

**Distribution.** Dominican Republic.

***tubicola*** Flint, 1964a:30 [Type locality: Puerto Rico, Maricao; NMNH; ♂; ♀; larva; pupa; biology]. —Flint, 1968b:80 [checklist]. —Botosaneanu, 2002:95 [checklist].

**Distribution.** Puerto Rico.

***wrighti*** Banks, 1941:400 [Type locality: Cuba, Santa Clara Prov., Soledad; MCZ; ♂]. —Flint, 1967c:5 [♂; lectotype]. —Flint, 1968b:80 [checklist]. —Botosaneanu, 1979:45 [distribution]. —Flint, 1996c:16 [checklist]. —Botosaneanu, 2002:95 [checklist]. —Naranjo López and González Lazo, 2005:150 [checklist].

**Distribution.** Cuba.

### Family Psychomyiidae

The family is widely distributed in most regions of the world, but notably lacking in the Neotropical region; two species in the family occur in Nearctic Mexico, while the other New World species are exclusively North American. Nine genera occur in total, with most species diversity occurring in the Oriental region.

The immature stages of most genera have been described (Lepneva 1970, Wiggins 1996). They all construct a long, silken tube attached to the substrate. They inhabit a great variety of sites: lakes, larger rivers and streams to small, spring fed brooklets. Gut contents are almost exclusively algae, organic matter, and detritus, which the larvae scrape off the surroundings (Wiggins 1996). One species cultivates algae in the silken mesh of the retreat to use as a food source (Hasselrot 1993, Ings et al. 2012).

#### Genus *Tinodes* Curtis [2]

*Tinodes* Curtis, 1834:216 [Type species: *Tinodes luridus* Curtis, 1834, by monotypy, a synonym of *Tinodes waeneri* (L.)].

This is a very wide-ranging genus of more than 200 species from the Palearctic, Oriental, Afrotropical and western Nearctic regions. The genus is only known from western North America in the New World, where two species also occur in Baja California, Mexico.

The immature stages construct long silken tubes mixed with mineral fragments. These tubes extend for several centimeters over the rock surface. The immature stages are found in flowing water, often on hygropetric surfaces. Wiggins (1996) reported gut contents to be largely detritus and algae.

***powelli*** Denning, 1964:131 [Type locality: Mexico, Baja California, Sierra San Pedro Martir, La Grulla; CAS; ♂]. —Bueno-Soria and Flint, 1978:198 [distribution].

**Distribution.** Mexico.

***provo*** Ross and Merkley, 1950:66 [Type locality: USA, Utah, Provo River, Highway 89-91, at bridge; INHS; ♂]. — Denning, 1983:208 [distribution; Baja California]. —Blinn and Ruiter, 2009b:187 [phenology, distribution].

**Distribution.** Mexico, U.S.A.

### Family Rhyacophilidae

This is a family primarily of the Northern Hemisphere, occurring as far south as the Atlas Mountains in northern Africa, Java in Indonesia, and northern Mexico. In addition to the type genus, it contains *Himalopsyche* from Asia and western North America, *Philocrena* from the Caucasas, and *Fansipangana* from Vietnam. *Rhyacophila* contains ca. 700 described species, *Himalopsyche* has ca. 50 species, and *Philocrena* and *Fansipangana* each have only a single species.

The larvae of all genera, except *Fansipangana*, and many species from various regions of the world, have been described (Flint 1962b, Lepneva 1970, Schmid and Botosaneanu 1966, Wiggins 1996). They are free-living predators in general, although *Rhyacophila verrula* Milne and a few others are phytophagous, feeding primarily on the freshwater green alga, *Prasiola* (Smith 1968).

#### Genus *Rhyacophila* Pictet [1]

*Rhyacophila* Pictet, 1834:181 [Type species: *Rhyacophila vulgaris* Pictet, 1834, subsequent designation of Ross 1944]. —Ross, 1956a:73 [revision]. —Schmid, 1970:29 [revision].

This is the dominant genus of the family, and, as is the whole family, primarily distributed in the Northern Hemisphere. Within these northern regions it is a very common and diverse group. In his classic monograph Schmid (1970) recorded the 465 species known at the time. In the New World, over 130 species are presently recognized, but new ones are being described at frequent intervals. Only a single species has been taken in the region covered by this catalog; however, several more may be found in northwestern Mexico.

The larvae of many species are known, and they are quite diverse in appearance. They are, however, all free-living, and with the exception of a few, predators on other small aquatic organisms (Wiggins 1996). The one Mexican species is unknown in its immature stages.

***rayneri*** Ross, 1951a:66 [Type locality: Mexico, Lower California, Valadares; INHS; ♂]. —Denning, 1956b:244 [distribution]. —Denning, 1964: 133 [checklist]. —Bueno-Soria and Flint, 1978:192 [distribution].

**Distribution.** Mexico, USA.

### Family Sericostomatidae

Many of the Southern Hemisphere genera once placed in the Sericostomatidae have been transferred to other families (Neboiss 1977, Scott 1985, Flint 1979, 1981c). As it was formerly understood, the family contained about 20 genera and 100 species. The combined distribution of these genera is cosmopolitan, except for Australia, New Zealand, and their biogeographically associated islands, but the individual genera are for the most part restricted within their regions. The Latin American fauna is composed of *Gumaga griseola* from Mexico, *Grumicha grumicha* from southeastern Brazil and neighboring Argentina, and *Myotrichia*, *Notidobiella*, *Parasericostoma*, and the obscure genus *Chiloecia*, together containing 18 species, mostly from Chile, although *Notidobiella* has been described recently from tropical regions of Ecuador and Brazil. Very recently, Johanson et al. (2016) established a new family, Parasericostomatidae for *Chiloecia* and *Parasericostoma*, including *Myotrichia* as a junior synonym of the latter.

The larvae build tubular cases of sand grains or silk alone. They inhabit streams and lakes, where the larvae often burrow in sandy substrates. Larvae are detritivores.

#### Genus *Chiloecia* Navás [1]

*Chiloecia* Navás, 1930b:365 [Type species: *Chiloecia lacustris* Navás 1930b, by monotypy; in Limnephilidae]. —Flint et al., 1999a:80 [to Sericostomatidae].

This obscure genus is known only from the original description. Its type is presumably lost, but the illustrations in the original description suggest that the genus is close to *Parasericostoma*.

***lacustris*** Navás, 1930b: 366 [Type locality: Chile, Panguipulli; collection Navás, now lost?; sex not stated]. —Flint, 1974e:88 [unidentified]. —Flint et al., 1999a:80 [status].

**Distribution.** Chile.

#### Genus *Grumicha* Müller [1]

*Grumicha* Müller, 1879b:134 [Type species: *Dicentropus flavipes* Ulmer, 1905a, first included species, a subsequent synonym of *Phryganea grumicha* Vallot, 1855]. —Flint et al., 1999:80 [nomenclatural history of *Grumicha* and its result].

*Dicentropus* Ulmer, 1905a:16 [Type species: *Dicentropus flavipes* Ulmer, 1905a, by monotypy]. — Ulmer, 1905b:97 [to synonymy].

The single known species, *Grumicha grumicha*, occurs in southeastern Brazil and adjacent Argentina. The larval stage and case have been described a few times (Müller 1880a, b, 1921), but not recently.

Larvae build slender, smooth, shiny cases entirely of darkened silk (Saint-Hilaire 1830). They live on rocks in medium sized streams and probably feed by scraping the periphyton.

***grumicha*** (Vallot), 1855:XII [Type locality: Brazil; type depository unknown; case; in *Phryganea*]. —Hagen, 1864a:226 [to *Leptocerus*?]. —Holzenthal, 1988a:191 [cases occupied by *Triplectides*]. —Flint et al., 1999a:80 [to *Grumicha*]. —Blahnik et al., 2004:5 [distribution]. —Crisci-Bispo et al., 2004:133 [cases occupied by *Triplectides*]. —Paprocki et al., 2004:16 [checklist]. —Calor, 2011:323 [checklist]. —Sganga et al., 2013:24 [cases occupied by *Triplectides misionensis*]. —Manzo et al., 2014:167 [distribution]. —Paprocki and França, 2014:92 [checklist].

—*flavipes* (Ulmer), 1905a:16 [Type locality: Brazil, Santa Catarina; PAN; ♂; in *Dicentropus*]. —Ulmer, 1905b:97 [to *Grumicha*]. —Thienemann, 1909:37 [larva]. —Ulmer, 1913:404 [distribution]. —Müller 1880a:106, 128 [case]. —Müller, 1921:432 [larva; pupa]. —Flint, 1966a:12 [lectotype; ♂]. —Flint et al., 1999a:80 [to synonymy].

**Distribution.** Argentina, Brazil.

#### Genus *Gumaga* Tsuda [1]

*Gumaga* Tsuda, 1938:100 [Type species: *Gumaga okinawaensis* Tsuda, 1938, by monotypy].

*Gumaga* occurs in Japan and the western United States. A total of six species are known. Only *Gumaga griseola* occurs in Mexico, in Baja California.

Larvae were described by Wiggins (1996). They build long, slender cases of minute sand grains. Larvae live in cold springs or occassionally warm streams and feed on fine organic particles.

***griseola*** (McLachlan), 1871:112 [Type locality: United States, California; BMNH; ♂; in *Notidobia*]. —Ross, 1951a:74 [as *Sericostoma* sp., distribution]. —Kimmins and Denning, 1951:138 [♂; redescription; in *Sericostoma*]. —Kimmins, 1957:123 [lectotype]. —Denning, 1964:134 [checklist]. —Ross and Wallace, 1974:46 [to *Gumaga*]. —Bueno-Soria and Flint, 1978:215 [distribution]. —Wiggins, 1996:380 [larva]. — Miller et al., 2002:1665 [biology]. —Blinn and Ruiter, 2006:329 [biology]. —Blinn and Ruiter, 2009a:303 [biology]. —Blinn and Ruiter, 2009b:187 [phenology, distribution]. —Ruiter and Blinn, 2009:5 [♀]. —Djernaes, 2011:24 [♂; ♀].

—*assimilis* Banks, 1907a:124 [Type locality: [United States], San Diego, Cal[ifornia], Field]; ♂; as *Notidobia*]. —Milne, 1936:125 [to synonymy].

**Distribution.** Mexico, U.S.A.

#### Genus *Myotrichia* Schmid [1]

*Myotrichia* Schmid, 1955a:153 [Type species: *Myotrichia murina* Schmid, 1955a, original designation].

A single species is known in the genus. The immature stages and biology were described and discussed by Valverde and Albariño (1999) and Brand and Miserendino (2011a).

***murina*** Schmid, 1955a:153 [Type locality: Chile, Santiago, Quebrada de Macul; NMNH; ♂]. —Flint, 1974e:91 [checklist]. —Valverde and Albariño, 1999:12 [larva; pupa; biology; distribution]. —Brand and Miserendino, 2011a:35 [biology; distribution]. —Brand and Miserendino, 2011b:143 [biology]. —Diáz Villanueva et al., 2012:2506 [biology]. —Brand et al., 2012:90 [biology]. —Brand and Miserendino, 2014:6 [community ecology].

**Distribution.** Argentina, Chile.

#### Genus *Notidobiella* Schmid [6]

*Notidobiella* Schmid, 1955a:152 [Type species: *Notidobiella parallelipipeda* Schmid, 1955a, original designation]. —Holzenthal and Blahnik, 2010:25 [revision; biogeographical discussion].

Six species are known in the genus, three endemic to Chile and the others from Brazil (north and southeast regions) and Ecuador. The Neotropical species of Sericostomatidae, including those in the genus *Notidobiella*, appear to be members of a southern Gondwana fauna (de Moor and Ivanov 2008).

The immature stages of *Notidobiella chacayana* were described by Flint (1967a). The very slightly tapered and curved cases are constructed of sand grains embedded in silk. Larvae are found in small streams and probably are detritivores.

***amazoniana*** Holzenthal and Blahnik, 2010:28 [Type locality: Brazil, Amazonas, AM 010, km 246; INPA; ♂]. —Paprocki and França, 2014:92 [checklist].

**Distribution.** Brazil.

***brasiliana*** Holzenthal and Blahnik, 2010:30 [Type locality: Brazil, São Paulo, Parque Estadual de Campos do Jordão, 1st. order trib. to Rio Galharada, 22°41.662'S, 45°27.783'W, el. 1530 m; MZUSP; ♂; ♀]. —Paprocki and França, 2014:92 [checklist].

**Distribution.** Brazil.

***chacayana*** Schmid, 1957:392 [Type locality: Chile, Maule, Chacay; NMNH; ♂]. —Flint, 1967a:63 [larva; pupa]. —Flint, 1974e:91 [checklist]. —Cohen, 2004:79 [distribution]. —Holzenthal and Blahnik, 2010:33 [diagnosis; ♂; ♀].

**Distribution.** Chile.

***ecuadorensis*** Holzenthal and Blahnik, 2010:30 [Type locality: Ecuador, Pastaza, Puyo; NMNH; ♂].

**Distribution.** Ecuador.

***inermis*** Flint, 1983a:90 [Type locality: Chile, Pcia. Cautín, near Pucón; NMNH; ♂]. —Holzenthal and Blahnik, 2010:37 [diagnosis; ♂; ♀].

**Distribution.** Chile.

***parallelipipeda*** Schmid, 1955a:152 [Type locality: Chile, Ñuble, Recinto; NMNH; ♂]. —Flint, 1974e:91 [checklist]. —Holzenthal and Blahnik, 2010:26 [diagnosis; ♂; ♀].

**Distribution.** Chile.

#### Genus *Parasericostoma* Schmid [10]

*Chrysostoma* Schmid, 1955a: 154 [Type species: *Chrysostoma peniai* Schmid, 1955a, original designation; preoccupied by *Chrysostoma* Swainson, 1840, in Mollusca]

*Parasericostoma* Schmid, 1957:393 [replacement name for *Chrysostoma* Schmid, 1955a].

Ten species are known in the genus, making it the largest member of the family in the Neotropics. None of the species has been reported to occur outside of Chile and adjacent Argentina.

The larva and pupa of *Parasericostoma laterale* were described by Flint (1967a), those of *Parasericostoma ovale* by Valverde and Miserendino (1997), and of *Parasericostoma cristatum* by Valverde and Albariño (1999). Larvae fashion cases entirely of silk, which are tapered and slightly curved. Small streams are the known habitat. The larvae of *Parasericostoma cristatum* are shredders, feeding on leaves and other organic matter in the environment (Albariño and Valverde 1998).

***abruptum*** Schmid, 1964:335 [Type locality: Chile, Contulmo, Palo Botado; NMNH; ♂]. —Flint, 1974e:91 [checklist]. —Flint, 1990:120 [distribution].

**Distribution.** Chile.

***acutum*** Flint, 1983a:89 [Type locality: Chile, Pcia. Maule, Alto Tregualemu, ~20 km SE Chovellén; NMNH; ♂].

**Distribution.** Chile.

***corniculatum*** Flint, 1983a:88 [Type locality: Chile, Pcia. Malleco, Cabrería, Cordillera Nahuelbuta; NMNH; ♂].

**Distribution.** Chile.

***cristatum*** Flint, 1983a:88 [Type locality: Chile, Pcia. Ñuble, Las Trancas, Cordillera Chillán; NMNH; ♂]. —Albariño and Valverde, 1998:131 [biology; distribution]. —Valverde and Albariño, 1999:14 [larva; pupa; biology; distribution]. —Brand, 2009:224 [distribution]. —Brand and Miserendino, 2011a:35 [biology]. —Brand and Miserendino, 2014:6 [community ecology].

**Distribution.** Argentina, Chile.

***dinocephalum*** Schmid, 1957:394 [Type locality: Chile, Linares, Esterro de Lleiva; NMNH; ♂]. —Flint, 1974e:91 [checklist].

**Distribution.** Chile.

***drepanigerum*** Flint, 1983a:86 [Type locality: Chile, Pcia. Malleco, Cabrería, Cordillera Nahuelbuta; NMNH; ♂].

**Distribution.** Chile.

***laterale*** Schmid, 1964:335 [Type locality: Chile, Malleco, Thermas de Tolhuaca; NMNH; ♂]. —Flint, 1967a:61 [larva; pupa]. —Flint, 1974e:91 [checklist].

**Distribution.** Chile.

***ovale*** (Schmid), 1955a:155 [Type locality: Chile, Santiago, El Manzano; NMNH; ♂; in *Chrysostoma*]. —Schmid, 1957:393 [to *Parasericostoma*]. —Flint, 1974e:91 [checklist]. —Flint, 1990:120 [distribution]. —Valverde and Miserendino, 1997:33 [larva; pupa; biology]. —Miserendino and Brand, 2007:312 [biology]. —Brand and Miserendino, 2011a:35 [biology]. —Brand et al., 2012:90 [biology]. —Brand and Miserendino, 2014:6 [community ecology].

**Distribution.** Argentina, Chile.

***peniai*** (Schmid), 1955a:154 [Type locality: Chile, Ñuble, Los Pellines; NMNH; ♂; in *Chrysostoma*]. —Schmid, 1957:393 [to *Parasericostoma*; variation; distribution]. —Flint, 1974e:92 [checklist].

**Distribution.** Chile.

***rufum*** Schmid, 1964:335 [Type locality: Chile, Ñuble, Atacalco; NMNH; ♂]. —Flint, 1974e:92 [checklist].

**Distribution.** Chile.

### Family Stenopsychidae

The stenopsychids are a family of only three known genera: *Stenopsyche*, of ca. 90 species, widely distributed throughout Asia and also with single known examples from Africa and Baltic amber, *Stenopsychodes* of nine Australian species, and *Pseudostenopsyche*, with two or three Chilean species (Neboiss 1986, Schmid 1969, Weaver 1987).

Larvae of several species of *Stenopsyche* have been described and their biology is well known (Lepneva 1970, Swegman and Coffman 1980, Tanida 2002, provided a detailed account). Larvae of *Pseudostenopsyche* and *Stenopsychodes* have not been described. *Stenopsyche* larvae have elongate heads and sclerotized labra with membranous margins bearing dense fringes of short setae. They use this labral brush to groom the tubular nets, as do philopotamids, their close relatives. Food includes other insects as well as organic material (Tanida 2002). The pupae are found under a domelike shelter of small stones, in a thin, loose cocoon of sparse grayish silk (Lepneva 1970). They are found on large cobble in small streams and brooks, but also larger rivers.

#### Genus *Pseudostenopsyche* Döhler [3]

*Pseudostenopsyche* Döhler, 1915:399 [Type species: *Pseudostenopsyche sugens* Döhler, 1915, by monotypy].

*Rhyncorheithrus* Schmid, 1964:330 [Type species: *Rhyncorheithrus gracilis* Schmid, 1964, original designation]. —Flint, 1974e:84 [to synonymy].

This is the single, known representative of the family in the Neotropical Region. It contains two species at a minimum, the probability being that *Pseudostenopsyche sugens* is the female of one of the other species, known only from males. The immature stages and biology of the genus are unknown.

***davisorum*** Flint, 1983a:21 [Type locality: Chile, Pcia. Maule, Alto Tregualemu, ca. 20km SE Chovellán; NMNH; ♂].

**Distribution.** Chile.

***gracilis*** (Schmid), 1964:332 [Type locality: Chile, (Arauco), Caramavida; NMNH; ♂; in *Rhyncorheithrus*]. —Flint, 1974e:91 [to *Pseudostenopsyche*].

**Distribution.** Chile.

***sugens*** Döhler, 1915:400 [Type locality: Chile; ZMHU; ♀]. —Flint, 1974e:91 [checklist].

**Distribution.** Chile.

### Family Tasimiidae

This is a small family of four genera and only nine species. *Charadropsyche* and *Trichovespula*, each with a single species, are known from Chile, whereas *Tasiagma* and *Tasimia*, with one and five species, respectively, are reported from eastern Australia and Tasmania.

The immature stages of *Charadropsyche* and *Trichovespula* are described (Flint 1999, 1967a) as are the immature stages of *Tasimia* (Riek 1968). They all build cases of sand grains, often with ballast stones laterally, and live in clear, fast flowing streams.

#### Genus *Charadropsyche* Flint [1]

*Charadropsyche* Flint, 1969b:510 [Type species: *Charadropsyche penicillata* Flint, 1969b, original designation]. —Flint, 1999:99 [redescription; placement, immature stages].

A single species occurs in the Chilean Subregion of the South American continent. The larva, pupa, and case have been described (Flint 1999). They construct flattened, sand grain cases, tapering strongly rearward. They were found in shallow, cascading brooklets in strongly shaded areas, often in a thin film of water on the surface of boulders.

***penicillata*** Flint, 1969b:510 [Type locality: Chile, Prov. Curico, Estero la Jaula, Los Quenes; NMNH; ♂]. —Flint, 1974e:89 [checklist]. —Flint, 1999:99 [♂; ♀; larva; pupa; case; biology].

**Distribution.** Chile.

#### Genus *Trichovespula* Schmid [1]

*Trichovespula* Schmid, 1955a:149 [Type species: *Trichovespula macrocera* Schmid, 1955a, original designation; in Lepidostomatidae]. —Flint, 1969b:509 [to Tasimiidae].

This is the second monotypic tasimiid genus from the Chilean subregion. It differs from the other known genera in the family in that the male palps are reduced to three segments.

The larva, pupa, and case were described by Flint (1967a). The larval cases are tubular and straight, incorporating large, lateral, ballast stones. They are found, often quite abundantly, in small to rather large, clear, fast flowing streams.

***macrocera*** Schmid, 1955a:150 [Type locality: Chile, Prov. Ñuble, Recinto; NMNH; ♂]. —Flint, 1967a:64 [larva; pupa; case]. —Flint 1974e:90 [checklist]. —Brand, 2009:225 [distribution].

**Distribution.** Argentina, Chile.

### Family Xiphocentronidae

This family occurs primarily in the tropical regions of the world, where the highest diversity of species occurs in the Oriental and Neotropical regions (Schmid 1982). A few are known from central Africa, the Middle East, Japan, and the extreme southwest of the United States.

Larvae and pupae of *Xiphocentron messapus* were described by Edwards (1961, as *Xiphocentron mexico*) and Wiggins (1996), *Xiphocentron cubanum haitiensis* by Flint (1964a), *Xiphocentron moncho* by Muñoz and Holzenthal (1997), and *Xiphocentron sclerothrix* by Pes et al. (2013). The larvae construct long, silken tubes, often covered with debris, fixed to the substrate. The tubes may extend several centimeters or more above the water surface if the substrate is constantly moist. In *Xiphocentron moncho*, from Costa Rican, the tubes were much shorter and carried about by the larvae like a case.

#### Genus *Cnodocentron* Schmid [6]

*Cnodocentron* Schmid, 1982:36 [Type species: Cnodocentron (Cnodocentron) girika Schmid 1982, original designation].

*Caenocentron* Schmid, 1982:42 [Type species: Cnodocentron (Caenocentron) pallas Schmid 1982, original designation, as a subgenus].

The genus was divided into two subgenera when it was established, with the nominotypical subgenus containing six species, all from the Oriental region. The subgenus *Caenocentron* was established for the New World species and now contains seven species, six of them occurring in the Neotropics and one in the United States (*Cnodocentron yavapai*).

The larva, pupa, and biology of *Cnodocentron yavapai* from Arizona were described by Moulton and Stewart (1997a); the immature stages appear to be morphologically indistinguishable from those of *Xiphocentron*. The female of *Cnodocentron yavapai* was described by Ruiter (2006). Larvae of the Arizona species built typical tubes and larval gut contents contained diatoms and fine mineral deposits. Adults are rarely collected, but when seen are found in the daytime displaying on foliage beside streams.

***galesus*** (*Caenocentron*) Schmid, 1982:44 [Type locality: Costa Rica, 2.8 mi E. of Golfito; NMNH; ♂]. —Holzenthal, 1988c:58 [distribution]. —Aguila, 1992:537 [distribution]. —Armitage et al., 2015b:5 [checklist]. —Armitage and Cornejo, 2015:193 [checklist].

**Distribution.** Costa Rica, Panama.

***ideolus*** (*Caenocentron*) Schmid, 1982:106 [Type locality: Mexico, Lomas de Chapultepec, D.F.; INHS; ♂].

**Distribution.** Mexico.

***immaculatum*** (*Caenocentron*) Flint, 1991:34 [Type locality: Colombia, Dpto. Antioquia, Quebrada la Jimenez, Sopetran; NMNH; ♂]. —Muñoz-Quesada, 2000:281 [checklist].

**Distribution.** Colombia.

***lausus*** (*Caenocentron*) Schmid, 1982:44 [Type locality: Nicaragua, 4.2 mi W of Villa Somoza; NMNH; ♂]. —Maes and Flint, 1988:3 [distribution]. —Maes, 1999:1188 [checklist]. —Chamorro-Lacayo et al., 2007:47 [checklist].

**Distribution.** Nicaragua.

***pallas*** (*Caenocentron*) Schmid, 1982:44 [Type locality: Panama, Canal Zone, Gamboa, Rio Agua Salud; NMNH; ♂]. —Aguila, 1992:537 [distribution]. —Armitage et al., 2015b:5 [checklist]. —Armitage and Cornejo, 2015:193 [checklist].

**Distribution.** Panama.

***trilineatum*** (*Caenocentron*) (Mosely), 1934a:140 [Type locality: Mexico, Teapa, Tabasco; BMNH; ♂; in *Melanotrichia*]. —Bueno-Soria and Flint, 1978:197 [distribution; in *Xiphocentron*]. —Schmid, 1982:112 [checklist].

**Distribution.** Mexico.

#### Genus *Machairocentron* Schmid [6]

*Machairocentron* Schmid, 1982:46 [Type species: *Machairocentron lucumon* Schmid, 1982, original designation].

The genus contains six described species, all from the Neotropics. The immature stages of the genus were described by Pes et al. (2013). The adult habits and habitats are similar to those of other genera in the family.

***ascanius*** Schmid, 1982:48 [Type locality: Panama, Dolega; NMNH; ♂]. —Aguila, 1992:537 [distribution]. —Armitage et al., 2015b:5 [checklist]. —Armitage and Cornejo, 2015:193 [checklist].

**Distribution.** Panama.

***echinatum*** (Flint), 1981a:17 [Type locality: Venezuela, Aragua, Maracay, Rio Limon, Estacion Piscicultura; NMNH; ♂; in *Xiphocentron*].

—*carmentis* Schmid, 1982:48 [Type locality: Venezuela, Arag., Ocumare; NMNH; ♂]. —Flint et al., 1999a:81 [to synonymy].

**Distribution.** Venezuela.

***falciforme*** Pes and Hamada in Pes, et al., 2013:562 [Type locality: Brazil, Amazonas, Manaus, Reserva Ducke, Igarapé do Acará, 02°56'29.3"S, 59°56'07.4"W; INPA; ♂; ♀; pupa; biology]. —Paprocki and França, 2014:93 [checklist].

**Distribution.** Brazil.

***lucumon*** Schmid, 1982:50 [Type locality: Mexico, Ver., Rio Tacolapan, rt 180, Km 551; NMNH; ♂].

**Distribution.** Mexico.

***tarpeia*** Schmid, 1982:46 [Type locality: Mexico (Mich.), San Lorenzo, Rt 15, Km 206; NMNH; ♂].

**Distribution.** Mexico.

***teucrus*** Schmid, 1982:48 [Type locality: Panama, Playa Santa Clara; NMNH; ♂]. —Aguila, 1992:537 [distribution]. —Armitage et al., 2015b:5 [checklist]. —Armitage and Cornejo, 2015:193 [checklist].

**Distribution.** Panama.

#### Genus *Xiphocentron* Brauer [46 + †1]

*Xiphocentron* Brauer, 1870:66 [Type species: *Xiphocentron bilimekii* Brauer, 1871, by monotypy]. —Brauer, 1871:103. —Schmid, 1982:50 [revision]. —Vilarino and Calor, 2015a:47 [Brazilian species; key].

*Antillotrichia* Banks, 1941:401 [Type species: *Antillotrichia cubana* Banks, 1941, original designation]. —Flint, 1964a:25 [to synonymy]. —Botosaneanu, 1988:221 [resurrected as a valid subgenus].

*Sphagocentron* Schmid, 1982:54 [Type species: Xiphocentron (Sphagocentron) evandrus Schmid, 1982, original designation, as a subgenus].

*Glyphocentron* Schmid, 1982:56 [Type species: Xiphocentron (Glyphocentron) alcmeon Schmid, 1982, original designation, as a subgenus].

*Rhamphocentron* Schmid, 1982:60 [Type species: Xiphocentron (Rhamphocentron) mexico Ross, 1949a, original designation, as a subgenus].

*Xirocentron* Schmid, 1982:66 [Type species: Xiphocentron (Xirocentron) rhamnes Schmid, 1982, original designation, as a subgenus]. —Botosaneanu, 1988:221 [as synonym of subgenus *Antillotrichia*].

This genus currently contains 43 species, divided among five subgenera. It is limited exclusively to the New World, where it is found from the southwestern United States, south to west-central Argentina, and both the Greater and Lesser Antilles. The only known fossil species was described by Wichard et al. (2006) from Mexican amber.

The larvae and pupae of three species have been described: *Xiphocentron messapus* (Edwards 1961, Wiggins 1996), *Xiphocentron cubanum haitiensis* (Flint 1964a), and *Xiphocentron moncho* (Muñoz and Holzenthal, 1997). The pupal shelter with enclosed larva of *Xiphocentron parentum* was illustrated by Botosaneanu (1988). Pes et al. (2013) described adults and pupa and presented biological notes for *Xiphocentron sclerothrix*. The descriptions do not offer any apparent differences between the species. Larvae are found on rocks in fast-flowing water or, more commonly, on moist rocks at or above the water line. Sturm (1960) reported larval and pupal cases of *Xiphocentron sturmi* from the underside of wet logs in the rain forest. Muñoz and Holzenthal (1997) described a semiterrestrial larva from Costa Rica that constructed a short, portable retreat. Pes et al. (2013) analyzed the gut contents of a *Xiphocentron sclerothrix* larva and characterized it as having scraper feeding habits.

***albolineatum*** (*Antillotrichia*) Flint, 1968b:18 [Type locality: Dominica, Pont Casse, 1.7 miles east; NMNH; ♂]. —Schmid, 1982:113 [checklist, in subgenus *Xirocentron*]. —Flint and Sykora, 1993:53 [distribution]. —Botosaneanu, 2002:91 [checklist]. —Botosaneanu and Thomas, 2005:47 [distribution].

**Distribution.** Dominica, Martinique, St. Vincent.

***alcmeon*** (*Glyphocentron*) Schmid, 1982:58 [Type locality: Guatemala, Dept. San Marcos, Pte. Petacalapa; NMNH; ♂].

**Distribution.** Guatemala.

***alecto*** (*Rhamphocentron*) Schmid, 1982:62 [Type locality: Mexico (S.L.P.), Tierra Blanca, Rt 85, km 348; NMNH; ♂].

**Distribution.** Mexico.

***asilas*** (*Xiphocentron*) Schmid, 1982:52, 108 [Type locality: Mexico (SLP), Tierra Blanca, Rt 85, Km 348; NMNH; ♂].

**Distribution.** Mexico.

***aureum*** (*Xiphocentron*) Flint, 1967b:10 [Type locality: Mexico (Vera Cruz), near Huatusco; NMNH; ♂]. —Bueno-Soria and Flint, 1978:197 [distribution]. —Schmid, 1982:52, 113 [discussion, ♂ wing venation]. —Aguila, 1992:537 [distribution]. —Armitage et al., 2015b:5 [checklist]. —Armitage and Cornejo, 2015:193 [checklist].

**Distribution.** Mexico, Panama.

***bilimekii*** (*Xiphocentron*) Brauer, 1871:104 [Type locality: Mexico; NMW; ♂]. —Flint, 1966a:4 [lectotype; ♂]. —Schmid, 1982:52 [discussion].

**Distribution.** Mexico.

***borinquensis*** (*Antillotrichia*) Flint, 1964a:29 [Type locality: [Puerto Rico] El Yunque; NMNH; ♂]. —Flint, 1968b:80 [checklist]. —Schmid, 1982:114 [checklist]. —Botosaneanu, 2002:91 [checklist].

**Distribution.** Puerto Rico.

***caenina*** (*Antillotrichia*) Schmid, 1982:110 [Type locality: Argentina [not Mexico as stated in original description], Quebrada Cainzo, Dept. Tafi Viejo, Tuc.; INHS; ♂; in *Xirocentron*]. —Isa Miranda and Rueda Martín, 2014:200 [distribution].

**Distribution.** Argentina.

† ***chiapasi*** (*Xiphocentron*) Wichard, Solórzano-Kraemer and Luer, 2006:46 [Type locality: Mexico; Collection Poinar; ♂; in amber].

**Distribution.** Mexico.

***cubanum caimitense*** (*Antillotrichia*) Kumanski, 1987:8 [Type locality: [Cuba], Province Pinar del Rio, ca. 15 km SE from La Palma, Rio El Caimito; NMNSB; ♂; as *caimitensis*]. —Botosaneanu, 1993:296 [to subspecific status of *cubanum*]. —Flint, 1996c:16 [checklist]. —Botosaneanu, 2002:91 [checklist]. —Naranjo López and González Lazo, 2005:150 [checklist].

**Distribution.** Cuba.

***cubanum cubanum*** (*Antillotrichia*) (Banks), 1941:401 [Type locality: Hanabanillo Falls, Trinidad Mts., Cuba; MCZ; ♂; in *Antillotrichia*]. —Flint, 1964a:26 [to *Xiphocentron*]. —Flint, 1967c:7 [genitalia missing from holotype]. —Flint, 1968b:80 [checklist]. —Botosaneanu, 1979:45 [distribution]. —Schmid, 1982:114 [checklist]. —Kumanski, 1987:8 [♂]. —Botosaneanu, 1993:293 [♂; relationship of Cuban subspecies]. —Flint, 1996c:16 [checklist]. —Botosaneanu, 2002:91 [checklist]. —López del Castillo et al., 2004:229 [distribution]. —González Lazo et al., 2005:261 [checklist]. —Naranjo López and González Lazo, 2005:150 [checklist]. —López del Castillo et al., 2007:171 [distribution; seasonal abundance].

**Distribution.** Cuba.

***cubanum orientale*** (*Antillotrichia*) Botosaneanu, 1993:296 [Type locality: Cuba, Oriente, Arroyo Seboruquito, Mayari, foot of Sierra Pinares de Mayari; ZMUA; ♂]. —Flint, 1996c:16 [checklist]. —Botosaneanu, 2002:92 [checklist]. —Naranjo López and González Lazo, 2005:150 [checklist].

**Distribution.** Cuba.

***cuyensis*** (*Antillotrichia*) Flint, 1983a:34 [Type locality: Argentina, Pcia. Tucuman, Rt 307, 33.7 km W Acheral; NMNH; ♂]. —Isa Miranda and Rueda Martín, 2014:200 [distribution].

**Distribution.** Argentina.

***erato*** (*Rhamphocentron*) Schmid, 1982:62 [Type locality: Mexico (S.L.P.), Tierra Blanca, Rt 85, km 348; NMNH; ♂].

**Distribution.** Mexico.

***euryale*** (*Glyphocentron*) Schmid, 1982:58 [Type locality: Costa Rica 14.1 m SE of Esparta; NMNH; ♂]. —Holzenthal, 1988c:58 [distribution].

**Distribution.** Costa Rica.

***evandrus*** (*Sphagocentron*) Schmid, 1982:56 [Type locality: Costa Rica, Juan Vinas, Rio Chiz; NMNH; ♂]. —Holzenthal, 1988c:58 [distribution]. —Armitage et al., 2015a:3 [distribution]. —Armitage et al., 2015b:5 [checklist]. —Armitage and Cornejo, 2015:193 [checklist].

**Distribution.** Costa Rica, Panama.

***fuscum*** (*Antillotrichia*) Flint, 1968b:16 [Type locality: Dominica, Brantridge; NMNH; ♂]. —Schmid, 1982:113 [checklist, in *Xirocentron*]. —Flint and Sykora, 1993:51 [distribution]. —Botosaneanu, 1994a:51 [distribution]. —Botosaneanu, 2002:92 [checklist]. —Botosaneanu and Thomas, 2005:55 [checklist].

**Distribution.** Dominica, Guadeloupe, St Lucia.

***haitiense*** (*Antillotrichia*) (Banks), 1941:402 [Type locality: Camp Perin, Haiti; MCZ; ♂; as *Antillotrichia haitiensis*]. —Flint, 1964a:26 [to *Xiphocentron*; ♂; ♀; larva; pupa; as *haitiensis*]. —Flint, 1967c:7 [holotype; ♂]. —Flint, 1968b:80 [checklist]. —Schmid, 1982:114 [checklist]. —Botosaneanu, 1993:293 [suggested synonymy with *cubanum*]. —Botosaneanu, 1996:12 [to subspecific status of *cubanum*]. —Flint and Pérez-Gelabert, 1999:43 [checklist; as *cubanum haitiense*]. —Botosaneanu, 2002:92 [checklist; as subspecies]. —Flint and Sykora, 2004:55 [to species status, as *haitiense*, distribution]. —Pérez-Gelabert, 2008:302 [checklist]. —Djernaes, 2011:12 [♂; ♀].

**Distribution.** Dominican Republic, Haiti, Puerto Rico.

***ilionea*** (*Antillotrichia*) Schmid, 1982:70 [Type locality: Brazil, [São Paulo], Estacion Biol. Boraceia, Pedreira; NMNH; ♂; in *Xirocentron*]. —Paprocki et al., 2004:16 [checklist]. —Dumas et al., 2009:361 [distribution]. —Calor, 2011:323 [checklist]. —Paprocki and França, 2014:93 [checklist]. —Vilarino and Calor, 2015a:52 [♂; key].

**Distribution.** Brazil.

***insulare*** (*Antillotrichia*) (Ulmer), 1913:386 [Type locality: Trinidad (Waterfall, Diego Martin); BMNH; ♂; in *Melanotrichia*]. — Flint, 1964a:26 [to *Xiphocentron*]. —Schmid, 1982:114 [checklist, in *Xirocentron*]. —Flint, 1996a:81 [identity, synonymy]. —Botosaneanu, 2002:92 [checklist].

—*trinitatis* (Lestage), 1926:382 [Type locality: same as *insulare*; BMNH; ♂; in *Melanotrichia*; type is the type of *insulare*]. —Fischer, 1962:233 [to synonymy].

—*nisus* (*Xirocentron*) Schmid, 1982:70 [Type locality: Trinidad, Simla; NMNH; ♂]. —Flint, 1996a:81 [to synonymy].

**Distribution.** Trinidad.

***julus*** (*Sphagocentron*) Schmid, 1982:56 [Type locality: Mexico (Ver.), Puente Nacional; NMNH; ♂].

**Distribution.** Mexico.

***kamakan*** (*Antillotrichia*) Vilarino and Calor, 2015a:47 [Type locality: Brazil, Bahia, Camacan, RPPN Serra Bonita, Córrego 3, Chuchuzeiro, 15°23'03"S, 039°34'00"W; MZUSP; ♂].

**Distribution.** Brazil.

***lavinia*** (*Rhamphocentron*) Schmid, 1982:64 [Type locality: Guatemala, Dept. Izabal, nr Matias de Galvez; NMNH; ♂]. —Bueno-Soria and Barba-Álvarez, 2011:361 [checklist].

**Distribution.** Guatemala, Mexico.

***lobiferum*** (*Antillotrichia*) Flint and Sykora, 1993:52 [Type locality: Grenada, Parish St. Patrick, Plains; FSCA; ♂]. —Flint and Sykora, 1993:49 [checklist]. —Botosaneanu, 2002:92 [checklist].

**Distribution.** Grenada.

***maiteae*** (*Antillotrichia*) Vilarino and Calor, 2015a:50 [Type locality: Brazil, Bahia, Camacan, RPPN Serra Bonita, Córrego 3, Chuchuzeiro, 15°23'03"S, 039°34'00"W; MZUSP; ♂].

**Distribution.** Brazil.

***messapus*** (*Rhamphocentron*) Schmid, 1982:64, 108 [Type locality: United States, Texas, Springs along Blanco River, below Blanco; ROM; ♂]. —Edwards, 1961:51 [as *Xiphocentron mexico* Ross; larva; pupa; biology]. —Wiggins, 1996:186 [larva; pupa; biology]. —Bowles et al., 2007:23 [distribution; biology]. —Bueno-Soria and Barba-Álvarez, 2011:362 [checklist].

**Distribution.** Mexico, U.S.A.

***mexico*** (*Rhamphocentron*) Ross, 1949a:4 [Type locality: Villa Santiago, Nueva Leon, Mexico; INHS; ♂]. —Bueno-Soria and Flint, 1978:197 [distribution]. —Schmid, 1982:82, 113 [discussion, to *Rhamphocentron*, male leg spurs]. —Bueno-Soria et al., 2005:75 [distribution; as *movica*, misspelling of *mexico*?].

**Distribution.** Mexico.

***mezencius*** (*Antillotrichia*) Schmid, 1982:108 [Type locality: Mexico, Rio Frio; INHS; ♂].

**Distribution.** Mexico.

***mnesteus*** (*Antillotrichia*) Schmid, 1982:69 [Type locality: Venezuela (Ba.), 30 km NW Barinitas; NMNH; ♂; in *Xirocentron*]. —Flint, 1991:33 [♂; distribution]. —Muñoz-Quesada, 2000:281 [checklist].

**Distribution.** Colombia, Venezuela.

***moncho*** (*Antillotrichia*) Muñoz and Holzenthal, 1997:357 [Type locality: Costa Rica, Alajuela, Reserva Forestal San Ramón, Río San Lorencito and tribs., 10.216°N, 84.607°W; NMNH; ♂; ♀; larva; pupa; case; biology].

**Distribution.** Costa Rica.

***nesidion*** (*Antillotrichia*) Flint, 1968a:22 [Type locality: [Jamaica], Trelawny, Martha Brae near Falmouth; NMNH; ♂]. —Flint, 1968b:80 [checklist]. —Schmid, 1982:114 [checklist, in *Xirocentron*]. —Botosaneanu and Hyslop, 1998:18 [as subspecies of *cubanum*]. —Flint et al., 1999b:186 [to species status]. —Botosaneanu, 2002:92 [checklist; as subspecies of *cubanum*].

**Distribution.** Jamaica.

***numanus*** (*Rhamphocentron*) Schmid, 1982:66 [Type locality: Mexico (Oax.), Tamazulapan; NMNH; ♂].

**Distribution.** Mexico.

***parentum*** (*Antillotrichia*) Botosaneanu, 1988:219 [Type locality; [Martinique], Ravine l’Abbé (Morne-Vert); ZMA; ♂; larva]. —Botosaneanu, 1990a:42 [♀]. —Flint and Sykora, 1993:49 [checklist]. —Botosaneanu, 2002:92 [checklist]. —Botosaneanu and Thomas, 2005:47 [distribution].

**Distribution.** Martinique.

***pintada*** (*Antillotrichia*) Flint, 1983a:34 [Type locality: Argentina, Pcia. Catamarca, Arroyo El Pintada, near La Vina; NMNH; ♂].

**Distribution.** Argentina.

***piscicaudum*** (*Antillotrichia*) Flint, 1996a:77 [Type locality: Tobago, Bridge B1/5, 6.5 km N Roxborough; NMNH; ♂]. —Botosaneanu, 2002:92 [checklist].

**Distribution.** Tobago, Venezuela.

***polemon*** (*Xiphocentron*) Schmid, 1982:108 [Type locality: Mexico, Pedregal, San Angel, D.F.; INHS; ♂].

**Distribution.** Mexico.

***prolixum*** (*Antillotrichia*) Flint, 1996a:78 [Type locality: Trinidad, Guanapo River, Lalaja Road, 10°43'N, 61°17'W; NMNH; ♂]. —Botosaneanu, 2002:92 [checklist].

**Distribution.** Trinidad.

***regulare*** (*Antillotrichia*) Flint, 1991:33 [Type locality: Colombia, Dpto. Antioquia, 12 km N Fredonia; NMNH; ♂]. —Muñoz-Quesada, 2000:281 [checklist].

**Distribution.** Colombia.

***rhamnes*** (*Antillotrichia*) Schmid, 1982:68 [Type locality: Mexico (Ver.), N. Huatusco; NMNH; ♂; in *Xirocentron*].

**Distribution.** Mexico.

***saltuum*** (unplaced) (Müller), 1921:531, fig. 184k [Type locality: Brazil; type depository unknown; pupal mandible; in *Hydropsyche*]. —Ulmer, 1957:316 [bibliography, to *Tinodes*]. —Flint et al., 1999a:81 [to *Xiphocentron*, as *nomen dubium*]. —Paprocki et al., 2004:16 [checklist]. —Paprocki and França, 2014:93 [checklist]. —Vilarino and Calor, 2015a:52 [as *nomen dubium*].

**Distribution.** Brazil.

***sclerothrix*** (*Antillotrichia*) Pes and Hamada in Pes, et al., 2013:568 [Type locality: Brazil, Amazonas, Presidente Figueiredo, igarapé da Caverna do Maroagoa, km 6 AM 240, 01°04'20.33"S, 59°58'54.24"W; INPA; ♂; ♀; pupa; biology]. —Paprocki and França, 2014:93 [checklist]. —Vilarino and Calor, 2015a:52 [♂; key].

**Distribution.** Brazil.

***serestus*** (*Antillotrichia*) Schmid, 1982:68 [Type locality: Mexico (Mich.), P.N. Morelos, nr Morelia; NMNH; ♂; in *Xirocentron*].

**Distribution.** Mexico.

***steffeni*** (*Antillotrichia*) (Marlier), 1964a:6 [Type locality: Brazil, São Paulo, Boracéia; IRSNB; ♂; in *Melanotrichia*]. —Schmid, 1982:114 [to Xiphocentron (Xirocentron)]. —Paprocki et al., 2004:16 [checklist]. —Dumas et al., 2009:361 [distribution]. —Dumas et al., 2010:9 [distribution]. —Calor, 2011:323 [checklist]. —Dumas and Nessimian, 2012:24 [checklist]. —Paprocki and França, 2014:93 [checklist]. —Vilarino and Calor, 2015a:52 [♂; key].

**Distribution.** Brazil.

***stenotum*** (*Antillotrichia*) Flint, 1996a:78 [Type locality: Tobago, Doctor River, 1 km NW Speyside, 11°18'N, 60°32'W; NMNH; ♂]. —Botosaneanu, 2002:92 [checklist].

**Distribution.** Tobago.

***sturmi*** (*Antillotrichia*) Sturm, 1960:387 [Type locality: Colombia, Strasse Cali-Buenaventura; unspecified; larval and pupal retreats]. —Schmid, 1982:114 [*nomen dubium*]. —Malicky 1987b:97 [available name, clarification of type locality: [label data] Colombia, Anchicaya (35 km WNW Cali); collection Malicky; ♂ lectotype]. —Muñoz-Quesada, 2000:281 [checklist].

**Distribution.** Colombia.

***surinamense*** (*Antillotrichia*) Flint, 1974c:32 [Type locality: Suriname, Brownsberg, mountain creek near Golddigers camp; RNH; ♂]. —Schmid, 1982:114 [checklist, in *Xirocentron*].

**Distribution.** Suriname.

***tarquon*** (*Xiphocentron*) Schmid, 1982:54 [Type locality: Mexico (Chis.), Rt 195, 5 km S. Ixtacomitan; NMNH; ♂]. —Bueno-Soria et al., 2005:76 [distribution]. —Bueno-Soria and Barba-Álvarez, 2011:362 [checklist].

**Distribution.** Mexico.

### Trichoptera, *incertae sedis*

#### Genus *Eutonella* Müller [1]

*Eutonella* Müller, 1921:531 [Type species: *Eutonella peltopsychoides* Müller 1921, by monotypy]. —Ulmer, 1957:316 [systematic placement].

The systematic position of this genus, for which only a figure of a pupal mandible is known, is not certain. As discussed in Flint et al. (1999a), it could be either a hydroptilid or psychomyiid, but was placed by them in the Hydroptilidae on the preponderance of evidence. Santos et al., (2016) further stated that the tibial spur formula of 2-4-4, as indicated by Müller (1880a, 1880b), does not match that of any Hydroptilidae. Accordingly, they considered it not to be in Hydroptilidae, but instead placed this pooly described species in Trichoptera *incertae sedis*.

***peltopsychodes*** Müller, 1921:531, fig. 184 1 [Type locality: Brazil; type depository unknown; pupal mandible]. —Ulmer, 1957:316 [bibliography]. —Flint et al., 1999a:76 [identity; to Hydroptilidae]. —Paprocki et al., 2004:11 [checklist]. —Paprocki and França, 2014:44 [checklist]. —Santos et al., 2016a:460 [to Trichoptera *incertae sedis*]

**Distribution.** Brazil.

## Index

### A

***abbreviata***, *Cernotina* 412

***abbreviata***, *Neotrichia* 246

***abbreviatoides***, *Neotrichia* 246

***abdominalis***, *Phylloicus* 29

***aberrans***, *Metrichia* 233

***aberrans***, *Monocosmoecus* 353

*abjurans*, *Centromacronema* 129

***abrachium***, *Austrotinodes* 37

***abrelata***, *Byrsopteryx* 206

***abrelata***, *Ochrotrichia* 261

***abrupta***, *Smicridea* 161

***abruptum***, *Parasericostoma* 450

***abruptus***, *Triaenodes* 342

***absona***, *Oxyethira* 281

*Abtrichia*, [Hydroptilidae] 293

***acaena***, *Culoptila* 48

***acahuana***, *Atopsyche* 100

***acalyptra***, *Cernotina* 412

***acanthogaster***, *Polycentropus* 422

***acanthostema***, *Oecetis* 333

***acanthus***, *Triaenodes* 342

***acauda***, *Mortoniella* 55

***acciptrina***, *Oecetis* 333

***acegua***, *Oxyethira* 281

**Achoropsyche** [Leptoceridae] 312

***acicula***, *Metrichia* 233

***acinaciformis***, *Chimarra* 370

***acinaciformis***, *Polycentropus* 422

**Acostatrichia** [Hydroptilidae] 192

*acreata*, *Scotiotrichia* See ***ocreata***, *Scotiotrichia*

***acula***, *Chimarra* 370

***aculea***, *Oxyethira* 281

***aculeatus***, *Phylloicus* 29

***acuminata***, *Atanatolica* 315

***acuminata***, *Hydroptila* 215

***acuminata***, *Metrichia* 233

***acuminata***, *Smicridea* 161

***acuta***, *Chimarra* 370

***acuti***, *Angrisanoia* 201

***acutiloba***, *Nectopsyche* 322

*acutissima*, *Hydroptila* 218

***acutiterga***, *Banyallarga* 26

***acutum***, *Leptonema* 135

***acutum***, *Parasericostoma* 450

***adamsae***, *Austrotinodes* 37

***adamsae***, *Chimarra* 370

***adamsae***, *Contulma* 21

***adamsae***, *Metrichia* 233

***adamsae***, *Phylloicus* 29

***adamsae***, *Polyplectropus* 433

***adela***†, *Leucotrichia* 225

***adella***, *Chimarra* 370

***adelphe***, *Chimarra* 370

***adunca***, *Cerasmatrichia* 208

***adusta***, *Nectopsyche* 322

***aegerfasciella***, *Orthotrichia* 279

***aeneus***, *Phylloicus* 29

*aeola*, *Nectopsyche* 329

***aequalis***, *Mortoniella* 55

***aequalis***, *Smicridea* 161

***aequatoria***, *Chimarrhodella* 400

***aequispina***, *Eosericostoma* 77

*aequispina*, *Eosericostoma* Schmid 1957, *nec* Schmid 1955a 78

***aequispina***, *Neotrichia* 246

***aequiunguis***, *Grumichella* 318

***aestheticella***, *Cernotina* 412

***affinis***, *Alterosa* 365

***affinis***, *Necrotaulius* [†] 10

***affinis***, *Ochrotrichia* 261

***affinis***, *Verger* 355

***agaboga***, *Anchitrichia* 200

***agazoka***, *Angrisanoia* 201

*agglutinans*, *Helicopsyche* 88

***aglae***, *Alisotrichia* 194

*agnathum*, *Macronema* 150

***agosta***, *Mortoniella* 56

***agosta***, *Oecetis* 333

***agraphum***, *Leptonema* 135

***aguilerai***†, *Culoptila* 48

***aguyje***, *Polycentropus* 422

***aitija***, *Alisotrichia* See ***hirudopsis aitija***

***aiuruoca***, *Marilia* 360

***ajax***, *Hydroptila* 215

***akantha***, *Chimarra* 371

***akantha***, *Mortoniella* 56

***alafimbriata***, *Mystacides* 321

***alajuela***, *Flintiella* 213

***alajuela***, *Helicopsyche* 80

***alajuela***, *Metrichia* 233

***alaluz***, *Oxyethira* 281

*alamosa*, *Chimarra* 385

***alarca***, *Zumatrichia* 301

***alargada***, *Ochrotrichia* 261

***alata***, *Chimarra* 371

***alata***, *Marilia* 360

***alata***, *Neotrichia* 246

***alatespinus***, *Polyplectropus* 433

***alatus***, *Polycentropus* 422

***alayoana***, *Alisotrichia* 194

*alayoi*, *Chimarra* 388

***albaeaquae***, *Oxyethira* 281

*albata*, *Smicridea* 165

*albescens*, *Smicridea* 168

***albicornis***, *Marilia* 360

***albifrontalis***, *Smicridea* 161

***albolineata***, *Mortoniella* 56

***albolineatum***, *Xiphocentron* 456

***albomaculata***, *Chimarra* 371

***albosignata***, *Smicridea* 161

***albovirens***, *Leptonema* 135

***album***, *Leptonema* 136

***alceatum***, *Leptonema* 136

***alcmeon***, *Xiphocentron* 456

*alconura*, *Atopsyche* 102

***aldama***, *Ochrotrichia* 262

***aldricki***, *Hydroptila* 215

***alecto***, *Xiphocentron* 456

***alexanderi***, *Itauara* 51

*alexanderi*, *Lepidostoma* 310

***alexanderi***, *Protoptila* 67

***alhoma***, *Metrichia* 233

***alibrachia***, *Leucotrichia* 225

***aliceae***†, *Ochrotrichia* 262

***alicia***, *Wormaldia* 404

***aliciae***, *Polycentropus* 422

***alicula***, *Mortoniella* 56

***aliena***, *Kumanskiella* 224

***alienus***, *Polyplectropus* 434

***alisensis***, *Leucotrichia* 225

**Alisotrichia** [Hydroptilidae] 194

***allani***, *Atopsyche* 100

***alleni***, *Polyplectropus* 434

**Alloecentrellodes** [Helicophidae] 76

***alsa***, *Neotrichia* 246

***altercoma***, *Helicopsyche* 80

**Alterosa** [Philopotamidae] 365

***alticola***, *Smicridea* 161

***altmani***, *Chimarra* 371

***altmani***, *Polycentropus* 422

***altura***, *Protoptila* 67

***aluca***, *Culoptila* 48

***alumnorum***, *Protoptila* 67

***alvarezi***†, *Plectropsyche* 159

***alyshae***, *Neotrichia* 247

***alza***, *Bredinia* 204

***amadoi***, *Alterosa* 365

***amazona***, *Helicopsyche* 81

***amazonas***, *Phylloicus* 30

**Amazonatolica** [Leptoceridae] 312

***amazonense***, *Leptonema* 136

***amazonense***, *Macronema* 150

***amazonensis***, *Austrotinodes* 38

***amazonensis***, *Taraxitrichia* 299

***amazoniana***, *Notidobiella* 449

***amazonica***, *Chimarra* 371

***amazonica***, *Itauara* 51

***amazonica***, *Oecetis* 333

***amazonicus***, *Polyplectropus* 434

***amberia***, *Culoptila* 48

*ambitiosa*, *Nectopsyche* 328

*americana*, *Orthotrichia* 279

***amica***, *Chimarra* 371

***amica***, *Helicopsyche* 81

***amnicola***, *Marilia* 360

***amorfa***, *Ochrotrichia* 262

**Amphichorema** [Hydrobiosidae] 98

***amphirhamphus***, *Polycentropus* 423

**Amphoropsyche** [Leptoceridae] 313

***amplector***, *Neotrichia* 247

***amplifurcatum***, *Leptonema* 136

***amplio***, *Neotrichia* 247

***amplispina***, *Smicridea* 161

***amplitudinis***, *Metrichia* 233

***anahua***, *Neotrichia* 247

***anakantha***, *Mortoniella* 56

**Anastomoneura** [Odontoceridae] 358

***anaticula***, *Smicridea* 161

***ancestralis***, *Hydropsyche* 133

**Anchitrichia** [Hydroptilidae] 200

***anchorus***, *Polyplectropus* 434

***ancistrion***, *Hydroptila* 215

***ancistrus***, *Polycentropus* 423

***ancora***, *Metrichia* 233

***ancylus***, *Austrotinodes* 38

***andicola***, *Smicridea* 162

***andina***, *Oxyethira* 281

***andina***, *Rhyacopsyche* 295

***andinensis***, *Polyplectropus* 434

***andinum***, *Leptonema* 136

***andrea***, *Leptonema* 136

***andrea***, *Wormaldia* 404

***andreae***, *Flintiella* 213

**Androchorema** [Hydrobiosidae] 98

*angelae*, *Helicopsyche* 90

***angelae***, *Oecetis* 333

***angelinae***, *Leucotrichia* 225

***angeloi***, *Helicopsyche* 81

***angelus***, *Orinocotrichia* 278

***angra***, *Rhyacopsyche* 295

***angrisanae***, *Ragatrichia* 294

**Angrisanoia** [Hydroptilidae] 201

***angularis***, *Chimarra* 371

***angularis***, *Ochrotrichia* 262

***angularis***, *Oecetis* 333

***angulata***, *Helicopsyche* 81

***angulata***, *Mortoniella* 56

***angulata***, *Neotrichia* 247

***angulata***, *Sortosa* 402

***angulata***, *Zumatrichia* 301

***angulatum***, *Microthremma* 78

***angulosa***, *Metrichia* 233

***angusta***, *Hydroptila* 215

***angustior***, *Austrotinodes* 38

***angustior***, *Phylloicus* 30

*angustipennis*, *Cailloma* 115

***angustipennis***, *Chimarra* 371

***anhanguera***, *Cernotina* 412

***anhelitus***, *Wormaldia* 404

***anisoscola***, *Metrichia* 233

*annulicornis* (Blanchard), *Smicridea* 168

*annulicornis* (Ulmer), *Smicridea* 168

*annulicornis*, *Helicopsyche* 82

***annulicornis***, *Polyplectropus* 434

***annulicornis***, *Smicridea* 162

***anomala***, *Ochrotrichia* 262

***anomala***, *Smicridea* 162

**Anomalocosmoecus** [Limnephilidae] 348

**Anomalopsyche** [Anomalopsychidae] 20

***anomaloptera***, *Zumatrichia* 301

***anomalum***, *Leptonema* 136

***anomalus***, *Neoathripsodes* 330

*anomalus*, *Polycentropus* 428

***anomalus***, *Setodes* 341

***anomalus***, *Triaenodes* 342

**Antarctoecia** [Limnephilidae] 348

*Antarctopsyche* [Hydropsychidae] 160

***antennata***, *Peltopsyche* 293

***anticheira***, *Chimarra* 372

***anticura***, *Smicridea* 162

***antigua***, *Chimarra* 372

*antillana*, *Oecetis* 337

***antilles***, *Streptopsyche* 187

***antilliana***, *Chimarra* 372

***antilliarum***, *Hydroptila* 215

***antilliensis***, *Zumatrichia* 301

**Antillopsyche** [Polycentropodidae] 444

***Antillotrichia*** [Xiphocentronidae] 455

***antillularum***, *Oxyethira* See ***tega antillularum***

***antisuya***, *Atopsyche* 100

***antonina***, *Cernotina* 412

*Antoptila* [Glossosomatidae] 51

**Apatanodes** [Hydrobiosidae] 98

*aperiens*, *Barypenthus* 359

***apicale***, *Centromacronema* 129

*apicale*, *Macronema* 150

*apicalis*, *Oecetis* 337

*apicata*, *Oecetis* 335

***apicauda***, *Helicopsyche* 81

***apiculata***, *Mortoniella* 56

***apinolada***, *Oxyethira* 281

***aplita***, *Atopsyche* 100

***appendicula***, *Smicridea* 162

***appendiculata***, *Bredinia* 204

***appendiculata***, *Smicridea* 162

***appendiculata***, *Sortosa* 402

***appendiculatus***, *Verger* 355

***apurimac***, *Atopsyche* 100

***aquaecadentis***, *Alisotrichia* 194

***aragua***, *Amphoropsyche* 313

***araguaiense***, *Smicridea* 162

***araguense***, *Leptonema* 136

***araguensis***, *Metrichia* 233

***arantala***, *Oxyethira* 282

***araucanica***, *Australobiosis* 114

***araujoi***, *Wormaldia* 404

***arawak***, *Cariboptila* 44

***arcana***, *Alisotrichia* 195

***archboldi***, *Leptonema* 136

*arcopa*, *Wormaldia* 408

***arctia***, *Hydroptila* 216

***arctodactyla***, *Oxyethira* 282

*arcuata*, *Macronema* See ***arcuatum***, *Macrostemum*

***arcuatum***, *Macrostemum* 154

***ardisia***, *Calosopsyche* 126

*arenifera*, *Helicopsyche* 82

***arenifera***, *Metrichia* 234

*areopagita*, *Cariboptila* 47

***argentata***, *Nectopsyche* 322

***argentea***, *Ochrotrichia* 262

***argentella***, *Chimarra* 372

***argentilinea***, *Alisotrichia* 195

***argentilineatum***, *Macronema* 150

***argentina***, *Smicridea* 162

***argentinensis***, *Oxyethira* 282

***argentinica***, *Banyallarga* 26

***argentinica***, *Betrichia* 203

***argentinica***, *Chimarra* 372

***argentinica***, *Hydroptila* 216

***argentinica***, *Metrichia* 234

***argentinica***, *Mortoniella* 56

***argentinicus***, *Anomalocosmoecus* 348

*argentinus*, *Badallus* See ***argentina***, *Smicridea*

*argentinus*, *Nyctiophylax* See ***muhnianus***, *Nyctiophylax*

*Argentitrichia* [Hydroptilidae] 232

***argylensis***, *Cerasmatrichia* 209

***Argyrobothrus*** , [Hydroptilidae] 280

***ariasi***, *Austrotinodes* 38

***ariensis***, *Polycentropus* 423

***aries***, *Mortoniella* 57

***aries***, *Smicridea* 163

***arima***, *Chimarra* 372

***arista***, *Neotrichia* 247

***arizela***†, *Alisotrichia* 195

***arizona***, *Oxyethira* 282

***arizonensis***, *Cheumatopsyche* 131

*arizonensis*, *Helicopsyche* 89

***arizonensis***, *Metrichia* 234

***arizonensis***, *Polycentropus* 423

***arizonensis***, *Smicridea* 163

***arizonensis***, *Wormaldia* 404, 405

***arizonica***, *Oecetis* 333

*armata*, *Chimarra* 372

***armata***, *Mortoniella* 57

***armata***, *Neotrichia* 247

***armatus***, *Verger* 356

***armiger***, *Austrotinodes* 38

***arotron***, *Cyrnellus* 418

***arranca***, *Ochrotrichia* 262

***arriba***, *Ochrotrichia* 262

***artiguensis***, *Cernotina* 412

***aruma***, *Cernotina* 412

***asancaru***, *Atopsyche* 100

***ascanius***, *Machairocentron* 455

***asclepium***, *Leptonema* 137

**Ascotrichia** [Hydroptilidae] 202

***asilas***, *Xiphocentron* 456

***aspersum***, *Leptonema* 137

***aspinosus***, *Polycentropus* 423

*assimilis*, *Notidobia* 449

*assimilis*, *Phylloicus* 34

***assita***, *Ochrotrichia* 262

***asta***, *Alisotrichia* 195

***astarte***, *Smicridea* 163

***astera***, *Cernotina* 412

***astilla***, *Flintiella* 213

***asymmetris***, *Mortoniella* 57

***atahuallpa***, *Atopsyche* 100

**Atanatolica** [Leptoceridae] 314

***atenuata***, *Mortoniella* 57

***aterrima***, *Smicridea* 163

***aterrimum***, *Leptonema* 137

***atezcae***, *Ochrotrichia* 263

***atilanoi***, *Chimarra* 372

***atmena***, *Smicridea* 163

***atmena***, *Zumatrichia* 301

*Atomyiodes* [Lepidostomatidae] 307

***Atopsaura*** [Hydrobiosidae] 99

**Atopsyche** [Hydrobiosidae] 99

*Atopsychodes* [Hydrobiosidae] 99

***atrobasis***, *Smicridea* 163

***attenuata***, *Cernotina* 412

***attenuata***, *Ochrotrichia* 263

***attenuata***, *Zumatrichia* 302

***aurantibasis***, *Chimarra* 373

***auratus***, *Phylloicus* 30

***aurea***, *Atanatolica* 315

***aureofasciata***, *Nectopsyche* 322

***aureoinclusa***†, *Setodes* 341

***aureopunctata***, *Chimarra* 373

***aureovittata***, *Nectopsyche* 322

***aureum***, *Xiphocentron* 456

***auricolor***, *Hydropsyche* 133

***auricula***†, *Antillopsyche* 444

***auriculatum***, *Leptonema* 137

*aurimacula*, *Smicridea* 183

***auripenne***, *Centromacronema* 129

***auriplicatus***, *Polyplectropus* 434

***aurivittata***, *Chimarra* 373

***auroa***, *Helicopsyche* 81

***aurra***, *Smicridea* 163

***aurulenta***, *Cariboptila* 44

*australis*, *Verger* 356

**Australobiosis** [Hydrobiosidae] 114

*Australochorema* [Hydrobiosidae] 99

*Australomyia* [Limnephilidae] 355

**Austrocentrus** [Helicophidae] 76

**Austrocosmoecus** [Limnephilidae] 349

**Austrotinodes** [Ecnomidae] 37

***aviceps***, *Chimarra* 373

***aviceps***, *Mortoniella* 57

***avicula***, *Ochrotrichia* 263

***avis***, *Ochrotrichia* 263

***avon***, *Metrichia* 234

***ayacucho***, *Atopsyche* 101

***ayahuaca***, *Atopsyche* 101

***ayama***, *Mayatrichia* 230

***ayaya***, *Ochrotrichia* 263

***aycara***, *Antillopsyche* 444

***aymara***, *Wormaldia* 404

***ayura***, *Amphoropsyche* 313

***ayura***, *Leucotrichia* 225

***azteca***, *Oxyethira* 282

***aztecum***, *Lepidostoma* 308

***aztecus***, *Polycentropus* 423

***azul***, *Metrichia* 234

***azulae***, *Culoptila* 48

***azulus***, *Polycentropus* 423

### B

***bacula***, *Contulma* 21

*Badallus* [Hydropsychidae] 160

***baja***, *Limnephilus* 351

***bakeri***, *Lepidostoma* 308

***balra***, *Ceratotrichia* 210

***balra***, *Ochrotrichia* 263

***bandeira***, *Alterosa* 365

***banksi***, *Atopsyche* 101

***banksi***, *Chimarra* 373

***banksi***, *Leptonema* 137

***banksi***, *Smicridea* 163

***banksianus***, *Polyplectropus* 434

**Banyallarga** [Calamoceratidae] 25

***baoruco***, *Chimarra* See ***spinulifera baoruco***

***baorucoensis***, *Ochrotrichia* 263

***barbai***, *Wormaldia* 404

***barinas***, *Chimarra* 373

***barinita***, *Chimarra* 373

***baritu***, *Neotrichia* 247

***baritu***, *Oxyethira* 283

*barranca*, *Chimarra* 381

***barrerai***, *Culoptila* 48

***barrettae***, *Chimarra* 373

***bartolus***, *Polycentropus* 423

**Barypenthus** [Odontoceridae] 359

***batesi***, *Atopsyche* 101

***batesi***, *Calosopsyche* 126

***baumanni***, *Marilia* 360

***beameri***, *Chimarra* 374

***beaumonti***, *Platycosmoecus* 354

*Beaumontia* [Limnephilidae] 354

***beccus***, *Polyplectropus* 434

***beckeri***, *Alterosa* 365

***beckeri***, *Chimarra* 374

***befela***, *Zumatrichia* 302

***befoga***, *Alisotrichia* 195

***begorba***, *Smicridea* 163

***belchioris***, *Austrotinodes* 38

***belizensis***, *Chimarra* 374

***bella***, *Nectopsyche* 322

***bellini***, *Neotrichia* 247

***bellus***, *Polycentropus* 423

***benji***, *Alisotrichia* 195

***benwa***, *Rhyacopsyche* 295

***bertioga***, *Phylloicus* 30

**Betrichia** [Hydroptilidae] 202

*betteni*, *Chimarra* 398

***bettyae***, *Oxyethira* 283

***beutelspacheri***, *Polyplectropus* 434

***bevagota***, *Zumatrichia* 302

***biappendiculatus***, *Polycentropus* 423

***bibrachiata***, *Cernotina* 412

***bicarinatus***, *Phylloicus* 30

***bicaudata***, *Ochrotrichia* 263

***bicolor***, *Chimarra* 374

***bicolor***, *Macronema* 150

***bicolorata***, *Atopsyche* 101

***bicoloroides***, *Chimarra* 374

***bicornuta***, *Leucotrichia* 225

***bicornuta***, *Mastigoptila* 54

***bicornuta***, *Oxyethira* 283

***bicornuta***, *Protoptila* 67

***bicornuta***, *Smicridea* 164

***bicuspis***, *Calosopsyche* 126

***bidactyla***, *Smicridea* 164

***bidens***, *Australobiosis* 114

***bidens***, *Chimarra* 374

***bidens***, *Hydroptila* 216

***bidentata***, *Chimarra* 374

***bidentata***, *Culoptila* 49

***bidentata***, *Itauara* 51

***bidentata***, *Metrichia* 234

***bidentata***, *Oxyethira* 283

***bidentata***, *Smicridea* 164

***bidigitatus***, *Phylloicus* 31

***bifasciata***, *Smicridea* 164

***bifida***, *Canoptila* 43

***bifida***, *Neotrichia* 247

***bifida***, *Smicridea* 164

***bifida***, *Sortosa* 402

***bifida***, *Zumatrichia* 302

***bifidum***, *Macronema* 150

***bifidum***, *Parachorema* 122

***bifidus***, *Austrocentrus* 77

***bifidus***, *Brachysetodes* 317

***bifidus***, *Cyrnellus* 419

***bifurcata***, *Mortoniella* 57

***bifurcata***, *Neotrichia* 247

***bifurcata***, *Smicridea* 164

***bifurcatodes***, *Leptonema* 137

***bifurcatum***, *Leptonema* 137

***bifurcatus***, *Brachysetodes* 317

***bika***, *Neotrichia* 248

***bilanceolata***, *Alterosa* 365

***bilimekii***, *Xiphocentron* 456

***bilineata***, *Mortoniella* 57

***biloba***, *Marilia* 360

***bilobatum***, *Leptonema* 137

***bilobosa***, *Oecetis* 334

***biparta***, *Limnephilus* 351

***bipartita***, *Costatrichia* 211

***bipartita***, *Ochrotrichia* 263

***bipartiterga***, *Byrsopteryx* 206

***bipartitum***, *Microthremma* 78

***bisectilis***, *Chimarra* 374

***biserrulata***, *Smicridea* 164

***bisetosa***, *Alisotrichia* 195

***bispicata***, *Cernotina* 412

***bispinosa***, *Atopsyche* 101

***bispinosa***, *Betrichia* 203

*bispinosa*, *Lepidostoma* 309

***bispinosa***, *Sortosa* 402

***bispinus***, *Verger* 356

***biuncifera***, *Neotrichia* 248

***biungulata***, *Metrichia* 234

***bivittata***, *Smicridea* 164

***blahniki***, *Atopsyche* 101

***blahniki***, *Grumichella* 318

***blahniki***, *Helicopsyche* 81

***blahniki***, *Itauara* 51

***blahniki***, *Phylloicus* 31

***blahniki***, *Polyplectropus* 435

***blanca***, *Ochrotrichia* 263

***blancasi***, *Anomalocosmoecus* 348

***blancasi***, *Helicopsyche* 81

*blanchardi*, *Psilopsyche* 410

***blantoni***, *Helicopsyche* 81

***blantoni***, *Mejicanotrichia* 231

***blepharophera***, *Chimarra* 375

**Blepharopus** [Hydropsychidae] 125

***bocaina***, *Mortoniella* 57

***bocainae***, *Alterosa* 366

***bohio***, *Calosopsyche* 126

***bola***, *Metrichia* 234

***bolivari***, *Atopsyche* 101

***bolivari***, *Wormaldia* 405

***bolivianus***, *Polyplectropus* 435

***bolivica***, *Mortoniella* 57

***boliviense boliviense***, *Leptonema* 137

***boliviense plumosum***, *Leptonema* 137

***boliviensis***, *Austrotinodes* 38

***boliviensis***, *Contulma* 21

***boneti***, *Atopsyche* 101

*boneti*, *Chimarra* 370

***bonita***, *Atanatolica* 315

***bonita***, *Metrichia* 234

***bonus***, *Polycentropus* 424

***boquillas***, *Ochrotrichia* 264

***boraceia***, *Chimarra* 375

***boraceia***, *Flintiella* 213

***boraceia***, *Grumichella* 319

***boraceia***, *Leptonema* 138

***boraceia***, *Polycentropus* 424

***boraceiae***, *Alterosa* 366

***borealis***, *Helicopsyche* 81

***borinquensis***, *Xiphocentron* 456

***boruca***, *Protoptila* 67

***bostrychion***, *Metrichia* 234

***boteroi***, *Wormaldia* 405

***botka***, *Neotrichia* 248

***botonia***, *Neotrichia* 248

***botosaneanui***, *Atanatolica* 315

***botosaneanui***, *Cariboptila* 44

***botosaneanui***, *Leucotrichia* 225

*brachiata*, *Orthotrichia* 279

***brachycerca***, *Atopsyche* 101

***brachyrhachos***, *Mortoniella* 58

***brachyscolus***, *Polyplectropus* 435

**Brachysetodes** [Leptoceridae] 317

***brachyterga***, *Apatanodes* 99

*brachytergum*, *Australochorema* See *brachyterga*, *Apatanodes*

***braconoides***, *Chimarra* 375

***bractea***, *Ochrotrichia* 264

***bracteatus***, *Austrotinodes* 38

***bracui***, *Metrichia* 235

***brailovskyi***, *Hydroptila* 216

*branchiatus*, *Monocosmoecus* 353

***brasilensis***, *Polyplectropus* 435

***brasiliana***, *Atanatolica* 315

*brasiliana*, *Atopsyche* See ***longipennis***, *Atopsyche*

***brasiliana***, *Chimarra* 375

***brasiliana***, *Itauara* 51

***brasiliana***, *Leucotrichia* 225

***brasiliana***, *Notalina* 331

***brasiliana***, *Notidobiella* 449

***brasiliana***, *Smicridea* 165

*brasilianus*, *Rhyacophylax* See ***brasiliana***, *Smicridea*

*brasilianus*, *Smicridea* See ***weidneri***, *Smicridea*

***brasiliense***, *Macrostemum* 154

***brasiliensis***, *Antarctoecia* 349

***brasiliensis***, *Oxyethira* 283

***brasiliensis***, *Tolhuaca* 76

***braueri***, *Macrostemum* 155

***bravoae***, *Polyplectropus* 435

***bravoi***, *Macrostemum* 155

***brayi***, *Ochrotrichia* 264

***brazilia***, *Helicopsyche* 82

***braziliensis***, *Helicopsyche* 82

**Bredinia** [Hydroptilidae] 204

***brborichorum***, *Polyplectropus* 435

***bredini***, *Polyplectropus* 435

*Brethesella* [Leptoceridae] 321

***brethesi***, *Nectopsyche* 322

***brevicornuta***, *Mastigoptila* 54

***brevicornutus***, *Polycentropus* 424

***brevior***, *Phylloicus* 31

***brevipenis***, *Acostatrichia* 192

***brevis***, *Austrotinodes* 38

***brevispina***, *Neoatopsyche* 119

***brevispina***, *Neotrichia* 248

***brevitas***, *Metrichia* 235

***breviterga***, *Helicopsyche* 82

***breviuncata***, *Smicridea* 165

***bribri***, *Protoptila* 67

*bridarollia*, *Nectopsyche* 326

*bridarollina*, *Oecetis* 339

***brochophora***, *Leucotrichia* 226

***brodzinskyi***†, *Ochrotrichia* 264

***bromeliarum***, *Phylloicus* 31

***browni***, *Neotrichia* 248

***bruchi***, *Nectopsyche* 322

***bruchinus***, *Verger* 356

***brunneofascia***, *Nectopsyche* 323

***brunnescens***, *Smicridea* 165

*brunosa*, *Cailloma* 115

*brustia*, *Chimarra* 385

***buborektala***, *Acostatrichia* 192

***buchwaldi***, *Polyplectropus* 435

***buenoi***, *Culoptila* 49

***buenoi***, *Mortoniella* 58

***buenoi***, *Neotrichia* 248

***buenorum***, *Wormaldia* 405

***bulara***, *Smicridea* 165

***bulbosa***, *Metrichia* 235

***bulbosa***, *Rhyacopsyche* 296

***bulbosa***, *Smicridea* 165

***bullata***, *Neotrichia* 248

***bunkok***, *Leptonema* 138

***bunkotala***, *Rhyacopsyche* 296

***burica***, *Protoptila* 67

***burmeisteri***, *Chimarra* 375

***burmeisteri***, *Macronema* 150

***butleri***, *Chimarra* 375

**Byrsopteryx** [Hydroptilidae] 205

### C

***caab***, *Cariboptila* 45

***caaete***, *Polycentropus* 424

***caatinga***, *Ochrotrichia* 264

***cacaulandia***, *Alisotrichia* 195

***cacha***, *Cernotina* 413

***cachoeira***, *Polycentropus* 424

***cachonera***, *Ochrotrichia* 264

***cadeti***, *Cernotina* 413

***caenina***, *Xiphocentron* 457

***Caenocentron*** [Xiphocentronidae] 453

***cafetalera***, *Metrichia* 235

**Cailloma** [Hydrobiosidae] 115

***caimita***, *Ochrotrichia* 264

***caimitense***, *Xiphocentron* See ***cubanum caimitense***

***cainguas***, *Alisotrichia* 195

***caissara***, *Alterosa* 366

***cajas***, *Atopsyche* 102

***calahuaya***, *Atopsyche* 102

***calcarata***, *Ochrotrichia* 264

***calcariga***, *Orinocotrichia* 278

***calcea***, *Cernotina* 413

*calceatus*, *Monocosmoecus* 354

***calcigena***, *Cariboptila* 45

***caldas***, *Atanatolica* 315

***caldensis***, *Contulma* 22

***calderonae***, *Wormaldia* 405

***caldwelli***, *Smicridea* 165

*californica*, *Helicopsyche* 82

***caligata***, *Helicopsyche* 82

***caligata***, *Smicridea* 165

***caliginosa***, *Cernotina* 413

***caligula***, *Ochrotrichia* 264

***callosa***, *Atopsyche* 102

***calopa***, *Atopsyche* 102

***calopa***, *Smicridea* 165

***calori***, *Chimarra* 375

***calosa***, *Calosopsyche* 126

**Calosopsyche** [Hydropsychidae] 125

*calva*, *Chimarra* 374

***camargoi***, *Phylloicus* 31

***camella***, *Chimarra* 375

***cameria***, *Neotrichia* 248

***campana***, *Metrichia* 235

***campana***, *Oecetis* 334

***campana***, *Smicridea* 165

***campanilla***, *Ochrotrichia* 264

***campanum***, *Leptonema* 138

***campesina***, *Oxyethira* 283

***camposae***, *Chimarra* 376

*Campsiophora* [Glossosomatidae] 44

***camura***, *Chimarra* 376

***camuriensis***, *Helicopsyche* 83

***cana***, *Nectopsyche* 323

***cana***, *Protoptila* 67

***canastra***, *Polyplectropus* 435

*candida* Hagen 1861, *Setodes* (now *Nectopsyche*) [extralimital] 327

*candida* Navás 1923, *Leptocella* (now *Nectopsyche*) 327

***candida***, *Nectopsyche* 323

***canicula***, *Ochrotrichia* 265

***canixa***, *Neotrichia* 248

***canoaba***, *Chimarra* 376

***canoabo***, *Austrotinodes* 38

**Canoptila** [Glossosomatidae] 43

*canosa*, *Chimarra* 372

*canossa*, *Chimarra* See *canosa*, *Chimarra*

***caparaonensis***, *Alterosa* 366

*capillata*, *Clavichorema* See ***capillatum***, *Clavichorema*

***capillatum***, *Clavichorema* 116

***capillatus***, *Verger* 356

***capixaba***, *Alterosa* 366

***caquetia***, *Atopsyche* 102

***caraca***, *Metrichia* 235

***carajas***, *Flintiella* 214

***caramba***, *Ochrotrichia* 265

***carara***, *Costatrichia* 211

***carara***, *Hydroptila* 216

***carbetina***, *Metrichia* 235

***carbonelli***, *Cernotina* 413

***cardela***, *Protoptila* 68

***cariba***, *Smicridea* 166

***caribea caribea***, *Chimarra* 376

*caribea surinamensis*, *Chimarra* 374

***caribea tobaga***, *Chimarra* 376

**Cariboptila** [Glossosomatidae] 44

***carinifera***, *Calosopsyche* 127

***carinula***, *Mortoniella* 58

***carioca***, *Byrsopteryx* 206

***carioca***, *Polycentropus* 424

***carlibanezae***, *Oecetis* 334

*carmentis*, *Machairocentron* 455

***carolae***, *Anchitrichia* 200

***carolae***, *Chimarra* 376

***carolae***, *Polycentropus* 424

***carolae***, *Polyplectropus* 436

***cartiensis***, *Smicridea* 166

***cascada***, *Chimarra* 376

***cascada***, *Culoptila* 49

***casicus***, *Polycentropus* 424

*castilleja*, *Oecetis* 335

***castroalvesi***, *Alterosa* 366

***catamarcensis***, *Hydroptila* 216

***cataracta***, *Contulma* 22

***catarina***, *Lepidostoma* 308

***catarina***, *Ochrotrichia* 265

***catarinae***, *Alterosa* 366

*catarinensis*, *Chimarra* 396

***catarinensis***, *Mortoniella* 58

***catherinae***, *Atopsyche* 102

***catherinae***, *Smicridea* 166

***cauca***, *Amphoropsyche* 313

***cauca***, *Chimarra* 376

***caudatum***, *Microthremma* 78

***caudicula***, *Mortoniella* 58

***caudifera***, *Zumatrichia* 302

***cautinensis***, *Nothotrichia* 260

***cavitectum***, *Ochrotrichia* 265

***caxima***, *Neotrichia* 248

***cayada***, *Neotrichia* 248

***caymmii***, *Alterosa* 366

***cebollati***, *Angrisanoia* 201

***ceciliae***, *Polycentropus* 424

***ceer***, *Metrichia* 235

***cekalovici***, *Austrotinodes* 39

**Celaenotrichia** [Hydroptilidae] 208

*cellare*, *Leptonema* 139

***centralis***, *Chimarra* 377

***centralus***, *Phylloicus* 30

***centrispina***, *Chimarra* 377

***centrocubana***, *Helicopsyche* 83

**Centromacronema** [Hydropsychidae] 128

**Cerasmatrichia** [Hydroptilidae] 208

*Ceratopsyche* [Hydropsychidae] 133

**Ceratotrichia** [Hydroptilidae] 209

***cerna***, *Acostatrichia* 192

**Cernotina** [Polycentropodidae] 411

**chacayana**, *Notidobiella* 450

***chaconi***, *Byrsopteryx* 206

***chalybeoides***, *Macronema* 151

*chalybeum*, *Macronema* See ***chalybeoides***, *Macronema*

***chalybeus***, *Phylloicus* 31

***championi***, *Leptonema* 138

***chana***, *Neotrichia* 249

**Charadropsyche** [Tasimiidae] 452

***charlesi***, *Polyplectropus* 436

***charlotta***, *Itauara* 52

***charrua***, *Neotrichia* 249

***chaulioda***†, *Ochrotrichia* 265

***cheesmanae***, *Leptonema* 138

***chela***, *Chimarra* 377

***cheliceratus***, *Polycentropus* 424

***chelifera***, *Cernotina* 413

**Cheumatopsyche** [Hydropsychidae] 131

***chiapa***, *Ochrotrichia* 265

***chiapaneca***, *Cernotina* 413

***chiapasi***†, *Xiphocentron* 457

***chiapense***, *Leptonema* 138

***chiapensis***, *Diplectrona* 133

*Chiasmoda* [Hydropsychidae] 188

***chicana***, *Mortoniella* 58

***chichotla***, *Rhyacopsyche* 296

***chicoana***, *Smicridea* 166

***chihuahua***, *Alisotrichia* 195

***chihuahua***, *Austrotinodes* 39

***chihuahua***, *Neotrichia* 249

***chila***, *Leptonema* 138

***chilense***, *Androchorema* 98

***chilense***, *Nolganema* 121

***chilensis***, *Helicopsyche* 83

***chilensis***, *Neoatopsyche* 119

***chilensis***, *Neotrichia* 249

***chilensis***, *Polycentropus* 424

***chilensis***, *Pomphochorema* 122

*chilensis*, *Smicridea* 162

***chilensis***, *Sortosa* 402

***chilensis***, *Triplectides* 345

*chilensis*, *Verger* 356

***chillana***, *Psilopsyche* 410

**Chilocentropus** [Ecnomidae] 42

*chiloeana*, *Clavichorema* See ***chiloeanum***, *Clavichorema*

***chiloeanum***, *Clavichorema* 116

**Chiloecia** [Sericostomatidae] 447

***chimalapa***, *Chimarra* 377

**Chimarra** [Philopotamidae] 369

*Chimarrha* [Philopotamidae] 369

**Chimarrhodella** [Philopotamidae] 400

*Chimarrhodes* [Philopotamidae] 369

***Chimarrita*** [Philopotamidae] 369

***chimpuocllo***, *Atopsyche* 102

***chimuru***, *Atopsyche* 103

***chinchacamac***, *Atopsyche* 103

***chipiriri***, *Oecetis* 334

***chiquitica***, *Alisotrichia* 195

***chirihuana***, *Atopsyche* 103

***chirimachaya***, *Atopsyche* 103

***chiriquensis***, *Helicopsyche* 83

***chiriquiense***, *Lepidostoma* 308

*chiriquiensis*, *Lepidostoma* 308

***chiriquiensis***, *Leucotrichia* 226

***chiriquiensis***, *Zumatrichia* 302

***chirripo***, *Triaenodes* 342

***chitaria***, *Protoptila* 68

*chloraemus*, *Macronema* 150

***choco***, *Amphoropsyche* 313

***choco***, *Atanatolica* 316

***chocoense***, *Leptonema* 138

***chocoensis***, *Chimarra* 377

***chocoensis***, *Helicopsyche* 83

***cholta***, *Smicridea* 166

***choluteca***, *Protoptila* 68

***chontala***, *Protoptila* 68

***choronica***, *Atopsyche* 103

***chorra***, *Alisotrichia* 196

***chrismark***, *Wormaldia* 405

***chrysosoma***, *Chimarra* 377

*Chrysostoma* [Sericostomatidae] 450

*chysopus*, *Barypenthus* 359

***cieneguilla***, *Ochrotrichia* 265

***cimarrona***, *Alisotrichia* 196

***cinctum***, *Leptonema* 138

***cinerascens***, *Oecetis* 334

***cinerea***, *Marilia* 360

***cingulata***, *Cernotina* 413

***cipo***, *Notalina* 331

***cipo***, *Triplectides* 345

***cipoensis***, *Chimarra* 377

***cipoensis***, *Helicopsyche* 83

***cipoensis***, *Polycentropus* 425

***cira***, *Atopsyche* 103

***circaverna***, *Oxyethira* 283

***circinata***, *Alisotrichia* 196

***circinata***, *Smicridea* 166

***circulata***, *Helicopsyche* 83

***circulatrix***, *Metrichia* 235

***circuliforme***, *Metrichia* 235

***cirrifera***, *Chimarra* 377

***cirrifera***, *Oxyethira* 284

***citra***, *Ochrotrichia* 265

***clara***, *Helicopsyche* 83

***clarkei***, *Atopsyche* 103

***clarus***, *Polycentropus* 425

*claudens*, *Barypenthus* 359

***clauseni***, *Polyplectropus* 436

***clauseni***, *Triaenodes* 342

**Clavichorema** [Hydrobiosidae] 116

***clavicornia***, *Oecetis* 334

***claviloba***, *Chimarra* 377

**Clistoronia** [Limnephilidae] 349

***clorito***, *Leptonema* 138

**Cnodocentron** [Xiphocentronidae] 453

***cochleara***, *Helicopsyche* 83

***Cochliopsyche*** [Helicopsychidae] 79

***coheni***, *Chimarra* 377

***coheni***, *Leptonema* 139

***colei***, *Rhyacopsyche* 296

***collaris***, *Cyrnellus* 419

***collegarum***, *Mortoniella* 58

***colmillo***, *Chimarra* 378

***colmillosa***, *Neotrichia* 249

***colombiana***, *Rhyacopsyche* 296

***colombianus***, *Polyplectropus* 436

***colombicus***, *Triplectides* 345

***colombiensis***, *Contulma* 22

***colombiensis***, *Helicopsyche* 83

***colombiensis***, *Neotrichia* 249

***colombiensis***, *Oxyethira* 284

*colombiensis*, *Polycentropus* 428

***colombiensis***, *Protoptila* 68

***colubrinosa***, *Rhyacopsyche* 296

***columbiana***, *Banyallarga* 26

***columbiana***, *Smicridea* 166

***columbianum***, *Leptonema* 139

*columbianus*, *Rhyacophylax* See ***columbiana***, *Smicridea*

***columbicus***, *Triaenodes* 342

***comma***, *Smicridea* 166

***comosa***, *Helicopsyche* 84

***compacta***, *Ochrotrichia* 265

***completa***, *Smicridea* 167

***complexum***, *Leptonema* 139

*complicatissima*, *Clavichorema* See ***complicatissimum***, *Clavichorema*

***complicatissima***, *Smicridea* 167

***complicatissimum***, *Clavichorema* 116

***complicornuta***, *Mastigoptila* 54

***compostella***, *Smicridea* 167

***compressa***, *Cernotina* 413

***comptum***, *Plectromacronema* 158

***concha***, *Ochrotrichia* 265

***concolor***, *Barypenthus* 359

*Condocerus* [Leptoceridae] 320

***condylifera***, *Protoptila* 68

***conformalis***, *Ochrotrichia* 266

***confusa***, *Ochrotrichia* 266

***conica***, *Chimarra* 378

***conjuncta***, *Smicridea* 167

***connata***, *Oecetis* 334

***connatus***, *Polycentropus* 425

***connori***, *Neotrichia* 249

***consimilis***, *Chimarra* 378

***constricta***, *Hydroptila* 216

***constricta***, *Ochrotrichia* 266

***constricta***, *Oecetis* 334

***continentalis***, *Calosopsyche* 127

***continentalis***, *Metrichia* 236

***contrerasi***, *Neotrichia* 249

***contrerasi***, *Ochrotrichia* 266

***contrerasi***, *Scelobotrichia* 298

***contrerasi***, *Wormaldia* 405

***contubernalis***, *Austrotinodes* 39

**Contulma** [Anomalopsychidae] 21

***conventica***, *Atopsyche* 103

***copayapu***, *Atopsyche* 103

***copina***, *Oxyethira* 284

***cora***, *Protoptila* 68

***cordatus***, *Phylloicus* 31

*Cordillopsyche* [Polycentropodidae] 433

***cordoba***, *Atopsyche* 103

***corneolus***, *Ochrotrichia* 266

***cornicula***, *Alisotrichia* 196

***corniculatum***, *Parasericostoma* 450

***corniculatus***, *Polyplectropus* 436

***corniculans***, *Neotrichia* 249

***cornuta***, *Chimarra* 378

***cornuta***, *Smicridea* 167

***cornuta***, *Wormaldia* 405

***coronata***, *Smicridea* 167

***coronatum***, *Leptonema* 139

***corosa***, *Zumatrichia* 302

***corralita***, *Smicridea* 167

***coscaroni***, *Hydroptila* 216

***costalis***, *Polyplectropus* 436

***costaricensis***, *Bredinia* 204

***costaricensis***, *Chimarra* 378

***costaricensis***, *Chimarrhodella* 400

***costaricensis***, *Contulma* 22

***costaricensis***, *Culoptila* 49

***costaricensis***, *Oxyethira* 284

***costaricensis***, *Polycentropus* 425

***costaricensis***, *Tizatetrichia* 299

**Costatrichia** [Hydroptilidae] 210

***costiferum***, *Amphichorema* 98

***cotopaxi***, *Atanatolica* 316

***cotopaxi***, *Helicopsyche* 84

***cranifer***, *Contulma* 22

*crassicosta*, *Stenochorema* See ***crassicostum***, *Stenochorema*

***crassicostum***, *Stenochorema* 124

***crassifimbriatum***, *Microthremma* 78

*crassum*, *Leptonema* of Mosely 1933 *See* ***divaricatum***, *Leptonema*

***crassum***, *Leptonema* 139

***crea***, *Marilia* 361

***creagra***, *Chimarra* 378

***crena***, *Chimarra* 378

***crenatus***, *Phylloicus* 31

***crenula***, *Metrichia* 236

***crescentis***, *Mortoniella* 58

***cressae***, *Austrotinodes* 39

***cressae***, *Chimarra* 378

***cressae***, *Costatrichia* 211

***cressae***, *Grumichella* 319

***cressae***, *Hydroptila* 216

***cressae***, *Leptonema* 140

***cressae***, *Phylloicus* 32

***cressae***, *Polycentropus* 425

***cressae***, *Polyplectropus* 436

***criollo***, *Polycentropus* 425

***cristata***, *Orthotrichia* 279

***cristata***, *Protoptila* 68

***cristatum***, *Parasericostoma* 450

***cristula***, *Protoptila* 68

***crucecita***, *Ochrotrichia* 266

***cruces***, *Ochrotrichia* 266

***csiga***, *Ochrotrichia* 266

***ctenacantha***, *Protoptila* 68

***ctenifer***, *Limnephilus* 351

***ctilopsis***, *Protoptila* 68

***cubana***, *Atopsyche* 104

***cubana***, *Calosopsyche* 127

***cubana***, *Cariboptila* 45

***cubana***, *Helicopsyche* 84

***cubana***, *Hydroptila* 216

***cubana***, *Nectopsyche* 323

***cubana***, *Smicridea* 167

*Cubanoptila* [Glossosomatidae] 44

***cubanorum***, *Chimarra* 378

***cubanum caimitense***, *Xiphocentron* 457

***cubanum cubanum***, *Xiphocentron* 457

***cubanum orientale***, *Xiphocentron* 457

***cubanus***, *Austrotinodes* 39

***cubanus***, *Phylloicus* 32

***cuchilla***, *Byrsopteryx* 206

***cuenca***, *Metrichia* 236

***cuernita***, *Alisotrichia* 196

***cuernuda***, *Neotrichia* 249

***cuernuda***, *Oxyethira* 284

***culebra***, *Oxyethira* 284

**Culoptila** [Glossosomatidae] 48

***cultellata***, *Chimarra* 379

***cuna***, *Smicridea* 168

***cuniapiru***, *Metrichia* 236

*cupreosquamosa*, *Nectopsyche* 325

*cupreum*, *Centromacronema* 129

***cupulifera***, *Tolhuaca* 76

***curfmani***, *Chimarra* 379

***Curgia*** [Philopotamidae] 369

***curiosa***, *Protoptila* 68

***curta***, *Metrichia* 236

***curtior***, *Verger* 356

***curvata***, *Hydroptila* 217

***curvata***, *Ochrotrichia* 266

***curvicornuta***, *Mastigoptila* 54

***curvipalpia***, *Helicopsyche* 84

***curvipenis***, *Chimarra* 379

***curvipenis***, *Smicridea* 168

***curvispinum***, *Isochorema* 118

***cuspidata***, *Metrichia* 236

***cuspidatus***, *Ochrotrichia* 267

***cuspidatus***, *Polycentropus* 425

***cuyensis***, *Xiphocentron* 457

***cuyotenango***, *Triaenodes* 342

***cuzcoensis***, *Polyplectropus* 436

***cyanolenos***, *Alisotrichia* 196

***cygnea***, *Cernotina* 413

*Cyllene* [Hydroptilidae] 245

**Cyrnellus** [Polycentropodidae] 418

***cystophora***, *Cernotina* 413

### D

***dachiardiorum***, *Wormaldia* 405

***dactylonedys***, *Oxyethira* 284

***dactylophora***, *Ochrotrichia* 267

***Dactylotrichia*** , [Hydroptilidae] 280

***dafila***, *Lepidostoma* 308

***dalmeria***, *Oxyethira* 284

***dampfi***, *Atopsyche* 104

***dampfi***, *Helicopsyche* 84

***dampfi***, *Hydropsyche* 134

***dampfi***, *Smicridea* 168

***dampfi***, *Wormaldia* 405

***Dampfitrichia*** , [Hydroptilidae] 280

***danielae***, *Oecetis* 334

***danieli***, *Cernotina* 414

***darda***, *Acostatrichia* 192

***darlingtoni***, *Calosopsyche* 127

***darlingtoni***, *Chimarra* 379

***davenporti***, *Bredinia* 204

***davidsoni***, *Atopsyche* 104

***davisi***, *Leptonema* 140

***davisorum***, *Atopsyche* 104

***davisorum***, *Pseudostenopsyche* 452

***davisorum***, *Streptopsyche* 188

***dearmasi***, *Calosopsyche* 127

***decimlobata***, *Chimarra* 379

***declinata***, *Cernotina* 414

***decora***, *Metrichia* 236

***decora***, *Smicridea* 168

*decorata*, *Nectopsyche* 324

***decumbens***, *Cernotina* 414

***delaca***, *Protoptila* 69

***delgada***, *Ochrotrichia* 267

***delgadeza***, *Neotrichia* 249

***delicatus***, *Triaenodes* 343

***delongi***, *Lepidostoma* 308

***delrio***, *Hydropsyche* 134

***deltoides***, *Polyplectropus* 436

***demma***, *Antillopsyche* 444

***denaia***†, *Ochrotrichia* 267

***denningi***, *Culoptila* 49

***denningi***, *Lepidostoma* 308

***dentatum***, *Centromacronema* 129

***denticulata***, *Leucotrichia* 226

***denticulata***, *Mortoniella* 58

***denticulus***, *Polyplectropus* 437

***dentifera***, *Smicridea* 168

***dentisserrata***, *Smicridea* 168

***dentoides***, *Polycentropus* 425

***dentosa***, *Chimarra* 379

***denza***, *Hydroptila* 217

***depressa***, *Cernotina* 414

***dereka***, *Zumatrichia* 302

***desadorna***, *Oxyethira* 284

***desiderata***, *Neoatriplectides* 25

***desirae***, *Chimarra* 379

***diablita***, *Protoptila* 69

***diacantha***, *Rhyacopsyche* 296

***diakis***, *Chimarra* 379

***diamantina***, *Atopsyche* 104

***diamphidia***, *Zumatrichia* 302

***dianae***, *Polycentropus* 425

***dianeae***, *Leucotrichia* 226

***diannae***, *Chimarra* 379

***diaphanus***, *Blepharopus* 125

***diaphora***, *Chimarra* 380

*Diaulus* [Hydroptilidae] 212

**Dicaminus** [Hydroptilidae] 212

*Dicentropus* [Sericostomatidae] 448

***dictynnum***, *Neochorema* 120

***didii***, *Neotrichia* 249

***didyma***, *Chimarra* 380

***dientera***, *Neotrichia* 250

***dietzi***, *Ragatrichia* 294

***difusa***, *Metrichia* 236

***digitata***, *Acostatrichia* 192

***digitata***, *Neotrichia* 250

***digitus***†, *Antillopsyche* 444

***digitus***, *Polycentropus* 425

***digramma***, *Macrostemum* 155

***dikeros***, *Neotrichia* 250

***dikrosa***, *Rhyacopsyche* 296

***diminuta***, *Nectopsyche* 323

***dinamica***, *Leucotrichia* 226

***dinocephalum***, *Parasericostoma* 451

***diosa***, *Metrichia* 236

**Diplectrona** [Hydropsychidae] 132

***discaelata***, *Oxyethira* 284

***discalis***, *Smicridea* 168

*discolor*, *Hudsonema* 320

***discolor***, *Limnephilus* 351

***disjuncta***, *Helicopsyche* 84

***disjuncta***, *Oecetis* Smith and Lehmkuhl *nec* Banks 340

***dispar***, *Smicridea* 168

***disparilis***, *Chilocentropus* 42

***disparilis***, *Metrichia* 236

*dissimile*, *Leptonema* 149

*distans*, *Phylloicus* 29

***distermina***, *Chimarra* 380

***disticha***, *Protoptila* 69

***ditalea***, *Hydroptila* 217

*ditata*, *Nectopsyche* 324

***dithyra***, *Smicridea* 169

***divaricatum***, *Leptonema* 140

*divisa*, *Smicridea* 170

***djalmasantosi***, *Neotrichia* 250

***doehleri***†, *Ochrotrichia* 267

***doesburgi***, *Oecetis* 335

***dolabrifera***, *Chimarra* 380

***Dolochorema*** [Hydrobiosidae] 99

***dolonis***, *Mortoniella* 58

*Dolotrichia*, [Hydroptilidae] 245

***dombora***, *Smicridea* 169

***domingensis***, *Calosopsyche* 128

***domingensis***, *Polycentropus* 425

***dominguezi***, *Oecetis* 335

***dominicana***, *Atanatolica* 316

***dominicana***, *Chimarra* 380

***dominicana***, *Helicopsyche* 84

***dominicana***, *Hydroptila* 217

***dominicensis***, *Bredinia* 204

***dominicensis***, *Cerasmatrichia* 209

***dominicensis***, *Protoptila* 69

*dommeli*†, *Chimarra* 380

***donamariae***, *Chimarra* 380

***doronca***, *Polycentropus* 426

***dorsalis***, *Nectopsyche* 323

*dorsalis*, *Orthotrichia* 279

***dorsata***, *Wormaldia* 405

***dorsocurvata***, *Helicopsyche* 85

***doublesi***, *Austrotinodes* 39

***drepanigerum***, *Parasericostoma* 451

***duarte***, *Smicridea* 169

***dubitans***, *Neotrichia* 250

***dubitans***, *Protoptila* 69

***dubitatus***, *Polyplectropus* 437

***duckworthi***, *Chimarra* 380

***dudosa***, *Chimarra* 381

***dulce***, *Ochrotrichia* 267

***dumasi***, *Phylloicus* 32

***duodecimpunctata***, *Achoropsyche* 312

***duplex***, *Sortosa* 402

*duplicicornuta*, *Mastigoptila* 54

***duplicispina***, *Rhyacopsyche* 296

***duplifurcata***, *Anchitrichia* 200

***dupliplex***, *Sortosa* 402

***durior***, *Neotrichia* 250

***dyeri***, *Leptonema* 140

### E

***eberhardi***, *Wormaldia* 406

***echinata***, *Banyallarga* 26

***echinata***, *Contulma* 22

***echinata***, *Zumatrichia* 302

***echinatum***, *Machairocentron* 455

***ecliptica***, *Synoestropsis* 189

*Ecnomodellina* [Polycentropodidae] 433

*Ecnomodes* [Polycentropodidae] 433

***ecornuta***, *Mastigoptila* 54

***ecotura***, *Cernotina* 414

***ectopium***, *Lepidostoma* 308

***ecuadorensis***, *Contulma* 22

***ecuadorensis***, *Notidobiella* 450

***ecuadoriensis***, *Polyplectropus* 437

***ecuatoriana***, *Ochrotrichia* 267

***eduardoi***, *Mortoniella* 59

***edwardi***, *Sortosa* 402

***edwardsi***, *Celaenotrichia* 208

***egleri***, *Triplectides* 345

***egsera***, *Smicridea* 169

***ekisi***, *Leptonema* 140

***elachista***, *Calosopsyche* 128

***elae***, *Mastigoptila* 54

***elata***, *Oecetis* 335

*elatus*, *Oecetis* See ***elata***, *Oecetis*

***electra***†, *Helicopsyche* 85

***elegans***, *Phylloicus* 32

***elektoros***, *Phylloicus* 32

***eleutheria***, *Marilia* 361

***elia***, *Chimarra* 381

***elisae***, *Smicridea* 169

***elongata***, *Marilia* 361

***elongata***, *Mortoniella* 59

***elongata***, *Neotrichia* 250

***elongata***, *Sortosa* 403

***elongatoides***, *Sortosa* 403

***elongatus***, *Alloecentrellodes* 76

***elongatus***, *Nyctiophylax* 421

***elongatus***, *Polyplectropus* 437

***eltera***, *Metrichia* 236

***elvesta***, *Acostatrichia* 192

***emarginata***, *Bredinia* 204

***embia***, *Chimarra* 381

***emilia***, *Itauara* 52

***emima***, *Chimarra* 381

***encera***, *Polycentropus* 426

***enchrysa***, *Mortoniella* 59

***encrypta***, *Cernotina* 414

***endonima***, *Wormaldia* 406

***enigmatica***, *Metrichia* 236

***enikolah***, *Leptonema* 140

***enonis***, *Cheumatopsyche* 131

***ensifera***, *Chimarra* 381

***ensifera***, *Protoptila* 69

**Eosericostoma** [Helicophidae] 77

***epara***, *Peltopsyche* 293

***ephippifer***, *Smicridea* 169

***ephippium***, *Phylloicus* 32

***erato***, *Xiphocentron* 457

***erda***, *Smicridea* 169

***erecta***, *Smicridea* 169

***erectiloba***, *Chimarra* 382

*Eremopsyche* [Lepidostomatidae] 307

***erichsoni***, *Macrostemum* 155

***erigia***, *Atopsyche* 104

*erinaceus*, *Cailloma* 115

***eroga***, *Neotrichia* 250

***erotica***, *Protoptila* 69

***escoba***, *Ochrotrichia* 267

***escova***, *Alterosa* 366

***esmalda***, *Neotrichia* 250

***espala***, *Atopsyche* 104

***esparta***, *Byrsopteryx* 206

***espera***, *Metrichia* 237

***esperonis***, *Wormaldia* 406

***espinada***, *Oxyethira* 284

***espinhosa***, *Byrsopteryx* 207

***espinosa***, *Bredinia* 205

***espinosa***, *Chimarra* 382

***estaquillosa***, *Mejicanotrichia* 231

***esterum***, *Macronema* 151

***eugnathum***, *Leptonema* 140

***euphrosyne***, *Alisotrichia* 196

***euryale***, *Xiphocentron* 457

***euryphlebia***, *Synoestropsis* 189

**Eutonella** [Hydroptilidae] 461

***evandrus***, *Xiphocentron* 457

***excisa***, *Metrichia* 237

***excisa***, *Oecetis* 335

***excisum***, *Centromacronema* 129

***exclamationis***, *Metrichia* 237

***exicoma***, *Neotrichia* 250

*exilis*, *Nectopsyche* 325

***exilis***, *Polyplectropus* 437

*Exitrichia*, [Hydroptilidae] 245

***exophthalma***, *Nectopsyche* 324

***exophthalmum***, *Macronema* 151

***explanata***, *Atopsyche* 105

***exsertus***, *Polycentropus* 426

***extensa***, *Helicopsyche* 85

*extensum*, *Centromacronema* 129

***extensum***, *Microchorema* 119

***extensus***, *Brachysetodes* 317

*externum*, *Leptonema* 139

***extragma***, *Metrichia* 237

***extraordinaria***, *Leucotrichia* 226

*extrema*, *Verger* 356

***eyipantla***, *Ochrotrichia* 267

### F

***facile***, *Centromacronema* 130

***fairchildi***, *Ceratotrichia* 210

***fairchildi***, *Leucotrichia* 226

***falcata***, *Alterosa* 366

***falcata***, *Cernotina* 414

***falcicula***, *Mortoniella* 59

***falcifera***, *Neotrichia* 251

***falciforme***, *Machairocentron* 455

***falcigona***, *Helicopsyche* 85

***falicia***, *Oecetis* 335

*falina*, *Atopsyche* 112

***fallaciosa***, *Cernotina* 414

***falsa***, *Leucotrichia* 227

***farkoska***, *Neotrichia* 251

***farofa***, *Metrichia* 237

***farri***, *Phylloicus* 32

***fasciatella***, *Smicridea* 169

***fasiculata***, *Marilia* 361

***fasthi***, *Polycentropus* 426

***favus***, *Metrichia* 237

*fazi*, *Hudsonema* 320

***faziana***, *Pangullia* 306

***felfela***, *Hydroptila* 217

***felfele***, *Centromacronema* 130

***felfesa***, *Zumatrichia* 302

***felgorba***, *Ceratotrichia* 210

***felipe***, *Ochrotrichia* 268

***felker***, *Macrostemum* 155

***felkurta***, *Neotrichia* 251

***felsa***, *Smicridea* 170

*fenestrata*, *Nectopsyche* 328

***fenestratus***, *Phylloicus* 32

***feolai***, *Neotrichia* 251

***ferelunatum***, *Leptonema* 140

***fernandezi***, *Chimarra* 382

***ferni***, *Ithytrichia* 223

***Feropsyche*** [Helicopsychidae] 80

***ferrugineum*** (Navás) 1924, *Centromacronema* 130

*ferrugineum* Bueno 1986, *Centromacronema* 130

***ferrugineus***, *Barypenthus* 359

*festiva*, *Nectopsyche* 324

***fesuka***, *Zumatrichia* 303

***fibra***, *Oecetis* 335

***figueroai***, *Smicridea* 170

***filicata***, *Smicridea* 170

***filifera***, *Neotrichia* 251

***filiforma***, *Ochrotrichia* 268

***filiformis***, *Cernotina* 414

*filiformiss*, *Cernotino* See ***filiformis***, *Cernotina*

***filosa***, *Zumatrichia* 303

***fimbriata***, *Acostatrichia* 192

***fimbriata***, *Alterosa* 367

***fimbriata***, *Chimarra* 382

***fimbriata***, *Protoptila* 69

***fioka***, *Ochrotrichia* 268

***fistulata***, *Helicopsyche* 85

***fittkaui***, *Chimarra* 382

*flagellata*, *Leptonema* 144

***flagellata***, *Ochrotrichia* 268

***flaminii***, *Hudsonema* 320

***flaveola***, *Grumichella* 319

*flaveolata*, *Oecetis* 337

***flavicoma***, *Ceratotrichia* 210

***flavicornis***, *Polyplectropus* 437

*flavida*, *Oecetis* 337

*flavipes*, *Grumicha* 448

***flavipunctata***, *Sortosa* 403

***flavofasciata***, *Nectopsyche* 324

***flavofuscum***, *Iguazu* 117

***flexispina***, *Protoptila* 69

***flexuosa***, *Cernotina* 414

***flexuosa***, *Marilia* 361

***flexura***, *Ochrotrichia* 268

***flinti***, *Alterosa* 367

***flinti***, *Amphoropsyche* 313

***flinti***, *Atanatolica* 316

***flinti***, *Atopsyche* 105

***flinti***, *Chimarra* 382

***flinti***, *Chimarrhodella* 401

***flinti***, *Costatrichia* 211

***flinti***, *Helicopsyche* 85

***flinti***, *Hydroptila* 217

***flinti***, *Itauara* 52

***flinti***, *Marilia* 362

***flinti***, *Mortoniella* 59

***flinti***, *Phylloicus* 33

***flinti***, *Rhyacopsyche* 296

***flinti***, *Smicridea* 170

***flinti***, *Wormaldia* 406

***flintiana***, *Alisotrichia* 196

***flintiana***, *Ochrotrichia* 268

**Flintiella** [Hydroptilidae] 213

***flintorum***, *Isochorema* 118

***flintorum***, *Leptonema* 140

***flintorum***, *Polyplectropus* 437

***flintorum***, *Triaenodes* 343

***flintorum***, *Triplectides* 345

***florecita***, *Metrichia* 237

***florecita***, *Smicridea* 170

***florestani***, *Hydroptila* 217

***florica***, *Mortoniella* 59

***florida***, *Oxyethira* 285

*floridana*, *Oecetis* 334

***flowersi***, *Neotrichia* 251

***fluminensis***, *Acostatrichia* 192

***fluminensis***, *Alterosa* 367

***fluminensis***, *Contulma* 22

***fluminensis***, *Polycentropus* 426

***foersteri***, *Mortoniella* 59

***fogaka***, *Neotrichia* 251

***fogasa***, *Smicridea* 170

***fontismoreaui***, *Metrichia* 237

***forceps***, *Metrichia* 237

***forcipata***, *Chimarra* 382

***forcipata***, *Smicridea* 170

***forcipatus***, *Brachysetodes* 317

***forficulum***, *Leptonema* 141

***formosinha***, *Metrichia* 238

***forrota***, *Leucotrichia* 227

***fortispinus***, *Polycentropus* 426

***fortuna***, *Banyallarga* 26

***fortunata***, *Austrotinodes* 39

***fortunum***, *Leptonema* 141

***fortunus***, *Polycentropus* 426

***fossilis***†, *Palaehydropsyche* 158

***fragile***, *Macronema* 151

*fragilis*, *Macronema* See ***fragile***, *Macronema*

***franciscana***, *Smicridea* 170

***francovilla***, *Wormaldia* 406

*fraterna*, *Chimarra* 393

*fraterna*, *Macronema* See ***fraternum***, *Macronema*

***fraternum***, *Macronema* 151

***fraternus***, *Cyrnellus* 419

***fredycarol***, *Wormaldia* 406

***frequens***, *Smicridea* 171

***freytagi***, *Austrotinodes* 39

***fridae***, *Helicopsyche* 85

***frijole***, *Limnephilus* 351

***froehlichi***, *Chimarra* 382

***froehlichi***, *Mortoniella* 59

***froehlichi***, *Neoatriplectides* 25

***froehlichi***, *Notalina* 331

***froehlichi***, *Oecetis* 335

***froehlichi***, *Polycentropus* 426

***froehlichi***, *Smicridea* 171

***frontale***, *Lepidostoma* 308

***frontalis***, *Ascotrichia* 202

***frontalis***, *Triaenodes* 343

***fugga***, *Metrichia* 238

***fulika***, *Cerasmatrichia* 209

***fulminea***, *Leucotrichia* 227

***fulva***, *Nectopsyche* 324

*fulvocapilla*, *Nectopsyche* 326

***fulvum***, *Macronema* 151

*fumosa*, *Oecetis* 334

***fundorai***, *Alisotrichia* 196

***furcata***, *Oecetis* 336

***furcata***, *Synoestropsis* 189

*furcatum*, *Leptonema* 144

***furciligerum***, *Leptonema* 141

***furesa***, *Smicridea* 171

***furthi***, *Marilia* 362

***furtiva***, *Hydroptila* 217

*fusca*, *Marilia* 361

*fusca*, *Pangullia* 306

*fusca*, *Sortosa* 402

***fuscatus***, *Polyplectropus* 437

***fuscifurca***, *Smicridea* 171

***fuscomaculata***, *Nectopsyche* 324

***fuscomarginatus***, *Austrotinodes* 40

***fuscovittatus***, *Verger* 356

***fuscum***, *Xiphocentron* 458

### G

***gabriel***, *Alisotrichia* 196

***gadzux***, *Leptonema* 141

***gaesum***, *Polyplectropus* 437

***galalcha***, *Chimarra* See ***spinulifera galalcha***

***galeata***, *Chimarrhodella* 401

***galesus***, *Cnodocentron* 454

***galharada***, *Atopsyche* 105

***galharada***, *Polycentropus* 426

***gallardoi***, *Wormaldia* 406

***galtena***, *Zumatrichia* 303

***garciai***, *Chimarra* 382

***garfio***, *Polycentropus* 426

***garifosa***, *Oxyethira* 285

***garra***, *Neotrichia* 251

***garrinchai***, *Neotrichia* 251

***garuhape***, *Ragatrichia* 294

***gelita***, *Cheumatopsyche* 132

***gemina***, *Smicridea* 171

***geminata***, *Metrichia* 238

***geminata***, *Oxyethira* 285

***gemma***, *Nectopsyche* 324

***gemmoides***, *Nectopsyche* 325

*genuosa*, *Nectopsyche* 324

*geolca*, *Cheumatopsyche* 132

***geranoides***, *Chimarra* 383

***gertschi***, *Polycentropus* 427

***giampaolina***, *Alisotrichia* 197

***gibba***, *Chimarra* 383

***gigas***, *Marilia* 362

***gilmari***, *Neotrichia* 251

***gilvimacula***, *Chimarra* 383

***giovannae***, *Polycentropus* 427

***glabra***, *Helicopsyche* 82

***glabra***, *Ochrotrichia* 268

***gladiator***, *Smicridea* 171

***gladiocincta***, *Australobiosis* 114

***glasa***, *Oxyethira* 285

***globigona***, *Nectopsyche* 325

**Glossosoma** [Glossosomatidae] 50

***Glyphocentron*** [Xiphocentronidae] 455

***goiana***, *Metrichia* 238

***goianensis***, *Notalina* 331

***goitiai***, *Protoptila* 70

***golfitoensis***, *Helicopsyche* 85

***gomboska***, *Metrichia* 238

***gomezi***, *Byrsopteryx* 207

***gomezi***, *Leucotrichia* 227

***gomezi***, *Smicridea* 171

***gomphotheria***, *Smicridea* 171

***gondela***, *Chimarra* 383

***gonzalezae***, *Wormaldia* 406

***gorba***, *Zumatrichia* 303

***gordita***, *Metrichia* 238

***gotera***, *Neotrichia* 251

*gracilis* Banks 1904, *Leptocella* (now *Nectopsyche*) 325

***gracilis***, *Marilia* 362

***gracilis***, *Nectopsyche* 325

***gracilis***, *Pseudostenopsyche* 452

***gracilis***, *Triplectides* 346

***graciosa***, *Alterosa* 367

***graciosa***, *Polycentropus* 427

***granda***, *Psilopsyche* 410

***grandis***, *Smicridea* 172

***grandisaccata***, *Smicridea* 172

***graniculata***, *Clistoronia* 350

***granpiedrana***, *Helicopsyche* 85

*graphica*, *Nectopsyche* 329

***gregarium***, *Metachorema* 118

***grenadensis***, *Helicopsyche* 86

***grenadensis***, *Hydroptila* 217

***grenadensis***, *Smicridea* 172

***gretae***, *Ochrotrichia* 268

***grimaldii***†, *Cariboptila* 45

***griseola***, *Gumaga* 448

***griseum***, *Lepidostoma* 309

***griseum***, *Metachorema* 118

***griseum***, *Microthremma* 78

***griseus***, *Austrocentrus* 77

***grisoli***, *Synoestropsis* 189

*grisolinum*, *Leptonema* 139

**Grumicha** [Sericostomatidae] 447

***grumicha***, *Grumicha* 448

**Grumichella** [Leptoceridae] 318

***guadaloupe***, *Triaenodes* 343

***guadeloupea***, *Alisotrichia* See ***orophila guadeloupea***

***guadeloupensis***, *Helicopsyche* 86

***guahybae***, *Anastomoneura* 358

***guahybae***, *Mortoniella* 59

***guaira***, *Marilia* 362

***guairica***, *Mortoniella* 60

***guajira***, *Cariboptila* 45

***guanacasteca***, *Bredinia* 205

***guanacasteca***, *Chimarra* 383

***guapa***, *Chimarra* 383

***guapimirim***, *Alterosa* 367

***guara***, *Helicopsyche* 86

***guarani***, *Itauara* 52

***guarani***, *Protoptila* 70

***guata***, *Protoptila* 70

***guatemalensis***, *Chimarra* 383

***guatemalensis***, *Polycentropus* 427

*guatemalum*, *Leptonema* 136

*Guerrotrichia*, [Hydroptilidae] 246

***gula***, *Zumatrichia* 303

**Gumaga** [Sericostomatidae] 448

***gundlachi***, *Macronema* 151

***gurneyi***, *Ochrotrichia* 268

***guyanae***, *Polyplectropus* 438

***guyanense***, *Leptonema* 141

***guyanensis***, *Chimarra* 383

***guyanensis***, *Itauara* 52

### H

***haesitationis***, *Chimarra* 383

***hispaniola***†, *Macronema* 152

***hageni***, *Helicopsyche* 86

***hageni***, *Macronema* 151

***hagenii***, *Rhyacopsyche* 296

***hairouna***, *Chimarra* 384

***haitiense***, *Xiphocentron* 458

*haitiensis*, *Antillotrichia* See ***haitiense***, *Xiphocentron*

***haitiensis***, *Helicopsyche* 86

***haitises***, *Oecetis* 336

***hajla***, *Neotrichia* 252

***hajla***, *Smicridea* 172

***hajtoka***, *Rhyacopsyche* 297

***halidus***, *Polycentropus* 427

***hamadae***, *Amazonatolica* 312

***hamata***, *Atopsyche* 105

***hamata***, *Culoptila* 49

***hamata***, *Hydroptila* 218

***hamatilis***, *Ochrotrichia* 268

***hamatus***, *Polyplectropus* 438

***hamifer***, *Limnephilus* 352

***hamiferus***, *Polycentropus* 427

***hamiltoni***, *Notalina* 331

***hamiltoni***, *Polycentropus* 427

***hamuli***, *Leptonema* 141

***hamulifera***, *Costatrichia* 211

***hamulus***, *Polyplectropus* 438

***hansoni***, *Phylloicus* 33

***haraga***, *Smicridea* 172

***haranga***, *Metrichia* 238

***harma***, *Flintiella* 214

***harmas***, *Ochrotrichia* 269

***haroma***, *Zumatrichia* 303

***harpagum***, *Leptonema* 141

*Harpax* [Hydrobiosidae] 99

***harrisi***, *Anchitrichia* 200

***harrisi***, *Cernotina* 414

***harrisi***, *Flintiella* 214

***harrisi***, *Mejicanotrichia* 232

***hasta***, *Rhyacopsyche* 297

***hastilis***, *Cernotina* 415

***hata***, *Ochrotrichia* 269

***hatunpuna***, *Atopsyche* 105

***hedamafera***, *Wormaldia* 406

***heleios***, *Neotrichia* 252

***helenae***, *Metrichia* 238

***helenae***, *Smicridea* 172

***helicina***, *Hydroptila* 218

***helicoidella***, *Helicopsyche* 87

**Helicopsyche** [Helicopsychidae] 79

***heligma***, *Chimarra* 384

***heppneri***, *Chimarra* 384

***heppneri***, *Leptonema* 141

***heredia***, *Flintiella* 214

***herrerai***, *Polyplectropus* 438

**Hesperophylax** [Limnephilidae] 350

**Heterochorema** [Hydrobiosidae] 117

***heveli***, *Lepidostoma* 309

***hiaspa***, *Neotrichia* 252

***hidala***, *Cerasmatrichia* 209

***hidalgoi***, *Atopsyche* 105

***hilosa***, *Oxyethira* 285

***hinnulus***, *Atopsyche* 105

***hintoni***, *Atopsyche* 105

***hirsutum***, *Leptonema* 141

***hirsutus***, *Austrocosmoecus* 349

***hirudopsis aitija***, *Alisotrichia* 197

***hirudopsis hirudopsis***, *Alisotrichia* 197

***hispaniola***†, *Macronema* 152

***hispaniolica***, *Cariboptila* 45

***hispaniolina***, *Alisotrichia* 197

***hispida***, *Atopsyche* 105

***hispida***, *Leucotrichia* 227

***Histricoverpa*** [Calamoceratidae] 26

***hodgesi***, *Mortoniella* 60

***hodgesi***, *Triaenodes* 343

***hoffmannae***, *Hydroptila* 218

***hollyae***, *Polyplectropus* 438

***holzenthali***, *Alisotrichia* 197

***holzenthali***, *Alterosa* 367

***holzenthali***, *Anchitrichia* 200

***holzenthali***, *Helicopsyche* 87

***holzenthali***, *Marilia* 362

***holzenthali***, *Neoathripsodes* 330

***holzenthali***, *Phylloicus* 33

***holzenthali***, *Polycentropus* 427

***holzenthali***, *Polyplectropus* 438

***holzenthali***, *Smicridea* 172

*Homoeoplectron* [Calamoceratidae] 28

***homora***, *Smicridea* 172

***hondurenia***, *Ochrotrichia* 269

***hoogstraali***, *Plectropsyche* 159

***horga***, *Smicridea* 172

***horgoska***, *Neotrichia* 252

***hornitos***, *Triaenodes* 343

***hossa***, *Hydroptila* 218

***hosulaba***, *Acostatrichia* 193

***hozosa***, *Oxyethira* 285

***huacachaca***, *Atopsyche* 105

***huacapuncu***, *Atopsyche* 106

***huachacuyac***, *Atopsyche* 106

***huainacapac***, *Atopsyche* 106

***huallaripa***, *Atopsyche* 106

***huamachucu***, *Atopsyche* 106

***huamantincoae***, *Marilia* 362

***huanapu***, *Atopsyche* 106

***huanucu***, *Atopsyche* 106

***huarcu***, *Atopsyche* 106

***huasteca***, *Protoptila* 70

***huava***, *Protoptila* 70

**Hudsonema** [Leptoceridae] 320

***huenga***, *Atopsyche* 107

***huismanae***, *Leptonema* 141

***humerosa***, *Marilia* 362

***hyadesi***, *Monocosmoecus* 353

*hyalina*, *Hydropsyche* See ***hyalinum***, *Macrostemum*

***hyalina***, *Oxyethira* 285

*hyalinum* var. Ulmer 1907, *Macronema* See ***ulmeri***, *Macrostemum*

*hyalinum* var. Ulmer 1913, *Macronema* See ***erichsoni***, *Macrostemum*

***hyalinum***, *Macrostemum* 156

***hybrida***, *Smicridea* 172

**Hydropsyche** [Hydropsychidae] 133

**Hydroptila** [Hydroptilidae] 215

***hymenochilus***, *Polyplectropus* 438

***hyoeides***, *Chimarra* 384

***hystricosa***, *Mortoniella* 60

***hystricosus***, *Polyplectropus* 438

### I

***iana***, *Atopsyche* 107

***iara***, *Oecetis* 336

***ibarrai***, *Lepidostoma* 309

***ibarrai***, *Polycentropus* 427

***icona***, *Hydroptila* 218

***ideolus***, *Cnodocentron* 454

*Igazu* See ***Iguazu***

***ignera***, *Protoptila* 70

***igrapiuna***, *Ochrotrichia* 269

**Iguazu** [Hydrobiosidae] 117

***iguazu***, *Oecetis* 336

***iguazu***, *Smicridea* 172

***ikonnikovi***, *Atopsyche* 107

***ildria***, *Ochrotrichia* 269

***ilionea***, *Xiphocentron* 458

***illiesi***, *Anomalocosmoecus* 348

***illiesi***, *Nothotrichia* 260

***illobia***, *Mayatrichia* 231

***imberti***, *Wormaldia* 406

***imitator***, *Leucotrichia* 227

***immaculata***, *Chimarra* 384

*immaculata*, *Macronema* See ***immaculatum***, *Macronema*

***immaculatum***, *Cnodocentron* 454

***immaculatum***, *Macronema* 152

***implexa***, *Atopsyche* 107

***impluviatus***, *Verger* 356

***inaequispina***, *Eosericostoma* 77

***inaequispina***, *Oxyethira* 285

***inaequispina***, *Smicridea* 173

***inappendiculata***, *Alterosa* 367

***inarmata***, *Smicridea* 173

***inarmatus***, *Polyplectropus* 438

***inbio***, *Austrotinodes* 40

***inca***, *Leptonema* 142

***inca***, *Wormaldia* 407

***incatupac***, *Atopsyche* 107

***inchoata***, *Chimarra* 384

***incipens***, *Chimarra* 384

***incisa***, *Helicopsyche* 87

***inconspicua***, *Oecetis* 336

***indefinida***, *Ochrotrichia* 269

**Indusia** [†] [Limnephilidae] 10

***inermis***, *Notidobiella* 450

***inflata***, *Chimarra* 384

***inflata***, *Oecetis* 337

***inflaticornis***, *Leucotrichia* 227

***infundibulum***, *Marilia* 362

***ingloria***, *Ochrotrichia* 269

***inops***, *Leucotrichia* 227

***inornata***, *Contulma* 22

***inornata***, *Hydroptila* 218

*inornata*, *Oecetis* 337

***insignis***, *Wormaldia* 407

***inspiratum***, *Leptonema* 142

***insulanum***, *Leptonema* 142

***insulare***, *Xiphocentron* 458

***insularis***, *Amphoropsyche* 313

***insularis***, *Ochrotrichia* 269

***insularis***, *Polycentropus* 427

***insularis***, *Polyplectropus* 438

*interclusus*, *Barypenthus* 359

***intermedia***, *Ochrotrichia* 269

***intermedium***, *Leptonema* 142

***interrupta***, *Leucotrichia* 227

***interrupta***, *Neotrichia* 252

***intersecta***, *Cernotina* 415

***intervales***, *Alterosa* 367

***intervales***, *Mortoniella* 60

*intervena*, *Nectopsyche* 325

*intortigona*, *Neotrichia* [nomen nudum] 255

***intortilis***, *Ochrotrichia* 269

***intraspira***, *Rhyacopsyche* 297

***inusitatus***, *Polycentropus* 428

***involuta***, *Ochrotrichia* 270

***ipixuna***, *Costatrichia* 211

***iridescens***, *Mortoniella* 60

***iridescens***, *Neotrichia* 252

***iridescens***, *Phylloicus* 33

***irregularis***, *Atopsyche* 107

***irroratum***, *Leptonema* 142

***irwini***, *Austrotinodes* 40

***irwini***, *Chimarra* 384

***isela***, *Wormaldia* 407

*islamarga*, *Leptonema* See *islamarga*, *Smicridea*

*islamarga*, *Smicridea* 174

***islena***, *Ochrotrichia* 270

***ismetla***, *Neotrichia* 252

*Isocentropus* [Limnephilidae] 353

**Isochorema** [Hydrobiosidae] 117

*Isochorema*†, junior homonym [Hydrobiosidae] 117

***itabaiana***, *Metrichia* 238

***itatiaia***, *Polycentropus* 428

***itatiaia***, *Triplectides* 346

***itatiaiae***, *Alterosa* 367

**Itauara** [Glossosomatidae] 51

**Ithytrichia** [Hydroptilidae] 223

***ixcateopana***, *Ochrotrichia* 270

***ixtala***, *Protoptila* 70

***ixtlahuaca***, *Lepidostoma* 309

***ixtlahuaca***, *Ochrotrichia* 270

***izabala***, *Chimarra* 384

### J

***jaba***, *Atopsyche* 107

***jaffuelli***, *Triplectides* 346

***jamaicae***, *Metrichia* See ***kumanskii jamaicae***

***jamaicensis***, *Cariboptila* 45

***jamaicensis***, *Chimarra* 384

***jamaicensis***, *Oxyethira* 286

***jamaicensis***, *Polycentropus* 428

***jamaicensis***, *Smicridea* 173

***jamapa***, *Culoptila* 49

***jamesii***, *Itauara* 52

***janella***, *Oxyethira* 286

***janethae***, *Atopsyche* 107

***janolah***, *Leptonema* 142

***janstockiana***, *Amphoropsyche* 314

***janzeni***, *Chimarra* 385

***japoda***, *Atopsyche* 107

***jarochita***, *Neotrichia* 252

***jaula***, *Neochorema* 120

***jeldesi***, *Polycentropus* 428

***jemima***, *Chimarra* 385

***jenseni***, *Nectopsyche* 325

***jimena***, *Rhyacopsyche* 297

***jobbra***, *Ceratotrichia* 210

***joergenseni***, *Polycentropus* 428

***jolandae***, *Ochrotrichia* 270

***jolandae***, *Protoptila* 70

***jonssoni***, *Ochrotrichia* 270

***jordaensis***, *Alterosa* 368

***jordanensis***, *Notalina* 331

*Jörgenseni*, *Polycentropus* See ***joergenseni***, *Polycentropus*

***jorobada***, *Metrichia* 238

***juana***, *Metrichia* 238

***juarox***, *Wormaldia* 407

***jugescens***, *Chimarra* 385

***julia***, *Itauara* 52

***juliae***, *Chimarra* 385

***juliae***, *Polyplectropus* 438

***julieta***, *Protoptila* 70

***julus***, *Xiphocentron* 458

***jundiai***, *Smicridea* 173

***juntada***, *Neotrichia* 252

***jureia***, *Grumichella* 319

### K

***kagyla***, *Betrichia* 203

***kalaom***, *Helicopsyche* 87

***kamakan***, *Atopsyche* 107

***kamakan***, *Xiphocentron* 458

***kamesa***, *Atopsyche* 108

***kampa***, *Neotrichia* 253

***kampoka***, *Neotrichia* 253

***kampoka***, *Smicridea* 173

***kana***, *Smicridea* 173

***kanalas***, *Centromacronema* 130

***kantala***, *Alisotrichia* 197

***kanukarum***, *Polyplectropus* 439

***kanukua***, *Alisotrichia* 197

***kapara***, *Smicridea* 173

***karenae***, *Kumanskiella* 224

***karikatla***, *Hydroptila* 219

***karima***, *Hydroptila* 219

***karukerae***, *Smicridea* 173

***kateae***, *Leucotrichia* 228

***kehelia***, *Neotrichia* 253

***Kelleyella*** , [Hydroptilidae] 280

***kerek***, *Oxyethira* 286

***kerekeda***, *Zumatrichia* 303

***ketaga***, *Ochrotrichia* 270

***ketaguka***, *Neotrichia* 253

***ketarca***, *Ochrotrichia* 270

***ketleben***, *Macronema* 152

***ketos***, *Leptonema* 142

***kettes***, *Ochrotrichia* 270

***ketvilla***, *Acostatrichia* 193

***kevera***, *Alisotrichia* 197

***kihara***, *Acostatrichia* 193

***kilambe***, *Triaenodes* 343

***kingi***, *Atopsyche* 108

***kingsolveri***, *Marilia* 362

***kingsolveri***, *Polyplectropus* 439

***kingstona***, *Helicopsyche* 87

***kisgula***, *Zumatrichia* 303

***kislaba***, *Zumatrichia* 303

***kjeri***, *Protoptila* 70

***knulli***, *Lepidostoma* 309

***knutsoni***, *Oecetis* 337

***kocka***, *Metrichia* 238

***koki***, *Chimarra* 385

***kolbiana***, *Psilopsyche* 410

***kontilos***, *Chimarra* 385

***kovera***, *Smicridea* 174

***koztesa***, *Zumatrichia* 303

**Kumanskiella** [Hydroptilidae] 224

***kumanskii jamaicae***, *Metrichia* 239

***kumanskii kumanskii***, *Metrichia* 239

***kunbenorum***, *Leptonema* 142

***kurta***, *Neotrichia* 253

***kurtika***, *Neotrichia* 253

***kurtitva***, *Neotrichia* 253

***kuscheli***, *Verger* 357

***kylistos***, *Polyplectropus* 439

### L

***labafura***, *Ochrotrichia* 270

***labiatus***, *Austrotinodes* 40

***labios***, *Neotrichia* 253

***lacandona***, *Hydroptila* 219

***lacanha***, *Smicridea* 174

*lacanja*, *Smicridea* See ***lacanha***, *Smicridea*

***lacertina***, *Neotrichia* 253

***lachlani***, *Macronema* 152

***lacinatum***, *Lepidostoma* 309

***lacuna***, *Metrichia* 239

***lacuniferum***, *Leptonema* 142

***lacustris***, *Chiloecia* 447

***ladislavii***, *Dicaminus* 213

*Lagenopsyche*, [Hydroptilidae] 280

***laguna***, *Chimarra* 385

***lagunita***, *Oxyethira* 286

***lahontanensis***, *Nectopsyche* 326

***lambda***, *Helicopsyche* 87

***laminatus***, *Polyplectropus* 439

***lanceolata***, *Cernotina* 415

***lanceolata***, *Contulma* 22

***laneblina***, *Helicopsyche* 87

***langleyae***, *Chimarra* 386

***lapa***, *Zumatrichia* 303

***laposka***, *Leucotrichia* 228

***lara***, *Helicopsyche* 87

***larica***, *Microchorema* 119

***larimar***, *Ochrotrichia* 270

***lasia***, *Cheumatopsyche* 132

***lata***, *Chimarra* 386

*latchani*, *Verger* 357

***laterale***, *Parasericostoma* 451

***lateralis***, *Marilia* 362

***laterospina***, *Protoptila* 70

***laticula***, *Cernotina* 415

***latiforceps***, *Chimarra* 386

***latior***, *Austrotinodes* 42

***latipala***, *Smicridea* 174

***latipalpis***, *Alisotrichia* 197

***latispina***, *Mortoniella* 60

*latum*, *Macronema* See *latus*, *Phylloicus*

*latus*, *Phylloicus* 35

***lauglo***, *Wormaldia* 407

***lausus***, *Cnodocentron* 454

***lavinia***, *Xiphocentron* 458

***lazzariae***, *Helicopsyche* 87

***lazzarii***, *Cernotina* 415

***lebena***, *Smicridea* 174

***leccii***, *Grumichella* 319

*leechi*, *Lepidostoma* 309

***leei***, *Mortoniella* 60

***lefela***, *Neotrichia* 253

***legeza***, *Ochrotrichia* 270

***legezoa***, *Smicridea* 174

*Leiochiton*, [Hydroptilidae] 300

***leloga***, *Flintiella* 214

***leloga***, *Smicridea* 174

***lemeza***, *Angrisanoia* 202

***lemeza***, *Smicridea* 174

***lemniscata***, *Metrichia* 239

***lenophora***, *Metrichia* 239

***leona***, *Ochrotrichia* 271

***leonensis***, *Neotrichia* 253

***leonilae***, *Lepidostoma* 309

***leonilae***, *Protoptila* 71

**Lepidostoma** [Lepidostomatidae] 307

*Leptocella* [Leptoceridae] 321

*Leptocellodes* [Leptoceridae] 318

**Leptonema** [Hydropsychidae] 135

***lerma***, *Leucotrichia* 228

***leroda***, *Mortoniella* 60

**Leucotrichia** [Hydroptilidae] 224

***lewalleni***, *Helicopsyche* 88

***lewisi***, *Nectopsyche* 326

***lezarda***, *Zumatrichia* 303

***lilicae***, *Atopsyche* 108

***limeirai***, *Ochrotrichia* 271

**Limnephilus** [Limnephilidae] 351

*limnophilus*, *Verger* 358

***limon***, *Chimarra* 386

***limona***, *Mortoniella* 60

***limonensis***, *Ochrotrichia* 271

***limpia***, *Leucotrichia* 228

***linabena***, *Helicopsyche* 88

***lineaticorne***, *Leptonema* 142

***lineatum***, *Macronema* 152

***lineatus***, *Austrotinodes* 40

***linguata***, *Helicopsyche* 88

***lingulatus***, *Polycentropus* 428

***linterna***, *Alisotrichia* 197

***liqua***, *Protoptila* 71

***lisae***, *Plectromacronema* 158

***lithus***, *Limnephilus* 352

***lituratus***, *Phylloicus* 33

***llaviuco***, *Phylloicus* 33

***lobata***, *Alisotrichia* 197

***lobata***, *Chimarra* 386

***lobata***, *Helicopsyche* 88

***lobata***, *Neotrichia* 253

***lobata***, *Smicridea* 174

***lobatum***, *Microthremma* 78

*lobatus*, *Rhyacophylax* See ***lobata***, *Smicridea*

***lobifera***, *Ochrotrichia* 271

***lobiferum***, *Neochorema* 120

***lobiferum***, *Xiphocentron* 458

***lobisomem***, *Cernotina* 415

***lobosa***, *Atopsyche* 108

*lobulifera*, *Rheochorema* See ***lobuliferum***, *Rheochorema*

***lobuliferum***, *Rheochorema* 123

***locula***, *Protoptila* 71

***lodora***, *Costatrichia* 211

***logana***, *Ochrotrichia* 271

***loja***, *Byrsopteryx* 207

***lojaense***, *Leptonema* 143

***lojaensis***, *Chimarra* 386

***longicornuta***, *Mastigoptila* 54

***longipenis***, *Oxyethira* 286

*longipenne*, *Psilochorema* See ***longipennis***, *Atopsyche*

***longipennis***, *Atopsyche* 108

***longipilosa***, *Mystacopsyche* 410

***longiscapa***†, *Cariboptila* 45

***longispina***, *Cernotina* 415

***longispina***, *Metrichia* 239

***longispina***, *Mortoniella* 61

***longispina***, *Ochrotrichia* 271

***longispina***, *Zumatrichia* 304

***longispinata***, *Protoptila* 71

***longispinosa***, *Oxyethira* 287

***longispinosus***, *Polycentropus* 429

***longispinum***, *Austrotinodes* 40

***longissima***, *Cernotina* 415

***longissima***, *Hydroptila* 219

***longissima***, *Metrichia* 239

***longissima***, *Neotrichia* 254

***longissima***, *Oxyethira* 287

*longissimus*, *Hydroptila* See ***longissima***, *Hydroptila*

***longistyla***, *Betrichia* 203

***longiterga***, *Chimarra* 386

***longitudinis***, *Metrichia* 239

*lonquimaya*, *Verger* 356

***lorada***, *Protoptila* 71

*Lorotrichia*, [Hydroptilidae] 246

***lourditae***, *Smicridea* 174

***loxana***, *Banyallarga* 27

*Loxinum* [Philopotamidae] 400

***Loxotrichia*** , [Hydroptilidae] 280

***luanae***, *Oxyethira* 287

*lucia*, *Chimarra* 389

***lucia***, *Protoptila* 71

***lucidula***, *Cailloma* 115

***lucinda***, *Itauara* 52

*lucipeta*, *Nectopsyche* 326

***lucrecia***, *Neotrichia* 254

***lucumon***, *Machairocentron* 455

***luma***, *Wormaldia* 407

***luna***, *Metrichia* 239

***lunatum***, *Leptonema* 143

***lupita***, *Ochrotrichia* 271

*luquillo*, *Chimarra* 371

*lustrica*, *Helicopsyche* 82

***lutea***, *Cernotina* 415

***lutea***, *Helicopsyche* 88

***luteipenne***, *Macronema* 152

***lutzi***, *Verger* 357

*lutzinus*, *Monocosmoecus* 353

### M

***macacu***, *Leptonema* 143

***macae***, *Alisotrichia* 198

***macara***, *Chimarra* 386

***macarenica***, *Mortoniella* 61

***machadoi***, *Chimarra* 386

***machadorum***, *Wormaldia* 407

**Machairocentron** [Xiphocentronidae] 454

***macherophora***, *Chimarra* 386

***machiguenga***, *Ochrotrichia* 271

***macilenta***, *Protoptila* 71

***maclachlani***, *Tupiniquintrichia* 300

*Mac-Queeni*, *Psilopsyche* See *macqueeni*, *Psilopsyche*

*macqueeni*, *Psilopsyche* 411

***macrocera***, *Trichovespula* 453

***macrocerca***, *Atopsyche* 108

**Macronema** [Hydropsychidae] 149

*Macronemum* [Hydropsychidae] 149

***macrophallata***, *Metrichia* 239

**Macrostemum** [Hydropsychidae] 154

***macrosterna***, *Oxyethira* 287

*macrostylus*, *Polycentropus* 422

*maculata*, *Smicridea* 161

***maculatum***, *Leptonema* 143

*maculatum*, *Macrostemum* 155

***maculatus***, *Phylloicus* 33

***maculatus***, *Polyplectropus* 439

***maculipennis***, *Nectopsyche* 326

***maculisternum***, *Helicopsyche* 88

***macuta***, *Mortoniella* 61

***madicola***, *Metrichia* 240

***madininae***, *Austrotinodes* 40

***madre***, *Metrichia* 240

***madremia***, *Cariboptila* 46

***maesi***, *Polyplectropus* 439

***maesi***, *Wormaldia* 407

***maga***, *Ochrotrichia* 271

***magas***, *Leptonema* 143

***magdalenae***, *Smicridea* 175

*magellanica*, *Rheochorema* See ***magellanicum***, *Rheochorema*

***magellanicum***, *Rheochorema* 123

*Magellomyia* [Limnephilidae] 355

***magna***, *Metrichia* 240

*magna*, *Smicridea* 177

***magnipinnata***, *Smicridea* 175

***magnus***, *Hesperophylax* 350

***magnus***, *Phylloicus* 34

*magnus*, *Rhyacophylax* See *magna*, *Smicridea*

***maitacapac***, *Atopsyche* 108

***maiteae***, *Xiphocentron* 459

***majada***, *Atopsyche* 108

***major***, *Atopsyche* 108

***major***, *Brachysetodes* 317

***major***, *Marilia* 363

***major***, *Phylloicus* 34

***majuscula***, *Chimarra* 386

***malada***, *Metrichia* 240

***maldonadoi***, *Chimarra* 387

***malica***, *Protoptila* 71

***malickyi***, *Neotrichia* 254

***mammillatus***, *Cyrnellus* 419

***manabi***, *Atanatolica* 316

***manabiensis***, *Bredinia* 205

***manauara***, *Flintiella* 214

***mancocapac***, *Atopsyche* 109

***mandeba***, *Cernotina* 415

***mandibulatum***, *Leptonema* 143

***mangaratiba***, *Smicridea* 175

***manicata***, *Synoestropsis* 189

***manopla***, *Neotrichia* 254

***manu***, *Osflintia* 340

***manuensis***, *Ochrotrichia* 272

***manuensis***, *Polyplectropus* 439

***manzanara***, *Smicridea* 175

***mara***, *Protoptila* 71

***marcanoi***, *Polycentropus* 429

***margaritae***, *Chimarra* 387

***margaritena***, *Neotrichia* 254

*margaritensis*, *Helicopsyche* 96

*marginalis*, *Cyrnellus* 419

***maria***, *Neotrichia* 254

***marica***, *Ochrotrichia* 272

***marica***, *Zumatrichia* 304

***marighellai***, *Hydroptila* 219

**Marilia** [Odontoceridae] 360

***marini***, *Mortoniella* 61

***marinonii***, *Alterosa* 368

***maritza***, *Chimarra* 387

***maritza***, *Hydroptila* 219

***maritza***, *Oecetis* 337

***marlieri***, *Smicridea* 175

***marqua***, *Protoptila* 71

***marquesi***, *Oecetis* 337

***martinae***, *Oecetis* 338

***martinica***, *Smicridea* 175

*martinmoselyi*, *Chimarra* 388

***martorelli***, *Hydroptila* 219

***marua***, *Smicridea* 175

***maryae***, *Oxyethira* 287

***masa***, *Zumatrichia* 304

***masafuera***, *Verger* 357

*masatierra*, *Verger* 357

***masinca***, *Leptonema* 143

***maskara***, *Zumatrichia* 304

***maskoska***, *Zumatrichia* 304

***maspeluda***, *Oecetis* 338

***mastelleri***, *Cernotina* 415

***mastigion***, *Leptonema* 143

**Mastigoptila** [Glossosomatidae] 53

***matadero***, *Oxyethira* 287

***matagalpa***, *Smicridea* 175

***matagalpa***, *Wormaldia* 407

***matancilla***, *Smicridea* 175

***matatlanticus***, *Polyplectropus* 439

***mathisi***, *Alisotrichia* 198

***mathisi***, *Cariboptila* 46

***mathisi***, *Marilia* 363

***mathisi***, *Neotrichia* 254

***mathisi***, *Polycentropus* 429

***mathisi***, *Polyplectropus* 439

***matthewsi***, *Macronema* 152

***matthiasi***, *Notalina* 331

***matthiasi***, *Rhyacopsyche* 297

***maxi***, *Atopsyche* 109

***maya***, *Limnephilus* 352

***maya***, *Neotrichia* 254

***maya***, *Ochrotrichia* 272

***maya***, *Oxyethira* 287

***mayana***, *Protoptila* 71

***mayanum***, *Leptonema* See ***simulans mayanum***

***mayanus***, *Polycentropus* 429

**Mayatrichia** [Hydroptilidae] 230

***maycoba***, *Ochrotrichia* 272

***mayucapac***, *Atopsyche* 109

***maza***, *Hydroptila* 219

***mechuda***, *Metrichia* 240

***medena***, *Smicridea* 175

***mederi***†, *Cariboptila* 46

*mediana* Banks 1905, *Wormaldia* (now *Chimarra*) 373

*mediana* McLachlan 1878, *Wormaldia* 373

***medinai***, *Hydroptila* 219

***medioloba***, *Cernotina* 416

***medioloba***, *Chimarra* 387

***medius***, *Phylloicus* 34

***meginca***, *Leptonema* 143

**Mejicanotrichia** [Hydroptilidae] 231

***melanochaeta***, *Helicopsyche* 88

***melleopicta***, *Leucotrichia* 228

***meloi***, *Contulma* 22

***meloi***, *Mortoniella* 61

***membrana***, *Ochrotrichia* 272

***menchuae***, *Wormaldia* 407

*mendocensis*, *Smicridea* 177

***menkei***, *Leptonema* 143

***meralda***, *Hydroptila* 219

***meralda***, *Mortoniella* 61

***merengue***, *Chimarra* 387

***merga***, *Oxyethira* 287

***merida***, *Helicopsyche* 89

***meridensis***, *Smicridea* 176

***meridiensis***, *Polycentropus* 429

*meridionalis*, *Verger* 355

**Merionoptila** [Glossosomatidae] 55

***mesembrina***, *Smicridea* 176

***mesodonta***, *Chimarra* 387

*Mesotrichia*, [Hydroptilidae] 280

***messapus***, *Xiphocentron* 459

***meta***, *Metrichia* 240

**Metachorema** [Hydrobiosidae] 118

**Metacosmoecus** [Limnephilidae] 353

***metlacensis***, *Oecetis* 338

**Metrichia** [Hydroptilidae] 232

***mexicana***†, *Antillopsyche* 445

***mexicana***, *Atopsyche* 109

***mexicana***, *Banyallarga* 27

***mexicana***, *Bredinia* 205

***mexicana***, *Chimarra* 387

***mexicana***, *Helicopsyche* 89

*mexicana*, *Hydropsyche* See ***mexicanus***, *Polycentropus*

***mexicana***, *Hydroptila* 220

***mexicana***, *Ithytrichia* 223

***mexicana***, *Marilia* 363

***mexicana***, *Mortoniella* 61

***mexicana***, *Oecetis* 338

***mexicana***, *Rhyacopsyche* 297

*mexicana*, *Wormaldia* See ***mexicana***, *Chimarra*

***mexicanum***, *Lepidostoma* 309

***mexicanus***, *Austrotinodes* 40

***mexicanus***, *Limnephilus* 352

***mexicanus***, *Phylloicus* 34

***mexicanus***, *Polycentropus* 429

***mexicanus***, *Triaenodes* 343

***mexico***, *Hesperophylax* 350

***mexico***, *Xiphocentron* 459

*Mexipsyche* [Hydropsychidae] 133

*Mexitrichia* [Glossosomatidae] 55

***mezencius***, *Xiphocentron* 459

*micans*, *Oecetis* 337

***michaelseni***, *Verger* 357

***michelbacheri***, *Sortosa* 403

***michoacanense***, *Leptonema* 143

***mickeli***, *Cheumatopsyche* 132

**Microchorema** [Hydrobiosidae] 118

***microps***, *Marilia* 363

***microsaccata***, *Smicridea* 176

*Microsiphon*, [Hydroptilidae] 245

**Microthremma** [Helicophidae] 78

***mignonae***, *Polyplectropus* 440

***milenae***, *Atopsyche* 109

***mina***, *Protoptila* 72

***minca***, *Chimarra* 387

***mincana***, *Smicridea* 176

***minensium***, *Polyplectropus* 440

***minera***, *Metrichia* 240

***minero***, *Polycentropus* 429

***minga***, *Chimarra* 388

*minima* Flint 1972, Smicridea (Rhyacophylax) 176

***minima***, *Helicopsyche* 89

***minimajada***, *Atopsyche* 109

***minima***, *Smicridea* 176

*minimus*, *Cyrnellus* 419

***minor***, *Marilia* 363

***minor***, *Monocosmoecus* 354

***minuscula***, *Helicopsyche* 89

***minuscula***, *Smicridea* 176

***minuta***, *Anomalopsyche* 21

***mirama***, *Smicridea* 176

*mirebalina*, *Oxyethira* Botosaneanu 1991 291

***mirebalina***, *Oxyethira* 287

***mirifica***, *Byrsopteryx* 207

***mirnae***, *Smicridea* 176

***misionensis***, *Atopsyche* 109

***misionensis***, *Cyrnellus* 420

***misionensis***, *Marilia* 363

***misionensis***, *Oxyethira* 288

***misionensis***, *Protoptila* 72

***misionensis***, *Triplectides* 346

***misolha***, *Hydroptila* 220

***misolja***, *Polyplectropus* 440

*mixta*, *Nectopsyche* 325

***mixteca mixteca***, *Protoptila* 72

***mixteca veracruzensis***, *Protoptila* 72

***mixteco***, *Polycentropus* 429

***mnesteus***, *Xiphocentron* 459

***mobilensis***, *Neotrichia* 254

***mocoi***, *Oxyethira* 288

***modesta***, *Marilia* 363

***modesta***, *Nectopsyche* 326

***modestus***, *Verger* 357

***modica***, *Hydroptila* 220

***moesta***, *Chimarra* 388

*moesta*, *Verger* 356

***molesta***, *Helicopsyche* 89

***molinai***, *Psilopsyche* 411

***mollicula***, *Banyallarga* 27

***moncho***, *Triaenodes* 343

***moncho***, *Xiphocentron* 459

***monda***, *Helicopsyche* 90

***monicae***, *Amphichorema* 98

***monneorum***, *Phylloicus* 34

**Monocosmoecus** [Limnephilidae] 353

*monotona*, *Triplectides* 346

***monsonorum***, *Wormaldia* 408

***montana***, *Helicopsyche* 90

***montanensis***, *Culoptila* 49

***monticola***, *Nectopsyche* 326

***monticolus***, *Phylloicus* 34

***morai***, *Triaenodes* 343

***morato***, *Alterosa* 368

***morio***, *Chimarra* 388

***morsei***, *Marilia* 363

***morsei***, *Notalina* 331

**Mortoniella** [Glossosomatidae] 55

*moselyi* Denning 1947, *Chimarra* 388

*moselyi* Ross 1956, *Chimarra* 388

***moselyi***, *Atanatolica* 316

***moselyi***, *Culoptila* 49

***moselyi***, *Leptonema* 144

***mosleyi***, *Ochrotrichia* 272

*Muangpaipsyche* [Glossosomatidae] 44

***mucronata***, *Smicridea* 176

***muelleri***, *Atopsyche* 109

***muelleri***, *Grumichella* 319

***muelleri***, *Helicopsyche* 90

***muelleri***, *Macronema* 152

***muelleri***, *Nectopsyche* 326

***muellita***, *Alisotrichia* 198

***muhni***, *Nectopsyche* 326

*muhnia*, *Oecetis* 335

***muhnianus***, *Nyctiophylax* 421

***mulata***, *Cariboptila* 46

*mulleri*, *Helicopsyche* See ***muelleri***, *Helicopsyche*

*mülleri*, *Macronema* See ***muelleri***, *Macronema*

***multidens***, *Smicridea* 177

***multilineata***, *Nectopsyche* 327

*multipunctatus*, *Triplectides* 347

***multisetosa***, *Zumatrichia* 304

***multispinosa***, *Amphoropsyche* See ***woodruffi multispinosa***

***munieca***, *Metrichia* 240

***munozi***, *Chimarra* 388

***munozi***, *Mortoniella* 61

***munozi***, *Nothotrichia* 260

***munozi***, *Phylloicus* 34

*Murielia* [Calamoceratidae] 28

***murina***, *Myotrichia* 449

***murina***, *Smicridea* 177

*Musarna* [Odontoceridae] 359

***mutica***, *Leucotrichia* 228

*mutila*, *Oecetis* 335

***mutisi***, *Rhyacopsyche* 297

***muybonita***, *Cariboptila* 46

***muyupampa***, *Atanatolica* 316

***mycterophora***, *Chimarra* 388

**Myotrichia** [Sericostomatidae] 449

***myriamae***, *Protoptila* 72

**Mystacides** [Leptoceridae] 321

**Mystacopsyche** [Philorheithridae] 410

### N

***naevosum***, *Leptonema* 144

***nahuatl***, *Culoptila* 49

***nahuatl***, *Smicridea* 177

***nanay***, *Notalina* 332

***nanda***, *Smicridea* 177

***napo***, *Amphoropsyche* 314

***napoa***, *Helicopsyche* 90

***napoensis***, *Neotrichia* 254

***narifer***, *Hydroptila* 220

***narifer***, *Polyplectropus* 440

***nasuta***, *Chimarra* 388

***navarroae***, *Wormaldia* 408

***navasi***, *Nectopsyche* 327

***nayaritensis***, *Polyplectropus* 440

***nea***, *Pangullia* 306

***neadelphus***, *Leptonema* 144

***neblina***, *Alisotrichia* 198

***neblina***, *Chimarra* 388

***neblinense***, *Leptonema* 144

***neblinensis***, *Austrotinodes* 40

***neblinensis***, *Helicopsyche* 90

***neblinensis***, *Polycentropus* 429

***neblinus***, *Triplectides* 346

***nebulosus***, *Polycentropus* 429

***necator***, *Smicridea* 177

***necopina***, *Metrichia* 240

**Necrotaulius** [†] [† Necrotaulidae] 10

**Nectopsyche** [Leptoceridae] 321

*neglecta*, *Oxyethira* 286

***negrense***, *Macrostemum* 156

***negroensis***, *Neotrichia* 254

***nelkula***, *Zumatrichia* 304

***nelsonferreirai***, *Costatrichia* 211

***nemorosa***, *Smicridea* 177

***nemtompa***, *Smicridea* 178

**Neoathripsodes** [Leptoceridae] 330

**Neoatopsyche** [Hydrobiosidae] 119

**Neoatriplectides** [Atriplectididae] 24

*Neoatriplectides* sp. 25

**Neochorema** [Hydrobiosidae] 120

*Neodinarthrum* [Lepidostomatidae] 307

***neofimbriata***, *Chimarra* 388

***neoleonensis***, *Hydroptila* 220

*Neoleptonema* [Hydropsychidae] 135

***neolobosa***, *Atopsyche* 109

***Neonotalina*** [Leptoceridae] 330

**Neopsilochorema** [Hydrobiosidae] 121

**Neotrichia** [Hydroptilidae] 245

***neotropicalis***, *Atopsyche* 109

***neotropicalis***, *Metrichia* 240

***neotropicalis***, *Nyctiophylax* 421

***neotropicalis***, *Tricholeiochiton* 300

***neotropicus***, *Triplectides* 346

***nesidion***, *Xiphocentron* 459

***nesiotes***, *Neotrichia* 254

***nessimiani***, *Alisotrichia* 198

***nessimiani***, *Alterosa* 368

***neuquenensis***, *Schajovskoya* 123

***nevada***, *Contulma* 22

***nevadus***, *Triplectides* 347

***nhundiaquara***, *Betrichia* 203

***nica***, *Banyallarga* 27

***nicaragua***, *Ochrotrichia* 272

***nicaraguensis***, *Polyplectropus* 440

***nicaraguensis***, *Triaenodes* 343

***nicehuh***, *Chimarra* 389

***nielseni***, *Austrotinodes* 40

***nigerrima***, *Smicridea* 178

***nigra***, *Atanatolica* 316

***nigra***, *Chimarrhodella* 401

*nigra*, *Helicopsyche* 89

***nigrescens***, *Marilia* 363

***nigricans***, *Smicridea* 178

***nigricapilla***, *Nectopsyche* 327

***nigriceps***, *Polycentropus* 429

***nigridentata***, *Cernotina* 416

***nigrifrons***, *Centromacronema* 130

***nigripenne***, *Centromacronema* 130

***nigripennis***, *Phylloicus* 35

***nigripennis***, *Smicridea* 178

***nigripennis***, *Triplectides* 347

***nigrisensilla***, *Helicopsyche* 90

***nigritta***, *Metrichia* 241

***nigrofasciatus***, *Metacosmoecus* 353

***nigrum***, *Macrostemum* 156

***niltonsantosi***, *Neotrichia* 255

***nimmoi***, *Ochrotrichia* 272

*nisus*, *Xiphocentron* 458

*nivea* Hagen 1861, *Setodes* (now *Nectopsyche*) [extralimital] 327

*nivea* Navás 1920, *Leptocella* (now *Nectopsyche*) 327

***nivea***, *Atanatolica* 316

*niveistigma*, *Centromacronema* 129

*nivosa*, *Smicridea* 176

*nivosus*, *Rhyacophylax* See *nivosa*, *Smicridea*

***nobsca***, *Marilia* 363

***noite***, *Costatrichia* 211

*Nolga* [Limnephilidae] 353

**Nolganema** [Hydrobiosidae] 121

***nordenskioeldii***, *Antarctoecia* 349

***nordestina***, *Atanatolica* 316

*Nosopus* [Lepidostomatidae] 307

*Nostrafila* [Limnephilidae] 355

**Notalina** [Leptoceridae] 330

***noteuna***, *Neotrichia* 255

**Nothotrichia** [Hydroptilidae] 260

**Notidobiella** [Sericostomatidae] 449

*Notiomyia* [Calamoceratidae] 28

***novafriburgensis***, *Polyplectropus* 440

*novamexicana*, *Hydropsyche* 134

***novara***, *Neotrichia* 255

***nowaczyki***, *Metrichia* 241

***nubestre***, *Leptonema* 144

***nublensis***, *Brachysetodes* 317

***numanus***, *Xiphocentron* 459

***nusagandia***, *Hydroptila* 220

**Nyctiophylax** [Polycentropodidae] 420

***nygmosum***, *Leptonema* 144

***nyultka***, *Oxyethira* 288

### O

***oaxaca***, *Chimarra* 389

***oaxacense***, *Lepidostoma* 310

***oaxacensis***, *Centromacronema* 130

***oaxacensis***, *Triaenodes* 344

***oaxaquensis***, *Polyplectropus* 440

***oberdorffi***, *Oecetis* 338

***obesa***, *Smicridea* 178

***obliqua***, *Cernotina* 416

***obliqua***, *Neoatopsyche* 120

***obliqua***, *Rhyacopsyche* 297

***obliqua***, *Smicridea* 178

***obliqua***, *Synoestropsis* 189

***obliquus***, *Alloecentrellodes* 76

***obliquus***, *Phylloicus* 35

***obliquus***, *Verger* 357

***oblongata***, *Ochrotrichia* 272

***obovata***, *Ochrotrichia* 272

***obscura***, *Chimarra* 389

***obscura***, *Helicopsyche* 90

***obscura***, *Oxyethira* 288

***obscurum***, *Centromacronema* 130

***obtecta***, *Ochrotrichia* 273

***obtusus***, *Monocosmoecus* 354

***obtusus***, *Polycentropus* 430

***occidentale***, *Helicopsyche* 90

***occidentalis***, *Betrichia* 203

***occidentalis***, *Hydropsyche* 134

*ocellata*, *Anomalopsyche* 21

***ochracea***, *Mystacopsyche* 410

*ochraceum*, *Leptonema* 140

**Ochrotrichia** [Hydroptilidae] 261

***ochthephila***, *Helicopsyche* 91

***ocosingua***, *Helicopsyche* 91

***ocreata***, *Scotiotrichia* 75

***octospina***, *Smicridea* 178

***oculatum***, *Centromacronema* 130

***odonta***, *Cernotina* 416

***odonta***, *Chimarra* 389

*Oecetina* [Leptoceridae] 332

*Oecetinella* [Leptoceridae] 332

**Oecetis** [Leptoceridae] 332

***okanoganensis***, *Ochrotrichia* 273

***oldala***, *Ochrotrichia* 273

***oldalia***, *Neotrichia* 255

*Olemira* [Lepidostomatidae] 307

*olens*, *Monocosmoecus* 354

***olivacea***, *Smicridea* 178

***oliveri***†, *Antillopsyche* 445

***olmos***, *Leptonema* 144

***olorina***, *Neotrichia* 255

***olvidada***, *Protoptila* 72

***onchyrhina***, *Chimarra* 389

***onima***, *Chimarra* 389

***onorei***, *Atopsyche* 109

***onyx***, *Nectopsyche* 327

***opalescens***, *Helicopsyche* 91

***opinionis***, *Mortoniella* 61

***orejona***, *Neotrichia* 255

***orellanai***, *Oxyethira* 288

***orgaosensis***, *Alterosa* 368

***orientale***, *Xiphocentron* See ***cubanum orientale***

***orientalis***, *Atopsyche* 109

**Orinocotrichia** [Hydroptilidae] 278

***orlandoi***, *Neotrichia* 255

***ormina***, *Mortoniella* 62

***ornata***, *Chimarrhodella* 401

*ornata*, *Nectopsyche* 327

*ornatus*, *Phylloicus* 30

***orophila guadeloupea***, *Alisotrichia* 198

***orophila orophila***, *Alisotrichia* 198

***orophila***, *Cariboptila* 46

***orotina orotina***, *Protoptila* 72

***orotina raposa***, *Protoptila* 72

**Orthotrichia** [Hydroptilidae] 279

***ortizi***, *Nectopsyche* 327

***ortiziana***, *Chimarra* 389

***ortizorum***, *Oxyethira* 288

***osa***, *Hydroptila* 220

**Osflintia** [Leptoceridae] 340

***oslari***, *Hydropsyche* 134

*ostina*, *Wormaldia* 407

***ostoroska***, *Ochrotrichia* 273

***otarosa***, *Angrisanoia* 202

***Otarrha*** [Philopotamidae] 369

***otuzcoensis***, *Chimarra* 389

***ovale***, *Parasericostoma* 451

***ovalis***, *Chimarra* 390

***ovis***, *Itauara* 52

***ovona***, *Neotrichia* 255

***oxima***, *Neotrichia* 255

**Oxyethira** [Hydroptilidae] 280

***Oxytrichia*** , [Hydroptilidae] 280

### P

***pablito***, *Chimarra* 390

***pachacamac***, *Atopsyche* 110

***pachacutec***, *Atopsyche* 110

***pacharurac***, *Atopsyche* 110

***pacifica***, *Ochrotrichia* 273

***pacuara***, *Mortoniella* 62

***padera***, *Leucotrichia* 228

***padrenavasi***, *Nectopsyche* 327

***pakitza***, *Metrichia* 241

***palaedominicana***†, *Chimarra* 390

**Palaehydropsyche** † [Hydropsychidae] 158

***palaenova***†, *Chimarra* 390

***palaeoelegans***†, *Calosopsyche* 128

***palida***, *Metrichia* 241

***palifera***, *Smicridea* 178

***paliferum***, *Macronema* 152

***palitla***, *Neotrichia* 256

***palitla***, *Ochrotrichia* 273

***pallas***, *Cnodocentron* 454

***pallida***, *Cernotina* 416

***pallida***, *Flintiella* 214

***pallidivittata***, *Smicridea* 179

***pallidum***, *Leptonema* 144

***palma***, *Neotrichia* 256

***palma***, *Wormaldia* 408

***palmar***, *Smicridea* 179

***palmara***, *Zumatrichia* 304

***palmata***, *Ochrotrichia* 273

***palmatiloba***, *Anchitrichia* 201

***palmitus***, *Polycentropus* 430

***paluguillensis***, *Contulma* 23

***pamelae***, *Neotrichia* 256

***pampeana***, *Smicridea* 179

*pampena*, *Smicridea* See ***pampeana***, *Smicridea*

***panama***, *Nothotrichia* 260

***panamensis***, *Alisotrichia* 198

***panamensis***, *Atanatolica* 316

***panamensis***, *Austrotinodes* 41

***panamensis***, *Costatrichia* 212

***panamensis***, *Flintiella* 214

***panamensis***, *Mortoniella* 62

***panamensis***, *Ochrotrichia* 273

***panamensis***, *Phylloicus* 35

***panamensis***, *Polyplectropus* 440

***pandeirosa***, *Helicopsyche* 91

**Pangullia** [Kokiriidae] 306

***pantosticta***, *Nectopsyche* 327

***papallacta***, *Contulma* 23

***papillata***, *Mortoniella* 62

***paprockevi***, *Wormaldia* 408

***paprockii***, *Alterosa* 368

***paprockii***, *Grumichella* 319

***paprockii***, *Helicopsyche* 91

***paprockii***, *Phylloicus* 35

***paprockii***, *Polycentropus* 430

***par***, *Macrostemum* 156

***parabullata***, *Neotrichia* 256

**Parachorema** [Hydrobiosidae] 121

***paracreagra***, *Chimarra* 390

***paradelphae***, *Polyplectropus* 440

***paradenza***, *Hydroptila* 220

***paradoxa***, *Cariboptila* 46

***paradoxicum***, *Heterochorema* 117

***paraenchrysa***, *Mortoniella* 62

***paraguaiensis***, *Helicopsyche* 91

***paraguaiensis***, *Mortoniella* 62

***paraguayensis***, *Austrotinodes* 41

***parahageni***, *Helicopsyche* 91

***paraldama***, *Ochrotrichia* 273

***paralineata***, *Mortoniella* 62

***parallelipipeda***, *Notidobiella* 450

***parana***, *Chimarra* 390

***paranaensis***, *Alterosa* 368

***parander***, *Streptopsyche* 188

***paranensis***, *Oecetis* 338

***paranensis***, *Smicridea* 179

***parany***, *Neotrichia* 256

***parany***, *Smicridea* 179

***paraortiziana***, *Chimarra* 390

*Paraprotoptila* [Glossosomatidae] 55

***pararusia***, *Culoptila* 49

**Parasericostoma** [Sericostomatidae] 450

***parati***, *Grumichella* 319

*Paratrichia* [Hydroptilidae] 201

***parauna***, *Atopsyche* 110

***parauna***, *Mortoniella* 62

***paraunota***, *Mortoniella* 62

***parazteca***, *Oxyethira* 288

***parce***, *Oxyethira* 288

***parene***, *Chimarra* 390

***parentum***, *Xiphocentron* 459

***parhuzam***, *Hydroptila* 220

***paria***, *Chimarra* 390

***paria***, *Chimarrhodella* 401

***parihuana***, *Atopsyche* 110

***parilis***, *Chimarra* 391

*parishi*, *Oecetis* 339

***paritentacula***, *Oxyethira* 288

***particeps***, *Chimarra* 391

*partita*, *Hydropsyche* 134

***partitum***, *Macronema* 152

*parva*, *Chimarra* 375

*parvula*, *Oecetis* 337

***parvum***, *Macronema* 152

***paschia***, *Hydroptila* 220

***passulatus***, *Phylloicus* 35

***patagonica***, *Metrichia* 241

*patagonica*, *Verger* 355

***patagonicum***, *Microthremma* 78

***patinae***, *Smicridea* 179

***patosa***, *Chimarra* 391

***patula***, *Oecetis* 338

***patulosa***, *Ochrotrichia* 273

***paucartambo***, *Phylloicus* 35

***paucartampu***, *Atopsyche* 110

***paucispina***, *Chimarra* 391

***paucispina***, *Helicopsyche* 91

***paulista***, *Notalina* 332

***pavida***, *Nectopsyche* 327

***paxilla***, *Alisotrichia* 198

***paxillifera***, *Sortosa* 403

***paysandu***, *Polyplectropus* 440

***pectinata***, *Leucotrichia* 228

***pectinata***, *Ochrotrichia* 274

***pectinella***, *Mortoniella* 62

***pectinifera***, *Ochrotrichia* 274

***pectinifera***, *Sortosa* 403

***pedernales***, *Polycentropus* 430

***pedicillata***, *Synoestropsis* 190

***pedophila***, *Cariboptila* 46

***peineta***, *Chimarra* 391

***pelaezi***, *Chimarra* 391

***pelei***, *Neotrichia* 256

*Pellopsyche* [Hydropsychidae] 160

**Peltopsyche** [Hydroptilidae] 293

***peltopsychodes***, *Eutonella* 461

***peluda***, *Metrichia* 241

***penai***, *Atanatolica* 316

***penai***, *Banyallarga* 27

***penai***, *Contulma* 23

***penai***, *Microchorema* 119

***penai***, *Smicridea* 179

***peniai***, *Parasericostoma* 451

***penicillata***, *Charadropsyche* 453

***penicillata***, *Metrichia* 241

***pennyi***, *Macronema* 153

***pequenita***, *Neotrichia* 256

***peravia***, *Atopsyche* 110

***percitans***, *Macronema* 153

***perdida***, *Protoptila* 72

***perija***, *Helicopsyche* 92

***perija***, *Phylloicus* 35

***perlucida***†, *Atopsyche* 110

***pernambucana***, *Metrichia* 242

***perpendicularis***, *Cernotina* 416

***perpendicularis***, *Polyplectropus* 441

***persimilis***, *Chimarra* 391

***pertyi***, *Macronema* 153

***peruana***, *Chimarra* 391

***peruana***, *Helicopsyche* 92

***peruana***, *Smicridea* 179

*peruanus*, *Rhyacophylax* See ***peruana***, *Smicridea*

***peruanus***, *Triaenodes* 344

***peruensis***, *Itauara* 52

***peruviana***, *Chimarra* 391

***peruviana***, *Chimarrhodella* 401

***peruviana***, *Oecetis* 338

***peruviana***, *Oxyethira* 289

***peruviana***, *Rhyacopsyche* 297

***peruvianus***, *Polyplectropus* 441

***pesae***, *Cernotina* 416

***pescaderum***, *Clavichorema* 116

***petasata***, *Smicridea* 180

***petei***, *Oxyethira* 289

***petersorum***, *Chimarra* 391

***petrae***, *Polyplectropus* 441

***petricola***, *Chimarra* 392

***peytoni***, *Chimarra* 392

*Phanopsyche* [Lepidostomatidae] 307

***philo***, *Hydropsyche* 134

***phraterus***, *Polycentropus* 430

***phthanorossi***, *Chimarra* 392

***phyllisae***, *Protoptila* 72

**Phylloicus** [Calamoceratidae] 28

***piacha***, *Protoptila* 72

***picada***, *Austrotinodes* 41

***picada***, *Neotrichia* 256

***picana***, *Polycentropus* 430

***picea***, *Chimarra* 392

***picigula***, *Zumatrichia* 304

***picita***, *Oxyethira* 289

***picteli***, *Macronema* 153

*picteti*, *Macronema* See ***picteli***, *Macronema*

***pictipes***, *Leucotrichia* 228

***picuda***, *Metrichia* 242

***pietia***, *Helicopsyche* 92

***pika***, *Acostatrichia* 193

***pilcomayo***, *Atopsyche* 110

***pilcopata***, *Bredinia* 205

***pilcopata***, *Chimarrhodella* 401

***piliferosa***, *Chimarra* 392

***piliferum***, *Leptonema* 144

***pillimpilli***, *Clavichorema* 116

***pinarenia***, *Neotrichia* 256

***pinotepa***, *Leptonema* 145

***pintada***, *Xiphocentron* 459

***pinula***, *Cheumatopsyche* 132

***pioneira***, *Centromacronema* 130

***pipila***, *Smicridea* 180

***pirapo***, *Phylloicus* 35

***piraya***, *Chimarra* 392

***piraya***, *Smicridea* 180

*pirioni*, *Hudsonema* 320

*pirioni*, *Verger* 357

***piroa***, *Helicopsyche* 92

***piscicaudum***, *Xiphocentron* 460

*pitella*, *Plectropsyche* 159

***pitu***, *Metrichia* 242

***pizotensis***, *Flintiella* 214

*Placocentropus* [Polycentropodidae] 421

***planae***, *Wormaldia* 408

***planata***, *Helicopsyche* 92

***plancki***, *Atopsyche* 110

***planorboides***, *Helicopsyche* 92

***platigona***, *Metrichia* 242

**Platycosmoecus** [Limnephilidae] 354

***platyrhina***, *Chimarra* 392

***plaumanni***, *Acostatrichia* 193

***plaumanni***, *Chimarra* 392

***plaumanni***, *Itauara* 53

***plaumanni***, *Phylloicus* 35

**Plectromacronema** [Hydropsychidae] 158

**Plectropsyche** [Hydropsychidae] 159

***plicatum***, *Leptonema* 145

***plumosum***, *Leptonema* See ***boliviense plumosum***

*plutonis*, *Chimarra* 389

***pluviale***, *Lepidostoma* 310

***poapi***, *Oxyethira* 289

***poblana***, *Ochrotrichia* 274

***pochutla***, *Smicridea* 180

***pocita***, *Mortoniella* 63

*Podomacronema* [Hydropsychidae] 158

***poeyi***, *Leptonema* 145

***poinari***†, *Cariboptila* 47

***polemon***, *Xiphocentron* 460

***poliochaeta***, *Helicopsyche* 92

***pollex***, *Chimarra* 392

***pollux***, *Limnephilus* 352

***polyacinata***, *Alterosa* 368

**Polycentropus** [Polycentropodidae] 421

***polyfasciata***, *Smicridea* 180

*polygramma*, *Macronema* 150

***polylepidum***, *Lepidostoma* 310

**Polyplectropus** [Polycentropodidae] 433

*Polytrichia* [Hydroptilidae] 261

**Pomphochorema** [Hydrobiosidae] 122

***ponta***, *Ochrotrichia* 274

***poolei***, *Chimarra* 392

***poquita***, *Cariboptila* 47

***pora***, *Ochrotrichia* 274

***porteri***, *Verger* 357

***potosi***, *Chimarra* 393

***potosina***, *Hydroptila* 221

***potosina***, *Metrichia* 242

*poujadi*, *Grumichella* 319

***powelli***, *Tinodes* 446

***poyanawa***, *Centromacronema* 131

***praecipua***, *Streptopsyche* 188

***pratherae***, *Polyplectropus* 441

***pratti***, *Oecetis* 338

***presilla***, *Oxyethira* 289

*pretiosella*, *Nectopsyche* 326

***priapo***, *Ochrotrichia* 274

*priapulus*, *Phylloicus* 33

***primerana***, *Protoptila* 72

***primula***, *Chimarra* 393

*princeps*, *Triplectides* 346

***probolophora***, *Smicridea* 180

***proboscidea***, *Neotrichia* 257

***procera***, *Tupiniquintrichia* 300

*proditus*, *Polycentropus* 442

***producta***, *Hydroptila* 221

***profaupar***, *Polyplectropus* 441

***profunda***, *Scelobotrichia* 298

***prolata***, *Chimarra* 393

***prolata***, *Metrichia* 242

***prolixa***, *Metrichia* 242

***prolixa***, *Sortosa* 403

***prolixa***, *Wormaldia* 408

***prolixum***, *Xiphocentron* 460

***prolixus***, *Austrotinodes* 41

***prolongata***, *Oecetis* 338

***propinqua***, *Helicopsyche* 92

***propinqua***, *Mortoniella* 63

***prorigera***, *Smicridea* 180

*Protarra* [Philopotamidae] 400

***protera***, *Smicridea* 180

**Protoptila** [Glossosomatidae] 66

***protrudens***, *Metrichia* 242

***protrusa***, *Oecetis* 339

***protuberans***, *Chimarra* 393

***provo***, *Tinodes* 446

***pseudoamazonica***, *Oecetis* 339

***pseudocinctum***, *Leptonema* 145

***pseudoinconspicua***, *Oecetis* 339

***pseudolobata***, *Smicridea* 180

**Pseudomacronema** [Hydropsychidae] 160

*pseudomeralda*, *Hydroptila* [nomen nudum] 217

***pseudopatagonica***, *Metrichia* 242

***pseudopiacha***, *Protoptila* 73

**Pseudoradema** [Hydrobiosidae] 122

***pseudoradula***, *Smicridea* 180

**Pseudosericostoma** [Helicophidae] 79

*Pseudosetodes* [Leptoceridae] 332

**Pseudostenopsyche** [Stenopsychidae] 452

***pseudostigmosum***, *Leptonema* 145

**Psilopsyche** [Philorheithridae] 410

***pucara***, *Smicridea* 181

***puertoricensis***, *Chimarra* 393

***puertoricensis***, *Oxyethira* 289

***pugiunculatus***, *Polyplectropus* 441

***puharcocha***, *Atopsyche* 110

***pulchella***, *Grumichella* 319

***pulcher***, *Monocosmoecus* 354

*pulcherrimus*, *Monocosmoecus* 354

***pulchra***†, *Cernotina* 416

***pulchra***, *Chimarra* 393

***pulchrus***, *Phylloicus* 36

***pulestoni***, *Hydroptila* 221

***pulgara***, *Neotrichia* 257

***pulgara***, *Ochrotrichia* 274

*pullatum*, *Macrostemum* 157

***pumida***, *Cailloma* 115

***pumila***, *Chimarra* 393

***pumila***, *Mortoniella* 63

***punctata***, *Nectopsyche* 328

***punctata***, *Oecetis* 339

***punctipennis***, *Oecetis* 339

***punctipennis***, *Synoestropsis* 190

*punctulata*, *Chimarra* 390

***punensis***, *Mortoniella* 63

***puposa***, *Ochrotrichia* 274

*purgatoria*, *Clavichorema* See ***purgatorium***, *Clavichorema*

***purgatorium***, *Clavichorema* 116

***puri***, *Cernotina* 416

***purisca***, *Chimarra* 393

***purpurea***, *Cariboptila* 47

***pusilla***, *Chimarra* 393

***pusilla***, *Mortoniella* 63

***puya***, *Chimarra* 394

***puyana***, *Ochrotrichia* 274

***puyoa***, *Helicopsyche* 93

***puyoensis***, *Polyplectropus* 441

***pygmaeum***, *Centromacronema* 131

***pylaea***, *Chimarra* 394

### Q

***qosqo***, *Triplectides* 347

***quadrata***, *Metrichia* 242

***quadratiterga***, *Chimarra* 394

***quadriappendiculatus***, *Polycentropus* 430

***quadricuspidis***, *Polycentropus* 430

***quadridigitatus***, *Phylloicus* 36

***quadrifidus***, *Brachysetodes* 317

*quadrifurca*, *Centromacronema* 129

***quadrifurcata***, *Chimarra* 394

***quadrispina***, *Austrotinodes* 41

***quadrispinosus***, *Polycentropus* 430

***quadrispinus***, *Verger* 357

***quadrosa***, *Helicopsyche* 93

***quasi***, *Ochrotrichia* 274

***quaternaria***, *Chimarra* 394

***quatuorguttata***, *Nectopsyche* 328

***quebrada***, *Amphoropsyche* 314

***quebrada***, *Ochrotrichia* 274

***quechua***, *Atanatolica* 316

*quelinda*, *Oxyethira* 289

***quemada***, *Scelobotrichia* 299

***quicha***, *Protoptila* 73

***quila***, *Lepidostoma* 310

***quina***, *Chimarra* 394

***quincemil***, *Banyallarga* 27

***quinealensis***, *Ochrotrichia* 275

***quinoi***, *Protoptila* 73

***quinquaginta***, *Oxyethira* 289

***quinuas***, *Mortoniella* 63

***quiramae***, *Oxyethira* 289

***quitacalzon***, *Chimarra* 394

***quitacalzon***, *Phylloicus* 36

### R

*radiale*, *Leptonema* 139

***radula***, *Smicridea* 181

***rafaeli***, *Metrichia* 243

***rafaeli***, *Oecetis* 339

***rafita***, *Chimarra* 394

***rafita***, *Leptonema* 145

***ragada***, *Ragatrichia* 295

**Ragatrichia** [Hydroptilidae] 294

***ralphi***, *Smicridea* 181

***ramona***, *Ochrotrichia* 275

***ramosi***, *Helicopsyche* 93

***ramosum***, *Leptonema* 145

***ramosum***, *Macrostemum* 156

*ramulorus*, *Triplectides* 346

***rancura***, *Mortoniella* 63

*ranea*, *Neotrichia* 259

***raposa***, *Ochrotrichia* 275

***raposa***, *Protoptila* See ***orotina raposa***

***rara***, *Mejicanotrichia* 232

***rara***, *Smicridea* 181

***rareza***, *Oxyethira* 289

*rarus*, *Smicridea* See ***rara***, *Smicridea*

***rastrilla***, *Hydroptila* 221

***rawlinsi***, *Atopsyche* 111

***rawlinsi***, *Metrichia* 243

***rawlinsi***, *Streptopsyche* 188

***rayada***, *Byrsopteryx* 207

***rayneri***, *Rhyacophila* 447

***real***, *Amphoropsyche* 314

***recintoi***, *Microchorema* 119

***recta***, *Austrotinodes* 41

***rectangulare***, *Lepidostoma* 310

*rectispina*, *Australochorema* See *rectispinum*, *Apatanodes*

*rectispinum*, *Apatanodes* 99

***recurvatus***, *Austrotinodes* 41

***recurvatus***, *Polyplectropus* 441

***redonda***, *Chimarra* 394

***redunca***, *Mortoniella* 63

***redunca***, *Oxyethira* 290

***redunca***, *Smicridea* 181

***refugia***, *Amphoropsyche* 314

***regina***, *Ochrotrichia* 275

***regiomontana***, *Ochrotrichia* 275

***regulare***, *Xiphocentron* 460

***reimoseri***, *Lepidostoma* 310

***reinburgi***, *Macronema* 153

***reinerti***, *Smicridea* 181

***rentzi***, *Helicopsyche* 93

***repanda***, *Leucotrichia* 229

*repula*, *Smicridea* 174

***resela***, *Smicridea* 181

***resinacapta***†, *Setodes* 341

***resinae***†, *Chimarra* 394

***resolda***, *Protoptila* 73

*reticulatus*, *Blepharopus* 125

***retrorsa***, *Chimarra* 394, 395

***rhamnes***, *Xiphocentron* 460

***rhamphisa***, *Rhyacopsyche* 298

***Rhamphocentron*** [Xiphocentronidae] 455

***rhamphodes***, *Chimarra* 395

***rhamphoides***, *Zumatrichia* 304

**Rheochorema** [Hydrobiosidae] 122

*rhino*, *Lepidostoma* 310

***rhomba***, *Leucotrichia* 229

**Rhyacophila** [Rhyacophilidae] 446

***Rhyacophylax*** [Hydropsychidae] 160

**Rhyacopsyche** [Hydroptilidae] 295

*Rhynchopsyche* [Kokiriidae] 306

*Rhyncorheithrus* [Stenopsychidae] 452

***rianus***, *Cyrnellus* 420

***ridleyi***, *Chimarra* 395

***riita***, *Smicridea* 181

***rinconi***, *Atopsyche* 111

*Rioptila* [Hydroptilidae] 194

***riosanjuanensis***, *Cernotina* 416

***riostoumae***, *Leucotrichia* 229

***riparia***, *Neotrichia* 257

***risi***, *Cyrnellus* 420

***ritae***, *Oxyethira* 290

***riva***, *Metrichia* 243

*rizona*, *Chimarra* 381

***robacki***, *Polyplectropus* 441

***robertsonae***, *Polyplectropus* 442

*robusta*, *Rheochorema* See ***robustum***, *Rheochorema*

***robustum***, *Rheochorema* 123

*robustus*, *Triplectides* 346

***rodmani***, *Itauara* 53

***rodmani***, *Mortoniella* 64

***rodmani***, *Polyplectropus* 442

***roldani***, *Mortoniella* 64

***rona***, *Metrichia* 243

***rondoniensis***, *Polyplectropus* 442

***rono***, *Hydroptila* 221

***roraima***, *Notalina* 332

***roraimense***, *Smicridea* 182

***rosalesi***, *Chimarra* 395

***rosalysae***, *Polycentropus* 431

*rosarius*, *Polycentropus* 430

***rosenbergi***, *Leptonema* 145

***rossi***, *Chimarra* 395

***rostrata***, *Grumichella* 319

***rostratum***, *Leptonema* 145

***rota***, *Protoptila* 73

***rothi***, *Limnephilus* 352

***rothi***, *Ochrotrichia* 275

***rotunda***, *Cailloma* 115

***rotundata***, *Neotrichia* 257

***rovatka***, *Betrichia* 203

***rovidka***, *Acostatrichia* 193

***rovira***, *Mortoniella* 64

***rualda***, *Mayatrichia* 231

***rubemarini***, *Cailloma* 116

***rubiginosum***, *Macronema* 153

*rufipes*, *Barypenthus* 359

***rufum***, *Parasericostoma* 451

***ruginasa***, *Smicridea* 182

***rugoka***, *Alisotrichia* 198

***ruiteri***, *Neotrichia* 257

***ruizi***, *Mastigoptila* 54

*ruiziana*, *Psilopsyche* 410

***ruschii***, *Alterosa* 368

***rusia***, *Culoptila* 50

### S

***saboriorum***, *Wormaldia* 409

***sacculifera***, *Metrichia* 243

*sagitta*, *Oecetis* 337

*sagittoides*, *Chimarrhodella* 401

*sagittosa*, *Phylloicus* 35

***sala***, *Neotrichia* 257

***salada***, *Neotrichia* 257

***salta***, *Marilia* 364

***salta***, *Protoptila* 73

***salta***, *Smicridea* 182

***saltena***, *Culoptila* 50

***saltuum***, *Xiphocentron* 460

***saluda***, *Zumatrichia* 305

***salvini***, *Leptonema* 145

***sana***, *Contulma* 23

***sanblasensis***, *Helicopsyche* 93

***sanchezi***, *Banyallarga* 27

***sancta***, *Contulma* 23

***sanctaeteresae***, *Alterosa* 368

***sancticaroli***, *Leptonema* 146

***sanctipauli***, *Alterosa* 369

***sanctipauli***, *Atopsyche* 111

***sandrae***, *Calosopsyche* 128

*Santae Ritae*, *Macronema* See ***santaeritae***, *Macrostemum*

***santaeritae***, *Macrostemum* 156

***santateresae***, *Polycentropus* 431

***santiaga***, *Mortoniella* 64

***santiagensis***, *Oxyethira* 290

***santiago***, *Polyplectropus* 442

***santosi***, *Leptonema* 146

***sarandi***, *Polycentropus* 431

***sarita***, *Leucotrichia* 229

***sarkos***, *Hydroptila* 221

***sarkoska***, *Smicridea* 182

***sarla***, *Smicridea* 182

***sarophora***, *Chimarra* 395

***sarvaka***, *Smicridea* 182

***sattleri***, *Smicridea* 182

***sauca***, *Hydroptila* 221

***saucia***, *Smicridea* 182

***savegra***, *Metrichia* 243

***scaeodactyla***, *Oxyethira* 290

***scalaris***, *Helicopsyche* 93

***scaloida***†, *Helicopsyche* 93

**Scelobotrichia** [Hydroptilidae] 298

***schadrackorum***, *Alterosa* 369

**Schajovskoya** [Hydrobiosidae] 123

***schiza***, *Chimarra* 395

*schmidi* Denning 1965, *Atopsyche* 102

*schmidi* Sykora 1991, *Atopsyche* 109

***schmidi***, *Alisotrichia* 198

***sclerothrix***, *Xiphocentron* 460

***scoparia***, *Oecetis* 339

***scopula***, *Chimarra* 395

***scopula***, *Sortosa* 403

***scopulina***, *Oxyethira* 290

***scopuloides***, *Chimarra* 395

**Scotiotrichia** [Glossosomatidae] 75

***scudderi***, *Marilia* 364

***scutellaris***, *Smicridea* 182

*secunda*†, *Isochorema* 117

***securigera***, *Chimarra* 396

***sedmani***, *Austrotinodes* 41

***segninii***, *Atopsyche* 111

***seiba***, *Ochrotrichia* 275

***selanderi***, *Helicopsyche* 93

***selva***, *Bredinia* 205

***selvatica***, *Hydroptila* 221

***sencilla***, *Metrichia* 243

***sencilla***, *Oxyethira* 290

***sensillata***, *Chimarra* 396

***sepala***, *Smicridea* 182

***separata***, *Metrichia* 243

***separata***, *Nectopsyche* 328

***septemlobata***, *Chimarra* 396

***septempunctata***, *Byrsopteryx* 207

***septifera***, *Chimarra* 396

***septifera***, *Helicopsyche* 93

***serestus***, *Xiphocentron* 460

***serica***, *Atopsyche* 111

***serra***, *Ochrotrichia* 275

***serrana***, *Ochrotrichia* 275

***serranum***, *Leptonema* 146

***serratum***, *Leptonema* 146

*serrei*, *Nectopsyche* 323

***sesquipedalis***, *Metrichia* 243

***setigera***, *Alisotrichia* 199

*setipes*, *Verger* 355

**Setodes** [Leptoceridae] 341

***setosa***, *Chimarra* 396

***sexspinosa***, *Cernotina* 417

***sexspinosa***, *Smicridea* 183

***shimigaya***, *Orthotrichia* 280

***shorti***, *Rhyacopsyche* 298

***sicilicula***, *Hydroptila* 222

***sicilicula***, *Neotrichia* 257

***sicula***, *Mortoniella* 64

***sidneyi***, *Leucotrichia* 229

***sieboldii***, *Peltopsyche* 294

***sierruca***, *Oxyethira* 290

***sigillata***, *Helicopsyche* 94

***signata***, *Smicridea* 183

***silex***, *Polycentropus* 431

***silva***, *Ochrotrichia* 276

*silvestrinum*, *Leptonema* 139

***silviae***, *Oecetis* 340

***sima***, *Zumatrichia* 305

***simanka***, *Oxyethira* 290

***similis***, *Metrichia* 243

***similis***, *Mortoniella* 64

***simla***, *Mortoniella* 64

***simmonsi***, *Smicridea* 183

***simples***, *Metrichia* 244

***simplex***, *Costatrichia* 212

***simplex***, *Itauara* 53

***simplex***, *Leptonema* 146

***simplex***, *Protoptila* 73

***simpliciforma***, *Chimarra* 396

***simplississimum***, *Pseudosericostoma* 79

***simulans mayanum***, *Leptonema* 146

***simulans simulans***, *Leptonema* 146

***simulans***, *Acostatrichia* 193

*simulatrix cubana*, *Oxyethira* 290

***simulatrix***, *Oxyethira* 290

***sinchicurac***, *Atopsyche* 111

***singri***, *Hydroptila* 222

***singri***, *Smicridea* 183

***singulare***, *Helicopsyche* 94

***sinistra***, *Oxyethira* 291

***sinosa***, *Cernotina* 417

***sinuatum***, *Leptonema* 146

***sinuatum***, *Neochorema* 120

***sinuosa***, *Cernotina* 417

***sinuata***, *Helicopsyche* 94

***sioli***, *Marilia* 364

***siolii***, *Atopsyche* 111

*siolii*, *Macrostemum* 157

***sirena***, *Smicridea* 183

*siva*, *Chimarra* 372

**Smicridea** [Hydropsychidae] 160

***smilodon***, *Smicridea* 183

***sociale***, *Leptonema* 147

***socialis***, *Atopsyche* 111

***sociata***, *Apatanodes* 99

***sokaga***, *Neotrichia* 257

***soleaferrea***, *Neotrichia* 257

*solex*, *Hydropsyche* 134

***solidus***, *Limnephilus* 352

***solisi***, *Byrsopteryx* 207

***solisi***, *Chimarra* 396

***solitaria***, *Diplectrona* 133

***soltera***, *Cariboptila* 47

***soniae***, *Polycentropus* 431

***sonora***, *Alisotrichia* 199

***sonora***, *Metrichia* 244

***sordida***, *Oecetis* 340

***soroa***, *Chimarra* 396

***sortetla***, *Zumatrichia* 305

**Sortosa** [Philopotamidae] 402

***soyatepecana***, *Smicridea* 183

***spada***, *Hydroptila* 222

***spangleri***, *Anchitrichia* 201

***spangleri***, *Ascotrichia* 202

***spangleri***, *Bredinia* 205

***spangleri***, *Chimarra*, Bueno 396

*spangleri*, *Chimarra*, Trivette ms. *See* ***pablito***, *Chimarra*

***spangleri***, *Hydroptila* 222

***spangleri***, *Leptonema* 147

***spangleri***, *Marilia* 364

***spangleri***, *Protoptila* 73

*sparsa*, *Nectopsyche* 324

***sparsum***, *Leptonema* 147

***spatulata***, *Chimarra* 397

***speciosum***, *Leptonema* 147

***spectabilis***, *Phylloicus* 36

***spectabilis***, *Sortosa* 403

*spegazzinia*, *Nectopsyche* 328

***sperryi***, *Atopsyche* 111

***Sphagocentron*** [Xiphocentronidae] 455

***spica***, *Metrichia* 244

***spicatus***, *Polycentropus* 431

*spicula*, *Chimarra* 381

***spiculifer***, *Polyplectropus* 442

***spiesae***, *Alterosa* 369

***spiloma***, *Nectopsyche* 329

***spina***, *Ochrotrichia* 276

***spinifera***, *Acostatrichia* 193

***spinifera***, *Amphoropsyche* 314

***spinifera***, *Costatrichia* 212

***spinifera***, *Sortosa* 403

***spinigera***, *Cernotina* 417

*spinosa* Mosely 1931, *Atopsyche* See ***iana***, *Atopsyche*

***spinosa***, *Atopsyche* 112

***spinosa***, *Cerasmatrichia* 209

***spinosa***, *Contulma* 23

***spinosella***, *Neoatopsyche* 120

***spinosella***, *Sortosa* 403

***spinosior***, *Cernotina* 417

***spinosissima***, *Ochrotrichia* 276

*spinosissima*, *Pseudoradema* See ***spinosissimum***, *Pseudoradema*

***spinosissimum***, *Pseudoradema* 122

***spinosula***, *Marilia* 364

***spinosus***, *Brachysetodes* 318

***spinosus***, *Verger* 357

***spinula***, *Ochrotrichia* 276

***spinulacolis***, *Phylloicus* 36

***spinulata***, *Mortoniella* 64

***spinulata***, *Ochrotrichia* 276

***spinulifera baoruco***, *Chimarra* 397

***spinulifera galalcha***, *Chimarra* 397

***spinulifera spinulifera***, *Chimarra* 397

***spinulosa***, *Ochrotrichia* 276

***spinulosa***, *Smicridea* 184

***spinulum***, *Leptonema* 147

***spira***, *Ochrotrichia* 276

***spiralis***, *Itauara* 53

***spirifera***, *Protoptila* 73

***spirillum***, *Leptonema* 147

***spirogyrae***, *Oxyethira* 291

***spirula***, *Hydroptila* 222

***spissa***, *Oxyethira* 291

***splendida***, *Nectopsyche* 329

***squalus***, *Polyplectropus* 442

***squamata***, *Mortoniella* 64

***squamigera***, *Metrichia* 244

***squamosa***, *Peltopsyche* 294

***stannardi***, *Cernotina* 417

***steffeni***, *Xiphocentron* 460

***steinhauseri***, *Lepidostoma* 310

***stella***, *Itauara* 53

***stellata***, *Amphoropsyche* 314

*stellula*, *Chimarra* 381

**Stenochorema** [Hydrobiosidae] 124

***stenopterus***, *Verger* 358

***stenotum***, *Xiphocentron* 460

***stictonota***, *Synoestropsis* 190

*stigmata*, *Verger* 356

***stigmatica***, *Nectopsyche* 329

***stigmaticum***, *Leptonema* 147

***stigmosum***, *Leptonema* 148

***stilula***, *Mortoniella* 65

***straminea***, *Chimarra* 397

***strepsicera***, *Protoptila* 73

**Streptopsyche** [Hydropsychidae] 187

***strobilina***, *Zumatrichia* 305

***strongyla***, *Chimarra* 397

***studiosorum***, *Macronema* 153

***sturmi***, *Xiphocentron* 460

***stylata***, *Ochrotrichia* 276

***subaequale***, *Macrostemum* 157

***subapicalis***, *Cernotina* 417

***subfuscum***, *Plectromacronema* 158

***subtropicalis***, *Anomalocosmoecus* 348

***succincta***, *Helicopsyche* 94

***succini***†, *Chimarra* 397

***sucrensis***, *Bredinia* 205

***sucrensis***, *Helicopsyche* 94

***sucusaria***, *Neotrichia* 257

***sudara***, *Smicridea* 184

***sugens***, *Pseudostenopsyche* 452

***suguioi***, *Indusia* [†] 10

***sunima***, *Chimarra* 397

***superbus***, *Phylloicus* 36

***surinamense***, *Macrostemum* 157

***surinamense***, *Xiphocentron* 461

***surinamensis***, *Ascotrichia* 202

***surinamensis***, *Hydroptila* 222

***surinamensis***, *Polycentropus* 431

***sylvana***, *Banyallarga* 27

**Synoestropsis** [Hydropsychidae] 188

### T

***tabasquensis***, *Byrsopteryx* 207

***tachira***, *Helicopsyche* 94

***tachuela***, *Chimarra* 397

***tacuarembo***, *Nyctiophylax* 421

***taeniata***, *Cernotina* 417

***tagala***, *Ochrotrichia* 276

***tagola***, *Orinocotrichia* 279

***taina***, *Atopsyche* 112

***tajo***, *Triaenodes* 344

***talamanca***, *Atopsyche* 112

***talamanca***, *Contulma* 23

***talamanca***, *Protoptila* 74

***talamanca***, *Smicridea* 184

***talamanca***, *Triaenodes* 344

***talamancense***, *Lepidostoma* 310

***talan***, *Centromacronema* 131

***talcana***, *Austrotinodes* 42

***taleola***, *Nectopsyche* 329

***talhada***, *Metrichia* 244

***tamandua***, *Oxyethira* 291

***tamaulipasa***, *Flintiella* 214

***tamaza***, *Mejicanotrichia* 232

***tamba***, *Chimarra* 398

***tampurimac***, *Atopsyche* 112

***tandayapa***, *Amphoropsyche* 314

***tanylobosa***, *Rhyacopsyche* 298

***Tanytrichia*** , [Hydroptilidae] 280

***tapada***, *Acostatrichia* 193

***tapadas***, *Helicopsyche* 94

***tapanti***, *Atopsyche* 112

***tapanti***, *Banyallarga* 27

***tapanti***, *Byrsopteryx* 207

***tapanti***, *Chimarra* 398

***tapanti***, *Chimarrhodella* 401

***tapanti***, *Contulma* 23

***tapanti***, *Culoptila* 50

***tapanti***, *Lepidostoma* 311

***tapanti***, *Leptonema* 148

***tapanti***, *Mortoniella* 65

***tapanti***, *Nectopsyche* 329

***tapanti***, *Smicridea* 184

***tapanti***, *Triaenodes* 344

***tapantia***, *Leucotrichia* 229

***taquaralis***, *Austrotinodes* 42

***tarahumara***, *Protoptila* 74

***tarasca***, *Smicridea* 184

***tarasca***, *Wormaldia* 409

***tarascanica***, *Culoptila* 50

**Taraxitrichia** [Hydroptilidae] 299

***tarpeia***, *Machairocentron* 455

***tarquon***, *Xiphocentron* 461

***tarsalis***, *Ochrotrichia* 277

***tauricornis***, *Neotrichia* 258

***taurina***, *Mortoniella* 65

***tavola***, *Smicridea* 184

***teapa***, *Zumatrichia* 305

***techila***, *Protoptila* 74

***tega antillularum***, *Oxyethira* 291

***tega tega***, *Oxyethira* 291

***teixeirai***, *Oxyethira* 292

***temascalapensis***, *Metrichia* 244

***temora***, *Helicopsyche* 94

***tenanga***, *Ochrotrichia* 277

*tenuispina*, *Rheochorema* See ***tenuispinum***, *Rheochorema*

***tenuispinum***, *Rheochorema* 123

***tenuivirga***, *Alisotrichia* 199

***tepui***, *Triplectides* 347

***tere***, *Metrichia* 244

***teresae***, *Chimarra* 398

***teribe***, *Zumatrichia* 305

***termitiformis***, *Leucotrichia* 229

***ternatia***, *Protoptila* 74

***tertia***, *Neotrichia* 258

*testacea*, *Banyallarga* 27

*Tetanonema* [Helicopsychidae] 79

*Tetracentron* [Leptoceridae] 344

***tetraespinosa***, *Alisotrichia* 199

***tetravittata***, *Protoptila* 74

***teucrus***, *Machairocentron* 455

***teutona***, *Mortoniella* 65

***teutonia***, *Neotrichia* 258

***texana***, *Chimarra* 398

***textor***, *Lepidostoma* 311

***thalia***, *Alisotrichia* 199

***thallina***, *Nectopsyche* 329

***thelidomus***, *Helicopsyche* 95

*therezieni*, *Smicridea* 183

***thermophila***, *Smicridea* 184

***thilus***, *Polyplectropus* 442

***thirysae***, *Metrichia* 244

***tholloni***, *Leptonema* 148

***thomasi***, *Atopsyche* 112

***thoracica***, *Culoptila* 50

***tica***, *Byrsopteryx* 207

***tica***, *Contulma* 23

***tica***, *Leptonema* 148

***tica***, *Oxyethira* 292

***tica***, *Protoptila* 74

***tico***, *Triaenodes* 344

***ticumanensis***, *Protoptila* 74

***tijuca***, *Contulma* 23

***timbira***, *Helicopsyche* 95

***timouchela***, *Alisotrichia* 199

***tina***, *Smicridea* 184

***tincuracu***, *Atopsyche* 112

**Tinodes** [Psychomyiidae] 445

*Tipulitides* [†] 10

***tirabuzona***, *Neotrichia* 258

***titschacki***, *Smicridea* 184

***tiza***, *Alisotrichia* 199

**Tizatetrichia** [Hydroptilidae] 299

***tlaloc***, *Atopsyche* 112

***tobada***, *Smicridea* 184

***tobaga***, *Chimarra* See ***caribea tobaga***

***tobaga***, *Hydroptila* 222

***tobagoensis***, *Chimarrhodella* 401

***toblet***, *Macronema* 153

***tocajoma***, *Wormaldia* 409

***tojana***, *Protoptila* 74

**Tolhuaca** [Glossosomatidae] 75

***tollas***, *Leptonema* 148

***tompa***, *Neotrichia* 258

***tompagula***, *Zumatrichia* 305

***topora***, *Acostatrichia* 193

***torpa***, *Smicridea* 185

***tortuosa***, *Chimarra* 398

***torulosa***, *Rhyacopsyche* 298

***torza***, *Oxyethira* 292

***toschiae***, *Hydropsyche* 134

*toussianti*, *Limnephilus* 352

***tragularius***, *Polyplectropus* 443

***traini***, *Oecetis* 340

*trancasica*, *Clavichorema* See ***trancasicum***, *Clavichorema*

***trancasicum***, *Clavichorema* 116

***tranquilla***, *Mortoniella* 65

***travertinera***, *Smicridea* 185

***tregala***, *Smicridea* 185

***tremenda***, *Macronema* 153

**Triaenodes** [Leptoceridae] 341

***triangula***, *Metrichia* 244

***triangularis***, *Austrotinodes* 42

***triangularis***, *Marilia* 364

***tricalcaratus***, *Phylloicus* 36

*tricarinata*, *Neopsilochorema* See ***tricarinatum***, *Neopsilochorema*

***tricarinatum***, *Neopsilochorema* 121

***trichoglossa***, *Protoptila* 74

**Tricholeiochiton** [Hydroptilidae] 300

***trichothylax***, *Phylloicus* 36

**Trichovespula** [Tasimiidae] 453

***tridens***, *Cariboptila* 47

***tridens***, *Leptonema* 148

***tridentata***, *Mejicanotrichia* 232

***trifida***, *Atopsyche* 112

***trifida***, *Mejicanotrichia* 232

***trifidum***, *Leptonema* 148

*trifidus*, *Atopsyche* See ***trifida***, *Atopsyche*

***trifidus***, *Brachysetodes* 318

***trifurcata***, *Anchitrichia* 201

***trigonella***, *Metrichia* 244

***trigramma***, *Macrostemum* 157

***trilineatum***, *Cnodocentron* 454

*trilobata*, *Leptonema* 135

***trilobatus***, *Polyplectropus* 443

***trinitatis***, *Cerasmatrichia* 209

***trinitatis***, *Ochrotrichia* 277

*trinitatis*, *Xiphocentron* 458

***tripartita***, *Costatrichia* 212

***tripartitum***, *Leptonema* 148

***tripartitus***, *Brachysetodes* 318

**Triplectides** [Leptoceridae] 344

***tripui***, *Contulma* 23

***tripui***, *Polycentropus* 431

***tripuiensis***, *Alterosa* 369

***tripuiensis***, *Mortoniella* 65

***tripunctata***, *Atopsyche* 112

***tripunctatum***, *Polyplectropus* 443

***triquetra***, *Metrichia* 244

***trispicata***, *Protoptila* 74

***trispicatum***, *Leptonema* 148

***trispina***, *Cernotina* 417

***trispinata***, *Cariboptila* 47

***trispinosa***, *Metrichia* 245

***triste***, *Macrostemum* 157

***tritoven***, *Leucotrichia* 229

***trondi***, *Wormaldia* 409

***trujilloi***, *Grumichella* 320

***truncata***, *Alterosa* 369

***truncata***, *Helicopsyche* 95

***truncata***, *Marilia* 364

***truncata***, *Metrichia* 245

***truncata***, *Mortoniella* 65

***truncata***, *Protoptila* 75

***truncata***, *Smicridea* 185

***truncatiloba***, *Chimarra* 398

*truncatus*, *Monocosmoecus* 354

***tuanis***, *Nectopsyche* 330

***tuberculatus***, *Polycentropus* 431

*tuberosum* var. *ramosa* See ***ramosum***, *Macrostemum*

*tuberosum*, *Macrostemum* 155

***tubicola***, *Antillopsyche* 445

***tubifex***, *Leucotrichia* 229

***tubulifera***, *Neotrichia* 258

***tucuna***, *Chimarra* 398

***tulatus***, *Limnephilus* 352

***tulipa***, *Hydroptila* 222

***tumida***, *Oecetis* 340

***tupacamara***, *Wormaldia* 409

***tupi***, *Nothotrichia* 261

**Tupiniquintrichia** [Hydroptilidae] 300

***turbida***, *Helicopsyche* 95

***turgida***, *Smicridea* 185

***turquino***, *Polycentropus* 431

***turrialbae***, *Rhyacopsyche* 298

***turrialbana***, *Smicridea* 185

***turrialbum***, *Leptonema* 149

***turuda***, *Zumatrichia* 305

***tusa***, *Zumatrichia* 305

***tuscaloosa***, *Mayatrichia* 231

***tusci***, *Itauara* 53

***tuskera***, *Acostatrichia* 193

***tuveva***, *Oxyethira* 292

***tuxtla***, *Neotrichia* 258

***tuxtlensis***, *Austrotinodes* 42

***tuxtlensis***, *Helicopsyche* 95

***tuxtlensis***, *Triaenodes* 344

### U

***uara***, *Cernotina* 417

***uara***, *Chimarra* 398

***ubajara***, *Metrichia* 245

***ubatuba***, *Alisotrichia* 199

***ujasa***, *Acostatrichia* 193

***ulmeri***, *Atopsyche* 113

***ulmeri***, *Chimarrhodella* 401

***ulmeri***, *Cyrnellus* 420

***ulmeri***, *Iguazu* 117

*ulmeri*, *Leptonema* 142

***ulmeri***, *Macrostemum* 157

***ulmeri***, *Oxyethira* 292

***ulmeriana***, *Polyplectropus* 443

***ultima***, *Alisotrichia* 199

***ultimus***, *Triplectides* 347

***ulva***, *Smicridea* 185

***umbonata***, *Helicopsyche* 95

***umbonata***, *Mortoniella* 65

***unamas***, *Neotrichia* 258

***uncata***, *Oecetis* 340

***uncatum***, *Leptonema* 149

***uncifera***, *Cernotina* 418

***unguiculata***, *Cernotina* 418

***unguiculata***, *Smicridea* 185

***unica***, *Ochrotrichia* 277

*unicolor* Banks 1938, *Smicridea* 163

***unicolor***, *Smicridea* 185

***unicolorata***, *Atopsyche* 113

***unicornia***, *Ochrotrichia* 277

***unicuspis***, *Hydroptila* 222

***unidentata***, *Itauara* 53

***unilineata***, *Mortoniella* 66

***unispina***, *Culoptila* 50

***unispina***, *Neoatopsyche* 120

***unispina***, *Neotrichia* 259

***unispina***, *Oxyethira* 292

***unispinus***, *Polycentropus* 432

Unknown family 1 [Atriplectididae] 24

***unota***, *Mortoniella* 66

***urra***, *Smicridea* 185

***urubici***, *Polycentropus* 432

***uruguaiensis***, *Mortoniella* 66

***uruguayensis***, *Atopsyche* 113

***uruguayensis***, *Austrotinodes* 42

***uruguayensis***, *Betrichia* 203

***uruguayensis***, *Protoptila* 75

***urumarca***, *Atopsyche* 113

***usingeri***, *Atopsyche* 113

***usitatissima***, *Chimarra* 398

***usseglioi***, *Mortoniella* 66

***utahensis***, *Chimarra* 399

*utico*, *Smicridea* 169

***utleyorum***, *Nectopsyche* 330

***utra***, *Chimarra* 399

### V

***vagotta***, *Smicridea* 186

***vaina***, *Oxyethira* 292

*vakaca*, *Nectoopsyche* 329

***vakara***, *Smicridea* 186

***valdiviensis***, *Polycentropus* 432

***valeni***, *Smicridea* 186

***valga***, *Marilia* 364

***valgiformis***, *Austrocentrus* 77

***valligera***, *Helicopsyche* 95

***valverdei***, *Contulma* 24

***vanderpooli***, *Polycentropus* 432

***vanderweeli***, *Monocosmoecus* 354

***varia***, *Smicridea* 186

***variipenne***, *Macronema* 154

***varrata***, *Zumatrichia* 305

***varratlana***, *Betrichia* 203

***vaskosa***, *Smicridea* 186

***vatucra***, *Atopsyche* 113

***vavai***, *Neotrichia* 259

***vaza***, *Oxyethira* 292

***vazquezae***, *Helicopsyche* 95

***vazquezae***, *Hydroptila* 222

***vegosa***, *Peltopsyche* 294

***vekona***, *Smicridea* 186

***vekonyka***, *Neotrichia* 259

***velascoi***, *Ochrotrichia* 277

***velascoi***, *Plectropsyche* 159

***velasquezi***, *Mortoniella* 66

*veleda*, *Lepidostoma* 310

***velteni***†, *Phylloicus* 36

***venezolanus***, *Polyplectropus* 443

***venezuelensis***, *Bredinia* 205

***venezuelensis***, *Costatrichia* 212

***venezuelensis***, *Helicopsyche* 96

***venezuelensis***, *Hydroptila* 222

***ventrale***, *Glossosoma* 50

*Ventrarma* [Hydrobiosidae] 99

***ventricornuta***, *Mastigoptila* 54

***ventricosa***, *Alisotrichia* 199

***ventricosta***, *Sortosa* 403

***ventridenticulata***, *Smicridea* 186

***veracruzensis***, *Hydroptila* 223

***veracruzensis***, *Polycentropus* 432

***veracruzensis***, *Protoptila* See ***mixteca veracruzensis***

***veracruzensis***, *Smicridea* 186

***verda***, *Ochrotrichia* 277

***vergelana***, *Helicopsyche* 96

**Verger** [Limnephilidae] 355

***vermiculata***, *Smicridea* 186

***verna***, *Cernotina* 418

***verrucula***, *Oecetis* 340

***verruculus***, *Polycentropus* 432

***verticalis***, *Cernotina* 418

***vespersus***, *Verger* 358

***vespertina***, *Hydropsyche* 134

***veva***, *Peltopsyche* 294

***vexillifera***, *Culoptila* 50

***vibrans***, *Neotrichia* 259

***vicaria***, *Banyallarga* 27

*vicarium*, *Ganonema* See ***yungensis***, *Banyallarga*

***vieja***, *Ochrotrichia* 277

***vieja***, *Zumatrichia* 305

***vilela***, *Smicridea* 187

***villa***, *Neotrichia* 259

***villa***, *Smicridea* 187

***villalobosi***, *Chimarra* 399

***villarenia***, *Ochrotrichia* 278

***villarricensis***, *Smicridea* 187

***villegasi***, *Helicopsyche* 96

***villosa***, *Banyallarga* 28

***villosum***, *Microthremma* 78

***vinai***, *Atopsyche* 113

***vipera***, *Oxyethira* 293

***viracocha***, *Atopsyche* 113

***virgencita***, *Chimarra* 399

***virginiae***, *Polycentropus* 432

***viridianum***, *Leptonema* 149

***viridis***, *Leucotrichia* 230

***vissa***, *Neotrichia* 259

***vitrea***, *Synoestropsis* 190

***vittatum***, *Pseudomacronema* 160

***vitum***, *Leptonema* 149

***viuda***, *Alisotrichia* 199

***voigti***†, *Helicopsyche* 96

***volcanus***, *Polycentropus* 432

*volenta*, *Chimarra* 393

***voluta***, *Protoptila* 75

***voluta***, *Smicridea* 187

***vonza***, *Neotrichia* 259

***vulgaris***, *Metrichia* 245

### W

***wallacei***, *Plectropsyche* 159

***warema***, *Metrichia* 245

*weaveri*, *Lepidostoma* 311

***weibezahni***, *Atopsyche* 114

***weidneri***, *Smicridea* 187

***weitschati***†, *Chimarra* 399

***wilcuma***, *Chimarra* 399

***williami***, *Canoptila* 43

***williammerrilli***, *Marilia* 364

***wilsoni***, *Chimarra* 399

***wirthi***, *Cerasmatrichia* 209

***woldai***, *Alisotrichia* 200

***woldai***, *Chimarra* 399

***woldai***, *Helicopsyche* 97

***woldai***, *Polyplectropus* 443

***woldai***, *Triaenodes* 344

***woldianum***, *Leptonema* 149

***woodruffi multispinosa***, *Amphoropsyche* 314

***woodruffi woodruffi***, *Amphoropsyche* 314

***woodruffi***, *Alisotrichia* 200

**Wormaldia** [Philopotamidae] 403

***woytkowskii***, *Helicopsyche* 97

***wrighti***, *Antillopsyche* 445

***wrighti***, *Marilia* 364

***wygodzinskii***, *Mortoniella* 66

***wygodzinskyi***, *Merionoptila* 55

### X

*xesta*, *Chimarra* 374

***xicana***, *Neotrichia* 259

***xingu***, *Chimarra* 399

***xinguensis***, *Helicopsyche* 97

**Xiphocentron** [Xiphocentronidae] 455

*Xirocentron* [Xiphocentronidae] 455

***xolotl***, *Lepidostoma* 311

***xus***, *Chimarra* 400

### Y

***yagua***, *Neotrichia* 259

***yalla***, *Metrichia* 245

***yanamona***, *Flintiella* 214

***yanayacuana***, *Ochrotrichia* 278

***yanomonoa***, *Neotrichia* 259

***yanura***, *Chimarra* 400

***yatay***, *Ragatrichia* 295

***yavesia***, *Metrichia* 245

***yavesia***, *Neotrichia* 259

***yavesia***, *Ochrotrichia* 278

***yepachica***, *Ochrotrichia* 278

***yetla***, *Ochrotrichia* 278

***yolandae***, *Phylloicus* 36

***yolandae***, *Polyplectropus* 443

***youngi***, *Atopsyche* 114

***ypsilon***, *Chimarra* 400

***yungarum***, *Leucotrichia* 230

***yungensis***, *Banyallarga* 28

***yunguensis***, *Atopsyche* 114

***yupanqui***, *Atopsyche* 114

***yurumanga***, *Protoptila* 75

### Z

***zagalloi***, *Neotrichia* 260

***zamora***, *Chimarra* 400

***zamoranoensis***, *Polyplectropus* 443

***zanclana***, *Cernotina* 418

*zanclophora*, *Smicridea* 177

***zanclus***, *Polycentropus* 432

***zaneta***, *Polycentropus* 432

***zapateriensis***, *Cyrnellus* 420

***zaragozai***, *Polyplectropus* 443

***zegla***, *Zumatrichia* 305

***zerbinae***, *Hydroptila* 223

***zernyi***, *Atopsyche* 114

*zernyi*, *Cyrnellus* 419

***zihuaquia***, *Ochrotrichia* 278

***zilaba***, *Oxyethira* 293

***zilbra***, *Betrichia* 203

*zion*, *Cheumatopsyche* 131

***zitoi***, *Neotrichia* 260

***zongo***, *Atanatolica* 317

***zopilote***, *Leucotrichia* 230

***zotheculum***, *Amphichorema* 98

***zulia***, *Bredinia* 205

***zuliae***, *Polyplectropus* 443

**Zumatrichia** [Hydroptilidae] 301

***zunigae***, *Wormaldia* 409

***zunigarceorum***, *Wormaldia* 409

***zurqui***, *Polycentropus* 433
